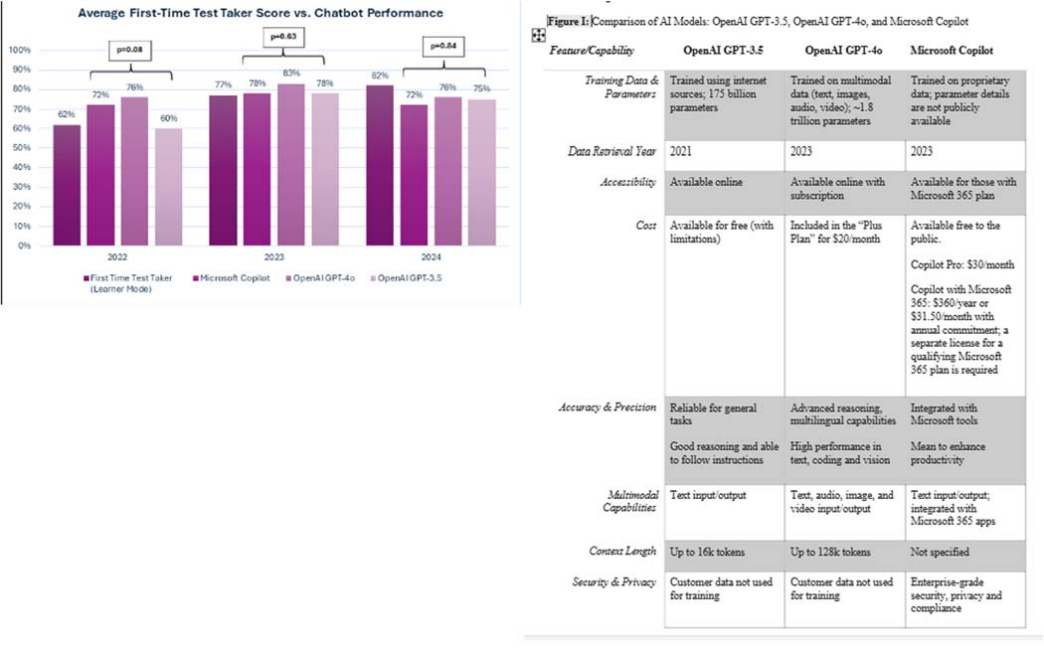# Abstract

**DOI:** 10.1002/jpr3.70080

**Published:** 2025-09-25

## 2* BUT IS IT REALLY SILENT? ANALYSIS OF SOUNDS MADE DURING FEEDING MAY PROVIDE INSIGHT INTO SWALLOW SAFETY IN CHILDREN WITH OROPHARYNGEAL DYSPHAGIA

Daniel Duncan^1^, Clare Golden^1^, Michael Kim^1^, Kara Larson^1^, Louisa Ferrara‐Gonzalez^2^, Simon Johnson^2^, Caroline Martinez^2^, Rachel Rosen^1^

^1^Aerodigestive Center/Gastroenterology, Boston Children's Hospital, Boston, MA; ^2^Sipple Care, Riverside, CT

**Background:** Diagnosis and management of oropharyngeal dysphagia in young children requires instrumental swallow assessment with videofluoroscopic swallow study (VFSS) since more than 80% of aspiration is silent in this age group. However, acoustic analysis of sounds made during feeding is a novel approach that may reveal key features of swallowing and breathing coordination which are associated with increased aspiration risk. We hypothesize that metrics derived from acoustic analysis could be used to discriminate between safe versus unsafe swallowing.

**Methods:** We recruited children under 2 years of age undergoing evaluation for oropharyngeal dysphagia. We used an iOS application (Sipple) to record sounds made during feedings which then underwent acoustic analysis. A machine learning model was used to classify each sound with a suck/swallow/breathe probability and to derive metrics of suck‐swallow‐breathe coordination. Medical records were reviewed for VFSS results including highest penetration aspiration scale (PAS) score and whether the liquid viscosity tested during audio recording was safe or unsafe, defined by any aspiration or laryngeal penetration seen with the given consistency on VFSS. Swallow coordination metrics were compared between safe and unsafe groups using t‐tests. Principal component analysis was used to identify key features that differed between the groups by swallow safety and PAS score.

**Results:** Recordings of a total of 3,141 swallows from 44 children with mean age 7.8 ± 0.8 months were analyzed. Comparison of swallow coordination metrics, percentage of sucks followed by swallows, average inhale duration, average exhale duration, and breathing pattern consistency composite scores all were found to be higher in the unsafe group. Subject characteristics and swallow coordination metrics are shown in Table 1. Principal components analysis clustered the metric data into two groups, including PC1 (Suck‐Swallow‐Breathe Integration) which explained 29.9% of the variance and PC2 (Swallow Timing Variability) which explained 17.2% of the variance between groups. Data were also found to cluster by PAS score, as shown in Figure 1.

**Conclusions:** Subtle differences in swallowing and associated breath sounds differentiate young children who aspirate from those that do not. Findings from this novel acoustic analysis suggest this approach may be used to discriminate safe from unsafe swallows in children with oropharyngeal dysphagia.


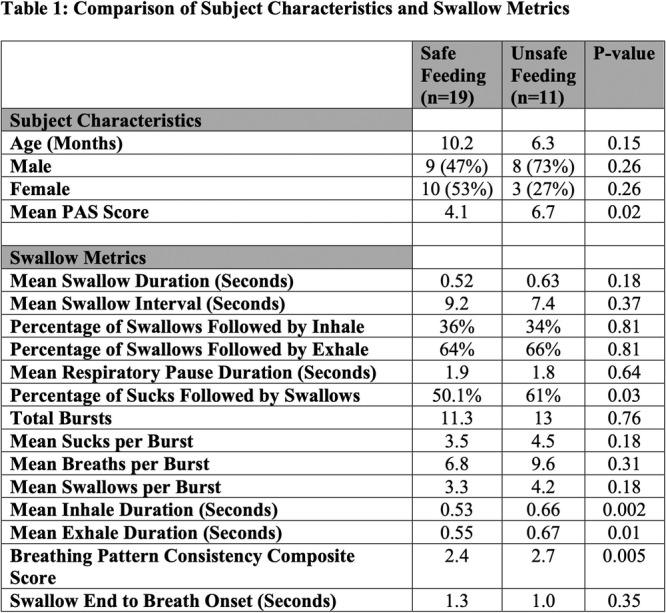



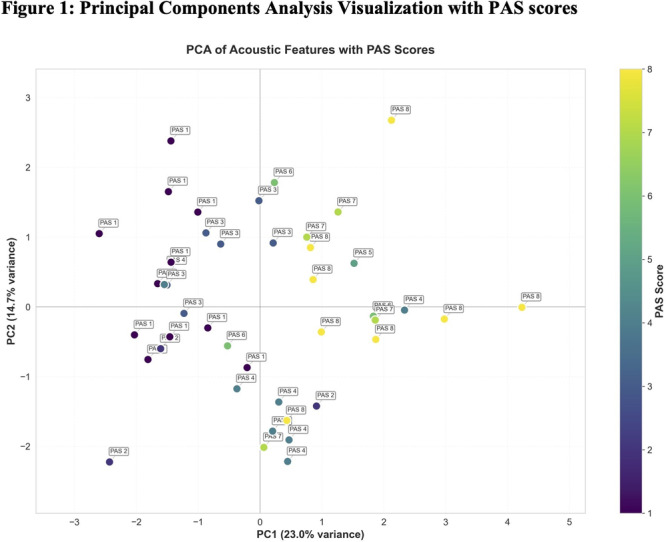


## 4 CORRELATION OF ACID EXPOSURE TIME AND REFLUX INDEX WITH HISTOLOGIC SEVERITY OF ESOPHAGITIS IN INFANTS UNDERGOING PH‐MII TESTING

*Eru Sujakhu* ^*1*^, *David Gremse* ^*2*^

^*1*^ *University of South Alabama*, *Mobile*, *AL*; ^*2*^ *University of South Alabama*, *Mobile*, *AL*

**Background:** The pH‐multichannel intraluminal impedance (pH‐MII) monitor is a highly sensitive tool used to detect gastroesophageal reflux. The pH‐MII results may be analyzed including all episodes of pH <4 or only reflux episodes associated with impedance changes. Low‐volume acid reflux without impedance changes may occur by proximal migration of a subcardial acid pocket or acid “film” that would not be captured by computer software that analyzes pH‐MII studies and recognizes only those reflux events that are associated with impedance change. The acid exposure time (AET) includes all esophageal pH measurements <4 with or without impedance changes and the reflux index (RI) calculated by proprietary software includes only episodes of pH<4 associated with impedance changes. However, the clinical significance of these two methods in predicting clinical outcomes such as the histologic severity of esophagitis remain underexplored.

**Objective:** To compare AET and RI and assess their respective correlations with histologic severity of esophagitis in infants who underwent pH‐MII testing.

**Methods:** We conducted a retrospective cohort study that included infants who underwent pH‐MII monitoring and EGD with biopsy between 2020 and 2025 at the University of South Alabama Children and Women's Hospital. The AET was determined by manual review of the pH versus time measurements for each patient while the RI was obtained using Sandhill Scientific software. The histologic severity of esophagitis was graded by a single pathologist using a standardized scale from 0 (none) to 4 (severe). Spearman's correlation and ordinal logistic regression assessed associations with histologic severity, and a Wilcoxon signed‐rank test compared AET and RI across severity grades.

**Results:** Among 203 infants who underwent pH MII monitoring, the mean age was 3.73 ± 2.19 months, with 58.6% being male and 72.4% identified as white. The median manual AET was 4.9% compared to 2.5% for the RI. AET and RI were compared across the histologic grades of esophagitis which were absent in 37.4%, minimal in 29.6%, mild in 29.1%, moderate in 3.5%, and severe in 0.5% of patients. Both AET and RI showed an upward trend with increasing severity as shown in Figure 1. Spearman's correlation analysis showed a weak but positive association between esophagitis grade and both AET (rs = 0.28) and RI (rs = 0.30). Ordinal logistic regression also demonstrated statistical significance in both AET (OR = 1.03; 95% CI: 1.004–1.062; p = 0.0308) and RI (OR = 1.04; 95% CI: 1.01–1.08; p = 0.0124) with increasing severity of esophagitis. RI showed a lower Akaike Information Criterion (AIC = 502.5 vs. 504.0 for manual), which suggested a better model fit and predictive performance. While comparing AET and RI values within esophagitis grades 1, 2, and 3 using Wilcoxon signed‐rank tests, AET was higher in all these histologic grades compared to RI. For grade 1, median (Q1–Q3) AET was 4.4 (2.68–6.43) vs. 1.95 (0.70–4.60) for RI (p < 0.001); for grade 2, 6.8 (4.00–9.40) vs. 3.7 (2.00–8.55) (p < 0.001); and for grade 3, 14.5 (12.30–17.55) vs. 4.1 (3.05–10.80) (p = 0.036). The RI demonstrated a slightly higher AUC (0.629 vs. 0.602) although DeLong's test comparing ROC curves showed no statistically significant differences (p = 0.41), indicating comparable diagnostic performance.

**Conclusion:** Both AET and RI were significantly associated with increasing histologic severity of esophagitis in infants undergoing pH‐MII monitoring. Although the computer‐generated index demonstrated a slightly stronger Spearman correlation and better predictive performance, the higher AET in patients with minimal, mild and moderate esophagitis, suggests that acid reflux without impedance changes may result in histologic changes of reflux esophagitis.


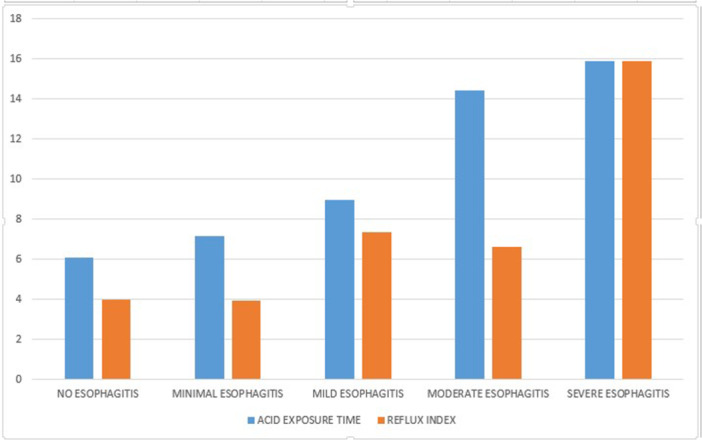


Figure 1: Mean Reflux Scores By Esophagitis Grade: Manual Vs Computer Analysis

## 7 BENEFITS OF A BLENDED DIET: SECONDARY ANALYSIS OF THE IMPACT OF DIET TYPE ON FEEDING TUBE WEANING SUCCESS

*Kristina Nash* ^*1*^, *Dana Bakula* ^*1*^, *Rachel Graham* ^*1*^, *Brenda Fetter* ^*2*^, *Sarah Bullard* ^*4*^, *April Escobar* ^*3*^, *Julianne Brogren* ^*5*^, *Amy Ricketts* ^*5*^, *Ryan Thompson* ^*5*^, *Lori Erickson* ^*5*^, *Sarah Edwards* ^*1*^

^*1*^ *Gastroenterology*, *Children's Mercy Kansas City*, *Kansas City*, *MO*; ^*2*^ *Hearing and Speech*, *Children's Mercy Kansas City*, *Kansas City*, *MO*; ^*3*^ *Nutrition Services*, *Children's Mercy Kansas City*, *Kansas City*, *MO*; ^*4*^ *Center for Children's Healthy Lifestyles and Nutrition*, *Children's Mercy Kansas City*, *Kansas City*, *MO*; ^*5*^ *Remote Health Solutions, Strategy, Innovation, and Partnerships*, *Children's Mercy Kansas City*, *Kansas City*, *MO*

**Introduction:** Enteral feeding tubes are needed to support growth and nutrition for a subset of children with pediatric feeding disorder (PFD). When medically ready, feeding tube weaning improves patient and family satisfaction, decreases clinic visits and hospital stays, decreases costs, and improves mealtime experiences. A commercially made formula is the route of many children's nutrition regimen, but many families report a preference for their child to have real foods that more closely mimic a typical oral diet. Blended tube feedings provide this option and can help improve feeding tolerance and bowel regularity, and decrease feeding aversion, reflux, and vomiting. It is possible that these benefits may help improve oral intake and support successful tube weaning. Our team recently completed the CHAMP for the Feeder study (NCT06052891), which evaluated tube weaning outcomes for children using a remote patient monitoring (RPM) application (CHAMP App®). We conducted secondary data analysis to test the hypothesis that children on blended diets would have improved tube weaning success.

**Methods:** We conducted a single site non‐randomized trial of RPM feeding tube weaning at a mid‐west tertiary care center. Children aged 0‐6 years of age and medically cleared to wean from their feeding tube were recruited through an interdisciplinary feeding clinic. Parents were taught how to use the CHAMP App® to enter weekly weights and daily diet records. Children were prescribed a hunger provocation protocol over 4 weeks. We collected data on medical history, demographics, and diet (blended vs. traditional formula). We defined a “blended diet” as a blend made by the family with consultation from a dietitian or a commercially made blended product. We defined a “formula diet” as a milk‐based, hypoallergenic, dairy free formula, or breastmilk. We measured whether a child weaned to all calories by mouth and the number of days it took to wean. We ran t‐tests and chi‐square tests for group comparisons. We used Cox regression to assess the impact of diet type on number of days it took a child to wean, controlling for % estimated energy requirement (EER) provided by tube at baseline, as this is a known predictor of success.

**Results:** Forty‐six children enrolled from October 2023‐May 2025. Most children (87.0%) successfully tube weaned with an average of 44.25 +/‐ 31.6 days. For the 13% of children who did not ever reach all calories by mouth, they were actively working on tube weaning from 130 +/‐ 69 days (p=.014). 90% of children on blended diets (18/20) and 84.6% on formula (22/26) were able to get to all calories by mouth (p=.591). There were no significant differences in demographics, but at baseline, children on a blended diet were older (44.3 +/‐ 18.8 vs. 26.8 +/‐ 13.8 months, p<.001), had a higher weight for length z‐score (‐.814 + 1.1 vs. ‐.042 + .99, p=.010), had higher % EER (71.9 + 35.9 vs. 51.9 + 30.8, p=.024), and had their tube for longer prior to weaning (35.5 + 17.9 vs. 18.5 + 12.9 months, p<.001). Children on blended diets showed a trend of weaning quicker (46.15 +/‐ 30.0) than children on formula (62.8 +/‐ 57.1; p=.122). The Cox regression model fit was statistically significant (p = .005) and showed that children on a blended diet had a hazard of 2.043 (95% CI [1.02, 4.084], p=.04; see Figure 1).

**Discussion:** When controlling for % EER at baseline, children on a blended diet reached all calories by mouth 200% more quickly than children receiving formula. It took an average of 44 days for children to wean using hunger provocation. Notably, even though children in this study received a higher % EER via tube at baseline, which is typically associated with worse tube weaning outcomes, they still were able to wean much more efficiently than children on formula diets. Blended tube feeding decreases gastrointestinal symptoms. This may lead to improved intake of nutrition by mouth and readiness to tube wean, but more research is needed to determine the causal nature of the findings in this sub‐analysis. Therefore, it is critical to better understand if prescribing blended diets could lead to improved tube weaning success. Future research should also assess caregiver satisfaction and caregiver‐reported challenges and benefits of using blended feedings.


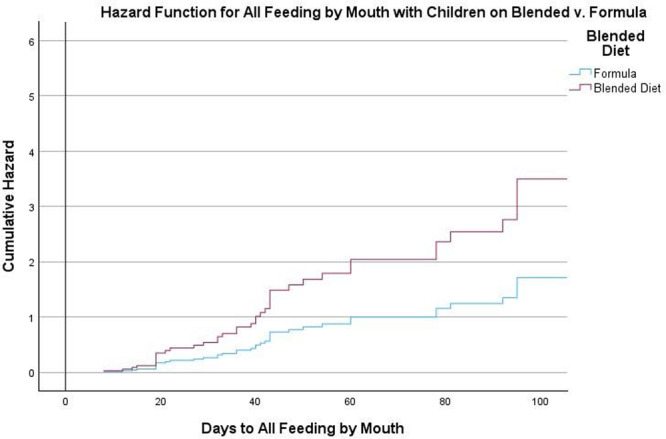


## 8 RADIATION‐ASSOCIATED IMAGING BURDEN IN PATIENTS WITH ESOPHAGEAL ATRESIA AND TRACHEOESOPHAGEAL FISTULA: RESULTS FROM A RETROSPECTIVE STUDY

*Hussain Alturki*, *Muhannad Abu Abthan*, *Nicholas Shkumat*, *Margaret Marcon*, *Priscilla Chiu*, *Theo Moraes*, *Michelle Gould*

*The Hospital for Sick Children*, *Toronto*, *ON*, *Canada*

**Background & Objectives:** Children and adolescents with esophageal atresia (EA) and tracheoesophageal fistula (TEF) require long term follow‐up to monitor for potential co‐morbidities that can occur across the lifespan. Often, imaging studies are used as part of this follow‐up. Previous studies have only assessed radiation burden in the neonatal period and first few years of life for patients with EA/TEF but it remains unclear how many imaging studies these patients undergo throughout childhood. We sought to quantify the number of studies involving ionizing radiation that patients with a history of EA/TEF complete from birth until transition to adult care. We also assessed whether this number has changed over time with further recognition of the negative effects of radiation.

**Methods:** A retrospective review of patients followed at the Hospital for Sick Children for EA/TEF, born between June 1999 and August 2011 was completed. Patients were included if they were followed consistently by at least one specialty involved in the multidisciplinary EA/TEF team (gastroenterology, respirology, and general surgery) from the time of their EA/TEF repair until at least 12 years of age. Abstracted data included patient demographics, type of fistula, gap type (short/long), presence of VACTERL association, and number of imaging scans (x‐ray, fluoroscopic scans and procedures, CT scans, and nuclear imaging). Imaging data for patients born before and after 2005 were compared using an unpaired t‐test.

**Results:** A total of 78 patients were identified who met inclusion criteria with median age of final follow up of 17 years (IQR 14.25‐17). 38 were male (49%), 22 (28%) had VACTERL association and 18 (23%) had long gap EA/TEF. 63 (81%) had a type C TEF. Median imaging studies per patient were: 30.5 x‐rays (IQR 15–58.25), 12 fluoroscopic studies (IQR 6.25–22), 0 CT scans (IQR 0–1.75), and 0 nuclear medicine studies (IQR 0–1). Upper gastrointestinal (UGI) contrast studies comprised 38% of fluoroscopic studies with a median of 5.5 per patient (IQR 3‐9), with each patient undergoing at least one UGI contrast study. Fluoroscopic‐guided esophageal dilation was performed at least once for 31 (40%) patients. For these patients, there was a median of 3 fluoroscopic‐guided dilations per patient (IQR 2‐5). No differences in overall number of studies were found between patients born before or after 2005 (p = 0.32) with the exception of a statistically significant but clinically negligible difference in the number of nuclear medicine studies (p = 0.03), amounting to less than one study on average.

**Conclusion:** Patients with EA/TEF undergo a large number of radiologic scans with associated radiation exposure across their childhood. Considering the potential risks associated with radiation exposure, particularly in infants and children, the development of follow‐up protocols which seek to minimize unnecessary radiation while still effectively monitoring for potential complications and long‐term comorbidities experienced by patients with EA/TEF is necessary.

## 12 MESENTERIC LYMPHATIC MALFORMATION MASQUERADING AS CHYLOUS ASCITES: A DIAGNOSTIC CHALLENGE IN PEDIATRICS

*Maria Altamar* ^*2*^, *Nicolas Mora* ^*2*^, *Adriana Rueda* ^*3*^, *JOSE VERA* ^*1*^, *Angie Vergara Espitia* ^*1*^

^*1*^ *Pediatric Gastroenterology*, *Hospital Universitario de la Fundacion Santa Fe de Bogota*, *Bogotá*, *Bogota*, *Colombia*; ^*2*^ *Pediatric surgery*, *Fundacion Santa Fe de Bogota*, *Bogotá*, *Bogota*, *Colombia*; ^*3*^ *Pathology department*, *Fundacion Santa Fe de Bogota*, *Bogotá*, *Bogota*, *Colombia*

**Introduction:** In the evaluation of intra‐abdominal cystic lesions in pediatrics, mesenteric lymphatic malformations (MLM) must be considered in the differential diagnosis. Formerly known as mesenteric cysts or lymphangiomas, these are rare entities with an incidence ranging from 1 per 20,000 to 250,000 hospital admissions. They represent 5% of all lymphatic malformations and 6% of benign abdominal tumors in childhood. MLMs may be asymptomatic or present with nonspecific symptoms or acute abdomen, depending on their size, location, and complications. Three subtypes are recognized: microcystic, macrocystic, and mixed. Macrocystic lesions can mimic ascites, particularly when containing chylous fluid. Chylous ascites (CA) presents similar clinical and biochemical features, making differential diagnosis essential given their distinct management and prognosis.

**Case Report:** We present a 5‐year‐old previously healthy male with a one‐year history of progressive abdominal distention following minor abdominal trauma. Initial ultrasound suggested an abdominal hematoma, but persistent distention prompted paracentesis, yielding milky fluid. Without immediate analysis, further ultrasound and magnetic resonance imaging (MRI) showed extensive loculated intra‐abdominal fluid displacing adjacent structures. Laboratory tests, including hepatic, renal, tumor markers, and serum proteins, were normal.

A second paracentesis retrieved 2100 mL of chylous fluid, with triglyceride levels of 960 mg/dL, confirming CA. Dietary management with medium‐chain triglyceride supplementation was initiated. Intestinal lymphangiectasia was ruled out by upper endoscopy with histology. Lymphangiography was not performed due to technical limitations. Revised MRI interpretation revealed a large, multiloculated, non‐free fluid collection. Surgical exploration identified a giant mesenteric cystic lesion arising from the ileal mesentery, with firm adhesions and vascular compression. Segmental bowel resection with end‐to‐end anastomosis was performed, draining 600 mL of chylous fluid. Histopathology confirmed a benign cystic lymphatic malformation with xanthogranulomatous inflammation.

**Discussion:** CA typically presents as progressive abdominal distention with few additional symptoms. While imaging is often non‐specific, it aids in characterizing cystic lesions. Definitive diagnosis relies on paracentesis, particularly when triglyceride levels exceed 200 mg/dL. Mesenteric cysts and lymphangiomas are now classified as MLM due to their shared vascular anomaly origin. Most occur in the neck and axilla; only 5% arise in the mesentery. They may remain asymptomatic or cause complications such as hemorrhage, infection, torsion, or rupture. In this case, paracentesis of a giant macrocystic MLM yielded chylous fluid, mimicking CA — a rarely reported diagnostic pitfall.

Definitive treatment is complete surgical excision, especially for macrocystic lesions, to prevent complications. Minimally invasive approaches, such as the single‐incision laparoscopy used here, are safe and effective.

**Conclusion:** This case highlights the diagnostic challenge between CA and MLM, which can share overlapping clinical and biochemical features. Recognizing MLM as a differential diagnosis in suspected CA prevents unnecessary treatments and allows for timely, appropriate surgical management, reducing associated morbidity.

## 13 DEMOGRAPHIC DISTRIBUTION OF ESOPHAGOGASTRODUODENOSCOPY IN EOSINOPHILIC ESOPHAGITIS BEFORE AND AFTER THE COVID PANDEMIC

*Charlotte Banayan* ^*3*^, *Klaudia Cios* ^*2*^, *Maile Ray* ^*1*^, *Shahzaib Khan* ^*3*^, *Ashley Shayya* ^*1*^, *Sandra McGinnis* ^*1*^, *Thomas Wallach* ^*3*^

^*1*^ *University at Albany*, *Albany*, *NY*; ^*2*^ *Maimonides Medical Center*, *New York*, *NY*; ^*3*^ *SUNY Downstate Health Sciences University*, *New York*, *NY*

**INTRO:** Esophagogastroduodenoscopy (EGD) is essential for the diagnosis and monitoring of eosinophilic esophagitis (EOE). There is limited literature illustrating the volume of EGD and how it varies by sex, ethnicity, and race. We previously demonstrated significant disparities in the distribution of endoscopic evaluation by race before and after the COVID pandemic. In this study, we seek to assess what variation, if any, exists in the distribution of EGD by sex, ethnicity, and race prior to the COVID pandemic (2019) and after the pandemic (2024) in pediatrics.

**METHODS:** We used the TriNetX database, which consists of electronic medical records from >120 million patients across >50 healthcare organizations, to identify EGD utilization among patients with EOE less than or equal to 22 years of age before (2019) and after (2024) the COVID pandemic. We repeated this for patients with EOE who received EGD within the same timeframe. We utilized Chi‐square analysis with a p‐value <0.05 to determine statistical significance.

Demographic data was available for sex, ethnicity, and race. Sex categories were male or female. Ethnicity categories were Hispanic or Latino (HL), Not Hispanic or Latino, and unknown ethnicity (UE). Racial subgroups included White, Black or African American (BAA), unknown race (UR), other race (OR), Asian, American Indian or Alaska Native (AIAN), and Native Hawaiian or Other Pacific Islander (NHOPI).

**RESULTS:** Prior to the pandemic in 2019, we identified 9,286 patients with EOE. Of those patients, 4,074 (43.9% of total EOE) also received EGD. After the pandemic in 2024, we identified 14,355 patients with EOE. Of those patients, 5,784 (40.3% of total EOE) also received EGD. The table provides percentage breakdown of sex, ethnicity, and race for EOE, EGD, and EOE & EGD.

Among all patients in both 2019 and 2024, there is a significant difference among patients who received an EGD to those that did not by ethnicity (p<0.001) and race (p<0.001). Specifically, we note improvement in procedure difference by race, with less alteration to representation of persons of color (POC), although worsening in disparity by ethnicity, with non‐hispanic patients becoming more overrepresented. Overall, demographics of all patients aged 0 to 22 years with EOE in 2019 compared to 2024 differed significantly by ethnicity (p<0.001) and race (p<0.001). There is a significant difference in distribution of EGD in 2019 versus 2024 by ethnicity (p=0.015) and race (p<0.001).

**DISCUSSION:** Among patients with EOE in 2019 and 2024, there are ethnic and racial differences in those who received an EGD in 2019 and 2024 separately, which have evolved between 2019 and 2024.

Pre‐ and post‐pandemic, we observed an overrepresentation of EGD in White and NHOPI patients and an underrepresentation of EGD in BAA and AIAN patients. However, the overrepresentation of the White population receiving EGD in 2024 decreased. While an improvement relative to 2019, there remains a significant difference between racial distribution of EGD in EOE patients in 2024, although it is a small functional difference.

After the COVID pandemic, we observed an increase in the number of pediatric patients with EOE, though a smaller percentage of them underwent EGD. Our findings suggest that previously documented alterations in practice associated with race are not as substantial in the EOE disease state, and that overall rates of endoscopy in EoE are declining relative to population.


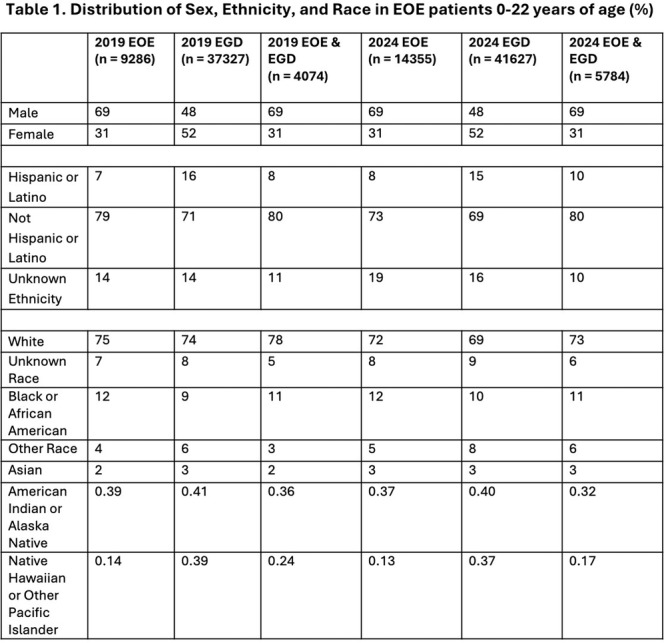


## 25* COMPARISON OF PROACTIVE VS. REACTIVE ENDOSCOPIC APPROACHES FOR SURVEILLANCE IN PATIENTS POST‐ESOPHAGEAL ATRESIA REPAIR

*Catherine Sanon* ^*2*^, *Jose Frias Mantilla* ^*1,3*^, *Aridj Bouragbi* ^*4*^, *Helene Bacha* ^*3*^, *Ana Sant'Anna* ^*1,3*^

^*1*^ *Pediatrics*, *McGill University*, *Montreal*, *QC*, *Canada*; ^*2*^ *Pediatrics*, *Dalhousie University*, *Halifax*, *NS*, *Canada*; ^*3*^ *Montreal Children's Hospital*, *Montreal*, *QC*, *Canada*; ^*4*^ *McGill University*, *Montreal*, *QC*, *Canada*

**Objectives and Study:** Esophageal atresia (EA) is a common congenital malformation, affecting up to 1 in 2,500 live births. Advances in surgical techniques have led to success rates of 90–100%. However, post‐surgical management remains challenging, particularly in addressing complications such as anastomotic strictures, which are commonly treated with endoscopic balloon dilation. The utility of routine endoscopic surveillance and dilation compared to selective endoscopy for symptomatic patients remains unclear. This study aims to evaluate the outcomes of these two approaches in our patient population.

**Methods:** We analyzed two groups of patients followed for up to 36 months post‐EA repair (n=24). The *proactive group* (n=13) underwent routine and symptom‐driven endoscopic surveillance from February 2016 to February 2019, with dilations performed as needed. The *reactive group* (n=11), managed from February 2020 to February 2023, received selective endoscopies only when symptomatic. Both groups underwent an initial endoscopic evaluation within 0–24 months post‐repair. Follow‐up strategies were determined based on the year of diagnosis. Variables in both groups such as gestational age, type of EA, age at EA repair, length of gap between the two esophageal segments, age at first endoscopy, symptoms pre‐ and post‐EA repair, presence of GERD, treatment with PPIs, number of endoscopies, presence of strictures, number of endoscopies and dilations were described.

**Results:** In the proactive group, 78 endoscopies were performed, of which 59 required dilations (75.64%). In the reactive group, 47 symptom‐driven endoscopies were conducted, with 46 requiring dilations (97.87%). The mean number of endoscopic interventions per patient was 6 (range: 2–10) in the proactive group and 4.27 (range: 0–9) in the reactive group, with similar mean numbers of dilations per patient: 4.53 (range: 0–10) in the proactive group and 4.18 (range: 0–9) in the reactive group. The odds ratio (OR) for comparing the effectiveness of endoscopies in detecting patients requiring dilations between the reactive and proactive groups was approximately 14.81. This difference reflects the symptom‐driven nature of the reactive group's procedures, which inherently increased the likelihood of requiring dilations compared to the routine approach in the proactive group. In the proactive group, two patients underwent 2 and 3 routine endoscopies, respectively, without requiring dilations. In the reactive group, one patient did not require a second endoscopy as they remained asymptomatic throughout the study period, while another underwent a single endoscopy due to symptoms but did not require dilations.

In the proactive group, most dilations (28, or 43.75%) were performed between 12 and 24 months, whereas in the reactive group, the majority (18, or 40.90%) occurred between 6 and 12 months.

Cough and dysphagia were the most common symptoms prompting endoscopy in both groups. There were two complications (leakages) in the proactive group and none in the reactive group. No dysplasia was detected in either group.

**Conclusions:** The reactive approach reduced the number of endoscopic procedures needed in our population. Patients who had a reactive follow‐up were more likely to require dilation therapy. This small study suggests that a reactive strategy can decrease the frequency of anesthetic exposure, risk and procedural costs by reducing the number of endoscopic events in patients who do not require dilatation.


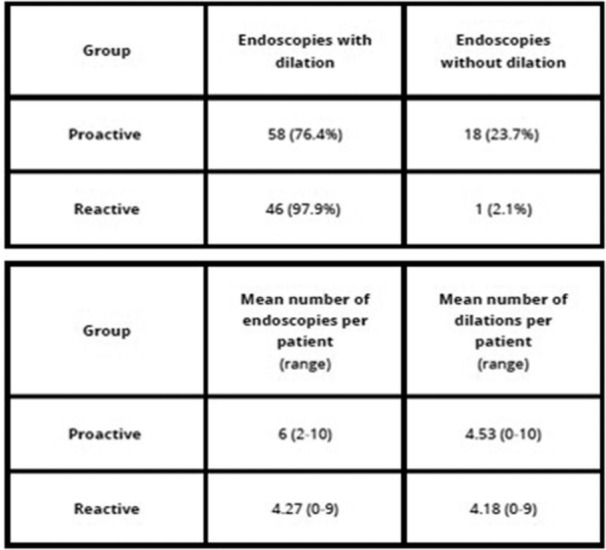


## 26* USING POINT‐OF‐CARE GASTRIC ULTRASOUND TO INFORM PRE‐ENDOSCOPY FASTING GUIDELINES IN POST‐PYLORICALLY TUBE FED CHILDREN: A PROSPECTIVE OBSERVATIONAL STUDY

*Stephanie Hum* ^*1*^, *Elaina Lin* ^*2,3*^, *Michael Manfredi* ^*1,3*^

^*1*^ *The Children's Hospital of Philadelphia Division of Gastroenterology Hepatology and Nutrition*, *Philadelphia*, *PA*; ^*2*^ *The Children's Hospital of Philadelphia Department of Anesthesiology and Critical Care Medicine*, *Philadelphia*, *PA*; ^*3*^ *University of Pennsylvania Perelman School of Medicine*, *Philadelphia*, *PA*

**BACKGROUND:** The optimal fasting time prior to endoscopy in post‐pylorically tube fed children is unclear. Current guidelines are often extrapolated from those of children who are formula fed by mouth. Because post‐pyloric feeds bypass the stomach, they may be associated with decreased gastric contents and thus a lower risk of aspiration. Applying standard fasting times to this population may unnecessarily prolong fasting, leading to increased discomfort, hypoglycemia, and worse nutritional status without improving patient safety. Alternatively, shortening fasting times in this population without sufficient evidence of decreased gastric contents may increase the risk of aspiration.

**OBJECTIVE:** To use point‐of‐care ultrasound to evaluate the gastric contents of post‐pylorically tube fed children at different fasting intervals in order to inform pre‐endoscopy fasting guidelines.

**METHODS:** We conducted a prospective observational study of post‐pylorically tube fed children under 18 years of age admitted to a large tertiary pediatric hospital. Patients were excluded if they had oral solid intake within 8 hours or gastric feeds within 4 hours of the ultrasound. Point‐of‐care ultrasound was performed by a trained investigator in the right lateral decubitus position in order to visualize the gastric antrum. Then, another trained investigator, who was blinded to fasting time, qualitatively assessed the gastric contents as solid appearing (solids and formula), clear liquid, or empty (Figure 1). Solid appearing contents of any volume and clear liquid contents ≥1.25 mL/kg were considered high risk for aspiration. Empty and clear liquid contents <1.25 mL/kg were considered low risk for aspiration. The total gastric volume of clear liquids was estimated using a published pediatric predictive model. Detailed feeding and clinical history were obtained at the time of the ultrasound. The study protocol was approved by our institutional review board.

**RESULTS:** A total of 13 patients ages 2 months old to 17 years old were included at fasting intervals of 0‐6 hours (Table 1). 8 patients had gastrojejunal tubes and 5 had nasojejunal tubes. Overall, 54% (7/13) of patients were on pro‐motility medications and 23% (3/13) were on opioids that could slow gastric motility; no patients had a formal diagnosis of gastroparesis. Solid appearing gastric contents were visualized in 100% of patients (n=5) at 0 hours, 50% of patients (n=2) at 1 hour, 33% of patients (n=3) at 2 hours, 100% of patients (n=2) at 4 hours and 0% of patients (n=1) at 6 hours (Table 1). One patient who fasted for 2 hours had clear liquid contents seen in the stomach, but the total gastric volume was considered to be low risk for aspiration (0.3 mL/kg).

**CONCLUSIONS:** Solid appearing gastric contents were frequently present in post‐pylorically tube fed children after fasting for up to four hours, even with venting and the use of pro‐motility agents. These findings may suggest that there is retrograde reflux of post‐pyloric feeds back into the stomach, which may cause gastric contents to persist longer than expected in this population. Underlying dysmotility in patients who are post‐pylorically tube fed may also play a role. Thus, until larger studies are performed, conservative fasting guidelines prior to endoscopy may be warranted in post‐pylorically tube fed children in order to minimize the risk of aspiration.


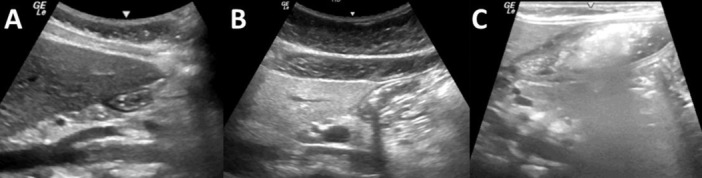


Figure 1. Representative images of point‐of‐care gastric ultrasound findings (A) empty, Patient 13 (B) clear liquid, Patient 9 (C) solid‐appearing, Patient 3


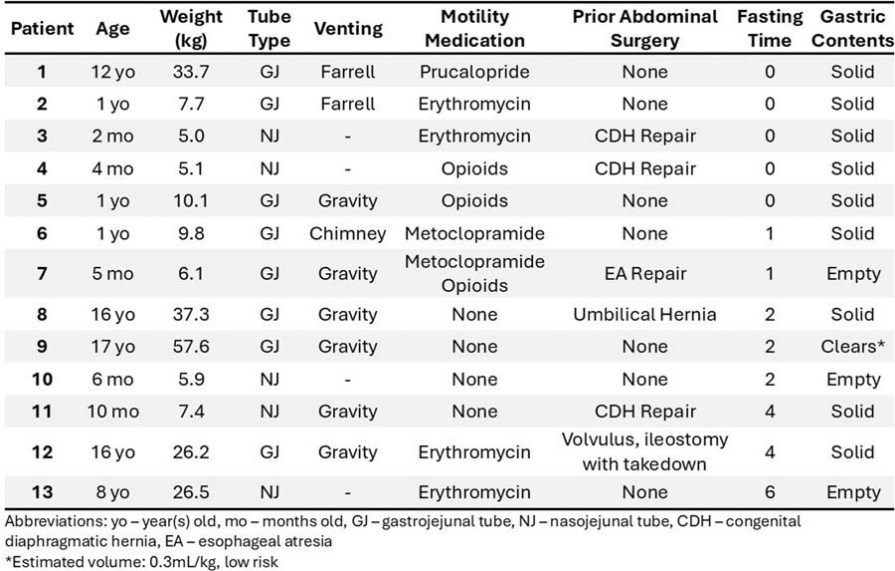


Table 1. Patient characteristics and point‐of‐care gastric ultrasound results at different fasting intervals

## 31 ARGON PLASMA COAGULATION TREATMENT FOR GASTRIC INLET PATCH IN SYMPTOMATIC CHILDREN: A RETROSPECTIVE CHART REVIEW

*Sara Alremawi* ^*1,2*^, *Erini Gamal Nessim Kostandy* ^*1*^, *Ajay Kaul* ^*1*^

^*1*^ *Gastroenterology*, *Cincinnati Children's Hospital Medical Center*, *Cincinnati*, *OH*; ^*2*^ *Department of Pediatric, Faculty of Medicine*, *The Hashemite University*, *Az‐Zarqa*, *Zarqa Governorate*, *Jordan*

**Introduction:** Gastric inlet patch (GIP) is a heterotopic gastric mucosal lesion in the proximal esophagus that can be asymptomatic or associated with various gastrointestinal and laryngopharyngeal symptoms. In rare cases, GIPs can lead to complications such as bleeding, ulceration, strictures, and even neoplastic transformation. The prevalence of a GIP in pediatric patients is reported to be between 1.4% and 6.3% based on endoscopic studies.

The clinical management of symptomatic GIPs includes proton pump inhibitors (PPIs) to reduce acid production. In cases where medical therapy is insufficient, endoscopic ablation of heterotopic gastric tissue may be performed using techniques such as argon plasma coagulation (APC). The American College of Gastroenterology and the American Society for Gastrointestinal Endoscopy recognize APC as an established modality for superficial mucosal ablation in the gastrointestinal tract in adults. This procedure involves non‐contact, endoscopic ablation in which ionized argon gas conducts monopolar electrical current to the tissue which coagulates the mucosa, typically performed under general anesthesia. Depending on the size of the GIP, multiple sessions may be required.

**Aim:** To evaluate the outcomes of APC treatment in symptomatic PPI‐resistant GIP.

**Method:** We retrospectively reviewed EMR of patients with histologically confirmed, PPI‐nonresponsive GIP who had received APC from April 2017‐ April 2025. We collected demographic and endoscopic details and outcomes in these children.

All APCs were performed under general anesthesia while the patients were intubated. Single‐use flexible monopolar APC probes with axial and radial tips were used with APC settings ranging from 40–60 W and gas flow rates of 1–2 L/min. The probe was held a few millimeters from the mucosal surface and activated for 1‐2 second pulses to achieve effective ablation while minimizing the risk of deep tissue injury. In cases with large, circumferential GIP, piecemeal ablation was performed over multiple sessions.

**Results:** A total of 17 symptomatic patients with GIP, despite a minimum of 4 weeks PPI therapy, were included (12 males, 5 females; median age 15 years).

Dysphagia was the most common symptom noted in 76%, while nausea, vomiting and retching were noted in 53% of cases. Other documented symptoms included heartburn, regurgitation, chest pain, chronic sore throat, globus sensation and weight loss.

Of the 17 patients, 15 (88%) had one or more prior endoscopy where GIP was not noted (ranging from 1‐5).

All GIPs were biopsied, and the presence of gastric epithelium confirmed in all with no histological evidence of intestinal metaplasia or dysplasia.

Most patients (76%) had one patch, while three had two patches, and one patient had three.

While 41% of patients received a single APC session, 59% received more than one session (ranging from 2‐6 sessions), mostly due to incomplete ablation in one session. Median duration between sessions was 3 months, (range 3 weeks to 17 months). One patient had complicated GIP with proximal esophageal stricture that required frequent dilatations.

Associated esophageal conditions included Eosinophilic Esophagitis noted in 29%, and 18% of the cases had distal (peptic) esophagitis. Additionally, there were single cases each of hiatal hernia, retrograde cricopharyngeal dysfunction, rumination and globus hystericus.

On post‐ablation, follow up endoscopy in 7 of the 17 patients (41%), all showed complete resolution of the heterotopic gastric tissue.

No serious post‐APC complications were reported in any patient.

**Outcome:** On follow up, 4 out of 17 patients (24%) had complete resolution of symptoms, 9 (53%) had at least 50% improvement, two patients had no changes in symptoms, and two were unsure. No patients experienced worsening symptoms.

**Conclusion:** APC is a safe and effective treatment modality in most symptomatic pediatric patients with PPI‐unresponsive GIP.

## 32 THE ENVIRONMENTAL IMPACT OF PEDIATRIC ENDOSCOPY: SMALL FEET HAVE A DISPROPORTIONATELY LARGE CARBON FOOTPRINT

*Monique Barakat* ^*1,2*^, *Praveen Kalra* ^*3*^, *Megan Quinn* ^*2,3*^, *Roberto Gugig* ^*2*^

^*1*^ *Pediatrics & Medicine*, *Stanford University School of Medicine*, *Stanford*, *CA*; ^*2*^ *Lucile Salter Packard Children's Hospital at Stanford*, *Palo Alto*, *CA*; ^*3*^ *Anesthesia*, *Stanford University School of Medicine*, *Stanford*, *CA*

**Introduction:** Endoscopy has a significant environmental impact, attributable to high procedure volume, energy‐intensive disinfection processes and abundant single‐use items. Studies evaluating the carbon footprint of adult endoscopy have demonstrated that 2.1 kg of disposable waste is produced during each procedure. We evaluate, for the first time, the carbon footprint of pediatric endoscopy.

**Methods:** We conducted a 2‐week audit of disposable waste produced in our pediatric endoscopy room, which is located within the operating room. Weeks evaluated capture busy and less busy weeks (one week in early August, one week in February) to maximize generalizability and minimize confounding. Waste generated from combined procedures was analyzed as a single data point. We used these data, coupled with established and institutional and device/product energy usage data to estimate the carbon footprint of pediatric endoscopy at our institution.

**Results:** 98 procedures performed on 71 patients were analyzed (62 procedures in Week 1, 36 in Week 2). 27 were combined EGD/Colonoscopy or Enteroscopy/Colonoscopy procedures. A mean of 3.48 ± 0.57 kg of disposable waste was generated per pediatric endoscopic procedure. This is 65.7% higher than the 2.1 kg of disposable waste produced during each adult endoscopic procedure. Among pediatric endoscopy procedures, ERCPs generated significantly more waste than other procedures (4.3 ± 0.41 kg, Figure 1) Combined procedures did not generate significantly more disposable waste compared with independent procedures (3.6 ± 0.41 vs. 3.2 ± 0.44). The amount of waste generated per procedure did not vary significantly between the high and low volume week evaluated.

In addition to the volume of disposable waste, 20% of what was disposed of in hazardous waste receptacles could have been disposed of in the trash and 32% of disposable waste in the trash bin was marked to indicate it was recyclable.

Total disposable waste was 1,484.4 kg for these two weeks. When the carbon impact of production and disposal of this waste is taken into account, this disposable pediatric endoscopy waste corresponds with 3.71 metric tons of CO_2_ over the study period and an estimated 96.51 metric tons of CO_2_ in one year,

In addition to disposable waste, taking into account high level disinfection and endoscope cleaning, air circulation, lights, cooling/heating, and anesthesia/endoscopy machines, we estimated the energy required to power endoscopy was 128.5 kWH, which corresponds with 90.85 lb CO_2_ daily and 11.81 metric tons of CO_2_ each year.

Disposable waste and energy for power results a carbon footprint of 108.32 metric tons of CO_2_ each year from our pediatric endoscopy center alone (Figure 2).

**Conclusions:** The environmental impact of pediatric endoscopy is high—even higher than that of adult endoscopy. There are several potential reasons for this, the most significant of which is that pediatric endoscopy procedures at our institution and many institutions are performed in the operating room rather than in an independent endoscopy unit. Air circulation and cleaning guidelines are typically more stringent and energy intensive in the sterile operating room environment. There are relatively straightforward opportunities for improvement: First, combining EGD and colonoscopy procedures is energy efficient compared with performing these procedures independently. Second, up to 40% of waste generated in U.S. endoscopy units may be recyclable or reusable, and hazardous waste disposal, which requires incineration and produces harmful toxins and greenhouse gases, is overused for endoscopy associated waste. Additional opportunities for improvement exist and must be considered to reduce the ecological footprint and achieve more sustainable pediatric endoscopy.


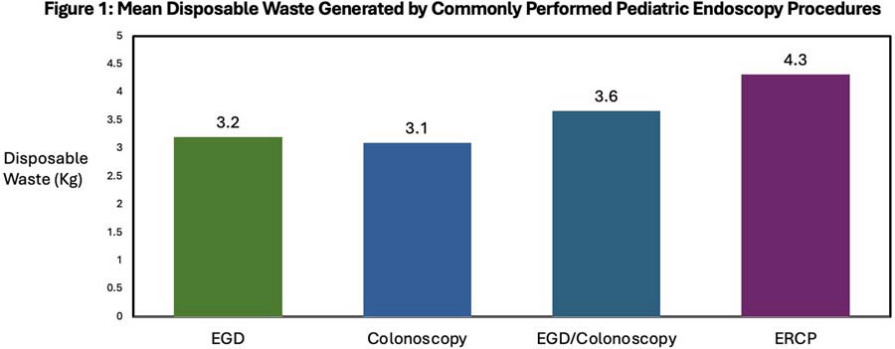



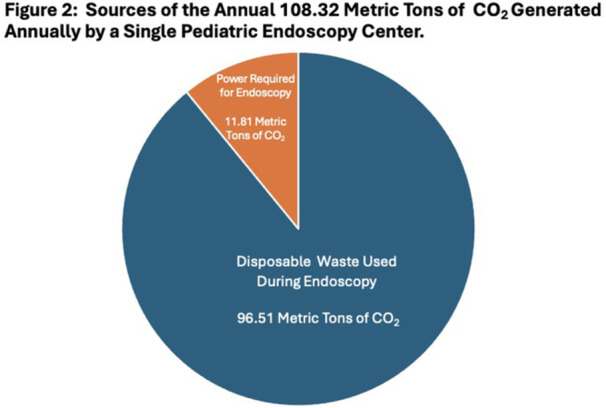


## 38 VIDEO CAPSULE ENDOSCOPY FINDINGS IN CHILDREN WITH PTEN HAMARTOMA SYNDROME: A RETROSPECTIVE COHORT STUDY

*HyeIn Cha* ^*2*^, *Hunter Friesen* ^*3*^, *Thomas Attard* ^*1*^

^*1*^ *Gastroenterology*, *Children's Mercy Kansas City*, *Kansas City*, *MO*; ^*2*^ *Kansas City University*, *Kansas City*, *MO*; ^*3*^ *Pediatrics*, *Children's Mercy Kansas City*, *Kansas City*, *MO*

**Background:** PTEN hamartoma tumor syndrome (PHTS) is associated with multisystem hamartomas including gastrointestinal (GI) polyps. While upper and lower GI manifestations are well described, small‐intestinal involvement in pediatric patients has not been systematically characterized. Video capsule endoscopy (VCE) enables non‐invasive small bowel imaging and may reveal subclinical pathology. This study aimed to characterize polyp burden, distribution, mucosal findings, and associated laboratory abnormalities in children with PHTS; as well as evaluate the **diagnostic utility of video capsule endoscopy** (VCE) in this population.

**Methods:** Patients (<21 years of age) with PHTS who were treated at our center were identified from our dedicated clinical registry and were eligible for inclusion if they underwent VCE between 2014 and 2024. Polyp and mucosal findings were extracted using a two‐step approach. First, narrative VCE reports were reviewed using a rule‐based natural language processing (NLP) strategy that identified predefined keywords (e.g., “inflammation,” “ulcer,” “erosion,” “exudate,” and descriptive polyp features. In parallel, all VCE videos were reviewed by two investigators to directly assess polyp number, size, distribution, and mucosal abnormalities. In cases of disagreement, final determinations were adjudicated by the senior author. Clinical symptoms and laboratory values (hemoglobin, serum total protein) were also abstracted and matched to each VCE study (median interval: 5 months).

**Results:** Our registry included 16 patients clinically diagnosed or suspected with PTEN‐HTS, and our cohort included 9 children and 21 VCE studies; 5 were female and the mean (SD) age at VCE was 15.4 (5.5) years. One patient carried a combined PTEN + BMPR1A deletion; the remainder had isolated PTEN pathogenic variants. Most studies (95.5%) demonstrated at least one abnormal finding, based on either structured documentation of polyps or mucosal abnormalities described in narrative reports. One study was incomplete, and capsule retention did not occur.

Small‐intestinal polyps were identified in 20 VCEs (95%), with counts ranging from 1 to 250 (median 70). There was a non‐significant trend (p = 0.069) toward different polyp counts across the three tertiles, with the highest median polyp count in the 1st tertile. In 3 of 4 patients undergoing repeated studies, an increase in total polyp count over time was observed. Median (range) polyp size was 6 (2 – 25) mm (Table 1). Differences across tertiles were not statistically significant, reflecting a fairly uniform size distribution regardless of location.

In addition, 8 of 22 studies (36%) demonstrated non‐polyp mucosal abnormalities, including inflammation (n=8), ulcers (5), exudate (3), and erosions (2). Overall mean Small Intestine video capsule transit time was 277 mins; however, patients with endoscopically placed capsules had a longer mean transit time of 303 minutes, which was consistent with published normative values.

**Clinical symptoms** at the time of VCE were inconsistent with findings: abdominal pain (7 studies) and abdominal distension/bloating (6) were the most common, while 5 studies (23%) were performed in asymptomatic patients; other reported symptoms included GI bleeding (4), constipation (3), nausea/vomiting (3), and anemia (2).

**Laboratory abnormalities** were frequent. Mean hemoglobin was 11.5 g/dL (range 7.9–14.5), with 50% of patients exhibiting usually mild, microcytic hypochromic anemia. Serum total protein ranged from 5.0 to 7.9 g/dL.

The child with a combined PTEN + BMPR1A deletion had the most severe clinical and endoscopic phenotype, including extensive duodenojejunal polyposis, gastrointestinal bleeding and anemia.

**Conclusions:** VCE yielded clinically relevant findings in the majority (9/16) of pediatric patients with PHTS. Children with PHTS have a high prevalence of diffuse but predominantly proximal small‐intestinal polyposis, often with nonspecific inflammatory mucosal abnormalities, detectable by VCE. These findings may contribute to anemia and protein loss. While polyp burden is greater than in other polyposis, polyp size rarely exceeds intussusception thresholds. VCE in this population is safe and allows for surveillance of polyp burden and detection of mucosal abnormalities. These abnormalities are amenable to enteroscopy for further diagnostic work‐up or therapy. Further research is needed to define optimal timing and frequency of small bowel surveillance in this population.


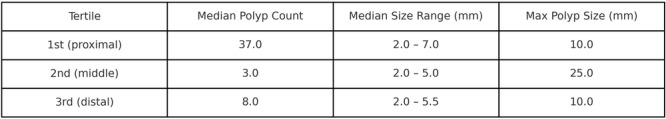


Table1. Small Intestinal Polyp Burden by Tertile in Pediatric PTEN

## 45 ASSOCIATION OF ESOPHAGOGASTRIC JUNCTION DISTENSIBILITY WITH GASTROESOPHAGEAL REFLUX DISEASE IN CHILDREN

*Sharef Al‐Mulaabed*, *Hiba Hajissa*, *Devendra Mehta*, *Yamen Smadi*, *Nishant Patel*

*Center for Digestive Health and Nutrition*, *Orlando Health Arnold Palmer Hospital for Children*, *Orlando*, *FL*

**BACKGROUND:** Increased esophagogastric junction (EGJ) relaxation is the most important pathophysiology of gastroesophageal reflux disease (GERD). Endoscopy, high‐resolution manometry, and 24‐hour pH‐ impedance all have limitations when it comes to evaluating the function of the EGJ. Endoluminal Functional Luminal Impedance Probe (EndoFLIP) has been established to objectively evaluate mechanical properties and distensibility at the EGJ. Previous reports demonstrated controversies surrounding the usefulness of EndoFLIP in GERD patients, with none of them included pediatric age group. In addition, those studies did not correlate EndoFLIP parameters with specific pH‐impedance study findings.

**AIMS:** We aim to examine the EndoFLIP parameters, including distensibility index (DI), maximum diameter (MD), and contractility pattern in patients with or without GERD. In addition, we aim to study the correlation between EndoFLIP parameters and impedance findings.

**METHODS:** A retrospective analysis of subjects (aged 1‐22 years) who completed EndoFLIP with a 24‐hour pH impedance study at the time of evaluation, between April 2021 and November 2024, at Arnold Palmer Hospital for Children, Orlando, FL. Patients with other diagnoses or comorbidities such as eosinophilic esophagitis, esophageal atresia, or achalasia were excluded. Diagnosis of GERD was based on elevated acid reflux index, DeMeester/Boix‐Ochoa scores, or increased 24‐hour total refluxes (according to the 95th percentile published values). Controls were patients with normal endoscopy, EndoFLIP, 24‐hour pH impedance, and esophageal biopsies.

**RESULTS:** Eighty‐four subjects were included in the study, mean (±SD) age 10.4 (±4.5) years, 37 (44%) males. Out of those 84, 46 (55%) were diagnosed as GERD, while 38 (45%) were controls. There was no significant difference in age and gender (p>0.05 in both), table 1. DI was significantly higher in GERD group (5.9 ± 2.6) compared to controls (4.2 ± 2), p=0.001. Both groups had comparable contractility pattern, with no significant difference in prevalence of diminished or absent contractility.

There was a significant correlation between DI and number of 24‐hour refluxes (r=0.339, p=0.003), figure 1. Area under the curve analysis for DI and GERD revealed a result of 0.708 (standard error of 0.058), which was statistically significant (p=0.005), indicating that DI performs moderately well in distinguishing between GERD and no GERD in the studied cohort. A distensibility cut‐off value of 5.35 (mm^2^/mmHg) resulted in a sensitivity of 58% and specificity of 82% in diagnosing GERD.

**CONCLUSIONS:** Evaluating distensibility at EGJ may serve as an additional tool to diagnose GERD in patients undergoing Endoscopic evaluation, and may facilitate defining reflux severity in those patients, therefore aiding in future management and therapy.


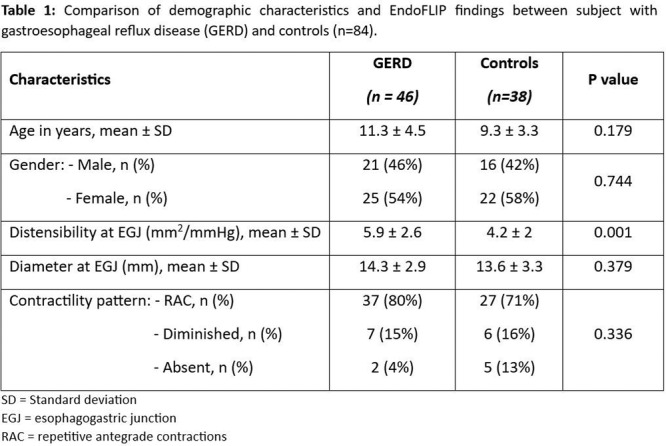



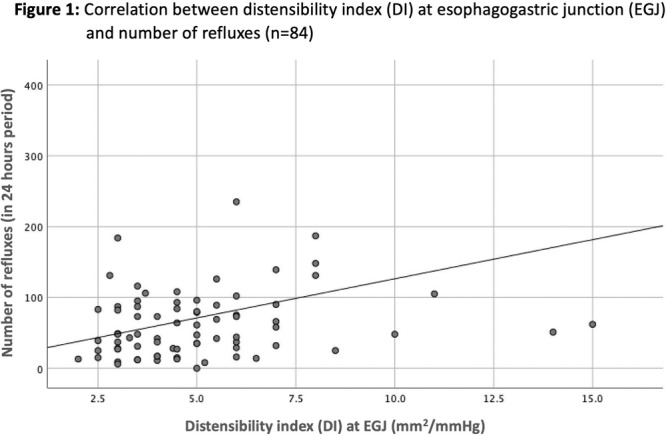


## 46 UNSEDATED TRANSNASAL ESOPHAGOGASTRODUODENOSCOPY FOR EVALUATION OF PEDIATRIC CELIAC DISEASE, A SINGLE CENTER PILOT EXPERIENCE

*Julia Sessions*, *Michael Joseph*, *Marisa Stahl*, *Edwin Liu*, *Nathalie Nguyen*, *Jacob Mark*

*Pediatric Gastroenterology/Digestive Health Institute*, *Children's Hospital Colorado*, *Aurora*, *CO*

**Background:** Adequate duodenal mucosal biopsies are critical for accurate diagnosis of celiac disease and are generally obtained through standard esophagogastro‐duodenoscopy (EGD) involving procedural anesthesia. Sedation‐free transnasal esophagoscopy (TNE) using topical anesthetic has been successful for evaluation of pediatric esophageal diseases, particularly in eosinophilic esophagitis (EoE). Here, we evaluated unsedated transnasal esophagogastroduodenoscopy (TN‐EGD) with topical anesthetic for duodenal tissue assessment in children with suspected or known celiac disease.

**Methods:** We performed a retrospective review of pediatric patients who underwent TN‐EGD for diagnosis or surveillance of celiac disease at a tertiary children's hospital from 2024 ‐ 2025. TN‐EGD was done in an outpatient clinic room designated for procedures. Patients were provided distraction using an Oculus virtual reality system (Reality Labs, Menlo Park, California USA). Aerosolized lidocaine spray was applied to the nares and oropharynx. TN‐EGD was performed by 1 of 2 gastroenterologists proficient in TN‐EGD. A 110 cm long, 3.5 mm outer diameter disposable gastroscope (EvoEndo Centennial, CO USA) was used along with 2 mm biopsy forceps to obtain biopsies. Patient and procedural variables were collected from the electronic medical record. Patient tolerance was quantified using the TNEase scoring system (1 = performed on patient with ease, 2 = performed with minimal complaints, 3 = performed with moderate complaints, 4 = performed but patient had significant complaints and resistance, 5 = patient did not tolerate, and procedure was terminated). Adverse events (AE) were assessed by using a prospective AE monitoring system which tracks all post‐procedural complaints within 72 hours of any endoscopic procedure. This study was approved by the institutional review board.

**Results:** Eleven patients underwent TN‐EGD for celiac disease evaluation (Table 1) from January 2024 ‐ April 2025 with an overall completion rate of 81% (9/11). The first two TN‐EGDs were unsuccessful. The first patient had a large amount of retained food in their stomach obscuring the pylorus which could not be traversed. The second patient was 8 years old and did not tolerate the procedure and asked to stop. After lengthening the nil per os (NPO) time and raising the minimum age to 12 years, the subsequent 9 procedures were successful. Indication for TN‐EGD was celiac disease diagnostic evaluation in 64% (7/11) and the remainder were for celiac disease surveillance for patients for whom assessment of mucosal inflammation was beneficial for clinical care. All biopsies were considered adequate for proper assessment for celiac disease. The average visit duration was 89 ± 25 minutes.

There were no intra‐ or post‐procedure AEs. Complications included one patient who vomited during the procedure and another procedure that had an endoscope malfunction requiring a replacement and repeat esophageal intubation; both patients were able to complete the exams successfully.

**Conclusions:** This study demonstrates the feasibility of TN‐EGD for the assessment of celiac disease in select adolescents. Optimizing patient selection and NPO times may facilitate successful procedures.


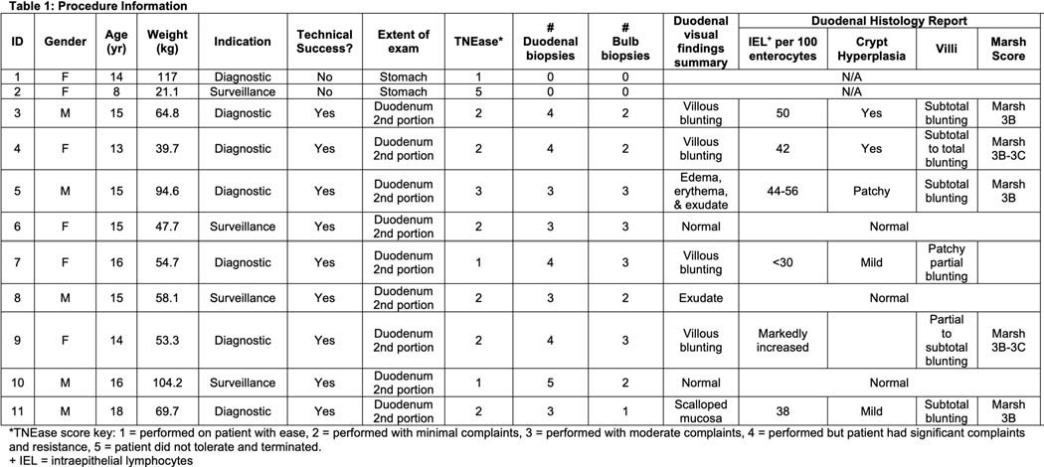


## 47 EXPANDING ROLE OF EUS IN PEDIATRIC CARE: RETROSPECTIVE REVIEW OF A SINGLE‐CENTER 5‐YEAR EXPERIENCE

*Daniel Reiss* ^*1,2*^, *Jenifer Lightdale* ^*1,2*^, *Amit Grover* ^*1,2*^

^*1*^ *Gastroenterology*, *Boston Children's Hospital*, *Boston*, *MA*; ^*2*^ *Harvard Medical School*, *Boston*, *MA*

**Background:** Indications for endoscopic ultrasound (EUS) in pediatric practice continue to evolve. Recent studies suggest that EUS is safe, technically successful, and clinically useful in a variety of pediatric conditions. The aim of this study is to chronicle the use of upper gastrointestinal (GI) tract EUS at a large, tertiary‐care, freestanding children's hospital to better understand evolving trends in utilization and indications.

**Methods:** IRB approval was granted to perform a single‐center retrospective descriptive study of all EUS procedures performed at Boston Children's Hospital from January 2020 through May 2025. Patient characteristics and procedure details, including disposition (hospitalized vs. outpatient), indication, anatomic focus, and complications, as well as tissue acquisition and technical success, were obtained through chart review. Descriptive statistics and trend analyses were performed for both parametric and nonparametric data.

**Results:** One‐hundred‐ten consecutive unique EUS procedures were performed in 101 patients during the study period. The median patient age was 14.2 years (IQR: 10.5, 18.5), with the youngest patient being 1.2 years old. Patients’ body weight ranged from 14.1 to 189 kg, median 50.5 kg (IQR: 30.1, 68). 57% (n=63) occurred as ambulatory procedures, with the remainder performed in hospitalized patients. The volume of EUS procedures performed per year increased from 8 in 2020 to a median of 25 between 2022‐2024. The number of indications similarly increased from 4 in 2020 to a median of 8 between 2022‐2024 (see Figure 1). EUS was performed for a total of 13 different indications during the study period, including for evaluation of a mass of the GI tract or surrounding structures (n=41 (37%)); chronic pancreatitis or abnormal pancreatic duct imaging (27 (24%)); and therapeutic drainage of peri‐pancreatic fluid collections via cystgastrostomy (18 (16%)). Forty (36%) EUS procedures involved attempted tissue acquisition, of which 35/40 (88%) yielded samples adequate for diagnosis. Beginning in 2023, 9 EUS‐guided liver biopsies (EUS‐LB) were performed, of which 7 (78%) yielded adequate tissue samples. Out of the 110 EUS procedures, a total of 3 post‐procedural complications (2.7%) were identified. All 3 followed EUS‐guided cystgastrostomy and included bleeding requiring repeat upper endoscopy (n=1), aspiration pneumonia (1), and ICU admission for post‐procedural fever (1).

**Conclusions:** At our institution EUS is being increasingly performed in children for a wide range of diagnostic and therapeutic indications. Our review found a higher proportion of EUS procedures performed for evaluation of masses in children than has previously been described, as well as a very recent increase in the use of EUS‐LB. In concordance with other published case series, we found EUS in children to be a safe and clinically useful procedural modality.


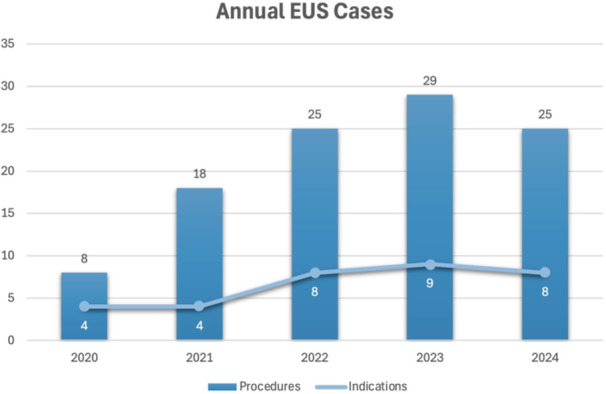


Figure 1: Procedure volume and indications for full calendar years during the study period.

## 57 THE CORRELATION BETWEEN ESOPHAGEAL DISTENSIBILITY WITH DYSPHAGIA SCORES IN PEDIATRIC EOSINOPHILIC ESOPHAGITIS IS DEPENDENT ON DISEASE ACTIVITY

*Joshua Thariath* ^*2*^, *Jose Peraza* ^*1*^, *Ritam Patel* ^*2*^, *Zeinab Dehghani* ^*1,2*^, *Kaitlyn Keeley* ^*1*^, *Michael White* ^*1,2*^, *Joshua Wechsler* ^*1,2*^

^*1*^ *Pediatrics*, *Ann & Robert H. Lurie Children's Hospital of Chicago*, *Chicago*, *IL*; ^*2*^ *Northwestern University Feinberg School of Medicine*, *Chicago*, *IL*

**Introduction:** Eosinophilic esophagitis (EoE) is a chronic, immune‐mediated disorder of the esophagus that often starts in childhood and can persist into adulthood. Endoscopic functional lumen imaging probe (EndoFLIP) is an invaluable tool to assess esophageal diameter in EoE. Decreased esophageal diameter is seen in EoE and is associated with a fibrostenotic phenotype, dysphagia, and food impaction. Validated measures of symptoms and quality of life include the Pediatric Eosinophilic Esophagitis Symptom Score (PEESS) v2.0 and Peds QL 4.0. However, studies to correlate esophageal diameter with these measures are lacking. We aimed to assess the relationship of diameter with symptom and quality of life scores in pediatric EoE.

**Methods:** This was a single‐center, prospective cohort study of patients aged 10‐21 years with EoE undergoing upper endoscopy and EndoFLIP at Lurie Children's Hospital between 1/2021‐3/2024. Patients completed the symptom questionnaires at time of endoscopy. Minimum diameter in the esophageal body at the distensibility plateau was determined (referred to as FLIP diameter). Comparisons were made between in EoE vs non‐EoE patients and further stratified by active EoE (>15 eosinophils per high power field) vs EoE in remission using Mann‐Whitney or chi‐square tests. Pearson correlation coefficients were determined to evaluate the relationship between PEESS composite and domain subscores as well as PedsQL 4.0 composite scores with FLIP diameter, adjusting for age, which can impact FLIP diameter.

**Results:** We recruited 61 patients with EoE and 8 non‐EoE controls. The mean age was 13±4 years old and 28% were female. Among EoE patients, 37 (61%) had active EoE.

Patients with EoE had decreased FLIP diameter compared to non‐EoE controls (14.6±2.7 vs 17.0 ±1.8 mm; p=0.007). There was no difference in FLIP diameter in patients with active vs inactive EoE (p=0.49). We observed a higher PEESS Child score (30±14 vs 21±13; p=0.019) and a higher PEESS Parent GERD score (21±19 vs. 12±13; p=0.048) in active EoE. No differences were observed in PedsQL by disease activity except a higher EoE Child QoL score in active EoE (90±11 vs. 75±16; p=0.008).

In patients with EoE, we observed a moderate inverse correlation between FLIP diameter and PEESS Child Dysphagia (r=‐0.34, p=0.015) and borderline significant weak‐moderate inverse trend between FLIP diameter and PEESS Parent Dysphagia (r=‐0.27, p=0.05).

To understand the impact of inflammation on the association between symptoms and distensibility, we separately analyzed patients with active EoE and remission (Figure 1). Among patients with active EoE, we observed a strong inverse correlation between FLIP diameter and PEESS Child Dysphagia score (r=‐0.55, p=0.002). No other PEESS Child or Parent Scores were significantly correlated with FLIP diameter in active EoE.

There was also no significant correlation between FLIP diameter and any PEESS Child or Parent scores or PedsQL in patients with EoE in remission. FLIP diameter was correlated with several PedsQL domains: Symptoms 2 (r=0.42, p=0.02) in active EoE, and Peds QL Parent School Function (r=‐0.43, p=0.049) in EoE in remission. No other PedsQL composite or sub‐domain scores were correlated with FLIP diameter regardless of disease activity.

**Discussion:** In those with EoE, child dysphagia symptom scores were moderately inversely correlated with FLIP diameter, with an additional moderate‐strength trend seen with parent dysphagia scores. When stratified by disease status, the association between FLIP diameter and child dysphagia scores was enhanced. No correlation was seen between diameter and symptoms scores in pediatric patients with EoE in remission.

Our findings of a significant correlation between physiological FLIP parameters and symptoms only in active EoE suggest that the perception of symptom severity may in part be driven by the presence of inflammation.


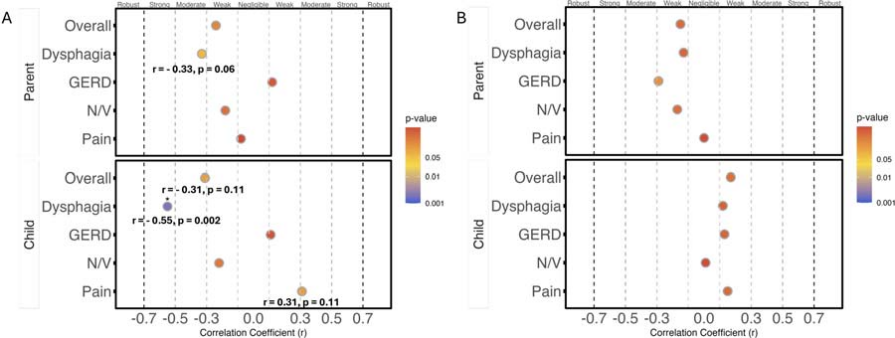


Figure 1. In Active EoE, minimum diameter in the esophageal body had a strong inverse correlation with Dysphagia Child scores and a trend with Dysphagia Parent Scores. B) In Remission, no correlation was observed with diameter and PEESS scores.

## 59 LONG‐TERM DUPILUMAB MAINTAINS HISTOLOGIC AND ENDOSCOPIC IMPROVEMENTS IN CHILDREN WITH EOSINOPHILIC ESOPHAGITIS (EOE): 100‐WEEK RESULTS FROM THE OPEN‐LABEL EXTENSION (OLE) OF THE EOE KIDS STUDY

*Mirna Chehade* ^*1*^, *Evan Dellon* ^*2*^, *Robert Pesek* ^*3*^, *Margaret Collins* ^*4*^, *Dhandapani Ashok* ^*5*^, *Ruiqi Liu* ^*6*^, *Margee Louisias* ^*7*^, *Allen Radin* ^*6*^

^*1*^ *Departments of Pediatrics and Medicine*, *Mount Sinai Center for Eosinophilic Disorders, Icahn School of Medicine at Mount Sinai*, *New York*, *NY*; ^*2*^ *University of North Carolina School of Medicine*, *Chapel Hill*, *NC*; ^*3*^ *University of Arkansas for Medical Sciences and Arkansas Children's Hospital*, *Little Rock*, *AR*; ^*4*^ *Cincinnati Children's Hospital Medical Center and University of Cincinnati College of Medicine*, *Cincinnati*, *OH*; ^*5*^ *London Health Sciences Centre Children's Hospital*, *London*, *ON*, *Canada*; ^*6*^ *Regeneron Pharmaceuticals Inc*, *Tarrytown*, *NY*; ^*7*^ *Sanofi*, *Cambridge*, *MA*

**Objectives and Study:** EoE is a chronic, progressive, type 2 inflammatory disease of the esophagus. Dupilumab, a fully human monoclonal antibody that blocks interleukin‐4 and interleukin‐13, significantly improved histologic and endoscopic outcomes versus placebo in Parts A and B of the EoE KIDS study in pediatric (1–11 years) patients with EoE (NCT04394351). We assessed safety and efficacy of long‐term dupilumab in the Part C OLE.

**Methods:** Patients completing Week (W)52 in Part B were eligible for Part C, where they received the weight‐tiered, open‐label dupilumab regimen later approved by the FDA (similar to the Part A and B higher‐exposure regimen). Part C ended early with FDA approval of dupilumab for pediatric patients with EoE; efficacy data to the last prespecified assessment at W100 are reported.

**Results:** 102 patients enrolled in Part A, 98 in Part B, and 61 in Part C. At W100, the proportions of patients achieving peak esophageal intraepithelial eosinophil counts of ≤6 and <15 eosinophils/high‐power field (eos/hpf) were similar/improved vs those observed with higher‐exposure dupilumab through W52 of Part B: 70.7% vs 62.9% (**Figure**) and 92.7% vs 85.7%, respectively. Additionally, mean (standard deviation) changes from Part A baseline at W100 were similar to those with higher‐exposure dupilumab through W52 of Part B in EoE‐Histologic Scoring System grade and stage scores (‐0.85 [0.39] vs ‐0.97 [0.39], and ‐0.85 [0.36] vs ‐0.89 [0.32], respectively) and EoE‐Endoscopic Reference Score (‐5.34 [2.54] vs ‐4.77 [3.08]). Overall, 53/61 (86.9%) patients reported mild or moderate adverse events (AEs) and 3/61 (4.9%) reported serious AEs (none deemed related to dupilumab). The most common treatment‐related AE during Part C was injection site reaction.

**Conclusions:** Dupilumab had a consistent, acceptable safety profile and maintained histologic and endoscopic efficacy in pediatric patients with EoE to W100. Future analyses will evaluate symptoms.


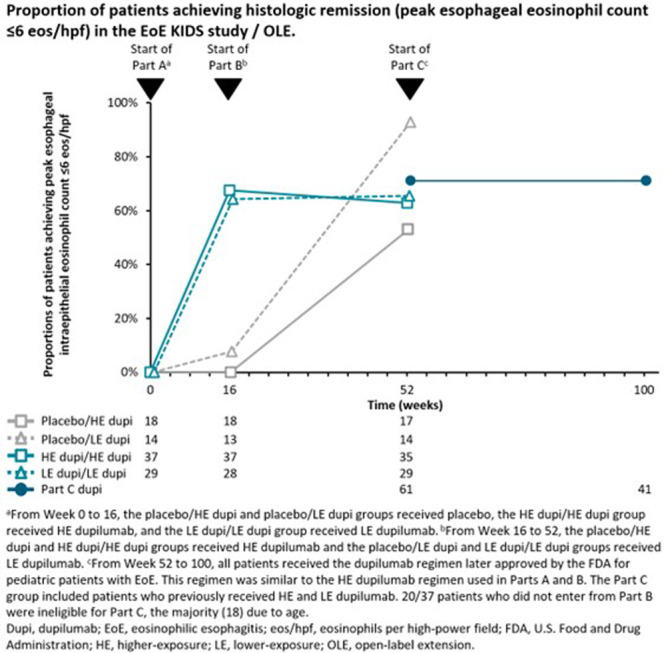


## 60 PREVALENCE AND CHARACTERISTICS OF EATING AND FEEDING DISORDERS AMONG CHILDREN WITH EOSINOPHILIC ESOPHAGITIS

*Matilda Francis* ^*1*^, *Thomas Sferra* ^*2*^, *Senthilkumar Sankararaman* ^*3*^, *Aravind Thavamani* ^*2*^

^*1*^ *Pediatrics*, *University Hospitals Rainbow Babies & Children's Hospital*, *Cleveland*, *OH*; ^*2*^ *Pediatric Gastroenterology, Hepatology and Nutrition*, *University Hospitals Rainbow Babies & Children's Hospital*, *Cleveland*, *OH*; ^*3*^ *Pediatric Gastroenterology, Hepatology and Nutrition*, *Cleveland Clinic Children's Hospital*, *Cleveland*, *OH*

**Introduction:** Patients with eosinophilic esophagitis (EoE) and resultant esophageal inflammation may have an increased risk of development of eating and feeding disorders. Further, symptoms of EoE such as dysphagia, decreased oral intake, and weight loss often overlap with symptoms associated with eating and feeding disorders and may masquerade their diagnosis. There is limited data on the prevalence of eating and feeding disorders and their clinical characteristics in the pediatric population, particularly among patients with EoE.

**Methods:** This study analyzed the Pediatric Health Information System (PHIS) database from Children's Hospital Association and collected data from 49 Children's Hospitals across the United States from 2008 to 2024 to include all patients up to 18 years of age with a diagnosis of EoE using the International Classification of Diseases (ICD)‐10 codes. EoE patients with one of the following diagnoses: anorexia nervosa, bulimia, avoidant/restrictive food intake disorder (ARFID), binge eating disorder, and unspecified eating disorders (not categorized) were categorized as the eating disorder group and those with pediatric feeding disorder, pica, and rumination disorder diagnoses were categorized as feeding disorder group. They were then compared with EoE patients without any of these comorbid eating/feeding disorders for various demographic factors and associated clinical comorbidities such as malnutrition, overweight and obesity, and chronic conditions involving various organ systems including malignancy, metabolic disorders, renal disorders, neurological disorders, mental health disorders, congenital or genetic disorders, and respiratory disorders.

**Results:** We analyzed a total of 73,291 patients with EoE during the 17‐year time period. Within this group of patients, the overall prevalence of eating and feeding disorders was 10.7% (7,823 patients). A total of 605 patients (0.8% of study cohort) had a diagnosis of an eating disorder. Amongst eating disorder diagnoses, ARFID was the most prevalent in 55.4% (n=335), followed by eating disorders, unspecified type in (38.7%), anorexia nervosa (9.4%), bulimia nervosa (2%), and binge eating disorder (1%). In the feeding disorder cohort (N=7132, 9.7% of the entire cohort), apart from pediatric feeding disorder diagnosis, rumination disorder was observed in 1.2% and pica in 1% of patients. Patients with feeding disorders were relatively younger and had a larger proportion of African American and Hispanic patients compared to controls, whereas patients with eating disorders had increased proportion of older and female pediatric patients, in addition to an increased prevalence of anxiety and depression. Malnutrition was more prevalent in both eating and feeding disorder groups. Separate logistic regression models showed that eating disorder was increasingly associated with the 6 to 12 year age group, female gender, anxiety, depression, and presence of metabolic and neuromuscular comorbidities (P<0.05). Feeding disorder was increasingly associated with younger age groups (0 to 5 years), male gender, African American and Asian race, public insurance, malnutrition and anxiety disorders along with presence of metabolic, neuromuscular, genetic, renal and respiratory comorbidities.

**Conclusion:** More than 1 in 10 children with EoE have an eating or feeding disorder. The prevalence of eating disorders and feeding disorders in EoE patients were 0.8% and 9.7% respectively. Further, pediatric patients with eating and feeding disorders have increased comorbidities and often had malnutrition. Prompt recognition and multidisciplinary management of EoE and comorbidities may positively impact health and nutritional outcomes in patients with EoE.


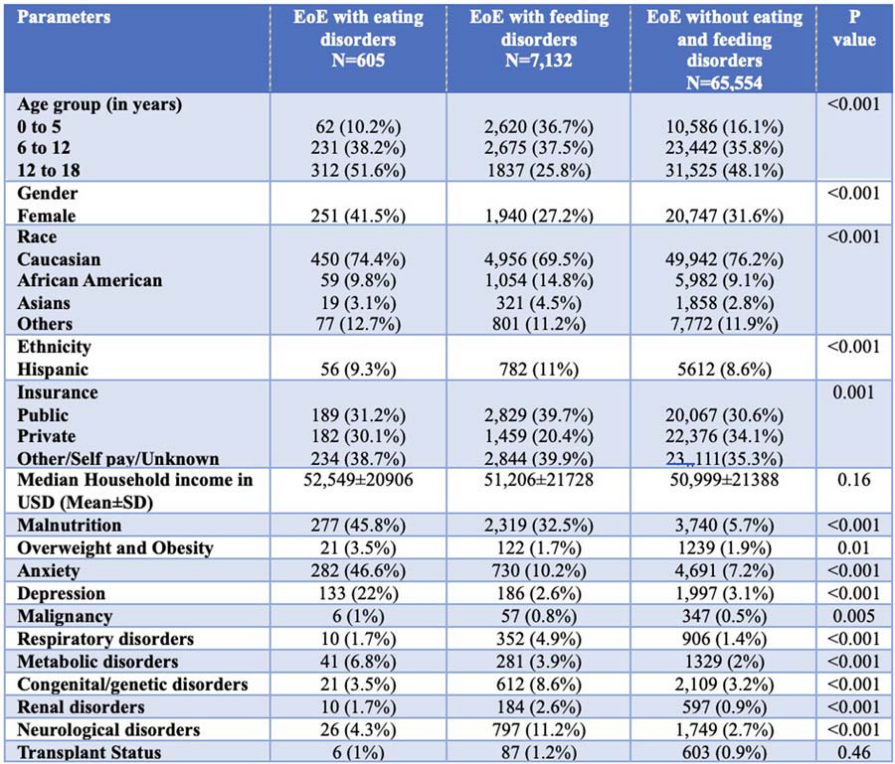


Table 1: Demographics of patients with eosinophilic esophagitis (EoE) and comparison between the groups

## 64 DUPILUMAB IMPROVES HISTOLOGIC AND ENDOSCOPIC FEATURES OF PEDIATRIC ONSET EOSINOPHILIC ESOPHAGITIS (EOE): POOLED RESULTS FROM THE EOE KIDS AND LIBERTY EOE TREET STUDIES

*Noam Zevit* ^*1*^, *Joshua Wechsler* ^*2*^, *Jonathan Markowitz* ^*3*^, *Francesca Racca* ^*4*^, *Carlos Gonzalez* ^*5*^, *Christine Cazeau* ^*6*^, *Bram Raphael* ^*5*^, *Cristina Almansa* ^*6*^, *Amr Radwan* ^*5*^

^*1*^ *Eosinophilic Gastrointestinal Disease Clinic, Institute of Gastroenterology, Hepatology, and Nutrition, Schneider Children's Medical Center of Israel, and Gray Faculty of Medical Sciences, Tel Aviv University*, *Tel‐Aviv*, *Israel*; ^*2*^ *Ann and Robert H. Lurie Children's Hospital of Chicago*, *Chicago*, *IL*; ^*3*^ *Prisma Health, University of South Carolina School of Medicine‐Greenville*, *Greenville*, *SC*; ^*4*^ *Personalized Medicine, Asthma and Allergy Clinic, IRCCS Humanitas Research Hospital*, *Milan*, *Italy*; ^*5*^ *Regeneron Pharmaceuticals Inc*., *Tarrytown*, *NY*; ^*6*^ *Sanofi*, *Cambridge*, *MA*

**Introduction:** Eosinophilic esophagitis (EoE) is a chronic, progressive, type 2 inflammatory disease of the esophagus that can affect people of all ages. Despite a common disease pathogenesis, age‐dependent differences in clinical presentation, endoscopic features, and response to certain therapies have been reported. In the randomized, placebo‐controlled, phase 3 EoE KIDS (NCT04394351) and LIBERTY EoE TREET studies (NCT03633617), dupilumab improved EoE outcomes, including histologic and endoscopic features, in children and adolescents/adults with active EoE, respectively. Given nuances in clinical characteristics and approach to treatment in children and adolescents with EoE across the ≥1 to <18 years age range, further exploration of dupilumab treatment response in such patients is warranted. This pooled analysis of the KIDS and TREET studies aimed to assess the efficacy of dupilumab on histologic and endoscopic outcomes in pediatric patients (aged ≥1–<18 years), stratified by age at baseline (≥1–<6, ≥6–<12, and ≥12–<18 years).

**Methods:** Patients randomized to weight‐tiered, higher‐exposure dupilumab or placebo in EoE KIDS, and weekly dupilumab 300 mg or placebo in LIBERTY EoE TREET, were included. At the end of each study's double‐blind treatment period (Week 16 in KIDS; Week 24 in TREET), patients either continued dupilumab or switched from placebo to dupilumab through Week 52. Proportions of patients achieving ≤6 or <15 eosinophils per high‐power field (eos/hpf), and percentage change in EoE‐Histologic Scoring System (EoE‐HSS) grade/stage scores and EoE‐Endoscopic Reference Score (EREFS) total score, calculated based on the worst observed region of the esophagus, were assessed at Weeks 16/24 and 52. Outcomes were analyzed by age at baseline: ≥1–<6, ≥6–<12, and ≥12–<18 years. All *P*‐values for dupilumab vs placebo are nominal.

**Results:** 143 patients were included: 74 received dupilumab (≥1–<6 years, n=14; ≥6–<12 years, n=23; ≥12–<18 years, n=37) and 69 received placebo (≥1–<6 years, n=8; ≥6–<12 years, n=26; ≥12–<18 years, n=35). At the end of the double‐blind treatment period, dupilumab resulted in significantly greater proportions of patients achieving ≤6 and <15 eos/hpf vs placebo in all age groups (≥1–<6 years: 85.7% vs 0.0% and 100.0% vs 0.0%, ≥6–<12 years: 56.5% vs 3.8% and 73.9% vs 3.8%, ≥12–<18 years: 51.4% vs 5.7% and 67.6% vs 5.7%, respectively; all *P*<0.0001) (**Figure**). Significant improvements were also observed in all age groups treated with dupilumab compared with placebo for percentage change from baseline in EoE‐HSS grade scores (–65.1% vs 12.2%, –64.7% vs 2.2%, –53.0% vs 3.8%; all *P*<0.0001), EoE‐HSS stage scores (–62.2% vs 15.7%, –63.7% vs 3.1%, –51.3% vs 6.9%; all *P*<0.0001), and worst‐observed EREFS total scores (–40.7% vs 25.1%, –56.5% vs 10.8%, –42.5% vs 21.1%; all *P*<0.001). At Week 52, outcomes were sustained or further improved with continued dupilumab, and improvements were observed in patients who switched from placebo to dupilumab at Week 16/24 (**Figure** shows proportions of patients achieving ≤6 and <15 eos/hpf).

**Conclusion:** In this pooled analysis of pediatric patients from the clinical trials of dupilumab, sustained improvements in histologic and endoscopic features of EoE were observed across age subgroups. Although patient numbers were small, and this analysis was not designed to draw comparisons between age groups, the youngest subgroup (≥1–<6 years) experienced the greatest numerical rate of histologic remission and response at Week 16.


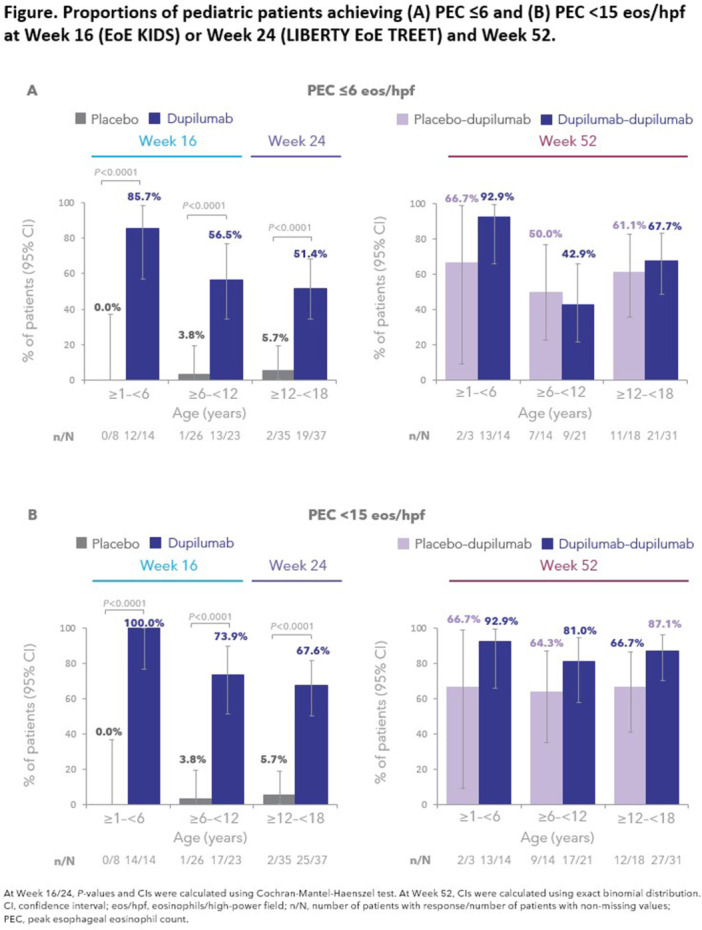


## 65 DUPILUMAB IMPROVES CAREGIVER‐REPORTED EOSINOPHILIC ESOPHAGITIS SYMPTOMS IN CHILDREN AGED 1–11 YEARS: 16‐WEEK RESULTS FROM THE PHASE 3 EOE KIDS STUDY

*Dhandapani Ashok* ^*1*^, *Marc Rothenberg* ^*2*^, *Benjamin Gold* ^*3*^, *Navneet Virk Hundal* ^*4*^, *Salvatore Oliva* ^*5*^, *Changming Xia* ^*6*^, *Sherif Zaghloul* ^*7*^, *Bram Raphael* ^*6*^, *Sarette Tilton* ^*7*^, *Ryan Thomas* ^*6*^

^*1*^ *London Health Sciences Centre Children's Hospital*, *London*, *ON*, *Canada*; ^*2*^ *Cincinnati Children's Hospital Medical Center and University of Cincinnati College of Medicine*, *Cincinnati*, *OH*; ^*3*^ *Gastroenterology*, *Children's Center for Digestive Healthcare, LLC*, *Atlanta*, *GA*; ^*4*^ *Food Allergy Center, Massachusetts General Hospital*, *Boston*, *MA*; ^*5*^ *Pediatric Digestive Endoscopy, Pediatric Gastroenterology and Liver Unit, Maternal and Child Health Department, University Hospital Umberto, Sapienza University of Rome*, *Rome*, *Italy*; ^*6*^ *Regeneron Pharmaceuticals Inc*., *Tarrytown*, *NY*; ^*7*^ *Sanofi*, *Morristown*, *NJ*

**Objectives and Study:** Eosinophilic esophagitis (EoE) is a chronic, type 2 inflammatory disease characterized by esophageal barrier dysfunction as well as mucosal inflammation, and, in some patients, esophageal narrowing and scarring. The validated Pediatric Eosinophilic Esophagitis Symptom Score, version 2.0 (PEESSv2.0) measures patient‐relevant domains (dysphagia, gastroesophageal reflux disease [GERD], nausea/vomiting, and pain). The objective of this research was to evaluate the proportion of pediatric patients with EoE symptoms achieving a measurable improvement in symptom response scores, as measured by the PEESSv2.0.

**Methods:** This post hoc analysis of data from the phase 3 EoE KIDS study (NCT04394351) included patients aged 1–11 years with EoE who were randomized to weight‐tiered dupilumab higher exposure, or placebo, to Week 16. PEESSv2.0 total score (0–100; higher scores indicate greater symptom burden) was evaluated at baseline and Week 16. Response rates in dupilumab‐ and placebo‐treated patients were compared across a range of percent reduction from baseline response thresholds (≥25%, ≥50%, ≥75%, and 100%).

**Results:** Dupilumab treatment led to a greater reduction in PEESSv2.0 total score versus placebo at Week 16 (least squares mean difference [95% confidence interval] ‐8.03 [‐15.39, ‐0.67]). Greater proportions of patients achieved a range of PEESSv2.0 total score reduction thresholds with dupilumab versus placebo at Week 16 (**Table**). Overall safety was consistent with the known dupilumab safety profile.

**Conclusions:** Sixteen weeks of dupilumab treatment led to an improvement in caregiver‐reported symptoms (including pain, dysphagia, GERD, nausea, and vomiting) versus placebo across all symptom scoring thresholds assessed using PEESSv2.0. These findings suggest that dupilumab is a viable treatment option for pediatric patients aged 1–11 years with EoE.


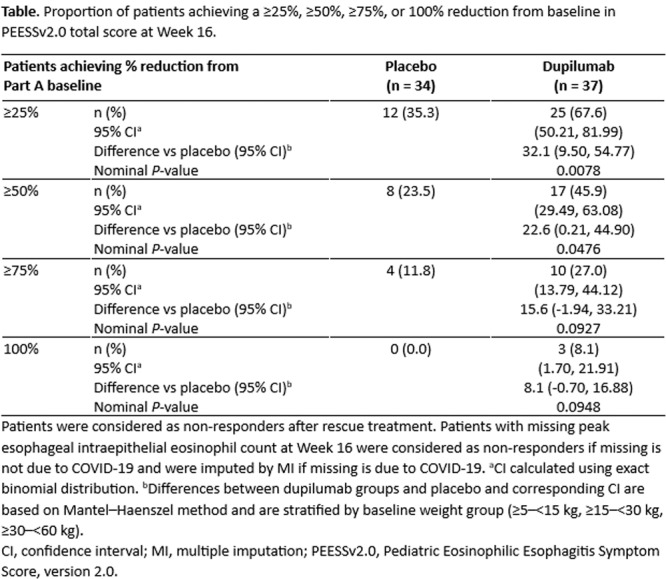


## 66 DUPILUMAB IS EFFECTIVE IN MAINTAINING LONG‐TERM IMPROVEMENTS IN DISEASE SEVERITY AND QUALITY OF LIFE IN CHILDREN WITH EOSINOPHILIC ESOPHAGITIS: 100‐WEEK RESULTS FROM THE OPEN‐LABEL EXTENSION OF THE EOE KIDS STUDY

*Mirna Chehade* ^*1*^, *Salvatore Oliva* ^*2*^, *Christos Tzivinikos* ^*3*^, *Ruiqi Liu* ^*4*^, *Margee Louisias* ^*5*^, *Allen Radin* ^*4*^, *Sarette Tilton* ^*5*^, *Ryan Thomas* ^*4*^

^*1*^ *Icahn School of Medicine at Mount Sinai*, *New York*, *NY*; ^*2*^ *University Hospital Umberto, Sapienza University of Rome*, *Rome*, *Italy*; ^*3*^ *Al Jalila Children's Speciality Hospital*, *Dubai*, *United Arab Emirates*; ^*4*^ *Regeneron Pharmaceuticals Inc*., *Tarrytown*, *NY*; ^*5*^ *Sanofi*, *Cambridge*, *MA*

**Introduction:** EoE is a chronic, progressive, type 2 inflammatory disease of the esophagus that can significantly impact patient quality of life (QoL). Dupilumab is approved in the USA and EU for patients with EoE aged ≥1 year, weighing ≥15 kg. The phase 3 EoE KIDS study (NCT04394351) evaluated the efficacy and safety of dupilumab in children aged 1 to 11 years with EoE vs placebo. For up to 52 weeks, dupilumab has been shown to improve disease severity as measured by the patient‐, caregiver‐, and clinician‐reported Global Impression of Severity (GIS‐P/C/Clin), and patient and caregiver QoL as measured by the Pediatric EoE Impact Scale (PEIS‐P/C).

**Aims & methods:** This post hoc analysis of the EoE KIDS study aimed to assess the impact of long‐term dupilumab treatment on patient and caregiver QoL, and patient‐ and observer‐reported disease severity during the Part C open‐label extension. The PEIS‐P and PEIS‐C, respectively, are patient‐ and caregiver‐reported outcome measures that assess the impact of the patients’ EoE on the patients’ and caregivers’ QoL during the past week. The PEIS‐P/C scores range from 0 to 4, with a higher score indicating a greater negative impact on QoL. The GIS‐P and GIS‐C/Clin, respectively, are single‐item, patient‐ and observer‐reported outcome measures of the severity of EoE over the past week. The GIS‐P/C/Clin are measured on a 4‐point scale from 1 (mild) to 4 (very severe). In Part A of the EoE KIDS study, patients were randomized to receive either placebo, dupilumab higher exposure (HE), or dupilumab lower exposure up to Week 16. In Part B, patients either continued dupilumab, or switched from placebo to dupilumab up to Week 52. Patients who completed Part B were eligible for Part C open‐label extension (OLE) period, during which they received the weight‐tiered dupilumab dose regimen, later approved by the FDA (similar to the Part A and B HE regimen), for up to 108 weeks. Percent change from baseline in PEIS‐P/C scores and change from baseline in GIS‐P/C/Clin scores were assessed in patients enrolled in Part C who previously received placebo or dupilumab HE during Parts A and B at Week 52, Week 76, and Week 100 (the last available timepoint for efficacy).

**Results:** PEIS‐P, PEIS‐C, and GIS‐P/C/Clin scores at Part A baseline are detailed in **Table 1**. At Week 52, the mean percent changes from baseline (95% confidence interval [CI]) in PEIS‐P and PEIS‐C scores were −42.5% (−75.9, −9.2) and −60.9% (−81.0, −40.9), respectively, with further reductions observed at Week 100 (−83.2% [−100, −62.9] and −80.7% [−97.6, −63.7]) (**Figure 1**). Mean changes from Part A baseline (95% CI) in GIS‐P/C/Clin scores were maintained, and in some cases, further reduced from Week 52 (−0.6 [−1.1, −0.1]/−0.9 [−1.2, −0.6]/−1.3 [−1.7, −1.0]) to Week 100 (−0.7 [−1.3, 0.0]/−0.9 [−1.2, −0.5]/−1.2 [−1.6, −0.8]). Dupilumab safety was consistent with its known safety profile.

**Conclusion:** Reductions in PEIS‐P/C scores and GIS‐P/C/Clin scores were maintained and, in some cases, further improved during the Part C OLE. For up to 100 weeks, dupilumab improved patient and caregiver QoL, and reduced EoE severity as reported by patients, caregivers, and clinicians.


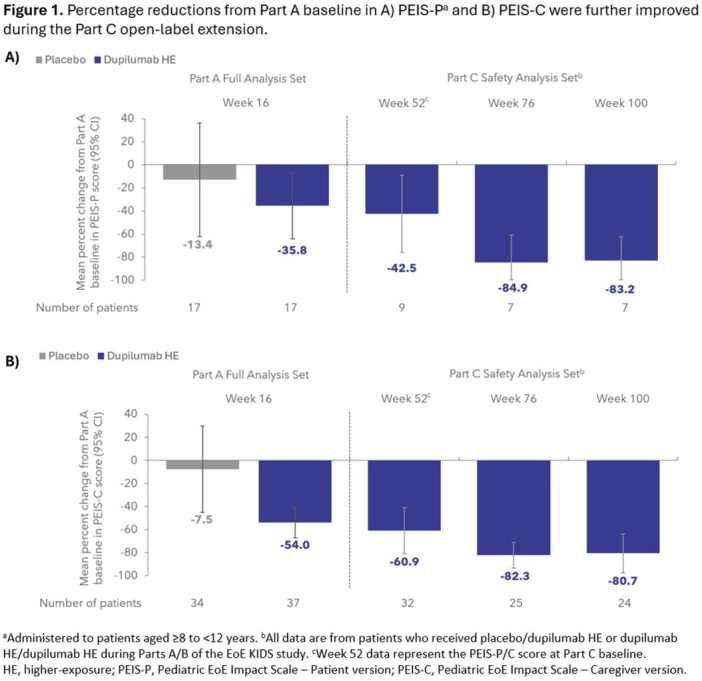



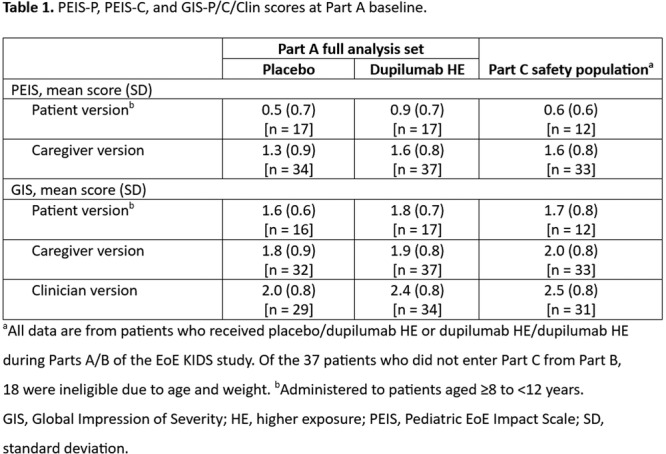


## 67 EFFICIENCY OF REDUCED DOSING FOR DUPILUMAB IN PEDIATRIC EOSINOPHILIC ESOPHAGITIS: A RETROSPECTIVE ANALYSIS

*Ebtihal Ahmed*, *Khalid M Al Kharraz*, *Belinda Kwartemaa Nti*, *Imad Absah*, *Puanani Hopson*

*Pediatric*, *Mayo Clinic Minnesota*, *Rochester*, *MN*

**Introduction:** Eosinophilic esophagitis (EoE) is a chronic, immune‐mediated esophageal disease characterized by eosinophilic infiltration and symptoms of esophageal dysfunction. Current treatment includes the use of biologics, specifically dupilumab, particularly in patients who are refractory to PPI and topical corticosteroids. Dupilumab is a monoclonal antibody targeting the interleukin‐4 receptor alpha (IL‐4rα), which inhibits IL‐4 and IL‐13 signaling.

The FDA approved dupilumab for use in children aged >12 years of age in 2022 and then in 1 to 11 years of age with EoE in 2024.

Dupilumab dosing and frequency for the treatment of EoE varies based on the age and weight of the patient. There are reports of continued efficacy of dupilumab despite weaning in other disorders such as asthma and atopic dermatitis. There is a paucity of data regarding the efficacy of reduced dosing strategy through interval prolongation of dupilumab for children with EoE. Our study aims to assess the effect of weaning dupilumab dose on clinical and histologic remission in children with EoE.

**Methods:** We performed a retrospective chart review of the Mayo Clinic electronic health records between 2019 and 2025. We included all children (<18 years) who were diagnosed with EoE and are being treated with dupilumab. The dose and frequency regimen of dupilumab at initiation and subsequent changes were at the discretion of the prescribing physician. We defined a reduced dose strategy as a planned adjustment following initial endoscopic follow‐up assessment to a less frequent dupilumab administration schedule (e.g., extending the interval from every week to every two weeks). Collected clinical endpoints included symptoms and histologic parameters, including peak eosinophil count (PEC). Histologic remission was characterized by a PEC <15.

**Results:** We identified 80 children with EoE who are being treated with dupilumab during the study period. The average age at dupilumab initiation was 12.6 yrs, 55% were males. Approximately 17 patients underwent reduced dose strategy (average age 11.9 yrs), with the majority (n=14, 82.4%) initially on weekly dupilumab injections (Figure 1). Four of the 17 patients discontinued the reduced dose strategy and returned to original dosing without follow‐up endoscopic evaluation due to symptom reoccurrence (abdominal pain (n=3) and atopic dermatitis (n=1). Of the remaining 13 patients who underwent follow‐up endoscopy (within average of 5.1 months from dose change) while on the reduced dose strategy, 11 (84.6%) met criteria for histologic remission. The two patients not meeting strict histologic remission criteria (both of which had PEC of 16 eos/hpf) demonstrated symptomatic improvement and reduced PEC from prior endoscopy and as such were considered to be in remission by their treating physician.

**Conclusion:** Given the chronic nature of EoE and the need for long‐term management, exploring reduced dose frequency strategies with dupilumab in the pediatric population could offer a balance between efficacy, patient adherence, and improved quality of life. The study shows promising results in sustained efficacy of dupilumab in children with EoE. Less frequent dosing can be considered to improve compliance, decrease side effects, and cost associated with long‐term treatment. Larger studies are needed to confirm these results.


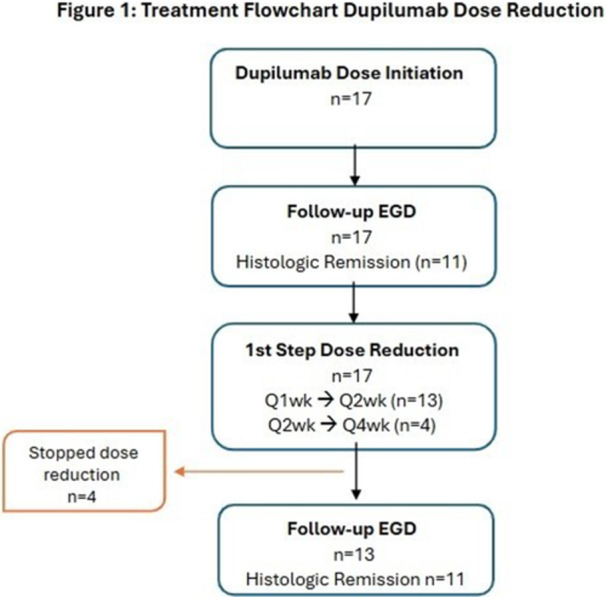


## 68* FROM PRIMARY CARE TO SPECIALTY CARE: HOW PATIENT BACKGROUND AFFECTS SUBSPECIALTY REFERRAL COMPLETION

*Doris Valenzuela‐Araujo* ^*1*^, *Alyssa Ciarlante* ^*2*^, *Ian Delorey* ^*3*^, *Katherine Yun* ^*2*^, *Jennifer Webster* ^*1*^

^*1*^ *Gastroenterology, Hepatology and Nutrition*, *Children's Hospital of Philadelphia*, *Portland*, *OR*; ^*2*^ *The Children's Hospital of Philadelphia*, *Philadelphia*, *PA*; ^*3*^ *University of Pennsylvania*, *Philadelphia*, *PA*

**Background:** Children in families facing challenges such as poverty, lack of access to transportation, and language barriers also face significant healthcare navigation challenges. These are augmented when families require pediatric subspecialty care due to wait times, complex referral processes, variable accommodations for language needs, and centralization of care outside of the neighborhoods where families live.

**Objective:** Our study examines how patient preferred language, race/ethnicity, and social vulnerability index are associated with both subspecialty scheduling and referral completion rates following a referral from primary care.

**Methods:** This retrospective study evaluated referral data in a large pediatric academic center with over 500,000 pediatric specialty visits per year and a large regional network of primary care practices. We analyzed referral orders placed during primary care visits within this network from January 2022‐ December 2023. We focused on specialties with longer wait times: Gastroenterology, Allergy, Dermatology, Developmental Pediatrics, and Pulmonology. We examined preferred language (English, Spanish, Other), interpreter use, age, race, ethnicity, insurance, zip code, and social vulnerability index (SVI).

**Results:** During the study period, primary care clinicians placed 48,881 specialty referrals orders for the services listed above. Of these, 49.8% of referrals resulted in a subspecialty visit, and the mean time from referral placement to referral completion was 96 days (SD 83). Most families had a preferred language of English (94%) and were Non‐Hispanic White (38.8%), Non‐Hispanic Black (31.7%), or Hispanic/Latino (10.8%). Participants were primarily residing in the lowest (34.3%) and highest (21.9%) SVI quartiles. When compared to Non‐Hispanic White children, the unadjusted odds ratio for completing a specialty referral was significantly lower for Non‐Hispanic Black (0.49, 0.43‐0.56), Hispanic/Latino (0.76, 0.63‐0.93), and Non‐Hispanic Asian (0.76, 0.59‐0.99) patients. Those in the highest SVI quartile had significantly lower odds of scheduling an appointment when compared to those in the lowest quartile. Similar trends were seen with referrals to Gastroenterology, where the groups that had the lowest odds of referral completion were Non‐Hispanic Black (0.32, 0.18‐0.57), Hispanic/Latino (0.36, 0.18‐0.71), and highest SVI quartile (0.46, 0.24‐0.86) patients.

**Conclusion:** In this single center study, we found that most children referred from primary care to pediatric subspecialties—including Gastroenterology—never had a subspecialty appointment. There were significant differences in rates of referrals completion by patient race/ethnicity and SVI quartile. Results from this study highlight the need for interventions to ensure equitable access to subspecialty care and improve timely completion of referrals for all patients.


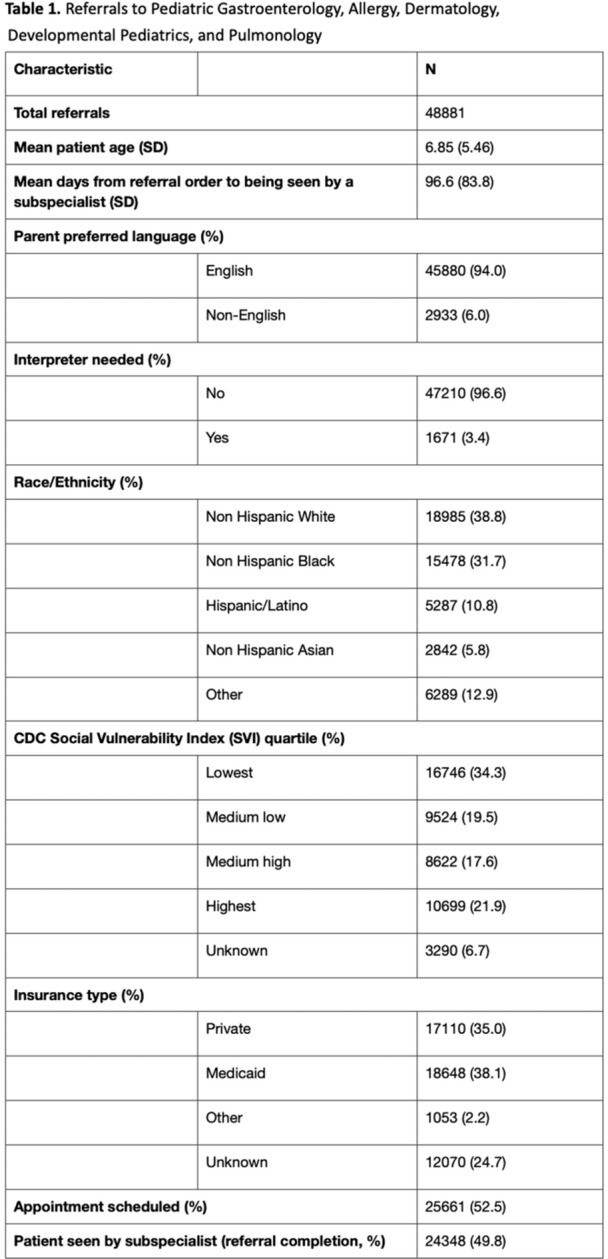



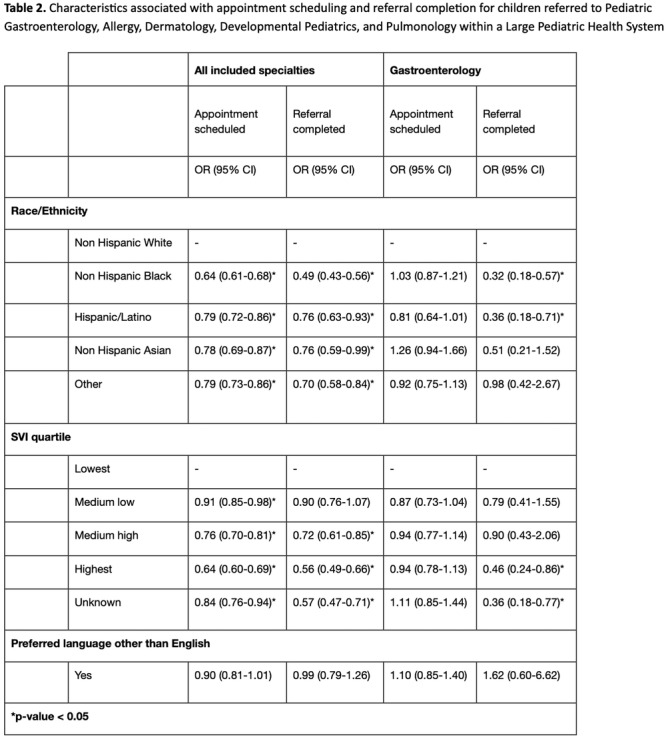


## 69 SOCIAL DETERMINANTS OF HEALTH AND ASSOCIATIONS IN PEDIATRIC INFLAMMATORY BOWEL DISEASE

*Laura Bauman*, *D. Brent Polk*, *Jeannie Huang*

*Pediatric Gastroenterology*, *University of California San Diego*, *La Jolla*, *CA*

Social determinants of health (SDOH) have been shown to significantly influence health outcomes across diverse populations. In pediatric inflammatory bowel disease (IBD), understanding the impact of SDOH is particularly important, given the complex interplay between social factors and disease management. Limited research exists on the prevalence of SDOH risk in pediatric IBD populations. In this study, we aimed to examine the prevalence of SDOH risk in pediatric IBD patients and their potential associations with demographic factors. Our broader work seeks to provide insights to inform tailored interventions to address health inequities in this vulnerable population.

Patients under 24 years of age followed at the Rady Children's Hospital IBD center from Jan 2023 to Nov 2024 were included. SDOH risks were assessed via electronic medical record questionnaires distributed every 6–12 months at patient encounters using the following screeners: the U.S. Household Food Security Survey Module (HFSSM), Housing Instability Screening Tool, Transportation Needs Screening Tool, CRAFFT Screening Test, University of Washington Caregiver Stress Scale, and Caregiver Health Literacy Assessment. Electronic medical record data extracted for analysis included demographics, Paris classification, and SDOH risk assessments. Analyses used Chi‐square and Fisher exact tests to assess associations with Bonferroni corrections for multiple comparisons.

Five‐hundred sixty‐four patients/families were screened via 2357 SDOH questionnaires, with 58 (10.28%) patients reporting SDOH risk. Low caregiver education/literacy was the most common SDOH risk (Table 1) followed by caregiver stress and food insecurity. The median age of patients was 17 with an interquartile range of 5 years, with 41.31% female, 66.31% white, 26.06% Hispanic/Latino, and 4.61% preferring a language other than English. 347 (61.52%) had Crohn's disease. Low caregiver education/literacy was strongly correlated with Hispanic/Latino ethnicity and preferred language other than English (Table 2). Food insecurity was correlated with female sex and preferred language other than English. Housing insecurity correlated with IBD diagnosis, with more housing insecurity among those with indeterminate colitis (16.67%) versus Ulcerative Colitis (1.69%) or Crohn's disease (2.75%).

This study highlights the significant prevalence of SDOH risks among pediatric IBD patients at a pediatric tertiary care center serving a large metropolitan, ethnically diverse area. Caregiver education/literacy challenges, food insecurity, and housing instability were notably correlated with demographic factors such as ethnicity, language preference, and IBD disease subtype. These findings suggest the need for widespread SDOH screening to provided needed resources and to develop targeted interventions to improve health outcomes.


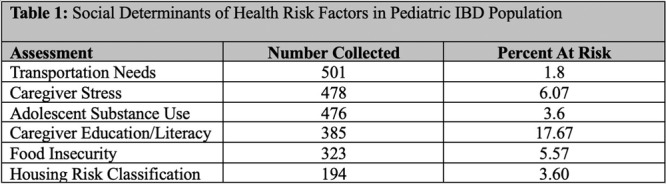


Table 1: Social Determinants of Health Risk Factors in Pediatric IBD Population


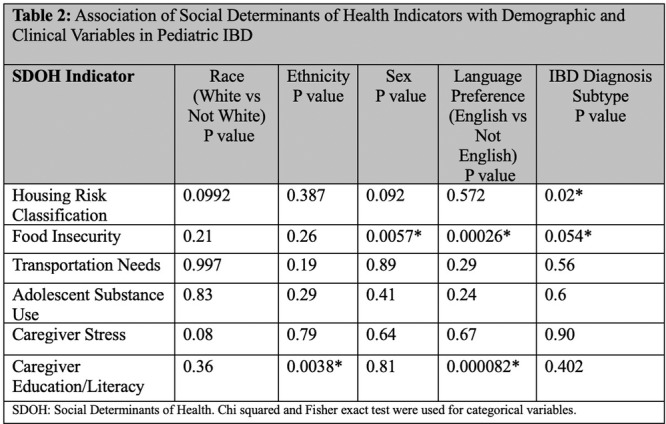


Table 2: Association of Social Determinants of Health Indicators with Demographic and Clinical Variables in Pediatric IBD

## 71 RACE AND NEIGHBORHOOD FACTORS AFFECT OUTCOMES IN CHILDREN HOSPITALIZED WITH ACUTE PANCREATITIS

*Amy Fan* ^*1*^, *Elizabeth Baker* ^*1,2*^, *Chinenye Dike* ^*1*^

^*1*^ *Pediatric Gastroenterology, Hepatology, & Nutrition*, *The University of Alabama at Birmingham Heersink School of Medicine*, *Birmingham*, *AL*; ^*2*^ *Department of Anesthesiology and Perioperative Medicine*, *The University of Alabama at Birmingham Heersink School of Medicine*, *Birmingham*, *AL*

**Background:** Disparities impacting acute pancreatitis (AP) outcomes have been reported. We have shown that Non‐Hispanic Black (NHB) and Hispanic children hospitalized with AP experience longer length of hospital stay (LOS) and costs. Although racial/ethnic disparities are known in AP, geospatial disparities are unexplored in pediatric AP.

**Aim:** To study the impact of area level social determinant of health (SDOH) factors like social vulnerability index (SVI) and area deprivation index (ADI) on outcomes in pediatric AP.

**Methods:** IRB approved retrospective study of children hospitalized with AP at a tertiary institution over 5 years. Clinical and demographic data including home addresses and zip codes were geocoded to determine area level factors for SDOH: SVI (with 4 themes) ADI, Segregation (Isolation, Dissimilarity, Integration), Environmental Justice Index (Health vulnerability, Built environment), distance to pediatric ICU, % Food desert, walkability index, urban and rural index. Descriptive statistics were used to summarize variables for the overall sample stratified by LOS (1‐4 or ≥5 days), AP severity (severe/moderate vs. mild), and complications using NASPGHAN classification. Significance tests are done using high dimensional fixed effects regression (log linear for LOS and linear probability for AP severity and complications) with multiway cluster‐robust standard errors to allow correction for dependence introduced by neighborhood level exposure variable and having multiple observations from the same individual while leveraging all hospitalization‐level observations. Next, we examined significant associations using a multivariate format. Stata software 17.0 was used for analysis, p value < 0.05 was considered significant.

**Results:** 248 encounters were identified for 187 participants living in 160 different census tracts with median age of 13.6 years (IQR: 9.8‐15.76). 51% were males, 58% Non‐Hispanic White (NHW), 29% NHB, 11% Hispanic and 2% other race/ethnic group. 82% experienced mild AP, 16% severe (11% moderately severe and 5% severe), 2% of unknown severity. Mean BMI% was 75% (SD: 30.54). NHB children were more likely to live in urban and more minority neighborhoods (measured by SVI theme 3, the integration, and the isolation indexes) compared to NHW children (p< 0.001). Children living in more urban neighborhoods had an increased mean LOS (p=0.041), AP severity (p=0.027) and complications (p <0.05) compared to those in rural neighborhoods. We found a significant association between living in more minority neighborhoods and having an increased LOS and AP severity. This was seen in SVI theme 3 which was associated with increased mean LOS (p=0.032), Isolation ‐associated with increased mean LOS (p=0.022) and AP severity (p=0.044) and Integration ‐associated with increased LOS (p=0.04). Increased mean LOS did not persist for children living in minority neighborhoods if race is adjusted for (p=0.28). However, increased mean LOS persisted in NHB children after adjusting for age, sex, chronic conditions, more minority and urban neighborhoods (p=0.031). Children diagnosed with biliary AP and hypertriglyceridemia were more likely to be overweight/obese and live in less walkable neighborhoods. ADI and EJI factors did not affect AP outcomes.

**Conclusions:** Children hospitalized with AP living in minority neighborhoods experience an increased LOS and AP severity. Living in an urban neighborhood is a risk for increased LOS and complications. Children with biliary AP and hypertriglyceridemia are more likely to be overweight/obese and live in less walkable neighborhoods. NHB children are more likely to live in urban and minority neighborhoods and have an increased mean LOS which persists after adjusting for covariates in a multivariate model. Therefore, NHB children who live in minority and urban neighborhoods experience worse AP outcomes than other children. This study lays the groundwork for improving neighborhood factors and AP outcomes in children.


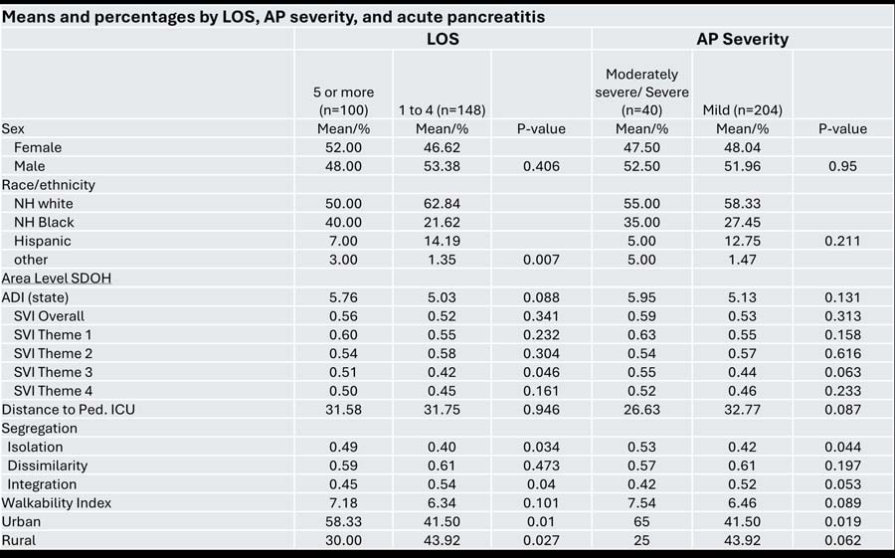


Mean LOS


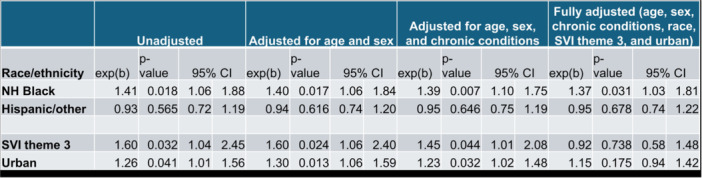


Multivariate LOS

## 74* LONG‐TERM QUALITY OF LIFE OUTCOMES FOR PATIENTS WITH ALAGILLE SYNDROME TREATED WITH ODEVIXIBAT: POOLED RESULTS FROM PHASE III, RANDOMIZED, DOUBLE‐BLIND ASSERT AND OPEN‐LABEL ASSERT‐EXT STUDIES

*Nadia Ovchinsky* ^*1*^, *Madeleine Aumar* ^*2*^, *Alastair Baker* ^*3*^, *Philip Bufler* ^*4*^, *Mara Cananzi* ^*5*^, *Piotr Czubkowski* ^*6*^, *Özlem Durmaz* ^*7*^, *Ryan Fischer* ^*8*^, *Giuseppe Indolfi* ^*9*^, *Wikrom Karnsakul* ^*10*^, *Way Lee* ^*11*^, *Giuseppe Maggiore* ^*12*^, *Mathias Ruiz* ^*13*^, *Etienne Sokal* ^*14*^, *Ekkehard Sturm* ^*15*^, *Wendy van der Woerd* ^*16*^, *Andrew Wehrman* ^*17*^, *Fatine Elaraki* ^*18*^, *Praneeta Nagraj* ^*19*^, *Judy Zhu* ^*19*^, *Henkjan Verkade* ^*20*^

^*1*^ *Pediatric Gastroenterology and Hepatology, Hassenfeld Children's Hospital, NYU Langone*, *New York*, *NY*; ^*2*^ *Univ Lille, CHU Lille, Pediatric Gastroenterology, Hepatology, and Nutrition, Inserm U1286 Infinite, CHU Lille Pôle Enfant*, *Lille*, *France*; ^*3*^ *Paediatric Liver Centre, King's College Hospital*, *London*, *United Kingdom*; ^*4*^ *Department of Pediatric Gastroenterology, Nephrology, and Metabolic Diseases, Charité Universitätsmedizin Berlin, Berlin, Germany; German Center for Child and Adolescent Health (DZKJ), partner site Berlin*, *Berlin*, *Germany*; ^*5*^ *Paediatric Gastroenterology, Digestive Endoscopy, Hepatology, and Care of the Child With Liver Transplantation, Department of Children's and Women's Health, University Hospital of Padova*, *Padova*, *Italy*; ^*6*^ *Department of Gastroenterology, Hepatology, Nutritional Disorders, and Pediatrics, The Children's Memorial Health Institute*, *Warsaw*, *Poland*; ^*7*^ *Istanbul University, Istanbul Faculty of Medicine*, *Istanbul*, *Turkey*; ^*8*^ *Division of Pediatric Gastroenterology, Hepatology, and Nutrition, Children's Mercy Hospital*, *Kansas City*, *MO*; ^*9*^ *Meyer Children's Hospital IRCCS*, *Florence*, *Italy*; ^*10*^ *Division of Pediatric Gastroenterology, Hepatology, and Nutrition, Department of Pediatrics, Johns Hopkins University School of Medicine*, *Baltimore*, *MD*; ^*11*^ *Department of Paediatrics, Faculty of Medicine, University of Malaya*, *Kuala Lumpur*, *Malaysia*; ^*12*^ *Hepatology and Liver Transplantation Unit, Bambino Gesù Children's Hospital IRCCS*, *Rome*, *Italy*; ^*13*^ *Hospices Civils de Lyon, Hôpital Femme‐Mère‐Enfant, Hépatologie Gastro‐entérologie et Nutrition Pédiatriques*, *Bron*, *France*; ^*14*^ *Université Catholique de Louvain, Cliniques St Luc, Service de Gastroentérologie et Hepatologic Pédiatrique*, *Brussels*, *Belgium*; ^*15*^ *Pediatric Gastroenterology and Hepatology, University Children's Hospital Tübingen*, *Tübingen*, *Germany*; ^*16*^ *Department of Pediatric Gastroenterology, Wilhelmina Children's Hospital, University Medical Center Utrecht*, *Utrecht*, *Netherlands*; ^*17*^ *Division of Gastroenterology, Hepatology, and Nutrition, Boston Children's Hospital*, *Boston*, *MA*; ^*18*^ *Ipsen Pharma*, *Boulogne‐Billancourt*, *France*; ^*19*^ *Ipsen*, *Cambridge*, *MA*; ^*20*^ *Pediatric Gastroenterology/Hepatology, Department of Pediatrics, University of Groningen, Beatrix Children's Hospital/University Medical Center Groningen*, *Groningen*, *Netherlands*

**Background:** Patients with Alagille syndrome (ALGS) often have debilitating pruritus with associated sleep disturbances and reduced quality of life (QoL). Robust long‐term data demonstrated sustained efficacy – including clinically significant improvements in pruritus – and tolerability for patients with ALGS remaining on odevixibat (ODX) for cholestatic pruritus after 72 weeks’ follow‐up in ASSERT‐EXT (NCT05035030). Here, we evaluate long‐term (≤96 weeks) pooled QoL data from ASSERT (NCT04674761) and ASSERT‐EXT.

**Methods:** Patients in ASSERT received 24 weeks of placebo or 120 µg/kg/day ODX; study completers were eligible for ASSERT‐EXT (120 µg/kg/day ODX for 72 weeks). Endpoints included: Sleep parameters (Observer‐Reported Outcome [ObsRO]) and caregiver‐assessed QoL (Pediatric Quality of Life Inventory [PedsQL]).

**Results:** 52 patients (median [range] age: 5.7 [1.0–15.5] years) received ODX during ASSERT and ASSERT‐EXT. Patients had significant improvements in 6 of 7 ObsRO sleep parameters from baseline (BL) up to weeks 93−96 and a numerical reduction in days taking medication to induce sleep (**Figure 1 A**). PedsQL scores reported by caregivers also showed numerical improvements from BL up to week 96 with ODX (**Figure 1B**).

**Conclusions:** Long‐term ODX given for up to 96 weeks improved multiple sleep parameters and caregiver‐reported QoL in patients with ALGS; data support long‐term improvements in pruritus seen with ODX.


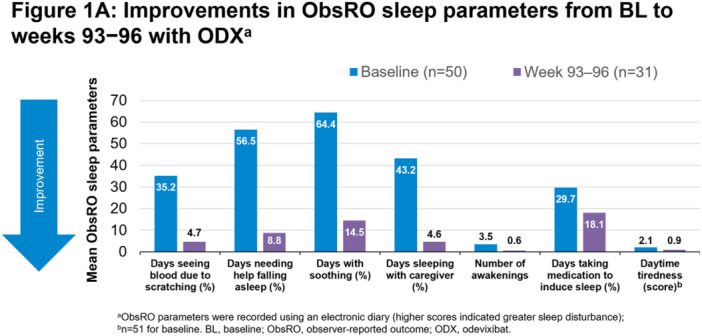



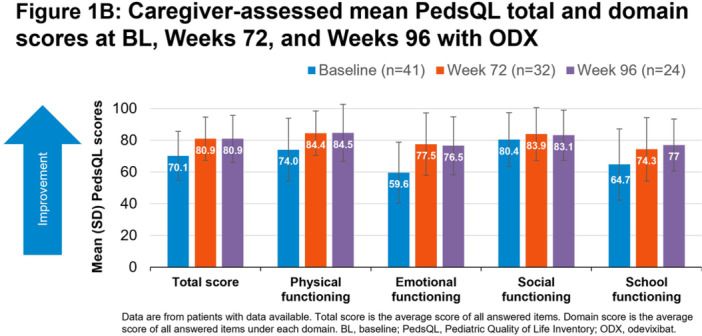


## 75* ULTRASOUND OF THE RECTUS FEMORIS MUSCLE IS ASSOCIATED WITH PEDIATRIC LIVER FAILURE ‐ PRELIMINARY ONE‐YEAR PILOT OUTCOMES DATA

*Christopher Chu* ^*2*^, *Victor Yu* ^*4*^, *Jennifer Dodge* ^*3*^, *Sindhura Kasturi* ^*4*^, *Patricia Acharya* ^*1*^, *David Rigual* ^*1*^, *Norah Terrault* ^*5*^

^*1*^ *Radiology*, *Children's Hospital Los Angeles*, *Los Angeles*, *CA*; ^*2*^ *Pediatric Gastroenterology*, *Rady Children's Hospital‐San Diego*, *San Diego*, *CA*; ^*3*^ *Research Medicine and Population and Public Health Sciences*, *University of Southern California*, *Los Angeles*, *CA*; ^*4*^ *Pediatric Gastroenterology*, *Children's Hospital Los Angeles*, *Los Angeles*, *CA*; ^*5*^ *Medicine ‐ Gastroenterology*, *University of Southern California*, *Los Angeles*, *CA*

**Background:** Sarcopenia predicts morbidity and mortality in adults with end‐stage liver disease. However, data on sarcopenia and liver‐related outcomes in children remain limited. This study evaluates the association between liver failure and ultrasound (US) measurements of the rectus femoris muscle in children with chronic liver disease (CLD).

**Methods:** A prospective corhort of participants ≤18 years of age with CLD underwent US measurement of the rectus femoris muscle for 4 sonographic parameters: cross‐sectional area (CSA), muscle thickness (MT), fascicle length (FL) and echogenic intensity (EI). Basic anthropometrics were recorded including mid‐upper arm circumference (MUAC). Liver failure status, defined as liver transplantation or death, was abstracted from participant clinical chart review up to 1 year ± 2 months from their original study measurement. The association of each US muscle parameter and liver failure were explored by logistic regression.

**Results:** A total of 66 participants were included in this study (43.9% male; median age 7.47 years (IQR 1.72‐13.65). One‐third of participants experienced liver failure. There was no significant difference in sex, BMI z‐score or ethnicity between those who had liver failure compared to those who did not. Participants with liver failure were younger and had lower MUAC z‐scores (**Table 1**). Participants with liver failure had decreased quantitative muscle parameters (CSA, MT, FL, all p<0.01), however there was no significant difference in EI, a qualitative muscle parameter. In univariable logistic regression, for every 1 standard deviation (SD) increase in mean CSA, MT and FL, the odds of liver failure decreased by 79%, 77% and 77%, respectively (p <0.001, 0.001, and 0.001, respectively). In age‐adjusted logistic regression, a similar trend was noted, however did not reach statistical significance. For every 1 SD increase in mean CSA, MT and FL, the odds of liver failure decreased by 68%, 55% and 57%, respectively (all p >0.05).

**Conclusions:** Younger age and decreased cross‐sectional area, muscle thickness and fascicle length of the rectus femoris muscle are associated with liver failure. Increasing muscle quantity (CSA, MT, FL) as measured by ultrasound are associated with decreasing odds of liver failure—although not statistically significant after adjusting for age. This study highlights the role that muscle health may have on waitlist outcomes and warrants further investigation.


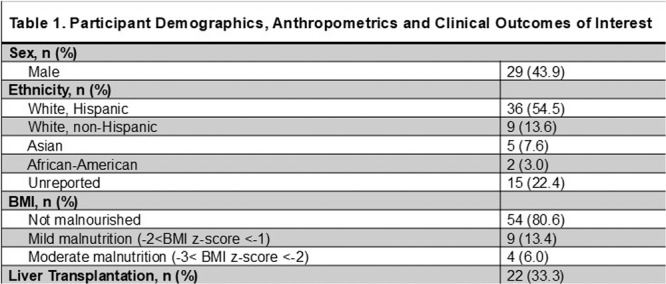



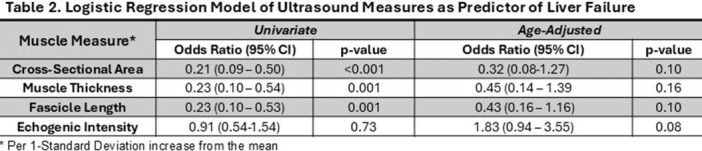


## 76* IRON METABOLISM AND FERROPTOSIS SIGNALING DURING ISCHEMIA REPERFUSION INJURY IS MODULATED BY DEFEROXAMINE IN A NOVEL NORMOTHERMIC PERFUSION PUMP SYSTEM

*Kento Kurashima* ^*1*^, *James Fox* ^*1*^, *Connor Whalen* ^*2*^, *Shaurya Mehta* ^*1*^, *Austin Sims* ^*1*^, *Chandrashekhara Manithody* ^*1*^, *Ashlesha Bagwe* ^*1*^, *Yasar Caliskan* ^*1*^, *Mustafa Nazzal* ^*1*^, *Ajay Jain* ^*1*^

^*1*^ *Pediatrics*, *Saint Louis University School of Medicine*, *Saint Louis*, *MO*; ^*2*^ *University of Notre Dame*, *Notre Dame*, *IN*

**Background:** Marginal donor livers (MDLs) have been used for liver transplantation to address major organ shortages. However, MDLs are notably susceptible to Ischemia/Reperfusion injury (IRI). Recent investigations have highlighted Ferroptosis, a new type of programmed cell death, as a potential contributor to IRI. We hypothesized that modulating ferroptosis by the iron chelator deferoxamine (DFO) could alter the course of IRI.

**Method:** Using our novel Perfusion Regulated Organ Therapeutics with Enhanced Controlled Testing (PROTECT) model (US provision Patent, US63/136,165), six human MDLs (liver A to F) were procured and split into paired lobes. Simultaneous perfusion was performed on both lobes, with one lobe subjected to DFO while the other serving as an internal control. Histology, serum chemistry, expression of ferroptosis‐associated genes, determination of iron accumulation, and measurement of lipid peroxidation, were performed.

**Results:** Histological analysis revealed severe macrovesicular steatosis (>30%) in liver A and D, while liver B and E exhibited mild to moderate macrovesicular steatosis. Majority of the samples noted mild inflammation dominantly in zone 3. No significant necrosis was noted during perfusion. Perl's Prussian blue stain and non‐heme iron quantification demonstrated a suppression of iron accumulation in liver A to D with DFO treatment (p<0.05). Based on the degree of iron chelation, 12 lobes were categorized into two groups: lobes with decreased iron (n=4) and those with increased iron (n=8). Comparative analysis demonstrated that ferroptosis‐associated genes (HIF1‐alpha, RPL8, IREB2, ACSF2, NQO1) were significantly downregulated in the former (p=0.0338, p=0.0085, p=0.0138, p=0.0138, p=0.0209, respectively). Lipid peroxidation was significantly suppressed in lobes with decreased iron (p=0.02). While serum AST was lower in iron chelated lobes this did not reach statistical significance.

**Conclusion:** This study affirmed that iron accumulation was driven by normothermic perfusion. Reduction of iron content suppressed ferroptosis‐associated genes and lipid peroxidation to mitigate IRI. Our results using human MDLs revealed a novel relationship between iron content and ferroptosis, providing a solid foundation for future development of IRI therapeutics.


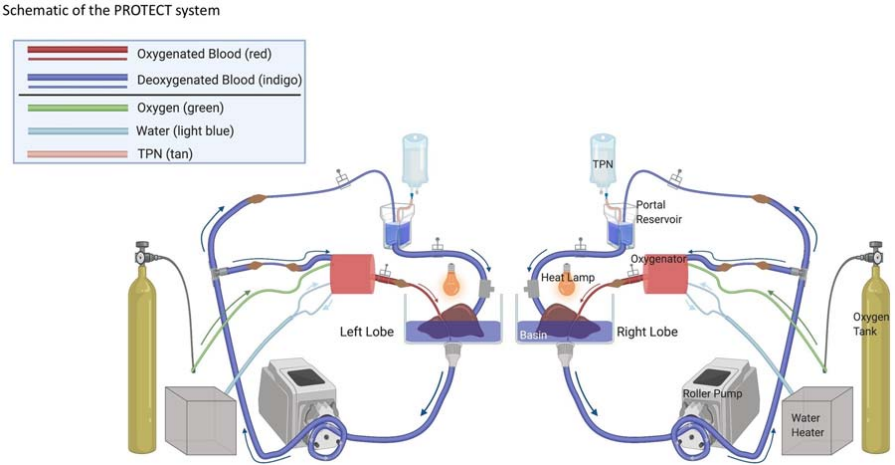



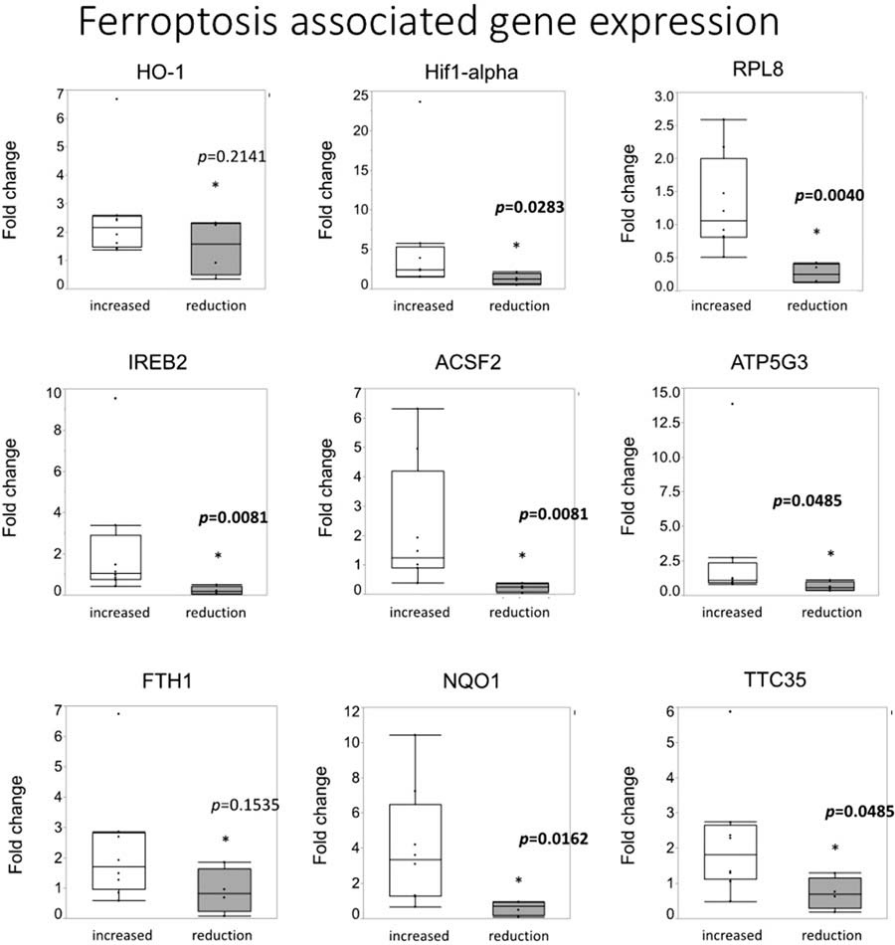


## 77* IMPROVEMENTS IN PRURITUS ARE ASSOCIATED WITH IMPROVEMENTS IN GROWTH IN PATIENTS WITH PROGRESSIVE FAMILIAL INTRAHEPATIC CHOLESTASIS: DATA FROM THE MARCH‐ON TRIAL

*Amal Aqul* ^*1*^, *Alexander Miethke* ^*2*^, *Chuan‐Hao Lin* ^*3*^, *Douglas Mogul* ^*4*^, *Tiago Nunes* ^*4*^, *Jolan Terner‐Rosenthal* ^*4*^, *Marshall Baek* ^*4*^, *Pamela Vig* ^*4*^, *Richard Thompson* ^*5*^

^*1*^ *The University of Texas Southwestern Medical Center*, *Dallas*, *TX*; ^*2*^ *Cincinnati Children's Hospital Medical Center*, *Cincinnati*, *OH*; ^*3*^ *Children's Hospital Los Angeles*, *Los Angeles*, *CA*; ^*4*^ *Mirum Pharmaceuticals Inc*, *Foster City*, *CA*; ^*5*^ *Institute of Liver Studies*, *King's College London*, *London*, *England*, *United Kingdom*

**Background:** Progressive familial intrahepatic cholestasis (PFIC) is a group of disorders resulting in disrupted bile composition and cholestasis leading to debilitating pruritus, growth deficits, vitamin deficiency, and progressive liver damage. Maralixibat, an IBAT inhibitor, is approved in the US for the treatment of cholestatic pruritus in individuals ≥12 months old with PFIC. MARCH, a Phase 3 trial of maralixibat, achieved its primary and key secondary endpoints of pruritus and sBA reduction. Long‐term maintenance of effect, including growth, for up to two years was observed in MARCH‐ON, an open‐label extension. We report on the relationship between pruritus response and growth improvements in MARCH‐ON.

**Methods:** Pruritus response was defined as a participant having ≥1 point reduction in ItchRO(Obs) from baseline to the average of the final 3 week periods of MARCH (Weeks 15‐18, 19‐22, 23‐26). Height and weight Z‐scores were analyzed. Wilcoxon signed‐rank test and rank sum test were used to determine CFB significance within groups and between groups.

**Results:** Sixty participants from the All‐PFIC cohort in MARCH‐ON were included in the analysis. Baseline characteristics were well‐balanced between groups. Growth (mean [SD]) for the pruritus responders (height z‐score: ‐1.8 [1.3]; weight‐score: ‐1.2 [1.1]) and non‐responders (height z‐score: ‐2.6 [1.2]; weight z‐score: ‐1.8 [1.5]) was stunted at Baseline. Thirty‐seven participants met the pruritus responder criteria in the All‐PFIC population and 23 were non‐responders. Pruritus responders had a significant improvement in height (mean CFB [SE]: 0.4 [0.09]; P<0.0001) and weight z‐scores (CFB: 0.38 [0.07]; P<0.0001) at Week 26, sustained out to 70 weeks of MRX treatment in MARCH‐ON (height CFB: 0.53 [0.10]; P<0.0001; weight CFB: 0.51 [0.15]; P=0.001). Significant differences between pruritus responders and non‐responders were sustained out to 70 weeks in height (CFB Δ: +0.63; P=0.0049) and numeric improvements in weight (CFB Δ: +0.52; P=0.11).

**Conclusions:** These consistent trends in growth for participants on maralixibat who were pruritus responders indicate a potential disease‐modifying effect of maralixibat treatment in PFIC.

## 84 THE UTILIZATION AND ACCURACY OF FIBROSCAN IN PEDIATRIC LIVER TRANSPLANT AT A SINGLE TERTIARY CENTER

*Chaowapong Jarasvaraparn* ^*3*^, *Iván González* ^*2*^, *Brendan Anderson* ^*1*^, *Ishanpreet Sran* ^*1*^, *Kyla Tolliver* ^*3*^, *Jean Molleston* ^*3*^

^*1*^ *Medicine*, *Indiana University School of Medicine*, *Indianapolis*, *IN*; ^*2*^ *Department of Pathology and Laboratory Medicine*, *Indiana University School of Medicine*, *Indianapolis*, *IN*; ^*3*^ *Division of Pediatric Gastroenterology, Hepatology and Nutrition*, *Indiana University School of Medicine*, *Indianapolis*, *IN*

**Background:** Liver biopsy remains the gold standard to evaluate fibrosis. However, it is invasive and associated with complications. Vibration‐Controlled Transient Elastography (VCTE) utilizing FibroScan allows for non‐invasive measurement of liver fibrosis in liver transplant (LT) recipients. This study investigated the utilization and accuracy of FibroScan in pediatric liver transplant recipients.

**Method:** In a retrospective analysis of children (≤18 years old) with LT who underwent at least one FibroScan between January 2020 and December 2024, we investigated the correlation of biomarkers such as AST‐to‐platelet ratio index (APRI), Fibrosis‐4 (FIB‐4), Controlled Attenuation Parameter (CAP), and Liver Stiffness Measurement (LSM) from FibroScan with severity of steatosis and fibrosis on liver biopsy. All laboratory tests and liver biopsies were obtained within 6 months of FibroScan. Advanced liver disease (ALD) was defined as bridging fibrosis and/or cirrhosis (fibrosis score 3‐4) on liver biopsy. Our pathology team (IG, IS) retrospectively reviewed the historical liver slides and were blinded for clinical information.

**Results:** A total of 166 FibroScans from 74 children with LT (13 split and 61 whole liver) were included. CAP did not significantly correlate with BMI. 47 children underwent liver biopsies. LSM was significantly correlated with ALD (Pearson correlation 0.63, p =0.001), but not APRI and FIB‐4. LSM greater than 7.85 kPa predicted ALD at AUC 0.96 with 99% sensitivity and 84% specificity (p = 0.001). Eleven children with CAP ≥225 dB/m did not have any biopsy evidence of steatosis or steatohepatitis. CAP correlated with LSM and liver fibrosis score from liver biopsy (0.62, p = 0.001, 0.41, p = 0.05, respectively). Split liver recipients had significantly higher LSM than whole liver recipients (10.7 vs. 5.9 kPa, p = 0.001).

**Conclusions:** VCTE is a practical tool to assess graft fibrosis in children with LT and performs better than APRI and FIB‐4. CAP may not be useful to assess steatosis in pediatric liver grafts. We speculate that VCTE can be useful for graft fibrosis surveillance in children with LT.

## 85 IMPLEMENTATION SCIENCE‐GUIDED ADOPTION OF THE 2023 CYSTIC FIBROSIS HEPATOBILIARY SCREENING GUIDELINES

*Alaa Abdelghani*, *Emily Perito*, *Addison Cuneo*

*Pediatric Gastroenterology*, *University of California San Francisco*, *San Francisco*, *CA*

**Background:** In 2023, the Cystic Fibrosis (CF) Foundation issued guidelines to improve early detection of CF hepatobiliary disease (CFHBD) through routine screening. However, clinical implementation remains limited. At our center, 18% of people with CF (PwCF) missed recommended annual labs, and 80% missed screening abdominal ultrasounds. Using an implementation science (IS) framework, we identified barriers, facilitators, and targeted strategies to improve CFHBD guideline adoption at our pediatric CF center.

**Methods:** We conducted a phased qualitative study using the Behavior Change Wheel (BCW)/COM‐B model. In Phase 1, pre‐implementation interviews with CF clinic team members identified barriers and facilitators. Phase 2 used these findings to design interventions, selected via APEASE criteria (Affordability, Practicality, Effectiveness, Acceptability, Safety, Equity). Phase 3 involved a chart review to assess changes in screening from baseline. Figure 1 summarizes the phases.

**Results**:

**Phase 1:** Pre‐Implementation Assessment: Qualitative interviews (n=10, 2 gastroenterologists, 3 pulmonologists, 2 nurses, 2 pharmacists and 1 patient navigator) revealed key barriers and facilitators to guideline implementation. Barriers identified included (1) limited knowledge among non‐GI team members, (2) systemic and workflow challenges such as scheduling complexity and time constraints during clinic visits, and (3) perceived low patient/family awareness of CFHBD. Facilitators included strong team communication, leadership support, and potential electronic medical record (EMR) tools to streamline care.

**Phase 2:** Strategy Selection and Implementation: Based on findings from Phase 1 and using the APEASE criteria, implementation strategies targeted four domains: **(1)** Education: Two tiered educational modules (basic and in‐depth) were delivered across team huddles and departmental conferences. Patient/family education materials were also developed. **(2)** EMR Enhancements: We integrated CFHBD screening into EMR checklists‐ using “Epic specialty snapshot”, note structures, including hard stops so providers are prompted to review obtaining screening US and blood tests for all PwCF. And smart phrases for the GI notes to automatically include recent results of CFHBD screening to the clinic notes guideline adherence. **(3)** Scheduling Optimization: we coordinated same‐day ultrasounds and consolidated lab work to reduce patient burden. **(4)** QI: PDSA cycles tracked progress and facilitated iterative refinement of interventions, ensuring ongoing alignment with team and patient needs (Figure 2).

**Phase 3:** Our cohort included 73 PwCF. All, except for one child, completed the annual laboratory screening bundle as recommended by the 2023 CFHBD guidelines increased from a baseline of 82%. Among 62 PwCF aged ≥3 years, 53 (85%) completed at least one abdominal US and 29 (46.8%) underwent at least one elastography. An improvement from a baseline of 20% for abdominal US.

**Conclusion:** Using IS approach, we identified knowledge gaps and workflow issues as key barriers. A multidisciplinary approach—engaging GI providers and EMR tools—enabled targeted interventions, including team education and workflow changes. This led to improved adherence: lab completion rose from 82% to 99%, and ultrasound screening from 20% to 85%.


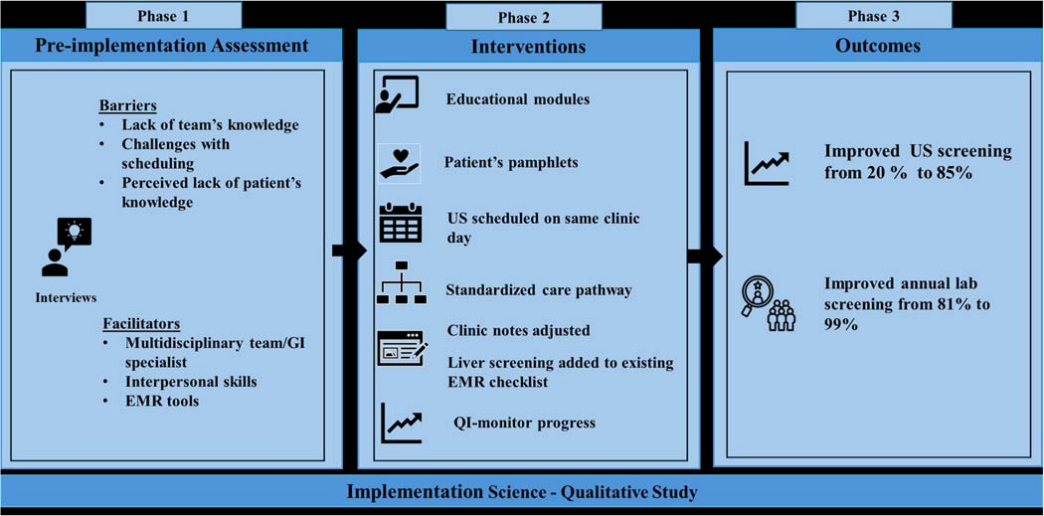


**Figure 1:** Study Phases


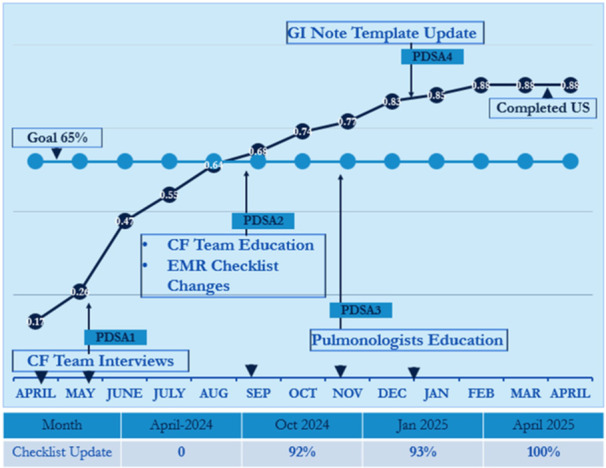


**Figure 2:** PDSA cycles timeline showing an increase in abdominal ultrasound completion over 9 months. EMR checklists were updated for all PwCF at our center.

## 86 LARGE‐SCALE PROTEOMIC STUDY REVEALS TOTAL SERUM IGG AS A CANDIDATE NOVEL DIAGNOSTIC BIOMARKER IN GESTATIONAL ALLOIMMUNE LIVER DISEASE

*Katie Conover* ^*1*^, *Priya Rolfes* ^*1*^, *Kathryn Kilpatrick* ^*2*^, *Anthony Saviola* ^*2*^, *Sarah Taylor* ^*1*^

^*1*^ *Pediatrics*, *University of Colorado Anschutz Medical Campus*, *Aurora*, *CO*; ^*2*^ *University of Colorado Anschutz Medical Campus*, *Aurora*, *CO*

**Background:** Gestational alloimmune liver disease (GALD) is a leading cause of neonatal acute liver failure (ALF) with high mortality, particularly if treatment with intravenous immunoglobulin (IVIG) and exchange transfusion (ET) is not initiated early. However, diagnosis of GALD remains challenging. We aim to identify novel non‐invasive diagnostic biomarkers for GALD.

**Methods:** We obtained serum samples from GALD (n=10) and non‐GALD (n=15) cases with neonatal ALF from the NIDDK Pediatric Acute Liver Failure study biorepository. Mass spectrometry and IgG enzyme‐linked immunosorbent assay (ELISA) were run on all samples. Proteomic analysis (MetaboAnalyst, Enrichr) was conducted in patients without prior IVIG or ET treatment (n=18); proteomic analysis by outcome included the full cohort (n=25). Statistical analysis was performed using Pearson's chi‐squared, Mann‐Whitney *U* and Student's t‐test.

**Results:** Age of liver failure onset and serum collection did not differ between groups (p>0.05 for both). Although not reaching statistical significance, a greater proportion of GALD patients died (67% vs 30%) and fewer were transplanted (0% vs 30%). After excluding post‐IVIG/ET cases, GALD cases demonstrated robust upregulation of immunoglobulin heavy and light chains (**Figure 1 A**). ELISA confirmed significant increase in total serum IgG in untreated GALD cases (p=0.005) with an area under receiver operating curve of 0.903 to predict GALD vs non‐GALD ALF (p=0.007, **Figure 1B,C**). Importantly, IgG level did not correlate with age (r=‐0.07, p=0.74). Pathway analysis showed upregulation of classical antibody‐mediated complement activation pathways in GALD (adjusted p=3.9e‐13) and scavenging by class A receptors in non‐GALD (adjusted p=1.2e‐6). Several proteins reflecting liver synthetic and regenerative function, including factor levels, alpha‐fetoprotein, angiotensinogen and fibrinogen were significantly decreased in GALD cases with poor outcome (raw p<0.05).

**Conclusions:** Differentially expressed serum proteins between GALD and non‐GALD neonatal ALF reflect understood pathogenic mechanisms involving complement activation and decreased liver synthetic proteins particularly in GALD infants with poor outcome. Total serum IgG may be significantly increased in GALD due to maternal placental transfer of pathogenic antibodies that destroy fetal hepatocytes. Total serum IgG should be explored prospectively as a non‐invasive GALD diagnostic biomarker.


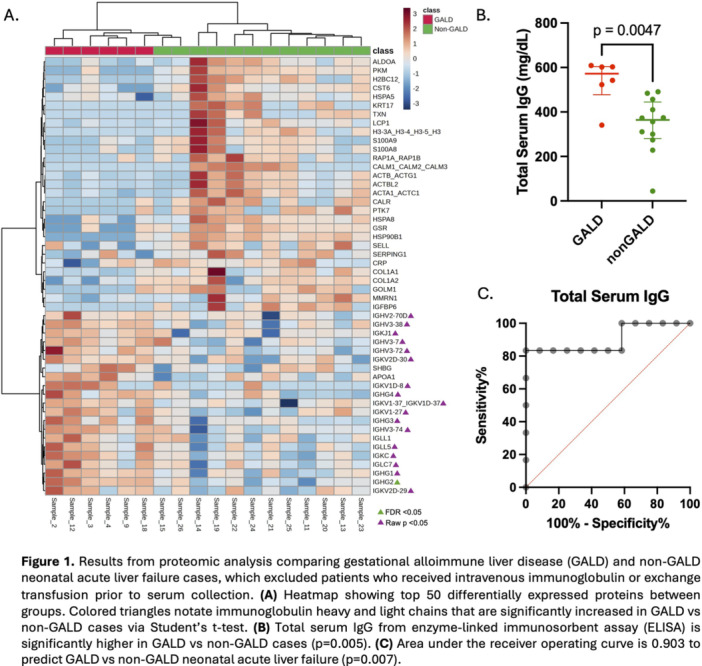


## 87 ANALYSIS OF PATIENT REPORTED OUTCOME MEASURES USING THE PEDIATRIC LIVER TRANSPLANT QUALITY OF LIFE (PELTQL) QUESTIONNAIRE: A PITTSBURGH EXPERIENCE

*Maria Amendola* ^*1*^, *Vikram Raghu* ^*1*^, *Mary Ayers, MD* ^*1*^, *George Mazariegos, MD* ^*2*^, *Daniel Pieratt* ^*2*^, *James Squires* ^*1*^

^*1*^ *Pediatric Gastroenterology, Hepatology and Nutrition*, *UPMC Children's Hospital of Pittsburgh*, *Pittsburgh*, *PA*; ^*2*^ *Pediatric Transplant*, *UPMC Children's Hospital of Pittsburgh*, *Pittsburgh*, *PA*

I**NTRODUCTION:** Past research in pediatric liver transplant (pLTx) has improved outcomes but mostly focuses on transplant rejection or graft failure and not real‐world outcomes affecting patients. More recently, there has been a shift toward determining patient reported outcomes (PROs) like quality of life (QoL). Here, we report an institutional experience of QoL assessments using a transplant‐specific validated tool – the PeLTQL.

**METHODS:** pLTx recipients, aged 8‐18 years old, completed a self PeLTQL questionnaire at annual transplant clinic at least 1‐year post‐transplant. The PeLTQL is a 26‐item questionnaire that addresses three different subdomains (Future Health, FH; Coping & Adjustment, CA; Social‐Emotional, SE). The responses are in the form of a reverse Likert scale (1‐5) with the highest points given for “1” responses. Values are then weighted to yield subdomain and total scores (0‐100). Proxy versions were completed by their guardian. Surveys were collected between March 2022 and May 2025 at UPMC Children's Hospital of Pittsburgh. Total and subdomain PeLTQL scores were analyzed and areas of concern were identified by recording which questions had the highest percentage of responses designated as “5”.

**RESULTS:** A total of 53 patients were included in the final cohort. The most common indication for transplant was Biliary Atresia (45.28%).The mean age at the time of pLTx was 1.3 years. The most common transplant was a living donor left lateral segment (41.8%), followed by a deceased whole liver (27.3%). The mean age at the time of the latest completed PeLTQL was 10.4 years. The subdomain with the lowest median score for self‐responses was Coping and Adjustment (75), while the Social and Emotional subdomain was the lowest for proxy respondents (69.44). The questions that had the highest number of “5” responses involved worrying about missing medications (16.9%) as well as ease of talking about feelings (13.2%). For the proxy respondents, the questions with the most “5 s” were worrying about staying away from others due to illness and concentration issues (9.43%). Eight patients (15%) in the cohort were identified as “at‐risk” for anxiety (scoring lower than 62.5 on the PeLTQL). Of the patients who were identified as “at‐risk” in the study population, 50% (n=4) had discordant scores with their proxies (proxy score was > 62.5). Five patients (9.1%) in the cohort were found to be “at risk” for depression (total PeLTQL score < 49.3). Every patient at risk for depression had a discordant score from their proxy (proxy response was > 49.3). The highest scoring questions of patients with living donor transplants were ease of talking about their feelings and worrying about missing medicine doses (20%). The highest scoring questions amongst patients with deceased donor transplants were worrying about family money problems related to their transplant (14.28%) and worrying about missing medicine doses (10.71%).

**CONCLUSIONS:** Successful PeLTQL implementation in annual transplant clinic can highlight unique areas to improve the lives of pLTx recipients not captured by traditional outcome measures. Discordance between patient and proxy reports, especially in high‐risk patients, supports the need for comprehensive routine monitoring. Unique to our study, different concerns between living donor and deceased donor recipients' responses highlight how donor sources and environmental aspects may play a role in a patient's post‐transplant QoL.


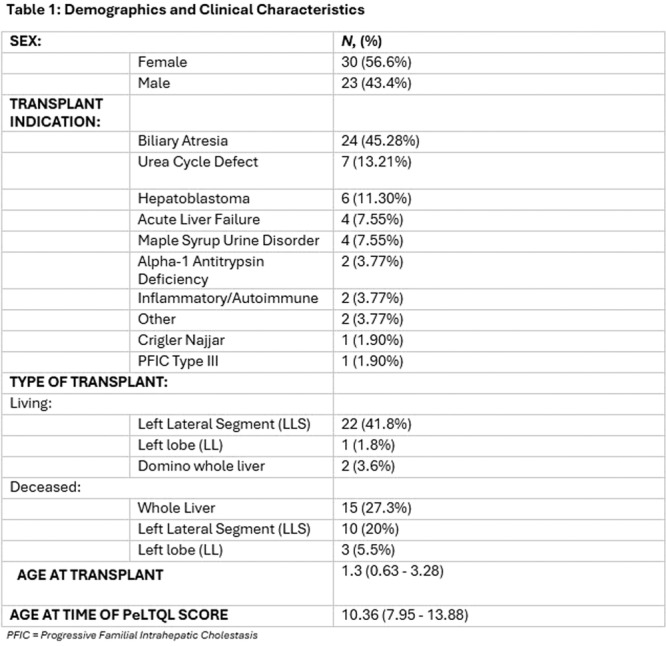


Table 1: Demographics and Clinical Characteristics


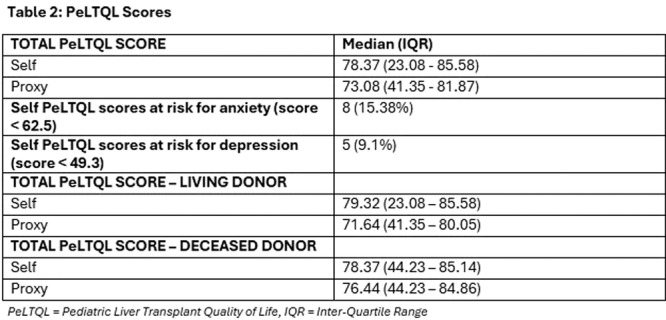


Table 2: PeLTQL Scores

## 90 COMPREHENSIVE ANALYSIS OF CHOLESTASIS GENETIC PANEL RESULTS FROM 2016 TO 2022 IN CHILDREN AND YOUNG ADULTS: INSIGHTS INTO DIAGNOSTIC YIELD

*Brett Hoskins* ^*1*^, *Tiziano Pramparo* ^*2*^, *Ethan Gough* ^*3*^, *Amy Ponte* ^*5*^, *Jolan Terner‐Rosenthal* ^*2*^, *Rana Dutta* ^*2*^, *Wikrom Karnsakul* ^*4*^

^*1*^ *Division of Pediatric Gastroenterology, Hepatology and Nutrition, Department of Pediatrics*, *Riley Hospital for Children, Indiana University School of Medicine*, *Indianapolis*, *IN*; ^*2*^ *Mirum Pharmaceuticals Inc*, *Foster City*, *CA*; ^*3*^ *Department of Biostatistics, Epidemiology, and Data Management (BEAD) Core*, *The Johns Hopkins University Bloomberg School of Public Health*, *Baltimore*, *MD*; ^*4*^ *Division of Pediatric Gastroenterology, Hepatology, and Nutrition, Department of Pediatrics*, *The Johns Hopkins University School of Medicine*, *Baltimore*, *MD*; ^*5*^ *Sanofi*, *Bridgewater*, *NJ*

**Background:** Cholestasis can arise from various underlying causes, with genetic factors playing a crucial role. Diagnosis is often challenging due to a wide range of clinical presentations and overlap between genetic conditions. Utilizing a cholestasis gene panel through next‐generation sequencing offers a more efficient and timely method for identifying genetic causes. This abstract summarizes results from over 10,000 cholestasis panel tests, providing valuable insights into the diagnostic yield.

**Methods:** Aggregate data from a 77‐gene cholestasis panel (initially 57 genes with 9 and 11 genes added in 2017 and 2022, respectively) were reviewed. Patients eligible for analysis had undergone genetic testing between February 2016 and February 2022 due to either a current or previous history of cholestasis without an identifiable cause or unexplained chronic liver disease. DNA sequencing was performed utilizing custom‐designed and optimized capture libraries [SureSelect by Eurofins/Emory Genetics Laboratory (2016–2021) and PGxome® by PreventionGenetics (2021–2024)]. Variants were classified according to diagnostic laboratory guidelines and ACMG criteria as benign, likely benign, variant of unknown significance (VUS), likely pathogenic (LP), or pathogenic (P). Definite diagnoses were assigned for genes with either two pathogenic or likely pathogenic alleles (either homozygous or compound heterozygous), or for cases with a single P or LP allele in *JAG1* or *NOTCH2*. Potential diagnoses included genes with one P or LP allele along with one VUS, or one VUS in *JAG1* or *NOTCH2*.

**Results:** A total of 10,894 samples were analyzed between February 2016 and February 2022. Most patients (51.1%) were younger than one year of age at the time of testing, and 9.2% were 18 years of age or older. Within the completed tests, there were 2,400 and 455 subjects with at least one P or LP variant, respectively. The overall diagnostic yield was 3.8% and 2.3% among subjects with a definitive diagnosis (411/10,894) and a potential diagnosis (256/10,894), respectively. Definitive diagnoses were most common in genes JAG1 (n=197), ABCC2 (n=41), POLG (n=31), NOTCH2 (n=25), and ABCB11 (n=24). Genes with the most potential diagnoses included ABCC2 (n=51), ABCB4 (n=41), ABCB11 (n=41), CFTR (n=26), and PKHD1 (n=17). Monoallelic variants occurred most often in genes SERPINA1 (n=705), CFTR (n=379), DHCR7 (n=158), ABCB4 (n=115), ABCC2 (n=109).

**Conclusions:** Data from 6 years of cholestasis next‐generation sequencing panels highlight its critical role in diagnosing and identifying complex genetic variants associated with cholestasis. This was especially beneficial for infants under 1 year of age, facilitating early detection. This panel has proven valuable in providing clearer insights into the condition's genetic basis, facilitating more accurate diagnoses and potential therapeutic strategies.

## 91 PROTEOMICS USED IN IDENTIFYING NOVEL CORRELATES OF DISEASE IN PEDIATRIC METABOLIC DYSFUNCTION‐ASSOCIATED STEATOTIC LIVER DISEASE

*Diego Paine‐Cabrera* ^*2*^, *Lisa Harvey* ^*1*^, *Michele Pritchard* ^*2,3*^, *John Thyfault* ^*4,5*^, *Antonio Artigues* ^*6*^, *Udayan Apte* ^*2,3*^, *Voytek Slowik* ^*1,5*^

^*1*^ *Pediatrics*, *Children's Mercy Kansas City*, *Kansas City*, *MO*; ^*2*^ *Department of Pharmacology, Toxicology and Therapeutics*, *The University of Kansas Medical Center*, *Kansas City*, *KS*; ^*3*^ *University of Kansas Liver Center*, *The University of Kansas Medical Center*, *Kansas City*, *KS*; ^*4*^ *Department of Molecular and Integrative Physiology*, *The University of Kansas Medical Center*, *Kansas City*, *KS*; ^*5*^ *Children's Center for Healthy Lifestyles and Nutrition*, *Kansas City*, *MO*; ^*6*^ *Department of Biochemistry and Molecular Biology*, *The University of Kansas Medical Center*, *Kansas City*, *KS*

Metabolic dysfunction‐associated liver disease (MASLD) is a leading cause of liver disease in children. There is a paucity of data on potential biomarkers and therapeutic targets, especially in pediatric MASLD. We used mass spectrometry (MS)‐mediated proteomics followed by enzyme‐linked immunosorbent assay (ELISA) to identify potential biomarkers and therapeutic targets in pediatric MASLD in pediatric MASLD.

Serum samples were collected from pediatric subjects without (n=56) and with MASLD (n=72). Initial screen using MS‐based proteomics identified 6 upregulated (adenosine deaminase 2, sex hormone‐binding globulin (SHBG), inter‐alpha‐trypsin inhibitor heavy chain H1 (ITIH1), fructose‐bisphosphate aldolase A, type II cytoskeletal 2 epidermal keratine, N‐acetylmuramoyl‐L‐alanine amidase) and 3 downregulated (alcohol dehydrogenase 4 (ADH4), fructose‐bisphosphate aldolase B (ALDOB), serum albumin) proteins in the MASLD group. Confirmatory studies using ELISA were performed for the 2 strongest upregulated proteins (SHBG and ITIH1) and two top downregulated proteins (ADH4 and ALDOB). Correlation of ELISA results with clinical data revealed that SHBG had strong associations with BMI, ALT, and HgbA1c (p < 0.05). ADH4 had strong associations with BMI and HgbA1c (p < 0.05). ITIH1 and ALDOB had no strong correlations with common clinical paramenters of MASLD. Area under ROC Curve revealed statistically significant ability of SHBG (494 nmol/L, sensitivity = 98%, specificity 80%) and ADH4 (2.14 ng/mL, sensitivity = 65%, specificity = 66%) to diagnosis MASLD (p < 0.05).

MS with confirmation ELISA identified SHBG and ADH4 as potential biomarkers of pediatric MASLD.

## 92 REAL‐WORLD EFFICACY AND SAFETY OF GLECAPREVIR/PIBRENTASVIR IN CHILDREN AGED 3 TO 11 YEARS WITH CHRONIC HEPATITIS C VIRUS INFECTION: A PROSPECTIVE MULTICENTER STUDY IN JAPAN

*Tatsuki Mizuochi* ^*1*^, *Daiki Abukawa* ^*2*^, *Ayano Inui* ^*3*^, *Yoshihiro Azuma* ^*4*^, *Takako Suzuki* ^*5*^, *Hiroko Yagi* ^*6*^, *Hideki Kumagai* ^*7*^, *Sotaro Mushiake* ^*8*^, *Daisuke Tokuhara* ^*9*^, *Naoya Tsumura* ^*1*^, *Ken Kato* ^*1*^, *Hitoshi Tajiri* ^*8,9*^

^*1*^ *Pediatrics and Child Health*, *Kurume University School of Medicine*, *Kurume*, *Japan*; ^*2*^ *Gastroenterology and Hepatology*, *Miyagi Children's Hospital*, *Sendai*, *Japan*; ^*3*^ *Pediatric Hepatology and Gastroenterology*, *Saiseikai Yokohamashi Tobu Hospital*, *Yokohama*, *Japan*; ^*4*^ *Pediatrics*, *Yamaguchi University Graduate School of Medicine*, *Ube*, *Japan*; ^*5*^ *Pediatrics*, *Nagoya University Graduate School of Medicine*, *Nagoya*, *Japan*; ^*6*^ *Pediatrics*, *Hirosaki University Graduate School of Medicine*, *Hirosaki*, *Japan*; ^*7*^ *Pediatrics*, *Jichi Medical University*, *Shimotsuke*, *Japan*; ^*8*^ *Pediatrics*, *Kindai University Nara Hospital*, *Ikoma*, *Japan*; ^*9*^ *Pediatrics*, *Wakayama Medical University*, *Wakayama*, *Japan*

**Background & Aim:** Part 2 of the DORA study, a global clinical trial investigating glecaprevir and pibrentasvir (G/P) in children aged 3–11 years with chronic hepatitis C virus (HCV) infection, demonstrated high efficacy and favorable safety outcomes. However, real‐world data in this pediatric population remain scarce. This prospective, multicenter study aimed to evaluate the real‐world efficacy and safety of G/P in children aged 3‐11 years with chronic HCV infection in Japan.

**Methods:** Children aged 3–11 years with confirmed chronic HCV infection were prospectively enrolled between January 2023 and March 2024. All participants received once‐daily oral G/P for either 8 or 12 weeks. The primary efficacy endpoint was sustained virologic response at 12 weeks after treatment completion (SVR12). Safety assessments included monitoring of adverse events, laboratory parameters, and growth metrics.

**Results:** Eighteen children (8 female) with a median age of 9 years (range: 3–11) were enrolled from 9 pediatric centers in Japan. The HCV genotype distribution was as follows: genotype 1b (n=3), 2a (n=8), 2b (n=5), 3a (n=1), and one case with an unclassified subtype of genotype 2. All patients were treatment‐naïve and completed therapy (17 received 8 weeks, 1 received 12 weeks). SVR12 was achieved in 17 of 18 patients (94%). Adverse events were predominantly mild, and no serious adverse events were reported. Treatment was associated with significant reductions in serum alanine aminotransferase and *Wisteria floribunda* agglutinin‐positive Mac‐2 binding protein levels. No negative impact on growth was observed.

**Conclusions:** In this real‐world, multicenter cohort, G/P treatment demonstrated high virologic efficacy and was well tolerated in children aged 3‐11 years with chronic HCV infection. These findings support the use of G/P as an effective treatment option in routine clinical practice for pediatric patients with HCV.

## 93 METABOLOMIC PROFILING IN PEDIATRIC METABOLIC DYSFUNCTION‐ASSOCIATED STEATOTIC LIVER DISEASE (MASLD)

*Shatha Qarooni* ^*1*^, *Caroline Blakley* ^*1*^, *Kevin Short* ^*2*^, *Sirish Palle* ^*1*^

^*1*^ *Pediatric Gastroenterology*, *Oklahoma Children's Hospital*, *Oklahoma City*, *OK*; ^*2*^ *Pediatric Endocrinology*, *Oklahoma Children's Hospital*, *Oklahoma City*, *OK*

**Background:** Alternation in circulating metabolomics is often found in Metabolic Dysfunction‐Associated Steatotic Liver Disease (MASLD). While Liver biopsy remains the gold standard for diagnosing MASLD, Glutamate/Serine+Glycine (GSG Index) and Branched Chain Amino Acids (BCAA) are biomarkers known to correlate with MASLD in adults. Few prior studies were done in children lacking the association with histopathological features. We are hypothesizing that circulating Amino Acids (AA), Bile Acids (BA) and/or Fatty acids (FA) can be better predictors of MASLD in pediatric age group compared to the standard alanine transaminase (ALT).

**Methods:** We compared three groups aged 12‐20 years (n/101, F/M= 38/63)1) Obese individuals with biopsy confirmed MASLD (MASLD, n=45, F/M=12/33), 2) Obese control (OB, n=27, F/M=14/13) and 3) Normal weight control (NW, n=29 F/M=12/17). Ultra‐performance liquid chromatography‐mass spectrometry (UPLC‐MS) was used to measure the concentrations of plasma AA and BA and Gas Chromatography‐Mass Spectrometry (GC‐MS) was used to measure plasma FA.

**Results:** The GSG index was higher in MASLD group (p =< 0.001) strongly correlating with trunk fat (r = 0.60, p = <0.001) and insulin sensitivity (r =‐0.40, p = <0.001). No significant difference was found in BCAA between the 3 groups. Among the tested FA, Palmitoleic Acid (MUFA, 16:1) and alpha Linolenic were significantly higher in the MASLD group (p =< 0.001).

**Conclusion:** The results suggest that GSG index can be a promising biomarker for predicting MASLD in children independent from body mass index (BMI) while still strongly correlating with trunk fat and insulin sensitivity. Larger studies are needed to determine the association between GSG level and severity of MASLD on histopathology.

## 94 GLP‐1 RECEPTOR AGONISTS REDUCE THE RISK OF INCIDENT FATTY LIVER CHANGES IN ADOLESCENTS WITH OBESITY

*Anisha Lobo* ^*3*^, *Shradha Chhabria* ^*3*^, *Priya Mohan* ^*3*^, *Roshan Patel* ^*1*^, *Jaime Pérez* ^*2*^, *Elleson Harper* ^*2*^, *Courtney Batt* ^*3*^

^*1*^ *Cleveland Clinic*, *Cleveland*, *OH*; ^*2*^ *University Hospitals Rainbow Babies & Children's Hospital*, *Cleveland*, *OH*; ^*3*^ *Pediatrics*, *University Hospitals Rainbow Babies & Children's Hospital*, *Cleveland*, *OH*

**Background:** Metabolic dysfunction‐associated steatotic liver disease (MASLD) has become a common cause of chronic liver disease in adolescents, paralleling rising rates of obesity and metabolic syndrome. As rates of obesity have risen, so has the incidence of fatty liver changes in the adolescent population. Although often asymptomatic, MASLD in youth can progress to fibrosis and long‐term hepatic complications. Adequate weight management may help reduce the rising incidence of fatty liver changes in adolescents. There is a paucity of randomized control trials comparing the efficacy of anti‐obesity medications (AOM) and GLP‐1 receptor agonists (GLP‐1 RA) in this population. Our study aims to investigate the risk of developing fatty liver changes amongst adolescents with obesity who are treated with GLP‐1 RA versus other AOM, five years after treatment initiation.

**Methods:** A large retrospective, multicenter database study was conducted using TriNetX. We utilized ICD‐10 codes to identify adolescents aged 10–19 years with obesity but without fatty liver changes. Patients included in the study were prescribed either GLP‐1 RA including semaglutide, liraglutide, or tirzepatide or AOM including orlistat, metformin, topiramate, phentermine, bupropion, or naltrexone. Two cohorts were formed based on the class of pharmacologic treatment. Baseline characteristics between the cohorts were compared using student's t‐test. Demographic and comorbidity data were used for 1:1 propensity score matching. Risk difference for developing incident fatty liver changes or non‐alcoholic steatohepatitis (NASH) was calculated at 5 years following initiation of either GLP‐1 RA or other AOM.

**Results:** Patients prescribed GLP‐1 RA (n=5,719) and those prescribed other AOM (n=23,700) were propensity score matched, yielding 5,719 patients in each cohort. Amongst the GLP‐1 RA cohort, 657 patients were excluded and amongst the AOM cohort, 382 patients were excluded because they had a diagnosis of fatty liver changes or NASH prior to the index event. Mean age of the patients was 15.9 years in both groups. Mean baseline BMI among those using GLP‐1 RA was 42.2 kg/m^2^ and among those using other AOM was 40.4 kg/m^2^. Among adolescents aged 10‐19 with obesity, use of a GLP‐1 RA was associated with a reduced risk of developing fatty liver changes (3.7% vs 4.6%; RD = ‐0.009; 95% CI [‐0.017, ‐0.002]) over 5 years compared to use of other AOM.

**Conclusion:** The use of GLP‐1 RA amongst adolescents with obesity was associated with a lower risk of developing fatty liver changes compared to other AOM five years after initiating treatment. Randomized prospective studies are needed to investigate the role of GLP‐1 RA in the prevention of obesity‐related comorbidities in the adolescent population.

## 103 RESOLUTION OF LIVER FIBROSIS AND CLINICAL IMPROVEMENT IN FOUR SIBLINGS WITH LYSOSOMAL ACID LIPASE DEFICIENCY FOLLOWING NINE YEARS OF CONTINUOUS ENZYME REPLACEMENT THERAPY

*Hernando Lyons* ^*1,2*^, *Premchand Anne* ^*2*^, *Sarah Lonardo* ^*3*^, *Sanjay Kumar* ^*1,2*^

^*1*^ *Pediatric Gastroenterology*, *Henry Ford St John Children's Hospital*, *Detroit*, *MI*; ^*2*^ *Pediatrics*, *Wayne State University School of Medicine*, *Detroit*, *MI*; ^*3*^ *Pathology*, *Henry Ford St John Hospital*, *Detroit*, *MI*

**Background:** Lysosomal acid lipase deficiency (LAL‐D) is a rare, autosomal recessive, lysosomal storage disorder characterized by an accumulation of cholesteryl esters and triglycerides in the liver, spleen, and macrophages throughout the body. The liver pathology in LAL‐D is characterized by predominantly microvesicular steatosis and progressive fibrosis that leads to cirrhosis and liver failure. The enzyme replacement with sebelipase alpha (SA) has shown clinical improvement in LAL‐D patients. We present 4 male siblings with LAL‐D who had clinical improvement and resolution of hepatic fibrosis after nine years of enzyme replacement.

**Methods:** Four male biological brothers (Current ages 19, 21, 24 and 26 years) from the same non‐consanguineous parents were diagnosed with the late onset phenotype of LAL‐D in 2015 by DNA sequencing of LIPA gene. All patients were start on SA through IV route at 1 mg/kg every 2 weeks. The SA dose was increased to 2 mgs/kg, IV, every other week after two and a half years in 2 of the siblings (19 and 24 years old), and also was increased to 2 mg/kg, IV, every 2 weeks after 6 years of treatment in the 26 year old because of insufficient improvement in the dyslipidemia. This was based on the recommendations of the national cholesterol education program expert panel cut‐offs for acceptable LDL‐cholesterol level of less than 110 mg/dl. Serum lipids, lipoprotein level, transaminases were assessed at baseline and sequentially every 16 weeks for 9 years. Liver biopsies were performed at baseline at two years and nine years of therapy. Hepatic steatosis was evaluated from hematoxylin‐eosin‐stained specimens using the modified Brunt classification as the percentage of area of the liver parenchyma occupied by lipid vacuoles (1=5‐33%, 2=34‐66%, 3=>66%). Fibrosis was scored utilizing the Metavir scoring system (F0= no fibrosis, F1=portal fibrosis without septa, F2=portal fibrosis with few septa, F3=numerous septa without cirrhosis, F4=cirrhosis).

**Results:** All 4 siblings had significant improvement in their serum lipids, transaminases, steatosis and resolution of hepatic fibrosis following treatment with SA for 9 years. Alanine aminotransferase (ALT) and aspartate aminotransferase (AST) decreased from baseline by an average of 50% and 59% respectively. The fasting triglycerides and low‐density lipoprotein cholesterol (LDL‐C) levels decreased from baseline by an average of 45% and 50%, respectively. Hepatic steatosis decreased from baseline of grade 3 to grade 1 post‐treatment. Hepatic fibrosis decreased from baseline stage F2‐3 to stage F0.

**Conclusions:** Treatment with SA for 9 years in 4 siblings with LAL‐D demonstrated improvement in serum lipid levels, transaminases, hepatic steatosis and resolution of fibrosis.

## 104 SURVEILLANCE OF FONTAN ASSOCIATED LIVER DISEASE IN CHILDHOOD AND ADOLESCENCE:A SINGLE‐CENTER EXPERIENCE IN LATIN AMERICA

*Maria Sanchez* ^*1*^, *Maris Belen Pallitto* ^*1*^, *Natalia Napoli* ^*2*^, *Victoria Fernandez de Cuevas* ^*1*^, *Gustavo Boldrini* ^*1*^, *Maria Loreno Cavalieri* ^*1*^, *Daniel D'Agostino* ^*1*^

^*1*^ *Pediatric Gastroenterology and Hepatology Unit*, *Hospital Italiano de Buenos Aires*, *Buenos Aires*, *Buenos Aires*, *Argentina*; ^*2*^ *Pediatric Cardiology*, *Hospital Italiano de Buenos Aires*, *Buenos Aires*, *Buenos Aires*, *Argentina*

**Background** Fontan‐associated liver disease (FALD) is increasingly recognized as a significant long‐term complication of the Fontan circulation. However, data on pediatric populations remain limited.

**Aim:** To describe the characteristics of FALD across different age groups and to identify risk factors associated with advanced liver changes over a 20‐year follow‐up period.

**Methods** A retrospective study was conducted at a tertiary care center, including patients who underwent the Fontan procedure between 2005 and 2020. Heart transplant recipients were excluded. Fontan‐associated liver disease (FALD) was diagnosed based on the presence of hepatic fibrosis on biopsy, focal nodular hyperplasia on imaging, laboratory evidence of hepatic injury, impaired synthetic liver function, or hepatocellular neoplastic lesions. Advanced liver disease (ALD) was defined as bridging fibrosis and/or cirrhosis confirmed by liver biopsy, or by evident clinical signs of advanced fibrosis. Non‐invasive fibrosis scores, including the AST‐to‐platelet ratio index (APRI), Fibrosis‐4 (FIB‐4), and liver stiffness measurement (LSM) using FibroScan, were utilized to assess liver fibrosis.

**Results** Of 84 patients, 41 (48.8%) underwent liver assessment after 5 years of fontan procedure (male: n =26, female: n=15) with a median age of 13.7 years (r 7.4‐25.8) and median follow‐up of 10.7 years. FALD occured in 55% at 5‐10 years and 100% at 10‐20 years. Ultrasound (US) abnormalities were detected in 21 patients (51.2%), including surface nodularity or blunted liver edge in 7 (17.9%), heterogeneous liver parenchyma in 18 (43.9%), splenomegaly in 7 (17.9%), and ascites in 1 patient (2%). Laboratory abnormalities were present in 27 patients (72.9%). Mean platelet count was 217000 (SD 52100) at 5‐10 years and 179600 (SD 68717) at 10‐20 years (p 0.004). Median elastography score at 5‐10 years was 13 Kpa (IQR 9,9‐15,5) at 10‐15 ys 19,7 Kpa (IQR 16,3.‐ 22,6) and at 15‐20 years 17,1 Kpa.(IQR13,5‐22,6). Median APRI and FIB‐4 Scores ≥10 years of follow‐up were 0,37 (0,24‐0,42) and 0,42 (0,29‐0,45). Of the 13 pacientes who underwent liver biopsy, 6 had bridging fibrosis (one patient 8 years after fontan). After 10 years of fontan, 55,5% (10/18) had histologic or clinical ALD and correlated with lower prothrombin time (51 vs 73, p = 0,01), a VAST ≥ 2 (p =0,027), splenomegaly (125 mm vs 109 mm p =0.05) and the presence of more than 3 US findings (p 0.002). Liver fibrosis correlated poorly with APRI, FIB‐4. Additionally, liver stiffness measurements did not significantly change over time nor correlate with disease severity.

**Conclusions** In this pediatric cohort, advanced liver disease was observed in half of the patients after 10 years of follow‐up. Ultrasound and laboratory data proved to be valuable tools in determining the appropriate timing for liver biopsy. These findings suggest that initiating liver assessments within the first decade post fontan could enhance the management of hepatic complications and lead to better clinical outcomes.


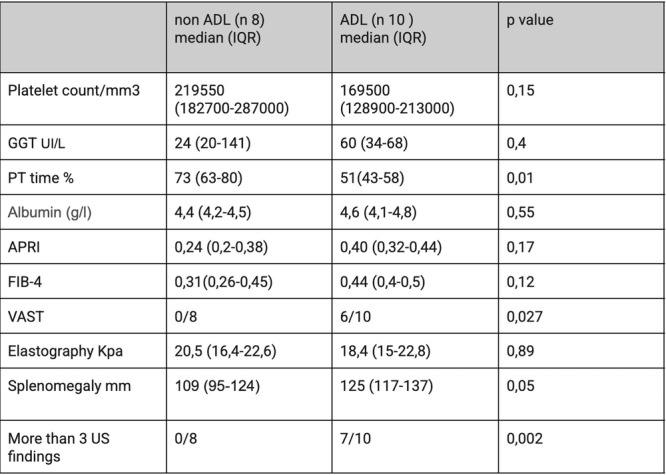



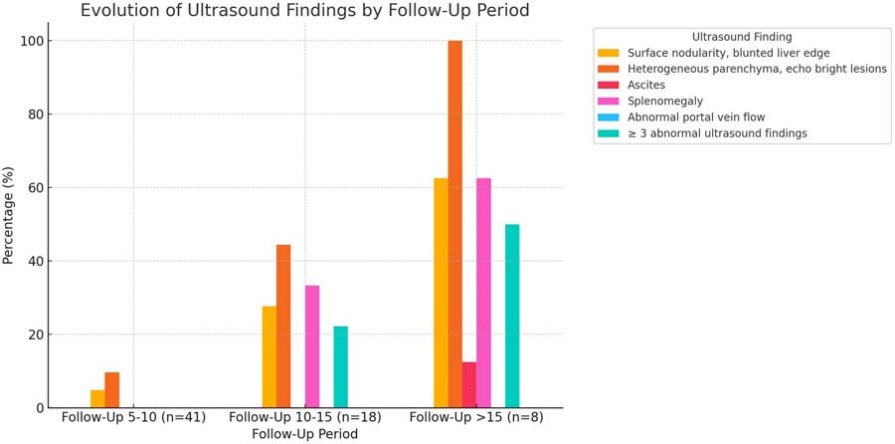


## 111 ODEVIXIBAT TREATMENT IN PATIENTS WITH FIC1 DEFICIENCY: SUSTAINED EFFICACY, PARTICULARLY IN PRURITUS, IN AN INTEGRATED ANALYSIS OF RESPONDERS (PEDFIC 1 AND PEDFIC 2)

*Henkjan Verkade* ^*1*^, *Richard Thompson* ^*2*^, *Reha Artan* ^*3,4*^, *Pier Luigi Calvo* ^*5*^, *Piotr Czubkowski* ^*6*^, *Buket Dalgic* ^*7*^, *Lorenzo D'Antiga* ^*8*^, *Özlem Durmaz* ^*9*^, *Tassos Grammatikopoulos* ^*10*^, *Girish Gupte* ^*11*^, *Winita Hardikar* ^*12*^, *Binita Kamath* ^*13*^, *Florence Lacaille* ^*14*^, *Kathleen Loomes* ^*15*^, *Cara Mack* ^*16*^, *Patrick McKiernan* ^*11*^, *Hasan Özen* ^*17*^, *Sanjay Rajwal* ^*18*^, *Eyal Shteyer* ^*19*^, *Etienne Sokal* ^*20*^, *Nisreen Soufi* ^*21*^, *James Squires* ^*22*^, *Ekkehard Sturm* ^*23*^, *Shikha Sundaram* ^*24*^, *Mary Elizabeth Tessier* ^*25*^, *Wendy van der Woerd* ^*26*^, *Jennifer Vittorio* ^*27*^, *Fatine Elaraki* ^*28*^, *Praneeta Nagraj* ^*29*^, *Alejandra Ramirez‐Santiago* ^*29*^, *Quanhong Ni* ^*29*^, *Emmanuel Gonzalès* ^*30*^

^*1*^ *Pediatric Gastroenterology‐Hepatology, Department of Pediatrics, University of Groningen, Beatrix Children's Hospital−University Medical Centre Groningen*, *Groningen*, *Netherlands*; ^*2*^ *Institute of Liver Studies, King's College London*, *London*, *United Kingdom*; ^*3*^ *Karolinska Institutet and Karolinska University Hospital*, *Stockholm*, *Sweden*; ^*4*^ *Department of Paediatric Gastroenterology, Akdeniz University*, *Antalya*, *Turkey*; ^*5*^ *Pediatric Gastroenterology Unit, Regina Margherita Children's Hospital, Azienda Ospedaliera‐Città della Salute e della Scienza di Torino*, *Turin*, *Italy*; ^*6*^ *Department of Gastroenterology, Hepatology, Nutritional Disorders and Pediatrics, The Children's Memorial Health Institute*, *Warsaw*, *Poland*; ^*7*^ *Department of Paediatric Gastroenterology, Gazi University Faculty of Medicine*, *Ankara*, *Turkey*; ^*8*^ *Department of Paediatric Hepatology, Gastroenterology, and Transplantation, Azienda Ospedaliera Papa Giovanni XXIII*, *Bergamo*, *Italy*; ^*9*^ *Istanbul University, Istanbul Faculty of Medicine*, *Istanbul*, *Turkey*; ^*10*^ *Paediatric Liver, GI and Nutrition Centre and MowatLabs, King's College Hospital NHS Trust*, *London*, *United Kingdom*; ^*11*^ *Liver Unit and Small Bowel Transplantation, Birmingham Women's and Children's NHS Foundation Trust*, *Birmingham*, *United Kingdom*; ^*12*^ *Department of Gastroenterology and Clinical Nutrition, Royal Children's Hospital Melbourne*, *Melbourne*, *Victoria*, *Australia*; ^*13*^ *Division of Gastroenterology, Hepatology and Nutrition, The Children's Hospital of Philadelphia and Department of Pediatrics, Perelman School of Medicine at the University of Pennsylvania*, *Philadelphia*, *PA*; ^*14*^ *Department of Paediatric Gastroenterology, Hepatology, and Transplant Medicine, University Children's Hospital Essen*, *Essen*, *Germany*; ^*15*^ *Department of Gastroenterology, Hepatology and Nutrition, Children's Hospital of Philadelphia*, *Philadelphia*, *PA*; ^*16*^ *Pediatric Gastroenterology, Hepatology, & Nutrition, Children's Wisconsin, Medical College of Wisconsin*, *Milwaukee*, *WI*; ^*17*^ *Paediatric Gastroenterology, Hepatology, and Nutrition, Hacettepe University Faculty of Medicine*, *Ankara*, *Turkey*; ^*18*^ *Children's Liver Unit, Leeds Teaching Hospitals NHS Trust, Leeds Children's Hospital*, *Leeds*, *United Kingdom*; ^*19*^ *Faculty of Medicine, Hebrew University of Jerusalem, Juliet Keidan Department of Paediatric Gastroenterology, Shaare Zedek Medical Centre*, *Jerusalem*, *Israel*; ^*20*^ *Université Catholique de Louvain, Cliniques St Luc, Service de gastroentérologie et hépatologie pédiatrique*, *Brussels*, *Belgium*; ^*21*^ *Pediatrics Department, Children's Hospital Los Angeles, Los Angeles*, *CA*; ^*22*^ *Division of Gastroenterology, Hepatology and Nutrition, The Children's Hospital of Pittsburgh*, *Pittsburgh*, *PA*; ^*23*^ *Paediatric Gastroenterology and Hepatology, University Children's Hospital Tübingen*, *Tübingen*, *Germany*; ^*24*^ *University of Colorado School of Medicine, Children's Hospital Colorado*, *Aurora*, *CO*; ^*25*^ *Department of Pediatrics, Section of Pediatric Gastroenterology, Hepatology, and Nutrition, Baylor College of Medicine−Texas Children's Hospital*, *Houston*, *TX*; ^*26*^ *Department of Pediatric Gastroenterology at the Wilhelmina Children's Hospital and University Medical Center Utrecht*, *Utrecht*, *Netherlands*; ^*27*^ *Department of Surgery, Center for Liver Disease and Transplantation, Columbia University Medical Center*, *New York*, *NY*; ^*28*^ *Ipsen*, *Boulogne‐Billancourt*, *France*; ^*29*^ *Ipsen*, *Cambridge*, *MA*; ^*30*^ *Hépatologie et Transplantation Hépatique Pédiatriques, Centre de Référence de l'Atrésie des Voies Biliaires et des Cholestases Génétiques, FSMR FILFOIE, ERN RARE LIVER, Hôpital Bicêtre, AP‐HP, Université Paris‐Saclay, Hépatinov, Inserm U 1193*, *Paris*, *France*

**Background:** Long‐term data demonstrate improvements in pruritus, reductions in serum bile acids (sBA), and manageable safety with odevixibat (ODX) across progressive familial intrahepatic cholestasis (PFIC) types in the phase 3 PEDFIC 1 study (NCT03566238) and PEDFIC 2 open‐label extension study (NCT03659916). It is important to better understand outcomes and response dynamics for patients with different PFIC types. Therefore, we have now characterized the responders to ODX with FIC1 deficiency (PFIC1), separately for sBA and pruritus.

**Methods:** Patients with FIC1 deficiency treated with ODX (40 or 120 µg/kg/day) in PEDFIC 1 or PEDFIC 2 who achieved an sBA response and/or a pruritus response at Week 24 of treatment were included in this *post hoc* analysis. sBA response was stringently defined as ≥70% reduction in fasting sBA from baseline (BL) or sBA ≤70 μmol/L. Pruritus response was defined as a ≥1‐point drop in observer‐reported (ObsRO) scratching score (0–4 scale) from BL. Here we report sBA and pruritus outcomes over time for these patients.

**Results:** 35 patients with FIC1 deficiency were included in this analysis. At Week 24 of ODX treatment, 5 patients were sBA responders and 14 were pruritus responders; 10 of the 14 pruritus responders had a pruritus response independent of an sBA response, and 15 patients had either sBA or pruritus response, or both. Sustained reductions in sBA and/or pruritus were observed from BL to 96 weeks (Table).

**Conclusions:** Patients with FIC1 deficiency who responded to ODX treatment at Week 24 experienced rapid improvements in pruritus and reductions in sBA that were sustained with long‐term treatment. Forty percent (n=14/35) of patients with FIC1 deficiency achieved a sustained, clinically relevant improvement in pruritus; 10/14 of these achieved a pruritus response without fulfilling the stringent criteria for an sBA response.


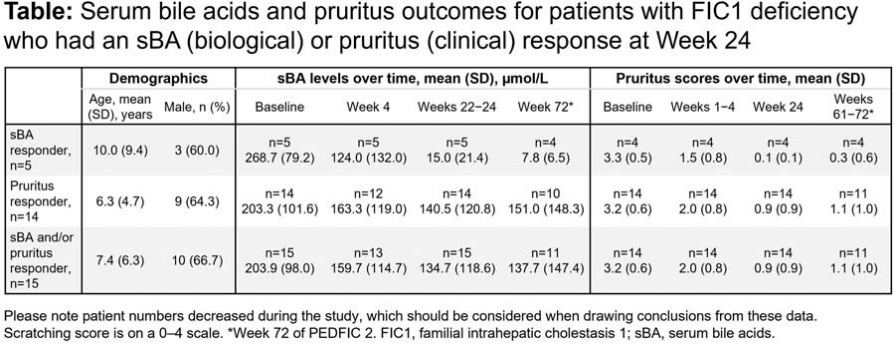


## 112 AUTOIMMUNE HEPATITIS AND RHEUMATOLOGIC DISEASE: AN UNDERRECOGNIZED CHALLENGE TO REMISSION?

*Kristen Solomon* ^*1*^, *Kelly Rouster Stevens* ^*2*^, *Nitika Gupta* ^*1*^

^*1*^ *Pediatric Gastroenterology and Hepatology*, *Emory University School of Medicine*, *Atlanta*, *GA*; ^*2*^ *Pediatric Rheumatology*, *Emory University School of Medicine*, *Atlanta*, *GA*

Autoimmune hepatitis (AIH) is a rare progressive inflammatory condition of the liver that can progress to end stage liver disease without proper treatment. The goal of the treatment of AIH is to induce biochemical remission by 6 months after initiation of treatment. Standard treatment with corticosteroids alone or in combination with azathioprine leads to remission rates of 75–80% in the majority of patients, however, disease outcomes can be worse in certain racial demographics. AIH can coexist with other autoimmune diseases, with up to 30–50% of AIH patients having concurrent autoimmune conditions. It is well described that Black, Hispanic, Asian and American Indians have higher rates of rheumatologic conditions. We sought to identify if concurrent diagnosis with a rheumatologic condition increased the likelihood of refractory AIH and subsequently a worse prognosis and to identify criteria which help identify patients at high risk of refractory disease.

**Methods:** A retrospective review of patients with AIH who attended hepatology clinic at Children's Healthcare of Atlanta between 2014 and 2021 was conducted. We identified 71 patients with Autoimmune hepatitis type 1 or type 2 based on biochemical lab testing or pathology using the International Autoimmune Hepatitis Group (IAHG) Scoring Criteria. Baseline demographics were collected, and clinical and biochemical factors were assessed to identify patients with co‐existing rheumatologic conditions and their outcomes were compared with patients with AIH who had negative screening for rheumatologic diseases.

**Results:** Seventy‐one patients were identified as having autoimmune hepatitis type 1 or type 2. Of those patients, 49 were female (69%) and 22 were male (31%). Forty‐eight percent identified as Black, 39 % White, 3 % Asian, 1 %American Indian or Alaskan Native, 3 % mixed race, and 6 % were unknown. Ten percent of patients identified as Hispanic or Latino. The median age at diagnosis with autoimmune hepatitis was 12 years [9 mo‐18yrs]. Out of the 71 patients with AIH, there was a clinical suspicion of a concurrent rheumatologic disease in 69 % (n=49) which resulted in additional laboratory evaluation. The level of evaluation varied amongst the patients, with a double stranded DNA antibody (dsDNA ab) being baseline inclusion criteria. Of the patients screened for co‐existing rheumatologic conditions, 9 were positive for an associated rheumatologic diagnosis. Of note, all 9 of these patients were female. Comparing the study group (patients with AIH and a co‐existing rheumatologic condition) and the control group (patients with AIH and negative rheumatologic screening), 89% of these patients (n=8/9) had refractory AIH compared to 37.5% (n=15/40) in the control group. Median follow up of patients with AIH plus a co‐existing condition versus the control group was 10 years and 8 years respectively [5 mo‐16yrs; 2‐18 yrs]. Two patients in the rheumatologic positive cohort received liver transplants, one was listed, with one deceased prior to transplantation (44%) versus in the control group 32.5% received liver transplants. The median age at time of AIH diagnosis in the rheumatologic positive cohort versus the control group was 10 years and 12 years respectively. There was a Black predominance in both groups with 66% in the rheumatologic positive group and 57% in the control group. The type of rheumatologic disease varied amongst the patients with six confirmed systemic lupus erythematosus (SLE) or lupus‐like syndrome, one with a mixed connective tissue disorder (MCTD), one with scleroderma and one with positive rheumatologic markers without a confirmed diagnosis. Also, 78% of the patients that screened positive for a rheumatologic condition had a clinical symptom concerning for a rheumatologic disease (rash, joint pain, Raynaud's), compared to the 20% in the control group.

**Conclusion:** In comparison between the two groups, majority of the patients in both the study population and the control group were Black. The majority of patients with a co‐existing rheumatologic condition had a younger median age at diagnosis and refractory AIH disease with increased incidence of liver transplant or death. We postulate that patients with a co‐existing rheumatologic disease or a lupus like profile have more refractory disease. Future multi‐center studies are needed to understand these patients’ clinical phenotype, establish comprehensive screening protocols and institute personalized therapies to improve patient outcomes.

## 113 LONG‐TERM LINEAR GROWTH OUTCOMES FOR PEDIATRIC LIVER TRANSPLANT RECIPIENTS WITH INBORN ERRORS OF METABOLISM

*Srinidhi Mereddy* ^*1*^, *Leila Mahdavi* ^*1*^, *Nada Yazigi* ^*1*^, *Gabriel Gondolesi* ^*1*^, *Kimberly Chapman* ^*2*^, *Udeme Ekong* ^*1*^, *Khalid Khan* ^*1*^

^*1*^ *Transplant*, *Georgetown University School of Medicine*, *Washington*,; ^*2*^ *Children's Hospital Los Angeles*, *Los Angeles*, *CA*

The second‐leading indication of liver transplantation (LT) in pediatric patients has recently evolved to be treatment of inborn errors of metabolism, such as urea cycle disorders, maple syrup urine disease, organic acidemias, etc.^1^ While liver transplantation is often able to stabilize the primary metabolic disorder in children, the long‐term implications of improved growth are not well characterized. This study investigates the linear growth in pediatric LT recipients over a 10‐year period at MedStar Georgetown University Hospital.

A retrospective chart review was conducted of pediatric patients who underwent LT as treatment for their metabolic disease at The Transplant Center for Children at MedStar Georgetown University Hospital between 2005 and 2020. Data extraction at key clinical timepoints post‐transplant (1 year, 5 years, 10 years) included the following parameters: body mass index (BMI) Z‐score, weight Z‐score, and height Z‐score, diagnosis, and steroid use. Inclusion criteria required: 1) a diagnosis of inborn error of metabolism including Intoxication diseases such as Urea Cycle Disorder, Maple Syrup Urine Disease, Organic Acidemia, or Non‐Intoxication diseases and 2) patients that only underwent one LT.

An unpaired sample t‐test was performed in Microsoft Excel to compare BMI Z‐score, Height Z‐score, and Weight Z‐score in patients with Intoxication diseases and Non‐Intoxication diseases. Significantly abnormal BMIs, Weights, and Heights compared to age were noted at the time of transplant (p=0.0181,p=0.0001,p=0.0021) and at 1 year post‐transplant for only Weight and Height (p=0.0427,p=0.01), but not at 5 years and 10 years. None of the patients were found to be taking steroid immunosuppression past 6 months post‐transplant, thus the relationship between steroid use and linear growth impact could not be assessed.

Patients with both Intoxication and Non‐intoxication diseases had abnormal Weight and Height up to 1 year post‐transplant and abnormal BMI at the time of transplant, but after these time points these metrics were normalized. These findings indicate that liver transplant improved growth for patients and normalized linear growth long‐term compared to solely medication management of metabolic disorders.^2,3^


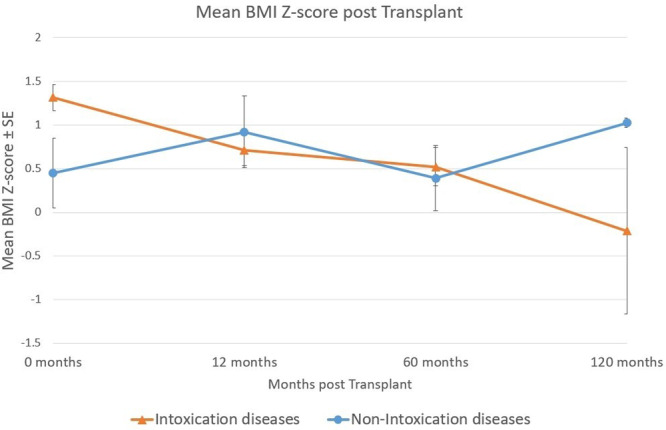


## 114 LONG TERM RISK FOR METABOLIC SYNDROME IN PEDIATRIC LIVER TRANSPLANT RECIPIENTS WITH METABOLIC DISORDERS: A RETROSPECTIVE CLINICAL CHART REVIEW

*Leila Mahdavi* ^*1,2*^, *Srinidhi Mereddy* ^*1,2*^, *Udeme Ekong* ^*2*^, *Khalid Khan* ^*2*^, *Kimberly Chapman* ^*3*^, *Carolina Rumbo* ^*4*^, *Stuart Kaufman* ^*2*^, *Thomas Fishbein* ^*2*^, *Gabriel Gondolesi* ^*2*^, *Nada Yazigi* ^*2*^

^*1*^ *Georgetown University School of Medicine*, *Washington*,; ^*2*^ *Pediatric Transplant Hepatology*, *MedStar Georgetown University Hospital*, *Washington*,; ^*3*^ *Pediatric Genetics*, *Children's National Hospital*, *Washington*,; ^*4*^ *Pediatric Gastroenterology*, *MedStar Georgetown University Hospital*, *Washington*

**Background:** The second‐leading indication for pediatric liver transplantation (LT) is the treatment of metabolic disorders. Most such recipients enter transplantation with a high BMI and elements of metabolic syndrome due to the strict low‐protein and high‐energy diet needed to medically stabilize their metabolic disorder. It is not clear if liver transplantation decreases their long‐term risk of metabolic syndrome. This study investigates the progression of metabolic syndrome risk factors in pediatric metabolic LT recipients over a 5‐year period.

**Methods:** A retrospective clinical chart review was conducted of pediatric patients who underwent isolated LT as treatment for their metabolic disease at The Transplant Center for Children at MedStar Georgetown University Hospital between 2005 and 2020 (N=85). Patients who received more than one transplant were excluded. Data was collected at time of transplant, 1‐ and 5‐years post‐transplantation. Data extraction included: body mass index (BMI), systolic blood pressure, lipid profile (total cholesterol and triglycerides), and hemoglobin A1c.Unpaired sample t‐test was used for data analysis; p‐values of less than 0.05 were considered significant.

**Results:** BMI z‐scores demonstrated a statistically significant improvement at 1 year for MSUD and UCD patients. By 5 years post‐transplantation, patients BMI z‐scores normalized regardless of their specific metabolic disease.

Systolic blood pressures and cholesterol remained statistically higher than normal for age in all patients at 1 and 5 years.

No significant anomalies were observed in triglyceride or hemoglobin A1c values.

**Conclusions:** Pediatric LT recipients with metabolic diseases normalize their BMI z‐score by 5 years post‐transplantation. Despite this improvement, they remain at increased risk for developing components of metabolic syndrome over time. Indeed, our data shows a statistically significant, persistent elevation in total cholesterol levels and blood pressure across all categories of metabolic patients. These findings underscore the need for proactive management of metabolic and cardiovascular risks for pediatric patients receiving liver transplantation for the treatment of their metabolic disease. Further studies are needed to delineate the pathophysiology and modifiable risk factors contributing to long term metabolic and cardiovascular morbidities in this patient population.


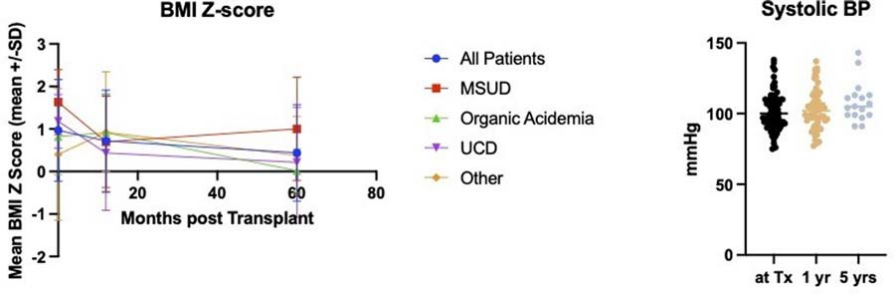


## 116 THE ITCH BURDEN: SURVEY INSIGHTS INTO PRURITUS IN BILIARY ATRESIA

*Natasha Dilwali* ^*2*^, *Rozanne Groen* ^*1*^, *Alex Brenner* ^*1*^, *Nadia Ovchinsky* ^*1*^, *Jennifer Vittorio* ^*1*^

^*1*^ *Pediatric Gastroenterology and Hepatology*, *New York University Grossman School of Medicine*, *New York*, *NY*; ^*2*^ *Johns Hopkins University*, *Baltimore*, *MD*

Biliary atresia (BA) is a progressive, fibro‐obliterative disorder affecting intra‐ and extrahepatic bile ducts. If left untreated, it leads to end‐stage liver disease and is ultimately fatal. It is the most common cause of cholestasis in infants under two months of age and is a leading indication for pediatric liver transplantation. While pruritus is a well‐characterized and burdensome symptom in other pediatric cholestatic conditions such as Alagille syndrome and progressive familial intrahepatic cholestasis (PFIC), it has also been reported in patients with BA. Despite its potential clinical significance, pruritus in BA remains poorly studied, and its impact on patient quality of life is not well defined.

A cross‐sectional survey was conducted to assess caregiver‐reported experiences and perspectives related to pruritus in pediatric patients with BA. Caregivers of children aged ≥6 months were recruited through the Biliary Atresia Research and Education (BARE) network and NYU Langone Health clinics. A structured survey instrument was developed to capture demographic data, relevant clinical history, current and prior medication use, and characteristics of pruritus. Responses were analyzed using descriptive statistics to summarize key findings.

A total of 48 caregiver surveys were included in the analysis; 16 were excluded due to prior liver transplantation or incomplete responses. The mean age of patients was 4.5 years (range: 0‐31 years), and all had undergone a Kasai portoenterostomy. Pruritus related to biliary atresia was reported by 46% (22/48) of caregivers. Commonly reported symptoms included irritability or fussiness (43.8%), difficulty falling or staying asleep (43.8%), excoriations from scratching (37.5%), skin discoloration or hyperpigmentation (25.0%), and blood on sheets (16.7%). Among 43 respondents, 23% noted pruritus was worse in the evening or at night. Most children (77%) had received at least one treatment for pruritus. The most frequently used medications were ursodeoxycholic acid (75%), antihistamines (27%), rifampin (25%), and topical agents (16%). In terms of perceived treatment effectiveness, 30% of caregivers reported complete relief, 35% partial relief, and 35% no improvement. Sleep disturbance due to pruritus was reported in 43% (18/42) of children. Among 35 respondents, 49% indicated that itching occasionally disrupted sleep, while 51% reported no impact. Fatigue, defined as frequent daytime napping, was reported in 26% (11/43) of children. Caregivers also reported personal burden: 44% (18/41) experienced sleep disruption themselves, and 10% (4/42) reported that their child's pruritus interfered with daily responsibilities.

Pruritus is a common and underrecognized symptom in children with BA, reported by nearly half of the surveyed caregivers. It significantly affects the quality of life for both patients and their families. These findings highlight the need for increased clinical awareness, routine standardized assessment of pruritus, and the development of targeted treatment strategies to improve outcomes in this population.


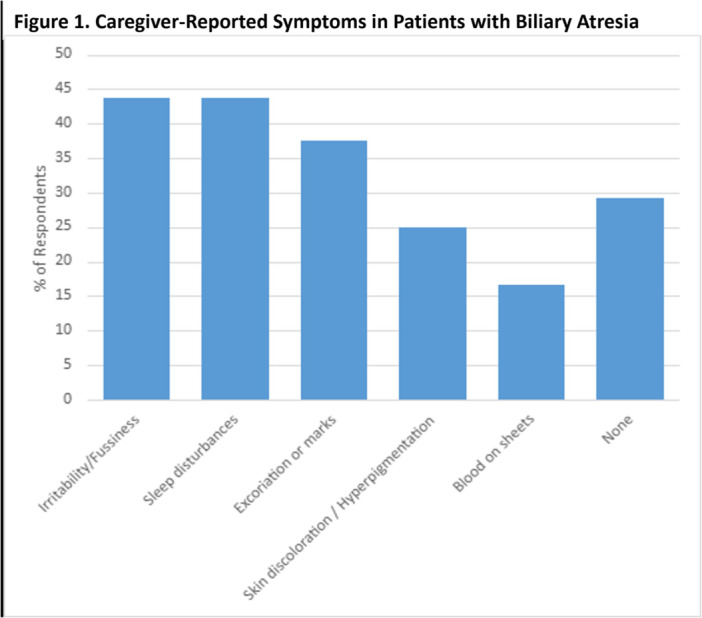



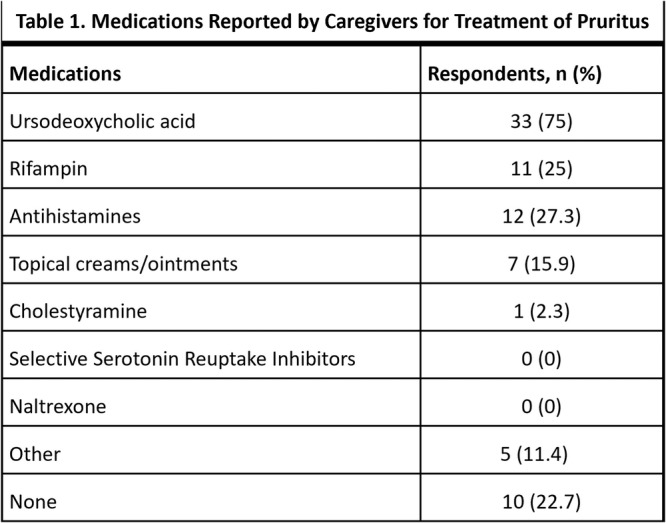


## 117 NEUTROPHIL PERCENTAGE‐TO‐ALBUMIN RATIO (NPAR) AS A PREDICTOR OF PORTAL HYPERTENSION PROGRESSION IN CHILDREN

*Mayra Paola Padilla‐Sánchez* ^*1*^, *Yunuen Rivera‐Suazo* ^*1,2*^, *Juan Suárez‐Cuenca* ^*1*^, *Carlos Ricardo Flores‐Soriano* ^*1*^, *Jaime Ernesto Alfaro‐Bolaños* ^*1,2*^

^*1*^ *Pediatria*, *Universidad Nacional Autonoma de Mexico*, *Mexico City*, *CDMX*, *Mexico*; ^*2*^ *Gastroenterología Pediátrica*, *Universidad Nacional Autonoma de Mexico*, *Mexico City*, *CDMX*, *Mexico*

**Background.** Portal hypertension (PH) in the pediatric population is a significant complication of chronic liver diseases, both cirrhotic and non‐cirrhotic. This condition can lead to life‐threatening complications, with variceal hemorrhage being one of the most serious and common.

The Neutrophil Percentage‐to‐Albumin Ratio (NPAR), a non‐invasive and easily obtainable biomarker of inflammation and nutritional status, has been proposed as a prognostic tool in this context. In adults, elevated NPAR is associated with a worse prognosis and higher mortality in liver cirrhosis and variceal bleeding. Its potential use in pediatrics remains underexplored.

**Objective.** To evaluate the relationship between NPAR and PH progression in pediatric patients.

**Methods.** *Study design*. A retrospective, observational, and analytical study was conducted in a tertiary center in Mexico City. *Population*. Pediatric patients (<18 years) with a newly diagnosed portal hypertension (PH), classified by the severity of gastroesophageal varices using the endoscopic GOV grading system. Cases with missing information and/or hypoalbuminemia of non‐hepatic origin were excluded. *Data collection*. Clinical, biochemical (neutrophil percentage and serum albumin levels), radiological, and endoscopic (GOV classification) data were collected from clinical electronic records. NPAR was calculated using the following formula: Neutrophil (%)/Serum albumin. Patients were categorized into two severity groups: 1) Low severity: grade I varices or absence of varices (GOV0–1); 2) High severity: grade II or greater esophagogastric varices (GOV2–3). *Statistical analyses*. High Categorical variables were resumed by n(%) and quantitative values with mean ± SD. Fisher test or mean comparison (T‐test or U‐Mann Whitney) were applied according to the nature of each variable.

**Results.** Study population consisted of 66 children with PH, 54.5% female, mean aged 76 days, showing mean values of BMI 16.6 and abnormal values in liver function test and blood cytometry (Table 1). Main etiology of PH was prehepatic with 84.8%. Based on GOV endoscopic criteria, 34 patients were classified as having low severity PH (GOV 0‐1), and 32 as high severity PH (GOV 2‐3); the group with higher PH was characterized by significantly older age, higher counts of leucocytes, % neutrophils and lower number of platelets.

**Conclusion.** Higher values of NPAR seem to be related with higher PH in pediatric population. This may be due to its ability to mainly reflect systemic inflammation as well as nutritional status.


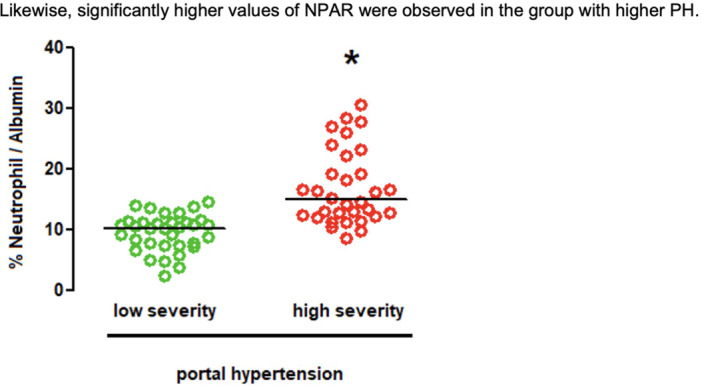



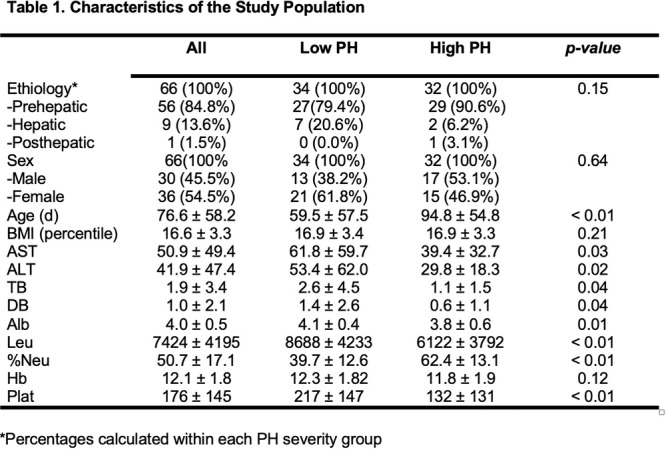


## 126 TRANSFORMING GENETIC DIAGNOSIS OF PEDIATRIC CHOLESTASIS USING A COMPREHENSIVE PHENOTYPE‐DRIVEN LIVER GENE PANEL

*Akihiro Asai* ^*1,2*^, *Wenying Zhang* ^*1*^, *Chunyue Yin* ^*1,2*^, *Emily Vincent* ^*1*^, *William Balistreri* ^*1,2*^

^*1*^ *Pediatrics*, *Cincinnati Children's Hospital Medical Center*, *Cincinnati*, *OH*; ^*2*^ *Pediatrics*, *University of Cincinnati College of Medicine*, *Cincinnati*, *OH*

**Background:** Chronic cholestatic jaundice affects approximately 1 in 2,500 full‐term infants. Inherited conditions account for 25–50% of cases. A precise genetic diagnosis can guide clinical management, inform family counseling, and improve outcomes. However, ~30% of children with isolated or primary cholestasis remain undiagnosed after standard gene panels or exome/genome sequencing, due in part to variants of uncertain significance in genes not clearly linked to liver disease.

**Methods:** To address this diagnostic gap, we developed the *Cincinnati Undiagnosed and Rare Liver (CURL) Disease Panel*, a phenotype‐driven genetic test based on exome sequencing. The panel targets 1,445 genes curated for their relevance to liver biology and cholestasis. The gene list is continuously refined by hepatologists, geneticists, and basic researchers to reflect the latest literature and emerging discoveries. We applied this test to patients with chronic cholestasis of unclear etiology, prioritizing those for whom a genetic diagnosis could inform care. Test interpretation was performed via multidisciplinary case review.

**Results:** We piloted the CURL panel in eight patients (seven pediatric, one adult), six with trio‐based testing. All had undergone prior clinical genetic testing, including the 72‐liver gene panel at CCHMC and, in two cases, commercial whole genome sequencing. In three cases, the CURL panel confirmed previously reported variants consistent with clinical features: **Patient 1:** Neonatal cholestasis due to compound missense heterozygous variants in *ABCB11*. **Patient 2:** Syndromic bile duct paucity with a heterozygous *NOTCH2* missense variant. **Patient 3:** Intrahepatic cholestasis of pregnancy with a heterozygous *ABCB4* in‐frame deletion. In three additional pediatric patients, novel variants were identified in genes consistent with the clinical phenotype: **Patient 4:** High‐GGT neonatal cholestasis with compound heterozygous *LARS1* missense variants. **Patient 5:** Cholestasis and pancreatic insufficiency with a hemizygous missense variant in *ATP6AP1*. **Patient 6:** Severe lobular cholestasis with a heterozygous *PLEC* missense variant.

**Conclusion:** The CURL panel provides a focused, phenotype‐guided approach to genetic testing for pediatric cholestasis. It shows promise to enhance diagnostic yield compared to standard gene panels and untargeted exome/genome sequencing, helping clinicians reach actionable diagnoses in previously unexplained cases. This approach may be especially valuable for general pediatric providers managing children with unexplained liver dysfunction.

## 127 WHAT DO WE KNOW ABOUT CHOLEDOCHAL CYSTS?

**EPIDEMIOLOGY, CLINICAL, HISTOLOGICAL CHARACTERISTICS, AND SURGICAL TREATMENT OF PEDIATRIC PATIENTS DIAGNOSED WITH CHOLEDOCHAL CYSTS OVER THE LAST 10 YEARS IN A TERTIARY CARE HOSPITAL**

*JIMENA AMEZCUA MARTINEZ*, *Andrea Isabel Fernández*, *Flora Zarate‐Mondragon*, *Dr. José Francisco Cadena León*, *Ericka Montijo‐Barrios*, *Roberto Cervantes Bustamante*, *Jaime Ramirez‐Mayans*, *Erick Toro Monjaraz*, *Karen R. Ignorosa‐Arellano*

*Pediatric Gastroenterology and Nutrition*, *Instituto Nacional de Pediatria*, *Mexico City*, *CDMX*, *Mexico*

**Introduction:** Choledochal cysts are a rare congenital biliary tract condition characterized by intra‐ or extrahepatic bile duct dilations. Up to 80% of cases are diagnosed during childhood. Clinical manifestations are nonspecific, needing a comprehensive approach to ensure timely diagnosis. In Mexico, there are currently no recent reports regarding this pathology; therefore, a comprehensive description of the disease will facilitate thorough evaluation and early diagnosis of its potential complications, including liver cirrhosis, pancreatitis, cholangitis, and biliary tract neoplasms.

**Objective:** To describe the epidemiology, clinical manifestations, histological characteristics, and surgical treatment of patients diagnosed with choledochal cysts in a tertiary care center in Mexico City over the last 10 years.

**Materials and Methods:** A descriptive, retrospective, cross‐sectional, and observational study was conducted. Medical records of patients diagnosed with choledochal cysts from January 1, 2014, to January 1, 2024, were included. The study analyzed epidemiological data, clinical presentation, diagnosis, histological features, and surgical treatment. Results were assessed using frequency measures, percentages, means, and standard deviations.

**Results:** A total of 47 patients were identified, 38 (80%) were female, with a mean age at diagnosis of 67 ± 7.8 months. According to Todani's classification, the most common type was Type I in 32 cases (68%), followed by, IV in 14 cases (29.7%) type V in 1 (2.1 %).

Clinical presentation included abdominal pain in 40 patients (85%), jaundice in 31 (65.9%), and abdominal mass in 11 (23.4 %). Complications included pancreatitis in 14 patients (29 %) and cholangitis in 7 (14%). One patient presented with upper gastrointestinal bleeding due to portal hypertension. Cholestasis was found in 25 patients (53%).

Initial diagnostic imaging included liver and bile duct ultrasonography in 12 patients (25 %), MR cholangiopancreatography in 9 (19.1%), and abdominal CT in 1 (2.1%).

Surgical treatment involved Roux‐en‐Y hepaticojejunostomy in 47 patients (98%), one patient required sphincteroplasty via ERCP. Biopsies were obtained in 29 patients (61%), revealing hepatic cirrhosis in 3 cases (6.3%) and congenital hepatic fibrosis in 2 cases (4.2%).

**Conclusions:** This study highlights that the epidemiological profile in Mexico aligns with international reports. Early diagnosis is crucial, particularly for patients presenting with chronic abdominal pain, jaundice, and abdominal masses, these are the most frequently reported clinical features. Identifying patients at risk of progression to liver cirrhosis is essential, as histological findings in this study included cirrhosis and congenital hepatic fibrosis. Biopsy during surgery is a cornerstone for prognosis and subsequent medical management.

Regarding surgical treatment, Roux‐en‐Y hepaticojejunostomy remains the procedure of choice, consistent with existing literature.


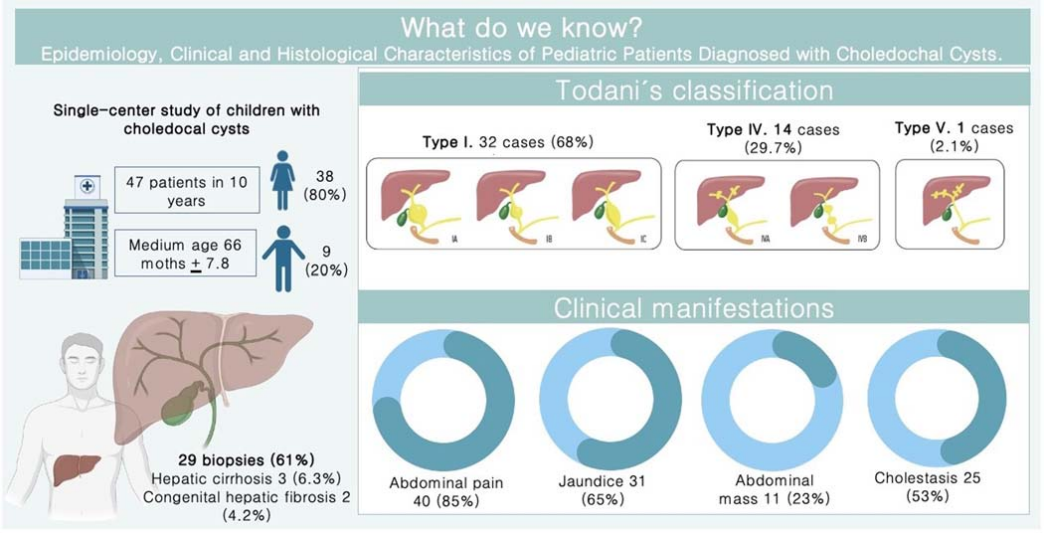


## 128 SERUM GDF‐15 AS A NOVEL BIOMARKER IN PEDIATRIC AUTOIMMUNE HEPATITIS: A PROSPECTIVE MULTICENTER STUDY IN JAPAN

*Naoya Tsumura* ^*1,2*^, *Ken Kato* ^*1,2*^, *Ryuta Nishikomori* ^*1,2*^, *Tatsuki Mizuochi* ^*1,2*^, *JPAILD Study Investigators* ^*2*^

^*1*^ *Department of Pediatrics and Child Health*, *Kurume University School of Medicine*, *Kurume*, *Fukuoka*, *Japan*; ^*2*^ *Japanese Pediatric Autoimmune Liver Disease (JPAILD) Study Group*, *Kurume*, *Japan*

**Background and Aim:** Autoimmune hepatitis (AIH) is a rare condition in children, and both its diagnosis and assessment of disease activity remain challenging. Although noninvasive biomarkers are highly desirable, no reliable markers have been established to date. This study aimed to evaluate the potential role of serum growth differentiation factor 15 (GDF‐15) as a novel, disease‐specific biomarker in pediatric AIH, with the potential to provide superior diagnostic utility compared to conventional markers such as alanine aminotransferase (ALT) and gamma‐glutamyl transpeptidase (GGT).

**Methods:** This prospective, multicenter study analyzed clinical data and serum samples collected from 18 participating institutions in Japan. The discovery cohort included pediatric AIH patients (n = 8) who achieved normalization of ALT (<30 U/L) and GGT (<40 U/L) after treatment, and age‐matched healthy controls (HC, n=7). Four serum markers previously reported as indicators of chronic liver disease, including GDF‐15, matrix metalloproteinase 7, calprotectin, and leucine‐rich α2‐glycoprotein, were measured using enzyme‐linked immunosorbent assay. Markers that showed significant differences between AIH and HC in the discovery cohort were further evaluated in the validation cohort, which included pediatric AIH patients (n=18) with normalized ALT and GGT after treatment, disease controls (DC, n=10) such as hepatitis B carriers and patients with metabolic dysfunction‐associated steatotic liver disease, and HC (n=14).

**Results:** In the discovery cohort, only GDF‐15 was significantly increased in AIH compared to HC (P<0.001). In the validation cohort, the median GDF‐15 levels were 460 pg/mL, 254 pg/mL (P<0.01), and 186 pg/mL (P<0.0001) for AIH, DC, and HC, respectively, with a significant increase in AIH. ROC analysis for differentiating AIH from DC yielded an AUC of 0.92. At a cut‐off value of 278 pg/mL, determined from the ROC curve, the sensitivity and specificity for diagnosing AIH were 80% and 94%, respectively.

**Conclusion:** Serum GDF‐15 may serve as a disease‐specific biomarker in pediatric AIH and could provide greater diagnostic utility than ALT or GGT.

## 129 GENETIC INSIGHTS INTO PFIC‐ASSOCIATED GENES IN UNEXPLAINED CHRONIC CHOLESTASIS AND LIVER DISEASE: FREQUENCY AND IMPLICATIONS OF VARIANT COMBINATIONS

*Brett Hoskins* ^*1*^, *Tiziano Pramparo* ^*2*^, *Ethan Gough* ^*3*^, *Jolan Terner‐Rosenthal* ^*2*^, *Rana Dutta* ^*2*^, *Wikrom Karnsakul* ^*4*^

^*1*^ *Division of Pediatric Gastroenterology, Hepatology and Nutrition, Department of Pediatrics*, *Riley Hospital for Children, Indiana University School of Medicine*, *Indianapolis*, *IN*; ^*2*^ *Mirum Pharmaceuticals Inc*, *Foster City*, *CA*; ^*3*^ *Department of Biostatistics, Epidemiology, and Data Management (BEAD) Core*, *The Johns Hopkins University School of Medicine*, *Baltimore*, *MD*; ^*4*^ *Division of Pediatric Gastroenterology, Hepatology, and Nutrition, Department of Pediatrics*, *The Johns Hopkins University School of Medicine*, *Baltimore*, *MD*

**Background:** Heterozygous pathogenic variants in PFIC‐associated genes (*ATP8B1*, *ABCB11*, *ABCB4*, *TJP2*, *NR1H4*, and *MYO5B*) may contribute to unexplained cholestasis and chronic liver disease. This study investigates the frequency and potential implications of compound heterozygous states involving pathogenic variants and variants of uncertain significance (VUS) in these genes.

**Methods:** Data from a 77‐gene cholestasis panel (updated in 2017 and 2022) were analyzed for cases tested between February 2016 and February 2022. Eligible cases had unexplained cholestasis or chronic liver disease. DNA sequencing was performed using Sure Select (2016–2021) and PGxome® (2021–2024). Analysis focused on pathogenic monoallelic variants with VUS in the same gene or across the six

PFIC‐associated genes. Clinical data were unavailable for review (ongoing consortium to collect real‐time data.

**Results:** Among 10,897 cases, 51.1% were under one year old, and 9.2% were ≥18 years. Monoallelic pathogenic variants were identified in *ATP8B1* (33 cases), *ABCB11* (100), *ABCB4* (115), *TJP2* (17), *NR1H4* (7), and *MYO5B* (6). Potential compound heterozygous states with VUS were identified in *ATP8B1* (8 cases), *ABCB11* (41), *ABCB4* (41), *TJP2* (4), and *MYO5B* (3). No cases exhibited concurrent monoallelic pathogenic variants across multiple genes, though VUS from other genes were observed. Of 33 *ATP8B1* monoallelic pathogenic variants, only 1 had an *ACBC4* VUS. Of 100 *ABCB11* monoallelic pathogenic variants, 1 case had an *ATP8B1* VUS, 1 had an *ABCB4* VUS, 3 had a *TJP2* VUS, 1 had an *NR1H4* VUS, and 2 had a *MYO5B* VUS. Of 115 *ABCB4* monoallelic pathogenic variants, 4 cases had a *ATP8B1* VUS, 2 had an *ABCB11* VUS, 1 had a *TJP2* VUS, 2 had an *NR1H4* VUS, and 2 had a *MYO5B* VUS. Of 17 *TJP2* monoallelic pathogenic variants, 1 case had an *ATP8B1* VUS and 3 had an *ABCB4* VUS. Of 7 *NR1H4* monoallelic pathogenic variants, 2 cases had 2 *ABCB11* VUS and 1 case had 1 *TJP2* VUS.

**Conclusions:** The frequent occurrence of monoallelic pathogenic variants with VUS in the same gene highlights the need for urgent reclassification of VUS, especially when clinically significant. These findings may aid in diagnosing PFIC‐related conditions and refining the interpretation of genetic results. Further studies are needed to engage in data‐sharing initiatives with other genetic testing centers to pool VUS data, collaboratively reclassifying VUS based on combined genetic, clinical, and functional data to improve diagnostic accuracy and explore the potential modifying effects of variants in other PFIC‐associated genes (e.g., *ABCB4* VUS inpatients with *ABCB11* monoallelic pathogenic variants).

## 130 REAL WORLD TREATMENT OF PEDIATRIC HEPATITIS C: THE FLORIDA EXPERIENCE

*Mohammad Adawi* ^*1*^, *Katherine McGoogan* ^*1*^, *Regino Gonzalez‐Peralta* ^*2*^

^*1*^ *Pediatric Gastroenterology*, *The Nemours Foundation*, *Jacksonville*, *FL*; ^*2*^ *AdventHealth Orlando*, *Orlando*, *FL*

**Objective:** Chronic Hepatitis C virus (HCV) infection is a global health problem. Before the approval of direct acting antivirals (DAA) for children with chronic HCV infection, treatment was challenging. In pediatric clinical trials, the use of DAA was effective and safe for children with chronic HCV infection, However, little is known about the effectiveness of DAA used in standard clinical practice. The purpose of this real‐world study was to describe our experience in treating children with chronic HCV infection with DAA prescribed outside of clinical trials.

**Methods:** We conducted a retrospective review of EMRs to identify children aged 3 – 17 years of age who were treated for chronic HCV infection at Nemours Children's Health in Florida between 4/17/2017 and 5/23/2025. Data analyzed included patient demographics, laboratory results, HCV RNA results, therapeutic interventions and sustained virologic response (defined as undetectable HCV RNA PCR 12 weeks after completion of treatment).

**Results:** A total of 32 patients were identified, all of whom acquired HCV vertically. The median age range for the cohort was 6 (3–17) years. The group was predominantly comprised of non‐Hispanic whites (88 %, n=28); 56% (n=18) were male and 44% (n=14) were female. DAA therapy was initiated in 32 patients, of whom 30 completed the medication course. 24 patients (75%) who completed treatment had an HCV PCR collected at least 12 weeks after finishing treatment. 100% of these 24 patients achieved SVR. There were no clinically significant side effects reported in the patients treated. Insurance challenges were a barrier to initiation of treatment. Nonadherence and palatability were barriers to treatment completion.

**Conclusions:** DAA are effective and safe in treating children with HCV infection in the real‐world setting. Addressing systemic barriers and promoting education for caregivers and providers are essential to optimizing outcomes.


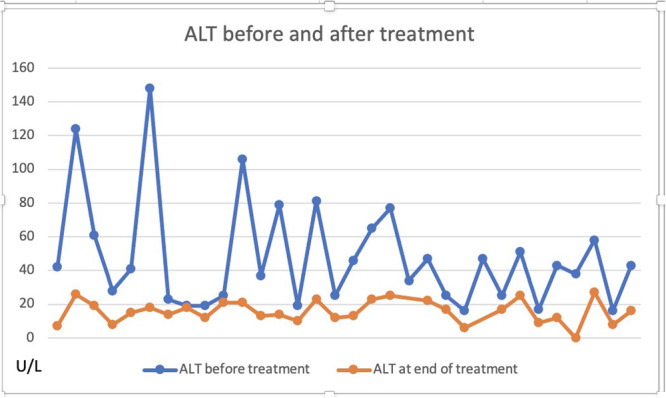


ALT before and after treatment


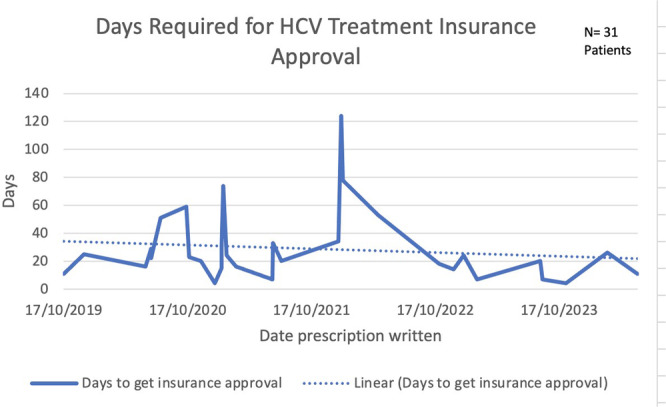


Days Required for HCV Treatment Insurance Approval

## 131 ANALYSIS OF SURVIVAL OUTCOMES BY TRANSPLANT TYPE IN PATIENTS WITH PROPIONIC ACIDEMIA AND METHYLMALONIC ACIDEMIA

*Maria Amendola*, *James Squires*, *Simon Horslen*, *Vikram Raghu*

*Pediatrics*, *University of Pittsburgh School of Medicine*, *Pittsburgh*, *PA*

**Background:** Methylmalonic acidemia (MMA) and propionic acidemia (PA) are two of the most common organic acidemias and can lead to chronic complications like developmental delay, cardiomyopathy, and renal failure. Liver transplant offers a therapeutic option via “partial enzyme replacement” that decreases recurrence of metabolic crises with the ability to liberalize protein restriction in the post‐transplant period. It is unknown whether partial liver grafts, which may contain less enzyme, provide sufficient enzyme replacement comparable to whole grafts. In this study, we aim to describe the types of technical variants used and analyze survival curves in these patient populations.

**Methods:** Liver transplant recipients with a diagnosis of propionic acidemia or methylmalonic acidemia were identified from the Scientific Registry of Transplant Recipients between 2013 and 2023. The primary exposure was transplant type, classified as decreased whole, deceased partial, or living donor. Analysis included descriptive statistics and survival analysis to examine differences in graft and patient survival by transplant type.

**Results:** We identified 71 liver recipients with PA and 88 liver recipients with MMA. Most recipients were in the pediatric age range (66/71 with PA; 82/88 with MMA). One PA recipient received a simultaneous kidney transplant, and three received simultaneous heart tranplants. Kidney transplant was performed simultaneously in 47/88 MMA receipients.

Among the PA recipients receiving isolated liver transplants, 37 received whole livers from deceased donors, 27 received partial livers from deceased donors, and 3 had living donors. 4/5 graft failure events including all 3 mortality events in PA recipients occurred in those with partial grafts. All events occurred within 30 days of follow‐up.

Among MMA recipients, 40/47 combined liver‐kidney recipients received whole livers. Among those receiving isolated liver transplants 6/41 had living donors and 10/41 had partial grafts from deceased donors. Among all MMA recipients, 5/7 graft failure events including 4/5 mortality events occurred within 60 days of transplant. Four of these events, including three within 60 days, occurred in those who received whole livers.

**Conclusions:** Children with PA and MMA receiving liver transplants are susceptible to early graft loss and mortality events. The majority of these events in PA recipients seem to occur in those with partial grafts. Whether partial grafts provide sufficient enzyme in PA recipients requires further inquiry.


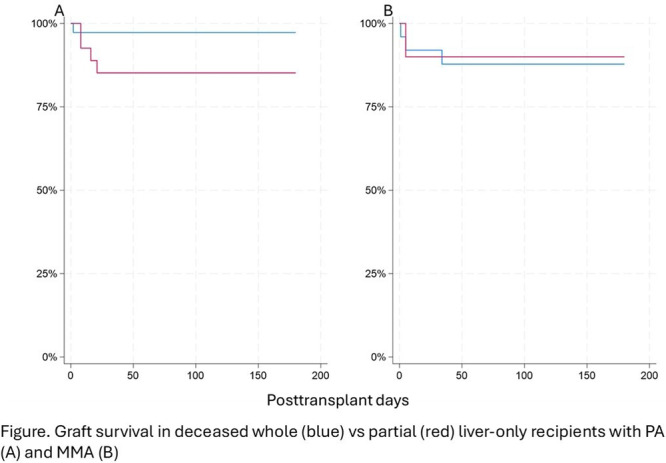


## 135* AVOIDANT/RESTRICTIVE FOOD INTAKE DISORDER (ARFID) IN YOUTH WITH INFLAMMATORY BOWEL DISEASE: PREVALENCE AND RELATIONSHIP WITH QUALITY OF LIFE

*Ally Koh* ^*1,2*^, *Ashley Dunn* ^*1,2*^, *Megha Tandel* ^*3*^, *Victor Ritter* ^*3*^, *Farah Mardini* ^*2*^, *Meeta Patel* ^*5*^, *Nandini Datta* ^*4*^, *Anava Wren* ^*1,2*^, *Alka Goyal* ^*1,2*^

^*1*^ *Pediatrics, Division of Pediatric Gastroenterology, Hepatology and Nutrition*, *Stanford University School of Medicine*, *Stanford*, *CA*; ^*2*^ *Center for Pediatric IBD and Celiac Disease*, *Stanford Medicine*, *Stanford*, *CA*; ^*3*^ *Medicine, Quantitative Sciences Unit*, *Stanford University School of Medicine*, *Stanford*, *CA*; ^*4*^ *Child and Adolescent Psychiatry*, *Stanford University School of Medicine*, *Stanford*, *CA*; ^*5*^ *Packard Children's Health Alliance*, *Stanford Medicine*, *Stanford*, *CA*

Patients with Inflammatory Bowel Disease (IBD) often perceive that diet influences their symptoms. Avoidant/Restrictive Food Intake Disorder (ARFID), characterized by restrictive eating behaviors due to sensory sensitivities, fear of aversive consequences, or lack of interest in food, is increasingly recognized in populations with chronic illnesses. Although ARFID has been identified in 10–17% of adults with IBD and 2–3% of the general pediatric population, its prevalence and clinical significance in pediatric populations with IBD remain poorly characterized. This study aimed to: 1) estimate the prevalence of ARFID symptoms in youth with IBD and 2) examine associations between ARFID symptoms and demographic, clinical (i.e., abdominal pain, diarrhea, dietary therapy), and psychosocial characteristics (i.e., anxiety, depression, and global health).

A cross‐sectional study was conducted with patients aged 8–25 years with IBD at Stanford Medicine Children's Health. ARFID symptoms were assessed using validated, age‐appropriate measures: the Eating Disorders in Youth Questionnaire (EDY‐Q) for children (ages 8–13), and the Eating Disorder Examination Questionnaire (EDE‐Q) combined with the Nine‐Item ARFID Screen (NIAS) for adolescents and young adults (ages 14–25). A positive ARFID screen was defined as meeting DSM‐5 criteria for ARFID, based on EDY‐Q criteria (children) or an EDE‐Q global score <2.3 with ≥1 elevated NIAS subscale (adolescents/young adults). Patient‐reported outcomes were measured using PROMIS Anxiety, Depressive Symptoms, and Global Health instruments. IBD clinical data were obtained from electronic medical records. Prevalence estimates were calculated using proportions and 95% confidence intervals (CIs). Associations between ARFID symptoms and participant clinical and psychosocial characteristics were evaluated using unadjusted and adjusted regression models, with adjusted models selected via uniLasso.

Among 100 participants (50% female; 63% White/European; 61% with Crohn's disease), 28% (95% CI: 19–38%) met criteria for ARFID symptoms. Prevalence was 32% (95% CI: 17–51%) in children and 26% (95% CI: 16–39%) in adolescents/young adults with IBD. Among children with ARFID symptoms, selective eating (71%) and weight/shape concerns (61%) were the most common subscales reported. Among adolescents/young adults with ARFID symptoms, picky eating (17%), low appetite (11%), and fear of adverse consequences (11%) were predominant.

Presence of ARFID symptoms was significantly associated with lower PROMIS Global Health T‐scores (estimated adjusted difference of ‐4.6, 95% CI: ‐8.4– ‐0.75, p<0.05), indicating poorer physical and psychological health among those with ARFID symptoms. Participants with ARFID symptoms reported higher PROMIS Anxiety scores (adjusted difference of 2.0; 95% CI: ‐2.5–6.4), PROMIS Depressive Symptoms scores (adjusted difference of 1.3; 95% CI: ‐3.1–5.8), and abdominal pain scores (OR = 3.03, 95% CI: 0.92‐10.0); however, these differences were not statistically significant.

Study results highlighted that ARFID symptoms were present in over a quarter of youth with IBD, with higher rates observed among children. This study provides preliminary evidence that the prevalence of ARFID symptoms in youth with IBD is notably greater than in the general population of children (2–3%). ARFID symptoms were associated with poorer global health, suggesting a negative impact on youth's holistic health and wellbeing. While anxiety and depressive symptoms scores were not statistically significant, their confidence intervals reflect a possible trend that requires further exploration with larger and more diverse samples of children and young adults with IBD. Further investigation, including chart review and qualitative interviews, is underway to better understand the drivers of ARFID behaviors and their impact on wellbeing and quality of life in pediatric IBD populations.

## 137* SUSTAINED REMISSION AND IMPROVED TROUGH LEVELS FOLLOWING TRANSITION FROM INTRAVENOUS TO SUBCUTANEOUS INFLIXIMAB IN PEDIATRIC INFLAMMATORY BOWEL DISEASE

*Patricia Hughes*, *Nanci Pittman*, *Keith Benkov*, *Virginia Baez*, *Juliana Kennedy*, *Shouli Tung*, *Marla Dubinsky*, *Elizabeth Spencer*

*Pediatric Gastroenterology*, *Mount Sinai Health System*, *New York*, *NY*

**Background:** Subcutaneous (SC) infliximab (IFX) maintenance therapy is now FDA‐approved for adults in the U.S. and has been used off‐label in pediatric inflammatory bowel disease (IBD) internationally since 2020. Despite emerging global interest, limited data exists on pharmacokinetics, clinical outcomes, and dosing strategies in pediatric patients. Further, prior adult studies suggest that pre‐switch intravenous (IV) IFX dosing may help predict the required SC dose. Escalated IV dosing regimens—common in pediatric patients—may not indicate the same need for higher SC doses, particularly in children with lower body weight.

**Aim:** To evaluate the impact of transitioning from IV to SC IFX on sustained clinical remission, to describe associated changes in IFX levels, and explore impact on objective markers of remission and safety in pediatric IBD.

**Methods:** In this prospective single‐center cohort study, pediatric patients with IBD (<18 years) who were clinically stable on maintenance IV IFX and transitioned to SC IFX were enrolled. Prospectively collected data included demographics, IBD phenotype, disease activity (assessed by Pediatric Crohn's Disease Activity Index (PCDAI) or Pediatric Ulcerative Colitis Activity Index (PUCAI), intestinal ultrasound (IUS), and endoscopy), and pharmacokinetics (IV trough level prior to switch [T0] and SC IFX level ≥12 weeks post‐switch [T1]). The primary outcome was sustained clinical remission at T1, defined as unchanged PCDAI≤10 or PUCAI<10, without need for hospitalization, surgery, or corticosteroids. Secondary outcomes included changes in pharmacokinetic markers, new inflammation on IUS (bowel wall thickness ≥3.0 mm and/or hyperemia), adverse effects (e.g., paradoxical dermatitis, infusion reactions), and qualitative assessment of tolerability and access barriers. Descriptive statistics were reported as medians with interquartile ranges [IQRs]. A paired t‐test was used to compare IFX levels between T0 IV trough and T1 SC steady state.

**Results:** Of 33 patients for whom SC IFX was ordered, 20 initiated therapy; insurance denial due to age was the primary reason for non‐initiation (9/13). Among the 20 initiators (CD n=15, UC n=5; median age 15 [14–16] years; 45% female), 16 (80%) received 120 mg every other week and 4 (20%) received 240 mg every other week, corresponding to a median weight‐based dose of 2.3 [2.0–3.2] mg/kg. Prior to transition, the median IV dose was 10 [10–11] mg/kg every 6 [5–7] weeks.

At the time of analysis, a total of 15 patients had reached T1. All patients achieved the primary outcome, sustaining clinical remission with no hospitalizations, surgeries, or steroid use. T0 and T1 IUS were both available in 8/15 (53%) patients, all of whom had stable findings, 88% with no IUS inflammation. Among the 11 with post‐switch IFX levels, median IV trough at T0 was 19.0 [12.8–22.9] μg/mL, rising to 29.5 [26.5–34.5] μg/mL on SC dosing (P<0.001). Antidrug antibodies present at T0 in two patients cleared; two patients developed new low‐titer antibodies at T1.

Median follow‐up on SC IFX was 130 [58–248] days. SC IFX was generally well tolerated: three patients reported injection site pain, and one (age 7) reverted back to IV after four SC doses due to discomfort. One patient with prior infusion reactions tolerated SC IFX without issue. Of the five patients with pre‐existing paradoxical psoriasiform dermatitis, two improved, one worsened (leading to IFX discontinuation), and two remained stable.

**Conclusions:** Transitioning from IV to SC IFX in pediatric IBD maintained remission and resulted in significantly higher levels without new safety concerns. Insurance barriers remain a major limitation. These findings support SC IFX as a viable long‐term maintenance option in pediatric IBD, with further research underway to define early predictors of successful transition and optimal dosing strategies as well as impact on adverse effects of the IV form.

## 138* INCIDENCE OF VENOUS THROMBOEMBOLISM AND PROPHYLAXIS IN PEDIATRIC PATIENTS WITH INFLAMMATORY BOWEL DISEASE: A SINGLE‐CENTER EXPERIENCE

*Meredith Kline* ^*1,4*^, *Tessa George* ^*2,4*^, *David Rubin* ^*3,4*^, *Jill de Jong* ^*5*^, *Amelia Kellar* ^*2,4*^

^*1*^ *Internal Medicine/Pediatrics*, *The University of Chicago Medicine*, *Chicago*, *IL*; ^*2*^ *Pediatric Gastroenterology*, *The University of Chicago Medicine*, *Chicago*, *IL*; ^*3*^ *Gastroenterology, Hepatology, and Nutrition*, *The University of Chicago Medicine*, *Chicago*, *IL*; ^*4*^ *Inflammatory Bowel Disease Center*, *The University of Chicago Medicine*, *Chicago*, *IL*; ^*5*^ *Pediatric Hematology/Oncology*, *The University of Chicago Medicine*, *Chicago*, *IL*

**Background:** Hospitalized pediatric patients with inflammatory bowel disease (IBD) are at significantly higher risk for venous thromboembolism (VTE) compared to the general pediatric population, with reported incidences ranging from 0.37 to 11.79 per 1,000 person‐years (PY). Despite this elevated risk, the absence of formal pediatric guidelines has led to inconsistent practices, with concerns about bleeding often limiting pharmacologic prophylaxis. This study aimed to design and implement a standardized VTE prophylaxis protocol for hospitalized pediatric patients with IBD, assess adherence to the protocol, and evaluate its impact on VTE incidence and prophylaxis.

**Methods:** We conducted a retrospective chart review of all hospitalizations for patients 12 to 18 years with IBD who were admitted to the hospital for an IBD exacerbation or infection at Comer Children's Hospital between January 1, 2012, and June 30, 2024. Patient demographics, clinical characteristics, hospitalization details (admission reason, length of stay [LOS], pharmacologic VTE prophylaxis, and outcomes) were reviewed. In July 2024, a standardized VTE prophylaxis protocol was developed by a multidisciplinary team including pediatric hospital medicine, hematology/oncology, and gastroenterology. The protocol called for VTE prophylaxis with enoxaparin 1 mg/kg/day and sequential compression devices (SCDs) for all hospitalized IBD patients and was implemented as a clinical decision‐support pathway in the electronic medical record (EMR). Education was provided to residents and hospitalist teams. Prospective data were collected post‐implementation from July 1, 2024 to March 30, 2025. Descriptive statistics, Fisher's exact test, and Wilcoxon rank‐sum test were used for analysis.

**Results:** Prior to implementation, 285 patients accounted for 583 hospitalizations. Of these, 11 VTE events occurred in 9 patients, corresponding to an incidence of 2.52 per 1,000 PY. The majority (64%) of VTEs were line‐associated, and 36% occurred despite prophylaxis. Median LOS was significantly longer in hospitalizations complicated by VTE compared to those without (13.9 vs. 4.9 days, p = 0.028, Table 1). Pharmacologic prophylaxis was prescribed in only 10% of hospitalizations prior to protocol implementation. In the nine months following protocol implementation, 24 patients were hospitalized in 27 admissions. Pharmacologic prophylaxis was administered in 48% of admissions, a significant increase from baseline (p < 0.001, OR 9.06, 95% CI 3.9–23.4). Notably, no VTE events occurred during the post‐implementation period.

**Conclusion:** Our findings highlight a previously underrecognized burden of VTE and a substantial gap in prophylaxis practices among hospitalized pediatric patients with IBD. Implementation of a standardized, EMR‐integrated protocol supported by targeted provider education resulted in a meaningful and sustained increase in pharmacologic prophylaxis rates. With no VTE events observed in the nine months following implementation, these data suggest that timely, protocolized intervention may play a critical role in reducing thromboembolic risk. Integration of such protocols into clinical workflows promotes consistency across disciplines and may serve as a model for broader adoption across pediatric healthcare systems. Prospective data collection and long‐term follow‐up are ongoing to evaluate provider compliance and patient outcomes.


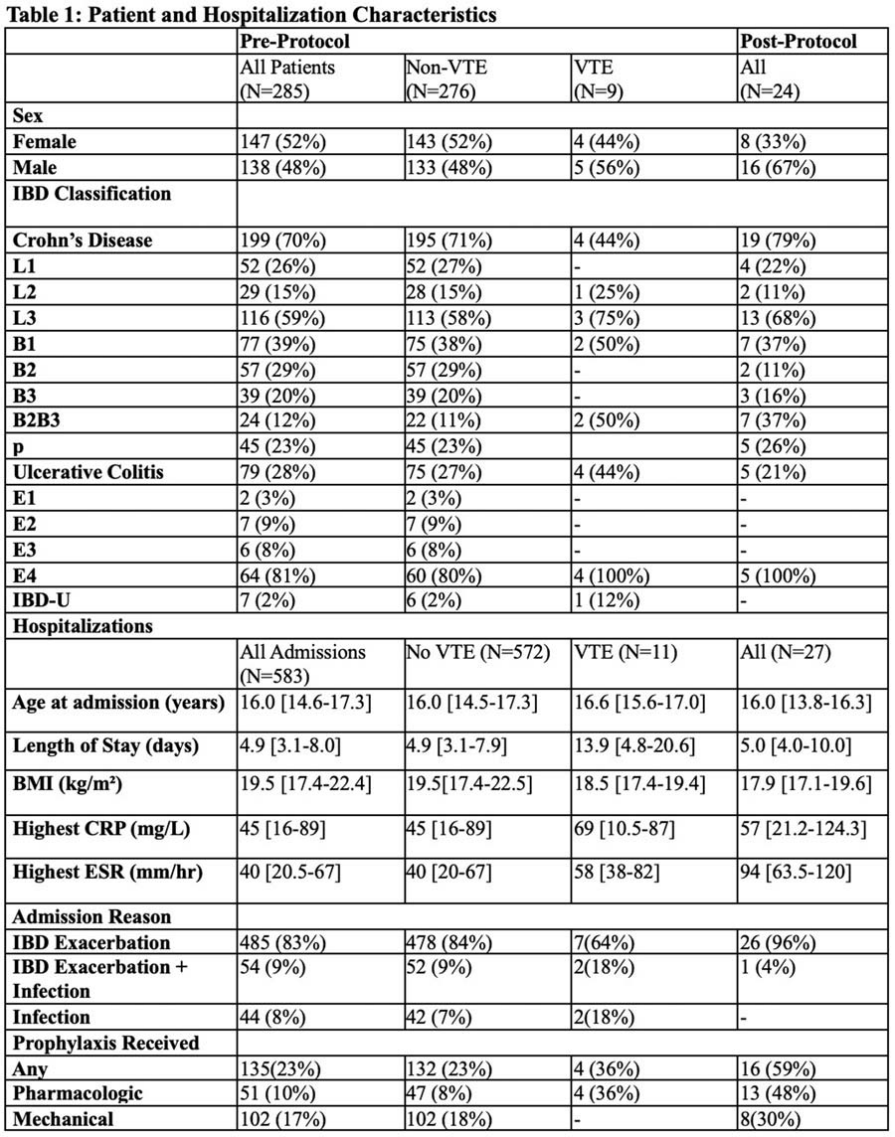


Table 1‐ Patient and Hospitalization Characteristics

## 139* FREQUENCY OF NORMAL LABORATORY EVALUATION AT THE TIME OF INFLAMMATORY BOWEL DISEASE DIAGNOSIS PRIOR TO TREATMENT

*Nicholas Litchin* ^*1*^, *Jeremy Adler* ^*1,2*^

^*1*^ *Pediatric Gastroenterology*, *University of Michigan Michigan Medicine*, *Ann Arbor*, *MI*; ^*2*^ *Susan B. Meister Child Health Evaluation and Research Center*, *University of Michigan Michigan Medicine*, *Ann Arbor*, *MI*

**Background:** Inflammatory bowel disease (IBD) includes Crohn's disease (CD), ulcerative colitis (UC), and IBD unclassified (IBD‐U). Timely diagnosis of IBD is important since delays in diagnosis and treatment are associated with increased disease activity, worse quality of life, and greater risk of developing disease complications. Primary care and emergency department physicians commonly rely on screening laboratory tests to triage patients with gastrointestinal symptoms. Normal laboratory evaluation may be seen as reassuring and may dissuade them from considering IBD or referring to pediatric gastroenterology thus contributing to diagnostic delay. Mack *et al*. (*Pediatr* 2007) reported that some children with IBD have normal labs at diagnosis. However, these findings have not been replicated in a larger patient cohort and have not been evaluated since fecal calprotectin has become routinely available. Our aim was to characterize the frequency of normal laboratory results (i.e. hematocrit, c‐reactive protein [CRP], erythrocyte sedimentation rate [ESR], albumin, fecal calprotectin) in the initial evaluation of patients at IBD diagnosis, prior to initiating treatment.

**Methods:** We evaluated data from the ImproveCareNow (ICN) Network registry. We included all patients who were enrolled in ICN within 30 days of IBD diagnosis and who provided consent/assent for research. We evaluated the first available laboratory results for each patient, if available prior to starting treatment. Laboratory results that were obtained after treatment was started were excluded. Patient and disease characteristics were evaluated. We used physician global assessment (PGA) to summarize disease activity since it is available across diagnoses. We used Chi‐squared testing to describe the associations between normal lab results and patient and disease characteristics.

**Results:** In total, 6,062 patients were included; 44% female, median age 13.9 yr (interquartile range [IQR] 11.2‐16.1), 79% White. 28% UC, 64% Crohn's disease, and 7% IBD‐U. Albumin was normal in 76% of patients (4,262/5,585), ESR was normal in 51% of patients (2,663/5,246), CRP was normal in 35% of patients (1,588/4,542), hematocrit was normal in 55% of patients (3,221/5,885), and fecal calprotectin was normal in 6% of patients (17/292). All blood labs were normal in 16% (629/3,898) of patients with complete bloodwork (Figure 1). Among the subset who also had stool test results, 1% had all labs normal. White patients were more likely to have all normal serum labs than Non‐White patients (17% vs. 13%; p=0.008). Patients with commercial insurance were more likely to have all normal serum labs than patients without commercial insurance (18% vs. 13%; p<0.001). Using the PGA, patients with complete bloodwork had all normal serum results 23% (213/927) of the time if disease was quiescent, 18% (279/1,591) with mild disease, 9% (99/1,091) with moderate disease, and 7% (10/145; p<0.001) with severe disease. Among patients with UC with complete bloodwork, 22% (223/1,025) had all normal results compared to 13% (344/2,594) among those with CD (p<0.001). Patients with UC with more extensive disease were less likely to have all normal serum results (proctitis, 45% [29/64]; left‐sided colitis, 29% [43/4,149]; extensive colitis, 31% (23/75); pancolitis, 18% [104/588]; p<0.001). There were no significant differences between different phenotypes of CD (p=0.76).

**Conclusion:** In a large multicenter pediatric IBD cohort, we found that 16% of patients had completely normal serum lab results at IBD diagnosis. When fecal calprotectin was included, 1% had all normal labs, but the sample size was considerably smaller. Normal serum lab evaluations were more common among patients with less severe and less symptomatic disease. This is important information for referring physicians. In patients for whom there is a high index of suspicion for IBD, the presence of normal laboratory findings does not exclude IBD. The addition of fecal calprotectin improves reliability.


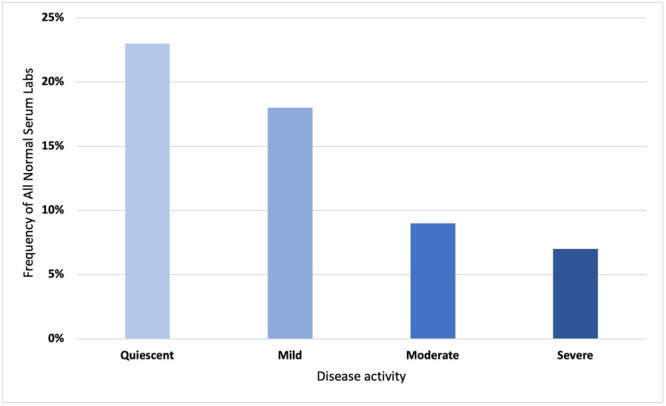


**Figure 1. Frequency of normal serum laboratory values stratified by disease activity.** Proportion of patients with all normal results among those with hematocrit, c‐reactive protein, erythrocyte sedimentation rate, and albumin available. Disease activity was classified according to physicain global assessment.

## 140* LOW SENSITIVITY OF COMMERCIAL ASSAYS FOR VARICELLA ZOSTER VIRUS SEROPOSITIVITY IN PEDIATRIC PATIENTS WITH INFLAMMATORY BOWEL DISEASE

*Freddy Caldera* ^*1*^, *Mary Hayney* ^*5*^, *Jennifer Strople* ^*2*^, *Arthur Kastl* ^*3*^, *Jeremy Adler* ^*4*^, *Margie Boccieri* ^*6*^, *Xian Zhang* ^*6*^, *Millie Long* ^*7*^, *Michael D Kappelman* ^*6*^

^*1*^ *Gastroenterology & Hepatology*, *University of Wisconsin*, *Madison*, *WI*; ^*2*^ *Northwestern University Department of Pediatrics*, *Chicago*, *IL*; ^*3*^ *The Children's Hospital of Philadelphia*, *Philadelphia*, *PA*; ^*4*^ *University of Michigan Department of Pediatrics*, *Ann Arbor*, *MI*; ^*5*^ *University of Wisconsin‐Madison School of Pharmacy*, *Madison*, *WI*; ^*6*^ *The University of North Carolina at Chapel Hill Department of Pediatrics*, *Chapel Hill*, *NC*; ^*7*^ *The University of North Carolina at Chapel Hill Department of Medicine*, *Chapel Hill*, *NC*

**BACKGROUND:** Pediatric gastroenterology guidelines recommend the assessment of varicella zoster virus (VZV) immunity in patients with inflammatory bowel disease (IBD) prior to starting immunosuppression. VZV immunity can be determined based on history of prior infection or documented immunization. Although the Advisory Committee on Immunization Practice (ACIP) does not recommend serologic testing to evaluate vaccine‐induced immunity, many physicians routinely order VZV serologies. We evaluated the sensitivity of the commercial VZV assay using a more sensitive glycoprotein (GP) test as a gold standard.

**METHODS:** We conducted a cross‐sectional study of pediatric IBD patients (ages 7‐18 years) from the PREVENT‐COVID study of COVID vaccine response. Study participants were assumed to be immunized against varicella as they were 1) born in an era and past the age for which varicella vaccination was recommended and 2) from vaccine‐interested families., Demographic and clinical information collected included age, sex, IBD type, disease duration, and medication use. Patients were classified as immunosuppressed if they received any immunomodulator (thiopurine or methotrexate), anti‐tumor necrosis factor (TNF), Janus kinase inhibitor, tacrolimus, ustekinumab, or systemic corticosteroid. Serum samples were tested using a commercial assay (Diasorin Liaison XL chemiluminescent immunoassay, Saluggia VC, Italy) and a more sensitive glycoprotein IgG ELISA (gpELISA) (Serion, Wurzburg, Germany), according to the manufacturer's instructions. We evaluated test characteristics of the commercial assay relative to that of the GP test (Gold Standard). We also utilized univariate logistic regression to identify the factors associated with seropositivity.

**RESULTS:** A total of 176 pediatric IBD patients were included (mean age 12.8 years, 49% female; 68% with Crohn's disease; mean disease duration 4.9 years). The majority (89%) of patients were receiving immunosuppressive therapy, primarily anti‐TNF agents (72%). The remaining baseline demographics are shown in Table 1. Using the GP test as the gold standard, 90% (159/176) of participants demonstrated VZV immunity, while only 69% (122/176) tested positive on commercial assays. Of the 54 participants with negative commercial results, 37 (69%) were positive on the GP test, yielding a sensitivity of 77%, specificity of 100%, positive predictive value of 100%, and negative predictive value of only 32%. The overall false‐negative rate was 69%. Seropositivity on the GP test was 84% in the immunosuppressed group compared to 94% in the non‐immunosuppressed group (P = 0.13). No significant associations were found between seropositivity and age, IBD diagnosis, time since diagnosis, or specific treatments.

**CONCLUSIONS:** Evidence of VZV immunity was found in 90% of pediatric patients with IBD using a highly sensitive GP test. However, commercial VZV serologic assays frequently fail to detect existing immunity in pediatric patients with IBD, with a high false‐negative rate (69%) and low negative predictive value (32%). These findings suggest the limited clinical reliability of commercial assays in evaluating vaccine‐induced immunity. Our results support following the ACIP recommendations to document vaccination history and/or natural infection rather than utilize currently available serological testing for assessing VZV immunity. It is reassuring that no significant differences in immunity were observed between patients receiving various immunosuppressive therapies, suggesting that advanced IBD therapy does not impair previously established VZV immunity. Future research should focus on developing improved commercial assays that can accurately detect vaccine‐induced immunity in immunocompromised populations.

## 149 RISANKIZUMAB FOR THE TREATMENT OF VERY EARLY ONSET INFLAMMATORY BOWEL DISEASE

*Marina Macchi* ^*2*^, *Shreya Gaddipati* ^*2*^, *Astrela Moore* ^*1*^, *Alyssa Baccarella* ^*2,3*^, *Yelizaveta Borodyanskaya* ^*2*^, *Maya Cohen* ^*2*^, *Andrea Cubero* ^*2*^, *Noor Dawany* ^*4*^, *Maire Conrad* ^*2,3*^, *Judith R Kelsen* ^*2,3*^

^*1*^ *Pharmacy*, *The Children's Hospital of Philadelphia*, *Philadelphia*, *PA*; ^*2*^ *Gastroenterology, Hepatology, and Nutrition*, *The Children's Hospital of Philadelphia*, *Philadelphia*, *PA*; ^*3*^ *University of Pennsylvania*, *Philadelphia*, *PA*; ^*4*^ *Biomedical and Health Informatics*, *The Children's Hospital of Philadelphia*, *Philadelphia*, *PA*

**Background:** Risankizumab (RIS), an interleukin (IL)‐23 specific inhibitor, is approved for adults with moderate to severe active Crohn disease (CD) and ulcerative colitis (UC), but limited data exists in the patients diagnosed <6 years, known as very early onset (VEO)‐IBD. We aimed to evaluate the effectiveness and safety of RIS in patients with VEO‐IBD.

**Methods:** We performed a single‐center retrospective cohort study of patients with VEO‐IBD, treated with RIS for IBD. Inclusion criteria were: completion of induction dosing (8 weeks), availability of clinical activity data at baseline and at 8 weeks and no known monogenic disease. Data collected included demographics, disease characteristics, laboratories, endoscopies, surgeries, medications and adverse events at baseline, 8 weeks, 6 months and 12 months.

The primary outcomes were clinical remission (Pediatric Crohn's Disease Activity Index (PCDAI) or Pediatric Ulcerative Colitis Activity Index (PUCAI) ≤10) and steroid‐free clinical remission (SFCR) at 8 weeks, 6 months and 12 months. Secondary outcomes included change in physician global assessment (PGA) and laboratory studies. The PGA was used for patients with ileostomies or colostomies to evaluate clinical activity as PCDAI/PUCAI are not applicable to this subset. Descriptive statistics were used to evaluate differences across time points.

**Results:** Of the 20 children with VEO‐IBD who were treated with RIS, 17 met the inclusion criteria while 3 patients were excluded due to incomplete data. 55% were female and 60% were non‐Hispanic white. The median age at presentation was 5.25 years (IQR 2.39 – 6.75) and the median age at diagnosis was 5.53 (4.34 – 6.8). The median age at RIS initiation was 11.95 years (7.25 – 15.92). 10 patients (59%) had CD, 40% IBDU and 10% UC. Of those with CD, 70% had ileocolonic disease and 20% had perianal disease, while all patients with UC/IBDU had pancolitis. The main indications for RIS were failure of biologic therapy, growth failure and severe endoscopic findings. At baseline, 10% had severe disease based on PGA and 19% had moderate‐severe disease based on PUCAI/PCDAI, and 60% of patients with UC/IBDU had history of severe disease (PUCAI ≥65 or PGA severe). There was a history of prior surgery in 30% of patients and 15 (88%) patients were refractory to ≥2 biologics or small molecules. Most were biologic exposed (16, 94%) and 2 (12%) were on systemic steroids at time of RIS initiation. RIS was used as dual therapy in 10 (59%) patients, including ruxolitinib (n=1), upadacitinib (n=2), tacrolimus (n=1), mercaptopurine (n=1).

RIS induction dosing was 15 mg/kg for patients <40 kg (n=8) and 600 mg for >40 kg (n=9). Two patients discontinued RIS during the follow up period (1 at 6 months and 1 at 12 months) due to lack of response (n=1) or insurance denial (n=1).

Data was available for SFCR in 11 patients at 8 weeks, 8 at 6 months and 5 at 12 months. At 8 week, 8/11 achieved SCFR, and 7 remained in SFCR at 6 months. At 12 months, 4 patients remained in SFCR and 1 had an exacerbation. In addition, 2 patients who did not achieve SFR at 8 weeks, achieved SFCR at 6 months (in total 8/11) and one additional patient 1 at 12 months (in total 5/11). Data was available for the PGA in all 17 patients at 8 weeks, 12/17 at 6 months and 7/12 at 12 months. At 8 weeks, 9/17 were in quiescent disease status, at 6 months, of these 9, 7 patients remained in quiescent disease and an additional 2 achieved quiescent disease (total 9/17) and one had mild disease. At 12 months, of the 7 with complete data, 5 patients continued to have quiescent disease, one had mild disease, and one had moderate disease.

There was a decrease in median PUCAI/PCDAI and an improvement in biochemical markers of disease at the follow‐up points compared to baseline (Table 1). Systemic steroid therapy was successfully discontinued by both patients by week 8 (n=1) and by 6 months (n=1). One patient underwent subtotal colectomy at 8 weeks due to uncontrolled pain and anemia. There were no severe hypersensitivity reactions to RIS or serious adverse events.

**Conclusion:** In our single center experience, risankizumab led to clinical response in most patients with VEO‐IBD during induction, with a favorable safety profile. Limitations include small sample size and retrospective design, However, further prospective data collection is ongoing. Risankizumab therapy may be an effective approach in a subset of patients with VEO‐IBD.


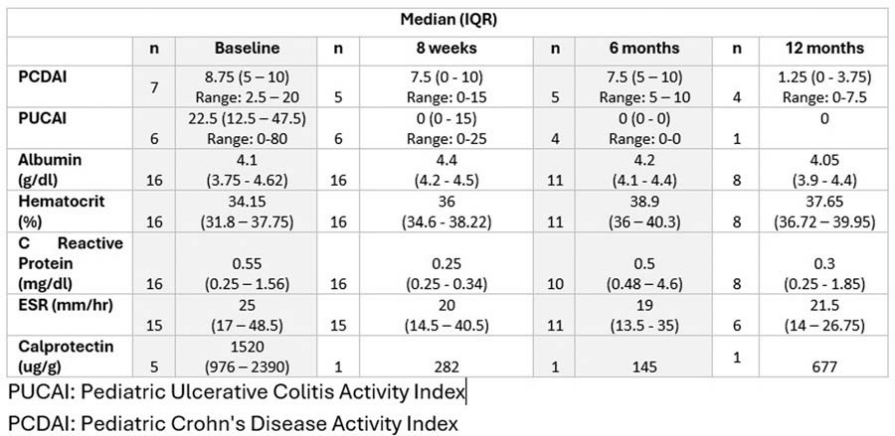


## 153 ANALYZING THE ROLE OF HMG‐COA REDUCATASE IN EPITHELIAL CELLS DURING INFLAMMATORY SIGNALING IN CROHN'S DISEASE

*Irina Geiculescu* ^*1*^, *Sachith Munasinghe* ^*2*^, *Jeeya Sharma* ^*2*^, *Sana Syed* ^*3*^, *Jason Matthews* ^*2*^, *Subra Kugathasan* ^*2*^

^*1*^ *Pediatric Gastroenterology*, *Emory University*, *Atlanta*, *GA*; ^*2*^ *Emory University School of Medicine*, *Atlanta*, *GA*; ^*3*^ *Duke University School of Medicine*, *Durham*, *NC*

**Background:** Although the immune system's role in the persistence of CD is known, emerging evidence suggests that dysregulation of epithelial metabolic pathways occurs during CD. Recent RNA‐seq analysis of patient‐derived ileal organoids points to changes in the mevalonate pathway during CD. The goal of this study was to test the effect of inhibiting the key enzyme in the mevalonate pathway, HMG‐CoA reductase, on inflammatory signals in epithelial cells.

**Methods:** Human organoids derived from ileal and rectal mucosal biopsies of consented patients at Children's Healthcare of Atlanta were cultured in either Intesticult™ Human Growth Medium (OGM) or in‐house organoid medium (OM) and stimulated for 48 hours with different combinations of IFN‐γ (1 ng/mL), TNF‐α (25 ng/mL), or IL‐13 (10 ng/mL), along with 10 µM atorvastatin. The effects of these treatments on IL‐8 and ICAM1 expression levels were measured by real‐time quantitative PCR (RT‐qPCR). Phosphorylated STAT1 (Y701 and S727) and STAT6 (Y641) levels were determined by Western blotting. Cell viability and apoptosis were evaluated using cleaved caspase‐3 levels (Western blot) and the CellTiter‐Glo® 3D Cell Viability Assay, respectively. Conditioned media collected at 48 hours post treatment was used to measure IL‐8 levels released by epithelial cells. The expression levels and patterns of IL‐8 were also examined in single cell RNA sequencing data from mucosal and organoid datasets.

**Results:** Stimulation of intestinal organoids with inflammatory cytokines showed varied levels of responses across patient samples in multiple assays. The most consistent responses from patient organoids were the increases in levels of STAT1 that were phosphorylated at Y701 and S727 during IFN‐γ treatments, and the increased levels of phosphorylation at Y641 of STAT6 with IL‐13 treatment. Stimulation of organoids with TNF‐α increased expression levels of the already highly expressed IL‐8, promoted higher levels of IL‐8 released from the organoids into the media, and it induced the expression of ICAM1 (**Figure 1**). Atorvastatin treatment did not mitigate the intracellular inflammatory signals but decreased the release of IL‐8 (**Figure 2**). When examined at the single cell level, the source of the IL‐8 expression in the mucosal epithelial cell subpopulations was predominantly enterocytes and goblet cells. Interestingly, the levels for IL‐8 and ICAM1 were higher in established CD patients on anti‐TNF than in treatment naïve patients or non‐IBD controls.

**Conclusion:** The results highlight inter‐patient heterogeneity in response to cytokine stimulation. While blocking HMG‐CoA reductase with atorvastatin did not mitigate cytokine‐induced inflammatory signaling measured in our experimental endpoints, the decrease in baseline IL‐8 secretion in intestinal organoids when HMG‐COA reductase is inhibited suggests a potential mechanistic role for cholesterol levels that affect lipid‐raft formation and exocytosis of IL‐8, and potentially affects other inflammatory mediators.


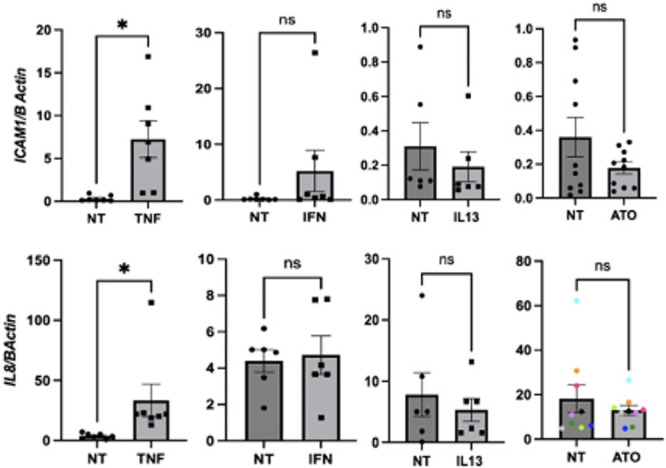


**Figure 1:** Effects of TNF‐a, IFN‐y, IL‐13, and Atorvastatin on Intracellular Signaling Dynamics in Ileal Organoids


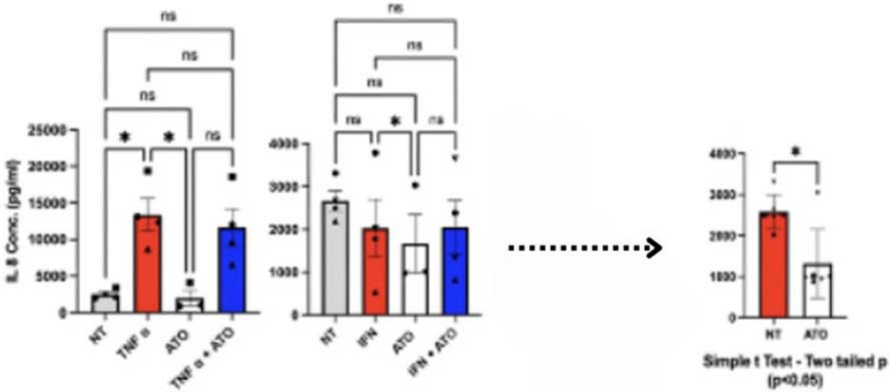


**Figure 2:** Modulation of IL‐8 Protein Secretion by TNF‐a and INF‐y with and without Atorvastatin in Ileal Organoids (ELISA)

## 154 PREVALENCE OF VENOUS THROMBOEMBOLISM IN HOSPITALIZED NON‐SURGICAL PATIENTS WITH INFLAMMATORY BOWEL DISEASE: A SYSTEMATIC REVIEW AND META‐ANALYSIS

*Marie Chen* ^*1*^, *Imad Absah* ^*1*^, *Erin Alexander* ^*1*^, *Puanani Hopson* ^*1*^, *Michael Stephens* ^*1*^, *Edward Loftus* ^*3*^, *Ahmad Al‐Huniti* ^*2*^

^*1*^ *Pediatric Gastroenterology*, *Mayo Clinic Minnesota*, *Rochester*, *MN*; ^*2*^ *Pediatric Hematology/Oncology*, *Mayo Clinic Minnesota*, *Rochester*, *MN*; ^*3*^ *Gastroenterology*, *Mayo Clinic Minnesota*, *Rochester*, *MN*

**Introduction:** Venous thromboembolism (VTE) is a life‐threatening and preventable adverse event in hospitalized patients. Patients with inflammatory bowel disease (IBD) are at a higher risk of VTE. However, the risk in hospitalized non‐surgical IBD patients has not been extensively studied. Our aim is to review the literature on the prevalence of VTE in hospitalized non‐surgical patients with IBD.

**Methods:** We performed a systematic literature review of all publications on hospital acquired venous thromboembolism in patients with IBD published until November 2024 using MEDLINE, EMBASE and the Cochrane Library. Two reviewers (A.A. and M.C.) screened the titles and abstracts of the identified articles. Our primary outcome was the prevalence of venous thromboembolism. Inclusion and exclusion criteria are summarized in Table 1. We calculated pooled prevalence using OpenMetaAnalyst with a random effect model.

**Results:** Our literature search identified 1198 articles, of which 27 met our inclusion criteria (Figure 1). Almost half of the studies were conducted in the United States (13/27, 48%). Seventeen studies focused on adults, five studies focused on pediatrics, four studies included pediatrics and adults, and one study did not report the study population. In the 27 studies, we included 8,986,455 patients (mean age 46.9 years, 57% female sex, 62% Crohn's disease, and 37% ulcerative colitis).

The pooled prevalence of VTE in hospitalized IBD patients is 1.9% (95% CI: 1.5% ‐2.2%, I^2^ 99%; Figure 2). When stratified by the population studied, the pooled prevalence of VTE in hospitalized adult IBD patients is 2.0% (95% CI: 1.5% ‐2.5%, I^2^ 99%), while in hospitalized pediatric IBD patients it is 1.8% (95% CI: 0.8% ‐2.7%, I^2^ 97%). Furthermore, the pooled prevalence of VTE in hospitalized patients with ulcerative colitis is 2.2% (95% CI: 1.2%‐3.2%, I^2^ 99%) and in hospitalized patients with Crohn's disease it is 1.4% (95% CI: 0.8%‐2.0%, I^2^ 99%). Risk of bias assessment indicated that only 2 studies (7%) were rated as high risk. The significant heterogeneity reflected in the I^2^ scores across all pooled prevalence estimates likely stems from variability in study populations as well as exposure and outcome ascertainment.

**Conclusion:** The existing body of evidence along with adult consensus guidelines supports the use of VTE prophylaxis in hospitalized non‐surgical IBD patients due to their increased risk of VTE. However, there is no consensus on VTE prophylaxis in hospitalized non‐surgical pediatric patients with IBD leading to inconsistent use of VTE prophylaxis in this population. Our literature review shows that the VTE prevalence between hospitalized non‐surgical pediatric and adult patients with IBD is comparable. Given the comparable prevalence, our study results suggest that thromboprophylaxis may be indicated in pediatric hospitalized non‐surgical IBD patients. The study limitations include significant heterogeneity among the studies and the potential for publication bias.


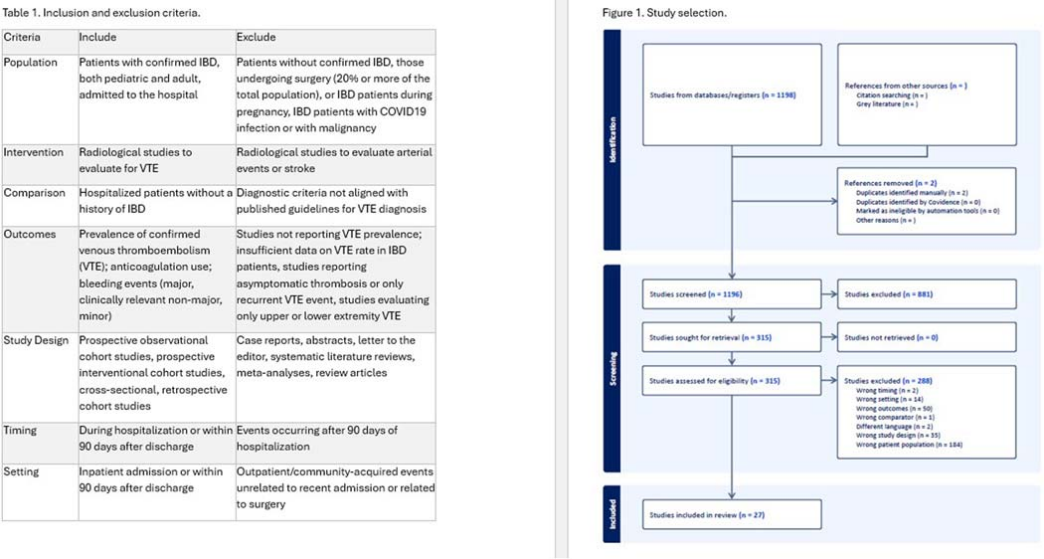



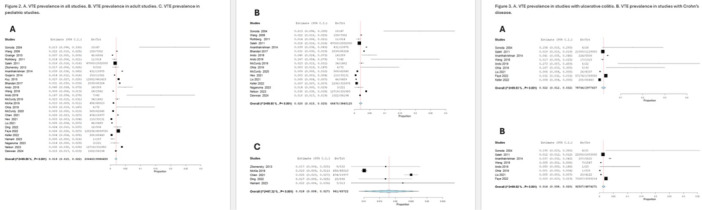


## 155 NEW PEDIATRIC CROHN'S DISEASE PATIENTS WITH STRICTURED ILEOCECAL VALVE AT DIAGNOSTIC COLONOSCOPY: BASELINE CHARACTERISTICS AND TREATMENT OUTCOMES OVER A MINIMUM OF ONE YEAR

*Alexandra Hudson* ^*1*^, *Daniela Isaac* ^*1*^, *Matthew Carroll* ^*1*^, *Adrienne Thompson* ^*2*^, *Troy Perry* ^*3*^, *Lorraine Lu* ^*1*^, *Deirdre McKay* ^*1*^, *Hien Huynh* ^*1*^

^*1*^ *Pediatric Gastroenterology*, *University of Alberta*, *Edmonton*, *AB*, *Canada*; ^*2*^ *Radiology and Diagnostic Imaging*, *University of Alberta*, *Edmonton*, *AB*, *Canada*; ^*3*^ *Surgery*, *University of Alberta*, *Edmonton*, *AB*, *Canada*

**Introduction:** It is known that development of small bowel strictures and need for intestinal resections are complications of medication refractory pediatric Crohn's disease (CD), but it is not well known how well patients respond to medication when they already have stricturing disease present at diagnosis. The aim of this study was therefore to describe characteristics and treatment outcomes in new pediatric CD patients with a strictured non‐traversable ileocecal valve (ICV) at diagnostic colonoscopy.

**Methods:** Patients with suspected inflammatory bowel disease (IBD) were prospectively enrolled into the Edmonton Pediatric Inflammatory Bowel Disease Clinic registry. A retrospective review (2018‐2023) of all newly diagnosed pediatric CD patients from this registry was performed. Demographic and baseline disease variables (endoscopic, biochemical, magnetic resonance enterography (MRE)) were collected. Statistics included descriptive, Kaplan‐Meier survival curves with log‐rank test, and univariate and multivariate logistic regression analyses.

**Results:** 168 patients were included (median age 14 years, IQR 11‐16), of which 16 (10%) had a non‐traversable ICV stricture at diagnosis on colonoscopy. Overall ileocecal resection rate was 13% (n=21/168) over a median follow‐up time of 4.6 years (IQR 4.1‐5.6). ICV stricture patients had a non‐significant trend towards more (n=8/16, 50% vs. n=13/152, 9% non‐stricture patients) and faster time to ileocecal resection (median 4.4 months, IQR 2.2‐6.8 vs. median 9 months IQR 4.4‐42) (p>0.05) **(Figure 1).** The majority of the baseline non‐strictured patients that ended up with ileocecal resection also had no evidence of traversable ICV and/or terminal ileum narrowing at diagnosis (n=11/13, 85%). Multivariate regression identified that endoscopic non‐traversable ICV stricture at diagnosis was the only significant predictor of need for future ileocecal resection (OR 4.67, 95% CI 1.20‐18.17, p=0.026) **(Table 1).** Non‐significant baseline variables included age, BMI z‐score, wPCDAI, penetrating disease, MRE ICV/terminal ileum narrowing with upstream dilation, and use of anti‐TNF biologic as first maintenance medication (p>0.05).

**Conclusions:** One in ten newly diagnosed pediatric CD patients were found to have a non‐traversable ICV stricture already present at diagnosis, which was the only independent baseline predictor of undergoing ileocecal resection within 5 years of diagnosis, even when accounting for penetrating disease. Early anti‐TNF biologic use did not reduce surgical risk. These patients may benefit from early surgical consultation or a combined pediatric gastroenterologist/surgeon clinical visit soon after diagnosis when feasible.


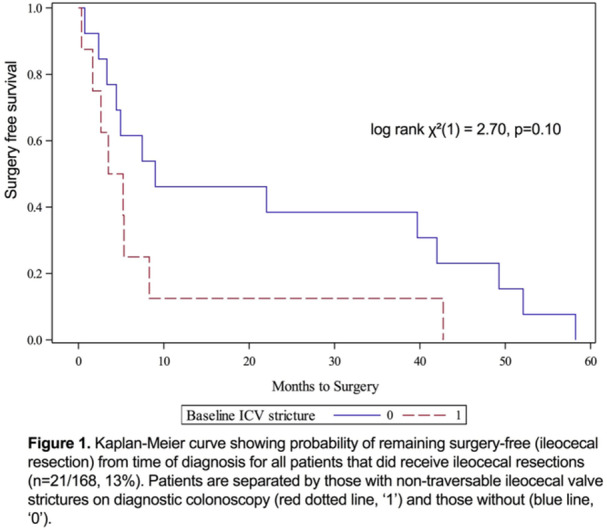



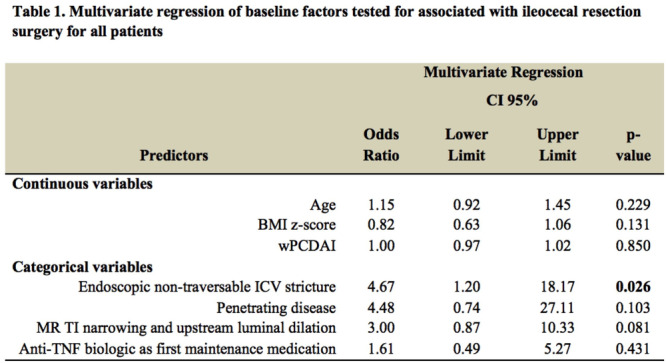


## 161 UPADACITINIB USE IN CHILDREN AND ADOLESCENTS WITH INFLAMMATORY BOWEL DISEASE (IBD): A MULTICENTER, RETROSPECTIVE STUDY

*Shova Subedi* ^*1*^, *Jeffrey Hyams* ^*2*^, *James F Markowitz* ^*3*^, *Joseph A Picoraro* ^*4*^, *Hillary Moore* ^*4*^, *Erica Rabinovich* ^*3*^, *linda Ineus* ^*1*^, *Dena Hopkins* ^*2*^, *Has Phinnara* ^*1*^, *Caroline Jankowich* ^*5*^, *Jason Shapiro* ^*1*^

^*1*^ *Pediatrics*, *Hasbro Children's Hospital*, *Providence*, *RI*; ^*2*^ *Connecticut Children's Medical Center*, *Hartford*, *CT*; ^*3*^ *Cohen Children's Medical Center*, *New York*, *NY*; ^*4*^ *NewYork‐Presbyterian Hospital*, *New York*, *NY*; ^*5*^ *Columbia University*, *New York*, *NY*

**Background:** Upadacitinib (UPA) is a selective JAK inhibitor FDA approved for adults with moderate‐to‐severe Crohn's disease (CD) and ulcerative colitis (UC). It is increasingly being used off‐label in children refractory to other advanced treatments. The aim of this study is to evaluate the safety and efficacy of UPA in pediatric IBD.

**Methods:** We conducted a multicenter, retrospective chart review of patients aged 4 to 18 years with CD and UC who initiated UPA at four pediatric IBD centers. Demographic data, clinical measures and adverse events (AEs) were collected over a 52‐week timeframe. Primary outcomes were clinical response at weeks 4, 8, 12, 26, and 52 after initiation of UPA. Secondary outcomes were rates and types of AEs.

**Results:** 44 children (20 with CD, 21 with UC and 3 with IBD unclassified) were identified (Table 1). Mean age at IBD diagnosis was 11.4 yrs. Mean age at UPA initiation was 14. All patients had previously been treated with an anti‐TNF agent. 91% had been on corticosteroids (CS) prior to UPA initiation with 58% on CS at the time of initiation. 70% had been treated with either an anti‐integrin (n=9), anti‐IL12/23 (n=7) or selective anti‐IL23 (n=1) prior to or during UPA initiation.

Clinical response was evaluated based on assessment of physician global assessment (PGA), weighted Pediatric Crohn's Disease Activity Index (wPCDAI), and Pediatric Ulcerative Colitis Activity Index (PUCAI). Prior to UPA initiation, 80% (n=35) had moderate or severe disease. This decreased to 30% by the end of standard induction at week 12 with 44% noted to be in clinical remission. Patients with UC responded better by week 12 with 67% and 33% in remission or with mild disease, respectively, based on PUCAI. 55% of CD patients were in remission at week 12 based on wPCDAI with 45% noted to have persistence of moderate or severe disease activity.

52 week outcomes were available for 29 patients, 85% of which were in clinical remission based on PGA. The remaining 15% had mild disease and all were improved from time of initiation. 1 patient remained on CS at the time of last available follow up. Declines in inflammatory markers were observed over the 52‐week period (Figure 2).

39 (85%) were induction with 45 mg dose. Of the 5 children who were started on 30 mg dose, 3 required dose escalation for inadequate response. Majority (75%) continued on updacitinib therapy at the end of the study period with good clinical response. 5 children with CD required surgery. One child experienced reactivation of a herpes simplex virus infection which was successfully treated with valacyclovir. No major adverse cardiovascular events (MACE), venous thromboembolism (VTE), or severe infections such as zoster reactivation, were observed.

**Conclusions:** UPA was safe and effective for the majority of patients with many demonstrating clinical response as early as 4 weeks. Patients with UC demonstrated better response compared to those with CD, many of which had advanced disease that required surgery. These results offer promising evidence for the efficacy and safety of UPA for children with IBD. Larger, prospective studies are urgently needed.

**Keywords:** upadacitinib, small molecules, pediatric IBD


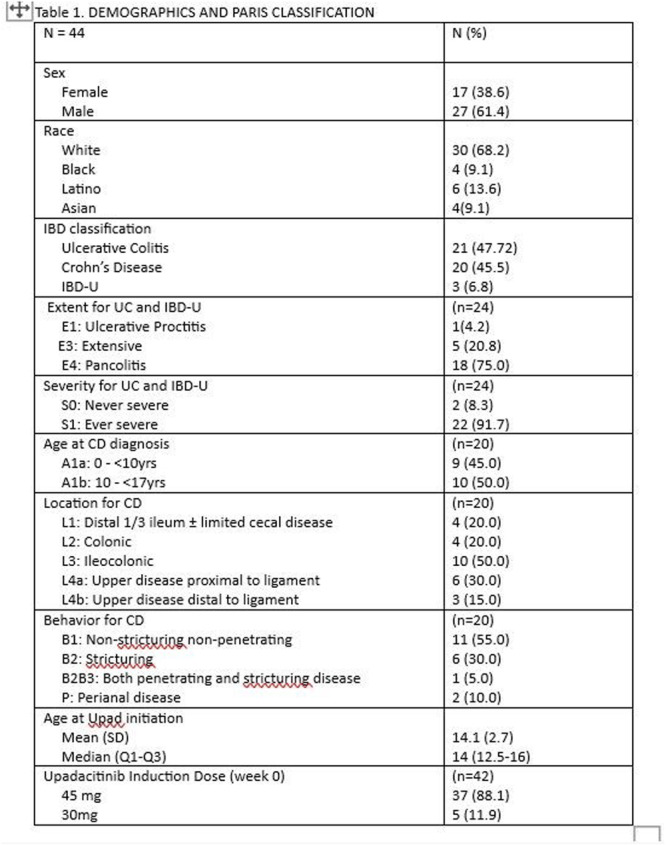


DEMOGRAPHICS AND DISEASE CLASSIFICATION


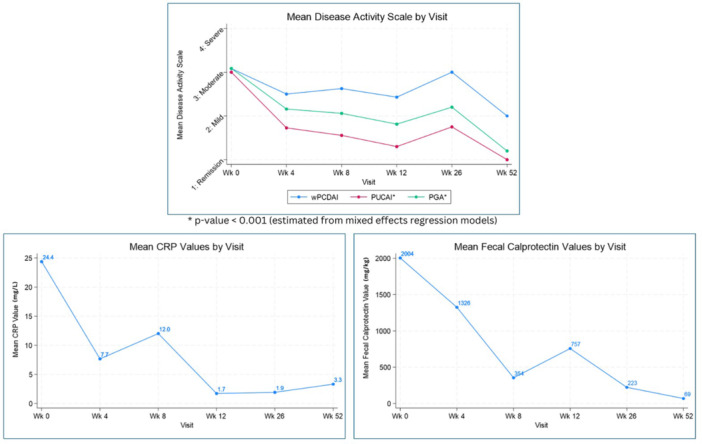


DISESAE ACTIVITY

## 162 LONGITUDINAL BEHAVIORAL HEALTH OUTCOMES IN PEDIATRIC INFLAMMATORY BOWEL DISEASE AT A TERTIARY PEDIATRIC CARE CENTER: EVALUATING THE IMPACT OF PSYCHOLOGICAL SERVICES

*Anjella Manoharan*, *Jasmine Holt*, *Cynthia Sepulveda*, *Laura Bauman*, *D. Brent Polk*, *Jeannie Huang*

*Pediatric Gastroenterology*, *Rady Children's Hospital‐San Diego*, *San Diego*, *CA*

Behavioral health issues have long been recognized as a concern in pediatric inflammatory bowel disease (pIBD). NASPGHAN has issued several position statements on this topic, with the latest update in 2020. These guidelines recommend routine behavioral health screening starting at age 12 y and emphasize the preference for psychological intervention over medication when such issues are identified.

We conduct behavioral health screenings beginning at age 12 y at every clinical encounter at our IBD center. Four standard screens are performed routinely: the Patient Health Questionnaire (PHQ)‐9 for depression if indicated after an initial PHQ‐2 pre‐screen, the Generalized Anxiety Disorder (GAD)‐7 scale for anxiety, and the Patient‐Reported Outcomes Measurement Information System (PROMIS) items for fatigue and pain. Screenings are performed in English or Spanish based on patient preference. In 2023, a psychologist joined our pIBD care team.

The goals of this evaluation were: a) to assess longitudinal behavioral health outcomes in our pIBD patient cohort, and b) to examine whether the involvement of clinic‐based psychological services influenced these behavioral health outcomes.

We identified youth with pIBD seen in 2023‐2024 who met two criteria: (1) confirmed IBD diagnosis, and (2) had at least two behavioral health screenings, allowing for comparisons over time. Behavioral health screening scores were categorized by clinical severity, based on established criteria. The direction of change in clinical severity over time was also assessed and classified into three groups: stable (no change in clinical severity), improved (latest severity better than the worst), and worsened (latest severity worse than the best). Demographic data were also collected. Sankey charts were used to visually represent changes in behavioral health status based on screenings over time (from worst to latest) in each behavioral health domain. Fisher's exact tests were used to analyze the impact of psychological services (yes vs. no) on directional changes in behavioral health outcomes.

Five hundred youth with IBD were evaluated (median age as of January 2024 ‐ 17.3 y, 58.4% male, 67.2% white, 26.4% Hispanic/Latino, 96.2% preferred surveys in English, 61.8% with Crohn's disease). Of these, seventy‐nine (15.8%) youth were evaluated by our psychologist. Overall, behavioral health outcomes improved over time (Figure 1). While improvements in behavioral health were observed in all youth with IBD, receiving clinic‐based psychological services was associated with greater improvements across all behavioral health domains (Table 1).

Behavioral health issues are prevalent in pIBD patients, and screening for these issues should be routinely performed as recommended. Psychological support can be beneficial and should be easily accessible as a standard component of care for youth with IBD.


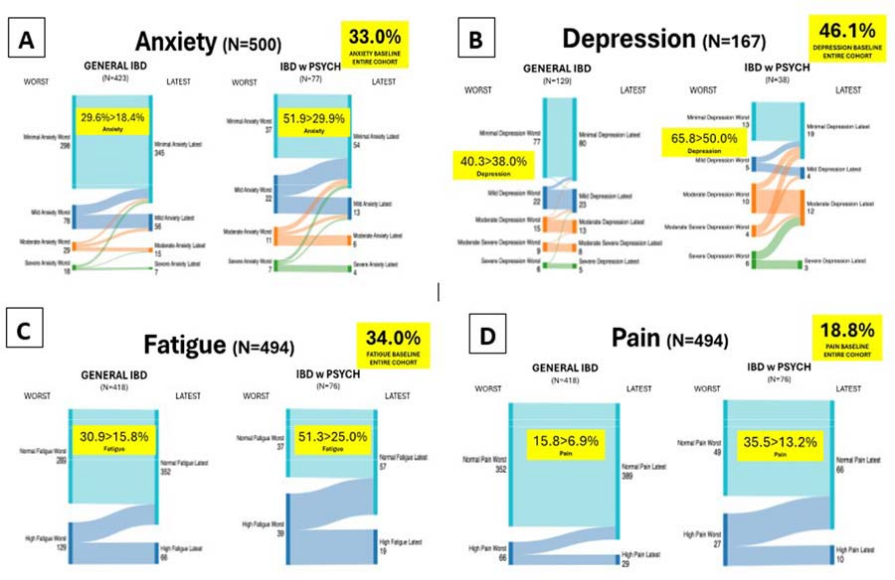


**FIGURE 1.** Sankey Diagrams of Behavioral Health Outcomes from Worst to Latest by Domain: (A) Anxiety, (B) Depression, (C) Fatigue, and (D) Pain.


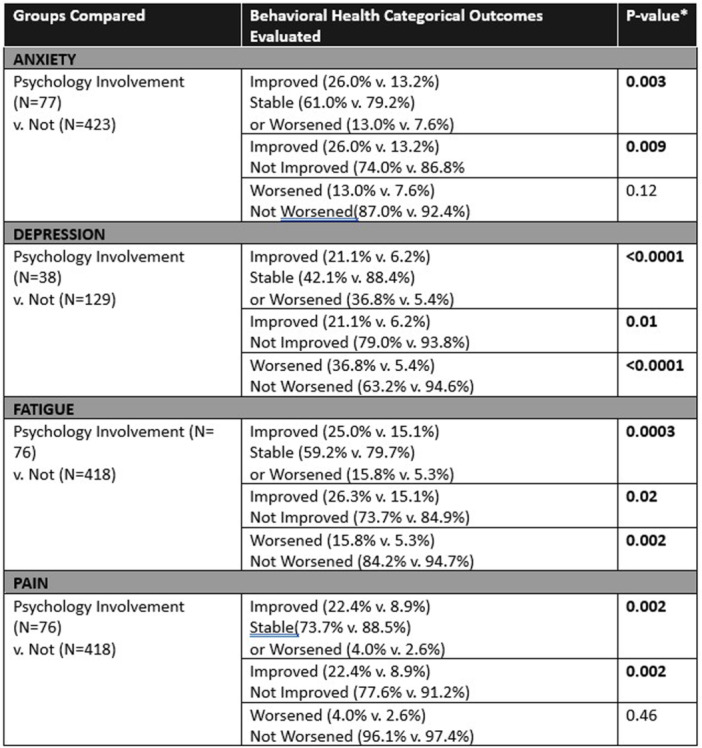


**TABLE 1.** Behavioral health outcomes by domain and psychology case involvement.

Outcomes reported as percentages. *By Fisher's exact test

## 163 LONG‐TERM OUTCOMES OF COMBINED INFLAMMATORY BOWEL DISEASE AND HEPATOBILIARY DISEASE IN CHILDREN AND YOUNG ADULTS: A 10 YEAR EXPERIENCE

*Sarah Bruce* ^*1*^, *Fatima Inam* ^*2*^, *Rhona Hubbard* ^*1*^, *Alenka Brooks* ^*2*^, *Claire Salmon* ^*2*^, *Natalia Nedelkopoulou* ^*1*^

^*1*^ *Paediatric Gastroenterology*, *Sheffield Children's NHS Foundation Trust*, *Sheffield*, *England*, *United Kingdom*; ^*2*^ *Gastroenterology*, *Sheffield Teaching Hospitals NHS Foundation Trust*, *Sheffield*, *England*, *United Kingdom*

**Introduction:** Inflammatory bowel disease (IBD) and concomitant Hepatopancreatobiliary (HPB) disease diagnosed in childhood have been considered to have a milder IBD phenotype, but paradoxically are associated with increased risk of HPB malignancy and colorectal cancer later in life [i],[ii].

**Aims & Methods:** A retrospective analysis of patients diagnosed with concomitant IBD and HPB disease before the age of 16 years, with a follow‐up period up to 30 years of age was undertaken at a linked paediatric‐adult centre with a structured transition program. Demographic data alongside biochemical, histopathological, endoscopic and radiological results were collected for all meeting the inclusion criteria between 2014‐2024.

**Results**:

A total of 38 patients were identied with IBD and concomitant HPB disease, with a mean age at diagnosis of 17.2 years (range 5‐30 years). 63.2% had a primary diagnosis of IBD, 26.3% presented with simultaneous IBD & HPB, whereas only 10.5% had a primary HPB diagnosis. The mean time interval between diagnoses was 17.5 months (0‐144). Ulcerative Colitis (UC) was the most common IBD subtype (26/38 (68.4%)) (Table 1). Concomitant HPB disease included Primary Sclerosing Cholangitis (PSC) (25/38 (65.8%)), Autoimmune Hepatitis (AIH) (9/38 (23.7%)) and Autoimmune Sclerosing Cholangitis (AISC) (4/38 (10.5%)). UC/PSC combination affected 50%,(19/38) UC/AIH (7/38) 18.4% (Table1).

Biologic treatment for IBD was required in 65.8%, of whom 34.2% required a second biologic and 24% of these required further escalation. Infliximab was the most common primary biologic, with vedolizumab the most common second line agent. JAK‐inhibitors were used in 7/38 (18.4%) and 13/38 (34.2%) achieved clinical remission with Azathioprine or 5‐ASA alone. 2/38 (5.26%) with UC/PSC required a subtotal colectomy. There were no cases of colorectal cancer.

In the 19 UC/PSC subtype patients, 3 (15.8%) patients developed HPB malignancy; 2/3 had intrahepatic cholangiocarcinoma, and 1/3 had gallbladder cancer. All 3 patients underwent extensive surgical resection but developed recurrence within 3 years. Palliative systemic therapy was offered, and 1 patient died aged 29 years. Non‐malignant outcomes include stage 4 liver fibrosis in 1 patient with UC/AIH and liver transplantation in another patient with IBD‐U/PSC. IBD/AIH/AISC subtypes were treated with prednisolone (61.5%), azathioprine (76.9%) or mycophenolate mofetil (15.4%). In our cohort, patients did not develop signicant portal hypertension.

**Conclusion:** This study demonstrates the complexity of managing children and young people with concomitant IBD and HPB disease. UC/PSC is a high‐risk subtype that requires intensive monitoring and structured care with implications for transition discussions about future prognosis. Consideration should be given to developing a joint IBD‐Hepatology clinical review to improve care.

**References:**

[i] Ordonez, Lacaille, Canioni, et al. Pediatric ulcerative colitis associated with autoimmune diseases: a distinct form of inflammatory bowel disease? Inflammatory Bowel Diseases. 2012;18: 1809–1817

[ii] Wang, Mousa, Friton et al. Unique Phenotypic Characteristics and Clinical Course in Patients with Ulcerative Colitis and Primary Sclerosing Cholangitis: A Multicenter US Experience. Inflammatory Bowel Diseases. 2020;26(5):774‐779.


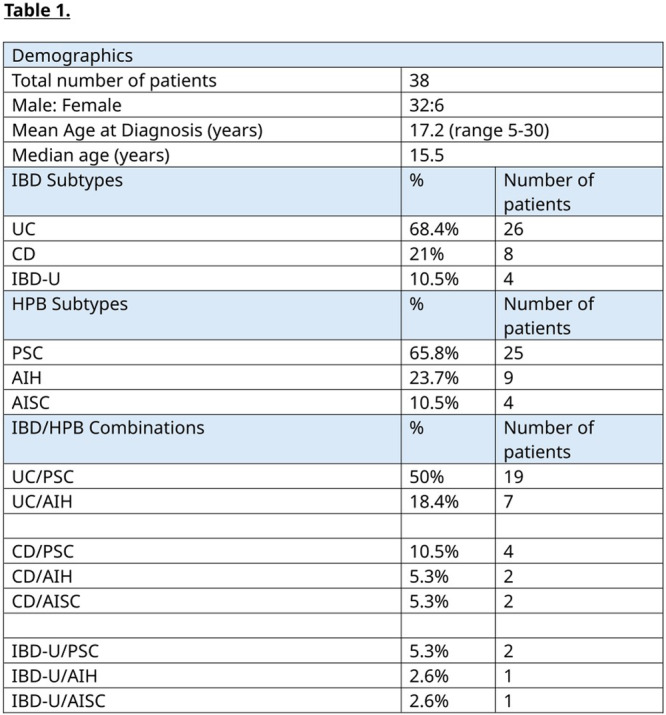


## 164 PERIANAL OUTCOMES IN CHILDREN USING UPADACITINIB FOR MANAGEMENT OF CROHN'S DISEASE

*Arthur Kastl* ^*1*^, *Rosa Hwang* ^*2*^, *Lindsey Albenberg* ^*1*^, *Robert Baldassano* ^*1*^, *Maire Conrad* ^*1*^, *Peter Mattei* ^*2*^

^*1*^ *The Children's Hospital of Philadelphia*, *Philadelphia*, *PA*; ^*2*^ *The Children's Hospital of Philadelphia Division of Pediatric General Thoracic and Fetal Surgery*, *Philadelphia*, *PA*

**Background:** Perianal disease (PD) occurs in nearly 25% of children with Crohn's disease (CD), with significant variation in presentation and disease progression. Unfortunately, perianal fistulae and other manifestations are often complex and refractory to conventional treatments, and upwards of one third to one half of patients will have recurrent complications requiring surgical intervention. Anti‐tumor necrosis alpha (TNFα) therapies are the mainstay of immunosuppression in patients with PD, but there is emerging evidence from adult clinical trials regarding efficacy of janus kinase inhibition in CD and potentially PD. Literature regarding use of upadacitinib in children with inflammatory bowel disease (IBD), particularly with PD, is incredibly limited. The aim of this study was to describe the perianal outcomes in children using upadacitinib for management of luminal CD.

**Methods:** We performed a retrospective descriptive case series of children who were prescribed upadacitinib for management of refractory luminal CD, with subsequent observation of their PD manifestations. The participants must have had a diagnosis of CD before 18 years of age, based on validated ICD‐9 or ICD‐10 diagnostic coding, and also developed perianal manifestations at some point during their PD course. Participants must have had gastroenterology and surgical care at the Children's Hospital of Philadelphia, and their medical and surgical assessments must have occurred during the study period of 1/2014‐12/2024. Abstracted clinical metadata included demographic information, IBD characteristics and treatments, radiologic studies, endoscopic reports, and operative notes. Active PD was defined as having needed surgical assessment or other intervention for PD within 1 year prior to starting upadacitinib. Inactive PD was defined as on‐going presence of PD manifestations, but not needing exam under anesthesia or other surgical intervention within 1 year prior to initiating upadacitinib. Upadacitinib was determined to have improved PD if there was improvement in perianal symptoms and imaging characteristics after its initiation, and relative to the previous immunosuppressive therapy. If there was no appreciable change in perianal symptoms and characteristics via imaging, but without need for additional surgical intervention, the PD was determined to be stable while on upadacitinib. If there was subsequent development of new PD or recurrence of PD complication requiring surgical intervention, then PD was determined to have worsened while on upadacitinib.

**Results:** A total of 15 children were included, with 8 males and 7 females. The median age at diagnosis of CD was 8 years (range 5‐17) and median age of diagnosis of PD was 12 years (range 6‐22). Seven (47%) participants developed PD after diagnosis of CD, while on biologic therapy in most cases, with a mean time to development of perianal disease of 6.7 years (range 2‐17). Perianal fistulae were present in the majority of the group (n=13, 87%), with most having deep fistulae (n=9, 69%). Each deep fistula had an associated deep abscess. Most perianal fistulae (n=10/13, 77%) were complex. Anal fissure occurred in 5 (33%) participants, and anorectal stricture occurred in one. Most of the participants (n=13, 87%) needed surgical assessment and intervention for their PD either prior to and/or after initiation of upadacitinib, and those who did often needed several interventions over time (n=8, 62%). Of the 15 participants, 12 (80%) had inactive PD upon initiation of upadacitinib, and 3 (20%) had active PD. Taking from both the active and inactive groups, PD improved or resolved while on upadacitinib in 4 (27%) participants, remained stable compared to prior immunosuppressive therapy in 5 (33%), and worsened in 6 (40%). All patients who had worsening PD while on upadacitinib had deep and complex fistula to begin with, requiring abscess drainage and seton or other drain placement. There were, however, a few patients with complex and deep fistulae with abscess who improved on upadacitinib. Conversely, nearly all participants with simple and superficial fistulae had either stable or improved PD while on upadacitinib.

**Discussion:** Complex PD may be refractory to conventional medical and surgical management, and new treatment targets are needed in this small but challenging subset of patients. Herein, we found that upadacitinib is potentially helpful in stabilizing or improving PD in pediatric IBD, particularly those with PD isolated to simple uncomplicated fistulae. Conversely, PD may worsen while on upadacitinib in select patients. Further evaluation is needed to ascertain efficacy and safety of upadacitinib in PD phenotypes.

## 165 5‐AMINOSALICYLIC ACID FOR THE TREATMENT OF MILD UNCOMPLICATED PEDIATRIC CROHN DISEASE

*Clarice Cook* ^*1*^, *Christine Quake* ^*1*^, *Maya Nagarkatte* ^*1*^, *Keely McManamon* ^*1*^, *Dazheng Zhang* ^*2*^, *Yong Chen* ^*2*^, *Robert Baldassano* ^*1,3*^, *Ronen Stein* ^*1,3*^

^*1*^ *Gastroenterology*, *The Children's Hospital of Philadelphia*, *Philadelphia*, *PA*; ^*2*^ *University of Pennsylvania*, *Philadelphia*, *PA*; ^*3*^ *University of Pennsylvania Perelman School of Medicine*, *Philadelphia*, *PA*

**INTRODUCTION:** Although infliximab and adalimumab are approved for moderate‐to‐severe pediatric Crohn disease (CD), there are no FDA‐approved treatment options for mild disease in children. While 5‐aminosalicylic acid (5‐ASA) preparations are recommended for adults with mild‐to‐moderate ulcerative colitis and have historically also been used for CD, recent systematic reviews have not shown 5‐ASA therapy to be beneficial for adults with CD. Yet, pediatric CD differs from adult CD in several important ways, including unique growth considerations and more common colonic involvement in children. Therefore, the role of 5‐ASA therapy in mild pediatric CD requires further study.

**AIMS:** To describe the clinical outcomes and adverse events associated with 5‐ASA therapy over 2 years in children with mild, uncomplicated, CD.

**METHODS:** We conducted a retrospective chart review of patients under 18 years of age diagnosed with mild inflammatory CD at the Children's Hospital of Philadelphia between 2014 and 2023. Mild disease was defined by physician global assessment and PCDAI <30. Patients were included if they initiated 5‐ASA therapy within six months of diagnosis and had two years of follow‐up data available. Patients were excluded if they started on an immunomodulator, biologic, or small molecule therapy prior to initiation of 5‐ASA. Additionally, patients with stricturing disease, penetrating disease, perianal disease, and growth failure were excluded. The primary outcome measure was the percentage of patients remaining on 5‐ASA therapy without the need to escalate to immunomodulator, biologic, or small molecule therapies at 2 years. Secondary measures included continued therapy at 1 year, steroid‐free clinical remission based on short PCDAI (<15), mucosal healing based on fecal calprotectin and/or colonoscopy, progression to complicated disease phenotypes, and adverse events—including pancreatitis, nephrotoxicity, and need for hospitalization or surgery.

**RESULTS:** Among the 104 children who met inclusion criteria (mean age 12.0 ± 3.4 years; 68 [65.4%] male), 69 (66.3%) remained on 5‐ASA as their primary therapy at 1 year, and 58 (56.7%) at 2 years respectively. Over the course of follow‐up, 42 (40.3%) patients required escalation to biologic therapy, and 2 discontinued all pharmacologic treatment. At 2 years of treatment, 47/54 (87.0%) of children remaining on 5‐ASA therapy met criteria for steroid‐free remission based on short PCDAI <15 (missing data for 5 participants). Endoscopic and/or calprotectin evaluation was available for 38 patients remaining on 5‐ASA therapy at 2 years of whom 23 (60.5%) showed evidence of mucosal healing. Within the 2‐year study period, 2 patients progressed to a more complicated disease phenotype: one developed both stricturing and fistulizing disease, while the other developed perianal disease. Seven patients were hospitalized due to CD while on 5‐ASA. Four adverse events potentially related to 5‐ASA therapy were reported: headache, decreased appetite, worsening diarrhea, and abdominal pain. No children developed pancreatitis or interstitial nephritis. Although no patients required surgery within the initial 2‐year period, two eventually underwent ileocecectomy at 30 months and 5 years post‐diagnosis, both after escalation to biologic therapy.

**CONCLUSION:** In this cohort of children with mild, uncomplicated Crohn's disease, over half were able to remain on 5‐ASA therapy for two years without requiring escalation to advanced therapies. Among those who continued 5‐ASA, the majority achieved steroid‐free clinical remission, and a substantial proportion demonstrated mucosal healing. Importantly, most patients who failed 5‐ASA did not progress to a complicated disease phenotype or require surgery or hospitalization. The treatment was also well‐tolerated with few adverse events. These findings support the potential role of 5‐ASA as an initial treatment option in carefully selected pediatric patients with mild CD. Further analysis is ongoing to compare 5‐ASA therapy to non‐pharmacological and dietary therapies in mild pediatric CD.

## 166 LEVERAGING QUALITY IMPROVEMENT AND TECHNOLOGY TO IMPROVE SUSTAINED REMISSION RATES IN PEDIATRIC INFLAMMATORY BOWEL DISEASE

*Sakina Neemuchwala* ^*3*^, *Halen Scott* ^*1,2*^, *Srisindu Vellanki* ^*4,2*^, *Jeremy Stewart* ^*4,2*^, *Bhaskar Gurram* ^*4,2*^, *Jacobo Santolaya* ^*4,2*^, *Phuong Luu* ^*4,2*^, *Rhiannon Akey* ^*2*^, *Lauren Lazar* ^*4,2*^

^*1*^ *The University of Texas Southwestern Medical Center Department of Pediatrics*, *Dallas*, *TX*; ^*2*^ *Children's Medical Center Dallas*, *Dallas*, *TX*; ^*3*^ *The University of Texas Southwestern Medical Center Medical School*, *Dallas*, *TX*; ^*4*^ *Division of Pediatric Gastroenterology*, *The University of Texas Southwestern Medical Center Department of Pediatrics*, *Dallas*, *TX*

**BACKGROUND:** Pediatric inflammatory bowel disease (IBD) is a chronic condition that can affect a child's growth and development when left untreated. ImproveCareNow (ICN) is a collaborative quality improvement (QI) network that benchmarks IBD care across pediatric gastroenterology (GI) centers aiming to improve IBD related outcomes. Our project focuses on improving sustained clinical remission rates (SCR), defined as patients with a Physician Global Assessment (PGA) of quiescent disease and no reported relapse for every visit in the last 365 days.

In June 2024, the University of Texas Southwestern/Children's Health Dallas (UTSWCHD) noted their SCR was 43%, below the ICN benchmark of 60%. A multidisciplinary team was assembled to improve this metric using QI methodology. Initial analysis revealed several issues, including variable use of ICN tracking within the Electronic Medical Record (EMR), rapid growth of faculty and patient volume, increased complexity of patients, insurance barriers, and limited visibility between visits. Interventions were designed to address these issues and analyze their effect on SCR.

**METHODS/RESULTS:** A current state assessment was performed by interviewing team members and creating a Process Map, Fishbone Diagram, and Key Driver Diagram. A Global Aim for the project was established to increase SCR to 80% with a SMART Aim of increasing SCR to 60% by 1/1/25. The Key Driver Diagram established ideas for possible interventions, including reeducation of physicians, reevaluating the included population, and use of Post‐Visit Planning (PoVP) with increased touchpoints after a visit via remote patient monitoring (RPM). Based on these tools, interventions were decided, and Plan‐Do‐Study‐Act (PDSA) cycles commenced.

PDSA 1 occurred between 8/1/24‐12/31/24 and addressed the Key Driver of faculty growth. Faculty grew by 40% within one year, expanded to additional clinical sites within the area, and were using 2 separate EMRs. The IBD Center physicians led an educational campaign to teach/re‐teach established and new faculty how to record necessary patient information via the existing embedded form which would be transferred to the ICN database. As a result, SCR improved to 53.9%. The SMART Aim was adjusted to achieve 60% SCR by 6/30/25.

The next Key Driver we addressed was the patient population being included in UTSWCHD ICN statistics. There were patients who had been seen for second opinions and returned to their primary GI, who had transitioned to adult care, or had been lost to follow‐up. In PDSA 2 (1/1/25‐3/31/25), these patients were excluded from the data set, and SCR rates improved to 55.81%.

In PDSA 3, PoVP/RPM was implemented via telephone calls. A list of IBD patients seen in the clinic in the past 2 weeks and not in remission was generated. Patients of “mild” status were called 2 weeks post‐visit, and patients of “moderate” or “severe” status were called 1‐week post‐visit. Symptoms were assessed as “better,” “same,” or “worse” since their visit, and barriers to care were identified (e.g. insurance authorization, health literacy, communication). The call was documented in their EMR chart using a standardized note template and routed to the QI team and their primary GI to ensure appropriate next steps (clinical change, utilization management, etc) were taken. If the patient was improving and intervention was started, their next call was scheduled 4‐6 weeks later, depending on their initial status (moderate/severe patients were followed sooner than mild patients). If their condition was the same or worsening, or an intervention had not been started due to a barrier, they were scheduled for a sooner follow‐up visit and call. PDSA 3 remains ongoing at this time, and the effect on SCR is pending.

**CONCLUSIONS:** Targeted QI interventions can improve SCR, and with the addition of RPM, we are capturing secondary data surrounding insurance approval delays, communication mismatches, and trouble with school accommodations, etc. We hypothesize this intervention will also improve other types of remission, such as prednisone‐free remission, as well as other aspects of patient care and patient satisfaction. In using RPM, patients can share symptom changes and barriers to care between visits, allowing for a dynamic and collaborative effort between the clinical team, patients, and their families to achieve SCR.

Our next PDSA cycle is a small adjustment to our interval patient monitoring: patients not in remission will initially be called two weeks after their appointment, regardless of severity. Future interventions may include utilization of the EMR portal for communication and use of an RPM application to improve monitoring in select populations, such as recently hospitalized patients or very‐early onset IBD. Technology integration should improve sustainability in this project and help us to address important barriers to care and improve remission rates, and therefore, quality of life of our IBD patients.

## 167 PREVALENCE OF FOOD PROTEIN‐INDUCED ALLERGIC PROCTOCOLITIS IN CHILDREN WITH FEEDING DIFFICULTIES BASED ON ENDOSCOPIC EVALUATION: A SINGLE CENTER EXPERIENCE

*Sharef Al‐Mulaabed*, *Joanne Thio*, *Vijay Mehta*, *Justin de Boer*, *Jeffrey Bornstein*, *Devendra Mehta*

*Center for Digestive Health and Nutrition*, *Orlando Health Arnold Palmer Hospital for Children*, *Orlando*, *FL*

**Background:** Children with autism spectrum disorder have a higher rate of feeding difficulties and are frequently referred for feeding therapy. Food protein‐induced allergic proctocolitis (FPIAP), a non‐IgE‐mediated food allergy, can contribute to feeding challenges in children with feeding difficulties due to associated gastrointestinal symptoms. Furthermore, the dietary restrictions required to manage this condition may complicate feeding therapy. FPIAP is primarily a clinical diagnosis, endoscopic evaluation may be warranted, particularly in children with limited communication skills ‐ many of whom are referred for feeding difficulties. Histopathology findings of FPIAP typically include marked eosinophilic infiltration of the rectal mucosa.

**Aims:** To determine the prevalence of FPIAP among patients referred for intensive feeding therapy at our pediatric Feeding Difficulties Center (FDC), based on endoscopic evaluation, and to assess whether the prevalence differs between children with and without autism.

**Methods:** A retrospective review of children who were evaluated for intensive feeding therapy at the FDC, Arnold Palmer hospital for children (Orlando, Fl), between May 2021 and April 2025. Children who underwent flexible sigmoidoscopy (FS) evaluation were included in the study. FS is typically done for children under 3 years of age who are evaluated at our FDC, however, other patients in the FDC who underwent FS for other indications were also included.

**Results:** Of 148 included patients, 102 (69%) were male, with a mean age of 2.96 years (SD 1.52). The cohort was predominantly Hispanic (36.5%), followed by White (32.4%) and African American (19.6%) children, table 1. FPIAP was diagnosed in 15 children (10%). All these diagnosed with FPIAP were ≤ 3 years old, resulting in a prevalence of 17% in that age group (15 of 90).

Among the 148 included children, 62 (41.9%) had autism. Of these, 8 (13%) had FPIAP, compared to 7 out of 86 (8.1%) in those with no autism (p >0.05), table 1. There was no statistically significant association between FPIAP and esophagogastroduodenoscopy (EGD) findings (including reflux or eosinophilic esophagitis), p > 0.05; table 1.

**Conclusions:** FPIAP was identified in a substantial portion of children with feeding difficulties referred to our FDC, particularly among those under 3 years of age. These patients likely had no overt symptoms reported to suggest food protein intolerance prior to evaluation at FDC, indicating a delayed diagnosis. Therefore, endoscopic evaluation should be considered in this population, given the potential for unrecognized gastrointestinal pathology to contribute to their feeding issues.


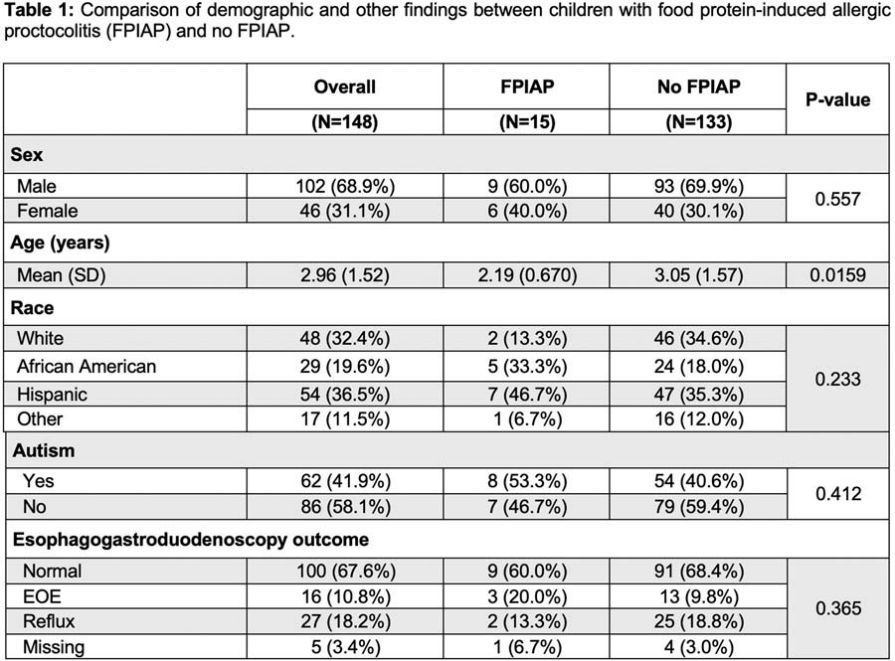


## 170* THE PREVALENCE AND IMPACT OF GLUTEN‐FREE FOOD INSECURITY IN PEDIATRIC CELIAC DISEASE

*Andrew Krueger* ^*1*^, *Rashmi Sahay* ^*5*^, *Emrah Gecili* ^*5*^, *Adrienne Henize* ^*3,6*^, *Andrew Beck* ^*2,6*^, *Melissa Klein* ^*3,6*^, *Daniel Mallon* ^*4,6*^

^*1*^ *Pediatric Residency Program*, *Cincinnati Children's Hospital Medical Center*, *Cincinnati*, *OH*; ^*2*^ *Divisions of General and Community Pediatrics and Hospital Medicine*, *Cincinnati Children's Hospital Medical Center*, *Cincinnati*, *OH*; ^*3*^ *Division of General and Community Pediatrics*, *Cincinnati Children's Hospital Medical Center*, *Cincinnati*, *OH*; ^*4*^ *Division of Gastroenterology, Hepatology and Nutrition*, *Cincinnati Children's Hospital Medical Center*, *Cincinnati*, *OH*; ^*5*^ *Division of Biostatistics & Epidemiology*, *Cincinnati Children's Hospital Medical Center*, *Cincinnati*, *OH*; ^*6*^ *Department of Pediatrics*, *University of Cincinnati College of Medicine*, *Cincinnati*, *OH*

**Introduction:** Celiac disease (CeD) is an autoimmune condition requiring lifelong adherence to a gluten‐free diet (GFD). Food insecurity (FI) refers to not having access to sufficient, quality food to meet one's needs. Given that gluten‐free (GF) foods are more expensive than wheat‐based alternatives, households that include members with CeD may be more prone to FI. Yet, there are few studies assessing FI in pediatric CeD patients. We aimed to 1) evaluate concordance between GF FI and general FI among households with a child with CeD, 2) assess demographic and social factors associated with GF FI, and 3) investigate the impact of GF FI on GFD adherence and tissue transglutaminase immunoglobulin A (TTG IgA) decline.

**Methods:** This was a single center cohort study of English‐speaking pediatric patients (≤18 years old) seen by a pediatric gastroenterologist between 11/1/18 and 11/1/24. A de novo survey assessing GF FI, general FI, GFD adherence, social determinants of health, and sociodemographic information was distributed electronically to all emails and phone numbers listed in the electronic health record (EHR) for caregivers of CeD patients receiving care at Cincinnati Children's.

The EHR was reviewed to collect demographic, serologic, diagnostic, and nutritional information about included patients. Patient addresses were geocoded and spatially joined to census tracts which were linked to community material deprivation index scores ranging from 0‐1, with higher scores indicating greater material deprivation. Four mutually exclusive groups were considered for comparison: GF FI/FI (GF and general food insecure), GF FI/FS (GF food insecure, general food secure), GF FS/FI (GF food secure, general food insecure), and GF FS/FS (both GF and general food secure). Univariate and multivariate logistic regression models were used to identify independent associations between GF FI and GFD adherence. Time to normalization of TTG IgA was assessed using a Kaplan‐Meier curve and log‐rank statistics; we adjusted for pertinent covariates in Cox regression models.

**Results**:

*Demographics*

A total of 1039 CeD patients from 994 households (43 households had multiple children with CeD) received survey invitations (response rate 34.8%; 346/994). Non‐respondents had a significantly higher median deprivation index (0.3 vs 0.2, *p*=0.018); there was no difference by sex, race, and ethnicity. The median age of children of respondents at survey invitation was 12 years (range 1.8‐18 years) and the majority were female (n=221, 64%) and identified as white (n=325, 94%).

More respondents reported GF FI (n=90, 26%) than general FI (n=68, 20%). Most identified as GF FS/FS (n=245, 71%), whereas 16.5% identified as GF FI/FI (n=57), 9.5% as GF FI/FS (n=33), and 3% as GF FS/FI (n=11). The GF FI group had a significantly higher median deprivation index score than the GF FS group (0.25 vs 0.23, *p*=0.0006). GF FI patients were more likely to report lower household incomes than those who were GF FS (34.5% earning less than $60,000 compared to 4.2% of GF secure patients; *p*<0.0001).

*Clinical Characteristics*

There was no significant difference between GF FI and GF FS respondents in the highest ever TTG IgA or proportion of respondents who normalized. However, GF FI respondents took significantly longer to normalize compared to GF FS respondents (*p*=0.01) (**Figure 1**), and this relationship persisted after adjusting for covariates identified as significant in univariate analysis (HR=0.55, 95% CI: 0.34–0.89, *p*=0.01).

*GFD adherence and barriers*

GF FI respondents were less likely to adhere to a GFD than GF FS respondents (*p*=0.0004). GF FI respondents were also significantly more likely than GF FS respondents to report gluten consumption due to increased cost of GF foods (both at the grocery store and while eating outside of the home such as at restaurants), transportation barriers, lack of accessibility to GF foods at nearby locations, personal choice, and by accident (all *p*<0.02).

**Conclusions:** In our cohort, 26% of pediatric CeD patients had GF FI which was associated with longer time to TTG IgA normalization and worse GFD adherence. Assessing CeD families for FI, in general, and GF FI, specifically, could inform actions to improve patient outcomes.


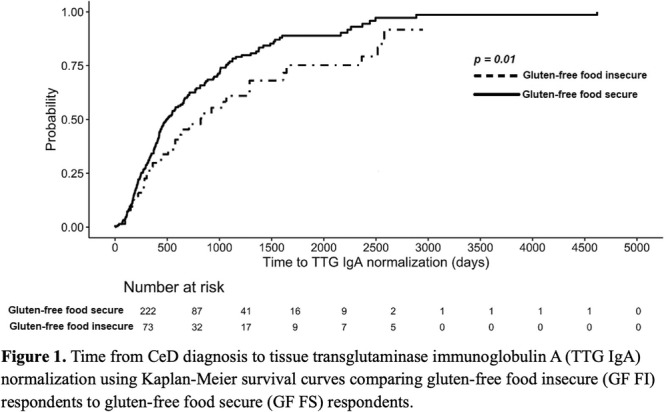


## 171 REAL‐WORLD EVIDENCE OF GROWTH IMPROVEMENT IN CHILDREN RECEIVING ENTERAL FORMULA ADMINISTERED THROUGH AN IMOBILIZED LIPASE CARTRIDGE: A CASE SERIES

*Alvin Freeman* ^*3,4*^, *Elizabeth Reid* ^*5*^, *Terri Schindler* ^*2*^, *Barbara Bice* ^*7*^, *Ashley Deschamp* ^*8*^, *Heather Thomas* ^*8*^, *Ann Remmers* ^*1*^, *David Recker* ^*6*^

^*1*^ *Clinical Operations*, *Alcresta Therapeutics*, *Boulder*, *CO*; ^*2*^ *Pediatric Pulmonology*, *University Hospitals Rainbow Babies & Children's Hospital*, *Cleveland*, *OH*; ^*3*^ *Pediatrics*, *The Ohio State University College of Medicine*, *Columbus*, *OH*; ^*4*^ *Gastroenterology, Hepatology & Nutrition*, *Nationwide Children's Hospital*, *Columbus*, *OH*; ^*5*^ *Clinical Nutrition*, *Children's Hospital of Philadelphia*, *Philadelphia*, *PA*; ^*6*^ *Clinical*, *Alcresta Therapeutics Inc*, *Waltham*, *MA*; ^*7*^ *Pediatric Cystic Fibrosis Center*, *Children's Nebraska*, *Omaha*, *NE*; ^*8*^ *Pediatrics*, *University of Nebraska Medical Center College of Medicine*, *Omaha*, *NE*

**Background:** RELiZORB (Alcresta Therapeutics, Inc.) immobilized lipase cartridge (ILC), a single‐use digestive enzyme cartridge that connects in‐line with enteral feeding circuits to hydrolyze triglycerides in enteral formulas, is cleared by the FDA for use in pediatric and adult patients. Although ILC use has been associated with improved fat absorption, anthropometrics, and enteral feeding tolerance in children and adults with exocrine pancreatic insufficiency and cystic fibrosis, limited data have been published regarding the effect of ILC use on growth in infants and children younger than 5 years of age.

**Methods:** This study was a retrospective evaluation of real‐world data extracted from a third‐party reimbursement program database to support insurance approval. All patients in the database aged 1 to < 5 years who received enteral formula administered through ILC between 2019 and 2024 were included. The change in weight, height/length, and BMI over 12 months was evaluated against Centers for Disease Control and Prevention (CDC) growth charts. Statistical significance of the efficacy endpoints was tested using a one‐sample t‐test at 2‐sided α=0.05. The central (WCG) Institutional Review Board approved the protocol prior to the conduct of the study.

**Results:** A total of 186 patients from 80 clinics in the United States were included in this study; 143 patients (efficacy analysis population) had baseline and follow‐up growth measurements. Patient demographics and baseline characteristics of the total and efficacy populations are comparable (Table 1). Most patients received enteral nutrition as overnight enteral feeds with a mean daily volume of 643 mL (range 120 to 1560 mL) using 1 ILC per 500 mL enteral formula.

There were significant improvements in mean weight and BMI z‐scores (0.63 [p<0.0001] and 0.53 [p=0.006], respectively) from baseline to 12 months after initiation of ILC in the efficacy population (Figure 1). Significant improvement in the mean weight z‐score (0.35 [p<0.0001]) was observed after 3 months of use. The fraction of patients with a BMI ≥ 50^th^ percentile in patients ≥2 years old improved from 28% to 45% over the course of 12 months (p=0.0045 by McNemar test). Post market surveillance did not identify any unexpected risks or increased risks in the 1‐ to 5‐year‐old population compared to older patients.

**Conclusion:** Real‐world evidence showed significant improvements in weight and BMI z‐scores among young children initiating ILC use. This improvement in growth is likely due to the increased availability of critical nutrition delivered through enteral feeding at a time of particularly important physical growth and development.


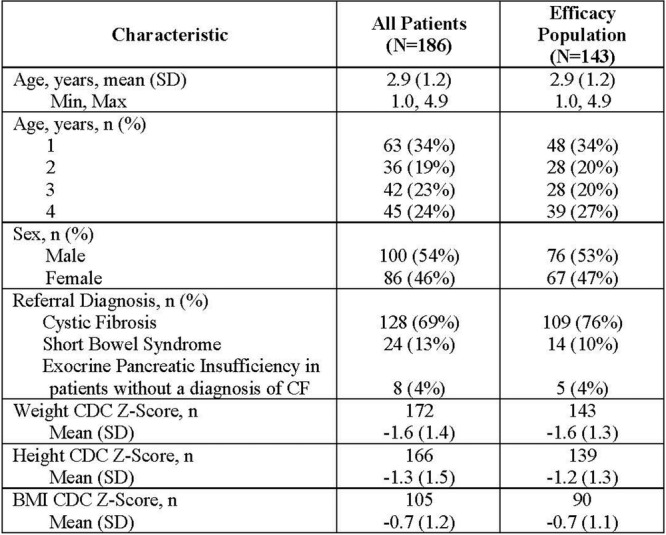


Table 1 Demographics and Baseline Characteristics


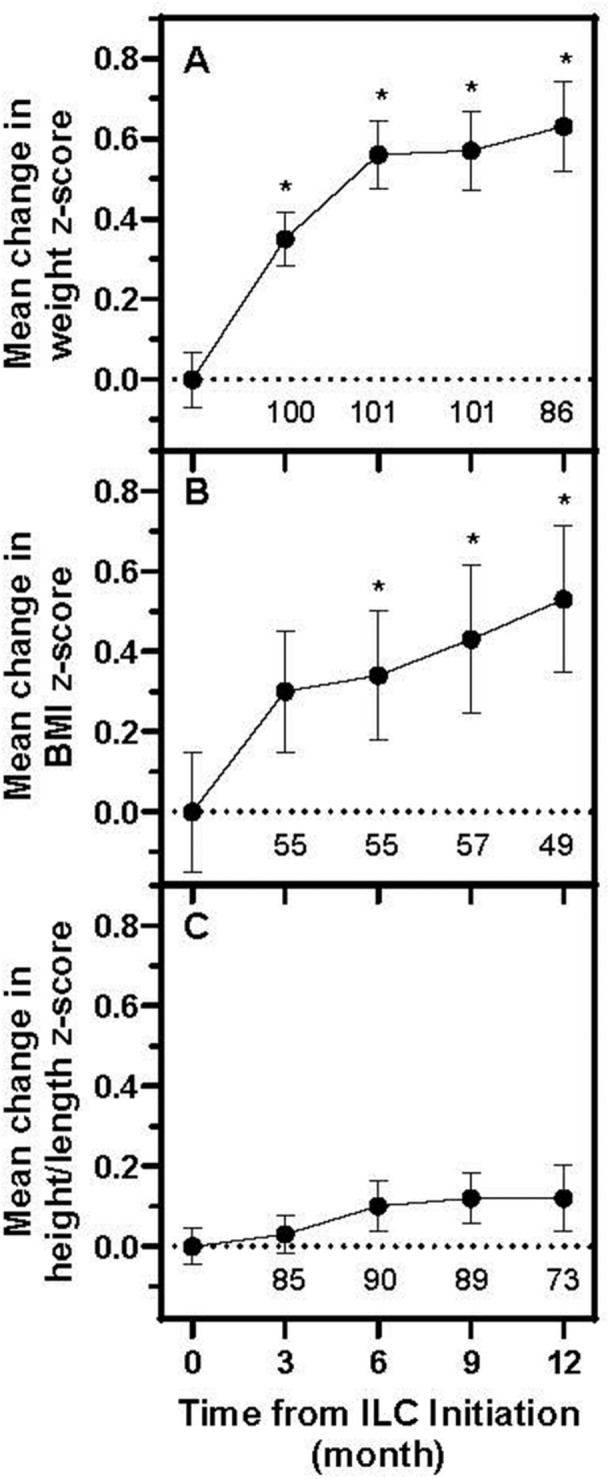


Figure 1 Mean changes in CDC z‐scores over 12 months of ILC use for A) weight, B) BMI in patients ≥2 years old, and C) height. Error bars indicate SEM and asterisks P

## 172 FEASIBILITY AND ACCEPTABILITY OF ROUTINE WEEKLY AT‐HOME GLUTEN IMMUNOGENIC PEPTIDE TESTING IN TEENS IN A 6‐WEEK BEHAVIORAL INTERVENTION FOR CELIAC DISEASE

*Catherine Raber* ^*6,3*^, *Geraldine Mendez‐Gonzalez* ^*1,3*^, *Paige Trojanowski* ^*2,3*^, *Jack Vagadori* ^*3*^, *Hong Bui* ^*5,3*^, *Shayna Coburn* ^*3,4*^

^*1*^ *School of Behavioral & Brain Sciences*, *Ponce Health Sciences University*, *Ponce*, *Ponce*, *Puerto Rico*; ^*2*^ *Primary Care Mental Health*, *Children's National Hospital*, *Washington*,; ^*3*^ *Center for Translational Research*, *Children's National Hospital*, *Washington*,; ^*4*^ *School of Medicine and Health Sciences*, *The George Washington University*, *Washington*,; ^*5*^ *College of Behavioral & Social Sciences*, *University of Maryland*, *College Park*, *MD*; ^*6*^ *Division of Gastroenterology, Hepatology and Nutrition*, *Children's National Hospital*, *Washington*

**Introduction:** Gluten immunogenic peptide (GIP) testing is an innovative tool that may enhance self‐management of the gluten‐free diet (GFD) in individuals with celiac disease (CD). The GIP manufacturer used for this study recommends using GIPs 3 times/week for the greatest adherence measurement accuracy. However, feasibility and acceptability of routine at‐home GIP testing has not been investigated in teens.

**Methods:** As part of a larger randomized controlled trial, weekly GIP testing was incorporated into a 6‐week behavioral intervention. Participants comprised 15 teens (66% female) with CD aged 12‐16 and their parents. Study staff mailed dyads test kits, instructions, gloves, and collection cups. Dyads were counseled on how to use GIPs, incorporate testing into their routine, and completed weekly electronic surveys to report results. Teens were asked to perform at least 2 tests/week and supplied up to 3/week. Dyads provided feedback on GIP user experience (e.g., emotional reaction, ease of use, perceived utility) in qualitative interviews, which were coded for themes. Data analyses examined testing frequency, positivity rate, and subjective experiences using the tests.

**Results:** Teens completed a mean of 2.48 (SD 0.53) tests/week, with 33.3% of the teens completing the requested minimum of 12 tests during the intervention. Eight teens missed one week or more of testing. 100% of GIP results were negative. 95.8% of GIPs were taken as part of the teen's routine, while only 3.6% were taken in response to suspected gluten exposure. Almost all dyads (93.3%) thought the instructions and materials provided by both the test kit and study staff were sufficient to take the tests, though three dyads expressed displeasure with the manufacturer‐provided collection cup. Parents had to remind teens to complete weekly testing in 73.3% of dyads. 46.67% of dyads reported that the parent did some or all of the sample processing, but just over half of the teens reported that they took complete responsibility for processing. 93.3% of teens had either neutral or pleasant feelings associated with weekly testing; one teen felt “disgusted” by testing. 26.67% of dyads had some level of difficulty interpreting test results. The main concern was ambiguity regarding the second stripe on the disc as an indicator of a positive result. Two dyads interpreted a positive result; however, manufacturer reviews confirmed they were negative. Dyads suggested clearer instructions showing that a positive test must change the line's color and real‐life images of positive and negative tests to improve interpretation.

**Conclusion:** Routine GIP testing is feasible and acceptable in teens with CD, but less frequent than recommended. Parental monitoring may be needed for some teens to execute routine testing. Testing instructions were adequate but required supplemental supplies. Finally, our 100% negativity rate indicates a highly adherent sample or perhaps undetected gluten exposure. The design of the kits may lead to false positive interpretations at times, which could cause anxiety.

**Coded Participant Quotes (P: Parent, T: Teen):**

***Adequacy of instructions & materials***

P: I don't think the directions are that great inside the packet. You know, the one that comes…in the kit…I think…the additional information the program gave was more helpful than…the provided literature.

P: The first time we took it…we didn't have the cups yet 'cause you mailed them later. That was a disaster…There's little paper cup things were a mess and…if we had to use that for the whole time…she would have quit…I wouldn't have gotten her to do it.

P: I thought the instructions were fairly straightforward.

***Parent reminders***

T: I forgot like, every time. So it's a good thing one of us is organized.

P: I think the only thing probably annoys her is that I keep reminding her to do it even though she doesn't need the reminder.

***Teen sample processing responsibility***

P: I did it all…Except for peeing.

P: I delegated that entire process to [teen] and wasn't involved in any way, shape or form.

***Difficulty interpreting results***

P: I thought I might have seen like a line, but it wasn't a red line. It was just like a mark…like, is that normal? Is it not normal?

P: I thought the verbal reminder that like the faint line is not necessarily a positive was helpful because I think we were like scrutinizing it and…especially in a generation that's so used to COVID tests and sort of like how any little tiny anything means a positive. And so I think…just a quick verbal reminder that like, nope, it's not exactly the same as a COVID test. You know, a tiny faint line is already existing. So just you need something more glaring than that.

***Feelings surrounding tests***

P: She was like, “I don't want to do it. I don't want to touch my pee.” And I was like, “I don't want to touch your pee.”

T: Happy that the test was negative, and happy that the tests have been showing I'm doing well on the GF diet

## 173 GLUTEN‐INDUCED IL‐2 RELEASE AS A BIOMARKER OF CELIAC DISEASE IN CHILDREN

*Marisa Stahl* ^*2*^, *Christy Hires* ^*2*^, *Melinda Hardy* ^*4*^, *Denis Chang* ^*1*^, *Glennda Smithson* ^*3*^, *Madison Wong* ^*1*^, *Joseph Maxwell* ^*3*^, *Mahsa Zarei* ^*3*^, *Jason Tye‐Din* ^*4*^, *Edwin Liu* ^*2*^, *Jocelyn Silvester* ^*1*^

^*1*^ *Boston Children's Hospital*, *Boston*, *MA*; ^*2*^ *Children's Hospital Colorado*, *Aurora*, *CO*; ^*3*^ *Takeda Pharmaceuticals International AG*, *Cambridge*, *MA*; ^*4*^ *Walter and Eliza Hall Institute of Medical Research Immunology Division*, *Melbourne*, *Victoria*, *Australia*

**Objectives and study:** Gluten‐reactive CD4+ T cells are central to the pathophysiology of celiac disease. Studies in adults have demonstrated that rise in circulating interleukin 2 (IL‐2) following single dose gluten challenge (GC‐IL2) is a highly specific marker of celiac disease. An *in vitro* whole blood assay (WBA) has also been used to detect gluten‐induced IL‐2 release without the need for oral gluten challenge. To date, all reports have been in adults. Therefore, we conducted an interventional study to determine whether gluten‐induced IL‐2 release is a marker of celiac disease in children.

**Methods:** Gluten‐free diet treated children (<18 years) and adults with and without celiac disease were recruited at two pediatric celiac disease centers. Participants had a baseline blood draw for the WBA and to determine baseline IL‐2. For GC‐IL2, a second blood draw was obtained 4 hours after ingestion of 5 grams vital wheat gluten. For the WBA, blood was incubated for 24 hours with gluten peptides or negative control. Plasma IL‐2 levels were determined after 24 hours incubation at 37°C using an electrochemiluminescent assay (MesoScale Discovery). Symptoms after gluten challenge were also queried.

**Results:** To date, 14 children (7 with celiac disease) and 12 adults have completed the study. Overall, GC‐IL2 was 100% specific and 75% sensitive for celiac disease whereas the WBA was 88% specific and 64% sensitive. Those with a false negative test on the whole blood assay tended to be younger, though this was not statistically significant.

**Conclusions:** Gluten‐induced IL‐2 release is a specific marker of celiac disease in children as well as adults. Additional studies are needed to standardize test conditions for GC‐IL2 and to optimize the conditions for the WBA before it can be adopted as a confirmatory test for pediatric celiac disease.

## 176 CLINICAL OUTCOMES OF CHILDREN WITH POTENTIAL CELIAC DISEASE IN NORTH AMERICA: A MULTICENTER RETROSPECTIVE STUDY

*Denis Chang* ^*1*^, *Jocelyn Silvester* ^*1*^, *Maureen Leonard* ^*7*^, *Edyth Moldow* ^*7*^, *Imad Absah* ^*2*^, *Belinda Kwartemaa Nti* ^*2*^, *Catherine Raber* ^*3*^, *Vahe Badalyan* ^*3*^, *Ankur Chugh* ^*6*^, *Abigail Moore* ^*6*^, *Arunjot Singh* ^*4*^, *Maya Khanna* ^*4*^, *Justine Turner* ^*5*^

^*1*^ *Division of Gastroenterology and Nutrition*, *Boston Children's Hospital*, *Boston*, *MA*; ^*2*^ *Division of Pediatric Gastroenterology and Hepatology*, *Mayo Clinic Minnesota*, *Rochester*, *MN*; ^*3*^ *Celiac Disease Program, Division of Gastroenterology, Hepatology, and Nutrition*, *Children's National Hospital*, *Washington*,; ^*4*^ *Division of Gastroenterology, Hepatology, and Nutrition*, *The Children's Hospital of Philadelphia*, *Philadelphia*, *PA*; ^*5*^ *Division of Pediatric Gastroenterology and Nutrition*, *Stollery Children's Hospital*, *Edmonton*, *AB*, *Canada*; ^*6*^ *Division of Pediatric Gastroenterology, Hepatology and Nutrition, Department of Pediatrics*, *Milwaukee Hospital‐Children's Wisconsin*, *Milwaukee*, *WI*; ^*7*^ *Center for Celiac Research and Treatment, Division of Pediatric Gastroenterology and Nutrition*, *Massachusetts General Hospital*, *Boston*, *MA*

**Background:** Small intestinal biopsy is the gold standard for celiac disease (CeD) diagnosis in children with positive celiac serology. Cases where serology is positive, but biopsies are normal or reveal non‐specific changes, including Marsh I lesions, are considered Potential Celiac Disease (PCD)^1^. Prospective studies of European children with PCD report many lost to follow‐up and the remainder having diverse outcomes with some progressing to CeD while others do not^2^. Our study aimed to describe the clinical outcomes of children with PCD in North America to determine if there are geographic differences in diagnosis and management.

**Methods:** Multicenter retrospective study of children (≤18 years) with elevated tissue transglutaminase (tTG) IgA within 6 months of a diagnostic endoscopy between January 2016 and December 2021. All children with small intestinal biopsies demonstrating either intraepithelial lymphocytosis without villous atrophy (i.e., Marsh 1), non‐specific duodenitis or normal histology were considered PCD and included. Cases with known CeD undergoing follow‐up endoscopy or those on a gluten‐free diet (GFD) were excluded. Chart review was performed to extract clinical data both at presentation and during follow up including the treating clinician's final diagnosis (CeD or non‐CeD). Children without a gastroenterologist visit more than 3 months after endoscopy were considered lost to follow‐up.

**Results:** 209 children (53% female, 14% Type 1 Diabetes, 3% Down Syndrome) met criteria for PCD. The most common initial symptoms were abdominal pain (57%), constipation (28%), and weight loss or poor weight gain (26%). The median initial tTG IgA level was 2.3 times the upper limit of normal (ULN); 25 (12%) had a tTG IgA ≥ 10X ULN. 69 children (33%) were lost to follow‐up after the initial endoscopy (**Table 1**). Of the remaining 140 children, 61 (44%) received a CeD diagnosis, including 11/16 (69%) with an initial tTG IgA ≥ 10X ULN. Only 14/61 (23%) children had histopathologic findings consistent with CeD on repeat endoscopy. Six children were diagnosed using the non‐biopsy pathway according to ESPGHAN guidelines, while the other children received a CeD diagnosis by the treating clinician based on Marsh I histopathology (n=18), clinical improvement on a GFD (n=20), or positive serology alone (n=3). More children diagnosed with CeD had a higher initial tTG IgA level, a positive endomysial IgA, and Marsh I histology on initial biopsies than those who remained PCD (**Figure 1**).

**Conclusions:** In our cohort, 44% of children with PCD received a CeD diagnosis, although established diagnostic criteria were used in only a third of cases. More than half of all children were either lost to gastroenterology follow‐up or remained PCD during the study period. Ongoing follow‐up is essential given the variable outcomes in this population.

**References:**

1. Ludvigsson JF, Leffler DA, Bai JC, et al. The Oslo definitions for coeliac disease and related terms. *Gut*. Jan 2013;62(1):43‐52.

2. Auricchio R, Mandile R, Del Vecchio MR, et al. Progression of Celiac Disease in Children With Antibodies Against Tissue Transglutaminase and Normal Duodenal Architecture. *Gastroenterology*. Aug 2019;157(2):413‐420 e3.


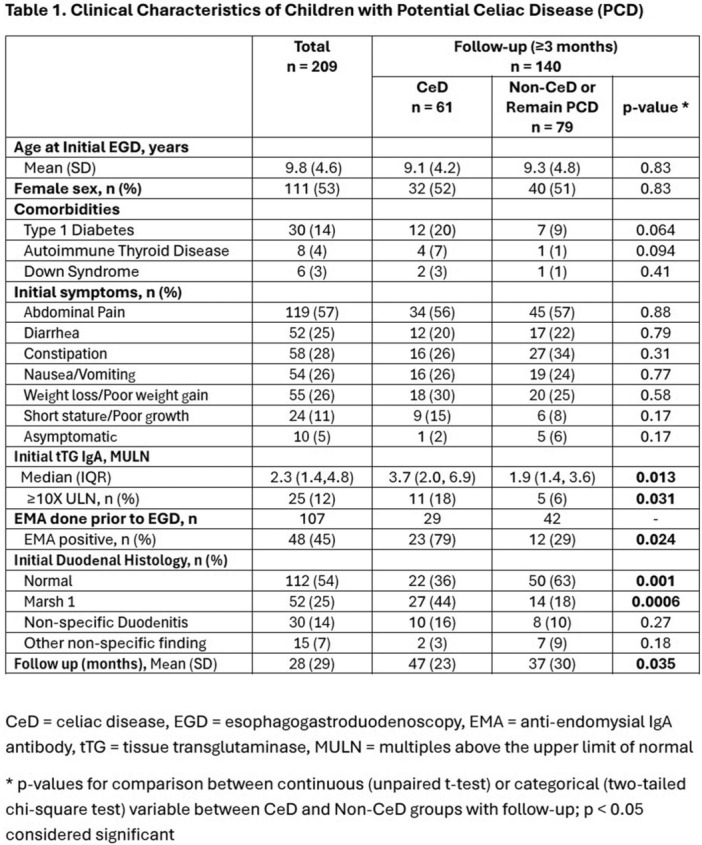



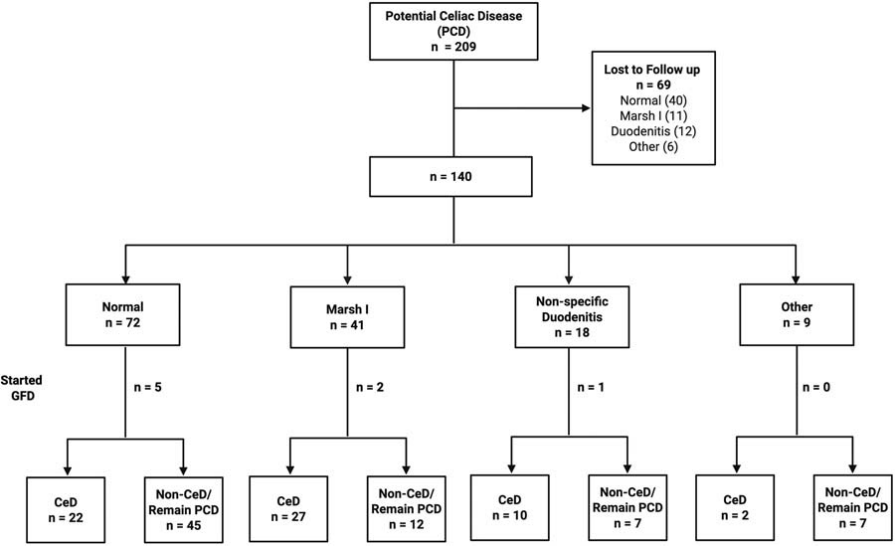


Clinical Outcomes based on initial duodenal biopsies in children with Potential Celiac Disease

## 177 THERAPEUTIC TRIAL OF SUCRAID® (SACROSIDASE) FOR ALLEVIATING GASTROINTESTINAL SYMPTOMS IN PEDIATRIC SUBJECTS STRATIFIED BY DUODENAL SUCRASE ACTIVITY LEVELS

*Tanaz Danialifar* ^*1,2*^, *Jonathan Markowitz* ^*3*^, *Eduardo Rosas‐Blum* ^*28*^, * * ^*4*^, *Mario Tano* ^*5*^, *Melissa Van Arsdall* ^*29*^, *Angela Shannon* ^*6*^, *Youhanna Al‐Tawil* ^*30*^, *Mary Lowry* ^*7*^, *Devendra Mehta* ^*8*^, *Daniel Gelfond* ^*10*^, *Adrian Miranda* ^*9*^, *Namrata Patel‐Sanchez* ^*31*^, *Rohit Kohli* ^*1*^, *Craig Friesen* ^*11*^, *Runa Watkins* ^*12*^, *Molly O'Gorman* ^*13*^, *Thomas Sferra* ^*14*^, *Lesley Small‐Harary* ^*15*^, *Barbara Verga* ^*16*^, *Kevin Watson* ^*17*^, *David Gremse* ^*32*^, *Christopher Hayes* ^*18*^, *Hillary Bashaw* ^*19*^, *Aldo Maspons* ^*20*^, *Shikib Mostamand* ^*21*^, *Miguel Saps* ^*22*^, *Maryam Shambayati* ^*23*^, *Julie Khlevner* ^*24*^, *Uwe Blecker* ^*25*^, *Stephanie Oliveira* ^*26*^, *Shaija Kutty* ^*27*^

^*1*^ *Children's Hospital Los Angeles*, *Los Angeles*, *CA*; ^*2*^ *University of Southern California Keck School of Medicine*, *Los Angeles*, *CA*; ^*3*^ *Prisma Health*, *Greenville*, *SC*; ^*4*^ *Children's Center for Digestive Healthcare*, *Atlanta*, *GA*; ^*5*^ *Kidz Medical*, *Hollywood*, *FL*; ^*6*^ *Happy Tummies*, *Flowood*, *MS*; ^*7*^ *GI Alliance*, *Flowood*, *MS*; ^*8*^ *Orlando Health*, *Orlando*, *FL*; ^*9*^ *Medical College of Wisconsin*, *Milwaukee*, *WI*; ^*10*^ *WNY Pediatric Gastroenterology*, *Buffalo*, *NY*; ^*11*^ *Children's Mercy Kansas City*, *Kansas City*, *MO*; ^*12*^ *University of Maryland Baltimore*, *Baltimore*, *MD*; ^*13*^ *Primary Children's Hospital*, *Salt Lake City*, *UT*; ^*14*^ *University Hospitals Health System*, *Cleveland*, *OH*; ^*15*^ *Stony Brook University Hospital*, *Stony Brook*, *NY*; ^*16*^ *Atlantic Health System Inc*, *Morristown*, *NJ*; ^*17*^ *Akron Children's Hospital*, *Akron*, *OH*; ^*18*^ *Children's National Hospital*, *Washington*,; ^*19*^ *Children's Healthcare of Atlanta Inc*, *Atlanta*, *GA*; ^*20*^ *Newco 3 A Research*, *El Paso*, *TX*; ^*21*^ *Lucile Salter Packard Children's Hospital at Stanford General Pediatrics*, *Palo Alto*, *CA*; ^*22*^ *University of Miami Miller School of Medicine*, *Miami*, *FL*; ^*23*^ *Measurable Outcomes Research*, *Oklahoma City*, *OK*; ^*24*^ *New York‐Presbyterian/Columbia University Irving Medical Center*, *New York*, *NY*; ^*25*^ *Nemours Children's Health*, *Pensacola*, *FL*; ^*26*^ *Cincinnati Children's Hospital Medical Center*, *Cincinnati*, *OH*; ^*27*^ *The Johns Hopkins Hospital*, *Baltimore*, *MD*; ^*28*^ *Pediatric GI of El Paso*, *El Paso*, *TX*; ^*29*^ *McGovern Medical School of UT Health*, *Houston*, *TX*; ^*30*^ *GI for Kids PLLC*, *Knoxville*, *TN*; ^*31*^ *UCSF Benioff Children's Hospital Oakland*, *Oakland*, *CA*; ^*32*^ *University Hospital*, *Mobile*, *AL*

**Introduction:** SSDXP‐13 was a Phase 4, open‐label, multicenter exploratory study designed to evaluate the therapeutic effects of a 7‐day trial of sacrosidase on GI symptoms in pediatric patients with suspected congenital sucrase‐isomaltase deficiency (CSID). Prior sacrosidase trials have largely focused on patients with confirmed sucrase deficiency, defined as duodenal sucrase activity <25 µmol/min/g protein. In contrast, SSDXP‐13 investigated treatment response across a broader range of sucrase activity, based on emerging evidence that hypomorphic sucrase‐isomaltase variants or moderate sucrase activities levels are associated with functional GI disorders and more frequent loose stool^1^. It was hypothesized that therapeutic response to sacrosidase treatment would follow a continuum, with greater benefit in those with lower sucrase activity, but with measurable improvements even in children with moderate sucrase activity (25‐35 µmol/min/g protein). Additionally, the study aimed to assess treatment response across a range of baseline symptom frequencies and to explore the potential modifying role of lactase deficiency—both of which have been understudied in this population.

**Methods:** Pediatric subjects were eligible if they presented with duodenal disaccharidase assay results for sucrase, lactase, maltase, and palatinase with normal histology and had at least one of the following symptoms (diarrhea, abdominal pain, bloating, gas, nausea, or borborygmi) occurring ≥3 times per week for ≥3 months. The study included a screening visit, a 7‐day run‐in period (no sacrosidase), a 7‐day treatment period (daily sacrosidase), and a follow‐up visit. Subjects were stratified into one of four sucrase cohorts: Cohort 1 (<25 µmol/min/g), Cohort 2 (≥25 to <35), Cohort 3 (≥35 to <55), and Cohort 4 (≥55). As part of the daily symptoms questionnaire (DSQ), participants or parent/caregiver reported the daily frequency of six functional GI symptoms (bloating, abdominal gas, borborygmi, bowel movement, nausea and stomach pain) using a 0–5 point scale during both run‐in and treatment periods. Between‐cohort comparisons in symptom outcomes were made using Cohort 4 as the reference. As part of treatment satisfaction questionnaire (TSQ), participants were asked to rate the change and severity in symptoms and overall satisfaction during treatment. Participants also recorded daily sacrosidase dosing times and meal types during treatment.

**Results:** A total of 312 subjects were treated across 32 U.S. centers: Cohort 1 (n=172), Cohort 2 (n=75), Cohort 3 (n=18), and Cohort 4 (n=47). Of these, 298 (95.5%) completed the study. The primary efficacy endpoint was change from run‐in to Day 7 of treatment in the daily average frequency score for the combined six GI symptoms. A restricted maximum likelihood‐based mixed‐effects model for repeated measures revealed a statistically significant reduction in mean daily symptom frequency scores for Cohort 1 versus Cohort 4 and Cohort 2 versus Cohort 4 (p<0.05). Based on an analysis of covariance, the weekly mean frequency scores were significantly lower for bloating in Cohorts 1 (p=0.006) and 2 (p=0.015) compared to Cohort 4; stomach pain in Cohort 1 vs Cohort 4 (p=0.024); and nausea in Cohorts 1 vs Cohort 4 (p=0.017). No statistically significant changes were observed for borborygmi, abdominal gas, or bowel movement.

Responder status was defined as a ≥1‐point decrease in the weekly mean composite symptom frequency score during treatment compared to run‐in, calculated only for subjects with at least four days of symptom data in both periods. Responder rates were 18.7% in Cohort 1, 22.2% in Cohort 2, 6.3% in Cohort 3, and 8.3% in Cohort 4. A logistic regression model adjusting for baseline symptom frequency, age group (<4 vs ≥4 years), and sucrase activity cohort showed that among children ≥4 years old, responder rates were significantly higher in Cohort 1 vs Cohort 4 (p=0.020). Among children <4 years old, the highest responder rate was observed in Cohort 1 (45.5%), whereas in children ≥4 years old, Cohort 2 had the highest responder rate (23.0%). Baseline lactase activity did not significantly modify response to sacrosidase.

**Conclusions:** This study provides evidence that sacrosidase is most consistently effective in subjects with sucrase activity <25 µmol/min/g protein but can also yield therapeutic benefit in those with moderate sucrase activity (25–35 µmol/min/g protein), particularly by Day 5 of treatment. Minimal response was observed in subjects with sucrase activity ≥35 µmol/min/g. These findings support the potential therapeutic benefit of sacrosidase in a broader range of pediatric patients with functional GI symptoms and suspected carbohydrate intolerance. An analysis of the relationships between dosing adherence and DSQ and TSQ responses is ongoing.

1. Deb C et al., Gastroenterology (2020) **72**:29‐35

## 178 CAN WE DO LESS? REDUCING DISCARDED BLOOD VOLUME IN LABORATORY TEST WITHDRAWALS FROM CENTRAL VENOUS CATHETERS IN PEDIATRIC INTESTINAL FAILURE PATIENTS

*Yochai Frenkel* ^*2,1*^, *Worood Matanis* ^*2*^, *Or Kobi* ^*3,4*^, *Marielle Kaplan* ^*5,7*^, *Halima Dabaja‐Younis* ^*6,7*^, *Inna Spector‐Cohen* ^*4,7*^

^*1*^ *Pediatrics*, *Clalit Health Services*, *Tirat HaCarmel*, *Haifa District*, *Israel*; ^*2*^ *Pediatrics A*, *Rambam Health Care Campus*, *Haifa*, *Haifa District*, *Israel*; ^*3*^ *Pediatrics B*, *Rambam Health Care Campus*, *Haifa*, *Haifa District*, *Israel*; ^*4*^ *Pediatric Gastroenterology and Nutrition Institute*, *Rambam Health Care Campus*, *Haifa*, *Haifa District*, *Israel*; ^*5*^ *Division of Laboratory Medicine*, *Rambam Health Care Campus*, *Haifa*, *Haifa District*, *Israel*; ^*6*^ *Pediatric Infectious Disease Unit*, *Rambam Health Care Campus*, *Haifa*, *Haifa District*, *Israel*; ^*7*^ *Technion Israel Institute of Technology The Ruth and Bruce Rappaport Faculty of Medicine*, *Haifa*, *Haifa District*, *Israel*

**Objectives and Study:** Central venous catheters (CVCs) play a crucial role in managing pediatric intestinal failure (PIF) patients reliant on parenteral nutrition (PN). Current protocols typically recommend discarding 5 mL of blood prior to collecting samples. This practice often exceeds the actual CVC "dead‐space" volume, leading to unnecessary blood loss. This study aimed to assess the reliability of blood test results when discarding smaller volumes compared to the standard 5 mL discard, with considerations for catheter size.

**Methods:** This prospective cohort study included 20 PIF patients with single‐lumen tunneled Hickman CVCs. Blood samples were obtained during routine clinical care, using varying discard volumes (1–5 mL). Laboratory parameters analyzed included hemoglobin, white blood cell count, platelets, sodium, potassium, glucose, and creatinine. Results from samples collected after discarding 1, 2, 3, and 4 mL of blood were compared to those obtained using the traditional 5 mL discard. Additionally, patients were stratified into subpopulations based on CVC size: small (2.7–6.6 Fr) and large‐bore (9.6 Fr).

**Results:** A total of 125 blood draws were analyzed. Across the entire cohort, a 2 mL discard volume yielded results comparable to 5 mL discard. For patients with larger CVCs, samples obtained after discarding 2 mL provided results comparable to samples after discarding 5 mL (Pvalue for Hemoglobin<0.001, White Blood Cells<0.001, Platelets=0.001, Glucose=0.25, Potassium=0.88, Sodium<0.001, Creatinine=0.29), while for smaller CVCs, discarding 1 mL was sufficient (Pvalue for Hemoglobin<0.001, White Blood Cells=0.067, Platelets<0.001, Glucose=0.86, Potassium=0.002, Sodium<0.001, Creatinine=0.081). Although some laboratory parameters showed statistically significant differences, these were clinically insignificant, indicating that reducing discard volume does not compromise clinical decisions.

**Conclusions:** Reducing discarded blood volume to 2 mL in 9.6 Fr, or 1 mL in 2.7–6.6 Fr CVCs, preserves diagnostic accuracy while minimizing blood loss in PIF patients, potentially mitigating iatrogenic anemia. Further research is warranted to validate these findings for additional laboratory parameters, such as coagulation studies, and across different CVC types.


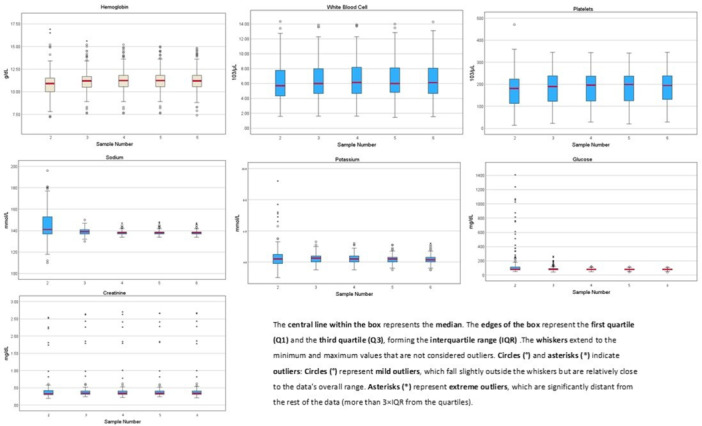


LABORATORY RESULTS ACROSS ALL THE ENTIRE

STUDY COHORT

## 179 NEIGHBORHOOD SOCIOECONOMIC DEPRIVATION ASSOCIATES WITH DECREASED SEROLOGIC NORMALIZATION RATES IN PEDIATRIC CELIAC DISEASE

*Telly Cheung* ^*2*^, *Mala Setty* ^*2*^, *Cynthia Fenton* ^*1*^, *Christine McDonald* ^*2*^, *Patrika Tsai* ^*2*^, *Jennifer Lai* ^*1*^, *Sharad Wadhwani* ^*2*^

^*1*^ *Medicine*, *University of California San Francisco*, *San Francisco*, *CA*; ^*2*^ *Pediatrics*, *University of California San Francisco*, *San Francisco*, *CA*

**Background:** Children with celiac disease (CeD) rely on strict dietary gluten elimination to reduce end‐organ damage. Adherence to a costly gluten‐free diet (GFD) can be challenging for low‐income families, leading to a persistently elevated tissue transglutaminase (tTG) IgA. We evaluated how neighborhood deprivation impacts tTG IgA normalization, a key marker of effective gluten elimination.

**Methods:** We conducted a single‐center retrospective cohort study of 207 children ≤18 years old with serology‐ or biopsy‐based diagnosis of CeD identified from electronic health records between 1/1/2013‐12/31/2023. The primary exposure was the neighborhood deprivation index, a continuous measure (0‐1) derived from the 2018 five‐year American Community Survey. We categorized children from lowest to highest deprivation by quartiles (Q): Q1 (<0.15), Q2 (0.15‐0.21), Q3 (0.21‐0.29), or Q4 (>0.29). The primary outcome was the normalization of tTG IgA over the 60 months after seropositivity. We used Cox regression models to estimate hazard ratios for tTG IgA normalization, adjusting for race, ethnicity, primary‐spoken language, and insurance type in multivariate analyses. We tested the interaction between deprivation and tTG IgA levels at diagnosis on tTG IgA normalization.

**Results:** Of 207 children, the median deprivation index was 0.22 (IQR: 0.14, 0.29). Higher deprivation was associated with participants identifying as Black or Other race, Hispanic, non‐English primary‐speaking, and having public insurance.

In univariate analysis, children in the highest quartile of neighborhood deprivation had a 50% lower normalization rate compared to those in the lowest quartile (**Q4**: HR 0.50; 95%CI 0.27, 0.92; p=0.03). In univariate models testing interaction, tTG IgA ≥10x upper limit of normal (ULN) uniformly reduced the normalization rate among children from deprived neighborhoods (**Q2**: HR 0.20; 95%CI 0.08, 0.53; p=0.001; **Q3**: HR 0.40; 95%CI 0.19, 0.87; p=0.02; **Q4**: HR 0.34; 95%CI 0.13, 0.92; p=0.03).

In multivariate analysis, children in the highest quartile of neighborhood deprivation sustained a 67% lower normalization rate after adjusting for race, ethnicity, primary‐spoken language, and insurance type (**Q4**: HR 0.33; 95%CI 0.15, 0.75; p=0.008). tTG IgA ≥10x ULN sustained reduced normalization rates among children in the 2nd and 4th deprivation quartiles (**Q2**: HR 0.14; 95%CI 0.05, 0.42; p<0.001; **Q4**: HR 0.34; 95%CI 0.12, 0.97; p=0.04).

**Conclusion:** Children living in socioeconomically deprived neighborhoods, especially those with markedly elevated tTG IgA at diagnosis, may have inadequate dietary adherence. Addressing the barriers to gluten elimination in deprived neighborhoods may improve outcomes.


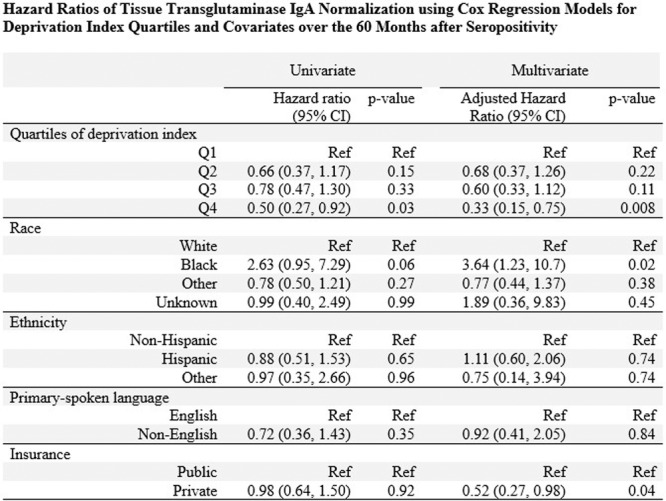


Cox regression models characterize the hazard ratio of tissue transglutaminase (tTG) IgA normalization among children over the 60 months after tTG IgA seropositivity based on deprivation index quartiles (Q), race, ethnicity, primary‐spoken language, and insurance type. Shown are 95% confidence intervals (95% CI).


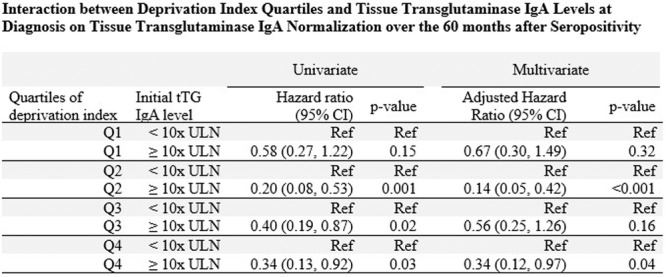


Cox regression models characterize the hazard ratio of tissue transglutaminase (tTG) IgA normalization among children with interaction between deprivation quartiles (Q) and tTG IgA levels at diagnosis (

## 180 EXAMINING THE FEASIBILITY OF DUODENAL PROTEIN SAMPLING THROUGH THE DUODENAL STRING TEST (DST)

*Patricia Dillawn* ^*1*^, *Edwin Liu* ^*1*^, *Nathalie Nguyen* ^*1*^, *Anthony Saviola* ^*2*^, *Alexandra Crook* ^*2*^, *Marisa Stahl* ^*1*^

^*1*^ *Pediatrics*, *University of Colorado Anschutz Medical Campus*, *Aurora*, *CO*; ^*2*^ *University of Colorado Anschutz Medical Campus School of Medicine*, *Aurora*, *CO*

**Background:** Pediatric celiac disease (CeD) diagnosis in North America relies on esophagogastroduodenoscopy (EGD) with duodenal biopsy, which requires children to undergo general anesthesia. The esophageal string test is a minimally invasive test that allows children to swallow a capsule from which an absorptive string is deployed and can sample luminal proteins in the esophagus with a dwell time of just one hour. This test is already employed in clinical care to measure eosinophilic proteins that reflect disease activity in patients with eosinophilic esophagitis (EoE) without undergoing repeat EGD. This study aimed to determine the feasibility of the duodenal string test (DST), utilizing similar technology to sample protein biomarkers in the duodenum.

**Methods:** In order to determine the feasibility of the DST to measure duodenal protein biomarkers, 2 participants swallowed the DST in this pilot study to explore optimal conditions. Pre‐study conditions were tested with the duodenal string to determine 1) the ability to actually measure protein 2) adequate “dwell time” following capsule swallowing, 3) cut string length for protein elution, and 4) ability to distinguish between gastric and duodenal string portions. Each participant underwent a 3‐hour “dwell time” after swallowing the capsule to allow for the capsule to pass into the duodenum and absorb luminal proteins. The esophageal, gastric, and duodenal portions of the string were separated based on pH change. Samples of varying length (6, 10, 15, and 30 cm) were collected from the duodenal portion. The string samples were frozen upon retrieval and eluted with 10% sodium dodecyl sulfate (SDS) for preparation for mass spectrometry. Protein concentration was determined with NanoDrop. Replicates of each test condition were performed. Peptide samples were analyzed by liquid‐chromatography tandem mass spectrometry using a Bruker nanoElute liquid chromatography system coupled to Bruker timsTOF SCP mass spectrometer (Bruker Daltonics) operating in data‐dependent positive ion mode. Mass spectra were interpreted against the UniProt human protein sequence database and the false discovery rate was 1%.

**Results:** With a 3‐hour dwell‐time, adequate quantity of proteins were obtained from a 6 cm portion of the string in the duodenum for mass spectrometry. There was a diverse pattern of proteins present in the duodenal versus the gastric portions of the string, allowing for discrimination (Figure 1‐Volcano Plot). Examples of proteins highly expressed in the duodenum compared to the stomach include *CLCA1*, *DEFA5*, and *LACTB2*.

**Conclusion:** This initial DST pilot testing reveals that a 3‐hour dwell time is sufficient to reach and sample duodenal luminal proteins. We are able to identify distinct protein profiles from the stomach and duodenum using mass spectrometry. In a continuation of our work, we will utilize the DST to compare the duodenal protein expression and cytokine profiles of patients with active CeD to patients without CeD. If this is successful, it would provide a less invasive test for CeD diagnosis and/or monitoring that could be applied similar in the clinical setting to EoE.


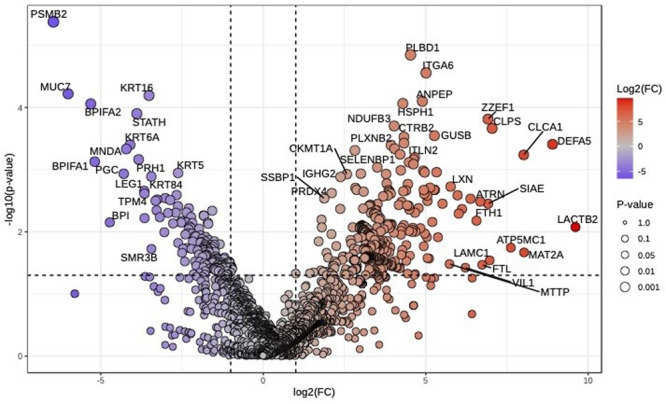


**Figure 1.** The proteins expressed in higher concentrations in the duodenum versus stomach are depicted in the upper right quadrant, and the proteins expressed in lower concentrations are depicted in the upper left quadrant. Statistical significance was set with a fold change threshold of 2.0 and a p <0.05.

## 186 EXCESSIVE POLY(ADP)RIBOSYLATION IN THE INFLAMED MUCOSA OF PEDIATRIC CELIAC DISEASE POINTS TO A NOVEL INFLAMMATORY MECHANISM AND THERAPEUTIC TARGET

*Tala Haddadin* ^*1,3*^, *Tarek Masannat* ^*3*^, *Belinda Sun* ^*2*^, *Fayez ghishan* ^*1,3*^, *Pawel Kiela* ^*3*^

^*1*^ *pediatric GI*, *Banner ‐ University Medical Center Tucson*, *Tucson*, *AZ*; ^*2*^ *Pathology*, *Banner ‐ University Medical Center Tucson*, *Tucson*, *AZ*; ^*3*^ *Pediatric*, *Daniel Cracchiolo Institute for Pediatric Autoimmune Disease Research, Steele Children's Research Center, University of Arizona Health Sciences*, *Tuscon*, *AZ*

**Background:** Celiac disease (CD) is a chronic immune‐mediated enteropathy triggered by gluten ingestion, primarily affecting genetically predisposed individuals carrying HLA‐DQ2/HLA‐DQ8 haplotypes. It is characterized by small intestinal inflammation, mucosal atrophy, and villous blunting, accompanied by gastrointestinal and extraintestinal symptoms. CD is marked by autoantibodies against tissue transglutaminase and deamidated gliadin peptides.

Multiple programmed cell death pathways, including apoptosis, necroptosis, pyroptosis, and ferroptosis, contribute to mucosal injury by promoting epithelial barrier loss and inflammation through cytokine release. However, Parthanatos—a distinct, caspase‐independent programmed cell death mediated by Poly (ADP‐ribose) polymerases‐1/2 (PARP‐1/2) and excessive poly(ADP)‐ribosylation (hyperPARylarion) —remain unexplored in CD.

Parthanatos arises from PARP‐1/2 hyperactivation following DNA damage, leading to depletion of NAD+ and ATP, accumulation of poly (ADP‐ribose) (PAR) polymers, and translocation of a heterodimer consisting of apoptosis‐inducing factor (AIF) and macrophage migration inhibitory factor (MIF) to the nucleus, causing chromatin condensation and large‐scale DNA fragmentation. This energy‐dependent process results in inflammatory cell death.

Given the established role of hyperPARylation in other inflammatory and autoimmune conditions, we hypothesized that cytotoxic stress in celiac disease (CD) may involve hyperPARylation and potentially Parthanatos, contributing to epithelial cell death and mucosal inflammation. To begin addressing this hypothesis, we employed immunofluorescence (IF) to assess PAR accumulation and CD3+ T cell infiltration in intestinal biopsies from pediatric CD patients compared to unaffected controls.

**Methods:** Formalin‐fixed paraffin‐embedded (FFPE) archived duodenal and bulb biopsies from pediatric patients with biopsy‐confirmed CD and non‐celiac controls were retrieved and sectioned at 5 µm. Sections were deparaffinized at 64°C, rehydrated, and antigen retrieval was performed using Tris‐EDTA buffer (pH 8.0).

Tissues were then permeabilized with 0.5% Triton X‐100 in PBS and blocked sequentially with AffiniPure F(ab) goat anti‐mouse antibody and 5% bovine serum albumin (BSA). Sections were incubated overnight at 4°C with primary antibodies against poly(ADP‐ribose) (PAR) and CD3 at 1:100 dilution. After PBS washes, secondary antibodies conjugated with Alexa Fluor (goat anti‐mouse 594, goat anti‐rabbit 488) were applied for 2 hours. Nuclear staining was done with DAPI, and slides were mounted with antifade medium.

Images were captured using the EVOS M7000 system under standardized conditions. Quantitative analysis of PAR and CD3 expression was conducted using ImageJ. Statistical differences were evaluated using unpaired Student's t‐test.

**Results:** Preliminary IF analysis (N=7) in each group showed a significant increase in both PAR (p<0.0001) and CD3 (p<0.03) expression in the duodenal and bulb mucosa of CD patients compared to controls. PAR signal intensity was increased within the epithelium and in subepithelial compartment, indicating enhanced PARP‐1/2 activation and PARylation associated with inflamed mucosa. CD3+ T cell infiltration was markedly elevated in the regions of marked hyperPARylation. These findings suggest active involvement of hyperPARylarion and T cell–mediated immune responses in CD pathology.

**Discussion:** Our data indicate that hyperPARylation and increased CD3+ T cell infiltration are prominent in the inflamed mucosa of pediatric CD. Elevated PAR abundance suggests that Parthanatos may contribute to epithelial damage and immune amplification in CD, although this requires further confirmation by AIF/MIF immunostaining. These findings position the PARP‐1/2 hyperactivation as a novel mechanistic link between immune activation and mucosal injury in CD. Given PARP inhibitors’ success in reducing immune responses in autoimmune models, targeting this pathway might offer therapeutic benefit in refractory or persistent CD despite gluten‐free diet adherence.

**Conclusion:** This study provides the first evidence of increased PARylation in the inflamed mucosa in pediatric CD, suggesting that hyperPARylation and T cell activation/homing may synergistically contribute to the CD pathogenesis by promoting epithelial cell death, barrier failure, and immune dysregulation. Further research is needed to validate and clarify the role of Parthanatos in CD and to evaluate PARP‐1/2 inhibition as a potential cytoprotective therapy.

## 187 CELIAC DISEASE IN CHILDREN WITH EQUIVOCAL HLA TYPES: CASE SERIES

*Lilas Alhalhooly*, *Imad Absah*

*Pediatric*, *Mayo Clinic Minnesota*, *Rochester*, *MN*

**Introduction:** Celiac disease (CeD) is a common autoimmune disorder affecting up to 1% of the North American population. HLA testing is increasingly used to identify permissive genotypes for CeD, particularly in high‐risk groups, patients already on a gluten‐free diet, and those with strong clinical suspicion despite negative serology or histology. However, little is known about the risk of CeD progression in children with equivocal HLA genotypes.

**Methods:** We conducted a retrospective review of electronic medical records at the Mayo Clinic between 2018 and 2025. Children (<18 years) with equivocal HLA results—defined as carrying only one allele of the permissive HLA genes—were included. We recorded clinical presentations, celiac serology, and histologic findings. CeD diagnosis was based on established serologic or histologic criteria. Histologic changes were classified using the Marsh system, and when discrepancies arose between the duodenal bulb and distal duodenum, the higher Marsh score was recorded. The study was approved by the Mayo Clinic Institutional Review Board.

**Results:** We identified 16 children who underwent CeD evaluation with equivocal HLA results. The mean age at CeD workup was 10.6 ± 5.6 years; 88% were non‐Hispanic White, and 38% were female. All presented with multiple symptoms, most commonly abdominal pain, diarrhea, and weight loss. All patients had positive celiac serology: 14 with tissue transglutaminase IgA (TTG IgA) and 2 with TTG IgG in the context of IgA deficiency. The average TTG IgA level was 53 U/mL (normal <4), with 31% (5/16) exceeding 10 times the upper limit of normal. Upper endoscopy was performed in 75% (12/16), revealing Marsh 3 lesions in 5, Marsh 1 in 4, and normal histology in 3 patients. Overall, 6/16 (38%) met diagnostic criteria for CeD (5 with Marsh 3 and 1 with TTG IgA >10× ULN plus positive endomysial antibodies), 4/16 (25%) were considered potential CeD (positive serology with Marsh 1 or normal histology), and 4/16 (25%) did not meet CeD criteria (normal histology). Follow‐up data were available for 14 patients (mean follow‐up 17 months). Eight patients (50%) were treated with a gluten‐free diet (6 CeD and 2 potential CeD), all of whom reported symptom resolution and serology normalization.

**Conclusion:** Although rare, children with equivocal HLA genotypes can progress to active CeD. These findings suggest that definitions of permissive HLA genotypes may need to be reconsidered to ensure that at‐risk patients are appropriately identified and monitored.


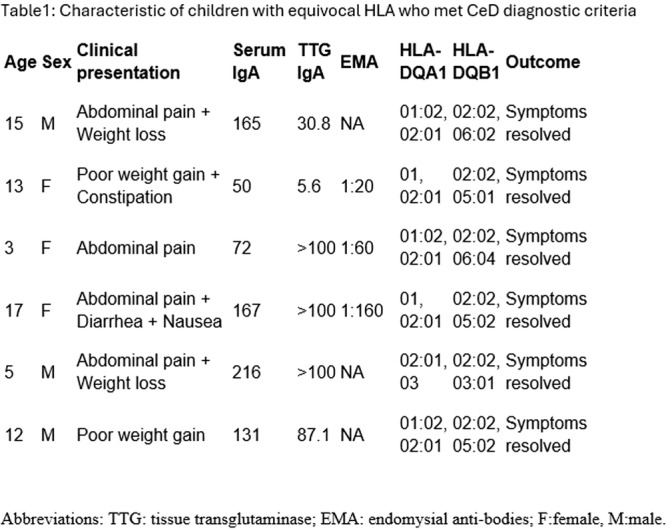


## 188 CULINARY MEDICINE AS A TOOL TO ENHANCE PROVIDER CONFIDENCE IN MANAGING DUAL DIAGNOSIS OF CELIAC DISEASE AND TYPE 1 DIABETES

*Vanessa Weisbrod* ^*1*^, *Meghan Donnelly* ^*1*^, *Jackie Jossen* ^*4*^, *Sharon Weston* ^*7*^, *Mary Shull* ^*3*^, *Jessica Lebovits* ^*4*^, *Marilyn Geller* ^*1*^, *Maria Ines Pinto‐Sanchez* ^*5*^, *Raquela Adelsburg* ^*6*^, *Ritu Verma* ^*2*^

^*1*^ *Education*, *Celiac Disease Foundation*, *Needham*, *MA*; ^*2*^ *Gastroenterology*, *The University of Chicago*, *Chicago*, *IL*; ^*3*^ *Gastroenterology*, *Children's Hospital Colorado*, *Aurora*, *CO*; ^*4*^ *Gastroenterology*, *Columbia University*, *New York*, *NY*; ^*5*^ *Gastroenterology*, *McMaster University*, *Hamilton*, *ON*, *Canada*; ^*6*^ *Mount Sinai Health System*, *New York*, *NY*; ^*7*^ *Nutrition*, *Boston Children's Hospital*, *Boston*, *MA*

**Introduction:** Culinary medicine is an evidence‐based field that blends medical, nutrition, and culinary knowledge to support patients in maintaining health and treating chronic disease. A dual diagnosis of celiac disease (CeD) and Type 1 Diabetes (T1D) requires significant dietary intervention to effectively manage both conditions. We aimed to determine whether participation in a culinary medicine program can improve confidence of physicians and dietitians in counseling patients with a dual diagnosis of CeD and T1D, as well as knowledge of appropriate dietary interventions.

**Methods:** We developed a pilot culinary medicine program, which was presented as a live webinar and accredited for CEUs for physicians and registered dietitians. The webinar was instructed by a gastroenterologist, pediatric and adult dietitians, and a medical chef. After obtaining consent, participants completed a pre‐and‐post survey to determine changes in confidence and strategies related to counseling patients with a dual diagnosis of CeD and T1D. Provider confidence levels before and after the webinar were compared using a chi‐squared test.

**Results:** A total of 29 health providers claimed CEUs for this webinar. Of those who provided consent to use their information in research, physicians (n=2) and dietitians (n=27) reported their primary reasons for attending was to improve their understanding of the management of co‐occurring CeD and T1D (80%) and to enhance their ability to educate these patients effectively (77.5%). The most common diet education strategies used among this sample were balanced meals and snacks (72.5%) and low glycemic foods (42.5%), and following the webinar, 62% indicated that they would modify their approach to educating patients with a dual diagnosis of CeD and T1D. Provider confidence significantly increased after the program compared to baseline (p<0.001).

**Discussion:** Integrating a disease‐specific culinary medicine program into continuing education programs can significantly improve provider confidence in counseling patients requiring complex dietary interventions, such as co‐occurring CeD and T1D. By combining practical cooking strategies with medical and nutritional education, this approach equips providers with actionable tools to support dietary adherence and optimize patient outcomes for both conditions.

## 189 LET'S BE CLEAR (CLARIFYING EXPECTATIONS TO ADVANCE RELATIONSHIPS): SUPPORTING GASTROENTEROLOGY INPATIENT STAFF, PATIENTS, AND FAMILIES IN MANAGING DISTRESS AND MITIGATING CONFLICT

*Lisa Fahey* ^*1,2*^, *JoAnn Duffy* ^*3*^, *Pam Nathanson* ^*3*^, *Lauren Boldizar* ^*3*^, *Miriam Stewart* ^*3,2*^

^*1*^ *Pediatrics; Division of Gastroenterology*, *The Children's Hospital of Philadelphia*, *Philadelphia*, *PA*; ^*2*^ *Perelman School of Medicine*, *University of Pennsylvania*, *Philadelphia*, *PA*; ^*3*^ *The Children's Hospital of Philadelphia*, *Philadelphia*, *PA*

**Objective:** Reports of employee stress and safety concerns resulting from challenging interactions with patients and families have increased across the Children's Hospital of Philadelphia (CHOP), including on the inpatient gastroenterology unit. These conflicts make it harder to deliver exceptional medical care. They can also negatively impact organizational culture, which can lead to employee burnout and turnover.

The objective was to design tools and strategies to help employees clarify expectations and improve communication with inpatient patients & families to help manage patient/family distress and engage in ways that can prevent and reduce conflict.

**Methods:** We developed a graded framework that provides guidance and resources for various team/family interactions, ranging from a Green Zone of no conflict, to a Purple Zone of intense conflict where potential legal action or patient relationship termination is considered, with several zones in between. See attached image of the CLEAR Framework Overview, as well as the Specific Strategies by Zone.

This framework enabled teams to engage with families proactively both by setting clear expectations at admission, and by addressing potential conflicts as early as possible. Expectations were communicated to families via an admission welcome letter. Standardized processes were implemented to respond to escalating conflict, with increased involvement from clinical leaders and increased frequency and structure of patient and family communication as conflict escalated.

**Results:** The framework was piloted during 1,607 admissions on 3 inpatient units including the gastroenterology unit over 8 months. Family complaints decreased on all units. All units showed improvements in the percentage of families with no conflict (the green zone) after pilot launch (from 91% to 95% on the GI unit, 86% to 100% on the neurology unit, and 96% to 100% on the complex care unit). % of GI patient families with ongoing conflict (the orange zone) decreased from 9% to 0%, % of neurology patient families with minimal conflict (the yellow zone) decreased from 14% to 0%, and % of complex care patient families with minimal conflict (the yellow zone) decreased from 4% to 0% during the pilot. There were no red zone (escalated conflict) or purple zone (intense conflict) conflicts during the pilot. In addition, the GI and neurology units had improvements in the ‘response to concerns and complaints’ question on the patient/family survey.

**Conclusion:** Challenging interactions with patients and families can be mitigated and sometimes even prevented all together by utilizing this innovative CLEAR framework to clarify expectations and support all parties, including patients, families and medical staff. Creating a standard approach to prevent conflict when possible and otherwise reduce its frequency and intensity propagates a safe, wellness‐centered organizational culture in which outstanding medical care can be safely delivered.


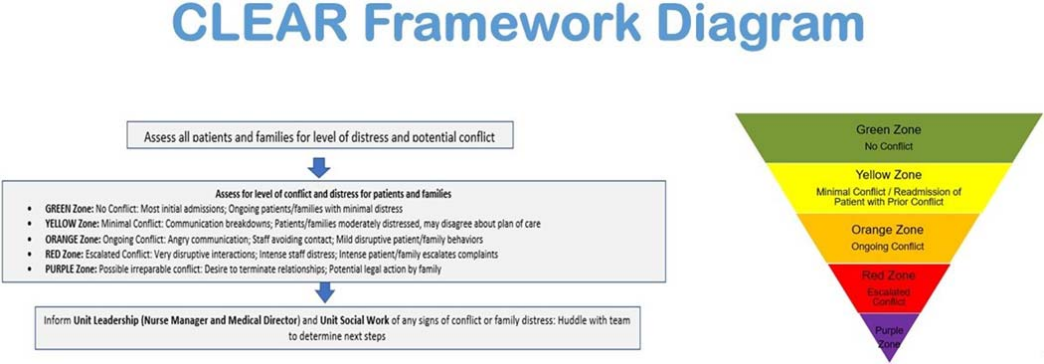


CLEAR Framework Diagram


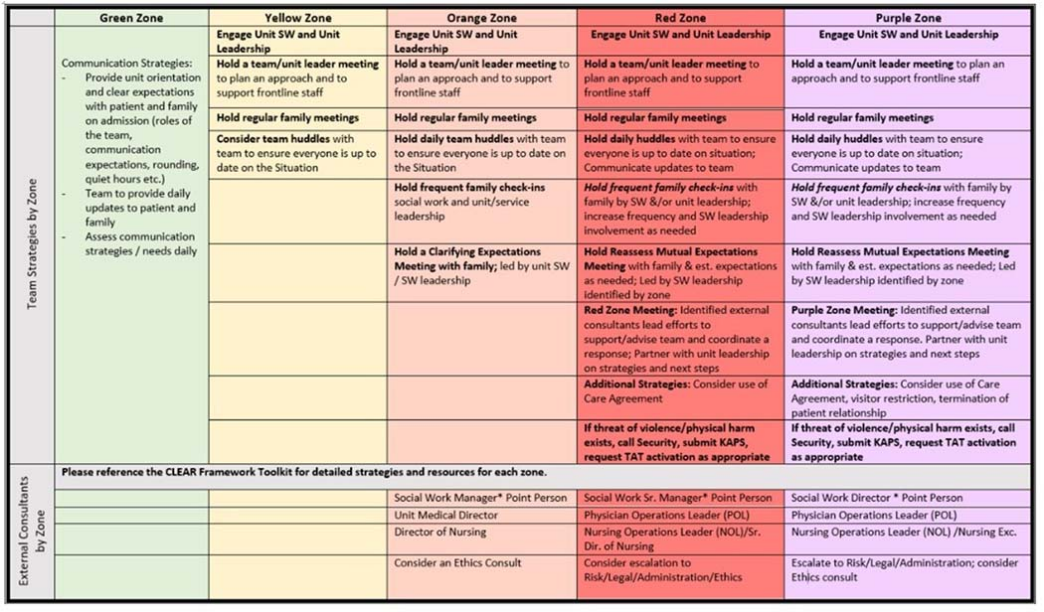


Mitigating Conflict ‐ Team Strategies by Zone

## 191 UNDERSTANDING PEDIATRIC FACULTY ENDOSCOPISTS' PERCEPTIONS AND ADOPTION OF STANDARDIZED COMMUNICATION TO TEACH ENDOSCOPY: AN INTERVIEW‐BASED STUDY

*Aayush Gabrani* ^*1*^, *Alec Mason* ^*2*^, *Catharine Walsh* ^*4,5*^, *Kinga Eliasz* ^*3*^

^*1*^ *Pediatrics*, *University of Minnesota Twin Cities*, *Minneapolis*, *MN*; ^*2*^ *Internal Medicine*, *The University of Texas Southwestern Medical Center*, *Dallas*, *TX*; ^*3*^ *Medicine*, *New York University Grossman School of Medicine*, *New York*, *NY*; ^*4*^ *The Hospital for Sick Children Department of Paediatrics*, *Toronto*, *ON*, *Canada*; ^*5*^ *Pediatrics*, *University of Toronto*, *Toronto*, *ON*, *Canada*

**Background:** Variability in instructional approaches and feedback during endoscopy training contributes to inconsistent learning experiences and may hinder the development of competence in pediatric colonoscopy. This study explored pediatric gastroenterologists’ perceptions of current endoscopy teaching practices and the use of standardized communication to structure instruction.

**Methods:** This was a prospective qualitative study using the constructivist grounded theory approach, using cognitive load theory and diffusion of innovation as sensitizing concepts and for designing interview guide. Pediatric gastroenterology faculty from an academic center participated in one‐on‐one semi‐structured interviews (60‐90 minute long) The first part of the interview focused on current practices of endoscopy teaching, and the second on endoscopists’ understanding of standardized communication to teach endoscopy and its adoption. Standardized communication refers to uniform teaching strategy used by an endoscopist trainer to give instructions to a trainee during an endoscopic procedure (Table 1). Interviews were transcribed and de‐identified, and inductive, multi‐staged and iterative coding was done by two independent coders. Quality and rigor were ensured by multiple coders, member‐checking and memo‐writing.

**Results:** Fifteen interviews were conducted (till saturation was achieved) and three key themes were generated. First theme showed that faculty endoscopists’ teaching styles were a result of complex interaction between constant factors (foundation factors influencing their approach in every endoscopy) and dynamic factors (with variable influence across procedures) [Figure 1]. The second theme described a spectrum of faculty's perceptions of standardized communication ranging from support to cautious optimism and hesitation. Although faculty supported standardized communication in theory (support), it was not incorporated in their daily practice (hesitation). The third theme identified factors influencing its adoption – trainees, training‐the‐trainer and time (3Ts). Trainee support and faculty training (training‐the‐trainers) emerged as powerful drivers of adoption of standardized communication. Time had multifaceted role ‐ faculty needing *time* to adopt, variable *time* to adopt these components (early vs late adopters) and *time* linked to facutly seniority (experienced faculty being more resistant to change).

**Conclusion:** This study uncovers how faculty developed their endoscopy teaching approach and highlights a theory‐practice mismatch of endoscopists’ perception regarding standardized communication. The identified facilitators (learner‐centered motivation, structured faculty development, and adequate time for integration) highlight important strategies for promoting adoption of not only standardized communication but also other educational innovations. Future research targeting the 3Ts will be important for bringing long‐lasting change to enhance the quality of endoscopy education.


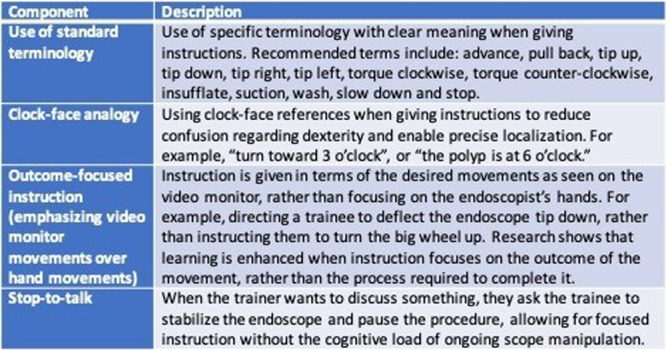


**Table 1:** Components of standardized communication for colonoscopy instructions


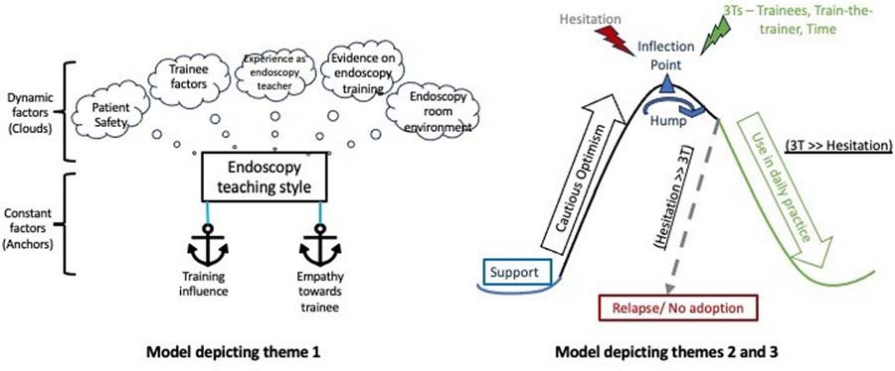


**Figure 1:** Model depicting theme 1 (left) ‐ Shifting dynamic factors (clouds) interact with constant factors (anchors) to influence real‐time instructional decisions.

Model depicting theme 2 and 3 (right) ‐ Adoption process for standardized communication in endoscopy teaching: Adoption is depicted as a hill ‐ theoretical support forms the base, with cautious optimism along the uphill. The inflection point—or 'hump'—represents the challenge of translating theory into practice. The 3 T's (trainees, training, and time) help faculty overcome this point, while hesitation can act as a barrier. If support from the 3 T's outweighs hesitation, adoption occurs; if not, relapse to previous teaching methods is likely.

## 192 EXPANDING EXPERTISE: IMPACT OF PEDIATRIC LEARN INTESTINAL FAILURE TELE‐ECHO ON PROVIDER EDUCATION AND PATIENT CARE

*Joanne Lai* ^*4*^, *Smita Ghosh* ^*1*^, *Marjorie Nisenholtz* ^*2*^, *Tessa McEniry* ^*2*^, *Maryanna Tosi* ^*3*^, *Kishore Iyer* ^*2*^

^*1*^ *Project LIFT‐ECHO*, *New York*, *NY*; ^*2*^ *Recanati/Miller Transplantation Institute at Mount Sinai*, *New York*, *NY*; ^*3*^ *Hackensack Meridian Hackensack University Medical Center*, *Hackensack*, *NJ*; ^*4*^ *Icahn School of Medicine at Mount Sinai*, *New York*, *NY*

**Background:** Pediatric Learn Intestinal Failure Tele‐ECHO (P‐LIFT‐ECHO) aims to improve the care of children with pediatric intestinal failure (PIF) by connecting providers with limited access to a multi‐disciplinary team of intestinal rehabilitation experts using the established ECHO™ tele‐mentoring/learning model.

**Methods:** We conducted a mixed method evaluation of 44 sessions that have been attended by 850 participants from July 2020 to July 2024. Modified appreciative inquiry (AI) guided focus group discussions using the SCORE (Strengths, Challenges, Opportunities/Aspirations, and Results) framework was conducted in September 2024. AI results informed a survey that was sent in December 2024 to all participants that attended ≥3 P‐LIFT‐ECHO sessions. Transcripts of AI were analyzed and triangulated with survey results to gain in‐depth understanding.

**Results:** Perceptions of the 19 AI participants highlighted the themes of opportunities to improve (39%) and challenges (25%). Survey responses were received from 67/206 (32.5%) and demonstrated global participation with 28% joining from 12 non‐US countries; US participation spanned 22 states [Figures 1 A, 1B]. 28/67 (42%) participants do not consider themselves PIF experts and 23/67 (34%) care for ≤10 PIF patients annually. Despite challenges to attending P‐LIFT‐ECHO, primarily due to conflicting patient schedules, 45% have attended ≥10 P‐LIFT‐ECHO sessions and 100% felt it benefitted professional growth, including learning from each other. Over half (59%) reported changing patient care and 90% reported new learning [Figure 1 C].

**Conclusions** Evaluation of P‐LIFT‐ECHO demonstrated value of continuing monthly as it connects PIF providers internationally, providing an effective platform for learning and impacting patient care.


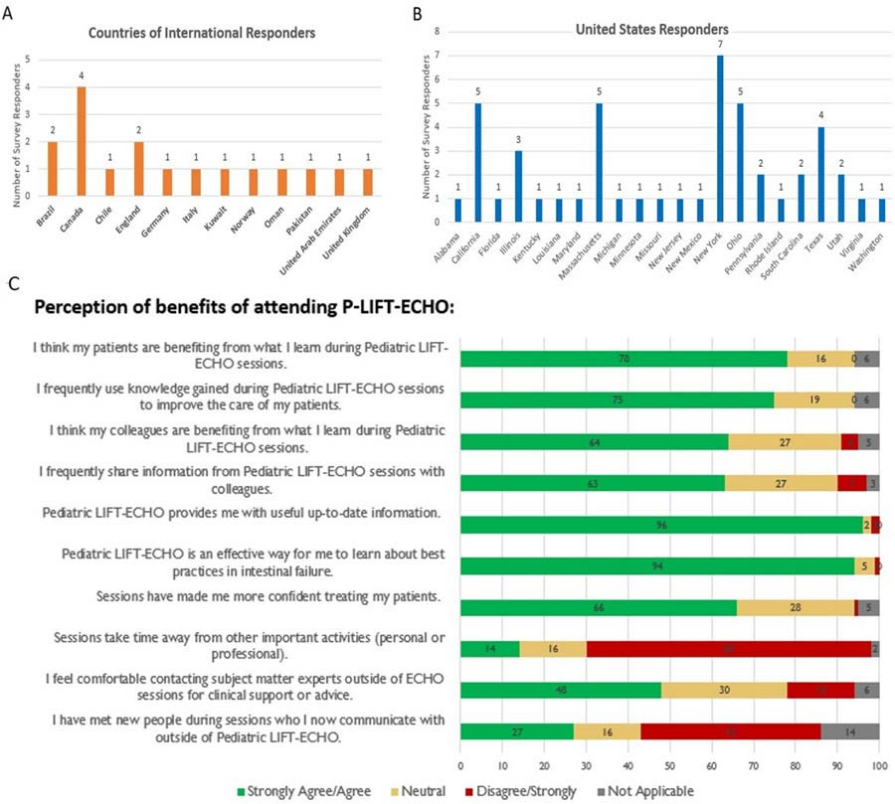


## 196 ASSOCIATIONS BETWEEN GUT MICROBIOTA AND NEONATAL JAUNDICE IN EXCLUSIVELY FORMULA‐FED INFANTS

*Kwang Kim* ^*2*^, *Kimin Kang* ^*1*^, *Sun Hyang Lee* ^*2*^, *Ga Young Park* ^*2*^, *Jieun Hwang* ^*2*^, *Yoo Min Lee* ^*2*^, *Sung Shin Kim* ^*2*^, *Jin Soo Moon* ^*3*^, *Donghyun Kim* ^*1*^, *Jae Sung Ko* ^*3*^

^*1*^ *Department of Biomedical Sciences*, *Seoul National University College of Medicine*, *Jongno‐gu*, *Seoul*, *Korea (the Republic of)*; ^*2*^ *Department of Pediatrics*, *Soonchunhyang University Hospital Bucheon*, *Bucheon‐si*, *Gyeonggi‐do*, *Korea (the Republic of)*; ^*3*^ *Department of Pediatrics*, *Seoul National University College of Medicine*, *Jongno‐gu*, *Seoul*, *Korea (the Republic of)*

**Background:** Neonatal jaundice (NJ) has been suggested to correlate with gut microbiota. Although it has recently been shown that an imbalance in the gut microbiome significantly affects the development of NJ, there is still controversy among studies about which microorganisms affect NJ. Therefore, we aimed to characterize and compare the gut microbiota of neonates with and without jaundice under controlled conditions.

**Methods:** From August 2024 to May 2025, we prospectively enrolled neonates admitted to the Neonatal Intensive Care Unit at Soonchunhyang University Bucheon Hospital. All enrolled infants were born via cesarean section with a gestational age between 30 and 40 weeks. Gestational age from 30 to less than 37 weeks was classified as preterm, and 37 to 40 weeks as term. To minimize maternal confounders, only neonates born to mothers without peripartum antibiotic exposure (within 4 weeks prior to delivery) and without maternal comorbidities were included. Only neonates who were exclusively formula‐fed from birth were included to eliminate the potential confounding effect of breast milk jaundice. Infants who received any postnatal antibiotics were excluded. Neonates with total bilirubin (TB) ≥ 15 mg/dL were classified as NJ, and those with TB <15 mg/dL as healthy control (HC). Neonates with hemolytic conditions (e.g., ABO or Rh incompatibility, hemolytic anemia), cephalohematoma, brain hemorrhage, or congenital hypothyroidism were excluded from the study. Alpha diversity was assessed using the Shannon index, Simpson index, and observed features. Beta diversity was evaluated using PERMANOVA to determine differences in microbial composition. To identify key taxa distinguishing between groups, Random Forest analysis was performed, and differential abundance was assessed using ANCOM‐BC.

**Results:** A total of 25 neonates were enrolled, including 7 with NJ and 18 HC. The median TB was 17.5 mg/dL (range: 15‐ 22.7) and 8.5 mg/dL (range: 7–9.9) for the NJ and HC, respectively. Among the NJ group, 3 were term neonatal jaundice (TNJ) and 4 were preterm neonatal jaundice (PNJ). The HC group consisted of 10 term healthy controls (THC) and 8 preterm healthy controls (PHC). Alpha diversity did not differ significantly between the NJ and HC or between the TNJ and THC (all *p* > 0.05). However, in the comparison between PNJ and PHC, observed features were significantly lower in the PNJ group (*p* = 0.012), while other alpha diversity metrics showed no significant differences (all *p* > 0.05). Beta diversity analysis using PERMANOVA revealed statistically significant differences across all comparisons between NJ and HC, TNJ and THC, and PNJ and PHC (*p* = 0.003, *p* = 0.034, and *p* = 0.023, respectively). Random Forest and ANCOM‐BC analyses identified family *Lactobacillaceae* as the key discriminatory taxon between NJ and HC, with significantly higher abundance in the HC group. In the comparison of TNJ and THC, family *Lactobacillaceae* and genus *Enterococcus* were significantly more abundant in THC. Between PNJ and PHC, family *Lactobacillaceae*, genus *Lactococcus*, and class *Actinobacteria* were all significantly enriched in PHC.

**Conclusions:** In this well‐controlled cohort of exclusively formula‐fed neonates, jaundice was associated with distinct differences in gut microbiota. The NJ group showed a significantly lower abundance of the family *Lactobacillaceae* compared to the HC group. Further studies incorporating functional metagenomics and metabolomics are warranted to clarify the role of gut microbiota in NJ.


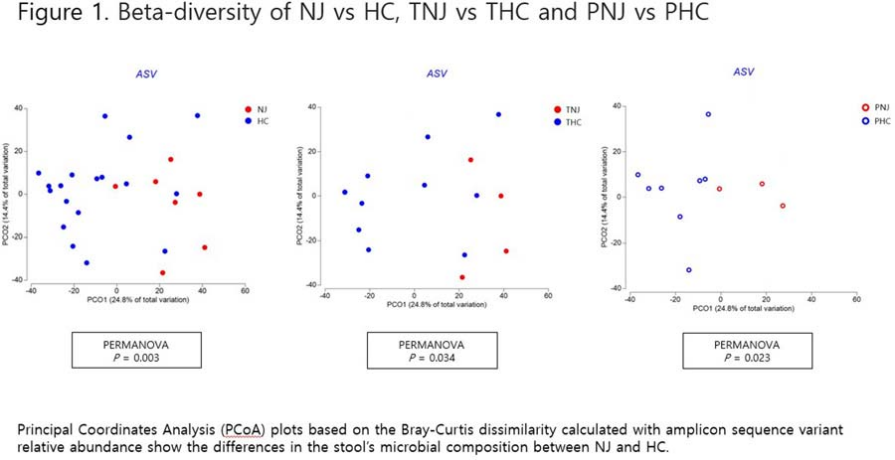



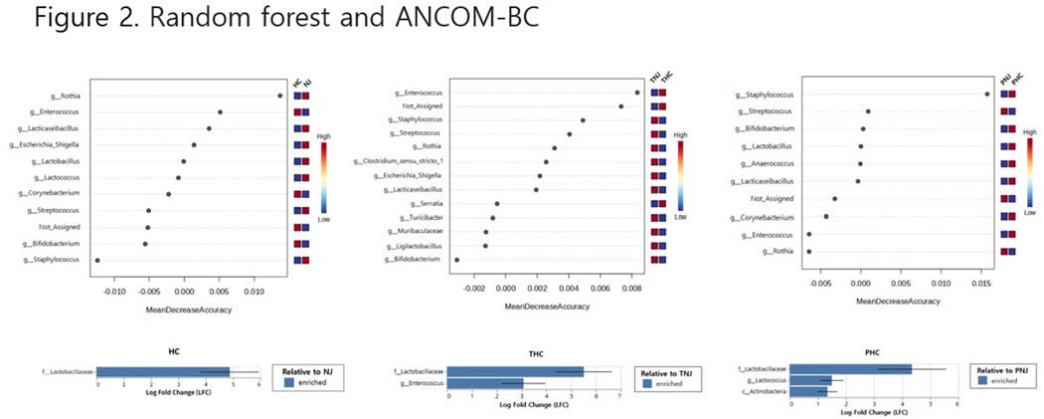


## 197 DECIPHERING THE STRUCTURE OF BACTERIAL STRAIN COMPETITION AND COEXISTENCE WITHIN COMPLEX MICROBIAL COMMUNITIES

*Eduardo Contijoch* ^*1*^, *Michael Fischbach* ^*2*^

^*1*^ *Pediatrics ‐ Gastroenterology*, *Stanford Medicine*, *Stanford*, *CA*; ^*2*^ *Bioengineering*, *Stanford University*, *Stanford*, *CA*

**Background:** The gut microbiome has emerged as an area of intense interest for its potential to deepen our understanding of the pathophysiology of and develop novel treatments for a wide range of human diseases, including metabolic disease, cancer, and inflammatory bowel disease. Notably, the microbiome has been shown to be a “druggable target”, as seen in the case of recurrent *Clostridium difficile* infection (rCDI), where fecal microbiota transplantation (FMT) has achieved greater than 90% efficacy in cases refractory to antibiotic treatment. However, efforts to employ FMT in other diseases such as inflammatory bowel disease and metabolic syndrome have been significantly less effective and have raised concerns regarding the safety of traditional approaches to FMT. The next generation of microbiome‐based therapies seeks to employ synthetic communities—collections of individually isolated and cultivated bacterial strains—as the donor material which improves the ability to track and characterize the fate of newly introduced strains and provides a heightened safety profile by allowing stricter control over what is introduced to patients. While the selection of representative strains of a species to include within a synthetic community can seem inconsequential or arbitrary, animal studies have shown that within‐species (i.e. strain‐to‐strain) phenotypic variability can have implications for the community composition, metabolic potential, and host‐physiologic consequences of a microbiome. In this work, we sought to understand the ecologic fitness and consequences of strain selection within the context of a full‐scale gut microbiome.

**Approach:** Synthetic microbiome communities are constructed by combining individual bacterial strains isolated from the stool of healthy human controls. Each strain in these communities has undergone whole genome sequencing to confirm its species identity and to create a genomic reference database for subsequent metagenomic data analysis. In this case, our core synthetic community represents a collection of over 400 distinct strains representing nearly 200 unique species of human gut commensals. Germ‐free Swiss Webster mice are colonized with the synthetic community and fecal pellets are collected for microbiome analysis. Animals are subjected to dietary and pharmacologic interventions (sequential dietary changes and antibiotic exposure) to assess whether these affect the niche occupancy and ecological fitness of the colonized strains as measured by their ability to persist within the gut microbiome, as well as their ability to resist displacement by other strains when challenged with a repeated colonization. Strain engraftment and abundance is measured over time by shotgun metagenomic sequencing of DNA extracted from mouse fecal pellets. By leveraging the reference database of strain genomes, we can accurately quantify the presence of low‐abundance and closely‐related strains. At the time of this submission, data collection is ongoing and preliminary results will be available for discussion at NASPGHAN.

Our experimental approach seeks to address how within‐species strain engraftment is determined in cases where there are more strains introduced to a community than the size of the niche for a species – (e.g., if there is only “room” for one *E. coli* strain in the gut microbiome and several *E. coli* strains are introduced, what rules govern which strain ultimately engrafts). We aim to determine whether, in the context of a full‐scale gut microbiome, there are within‐species strain fitness differences that lead to the preferential colonization and persistence of individual strains over other strains within that species, or if the selection is random. We will then also address whether these patterns can be influenced or overcome by external factors such as nutrient availability or relative susceptibility to antibiotic exposure.


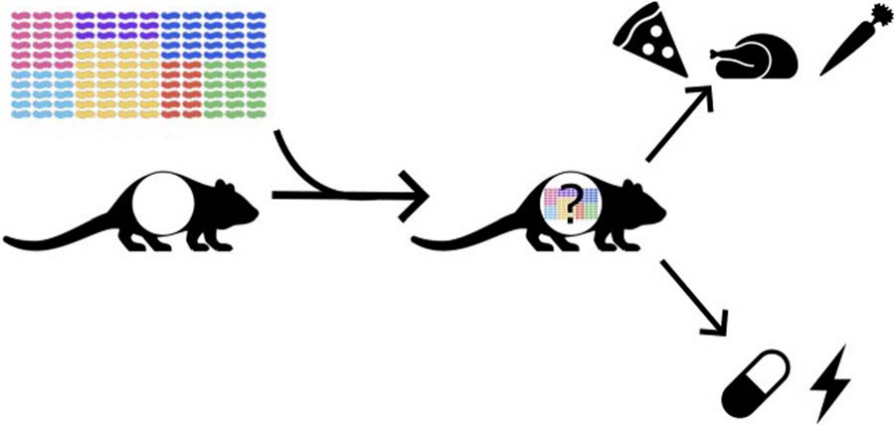


**Figure 1 ‐ Experimental Approach:** Germ free mice are colonized with a fully‐defined, full‐scale microbial community containing within‐species strain diversity to identify features that determine strain fitness, including the ability to tolerate changes to host diet and antibiotic exposure.

## 198 HELICOBACTER PYLORI INFECTION AND IMMUNE‐MEDIATED DIGESTIVE DISEASES IN CHILDREN OF THE AMERICAS: A CROSS‐SECTIONAL STUDY OF COMPARATIVE PREVALENCE

*Silvana Bonilla* ^*1,8*^, *JOSE VERA* ^*3*^, *AILIM CARIAS* ^*3*^, *Carlos Cuadros* ^*2*^, *Michelle Higuera* ^*4*^, *JUAN PABLO RIVEROS LOPEZ* ^*3*^, *Natali Gonzalez Rozo* ^*9*^, *Gloria Toscano* ^*7*^, *Ana Beatriz Munoz* ^*7*^, *Idalmis Aguilera* ^*6*^, *Enju Liu* ^*1*^, *Paul Harris* ^*5*^

^*1*^ *Pediatrics*, *Boston Children's Hospital*, *Boston*, *MA*; ^*2*^ *Hospital Internacional de Colombia*, *Bucaramanga*, *Colombia*; ^*3*^ *Hospital Universitario de la Fundacion Santa Fe de Bogota*, *Bogotá*, *Colombia*; ^*4*^ *Universidad Nacional de Colombia*, *Bogotá*, *Colombia*; ^*5*^ *Pontificia Universidad Catolica de Chile*, *Santiago*, *Santiago Metropolitan Region*, *Chile*; ^*6*^ *Instituto de Gastroenterologia*, *Havana*, *Havana*, *Cuba*; ^*7*^ *Universidad Peruana Cayetano Heredia*, *Lima District*, *Lima Region*, *Peru*; ^*8*^ *Harvard Medical School*, *Boston*, *MA*; ^*9*^ *Hospital Universitario Erasmo Meoz Cucuta*, *Cúcuta*, *North Santander*, *Colombia*

**Background:** *Helicobacter pylori* infection (HPI) remains common across the Americas. While eradication in adults helps prevent complications, the evidence in children is unclear. The 2023 joint NASPGHAN/ESPGHAN guidelines conditionally recommend against routine biopsies for HPI when evaluating other conditions such as inflammatory bowel disease (IBD), celiac disease (CeD), or eosinophilic esophagitis (EoE), citing limited, low‐quality evidence suggesting a potential protective role of HPI in some immune‐mediated digestive diseases (IMDD). We conducted a study to assess the prevalence of HPI in children with newly diagnosed IMDD.

**Methods:** Multicenter prospective study of children ≤18 years with newly diagnosed IBD, CeD, or EoE (biopsy‐confirmed), with or without HPI (defined by the presence of HPI in gastric biopsies), between July 2024 and April 2025. Demographic, social, anthropometric, and perinatal variables were collected using a study questionnaire that included items adapted from the ISAAC Phase III survey. We compared children with and without HPI and assessed HPI prevalence in IMDD versus HPI prevalence in a comparison group of children referred for upper endoscopy due to nonspecific digestive symptoms without any diagnosis of organic or structural disease.

**Results:** A total of 365 patients were included from five countries: USA (86.6%), Colombia (7.4%), Chile (3.8%), Peru (1.1%), and Cuba (1.1%). The mean age was 11.6 ± 4.8 years, and 53.4% were male. Diagnoses included EoE (106), CeD (184), and IBD (84), with IBD subtypes as follows: ulcerative colitis (48, 57.1%), Crohn's disease (34, 40.5%), and indeterminate colitis (2, 2.4%). Overall, 31/365 (9.3%) tested positive for HPI, with significant geographic variation: Colombia accounted for over half (16/31, 51.6%) of HPI‐positive cases, followed by the USA (9/31, 29%), Cuba (4/31, 12.9%), and Chile (2/31, 6.5%) (p < 0.0001). EoE was significantly more common in the HPI‐positive group (48.4% vs. 27.2%, p = 0.0131), while CeD was more common in the HPI‐negative group (52.1% vs. 32.3%, p = 0.0346). There was no significant difference in overall IBD prevalence (p = 0.6129). Significant differences between HPI‐positive and ‐negative groups were also observed in environmental and allergic factors. HPI prevalence was consistently lower in children with IMMD compared to those with non‐specific digestive symptoms, but none of the differences reached statistical significance (p > 0.05).

**Conclusion:** There is marked geographic variation in HPI among children with new‐onset IMDD. Children with HPI were significantly more likely to have EoE and less likely to have CeD, with no significant difference in overall IBD prevalence. Compared to children with nonspecific digestive symptoms, HPI prevalence was lower in children with new‐onset IMDD, particularly in Latin America, though the difference was not statistically significant.

## 199 LPS INDUCES TLR4 AND CYTOKINES IN A 3D PORCINE ENTEROID SYSTEM TO DRIVE MULTISYSTEM INJURY

*Chandrashekhara Manithody*, *Ashlesha Bagwe*, *Shaurya Mehta*, *Kento Kurashima*, *Marzena Swiderska‐Syn*, *Sree Kolli*, *Uthayashanker Ezekiel*, *Ajay Jain*

*Pediatrics*, *Saint Louis University School of Medicine*, *Saint Louis*, *MO*

**Background:** Intestinal epithelium plays an important role in maintaining gut homeostasis, acting as both a physical barrier as well as participating in immune responses to microbial stimuli. This is disrupted when using Total Parenteral Nutrition (TPN) and bypassing enteral nutrition. Lipopolysaccharide (LPS), a major component of Gram‐negative bacterial membranes, is a key mediator of pro‐inflammatory signaling. We hypothesized that stem cell‐derived intestinal enteroids would offer a valuable system to study epithelial functionality and used a porcine 3D enteroid model to interrogate LPS modulation of intestinal inflammation.

**Methods:** We developed a porcine protocol for matrigel‐based 3D culture systems to generate enteroids from the small bowel of neonatal yorkshire pigs. After 7 days, cultures were passaged and expanded. To investigate the effects of lipopolysaccharide (LPS), escalating concentrations of LPS µg/mL (1, 2, 5) were prepared by diluting a stock solution in organoid media. Serum cytokines were also measured in animals on enteral nutrition (EN control) or TPN as historical references.

**Results:** Zonula Occludens‐1 (ZO1), a well‐known marker for intestinal barrier integrity and gut permeability, was significantly decreased (3.6X) at the 24‐hour mark in enteroids exposed to 5 µg/mL LPS, with no significant changes with lower concentrations (2 µg/mL). LPS drove a significant upregulation of TLR4 gene expression in both the 2 µg and the 5 µg dose groups. TLR4 upregulation reached peak levels at 2 µg/ml LPS and paradoxically were highest in this group. To further evaluate host immune response, we evaluated Interleukin 10 and 8 (IL‐10 and IL‐8). IL‐10 and IL‐8 was increased in both the 2 µg and 5 µg LPS groups. We next evaluated serum cytokine profiles among the groups. The inflammatory cytokine interferon‐γ (IFN‐γ) was elevated in TPN vs EN, p=0.009. IL‐8 and LPS was higher in TPN vs EN (p=0.011, p<0.0001). Serum IL‐10 did not show any group differences.

**Conclusions:** LPS‐treated enteroids demonstrate the ability of our system to replicate epithelial responses. These are exemplified via enhanced innate immune signaling through TLR4, increased cytokine production (IL‐8 and IL‐10), and modulation of epithelial barrier integrity via ZO1 expression. Our findings suggest that the intestinal epithelium orchestrates both inflammatory and protective mechanisms in response to LPS, balancing immune activation and barrier maintenance.

## 200 HELICOBACTER PYLORI ANTI‐MICROBIAL RESISTANCE IN THE PEDIATRIC POPULATION

*Eru Sujakhu* ^*2*^, *Osman Altun* ^*1*^

^*1*^ *Gastroenterology*, *University of South Alabama*, *Mobile*, *AL*; ^*2*^ *University of South Alabama*, *Mobile*, *AL*

**BACKGROUND:** *Helicobacter pylori* infection is typically acquired in childhood with an estimated global prevalence of 32.3% in the pediatric population, and can become lifelong if not eradicated. Regimens involving a proton pump inhibitor and multiple antibiotics are used for eradication. However, antibiotic resistance is a major reason for treatment failure. Clarithromycin and Levofloxacin resistance rates of 20%‐30% have been reported from single‐center small adult studies. The H. pylori susceptibility for our patient population is unknown.

**METHODS:** A retrospective analysis of 3424 pediatric medical records for patients who had esophagogastroduodenoscopies at the University of South Alabama CW was conducted between November 2018 and February 2025. We found 141 (4%) patients with H. pylori infection. Electronic medical records were used to extract patient information, H. pylori treatment plans, and eradication test results. Using SPSS software, antimicrobial resistance profiles were examined with the above‐mentioned variables.

**RESULTS/DISCUSSION:** 141 patients were found to have H.pylori infection, with a mean age of 12.8 years; 74 (52.5%) were female, 86 (61%) were White, and 45 (33%) were African‐American. Treatment data were available in 140 patients, with the most common treatment regimens being proton pump inhibitor (PPI)‐Clarithromycin‐Amoxicillin (47.1%), PPI‐Amoxicillin‐Metronidazole (29.3%), and PPI‐Bismuth‐Metronidazole‐Tetracycline (16.4%) Table 1. Eradication data were available for 59.1% of the treated patients (84/140). Table 2. The overall eradication rate was 59.5% (50/84). Because of the persistent infection, 34 patients (40.5%) required second‐line therapy. The culture growth rate was 71.1% (37/52), and 28 antibiotic susceptibility data were available. Eight patients had antimicrobial resistance, six (21.5%) being clarithromycin‐resistant and two (7.1%) levofloxacin‐resistant. These rates are consistent with the calculated U.S.‐pooled prevalence and small single‐center studies in U.S. adults and children from the New England region.

Males had higher levels of antibiotic resistance than females (p=0.038). Resistance and increasing age showed a moderately positive correlation (r=0.515).

Among the 39 patients who received clarithromycin‐containing regimens, strains with clarithromycin resistance were statistically significantly higher in eradication failures (5/13, 38.5%) than in eradication success groups(1/26, 3.8%; p=0.004).

**CONCLUSION:** Routine susceptibility testing ensures effective treatment and prevents H.pylori resistance in children. Further large‐scale studies are necessary for reliable conclusions on H.pylori susceptibility in the pediatric population.


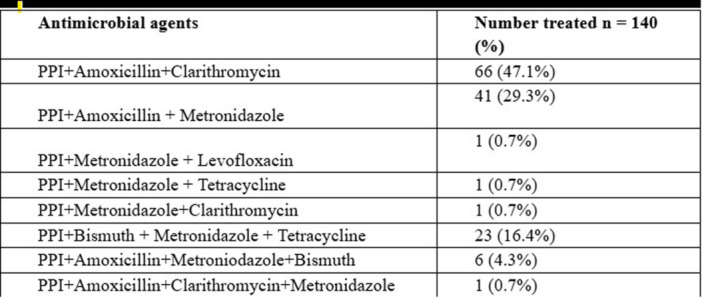


Table 1: Treatment regimens used for H.pylori eradication


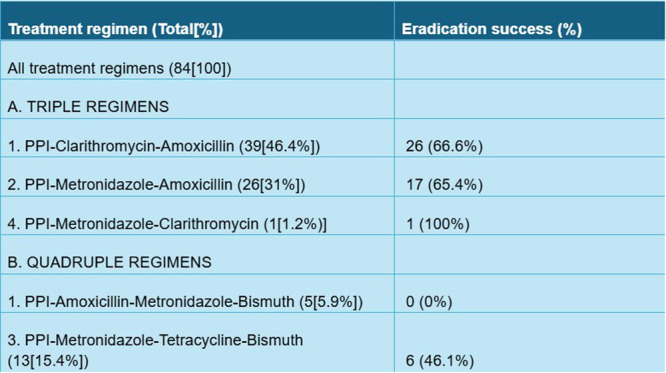


Table 2: Antimicrobial resistance, treatment regimens, and eradication rates

## 203* DEVELOPMENT AND VALIDATION OF A NOVEL NUTRITIONAL THERAPY FOR BILE ACID GASTROESOPHAGEAL AND EXTRAESOPHAGEAL REFLUX DISEASE

*Rachel Rosen* ^*1*^, *Toni Solari* ^*1*^, *Jasper Brezsny‐Feldman* ^*1*^, *Lauren Jalali* ^*1*^, *Elise Delaney* ^*1*^, *Christopher Chalmers* ^*1*^, *Michael Kim* ^*1*^, *Samuel Nurko* ^*1*^, *Monica Narvaez Rivas* ^*2*^, *Kenneth Setchell* ^*2*^, *Bridget Hron* ^*1*^

^*1*^ *Gastroenterology*, *Boston Children's Hospital*, *Boston*, *MA*; ^*2*^ *Cincinnati Children's Hospital Medical Center*, *Cincinnati*, *OH*

**Background:** Bile acid reflux has been implicated in vitro and in vivo as a cause for esophagitis, gastritis, Barret's esophagus, lung transplant allograft dysfunction, pulmonary fibrosis and other aerodigestive disorders. One population at greatest risk for bile acid‐related extraesophageal reflux disease is patients with oropharyngeal dysphagia fed by gastrostomy tube. Despite high prevalence of bile acid reflux in these patients, there are no commercially available therapies that effectively sequester bile acids in the stomach to prevent their retrograde flow into the esophagus and lungs. Based on animal studies showing that increased fiber intake results in increased fecal bile acid elimination, we hypothesized that patients receiving high fiber diets would have lower gastric bile acid concentrations and, as a result, higher serum C4 levels. Recognizing this unmet need for a gastric bile acid sequestrant, we developed an enteral tube feed composed of high bile acid sequestering whole foods pureed into a blenderized feed. We hypothesized that this provisionally patented diet would sucessfully bind human bile acids.

**Methods:** Our prospective study consisted of three parts. First, we measured gastric bile acid concentrations in a cohort of 104 patients with oropharyngeal dysphagia receiving oral feeds, enteral feeds with a standard commercial formula or enteral feeds containing at least one high bile‐acid binding ingredient. Second, in a small subgroup of enterally‐fed patients receiving a standard formula or a blenderized diet with at least one high bile binding ingredient, we measured serum C4 levels a proof of concept that successful bile acid sequestration would result in compensatory changes in C4 levels. Finally, based on the results of the first two aims, we created a nutritionally complete, provisionally patented, enteral tube feed that contained 78% (dry weight) of high bile acid binding ingredients. We then tested the bile sequestering capacity of this novel therapy *in vitro* by incubating the high bile acid‐binding feed with human bile acids to create a time curve of bile acid binding. All bile analyses were done using liquid chromatography‐mass spectrometry.

**Results:** First, in our prospective cohort of patients, gastric bile acids differed by diet category; orally fed patients had the highest concentration of gastric bile acids followed by patients receiving a standard enteral formula and then patients receiving at least one high bile binding ingredient (Figure 1 A). In a subset (N=16) of patients receiving enteral feeds, C4 was suprisingly reduced in patients receiving high bile acid‐binding ingredients compared to standard enteral formula (Figure 1B). Finally, when we tested our novel enteral tube feed made from high bile‐acid binding ingredients, 70‐80% of bile acids were successfully bound even within seconds of the initial exposure and this binding persisted for at least an hour (Figure 2).

**Conclusions:** For patients at high risk for bile acid‐related aerodigestive complications, nutritional sequestration of bile acids may be an effective therapy that merits additional study *in vivo*.


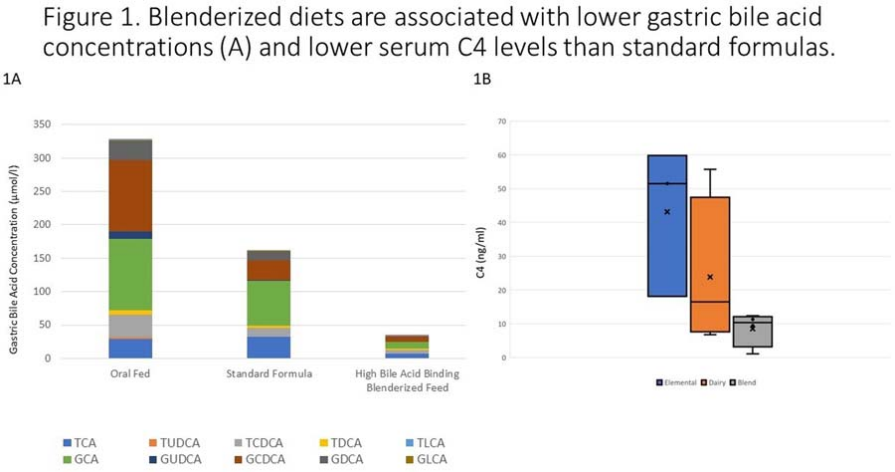



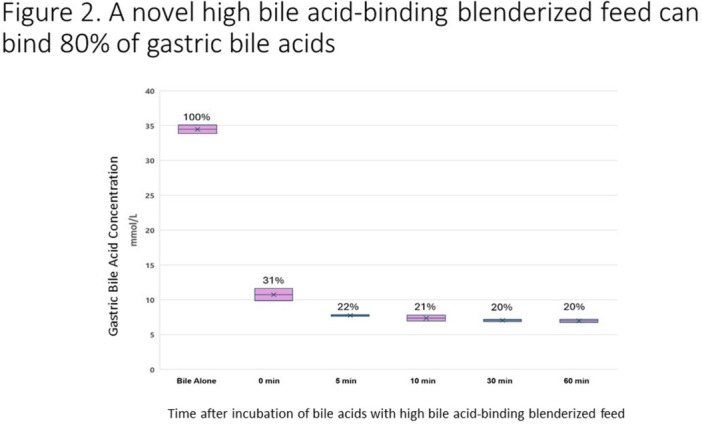


## 204* EVALUATING THE EFFECTS OF AURICULAR NEUROSTIMULATION ON QUALITY OF LIFE AND GASTROINTESTINAL SYMPTOMS IN PEDIATRIC POST‐COVID SYNDROME: A PROSPECTIVE STUDY

*Pari Mokhtari*, *Jamie Janchoi*, *Grace Mucci*, *Tammy Tran*, *Alda Taube*, *Asish Chogle*

*Gastroenterology*, *Children's Hospital of Orange County*, *Orange*, *CA*

**Background:** Post‐COVID Syndrome (PCS) in children frequently includes persistent gastrointestinal (GI) symptoms such as abdominal pain, nausea, and altered bowel habits, alongside fatigue, cognitive difficulties, and functional impairment. These GI symptoms resemble those seen in pediatric Disorders of Gut‐Brain Interaction (DGBIs). Percutaneous electrical nerve field stimulation (PENFS), a non‐invasive vagal neuromodulation therapy shown to improve functional GI symptoms, is being investigated as a potential treatment for PCS‐related GI symptoms. This study evaluates the impact of PENFS on GI symptoms and quality of life in children with PCS.

**Methods:** This prospective study enrolled children aged 11–18 years with PCS lasting over three months. Participants received six weekly PENFS treatments. Validated questionnaires were completed at baseline, during treatment, and at 1‐week and 1‐month follow‐up. Outcomes included the Pediatric Quality of Life Inventory (PedsQL), Functional Disability Inventory (FDI), Abdominal Pain Index (API), Children's Somatization Inventory (CSI), and PROMIS scales (Global Health, Anxiety, Depression), reported by both children and parents. Longitudinal changes were assessed using linear mixed‐effects models.

**Results:** Eighteen children completed the study. At baseline, all participants reported at least one gastrointestinal (GI) symptom. The average time between reported COVID‐19 infection and initiation of PENFS treatment was 2.2 years. Significant improvements were observed from baseline to 1‐month follow‐up in child‐reported outcomes, including FDI (*p* = 0.033), CSI (*p* < 0.001), API (*p* = 0.019), and PROMIS Global Health (*p* < 0.001). Depression scores also significantly improved (*p* < 0.001).

Parent‐reported outcomes showed significant improvements in PedsQL Physical (*p* = 0.001), Emotional (*p* < 0.001), School Functioning (*p* = 0.027), and General Well‐Being (*p* < 0.001). Parent‐reported FDI also decreased significantly (*p* < 0.001).

**Conclusions:** PENFS was associated with improvements in GI and non‐GI symptoms, including disability, somatic distress, pain, and emotional well‐being. PENFS promises a non‐invasive treatment for pediatric Long COVID. Randomized trials with larger sample sizes are needed to confirm efficacy and assess long‐term benefit.

## 205* COMPARISON OF RESPONSE TO TEMPORARY GASTRIC ELECTRICAL STIMULATION VERSUS STIMULATOR IMPLANTATION IN CHILDREN WITH REFRACTORY NAUSEA AND VOMITING

*Eduardo Castillo Leon* ^*1*^, *Anya Kirsch* ^*1*^, *Amina Usman* ^*1*^, *Raul Sanchez* ^*1*^, *Neetu Bali Puri* ^*1*^, *Karla Vaz* ^*1*^, *Desale Yacob* ^*1*^, *Md‐Rejuan Haque* ^*2*^, *Karen Diefenbach* ^*3*^, *Carlo Di Lorenzo* ^*1*^, *Peter Lu* ^*1*^

^*1*^ *Gastroenterology*, *Nationwide Children's Hospital*, *Columbus*, *OH*; ^*2*^ *Center for Biostatistics, Department of Biomedical Informatics*, *The Ohio State University*, *Columbus*, *OH*; ^*3*^ *Center of Colorectal and Pelvic Reconstruction*, *Nationwide Children's Hospital*, *Columbus*, *OH*

**Background:** Gastric electrical stimulation (GES) improves gastrointestinal symptoms and quality of life in children with refractory nausea and vomiting. At our institution, patients undergo a trial of temporary GES to assess response prior to permanent implantation. Our objectives are to compare response during temporary vs. permanent GES and to evaluate how changes in permanent GES settings impact symptoms.

**Methods:** We completed a prospective cohort study. We identified patients <21 years old treated with permanent GES at our institution between 2016‐2024. Encounters were selected at baseline, during the trial of temporary GES, and at 2, 4, 6, and 12 months follow up during permanent GES. At each encounter, GES settings were recorded and patients completed the Symptom Monitor Worksheet (SMW) and the Pediatric Quality of Life Inventory (PedsQL). We used paired t‐test to compare SMW and settings between temporary and permanent GES. Improvement or worsening of symptoms were defined as an increase or decrease in >7 points in SMW. We used a linear mixed effect regression model to evaluate changes over time and mixed effect logistic regression to evaluate association between settings and SMW.

**Results:** We included 38 patients (73% F, median age 15 years). Nearly all had a history of gastroparesis (97%), 53% had functional dyspepsia, 42% had postural orthostatic tachycardia syndrome, and 72% had anxiety/depression. Twenty‐six patients had data available during both temporary and permanent GES; 20/26 improved during the temporary trial before proceeding with permanent GES. Patients who experienced further improvement, maintained improvement, or worsened after permanent GES are shown in **Figure 1**. Patients with permanent GES had a lower stimulation voltage than those with temporary GES; 7 volts versus 10 volts (p<0.001) respectively. However, SMW and PedsQL in temporary and permanent phases were similar. Thirty‐three patients had >1 follow up available during permanent GES. During the first year of permanent GES, SMW and PedsQL scores remained stably improved from baseline. Stimulation voltage and frequency gradually increased with time, while lead impedance increased at 6 months before decreasing at 12 months. We identified 74 permanent GES follow up encounters. When compared with the preceding encounter, 34% (25/74) of these encounters showed improvement, 19% (14/74) showed worsening, and 58% (43/74) showed no change. As shown in **Figure 2**, there was no association between changes in voltage or frequency and changes in SMW.

**Conclusion:** In this prospective study of children with refractory nausea and vomiting treated with GES, improvement in symptoms and quality of life reaches its peak during the temporary phase, and few experience further improvement after implantation and starting permanent GES. This supports the use of temporary GES to assess response prior to deciding to proceed with stimulator implantation. Our findings also highlight the limited benefit of additional changes to voltage or frequency after permanent implantation.


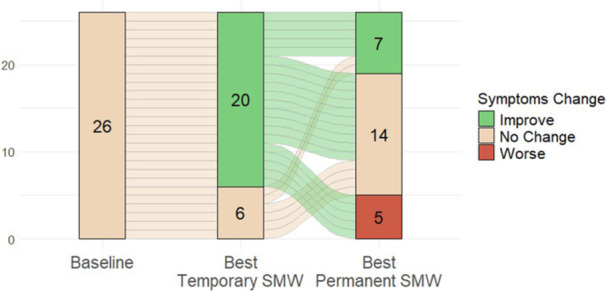


**Figure 1.** Alluvial plot showing symptom changes from baseline to follow up during the trial of temporary gastric electrical stimulation (GES) and during permanent GES after stimulator implantation.


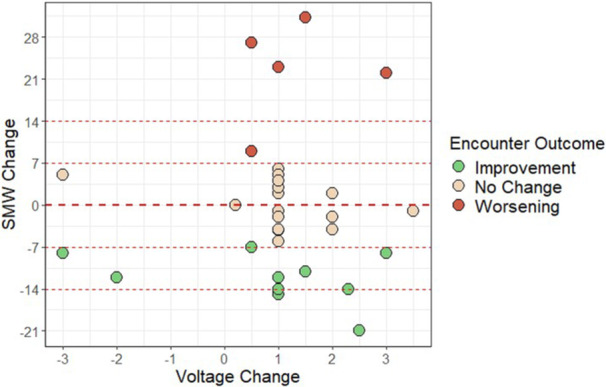


**Figure 2.** Scatterplot showing changes in Symptom Monitor Worksheet (SMW) score with changes in voltage of gastric electrical stimulation (GES). Points are colored by clinical outcome: improvement (green), no change (tan), and worsening (red).

## 206* EFFICACY AND SAFETY OF LINACLOTIDE IN PEDIATRIC PATIENTS AGED 2–5 YEARS WITH FUNCTIONAL CONSTIPATION: RESULTS FROM PART 1 OF A PHASE 3, RANDOMIZED, PLACEBO‐CONTROLLED STUDY

*Carlo Di Lorenzo* ^*1*^, *Miguel Saps* ^*2*^, *Samuel Nurko* ^*4*^, *Gerardo Rodriguez‐Araujo* ^*3*^, *Mena Boules* ^*5*^, *Valentina Shakhnovich* ^*5*^, *Wangang Xie* ^*3*^, *Julie Khlevner* ^*6*^, *Jeffrey Hyams* ^*7*^

^*1*^ *Pediatric Gastroenterology*, *Nationwide Children's Hospital*, *Columbus*, *OH*; ^*2*^ *University of Miami Miller School of Medicine*, *Miami*, *FL*; ^*3*^ *AbbVie Inc*, *North Chicago*, *IL*; ^*4*^ *Boston Children's Hospital*, *Boston*, *MA*; ^*5*^ *Ironwood Pharmaceuticals Inc*, *Boston*, *MA*; ^*6*^ *Department of Pediatrics*, *Columbia University Vagelos College of Physicians and Surgeons*, *New York*, *NY*; ^*7*^ *Connecticut Children's Medical Center*, *Hartford*, *CT*

**Introduction:** Linaclotide (LIN) is FDA‐approved to treat functional constipation (FC) in patients aged 6–17 years. This study evaluated the efficacy and safety of LIN in patients aged 2–5 years with FC during Part 1 of a double‐blind, randomized, placebo (PBO)‐controlled Phase 3 study (NCT05652205).

**Methods:** Patients aged 2–5 years who met modified Rome IV criteria for FC were randomized 1:1 to receive LIN 72 µg or PBO once daily for 12 weeks (stratified by age [2–3 and 4–5 years]; Part 1); Part 2 is an ongoing 24‐week, open‐label, long‐term safety extension. The primary efficacy endpoint was the change from baseline (CFB) in 12‐week spontaneous bowel movement (SBM) frequency rate (SBMs/week) based on SBMs reported by primary caregivers only. As children may have other caregivers (e.g., grandparents, at daycare) who can report SBMs, a supplementary analysis included all SBMs reported by primary and secondary caregivers. Secondary efficacy endpoints included the CFB in 12‐week stool consistency (assessed using the Bristol Stool Form Scale) and straining observed by primary caregivers. Efficacy data were collected in eDiaries by caregivers. Missing data were imputed with a control‐based imputation assuming missing not at random. A mixed effect model for repeated measures estimated treatment effects for LIN vs PBO. Use of rescue medication was assessed. Safety assessments included the frequency of treatment‐emergent adverse events (TEAEs). Endpoints were assessed in all randomized patients who received ≥1 dose of study drug. LIN is not FDA‐approved for use in patients aged 2–5 years with FC.

**Results:** A total of 123 randomized patients (LIN 72 µg, n=62; PBO, n=61) received ≥1 dose of study drug. Demographics and baseline clinical characteristics were balanced between groups (**Table 1**). The least squares (LS) mean CFB in 12‐week SBM frequency rate reported by primary caregivers was 1.74 vs 1.42 SBMs/week for patients treated with LIN 72 µg vs PBO; statistical superiority was not met (*P*=0.293). In the supplementary analysis including all SBMs reported by primary and secondary caregivers, the LS mean CFB was 2.07 vs 1.40 SBMs/week for patients treated with LIN 72 µg vs PBO, reaching nominal statistical significance (*P*=0.040). LIN 72 µg showed an improvement in CFB over 12 weeks in stool consistency vs PBO (LS mean CFB of 1.00 vs 0.62 [*P*=0.020]). For straining, the LS mean CFB over 12 weeks for LIN 72 µg vs PBO was −0.54 vs −0.53 (*P*=0.843). Rescue medication was used less frequently by patients treated with LIN 72 µg (64.5%) vs PBO (75.4%). TEAE frequency was similar between groups, with no serious TEAEs or TEAEs leading to treatment discontinuation (**Table 2**). Diarrhea was reported in one patient (1.6%) treated with LIN 72 µg and two patients (3.3%) treated with PBO.

**Conclusion:** Stool consistency improved in patients aged 2–5 years with FC treated with LIN 72 μg. While the primary endpoint was not met, a supplementary analysis including all SBMs reported by primary and secondary caregivers demonstrated a nominally significant treatment effect of LIN 72 µg vs PBO for the SBM frequency rate. LIN 72 µg was well tolerated with a low incidence of diarrhea (n=1, not treatment related).


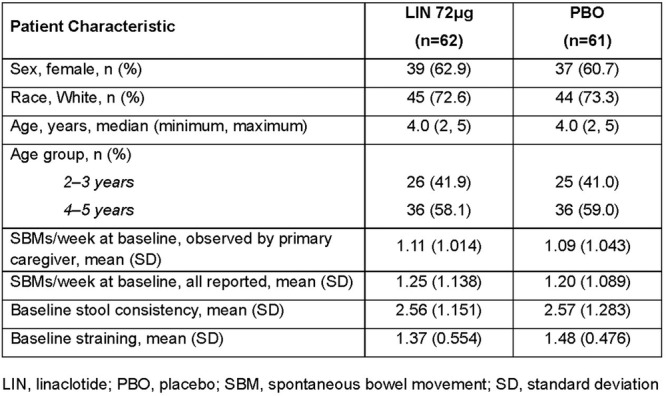


**Table 1.** Demographics and Baseline Clinical Characteristics


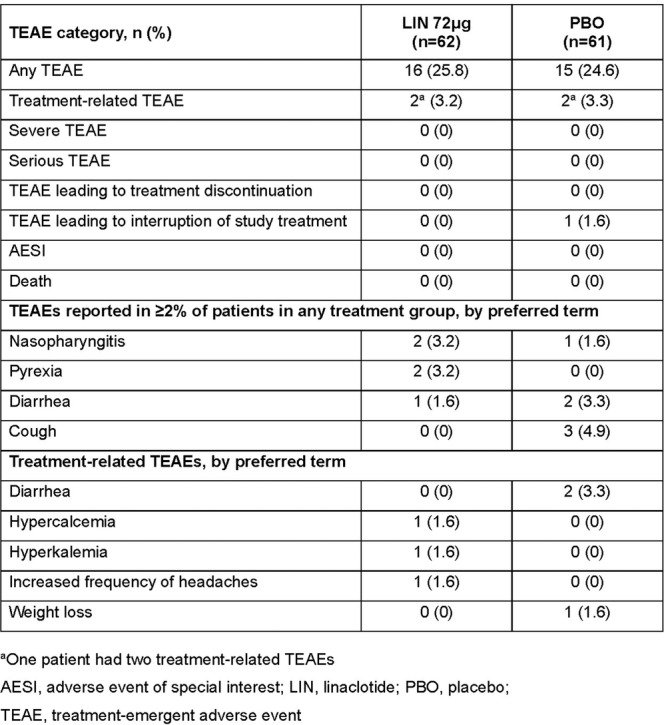


**Table 2.** Summary of TEAEs

## 212 EFFICACY OF APREPITANT AS AN ADJUNCTIVE THERAPY FOR CANNABINOID HYPEREMESIS SYNDROME IN PEDIATRIC PATIENTS: A RETROSPECTIVE COMPARATIVE STUDY OF CLINICAL OUTCOMES

*Nikolai Aksenov*, *Allen Hong*, *Taiwo Solarin*, *Christina Morrissey*, *Guru Bhoojhawon*

*Driscoll Children's Hospital*, *Corpus Christi*, *TX*

**Background:** Cannabinoid hyperemesis syndrome (CHS) is a challenging condition characterized by cyclic episodes of nausea, vomiting, and abdominal pain in chronic cannabis users. Initially described in adults, CHS is increasingly recognized in pediatric populations due to rising cannabis use among adolescents. The pathophysiology of CHS remains poorly understood, but it is thought to involve dysregulation of the endocannabinoid system, particularly CB1 receptors in the gastrointestinal tract and central nervous system; chronic cannabis use may lead to desensitization of these receptors, causing paradoxical hyperemesis.

Conventional antiemetics, such as Ondansetron, often fail to provide adequate symptom relief in CHS, highlighting the need for alternative therapeutic strategies. Aprepitant, an NK‐1 receptor antagonist, has shown promise in managing refractory nausea and vomiting in other conditions, such as chemotherapy‐induced nausea and vomiting. Emerging case reports and small studies suggest that Aprepitant may be effective in CHS, but robust clinical evidence in pediatric populations is lacking. The purpose of the study is to show that the addition of Aprepitant to standard antiemetics improves the outcomes of pediatric patients with CHS.

**Objective:** To evaluate the efficacy of Aprepitant as adjunctive therapy compared to standard antiemetics alone in pediatric patients hospitalized with CHS.

**Methods:** We conducted a retrospective chart review of all CHS admissions (ages 12‐21 years) at Driscoll Children's Hospital (Corpus Christi, USA, TX) from 01/01/2020 to 05/01/2025. Patients were identified using ICD‐10 codes and confirmed by positive urine drug screen for cannabinoids plus clinical criteria. Admissions were stratified into two groups: standard antiemetics alone (ondansetron, metoclopramide, promethazine, haloperidol, lorazepam, etc.) versus standard antiemetics plus Aprepitant. Primary outcomes included hospital length of stay (LOS), time to achieve a 6‐hour vomit‐free period, and total vomiting episodes. Secondary outcomes included readmission rates and emergency department (ED) visits. Statistical analyses included Mann‐Whitney U tests, chi‐square tests, and Kaplan‐Meier survival analysis with log‐rank testing.

**Results:** We analyzed 122 admissions (69 control, 53 Aprepitant). Baseline characteristics were comparable between groups. Aprepitant therapy was associated with significant reductions in:

‐ Hospital LOS: 58.2 hours (95% CI: 47.9‐68.4) vs 33.8 hours (95% CI: 29.8‐37.8); difference 24.4 hours (95% CI: 13.3‐35.4), p<0.001

‐ Mean vomiting episodes: 7.1 (95% CI: 4.6‐9.6) vs 1.5 (95% CI: 0.8‐2.3), p<0.001

‐ Time to 6‐hour vomit‐free period: 11.4±14.5 vs 7.3±2.4 hours, p<0.05

Notably, 52.8% (95% CI: 39.4‐66.3%) of Aprepitant‐treated patients achieved zero vomiting episodes during hospitalization compared to 17.4% (95% CI: 8.4‐26.3%) of controls (p<0.001), yielding a number needed to treat (NNT) of 2.8 (95% CI: 1.9‐5.2). Effect sizes were large for both LOS (Cohen's d=0.75) and vomiting reduction (d=0.73). Secondary outcomes showed numerical reductions in healthcare utilization that did not reach statistical significance: 7‐day readmissions 11.4% (95% CI: 4.0‐18.9%) vs 3.8% (95% CI: 0.0‐8.9%), p=0.124; 7‐day ED visits 20.6% (95% CI: 11.0‐30.2%) vs 9.8% (95% CI: 1.6‐18.0%), p=0.112.

**Conclusions:** In this first large retrospective study of Aprepitant for pediatric CHS, NK‐1 antagonist therapy demonstrated clinically meaningful and statistically significant benefits, reducing hospitalization duration by nearly 24 hours and achieving complete symptom control in over half of the treated patients. These findings support incorporating Aprepitant into standard CHS treatment protocols. The favorable NNT of 2.8 (95% CI: 1.9‐5.2) and substantial reduction in LOS indicate both clinical efficacy and potential healthcare savings. While trends toward reduced readmissions and ED visits were observed, prospective trials are warranted to confirm these findings and optimize dosing strategies. We suggest that NK1 receptor antagonism may target key pathophysiologic mechanisms in CHS that are not addressed by conventional antiemetics.

## 213 SINGLE‐CELL RNA SEQUENCING AND DEEP LEARNING ANALYSIS OF PEDIATRIC DISORDERS OF GUT‐BRAIN INTERACTION (DGBI) REVEAL IMMUNE CELL COMPOSITIONAL SHIFTS ACROSS DIAGNOSTIC SUBGROUPS

*Zoë Steier* ^*1*^, *Hengqi Zheng* ^*3*^, *Julianne Flusche* ^*1*^, *Kyle Kimler* ^*2*^, *Sara Rosenbaum* ^*2*^, *Brandi Bratrude* ^*2*^, *Paula Keskula* ^*2*^, *Jillian Zavistaski* ^*2*^, *Alexandre Albanese* ^*2*^, *Jeffrey Goldsmith* ^*2*^, *Leonard Nettey* ^*1*^, *Joseph Devlin* ^*4*^, *Hannibal Person* ^*3*^, *Lusine Ambartsumyan* ^*3*^, *Ghassan Wahbeh* ^*3*^, *David Suskind* ^*3*^, *Scott Snapper* ^*2*^, *Wei Lim* ^*4*^, *Alex Shalek* ^*1*^, *Jose Ordovas‐Montanes* ^*2*^, *Leslie Kean* ^*2*^

^*1*^ *Institute for Medical Engineering and Science*, *Massachusetts Institute of Technology School of Engineering*, *Cambridge*, *MA*; ^*2*^ *Boston Children's Hospital*, *Boston*, *MA*; ^*3*^ *Seattle Children's Hospital*, *Seattle*, *WA*; ^*4*^ *Regeneron Pharmaceuticals Inc*, *Tarrytown*, *NY*

**Background:** Pediatric Disorders of Gut‐Brain Interaction (DGBIs), also referred to as Functional Gastrointestinal Disorders (FGIDs), are highly prevalent yet lack quantitative positive diagnostics and targeted treatments. To further the development of diagnostics and therapeutics, greater understanding of DGBIs is needed on the cellular and molecular levels. Thus far, single‐cell RNA sequencing (scRNA‐seq) comparisons of pediatric DGBIs and healthy controls have not been explored. We aimed to characterize pediatric DGBIs as compared to healthy controls using scRNA‐seq and deep learning analysis of gene expression, cell type composition, and inter‐sample heterogeneity.

**Methods:** Samples were collected at the time of diagnostic endoscopy from the duodenum, terminal ileum, colon (each physically dissociated into lamina propria and epithelial layers), and blood (either peripheral blood mononuclear cells or CD3+ cells enriched via magnetic column). The study included 24 pediatric DGBI participants (ages 6‐21), of which 11 were classified as Functional Abdominal Pain ‐ Not Otherwise Specified (FAP) and 8 as Irritable Bowel Syndrome (IBS) by the Rome IV Criteria. The same samples were collected from 9 young adult healthy control participants (ages 18‐24). scRNA‐seq was performed on each sample with 10x Genomics. Adjacent tissue samples were processed for histological imaging. scRNA‐seq analysis was performed in python. We used Scanpy for basic preprocessing, scvi‐tools for batch correction and differential expression testing, scArches for integration and label transfer, and scCODA for differential abundance testing. We used Aperio ImageScope and QuPath for image analysis.

**Results:** scRNA‐seq data (**Fig. 1A‐E**) analyzed by differential abundance testing identified significant shifts (FDR 0.05) in lamina propria cell type composition between DGBIs and healthy controls, and between the DGBI diagnostic subgroups FAP and IBS. DGBI showed a 2‐fold reduction in CCL11+ fibroblasts in the ileum relative to healthy controls. In the ileum, IBS had 2‐fold increases in CD4+ and CD8+ memory T cells relative to healthy controls, which were not observed in FAP. FAP had 2‐fold increases in T follicular helper (Tfh) cells and germinal center B (B GC) cells and a 2‐fold decrease in IgA Plasma B cells relative to healthy controls, which were not observed in IBS. FAP and IBS did not significantly differ in abundance in the duodenum or colon, and significant abundance changes were not detected in the epithelium or blood. Significant differential gene expression was negligible between diagnoses and subgroups. Based on the concerted shifts in Tfh cell and B GC cell abundance in the FAP ileum (**Fig. 1 F**), we hypothesized that there might be changes in ileal tertiary lymphoid structures. Quantitative analysis of histological images showed a significant increase in lymphoid aggregate density in FAP relative to healthy controls (p = 0.002; **Fig. 1 G**).

**Conclusions:** Our scRNA‐seq data show shifts in lamina propria cell type composition between DGBI and healthy controls, and ileal immune cell compositional differences between the DGBI subgroups FAP and IBS. Our findings suggest that the underlying mechanisms of FAP and IBS might be distinct, motivating subgroup‐specific treatment strategies. The cellular compositions observed in this study merit further investigation as potential tissue biomarkers and eventual targets for DGBI diagnostics and treatments. Future work is warranted to understand the cellular changes and their interactions with enteric nervous system signaling for treatment development.


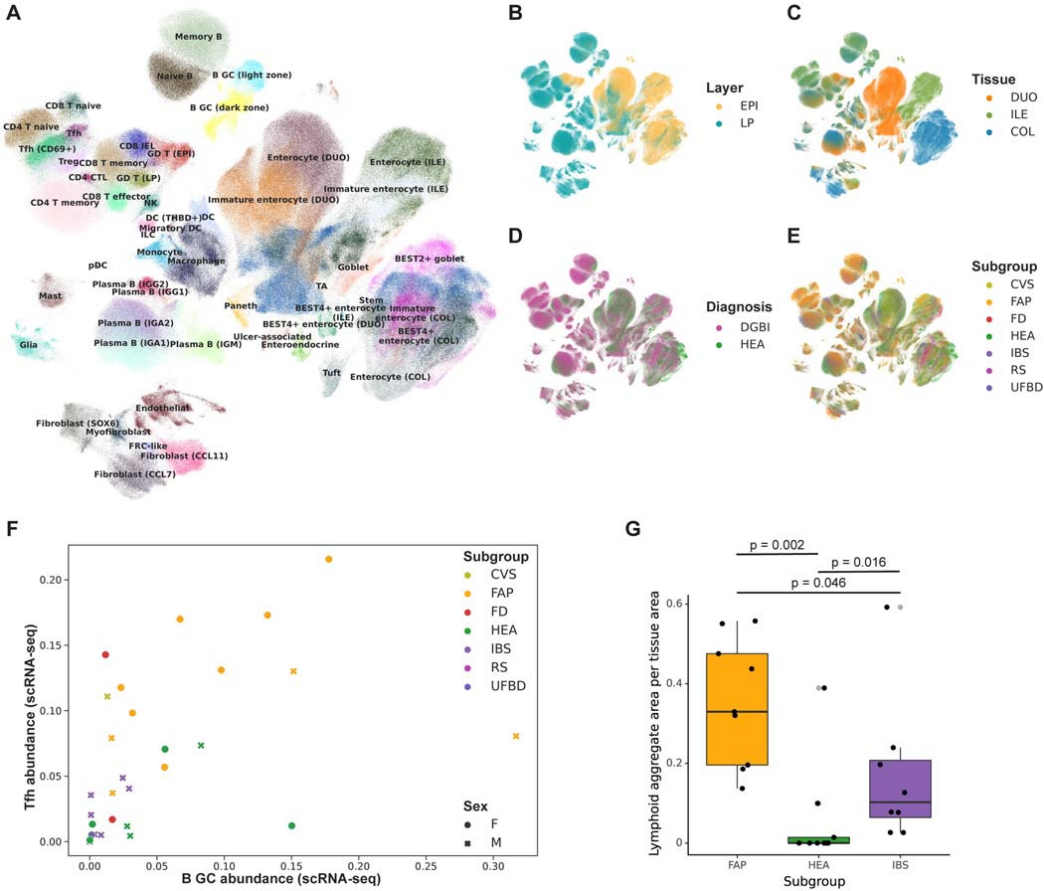


**Fig. 1:** scRNA‐seq analysis of pediatric DGBI identifies shifts in immune cell composition. **(A‐E)** UMAP representation of scANVI latent space for all cells from gastrointestinal tissue samples colored by cell type annotation (A), layer (B), tissue (C), diagnosis (D), and subgroup (E). **(F)** Abundance of B GC cells versus Tfh cells in each ileum lamina propria scRNA‐seq sample. **(G)** Lymphoid aggregate area per total tissue area in H&E images of ileum tissue samples.

## 214 COMPARISON OF THE THERAPEUTIC EFFICACY OF MACROGOL 3350 VS BIOFEEDBACK IN PEDIATRIC PATIENTS WITH DYSSYNERGIC DEFECATION DIAGNOSED WITH HIGH RESOLUTION ANORECTAL MANOMETRY

*Vasiliki‐ Maria Karagianni* ^*1*^, *Eirini Chantzi* ^*2*^, *Maria Rogalidou* ^*1*^, *Konstantina Dimakou* ^*1*^, *Vasiliki Karava* ^*3*^, *Panayiota Kafritsa* ^*1*^, *George Karamanolis* ^*4*^, *Alexandra Papadopoulou* ^*1*^

^*1*^ *1Division of Gastroenterology and Hepatology, First Department of Pediatrics, University of Athens*, *Nosokomeio Paidon e Agia Sophia*, *Athens*, *Attica*, *Greece*; ^*2*^ *Pediatric Centre for Anorectal and Urogenetic Disorders*, *Athens*, *Greece*; ^*3*^ *First Department of Pediatrics, University of Athens*, *Agia Sofia Children's Hospital*, *Athens*, *Greece*; ^*4*^ *Academic Department of Gastroenterology*, *Medical School, National and Kapodistrian University of Athens, Laiko General Hospital*, *Athens*, *Greece*

**Objectives and Study:** Functional constipation has a worldwide prevalence of 9,5% [1]. Functional constipation's main subtypes are normal transit constipation, slow transit constipation and outlet obstruction. A common form of outlet obstruction is dyssynergic defecation (DD). It is an acquired behavioral problem, due to the inability to coordinate the abdominal and pelvic floor muscles to evacuate stools. Anorectal manometry is essential for a diagnosis of DD. Normally, when a subject bears down or attempts to defecate, there is a rise in rectal pressure, which is synchronized with a relaxation of the external anal sphincter. The inability to perform this coordinated movement represents the key pathophysiologic abnormality in DD. According to Rao's Criteria there are 4 types of DD [image 1] [2].

The aim of the study is to compare the efficacy of osmotic laxatives with biofeedback therapy in the treatment of children with DD.

**Methods:** Over a 24‐month period, high‐resolution anorectal manometry was performed in 71 children aged 4‐17 years with functional constipation, after written parental consent for disclosure of possible DD. A questionnaire to measure specific symptoms of constipation (PAC‐SYM questionnaire) [image 2] was used to assess the severity of constipation. Moreover, Bristol Stool Scale Score and presence of soiling and retentive behaviour were estimated. Patients diagnosed with DD, according to Rao's criteria, were randomly assigned to treatment with Macrogol 3350 (dosage: 0,8‐1 gr/kg/day) for 2 months or biofeedback for 6 sessions (approximately 1 session/week).

**Results:** 48 out of 71 patients with functional constipation (67 %) were diagnosed with dyssynergic defecation. 22/48 (46%) patients were diagnosed with type 3 and 26/46 (54%) with type 1 DD.Their median age was 6,5 (4‐17) years. The majority of patients with DD were males (52%). 29/48 (60%) presented with soiling.Seventeen patients were randomized to macrogol 3350 treatment and 26 to biofeedback, while 5 patients did not complete treatment protocol.The median percentage of score reduction after treatment was 59% (33‐100%) overall:

‐ 70% (33‐100%) median percentage of score reduction after treatment with biofeedback

‐ 48 % (16‐90%) median percentage of score reduction after macrogol treatment (p= 0,023)

There was not statistically significant difference in the median percentage of PAC ‐SYM score reduction between the two treatment groups, neither in the patient group with DD type 1 (p=0,073), nor in the patient group with DD type 3 (p= 0,238). In the group of patients with soiling the median percentage of score reduction was not higher in a statistically significant degree withbiofeedback therapy compared to macrogol treatment (p=0.194).

**Conclusions:** Biofeedback therapy is more effective than the common macrogol treatment in the subgroup of pediatric patients with functional constipation that present dyssynergic defecation in high resolution anorectal manometry. The superiority of biofeedback therapy is statistically significant.

**References:**

1) Koppen, I. J. N. et al. Prevalence of functional defecation disorders in children: a systematic review and meta‐analysis. J. Pediatr. 198, 121–130 (2018). 2) Rao SS, Patcharatrakul T. Diagnosis and Treatment of DyssynergicDefecation. J Neurogastroenterol Motil. 2016 Jul 30;22(3):423‐35.

## 216 INTRAPYLORIC BOTULINUM INJECTION AS A TREATMENT FOR GASTROPARESIS IN PEDIATRIC LUNG TRANSPLANT

*Suzanna Hirsch* ^*1*^, *Timothy Klouda* ^*2*^, *Enju Liu* ^*1*^, *Michael Kim* ^*1*^, *Samuel Nurko* ^*1*^, *Gary Visner* ^*2*^, *Rachel Rosen* ^*1*^

^*1*^ *Gastroenterology*, *Boston Children''s Hospital*, *Boston*, *MA*; ^*2*^ *Pulmonary*, *Boston Children's Hospital*, *Boston*, *MA*

**Background:** Gastroparesis is common in lung transplant recipients as a pre‐transplant comorbidity or a post‐transplant complication, and it can contribute to gastrointestinal (GI) and pulmonary morbidity. Intrapyloric botulinum injection (IPBI) has been shown to improve gastric emptying in one small case series of adult lung transplant recipients, however the impact of IPBI on clinical symptoms and lung transplant outcomes is not known and there is no pediatric research. The objective of the current study was to evaluate the impact of IPBI on clinical symptoms, gastric motility, and graft and survival outcomes following pediatric lung transplant.

**Methods:** This was a retrospective observational study of pediatric lung transplant recipients who underwent lung transplant at a tertiary center between January 2008 and March 2024. Baseline data were recorded including demographic and clinical characteristics at the time of transplant, gastric emptying scintigraphy (GES) after transplant, and GI symptoms preceding IPBI. Gastroparesis was defined as a gastric residual > 10% for a 4‐hour study or > 60% for a 1‐hour study. Covariates included age, sex, transplant indication, diabetes, fundoplication, intraoperative ischemic time, and post‐operative ECMO. GI outcomes included changes to GI symptoms (improvement vs. no improvement based on the first GI or pulmonary note after IPBI) and changes to gastric emptying on repeat GES within 6 months after IPBI. Pulmonary outcomes included time to chronic lung allograft dysfunction (CLAD) and time to retransplantation or death. Gastric residuals pre‐ and post‐IPBI were compared using Wilcoxon signed‐rank test. Time to CLAD or retransplantation/death was compared between the three groups (Group 1: no gastroparesis; Group 2: gastroparesis and received IPBI; Group 3: gastroparesis and did not receive IPBI) using Cox regression controlling for the above covariates.

**Results:** Of 63 pediatric lung transplant recipients, 26 (41%) had gastroparesis identified on GES, including 18 patients who underwent IPBI for management of gastroparesis symptoms. Patients had a mean age of 12.5±6.3 years, and the most common transplant indications were cystic fibrosis (24/63, 38%) or pulmonary hypertension (14/63, 22%). There were no significant differences in baseline patient characteristics across groups. The most common symptoms in patients undergoing IPBI included nausea (12/18, 67%), vomiting (10/18, 56%), excessive fullness/early satiety (6/18, 33%), gagging/retching (4/18, 22%), or gastric feeding intolerance (4/18, 22%). Sixteen patients who underwent IPBI had follow‐up GI symptom data available, and 11/16 (69%) showed symptomatic improvement 23.19±18.58 days after IPBI. Six patients underwent repeat GES occurring 3.46±2.12 months after IPBI. Mean gastric residuals were reduced after IPBI, though this did not reach statistical significance (pre‐IPBI gastric residual 51.83 ± 25.42 vs. post‐IPBI 35.67 ± 22.53, *P* = 0.17; Figure 1). Of the total sample, 31/63 (49%) had CLAD and 31/63 (49%) had retransplantation/death. There were no significant differences between time to CLAD or time to retransplantation/death across groups.

**Discussion:** This is the largest study to investigate IPBI as a treatment for gastroparesis in lung transplant recipients and the first study in pediatrics. We found that gastroparesis was common in pediatric patients following lung transplant and that treatment with IPBI was associated with improvements in both upper GI symptoms and motility. Despite notable improvements in GI outcomes, IPBI was not associated with improved graft or survival outcomes in this sample.


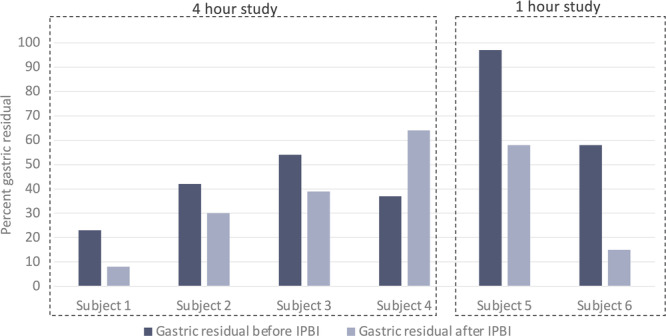


Comparing gastric residuals before and after intrapyloric botulinum injection in pediatric lung transplant recipients


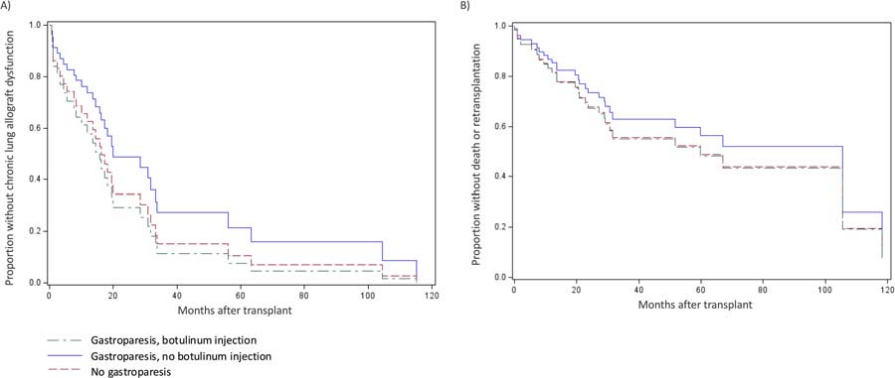


No significant group differences in time to (A) chronic lung allograft dysfunction (CLAD) or (B) retransplantation or death

## 217 ARE CHILDREN WITH CEREBRAL PALSY MORE LIKELY TO DEVELOP ESOPHAGITIS, REGARDLESS OF GASTROESOPHAGEAL REFLUX SYMPTOMS?

*Cecilia Zubiri* ^*1*^, *Cristina Lorenzo* ^*1*^, *Anabella Sozi* ^*1*^, *Ana Rocca* ^*2*^, *Maria Neder* ^*2*^, *Judith Cohen Sabban* ^*3*^, *Sandro Miculan* ^*1*^, *Roman Bigliardi* ^*4*^, *Maria Florencia Biasoli* ^*5*^, *Manuela Manterola* ^*6*^, *Luis Orlando Perez* ^*7*^, *Maria de los Angeles Savia* ^*8*^, *Renata Weinschelbaum* ^*9*^, *Maria Soledad Arcucci* ^*3*^, *Veronica Plante* ^*10*^, *Christian Boggio* ^*10*^, *Maria Alejandra Mortarini* ^*11*^, *Carlos Ruiz Hernandez* ^*12*^, *Erick Toro Monjaraz* ^*15*^, *Soraia Tahan* ^*14*^, *Ana Cristina Fontenele Soares* ^*14*^, *Samantha Arrizabalo* ^*13*^, *Miguel Saps* ^*13*^

^*1*^ *Gastroenterology*, *HIAEP Sor Maria Ludovica*, *La Plata*, *Buenos Aires*, *Argentina*; ^*2*^ *Hospital JP Garrahan*, *buenos Aires*, *Argentina*; ^*3*^ *Hospital Italiano de Buenos Aires*, *Buenos Aires*, *Buenos Aires*, *Argentina*; ^*4*^ *Hospital Posadas*, *Buenos Aires*, *Argentina*; ^*5*^ *Hospital Ricardo Gutierrez*, *Buenos Aires*, *Argentina*; ^*6*^ *Hospital El Cruce*, *Buenos Aires*, *Buenos Aires*, *Argentina*; ^*7*^ *Instituto Patagonico de ciencias sociales y humanas*, *Puerto Madryn*, *Argentina*; ^*8*^ *Hospital Horacio Cestino*, *Ensenada*, *Argentina*; ^*9*^ *Sanatorio de Niños de Rosario*, *Rosario*, *Argentina*; ^*10*^ *Hospital Pirovano*, *Buenos Aires*, *Argentina*; ^*11*^ *Hospital Austral*, *Buenos Aires*, *Argentina*; ^*12*^ *Hospital Sant Joan de Deu*, *Barcelona*, *CT*, *Spain*; ^*13*^ *University of Miami Miller School of Medicine*, *Miami*, *FL*; ^*14*^ *Universidade de Sao Paulo*, *São Paulo*, *SP*, *Brazil*; ^*15*^ *Instituto Mexicano de pediatria*, *Mexico*, *Mexico*

**AIM:** To determine if children with cerebral palsy (CP) have a higher risk of developing gastroesophageal reflux disease (GERD) based on MII‐pH monitoring findings regardless of gastroesophageal reflux (GER) symptoms.

**METHODS:** A multicenter, retrospective study was conducted from May 2017 to January 2024. This research involved evaluating MII‐pH tracings of children aged 1 to 15 with CP. It was carried out by members of the LASPGHAN Motility Working Group across 11 centers in four countries (Argentina, Brazil, Mexico, and Spain).

The study population was divided into two groups. Group 1 consisted of children with CP who exhibited symptoms of GER, while Group 2 comprised children with CP without GER symptoms. The studies were conducted prior to the implementation of gastrostomy for feeding.

Exclusion criteria included children younger than 1 year, patients with esophageal atresia, patients who underwent Nissen fundoplication, those with corrected or uncorrected diaphragmatic hernias, children receiving antacids or prokinetics during the study period, and studies shorter than 18 hours.

All analyses and visualizations were carried out using the R statistical software. A p‐value of less than 0.05 was considered statistically significant.

*Ethical approval*

The study received ethical approval from the research ethics committee of Alejandro Posadas National Hospital in Argentina. It adhered to the Helsinki Declaration, the CIOMS guidelines (2016), and the Ministry of Health of Argentina's Resolution No. 1480/11. The project's registration code with the CEIHP is ref 878 EMiP0S0/24.

**RESULTS:** A total of 201 children with CP were included in the study. Among them, 108 exhibited GER symptoms (60 males, mean age 6), while 93 did not (48 males, mean age 5,4).

All children were classified under categories IV and V of the Gross Motor Functional Classification System (GMFCS). Of the 110 participants, 81 (73%) had a nasogastric tube in place for feeding during the MII‐pH procedure.The symptomatic group included 75 patients with nasogastric tubes.

A total of 37 children had a reflux index of 7 or higher. Among them, 25 (24.3%) were symptomatic, while 12 (12.95%) were asymptomatic (t‐student = 2.081, p = 0.03).

There were no statistically significant differences in symptom and reflux index distributions between groups (chi‐square = 2.8432, df = 1, p = 0.09176).The number of gastroesophageal reflux episodes was 47 in symptomatic children and 37 in asymptomatic children (p = 0.10).Symptomatic children had a mean of 23.9 (AGER)Acidic Gastroesophageal reflux episodes, while asymptomatic children had a mean of 22.9 (p=0.57).The mean acid clearance time was 144.2 seconds (SD=169) in symptomatic children and 143.8 seconds (SD=162) in asymptomatic children, with no statistically significant difference (p = 0.57).

**CONCLUSION:** Our study shows that pathological RI is more common in CP children and symptoms, but no significant differences were found between the groups with and without symptoms in the cross‐sectional analysis of variables. Up to 12% of asymptomatic children had pathological RI. Shorter acid clearance time was demonstrated in children with CP, and due to the difficulty in symptom expression in this population, we consider important to study these children regardless of the presence of symptoms.

## 218 BOWEL HABITS AND FUNCTIONAL CONSTIPATION IN HEALTHY CHILDREN ‐ A LONGITUDINAL BIRTH COHORT STUDY FROM SWEDEN

*Cathrine Gatzinsky* ^*1,2*^, *Ulla Sillén* ^*1,2*^, *Matilda Brautigam* ^*1,2*^, *Carola Kullberg‐Lindh* ^*3*^, *Sarah Thornberg* ^*1*^, *Sofia Sjostrom* ^*1,2*^

^*1*^ *Pediatric Surgery*, *Queen Silvia's Hospital for Children and Young People, Sahlgrenska universitetssjukhuset Drottning Silvias barn‐ och ungdomssjukhus, Goteborg, SE, hospital/children*, *Goteborg*, *Sweden*; ^*2*^ *Goteborgs universitet Institutionen for kliniska vetenskaper*, *Gothenburg*, *Västra Götaland County*, *Sweden*; ^*3*^ *Lerum*, *Pedaitric Outpatient Clinic*, *Lerum*, *Sweden*

**Aim:** During childhood functional constipation is common worldwide. Parents’ concern about the child's bowel habits is a common reason for seeking healthcare. We aim to report data on normal bowel habits, prevalence of functional constipation, long term outcome and search for early risk factors in children during the first 2.5 years in life.

**Methods:** This prospective longitudinal birth‐cohort study included 122 healthy term infants. Outpatient visits were undertaken at 2, 6, 12, 18 and 30 months of age with a medical history and a physical examination. Questionnaires were answered by their parents.

**Result:** Stool frequency declined with age and stabilized after 6 months. Stool consistencies changed, with increasing hard and very hard stools with age. Functional constipation was found in 22.1% and at last follow‐up 69.6% were still on medication. Relapse was seen in 26.1% during follow up. Risk factors at baseline or at 2 months of age that increased the odds of having functional constipation thereafter were not found. Breastfeeding at 2 weeks was protective the first 2,5 years of life.

**Conclusion:** We report data on normal bowel habits from birth to 2.5 years of age in healthy children. One fifth of the children were diagnosed with functional constipation and relapse was common. Endurance of treatment, repeated visits and long follow up is necessary since many children need medication for several years. Breastfeeding at 2 weeks of age was protective for being diagnosed with functional constipation the first 2,5 years of life.

## 219 LONG‐TERM SAFETY OF LINACLOTIDE IN TREATING PEDIATRIC PATIENTS AGED 7–17 YEARS WITH IRRITABLE BOWEL SYNDROME WITH CONSTIPATION (IBS‐C): INTERIM RESULTS FROM A PHASE 3 STUDY

*Miguel Saps* ^*1*^, *Samuel Nurko* ^*2*^, *Julie Khlevner* ^*3*^, *Gerardo Rodriguez‐Araujo* ^*4*^, *Wangang Xie* ^*4*^, *Darren Weissman* ^*4*^, *Valentina Shakhnovich* ^*5*^, *Steven S Wu* ^*5*^, *James Wu* ^*5*^, *Hannibal Person* ^*6*^, *David Jativa* ^*7*^, *Jeffrey Hyams* ^*8*^

^*1*^ *University of Miami Miller School of Medicine*, *Miami*, *FL*; ^*2*^ *Boston Children's Hospital*, *Boston*, *MA*; ^*3*^ *Department of Pediatrics*, *Columbia University Vagelos College of Physicians and Surgeons*, *New York*, *NY*; ^*4*^ *AbbVie Inc*, *North Chicago*, *IL*; ^*5*^ *Ironwood Pharmaceuticals Inc*, *Boston*, *MA*; ^*6*^ *Department of Pediatrics*, *University of Washington School of Medicine*, *Seattle*, *WA*; ^*7*^ *Internal Medicine*, *Mount Sinai Medical Center of Florida*, *Miami Beach*, *FL*; ^*8*^ *Connecticut Children's Medical Center*, *Hartford*, *CT*

**Background:** Irritable bowel syndrome (IBS) – predominantly the IBS with constipation (IBS‐C) subtype – is estimated to affect 6.2–11.9% of children and can substantially impact their quality of life. Despite this disease burden, there are no FDA‐approved pharmacologic treatments for pediatric IBS‐C and there is limited understanding of the long‐term safety of those used off‐label to manage symptoms. Linaclotide is approved for the treatment of IBS‐C and chronic idiopathic constipation in adults and functional constipation in children aged 6–17 years. We report long‐term safety from an ongoing study of linaclotide treatment in children with IBS‐C.

**Methods:** This ongoing Phase 3 safety study (NCT04166058) assessed once‐daily linaclotide treatment for 52 weeks in pediatric patients aged 7–17 years with IBS‐C. Patients weighing ≥18 kg who met modified Rome III criteria for pediatric IBS‐C and had completed the treatment period in studies NCT02559817 or NCT04026113 were eligible. NCT02559817 completers received open‐label linaclotide 290 μg, except for those who had received linaclotide ≤145 μg or placebo, who had the option to receive open‐label linaclotide 145 μg. NCT04026113 completers had the option to remain on the same blinded linaclotide dose or to receive open‐label linaclotide 290 μg. Safety assessments included the incidence of treatment‐emergent adverse events (TEAEs; primary endpoint) and adverse events of special interest (AESIs; significant volume depletion, significant electrolyte abnormalities, or electrocardiogram abnormalities considered related to diarrhea). Safety was assessed in enrolled patients who received ≥1 dose of study drug (safety population).

**Results:** In total, 98 patients with IBS‐C (linaclotide 145 μg, n=22; linaclotide 290 μg, n=76) were enrolled (median [min, max] age: 14.0 [7, 17] years). The majority of patients were female (61.2%) and White (71.4%; **Table 1**). Overall, 34 patients (34.7%) had TEAEs, and 10 patients (10.2%) had treatment‐related TEAEs. The incidence of diarrhea as a TEAE was low (n=6/98 [6.1%]). In the linaclotide 290 μg group, two patients (2.6%) had serious TEAEs not related to treatment. No AESIs were reported (**Table 2**). Of the 18 patients (18.4%) who discontinued treatment, ‘withdrawal by patient’ was the most common reason (n=11/18); no patients discontinued treatment owing to TEAEs.

**Conclusions:** In pediatric patients aged 7–17 years with IBS‐C, linaclotide was well tolerated, with findings consistent with the known safety profile. The incidence of diarrhea was low (6.1%) and was less than the reported rates from adult IBS‐C clinical studies with linaclotide.


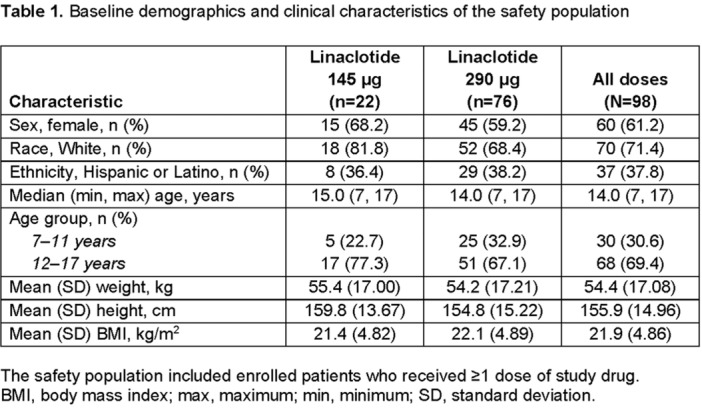



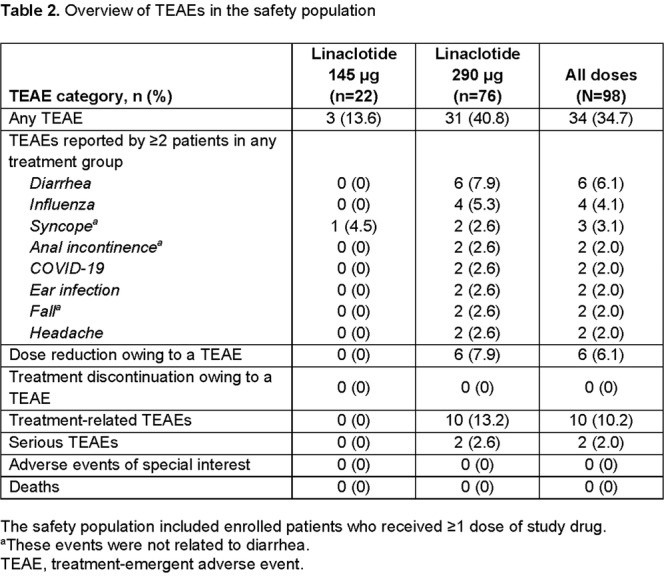


## 221 EFFICACY AND SAFETY OF LINACLOTIDE IN TREATING PEDIATRIC PATIENTS AGED 7–17 YEARS WITH IRRITABLE BOWEL SYNDROME WITH CONSTIPATION (IBS‐C): RESULTS FROM A PHASE 3 STUDY

*Jeffrey Hyams* ^*1*^, *Miguel Saps* ^*2*^, *Julie Khlevner* ^*3*^, *Gerardo Rodriguez‐Araujo* ^*4*^, *Wangang Xie* ^*4*^, *Valentina Shakhnovich* ^*5*^, *Steven S Wu* ^*5*^, *James Wu* ^*5*^, *Marcella E Hewson* ^*4*^, *Hannibal Person* ^*6*^, *David Jativa* ^*7*^, *Samuel Nurko* ^*8*^

^*1*^ *Connecticut Children's Medical Center*, *Hartford*, *CT*; ^*2*^ *University of Miami Miller School of Medicine*, *Miami*, *FL*; ^*3*^ *Department of Pediatrics*, *Columbia University Vagelos College of Physicians and Surgeons*, *New York*, *NY*; ^*4*^ *AbbVie Inc*, *North Chicago*, *IL*; ^*5*^ *Ironwood Pharmaceuticals Inc*, *Boston*, *MA*; ^*6*^ *Department of Pediatrics*, *University of Washington School of Medicine*, *Seattle*, *WA*; ^*7*^ *Internal Medicine*, *Mount Sinai Medical Center of Florida*, *Miami Beach*, *FL*; ^*8*^ *Boston Children's Hospital*, *Boston*, *MA*

No FDA‐approved pharmacologic therapies exist for pediatric irritable bowel syndrome with constipation (IBS‐C). Linaclotide (LIN) is FDA‐approved for functional constipation in children 6–17 yrs and adults with IBS‐C. We evaluated LIN efficacy and safety for pediatric IBS‐C in a multicenter, double‐blind, parallel‐group, Phase 3 study (NCT04026113).

Children aged 7–17 who met Rome III IBS‐C criteria were randomized to LIN 145 μg or 290 μg daily for 12 wks. The primary endpoint, based on twice‐daily eDiary entries, was the proportion of patients who achieved ≥30% reduction in abdominal pain and an increase of ≥2 spontaneous bowel movements (SBM)/wk from baseline (APS+2 responder) for ≥6/12 wks (6/12 wks APS+2 responder). Those with <4/7 days of twice‐daily eDiary entries/wk were considered APS+2 non‐responders for that week. In the absence of a placebo (PBO) arm, a meta‐analysis of adult IBS‐C studies using the same 6/12 wks APS+2 endpoint estimated a 16% PBO responder rate, with an 18% statistical superiority threshold (95% CI upper bound of the 16% PBO responder rate). Adult LIN data were used to construct a prior distribution for Bayesian analysis that assessed the posterior 2.5^th^ percentile against the statistical superiority threshold of 18%. Additional analyses evaluated impact of missing eDiary data on the primary endpoint. Secondary endpoints and safety were also assessed.

Of 100 patients in mITT who received ≥1 LIN dose (145 μg, n=53; 290 μg, n=47), 61% were female, 70% White, 68% non‐Hispanic/Latino; mean (SD) age was 12.6 (3.00) yrs. 6/12 wks APS+2 responder rates were 22.6% (145 μg) and 23.4% (290 μg), exceeding the adult PBO responder rate of 16%, but not the superiority threshold of 18% (**Table 1**). However, >1/3 of patients defaulted to 6/12 wks APS+2 non‐responder by missing the minimum required twice‐daily eDiary entries/wk. A prespecified supplemental analysis using a missing at random assumption showed the 95% CI lower bound of the 6/12 wks APS+2 responder rate was >18%, confirming statistical superiority (**Table 1**). Two *post‐hoc* Bayesian analyses also showed their posterior 2.5^th^ percentiles of 6/12 wks APS+2 responder rate >18% by leveraging more of the available data by using less stringent eDiary completion criteria (≥8/14 eDiary entries/wk or ≥4/7 daytime symptom entries/wk; **Table 1**). Both LIN doses improved SBM frequency, stool consistency and abdominal pain from baseline. All treatment‐emergent AEs were mild/moderate; none led to treatment discontinuation (**Table 2**).

In children with IBS‐C aged 7–17, LIN 145 μg or 290 μg improved symptoms from baseline with 6/12 wks APS+2 responder rates exceeding the estimated PBO rate, though statistical superiority was not met. Statistical superiority was met in supplemental analyses with less stringent missing eDiary data handling. LIN was well tolerated, aligning with the established safety profile.


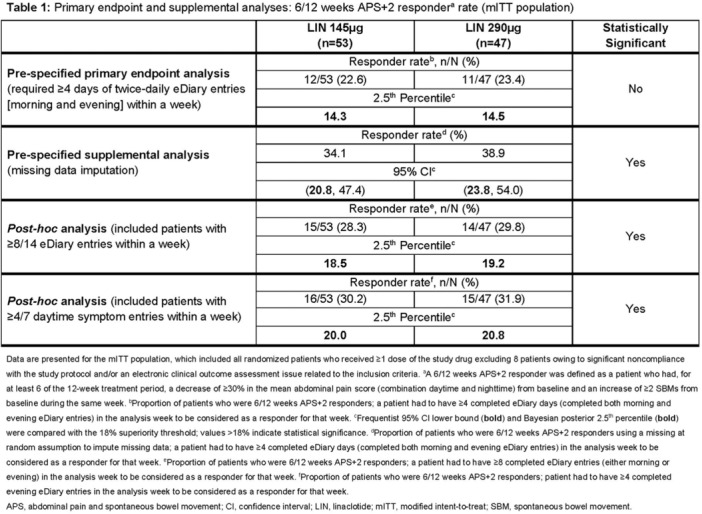



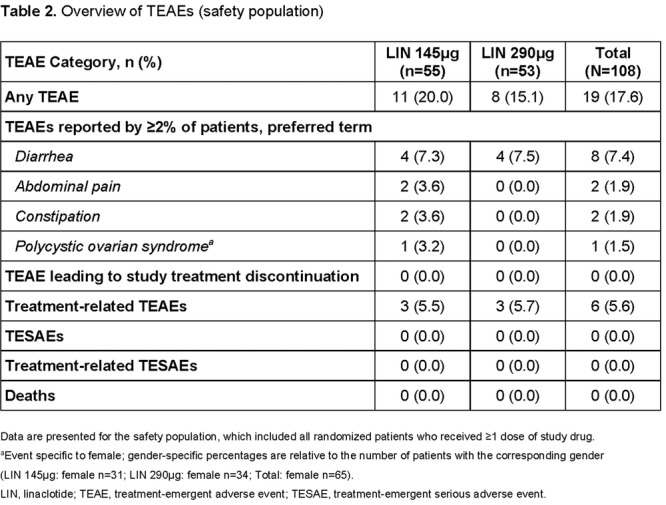


## 222 ANAL ENDOLUMINAL FUNCTIONAL IMAGING PROBE IN PATIENTS RECEIVING ANAL SPHINCTER BOTULINUM TOXIN

*Laura Hamant* ^*1*^, *Carlo Di Lorenzo* ^*1*^, *Peter Lu* ^*1*^, *Raul Sanchez* ^*1*^, *Neetu Bali Puri* ^*1*^, *Md‐Rejuan Haque* ^*2*^, *Desale Yacob* ^*1*^, *Richard Wood* ^*3*^, *Karla Vaz* ^*1*^

^*1*^ *Pediatric Gastroenterology*, *Nationwide Children's Hospital*, *Columbus*, *OH*; ^*2*^ *Center for Biostatistics*, *The Ohio State University*, *Columbus*, *OH*; ^*3*^ *Center for Colorectal & Pelvic Reconstruction*, *Nationwide Children's Hospital*, *Columbus*, *OH*

**Background:** Endoluminal functional luminal imaging probe (EndoFLIP) testing has been used to measure luminal diameter and distensibility primarily in the esophagus. Distensibility (DI) has been proposed as a primary determinant of anal canal function. Botulinum toxin weakens both smooth and striated muscle in a focal and transient fashion, and internal anal sphincter botulinum toxin injection has been shown to be safe and effective in children with constipation. We hypothesize that lower anal sphincter DI correlates with increased constipation severity and that patients with lower DI are more likely to respond to anal sphincter botulinum toxin injection. Our objective is therefore to use EndoFLIP to measure DI of the anal sphincter and evaluate for correlation with constipation severity and clinical response to anal sphincter botulinum toxin injection.

**Methods:** We performed a single‐center prospective cohort study at a tertiary children's hospital. We included patients (<21 years of age) who were scheduled to receive anal sphincter botulinum toxin injection. Anal EndoFLIP was performed under anesthesia before anal sphincter botulinum toxin injection. Probability of moderate or severe constipation as measured by the Columbus Pediatric Constipation Score (CPCS) was obtained at baseline, 2 days, 2 weeks, and 12 weeks post‐procedure, with higher scores indicating more severe constipation. We defined treatment response as CPCS<0.3. We used descriptive statistics, including median and interquartile range (IQR) for continuous variables, and frequencies and percentages for categorical variables, to characterize the baseline characteristics of the patient population. Two sample t‐tests and a Chi‐square test were used to assess the association between continuous and categorical variables with the presence or absence of Hirschsprung disease. Wilcoxon rank sum was used to compare CPCS to DI and diameter.

**Results:** We enrolled 33 patients (39% female, median age 4.7 years). Underlying diagnoses included functional constipation in 21 (64%), Hirschsprung disease (HD) in 9 (27%), and internal anal sphincter achalasia in 3 (9%). All 9 children with HD had a prior transanal pull‐through surgery, and none of the other patients had a history of rectal surgery. As shown in **Table 1,** the HD group had a smaller anal canal diameter (p<0.001) and lower DI (p<0.001) than the non‐HD group. The HD group had overall lower CPCS than the non‐HD group (difference ‐0.055) after controlling for time point and DI, but this difference was not statistically significant. As shown in **Figure 1**, CPCS decreased over time for both groups, reaching statistical significance at 2 and 12 weeks. Response to anal sphincter botulinum toxin injection was not associated with baseline anal canal diameter or DI at any time point. In addition, there was no correlation between diameter or DI and CPCS.

**Conclusions:** In the first prospective study of anal canal diameter and DI in children with constipation, we found that patients with post‐surgical HD had a smaller anal canal diameter and lower DI compared to patients with functional constipation or internal anal sphincter achalasia. This is consistent with our understanding of their underlying disease process and post‐surgical physiology. Patients experienced improvement in constipation severity after anal sphincter botulinum toxin injection. However, response was not associated with baseline anal canal diameter or DI. Our findings suggest that anal sphincter botulinum toxin injection should be considered for patients with refractory constipation regardless of anal canal diameter or DI.


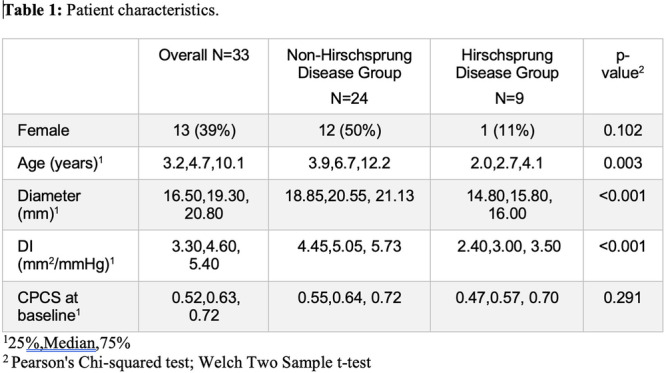



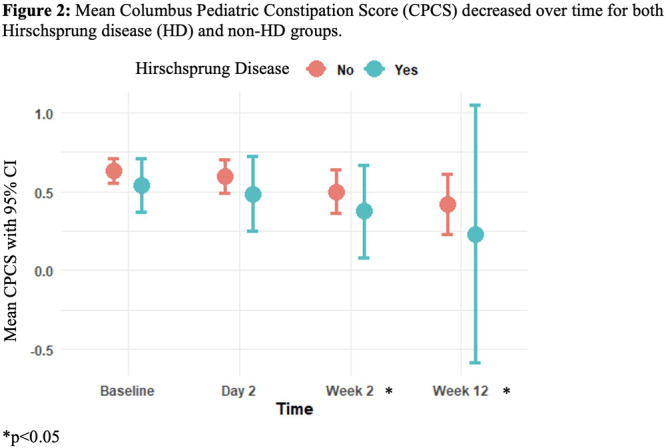


## 223 INTRAPYLORIC BOTOX INJECTION IMPROVES SYMPTOMS FOR PEDIATRIC PATIENTS WITH GASTROPARESIS

*Binghong Xu* ^*1*^, *Carolyn Orians* ^*1*^, *Christian Sadaka* ^*1*^, *Lexi Roshkovan* ^*1*^, *Tara Mcwilliams* ^*2*^, *Gayl Humphrey* ^*3,4*^, *Armen Gharibans* ^*3,4*^, *Alain Benitez* ^*1,2*^, *Hayat Mousa* ^*1,2*^

^*1*^ *The Children's Hospital of Philadelphia*, *Philadelphia*, *PA*; ^*2*^ *University of Pennsylvania Perelman School of Medicine*, *Philadelphia*, *PA*; ^*3*^ *The University of Auckland*, *Auckland*, *New Zealand*; ^*4*^ *Alimetry Ltd*, *Auckland*, *New Zealand*

**Background:** Body surface gastric mapping (BSGM) (Alimetry, Ltd) is an FDA‐approved noninvasive diagnostic tool for measuring gastric myoelectrical activity with real‐time symptom association for individuals aged 12 years and older. Intrapyloric Botox (IPB) injection has been used to treat gastroparesis in adults and pediatrics. This study aims to evaluate the effectiveness of IPB for pediatric gastroparesis via BSGM measurement, real‐time logging, and validated questionnaires.

**Methods:** Through an IRB‐approved protocol, the BSGM pediatric study at the Children's Hospital of Philadelphia enrolled patients aged 8‐25 years old with confirmed delayed gastric emptying. Patients scheduled for clinical IPB injection were invited for BSGM studies before and after the injection, with post‐IPB BSGM scheduled 2‐4 weeks after the injection. Baseline BSGM was done any day prior to IPB, but at least 3 months after the previous injection for those receiving recurrent treatment. All prokinetics were discontinued before the BSGM study. Participants fasted for 6 hours, starting with 30 minutes of pre‐prandial BSGM monitoring, 10 minutes consuming a standard meal, followed by 4 hours of post‐prandial monitoring. Real‐time symptom logging for upper gut pain, nausea, bloating, heartburn, stomach burn, and excessively full was captured every 15 minutes. Validated questionnaires were administered during the study visit, including Gastroparesis Cardinal Symptom Index Daily Diary (GCSI‐DD), Abdominal Pain Index (API), Nausea Severity Scale (NSS), Functional Disability Inventory (FDI), PedsQL GI Symptom Module and Quality of Life, PROMIS‐25. BSGM metrics (Principal Gastric Frequency (PGF), BMI‐Adjusted Amplitude, Gastric Alimetry Rhythm Index (GA‐RI), fed:fasted amplitude ratio (ffar)) and real‐time symptom burden were analyzed and reported by Alimetry. We used Generalized Estimating Equation (GEE) models to assess changes in questionnaire and BSGM‐derived scores between pre and post IPB injection, accounting for the correlation introduced by repeated measurements from the same subjects across multiple injections.

**Results:** We collected 14 sets of pre and post IPB injection BSGM studies from 6 participants. Age 12‐ 22 years old, 5 females (83.3%), BMI mean (SD) is 20.61 (1.87). Four hours of emptying rate from Gastric emptying studies (GES) ranges from 50% to 81%, one patient only had 2‐hour GES completed with 35% emptied at 2 hours. Two participants received IPB every 3‐5 months to manage gastroparesis symptoms.

**BSGM Metrics Monitoring:** After IPB injection, overall BMI‐Adjusted Amplitude decreased by 7.26 uV, PGF increased slightly by 0.04 cpm, GA‐RI decreased by 0.06, and ffar decreased by 0.14.

**BSGM Study Real‐time Symptom Burden:** Post IPB injection, improvements were noted in all real‐time symptoms, with excessive fullness showing the most improvement and upper gut pain the least.

**Validated Questionnaires:** After IPB injection, NSS overall score decreased by 0.12, API decreased by 0.26, FDI decreased by 0.23. PedsQL GI Module increased by 4.1, and Quality of Life Core increased by 1.38. PROMIS Physical Function Mobility, Fatigue, Pain Interference showed improvement; however, Anxiety, Depressive Symptoms, and Peer Relationships deteriorated. GCSI‐DD was added later in the study, collected 4 sets of pre and post IPB comparison, with the mean decreasing by 0.95 (p<0.01).

**Conclusion:** Both real‐time symptom logging during the BSGM study and data from validated questionnaires consistently demonstrated improvements in GI symptoms and quality of life among pediatric patients with gastroparesis receiving IPB injection, which suggests that IPB injections represent a viable therapeutic option for managing gastroparesis in children. Notably, the BSGM effectively captured a decrease in gastric myoelectrical amplitude, highlighting its utility in monitoring physiological changes.


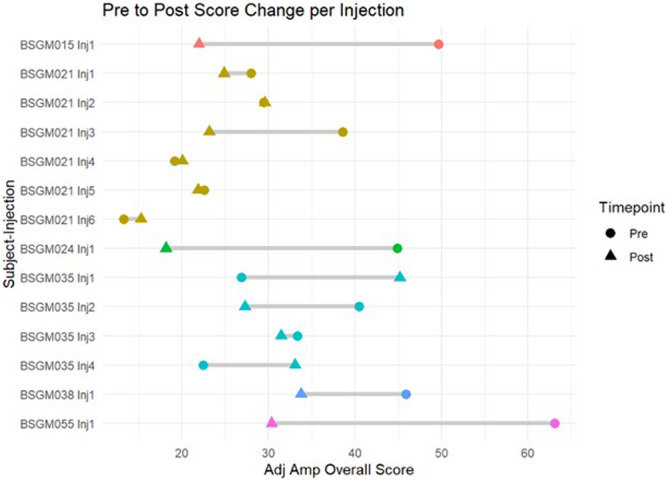


Figure 1. BMI‐Adjusted Amplitude changed captured by BSGM

## 224 EXPLORE THE ASSOCIATION BETWEEN GASTRIC EMPTYING STUDY & BODY SURFACE GASTRIC MAPPING IN PEDIATRICS

*Binghong Xu* ^*1*^, *Christian Sadaka* ^*1*^, *Lexi Roshkovan* ^*1*^, *Carolyn Orians* ^*1*^, *Alain Benitez* ^*1,2*^, *Gayl Humphrey* ^*3,4*^, *Armen Gharibans* ^*3,4*^, *Hayat Mousa* ^*1,2*^

^*1*^ *The Children's Hospital of Philadelphia*, *Philadelphia*, *PA*; ^*2*^ *University of Pennsylvania Perelman School of Medicine*, *Philadelphia*, *PA*; ^*3*^ *The University of Auckland*, *Auckland*, *New Zealand*; ^*4*^ *Alimetry Ltd*, *Auckland*, *New Zealand*

**Background:** Body surface gastric mapping (BSGM) (Alimetry, Ltd) is an FDA‐approved diagnostic technique for measuring bowel myoelectrical activities combining with real‐time patient‐reported symptoms for individuals aged 12 years and older. Studies have shown BSGM provides highly reproducible metrics over short (1 week) and long (>6 months) periods in gastric patients (Law et al., 2024). Gastric Emptying Study (GES) is a gold standard diagnostic test for gastric motility. A study compared BSGM with GES in adults with chronic gastroduodenal symptoms indicating that BSGM improves patient phenotyping and benefits individualized management (Wang et al., 2024). This study aims to explore the correlation between BSGM and GES in children with gastric functional disorders.

**Methods:** Through an IRB‐approved protocol, the BSGM pediatric study at the Children's Hospital of Philadelphia enrolled patients aged 8‐25 years old with functional gastrointestinal (GI) disorders confirmed via ROME IV criteria. Medical chart review was conducted, and any GES occurring within 180 days of the BSGM study visit were included in this analysis. BSGM measurements comprised 30 minutes of pre‐prandial monitoring, 10 minutes consuming a standard meal, followed by 4 hours of post‐prandial monitoring. BSGM metrics (Principal Gastric Frequency (PGF), BMI‐Adjusted Amplitude, Gastric Alimetry Rhythm Index (GA‐RI), fed:fasted amplitude ratio (ffar)) and real‐time symptom burden were analyzed and reported by Alimetry. GES 2 or 4 hours with a solid meal protocol were included; the emptied rate was reviewed and reported every 30 minutes by the Radiology department. Variables were reported as mean with standard deviation (SD) or median and interquartile range (IQR) based on normality. Pearson's correlation was employed for correlation analysis.

**Results:** Thirty‐eight pairs of GES & BSGM within 180 days were included. Participants' mean (SD) age was 15.6 (3.0) years old, 87.5% female, 96.9% non‐Hispanic, and 87.5% White. The mean (SD) weight was 53.8 (10.1) kg, BMI median (IQR) was 19.8 (4.7). The interval between BSGM and GES ranged from ‐163 to 180 days, with an absolute mean of 76.4 days. Due to the variability of GES reports, 8 GES didn't report 30 min emptied rate, 2 studies missed 60 min rate, 11 studies missed 90 min rate, and 3 studies missed 120 min rate.

The emptied rates from GES didn't show strong correlations with any BSGM metrics, however, moderate negative correlations were found between 60 and 90 min emptied rate with PGF, R values were ‐0.50 and ‐0.54 (both p<0.01). Further exploration of correlations between 60 and 90 min emptied rate and post‐prandial 2^nd^ hour PGF revealed that R decreased and was no longer significant.

**Conclusion:** This exploratory analysis indicates minimal associations exist between BSGM and GES in children with upper functional GI disorders. BSGM Principal Gastric Frequency shows a moderate relationship with the rate of stomach contents emptied at 60 and 90 minutes. The instability of GES may contribute to the inconsistent correlations observed. A larger sample size is needed for confirmation. Additionally, establishing BSGM normative value for the pediatric cohort could provide further insights, offering another analysis perspective that, potentially, may enhance individualized management strategies for pediatric upper functional GI disorders.


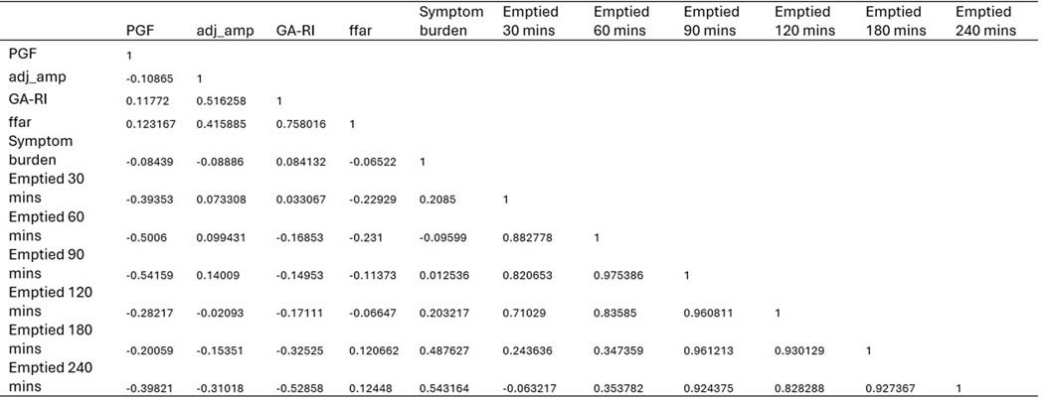


Table 1. Correlation Matrix for BSGM Metrics and GES Results

## 225 MEDICAL EXPERIENCES AND NEEDS IN VISCERAL MYOPATHY SYNDROMES: A PATIENT‐REPORTED COHORT STUDY THROUGH THE MMIHS FOUNDATION

*Sharon Wolfson* ^*3,1*^, *Sophie Sax* ^*1*^, *Kelly Rodriguez* ^*2*^, *Jessica Richards* ^*4*^, *Katherine Beigel* ^*3*^, *Robert Heuckeroth* ^*3,1*^

^*1*^ *Department of Pediatrics*, *University of Pennsylvania Perelman School of Medicine*, *Philadelphia*, *PA*; ^*2*^ *Department of Psychiatry*, *University of Pennsylvania Perelman School of Medicine*, *Philadelphia*, *PA*; ^*3*^ *The Children's Hospital of Philadelphia*, *Philadelphia*, *PA*; ^*4*^ *Washington University in St Louis School of Medicine*, *St. Louis*, *MO*

**Background:** Visceral myopathy syndromes represent rare but life‐threatening causes of pediatric intestinal pseudo‐obstruction (PIPO). These disorders result from profoundly impaired intestinal smooth muscle contractility, often due to mutations in genes such as *ACTG2*, which encodes intestinal smooth muscle actin. The goal of this study was to explore the medical experiences of affected individuals and families, with the aim of identifying patterns in care delivery, burdens, and unmet needs.

**Methods:** This was a prospective, cross‐sectional survey conducted in collaboration with the MMIHS Foundation. Exempt IRB approval was obtained through Children's Hospital of Philadelphia. The survey, distributed internationally from February to April 2025, included 190 questions on diagnoses, medical history, and therapies, and 38 questions on social and economic impact. Responses were de‐identified and analyzed using descriptive statistics and thematic analysis of open‐ended responses.

**Results:** Of 76 individuals who consented, 60 completed baseline medical information, 53 completed the medical history section, and 49 completed the socioeconomic survey. Respondents included caregivers of affected children (55/60), affected adults (5/60), and bereaved parents (3/60). Participants were from four countries, predominantly the United States (54/60).

• Genetic diagnoses were reported in 47/60 cases: 46 with *ACTG2* mutations (R257C most common, in 16/45) and one with an *MYH11* mutation.

• Symptom onset occurred before 3 months of age in 77% (46/60).

• Participants received care at 48 different institutions.

• Prenatal bladder ultrasound anomalies were noted in 41% (23/56), and 50% (28/56) were born prematurely. NICU care was required in 79% (44/56), often for prolonged durations.

• Nearly all (55/56) underwent an abdominal surgery; six underwent small bowel transplantation, four of whom received multi‐visceral transplants due to TPN‐associated liver failure.

• Most (45/56) had a current stoma.

• Nutritional support included enteral feeding in 75% (42/56) and parenteral nutrition in 80% (43/54); 63% (34/54) required IV fluid supplementation.

• In the past 12 months, 72% (39/54) experienced at least one hospitalization. Strategies cited as reducing hospitalizations included access to home IV hydration, ability to obtain urgent labs, and ethanol lock use.

• Common triggers for pseudo‐obstruction flares included infection (32/53), stress, medications, immunizations, and air travel.

• The most frequently trialed promotility medications included erythromycin (30/52), Augmentin (27/52), prucalopride (20/52), and pyridostigmine (12/52), with reported benefit in 74% for Augmentin, 80% for prucalopride, and 83% for pyridostigmine.

• Some (16/49) respondents reported relocating due to the condition, primarily for proximity to hospitals (7), access to better medical care (7), family support (2), and insurance (2).

• Financial strain was reported by most(38/49) families, with 31 noting career impacts and 36 reporting schooling disruptions. Psychosocial concerns, including medical trauma, were widely described.

**Conclusions:** This study represents the largest patient‐reported cohort of individuals with visceral myopathy syndromes to date. The findings underscore the medical complexity, surgical burden, and psychosocial challenges faced by these patients and families. Marked variability in care and access to services highlights the urgent need for multidisciplinary care models and targeted supports, particularly in GI motility, general surgery, nutrition, and behavioral health. These data call for increased collaboration and awareness to improve outcomes for this vulnerable and underrecognized population.


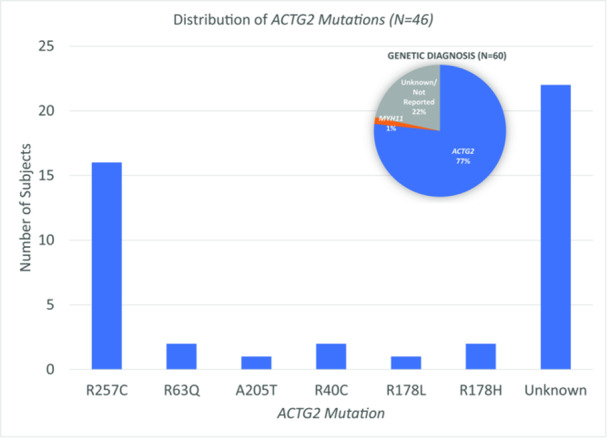


Figure 1. Bar chart showing distribution of *ACTG2* mutations among genetically confirmed patients (n=46), with a pie chart inset showing proportions of genetic diagnoses.

## 226 THE IMPACT OF SLEEP DISTURBANCE ON THE RELATIONSHIP BETWEEN ADVERSE CHILDHOOD EXPERIENCE AND IRRITABLE BOWEL SYNDROME

*Michelle Binod* ^*1,2*^, *Ming Hung* ^*2,3*^, *Tien Dong* ^*2,3*^, *Arpana Church* ^*2,3*^

^*1*^ *Division of Pediatric Gastroenterology*, *University of California Los Angeles David Geffen School of Medicine*, *Los Angeles*, *CA*; ^*2*^ *Goodman‐Luskin Microbiome Center*, *University of California Los Angeles*, *Los Angeles*, *CA*; ^*3*^ *Vatche and Tamar Manoukian Division of Digestive Diseases*, *University of California Los Angeles*, *Los Angeles*, *CA*

**Introduction:** Adverse Childhood Experiences (ACEs) are associated with an increased risk of sleep disturbances in adulthood, including insomnia, short sleep duration, and poor sleep quality. Meta‐analyses show that even a single ACE increases the likelihood of adult sleep problems (OR = 1.14), with higher risk in those with multiple ACEs (OR = 1.33). Sleep disturbances are also common in individuals with irritable bowel syndrome (IBS), affecting 37.6% of patients (OR = 2.6). Chronic sleep loss dysregulates the hypothalamic‐pituitary‐adrenal (HPA) axis and elevates cortisol, amplifying visceral pain sensitivity. Given these overlapping mechanisms, we examined whether sleep disturbances mediate or moderate the relationship between ACEs and IBS.

**Methods:** We analyzed data from 276 adult participants. The PROMIS Sleep Disturbance scale, assessed perceived sleep quality over the past week (T‐score mean = 50, SD = 10; higher scores = greater sleep disturbance). ACE scores (mean = 1.19, median = 1) were derived from the standardized ACE questionnaire. IBS status was binary (presence vs. absence). Missing data were addressed using k‐nearest neighbors (KNN) imputation. We performed two analyses: (1) mediation analysis to test if sleep disturbance mediates the association between ACE and IBS, and (2) moderation analysis to assess if sleep modifies the strength of the ACE‐IBS relationship. Mediation was assessed using linear regression and the mediate package in R. Moderation was tested using logistic regression with an ACE‐by‐sleep interaction term.

**Results:**

**Mediation analysis:** KNN imputation revealed a significant total effect of ACE on IBS (coefficient = 0.077, p = 5.07 x 10^‐4^). ACE was significantly associated with sleep disturbance (coefficient = 0.666, p = 9.88 x 10^‐3^). When both ACE and sleep were included in the model, the ACE coefficient decreased from 0.077 (total effect) to 0.068 (direct effect). Mediation accounted for 11.48% of the total effect with statistically significant Average Causal Mediation Effect (ACME; p = 8.0 x 10^‐3^) and Average Direct Effect (ADE, p = 4.0 x 10^‐3^), supporting partial mediation.

**Moderation analysis:** In logistic regression model with KNN‐imputed data, the ACE‐by‐sleep interaction term was significant (β = 0.053, p = 1.25 x 10^‐2^), indicating that the strength of the ACE‐IBS association increases with higher levels of sleep disturbance. Predicted probability plots (Figure 1) revealed a steeper ACE‐IBS slope at high sleep disturbances (+1 SD) compared to low (‐1 SD).

**Discussion:** Sleep disturbance plays a dual role in the ACE‐IBS relationship, functioning both as a mediator and a moderator. Mediation analysis supports a partial indirect pathway through sleep disturbance, while moderation analysis shows that the ACE‐IBS association is stronger at higher levels of sleep disruption. The use of a validated sleep assessment tool (PROMIS) and robust imputation methods strengthens the reliability of these findings. Our results align with prior research linking poor sleep to chronic pain, dysregulated stress response, and gastrointestinal dysmotility, underscoring the central role of sleep within the gut‐brain axis, particularly in those with early life adversity.

**Conclusion:** Sleep disturbance is a key mechanistic and modifying factor in the ACE‐IBS relationship. These results highlight the importance of assessing and addressing sleep quality in individuals with early life adversity, especially those presenting with IBS. Interventions aimed at improving sleep may represent a promising strategy for reducing the long‐term gastrointestinal burden associated with early life adversity.


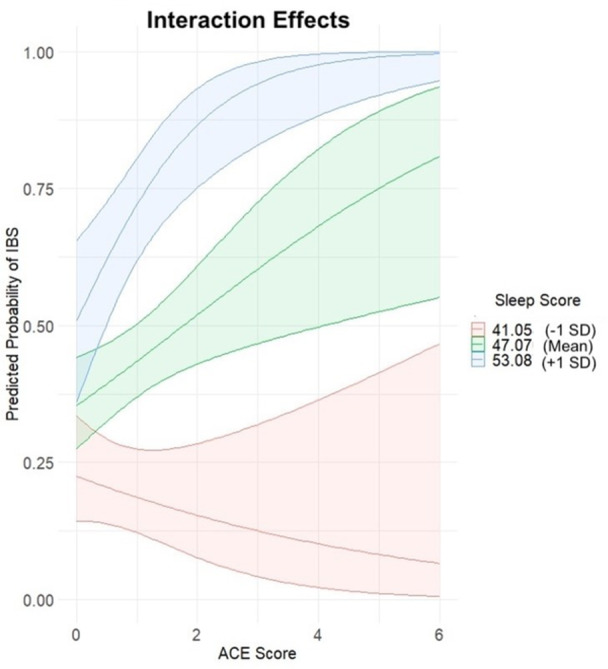


**Figure 1: Predicted probability plots with KNN imputated data highlighting the interaction effect of ACE and IBS at 3 different sleep disturbance levels.**

## 227 CHARACTERIZATION AND OUTCOMES OF FUNCTIONAL LUMEN IMAGING PROBE IN CHILDREN WITH ESOPHAGEAL ATRESIA‐TRACHEOESOPHAGEAL FISTULA

*Trevor Davis* ^*1*^, *Neetu Bali Puri* ^*2*^, *Raul Sanchez* ^*2*^

^*1*^ *Pediatric Gastroenterology*, *Washington University School of Medicine*, *St. Louis*, *MO*; ^*2*^ *Pediatric Gastroenterology*, *Nationwide Children's Hospital*, *Columbus*, *OH*

**Background:** Recurrent esophageal symptoms, primarily dysphagia, are common in children with a history of esophageal atresia (EA) and tracheoesophageal fistula (TEF). While high‐resolution esophageal manometry (HRM) is the gold standard in assessment of esophageal motility disorders, tolerance and cooperation may limit interpretability in pediatrics. More recently, esophageal functional lumen imaging probe (FLIP) has become available to assess metrics and contractility in response to volumetric distension during sedated esophagoscopy. It is also known that EA/TEF patients are at risk for gastroesophageal reflux (GER) and esophagitis. We aimed to characterize FLIP findings in symptomatic children with EA/TEF in association with pathologic GER, and determine how findings influence subsequent pharmacologic and endoscopic management.

**Methods:** In this retrospective cohort study at a large volume pediatric referral center, patients ≤23 years old with EA/TEF who underwent FLIP for esophageal symptoms between 2022‐2024 calendar years were identified for inclusion. Patient characteristics and adjunctive esophageal tests were collected from the electronic medical record. FLIP measurements, including esophageal diameter, distensibility index (DI), intra‐bag pressure, and contractile response (CR), were collected from procedural notes and confirmed by review of intra‐procedural FLIP topography files. Findings on FLIP were compared between patients with and without histologic evidence of esophagitis.

**Results:** Of 17 children, median age was 10.6 years (IQR 4.4‐14.7) with 9 (52.9%) males. A majority of patients had type C EA/TEF (14, 82.4%), while the remainder were type A (3, 17.6%). Primary indication for FLIP was dysphagia in 15 (88.2%), persistent heartburn in 1 (5.9%), and recent brief response unexplained event in 1 (5.9%). Median duration of symptoms was 12 months (IQR 2‐20). Most patients were eating solely by mouth (15, 88.2%), while the remainder were receiving supplemental gastric tube feeds. Acid suppression was utilized in 82% (14), and 1 patient (5.9%) was receiving bethanechol. Eight patients (47.1%) had histologic evidence of esophagitis.

Three patients underwent pH/multichannel intraluminal impedance (pH/MII) all with normal acid exposure time. Three patients underwent HRM all with absent contractility; however, one also had features concerning for type I achalasia.

Normal CR was observed in 3 patients (17.6%), while the remainder had a diminished (2, 11.8%) or absent (12, 70.6%) response. Management based on FLIP findings included esophageal dilation (9, 53.0%), change in medication regimen (4, 24.0%), and myotomy (1, 5.9%). For patients with histologic evidence of esophagitis in comparison to those without, diameter assessed by FLIP at the site of anastomosis was significantly larger at a max volume of 50 mL (15.1 mm vs 10.7 mm; p=0.036). Conversely, there was no significant difference in DI between the groups regardless of intra‐bag volume at the site of anastomosis (p>0.999) or esophagogastric junction [EGJ] (p=0.133). There was no difference in CR between the groups (p>0.999). For patients with esophagitis, management trended away from dilation in comparison to those without (p=0.091). For patients with normal CR, DI at the EGJ was significantly higher in comparison to those with abnormal CR up to a max volume of 50 mL (9.8mm^2^/mmHg vs 2.35mm^2^/mmHg; p=0.044). Five patients (36%) with abnormal CR had evidence of esophagitis compared to no patient (0.0%) with normal CR (p=0.593).

**Conclusion:** In symptomatic patients with EA/TEF, FLIP provided useful real‐time data to direct clinical management. Patients with esophagitis were more likely to have a larger anastomotic diameter in comparison to those without, suggesting a protective advantage of variation in anastomotic caliber. In patients with an abnormal CR, reduced EGJ distensibility may reflect a protective advantage in preventing retrograde movement of gastric contents.

## 236 OVERLAP OF RUMINATION WITH ABDOMINAL PAIN RELATED DGBIS IN SUBSPECIALTY PRACTICE: IMPLICATIONS FOR CONCEPTUALIZATION AND CARE

*Jennifer Schurman* ^*1,2*^, *Craig Friesen* ^*1,2*^

^*1*^ *Division of Gastroenterology, Hepatology, & Nutrition*, *Children's Mercy Kansas City Division of Pediatric Gastroenterology*, *Kansas City*, *MO*; ^*2*^ *University of Missouri‐Kansas City School of Medicine*, *Kansas City*, *MO*

Rumination Syndrome (RS) is a Disorder of Gut‐Brain Interaction (DGBI) in which stomach contents are regurgitated repeatedly in small volumes with re‐swallowing or spitting out. RS has long been recognized in infants and children/teens with developmental and/or neurodevelopmental differences but has more recently been recognized as occurring in typically developing children/teens. Prevalence estimates in community samples range from 0‐9.7% in children/teens (Martinez et al. 2021), with prevalence much higher in pediatric patients with treatment‐refractory gastroesophageal reflux disease (44%) and chronic vomiting (60%; Nikaki et al. 2020; Malik et al. 2020). This suggests that RS may not be easily distinguished from other GI conditions and/or may co‐occur with them. Understanding the relationship between RS and other DGBIs that present in clinical practice is likely to be important in selecting and prioritizing treatment targets, as well as in assessing treatment effectiveness.

**Method:** This study describes the experience of a single subspecialty clinic for pediatric DGBIs in the Midwest in which patients (8‐17 years) can be referred for a primary concern of abdominal pain (AP) or rumination (RUM). A total of 383 sequential referrals were retrospectively reviewed with IRB approval. As standard of care at initial evaluation, patients provided information on: 1) primary referral concern; 2) presence of abdominal pain (Abdominal Pain Inventory; API), nausea (Nausea Severity Scale; NSS), and regurgitation (a Rumination Severity Scale created by the current authors to parallel the API and NSS; RSS) in the past 2 weeks; 3) presence of meal‐related early satiety, bloating, nausea, and pain; 4) their 3 worst/most bothersome symptoms; and, 5) health‐related quality of life (HRQoL; PedsQL Generic Core Scales).

**Results:** The primary referral complaint was abdominal pain in 89% (n=342; AP) and rumination in 11% (n=41; RUM). Across both groups, 16% reported only 1 of these 3 symptoms, 39% reported 2 symptoms, and 44% reported all 3 symptoms as present in the past 2 weeks. To better understand this overlap, the AP group was further subdivided based on presence (AP+; n=168) or absence (AP‐; n=174) of regurgitation as a secondary symptom. Statistical comparison of the 3 groups (AB‐, AB+, and RUM) yielded significant differences on presence of abdominal pain and nausea in the past 2 weeks, severity of pain, nausea, and regurgitation in the past 2 weeks, presence of specific meal‐related symptoms, and overall health‐related quality of life (see Table 1). When asked to name the 3 most bothersome symptoms, AP‐ identified “abdominal pain in general” as the worst symptom most often (66%), followed by nausea (12%). AP+ also identified “abdominal pain in general” as the worst symptom most often (58%), followed by nausea (15%). Finally, RUM identified “vomiting” as the worst symptom most often (40%), followed by “abdominal pain in general” (20%), and nausea (17%).

**Conclusion:** Results suggest significant overlap in the hallmark symptoms of abdominal pain, nausea, and regurgitation for pediatric patients referred for abdominal pain versus rumination. Across all groups, meal‐related symptoms of pain, nausea, bloating, and early satiety also were commonly reported and average scores were consistent with impaired health‐related quality of life (all group means placing below cutoff score of 78). Overlap among symptoms and differences in reporting on “worst symptom” suggest that the primary reason for referral may better reflect the symptom deemed most bothersome by the patient rather than true differences in diagnosis. Further, results suggest that evaluation for abdominal pain, nausea, and meal‐related symptoms may be appropriate in patients referred for rumination, and vice versa, given that patients with a combination of these symptoms are common and at greater risk for more severe symptoms and impairment in quality of life. For the small number of patients with regurgitation only (~13%, or n=4, of those referred for rumination in our sample), a direct referral to a behavioral health provider to work on diaphragmatic breathing and habit reversal may be appropriate once screened negative for co‐morbid symptoms. More research is needed to determine how best to prioritize and optimize treatment for pediatric patients presenting with a combination of abdominal pain, nausea, and regurgitation, irrespective of the presenting complaint, to improve both clinical outcomes.


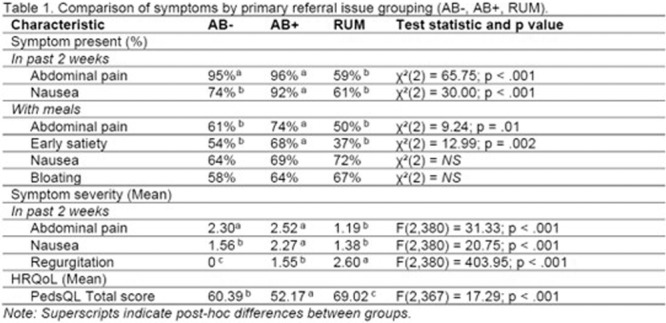


## 237 TREATMENT WITH THE PERCUTANEOUS ELECTRICAL NERVE FIELD STIMULATOR (PENFS) IMPROVES OTHER SYMPTOMS IN ADOLESCENTS WITH FUNCTIONAL ABDOMINAL PAIN OR FUNCTIONAL NAUSEA

*Sydney Kuzoian* ^*3*^, *Christine Brutus* ^*2*^, *Corey Baker* ^*1*^

^*1*^ *Gastroenterology*, *Connecticut Children's Medical Center*, *Hartford*, *CT*; ^*2*^ *Quinnipiac University*, *Hamden*, *CT*; ^*3*^ *University of Connecticut School of Medicine*, *Farmington*, *CT*

**Background:** The Percutaneous Electrical Nerve Field Stimulator (PENFS), also known as IB‐Stim, is an FDA approved treatment for 8‐21 year olds with functional abdominal pain, functional nausea, and functional dyspepsia. However, many pediatric patients who undergo treatment with IB‐Stim have many additional comorbid symptoms and diagnoses. We therefore sought to characterize these symptoms in this population while also identifying whether or not treatment with IB‐Stim improved these other symptoms.

**Methods:** This is a single center prospective survey‐based quality improvement study. All patients who received treatment with IB‐Stim for functional abdominal pain or functional nausea at Connecticut Children's, a free‐standing tertiary pediatric center, were asked to complete the same survey each week of treatment. The survey asked about the presence, duration, and severity of a variety of symptoms. Severity of symptoms were reported on a Likert scale with 1 being the least severe, 10 being the most. The symptoms questioned included reflux/vomiting, abdominal distention, decreased appetite, trouble swallowing, noise and/or light sensitivity, headaches, heart palpitations, shortness of breath, dizziness, generalized muscle weakness, frequent fatigue, frequent dehydration, frequent joint pain, and joint hypermobility. Answers to these weekly surveys were tracked longitudinally for each patient. Patients who did not receive the standard protocol of four weekly consecutive IB‐Stim treatments or who did not fill out a single survey were excluded from the study. Additionally, only patient survey answers from the first 4‐week IB‐Stim treatment were included. Demographic information of patients at presentation of at their first IB‐Stim treatment was characterized. Improvement of symptoms were defined as any reported decrease of severity score in comparison to the severity score reported at initial presentation. Symptoms that were not reported at initial presentation were not included. A retrospective manual chart review was then performed to confirm whether or not patients reported that treatment with IB‐Stim improved their primary symptom of abdominal pain or nausea. Percentage of patients who had improvement in other symptoms were compared between those who reported improvement in their primary symptom (abdominal pain or nausea) with IB‐Stim versus those who did not.

**Results:** A total of 25 patients met inclusion criteria. The average age at initial presentation was 16.7 years, 80% were female. The primary symptom reported for IB‐Stim use was abdominal pain (68%), the remaining were nausea (32%). The average number of therapies tried for their primary symptom prior to treatment with IB‐Stim was 9.92 (range 5‐20). On average, patients had 2.96 comorbidities at presentation, with the most common being anxiety (48%), depression (28%), AMPS (24%), POTs/dysautonomia (20%), and migraine headaches (20%). 14 patients (56%) verbally reported improvement in their primary symptom at the end of the treatment program (44% (n=9) no, 8% (n=2) unknown). The 5 most common other symptoms reported at presentation were frequent fatigue (80%, n=20), headache (76%, n=19), decreased appetite (76%, n=19), reflux/vomiting (72%, n=18), and dizziness (68%, n=17). Patients who reported that there was no improvement in their primary symptom, more frequently reported having a decreased appetite in comparison to the patients where IB‐Stim improved their primary symptom (100% vs 64%, p=0.045). However, when compared to adolescents who had no improvement in their primary symptom with IB‐Stim treatment, patients who did have improvement in their primary symptom more often had improvement in other symptoms of dizziness (60% vs 89%, p=0.026) and frequent fatigue (70% vs 22%, p=0.039).

**Conclusions:** This a single center prospective survey‐based quality improvement study that sought to characterize the presence of symptoms in adolescents receiving treatment for functional abdominal pain or functional nausea with IB‐Stim. The majority of these patients also reported having symptoms of frequent fatigue, headache, decreased appetite, reflux/vomiting, and dizziness. Adolescents who reported an improvement in their primary symptom (abdominal pain or nausea) more frequently had improvement in other symptoms of dizziness and frequent fatigue while those patients who did not have an improvement in their primary symptom more often reported having an additional symptom of decreased appetite. Further research is necessary to further validate these findings.

## 238 BINATIONAL REFERENCE STANDARDS FOR HIGH‐RESOLUTION ESOPHAGEAL MANOMETRY IN PEDIATRIC PATIENTS WITH NORMAL ESOPHAGEAL MOTILITY ACCORDING TO THE CHICAGO CLASSIFICATION 4.0

*Sofia Gonzalez Garcia* ^*1*^, *Erick Toro‐Monjaraz* ^*1*^, *Flora Zarate‐Mondragon* ^*1*^, *Ericka Montijo* ^*1*^, *Jose Francisco Cadena* ^*1*^, *Karen Ignorosa* ^*1*^, *Roberto Cervantes* ^*1*^, *Jaime Ramirez Mayans* ^*1*^, *Ana Rocca* ^*2*^, *Maria Neder* ^*2*^

^*1*^ *Pediatric Gastroenterology and Nutrition*, *Instituto Nacional de Pediatria*, *Mexico City*, *CDMX*, *Mexico*; ^*2*^ *Fundacion Garrahan*, *Buenos Aires*, *Buenos Aires*, *Argentina*

**Introduction:** High‐resolution esophageal manometry (HRM) is the gold standard for diagnosing esophageal motility disorders. However, its application in the pediatric population faces significant limitations due to the lack of standardized reference values. Protocols and equipment designed for adults are not directly transferable to children, risking misdiagnosis. Manometric metrics are influenced by variables such as age, height, and growth, highlighting the need for pediatric specific reference ranges for accurate interpretation.

**Objective:** To describe HRM values in patients under 18 years of age with normal esophageal motility according to the Chicago Classification 4.0, assessed at two leading pediatric centers in Latin America: National Institute of Pediatrics in Mexico City and the Garrahan Pediatric Hospital in Buenos Aires, Argentina, between 2015 and 2024. Additionally, to establish reference percentiles for key manometric parameters, adjusted by age and height.

**Methods:** We conducted an observational, retrospective, cross‐sectional, and descriptive study. Medical records of pediatric patients who underwent HRM and were diagnosed with normal esophageal motility based on the Chicago Classification 4.0 were included. Exclusion criteria comprising confirmed motility disorders, incomplete data, lack of follow‐up, or insufficient technical quality were excluded. Data from both institutions were merged into a unified database and analyzed using IBM SPSS Statistics v20.0. Descriptive statistics were applied, and 5th to 95th percentiles were calculated for each parameter, stratified by age and height.

**Results:** Out of 185 patients evaluated, 72 met the inclusion criteria. The following percentiles ranges were The following percentile ranges were established for key HRM parameters: Distal Contractile Integral (DCI) at the 5th percentile was 497.8 mmHgscm, with a 95th percentile of 3286.7 mmHgscm (mean: 1501.7 mmHgscm). Integrated relaxation Pressure (IRP) had a 5th percentile value of 5.00 mmHg and reached 21.00 mmHg at the 95th percentile (mean: 12.58 mmHg). For the LES pressure, the 5th percentile was 4.65 mmHg and the 95th percentile was 41.35 mmHg (mean: 22.42 mmHg). UES pressure ranged from 35.25 mmHg at the 5th percentile to 254.55 mmHg at the 95th percentile (mean: 113.87 mmHg). A statistically significant positive correlation was observed between distal latency, esophageal length, and patient height.

**Discussion:** Using reference values adjusted for age and height allows for more accurate interpretation of HRM in pediatric patients, reducing the risk of misclassification associated with applying adult norms. Distal latency, in particular, is significantly influenced by esophageal length and height, underscoring the importance of considering developmental anatomy in motility assessments.

**Conclusion:** This binational study provides valuable reference data for pediatric esophageal manometry. The establishment of age and height adjusted percentiles for HRM metrics enhances diagnostic accuracy and supports clinical decision‐making in children. The observed correlation between anatomical growth and manometric values highlight the need of pediatric specific standards in esophageal motility evaluation.

## 239 DIAGNOSTIC VALUE OF MR DEFECOGRAPHY IN CHILDREN WITH INTRACTABLE FUNCTIONAL CONSTIPATION AND COMPARISION WITH ANORECTAL MANOMETRY RESULTS

*Kyle Glisson* ^*3*^, *Kirk Thame* ^*3*^, *Gabriella Crane* ^*2*^, *Ajay Kaul* ^*1*^

^*1*^ *Gastroenterology*, *Cincinnati Children's Hospital Medical Center*, *Cincinnati*, *OH*; ^*2*^ *Radiology*, *Monroe Carell Junior Children's Hospital at Vanderbilt*, *Nashville*, *TN*; ^*3*^ *Pediatric Gastroenterology*, *LSU Health New Orleans*, *New Orleans*, *LA*

**Introduction:** Functional constipation is the most common cause of constipation in children, and most do not need any testing and respond to conventional management. Inability to achieve resolution is likely due to non‐compliance and inadequate therapy in some children; however, 20 to 30% of these children do not respond to conventional management. Outlet dysfunction is an important cause of intractable constipation in children. Currently, anorectal manometry is used to identify dyssynergic defecation, but this modality has a high risk of false positives and cannot detect structural abnormalities of the pelvic floor or rectum that may be contributing to the dyssynergic pattern. MRI defecography (MRD) is a radiation‐free method that enables dynamic visualization of the pelvic floor and rectum during defecation. The presence of these structural abnormalities has not been well studied in children, and there is a critical knowledge gap that can potentially impact our management of intractable pediatric functional constipation (PFC).

**AIM:** to study the feasibility and utility of MRD in children with intractable PFC and compare its results with those of anorectal manometry

**Methods:** A retrospective review was performed for all children ages 4 through 18 years of age who underwent MRI defecography between the years 2014 through 2023.

MRI defecography was performed according to previously published protocols, and the results were classified into the following three groups:

(A) Normal study (B) Pelvic floor dyssynergia, and (C) Structural abnormality, using adult norms.

Anorectal Manometry was performed with a solid state 12 F high resolution catheter according to standard protocol.

**Results:** During the study period, a total of 27 patients underwent 29 MRD studies (16 males and 11 females, mean age 11 years, age range 6‐18 years). All 27 patients had intractable constipation‐related symptoms despite appropriate conventional therapy. Twenty‐two of these children also had anorectal manometry. Two patients older than 18 were excluded. For the remaining 25 patients, the MRD was:

**1. Normal:** in a 13‐year‐old boy who spent long periods on the toilet despite normal frequency and consistency of BM and normal KUB on laxative therapy.

**2. Dyssynergia:** in 13 children (mean age 10.8 years). Attempted evacuation resulted in no change in the anorectal angle (ARA) in two and narrowing of the ARA in nine. In two cases, there was normal movement but no evacuation of gel.

**3. Structural abnormalities:** diagnosed in 9 (5 females, 4 males, mean age 11.5 years). All 9 had descending perineum syndrome, and 7 rectoceles, 3 cystoceles, 3 enteroceles and 1 recto‐anal intussusception were identified. Structural abnormalities caused pressure effect on the genitourinary system in 6 patients.

Two studies had features of both structural abnormalities and dyssynergia (both females). One showed narrowing of the ARA with a small anterior rectocele with pressure effect on the vagina and urethra. The other revealed failure of the ARA to open, descent of all pelvic organs and a 3.2 cm rectocele causing pressure on the bladder and urethra.

**Anorectal Manometry and MRD:** Twenty patients underwent both MRD and ARM. The single patient with a normal MRD also had a normal ARM. The remaining 19 patients had dyssynergic defecation on ARM. Twelve of these 19 patients (63%) also exhibited dyssynergic defecation on MRD. However, 2 with dyssynergic defecation on MRD also had structural abnormalities. The remaining 7 patients found to have dyssynergic defecation on ARM had a variety of structural abnormalities on MRD without evidence of dyssynergia.

**Conclusion:** While MRD successfully identified dyssynergic defecation like ARM, it also detected structural abnormalities that ARM could not. Identifying these structural abnormalities is crucial, as patients with descending perineum syndrome require an alternative approach to pelvic floor therapy, and other structural abnormalities may necessitate monitoring or surgical intervention.

Despite the relatively small sample size and retrospective nature of the study, it was able to show the feasibility and utility of performing MRD in the pediatric age group. MRD is likely most beneficial for patients with persistent symptoms despite adherence to conventional treatment. The percentage of patients with structural abnormalities (33%) in this study was significantly higher compared to other published pediatric studies (barium defecography), which reported rates of 14‐22%. This suggests that a substantial proportion of patients with refractory symptoms may have unrecognized underlying structural abnormalities contributing to their condition.

Currently, the norms used for MRD are based on the adult population. The measurements used to define normal pelvic descent, and a significant rectocele may lead to underestimation of abnormalities in smaller pediatric patients. Establishing pediatric‐specific norms would improve the accuracy of these evaluations.

## 240 PEDIATRIC FUNCTIONAL ABDOMINAL PAIN DISORDERS: EXAMINING THE RELATIONSHIP BETWEEN UNDERSTANDING AND HOPE

*Zoha Shahabuddin* ^*1*^, *Laurence Feinstein* ^*2*^

^*1*^ *Pediatrics Residency Program*, *St Christopher's Hospital for Children*, *Philadelphia*, *PA*; ^*2*^ *Department of Pediatric Gastroenterology, Hepatology, and Nutrition*, *St Christopher's Hospital for Children*, *Philadelphia*, *PA*

**Introduction:** Functional abdominal pain disorders (FAPDs) are characterized by chronic abdominal pain without identifiable underlying pathology and with significant functional disability. The etiology of FAPDs is complex and multifactorial, and management is founded on a biopsychosocial approach. Patient education is considered a mainstay of management and has the added benefit of being cost‐effective, readily accessible, and easily implementable. It has been found effective in improving gastrointestinal symptoms and quality of life in individuals with FAPDs. Hope has been found to be an important protective factor for pediatric patients with chronic illnesses like diabetes and cancer, but few studies have considered hope in relation to FAPDs. This study aims to assess the impact of functional abdominal pain education and the resulting level of patient understanding on the outcome of hope in pediatric patients with FAPDs.

**Methods:** Children 8‐17 years old managed outpatient by the institutional pediatric gastroenterology clinic for a diagnosed FAPD were recruited to complete an anonymous online survey. Participants were identified by chart review and recruited in‐person during routine clinic visits and via phone call/email. The survey gathered information regarding experiences with FAPD‐related education, including educational modalities (verbal, written, online) that had previously been utilized with the patient. The survey also included measures of objective understanding of pain science (Concept of Pain Inventory‐ Child; COPI‐C), subjective understanding of functional abdominal pain (developed by researchers for the study), and hope (Children's Hope Scale; CHS). Survey responses were collected on RedCap, and data analysis was performed using Microsoft Excel and SPSS. Statistical tests utilized in data analysis included two‐sample T‐testing, Analysis of Variance, Shapiro‐Wilk normality testing, Pearson correlation, and Spearman correlation.

**Results:** A total of 33 survey responses were collected. The mean age of respondents was 14.70 years, and the most represented FAPD diagnosis was irritable bowel syndrome (N= 27, 82%). The mean CHS score of the sample was 16.18, corresponding with slight hope. Most respondents indicated experiencing only 1 type of educational modality (N=16, 48%), with verbal education being the most frequently employed (N=30, 91%). Pearson correlation coefficients were utilized to assess for significant associations between COPI‐C scores and CHS scores. COPI‐C scores were positively correlated with CHS total scores (r= 0.348, p= 0.047) and CHS pathway sub‐scores (r= 0.412, p= 0.017). Because subjective understanding scores did not meet assumption of normality (Shapiro‐Wilk p= 0.007), Spearman correlation coefficients were utilized to assess for significant associations between subjective understanding scores and CHS scores. Subjective understanding scores were positively correlated with CHS total scores (ρ= 0.438, p= 0.011) and CHS pathway scores (ρ= 0.558, p= <0.001). There were no statistically significant associations between the number or type of utilized educational modalities and COPI‐C scores, subjective understanding scores, or CHS scores, though analysis was significantly limited by sample size.

**Conclusion:** In pediatric patients with FAPDs, better subjective and objective understandings of functional abdominal pain are associated with increased hope. Future directions for this study include continuing data collection and analysis to further elucidate the role played by educational interventions in shaping both understanding and hope outcomes.


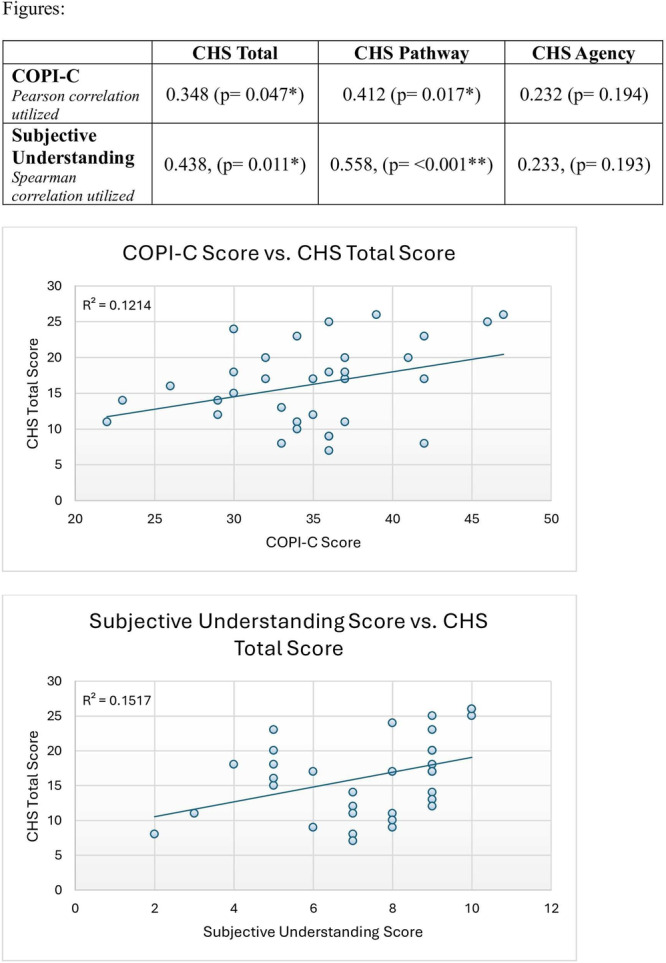


## 241 FUNCTIONAL LUMEN IMAGING PROBE (FLIP) IN CHILDREN WITH SYSTEMIC SCLEROSIS: CHARACTERISTICS AND CLINICAL UTILITY

*Alex Cohen* ^*1*^, *Justin Wheeler* ^*2,1*^

^*1*^ *Pediatrics*, *University of Utah Health*, *Salt Lake City*, *UT*; ^*2*^ *Division of Pediatric Gastroenterology*, *University of Utah Health Hospitals and Clinics*, *Salt Lake City*, *UT*

**Background:** Pediatric systemic sclerosis (SSc) is a rare, complex disease with cutaneous and extracutaneous morbidity. Up to 90% of patients with SSc have gastrointestinal organ involvement, including gastroesophageal reflux disease, esophageal dysmotility, or gastroparesis. Diagnostic testing like esophagogastroduodenoscopy (EGD), pH‐impedance testing, and High Resolution Esophageal Manometry (HREM) can identify affected areas and guide targeted therapy. HREM can be challenging for pediatric patients to tolerate, and may not fully characterize sphincter function or tissue fibrosis. Functional luminal imaging probe (FLIP) technology utilizes impedance to measure hollow organ cross sectional area, distensibility, compliance, and secondary peristalsis in a sedated patient. Use of FLIP to characterize esophageal function in adult patients with SSc has been described, but there are no published studies on use of FLIP in pediatric SSc. This study highlights a single center experience using FLIP testing during diagnostic EGD as an adjunctive tool to assess esophageal motility, distensibility, and function to help guide diagnosis and treatment of this rare disease.

**Methods:** Electronic medical record review was performed on 5 pediatric patients ages 8‐16 years with SSc and upper gastrointestinal involvement (dysphagia, esophagitis, and/or chronic abdominal pain) treated at Primary Children's Hospital in Salt Lake City, UT. Patient's underwent EGD with Functional Lumen Imaging Probe (FLIP) to assess esophageal function, including measurement of the esophagogastric junction distensibility index (EGJ‐DI), contractile pattern, and esophageal sphincter and body diameter. Demographics and diagnostic findings are described.

**Results:** 5 patients (female=3), mean age 10 years, had an EGD with FLIP study performed. There were no complications. 60% (n=3) had visual and histologic esophagitis. 40% (n=2) had increased EGJ‐DI (>9 mm^2^/mmHg). No patients had low EGJ‐DI (<3 mm^2^/mmHg). 60% (n=3) had absent Repetitive Antegrade Contractions (RAC) typically observed in normal esophageal function. 20% (n=1) had abnormal Repetitive Retrograde Contractions (RRC).

**Conclusions:** This is the first study describing use of FLIP to characterize esophageal function in pediatric patients with SSc. While the sample size is small, FLIP appears to be safe and effective adjunct to screen for esophageal dysfunction and guide management in patients with pediatric SSc.

## 242 HIGH PREVALENCE OF MENTAL HEALTH COMORBIDITIES IN CHILDREN WITH ACUTE AND RECURRENT PANCREATITIS

*Neha Santucci* ^*1,2*^, *Hailey Kramer* ^*1*^, *Olivia Lee* ^*1*^, *Juan Gurria* ^*1*^, *Todd Jenkins* ^*1*^, *Kristin Rich* ^*1*^, *Cheryl Hartzell* ^*1*^, *Maisam Abu‐El‐Haija* ^*1*^

^*1*^ *Gastroenterology*, *Cincinnati Children's Hospital Medical Center*, *Cincinnati*, *OH*; ^*2*^ *Pediatrics*, *University of Cincinnati College of Medicine*, *Cincinnati*, *OH*

**Background:** Multiple psychological factors and other related comorbidities affect children with chronic pancreatitis (CP) or acute recurrent pancreatitis (ARP). We aimed to describe the frequency of psychological symptoms and diagnoses in children with CP or ARP, as well as predict the severity of the disease burden based on these factors.

**Methods:** We identified patients ages five years and above with a prior history of CP or ARP who were seen by the Behavioral Medicine and Clinical Psychology Clinic (BMCP) at a single center. During these visits, symptoms of mental health disorders as well as any prior mental health diagnoses were identified. Additionally, patients or their families completed pain questionnaires using the NRS pain scale as part of clinical care. This data, as well as other demographic and pancreatic disease data collected from their EMR, was then analyzed.

**Key Results:** Of 105 children in the study (mean age 12.1 3.5 years, 54.3% females), 37.1% of the patients had a psychiatric/behavioral health diagnosis (20.0% anxiety, 13.3% depression, 23.8% ADHD, and 4.8% PTSD). In addition, 86.7% of patients displayed symptoms of psychiatric diagnoses based on DSM‐5 criteria of the disorder (75.7% anxiety, 32.3% depression, 21.1% suicidal ideation, and 40.2% panic, **Table 1)**. Additional systemic comorbidities were seen in the patients (5.7% joint hypermobility syndrome/EDS, 2.9% orthostatic intolerance, 17.1% DGBI, 3.8% biliary dyskinesia, 16.2% migraines, 17.1% other musculoskeletal pain, and 10.5% developmental delay, **Table 1)**.

Regarding pain, 38.8% of patients reported daily abdominal pain, 24.3% had pain weekly, 14.6% had pain monthly, and 22.3% had pain less than monthly (**Table 2**). In the six months prior to their BMCP appointment, 70.9% of patients used opioids in the inpatient setting only, while 25.7% of patients used opioids outpatient to control their pain, and 45.2% of patients were on a neuromodulator drug to control their pain (**Table 2**). 76.2% of patients underwent an endoscopic retrograde cholangiopancreatography treatment (ERCP) and 50.5% of patients underwent Total Pancreatectomy with Islet Autotransplantation (TPIAT, **Table 2**). Median pain severity did not statistically differ by those with a mental health diagnosis, those with mental health symptoms but no prior diagnosis, or those with neither a diagnosis or symptoms (p=.16).

**Conclusion & Inferences:** There was a high prevalence of mental health diagnoses and symptoms in patients with ARP and CP including suicidal ideation. This highlights the need for future prospective studies to explore the role of mental health in affecting disease outcomes in children with CP or ARP.


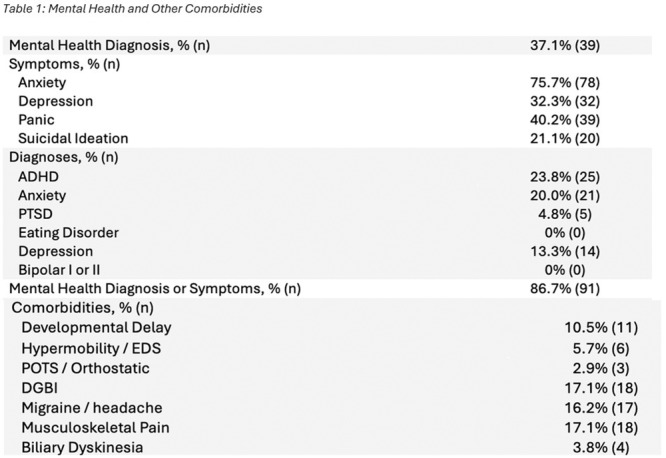



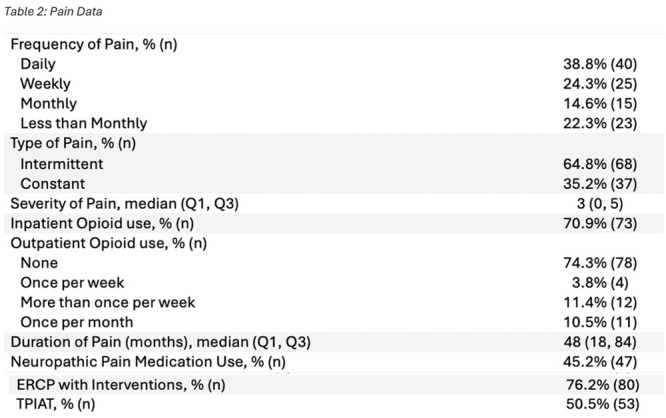


## 254 CONJUGATION OF PEPTIDE LIGANDS TO AAV TOWARDS PANCREATIC CELL GENE THERAPY

*Stephen Trisno* ^*1*^, *Mark Kay* ^*2,3*^

^*1*^ *Pediatric Gastroenterology, Hepatology and Nutrition*, *Stanford Medicine*, *Stanford*, *CA*; ^*2*^ *Pediatrics*, *Stanford University*, *Stanford*, *CA*; ^*3*^ *Genetics*, *Stanford University*, *Stanford*, *CA*

Adeno‐associated virus (AAV) is a small DNA virus that has been closely studied for its potential for human gene therapy. Multiple serotypes of AAV and recombinant AAV possess differential transduction efficiency and specificity towards different tissues; however, no cell type‐specific AAV vectors have been created to date. In order to improve cell/tissue transduction efficiency and specificity, our lab explored azide unnatural amino acid substitution on the AAV capsid that allows for site‐specific modification via click‐chemistry to conjugate targeting molecules to the AAV (Puzzo et al., 2023). In this study, we seek to demonstrate an expansion of these capabilities towards targeting pancreatic cells to develop vectors for *in vivo* gene therapy for pancreatic and endocrine genetic disorders, such as hereditary pancreatitis, cystic fibrosis, and diabetes. To target pancreatic exocrine and endocrine cells, we selected peptide ligands that bind to surface receptors known to be expressed on those cells. Specifically, we selected ligands that bind to SCTR (secretin receptor, for targeting pancreatic exocrine cells) or GLP1R (glucagon‐like peptide 1 receptor, for targeting pancreatic endocrine cells). The peptides secretin (SCT) and eGLP1 (a GLP1 agonist) were successfully conjugated to the azide amino acid‐substituted AAV‐DJ R/A‐A587. The secretin peptide‐conjugated AAVs (AAV‐SCT) demonstrated a several‐fold increase in transduction efficiency towards cell lines exogenously expressing SCTR when compared to unconjugated AAVs or when compared to transducing control cell lines not expressing SCTR. This improvement in transduction efficiency was dependent on the degree of conjugation of the AAV, with an intermediate fraction of viral proteins conjugated by SCT being most optimal. In contrast, the eGLP1 peptide‐conjugated AAVs (AAV‐eGLP1) demonstrated a decrease in transduction efficiency in various cell lines, including GLP1R‐expressing cells, compared to unconjugated AAV. The cause for this reduction is unclear. Next steps include interrogating how the AAV‐ligand‐receptor interaction improves transduction, understanding why eGLP1 peptide‐conjugated AAVs have decreased transduction efficiency overall and if steps can be taken to improve this. We also plan to test these vectors *in vivo* to evaluate whether this improved transduction efficiency is translatable in tissue/organs and whether this improved efficiency is specific to the pancreatic cells expressing the corresponding receptors. Overall, improving the efficiency and specificity of AAV‐based gene delivery will help advance the development of safer gene therapy for genetic disorders of the pancreas and beyond.

## 261* ADVERSE CHILDHOOD EXPERIENCES AND SYMPTOMS OF DISORDERS OF GUT‐BRAIN INTERACTION IN A NATIONAL SAMPLE

*Jamie Klapp* ^*1,2*^, *Sierra Martin* ^*4*^, *Jacqueline Shiels* ^*5*^, *Christin Ogle* ^*4*^, *Jeffrey Schwimmer* ^*3*^

^*1*^ *Pediatrics*, *University of California San Diego*, *San Diego*, *CA*; ^*2*^ *Pediatrics*, *Rady Children's Hospital‐San Diego*, *San Diego*, *CA*; ^*3*^ *Pediatric Gastroenterology, Hepatology and Nutrition*, *Rady Children's Hospital‐San Diego*, *San Diego*, *CA*; ^*4*^ *Center for the Study of Traumatic Stress, Department of Psychiatry*, *Uniformed Services University of the Health Sciences*, *Bethesda*, *MD*; ^*5*^ *Psychiatry*, *Kaiser Foundation Hospitals*, *Oakland*, *CA*

**Background:** Disorders of gut–brain interaction (DGBI) are among the most common reasons for referral to pediatric gastroenterology. Mounting evidence suggests that early‐life adversity, particularly adverse childhood experiences (ACEs), can disrupt neuroendocrine, immune, and autonomic signaling between the gut and brain. While these links are well‐documented in adults, the impact of specific ACEs on pediatric gut–brain health remains poorly understood. This study aimed to identify which ACEs are most strongly associated with DGBI symptoms in children and to evaluate psychosocial mechanisms and contextual factors that may mediate or moderate risk.

**Methods:** We analyzed data from 73,055 children ages 6–17 years from the 2021–2023 National Survey of Children's Health. DGBI was defined as caregiver‐reported frequent or chronic gastrointestinal symptoms, chronic pain, or both, reflecting common clinical presentations in pediatric gastroenterology. ACEs were coded from validated survey items, including household dysfunction, exposure to violence, and discrimination. We used Least Absolute Shrinkage and Selection Operator (LASSO) logistic regression to identify ACEs most strongly associated with DGBI. We conducted causal mediation analysis to evaluate the role of emotional and behavioral difficulties and examined moderation by protective factors such as perceived safety and community support.

**Results:** DGBI symptoms were reported in 13% of children. LASSO identified five ACEs most strongly associated with DGBI: household mental illness (aOR 1.63; 95% CI: 1.52–1.75), economic insecurity (aOR 1.42; 95% CI: 1.32–1.53), racial discrimination (aOR 1.42; 95% CI: 1.29–1.57), neighborhood violence (aOR 1.38; 95% CI: 1.25–1.53), and household substance use (aOR 1.07; 95% CI: 1.00–1.15). Emotional and behavioral difficulties mediated 28–31% of the association between DGBI and exposures such as household mental illness, substance use, and neighborhood violence. Food insecurity explained 24% of the effect of economic hardship on gut–brain symptoms. Perceived safety at school and in the neighborhood moderated multiple associations: children with high adversity who felt safe had lower symptom burden. Subgroup analyses revealed that racial discrimination was significantly associated with chronic pain symptoms but not with gastrointestinal symptoms, suggesting possible symptom‐specific pathways.

**Conclusions:** ACEs are potent risk factors for DGBI in children, with household mental illness and financial hardship emerging as the strongest predictors. Emotional health, food insecurity, and perceived safety partially explain or buffer these associations. These findings support the need for trauma‐informed care in pediatric GI settings and support routine psychosocial screening and cross‐sector partnerships to improve gut–brain outcomes and reduce long‐term disparities.


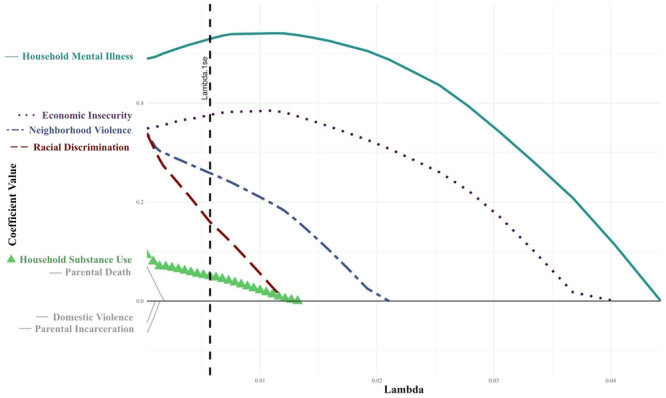


**Figure 1.** LASSO Coefficient Path Plot for the Association Between ACEs and DGBI

Coefficient paths of ACEs and covariates across a range of penalty values (lambda). Vertical dashed line is the lambda.1se value. Colored lines indicate ACE variables retained at lambda.1se; positive coefficients reflect stronger associations with DGBI. Gray lines represent ACEs whose coefficients were shrunk to zero before lamda.1se. ACE3 (parental divorce) is excluded from depiction as its coefficient remained effectively zero across the entire regularization path.

## 262 CHILDREN AND ADOLESCENTS WITH DISORDERS OF GUT‐BRAIN INTERACTION AND ATTENTION DEFICIT/HYPERACTIVITY DISORDER HAVE WORSE PSYCHOLOGICAL FUNCTIONING IN A CASE CONTROL STUDY

*Neha Santucci* ^*1,2*^, *Priyanshi Shah* ^*1*^, *Jen Hardy* ^*1*^, *Rashmi Sahay* ^*1*^

^*1*^ *Gastroenterology*, *Cincinnati Children's Hospital Medical Center*, *Cincinnati*, *OH*; ^*2*^ *Pediatrics*, *University of Cincinnati College of Medicine*, *Cincinnati*, *OH*

**Introduction:** Attention‐deficit/hyperactivity disorder (ADHD) is a common comorbidity within pediatric populations with disorder of the gut‐brain interaction (DGBI). Children with DGBI often have numerous concomitant psychiatric diagnoses. We aimed to analyze GI and psychological outcomes in children with DGBI and ADHD and compare them to children with DGBI without ADHD.

**Methods:** We retrospectively reviewed charts of DGBI patients who met Rome IV criteria and were seen in the DGBI clinic at Cincinnati Children's Hospital Medical Center ages 9‐20 y. We divided the cohort based on those who had a diagnosis of ADHD and those without. We included demographics, anthropometrics, medical and treatment history, and validated questionnaire responses routinely used in clinical care: Abdominal pain (API), Nausea (NSS), Pain Catastrophizing (PCS‐C), Disability (FDI), PROMIS – Anxiety, Depression (PHQ‐9), and Somatization (CSI) at baseline and at 6‐12 month follow up. We analyzed outcomes of those with ADHD compared to those without ADHD.

**Results:** Sixty‐five patients with ADHD (mean age 15.62 ± 2.4 y, 67.7% females, 90.8% Caucasian) and at least one DGBI were identified. We included 266 DGBI patients without ADHD (mean age 15.66 ± 2.7 y, 82% females, 88.7% Caucasian). Patients with ADHD had worse baseline functioning (p=0.02), somatization (p=0.0002), depression (p=0.001) and a trend for worse anxiety (p=0.09) compared to those without (Figure 1). Furthermore, functioning (p=0.01) and somatization (p=0.05) were worse at follow up in those with ADHD compared to those without. BMI for age percentile trended to be lower in those with ADHD compared to those without ADHD at baseline (p=0.08) and follow‐up visits (p=0.09) respectively.

**Conclusion:** Thus, DGBI patients with ADHD have worse psychological functioning with less improvements in functioning and somatization after treatment. This should be taken into consideration while tailoring therapy. Future treatment studies can be geared towards approaches targeting both DGBI and ADHD to improve psychosomatic outcomes in this population.


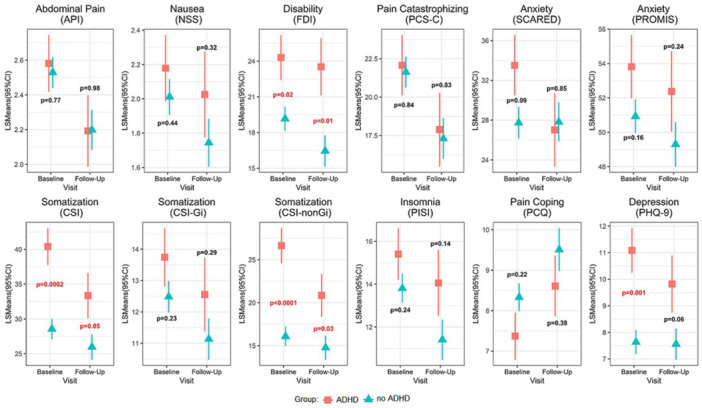


Outcomes in DGBI patients with and without ADHD

## 263 AGREEMENT BETWEEN YOUTH‐ AND PARENT‐REPORT OF PHYSICAL AND EMOTIONAL SYMPTOMS IN DISORDERS OF GUT‐BRAIN INTERACTION

*Erika Chiappini* ^*1*^, *Seoyeon Yoo* ^*1*^, *Ching‐Yuan Want* ^*1*^, *Sofia Wicker Velez* ^*2*^, *Melanie Brown* ^*3*^, *Shaija Kutty* ^*2*^

^*1*^ *Psychiatry and Behavioral Sciences*, *Johns Hopkins University*, *Baltimore*, *MD*; ^*2*^ *Pediatric Gastroenterology Hepatology and Nutrition*, *Johns Hopkins Children's Center*, *Baltimore*, *MD*; ^*3*^ *Pediatrics*, *Johns Hopkins Children's Center*, *Baltimore*, *MD*

**Background:** Youth with disorders of gut‐brain interaction (DGBI) experience several associated negative impacts, including worse anxiety and mood symptoms, poorer quality of life and functional disability. Important to assessing these challenges is obtaining a reliable report of symptoms. For providers, it is essential to obtain the youth's perception of their symptoms for treatment planning. However, caregiver's interactions with their children are based on their perceptions of their child's symptoms. As parenting factors have been identified to influence treatment outcomes in youth with DGBI, the agreement between youth and parent report becomes relevant to appropriately intervene. Though previous studies have examined agreement in symptoms in pain disorders broadly and mental health symptoms, they have not examined agreement in DGBIs specifically.

**Methods:** Patients were 67 youth and young adults (M=15.31 years, SD=2.57, range 9‐21 years) with diagnoses of abdominal pain (n=34, 64.2%), irritable bowel syndrome (IBS; n=32, 47.8%), nausea (n=26, 38.8%), constipation (n=18, 26.9%), and/or rumination (n=4, 6.0%). Youth and their families presented to a multidisciplinary clinic for DGBI, where they collectively met with a pediatric gastroenterologist, psychologist, integrative medicine, and dietitian for assessment and recommendations of abdominal pain and associated symptoms. Patients and caregivers completed a survey during their clinic visit that assessed somatic symptoms (CSSI), functional disability (FDI), and PROMIS measures of anxiety, depression, pain behaviors, and sleep. Agreement was evaluated using intraclass correlation (two‐way, random effects, absolute agreement). Mean differences between parents and children were evaluated with t‐tests.

**Results:** Agreement between youth and their caregivers on measures of anxiety was poor (ICC=0.47, CI=.24‐.64, p<.001), with parents endorsing more anxiety than their children. Agreement between youth and their caregivers was moderate on measures of somatic symptoms (ICC=.74, CI=.50‐.86, p<.001), depression (ICC=.59, CI=.40‐.73, p<.001), sleep disturbance (ICC=.69, CI=.49‐.83, p<.001) and pain behaviors (ICC=.74, CI=.56‐.85, p<.001). Agreement was good on a measure of functional disability (ICC=.76, CI=.62‐.85, p<.001). Parents reported significantly more sleep difficulties than youth (t(39)=‐2.20, p=.03. Youth reported significantly more somatic symptoms than parents (t(62)=4.46, p<.001). No other significant mean differences were observed.

**Conclusions:** Youth with DGBI and their parents show good agreement about how they are functioning in daily living. However, youth and their parents show less agreement with more experiences that are more internal to the youth (e.g., anxiety, pain symptoms, depression). Additionally, parents report more sleep challenges and youth report more pain symptoms overall. These findings highlight the importance of obtaining multiple perspectives of a youth's symptoms for a more complete impression.

## 264 CONNECTING MINDS AND GUTS: IMPLEMENTATION OF AN EREFERRAL FOR PEDIATRIC PSYCHOGASTROENTEROLOGY SERVICES

*Catherine Naclerio* ^*1,2*^, *Olivia Adams* ^*3*^, *Ryan Morrow* ^*2*^, *Morgan Drake* ^*1,2*^, *Monique Germone* ^*1,2*^, *Julie Rinaldi* ^*1,2*^, *Christine Reinhard* ^*1,2*^

^*1*^ *Psychiatry*, *University of Colorado System*, *Denver*, *CO*; ^*2*^ *Division of Gastroenterology, Hepatology, and Nutrition*, *University of Colorado System*, *Denver*, *CO*; ^*3*^ *Digestive Health Institute*, *Children's Hospital Colorado*, *Aurora*, *CO*

**Introduction:** Poor communication within medical teams can negatively impact patient care and is associated with wasted time for clinicians, prolonged patient stays, and increased medical costs. In pediatrics, delays due to poor communication can affect a child's development and psychosocial well‐being. Electronic referral systems (eReferrals), integrated into electronic medical records (EMRs), allow providers to initiate, track, and communicate referrals across specialties. eReferrals were developed to improve consistency of key clinical information shared between providers. Research has been somewhat limited on the topic of eReferrals; however, findings suggest that eReferrals are cost‐effective and increase the quantity and quality of referrals. While the impact of eReferrals has been examined in other medical populations, it has yet to be studied in pediatric gastroenterology (GI) populations. The following project looks at the novel implementation of an eReferral for access to specialized pediatric psychology services within a large GI division at a regional children's hospital. In pediatric GI, it has been well established that integrated psychology is a vital component of care given the role of stress and sympathetic nervous system activation on symptom severity and chronicity. Timely access to psychology services is therefore critical, highlighting the need for integrated and efficient referral pathways to psychological care.

**Method:** Prior to implementation, referrals to psychology within the GI clinic were submitted by medical providers informally via inconsistent routes (e.g., verbal handoffs, messages to scheduling team, advising families to schedule themselves). These referrals often lacked key clinical details, leading to delays, inconsistent triage, and administrative burden. In July 2023, the psychology team implemented a standardized eReferral that required referring gastroenterologists to indicate relevant clinical information, including diagnosis and reason for referral. eReferrals were routed to the scheduling queue and available to psychologists for review. We conducted a retrospective pre‐post analysis at a large pediatric GI clinic. Patient visit data from the pre‐eReferral period was collected between January 2022 and June 2023 while eReferral data, patient visit data, and patient demographics were collected from July 2023 to December 2024. Patient demographics included age, identified gender, and referral diagnosis. The primary outcome was the number of patient visits seen per month, both pre‐ and post‐implementation of the eReferral. An independent samples t‐test was conducted to compare mean patient volume between the two periods.

**Results:** In the 18‐month period post‐eReferral, a total of 2090 eReferrals were placed to the psychology service. The average number of referrals per month was 116 (SD= 35.1). The average age of referred patient was 12.2 years (SD=4.3) and 58.6% identified as female. The most common referral diagnoses were Abdominal Pain (38.3%) and Constipation (23.7%). The most common reasons for referral were Pain/Symptom management (30.3%), Adjustment and Coping with Diagnosis (29.8%), and Assessment of Psychosocial Stressors and Individual Coping (21.9%). Psychology providers met with 42% of referred patients. Analyses showed that number of patient visits seen by the psychology service significantly increased following the eReferral system (*n*_*pre*_=1766, *n*_*post*_=2441, p<0.001) with an average of 37.5 more patients seen per month (Figure 1). Lastly, over time, patients were seen more quickly after the referral was placed, indicating that access improved following the system's implementation. The average time from referral to receiving behavioral services during the first nine‐month period of the eReferral was 37.9 days, while the average time to be seen from referral for the second 9 nine‐month period was 26.6 days.

**Conclusions:** Implementation of an eReferral in pediatric GI is promising and feasible. Findings show significantly increased access to psychological care and reduced wait times, suggesting that systems‐levels changes can improve multidisciplinary care delivery. Future research should explore barriers to scheduling and scalability across subspecialities.


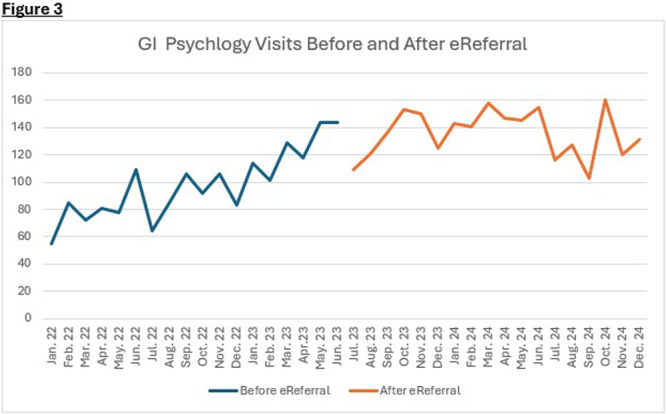


## 265 GLUTENFREE BUDDIES: A PILOT PEER‐TO‐PEER MENTORSHIP PROGRAM FOR YOUTH WITH CELIAC DISEASE

*Peacha Sokzini* ^*2*^, *Ashley Dunn* ^*2*^, *Bren Botzheim* ^*1*^, *Derek Boothroyd* ^*1*^, *Parnia Abrishamchian* ^*2*^, *Ally Koh* ^*2*^, *Jennifer Iscol* ^*3*^, *Garret Forshee* ^*2*^, *Anava Wren* ^*2*^, *Hilary Jericho* ^*2*^

^*1*^ *Department of Medicine, Quantitative Science Unit*, *Stanford University School of Medicine*, *Stanford*, *CA*; ^*2*^ *Pediatrics*, *Stanford University School of Medicine*, *Stanford*, *CA*; ^*3*^ *Celiac Community Foundation of Northern California*, *Healdsburg*, *CA*

**Background:** Celiac disease (CeD) is an autoimmune enteropathy triggered by gluten and treated with a gluten‐free diet (GFD). Youth with CeD often experience higher rates of depression and anxiety and a reduced quality of life. While peer mentorship programs benefit youth with chronic illnesses, their impact on youth with CeD is unknown. This study aimed to develop and pilot a peer‐to‐peer mentorship program for youth with CeD.

**Methods:** GlutenFree Buddies (GFB) was a 6‐month peer‐to‐peer mentorship program for youth aged 13‐25 with CeD that ran from February 2024 to August 2024. Participants were recruited via community and patient email databases and invited to join the GFB intervention group, consisting of peer mentor training, monthly meetings, and safety check‐ins, or a control group. Participants in the intervention group were matched with a peer mentor based on age and interest to foster stronger peer‐to‐peer connections. Both intervention and control groups completed validated surveys to assess GFD compliance and psychosocial wellbeing at baseline, 3 months, 6 months, and 8 months (Table 1). The intervention group also completed a feedback survey at the 6‐month time point. Standardized mean differences (SMDs) between intervention and control participants were calculated for the survey instruments at baseline. Descriptive statistics were calculated for baseline demographic variables and for each of the outcomes at each time point. An equal variance two‐sample t‐test was performed for the primary outcomes at 6 and 8 months. The feedback survey responses were analyzed quantitatively, with means calculated for each item to identify overall trends in participant experience and satisfaction.

**Results:** The study included 92 participants [mean age = 17.5; age range = 13‐25; 77% female; 88% white or European], with 42 in the intervention group and 50 in the control group. Sixty percent of program participants completed the 6‐month feedback survey and 68% completed the 8‐month survey. While participants were not randomized, baseline SMDs were small. At 6 months, the peer mentorship group showed statistically significant improvements in GFD adherence compared to the control group (p = 0.02), as well as positive trends in depression, quality of life, and psychosocial wellbeing. There were no statistically significant group differences in peer relationships, resilience, or global health. Participants reported high satisfaction with the program and their peer mentor match, and most were very likely to recommend the program to others.

**Conclusion:** Findings highlight the potential of peer mentorship to be a valuable complement to clinical care for youth with celiac disease. This pilot study has established a strong foundation for future iterations and broader programmatic efforts. Participant feedback will guide targeted revisions to the program, including enhancements to mentor training and development of strategies to foster sustained engagement, thereby strengthening the program's long‐term impact and scalability. Future research should be conducted in larger and more diverse populations of adolescents and young adults with celiac disease to increase the program's generalizability.


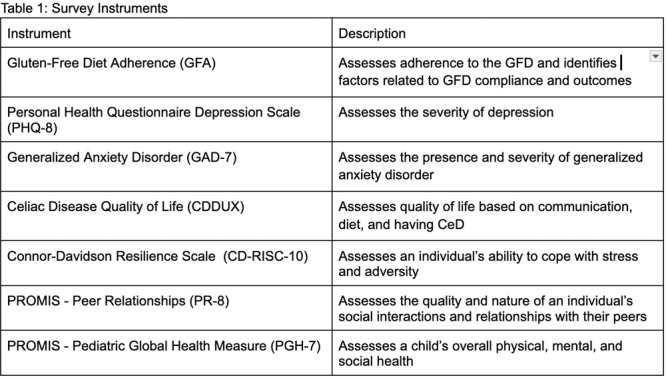


## 266 HELICOBACTER PYLORI EN NIÑOS: FACTORES DE RIESGO Y DIAGNÓSTICO DE INFECCIÓN EN LOS MIEMBROS DE LA FAMILIA

*Jessica Merino* ^*1*^, *Idalmis Matos* ^*1*^, *Daime Guilarte Falcon* ^*1,2*^, *Elsa García Bacallao* ^*1*^, *Licet González Fabián* ^*1*^, *Maidilys Soria Larduet* ^*1*^, *Enrique Galbán García* ^*1*^, *Maria Pérez Rodríguez* ^*1*^

^*1*^ *Plaza de la Revolución*, *Instituto de Gastroenterologia*, *Havana*, *Havana*, *Cuba*; ^*2*^ *Gastroenterology and Endoscopy*, *St. Joseph Mercy Hospital*, *Georgetown*, *Demerara‐Mahaica*, *Guyana*

**Abstract:** Background: Helicobacter pylori *(H. pylori)* infection continues to be highly prevalent, especially in developing countries. The most frequently described risk factors are related to socioeconomic conditions. Few studies have been documented in Cuba on the subject. This research aimed to identify the main risk factors and the role of the family in its transmission

**Methods:** Institution based cross‐sectional study was conducted on 165 children ≤18 years, who underwent upper digestive endoscopy in which two invasive methods were performed for the diagnosis of *H. pylori* and 502 cohabitants, who were tested for *H. pylori*. All candidate variables were entered into a multivariate logistic regression.

**Results:** The prevalence of *H. pylori* infection among children and adolescent undergoing upper digestive endoscopy was 46.3%, and 27.6% in family members. Rural origin [OR 2.4 95% CI (1.1‐5.1) p=0.003] and having an infected cohabitant [OR =2.4 95%CI (1.2‐4.6) p=0.001] were the risk factors identified. There was statistical significance in having infected siblings [OR =3.1 95% CI (1‐9.1) p=0.03] and grandparents [OR =3.6 95%CI (1.2‐10.4) p=0.01].

**Conclusions:** In Cuba, the family environment and residence in rural areas constitute significant risk factors for the transmission of Helicobacter pylori infection. Notably, male sex, rural domicile, and cohabitation with infected family members particularly grandparents and siblings have been identified as key variables associated with increased infection risk. These findings underscore the necessity of developing tailored preventive strategies that incorporate both familial and geographic determinants. Moreover, the enhancement of sanitary infrastructure and a thorough investigation of the family milieu are imperative for the effective and sustained eradication of H. pylori in regions with high prevalence. Such interventions are expected to contribute to a reduction in both the incidence and recurrence of infection, as well as in the burden of related complications, thereby advancing public health outcomes within these populations.

**Keywords:** *Helicobacter pylori*, prevalence, children, family, risk factor.


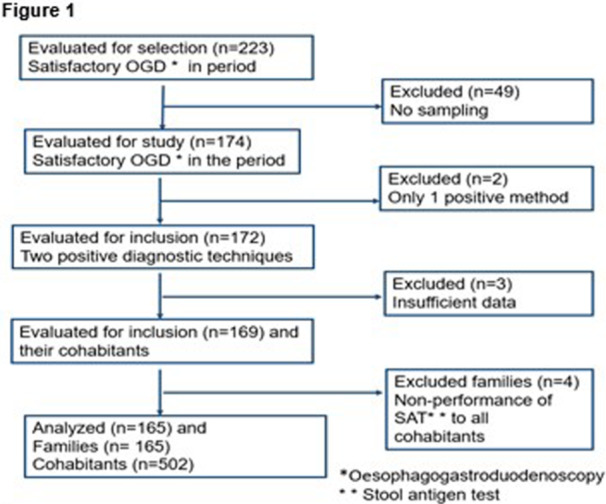



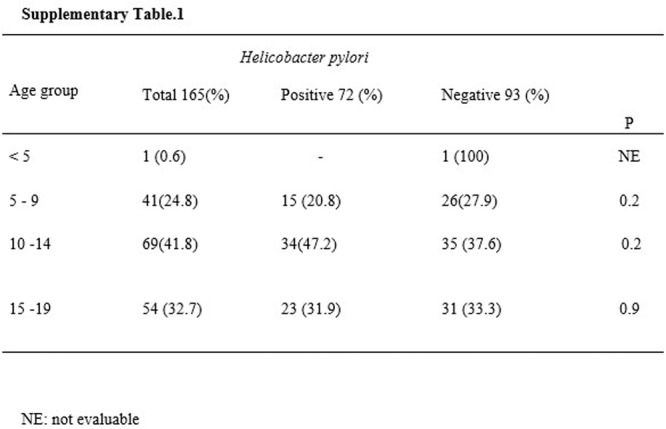


## 267 ENHANCING FOLLOW‐UP AND INTERVENTION AFTER DEPRESSION SCREENING IN PEDIATRIC IBD

*Brittany Gresl*, *Amanda Wenzel*, *Forum Patel*, *Jose Cabrera*, *Abdul Elkadri*, *Joshua Noe*, *Joann Samalik*

*Department of Pediatrics*, *Medical College of Wisconsin*, *Milwaukee*, *WI*

**Background:** Youth with inflammatory bowel disease (IBD) experience an increased risk of depression (up to 31%) compared to the general population (20%), and depressive symptoms can lead to poor health outcomes. Early identification of depressive symptoms is crucial for timely intervention, which has been shown to be beneficial in improving physical and mental health. The ImproveCareNow (ICN) clinical guidelines for depression screening recommend that all patients with IBD ages ≥12 years be screened for depression annually, with documentation of score, disposition and appropriate follow‐up. At our institution, depression screening using the Patient Health Questionnaire (PHQ‐9) is reliably completed and scores are documented in the electronic medical record (EMR); however, the disposition of results and subsequent interventions for patients, particularly those with mild symptoms (PHQ‐9 scores 5‐10) are not consistently documented or addressed.

**Objective:** Assess current adherence to ICN guidelines for depression screening follow‐up in pediatric IBD patients. Improve documentation of disposition of PHQ‐9 scores and increase delivery of depression educational resources for patients who report mild depressive symptoms (PHQ‐9 scores 5‐10).

**Methods:** We initiated a quality improvement (QI) project using the Model for Improvement framework from June 1, 2023. Baseline assessment confirmed that while annual PHQ‐9 screening and score documentation occur consistently for the majority of eligible IBD patients, documentation of disposition and provision of follow‐up resources remain inconsistent.

Barriers identified through process mapping and stakeholder feedback included: perceived negative impact on clinic workflow, challenges with EMR integration, lack of provider awareness regarding documentation expectations, limited familiarity with ICN depression screening clinical practice guidelines, and absence of a structured reminder system.

PDSA Cycle 1 (April 2024‐April 2025):

‐Focus: Increase provider awareness of follow‐up expectations and improve documentation of disposition for elevated PHQ‐9 scores.

‐Intervention: Developed Smartphrase in EMR to document disposition efficiently. Developed standardized educational resource handouts for patients with mild depressive symptoms.

PDSA Cycle 2 (Underway):

‐Focus: Streamline follow‐up documentation and resource distribution.

‐Intervention: Integrate Smartphrase in EMR note template to document disposition efficiently and prompt providers to offer educational materials to patients with PHQ‐9 scores of 5‐10. Conduct monthly audits and provide feedback on individual and team performance. Implement recurring provider reminders via team meetings and visual aids (e.g., ICN algorithm).

**Results:** As of May 2025, ≥90% of eligible patient visits (N= 1356; mean age 15.3 years, SD = 1.9; 54.1% Male; 84.2% White) had documented annual PHQ‐9 screening and score in the EMR. 64% had documented disposition of PHQ‐9 results. No consistent interventions or educational resources were provided for patients with mild depressive symptoms. PDSA Cycle 1 interventions were developed and implemented but had low utilization due to workflow and systems challenges. Implementation of PDSA Cycle 2 started May 2025 with EMR integration, periodic feedback, and reminders.

**Discussion:** We consistently screen IBD patients ≥12 years of age annually for depression commensurate with ICN clinical guidelines, but there is a significant gap in follow‐up documentation and provision of appropriate interventions. Identified barriers including workflow concerns and lack of structured reminders have informed the design of targeted interventions. Planned EMR SmartPhrases and educational resources aim to improve adherence to ICN guidelines and support early, standardized intervention for depressive symptoms in pediatric IBD. Future data collection will evaluate improvements in disposition documentation and intervention.


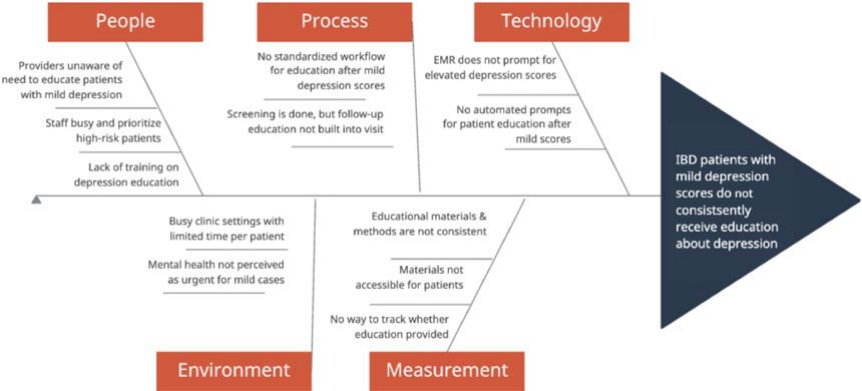


## 268 DIAPER FADING TO TREAT TOILET PHOBIA IN CHILDREN

*Andrea Begotka* ^*1,2*^

^*1*^ *Pediatrics*, *Medical College of Wisconsin*, *Milwaukee*, *WI*; ^*2*^ *Gastroenterology*, *Children's Hospital and Health System Inc*, *Milwaukee*, *WI*

**Introduction:** Constipation occurs in 68‐86% of children. Of those children, over 90% have painful stooling leading to withholding and fear of defecation. This can lead to children only defecating in a diaper because they are too afraid to use the toilet. Behavioral therapy has been shown to be an effective intervention for encopresis along with medical management. However, there has been minimal research on treating toilet avoidance or toilet phobia, with no prospective studies at this time. The purpose of the current study is to describe and demonstrate the outcomes of a prospective study using the diaper fading protocol to toilet train children without significant fear or resistance. Additionally, this study evaluates caregiver stress and family quality of life pre‐ to post‐treatment to determine the family impact of this intervention.

**Methods:** A diaper fading protocol was used to achieve continence in children with toilet phobia, resulting in them only defecating in their diaper. The protocol consists of having children defecate in a diaper while physically moving closer to and then onto the toilet. Once the child is defecating in the diaper while sitting on the toilet, the caregivers cut a slit in the diaper progressively wider, allowing stool to pass through into the toilet, until the child no longer needs a diaper at all. Fading the diaper in this manner allows the child to have the security of wearing a diaper while also gradually eliminating their fear of stooling in the toilet. Family completed the following measures pre‐ and post‐treatment: Parental Opinions of Pediatric Constipation (POOP‐C), Peds QL Family Impact Module, and the Pediatric Inventory for Parents (PIP).

**Results:** Currently, nineteen children with encopresis and their caregivers have been enrolled with five children having completed the study. The majority of participants are white (83%) males (67%) with a mean age of 6.34 years old. The majority of participants only defecate in a diaper (73%) with 27% having occasionally bowel movements in the toilet. Pre‐treatment data showed that all participants have soft, consistent bowel movements (every to every other day), but 67% also have stool leakage. Statistical analyses will be completed when at least 15 participants have completed treatment with the accompanying caregiver questionnaires. Paired t‐tests will be used to evaluate the patient toileting outcomes as well as caregiver questionnaire data pre‐ to post‐treatment.

**Conclusions:** The current study explains a novel protocol to treat toilet phobia for children with a history of constipation and/or painful defecation. Caregivers and families experience a lot of stress when their child refuses to toilet train. This project aims to demonstrate the effectiveness of this protocol to toilet train children as well as decrease caregiver stress and increase family quality of life. Further research is needed to replicate this protocol and further assist in the development of an evidenced based intervention for a condition that plagues many children and their families.


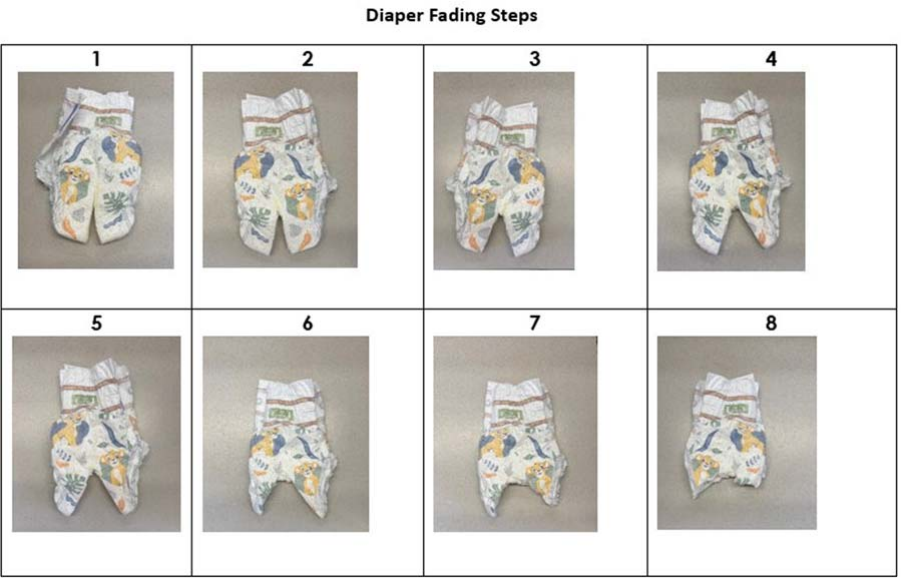


## 269 DISTRESS AMONG PARENTS AND CAREGIVERS OF PATIENTS WITH CONGENITAL COLORECTAL CONDITIONS

*Zoe Flyer* ^*1*^, *Andreina Giron* ^*1*^, *John Schomberg* ^*1*^, *Asish Chogle* ^*2*^, *Andreina Urrutia Gonzalez* ^*1*^, *Krystle Lemly* ^*1*^, *Yigit Guner* ^*1,3*^, *Donald Shaul* ^*1*^, *Hira Ahmad* ^*1,3*^

^*1*^ *Pediatric Surgery*, *Children's Hospital of Orange County*, *Orange*, *CA*; ^*2*^ *Pediatric Gastroenterology*, *Children's Hospital of Orange County*, *Orange*, *CA*; ^*3*^ *Pediatric Surgery*, *University of California Irvine*, *Irvine*, *CA*

**Purpose:** Hospital anxiety and depression occurs commonly among caregivers for patients with congenital anorectal disorders. Better understanding the prevalence is needed.

**Methods:** After IRB approval, a validated DT‐P (distress thermometer for parents) test was given to 98 caregivers of the above patients at the Pull‐Through Network (PTN) conference, asking them to rank their degree of distress from 0 (no distress) to 10 (extreme distress) within the past week in a variety of areas including practical, emotional, family/social, physical, support and communication categories. Scores greater than 4 strongly correlate to hospital anxiety, depression and parenting stress index. Diagnosis and demographic data were also collected. Participants were also asked to rank their most significant stressors with 1 being the most significant stressor and 5 was least significant.

**Results:** Among 98 caregivers that completed the survey, 90% of respondents were parents, 68% were female, and 61% were 35‐54 years old. The diagnosis groups included isolated ARM (n=61), cloaca (n=11), Hirschprung disease (HD, n=9) and VACTERL (n=17). The DT‐P scores over 4 occurred in 50% of ARM/cloaca, 35% of VACTERL and 77% of HD caregivers (p=0.03), among those completing the scoring. High proportions of surveyors reported high s tress (scores of 1‐2): anal invasive treatment such as dilations, enemas, irrigations (36%) and unexpected diagnosis (46.9%).

**Conclusions:** Anxiety and/or depression was present in over half of caregivers of congenital anorectal disorders even though respondents were asked to simply quantify their stress over the past week. Unexpected ARM or HD diagnosis in the newborn period was a source of significant stressor in almost half of the caregivers. These findings suggest that mental health interventions should be utilized early, perhaps as soon as the diagnosis of an ARM or HD is made.


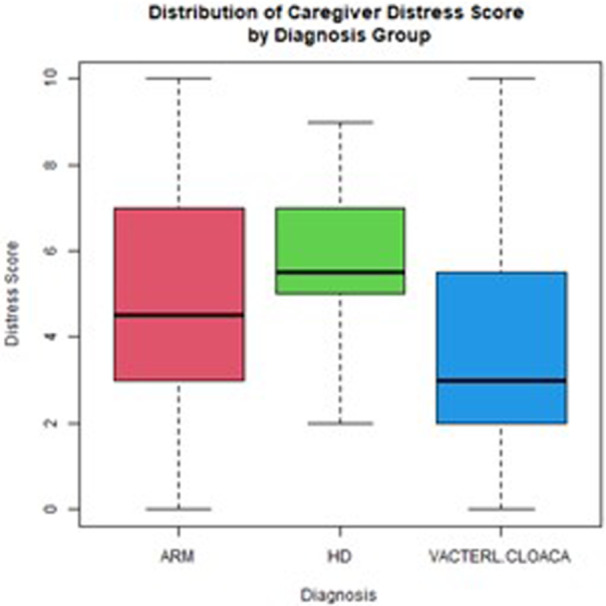


## 270 MENTAL HEALTH SCREENING IN PEDIATRIC INFLAMMATORY BOWEL DISEASE

*Erica Rabinovich* ^*2,1*^, *Stacey Jackson* ^*2*^, *Kristine Pascuma* ^*2,1*^, *Jodi Gary* ^*2,1*^, *Anu Subramony* ^*2*^

^*1*^ *Pediatric Gastroenterology*, *Northwell Health*, *New Hyde Park*, *NY*; ^*2*^ *Pediatrics*, *Donald and Barbara Zucker School of Medicine at Hofstra/Northwell*, *New Hyde Park*, *NY*

**INTRODUCTION:** Children with chronic illnesses have elevated rates of co‐morbid psychiatric disorders. Pediatric patients with Inflammatory Bowel Disease (IBD) are at increased risk for the development of anxiety and depression. Studies have shown that up to 25% of patients with IBD display symptoms of depression and that 97% would go unrecognized if not specifically screened. The Patient Health Questionnaire‐9 (PHQ‐9) is a validated depression screening questionnaire for pediatric patients and can be an important tool in identifying at risk patients.

**S.M.A.R.T. GOAL & TIMEFRAME:** At baseline, our practice did not formally screen for mental health conditions. At the initiation of this quality improvement project our aim was to increase depression screening by 50% in our pediatric IBD population over 1 year. This project was first proposed in August 2023 and in the interim, there have been three PDSA cycles spanning October 2023 to January 2025.

**METHODS:** The first PDSA cycle required workflow creation to construct a starting point for initial screening. It was presented to the Pediatric GI Division for support of the project. The initial plan was developed, and the IBD Mental Health QI Team was established, including providers, social workers (SWs) and medical office assistants (MOAs). Depression screening with PHQ‐9 questionnaires was implemented for the first PDSA cycle. With each cycle, changes were made to improve rates of screening.

Currently, the IBD Mental Health QI Team reviews the weekly schedule for eligible patients to screen. Then, the MOAs hand out the questionnaire to eligible patients at the beginning of the visit. After completion, the SW or medical provider reviews the results and input the score into the EMR. Any patient with scores from 0‐9 is rescreened in 6‐12 months. Any score > 10, a social work consult is placed to assess for active suicidal ideation (SI). A patient with active SI is immediately referred to emergency behavioral health services. A patient with scores >10 but no active SI is referred to urgent care behavioral health.

**RESULTS:** After the initial PDSA cycle, a run chart was created with pilot data to assess initial screening rates. The pilot analysis was presented to the division to readdress workflow to increase the percentage of patients screened. There were several lessons learned early on. First, is that the screening itself takes time to complete. Adjustments were made to allow patients to complete screens at the start of the visit while waiting for the provider. Initially, the social worker wasn't involved in every visit. Providers felt cognitive overload associated with adding mental health concerns to an already complex medical visit. This was addressed by assuring SW presence at every screening visit to more closely address the patient's individual mental health needs.

Data from the second PDSA cycle showed that a median of 67% of patients were screened in early 2024. The attached run chart reflects the project's third PDSA cycle. A median of 89% of patients were screened with a decrease in week‐to‐week variability. With each PDSA cycle, the screening percentages are reflective of effective improvements to the screening process. Providers note a smoother process better adapted to the divisional needs and existing workflow.

**CONCLUSION/FUTURE WORK:** We learned the importance of overlapping roles of team members to ensure the ability to create an eligible patient list, screen and score patients on a weekly basis. There was substantial increased awareness of mental health resources, which highlighted the importance of consistent SW support. We are proud of our ability to provide this crucial screening to our IBD patients and offer relevant resources to those who need it. This is reflective of our continued efforts to improve the holistic and comprehensive care we provide. Upcoming, we plan to expand our current screenings by adding an anxiety screening tool as well. Ongoing considerations include screening for other chronic GI conditions. We plan to enhance team communication and response times as well as monitor outcomes for continuous improvement in our current processes.


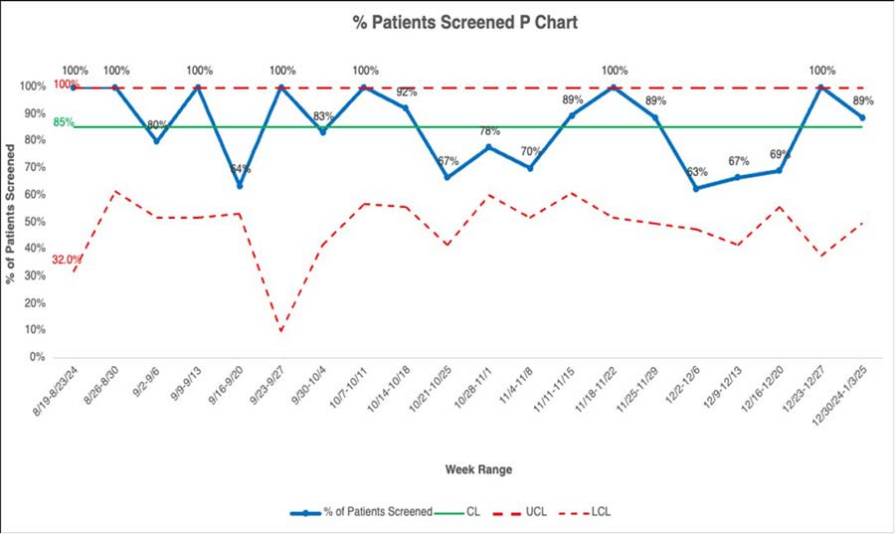


Run Chart Showing Percentage of Patient's Screened in Third (Most Recent) PDSA Cycle

## 271 EXPLORING THE FINANCIAL AND PSYCHOSOCIAL DIMENSIONS OF THE GLUTEN‐FREE DIET IN CELIAC PATIENTS

*Martina Williams* ^*1*^, *Shanaz Daneshdoost* ^*2*^, *Peacha Sokzini* ^*2*^, *Ashley Dunn* ^*2*^, *Parnia Abrishamchian* ^*2*^, *Nasim Khavari* ^*2*^, *Ritu Verma* ^*1*^, *Hilary Jericho* ^*2*^

^*1*^ *The University of Chicago Comer Children's Hospital*, *Chicago*, *IL*; ^*2*^ *Lucile Salter Packard Children's Hospital at Stanford*, *Palo Alto*, *CA*

**Background:** Strict adherence to a gluten‐free diet (GFD) is the only treatment for celiac disease (CeD), yet it is not covered by insurance despite functioning as essential therapy. This cross‐sectional pilot survey study aims to quantify the financial and psychosocial burden of the GFD on patients and families, with the goal of establishing a framework for insurance coverage of "medically‐approved gluten‐free foods.”

**Methods:** This prospective study includes two groups: 16 case families of children newly diagnosed with celiac disease (CeD) and 16 control families with a diagnosis >1 year. Case families completed a 25‐item Likert‐scale survey on demographics, changes to grocery lists over time and psychosocial impacts of adherence to a GFD in addition to standardized perceived stress and food insecurity questionnaires at diagnosis and 4 weeks after starting a GFD. They also submitted five grocery bills (pre‐GFD and at 2 weeks, 1, 3, and 6 months post‐GFD) to assess cost changes. Control families completed the same surveys and submitted one grocery bill at enrollment. Participants were recruited via email through EMR review and completed surveys via REDCap links. All participants were compensated for their time.

**Results:** Thirty‐two participants with celiac disease were enrolled (73% female; mean age 9 years, SD 3.8, mode 6), including 16 newly diagnosed and 16 on a gluten‐free diet (GFD) for >1 year (control). All control participants completed surveys and submitted one grocery receipt. Among newly diagnosed participants, 12 completed at least one survey set, 7 completed both pre‐ and post‐GFD surveys, and 9 submitted at least one grocery receipt; 2 completed all study components. In the newly diagnosed group, perceived stress appeared to decrease slightly post‐GFD while the control group reported significantly lower stress levels. Food insecurity scores were high in the newly diagnosed group and remained elevated in the control group. Grocery data indicated an initial increase in spending on processed gluten‐free products, with a subsequent shift toward naturally gluten‐free foods. A GFD was approximately 11% more expensive per unit than a gluten‐containing diet, corresponding to an estimated $666 increase in annual grocery costs.

**Conclusions:** In this study of families managing pediatric celiac disease, perceived stress decreased significantly after one year on a gluten‐free diet (GFD), indicating improved coping over time. However, food insecurity remained prevalent, and the GFD continued to cost approximately 11% more per unit than a gluten‐containing diet. Although families showed a gradual shift toward naturally gluten‐free foods, financial strain and barriers to food access persisted, even within a well‐resourced clinical environment. These findings support the need for a policy framework that enables insurance coverage of "medically‐approved gluten‐free foods” to reduce the long‐term economic burden on affected families.

## 272 CHILD HEALTH UTILITY CONTENT VALIDITY IN ADOLESCENTS WITH INTESTINAL FAILURE

*Claire Josey* ^*1*^, *Lisa Lakkis* ^*2*^, *Flor de Abril Cameron* ^*2*^, *Lane Alexander* ^*2*^, *Daniela Gattini* ^*3,4*^, *Beverly Kosmach‐Park* ^*1*^, *Janel Hanmer* ^*2*^, *Vikram Raghu* ^*1,2*^

^*1*^ *UPMC Children's Hospital of Pittsburgh*, *Pittsburgh*, *PA*; ^*2*^ *University of Pittsburgh School of Medicine*, *Pittsburgh*, *PA*; ^*3*^ *Institute of Health Policy, Management and Evaluation*, *University of Toronto*, *Toronto*, *ON*, *Canada*; ^*4*^ *The Hospital for Sick Children*, *Toronto*, *ON*, *Canada*

**Background:** Quality of life (QoL) plays an increasingly significant role in the medical care and decision‐making for children with intestinal failure, particularly as life expectancy increases. The Child Health Utility instrument (CHU9D) is a generic measure of nine QoL domains for children ages 7‐17 years which has been validated among caregivers of children with intestinal failure. This study aims to further examine QoL themes in adolescent patients with intestinal failure utilizing CHU9D domains.

**Method:** Semi‐structured interviews focused on quality of life were conducted with adolescents age 12‐26 with intestinal failure and those post‐intestine transplant from a single site. Content analysis was performed utilizing CHU9D domains.

**Results:** 15 interviews were conducted and analyzed. 40% (n = 6) of participants had previously received an intestine transplant. Without prompting, most participants suggested the importance of activities (n = 14) and daily routine (n = 10) while no participants mentioned sleep. Once prompted, all nine domains had over 70% of respondents affirming their importance. Over 90% affirmed the importance of pain, school, sleep, daily routine, and activities. When asked to indicate the most important domains, participants indicated the special significance of school (n = 11), daily routine (n = 10), activities (n = 10), and pain (n = 10). Among transplant recipients, 67% (n = 4) indicated all domains as “important,” whereas only one non‐recipient did so. The most infrequent domain cited as most important (annoyance) still had 5 participants who included it among the most important.

**Conclusion:** Adolescent patients corroborate the content validity of the CHU9D in assessing QoL in children with intestinal failure. Compared to caregivers, adolescents placed a similar emphasis on the domains of daily routine and activities but considered school and pain domains to be equally important.


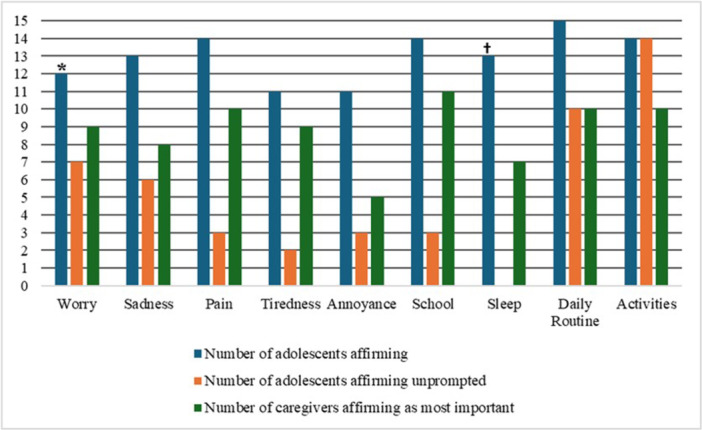


Figure 1: Quality of life domains identified by adolescent patients with intestinal failure (* = responses obtained from 14 patients; † = responses obtained from 13 patients).

## 274  A QUALITY IMPROVEMENT PROJECT TO IMPROVE ACCESS TO BEHAVIORAL HEALTH TREATMENT FOR PEDIATRIC RUMINATION SYNDROME: ASSESSING CAREGIVER‐REPORTED BARRIERS AND EDUCATION PREFERENCES

*Kelsey Jong* ^*1*^, *Sarah Mayer‐Brown* ^*1,5*^, *Jennifer Webster* ^*2,3*^, *Maura Downing* ^*4*^, *Elizabeth Maxwell* ^*2,3*^, *Kari Baber* ^*1,5*^

^*1*^ *Department of Child and Adolescent Psychiatry and Behavioral Sciences*, *The Children's Hospital of Philadelphia*, *Philadelphia*, *PA*; ^*2*^ *Division of Gastroenterology, Hepatology & Nutrition*, *The Children's Hospital of Philadelphia*, *Philadelphia*, *PA*; ^*3*^ *Department of Pediatrics, Perelman School of Medicine*, *University of Pennsylvania*, *Philadelphia*, *PA*; ^*4*^ *Population Health Innovation Team*, *The Children's Hospital of Philadelphia*, *Philadelphia*, *PA*; ^*5*^ *Department of Psychiatry, Perelman School of Medicine*, *University of Pennsylvania*, *Philadelphia*, *PA*

**Background:** Rumination syndrome (RS) is a disorder of gut‐brain interaction (DGBI) characterized by a habitual regurgitation response that is highly amenable to behavioral treatment (BT). Integrating behavioral health (BH) providers into pediatric gastroenterology (GI) settings improves access to BT; however, barriers limit utilization, particularly for historically marginalized populations. Previous quality improvement (QI) efforts have identified GI provider‐reported barriers to referring patients with RS to an integrated BH program (GI Psychology), including geographic distance, families declining the referral, insurance issues, appointment availability, and limited time to educate families about BT for RS (Mayer‐Brown et al., 2023). The current project aimed to expand on an ongoing QI initiative to increase referrals to GI Psychology for BT for RS (Mayer‐Brown et al., 2024) by obtaining caregiver perspectives on education materials and referral processes.

**Methods:** The ongoing QI initiative began with a root cause analysis of variation in referrals to GI Psychology for BT. The root cause analysis identified primary and secondary drivers, including several related to caregiver preferences and perspectives about BT referral. The current project used two interventions from the ongoing QI initiative: standardized education materials and an electronic medical record (EMR)‐based population health management tool (Mayer‐Brown et al., 2024).

Referral and visit status were obtained from the EMR‐based tool. Patients were eligible for caregiver outreach if they met the following criteria: (1) referred to GI Psychology for RS between March 20, 2024 and March 20, 2025, (2) had not completed GI Psychology visit as of May 15, 2025 and (3) the reason for not being seen by GI Psychology was unknown. Following standard clinic outreach practices, the lead author (KJ) attempted to contact eligible caregivers via two phone calls and one EMR message.

A semi‐structured interview guide was developed in REDCap by the lead author. Telephone interviews were conducted with caregivers to explore: (1) experiences and preferences for receiving RS education, (2) awareness and acceptability of the GI Psychology referral, and (3) perceived barriers and facilitators to initiating GI Psychology services for RS. A thematic analysis was used to identify key themes, which were included in the results if endorsed by two or more caregivers.

**Results:** Ninety‐six patients were newly diagnosed with RS and 49 (51%) of these patients were referred for GI Psychology for RS. Of those referred, 28 (57%) had at least one GI Psychology visit after RS diagnosis. Sixteen patients not seen by GI Psychology were eligible for outreach. Three patients were excluded from outreach due to an upcoming GI Psychology visit, and two were excluded due to either declining the GI Psychology referral or inappropriate referral. Interviews were conducted with four caregivers, all of whom identified as mothers. Six key themes emerged:

***1.Gaps in education delivery (n = 3, 75%)***: Although all caregivers were given education materials about RS via their GI provider or the EMR, most reported not receiving or reviewing these materials.

***2. Education preferences (n = 4, 100%):*** Caregivers expressed interest in receiving both printed and electronic RS education materials.

***3. High acceptance of BH referral (n = 4, 100%):*** Caregivers reported that the referral to GI psychology for RS was appropriate and would be helpful in managing RS.

***4. Impact of co‐occurring BH diagnoses (n = 4, 100%):*** Co‐occurring conditions, such as autism spectrum disorder and eating disorders, often took precedence over RS or treatment for these conditions was more accessible, reducing the perceived importance or urgency of pursuing RS‐specific treatment.

***5. Caregiver reassurance about the RS diagnosis (n = 2, 50%):*** Receiving a diagnosis of RS and education that it was not physically harmful provided caregivers with relief, reducing anxiety about their child's symptoms.

***6. Scheduling delays (n = 2, 50%):*** Caregivers reported difficulties in accessing timely new patient appointments, which served as a barrier to initiating GI Psychology services.

**Conclusions:** While embedding BH providers in GI settings may increase access and acceptance of BT for RS, barriers remain that limit treatment utilization. Some caregiver‐reported barriers echoed those previously reported by providers, such as appointment availability. However, new themes emerged, including gaps in education delivery, the prioritization and greater accessibility of treatment for co‐occurring BH conditions, and caregiver reassurance about RS symptoms after diagnosis. These findings highlight the need for tailored, timely education, and streamlined referral processes. In the next stage of the ongoing QI project, efforts will be made to improve gaps in education, outreach, and scheduling processes to address these issues.

## 275 ONE CLICK CLOSER: PROVIDER FEEDBACK ON THE IMPLEMENTATION OF AN E‐REFERRAL FOR SPECIALIZED PEDIATRIC PSYCHOLOGY SERVICES WITHIN GASTROENTEROLOGY

*Morgan Drake* ^*3,1*^, *Catherine Naclerio* ^*2,1*^, *Monique Germone* ^*2,1*^, *Julie Rinaldi* ^*2,1*^, *Christine Reinhard* ^*2,1*^

^*1*^ *Children's Hospital Colorado*, *Aurora*, *CO*; ^*2*^ *University of Colorado Anschutz Medical Campus School of Medicine*, *Aurora*, *CO*; ^*3*^ *University of Colorado School of Medicine Colorado Springs Branch*, *Colorado Springs*, *CO*

**Introduction:** Youth with gastrointestinal (GI) conditions are at increased risk for psychosocial difficulties. GI psychologists receive specialized training in treating youth with GI conditions and are often integrated into GI clinics to help address psychosocial needs. Research indicates that medical clinics with integrated psychological support experience increased patient and provider satisfaction and reduced healthcare costs. Although integrating psychology can be beneficial, it is imperative that systems are created to streamline communication and connect patients with the appropriate services. Poor communication between providers can lead to confusion, frustration, and delays in care. Internal eReferrals can be an efficient and effective means for connecting GI patients with pediatric GI psychologists. The current project examines provider feedback regarding the implementation of an eReferral for integrated GI psychology services.

**Methods:** Prior to the new eReferral, GI medical providers referred patients to GI psychology verbally, with a message in the electronic medical record, or have the patient directly call to schedule, which often led to confusion and miscommunication. The GI psychology team created an eReferral to streamline referrals from GI medical providers to the integrated GI psychology team. GI medical providers and GI psychologists at a large academic children's hospital were surveyed regarding implementation of the new internal eReferral. The online survey utilized a five‐point Likert rating scale and included areas of free text for additional comments. Descriptive statistics and thematic analysis were utilized to assess provider perceptions of the eReferral.

**Results:** Twenty GI medical providers (physicians, nurse practitioners, and physician assistants) and four GI psychologists in a pediatric GI department completed the survey. 95% of medical providers (Figure 1) and 100% of psychologists strongly agreed or agreed that the eReferral integrated smoothly within their workflow. 90% of medical providers strongly agreed or agreed that the eReferral allows them to clearly communicate their referral question (Figure 2), while 100% of the psychologists strongly agreed or agreed the eReferral effectively communicated the provider's reason for referral. 10% of medical providers (Figure 3) and 0% of psychologists reported needing to further follow‐up with eReferrals due to technological system glitches or missing information. 100% of psychologists strongly agreed that the eReferral facilitated communication between themselves and the medical provider. Themes included scheduling follow‐up, more streamlined communication between providers, and increased perception of psychologist effectiveness and efficiency.

**Conclusion:** Communication between medical and psychological providers is imperative for effective and timely care. Results from this study indicate that GI medical providers and psychologists believe that an electronic eReferral is an effective and helpful way to refer GI patients to integrated psychology and improves communication between specialties. Although the eReferral was found to be helpful, respondents identified several areas for improvement, including technological glitches, simplification of the referral, and follow‐through on patient scheduling.


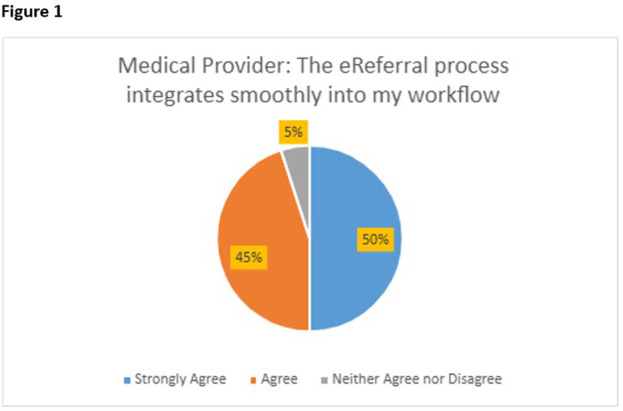



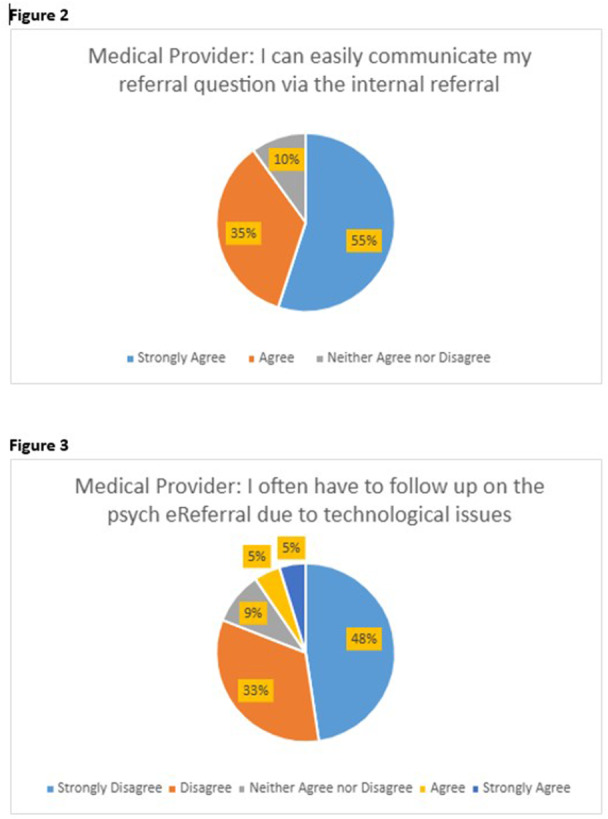


## 276 DESIGN AND IMPLEMENTATION OF A MULTIDISCIPLINARY PATIENT SUPPORT PROGEAM FOR PEDIATRIC FOOD ALLERGY AND INTOLERANCES (FAIT)

*Aliza Solomon*, *Liza Goldberg*, *Ayelet Goldberg*, *jennifer lentine*, *Arielle Bergman*

*Pediatric Gastroenterology Hepatology and Nutrition*, *Weill Cornell Medicine*, *New York*, *NY*

**Background:** Eosinophilic gastrointestinal diseases (EGIDs) and celiac disease are chronic, immune‐mediated food related conditions requiring complex dietary and medical management. These diseases impact nutritional status, social functioning, and emotional well‐being in children and their families. While diagnostic strategies have advanced, there remains a significant gap in ongoing patient education, psychosocial support, and coordinated care following diagnosis.

**Objective:** To design, implement, and evaluate a comprehensive, multidisciplinary patient support program for children and adolescents with EGIDs and celiac disease in a tertiary pediatric care setting.

**Methods:** A team comprising pediatric gastroenterologists, registered dietitians, social workers, and coordinators developed a structured support program targeting patients newly diagnosed or with established disease. Program components included structured group education sessions, peer support initiatives, and digital tools for disease self‐management. Outcome measures included patient and caregiver satisfaction surveys.

**Results:** Survey was sent to identified families to gauge interest. 85% respondents were interested in participating (23/27) with interest in in person events (8.7%), virtual events (47.8%) and both formats (43.5%). In the initial 18‐month period, 82 families participated in events during the program. Events were both in person hands on activities or virtual seminars. Topics included were Navigating Social Situations, Kids in the Kitchen: Cooking Demo, Night of Advocacy, Decorate a Teal Pumpkin to indicate a food awareness concern for Halloween, and Navigating Food‐Related Choices & Accommodations in College and Beyond. Satisfaction rates were high with 100% reporting they would attend more events.

**Conclusion:** This multidisciplinary patient support model offers meaningful benefits for pediatric patients with EGIDs and celiac disease, addressing unmet needs in disease education, mental health, and care navigation. The program shows promise for replication in other clinical settings and may offer opportunity to impact quality of life. Additional measures to be collected in the future to include health‐related quality of life (HRQoL), dietary adherence, and frequency of unscheduled clinical contacts.

## 277  A CROSS‐SECTIONAL STUDY OF SLEEP DISTURBANCES IN CHILDREN AND ADOLESCENTS WITH ABDOMINAL PAIN‐ASSOCIATED DISORDERS OF GUT‐BRAIN INTERACTION

*Pierce Thompson* ^*1,2*^, *Hunter Friesen* ^*4*^, *Jennifer Schurman* ^*2,3*^, *Jennifer Colombo* ^*2,3*^, *Craig Friesen* ^*2,3*^

^*1*^ *Student*, *Kansas City University*, *Kansas City*, *MO*; ^*2*^ *Children's Mercy Kansas City Division of Pediatric Gastroenterology*, *Kansas City*, *MO*; ^*3*^ *University of Missouri‐Kansas City School of Medicine*, *Kansas City*, *MO*; ^*4*^ *The University of Kansas School of Medicine*, *Kansas City*, *KS*

The aims of the current study were to determine the frequencies of specific sleep disturbances in youth with abdominal pain‐associated disorders of gut‐brain interaction (AP‐DGBIs) and to assess relationships with psychological dysfunction. This was a retrospective evaluation of 226 consecutive patients diagnosed with an AP‐DGBI. All had undergone a systematic evaluation of gastrointestinal symptoms, the Sleep Disturbance Scale for Children, and the Behavior Assessment System for Children. Disorders of initiation and maintenance of sleep (DIMS; 40%) and disorders of excessive daytime somnolence (DOES; 14%) were each present in more than 10% of the patients. Both DIMS and DOES scores were more likely to be elevated in patients with anxiety and/or depression scores in the at‐risk or elevated‐risk ranges. Sleep disorders are common in youth with AP‐DGBIs and are associated with anxiety and depression, even in those patients with anxiety and depression in the at‐risk range.


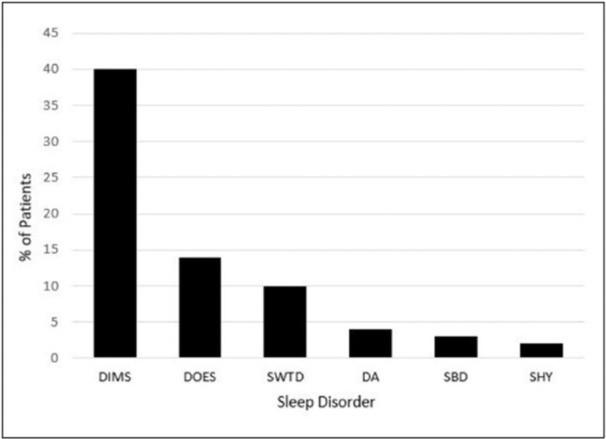


Figure 1. Frequency of elevated subscales of the Sleep Disturbance Scale for Children (SDSC) in youth with abdominal painassociated disorders of gut‐brain interaction. Abbreviations: DIMS, disorders of initiating and maintaining sleep; DOES, disorders of excessive somnolence; SWTD, sleep‐wake transition disorders; DA, disorders of arousal/nightmares; SBD, sleep breathing disorders; SHY, sleep hyperhidrosis


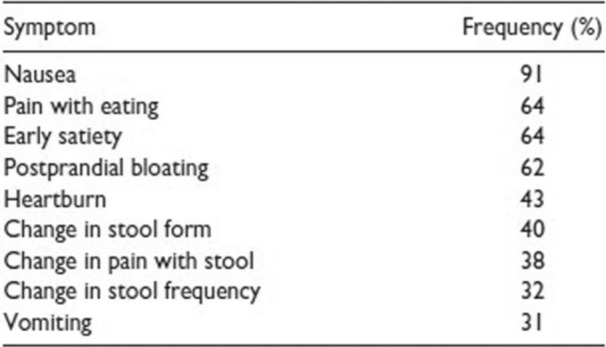


Table 1. Frequency of Gastrointestinal Symptoms in Youth With Abdominal Pain‐Associated Disorders of Gut‐Brain Interaction.

## 278 HARNESSING THE ELECTRONIC HEALTH RECORD FOR IMPLEMENTATION OF CYSTIC FIBROSIS HEPATOBILIARY INVOLVEMENT GUIDELINES: FROM CLASSIFICATION TO SCREENING

*Keishla Valentin‐Martinez* ^*1*^, *Alexandra Pottorff* ^*2*^, *Prigi Varghese* ^*2*^, *Lauren Lazar* ^*2*^, *Derek Ngai* ^*2*^, *Meghana Sathe* ^*2*^

^*1*^ *Pediatrics*, *The University of Texas Southwestern Medical Center*, *Dallas*, *TX*; ^*2*^ *Pediatric Gastroenterology*, *The University of Texas Southwestern Medical Center*, *Dallas*, *TX*

**BACKGROUND:** In 2024, new guidelines for screening and management of liver disease for people with Cystic Fibrosis (PwCF) were published. A new classification system was established to differentiate the presentation and severity of liver involvement, now termed CF hepatobiliary involvement (CFHBI). These updates aimed to improve care and understanding of CFHBI, but also presented challenges to CF care centers, patients, and families. Our 284‐person center is co‐directed by gastroenterology (GI) and pulmonology. Although GI care is well‐integrated in our center, we still noted deficits in liver screening. In 2024, our center launched a Quality Improvement (QI) project with the global aim of implementing the standardized CFHBI classification to enhance screening, evaluation, and management of CFHBI. An Electronic Health Record (EHR)‐based SmartForm tracked CFHBI classification, imaging screening, and prior liver disease evaluation. This intervention successfully led to increased identification of CFHBI, but revealed gaps in subsequent evaluation, specifically in biochemical testing. In response, we created a new Specific Measurable Attainable Realistic Timely (SMART) aim: increase evaluation for underlying liver disease in PwCF with CFHBI from 9% to 25% over the next 5 months.

**METHODS:** We used several EHR‐based interventions to incorporate new CFHBI guidelines. Specific QI tools, including a Process Map, Key Driver Diagram, and System Failure Mode and Effects Analysis, guided our iterative plan‐do‐study‐act (PDSA) cycles. During PDSA 1, we reviewed all PwCF to assess if CFHBI screening had been performed, and if further evaluation for underlying liver disease was needed. The above information was recorded in a shared “sticky” note, an EHR‐based tool. PDSA 2 will implement use of the “sticky” note in daily clinic workflow amongst all CF team members. PDSA 3 will focus on completing CFHBI classification for patients not seen by GI and identifying patients who need additional evaluation. PDSA 4 will involve distribution and discussion of a co‐produced education sheet to promote understanding of CFHBI and the screening and monitoring of such in PwCF.

**RESULTS:** Prior to new CFHBI classification and interventions, only 19 (7%) of patients in our clinic were identified to have CF related liver disease. After implementation of the EHR SmartForm, 206 (72.5%) patients were screened for CFHBI (Table 1). Of those screened, 64 (31.2%) met criteria for CFHBI and 7 (3.4%) for advanced CF liver disease (aCFLD) with 65 (90.2%) having a partial evaluation for underlying liver disease. Only 7 (9.7%) patients had the full recommended evaluation, and 4 (6%) had confirmed underlying liver disease. Thyroid testing, celiac screening, and alpha‐1 anti‐trypsin phenotyping were the tests most reliably obtained, occurring between 35‐50% each (see Table). Other testing occurred less reliably. For example, only 10 (14%) patients were screened for infectious hepatitis. We will continue to monitor these metrics with future PDSAs.

**CONCLUSIONS:** The integration of EHR tools to reliably screen and evaluate CFHBI has led to significant improvements in documentation and recognition of CFHBI in PwCF. Gaps in completion of non‐CF liver disease evaluation persist even in a CF center with significant GI involvement. Our continued QI efforts are aimed at closing gaps over the next 5 months and identifying strategies for implementation to share with the greater CF community.


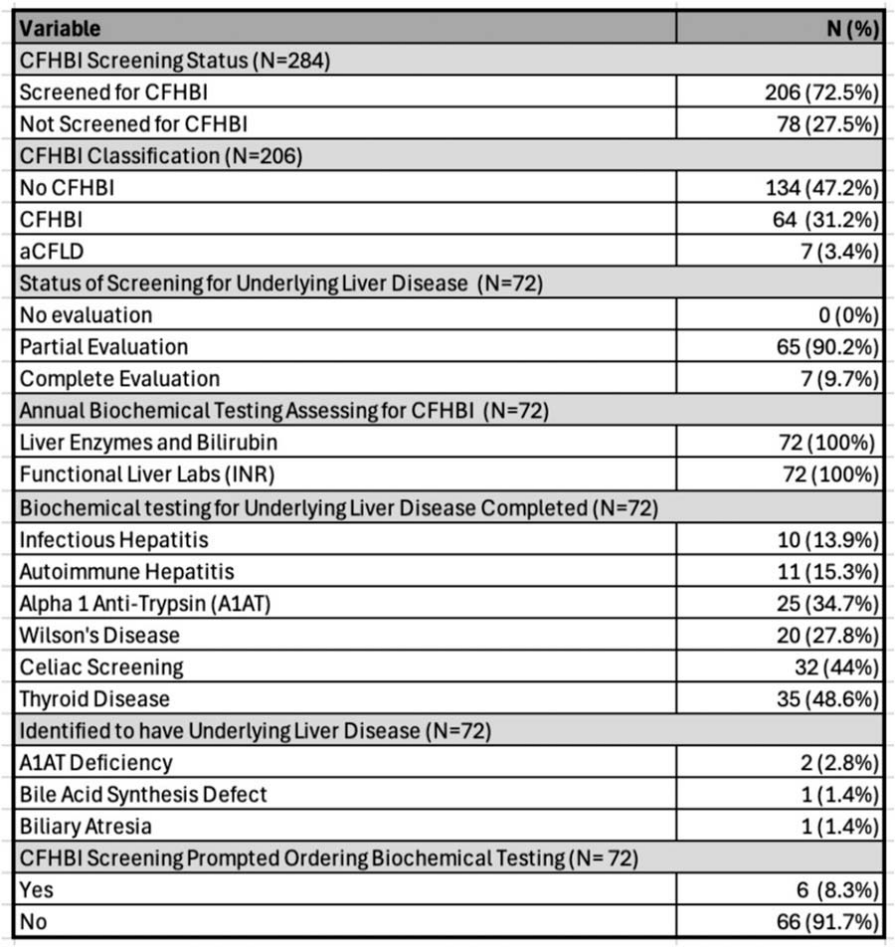


## 279 RISING FOOD‐RELATED ADVERSE EVENTS IN U.S. CHILDREN (2004‐2024): THE URGENT NEED FOR BETTER DOCUMENTATION AND INFORMATION SHARING

*Mary Abigail Garcia* ^*1,2*^, *Mandeep Bajwa* ^*1,2*^, *Jeannie Huang* ^*1,2*^

^*1*^ *Pediatric Gastroenterology*, *University of California San Diego*, *La Jolla*, *CA*; ^*2*^ *Rady Children's Hospital‐San Diego*, *San Diego*, *CA*

Currently there is no standard mechanism for reporting dietary or food associated adverse events in healthcare institutions. The U.S. Food and Drug Administration (FDA) has an adverse event reporting system managed by the Center for Food Safety and Applied Nutrition (CFSAN).The CFSAN Adverse Event Reporting System (CAERS) contains information on adverse event reports submitted for foods, dietary supplements, and cosmetics.

Using CAERS data, we aimed to A) identify time trends related to dietary or food‐related adverse events in children, and B) identify product and patient characteristics associated with poor outcomes.

CAERS includes adverse event data voluntarily reported by consumers and healthcare practitioners, as well as data from mandated industry reports, covering the period from January 2004 to March 2024. Reported outcomes included death, hospitalization, and serious adverse events (SAEs), which encompass disability, hospitalization, and death. Events are further characterized as suspect (reasonably associated) or concomitant (indicates another potential cause present at the same time).

To obtain pediatric‐specific data, CAERS data were limited to reports involving youth under 18 years. Descriptive statistical analyses were performed, and the volume of serious adverse events (SAEs) was plotted over time for events from 2004 to 2023 (Figure 1). Group comparisons were performed using chi‐squared and Wilcoxon rank‐sum tests.

A total of 8,814 CAERS adverse events were reported among youth under 18 years during the study period. The majority of these events were classified as suspect (N=7,950, 90.2%). Among these, there were 152 reported deaths, 1,388 hospitalizations, and 5,058 SAEs. The median age of the affected youth was 5 years, with a range from 0.003 to 17.96 years. Of the 8,109 cases reporting sex, 4,026 involved females and 4,083 involved males.

Youth who died in these events were younger than those survived (median (range): 5 (1, 17) v. 7 (1, 17.96) years, death v. survival, p=0.03). In contrast, there were no significant age differences by occurrence of SAE or hospitalization (both P>0.05). Overall, male youth were more likely to suffer serious adverse events (51.7% male v. 48.3% female, P<0.01), but not when those events were further characterized by hospitalization or death (P>0.05).

The volume and severity of events increased over time (Figure 1). The greatest number of serious adverse events (N=1,053) occurred in 2022.

Product categories most associated with deaths, hospitalizations, and serious adverse events were vitamins/minerals, infant formula, and baby products (Table 1).

Adverse events related to food and dietary products, reported through the FDA's CAERS, have increased over the past two decades. These events highlight the need for better healthcare system documentation and information‐sharing to ensure quality care and patient safety.


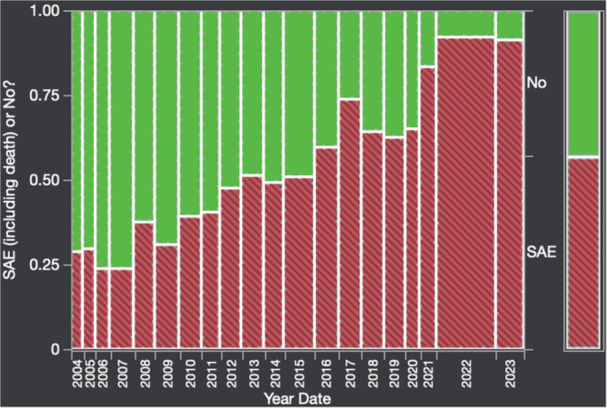


FIGURE 1. Serious Adverse Event (SAE) Proportions by Year (2004‐2023)


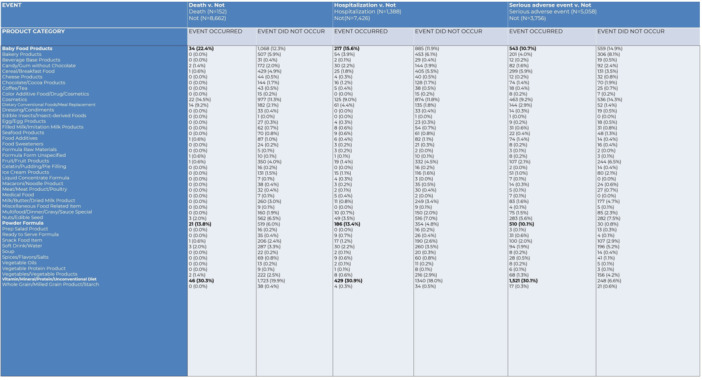


TABLE 1. Serious Adverse Events and Associated Outcomes. Events Reported as N (%).

## 280 EVALUATING ACCURACY, RELIABILITY, AND INTERPRETABILITY OF LARGE LANGUAGE MODEL RESPONSES TO PEDIATRIC CONSTIPATION QUESTIONS IN ENGLISH AND SPANISH

*Serena Haver* ^*1*^, *Erica Yi* ^*1*^, *Rakshana Selvarajan* ^*1*^, *Evangelos Katsanos* ^*1*^, *Iris Jo‐Shi* ^*1,2*^

^*1*^ *Pediatrics*, *Inova Fairfax Hospital*, *Falls Church*, *VA*; ^*2*^ *Pediatric Specialists of Virginia*, *Fairfax*, *VA*

**Background and Objectives:** Large language models (LLMs), such as ChatGPT and Google's AI Overview, have the potential to improve patient communication and education. However, few studies have evaluated the accuracy, reliability, and cross‐language performance of LLMs in pediatric gastroenterology. We aimed to (1) assess the reliability and accuracy of responses to common patient questions about pediatric constipation from ChatGPT‐4o, Google's AI Overview, and NASPGHAN's GIKids.org; and (2) explore perceptions of LLMs among pediatricians and pediatric gastroenterologists.

**Methods:** Four patient‐facing questions were selected from GIKids.org and adapted with the phrase “in children” to direct responses toward pediatric care. Three independent responses per question were generated using ChatGPT‐4o in new chat windows, and one response was retrieved from Google's AI Overview using an account created for the purpose of this study. Two resident physicians and one board certified pediatric gastroenterologist evaluated the (1) accuracy of responses from all three sources, (2) consistency of ChatGPT‐4o's multiple responses (reliability), and (3) response to use in patient care. We then conducted a cross‐sectional survey of Spanish‐speaking pediatricians and English‐speaking gastroenterologists who rated ChatGPT‐4o generated responses on a 5‐point scale (1 = neither accurate, relevant, nor interpretable; 5 = accurate, relevant, interpretable, and complete).

**Results:** Accuracy of the responses to the patient‐facing questions was 100% across all sources for all 4 questions. ChatGPT‐4o responses were rated as the most preferred by evaluators 93% of the time, with “organized and complete information” as the most cited reason. ChatGPT‐4o demonstrated internal reliability across its three responses per question. On average, Spanish‐speaking Pediatricians rated Spanish responses a 3.7 out of 5, and English‐speaking pediatric gastroenterologists rated English responses a 3.8 out of 5.

**Conclusions:** LLMs like ChatGPT‐4o and Google's AI Overview can provide accurate and reliable responses to patient questions about constipation. Clinicians frequently preferred ChatGPT responses over existing patient education websites due to completeness and organization. However, limitations in interpretability and completeness were evident in both Spanish and English responses. Future work will survey Spanish and English‐speaking families to assess familiarity and use of LLMs, as well as its utility in their diagnostic and management journey.

Declaration of Generative AI and AI‐assisted technologies in the writing process:

During the preparation of this work the author(s) used ChatGPT for editing. After using this tool/service, the author(s) reviewed and edited the content as needed and take(s) full responsibility for the content of the publication.

## 281 DEVELOPMENT AND IMPLEMENTATION OF PEDIATRIC AMBULATORY PARENTERAL NUTRITION PRESCRIBING VIA THE ELECTRONIC HEALTH RECORD

*Micah Morris*, *Meighan Marlo*, *Shawn Pierson*, *Zach Thompson*, *Ethan Mezoff*

*Nationwide Children's Hospital*, *Columbus*, *OH*

**Introduction/Background:** Parenteral nutrition (PN) remains a high‐risk therapy, typically containing more than 40 ingredients. PN is most commonly prescribed in the inpatient setting but in certain populations, notably patients with intestinal failure, ambulatory prescribing is performed. Incorporation of PN prescribing into the electronic health record (EHR) has been recommended to minimize the risk of transcription errors and allow for implementation of important safety alerts for prescribers. Ambulatory PN prescribing workflows typically require manual transcription of orders without robust EHR interoperability between systems used for transitions of care. Pediatric patients receiving ambulatory PN have an additional increased risk of medication events due to the need for weight‐based customization. Development and implementation of a prescribing system incorporating inpatient and outpatient functionality within the EHR is necessary to improve the quality and safety of ambulatory PN in pediatric patients. The primary goal was to create a workflow that improved transitions of care and safety through minimization of manual transcription. We describe modification of standard EHR tools to achieve this aim.

**Methods:** Utilizing a multidisciplinary team, development and incorporation of ambulatory PN prescribing within the EHR at Nationwide Children's Hospital was completed. Inpatient and outpatient variances, safety parameters to provide appropriate alerts to prescribers, and legal requirements were considered and evaluated for optimization within the new system.

**Results:** The final product successfully incorporated ambulatory PN prescribing while allowing seamless transfer of prescriptions between care settings. Patient‐specific ambulatory PN can now be ordered through the EHR in an inpatient or outpatient encounter. The order is subsequently queued for pharmacist review/verification to assess the adjustments and determine extended stability considerations in the ambulatory setting. After pharmacist review, the prescription prints and is signed by the provider to be faxed to the filling pharmacy.

**Conclusion:** To our knowledge, this is a novel solution developing and incorporating pediatric PN prescribing into the EHR that transfers between the inpatient and ambulatory settings independent of manual transcription while still allowing for customization of PN.

## 282 ONDANSETRON USE IN PEDIATRIC ACUTE GASTROENTERITIS DEMONSTRATES HIGH SAFETY AND TOLERABILITY IN REAL‐WORLD EVALUATION USING A LARGE‐SCALE DATASET (TRINETX)

*Samyuktha Sivakumar* ^*1*^, *Melissa Ramirez Escobar* ^*1*^, *Charlotte Banayan* ^*1*^, *Thomas Wallach* ^*2*^

^*1*^ *Pediatrics*, *SUNY Downstate Health Sciences University*, *New York*, *NY*; ^*2*^ *Pediatric Gastroenterology*, *SUNY Downstate Health Sciences University*, *Brooklyn*, *NY*

**Background:** Ondansetron is an antiemetic medication used to treat nausea and vomiting by blocking both the central and peripheral 5‐HT3 Serotonin receptors. Previous work has raised concerns about the side effect profile and safety in pediatric use.

**Methods:** We conducted a retrospective quantitative cohort study using TriNetX Database. Children from 2 to 18 years of age who required emergency department services between January 1, 2024, and December 31, 2024, who were treated with Ondansetron, were age‐stratified into cohorts. Children below the age of 1 year were excluded from the study. Inclusion criteria included ICD‐10 diagnoses of vomiting, diarrhea, and viral gastroenteritis. Outcomes included the incidence of common adverse effects of Ondansetron, such as headache, fatigue, and constipation, within 1 month of administering Ondansetron, and the serious adverse effects of QT prolongation and unspecified arrhythmias, within 24 hours of receiving the medication. Chi‐squared analysis was used to assess for significance.

**Results:** Pediatric patients from 2‐18 years of age from 66 healthcare organizations were categorized into different age groups: 2‐5 years, 6‐11 years, and 12 – 18 years. A total of 3,204,018 patients were studied in the 2‐5 age cohort, 6,632,809 patients in the 6‐11 age cohort, and 8,833,999 patients in the 12‐18 age group. The incidence of common adverse effects of Ondansetron in children was not significantly different between groups, except for the 2‐5 year age range for which associated drug effects were statistically lower (p = 0.0036). Incidence for patients with AGE receiving ondansetron was 1.04 per 1000 patients, 0.659 per 1000 patients, and 0.429 per 1000 patients, compared with 1.68 per 1000 patients, 1.02 per 1000 patients, and 0.676 per 1000 patients in patients with AGE not receiving ondansetron. The incidences of QT prolongation in the above age groups were 0.015 per 1000 patients, 0.01 per 1000 patients, and 0.021 per 1000 patients compared with 0.04 per 1000 patients, 0.02 per 1000 patients, and 0.036 per 1000 patients in patients with AGE not receiving ondansetron. These results did not significantly statistically differ.

**Conclusion:** This large real‐world study demonstrated that use of ondansetron in the setting of pediatric AGE was not associated with any increased risk in the surveyed domains and may, in fact, have a protective effect in smaller children. This finding aligns this medication easily with the safety and tolerability listed in multiple over‐the‐counter drugs. This data is highly reassuring regarding the safety and tolerability of the use of Ondansetron in pediatric patients above the age of 1.

## 283 TITLE: CHARACTERIZATION OF CURRENT TRENDS IN PEDIATRIC ACUTE GASTROENTERITIS MANAGEMENT USING A LARGE‐SCALE NATIONAL DATASET

*Samyuktha Sivakumar* ^*1*^, *Charlotte Banayan* ^*1*^, *Melissa Ramirez Escobar* ^*1*^, *Thomas Wallach* ^*2*^

^*1*^ *Pediatrics*, *SUNY Downstate Health Sciences University*, *New York*, *NY*; ^*2*^ *Pediatric Gastroenterology*, *SUNY Downstate Health Sciences University*, *Brooklyn*, *NY*

**Background:** Acute gastroenteritis is a common diagnosis encountered in the pediatric population that necessitates frequent visits to the emergency department. The symptoms are managed by fluid restoration, correcting electrolyte imbalance, and medications to control nausea and vomiting using antiemetics like Zofran. Global guidance in AGE management (WHO guidelines and Guarino et al, ESPGHAN Guidelines 2014) recommends the use of oral rehydration and antiemetic therapy, but historically, US practice has been more reliant on IV therapy with more limited use of antiemetics.

**Methods:** Retrospective quantitative analysis was done using TriNetX database. The inclusion criteria consisted of children from 0‐18 years of age who received emergency department services for vomiting, dehydration, or acute gastroenteritis with intravenous fluids, intravenous electrolytes mixed in fluid, or Ondansetron. The time was set from January 1, 2024, to December 31, 2024. Patients with an AGE/Diarrhea/Vomiting ICD10 code who did not receive IV fluids were categorized as receiving oral rehydration.

**Results:** Patients were categorized according to their age, with 829,035 within 0‐1 years of age, 9,836,827 of them aged 2‐11 years, and 8,833,999 in the 12 – 18 years age group. Analysis of these cohorts revealed that a majority of patients with vomiting, diarrhea, or gastroenteritis presenting to the emergency room were treated with intravenous fluids and electrolytes, with 69%, 63%, and 78%, respectively. Of these, 60% or more patients received sodium chloride with additional electrolytes (LR vs. electrolyte additive. 77% of patients aged between 0‐1 received gastrointestinal medication, while 95% of patients in the 2‐11 years cohort and 97% of patients in the 12‐18 years cohort received the same. In the newborns and infants’ group, 63% received Ondansetron, 17% got hyperosmolar laxatives, 16% got Famotidine, 11% got Simethicone, and 6% got Sodium bicarbonate. Ondansetron was the drug of choice, with 91% of children between 2‐11 years of age and 94% of children aged 12‐18 receiving it. Laxatives were the second‐most preferred medication, with 32% between 2‐11 years and 46% between 12‐18 years receiving it. There was also an uptrend in usage of Famotidine with increasing age, where 15% in the 2‐11 years group and 28% in the 12‐18 year group got the drug. Interestingly, a dip in usage of Omeprazole was noted in the 2‐11 year group, with only 5%, but an uptick was seen in the 12‐18 year group, with 17% of them getting the medication.

**Conclusion:** Clinical practice in North American ED settings has moved to incorporate more pharmacologic management of AGE than previously reported, however, IV rehydration remains the mainstay of ED setting AGE therapy. It is generally clear in other work that IV fluid resuscitation in AGE is both more expensive and associated with no clinical superiority, but longer hospital stays (Hartling et al, *Cochrane Reviews* 2006). Our study is limited by the inability to assess attempts at oral rehydration, as no relevant code exists.

## 284 OPTIMIZING DISACCHARIDASE TESTING RESULTS THROUGH TARGETED INTERVENTIONS

*Stephanie Bou‐Anak* ^*1*^, *Kevin Watson* ^*2*^, *Matthew Wyneski* ^*2*^, *Reinaldo Garcia* ^*2*^, *Christine Carter‐Kent* ^*2*^

^*1*^ *Pediatrics*, *Akron Children's Hospital*, *Akron*, *OH*; ^*2*^ *Pediatric Gastroenterology*, *Akron Children's Hospital*, *Akron*, *OH*

**Introduction:** Intestinal disaccharidase analysis is the gold standard test for disaccharidase deficiency in patients with symptoms suspicious for carbohydrate intolerance. This test is a multi‐step process, starting with an esophagogastroduodenoscopy (EGD) with duodenal tissue biopsy collection, followed by specimen storage and shipment to a laboratory for processing of disaccharidase enzyme quantification. Of note, proper storage of the disaccharidase enzymes is crucial to maintain the enzyme integrity for accurate testing. During a regular quality control review within Akron Children's Hospital (ACH) pediatric gastroenterology department, there was discovery of a higher‐than‐average percentage of cases with disaccharidase analysis showing pan‐disaccharidase insufficiency, suggesting inadequate results. This led to an investigation of the processes related to collection, storage and shipment of disaccharidase specimens, implementing changes to reinforce or correct processes involved in the test acquisition.

**Methods:** This quality improvement initiative utilized the Model for Improvement framework to assess the processes involved in disaccharidase analysis. This timeline of this project was 14 months. Our interventions included updating our institutional disaccharidase collection guidelines, educating the gastroenterologists and surgical staff about the guidelines, including specimen collection and storage, and optimizing shipment procedures with our recipient laboratory. The sample collection tube was downsized for improved sample storage in media. Additionally, we recorded the biopsy forceps size to monitor for possible effects on biopsy specimens. The primary aim of this quality improvement project is to increase the accuracy of disaccharidase data collected during EGD at all Akron Children's Hospital endoscopy locations by decreasing the percentage of inadequate resulting tests from 42% to 15% by December 1, 2025.

**Results:** During the timeline from January 31, 2024 to March 31, 2025, there were 493 cases with disaccharidase analysis, with an average of 28% of cases with inadequate results during the project timeline. The final process change led to a remarkable downtrend from 41% to 17% of cases with inadequate results. This considerable improvement was largely due to the final process change of reinforcing specimen storage on dry ice immediately following collection, with complete submersion of the sample collection tube to ensure enzyme integrity within the sample. Of note, a Pareto analysis determined no significant change between the various ACH endoscopy sites and inadequate disaccharidase testing results.

**Conclusion:** This quality improvement initiative at Akron Children's Hospital investigated the process map of disaccharidase analysis from specimen collection to laboratory shipment, reviewed and implemented key process changes, and achieved the project goal of mitigating the accuracy of disaccharidase data and decreasing the number of cases with inadequate disaccharidase testing results.

## 285 PROMOTING WELLBEING BY CREATING ELECTRONIC HEALTH RECORD EFFICIENCY AT THE CHILDREN'S HEALTH SYSTEM OF TEXAS

*Rinarani Sanghavi* ^*1*^, *Derek Ngai* ^*1*^, *Philip Bernard* ^*2*^

^*1*^ *Pediatrics*, *The University of Texas Southwestern Medical Center*, *Dallas*, *TX*; ^*2*^ *Childrens Health System of Texas*, *Dallas*, *TX*

**Introduction and background:** Physician burnout is at its highest level in decades nationally. It has worsened post‐pandemic in part due to the burden placed on physicians by electronic health records (EHRs). [1] Interventions for burnout have traditionally focused on personal resilience, while largely ignoring the role of the EHR.In a recent survey, 63% of pediatric physicians working at the Children's Health System of Texas (CHST) who are employed by the University of Texas Southwestern Medical Center (UTSW) were moderate to severely burnt out. CHST uses the Epic (Verona, WI, USA) EHR. The number of in‐basket messages has increased from 10,000/year in 2021 to 30,000/year in 2024. EHR message volume correlates with burnout.(2)

At the time of the survey, CHST was also among the top pediatric hospitals with the highest number of medication pop‐up alerts (source: Epic's reporting tools). Nationally over 80% of these warnings are overridden without interaction (3) (4) suggesting that they are distracting to the ordering physicians rather than the intended safety tool. This increases a physician's cognitive load and further worsens burnout[5].

Physicians in our pediatric gastroenterology(GI) clinic were spending an average of 63 minutes of pajama time (time outside of work hours) per day. Utilizing the Stanford Model of Professional Fulfillment, this study focused on improving the Efficiency of Practice through the EHR as one aspect of improving physician professional fulfillment.

**Objectives:** We focused on:

(1)Optimizing the in‐basket using Augmented Response Technology (ART), Epic's AI generated draft responses to patient portal messages.

(2)Eliminating unnecessary medication alerts by collaborating with the CMIO and individual divisions to eliminate low utility medication warnings.

We assessed response times to patient messages, draft times to patient replies, number of alerts shown per medication order (inpatient and outpatient), pajama time.

**Results to date:** **In‐basket optimization:** 88% of patient messages were handled within 2 days after implementing ART. Draft time has decreased from 3:06 to 2:07 minutes per draft (32% decrease) with a 25% acceptance rate. This equates to **128 hours/year saved:** **Eliminating unnecessary medication alerts:** CHST had 26 warnings shown per 100 inpatient orders(0.26) (national pediatric average is 0.16), and 0.35 for outpatient orders (national average is 0.25). Through advocacy and working with the CMIO, alerts improved to 0.13and 0.26 for inpatient and outpatient orders respectively. Importantly, no increase in Serious Safety Events (SSE) were observed. There was a 50% reduction in redundant warnings. The average provider spends 90 seconds to recalibrate cognitive attention. [6]The total monthly warnings were 100,943.,thus we estimate 1,262 hours/month saved. Similar calculations with outpatient data yield a total of 505 hours/month saved.

Overall pajama time for GIreduced from 63 minutes at the start in July 2024 to 50 minutes in March 2025.

**Discussion:** Physician burnout is multifactorial. Traditional strategies have focused on personal resilience. The Stanford Model of Professional Fulfillment[7] emphasizes instead focusing on culture of wellness and efficiency of practice. By leveraging new AI technologies and thoughtful removal of nuisance alerts within the EHR, we streamlined complex tasks while decreasing cognitive load. This is evidenced by decreased physician pajama time and enhanced clinical efficiencies such as documentation, both of which have been associated with improved clinician wellness.

**Conclusion:** Strategic use of AI and deliberate elimination of low‐yield alerts are successful tactics to help improve clinical efficiency, which may likely decrease burnout in healthcare.

**References:**

1. Sinsky, C.A. and M.R. Privitera. JAMA Intern Med, 2018. **178**(6): p. 741‐742.

2. Adler‐Milstein, J., et al., J Am Med Inform Assoc, 2020. **27**(4): p. 531‐538.

3. Weingart, S.N., et al., Arch Intern Med, 2003. **163**(21): p. 2625‐31.

4. Lin, C.P., et al., J Am Med Inform Assoc, 2008. **15**(5): p. 620‐6.

5. Patel, V.L., et al., J Am Med Inform Assoc, 2000. **7**(6): p. 569‐85.

6. Bailey, B.P. and J.A. Konstan, Computers in Human Behavior, 2006. **22**(4): p. 685‐708.

7. Bohman, B.D., et al.,. Acad Med, 2025.


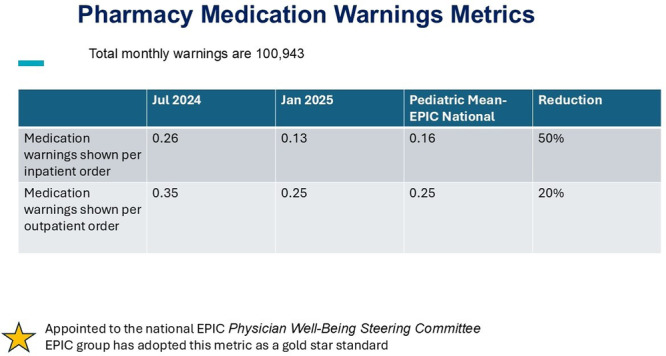


## 286  A NUDGE IN THE RIGHT DIRECTION: UTILIZING ELECTRONIC HEALTH RECORD SOLUTIONS TO IMPLEMENT NEW CYSTIC FIBROSIS HEPATOBILIARY INVOLVEMENT GUIDELINES

*Alexandra Pottorff* ^*1,3*^, *Keishla Valentin‐Martinez* ^*1*^, *Lauren Lazar* ^*1,3*^, *Meghana Sathe* ^*1,3*^, *Megha Mehta* ^*1,3*^, *Prigi Varghese* ^*3*^, *Philip Bernard* ^*2*^, *Derek Ngai* ^*1*^

^*1*^ *Pediatric Gastroenterology*, *The University of Texas Southwestern Medical Center*, *Dallas*, *TX*; ^*2*^ *Pediatric Criticial Care*, *The University of Texas Southwestern Medical Center*, *Dallas*, *TX*; ^*3*^ *Cystic Fibrosis*, *Children's Medical Center Dallas*, *Dallas*, *TX*

**Introduction:** New 2024 guidelines and classification system for Cystic Fibrosis Hepatobiliary Involvement (CFHBI) aim to improve monitoring and classification of liver disease in Cystic Fibrosis (CF) but are difficult to implement in clinics [1,2]. This led to a quality improvement project in our CF clinic to aid in the implementation of these guidelines utilizing an updated electronic health record (EHR) solution. A ‘nudge’ describes a configuration of choices to encourage certain courses of action without taking away the freedom of choice, which have been shown to be effective [3]. We employed nudge‐type strategies in collaborative redesigning of an inefficient and interruptive clinical decision support (CDS) tool to increase adherence to the latest CFHBI guidelines.

**Methods:** The informatics team collaborated with GI physicians in the CF clinic (n=4), who were the primary users of the CFHBI EHR form. Concerns regarding existing EHR tool included repetitive documentation for elements that are unlikely to change from visit to visit (e.g. prior imaging). The form automatically calculated predictive scores but failed to provide reference ranges for interpretation. Lastly, chart open alerts to order surveillance imaging per guidelines were disruptive and ineffective.

We decreased repetitive documentation by changing relevant documentation elements to the patient‐level rather than encounter‐level, allowing previously documented findings to persist into subsequent visits, which imitates the nudge concept of setting defaults. We added reference ranges for predictive scores with automatic background color changes based on severity, blending the nudge concepts of information transparency, environmental cueing, and EHR interface design. Lastly, we replaced the interruptive chart‐open alert with a disappearing text reminder within the clinic note that fits better with the clinic workflow.

Following rollout of the new form in September 2024, we measured adherence to imaging guideline intervals. A survey was sent out to the GI physicians after 2 months to elicit user feedback regarding this tool.

**Results:** In 2024, the CF clinic saw 42 patients in July and 71 in October. CF patients deficient in biennial ultrasounds had appropriate ultrasounds ordering increase from 53.6% (15 of 28) to 61.8% (21 of 34), and annual Fibroscan ordering increase from 53.6% (15 of 28) to 75.0% (27 of 34) [Fig 1a]. Survey responses (4/4 CF GI physicians) show that this tool was associated with improved user rating on effectiveness, efficiency, and usability [Fig 1b].

**Discussion:** Incorporating nudges into feedback from end users improved adherence to CFHBI imaging guidelines while simultaneously increasing physician satisfaction. Following this intervention, the CF team was able to identify inefficiencies within the clinic and made modifications to effectively order and track imaging. Redesigning underperforming CDS with nudges increases adherence to guidelines while enhancing user perception of usability, effectiveness, and efficiency.

**References:**

1. Sellers, Z. M., Assis, D. N., Paranjape, S. M., … & Narkewicz, M. R. Cystic fibrosis screening, evaluation, and management of hepatobiliary disease consensus recommendations. Hepatology. 2024 May 1;79(5):1220‐1238.

2. Bodewes FAJA, Freeman AJ, Weymann A, … & Narkewicz MR. Towards a Standardized Classification of the Hepatobiliary Manifestations in Cystic Fibrosis (CFHBI): A Joint ESPGHAN/NASPGHAN Position Paper. J Pediatr Gastroenterol Nutr. 2024 Jan;78(1):153‐165.

3. Wolf A, Sant'Anna A, Vilhelmsson A. Using nudges to promote clinical decision making of healthcare professionals: A scoping review. Preventive Medicine. 2022 Nov 1;164:107320.


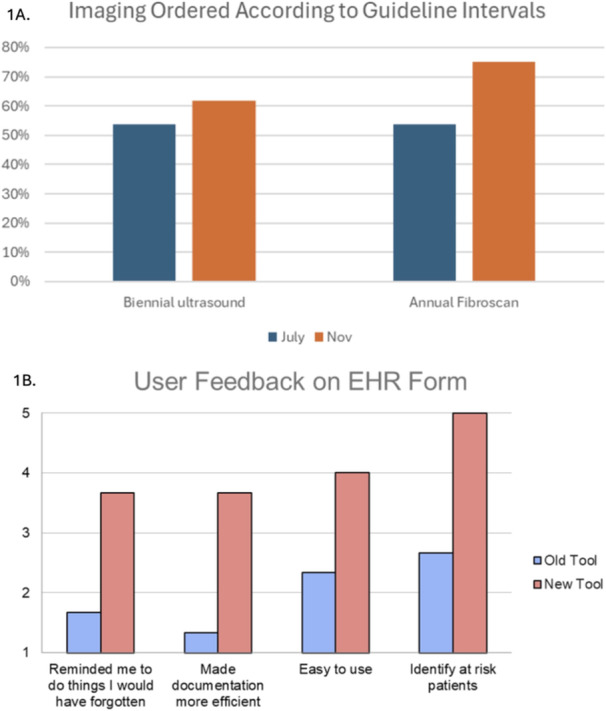


**Figure 1. 1 A:** Imaging ordered per guidelines before and after new EHR form implemented in September. **1B:** GI provider satisfaction with new EHR form universally increased user satisfaction compared to the old form. Each bar shows average score per domain. 1 = Strongly Disagree; 5 = Strongly Agree.

## 287 SAVING TIME AND EASING THE LOAD: IMPACT OF A DIGITAL SCRIBE ON PEDIATRIC GASTROENTEROLOGY WORKFLOWS

*Kevin Watson* ^*1*^, *Jonathan Pelletier* ^*2*^, *Sarah Rush* ^*3*^

^*1*^ *GI*, *Akron Children's Hospital*, *Akron*, *OH*; ^*2*^ *PICU*, *Akron Children's Hospital*, *Akron*, *OH*; ^*3*^ *Medical Informatics*, *Akron Children's Hospital*, *Akron*, *OH*

**Background:** Healthcare providers in the United States have a higher documentation burden than other nations. Half of healthcare providers report burnout symptoms, with electronic health records (EHRs) frequently cited as a source. Whether digital scribes can reduce documentation time and improve job satisfaction for pediatricians is unknown.

**Objective:** To assess the effect of a digital scribe among pediatric gastroenterologists

Design/Methods: We implemented a digital scribe for a 7‐month pilot across general pediatric generalists and subspecialists including pediatric gastroenterologists. After consent, recordings of outpatient visits were transcribed using automatic speech recognition and passed to a generative artificial intelligence model to create a structured clinical note which was edited by providers prior to filing to the EHR. Here we focus on all pediatric gastroenterology providers participating in the pilot with EHR usage logs available. The primary outcome was documentation time savings. Secondary outcomes included task load index, burnout, and patient satisfaction ratings. We analyzed EHR usage records, provider surveys including NASA task load index and mini‐z burnout index, and patient satisfaction surveys using descriptive statistics and interrupted time series analysis.

**Results:** 80% (12/15) providers offered the digital scribe adopted it for the pilot period. The digital scribe was used to generate notes for 67% of encounters (5,471/8,161) across the 12 providers during the pilot period. Digital scribe use was associated with a mean time savings of 4.7 (95% CI 2.3, 7.1) minutes per appointment. The total time savings across the 8,162 appointments was 639.3 (95% CI 312.8, 965.7) hours, representing 2.1 (95% CI 1, 3.1) hours per provider‐week. Interrupted time series analysis showed a 33.5% (95% CI 24%, 43%) time savings after implementation. Response rates for the pre‐ and post‐implementation surveys were 58.3% (7/12) and 41.7% (5/12), respectively. There were significant reductions in cognitive and temporal task load (all p < 0.005). There was not a significant reduction in burnout scores and no significant changes in patient satisfaction scores.

**Conclusion(s):** Digital scribe implementation was associated with decreased documentation time and task load in pediatric gastroenterologists this pilot study. Further work is needed to understand whether these results are replicable on a larger scale.

## 288 FGFBP1+ UPPER CRYPT STEM CELLS REGENERATE INJURED INTESTINAL EPITHELIUM

*Jonathan Miller* ^*1*^, *Claudia Capdevila* ^*2*^, *Liang Cheng* ^*2*^, *Hyeonjeong Lee* ^*2*^, *Joel Johnson George* ^*2*^, *Timothy Wang* ^*2*^, *Kelley Yan* ^*2*^

^*1*^ *Pediatrics*, *NewYork‐Presbyterian Hospital*, *New York*, *NY*; ^*2*^ *Medicine*, *New York‐Presbyterian/Columbia University Irving Medical Center*, *New York*, *NY*

**Background:** The intestinal epithelium is supported by actively cycling stem cells during homeostasis. The identity of intestinal stem cells (ISCs) is debated with a prevailing model that *Lgr5*+ crypt base columnar (CBC) cells are the sole homeostatic ISC population generating progeny that migrate up from the crypt base through an upper crypt transit amplifying (TA) population to become post‐mitotic villus cells. Paradoxically, loss of *Lgr5*+ cells does not disrupt intestinal crypts, proliferation, or epithelial homeostasis. Moreover, the epithelium demonstrates robust capacity for healing when CBCs are lost to injury. Various mechanisms have been proposed to explain these phenomena though no consensus exists. Recently, our lab identified an upper crypt population marked by expression of *Fgfbp1*. Using a novel knock‐in allele for time‐resolved fate mapping, it was shown that *Fgfbp1*+ cells are multipotent and give rise to *Lgr5*+ CBC cells during homeostasis, consistent with their function as stem rather than TA cells. Moreover, *Fgfbp1*+ cells sustain epithelial homeostasis after CBC cell loss by toxin‐mediated ablation, suggesting a role for *Fgfbp1*+ cells in other mechanisms of injury repair as well. We hypothesized that *Fgfbp1*+ ISCs are responsible for injury‐induced regeneration of the intestinal epithelium with implications for treatment of GI disease.

**Methods:** We developed *Fgfbp1‐CreERT2* mice for inducible lineage tracing and 2 clinically relevant disease models of tissue injury to define the role of *Fgfbp1*+ cells in injury‐repair. First, 12 Gy whole body irradiation (IR) which specifically damages mitotic crypt populations including rapid loss of *Lgr5*+ cells. Second, a murine Rotavirus (RV) infection model as this virus infects villus enterocytes inducing a regenerative response without direct injury to the crypt.

**Results:** Mice subjected to IR were harvested at early timepoints when *Lgr5*+ CBC cells are known to be damaged and lost. *ISH* in murine gut 2 and 3 days post‐IR revealed persistence of *Fgfbp1*‐expressing cells within the upper crypt and loss of *Lgr5*+ expression (Figure 1 A). Proliferative upper crypt cells at the initiation of regeneration largely overlapped with *Fgfbp1* expression (yellow arrowheads in 1 A). Lineage tracing of *Fgfbp1*+ cells by a single tamoxifen injection 24 hours prior to IR revealed sparse initial labeling of the upper crypt (Figure 1B). Over the course of 1 week, labeled progeny expanded bi‐directionally to reconstitute entire crypt‐villus units, similar to the timeframe observed during homeostasis. Next, mice were infected orally with murine RV and harvested at multiple timepoints. The proliferative upper crypt region is expanded during the peak of infection (not shown). Moreover, lineage tracing of *Fgfbp1*+ cells revealed accelerated expansion and upward migration of *Fgfbp1*‐progeny as early as 6 hours post‐infection (Figure 1 C,D).

**Conclusions:** *Lgr5*+ cells are accepted as the sole homeostatic ISC population. Their apparent dispensability and susceptibility to injury and disease challenge this model. Our recent identification of *Fgfbp1*+ ISCs suggests an alternative homeostatic model and may reconcile prior observations in injury‐repair. Here we suggest a role for *Fgfbp1*+ cells in 2 distinct injury‐repair models. In IR, where the regenerative response is initiated in the absence of *Lgr5*+ cells, *Fgfbp1*+ ISCs are capable of complete crypt reconstitution. In RV infection, which triggers crypt populations to replace infected enterocytes, *Fgfbp1*+ ISCs are recruited within 6 hours of infection, well before *Lgr5*+ cells undergo proliferative changes. Regenerative capabilities of *Fgfbp1*+ ISCs warrant further investigation to understand their role and therapeutic potential in human disease.


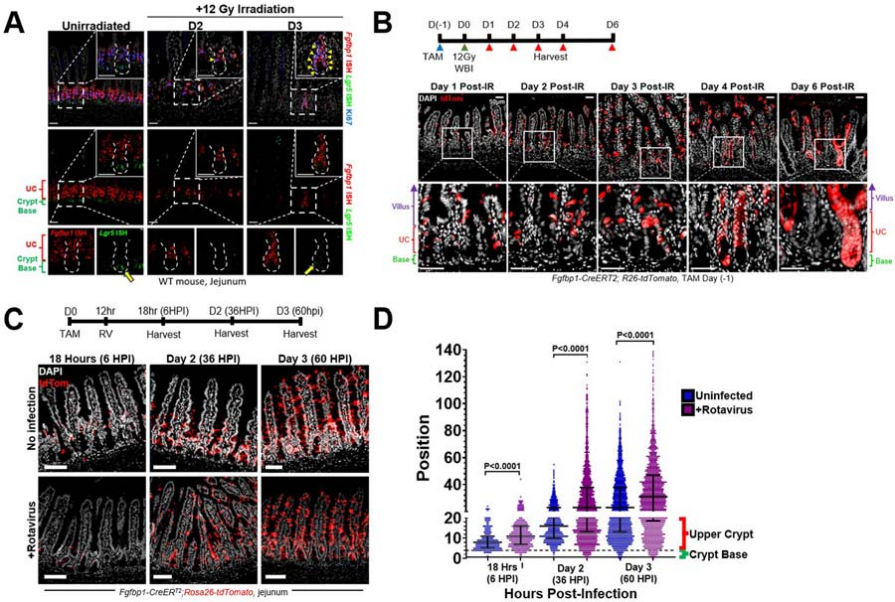


**Figure 1.** ***Fgfbp1*** **+ cells repair injured epithelium.** A. ISH in murine intesine post‐12 Gy IR with *Lgr5*+ cell loss but persistence of *Fgfbp1* expression. B. (Top) Lineage tracing schema. (Bottom) *Fgfbp1‐CreERT2; R26‐tdTomato* traces originate in the upper crypt and reconstitute the base post‐IR. C. (Top) Lineage tracing schema. (Bottom) *Fgfbp1* traces undergo accelerated villus migration post‐infection. D. Quantitation with significantly accelerated villus migration within 6 hours post‐infection. Scale bars 50μm.

## 289 VALIDATION OF URINARY FATTY ACID BINDING PROTEIN 2 (FABP2) AS A NON‐INVASIVE DIAGNOSTIC AND PROGNOSTIC BIOMARKER OF CELIAC DISEASE: EVIDENCE FROM A LARGE PEDIATRIC COHORT

*Nidhi Kapoor* ^*1*^, *Noah Stoeckel* ^*1*^, *Edwin Ballelos* ^*2*^, *Katrina Tiqui* ^*2*^, *Mary Schreck* ^*3*^, *Chirajyoti Deb* ^*1*^, *Bassam Abomoelak* ^*1*^, *Vijay Mehta* ^*4*^, *Akash Pandey* ^*4*^, *Devendra Mehta* ^*4*^

^*1*^ *specialty diagnostic and translational research lab*, *Orlando Health*, *Orlando*, *FL*; ^*2*^ *University of Central Florida*, *Orlando*, *FL*; ^*3*^ *OHMG Peds Spec Prac Resources*, *Orlando Health*, *Orlando*, *FL*; ^*4*^ *Center for digestive health and nutrition*, *Orlando Health Arnold Palmer Hospital for Children*, *Orlando*, *FL*

**Introduction:** Celiac disease (CD) is an autoimmune enteropathy (AIE), triggered by gluten ingestion in genetically predisposed individuals. As disease progresses it can lead to malabsorption, intestinal cell damage, inflammation, and compromised gut barrier integrity. Current diagnostic methods to determine the gut integrity, such as histopathology of intestinal biopsies and sugar absorption tests are complex, time‐consuming, and often difficult to use in routine clinical practice. A promising alternative approach is to measure biomarkers of gut integrity in blood or urine. Intestinal fatty acid binding protein (I‐FABP or FABP2), a member of FABP family, is a cytosolic protein exclusively expressed in the enterocytes of the small intestine and plays a role in the uptake and trafficking. Elevated FABP2 level in circulation have been shown to reflect intestinal epithelial cell damage. Our previous exploratory study demonstrated significantly elevated urinary FABP2 levels in pediatric patients with active CD. In this study, we validate those findings in a larger cohort of celiac patients and establish a diagnostic threshold of FABP2 for its use as a biomarker for diagnosis and prognosis of CD.

**Methods:** Retrospective clinical data and urine samples from a total of 125 patients were used for this study. Based on celiac serology and clinical symptomatology, patients were categorized as either normal (n=107, no CD or celiac resolved) or active CD cases (n=18). The FABP2 concentrations in urine were measured using a commercially available ELISA kit, (Abcam, Inc., USA) following the manufacturer's instructions.

**Results:** The demographic data is summarized in Table1. Urinary FABP2 levels were significantly elevated in patients with active CD (Avg. 128.0 pg/ml) compared to controls (Avg 21.4 pg/ml) (Figure 1 A). Receiver operating characteristic (ROC) curve analysis confirmed the diagnostic utility of FABP2 as a biomarker for celiac disease, with an area under the curve (AUC) of 0.7414 (p = 0.001) and a cut off value of 16.76 pg/ml (sensitivity, 83.3%; specificity 63.5%) (Figure 1B). Additionally, urine FABP2 levels were significantly associated with elevated serum anti‐tissue transglutaminase IgA (tTG‐IgA) levels.

**Conclusion:** This study confirms that the levels of urinary FABP2 are elevated in pediatric patients with active celiac disease compared to those with resolved or no CD. Higher levels of FABP2 in the urine samples of celiac patients indicate intestinal injury, resulting in its release from the epithelial cells into the urine. These findings support the use of FABP2 as a non‐invasive biomarker for intestinal injury and gut barrier dysfunction in CD. Given its specificity to small intestinal enterocytes, FABP2 holds promise as a diagnostic and prognostic tool for monitoring disease activity and treatment response in clinical practice.


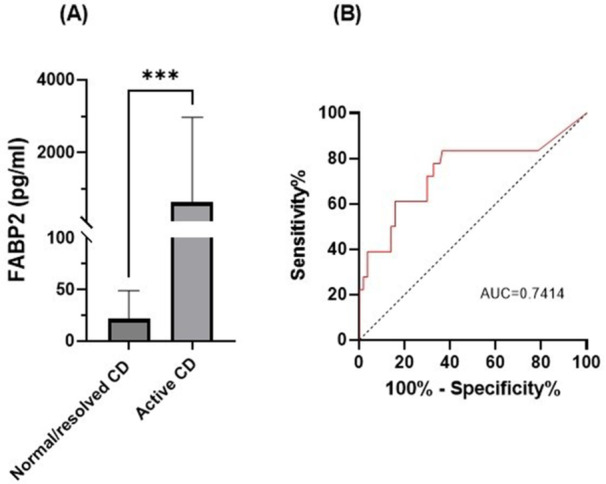


**Figure 1:** Mann‐Whitney Test shows a statistically significant higher concentration of FABP2 in active celiac disease patients compared to normal/resolved CD cases (*p

## 290 COMPREHENSIVE PROTEOMIC EVALUATION TO IDENTIFY NOVEL PLASMA PROTEINS ASSOCIATED WITH ENDOSCOPIC ACTIVITY IN PEDIATRIC CROHN'S DISEASE

*Thomas Aviles* ^*1*^, *Rebekah Karns* ^*1*^, *Jeffrey Hyams* ^*2*^, *Brendan Boyle* ^*3*^, *Joshua Noe* ^*4*^, *Kimberly Jackson* ^*1*^, *Kimberly Lynch* ^*1*^, *Lee Denson* ^*1,5*^, *Phillip Minar* ^*1,5*^

^*1*^ *Gastroenterology, Hepatology and Nutrition*, *Cincinnati Children's Hospital Medical Center*, *Cincinnati*, *OH*; ^*2*^ *Gastroenterology*, *Connecticut Children's Medical Center*, *Hartford*, *CT*; ^*3*^ *Gastroenterology, Hepatology and Nutrition*, *Nationwide Children's Hospital*, *Columbus*, *OH*; ^*4*^ *Gastroenterology*, *Medical College of Wisconsin*, *Milwaukee*, *WI*; ^*5*^ *University of Cincinnati College of Medicine*, *Cincinnati*, *OH*

**Background:** The STRIDE II guidelines highlight biochemical remission and endoscopic healing (EH) as key long‐term treatment targets for patients with Crohn's disease (CD). Assessment of EH is limited to repeat endoscopy, which is associated with high cost, burdensome bowel preparation, and a low, but not insignificant, risk of adverse events. This study therefore aims to identify novel plasma biomarkers for endoscopic activity (EA), addressing the need for a convenient, noninvasive method to assess anti‐TNF response and guide subsequent therapy selection when EA is present.

**Methods:** Eighty patients with luminal CD were enrolled in this prospective multicenter study from October 2019 to December 2022. All were anti‐TNF‐naïve at enrollment and started infliximab or adalimumab. Plasma protein abundance was assessed at baseline and year 1 using a 7322‐protein aptamer‐based assay (SOMAscan®, SomaLogic, Boulder, CO). Patients underwent colonoscopy at year 1 to assess for EH, defined as a Simple Endoscopic Score for CD (SES‐CD) <3. Each colonoscopy was blindly scored by study personnel (PM) unless a video recording was unavailable, in which case the primary endoscopist provided the SES‐CD. Protein abundance was compared between EH and EA using the limma package in R, with false discovery rate (FDR) correction applied due to the large number of proteins tested. Ontological analyses were done in ToppGene. Bootstrapped LASSO regression was then used to select biomarker candidates for predictive modeling. Anti‐TNF trough concentrations (Esoterix, LabCorp) and calculated drug clearance were assessed at year 1.

**Results:** Of 80 enrolled patients, 59 underwent a year 1 colonoscopy. Ten declined colonoscopy, 6 had CD surgery and 5 discontinued anti‐TNF prior to year 1. We found 37 patients achieved EH and 22 had EA. No difference in baseline demographics, disease location, or disease phenotype was seen between EH and EA. Those with EA had significantly higher baseline fecal calprotectin (FCP; p=0.007) and erythrocyte sedimentation rate (p=0.024). At year 1, no difference in anti‐TNF trough concentration was seen between EH and EA, but FCP (p<0.0001) and infliximab clearance (p=0.0008) differed significantly. Proteomics analysis at year 1 identified 22 proteins which differed significantly between EH and EA with FDR<0.05; however, since this was in the exploratory phase of analysis, we included all proteins with raw p<0.01 for ontological enrichment (Fig. 1; 104 down, 29 up in EH vs. EA). Bootstrapped LASSO regression identified several biomarker candidates with known roles in inflammatory responses: IL‐10, IL‐22, MRC1, SH2B3, MMP12 and TXNDC5 were all significantly less abundant in EH. Multiple regression modeling using IL‐10 and MRC1 to predict EH produced an AUROC of 0.83 (95% CI 0.728, 0.932). Anti‐TNF trough concentration did not improve the model, yet adding predicted infliximab clearance at year 1 improved the AUROC to 0.89 (Fig. 2; 95% CI 0.799, 0.981).

**Conclusion:** Using plasma proteomics, we identified novel biomarker candidates associated with EA after one year of anti‐TNF therapy. A model using IL‐10 and MRC1 predicted EH well in this small dataset and was strengthened by adding infliximab clearance. While validation in a larger cohort is ongoing, our biomarker panel shows promise for guiding therapy selection in anti‐TNF‐refractory CD patients.


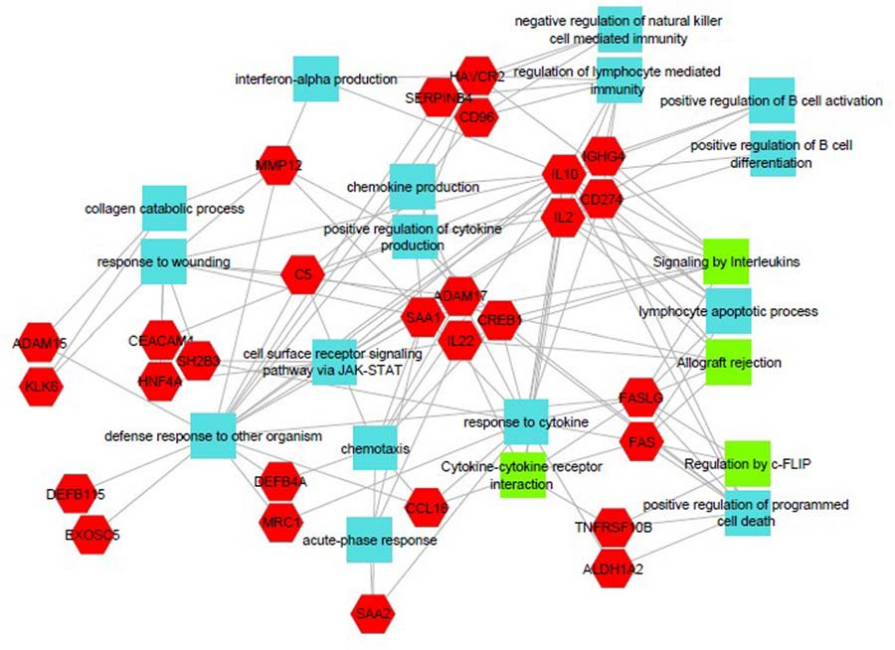


Fig. 1: Ontology map of proteins less abundant in EH.


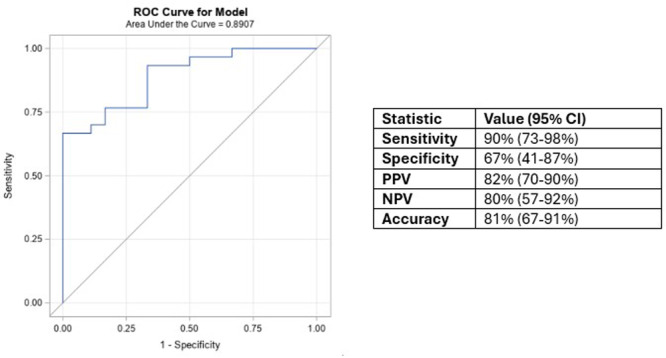


Fig. 2: ROC curve for the IL10+MRC1+IFXCL model. This was analyzed only in patients on infliximab with clearance data (n=48). Sensitivity, specificity, predictive values and accuracy are shown in the table.

## 291 NOVEL POINT‐OF‐CARE DIAGNOSTIC RISK MODEL AND CLINICAL CALCULATOR FOR BILIARY ATRESIA

*Sandra Rios‐Melendez* ^*1*^, *Leon Yuan* ^*4*^, *Song Zhang* ^*5*^, *Reena Mourya* ^*1*^, *Pranavkumar Shivakumar* ^*1*^, *Estella Alonso* ^*6*^, *Stephen Guthery* ^*7*^, *Sanjiv Harpavat* ^*8*^, *Simon Horslen* ^*9*^, *Binita Kamath* ^*10*^, *Saul Karpen* ^*11*^, *Rohit Kohli* ^*12*^, *Kathleen Loomes* ^*10*^, *John Magee* ^*13*^, *Alexander Miethke* ^*14*^, *Benjamin Shneider* ^*8*^, *Ron Sokol* ^*3*^, *Pamela Valentino* ^*2*^, *Jorge Bezerra* ^*1*^, *Sindhu Pandurangi* ^*1*^

^*1*^ *Pediatrics*, *The University of Texas Southwestern Medical Center*, *Dallas*, *TX*; ^*2*^ *Pediatrics*, *Seattle Children's Hospital*, *Seattle*, *WA*; ^*3*^ *Pediatrics*, *Children's Hospital Colorado*, *Aurora*, *CO*; ^*4*^ *Biostatistics*, *Southern Methodist University*, *Dallas*, *TX*; ^*5*^ *Statistics*, *The University of Texas Southwestern Medical Center*, *Dallas*, *TX*; ^*6*^ *Pediatrics*, *Ann and Robert H Lurie Children's Hospital of Chicago Foundation*, *Chicago*, *IL*; ^*7*^ *Pediatrics*, *Primary Children's Hospital*, *Salt Lake City*, *UT*; ^*8*^ *Pediatrics*, *Texas Children's Hospital*, *Houston*, *TX*; ^*9*^ *Pediatrics*, *UPMC Children's Hospital of Pittsburgh*, *Pittsburgh*, *PA*; ^*10*^ *Pediatrics*, *The Children's Hospital of Philadelphia*, *Philadelphia*, *PA*; ^*11*^ *Pediatrics*, *Emory University*, *Atlanta*, *GA*; ^*12*^ *Pediatrics*, *Children's Hospital Los Angeles*, *Los Angeles*, *CA*; ^*13*^ *Surgery*, *University of Michigan*, *Ann Arbor*, *MI*; ^*14*^ *Pediatrics*, *Cincinnati Children's Hospital Medical Center*, *Cincinnati*, *OH*

**Background/Objective:** Early diagnosis of biliary atresia (BA) is essential for timely surgical intervention and improved outcomes. Matrix metalloproteinase‐7 (MMP‐7) has been shown to be an accurate diagnostic predictor of BA; however, clinicians frequently incorporate other labs and clinical features into determining the clinical urgency of intraoperative cholangiogram evaluation. We aimed to create an integrated model which enhances interpretability of MMP‐7 in context of other patient factors and provides individualized risk estimates to support real‐time clinical decisions.

**Methods:** This is a case‐control study of 399 cholestatic infants enrolled in the ChiLDReN Prospective Database of Infants with Cholestasis study. The primary aim was to create a multivariable risk prediction model of BA diagnosis. The secondary aim was to create a user‐friendly risk prediction tool for future validation and clinical use. All serum MMP‐7 levels were quantified at diagnosis using a time resolved fluorescence resonance energy transfer (TR‐FRET) assay. All statistical analyses were conducted in R, and the clinical web calculator was created using the R *shiny* package (R Core Team, version 4.4.1, 2024).

**Results:** 201 infants with BA (median age 64 days) and 198 with non‐BA cholestasis (median age 59 days) were studied (p =0.81). BA was diagnosed by histological findings of luminal obstruction in extrahepatic bile duct remnants. Baseline laboratory and clinical features that were available for analysis were age (days), race, presence of acholic stool (defined by provider as binary yes/no), AST, ALT, total bilirubin, direct bilirubin, GGT, and MMP‐7 levels. MMP‐7 was drawn at the same baseline timepoint as the clinical labs. At baseline, total bilirubin and GGT levels were higher in the BA cohort (p =0.01 and p<0.001, respectively). In multivariable logistic regression, age, stool color, MMP‐7, and GGT were strongest predictors of BA (all p < 0.001) and included in the final model. The final model had an excellent discriminative performance (AUROC 0.9073 CI: 0.88‐0.94). The model achieved 94% sensitivity and 70% specificity, with a false negative rate of 6%. In stepwise models, diagnostic performance improved incrementally with each additional variable to MMP‐7 alone (Fig 1 A). Net reclassification analysis demonstrated that the full model correctly reclassified 19% of BA cases to higher‐risk categories and 30% of non‐BA patients to lower‐risk categories, yielding a total net reclassification improvement (NRI) of 49% (Fig 1B). Compared to a model including only GGT, stool color, and age (AUROC = 0.82; 95% CI: 0.78–0.86), the addition of MMP‐7 improved discrimination and significantly enhanced risk classification (NRI = 0.71), correctly reclassifying 33% of BA cases to higher‐risk and 39% of non‐BA cases to lower‐risk categories. Across age strata (<30 days, 30‐60 days, > 60 days of age), the prediction model shows good‐to‐excellent discrimination (AUC 0.72–0.93) and accuracy (70–84 %), with peak performance in infants 30–60 days old. A user‐friendly clinical risk calculator was created from the final model (Fig 1 C).

**Conclusion:** We present a clinically actionable diagnostic model for BA that integrates age, stool color, GGT, and serum MMP‐7 levels to generate individualized risk predictions in infants with cholestasis. The full model demonstrated excellent discrimination and reclassification performance compared to MMP‐7 alone, and maintains accuracy across age groups, with highest performance among infants 30–60 days old. A point‐of‐care clinical calculator was developed to facilitate real‐time application. This tool may support earlier recognition and referral for BA evaluation, potentially improving timing of surgical intervention, but requires external validation before clinical utilization. Future directions include prospective validation in external cohorts and assessment of clinical impact.


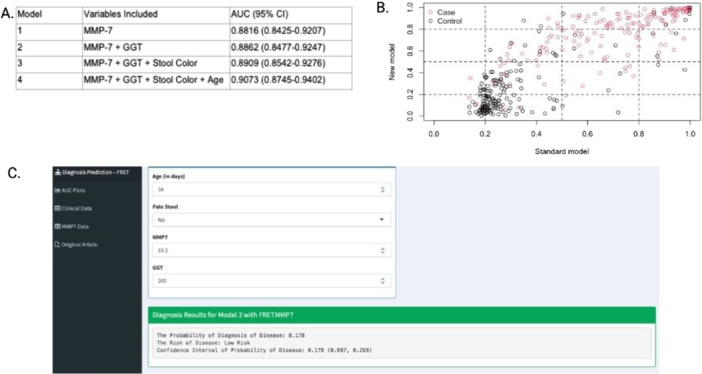


**Figure 1.** Performance of diagnostic model. (A) AUROC for stepwise logistic regression models. (B) Reclassification plot comparing predicted probabilities from the standard model (MMP‐7 alone; x‐axis) and the full model (MMP‐7, GGT, stool color, age; y‐axis). Red points represent BA cases and black points represent non‐BA controls. (C) Screenshot of web‐based clinical risk calculator derived from the final model.

## 298 ASSOCIATIONS BETWEEN SOCIAL DETERMINANTS OF HEALTH AND GASTROSCHISIS SEVERITY: A RETROSPECTIVE UNIVERSITY OF CALIFORNIA FETAL CONSORTIUM STUDY

*Melissa Ada* ^*1*^, *Kara Calkins* ^*2*^, *Daniel DeUgarte* ^*3*^, *Geoanna Bautista* ^*4*^, *Alexandra Leegwater* ^*4*^, *Cherry Uy* ^*5*^, *Helena Olivieri* ^*6*^, *Teresa Sparks* ^*7*^, *Christine Blauvelt* ^*7*^, *Shiyu Sherry Bai‐Tong* ^*8*^, *Sarah Lazar* ^*8*^, *Keren Chen* ^*9*^, *Nicholas Jackson* ^*9*^, *Rashmi Rao* ^*10*^

^*1*^ *Pediatric Gastroenterology*, *University of California Los Angeles*, *Los Angeles*, *CA*; ^*2*^ *Neonatology*, *University of California Los Angeles*, *Los Angeles*, *CA*; ^*3*^ *Pediatric Surgery*, *University of California Los Angeles*, *Los Angeles*, *CA*; ^*4*^ *Neonatology*, *UC Davis Children's Hospital*, *Sacramento*, *CA*; ^*5*^ *Neonatology*, *University of California Irvine*, *Irvine*, *CA*; ^*6*^ *Pediatrics*, *University of California Irvine*, *Irvine*, *CA*; ^*7*^ *Maternal‐Fetal Medicine*, *University of California San Francisco*, *San Francisco*, *CA*; ^*8*^ *Neonatology*, *University of California San Diego*, *La Jolla*, *CA*; ^*9*^ *Medicine Statistics Core*, *University of California Los Angeles*, *Los Angeles*, *CA*; ^*10*^ *Maternal‐Fetal Medicine*, *University of California Los Angeles*, *Los Angeles*, *CA*

**Background:** Infants with complex gastroschisis face a higher mortality and morbidity than those with simple gastroschisis. It remains unclear if infants with simple and complex gastroschisis share the same risk factors and social determinants of health.

**Objectives:**

1. Compare neonatal outcomes in simple vs complex gastroschisis

2. Assess maternal demographics and neonatal outcomes in infants with simple gastroschisis

3. Examine associations between maternal social determinants of health and gastroschisis severity

**Methods:** This multi‐center retrospective cohort study (n=337 maternal‐neonatal dyads) included gastroschisis infants born between 2015‐2025 and cared for at one of the University of California Fetal Consortium (UCFC) sites. Complex gastroschisis was defined as the presence of intestinal atresia, stricture, or ischemia prior to closure. Zip codes were categorized as rural or non‐rural. Bivariate differences in demographic characteristics between simple vs complex gastroschisis were assessed, and logistic regression models were used to examine interactions among geographic location, race/ethnicity, insurance status, and gastroschisis severity.

**Results:** Compared to infants with simple gastroschisis, infants with complex gastroschisis were more likely to be born premature, have lower birth weight and smaller head circumference, experience longer hospital stays and delayed abdominal closure, take longer to achieve full enteral feeds, require gastrostomy tube placement, and be discharged on parenteral nutrition (Table 1). Among infants with simple gastroschisis, neonatal outcomes did not significantly differ by maternal race, ethnicity, rural vs non‐rural residence, or insurance status. After adjusting for geography, Non‐Hispanic maternal ethnicity (odds ratio [OR] 2.0 [95% CI: 1.1, 3.6]) and White maternal race (OR 1.9 [95% CI: 1.1, 3.7]) were more likely to have complex gastroschisis. There was no increase in complex gastroschisis in maternal rural residence or in those with public insurance.

**Conclusions:** Complex gastroschisis is associated with poor neonatal outcomes compared to simple gastroschisis. Our study revealed associations between maternal demographic factors and gastroschisis severity. Mothers of Non‐Hispanic ethnicity and White maternal race had an increased risk of giving birth to a neonate with complex gastroschisis. These findings underscore the need for targeted research into unmeasured factors that may contribute to gastroschisis severity with a focus on racial and socioeconomic differences in prenatal care and lifetime exposures.


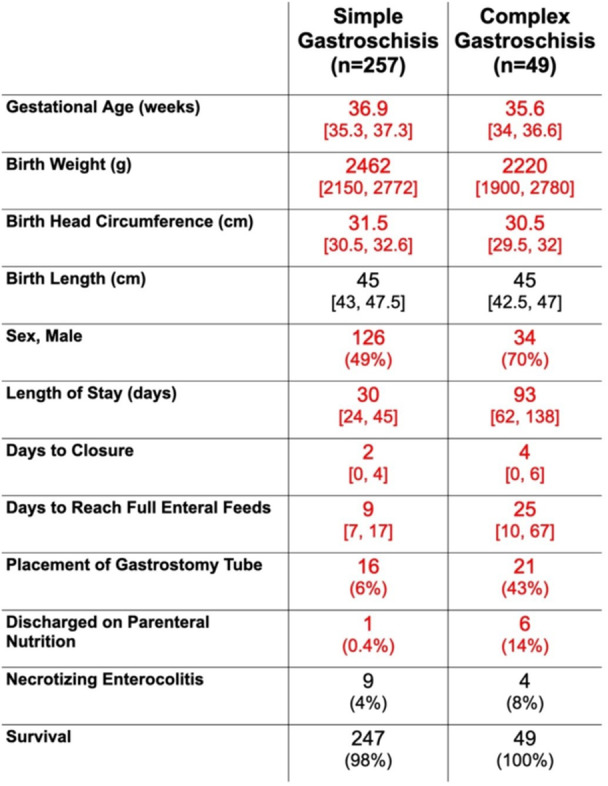


**Table 1:** Neonatal clinical information. Data are presented as median [IQR] or n (%). Full enteral feeds are defined as 100 kcal/kg/day. P

## 299 DISPARITIES IN PERIANAL FISTULIZING DISEASE AMONG CHILDREN WITH CROHN'S DISEASE BY INSURANCE AND PROVIDER TYPE

*Jeremy Adler* ^*1,2*^, *Lauren Manning* ^*2*^, *Moshiur Rahman* ^*2*^, *Alison Tribble* ^*3*^, *Samir Gadepalli* ^*4,2*^

^*1*^ *Pediatric Gastroenterology*, *University of Michigan Michigan Medicine*, *Ann Arbor*, *MI*; ^*2*^ *Susan B. Meister Child Health Evaluation and Research Center*, *University of Michigan Michigan Medicine*, *Ann Arbor*, *MI*; ^*3*^ *Pediatric Infectious Disease*, *University of Michigan Michigan Medicine*, *Ann Arbor*, *MI*; ^*4*^ *Pediatric Surgery*, *University of Michigan Michigan Medicine*, *Ann Arbor*, *MI*

**Background:** Nearly 30% of children with Crohn's disease (CD) develop perianal fistulizing disease (PFCD), more commonly than among adults. PFCD can be severe and difficult to treat, increasing likelihood of surgery, and worsening quality of life.

Racial and ethnic disparities in PFCD development are well described. However, differences in PFCD by care setting have not been characterized. We aimed to determine the variation in care and outcomes by insurance and provider type in a national administrative database.

**Methods:** Using Merative Marketscan (US administrative database including both commercial and Medicaid), we identified children (1‐18 yr) diagnosed with CD, and identified those with PFCD (including perianal fistula and/or abscess), using validated case definitions. We required enrollment 1 yr before and 4‐yr after CD diagnosis. We categorized PFCD development as “before/at Dx” as 1 yr before to 30 d after CD diagnosis, and “after Dx” as 31 d to 4 yr after CD diagnosis and characterized associated healthcare utilization. Provider type was classified as pediatric gastroenterologist (Ped GI) or other (Non‐Ped GI), which constituted a mix of adult GI and non‐GI providers. Insurance was dichotomized as commercial or not. We used descriptive statistics including chi‐square and Wilcoxon rank sum to compare groups.

**Results:** We identified 7,161 patients who met inclusion criteria: 57% male, 80% commercial insurance. Age at CD diagnosis was 63% 15‐18 yr, 28% 10‐14 yr, 8% 5‐9 yr, and 1% 1‐4 yr. Of the total patients, 2,444 (34.1%) saw Ped GI Before/at Dx. A total of 1,226 (17.1%) developed PFCD during the observation period (1 yr before to 4 yr after CD diagnosis), more commonly among males (18.0%) than females (16.0%; p=0.03), and more commonly among those diagnosed 15‐18 yr (18.4%) compared to 10‐14 yr (15.7%), 5‐9 yr (11.9%) and 1‐4 yr (14.9%; p<0.001). PFCD developed before/at Dx among 548 (7.7%) patients. Patients without commercial insurance were 27% more likely to present with PFCD before/at Dx compared to those with commercial insurance (9.2% vs. 7.3%; p=0.013). PFCD developed after Dx among 678 (9.4%) patients, which was 25% more common among those cared for by Non‐Ped GI provider (10.2% vs. 8.1%; p=0.006). When restricting analyses to patients without PFCD before/at Dx (N=6,613), the difference persisted. Among patients in Non‐Ped GI care 479 (11.0%) developed PFCD, more compared with 199 in Ped‐GI care (8.9%; p=0.008). There were no differences between Ped‐GI and Non‐Ped‐GI providers perianal drainage procedures (p=0.39) in ostomy (p=0.31).

**Discussion:** PFCD are common, occurring in 17.1% of children within 4 yr of CD diagnosis. Only 1 in 3 children saw Ped GI at the time of CD diagnosis. Lack of commercial insurance is associated with increased incidence of PFCD before diagnosis, suggesting a disproportionate delay in diagnosis. PFCD after diagnosis were more common among patients not cared for by Ped GI and though reasons are unclear, may be related to delay in appropriate therapy for severity of disease.

## 300 DISTINCT DISEASE PATTERNS AND ELEVATED SOCIAL RISK FACTORS IN HISPANIC/LATINO PEDIATRIC INFLAMMATORY BOWEL DISEASE PATIENTS: A SINGLE‐CENTER STUDY

*Laura Bauman*, *D. Brent Polk*, *Jeannie Huang*

*Pediatric Gastroenterology*, *University of California San Diego*, *La Jolla*, *CA*

**Introduction:** Limited data exist on the disease phenotypes of pediatric Hispanic/Latino (H/L) patients with inflammatory bowel disease (IBD). The National Institutes of Health (NIH) defines race and ethnicity as sociocultural constructs, emphasizing their roles as social determinants rather than genetic factors. These constructs, shaped by historical, social, and cultural contexts, influence experiences, behaviors, and access to resources. Understanding phenotypes in H/L pediatric IBD requires examining social determinants of health (SDOH). This study explored disease phenotypes and outcomes in H/L pediatric IBD patients alongside SDOH metrics.

**Methods:** Patients under 24 years of age followed at the Rady Children's Hospital IBD center registry from January 1, 2023, to November 4, 2024, were included. SDOH metrics were collected every six months. Data on demographics, Paris classification, remission status, and SDOH questionnaire responses were included. Questionnaires covered the U.S. Household Food Security Survey Module (HFSSM), Housing Instability and Homelessness Screening Tool, Transportation Needs Screening Tool, CRAFFT Screening Test for Adolescent Substance Use, University of Washington Caregiver Stress Scale, and Caregiver Health Literacy and Education Assessment. Some questionnaires were collected multiple times per patient. Categorical variables were analyzed using Chi‐squared and Fisher exact tests, with post hoc Z tests and Bonferroni corrections for pairwise comparisons.

**Results:** The cohort included 691 patients, 187 (27.06%) of whom self‐reported as H/L. Demographic data, Paris classification, and remission status are summarized in Table 1. H/L patients were more likely to have indeterminate colitis (IC), while non‐Hispanic/Latino (nH/L) patients were more likely to have Crohn's disease (CD). H/L patients were diagnosed later than nH/L patients, primarily due to a higher proportion of nH/L patients diagnosed before age six, while H/L patients were more often diagnosed after age 17. Growth delay was more common in nH/L patients. Among patients with ulcerative colitis (UC) or IC, disease location and severity did not vary by ethnicity. For CD, disease behavior was similar across ethnicities, but disease location differed, with H/L patients having higher rates of L2 and L4a disease. Remission status and recent medication use (across 10 categories) were comparable across ethnicities.

A total of 2192 SDOH surveys were collected. H/L patients were significantly more likely to report high‐risk SDOH metrics, including inadequate caregiver health literacy and preference for a language other than English (Table 2). Although H/L patients showed higher risk across most SDOH metrics, these differences were not statistically significant, potentially due to small sample sizes.

**Conclusion:** This single‐center study of pediatric IBD patients identified significant differences in disease subtypes and growth delay between H/L and nH/L patients. H/L patients more frequently presented with UC and IC. Among CD patients, H/L patients showed distinct disease locations, with higher prevalence of colonic disease. Additionally, H/L patients were more likely to report high‐risk SDOH metrics, particularly a preference for a language other than English and lower caregiver health literacy.


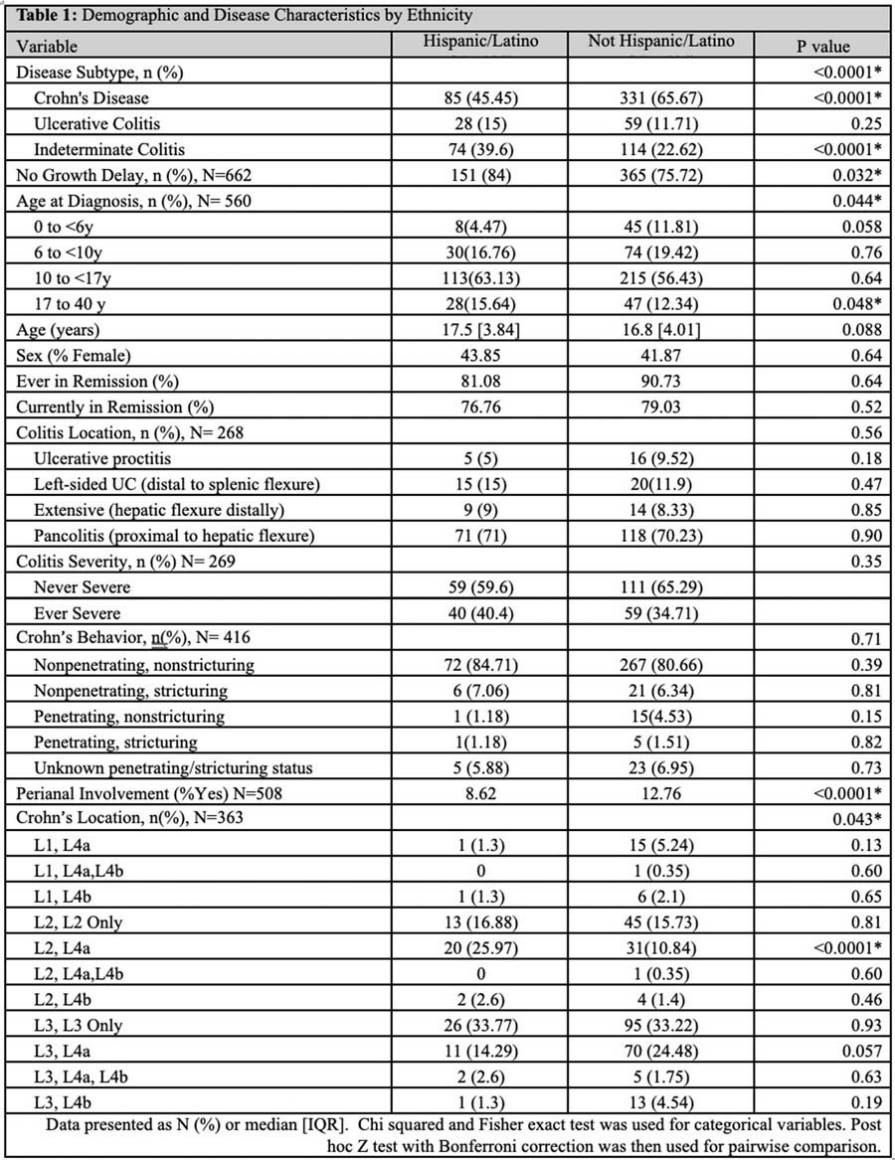


Table 1: Demographic and Disease Characteristics by Ethnicity


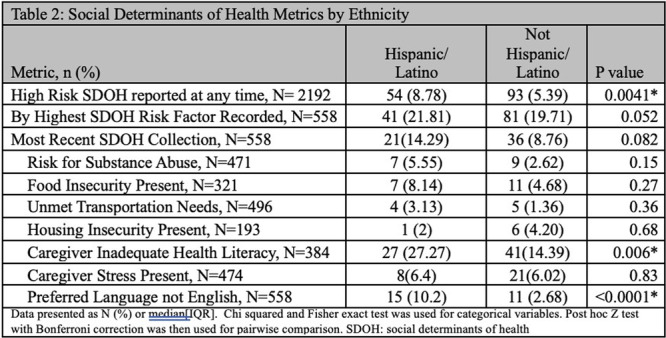


Table 2: Social Determinants of Health Metrics by Ethnicity

## 301 THE GLOBAL EPIDEMIOLOGY OF VEO‐IBD: EXAMINING THE IMPACT OF SOCIOECONOMIC AND HEALTHCARE FACTORS

*Laura Bauman* ^*1*^, *Gretchen Bandoli* ^*2*^, *Phillipp hartman* ^*1*^

^*1*^ *Pediatric Gastroenterology*, *University of California San Diego*, *La Jolla*, *CA*; ^*2*^ *University of California San Diego*, *La Jolla*, *CA*

**Introduction:** The global incidence of pediatric inflammatory bowel disease (IBD) has risen, with approximately 25% of cases diagnosed in childhood. Among these, very early‐onset IBD (VEO‐IBD), defined as onset before age 5 or 6, is of particular interest due to its strong genetic component and diagnostic challenges in resource‐limited settings. However, it remains unclear whether observed increases in VEO‐IBD prevalence reflect true epidemiological trends or improvements in diagnostic capacity driven by socioeconomic development.

**Methods:** Using data from the Global Burden of Disease (GBD) Study 2019, we examined VEO‐IBD prevalence and mortality across 204 countries between 1990–2019. We assessed associations between disease metrics and socioeconomic and healthcare indicators—including the Socio‐demographic Index (SDI), GDP per capita, education, health workforce density, hospital beds, and the Universal Health Coverage Index—using linear and multiple regression analyses.

**Results:** Global VEO‐IBD prevalence remained stable (0.02 per 100,000) from 1990 to 2019, with the largest increases observed in Africa, the Middle East, and Southeast Asia (Figure 1). SDI was a significant positive predictor of prevalence (adjusted R squared = 0.392, *p* < 2e‐16) and a modest negative predictor of death rate (*p* = 0.004). Figure 2. Change in SDI explained 65% of the variance in change in prevalence. In multivariate analysis, healthcare worker density and universal health coverage were the most significant predictors of VEO‐IBD prevalence (*p* < 0.001), while SDI and GDP were not.

**Conclusions:** While regional increases in VEO‐IBD prevalence are evident, particularly in developing regions, these trends are more closely linked to improvements in healthcare infrastructure and diagnostic capability than to true rises in disease incidence. Our findings underscore the importance of healthcare access and surveillance systems in shaping observed epidemiological trends and caution against overinterpreting prevalence increases as rising disease burdens in low‐resource settings.


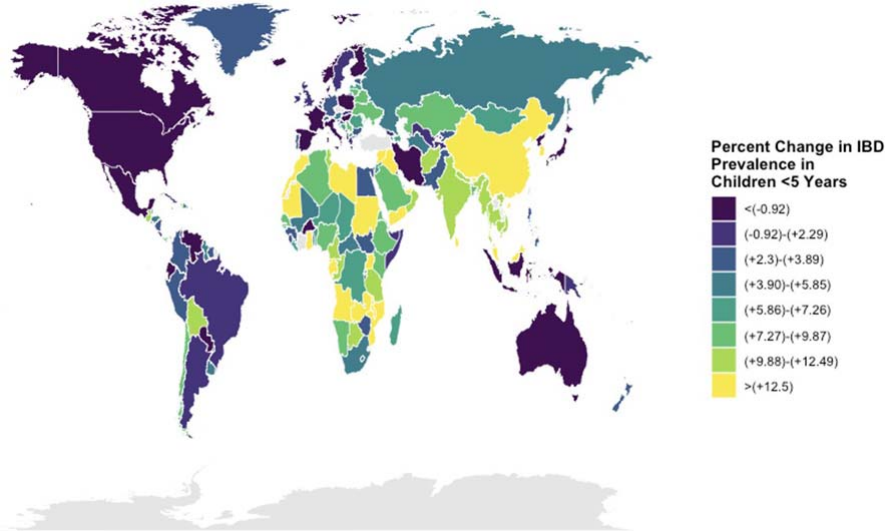


Figure 1: Global Change in Prevalence of IBD in Children Under 5 Years, 1990‐2019


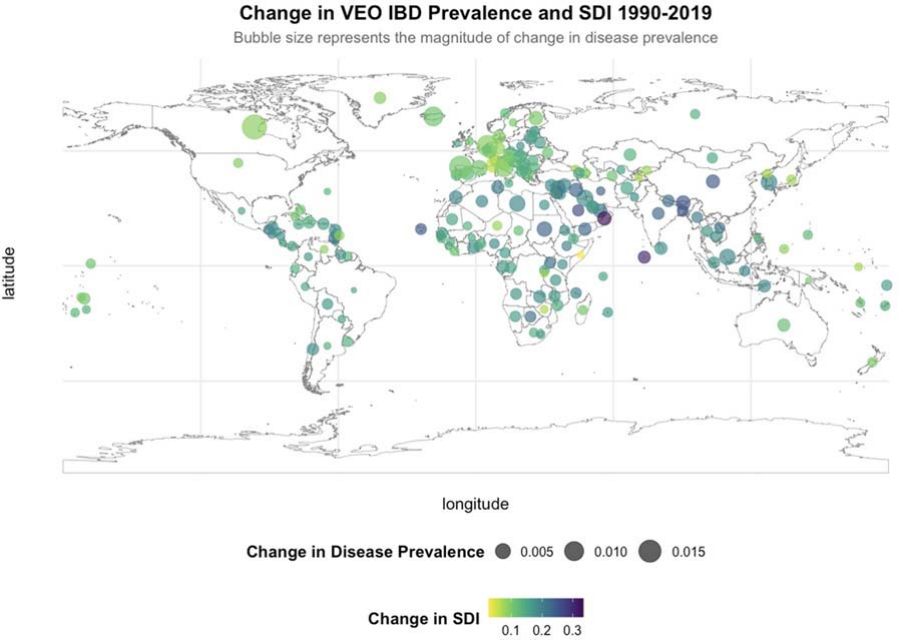


Figure 2: Change in VEO IBD Prevalence and SDI, 1990‐2019

## 302 ASSESSING BARRIERS TO CARE IN PATIENTS WITH EOSINOPHILIC ESOPHAGITIS

*Darius Blanding* ^*1*^, *Brandi Freeman* ^*2*^, *Kaitlin Olson* ^*3*^, *Calies Menard‐Katcher* ^*1*^

^*1*^ *Pediatric Gastroenterology, Hepatology, and Nutrition*, *University of Colorado Anschutz Medical Campus*, *Aurora*, *CO*; ^*2*^ *Pediatrics*, *University of Colorado Anschutz Medical Campus*, *Aurora*, *CO*; ^*3*^ *University of Colorado Anschutz Medical Campus*, *Aurora*, *CO*

**Background and Aim:** Eosinophilic esophagitis (EoE) is a chronic inflammatory disease of the esophagus. Timely diagnosis and consistent treatment is needed to prevent sequalae such as food impactions and strictures. Access to healthcare resources can be a significant barrier for families. Given EoE is an endoscopic diagnosis requiring subspeciality referral, it is likely that EoE is underdiagnosed in disadvantaged populations. There have been minimal studies in the pediatric population specifically assessing barriers to care directly from patients and families. This study aims to identify self‐expressed barriers to care for caregivers of patients with EoE and determine if socioeconomic factors and demographics are associated with barriers in this patient population.

**Methods:** This study is a single center, cross sectional study consisting of a survey disseminated through the electronic medical record to caregivers of patients 18 years of age or younger diagnosed with EoE residing in the state of Colorado and seen by a gastroenterologist between the years of 2019‐2024. The questionnaire includes a validated 37 question multiple‐choice Barriers to Care Questionnaire (BCQ) regarding their experience with their gastroenterology care for EoE, along with 13 multiple‐choice demographic questions. The BCQ questions are divided into five subscales: *expectations, pragmatics, skills, marginalization, and knowledge*. Responses range from 0 to 100 in 25‐point increments with 100 being “no problem” and 0 being a “very big problem”. Demographic questions incorporate socioeconomic factors including food insecurity, insurance type, geographical area, and household income. Responses to the BCQ were compared across each of the 13 demographic responses through use of non‐parametric tests and corrected for multiple testing using Bonferroni.

**Results:** There were 125 questionnaires were completed and analyzed. The mean age of patients was 11.9 years (SD 4.9), with 82% of subjects identifying as white, 9% as two or more races, 4% Black, 1% Asian, and 4% other. 12% identified as Hispanic or Latino and 3 respondents (2%) reported speaking a language other than English primarily in the home. 78.4% of respondents said they preferred in person visits over telehealth visits. 72% of respondents had private insurance (n=84) or Tricare (n=6) with 28% having public insurance (n=35).

Median scores from each of the BCQ subscales ranged from the lowest of 80.6 in the *pragmatics* score to the highest of 100 in the *knowledge* score, with an overall BCQ median score of 86.8. Total BCQ score was lower in those who used cellphone service instead of WIFI for internet access at home (71.2 vs. 89.1, p=0.01) and all subscales other than the skills subscale (p <0.035), though cellphone service users consisted of only 5 of the total respondents (4%). No other demographic response categories differed for total BCQ score.

The expectations total subscale score, questions regarding personal perception of care from their gastroenterologist, was also lower in respondents that identified as white (87.5 vs. 95.8, p=0.026). The pragmatics subscale total also trended towards lower for caregivers that lived >30 minutes from the health care facility (75 vs 80.6, p=0.052). Other subscale total scores did not significantly differ between other self‐reported demographics. The presence of food insecurity did not associate with any specific barrier subscale however did have lower scores to questions related to “having enough information about how the health care system works (50.0 with vs 75.0 without food insecurity, p=0.002), accessing care after hours or on the weekends (75.0 vs 100.0, p=0.035), having to take care of household responsibilities (50.0 vs 100.0, p=0.003) and meeting the needs of other family members (75.0 vs 100.0, p=0.024).

**Conclusions:** Findings from this administration of the BCQ survey identified several statistically significant barriers across demographic descriptors of respondents. These results highlight the complex and varied nature of barriers to care, underscoring the need for providing care that address both practical access issues and individual expectations that are affected by each patients' diverse demographic background. Asking regarding access to WIFI and food insecurity may help identify patients at most risk of barriers to care. Additional recruitment efforts are underway to include a larger patient/caregiver population.

## 303 ACALCULOUS CHOLECYSTITIS: A BRIEF OVERVIEW OF REAL WORLD PEDIATRIC MANAGEMENT

*Shahzaib Khan*, *Charlotte Banayan*, *Tina Maria Moreck*, *Thomas Wallach*

*Pediatric Gastroenterology*, *SUNY Downstate Health Sciences University*, *New York*, *NY*

**Background:** Acute acalculous cholecystitis is defined as acute inflammation of the gallbladder in the absence of gallstones. Although it accounts for only 5‐10% of cases in critically ill adults, recent epidemiological data has suggested an increasing incidence with acalculous cholecystitis becoming a more common etiology of acute cholecystitis in the pediatric population. Previously thought to be a rare disease described mostly in patients with acute systemic illness, chronic disease and post‐surgical patients, more recent pediatric cases have been observed secondary to viral infections. Given its fast‐rising incidence in previously health children, poor understanding of disease etiology and lack of data on management guidelines in the pediatric population has become increasingly significant. We describe a retrospective cohort of pediatric patients with acalculous cholecystitis, and the strategies employed in the management of their disease.

**Methods:** We obtained our data from TriNetX, which is a database with electronic medical records from over 120 million patients across 80 healthcare organizations. Using TrinetX, we identified patients who are either currently, or were previously at the time of their diagnosis, less than or equal to 21 years of age with acalculous cholecystitis. There is no ICD‐10 code for acalculous cholecystitis, therefore, we narrowed our search by identifying patients who had unspecified cholecystitis (ICD‐10 K81.9) and who did not have obstruction of the gallbladder (ICD‐10 K82.0) or cholelithiasis (ICD‐10 K80) upon diagnosis. To evaluate the various management strategies, we further narrowed our group to assess for antibiotic use alone, antibiotics followed by gallbladder surgery, and a third group who received ERCP along with antibiotics and surgical management.

**Results:** Our search identified 4609 pediatric patients with acalculous cholecystic. Of these, 2158 (47%) patients were managed with antibiotics alone. The second group of patients received surgical intervention along with antibiotics and consisted of 839 (39%) patients. A small subset of 21 (1%) patients received ERCP on top of antibiotics and surgical management.

**Discussion:** Our results, which looked at a large cohort of pediatric patients with acalculous cholecystitis, found nearly half of patients responding to antibiotics alone, with a little over one third requiring surgical intervention. This contrasts with adults, where the traditional treatment has been surgical intervention. It is important to note that no ICD code exists for acalculous cholecystitis, which is in line with the lack of management guidelines or multi‐center clinical studies. This highlights the unmet need for larger studies with development of appropriate treatment strategies in the pediatric population.


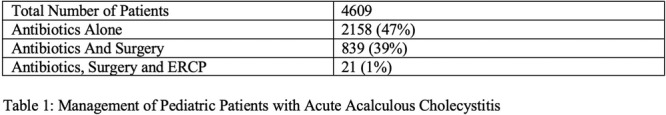


Table 1: Management of Pediatric Patients with Acute Acalculous Cholecystitis

## 304 NEITHER TENAPANOR NOR ITS MAJOR METABOLITE WERE DETECTED IN THE BREAST MILK OF HEALTHY LACTATING FEMALES AFTER 4 DAYS OF DOSING: A PHASE 1, OPEN‐LABEL, PHARMACOKINETIC STUDY

*Nastassja Williams* ^*1*^, *Darren Brenner* ^*2*^, *Karishma Raju* ^*1*^, *Kenji Kozuka* ^*1*^, *Yang Yang* ^*1*^, *Suling Zhao* ^*1*^, *Susan Edelstein* ^*3*^

^*1*^ *Ardelyx, Inc*., *Newark*, *CA*; ^*2*^ *Northwestern University Feinberg School of Medicine*, *Chicago*, *IL*; ^*3*^ *Ardelyx, Inc*., *Waltham*, *MA*

**Background:** Tenapanor, a first‐in‐class, small‐molecule inhibitor of intestinal sodium/hydrogen exchanger isoform 3 (NHE3), is FDA approved for the treatment of irritable bowel syndrome with constipation (IBS‐C) in adults. By inhibiting NHE3, tenapanor reduces absorption of sodium from the small intestine and colon, resulting in increased water retention within the intestinal lumen, which leads to softer stools and faster transit. Tenapanor is minimally absorbed following repeated twice daily (bid) oral administration. Plasma concentrations were below the limit of quantitation (<0.5 ng/mL) in the majority of samples from healthy subjects and patients with IBS‐C following repeated oral administration of tenapanor 50 mg bid.

At present, there is a paucity of clinical guidance for medication use in patients who are breastfeeding. Therefore, we conducted this study to determine the pharmacokinetics (PK) of tenapanor and its primary metabolite (M1) in breast milk and to assess the safety and tolerability of tenapanor in 7 healthy lactating females (a standard population for this type of study).

**Methods:** Participants received tenapanor 50 mg bid on days 1 through 3 and a single dose on day 4 (before breakfast). The first and last doses were administered at the clinic; interim doses were taken at home. Breast milk was collected on day 4 pre‐dose (hour 0), then at 1, 2, 4, 6, 8, and 24 hours post dose. Patients attended a follow‐up visit 5‐7 days after the last dose. Samples were analyzed using a validated high‐performance liquid chromatography–tandem mass spectrometry method to evaluate concentrations of tenapanor and its M1 metabolite. Any breast milk not used for PK analyses was discarded. PK and safety analyses were descriptive and exploratory.

**Results:** In total, 7 participants completed this study. They were predominantly White (71.4%; 5 of 7). Mean (SD) age at informed consent was 30.0 (5.07) years. Mean (SD) infant gestational age at delivery was 39.0 (1.91) weeks. Mean maximum observed concentration and lowest observed concentration during dosing interval estimates of tenapanor and M1 were 0.000 ng/mL, since all sample concentrations were below the limit of quantitation of 1.00 ng/mL at all timepoints (**Table 1**). Mean daily infant dose was 0.000 µg/kg/d for both tenapanor and M1. Mean (SD) maternal dose of tenapanor was 1275 (211.37) µg/kg/d and mean relative infant dose was 0.000 µg/kg/d. No unexpected treatment‐emergent adverse events (TEAEs) were reported (**Table 2**). The most common TEAEs were diarrhea (3 of 7 participants), flatulence (2 of 7), and nausea (1 of 7).

**Conclusions:** This study showed that tenapanor and its M1 metabolite were not present at detectable levels in the breast milk of healthy lactating females after repeated oral administration of tenapanor. The safety profile of tenapanor was similar to that seen in previous clinical trials of healthy volunteers.


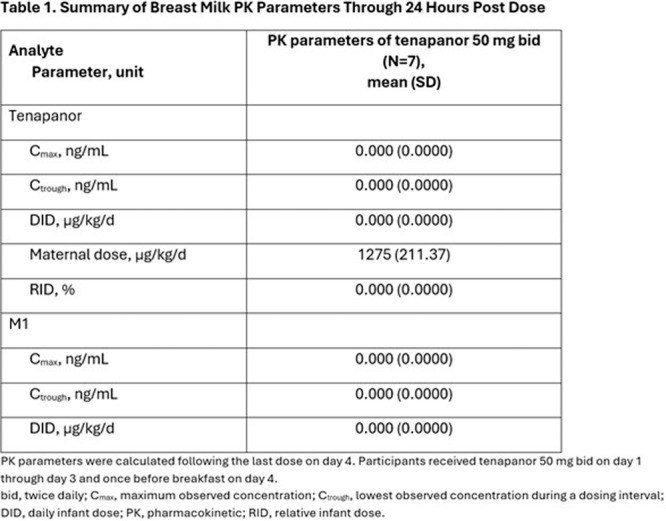



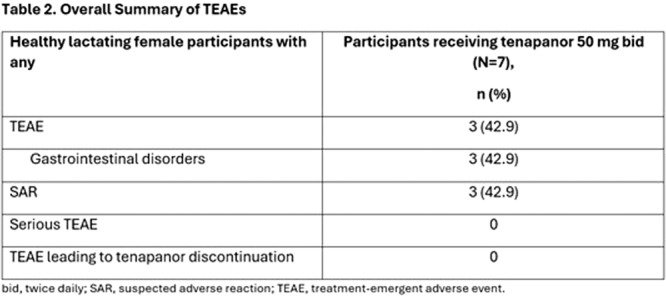


## 307 PRUCALOPRIDE FOR REFRACTORY UPPER TRACT SYMPTOMS IN PEDIATRIC ESOPHAGEAL ATRESIA

*Sabrina Karim*, *Shivani Mehta*, *Denis Chang*, *Peter Ngo*, *Jessica Yasuda*

*Gastroenterology, Hepatology & Nutrition*, *Boston Children's Hospital*, *Boston*, *MA*

**INTRODUCTION:** While prucalopride, a highly selective 5‐HT4 receptor agonist, is used to promote motility in gastroparesis, pseudo‐obstruction, and constipation, its role in upper tract symptoms in pediatric esophageal atresia (EA) is less well studied. Patients with a history of EA generally have some degree of esophageal dysmotility and often associated reflux and gastric emptying dysfunction. We therefore hypothesized that EA patients may benefit from prucalopride for symptoms of upper tract dysmotility.

**OBJECTIVES:** To describe clinical and histologic outcomes in children with EA started on prucalopride for symptoms of upper tract dysmotility.

**METHODS:** This was a single‐center retrospective cohort study of pediatric EA patients recommended to initiate prucalopride from October 2019 to April 2025. Children status post esophago‐esophageal anastomoses for repair of EA were included. Patients with non‐EA diagnoses and other anastomosis types were excluded. Primary outcome was clinical response following initiation of prucalopride, which was defined as “full” if symptoms were nearly or completely resolved, “partial” if partially improved, and “none” if no improvement was reported. Fisher's exact test was performed, comparing prucalopride starters to non‐prucalopride starters on the distribution of symptom resolution. Secondary outcomes included pre‐ and post‐prucalopride diagnostics (endoscopy, histology) and side effects related to prucalopride.

**RESULTS:** 66 EA patients with esophago‐esophageal anastomoses were prescribed prucalopride during the study period. The most common symptoms for medication initiation were nausea with or without vomiting (reported 48 times amongst sample) and dysphagia (reported 41 times). Twenty‐four patients (36%) did not start the medication, most commonly due to insurance denial. Of the 42 children who were treated with prucalopride, 16 (38%) reported full response, 11 (26%) reported partial, and 13 (31%) reported no response (**Table 1**). Two children have yet to follow‐up after medication initiation. Fisher's exact test comparing prucalopride starters to non‐prucalopride starters on the distribution of symptom resolution (“full”/“partial”/“none”) showed a significant association of prucalopride with symptom outcome (p = 0.02, **Table 2**).

Endoscopically, 20/42 of prucalopride starters had both pre‐ and post‐trial esophagogastroduodenoscopy (EGD) as shown in **Table 3**. While there was no clear trend for change in histopathologic inflammation in pre vs post‐prucalopride EGDs, there was an anecdotal resolution of erosive esophagitis in 2 patients (**Table 4**).

Prucalopride was discontinued in 26/42 (62%) children due to side effects (13/26), symptom resolution (8/26), or insurance denial/expense (5/26). On average, patients took prucalopride for 289 days prior to termination.

**CONCLUSION:** Prucalopride in this cohort was significantly associated with greater rates of symptomatic improvement. These findings suggest that prucalopride may improve symptoms in some pediatric EA patients, particularly those with dysphagia or gastric emptying dysfunction, and may help non‐responders to other multi‐modal therapy. Future prospective studies may clarify the role for prucalopride in improving objective markers of motility and/or endoscopic outcomes in pediatric EA.


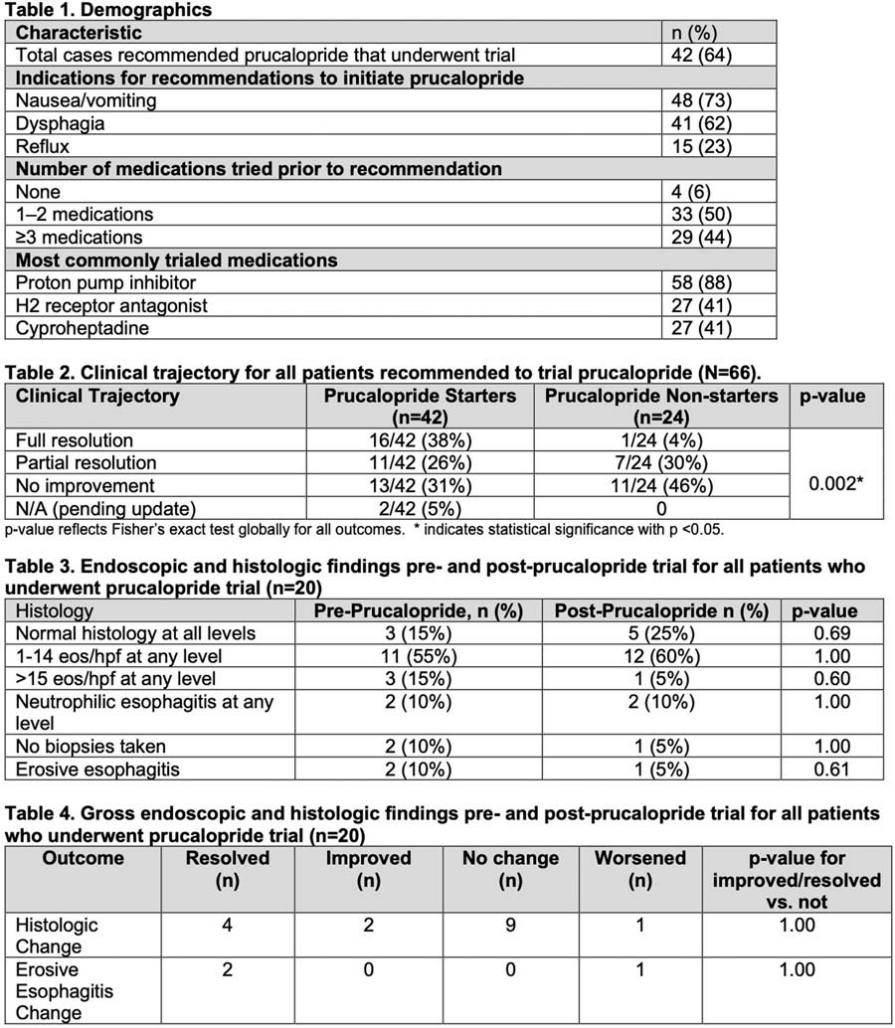


## 308 MORE THAN A FEEDING DISORDER: DIAGNOSTIC INSIGHTS AND COMORBIDITY PATTERNS IN ARFID

*Samantha Pendleton* ^*1*^, *Mojdeh Mostafavi* ^*2*^, *Gabby Bilyeu* ^*4*^, *Paula Braverman* ^*5*^, *Wael Sayej* ^*3*^

^*1*^ *Department of Internal Medicine and Pediatrics*, *Baystate Medical Center*, *Springfield*, *MA*; ^*2*^ *Department of Pediatric Gastroenterology & Nutrition*, *Massachusetts General Hospital*, *Boston*, *MA*; ^*3*^ *Department of Pediatric Gastroenterology & Nutrition*, *Baystate Medical Center*, *Springfield*, *MA*; ^*4*^ *Department of Emergency Medicine*, *Baystate Medical Center*, *Springfield*, *MA*; ^*5*^ *Department of Adolescent Medicine*, *Baystate Medical Center*, *Springfield*, *MA*

**Background:** Avoidant/restrictive food intake disorder (ARFID) is characterized by the sudden onset of picky eating and lack of appetite leading to significant weight loss or nutritional deficiency. Most often seen among adolescents, ARFID has shown an unprecedented eruption of diagnoses in the years of and following the COVID‐19 pandemic.

**Purpose:** We aimed to describe the clinical presentation and evaluation of patients presenting to pediatric clinics with gastrointestinal (GI) symptoms and ARFID. In particular, we aimed to identify factors precipitating ARFID symptoms, characterize diagnostic evaluation, and estimate the prevalence of organic and non‐organic GI disease as well as prevalence of pre‐existing psychiatric disorders.

**Methods:** Retrospective chart review was performed on all patients, aged 0‐21 years, who were diagnosed with ARFID (ICD‐10 Diagnostic Code F50.82) between 01/01/2019 and 06/30/2022, in one of our six pediatric care locations. Labs/interventions were only included if they occurred within 6 months prior and after the initial appointment. Of the 158 patients identified, 23 were excluded as they did not meet ARFID criteria upon secondary review. Descriptive analytics were performed on the remaining 136 patients to assess the prevalence of psychological factors and GI disease (organic and non‐organic) in patients with ARFID.

**Preliminary Results:** A GI complaint was present in 72.8% of patients, most commonly abdominal pain or nausea/vomiting, while 69.9% of patients presented with weight loss and 36.8% presented with changes in eating behavior.

Laboratory work up (CBC, ESR/CRP, celiac panel, and/or LFTs) was performed in 86.8% of patients, almost all of which occurred within a month of initial appointment. Endoscopy was performed in 29.4% of patients.

Additional GI diagnoses were present in 57.4% of patients, either prior to or during the workup of ARFID. A functional GI disease was diagnosed in 56.6%, most commonly IBS. 11.8% of patients were diagnosed with an organic GI disease, though notably 62.5% of organic diseases were present prior to diagnosis of ARFID. Only 5 patients were diagnosed with organic disease during their workup for ARFID, and all continued to have symptoms of ARFID after treatment of their organic disease.

GERD was commonly described as a symptom and present in 12.5% of patients, though only 2 patients who underwent endoscopy had findings of organic disease.

Pre‐existing psychiatric comorbidities were present in 85.3% of patients, most commonly anxiety (64.4%), depression (28.1%) and ADHD (21.5%).

In 32.6% of patients, family/friends described the patient as picky eaters in early childhood. Only 6.6% of patients reported a choking event prior to diagnosis, of which 77.8% reported the event occurring within a year prior to diagnosis. COVID was diagnosed prior to presentation in 6.6% of patients.

**Conclusion:** Our study supports previous findings of a strong prevalence of psychiatric and gastrointestinal comorbidities in patients with ARFID, particularly anxiety and IBS. Regardless of location of presentation (e.g. GI clinic, adolescent clinic, etc.), most patients underwent laboratory workup to rule out organic causes. Despite further workup after abnormal labs, only a few patients were diagnosed with organic disease, and all patients with organic disease still required additional treatment of their ARFID after treatment of their GI disorder. This suggests the importance to continue multidisciplinary treatment of ARFID focused not only on symptom management, but continued psychiatric support and family based therapy.

We hypothesized there would be a large prevalence of patients described as picky eaters in early childhood, which was confirmed by our study. We also hypothesized there would be a significant prevalence of patients with previous choking episodes and COVID, however these events were less prevalent than predicted. Choking episodes did occur in over half of the patients presenting with a chief complaint of dysphagia, so may represent an inciting event in a very specific subset of ARFID. We did not collect data on the presence of any other viral illness other than COVID prior to symptom onset, which may be an important factor to assess as an inciting event in the future.

Our design is limited in that review of charts was only done within 6 months of the initial appointment. This has likely led to underreporting of previous workup as well as future workup that may have occurred later in the disease process. An additional study could look at subsequent visits in the same set of patients and analyze the effectiveness of interventions as well as prevalence of any additional GI and/or psychiatric disorders diagnosed later in adolescence and as they transition into adulthood. This would be especially interesting given the lack of awareness of ARFID in the adult population.

## 309 FEASIBILITY OF USING CAREGIVER OBTAINED MUAC Z‐SCORE MEASUREMENT IN MONITORING MALNUTRITION IN THE PEDIATRIC PATIENT

*Michelle Roach*, *Dana Bakula*, *Saiyara Baset*, *April Escobar*, *Rachel Graham*, *Aileen Har*, *E. Nicole Jennison*, *Amanda May*, *Elizabeth Melia*, *Kristina Nash*, *Corey Schurman*, *Kathryn Yeldell*, *Sarah Edwards*

*Gastroenterology*, *Children's Mercy Kansas City*, *Kansas City*, *MO*

**Introduction:** One in 37 children under the age of 5 has pediatric feeding disorder (PFD) and is at risk for malnutrition. Over the last 5 years we have seen a large increase in the number of telehealth visits for pediatric medical visits. This improves access to care but makes it challenging to monitor weight and nutrition status. Alternative evidence‐based methods, beyond in‐clinic anthropometrics (height/weight), are needed to monitor nutrition status. Mid‐upper arm circumference (MUAC) insertion tape is a highly effective tool for monitoring nutritional status with a single data point. It is also made of paper and is cost effective to send home or mail to families. There has been a great deal of research outside of the US in undeveloped areas using MUAC insertion tape to screen infants and children for severe acute malnutrition, including studies which found that even uneducated caregivers in other countries were able to accurately measure with MUAC insertion tape following brief training and that these caregivers maintained those skills a month after training. There have been no US studies of the feasibility of teaching caregivers in the US to use MUAC insertion tape since 2001. Further research on the feasibility and accuracy of parent/caregiver obtained MUAC measurement is needed in the US.

**Methods:** We conducted a prospective cohort‐based study of the feasibility of teaching caregivers of children aged 2 months to 16 years to use MUAC z‐score insertion tape. Families were recruited from an interdisciplinary feeding and swallowing program. Families were eligible if they spoke English or Spanish. Families were taught how to use MUAC tape during a clinic visit by a registered dietitian (RD). Education included how to choose the correct side of tape based on child age, how to determine mid‐point in upper arm, how to feed the end of the tape through the slots, how to tighten the tape appropriately, correct arm position to read tape, and correct line for age when reading tape. Families were sent home with a kit including MUAC tape, an instructional guide with step‐by‐step images demonstrating the correct technique for using MUAC tape, Ziplock bag, and magnet clip. At the child's next clinic visit (2‐6 months later) parents were asked to measure their child's arm with the MUAC tape in front of a study team member with no assistance and give their reading. If any part of their technique was incorrect, reteaching was performed and the caregiver obtained a second measurement. After caregiver measurements were complete, a measurement was done by a RD. Parents completed a survey to assess the feasibility and acceptability of completing MUAC measurements at home.

**Results:** All participants (N=44) were able to use the tape appropriately and match color designation with the score obtained by the RD after teaching in the initial visit. Most families were White (69%), followed by Hispanic (23.8%), Black (12%), Asian (2%), and Native Hawaiian/Pacific Islander (2%). Most (86%) families spoke English, while14% spoke Spanish. Half (52%) had Medicaid and 38% were WIC‐eligible. 15 families completed the second study visit. Most families (84%) required re‐teaching by the RD at the second study visit. After re‐teaching, 100% of parents were able to report correct color designation and MUAC measurements in cm to nearest tenth as witnessed by team. A subset of families (n=10) completed the feasibility survey. Most parents reported no difficulty (45.5%) or some difficulty (45.5%). Only one parent was not able to use the tape at home. More than half (64%) reported needing assistance to hold their child still when using the MUAC tape at home. All families (100%) said they would be willing to continue using the MUAC tape at home.

**Discussion:** This was the first study since 2001 to assess the feasibility of teaching parents in the US to use MUAC insertion tape at home to monitor nutritional status. In a diverse group of families, we identified that caregivers found MUAC insertion tape measurement at home to be extremely feasible and acceptable. Caregivers were highly successful in learning to use MUAC insertion tape. Most caregivers required re‐teaching of the skill, but with re‐teaching were highly successful 2‐6 months after initial training. There were some potential barriers to home implementation, such as needing a second caregiver to hold the child, but all caregivers reported willingness to continue using the tape at home. This study supports the feasibility of using MUAC insertion tape as a method to monitor child nutritional status from home in the context of telehealth visits or for home‐based monitoring between medical appointments.

## 311 EPIDEMIOLOGY AND PROCEDURAL TRENDS OF ESOPHAGEAL ACHALASIA IN PEDIATRIC POPULATION: A NATIONAL ANALYSIS

*Judy Suh*, *Collins Odhiambo*, *Manu Sood*

*Pediatrics*, *University of Illinois Chicago College of Medicine*, *Chicago*, *IL*

**Introduction:** Lower esophageal sphincter achalasia in children is a rare disease, and most published data regarding its epidemiology are based on single‐center experiences. This study characterizes patient epidemiology and procedural trends in treatment options using a nationally representative dataset.

**Methods:** This study utilized data from the Healthcare Cost and Utilization Project (HCUP), the largest publicly available longitudinal hospital care database in the United States. Specifically, data were extracted from the Kids’ Inpatient Database (KID) covering a 12‐year span from 2012 to 2024 to investigate national inpatient trends related to achalasia in pediatric populations. Comprehensive data cleaning and preprocessing were performed to ensure accuracy and consistency across study variables. Data coding and mining were conducted using SAS version 8.3. The cleaned dataset was analyzed to evaluate primary outcomes, including the primary diagnosis of achalasia, procedural differences, and other comorbidities. Additional variables of interest included patient demographics (e.g., age, sex, race/ethnicity), comorbid conditions, diagnostic evaluations, frequency of therapeutic interventions, and length of stay stratified by procedure type. Results were summarized using descriptive statistics, with categorical variables presented as frequencies and percentages. All data were organized and reported in tabular format for clarity and ease of interpretation.

**Results:** Among the 737 inpatient pediatric patients with a primary diagnosis of achalasia, no in‐hospital deaths were reported, indicating a favorable safety profile for the evaluated procedures. Most patients were over 12 years of age (68%), with the remainder distributed across the 5–12 years (26%) and ≤5 years (6%) age groups. Males comprised a slightly higher proportion (53%) compared to females (48%).

The most commonly documented comorbidities among patients included gastroesophageal reflux disease (GERD) without esophagitis (n = 70), severe protein‐calorie malnutrition (n = 34), uncomplicated asthma (n = 13), and pneumonitis due to aspiration (n = 11), reflecting the complexity of patient presentations.

Regarding procedural trends, Peroral Endoscopic Myotomy (POEM) emerged as the most frequently performed procedure, accounting for 34% (n = 240) of all cases. This was followed by esophagomyotomy, including Heller myotomy (open or laparoscopic, with or without fundoplication), at 17% (n = 122); open Heller myotomy alone at 7% (n = 47). Transendoscopic balloon dilation of the upper/lower esophagus or esophagogastric junction was performed less frequently, at 5% (n = 36). 72% of patients required hospitalization for more than one day, regardless of procedure type. A stratified analysis comparing POEM with other procedures revealed no statistically significant differences in age distribution (p = 0.194) or length of stay (p = 0.301).

**Conclusions:** Achalasia in the pediatric population is most prevalent among those over 12 years of age, with a slightly higher incidence in males. POEM is shown to be the most frequently performed procedure, followed by Heller myotomy and transendoscopic balloon dilation. No significant differences in age distribution or length of hospital stay were found between POEM and other procedures.

## 313 TRENDS IN THE DIAGNOSIS AND MANAGEMENT OF INFANTILE REFLUX AND GERD IN PRIMARY PEDIATRIC CARE: A 10‐YEAR RETROSPECTIVE ANALYSIS

*Kirsten Young* ^*3*^, *Mark Fishbein* ^*1,2*^

^*1*^ *Pediatrics*, *Ann and Robert H Lurie Children*, *Chicago*, *IL*; ^*2*^ *Pediatrics*, *Northwestern University Feinberg School of Medicine*, *Chicago*, *IL*; ^*3*^ *Family Medicine*, *Northwestern Medicine Delnor Hospital*, *Geneva*, *IL*

**Background:** Gastroesophageal reflux (GER) is a common physiologic process in infants, while gastroesophageal reflux disease (GERD) represents a pathologic condition warranting clinical intervention. Over the past decade, national guidelines, including those from NASPGHAN, have recommended a conservative approach to managing infantile reflux, discouraging routine pharmacologic treatment in favor of behavioral modifications and limiting the use of acid‐suppressing medications to cases of confirmed GERD. Despite these recommendations, prior studies suggest persistent overdiagnosis of GERD and continued pharmacologic management without clear indications. This study investigates whether primary care pediatricians have adjusted their diagnostic and treatment approaches to align with updated guidelines.

**Methods:** We conducted a retrospective analysis of electronic health record data from pediatric primary care clinics within the Lurie Pediatric Primary Care Network between 2014 and 2024. The study population included infants aged 0–6 months diagnosed with GERD (with or without esophagitis) or infantile reflux. Co‐morbid conditions listed were dysphagia, milk allergy, and colic. Referrals to speech language pathologist, occupational therapist, and gastroenterologist were also recorded. Infants with prematurity or major congenital, genetic, or neurologic conditions were excluded. Annual rates of diagnoses were determined by comparison to the total number of age‐matched healthy infants seen during the same time intervals.

**Results:** Cumulative diagnoses included GERD with or without esophagitis (n=12311) and infantile reflux (n=420). Co‐morbid conditions included dysphagia (n=640), milk allergy (n=456), and colic (n=1321). Healthy controls included n=83705 infants. The average age at initial diagnosis was 2.59 months. The cumulative rate of GERD with or without esophagitis was 14.7%, infantile reflux was 0.5%, dysphagia was 0.8%, milk allergy was 0.5%, and colic was 1.6%. From 2014–2024, GERD diagnoses declined (R^2^=0.8784, p=0.00002), while infantile reflux (R^2^=0.7229, p=0.00091) and colic (R^2^=0.4705, p=0.01979) diagnoses increased slightly. H2 blocker therapy is currently peaking at 59.23% and PPI therapy is at a nadir at 3.78% in GERD diagnoses. Referrals to specialty services were infrequent across all diagnostic groups, with average referral rates of less than 0.5% for speech language pathologist, 0.2% for occupational therapy, 0.05% for pediatric gastroenterology.

**Conclusions:** Among primary care physicians, GERD diagnoses have declined over the past decade in accordance with present guidelines. H2 blocker therapy is much preferred over PPI in this cohort. Infantile reflux was diagnosed less and its relationship to GER was unclear. Few infants received co‐morbid diagnoses of dysphagia, milk allergy, and colic. Referrals for GERD‐like symptoms related to dysphagia and colic/unsettledness to speech language pathology and occupational therapy respectively appeared sparse. Referrals to pediatric gastroenterologist in this cohort were also quite low.

**Summary:** The trend of reduced GERD diagnoses is welcome, however more attention is required to properly address infants with GERD‐like symptoms including dysphagia and colic.


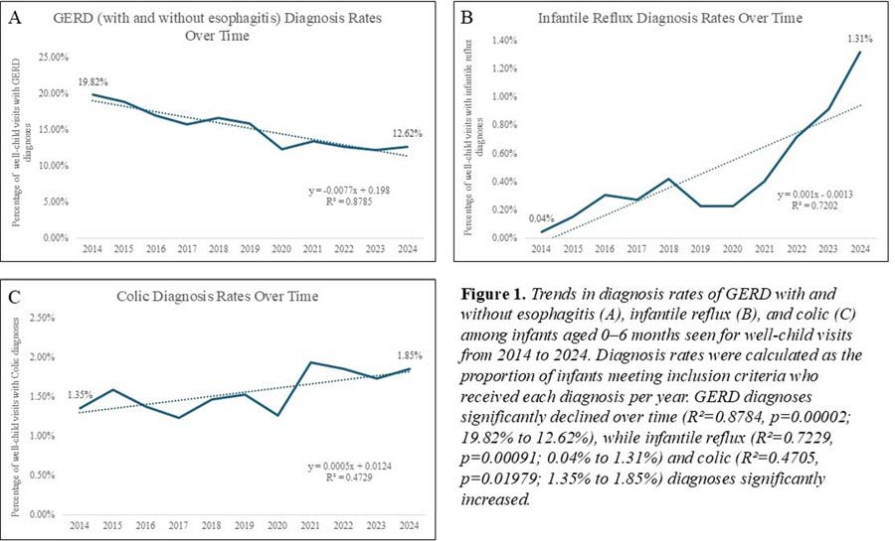


Changing rate of GERD, infantile reflux, and colic diagnoses

## 318 SAFETY AND EFFICACY OF RECTAL INDOMETHACIN FOR PREVENTION OF POST‐ERCP PANCREATITIS IN PEDIATRIC PATIENTS

*Mary Camacho* ^*1*^, *Emma Blythe* ^*4*^, *Badih Elmunzer* ^*5*^, *Elaine Odiase* ^*3*^, *Devika Gandhi* ^*2*^, *Christopher Fritzen* ^*3*^, *Reuven Cohen* ^*3*^, *Cary Sauer* ^*3*^, *Field Willingham* ^*2,3*^

^*1*^ *Medicine*, *Emory University School of Medicine*, *Atlanta*, *GA*; ^*2*^ *Digestive Diseases*, *Emory University*, *Atlanta*, *GA*; ^*3*^ *Children's Healthcare of Atlanta Arthur M Blank Hospital*, *Atlanta*, *GA*; ^*4*^ *Health Services Research Center*, *Emory University*, *Atlanta*, *GA*; ^*5*^ *Gastroenterology and Hepatology*, *Medical University of South Carolina*, *Charleston*, *SC*

**Introduction:** Peri‐procedural rectal indomethacin has been shown to decrease the incidence of post‐endoscopic retrograde cholangiopancreatography (ERCP) pancreatitis (PEP) in adult patients undergoing ERCP. This practice has been generalized to pediatric populations, despite an absence of data on safety or efficacy. This study evaluated the safety and efficacy of rectal indomethacin in pediatric patients undergoing ERCP.

**Methods:** We conducted a retrospective cohort study of pediatric patients who underwent ERCP at two tertiary academic hospitals from November 2009 to August 2024. Safety outcomes included bleeding and acute kidney injury (AKI). The efficacy outcome was the incidence of PEP with and without indomethacin at 100 mg >30 kg and 50 mg <30 kg. Descriptive statistics, univariate analysis, and binary logistic regression adjusting for 18 covariates were performed.

**Results:** 906 ERCPs were performed in 448 patients. Indomethacin was administered in 757 (83.6%) procedures and not in 148 (16.3%). Bleeding occurred in 3.2% vs. 3.4% (p=0.90), and AKI in 1.1% vs. 0.7% (p=0.67). PEP incidence was 3.0% with indomethacin vs. 9.5% without (p<0.05). On multivariate analysis, indomethacin significantly reduced PEP risk (OR=0.28, p<0.05) without increasing bleeding or AKI. Findings were similar in subgroup analysis of index ERCPs with no increased odds of bleeding or AKI and significantly lower odds of PEP (OR=0.16, p<0.05).

**Discussion:** The use of peri‐procedural rectal indomethacin was safe and effective, with no increased bleeding or AKI and a greater than threefold reduction in PEP in pediatric patients. These findings support the use of indomethacin prophylaxis in pediatric patients at 100 mg >30 kg and 50 mg <30 kg.


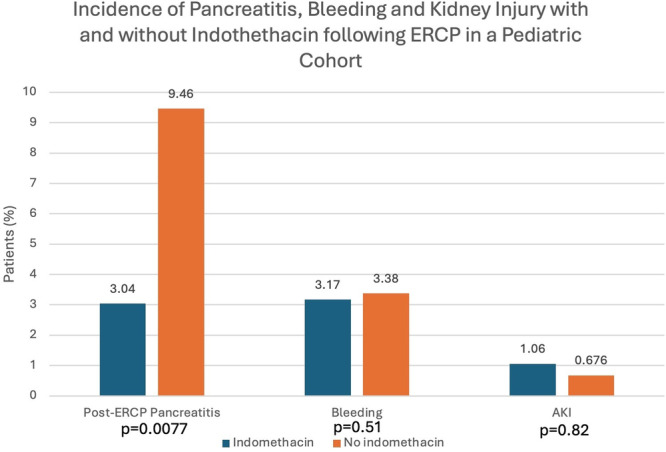


Post‐ERCP Pancreatitis developed in 23 of 757 (3.0%) of the indomethacin group and 14 of 148 (9.5%) of the control group (OR=0.28, 95% CI [0.11‐0.71], p=0.0077). Bleeding occurred in 24 of 757 (3.2%) of the indomethacin group and 5 of 148 (3.4%) of the control group (OR=0.66, 95% CI [0.19‐2.28], p=0.514). AKI occurred in 8 of 757 (1.1%) of the indomethacin group and 1 of 148 (0.7%) of the control group (OR=1.31, 95% CI [0.13‐13.65], p=0.822).


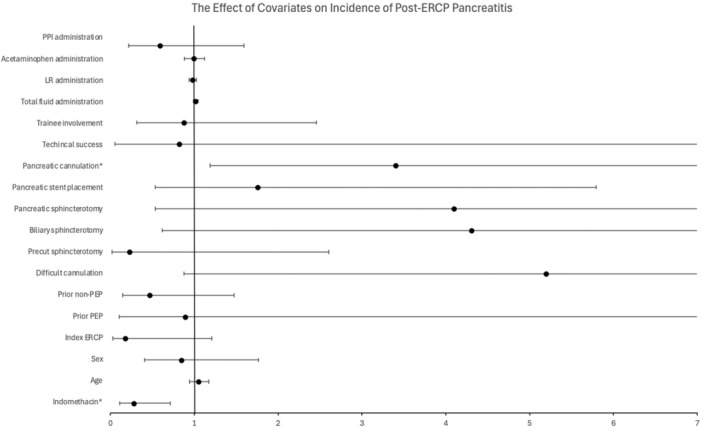


On logistic regression, only pancreatic cannulation was significantly associated with increased odds of PEP (OR=3.40, 95% CI [1.19‐9.72], p=0.022). No other covariates had a statistically significant effect on the incidence of PEP.

## 320 REDUCING WAIT TIME FOR PEDIATRIC GASTROINTESTINAL ENDOSCOPIC PROCEDURES: A QUALITY IMPROVEMENT PROJECT

*Jennifer Colombo* ^*5*^, *Nicholas Clark* ^*1*^, *Laura Shroyer* ^*5*^, *Dustin Hahn* ^*2*^, *Jonathan Patterson* ^*3*^, *Kenneth Sam* ^*4*^

^*1*^ *Pediatrics*, *Children's Mercy Kansas City*, *Kansas City*, *MO*; ^*2*^ *Ambulatory Services*, *Children's Mercy Kansas City*, *Kansas City*, *MO*; ^*3*^ *Perioperative Services*, *Children's Mercy Kansas City*, *Kansas City*, *MO*; ^*4*^ *Performance Improvement*, *Children's Mercy Kansas City*, *Kansas City*, *MO*; ^*5*^ *Gastroenterology*, *Children's Mercy Kansas City*, *Kansas City*, *MO*

**Background:** Diagnostic delays can lead to poor health outcomes and suboptimal patient and family experiences. We aimed to reduce the average wait time for gastrointestinal endoscopic procedures (procedure request to procedure completion) from 145 days to 17 days (88% reduction) within 5 months (11/2023 to 3/2024).

**Methods:** A3 methodology was used. Primary intervention: multi‐day, multidisciplinary improvement workshop. Outcome Measures: 1) procedure request date to procedure completion date; 2) percent favorable patient satisfaction score for timeliness of access to care. Process Measures: 1) procedure request date to scheduled date; 2) procedure scheduled date to procedure completion date. Balancing Measure: number of procedures scheduled per weekday. Control charts assessed the impact of interventions.

**Results:** Following the primary intervention (10/2023), special cause improvement was seen for all but the Balancing Measure. Outcome Measure 1 achieved sustained reduction in mean monthly wait time from 145 to 26.4 days (82% reduction) while Outcome Measure 2 achieved sustained improvement in patient satisfaction from 41.3% to 50.2% (22% relative improvement; Fig 1). Process Measures 1 and 2 had sustained reductions from 56.5 to 14.7 days (74% reduction) and 89.1 to 20.7 days (77% reduction), respectively (Fig 2). Balancing Measure also remained unchanged at a monthly average of 11.7 procedures scheduled per weekday.

**Conclusions:** Using A3 improvement methodology, we achieved an 82% sustained reduction in endoscopic procedure wait times with a concurrent 22% improvement in patient satisfaction. Procedures are now routinely completed within 1 month of the request date.

## 323 FORREST CLASSIFICATION: A NEW APPROACH IN THE PEDIATRIC SETTING

*Heather Grabow* ^*1*^, *Claire Woods* ^*2*^, *Lauren Hamilton* ^*3*^, *Paul Tran* ^*4*^

^*1*^ *Medical Education ‐ Residency*, *Phoenix Children's Hospital*, *Phoenix*, *AZ*; ^*2*^ *Creighton University School of Medicine ‐ Phoenix Health Sciences Campus*, *Phoenix*, *AZ*; ^*3*^ *The University of Arizona College of Medicine Phoenix*, *Phoenix*, *AZ*; ^*4*^ *Gastroenterology*, *Phoenix Children's Hospital*, *Phoenix*, *AZ*

**Background:** The Forrest Classification tool, developed in 1974 for grading non‐variceal upper gastrointestinal (GI) bleeds, is widely used by adult gastroenterologists to assess rebleed risk. However, its use in pediatrics is undocumented. Building on a 2024 proof‐of‐concept study, we analyzed an expanded pediatric cohort.

**Objective:** The primary aim was to assess inter‐rater reliability of Forrest Classification in pediatrics. Secondary aims included evaluating associations between score and rebleeding, time to endoscopy, pre‐procedure interventions, blood products, and hemostatic intervention. Rebleed rates were also analyzed in context of these variables to assess the value of temporizing measures and a new approach was developed.

**Methods:** A retrospective chart review (2016–2023) using ICD‐10 codes for upper GI hemorrhage identified 82 pediatric cases with visible lesions on endoscopy. Poor image quality cases were excluded. Five reviewers (ranging from first‐year fellow to 20+ years of experience) assigned Forrest scores (Ia, Ib, IIa, IIb, IIc, III) to de‐identified photos. Inter‐rater reliability was calculated. For secondary analysis, consensus was defined as >40% agreement without split. Forrest scores were grouped based on proposed tool: active bleeding (Ia, Ib), suspected vessel (IIa, IIb), and no vessel (IIc, III).

**Results:** No type Ia lesions were identified. 8 split‐agreement cases were excluded. Final breakdown: III (38), IIc (15), IIb (5), IIa (7), Ib (9).

*Inter‐rater reliability*

Fleiss Kappa was statistically significant at 0.385. Consensus reached in 90% (74/82); unanimous in 24%, four‐agree/one‐disagree in 33%, and three‐agree/two‐disagree in 29%. In 13%, consensus came from two un‐split matching responses. Graders were conflicted between IIc and III 45% of the time.

*Secondary Aims*

Pre‐Endoscopy: Lesions with bleeding/suspected vessels had longer time to endoscopy (p=0.0312) and were more likely to receive octreotide (p=0.0019) yet patients who had octreotide pre‐endoscopy often rebled (p<0.0001). Proton pump inhibitor (PPI) use prior to endoscopy was also linked with rebleed (p=0.0009) and need for blood products (p<0.0001). 72.2% of PPI users were inpatients with higher‐acuity lesions as inpatient status was higher in vessel‐related lesions Ib‐IIb (62%) vs non‐vessel lesions IIc and III (30%). Of those with high dose PPI use, 58.8% did not rebleed while those without PPI use had 97.8% that did not rebleed.

Hemostasis: Endoscopic intervention occurred 44.4% of the time for active bleed, 66.6% for suspected vessel lesions and 19% in those with no vessel. Use of clips differed significantly between vessel categories (p=0.0079); 58% in suspected vessel, 22% in active bleeding, 15% in non‐vessel. Cautery use was 44% for active bleeds, 42% for suspected vessels, and 11% for non‐vessel. Epi use was not significant. Overall, clip use was 24.4%, cautery 22%, and epi 3.7%. Lesions that received any intervention had higher rebleed rates.

Rebleed: Rebleed occurred in 12.2% overall. IIa had the highest rebleed rate of 42.9% as a part of suspected vessel subcategory which had a rebleed rate of 33.3% including IIb (20%). Remaining lesion rates include Ib/bleeding (22%), and non‐vessel (5.6%) with III (7.9%), and IIc (0%). Blood products were used most in IIa (71.4%) and IIb (60%). Rebleeds mostly occurred within 1–4 days of endoscopy (average: 1.75 days); two outliers occurred later.

**Discussion:** Forrest Classification is reliable in pediatrics but may benefit from simplification. Original Forrest rebleed patterns mostly align with our data, though Ib and IIc had lower rates in our cohort. IIa/IIb and IIc/III lesions showed enough vessel and acuity similarity to warrant category grouping. With such, a simplified, vessel‐focused classification could improve pediatric risk stratification and guide interventions.

Octreotide and PPI use did not reduce rebleed risk. High‐dose PPI and octreotide use were more common in hospitalized patients with higher‐acuity lesions, suggesting severity driving rebleeds may not respond to medical therapy. This underscores the importance of timely endoscopy over reliance on medication.

Hemostatic intervention was rare but linked to higher rebleed, possibly due to higher initial severity in those cases. Whether interventions are insufficient or simply used on high‐risk lesions remains unclear. Regardless, monitoring high‐risk lesions (bleeding or vessel suspected) for 2–4 days post‐endoscopy may be prudent.

**Conclusion:** This study presents a simplified, vessel‐based rebleed risk model derived from pediatric cases at a high‐volume center. Findings suggest this Forrest Classification model can be adapted to pediatric use and inform procedural decisions, monitoring, and pre‐endoscopy therapy. Future work will assess correlation with BUN/Cr ratios and further validate this modified tool.

## 326 FLUSHING OUT THE PROBLEM: A STANDARDIZED, VISUAL‐BASED PROTOCOL TO IMPROVE PEDIATRIC BOWEL PREPARATION

*Tala Haddadin* ^*3,2*^, *Jessica Black* ^*1*^, *Connor P. Kelley* ^*2*^, *Anas Bitar* ^*3*^

^*1*^ *Pediatrics*, *Banner University Medical Center*, *Tuscon*, *AZ*; ^*2*^ *Pediatrics*, *Steele Children's Research Center, University of Arizona*, *Tuscon*, *AZ*; ^*3*^ *Pediatrics Gastroenterology*, *Banner University Medical Center*, *Tuscon*, *AZ*

**Introduction:** Colonoscopy plays a vital role in pediatric gastroenterology as a key diagnostic tool for identifying various gastrointestinal conditions. Its effectiveness is closely tied to bowel preparation quality, which ensures adequate visualization and diagnostic accuracy. However, inadequate bowel preparation occurs in over 25% of pediatric colonoscopies, often leading to prolonged or incomplete procedures, increased healthcare costs, or repeat procedures with additional anesthesia exposure. Patients and guardians often struggle with adherence to prep regimens, and the lack of a standardized pediatric protocol contributes to variability in clinical practice. To address this, we developed and implemented a standardized preparation protocol for pediatric colonoscopy patients and their caregivers.

**Methods:** We created an evidence‐based bowel preparation protocol specifically for pediatric patients. Key features included weight‐based stratification of preparation regimens; visual shopping lists of required products; a stool chart to assess the level of cleanout and determine if additional laxatives are needed; and guidance on hydration and dietary restrictions. A detailed 24‐hour timeline outlined specific actions and expectations, enabling caregivers to track preparation progress.

This protocol was introduced at Banner University Medical Center ‐ Tucson. To evaluate its effectiveness, we performed a retrospective review of pediatric colonoscopy reports over a 12‐month period before implementation and an 8‐month period after. Data collected included patient age, sex, procedure indication, bowel prep quality, cecal intubation time (from scope insertion to cecum), ileal intubation success, and proceduralist identity. We used linear regression to assess changes in cecal intubation time.

**Results:** We analyzed data from 130 pediatric patients—72 pre‐intervention (Aug 2024–Jul 2025) and 48 post‐intervention (Aug 2025–Apr 2026). Females predominated in both groups (58.1% pre vs. 70.8% post). The median age was closely similar (14 vs. 15 years). Abdominal pain was the most common indication for colonoscopy.

Unadjusted linear regression showed a statistically significant reduction in cecal intubation time following protocol implementation—an average decrease of 5.6 minutes (95% CI: 9.4 to 1.7; p < 0.01). Including other variables did not improve the model. Although proceduralist identity had some influence, it was excluded due to heterogeneity across groups.

**Discussion:** The purpose of this quality improvement project was to develop and implement a standardized pediatric bowl cleanout protocol for our pediatric patients undergoing colonoscopy. Our results indicate that this protocol effectively reduced the cecal intubation time in pediatric colonoscopies.

Finishing endoscopic procedures in a timely manner provides several benefits spanning patient care, clinical efficiency, healthcare costs, and overall institutional performance. Timely completion reduces anesthesia‐related complications, shortens wait times, and facilitates faster transitions to recovery and discharge. It also enhances patient satisfaction by minimizing delays and reducing anxiety for both patients and caregivers. From an operational standpoint, it optimizes scheduling and improves resource utilization, including staff time, endoscopy suite availability, and reprocessing workflows. Additionally, it helps lower staffing costs by reducing the need for overtime and contributes to a healthier work environment by minimizing stress and burnout among healthcare staff.

The updated protocol's strengths lie in its evidence‐based foundation and its attention to common barriers in pediatric care. By providing visual aids for shopping lists of necessary products and cleanout levels, and tailoring instructions to specific weight categories, the protocol improved the clarity and accessibility of bowel preparation instructions, which contributed to better understanding and adherence among our patients and caregivers. As a single‐center initiative involving training fellows, the findings of this relatively short‐term quality improvement project are inherently limited in generalizability. Future work using similar or optimized protocol for a longer period of time and analyzing procedures performed by faculties vs trainee would allow us to gain a more comprehensive understanding of the impact of our protocol.

## 327 PEDIATRIC ENDOSCOPIC MUCOSAL RESECTION: A 10‐YEAR SINGLE‐CENTER EXPERIENCE

*Brett Hoskins* ^*1*^, *Jared Grabau* ^*3*^, *Douglas Rex* ^*2*^

^*1*^ *Division of Pediatric Gastroenterology, Hepatology, and Nutrition, Department of Pediatrics*, *Indiana University School of Medicine*, *Indianapolis*, *IN*; ^*2*^ *Division of Gastroenterology and Hepatology, Department of Medicine*, *Indiana University School of Medicine*, *Indianapolis*, *IN*; ^*3*^ *Department of Medicine*, *Marian University Wood College of Osteopathic Medicine*, *Indianapolis*, *IN*

**Objectives:** Endoscopic mucosal resection (EMR) is well established in adult gastroenterology but remains underutilized in pediatrics due to limited data, training opportunities, and equipment. This study presents a 10‐year, single‐center experience with conventional hot and cold snare EMR, band‐assisted (B‐EMR), and underwater EMR (U‐EMR) techniques in pediatric patients.

**Methods:** A retrospective review was conducted of all EMR procedures performed in patients under 21 years of age between 2015 and 2025 at a tertiary care children's hospital. Data on patient demographics, lesion characteristics, procedural details, pathology, and outcomes were collected and analyzed descriptively.

**Results:** Twenty EMRs were performed in 18 patients (mean age 17.1 years, range 3–20). The most common underlying diagnoses included familial adenomatous polyposis (*n*=7), sporadic mucosal polyps (*n*=4), subepithelial lesions (*n*=4), juvenile polyposis syndrome (*n*=2), Peutz‐Jeghers syndrome (*n*=1), and Lynch syndrome (*n*=1). Lesions ranged from 6 to 80 mm and were located throughout the gastrointestinal tract, most commonly in the colon (*n*=9), duodenum (*n*=5), and esophagus (*n*=3). Techniques included hot snare EMR (*n*=9), cold snare EMR (*n*=6), B‐EMR (*n*=4), and U‐EMR (*n*=1). Complete resection was achieved in 95% of cases, with one incomplete resection requiring surgical management for adenocarcinoma. B‐EMR was safely applied to subepithelial lesions. No delayed complications occurred.

**Conclusions:** EMR is feasible, safe, and effective in pediatric patients for both mucosal and subepithelial lesions. Broader adoption in pediatric practice will require expanded training, multidisciplinary collaboration, and development of pediatric‐specific guidelines. These findings support EMR as a valuable therapeutic option in complex pediatric gastrointestinal disease.


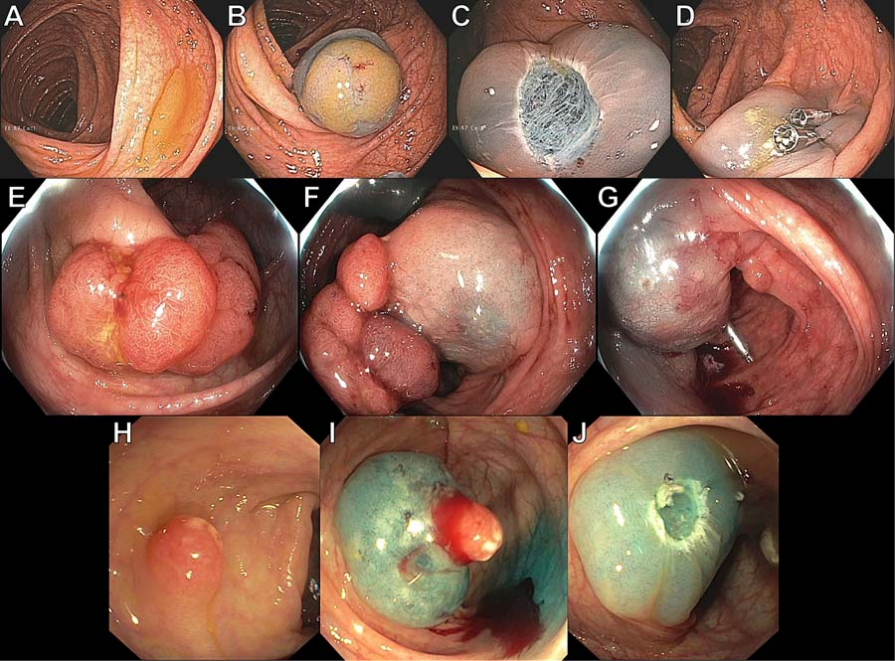


Representative cases of hot snare endoscopic mucosal resection (HS‐EMR) used for polyp removal in pediatric patients.

**A–D:** Flat polyp in the ascending colon of a 20‐year‐old patient, shown pre‐submucosal injection (A), post‐lifting (B), following en bloc resection (C), and after clip closure of the mucosal defect (D).

**E–G:** Semi‐pedunculated polyp in the hepatic flexure of a 17‐year‐old patient, shown pre‐submucosal lifting (E), post‐lifting (F), and following piecemeal resection with clip closure (G).

**H–J:** Semi‐pedunculated polyp in the transverse colon of a 5‐year‐old patient, shown pre‐submucosal lifting (H), post‐lifting (I), and following en bloc resection (J).


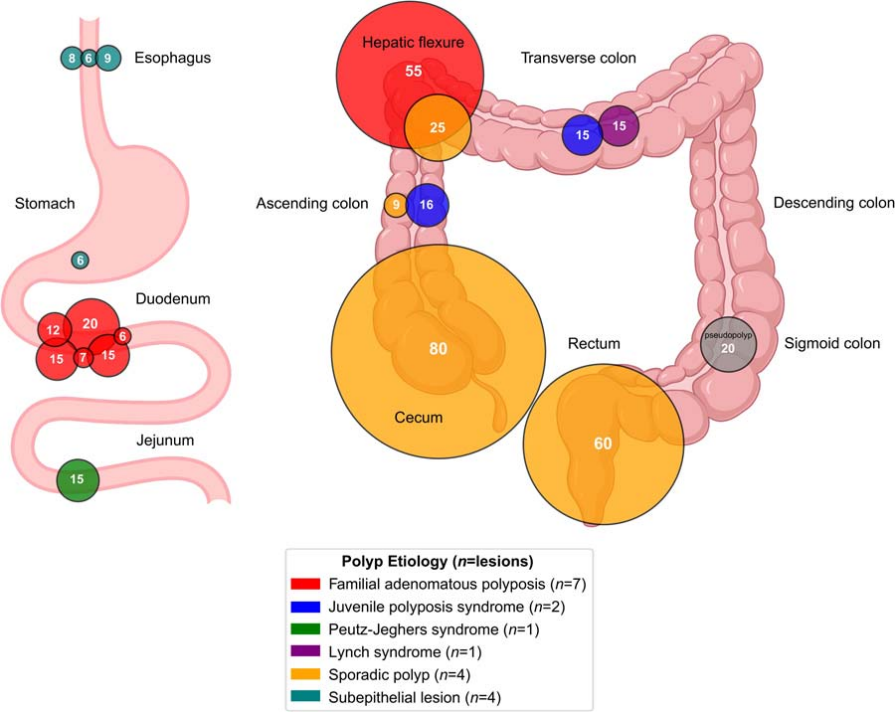


Anatomic distribution and size of resected polyps during pediatric endoscopic mucosal resection (EMR). Each circle corresponds to a polyp showing size comparison, with the number inside the circle indicating lesion size in millimeters (mm).

## 328 PEDIATRIC VASCULAR COMPRESSION OF THE ESOPHAGUS: ENDOFLIP AS A DIAGNOSTIC ADJUNCT

*Brett Hoskins*, *Paroma Bose*, *Ryan Pitman*

*Division of Pediatric Gastroenterology, Hepatology, and Nutrition, Department of Pediatrics*, *Indiana University School of Medicine*, *Indianapolis*, *IN*

**Background:** Vascular anomalies can cause external esophageal compression, leading to dysphagia or feeding intolerance in children. Diagnosis is based on imaging and endoscopy, though radiographic and visual inspection may under‐ or overestimate functional narrowing. Functional Lumen Imaging Probe (FLIP) offers real‐time assessment of esophageal distensibility and diameter, but its utility in detecting vascular compression has not been well‐described in either pediatric or adult populations.

**Methods:** We retrospectively reviewed pediatric patients with known vascular anomalies who underwent upper endoscopy with EndoFLIP between 7/2021 and 4/2025. Vascular anatomy, imaging, EndoFLIP findings, and treatment were compared. In two cases, measurements at the lower esophageal sphincter (LES) and compression site were compared at matched inflation volumes.

**Results:** Eight pediatric patients (mean 12.1 years, range 6–17; 5 female, 3 male) with known vascular anomalies underwent upper endoscopy with EndoFLIP evaluation for dysphagia. The most common anomaly was left aortic arch with aberrant right subclavian artery (n=5, one post‐reconstruction); others included double aortic arch (n=2, one post‐reconstruction) and right aortic arch with anomalous left subclavian artery (n=1). EndoFLIP detected esophageal narrowing in 6 of 8 patients (75%), compared to 4 of 8 (50%) on visual endoscopy and 6 of 7 (86%) on upper GI (UGI) contrast studies. Two patients had narrowing detected by EndoFLIP not appreciated on EGD, while one patient had narrowing evident on UGI but not on EndoFLIP. Four patients had concordant findings across all modalities (either all positive or negative for compression).

Esophageal contractility was normal in 7 patients (88%), with one patient showing diminished contractility of unclear etiology. Mucosal biopsies were normal in 7 patients, with one case of eosinophilic esophagitis (up 48 eosinophils per high‐power field). No procedural complications occurred. In two patients, EndoFLIP measurements at 30 mL balloon inflation were compared between the LES and the site of vascular compression. Patient 3 showed a marked reduction in both diameter (14.0 mm to 4.8 mm) and distensibility index (DI; 9.0 to 1.2 mm2/mmHg) at the compression site. Patient 4 showed similar mechanical restriction, with diameter decreasing from 20.0 mm to 10.3 mm and DI from 6.0 to 2.44 mm2/mmHg.

Treatment included surgical repair (n=3), dietary modifications alone (n=2), and proton pump inhibitor (PPI) therapy (n=3). Surgery led to resolution of dysphagia in one patient and partial improvement in another, with one patient awaiting intervention. Dietary modifications alone resulted in partial improvement in both cases. PPI therapy led to symptom resolution in all three patients, including two without significant anatomic narrowing on endoscopy or EndoFLIP, and one with eosinophilic esophagitis.

**Conclusions:** EndoFLIP appears to be safe and well‐tolerated in pediatric patients with vascular anomalies involving the esophagus. In this small cohort, EndoFLIP identified esophageal narrowing in cases both apparent and not apparent on endoscopy, suggesting added diagnostic value. The ability to quantify regional changes in distensibility and diameter may enhance characterization of extrinsic esophageal compression. These findings support the potential role of EndoFLIP as a complementary tool in the evaluation of children with suspected vascular compression. Larger, prospective studies are needed to validate these findings and define the clinical utility of EndoFLIP in this population.


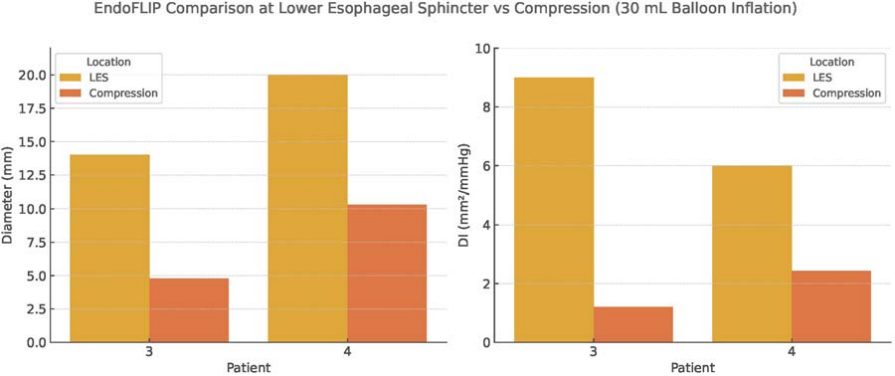


## 329* POST‐ERCP PANCREATITIS PROPHYLAXIS: EFFECTIVENESS OF RECTAL INDOMETHACIN VS INTRAVENOUS KETOROLAC IN THE PEDIATRIC POPULATION

*Sherif Ibrahim* ^*2,1*^, *Ethan Estes* ^*2*^, *Lindsey Hornung* ^*3*^, *Tyler Thompson* ^*2*^, *Tom Lin* ^*4*^, *Lin Fei* ^*3*^, *Maisam Abu‐El‐Haija* ^*2*^, *David Vitale* ^*1*^

^*1*^ *Division of Pediatric Gastroenterology*, *Medical University of South Carolina*, *Charleston*, *SC*; ^*2*^ *Division of Pediatric Gastroenterology*, *Cincinnati Children's Hospital Medical Center*, *Cincinnati*, *OH*; ^*3*^ *Division of Biostatistics and Epidemiology*, *Cincinnati Children's Hospital Medical Center*, *Cincinnati*, *OH*; ^*4*^ *Division of Pediatric Gastroenterology*, *Rady Children's Hospital‐San Diego*, *San Diego*, *CA*

**Background:** Endoscopic retrograde cholangiopancreatography (ERCP) is an essential interventional procedure but can be complicated by post‐ERCP pancreatitis (PEP). Rectal indomethacin has been shown to reduce rates of PEP in adult populations and a recent retrospective study showed reduced rates of PEP in pediatric patients receiving intravenous (IV) ketorolac. There have been no prospective trials evaluating medications for PEP prevention in pediatric patients. Both rectal indomethacin and IV ketorolac are currently standards of care, with IV ketorolac allowing more weight specific dosing for pediatric patients. This prospective randomized trial aims to compare effectiveness of therapy with rectal indomethacin versus IV ketorolac in PEP prevention.

**Methods:** This study conducted at a single, large pediatric tertiary care center. Patients (age 6 months to 21 years) at high risk for PEP were randomly assigned to receive rectal indomethacin (>=50 kg, 100 mg; 30‐49 kg, 50 mg; 10‐29 kg, 25 mg) or IV ketorolac 0.5 mg/kg (maximum dose: 15 mg). For this study, high risk patients included those with a native papilla, performance of a pancreatic sphincterotomy, or pancreatic duct injection with contrast. Patients were masked to group assignment, but clinicians were aware of group assignment to ensure medication administration. Primary outcome was rate of PEP with secondary endpoints including but not limited to pain, laboratory markers, and length of stay. Post‐procedurally, patients were admitted and started on D5 Lactated Ringers (D5LR) at 1.5 maintenance rate as per a standard order set (maximum rate 150 mL/hr) and a clear liquid diet to be advanced as tolerated. A minimum of 3 pain assessments were completed by nursing staff/anesthesia during admission in the post operative unit, the evening of admission, and the next morning. If the patient experienced pain worse than on admission based on scoring systems (Numeric Rating Scale (NRS) or Face, Legs, Activity, Cry, Consolability (FLACC)) or nausea this triggered a best practice order set for the primary provider to obtain a standard set of lab work assessing for pancreatitis. The lab work included: CRP, CBC, Amylase, and Lipase. If patient met criteria for PEP, they remained on D5LR. PEP was considered within a 2‐week period post procedure. This clinical trial is complete and registered with ClinicalTrials.gov (NCT05664074).

**Results:** A total of 193 cases were completed on 100 unique patients. Rates of pancreatic stent placement, both prophylactic and therapeutic, were similar in both groups. PEP rate for the Ketorolac group was higher than the indomethacin group but not statistically significant (22% vs 16%, p= 0.28). For those undergoing pancreatic sphincterotomy, the odds of developing PEP were significantly higher at 3.9 (95% CL: 1.1‐13.7) times higher for those given ketorolac compared to those given rectal indomethacin (p=0.03). In patients with a native papilla, though not significantly different, the odds of developing post‐ERCP pancreatitis were 1.8 (95% CL: 0.7‐4.8) times higher for those given ketorolac compared to those given rectal indomethacin (p=0.24). A number needed to treat analysis showed treating 17 patients with ketorolac ($3.75/15 mg vial) will result in 1 more pancreatitis case than use of indomethacin ($361.91/50 mg suppository), costing $6,008.72 per additional case of pancreatitis avoided by using indomethacin over ketorolac.

**Discussion:** This was a randomized, single‐blinded clinical trial in pediatric patients undergoing ERCP. Overall, this study shows a potential advantageous role for prevention of PEP of rectal indomethacin over ketorolac in pediatric patients, specifically those in high‐risk subgroups.


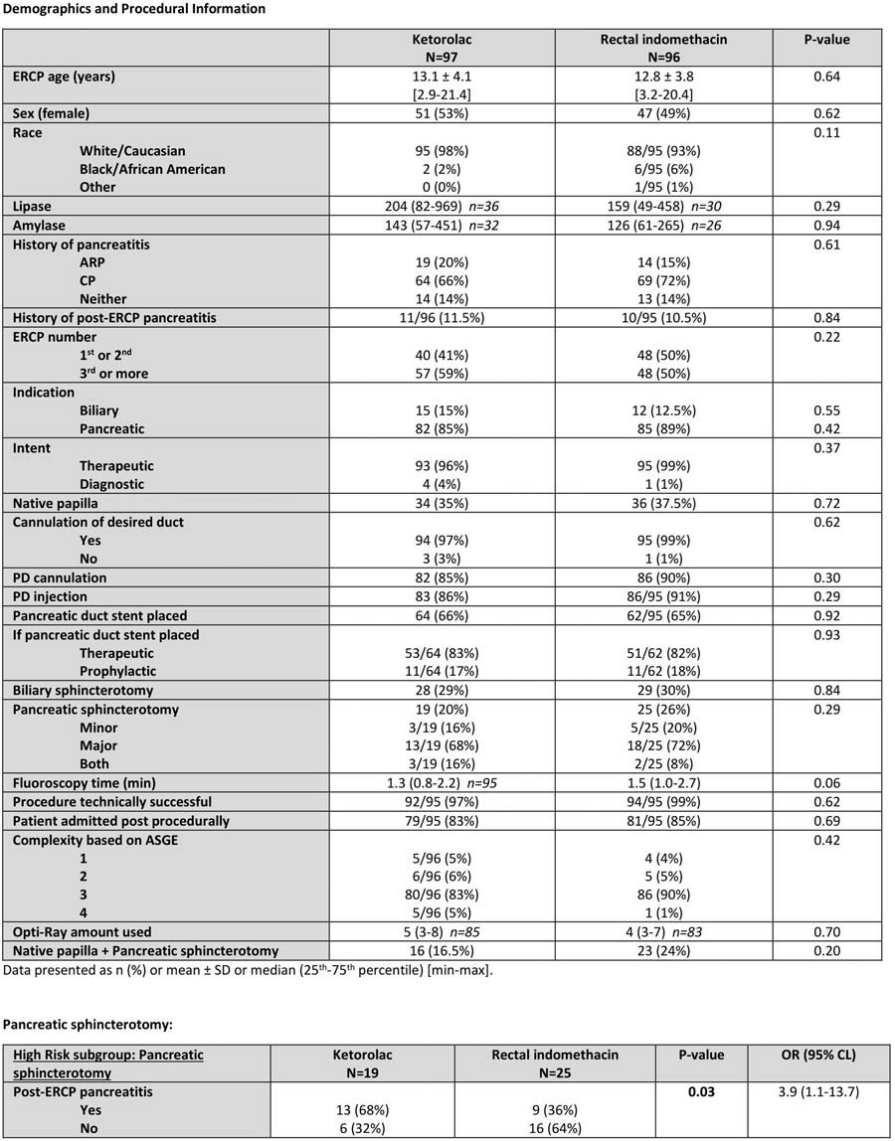


Demographics and High Risk Subgroup Analysis

## 333 COLLAGENOUS GASTRITIS IN PEDIATRICS: A CASE SERIES OF A RARE DISEASE

*Bertrand Leduc* ^*1*^, *Julie Castilloux* ^*2*^, *Isabelle Harvey* ^*3*^, *Anna Wieckowska* ^*2*^, *Guillermo Costaguta* ^*2*^

^*1*^ *Pediatrics*, *Centre Hospitalier de l'Universite Laval*, *Quebec*, *QC*, *Canada*; ^*2*^ *Pediatric Gastroenterology*, *Centre Hospitalier de l'Universite Laval*, *Quebec*, *QC*, *Canada*; ^*3*^ *Anatomopathology*, *Centre Hospitalier de l'Universite Laval*, *Quebec*, *QC*, *Canada*

**Background:** Collagenous gastritis (CG) is a rare gastrointestinal disease, the pathogenesis and optimal treatment of which have yet to be established. Histologically, CG is characterized by gastric subepithelial collagen deposition and chronic inflammation. Pediatric CG is particularly understudied, and the available literature is scarce. We report 12 pediatric cases from our tertiary care center in Québec, Canada.

**Objectives:** The aim of the study was to describe the clinical, endoscopic and histologic presentation, as well as the treatment outcomes of pediatric patients with CG at Centre hospitalier de l'Université Laval (CHUL).

**Methods:** We conducted a retrospective review of pediatric patients diagnosed with CG at our tertiary care center between 2014 and 2024. Patients aged 0–18 years with a confirmed histological diagnosis of CG were included. We excluded patients who were positive for Helicobacter pylori (H. pylori), who had hypertrophic gastritis on immunohistochemistry, or who were diagnosed with Crohn's disease. Demographic, clinical, endoscopic and histologic data, as well as treatments and responses, were reviewed.

**Results:** Twelve patients (ages 9‐17 years at first presentation) were included in the study, of whom ten were female (83%). On upper endoscopy, all patients (100%) had nodular gastritis (all negative for *H. pylori* infections) with eosinophilic infiltration and subepithelial collagen bands on histology consistent with CG. Most patients (8/12) had at least one gastrointestinal symptom, namely abdominal pain, decreased appetite, weight loss, pyrosis, nausea, or vomiting. One patient presented with two episodes of spontaneous gastric ulcer perforation and underwent gastrorrhaphy with epiploonoplasty, followed by a partial gastrectomy two years later. Ten patients (83%) presented with symptomatic iron‐deficiency anemia, of whom six patients (50%) presented with severe‐to‐moderate iron‐deficiency anemia (hemoglobin 26‐85 g/L), with three of them (25%) requiring blood transfusions. Eight patients (67%) achieved normalization of hemoglobin levels with oral iron supplementation alone. Eight patients (67%) were prescribed proton pump inhibitors (PPIs), while four patients (25%) were additionally treated with oral budesonide. The patient with the most severe iron‐deficiency anemia (hemoglobin 26 g/L), who required multiple blood transfusions, oral iron supplementation, oral budesonide, and PPI therapy, demonstrated resolution of CG on repeat endoscopy three years later. At the last follow‐up visit, seven patients (58.3%) had complete resolution of symptoms, although 11 patients (91.7%) continued to exhibit subepithelial collagen bands on the most recent endoscopic assessment.

**Discussion:** This study adds a new cohort of 12 pediatric patients with CG to the paucity of data in the literature. Symptomatic iron‐deficiency anemia was the most common presentation in our patients. Our treatment approach was consistent with the current body of knowledge on pediatric CG, including the use of oral and intravenous iron to address anemia, PPIs and topical budesonide. However, a proven therapeutic regimen for CG has not yet been established. The majority of the patients included in our cohort had complete resolution of symptoms at their last follow‐up visits despite the fact that almost all of them had persistence of subepithelial collagen bands, suggesting that histologic findings are not necessarily associated with clinical outcomes. Although CG is described in the literature as an exceedingly rare disease, the number of cases reported at our center suggests that it is likely to be better recognized nowadays, or that its incidence is on the rise. We are still in the early stages of analyzing the outcomes of these patients, as their long‐term evolution remains uncertain and understudied.

## 334 LEFT LATERAL DECUBITUS POSITION DURING SEDATION‐FREE TRANSNASAL ENDOSCOPY: A PILOT STUDY

*Rose Lee*, *Yonna Oparaugo*, *Molly Mackensen*, *Katherine Vaidy*

*Pediatric Gastroenterology*, *Medical College of Wisconsin*, *Milwaukee*, *WI*

**Background:** Sedation‐free transnasal endoscopy (TNE) is a safe, feasible, and well tolerated procedure performed in children to evaluate the upper gastrointestinal tract. The procedural technique of TNE in children is adopted from procedural standards in adults, typically using the upright seated position. The left lateral (LL) decubitus position may be preferred for optimal safety and visualization during TNE. This pilot study explored the feasibility and tolerance of TNE in pediatric patients using the LL decubitus position.

**Methods:** This was a retrospective review of thirteen children who underwent sedation‐free TNE in the LL decubitus position from October 2024 to February 2025 in an outpatient gastroenterology procedure suite. Procedure time, patient tolerance (TNEase score), adverse events, and patient demographics were collected and analyzed.

**Results:** A total of 13 TNE procedures were successfully completed in the LL decubitus position. The mean (SD) age of the cohort was 12 years (2.7); 38% were female. The mean (SD) procedural time for esophagoscopy was 5.1 minutes (1.6). All patients had TNEase score of 2 or lower. Ten (77%) of patients had a TNEase score of 1. Two patients with history of anxiety and orthostasis experienced syncope in the upright seated position but subsequently completed the TNE in the LL decubitus position without adverse events.

**Conclusions:** LL decubitus position for sedation‐free TNE is feasible and well tolerated in children. Findings should prompt further, prospective investigations of the benefits of LL decubitus vs upright seated position, particularly in children with orthostatic intolerance.

## 338 CAN GASTRIC BRUSHING AND FLUID REPLACE BIOPSY FOR DIAGNOSING HELICOBACTER PYLORI INFECTION IN PEDIATRIC PATIENTS?

*Akash Pandey* ^*1*^, *Devendra Mehta* ^*1*^, *Nishant Patel* ^*1*^, *William Morgan* ^*2*^, *Noah Stoeckel* ^*2*^, *Mary Schreck* ^*3*^, *Chirajyoti Deb* ^*2*^

^*1*^ *Pediatric Gastroenterology, Arnold Palmer Children's Hospital*, *Wintergarden*, *FL*; ^*2*^ *Specialty Diagnostic Clinical and Translational Research Lab*, *Orlando Health Arnold Palmer Hospital for Children*, *Orlando*, *FL*; ^*3*^ *Orlando Health Medical Group Pediatric Specialty Practices*, *Orlando Health Arnold Palmer Hospital for Children*, *Orlando*, *FL*

**Background:** *Helicobacter pylori* (*H. pylori*) is a class I human carcinogen associated with various gastrointestinal disorders in both children and adults. Current diagnostic methods rely primarily on invasive gastric biopsies for detection via culture, rapid urease test (RUT), histopathology, PCR, and fluorescence in situ hybridization (FISH). However, these methods have limitations in sensitivity, specificity, and turnaround time. Noninvasive tests such as stool antigen and urea breath tests are less reliable and not recommended as standalone diagnostic tools. Previous studies have suggested that cytology brushing, and gastric fluid may serve as less invasive alternatives. This study evaluates the diagnostic performance of a novel real‐time PCR assay using gastric brushing and fluid samples compared to biopsy‐based methods in pediatric patients. These methods may help reduce patient discomfort, sedation time, and procedural costs, making them attractive alternatives in pediatric settings.

**Methods:** In this prospective study, 150 pediatric patients (ages 2–21) undergoing esophagogastroduodenoscopy (EGD) for suspected or confirmed *H. pylori* infection were enrolled. Gastric biopsies, brushings (from the antrum and body), and available gastric fluid samples were collected during EGD. Total genomic DNA was extracted using the DNeasy Powersoil Pro Kit (Qiagen), and a novel real‐time PCR assay targeting *H. pylori*‐specific genes was developed and applied. Clinical data, including diagnostic outcomes and symptoms, were collected via chart review. PCR results were compared to clinical diagnoses to assess sensitivity, specificity, predictive values, and statistical concordance.

**Results:** Of the 150 enrolled patients, 138 (mean age 11.9 ± 4.81 years) were included in the final analysis at the time of data analysis for poster submission. The prevalence of *Helicobacter pylori* infection, based on clinical diagnosis, was 15.94%. Among 133 paired brushing and biopsy samples, *H. pylori* was detected in 21 (15.79%) and 19 (14.29%) samples, respectively. Of 71 gastric fluid samples, 13 (18.3%) tested positive. Two cases showed discordance between PCR and clinical diagnosis, both involving positive stool antigen tests but negative biopsy and culture results. One additional case was PCR‐positive despite a negative clinical diagnosis. Overall, there was no statistically significant difference between PCR results and clinical diagnoses across all sample types. The diagnostic performance metrics are presented in Figure 1.

**Conclusion:** This study demonstrates that gastric brushing and fluid samples analyzed via a novel real‐time PCR assay offer diagnostic accuracy comparable to biopsy‐based methods for detecting *H. pylori* in pediatric patients. These less invasive approaches may serve as viable alternatives to biopsy, particularly in settings where endoscopy is not feasible. Further testing, including expanded gene target analysis and next‐generation sequencing, is ongoing to validate these findings and explore microbial community profiles.

**Figure 1. Diagnostic Performance of real‐time PCR for H. pylori detection using three different sample types including gastric brushing, biopsy, and gastric fluid.:**


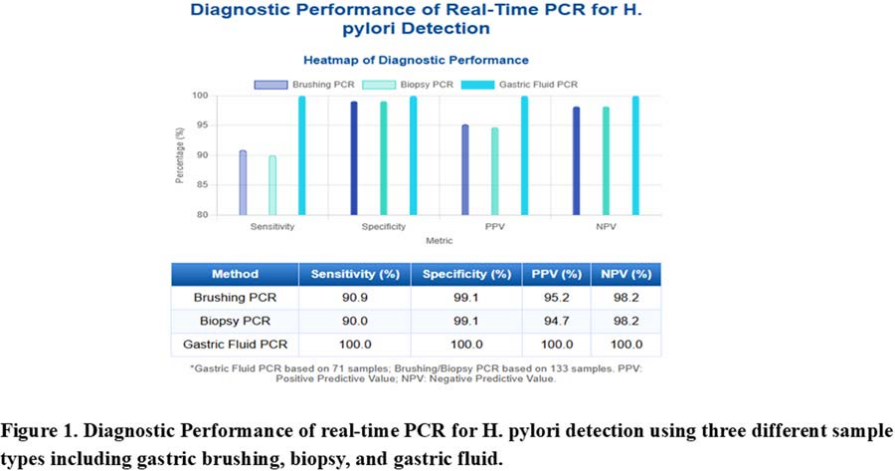


Diagnostic Performance of real‐time PCR for H. pylori detection using three different sample types including gastric brushing, biopsy, and gastric fluid.

## 341 FLUOROSCOPY SAFETY AND UTILIZATION IN PEDIATRIC ENDOSCOPIC PROCEDURES

*Alison Purcell* ^*1,2*^, *Eric Chiou* ^*1,3*^, *Adam Vogel* ^*5,6*^, *Wenly Ruan* ^*4*^, *Sherry Brogan* ^*6*^, *Ernestina Melicoff‐Portillo* ^*6,3*^, *Howard Pryor* ^*5,6*^, *Douglas Fishman* ^*1,5*^

^*1*^ *Division of Gastroenterology, Hepatology, and Nutrition*, *Texas Children's Hospital*, *Houston*, *TX*; ^*2*^ *The University of Texas Health Science Center at Houston John P and Katherine G McGovern Medical School*, *Houston*, *TX*; ^*3*^ *Baylor College of Medicine Department of Pediatrics*, *Houston*, *TX*; ^*4*^ *Unaffiliated*, *Houston*, *TX*; ^*5*^ *Division of Pediatric Surgery*, *Baylor College of Medicine Michael E DeBakey Department of Surgery*, *Houston*, *TX*; ^*6*^ *Department of Pediatrics*, *Texas Children's Hospital*, *Houston*, *TX*

Background and Aims: The utilization of fluoroscopy has significantly widened the scope of pediatric endoscopy. However, fluoroscopy is also associated with ionizing radiation exposure, for which pediatric patients are at increased risk. Training in radiation exposure and protection is not strongly emphasized during endoscopic training. Additionally, there is limited pediatric data on the use of fluoroscopy in general endoscopy procedures such as esophageal dilation. Therefore, the aim of our study was to determine the current utilization and documentation of fluoroscopy in pediatric endoscopy.

**Methods:** We retrospectively reviewed all cases of endoscopy with fluoroscopic guidance at a quaternary children's hospital from November 2019‐December 2023, including cases such as luminal balloon dilation, manometry catheter placement, and endoscopic retrograde cholangiopancreatography (ERCP). Procedure characteristics and quality measures were assessed, including: fluoroscopy dose (mGy) and dose area product (mGym^2^), fluoroscopy time (seconds), continuous vs. pulsed fluoroscopy use, procedure location (eg. GI unit or operating room), presence of a pediatric gastroenterologist, and differences among individual providers.

**Results:** A total of 734 endoscopic procedures with fluoroscopic guidance were identified, of which 41% were balloon dilations, 44% were ERCPs, and 13% were manometry catheter placements with the remaining 2% of cases a variety of lower‐volume procedure types. There were significant differences across the three most common procedure types in fluoroscopy total time, dose, and dose area product (Table 1). Not surprisingly, cases with continuous fluoroscopy had significantly higher times and dosages (p<0.001). There were no differences in fluoroscopy usage comparing pediatric surgeons (n=7) to pediatric gastroenterologists (n=8). For endoscopic dilation procedures, there was significant inter‐provider variation in fluoroscopy dose and time confirmed by ANOVA (p<0.001).

Conclusions: These data provide important insights into the usage of fluoroscopy in pediatric endoscopy. Significant differences in fluoroscopy time and dose between individual endoscopists may represent a future opportunity for fluoroscopy education and training. Limitations of this study include the heterogeneity in case complexity and procedure time. The present data allow us to better understand the current state of fluoroscopy use across pediatric endoscopic procedures and potentially alter current practice to further reduce ionizing radiation exposure to pediatric patients.


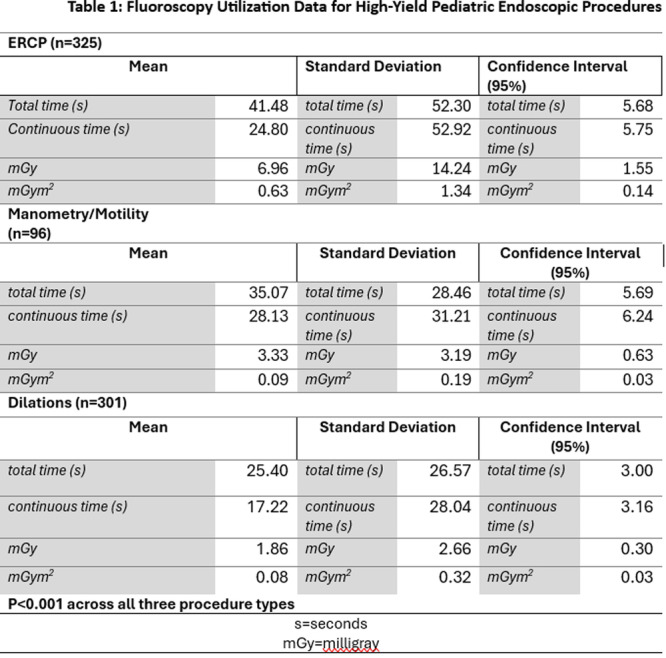


## 346 FIRST ONE HUNDRED CASES OF SEDATION‐FREE ENDOSCOPY USING SINGLE‐USE GASTROSCOPES. A PEDIATRIC SINGLE‐CENTER EXPERIENCE

*Yamen Smadi*, *Jeffrey Bornstein*, *Vijay Mehta*, *khaled bittar*

*Pediatric Gastroenterology*, *Orlando Health Arnold Palmer Hospital for Children*, *Orlando*, *FL*

**Background:** Unsedated transnasal endoscopy (TNE) has emerged as a promising alternative to esophagogastroduodenoscopy (EGD) under sedation. We report our center's experience using single‐use gastroscopes to perform over a hundred sedation‐free endoscopies.

**Methods:** A retrospective review was performed on patients who underwent sedation‐free endoscopy using a the EvoEndo (Grayslake, IL) Model LE, 85 or 110 cm, sterile, single‐use, ultra‐slim 3.5 mm outer diameter, gastroscope. Data including demographics, procedural success rate, total visit time, procedure time, procedural tolerance, and adverse events were collected.

**Results:** Sedation‐free endoscopy was performed in 105 subjects (age range 0.5‐43, mean age 14.6 years). The scope was advanced through the nares or through a gastrostomy site in 101 and 4 subjects respectively. Indications were eosinophilic esophagitis (n=95) celiac disease (n=6), gastrojejunostomy tube placement (n=4), dysphagia (n=2) and others (n=8). Procedure was successful in 98.1% (n=103). The scope was advance successfully to the small bowel through nares in 10 cases and through gastrostomy in 4 cases. The procedure was performed for the first time in 82 subjects (78%) and was a repeated in 23 subjects (22%, range 1‐5, average 1.6 endoscopies). TNEase score as previously reported was reported by endoscopist as 1 (n=94, 89.5%), 2 (n=6, 5.7%) 3 (n=3, 2.8%), 4 (n=0, 0.0%), and 5 (n=2, 1.9%). Average facility visit time was 35 minutes and average procedure duration was 6 minutes. Vomiting (n=5, 4.7%) and near syncope (n=3, 2.8%) were observed as the only adverse events of the procedure.

**Conclusion:** Sedation‐free endoscopy using single‐use ultra‐slim gastroscope is safe and successful in more than 100 cases performed in a single pediatric center and has the potential to improve patient care and efficient access to endoscopy.

**Acknowledgment:** Thank you to Joel Friedlander, MD from EvoEndo

## 349 TRANSNASAL ENDOSCOPY, A RETROSPECTIVE STUDY IN A LARGE PEDIATRIC POPULATION AT CHILDRENS HOSPITAL OF WISCONSIN

*Katherine Vaidy*, *Molly Mackensen*, *Rose Lee*

*Pediatrics*, *Medical College of Wisconsin*, *Milwaukee*, *WI*

**Background:** The aim of the retrospective study was to examine the efficacy and tolerance of transnasal endoscopy (TNE). We obtained study data at a single ambulatory center in a large pediatric patient population (n=120) who have undergone TNE using a disposable ultra‐thin gastroscope. The objectives were to 1) describe the experience of TNE using TNEase scoring 2) describe TNE technique including procedure type, duration, position, preparation, and scope length and 3) analyze success rates and adverse events.

**Methods:** Medical records using Children's of Wisconsin EPIC database included patients who have undergone the TNE procedure from (10/2022 – 04/2025). Deidentified study data incorporated in QHS REDCAP included diagnosis, procedure type, patient positioning, TNEase scoring, duration of procedure/visit, adverse events, and pre‐procedural preparation.

**Results:** One hundred and twenty unique patients enrolled in the study with 164 completed TNEs. We performed transnasal esophagoscopy (TN‐Eso) and esophagogastroscopy (TN‐EG) for eosinophilic esophagitis in the upright position (84%) using a 110 cm (76%) and 85 cm (24%) ultra‐slim gastroscope. We implemented pre‐procedural techniques with the use of lidocaine and oxymetazoline nasal spray, virtual reality goggles, and child life specialist 83‐100% of cases. Ninety three percent of patients had TNEase scoring of 1 or 2. One hundred sixty of 164 procedures obtained tissue biopsies. Eleven percent of procedures (N=18) noted minimal patient adverse side effects, which included syncope (n=5, 27%), near syncope (n=5, 27%), vomiting (n=6, 33%), and post‐procedure abdominal pain (n=2, 11%). Visit time average for ambulatory TNE was 1 hour 26 minutes with an average procedure time of 6 min 36 seconds compared to the sleep endoscopy visit duration of 4 hours 21 mins and procedure time of 26 min.

**Conclusion:** This study shows TNE is an efficient, well tolerated procedure and is the first to evaluate this in a large pediatric population (N=120 patients, N=164 TNE) using an ultra‐slim gastroscope. At our center, TNE is shorter visit and procedure compared to sleep endoscopy for monitoring eosinophilic esophagitis.

## 350 PEDIATRIC GASTRO ENDOSCOPIC ULTRASOUND (PEGASEUS) DATABASE

*David Vitale* ^*1*^, *Jacob Mark* ^*2*^, *Sagar Pathak* ^*3*^, *Alexander Coe* ^*4*^, *Lindsey Hornung* ^*1*^, *Brett Hoskins* ^*5*^, *Quin Liu* ^*6*^, *Garrett Sprague* ^*1*^, *David Troendle* ^*7*^, *Petar Mamula* ^*4*^

^*1*^ *Pediatrics*, *Cincinnati Children's Hospital Medical Center*, *Cincinnati*, *OH*; ^*2*^ *Pediatrics*, *Children's Hospital Colorado*, *Aurora*, *CO*; ^*3*^ *Pediatrics*, *Rady Children's Hospital‐San Diego*, *San Diego*, *CA*; ^*4*^ *Pediatrics*, *The Children's Hospital of Philadelphia*, *Philadelphia*, *PA*; ^*5*^ *Pediatrics*, *Riley Hospital for Children at Indiana University Health*, *Indianapolis*, *IN*; ^*6*^ *Cedars‐Sinai Medical Center*, *Los Angeles*, *CA*; ^*7*^ *Pediatrics*, *The University of Texas Southwestern Medical Center*, *Dallas*, *TX*

**Introduction:** Pediatric interventional endoscopy is being increasingly utilized in pediatric centers with a notable rise in indications for endoscopic ultrasound (EUS) procedures in children. Nonetheless, these procedures are much less utilized in children as compared to adults. Prior investigations of EUS in children are single center, small and retrospective. The study aims to establish and maintain a multi‐institutional pediatric EUS registry to further evaluate indications, safety, efficacy and outcomes for of EUS procedures in children.

**Methods:** This study is a multicenter prospective cohort study with a component of retrospective chart review. Patients ≤21 years of age undergoing EUS at participating institutions during enrolled study periods were included. The PEGASEUS database was initially developed in January 2020 with central data collection at a database at Cincinnati Children's Hospital. When available, participating centers also collected retrospective procedure data for up to 5 years prior to joining the database. Demographic, procedural and adverse event data were collected.

**Results:** A total of 832 procedures in 714 unique patients were collected (Table 1) at 5 centers, with 52% females and median age of 14.7 years (IQR: 10.5 ‐ 17.3). The most frequent indications were suspected pancreatic or biliary origin pain (156, 19%), elevated liver enzymes (124, 15%) recurrent pancreatitis (125, 15%) or chronic pancreatitis (125, 15%). Most procedures (729, 88%) were performed by a pediatric gastroenterologist alone. Interventions or biopsies were performed in 355 procedures (Table 2). Two week follow up for adverse events was obtained in 756 procedures (92%), with 47 of 804 procedures documented (5.9%) experiencing any adverse event. There were 16 adverse events (2.1%) ‘definitely’ or ‘probably’ related to the EUS procedure, including 5 instances of pain, 5 infections of cyst or lesion, 4 episodes of bleeding, 1 perforation and 1 fever.

**Conclusion:** This initial report from the PEGASEUS database identified 832 successful EUS procedures performed in children, primarily by pediatric gastroenterologists, with a low adverse event rate. Data collection is ongoing and will continue to examine the safety and efficacy for all types of EUS procedures in pediatric patients.


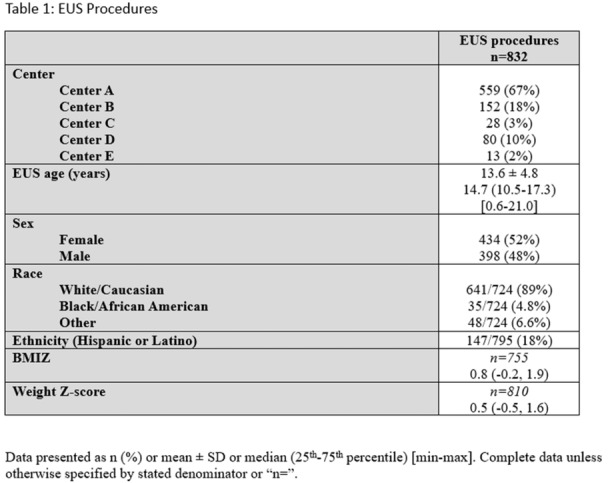



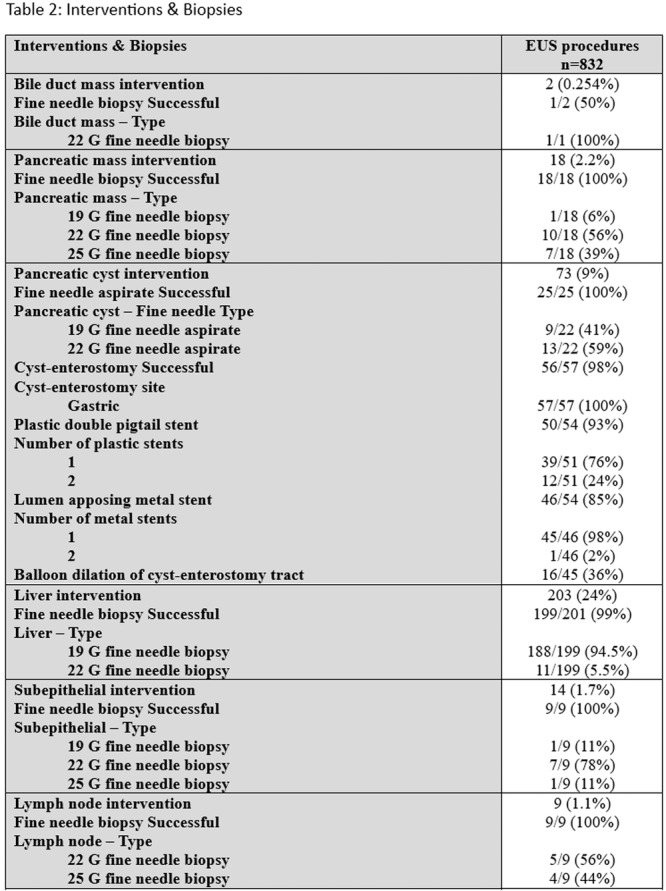


## 353 PREDICTORS OF PERSISTENCE OF SYMPTOMS IN PEDIATRIC PATIENTS WITH EOSINOPHILIC ESOPHAGITIS AFTER HISTOLOGIC REMISSION

*Rasha Abi Radi Abou Jaoudeh* ^*1*^, *sophia Patel* ^*2*^, *Claire Beveridge* ^*3*^

^*1*^ *Pediatric and Adolescent Medicine*, *Cleveland Clinic*, *Cleveland*, *OH*; ^*2*^ *Pediatric Gastroenterology Hepatology and Nutrition*, *Cleveland Clinic*, *Cleveland*, *OH*; ^*3*^ *Gastroenterology Hepatology and Nutrition*, *Cleveland Clinic*, *Cleveland*, *OH*

**Background:** Eosinophilic Esophagitis (EoE) is a chronic inflammatory disorder of the esophagus, frequently presenting with dysphagia, chest pain, and heartburn. Symptom persistence despite histologic remission remains a common challenge, with limited data on contributing factors, particularly in pediatric populations. This study aims to identify factors associated with the persistence of esophageal symptoms in patients with EoE following histologic remission.

**Methods:** We performed a retrospective cohort study of pediatric patients with EoE in histologic remission (HR) (<15 eosinophils per high power field [eos/hpf]). Data regarding patient demographics, disease history, endoscopy reports, EoE endoscopic reference score, and histology were collected. Univariate logistic regression analysis was performed to assess the association between each risk factor and outcomes. Multivariable logistic regression analysis was performed to assess the association between multiple risk factors and outcomes.

**Results:** Among 55 patients with EoE in HR (mean age: 11.5 ± 6.0 years), 27 (49.1%) presented with dysphagia, 20 (36.3%) with heartburn, and 19 (34.5%) with epigastric pain. Despite achieving HR, 33 (62.3%) experienced persistent symptoms, with epigastric pain (N=19; 34.5%), vomiting (N=15; 27.3%), heartburn (N=10; 18.2%), dysphagia (N=9; 16.4%), and dyspepsia (N=8; 14.5%) being the most common. Multivariate analysis revealed that males had a lower risk of persistent epigastric pain compared to females (OR 0.14; CI 0.02–0.58, p=0.0055), while functional disorders were strongly associated with persistent epigastric pain (OR 12.0; CI 2.2–100.7, p=0.0037). On univariate analysis, functional disorders were linked to increased risk of persistent heartburn (OR 5.3; CI 1.1–25.6, p=0.0255), though this was not confirmed on multivariate analysis. Anxiety was associated with increased risk of persistent dyspepsia in both univariate (OR 25.9; CI 2.8–235.8, p=0.0002) and multivariate (OR 23.1; CI 3.3–475.3, p=0.0008) analyses, while depression was only associated with dyspepsia on univariate analysis (OR 11.1; CI 1.9–63.6, p=0.0020). No variables were significantly associated with persistent dysphagia in either univariate or multivariate analysis.

**Conclusion:** This study shows a high prevalence of persistent symptoms in pediatric patients with EoE who are in histologic remission. Risk factors include anxiety, depression, and disorders of gut‐brain axis. These findings can help direct patient care, specifically by highlighting the need for better management of comorbid psychiatric diseases.

## 354 EPITHELIAL EOSINOPHIL‐DERIVED NEUROTOXIN PREDICTS HISTOLOGICAL FINDINGS IN EOSINOPHILIC ESOPHAGITIS ESPECIALLY LAMINA PROPRIA FIBROSIS

*Sharef Al‐Mulaabed*, *Justin Baba*, *Ahmad Abu Sulb*, *Lubna Rahman*, *Yamen Smadi*

*Center for Digestive Health and Nutrition*, *Orlando Health Arnold Palmer Hospital for Children*, *Orlando*, *FL*

**BACKGROUND and AIM:** Eosinophilic esophagitis (EoE) is characterized histologically by eosinophil‐predominate inflammation, typically >15 per high‐power field. Other histological findings (HF) to evaluate the severity and the extent of EoE include eosinophil microabscess, eosinophil surface layering, eosinophilic granules, basal zone hyperplasia, spongiosis, and lamina propria fibrosis. Esophageal epithelial eosinophil‐derived neurotoxin (EDN) obtained from esophageal brushing samples is a measure of disease activity with level ≥ 10 mcg/mL is highly sensitive (97%) and specific (89%) for EoE diagnosis. We aim to examine the correlation between EDN and HF in patients with active EoE.

**METHODS:** A retrospective analysis of patients with EoE who underwent endoscopy from Apr 2018 to Oct 2024 at Arnold Palmer Hospital for Children, Orlando, FL. EoE histology is routinely scored based on the above HF in our institution. Patients who had incomplete evaluation of the histopathology findings were excluded.

**RESULTS:** We included 317 patients with confirmed EoE, mean (SD) age of 10.2 (4.9) years, 257 (81%) males, who underwent 489 upper endoscopies with esophageal biopsies and EDN measured at the same time. Peak eosinophilic count median (IQR) was 50 (30‐61), and EDN median (IQR) was 79 ug/ml (24.7‐160.5).

The most prevalent HF was spongiosis and basal cell hyperplasia (76% and 74%, respectively), while the least prevalent feature was lamina propria fibrosis in 135 (31%) out of 435 specimens that contained submucosa. At least one HF was present in 430 (88%), while all six features were only present in 87 (18%). EDN was elevated in 464 out of the 489 (94.9%) of the subjects.

Correlation between EDN and number of abnormal HF was significant with Spearman's coefficient (rho= 0.309), p <0.001. In addition, comparison of EDN levels between groups with different HF revealed significantly higher level in those with microabscesses, superficial layering, eosinophilic granules, as well as lamina propria fibrosis (p<0.001). On the other hand, EDN was not different between patients with or without basal cell hyperplasia or spongiosis (p>0.05).

Area under the curve analysis for EDN and lamina propria fibrosis revealed a result of 0.728 (standard error of 0.029), which was statistically significant (p<0.001), indicating that EDN performs moderately well in distinguishing presence of lamina propria fibrosis. An EDN cut‐off value of 149.6 resulted in a sensitivity of 43% and specificity of 85% in predicting lamina propria fibrosis.

**CONCLUSIONS:** This study confirms the utility of epithelial EDN in the diagnosis of EoE, with significant correlation between EDN levels and multiple histopathology features of EoE. Lamina propria fibrosis which is not commonly detected due to inadequate sampling may be predicted by a higher EDN level which is easily obtained through esophageal brushing.


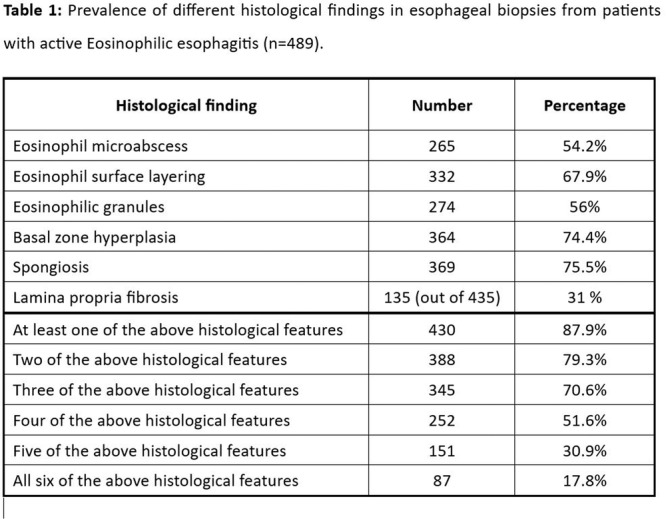



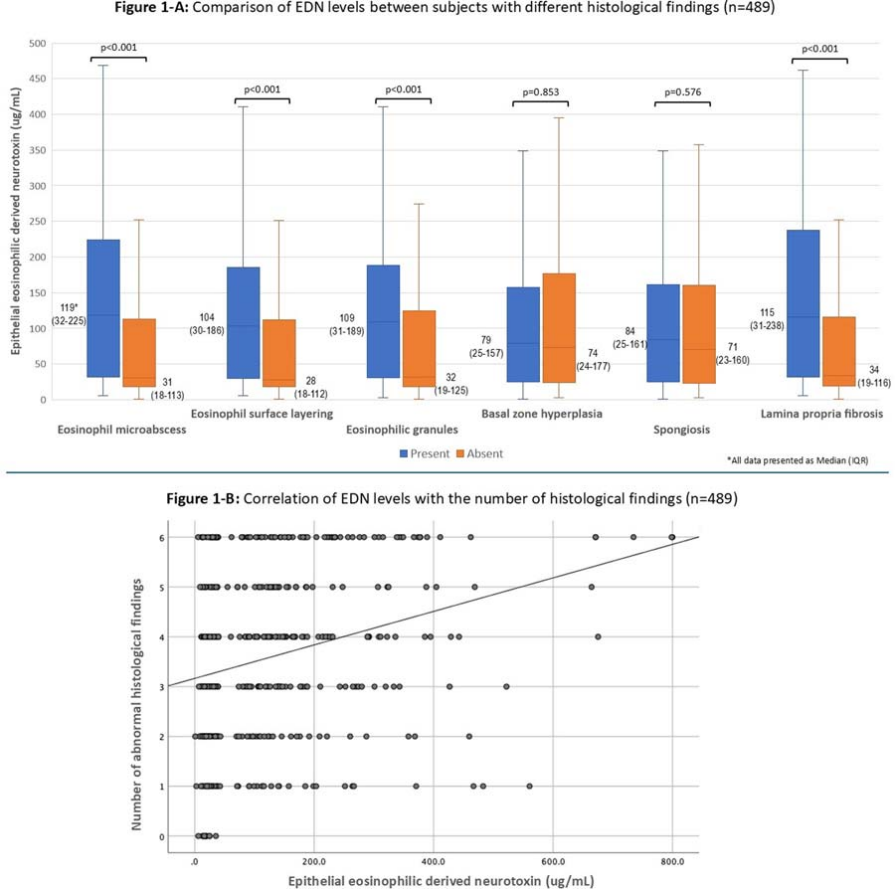


## 357 EOSINOPHIL DEGRANULATION PATTERNS ON TRANSMISSION ELECTRON MICROSCOPY IN EOSINOPHILIC ESOPHAGITIS IN PEDIATRIC PATIENTS TREATED WITH PROTON PUMP INHIBITORS

*Jenna Berson* ^*1*^, *Reid Wilkins* ^*2*^, *Kristen Thomas* ^*2*^, *Melanie Greifer* ^*1*^, *Jason Yin* ^*3*^, *Xiangxi Liang* ^*3*^, *Feng‐Xia Liang* ^*3*^, *Jeremiah Levine* ^*1*^

^*1*^ *Pediatric Gastroenterology*, *NYU Grossman School of Medicine*, *New York*, *NY*; ^*2*^ *Pathology*, *New York University Grossman School of Medicine*, *New York*, *NY*; ^*3*^ *Microscopy Laboratory*, *New York University Grossman School of Medicine*, *New York*, *NY*

**Introduction:** Eosinophilic esophagitis (EoE) is a clinicopathologic condition with increasing prevalence worldwide. It is a chronic, immune/antigen mediated esophageal disease in which there is activation of a Th2 immune response resulting in the production of Th2 cytokines such as IL‐13 and IL‐4. These cytokines then stimulate the esophagus to express eotaxin‐3, a potent eosinophil chemoattractant which leads to eosinophil activation and release of intracellular granules. Proton pump inhibitors (PPI) have been shown to block Th2 cytokine‐induced expression of eotaxin‐3. Transmission electron microscopy (TEM) is the only technique that can clearly identify and distinguish between different modes of cell secretion and is beneficial in evaluating the activation states of eosinophils in esophagitis.

**Objective:** The primary aim of our study was to evaluate eosinophil activation via TEM in patients with EoE vs controls. Our secondary aim was to evaluate the difference in eosinophil activation via TEM in patients with EoE treated with PPI monotherapy with our overall hypothesis being that TEM is an adequate way to monitor activation of eosinophils and that patients with EoE treated with PPI monotherapy should demonstrate stabilization of eosinophils.

**Methods:** After approval from the Institutional Review Board of New York University (NYU) School of Medicine, New York, NY, USA, we performed a prospective cohort study in patients ranging from 0‐18 years who were undergoing an esophagogastroduodenoscopy (EGD) for suspected EoE. Standard of care distal and proximal esophageal biopsies were obtained with a small fragment of tissue placed in TEM fixative. Patients ultimately diagnosed with EoE and placed on PPI monotherapy were followed and repeat biopsies (standard and TEM) were collected for analysis. Eosinophil activation was measured via TEM by analyzing and characterizing the degree and pattern of eosinophil degranulation. Results were compared by unpaired t‐test with follow‐up data compared with paired t‐test with significance assigned by P value < 0.05.

**Results:** Thirty‐four patients were enrolled in the study; 15 had EoE (53% male, average age 7 yo) and 19 were controls (68% male, average age 10 yo). Among the EoE patients, 10 were on PPI monotherapy. The average number of eosinophils per high‐powered field (eos/hpf) were significantly increased in the EoE cohort compared to controls (p < 0.0001). Using TEM, the average percent of activated eosinophils was significantly increased in the EoE cohort compared to controls (distal esophagus 26.99% vs 11.56% in controls, proximal esophagus 25.18% vs 12.54% in controls; p < 0.001). The total number of activated eosinophils (eos/hpf X percent activated eosinophils) was significantly increased in the EoE cohort versus controls (p = <0.0001) (Figure). Within our EoE cohort, the average number of eos/hpf during initial EGD was 54.0 ± 7.6 vs 21.5 ± 10.9 on follow‐up EGD after being on PPI monotherapy (p = 0.12). The total number of activated eosinophils during initial EGD was 1493.0 ± 221.9 vs 430.4 ± 201.6 on follow‐up EGD after being on PPI monotherapy (p < 0.05) (Table).

**Conclusion:** Our EoE cohort had significantly more eosinophils per high‐powered field than our control cohort and their eosinophils were significantly more activated. Although there was not a significant decrease in number of eos/hpf within our EoE cohort on PPI monotherapy, there was a statistically significant difference between the percent of activated eosinophils at initial EGD vs follow‐up EGD. We conclude that TEM is an adequate method to monitor activation of eosinophils and that PPI therapy leads to stabilization of eosinophils.


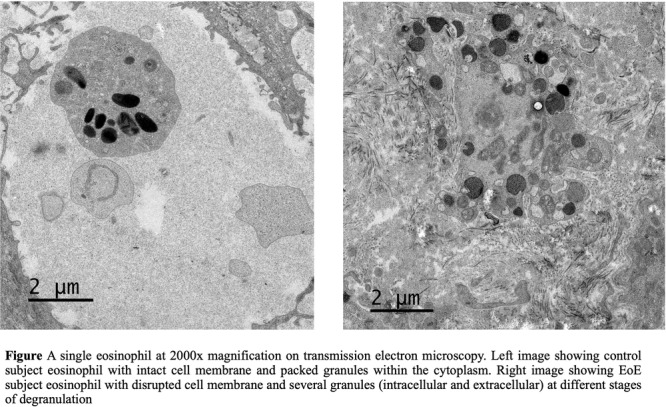



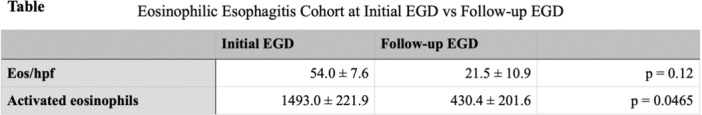


## 358 EVALUATING THE IMPACT OF SWALLOWED TOPICAL STEROIDS ON GROWTH IN CHILDREN WITH EOSINOPHILIC ESOPHAGITIS

*Serena Haver* ^*1*^, *Erica Yi* ^*1*^, *Rakshana Selvarajan* ^*1*^, *Dina Nour* ^*1*^, *Evangelos Katsanos* ^*3,1*^, *Nidhi Talasani* ^*1*^, *Diana Jo* ^*4,1*^, *Victoria Kim* ^*1*^, *Alaa Abdelghani* ^*2*^, *Yves‐Smith Benjamin* ^*2*^, *Michael Sheridan* ^*2*^, *Otto Louis‐Jacques* ^*1,2*^

^*1*^ *Pediatrics*, *Inova Fairfax Hospital*, *Falls Church*, *VA*; ^*2*^ *Pediatric Specialists of Virginia*, *Fairfax*, *VA*; ^*3*^ *University of California Los Angeles*, *Los Angeles*, *CA*; ^*4*^ *Children's National Hospital*, *Washington*

**Background:** Swallowed topical corticosteroids (STCs), such as fluticasone and budesonide, are standard treatments for eosinophilic esophagitis (EoE). Inhaled corticosteroids have been linked to reduced linear growth in children with asthma, but long‐term data on STCs and growth in pediatric EoE are limited.

**Objective:** To assess the long‐term impact of STCs on anthropometric outcomes in children with EoE.

**Methods:** This single center retrospective cohort study included patients diagnosed with EoE between the ages 8–18 years and treated with STCs for ≥2 months at a pediatric multispecialty practice from 2012–2024. Patients with comorbidities known to affect growth such as growth hormone deficiency were excluded. Anthropometric data from steroid initiation (or initial visit if already on steroids at first presentation to the clinic) were compared with data from the last endoscopy before transfer of care, age 20 years, or loss to follow‐up. Z‐scores were calculated in RStudio Version 2024.12.1+563 using the CDCAnthro package based on the 2000 CDC growth charts. Primary outcomes were changes in weight‐for‐age z‐score (WAZ), height‐for‐age z‐score (HAZ), and body mass index z‐score (BMIz). Wilcoxon signed‐rank tests with bootstrapped confidence intervals were used for analysis.

**Results:** In an analysis of 384 participants who were enrolled in the study, 57 met the inclusion criteria and had sufficient data available. The population consisted of 41 males and 16 females, the majority of which were White (66%). Change in WAZ (CI: ‐0.09 to 0.12), HAZ (CI: ‐0.077, 0.10), and BMIz (CI: ‐0.14, 0.08) showed no statistically significant difference in z‐score before and after STC use. The average weight gain on steroids was 0.23 kg/month and average height gain was 0.29 cm/month.

**Conclusion:** STC use in pediatric EoE was not associated with significant changes in weight‐for‐age, height‐for‐age, or body mass index‐z‐score. Further analysis will assess the effect of STCs on peak eosinophil count at each endoscopy as well as the impact of exclusion diets and proton‐pump‐inhibitors on growth.

Declaration of Generative AI and AI‐assisted technologies in the writing process:

During the preparation of this work the author(s) used ChatGPT for editing. After using this tool/service, the author(s) reviewed and edited the content as needed and take(s) full responsibility for the content of the publication.

## 359 RETHINKING FOOD TRIGGERS: A NOVEL ELIMINATION METHOD IN PEDIATRIC EOSINOPHILIC ESOPHAGITIS

*Charles Kang* ^*2,1*^, *Nathalia Albarracin* ^*1,2*^, *Rachel Silverman* ^*1*^, *Jamie Lombardo* ^*3*^, *Danielle Barnes* ^*1,2*^

^*1*^ *Pediatrics*, *Walter Reed National Military Medical Center*, *Bethesda*, *MD*; ^*2*^ *Pediatrics*, *Uniformed Services University of the Health Sciences*, *Bethesda*, *MD*; ^*3*^ *Pathology*, *Walter Reed National Military Medical Center*, *Bethesda*, *MD*

**Background:** Eosinophilic esophagitis (EoE) is a chronic, immune‐mediated gastrointestinal disorder characterized by eosinophilic inflammation of the esophagus in response to dietary allergens exposures in susceptible children and adults. The available treatments for EoE include medications and trial‐and‐error‐based dietary elimination with population level data showing that dairy, eggs, wheat, soy, nuts, and seafood are the most common triggers. No reliable method currently exists for prospectively individualizing elimination diets; therefore, the foods eliminated are selected empirically.

**Objective:** This study aims to assess the feasibility of a novel approach to patient symptom‐based food‐selection for elimination diets in the treatment of pediatric EoE, through a series of dose‐concentrated food trials.

**Methods:** This IRB‐approved, prospective, non‐blinded pilot study is enrolling pediatric EoE patients aged 2 to 20 years with active disease. Inclusion criteria include: a diagnosis of EoE (≥15 eosinophils per high‐powered field [eos/hpf] on esophageal biopsy and esophageal symptoms). Exclusion criteria include: other gastrointestinal disorders, contraindications for endoscopy, severe comorbidities, and recent steroid use. No power calculation is utilized in this initial pilot, which utilizes a convenience sample of 10 patients.

Eligible subjects are taught to perform the novel approach of self‐identifying their food triggers, called the “Dose‐Concentration Phase”. During this phase, subjects serially increase their dietary intake of the six most common EoE triggers: nuts, seafood, wheat, soy, eggs, and dairy one by one for 5 days, with a 2‐day wash‐out of baseline diet between each. During each of these weeks, subjects increase their intake of one potential EoE trigger while maintaining baseline intake of the other 5 foods. For example, during the first week, subjects double their daily intake of nuts while maintaining other food intake at baseline. Throughout this phase, subjects log their symptoms using a Likert scale for severity during each food trial

After completing the Dose Concentration Phase, subjects review their symptom scores with the research team to identify during which food weeks they experiences increased esophageal symptoms. The identified foods, only, are then excluded from their diet, creating an elimination diet guided by their symptomatology. Subjects then complete 6‐8 weeks of their personalized elimination diet. After which, they undergo endoscopy to evaluate for histologic response.

The primary outcome measure is histologic response categorized as remission (<6 eos/hpf), response (<15 eos/hpf), partial response (>15 eos/hpf with >50% eosinophil count reduction), or non‐response. Histology is reviewed by a subspecialty‐trained gastrointestinal pathologist. Secondary outcomes include symptom and quality of life scores measured using the validated Pediatric Quality of Life Inventory Eosinophilic Esophagitis Module (Peds QL EoE) and endoscopic improvement using the Endoscopic Reference Score (EREFS). The Peds QL EoE questionnaires are administered to subjects and guardians at enrollment, after the Dose‐Concentration Phase, and their elimination diet. EREFS scores are assessed at the pre‐study endoscopy and at the post‐elimination diet endoscopy. Gross and microscopic endoscopy results are shared with subjects and guardians. A single follow‐up is completed 6 months after the endoscopy to assess whether subjects have remained on the identified exclusion diet or changed, therapies, and whether they are experiencing symptoms.

**Discussion:** The patient symptom‐guided dietary exclusion method has potential to fill a gap in the dietary treatment of EoE, should it be shown effective. Completing this initial pilot study will provide an opportunity to identify potential early safety signals, and barriers to the feasibility of rolling out a future, powered efficacy study of the method. If patients can identify their dietary EoE triggers based on symptomatology during dose‐concentration, this approach has the potential to reduce the number of endoscopies and sedation events by foregoing extensive and nutrition‐limiting elimination diets with staged food reintroductions. The approach may also improve adherence by empowering the child and family to self‐identify symptoms and guide dietary treatment in a patient‐centered approach.


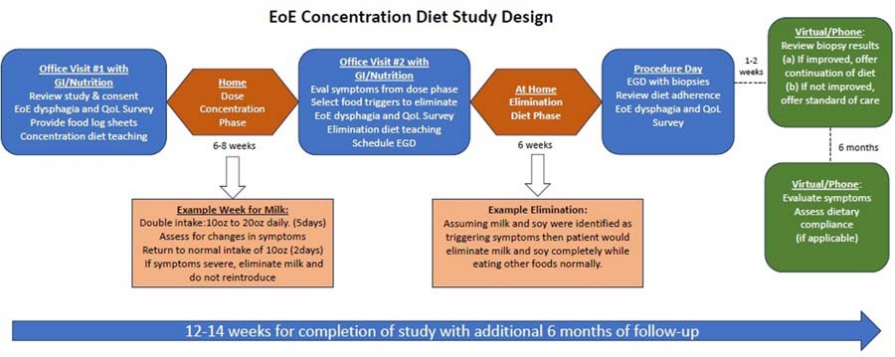


## 361 DISORDERS OF GUT‐BRAIN INTERACTION AS SOURCES OF UNRESOLVED SYMPTOMS IN PEDIATRIC PATIENTS WITH EOSINOPHILIC ESOPHAGITIS

*Olivia Lee* ^*3*^, *Benjamin Stamler* ^*3*^, *Vaidehi Patel* ^*3*^, *Vincent Mukkada* ^*1,2*^, *Scott Bolton* ^*1,2*^, *Neha Santucci* ^*1,2*^

^*1*^ *Gastroenterology, Hepatology and Nutrition*, *Cincinnati Children's Hospital Medical Center*, *Cincinnati*, *OH*; ^*2*^ *Department of Pediatrics*, *University of Cincinnati College of Medicine*, *Cincinnati*, *OH*; ^*3*^ *University of Cincinnati College of Medicine*, *Cincinnati*, *OH*

**Introduction:** Improved understanding of disease pathogenesis and therapies have improved the treatment of eosinophilic esophagitis (EoE) in pediatric patients. However, a subset of patients experience continued or new symptoms after their esophageal biopsies normalize. We hypothesize that these patients have disorders of gut‐brain interaction (DGBI) and attempt to describe them here.

**Methods:** In this single‐center retrospective study, patients with histologically confirmed EoE seen for their initial visit from 12/1/2020 to 8/31/2024 had electronic health records reviewed for demographics, esophageal histology, symptoms, treatments, family history, and comorbidities. Patients were grouped by remission status (<15eos/hpf) and presence of symptoms at the most recent visit. Differences between the suspected DGBI (patients with continued or new onset symptoms after histological remission) and non‐DGBI groups (asymptomatic in remission and active disease with or without symptoms) were analyzed.

**Results:** 351 patients (71% male, 29% female) with EoE, mean age of 12 (range 5‐19) were included. 31% met criteria for a suspected DGBI, 34% were asymptomatic in remission and 33% had active disease. 71% of the patients in the DGBI overlap group reported abdominal pain at their initial visit, compared to only 40% of patients who were asymptomatic in remission (p<0.001). Similarly, 31% of the DGBI group reported constipation at the initial visit compared to only 11% of the remission group (p<0.001) and 19% of the DGBI group reported postprandial distress compared to 7% of those in remission (p=0.017). The most commonly diagnosed DGBI in our study were functional abdominal pain (32%), irritable bowel syndrome (26%), and functional constipation (23%). Mast cell disorders were significantly increased in the DGBI group, with Mast Cell Activation Syndrome present in 3% of DGBI patients vs 0% of remission patients (p=0.023) and Chronic Urticaria seen in 7% of DGBI patients vs 3% of those in remission (p=0.037). Type 3 Ehlers‐Danlos Syndrome/joint hypermobility syndrome (15%, p<0.001), postural orthostatic tachycardia syndrome (7%, p=0.003), dysautonomia (9%, p=0.004) and muscle pain (10.3%, p=0.048) were significantly more common in the DGBI group compared to the control groups. Psychiatric comorbidities (61%, p<0.001), specifically anxiety (50%, p <0.001), depression (20%, p=0.042) and sleep disturbance (21%, p=0.006) were also statistically significant. Increased use of swallowed fluticasone (p=0.031) and famotidine (p<0.001) were found in the suspected DGBI group, but all other standard EoE treatments were used similarly across groups (p>0.05). Treatments often used for functional disorders, such as cyproheptadine (29%, p=0.002), tricyclic antidepressants (10%, p=0.049), neuromodulators (8%, p=0.003), PRN medications such as polyethylene glycol and ondansetron (59%, p<0.001), antispasmodics (25%, p<0.001) and prokinetics (9%, p=0.03).

**Conclusions:** This study noted increased reporting of abdominal pain, constipation, and postprandial distress at the initial visit in patients with continued or new onset symptoms after their EoE is in histologic remission. As these are not typical EoE symptoms, their presence at the first visit suggests they could act as ‘red flag’ symptoms for a DGBI. Of note, our suspected DGBI group is 50% larger than the group of patients with an official diagnosis of a DGBI. Other findings, such as associations with mast cell disorders and psychiatric comorbidities, support known literature about DGBI, supporting the hypothesis that our suspected DGBI patients with continued symptoms after remission do have an untreated functional disorder.

## 362 IMPACT OF SOCIAL DETERMINANTS OF HEALTH ON CLINICAL PRESENTATION AND INITIAL TREATMENT IN PEDIATRIC EOSINOPHILIC ESOPHAGITIS

*Stephanie Leon Paredes* ^*4*^, *Ankona Banerjee* ^*3*^, *Duc Nguyen* ^*3*^, *Savannah Sims* ^*2*^, *Riyad Abdalla* ^*2*^, *Sara Anvari* ^*1*^, *Anthony Olive* ^*4*^, *Eric Chiou* ^*4*^

^*1*^ *Texas Children's Hospital Department of Allergy and Immunology*, *Houston*, *TX*; ^*2*^ *Baylor College of Medicine*, *Houston*, *TX*; ^*3*^ *Pediatrics*, *Baylor College of Medicine*, *Houston*, *TX*; ^*4*^ *Pediatric Gastroenterology*, *Baylor College of Medicine*, *Houston*, *TX*

**Background:** Eosinophilic esophagitis (EoE) is a chronic inflammatory disorder that is characterized by eosinophil‐predominant infiltration to the esophagus. While genetics and pathophysiology are key to understanding EoE, emerging evidence suggests social determinants of health (SDOH) strongly influence other chronic atopic conditions such as asthma. Little is known however about the impact of SDOH on onset, severity and outcomes in EoE. The aim of our study was to explore the relationship between SDOH and the clinical presentation and initial choice of treatment of EoE in children.

**Methods:** We conducted a retrospective cohort study of children aged 0‐18 years diagnosed with EoE at Texas Children's Hospital between January 2014 to October 2024. Patients without esophagogastroduodenoscopy at our institution or whose initial endoscopic biopsy results weren't available were excluded. Demographic details at the time of initial diagnosis of EoE, as well as presenting clinical characteristics including BMI, history of food impaction, history of esophageal dilation, comorbid atopic diagnoses, and peak eosinophil count from esophageal biopsies, and initial treatment data were obtained from the electronic medical record. Assessment of subjects’ SDOH was stratified based on the Child Opportunity Index (COI), a validated multidimensional measure of neighborhood features associated with child health based on patient physical addresses. Logistic regression was used to identify factors associated with higher or lower COI scores.

**Results:** A total of 610 patients were included in the study. Assessment of subjects’ SDOH was stratified based on the COI index and distributed into quintiles as follows: Very Low 18.9%, Low 15.8%, Moderate 17.9%, High 19.9%, Very High 27.6%. For the purpose of our analysis, patients were dichotomized as Lower COI (Very Low/Low, n=211) vs Higher COI (Moderate/High/Very High, n=398). Race/ethnicity and primary language spoken were significantly associated with COI (Table 1), but after multivariable logistic regression EoE patients with Higher COI had a significantly higher rate of other comorbid atopic conditions compared with Lower COI (85.4% vs. 77.7%, p=0.02). Higher COI was also associated with significantly lower median BMI percentile for age at the time of diagnosis compared to Lower COI (47.8%ile vs. 63.3%ile, p=0.01). There was no significant correlation between COI and age of presentation, peak eosinophil count in the esophagus, history of food allergy, history of food impaction requiring endoscopic removal, or history of endoscopic esophageal dilation (Table 2). There was also no significant correlation between COI and choice of initial treatment of EoE: oral medication, dietary elimination, biologic therapy, or combination therapy.

**Conclusions:** Children with EoE and Higher COI were significantly more likely to have concomitant atopic conditions and lower BMI percentile, compared to patients with Lower COI. However, COI was not correlated with age at diagnosis, histology of EoE at diagnosis, or choice of initial EoE treatment. Future studies are warranted to analyze the utilization of healthcare resources, the accessibility to subspecialty services and long‐term clinical outcomes following the diagnosis of EoE.


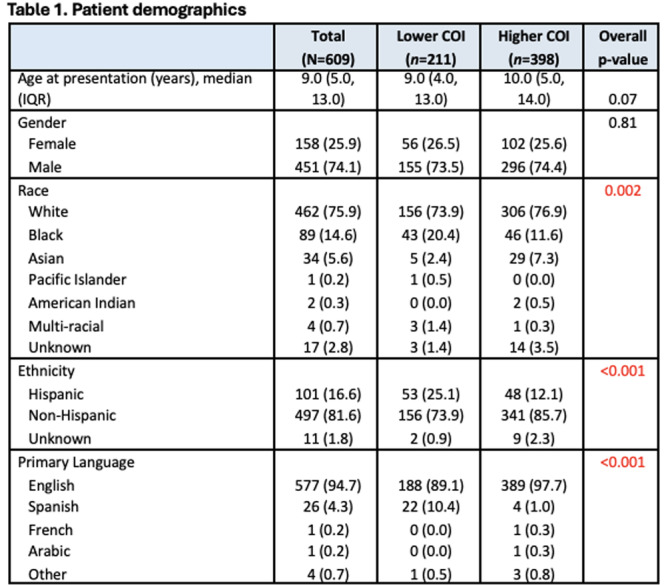



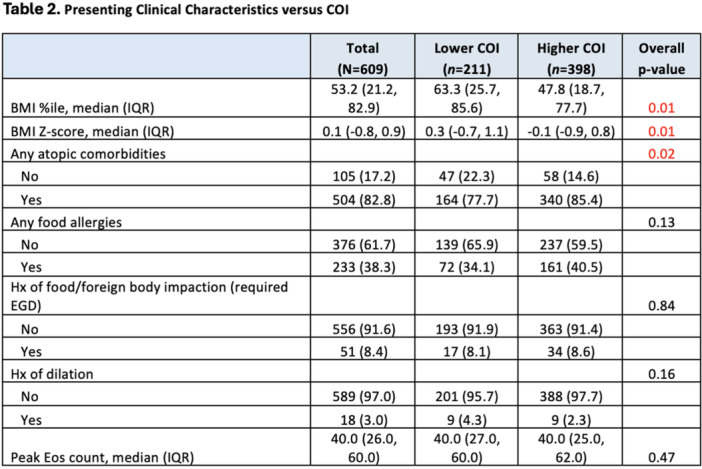


## 363 INCREASED DYSAUTONOMIA IN HYPERMOBILE CHILDREN WITH EOSINOPHILIC ESOPHAGITIS: A DISTINCT PHENOTYPE

*Ritam Patel* ^*2*^, *Jose Peraza* ^*1*^, *Kaitlyn Keeley* ^*1*^, *Zeinab Dehghani* ^*1,2*^, *Natalie Hoffmann* ^*1,2*^, *Michael White* ^*1,2*^, *Joshua Wechsler* ^*1,2*^

^*1*^ *Pediatrics*, *Ann & Robert H. Lurie Children's Hospital of Chicago*, *Chicago*, *IL*; ^*2*^ *Northwestern University Feinberg School of Medicine*, *Chicago*, *IL*

**Background:** The extent to which pediatric Eosinophilic Esophagitis (EoE) patients experience non‐atopic co‐morbidities is poorly understood. Prior research suggests an increased prevalence of hypermobile connective tissue disorders and dysautonomia in EoE. A close association between dysautonomia, hypermobility, and mast cell (MC) activation has been described. While esophageal MCs are increased in EoE, gastric and duodenal MCs have never been quantified. Patients with dysautonomia experience significant gastrointestinal (GI) symptoms, which studies suggest may be explained by increased gastric or duodenal MCs. However, no study has established the relationship between dysautonomia, hypermobility, and MCs in pediatric EoE.

**Methods:** 80 patients aged 10‐21 with EoE undergoing upper endoscopy at Lurie Children's Hospital 1/2021 to 3/2024 were prospectively enrolled. Demographics and peak eosinophil count were collected. Patients/caregivers completed validated surveys to assess symptoms (Pediatric Eosinophilic Esophagitis Symptom Survey [PEESS] and Dyspepsia Symptom Survey [DSS]), quality of life (PedsQL 4.0), and dysautonomia (Composite Autonomic Score [COMPASS‐31]). Hypermobile status was established through a hypermobility survey obtained from a prior publication: International Classification of the Ehlers‐Danlos Syndromes (Malfait et al. 2017) and Beighton scores. Active EoE status was based on at least 15 eosinophils per high power field (EOS/HPF). COMPASS‐31 scores for EoE were compared to a previously published pediatric control group (N=223, PMID: 39223749). Gastric and duodenal biopsies were stained with tryptase and quantified. Comparisons were made using Mann‐Whitney, Pearson correlation, and linear regression.

**Results:** COMPASS‐31 scores were increased for EoE patients compared to controls (P<0.001, Fig 1 A). Among EoE patients, COMPASS‐31 was increased with Active EoE compared to Remission (P=0.02, Fig 1B). As shown in Fig 2, COMPASS‐31 scores positively correlated with child (r=0.4, p<0.001) and parent (r=0.3, p=0.004) composite PEESS scores as well as GERD, Nausea/Vomiting, and Pain domain scores. Positive correlations were also present between COMPASS‐31 and Dyspepsia Symptom Survey scores (r=0.5, p<0.001). COMPASS‐31 scores negatively correlated with Parent (r=‐0.3, p=0.01) and Child (r=‐0.4, p<0.001) PedsQL 4.0 scores. COMPASS‐31 Gastrointestinal and Vasomotor domains positively correlated with composite DSS and PEESS Parent and Child scores and were inversely associated with Parent and Child PedsQL 4.0 scores. Among EoE patients, 21 (26%) were hypermobile and had increased COMPASS‐31 scores (P=0.003, Fig 1 C). As age was associated with COMPASS‐31 scores (P=0.04, r=0.23), we performed linear regression and confirmed that EoE disease activity (p<0.01) and hypermobile status (p<0.01) independently predicted COMPASS‐31 scores, independent of age. MCs were not correlated with COMPASS‐31 composite and domain scores, and no differences were found in MCs based on hypermobility status.

**Conclusions:** Pediatric patients with active EoE and hypermobility experience increased dysautonomia symptoms, which are associated with increased EoE symptom burden and a lower quality of life. Gastric/duodenal MCs are not associated with dysautonomia or hypermobility. This study highlights a significant co‐morbidity in pedaitric EoE and the need for comprehensive assessment.


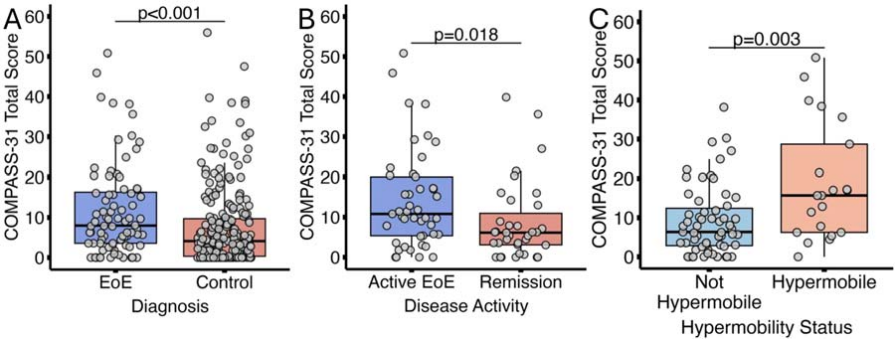


Fig 1: Increased COMPASS‐31 in A) EoE vs Control, B) Active EoE vs Remission, and C) Hypermobile vs Non‐Hypermobile EoE


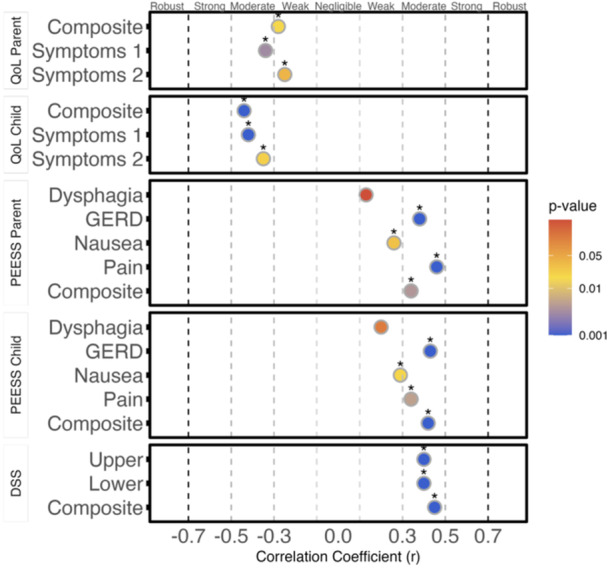


Fig 2. Correlation of COMPASS‐31 with Symptom and Quality‐of‐Life

## 365 PROVIDER PERSPECTIVES ON ESOPHAGEAL STRING TESTING AND HOW IT IMPACTS MANAGEMENT: A PROSPECTIVE STUDY

*Laura Quinn* ^*1*^, *Rachel Andrews* ^*1*^, *Stephanie Skirka* ^*1*^, *Jordon Koplon* ^*1*^, *Maureen Bauer* ^*2*^, *Nathalie Nguyen* ^*1*^

^*1*^ *Pediatric Gastroenterology, Hepatology, and Nutrition*, *University of Colorado*, *Denver*, *CO*; ^*2*^ *Allergy*, *University of Colorado Anschutz Medical Campus*, *Aurora*, *CO*

**Background:** The esophageal string test (EST) is a minimally invasive diagnostic tool used for EoE disease surveillance. While recent studies in children have reported on completion rates, complications, and predictors of successful administration of this novel assay, the ideal context for leveraging this test is yet to be defined. We hypothesized that esophageal string testing would primarily be used in patients with EoE in clinical remission to understand the efficacy of their current treatment regimen, and that results would align with provider's expectations but also facilitate EoE treatment changes if needed. We prospectively assessed the primary rationale for ordering ESTs, providers’ perspectives on EST results, and resultant management changes.

**Methods:** We performed a prospective survey of providers who ordered an EST for a patient at Children's Hospital Colorado between 3/1/2024 and 3/1/2025. Patients scheduled to undergo EST and the provider who ordered the testing were identified via review of the medical record. Providers were sent a survey link via REDCap 1‐2 weeks after the EST was completed, at which time EST results were available. The survey asked providers two yes/no questions: “Were the results aligned with what you expected?” and “Did the results change your management?”. Providers were asked to explain their answers if they indicated that EST results were not what they expected or if they led to a change in management. Assay‐specific data including the primary reason for ordering the EST, symptoms during EST administration, EST results, and time spent on EST administration were recorded. Patient‐specific information including EoE symptom scores (PEES 2.0) at time of EST were retrospectively collected.

**Results:** Thirty eight ESTs were ordered by nine different eligible providers during the study period. Of these 38 ESTs, 32 (84%) were successfully completed by patients, prompting subsequent provider survey. The 32 ESTs were completed by 27 patients (3 patients with 2 ESTs,1 patient with 3 ESTs). Patients were mean 12±3.5 years old and 70 percent were male. Thirty percent (n=8) had previously completed EST. The majority (n=19, 70%) had active EoE on last assessment and a minority (n=8,30%) had EoE in remission on last assessment. The results of the 32 completed ESTs reflected active EoE in 11 patients (34%,EoEScore 0.86±0.14), and 66% (n=21) reflected inactive EoE (EoEScore 0.36±0.05). Survey response rate was 97% (n=31). Providers reported their primary reason for ordering the EST, these are available in table 1. Providers answered “yes” to the question “were EST results what you expected?” in most (n=23, 74%) cases. EoE Scores were higher in the patients with unexpected results compared to those with expected results (mean 0.92±SD vs 0.43±SD, p<0.0001). Providers reported that EST results led to management changes in 90% of cases (n=28). None of the patients who underwent EST had an EGD in the subsequent two months. Scores were no different between cases where the assay was reported to drive management changes or not (0.55+SD vs 0.39+SD, p=0.32).

**Conclusions:** Physicians report that EST results drive EoE management changes in a variety of ways which prior to the availability of EST may have required endoscopy to assess these changes. EST results aligned with physician expectations in most cases.


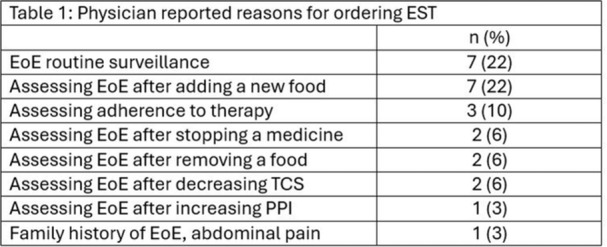


## 366 COMPARATIVE OUTCOMES OF DUPILUMAB VS. BUDESONIDE IN PEDIATRIC PATIENTS WITH EOSINOPHILIC ESOPHAGITIS AND COMORBID ASTHMA: A REAL‐WORLD ANALYSIS OF COMPLICATION RATES

*Melissa Ramirez Escobar*, *Shagun Sharma*, *Thomas Wallach*

*Pediatric Gastroenterology*, *SUNY Downstate Health Sciences University*, *Brooklyn*, *NY*

**Introduction:** Eosinophilic esophagitis (EoE) is a chronic and progressive type 2 inflammatory condition of the esophagus. Incidence is increasing annually, and there remains substantial evidence that the condition is underdiagnosed, in particular in patients of color. The disease comes with a high rate of association with other atopic diseases, as well as a substantial burden of medical care, with most patients having at least 1 endoscopy per year. Traditional therapy included dietary exclusion, proton‐pump inhibitors, and topical steroid usage, however a novel biologic therapy targeting IL‐4 and IL‐13 (dupilumab), has offered substantially higher efficacy, as well as impact on multiple atopic and respiratory disease types. Given impact on multiple disease domains, we set out to assess the impact of standard (budesonide) vs new therapy (dupilumab) in the clinical burden faced by patients with EoE and asthma.

**Methods:** We conducted a retrospective cohort study using the Global Collaborative TriNetX Network. Pediatric patients (ages 0–18 years) with ICD‐10 diagnosis of Asthma (J45) and eosinophilic esophagitis (K20.0) were identified and stratified into two cohorts: those treated with isolated oral budesonide and those on isolated dupilumab treatment. Propensity score matching was performed to balance cohorts by gender and race, minimizing confounding variables. Outcomes were identified using ICD‐10 codes for asthma exacerbation (J45.901), endoscopy [(CPT 1007241, 43239, 43235, 43259, 43242, 43237, 43248, 43249, 43238, 43241), (SNOMED 49230008, 446014007)], use of oral steroids (RXNORM 8640, 8638), and emergency visits occurring within a time window up to 1 year. Index events were defined based on the criteria used in the original cohort definitions. Number of instances was reported for the outcomes, including occurrences with zero instances. In addition, T‐Test statistics testing for the difference between the cohorts was included.

**Results:** The initial dataset included 1,160 pediatric patients with asthma and eosinophilic esophagitis on isolated dupilumab treatment and 688 pediatric patients with asthma and eosinophilic esophagitis on isolated budesonide treatment. After propensity score matching by sex (female and male) and race (White, Asian, African American), both cohorts were balanced, with 682 patients in each group.

The mean follow‐up duration in the isolated dupilumab cohort was 259 days (SD 130.44), with a median follow‐up of 355 days. In contrast, the isolated budesonide cohort had a longer mean follow‐up of 299 days (SD 118.52) and a median of 365 days.

Pediatric patients receiving dupilumab experienced fewer asthma exacerbations (t‐test ‐3.367, df=1362, p=0.001), had less use of oral steroids (t‐test ‐3.082, df=1362, p=0.002), and less emergency visits (t‐test ‐3.217, df=1362, p=0.001) than patients receiving budesonide. There was no statistically significant difference in the number of endoscopies.

**Conclusions:** EoE is a condition with substantial associated clinical burden in the form of atopic disease and endoscopic evaluation. While food elimination diets demonstrate histologic remission rates ranging from 45% to 96%, adherence remains a major challenge. High‐dose proton pump inhibitors (PPIs) show variable response rates (30%–70%) across different EoE phenotypes. Topical corticosteroids yield remission rates between 64% and 71%, though relapse after withdrawal and systemic side effects limit their long‐term utility.

In our study, dupilumab use was associated with lower instances of unspecified asthma exacerbation, use of oral steroids, and emergency visits suggesting potential benefit as a first‐line therapy in patients with comorbid atopic conditions. The study has limitations inherent to the TriNetX database and the impact of propensity score matching on reducing sample size and statistical power. Further research with larger cohorts and more granular control of confounding variables is warranted to better elucidate the therapeutic potential of dupilumab in EoE.

## 367 REWRITING THE SCRIPT: DUPILUMAB THERAPY AND ATOPIC AND COMORBID DISEASE PREVENTION IN PEDIATRIC EOSINOPHILIC ESOPHAGITIS

*Melissa Ramirez Escobar*, *Shagun Sharma*, *Thomas Wallach*

*Pediatrics*, *SUNY Downstate Health Sciences University*, *Brooklyn*, *NY*

**Introduction:** Eosinophilic esophagitis (EoE) is a chronic, progressive, type 2 inflammatory disorder of the esophagus. Its incidence continues to rise annually. EoE is frequently associated with other atopic conditions and carries a substantial burden of care. Traditional therapies, including dietary elimination, proton pump inhibitors (PPIs), and topical corticosteroids, aim to achieve both clinical and histologic remission, thereby reducing the risk of long‐term complications. Dupilumab, a novel biologic therapy targeting IL‐4 and IL‐13, has demonstrated efficacy in EoE and multiple other atopic conditions. Modification of Th2 signaling may alter disease formation or risk of other atopic conditions, or disease states of other conditions. This study aims to compare dupilumab and budesonide therapy in pediatric EoE patients, with a focus on the new incidence of comorbid atopic conditions.

**Methods:** We conducted a retrospective cohort study using the Global Collaborative TriNetX Network. Pediatric patients (ages 0–18 years) with ICD‐10 diagnosis of Eosinophilic Esophagitis (K20.0) were identified and stratified into two cohorts: those treated with budesonide who had never received dupilumab, and those on isolated dupilumab treatment. Propensity score matching was performed to balance cohorts by age at index, gender and race, minimizing confounding variables. Outcomes were identified using ICD‐10 codes for asthma exacerbation (J45.901), asthma (J45), eczema (L30), allergy (T78.40XA), anaphylactic shock (T78.2), depression (F32), and anxiety (F41) occurring within a time window up to 1 year. Index events were defined based on the criteria used in the original cohort definitions. Number of instances was reported for the outcomes, including occurrences with zero instances. In addition, T‐Test statistics testing for the difference between the cohorts was included. Kaplan‐Meier survival analysis was used to compare event‐free survival between cohorts, with results reported as hazard ratios (HRs) and corresponding 95% confidence intervals.

**Results:** The initial dataset included 3,072 pediatric patients with EoE on isolated dupilumab treatment and 13,511 pediatric patients with EoE on budesonide treatment. After propensity score matching by sex (female and male), age at index, and race (White, Asian, African American), both cohorts were balanced, with 3,072 patients in each group.

The mean follow‐up duration in the isolated dupilumab cohort was 262 days (SD 129.63), with a median follow‐up of 359 days. In contrast, the budesonide cohort had a longer mean follow‐up of 313 days (SD 107.97) and a median of 365 days.

Pediatric patients receiving dupilumab experienced fewer asthma exacerbations (t‐test ‐2.940, df=6142, p=0.003), as well as less disease formation for asthma (HR=0.702, 95% CI 0.54‐0.910), than patients receiving budesonide. There was no statistically significant difference in the disease formation for eczema, allergy, anaphylactic shock, and mental health disorders, however a trend towards less disease formation was identified in eczema, food allergy, depression, and anxiety.

**Conclusions:** Dupilumab plays a pivotal role in the treatment of immune‐mediated atopic conditions and is currently approved for multiple pediatric indications, including moderate‐to‐severe atopic dermatitis (≥6 months), moderate‐to‐severe asthma (≥6 years), eosinophilic esophagitis (≥1 year and >15 kg), and chronic spontaneous urticaria (≥12 years).

Our findings support its therapeutic benefit by demonstrating reduced asthma exacerbations and a lower incidence of new asthma diagnoses among patients treated with dupilumab compared with those treated with budesonide. We also observed trends suggesting a potential protective effect for disease formation of other atopic conditions, including eczema and allergy. While some associations did not reach statistical significance, likely due to limitations of the TriNetX database and reduced power from propensity score matching, these findings highlight the need for further research in larger pediatric cohorts to better understand the broader immunomodulatory effects of dupilumab.

## 368 CLINICAL PREDICTIVE MODEL FOR EOSINOPHILIC ESOPHAGITIS IN CHILDREN: EXTERNAL VALIDATION IN AN INDEPENDENT COHORT

*Bianca Sanchez* ^*1*^, *Zhaoxing Pan* ^*2*^, *Stephanie Borinsky* ^*3*^, *Evan Dellon* ^*3*^, *Pooja Mehta* ^*1*^

^*1*^ *Pediatrics*, *University of Colorado Anschutz Medical Campus School of Medicine*, *Aurora*, *CO*; ^*2*^ *Biostatistics*, *University of Colorado Anschutz Medical Campus School of Medicine*, *Aurora*, *CO*; ^*3*^ *The University of North Carolina at Chapel Hill*, *Chapel Hill*, *NC*

**Background:** Eosinophilic esophagitis (EoE) is a chronic, immune‐mediated disease of the esophagus with increasing prevalence in children. EoE can only be diagnosed via endoscopy – a costly and invasive procedure often requiring general anesthesia. Recently, a clinical predictive model was developed to help identify children at higher risk for EoE. This model effectively distinguished EoE from non‐EoE cases, achieving an area under the curve of 0.81 during initial analysis. The aim of this study was to externally validate the predictive model by applying it to an independent cohort.

**Methods:** Clinical characteristics were extracted from the medical record of 100 children with newly diagnosed EoE and 100 date‐matched non‐EoE controls undergoing endoscopy from November 2024 to May 2025. The clinical predictive model included the following variables: sex, history of food allergy, history of food impaction, and presence of adaptive eating behaviors, regurgitation, abdominal pain, and failure to thrive. The performance of the clinical tool was assessed using receiver operating characteristic (ROC) curve analysis. Assuming a true area under the curve (AUC) of 0.81, this sample size provided 89% power to detect that the AUC is significantly greater than 0.7 at a 0.05 significance level. Sensitivity, specificity, positive predictive value (PPV), and negative predictive value (NPV) were calculated across score thresholds. The optimal threshold was determined using Youden's J statistic.

**Results:** Demographic features of cases and controls are depicted in Table 1. Children with EoE scored significantly higher on the clinical predictive model than controls with a mean score of 0.68 (+/‐.26) and 0.40 (+/‐.26, respectively (p<.0001). The model's diagnostic performance yielded an AUC of 0.78 (95% CI: 0.71‐0.84) (Figure 1) which was in line with the original model's AUC of 0.81. The optimal cut‐off point, based on the highest Youden's J index (0.627), was 0.733, providing a sensitivity of 0.71, specificity of 0.91, PPV 0.87, NPV of 0.72, and overall classification accuracy of 80.4%.

**Conclusions:** The EoE clinical predictive model demonstrated external validity by accurately discriminating between cases and controls in an independent dataset, confirming its robustness and generalizability. The model demonstrated a favorable balance of sensitivity and specificity, supporting its potential role in identifying children at high risk for EoE who may benefit from early diagnostic intervention, while also aiding in the avoidance of unnecessary endoscopies in low‐risk patients. In summary, these results highlight the tool's promise as a clinical decision aid to prioritize patients for endoscopy and minimize unnecessary diagnostic interventions.


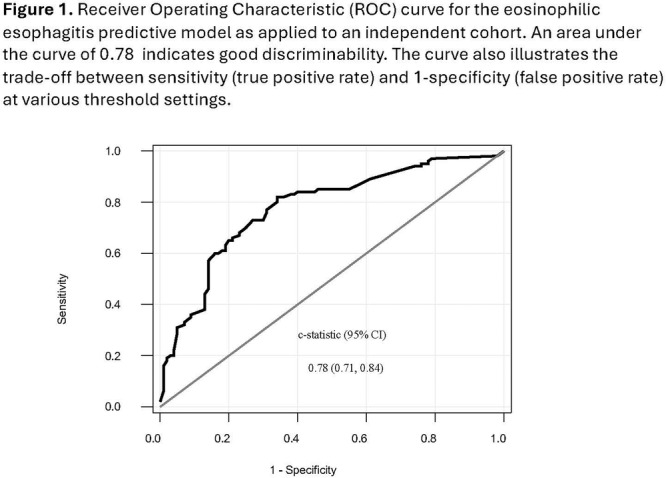



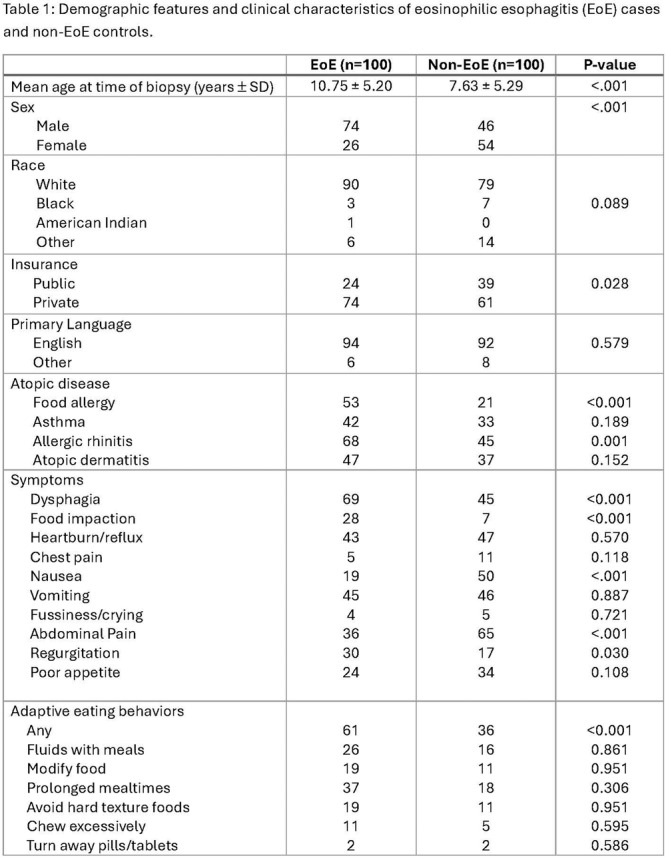


## 369 CHILDREN PRESENTING WITH INITIAL ESOPHAGEAL FOOD IMPACTIONS DEMONSTRATE IMPROVEMENT IN SYMPTOMS, HISTOLOGIC FINDINGS, AND FUNCTIONAL ESOPHAGEAL MEASURES ON REPEAT ENDOSCOPY AFTER TREATMENT

*Raul Sanchez* ^*1*^, *Md‐Rejuan Haque* ^*2*^, *Rajitha Venkatesh* ^*1*^, *John Russo* ^*1*^, *Elizabeth Erwin* ^*3*^, *Karla Vaz* ^*1*^, *Neetu Bali Puri* ^*1*^, *Desale Yacob* ^*1*^, *Peter Lu* ^*1*^, *Carlo Di Lorenzo* ^*1*^, *Muhammad Khan* ^*1*^

^*1*^ *Gastroenterology*, *Nationwide Children's Hospital*, *Columbus*, *OH*; ^*2*^ *Biomedical Informatics*, *The Ohio State University*, *Columbus*, *OH*; ^*3*^ *Allergy and Immunology*, *Nationwide Children's Hospital*, *Columbus*, *OH*

**Background:** Chronic inflammation in eosinophilic esophagitis (EoE) can lead to remodeling and subepithelial collagen deposition in the esophagus as well as strictures. This pattern of disease places patients at higher risk of esophageal food impaction (EFI). Functional lumen imaging probe (FLIP) provides information on esophageal structure and function in patients with EoE which has not been able to be well assessed previously. Our objective is to evaluate esophageal characteristics as measured by FLIP before and after treatment in children who present with EFI.

**Methods:** We performed a single‐center, prospective cohort study. Children 2‐18 years of age presenting with EFI requiring urgent endoscopic removal were recruited. Subjects and guardians each completed the Pediatric Eosinophilic Esophagitis Symptom Scores Version 2.0 (PEESS) at time of endoscopy to assess symptoms. Endoscopic food impaction removal was followed by FLIP by standard protocol and collection of esophageal biopsies. Subjects were then started on therapy at the managing physician's discretion with plan for follow up endoscopy. Follow up endoscopies with repeat FLIP and biopsies were completed at least 2 months after initial presentation. Repeat PEESS survey was also completed by subjects and guardian at that time. Symptom scores, FLIP measurements (presence of secondary peristalsis, maximum distensibility index (DI), maximum esophagogastric junction (EGJ) diameter), and histologic findings (eosinophil count and presence of fibrosis) at the index and the follow‐up endoscopy were compared with paired Wilcoxon rank sum tests.

**Results:** We have recrruited 15 children with initial EFI with FLIP assessment and 13 who have had follow up endoscopy and FLIP testing completed. Median age was 15.0 years (IQR 11.8–16.4) and subjects were mostly male (11, 85%). No patients had a diagnosis of EoE or prior dilation before their index endoscopy with EFI. At baseline, the median proximal esophageal eosinophils/high‐powered field (eos/hpf) was 38 (IQR 30–60) and median distal esophageal eos/pf was 22 (IQR 8–63). Eleven (85%) had fibrosis noted on esophageal biopsies. Total PEESS score and several PEESS subscores improved at time of follow up endoscopy, including dysphagia, nausea/vomiting, and pain subscores (**Table 1**). In the guardian survey alone there was improvement in GERD subscore (p=0.025). There was a significant decrease in proximal eosinophil count and fibrosis (p=0.003, p=0.016 respectively) but no improvement in distal esophageal eosinophil count or fibrosis (p = 0.366, p=0.371). There was significant improvement in maximum EGJ diameter on follow up endoscopy (13.2 mm vs. 15.7 mm, p=0.023). Maximum EGJ DI increased at follow up, but this change did not reach statistical significance (p=0.087).

**Conclusion:** In the first prospective study of children evaluated with FLIP at their index presentation with EFI prior to diagnosis of EoE, we found that treatment led to improvement in not only symptom scores and histological measures but also EGJ diameter. We observed a trend towards improvement in EGJ DI as well. This study provides insight into which esophageal charactersitics may play a role in EoE symptom generation.


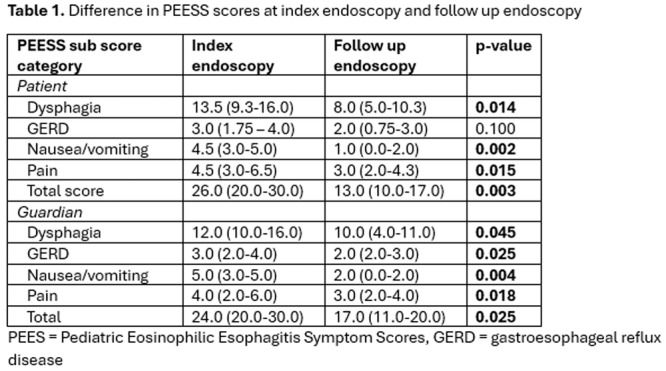



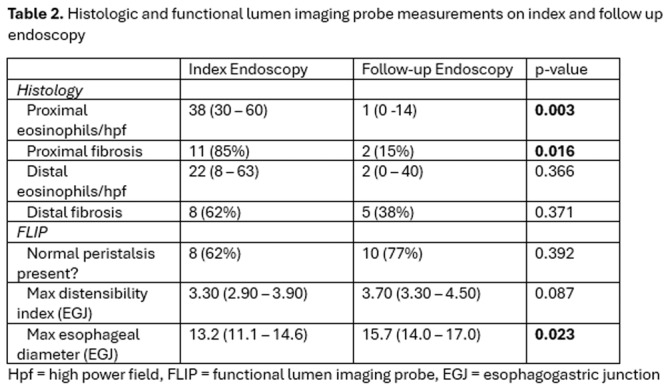


## 370 REAL‐WORLD EFFICACY AND SAFETY OF DUPILUMAB IN A SINGLE SITE LARGE CLINICAL EOE COHORT

*Richard Taylor* ^*1,2*^, *Colby Sharlin* ^*3*^, *Erick Madis* ^*4*^, *Kara Kliewer* ^*5*^, *Tetsuo Shoda* ^*1,5*^, *Scott Bolton* ^*1,2*^, *Philip Putnam* ^*1,2*^, *Marc Rothenberg* ^*1,5*^, *Vincent Mukkada* ^*1,2*^

^*1*^ *Pediatrics*, *University of Cincinnati College of Medicine*, *Cincinnati*, *OH*; ^*2*^ *Pediatric Gastroenterology*, *Cincinnati Children's Hospital Medical Center*, *Cincinnati*, *OH*; ^*3*^ *Gastroenterology*, *Phoenix Children's Hospital*, *Phoenix*, *AZ*; ^*4*^ *University of Cincinnati College of Medicine*, *Cincinnati*, *OH*; ^*5*^ *Allergy and Immunology*, *Cincinnati Children's Hospital Medical Center*, *Cincinnati*, *OH*

**Introduction:** Eosinophilic Esophagitis (EoE) is a chronic, immune‐mediated condition of the esophagus defined as ≥15 eos/hpf and symptoms of esophageal dysfunction. Dupilumab is a monoclonal antibody that binds IL4Rα and inhibits signaling of both IL4 and IL13 and is used to treat atopic conditions. It is a subcutaneous injection approved for EoE patients starting at 1 year of age at a weight based dose given every other week, and at a weekly 300 mg dose in patients 12 years and older. Our aim in this study is to assess real‐world efficacy and safety of dupilumab for treatment of EoE, with a focus on adolescents prescribed 300 mg every 2 weeks.

**Methods:** This is a retrospective chart review of patients seen at Cincinnati Children's Gastroenterology Clinic and/or the Cincinnati Center for Eosinophilic Disorders. Inclusion criteria were as follows: prescribed dupilumab for any indication up to June 1, 2023, aged 0 to 22 years at dupilumab start, and diagnosed with EoE. The subject population was assessed using descriptive statistics. Symptom data was recorded per patient report with dysphagia defined as pain or difficulty with swallowing. Side effects were defined as new symptoms or peripheral blood eosinophilia that began after dupilumab started. Histologic remission was defined as <15 eos/hpf, and further subdivided into the following groups:

*Achieved:* new remission after 1 follow‐up endoscopy

*Sustained:* remission on multiple follow‐up endoscopies

*Achieved with escalation:* remission only after increasing dose/frequency

*Maintained:* remission achieved prior to dupilumab and maintained on dupilumab

*Not achieved:* active EoE throughout.

**Results:** *Patient characteristics*

A total of 211 patients met inclusion criteria. Most patients (n=121, 57%) were between 12 and 17 years old at dupilumab start. Forty‐one (19%) patients were 11 years or younger and 49 (23%) patients were 18 years or older. There was a male predominance (72%). Only 15% had eosinophilic gastrointestinal disease in additional sites besides the esophagus.

*Histologic response*

Histologic response was assessed in the 156 patients with repeat endoscopy during the study period. At follow up endoscopy, 138 (88%) were in remission with only 5 (3%) requiring escalated dose or more frequent interval to achieve remission (Figure 1a). In the 96 patients 12 years and older who started dupilumab 300 mg every 2 weeks with follow up endoscopy, 85 (89%) achieved histologic remission with 2 (2%) requiring escalated dosing to achieve remission (Figure 1b).

*Symptomatic Response*

Initial and follow up symptom data was recorded in 155 patients, and dysphagia response to dupilumab was analyzed. Of the 39 (25%) patients who reported dysphagia at dupilumab initiation, 27 (69%) had their dysphagia resolve and none reported worsening dysphagia (Figure 2a). Out of the 100 patients 12 years and older on 300 mg every 2 weeks, 28 (28%) reported dysphagia at dupilumab initiation, and of these 23 (82%) had their dysphagia resolve (Figure 2b).

*Side Effects*

Among the 211 patients treated with dupilumab, 45 (21%) had at least 1 side effect. The most common side effects were ocular symptoms (11, 5%), injection site reaction (10, 5%), and blood eosinophilia (6, 3%). Of these 6 patients, 2 had extra‐esophageal involvement. There was no statistically significant difference in side effect occurrence rates between different median dosages. A total of 3 patients permanently discontinued dupilumab due to side effects (1 for needle phobia, 1 for hip pain, and 1 for severe hives at the injection site).

**Discussion:** This is the largest reported cohort of EoE patients treated with dupilumab in a clinical real‐world setting. We found that dupilumab was effective in inducing and maintaining both clinical and histologic remission, with a generally well‐tolerated side effect profile. In patients 12 years and older every 2 weeks dosing successfully induced symptomatic and histologic remission.


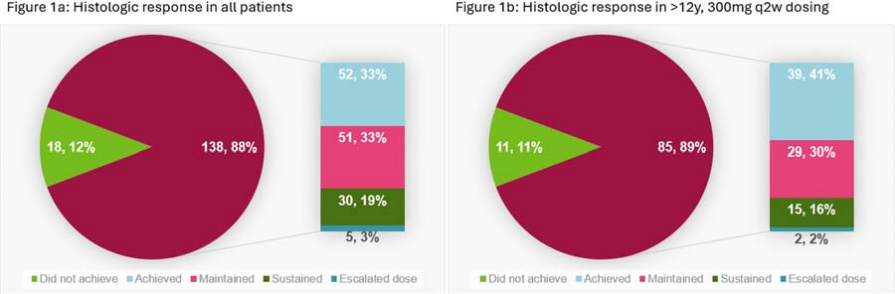


Figure 1: Histologic response to Dupilumab

**a.** Response in all patients and dosing regimens

**b.** Response in patients aged ≥12 years on 300 mg q2w dosing


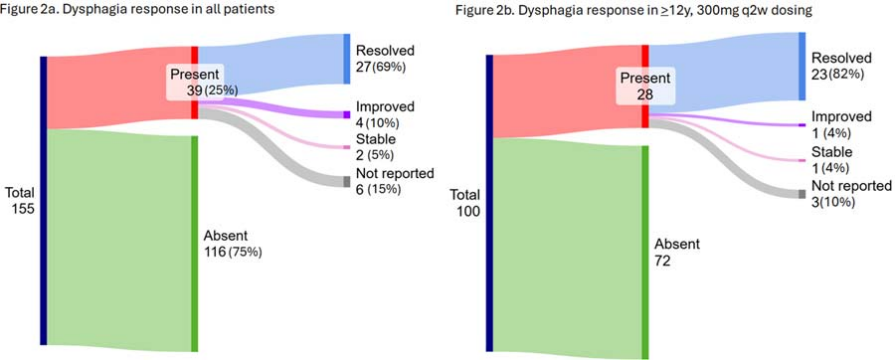


Figure 2: Dysphagia response to Dupilumab

a. Response in all patients

b. Response in patients aged >12 years on 300 mg q2w dosing

## 376 EXPLORING ALPHA‐FETOPROTEIN AS A PROGNOSTIC MARKER IN PEDIATRIC MASLD

*Samira Ali* ^*4*^, *Walaa Elfar* ^*4*^, *Keith Hazleton* ^*1*^, *Yasemin Cagil* ^*2*^, *Dorothy Gilbertson‐Dahdal* ^*3*^, *Pawel Kiela* ^*5*^

^*1*^ *Pediatric Gastroenterology,Hepatology and Nutrition*, *Children's Hospital Los Angeles*, *Los Angeles*, *CA*; ^*2*^ *Pediatric Gastroenterology,Hepatology and Nutrition*, *Stanford Medicine*, *Stanford*, *CA*; ^*3*^ *Pediatric Radiology and Imaging*, *Banner ‐ University Medical Center Tucson*, *Tucson*, *AZ*; ^*4*^ *Pediatirc Gastroenterology,Hepatology and Nutrition*, *Diamond Children's Medical Center*, *Tucson*, *AZ*; ^*5*^ *Clinical and Translational Research*, *Diamond Children's Medical Center*, *Tucson*, *AZ*

**Background:** Metabolic dysfunction‐associated steatohepatitis (MASLD), formerly known as nonalcoholic fatty liver disease, is the most common form of liver disease in children, with prevalence estimates as high as 11% in children over nine years of age. Without intervention, approximately 20% of affected children may develop hepatic fibrosis, which could ultimately necessitate liver transplantation.

Childhood obesity, a key risk factor for MASLD, affects 18.8% of children aged 10–17 in Arizona, with higher prevalence among Hispanic and Native American/Alaskan Native populations compared to non‐Hispanic White children. Monitoring and preventing MASLD progression is essential. While noninvasive diagnostic tools are effective in adults, they are less accurate in pediatric populations.

**Objective:** This study aims to evaluate the prognostic potential of alpha‐fetoprotein (AFP) as a noninvasive biomarker for MASLD in children. Specifically, we assess whether AFP correlates with standard serum biomarkers and MRI elastography (MRE) findings and whether AFP levels change over time in pediatric MASLD cases.

**Methods:** This ongoing prospective study enrolls pediatric patients aged 9–18 diagnosed with MASLD. Patients with elevated liver enzymes due to causes other than MASLD are excluded. Enrollment began in April 2024 and will continue through September 2025.

Data collection includes blood analysis, imaging, and chart review. Blood samples are collected at baseline, 3–4 months, and 6–9 months and include measurements of AFP, complete metabolic panel, complete blood count, lipid profile, hemoglobin A1c (HbA1c), gamma‐glutamyl transferase (GGT), body mass index (BMI), and blood pressure.

Spearman rank correlation tests are used to assess relationships between AFP levels and clinical variables.

**Results:** To date, 12 participants have been enrolled. The average age is 15 years (SD: 3), with 92% male and 50% identifying as Hispanic or Latino. No participants are on blood pressure, lipid‐lowering, or GLP‐1 medications; one participant uses metformin.

**Mean weight:** 101 lbs (SD: 38)

**Mean height:** 168 cm (SD: 15)

**Mean BMI:** 35 (SD: 9)

**Mean HbA1c:** 5.54 (SD: 0.34)

**LDLC:** 101 mg/dL (SD: 24)

**HDLC:** 42.6 mg/dL (SD: 6.1)

AFP levels are currently available for 5 participants (mean: 1.72, SD: 0.27).

Preliminary Spearman correlation results show:

**Strong positive correlations** between AFP and BMI (ρ = 0.949, p = 0.051), HbA1c (ρ = 0.949, p = 0.051), and LDLC (ρ = 0.949, p = 0.051)

**Strong negative correlation** between AFP and HDLC (ρ = ‐0.738, p = 0.262)

These findings suggest potential relationships but are highly preliminary due to the small sample size.

**Conclusion:** Despite the limited sample size, preliminary findings suggest a possible association between AFP and several metabolic markers in pediatric MASLD. Notably, trends observed in the AFP vs. BMI and AFP vs. HbA1c indicate strong positive correlations. These trends are consistent with previously established findings in adult MASLD populations, supporting the potential of AFP as a prognostic biomarker. While early, these observations suggest AFP may provide additional clinical insight alongside current diagnostic tools and merit continued investigation as more data become available.

**Limitations:** Small sample size, with only 5 AFP results currently available. Ongoing recruitment and data collection.

Scheduling difficulties related to school absences, though participation is expected to increase during the summer months.

## 379 COVID‐19 VACCINATION DECREASES HEPATOBILIARY MANIFESTATIONS OF COVID‐19 INFECTION

*Mandeep Bajwa* ^*1*^, *Janet Rosenbaum* ^*2*^, *Thomas Wallach* ^*2*^, *Amber Hildreth* ^*1*^

^*1*^ *Pediatrics*, *University of California San Diego*, *La Jolla*, *CA*; ^*2*^ *SUNY Downstate Health Sciences University*, *New York*, *NY*

**Background:** COVID‐19 has demonstrated the capacity to impact multiple tissue types, but particularly tissue expressing high levels of ACE2. Cholangiocytes are one of these tissue types, and this is consistent with anecdotal reporting of cholestasis and hepatobiliary complications seen in COVID‐19 infected patients. We aim to assess the natural history of this COVID‐19‐associated hepatobiliary injury in patients who received the COVID‐19 vaccine versus those who were unvaccinated.

**Methods:** Retrospective chart review of patients ages 0‐17 presenting to Rady Children's Hospital San Diego who had a documented COVID‐19 infection (antibody, antigen, PCR, diagnosis list) and Comprehensive Metabolic Panel (CMP) obtained at time of presentation. COVID‐19 vaccination status was collected on all patients. Pearson chi‐squared test was used for statistical analysis.

**Results:** Among the 1751 patients in this study, 20.5% had at least one COVID‐19 vaccine at least 14 days prior to COVID‐19 infection. Vaccinated patients had lower bilirubin, alkaline phosphatase, and aspartate aminotransferase (AST) than unvaccinated: on average, bilirubin was 0.59 versus 0.90 mg/dL; alkaline phosphatase was 161 versus 210 U/L; and AST 45 versus 52 U/L (Table 1, Figure 1 and 2). Vaccinated patients were also older than unvaccinated: 11.8 versus 6.0 years (Table 1). Vaccination status was not associated with alanine aminotransferase (ALT) or albumin levels (Table 1).

**Conclusions:** In this single center retrospective study, we found that vaccination against COVID‐19 decreases severity of hepatobiliary injury seen during an active COVID‐19 infection. This finding is consistent with the known pathogenicity of the SARS‐CoV‐2 virus and affinity for tissues expressing ACE2, in this case cholangiocytes. Immunity to COVID‐19 infection improves liver tropism and results in less severe hepatobiliary manifestations of disease.


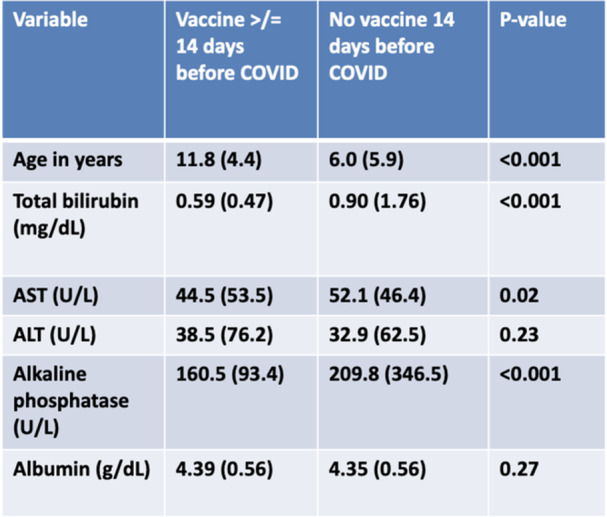



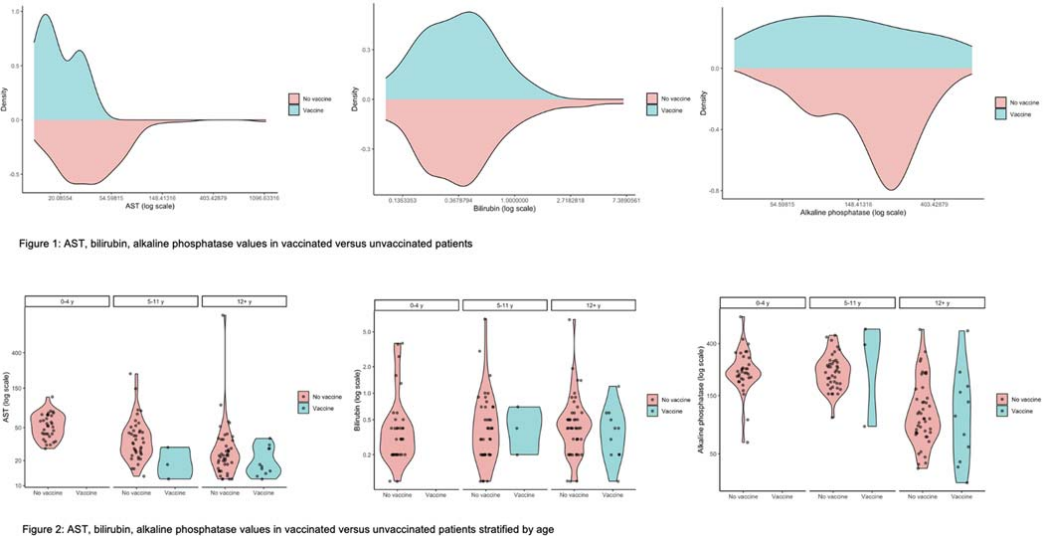


## 386 POST‐TRANSPLANT LYMPHOPROLIFERATIVE DISORDER (PTLD) VS EPSTEIN‐BARR VIRUS (EBV) DNAEMIA IN PEDIATRIC LIVER TRANSPLANT PATIENTS: A SINGLE‐CENTER EXPERIENCE

*Erin Chen* ^*1,2*^, *Natasha Dilwali* ^*3*^, *Wikrom Karnsakul* ^*3*^, *Sara Kathryn Smith* ^*3*^

^*1*^ *Pediatrics*, *The Children's Hospital of Philadelphia*, *Philadelphia*, *PA*; ^*2*^ *Pediatrics*, *The Johns Hopkins University School of Medicine*, *Baltimore*, *MD*; ^*3*^ *Pediatric Gastroenterology*, *Johns Hopkins Children's Center*, *Baltimore*, *MD*

**Background:** Liver transplantation is a life‐saving intervention for children with end‐stage liver disease. Advances in immunosuppressive therapy and surgical techniques have improved survival and graft outcomes. However, these advancements have also led to new complications, notably post‐transplant lymphoproliferative disorder (PTLD). PTLD is strongly associated with Epstein‐Barr Virus (EBV) infection, which is particularly relevant in children, who are often EBV‐seronegative at the time of transplant. Previous studies have identified several key risk factors for PTLD, including donor‐recipient EBV serostatus mismatch, elevated EBV viral load, early post‐transplant EBV infection, and exposure to high levels of immunosuppression. While many pediatric transplant patients may experience EBV DNAemia after transplantation, distinguishing which patients will progress to PTLD remains a clinical challenge. Improved characterization of patients who develop PTLD compared to those with isolated, asymptomatic EBV DNAemia could enhance risk stratification and inform monitoring and management strategies.

**Methods:** We conducted a single‐center retrospective chart review of all pediatric liver transplant recipients over the past 10 years at Johns Hopkins Children's Center. Patients with either PTLD or isolated EBV DNAemia were identified using ICD‐9 and ICD‐10 codes. Isolated EBV DNAemia was defined as any detection of EBV virus in plasma through polymerase chain reaction (PCR) testing in a pediatric liver transplant patient who did not develop PTLD. Data included PTLD status, EBV titers (initial and peak), donor and recipient EBV serostatus, immunosuppression regimen at the time of peak EBV titer, and post‐transplant induction therapy. All data was recorded in REDCap, and descriptive statistics was used to summarize findings.

**Results:** Twelve pediatric liver transplant patients met the inclusion criteria. Four developed PTLD, while eight had isolated EBV DNAemia. Among patients with PTLD, only one (25%) received a liver from a living donor, compared to seven of eight patients (87.5%) in the EBV DNAemia group. Donor EBV serostatus also varied between groups: two (50%) PTLD patients received grafts from EBV‐seropositive donors, compared to seven patients (87.5%) in the EBV DNAemia group. Mean peak EBV titers were higher in PTLD patients (mean log 4.12 ± 0.78) than in those with isolated EBV DNAemia (mean log 3.48 ± 0.99). The rate of EBV titer increase was also greater in the PTLD group (0.197 ± 0.074 log/week) compared to the EBV DNAemia group (0.018 ± 0.016 log/week). Additionally, the time to initial EBV detection and to peak titer was shorter in PTLD patients (initial: 2.1 ± 0.85 months; peak: 5.3 ± 1.6 months) than in those with EBV DNAemia (initial: 7.5 ± 10.4 months; peak: 42.9 ± 37.9 months). Immunosuppression at the time of peak EBV titer was more intensive among PTLD patients: one was on triple therapy, two on dual therapy, and one on monotherapy. In contrast, all patients with isolated EBV DNAemia were on monotherapy.

**Conclusions:** In this single‐center retrospective cohort of pediatric liver transplant recipients, differences emerged between patients who developed PTLD and those with isolated EBV DNAemia. PTLD was associated with earlier EBV onset, higher peak viral loads, a more rapid rise in viral titers, and more intensive immunosuppression regimens. These findings suggest potential clinical patterns that could be used to risk‐stratify and improve surveillance in patients. However, given the limited sample size, further studies are warranted to validate these.

**Acknowledgements:** We would like to thank the Johns Hopkins Biostatistics, Epidemiology, and Data Management service (BEADCore) for their invaluable assistance in setting up and managing the RedCAP database used for data collection.


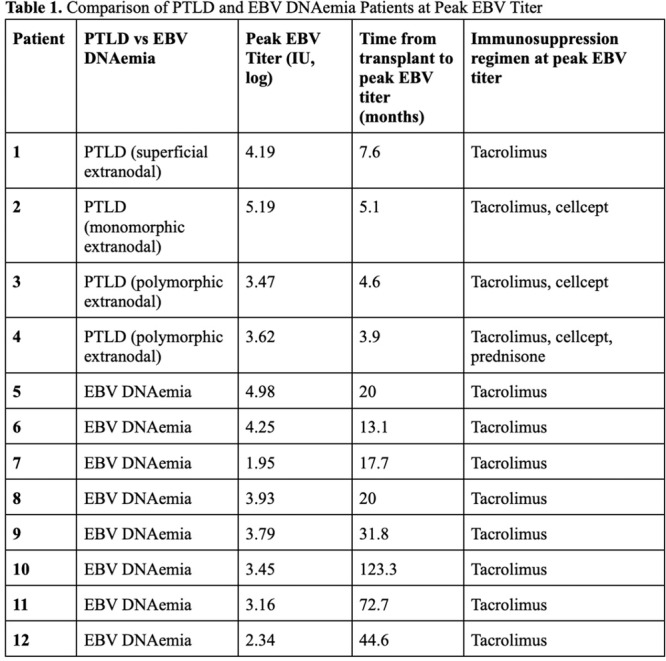


## 387 FROM SNEEZES TO STEATOSIS: PEDIATRIC COVID‐19 INFECTION INCREASES THE RISK OF METABOLIC DYSFUNCTION‐ASSOCIATED LIVER DISEASE

*Jacob Coene* ^*1*^, *Zella Beril* ^*2,3*^, *Kyle Sexton* ^*4*^, *Apryl Susi* ^*2,3*^, *Kristan Madison* ^*3*^, *Cade Nylund* ^*3*^

^*1*^ *Pediatrics*, *87th Medical Group*, *McGuire Air Force Base*, *NJ*; ^*2*^ *The Henry M. Jackson Foundation for the Advancement of Military Medicine*, *Bethesda*, *MD*; ^*3*^ *Pediatrics*, *Uniformed Services University of the Health Sciences F Edward Hebert School of Medicine*, *Bethesda*, *MD*; ^*4*^ *Pediatrics*, *Tripler Army Medical Center*, *Tripler Army Medical Center*, *HI*

**Background:** Metabolic dysfunction‐associated steatotic liver disease (MASLD) and steatohepatitis (MASH) are prevalent causes of chronic liver disease in children and youth, significantly impacting quality of life and healthcare resources. SARS‐Cov‐2 can directly infect adipocytes leading to inflammation and possibly adipocyte dysfunction, a known contributor to MASLD/MASH. Although there is developing evidence that COVID‐19 may increase the risk of adiposopathy related conditions, its effect on metabolic dysfunction‐associated liver disease remains unclear. This study investigated the potential association between prior COVID‐19 infection and new‐onset MASLD/MASH in children and youth.

**Methods:** We conducted a retrospective, propensity score‐matched cohort study using the Military Health System Database for dependent children and youth ages 8‐24 years. COVID‐19 infections (07/2020‐06/2021) were identified by ICD‐10 diagnosis codes and laboratory results. COVID‐19 were matched 1:2 to un‐exposed controls using 1800 clinical variables representing diagnoses, hospitalizations, procedures, and prescriptions converted to a propensity score, age, sex, beneficiary type and month. There are no ICD‐10 codes specific for the updated terminology and stricter diagnostic criteria of MASLD/MASH. For this reason outcomes were identified using ICD‐10 codes K75.81 (non‐alcoholic steatohepatitis) and K76.0 (non‐alcoholic fatty liver disease). To focus on incident cases, those with pre‐existing diagnoses were excluded. Proportional Hazards regression, adjusted for prior overweight/obesity (identified by ICD‐10 codes), estimated hazard ratios (HR) for incident MASLD/MASH.

**Results:** Among 109,014 participants (after excluding prevalent cases), 213 (0.2%) developed metabolic dysfunction‐associated liver disease (207 (97.2%) with MASLD, 8 (3.8%) with MASH (2 with both)). The median age at diagnosis was 18 years (interquartile range [IQR] 16‐20), slightly older than those without an incident diagnosis (median 16 years (IQR 13‐19)). Compared to the overall cohort (54.1% female), there was a modest female predominance in those affected by liver disease (58.2%). Crucially, prior COVID‐19 infection was independently associated with an increased risk of developing MASLD/MASH (HR 1.50; 95% CI 1.09‐2.05), even after adjusting for overweight/obesity status. As anticipated, overweight (HR 3.60; 95% CI 1.58‐8.22) and obesity (HR 5.56; 95% CI 3.56‐8.69) were also strong independent risk factors.

**Conclusion:** This large propensity score‐matched cohort study demonstrates a significant association between COVID‐19 infection and an increased risk of subsequent MASLD/MASH in children and youth, independent of pre‐existing weight status. These findings suggest the importance of targeted monitoring for steatosis/steatohepatitis in children and youth post‐COVID‐19. Early identification and interventions are critical to mitigate long‐term complications, especially given the bidirectional relationship between MASLD/MASH and type 2 diabetes mellitus as well as hypertension. These results may underscore a potential indirect benefit of COVID‐19 vaccination in preventing pediatric liver disease, warranting further investigation.

*The opinions and assertions expressed herein are those of the author(s) and do not reflect the official policy or position of the Uniformed Services University of the Health Sciences or the Department of Defense*


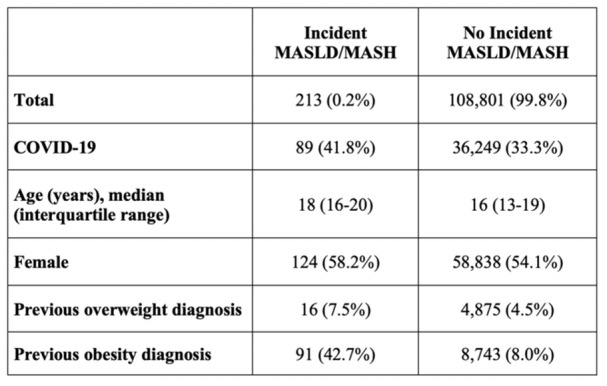


Table 1: Demographics of the study cohort with values reported separately for those with and without incident MASLD/MASH


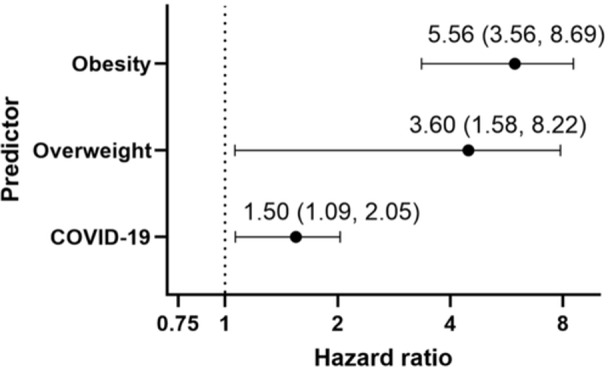


Figure 1: Hazard ratios, from a Cox proportional hazards model, for incident MASLD/MASH based on weight status and COVID‐19 exposure

## 388 ALAGILLE SYNDROME:A NEW CHALLENGE IN TRANSITION TO ADULT CARE

*Daniel D'Agostino* ^*2,1*^, *Carol Lezama* ^*3,4*^, *Maria Belen Pallitto* ^*2*^, *Gustavo Boldrini* ^*2*^

^*1*^ *Department of Pediatrics*, *Hospital Italiano de Buenos Aires*, *Buenos Aires*, *Buenos Aires*, *Argentina*; ^*2*^ *Gastroenterology‐ Hepatology Unit Department of Pediatrics*, *Hospital Italiano de Buenos Aires*, *Ciudad Autónoma de Buenos Aires*, *Buenos Aires*, *Argentina*; ^*3*^ *Hepatology*, *Children's*, *Buenos Aires*, *Buenos Aires*, *Argentina*; ^*4*^ *Hepatologia*, *Hospital de Niños*, *Buenos Aires*, *Buenos Aires*, *Argentina*

**Background:** Alagille syndrome (ALGS) is a complex and rare genetic disorder, historically regarded as a disease of childhood. Medical advances have allowed patients to transition to adult care.

**Aim**. To understand the evolution of adult ALGS patients referred from pediatric care.

**Methods:** This retrospective, descriptive study reviewed the clinical records of 91 ALGS patients from two pediatric centers between 1990 and 2024. Thirteen were lost to follow‐up; of the remaining 78, complete data were obtained from 16 patients, who reached adulthood.

**Results:** A total of 16 clinical histories were evaluated. Median age of patients was 30.3 years (IQR: 20‐40 years); 9 were males. The patients were classified into two groups: those who had undergone a liver transplant in childhood (Group 1) and those who preserved their native liver (Group 2) at transition to adult care.

Group 1 included 9 patients, with a median age of 29.3 years (IQR: 21‐36) and a mean age of 3.4 years at the time of transplantation. All patients in this group receive tacrolimus as their immunosuppressive medication. In these patients, the major complication was chronic renal failure; 5 out of 9 (55 %) experienced a mild to severe decrease in glomerular filtration rate (GFR): one needed a kidney transplant (age: 34), one died from chronic renal failure and multi‐organ failure (age: 29). Another patient died from a biliary complication with sepsis (age: 28) while listed for liver retransplantation.

Group 2 comprised 7 patients, with a median age of 31.7 years (IQR: 22‐40). Four experienced mild to moderate cholestasis and pruritus. One required a liver transplant at 30 years old, another was diagnosed with hepatocarcinoma (HCC), and a third suffered from ascitic syndrome. The last 2 patients passed away.

After a prolonged follow‐up, the survival rate for patients with ALGS was 75 %.

**Conclusion:**Alagille syndrome is a rare disease associated with cholestasis in children. Although it was largely unfamiliar to adult practitioners in the past, many patients now transition into adulthood. It is vital to alert adult healthcare providers who receive these patients about the renal involvement of those who have been transplanted and certain aspects of the disease's natural history that require lifelong surveillance.

## 393 RETROSPECTIVE ANALYSIS OF WHOLE‐GENOME SEQUENCING IN PATIENTS PRESENTING WITH CHOLESTASIS

*Jackson Fein* ^*1*^, *Lauren Chun* ^*1*^, *Laura Tobin* ^*2*^, *Amber Hildreth* ^*1*^

^*1*^ *Pediatrics*, *University of California San Diego*, *La Jolla*, *CA*; ^*2*^ *Rady Children's Institute for Genomic Medicine*, *San Diego*, *CA*

**Background:** Genetic disorders account for approximately 25% of cholestatic liver diseases. Early genetic testing in infants with cholestasis may yield significant changes in clinical management. At the Rady Children's Institute for Genomic Medicine (RCIGM), only variants with a high likelihood of being diagnostic are reported, compared to genetic panels which often report multiple variants possibly related to the patient's phenotype. In the first stage of this retrospective study, we collected genetic testing data on all patients admitted to Rady Children's Hospital who underwent WGS with cholestasis as a part of their presenting phenotype.

**Methods:** The RCIGM Database and Rady Children's Hospital Electronic Medical Record (EMR) was queried for patients ≥ 34 weeks gestational age and ≤6 months of age at presentation between the 2016‐2024 and with cholestasis (cases before 12/2017 unable to be included by time of submission due to data storage change). Cholestasis was defined as a direct bilirubin (DB) level ≥ 1 mg/dL at the time of presentation. A comprehensive list of Human Phenotype Ontology (HPO) terms related to cholestasis were used to identify subjects of interest (Table 1) WGS and genetic panel results were collected.

**Results:** A total 212 patients had at least one HPO term related to cholestasis. 91 patients met the definition of cholestasis by having DB ≥ 1 mg/dL, and 73 of these patients were greater than 34 weeks gestational age. Of the 73 patients meeting inclusion criteria, 40 patients had at least one genetic variant reported on either WGS or genetic panels (55%). 22 patients (30%) had genetic variants reported on WGS. 21 patients had genetic panel testing in addition to WGS, and all 21 patients (100%) had at least one genetic variant reported. 3 patients had findings reported on both WGS and genetic panels, with 1 patient having concordant diagnoses reported.

The most common variants reported on WGS was G6PD (5 patients). The most common variants reported on panel testing was in UGT1A1 (10 patients) with the risk allele for Gilbert's Syndrome being the most common (9 patients). 3 patients had CFTR c.1210‐34TG[11]T[5] intronic variant reported, and interestingly the earliest report classified this variant as likely benign; however, the later cases had up scored the variant to likely pathogenic.

**Conclusions:** In the first stage of this retrospective study, we describe the genetic findings in patients greater than 34 weeks gestational age who had cholestasis as a part of their presenting phenotype. Over half of the patients had a genetic variant identified, however further analysis is needed to understand how these findings contributed to the patient's diagnosis and management. Interestingly, one variant was up scored during this time period, highlighting the importance of frequent re‐analysis of genetic findings. This is in particular important for VUS's, as well as previously non‐diagnostic WGS results, and this analysis will be included in the next steps of our study.


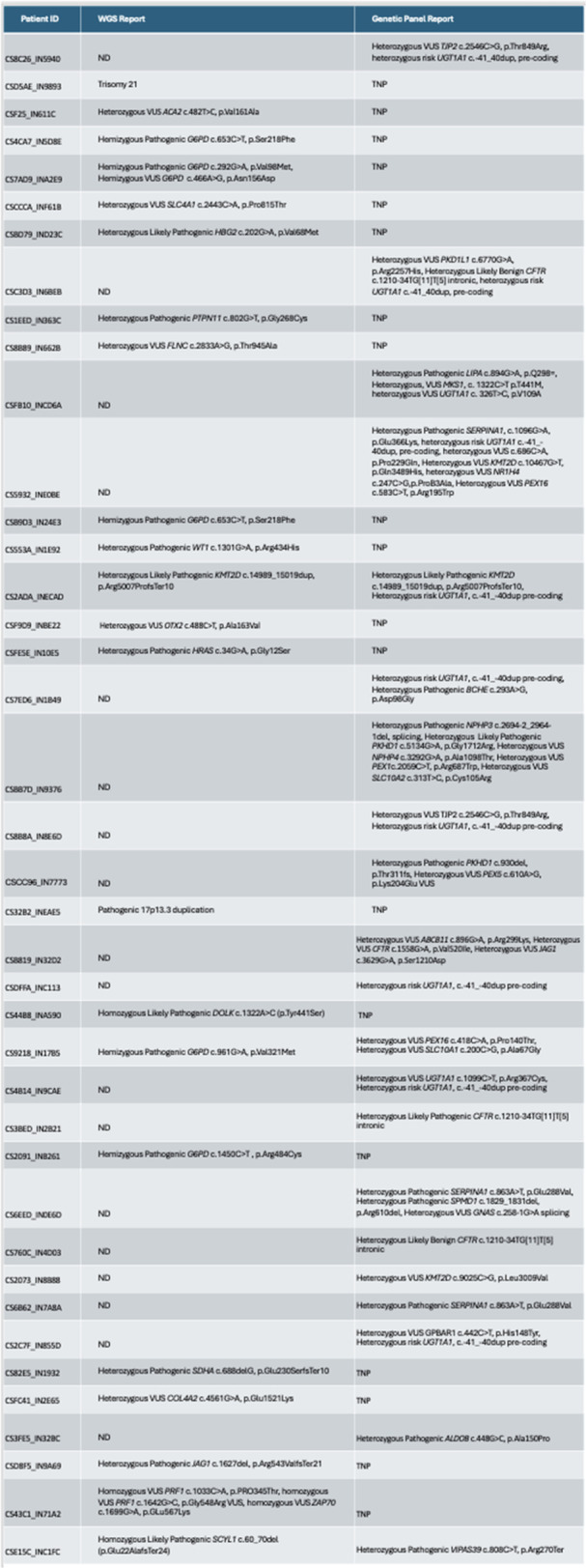



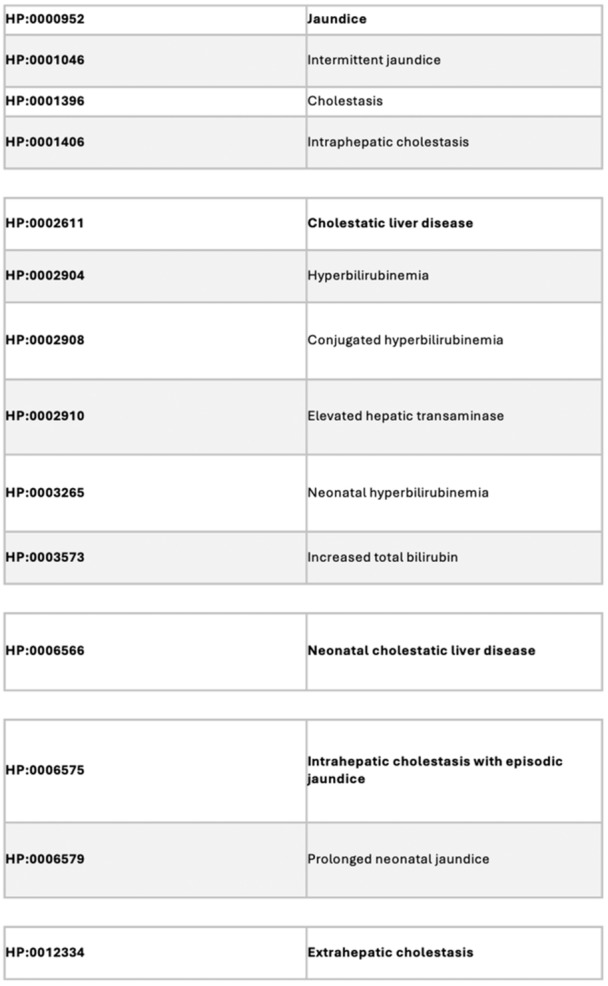


## 395 IMPLEMENTATION OF A FRACTIONATED BILIRUBIN‐BASED SCREENING PROTOCOL FOR BILIARY ATRESIA: EXPLORING NEW THRESHOLDS

*Sonia Hernandez*, *Jay Shah*

*Pediatrics*, *The University of Texas Health Science Center at San Antonio*, *San Antonio*, *TX*

Biliary atresia (BA) is the leading cause of pediatric liver transplantation. Early Kasai portoenterostomy (KP) can improve native liver survival but diagnosis delays are common. The United States lacks a universal biliary atresia screening program. A two‐stage direct bilirubin‐based screening protocol has shown promise. The initial study conducted at University Hospital in San Antonio Feb 2021‐ Jan 2024 showed high specificity and low false positive rates which demonstrated that this protocol is reliable. This study explores the continuation of this screening protocol by using a higher threshold of screening along with its challenges.

The study included all live births at University Hospital in San Antonio, Texas from February 1^st^ 2021 to May 21, 2025. The two‐stage screening involved measuring direct bilirubin (DB) levels between 24‐ and 48‐ hours of life, with follow‐up testing at two weeks if initial levels were elevated. Infants with persistent or rising DB levels were referred to pediatric hepatology for confirmatory testing. The initial protocol (Feb 2021‐ Jan 2024) used DB of 0.5 mg/dL as the threshold for retesting in 2 weeks while the newer protocol (Oct 2024 – now) used DB of 1.0 mg/dL. The primary outcome was diagnostic accuracy between the two different thresholds. Secondary outcomes included screening completion rates, successful referral rates, timeliness of diagnosis, and age at KP.

This ongoing study aims to evaluate the effectiveness of a revised direct bilirubin threshold for early screening of biliary atresia. Preliminary results have led to the identification of one confirmed case, demonstrating the potential utility of the new protocol. As data collection continues, the study seeks to refine and validate a standardized threshold that could be adopted across hospital systems to improve early detection and intervention outcomes.

## 397 SYSTEMATIC LITERATURE REVIEW OF THE CLINICAL AND HUMANISTIC BURDEN OF BILIARY ATRESIA

*Emmanuelle Kaltenbach* ^*1*^, *Claudia Mighiu* ^*2*^, *Sushma Pabbineedi* ^*2*^, *Pushpa Hossain* ^*2*^

^*1*^ *Ipsen*, *London*, *United Kingdom*; ^*2*^ *Prime HCD*, *Knutsford*, *United Kingdom*

**Background:** Biliary atresia (BA) is a rare, progressive fibroinflammatory cholangiopathy in infants. Primary treatment involves surgical intervention with Kasai portoenterostomy (KPE), aiming to reconstruct bile flow to prevent liver failure. Despite KPE, many affected children will develop end‐stage liver disease, requiring liver transplantation. Despite improvement in diagnosis and management, BA is a major pediatric health issue worldwide, and no approved drug is available to delay or prevent complications. The aim of this systematic literature review (SLR) was to describe the clinical and humanistic burden of BA.

**Methods:** Databases were searched for interventional and observational studies (SLRs and meta‐analyses) published from 2014–2024, including articles reporting on patients of any age diagnosed with BA and receiving any or no intervention. This SLR focused on natural history of the disease, treatment patterns, and patient‐reported outcomes, describing health‐related quality of life (HRQoL).

**Results:** The number of included studies is presented in Figure 1. In studies reporting clinical outcomes, age at KPE varied with a mean of 45 days at the lower end, and most studies reported the procedure around 50–60 days. Native liver survival and graft survival were reported in 13 and 4 studies, respectively; reported rates are described in Table 1. Twelve studies reported drug therapies, including nutritional supplements, immunosuppressants, postoperative antibiotics, and steroid therapy. Studies indicated lower HRQoL in BA patients compared with healthy peers, with mean Pediatric Quality of Life Inventory (PedsQL) scores of 80–90. Caregivers reported higher anxiety levels during hospitalization, with anxiety 36% higher compared with reference values.

**Conclusions:** Patients with BA undergo many interventions early in life and require continuous medication to manage symptoms and complications from surgery, contributing to a substantial humanistic and clinical burden for patients and caregivers. However, there is a gap in knowledge regarding the long‐term evolution of patients' HRQoL, and data on caregivers is scarce, highlighting the need for further research.


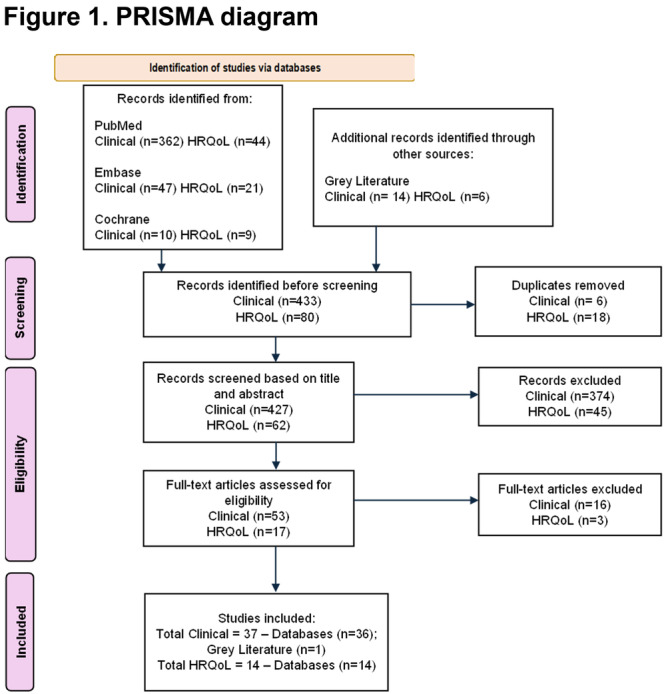



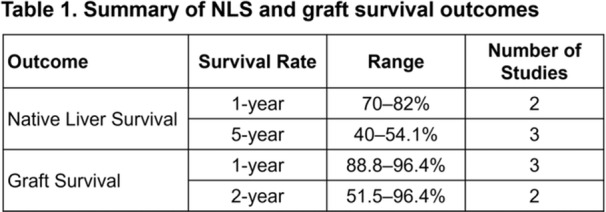


## 399 SALVAGING REFRACTORY ANTIBODY‐MEDIATED REJECTION IN PEDIATRIC LIVER TRANSPLANT PATIENTS AT A HIGH‐VOLUME TRANSPLANT CENTER

*Parastou Khalessi Hosseini* ^*1,2*^, *Arianna Barbetta* ^*2*^, *Sarah Bangerth* ^*2*^, *Vivienne Reed* ^*2*^, *Ronald Martinez* ^*2*^, *Shengmei Zhou* ^*3,2*^, *Kambiz Etesami* ^*4,2*^, *Rohit Kohli* ^*1,2*^, *Juliet Emamaullee* ^*4,2*^

^*1*^ *Gastroenterology, Hepatology, Nutrition*, *Children's Hospital Los Angeles*, *Los Angeles*, *CA*; ^*2*^ *University of Southern California*, *Los Angeles*, *CA*; ^*3*^ *Pathology*, *Children's Hospital Los Angeles*, *Los Angeles*, *CA*; ^*4*^ *Transplant Surgery*, *Children's Hospital Los Angeles*, *Los Angeles*, *CA*

**Background:** Antibody‐mediated rejection (AMR) is an underrecognized cause of graft dysfunction and late graft loss in pediatric liver transplant (pLT) recipients, often necessitating re‐transplantation. Current management strategies can include high‐dose steroids, intravenous immunoglobulin (IVIG), plasmapheresis (PLEX), monoclonal antibodies, and increased maintenance immunosuppression. This study highlights the diagnostic and management strategies that have prevented graft loss and re‐transplantation at a high‐volume pLT center.

**Methods:** Children (<18 years) transplanted between 1/1998‐12/2023 were reviewed for biopsy‐proven rejection. The diagnosis of AMR was based on clinical and biochemical findings consistent with graft dysfunction and Banff criteria. Diagnostic approach, treatment protocol, and clinical outcomes were reviewed for each patient. Primary outcomes included graft survival versus resolution of rejection, and any long or short‐term complications were reviewed as secondary outcomes.

**Results:** Eighteen children (6 female and 12 male) out of 492 LT were diagnosed with AMR over the study period (3.7%). Ten patients were diagnosed with AMR in the first year post‐LT, at a median of 5.0 months post‐LT. Sixteen patients had previously experienced T‐cell‐mediated rejection and 1 patient had suspected recurrent AMR. Fourteen patients were found to have Class II DSA. Two patients were treated with escalation of maintenance immunosuppression, 15 patients with high‐dose steroids, 9 with a combination therapy of IVIG, ATG, and/or agents like Bortezomib, Rituximab, Eculizumab, Basiliximab, or Alemtuzumab. (Table 1). Thirteen patients had graft stabilization, four required re‐transplantation, one remains under treatment, and two had fatal outcomes—one from decompensated liver disease with disseminated fungal infection, and the other from acute liver failure.

**Conclusion:** This study highlights the potential to salvage graft function in pediatric LT recipients with AMR through aggressive, targeted immunosuppressive therapy. The findings underscore the importance of early recognition with individualized treatment, and that further studies are needed to optimize diagnostic, screening, and therapeutic strategies to improve outcomes.


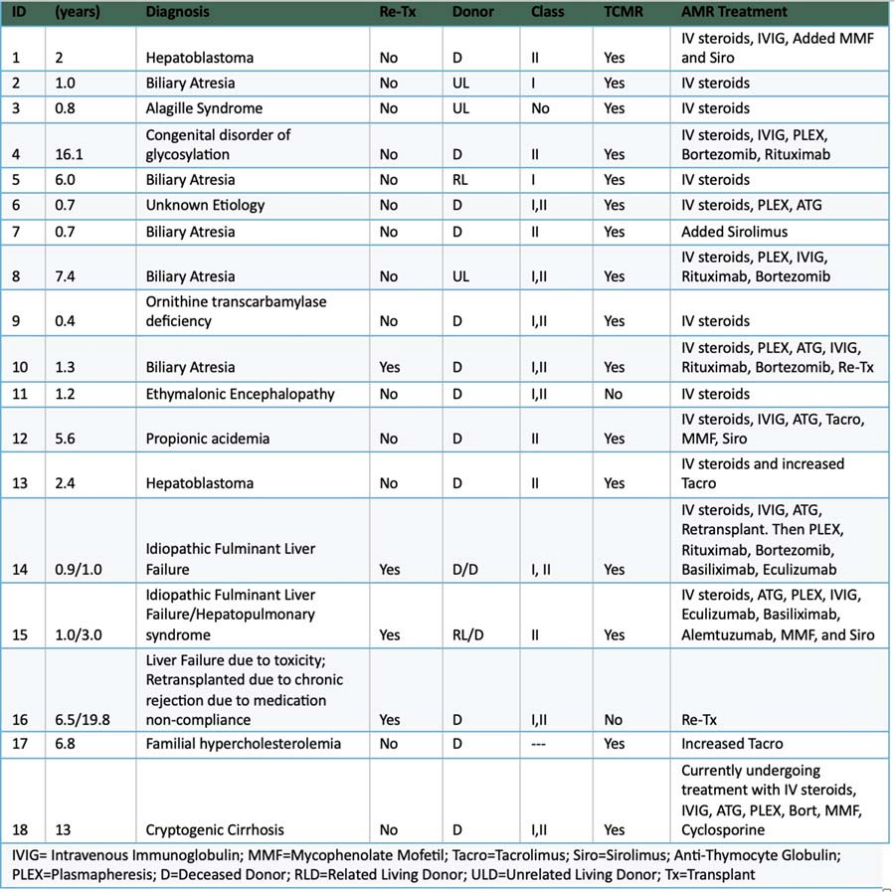


Clinical features of patients diagnosed with antibody mediated rejection

## 401 TITLE: ABCB11 P.GLU137LYS: A NOVEL DELETERIOUS VARIANT FOUND IN A 50‐YEAR‐OLD PARAFFIN LIVER BLOCK OF A PATIENT WITH SUSPECTED PROGRESSIVE FAMILIAL INTRAHEPATIC CHOLESTASIS

*Karis Kosar* ^*1*^, *Chunyue Yin* ^*1*^, *Aitaro Takimoto* ^*1*^, *Ammar Husami* ^*2*^, *Jaroslaw Meller* ^*3*^, *Kevin Bove* ^*4*^, *Wenying Zhang* ^*2*^, *William Balistreri* ^*1*^, *Akihiro Asai* ^*1*^

^*1*^ *Gastroenterology*, *Cincinnati Children's Hospital Medical Center*, *Cincinnati*, *OH*; ^*2*^ *Human Genetics*, *Cincinnati Children's Hospital Medical Center*, *Cincinnati*, *OH*; ^*3*^ *Biomedical Informatics*, *Cincinnati Children's Hospital Medical Center*, *Cincinnati*, *OH*; ^*4*^ *Pathology*, *Cincinnati Children's Hospital Medical Center*, *Cincinnati*, *OH*

**Background:** Approximately 30% of children with isolated/primary cholestasis lack a genetic diagnosis, limiting the ability to tailor treatment and assess prognosis. Advances in sequencing and histopathology have expanded the ability to achieve a precise diagnosis to an ever‐increasing number of patients. Here, we present a 13‐year‐old male who died 50 years ago with an unknown form of low‐GGT cholestasis; this undefined disease also led to the death of his 15‐year‐old male sibling. The tragic and mysterious death of two siblings engraved the family's everlasting fear of hereditary liver disease for the surviving sibling and his children. To establish a genetic diagnosis and its hereditary pattern, we obtained paraffin blocks of the liver collected at autopsy and blood samples from the unaffected mother and the surviving adult sibling.

**Methods:** To identify disease‐causing variants, we employed immunostaining of bile acid transporters, targeted next‐generation sequencing of the family members, and Sanger sequencing of DNA from the paraffin blocks. We used in silico structure modeling to predict the impact of the identified missense variants. To functionally validate the variant's pathogenicity, we introduced the variant into an induced hepatocyte (iHep) system derived from human induced pluripotent stem cells (iPSC) using mRNA lipofection.

**Results:** The patient's liver biopsy revealed canalicular cholestasis, giant cell transformation, and fibrosis. Immunostaining revealed a loss of bile canalicular localization of the bile salt export pump protein (BSEP, coded by *ABCB11*), whereas other transporters, MDR3 and MRP2, were not affected. Through targeted sequencing of 82 cholestasis‐related genes, we identified that his mother is a carrier for a novel BSEP variant, p.Glu137Lys. Sequencing of the patient's DNA confirmed that he was homozygous for this variant. In silico analysis predicted the novel variant to be likely pathogenic. In a Transwell culture system using iPSC, we established a monolayer iHep lacking BSEP by CRISPR genome editing. iHep treated with wild‐type *ABCB11* mRNA showed active transport of bile acids from the lower to the upper chamber of the Transwell, while iHep treated with p.Glu137Lys *ABCB11* mRNA did not. The BSEP‐Glu137Lys protein expression, measured by the HiBiT‐tag system, was diminished compared to WT.

**Conclusion:** This case demonstrates the power of combining DNA extracted from archived tissue with family‐based sequencing to achieve a definitive diagnosis. The identification of a novel homozygous variant, supported by results from histology, immunostaining, and in vitro functional assay, provides strong evidence for pathogenicity and expands the spectrum of PFIC2. Importantly, testing of family members clarified the inheritance pattern and alleviated concerns for future affected individuals. This approach underscores the value of genetic diagnosis in informing family counseling and advancing the understanding of gene function in rare liver diseases.

## 409 GREATER IMPROVEMENTS IN BILIRUBIN WERE OBSERVED IN PRURITUS RESPONDERS AFTER MARALIXIBAT TREATMENT IN PATIENTS WITH PFIC: DATA FROM THE MARCH/MARCH‐ON TRIALS

*Nadia Ovchinsky* ^*1*^, *Richard Thompson* ^*2*^, *Lorenzo D'Antiga* ^*3*^, *Chuan‐Hao Lin* ^*4*^, *Douglas Mogul* ^*5*^, *Tiago Nunes* ^*5*^, *Shannon Vandriel* ^*5*^, *Cheng Chen* ^*5*^, *Joanne Quan* ^*5*^, *Pamela Vig* ^*5*^, *Alexander Miethke* ^*6*^

^*1*^ *New York University Grossman School of Medicine*, *New York*, *NY*; ^*2*^ *Institute of Liver Studies*, *King's College London*, *London*, *England*, *United Kingdom*; ^*3*^ *Pediatric Hepatology, Gastroenterology and Transplantation*, *ASST Papa Giovanni XXIII*, *Bergamo*, *Italy*; ^*4*^ *Children's Hospital Los Angeles*, *Los Angeles*, *CA*; ^*5*^ *Mirum Pharmaceuticals Inc*, *Foster City*, *CA*; ^*6*^ *Cincinnati Children's Hospital Medical Center*, *Cincinnati*, *OH*

**Background:** Progressive familial intrahepatic cholestasis (PFIC) comprises a heterogeneous group of disorders linked to genetic defects in hepatocanalicular transporters. Prior data has demonstrated total bilirubin (TB) is associated with improved transplant‐free survival in this disorder. Maralixibat, an IBAT inhibitor, is approved in the US for the treatment of cholestatic pruritus in individuals ≥12 months old with PFIC, has been shown to reduce TB. Here, we evaluate the relationship between pruritus response and changes in TB in MARCH and MARCH‐ON.

**Methods:** MARCH/MARCH‐ON have been previously described. Changes in TB were evaluated during the first 26 weeks of MRX treatment (average of weeks 18, 22, and 26). Pruritus was assessed with average morning ItchRO(Obs)severity score in week 15‐26 and response was defined as ≥1‐point reduction from baseline or having the average score ≤ 1 point. Differences between pruritus responders and non‐responders were analyzed using Wilcoxon signed‐rank test and Fisher's exact test.

**Results:** Fifty‐nine children were treated with MRX [PFIC1: n=12 (20%); nt‐PFIC2: n=28 (47%); PFIC3: n=9 (15%); PFIC4: n=7 (12%); PFIC6: n=3 (5%)]. Pruritus response was observed in 37 (63%). Pruritus responders had lower Baseline TB compared to non‐responders (3.0 vs. 6.3 mg/dL, *p=*0.006) and had greater reductions in TB following treatment (‐1.4 vs. ‐0.6 mg/dL, *p*=0.048). Normalization of TB occurred more frequently in pruritus responders (*p*<0.01).

**Conclusions:** Changes in bilirubin and pruritus are linked in patients on MRX with greater reductions in bilirubin, an important marker of liver health, being observed in pruritus responders. People treated earlier in the course of disease (i.e., lower bilirubin) may have greater likelihood of improvement in pruritus.

## 411 CHARACTERIZATION OF PRODROMAL EVENTS PRECEDING THE DIAGNOSIS OF PEDIATRIC AUTOIMMUNE HEPATITIS‐ A RETROSPECTIVE STUDY

*Forum Patel* ^*1*^, *Liyun Zhang* ^*2*^, *Ke Yan* ^*2*^, *Bernadette Vitola* ^*1*^, *Cara Mack* ^*1*^

^*1*^ *Pediatric Gastroenterology*, *Medical College of Wisconsin*, *Milwaukee*, *WI*; ^*2*^ *Medical College of Wisconsin*, *Milwaukee*, *WI*

**Background:** Pediatric autoimmune hepatitis (AIH) is a chronic progressive liver disease of multifactorial etiology characterized by immune‐mediated hepatocellular inflammation, often leading to end‐stage liver disease if untreated. While effective treatment exists, early diagnosis is often difficult due to nonspecific presenting symptoms. Timely recognition is critical to improve outcomes. Identification of specific prodromal symptoms preceding the diagnosis of AIH may enable earlier recognition and intervention. The objective of this study is to identify and characterize prodromal events associated with the diagnosis of AIH and determine the correlation of prodromal events with outcomes.

**Methods:** Retrospective chart review of patients <18 years of age diagnosed with AIH at a tertiary pediatric center was conducted between the years of 2009 and 2024 using the Epic electronic health record. Exclusion criteria included chronic liver disease such as Wilson disease, hereditary hemochromatosis, hepatitis B/C, alpha‐1 antitrypsin deficiency and de novo AIH in liver transplant patients. Collected data included demographics, presenting symptoms and viral testing, examination findings, laboratory results (at presentation and follow‐up time points), liver histology, and treatment medications. Prodromal symptoms were categorized into three domains: gastrointestinal (GI), systemic, and upper respiratory (URI) and occurring within four weeks of initial presentation of AIH evaluation. Data were de‐identified, entered in REDCap and analyzed using descriptive and comparative statistics. Statistical significance was defined as p<0.05.

**Results:** A total of 95 patients were identified: mean age at presentation 12.5 ± 4 years, median time from presentation to diagnosis 35 days, 64% female, 75% White, 77% non‐Hispanic, 82% AIH type 1. At least one prodromal symptom was identified in 61% (n=58) of patients. No significant differences in age, sex, race/ethnicity, insurance status, or hepatotoxic medication were identified between patients with or without prodromal symptoms. Among symptomatic patients, GI symptoms (abdominal pain, vomiting, diarrhea) were most common (48%), followed by systemic symptoms (fatigue, fever) (23%) and URI symptoms (congestion, cough, rhinorrhea) (16%). All patients received steroids; 73% received azathioprine. At 6 months after treatment initiation, biochemical remission based on ALT normalization (<40 U/L) was acheived in 36% patients and was higher in patients with prodromal symptoms (50% remission) compared to without symptoms (21%), p=0.005. Additional analyses of physical signs at presentation showed that jaundice was present in 25% (n=24) of patients. Among these, 95% (n=23) reported at least one prodromal symptom (p<0.0001). In patients presenting with jaundice, the time to prednisone induction was shorter compared to those without jaundice p =0.001.

**Conclusion:** In this single‐center retrospective study, 61% of pediatric AIH patients had at least one prodromal symptom prior to diagnosis, with GI symptoms being the most frequently observed. The presence of prodromal symptoms—particularly in patients with concurrent jaundice—was associated with a shorter time to diagnosis and higher rate of biochemical remission at 6 months. Additionally, among patients with both prodromal symptoms and jaundice, the time from presentation to initiation of prednisone induction was significantly shorter compared to patients with neither symptoms nor jaundice. This likely reflects expedited medical evaluation due to the presence of overt clinical signs. Ultimately, identification of early AIH symptoms may lead to more timely diagnosis, treatment and improved long‐term outcomes.


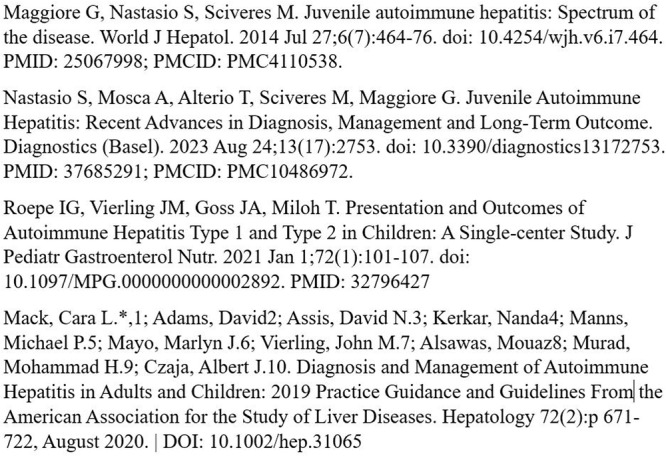


## 412 REAL‐WORLD ECONOMIC BURDEN OF BILIARY ATRESIA IN INFANTS UNDER 1 YEAR OF AGE: A US CLAIMS‐BASED ANALYSIS

*Tebyan Rabbani* ^*1*^, *Sonia Lee* ^*2*^, *Dannielle Lebovitch* ^*3*^, *Colin Navickas* ^*3*^, *Elizabeth Nagelhout* ^*3*^, *Nisreen Shamseddine* ^*2*^, *Cara Mack* ^*4*^

^*1*^ *Pediatric Gastroenterology, Hepatology, & Nutrition, Stanford University*, *Stanford*, *CA*; ^*2*^ *Ipsen*, *Cambridge*, *MA*; ^*3*^ *Genesis Research Group*, *Hoboken*, *NJ*; ^*4*^ *Pediatric Gastroenterology, Hepatology, & Nutrition, Children's Wisconsin, Medical College of Wisconsin*, *Milwaukee*, *WI*

**Background:** Biliary atresia (BA) is a rare, progressive pediatric liver disease requiring early intervention to prevent irreversible liver damage. Without timely Kasai portoenterostomy (KP), many patients progress to liver failure, with BA accounting for approximately one‐third of pediatric liver transplantations (LT) in the US.

**Objective:** This study evaluated the healthcare resource utilization (HCRU) and direct medical costs associated with BA in commercially insured infants in the US who were under 1 year of age and received KP.

**Methods:** A retrospective cohort analysis was conducted using Merative™ MarketScan® Commercial Database data from 2016 to 2023. Infants with a first observed diagnostic claim of BA (index date) during their birth year and a claim for KP were included. LT rates and time‐to‐procedure intervals were assessed using unrestricted follow‐up. HCRU and cost outcomes were evaluated descriptively per patient per year (PPPY) within the 12‐month post‐index period.

**Results:** Among 72 infants (53% female), a total of 21 (29%) progressed to LT. The median follow‐up was 1.24 years (interquartile range [IQR]: 0.42–3.20) for the KP‐only cohort and 2.02 years (IQR: 1.56–3.79) for those who received both KP and LT (KP + LT cohort). Median time from index to KP among infants who underwent KP only (n=51) was 2 days (IQR: 0–7). Among infants who underwent both KP + LT (n=21), the median time from index to KP was 0 days (IQR: 0.0–1.0) and time from KP to LT was 274 days (IQR: 149–367). Compared with the KP‐only cohort, infants in the KP + LT cohort experienced higher mean numbers of inpatient admissions (3.05 vs 1.73 PPPY) and outpatient visits (64.71 vs 21.94) during the 12 months post‐index. The KP + LT cohort also demonstrated modestly higher intensive care unit (1.14 vs 0.45) and emergency room (ER) utilization (2.00 vs 1.04). Median total all‐cause costs PPPY were $123,264 (IQR: $49,184–$248,297) for the KP‐only cohort and $412,820 (IQR: $101,344–$739,121) for the KP + LT cohort. Median inpatient costs per visit were $61,655 (KP‐only) and $100,571 (KP + LT). Outpatient and ER per‐visit costs were also elevated in the KP + LT cohort.

**Conclusions:** This study highlights the substantial burden of care among infants with BA, greatest among those with suboptimal outcomes post‐KP leading to LT, and underscores the unmet need for disease‐modifying therapies. Additionally, persistently high costs driven by hospitalizations suggest that coordinated care pathways and targeted interventions may improve patient outcomes and reduce economic impact.

## 414 IGG AS A SERUM BIOMARKER FOR HISTOLOGICAL ACTIVITY AND FIBROSIS IN CHILDREN WITH AUTOIMMUNE HEPATITIS AT DIAGNOSIS

*Yunuen Rivera‐Suazo* ^*1*^, *Jaime Ernesto Alfaro‐Bolaños* ^*1*^, *Juan Suárez‐Cuenca* ^*2,3*^, *Carlos Ricardo Flores‐Soriano* ^*3*^

^*1*^ *Pediatric Gastroenterology*, *Centro Medico Nacional 20 de Noviembre*, *Mexico City*, *Mexico City*, *Mexico*; ^*2*^ *Research*, *Centro Medico Nacional 20 de Noviembre*, *Mexico City*, *Mexico City*, *Mexico*; ^*3*^ *Pediatrics*, *Centro Medico Nacional 20 de Noviembre*, *Mexico City*, *Mexico City*, *Mexico*

**Background:** Autoimmune hepatitis (AIH) stands as a rare, necro‐inflammatory, progressive, immune‐mediated liver disease of unknown etiology, characterized by specific circulating autoantibodies, elevated IgG concentrations and particular histopathology.

Liver biopsy is needed to confirm diagnosis and to evaluate the severity of liver damage. Current serum biomarkers of liver injury in AIH that are used in practice include aminotransferases (AST and ALT) and IgG. There is a relative paucity of data about the role of IgG in histological activity and fibrosis in children with AIH.

We aim to establish an IgG cohort cut‐off with the highest performance and correlation with necro‐inflammatory activity and fibrosis in children with AIH.

**Methods:** *Study design*. Data were obtained by retrospective medical records review using K75.4 code for IAH in a tertiary center in Mexico City over a decade (2014‐2024).

*Inclusion criteria*. AIH was confirmed according to ESPGHAN 2018 proposed scoring criteria (>7: probable; >8: definite).

*Exclusion criteria*. Charts with missing information and biopsies not available for evaluation (assessment for same pediatric pathologist).

*Data collection*. Demographic, clinical, laboratory, serological and histological features were collected.

*Statistical analysis*. An analytical cross‐sectional study was conducted. Results are expressed as numbers (%) or mean (+ SD). P values <0.05 were considered statistically significant. Scheuer scoring system was used to evaluate necro‐inflammatory activity and histological changes. A score of 0 indicated absence of necroinflammation/fibrosis, 1‐2 represented mild necroinflammation/fibrosis, and 3‐4 severe necroinflammation/fibrosis. A 5‐point ROC curve was used to establish IgG cohort cut‐off with the highest performance and correlation with necro‐inflammatory activity and fibrosis. IgG values were those reported 6 months prior to biopsy.

**Results:** There were 29 patients that met AIH criteria (Table 1). A total of 16 native liver biopsies from 16 patients were eligible for evaluation. Sensitivity was 87% and specificity was 62% for necro‐inflammatory activity, with a negative predictive value (NPV) of 83% in the cohort with IgG levels of 2000 mg/dL. Regarding fibrosis, sensitivity was 75% and specificity 90%, with an NPV of 90% in the cohort with IgG levels of 3000 mg/dL (Table 2).

**Conclusions:** IgG elevated level is highly associated with histological activity in pediatric patients with AIH. These findings suggest that IgG concentrations could serve as a marker for assessing disease activity and progression and should be assessed serially in routine follow‐up. However, IgG should not be considered a standalone diagnostic criterion but rather used in conjunction with other clinical and histological biomarkers.

**Contact:** rivera.suazo@outlook.com


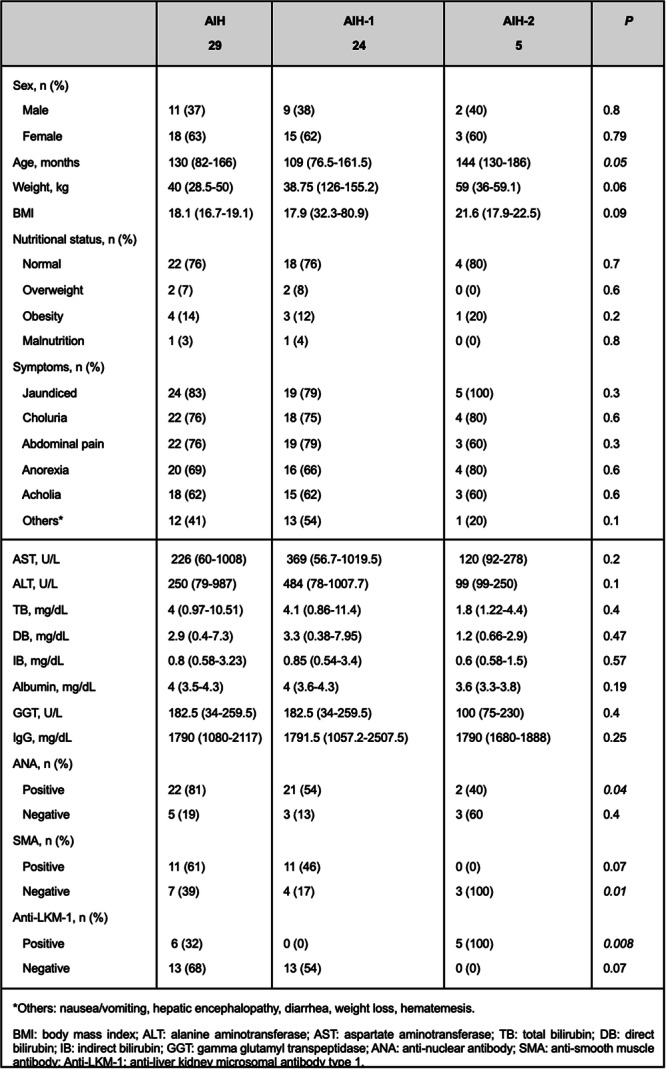


**Table 1. Demographic characteristics of autoimmune hepatitis in the study population:**


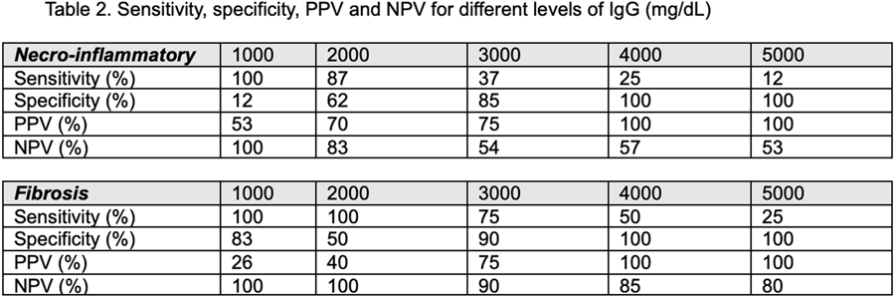


**Table 2. Sensitivity, specificity, PPV and NPV for different levels of IgG (mg/dL)**

## 415 ULTRASOUND ASSISTED PERCUTANEOUS LIVER BIOPSY IN PEDIATRIC PATIENTS A SINGLE CENTER EXPERIENCE

*Maria Rogalidou* ^*1,6*^, *Aglaia Zellos* ^*1*^, *Konstantina Dimakou* ^*3,6*^, *Vasiliki‐ Maria Karagianni* ^*1,6*^, *Eleanna Stasinou* ^*1,6*^, *Dafne Margoni* ^*1,6*^, *Kalliopi Stefanaki* ^*4*^, *Paraskevi Galina* ^*5*^, *David Oikonomopoulos* ^*2*^, *Alexandra Papadopoulou* ^*1,3*^

^*1*^ *1st Pediatrics Dep, Division of Gastroenterology & Hepatology*, *Ethniko kai Kapodistriako Panepistemio Athenon*, *Athens*, *Attica*, *Greece*; ^*2*^ *Anesthesiology Department*, *Nosokomeio Paidon e Agia Sophia*, *Athens*, *Attica*, *Greece*; ^*3*^ *Gastroenterology Department*, *Agia Sofia Children's Hospital*, *ATHENS*, *Type a choice below* …, *Greece*; ^*4*^ *Pathology Deaprtment*, *Nosokomeio Paidon e Agia Sophia*, *Athens*, *Attica*, *Greece*; ^*5*^ *Radiology Department*, *Nosokomeio Paidon e Agia Sophia*, *Athens*, *Attica*, *Greece*; ^*6*^ *Nosokomeio Paidon e Agia Sophia*, *Athens*, *Attica*, *Greece*

**Introduction:** Liver biopsy is the gold standard for diagnosing and staging liver diseases. The image‐guided technique involves using ultrasound (US) to mark the biopsy site before needle insertion.

**The aim** of this study was to retrospectively assess the safety and efficacy of ultrasound assisted percutaneous liver biopsy in pediatric patients at our center between 01/01/2018‐31/03/2025.

**Procedure Protocol:** Biopsies were obtained under minimal sedation. The patient is positioned supine on the surgical bed, with right arm, placed above the head, to best allow for intercostal space expansion. After palpation, and percussion ultrasound confirm the appropriateness of the site and tract of the biopsy. The skin is then prepped and draped in a sterile fashion. Lidocaine solution (1% or 2% solution are options) is injected along the upper border of the rib followed by a small incision with a surgical blade. A 18 G fine biopsy needle was used for all patients. After removing the needle, pressure is applied to the biopsy site for a few minutes, and a bandage is applied. The patient is then placed in the right lateral decubitus position. Following the procedure, the patient's blood pressure, heart rate, and pain level are measured every 15 minutes for the first hour, 30 minutes for the next hour, and every hour until the first haemoglobin value is obtained. Post biopsy, all patients were admitted for a 24‐hour observation to monitor for complications. Haemoglobin and Hematocrit levels were obtained at 4 h and 24 h after biopsy. Histology was reviewed by pathologists, including length (cm) and complete portal tract (CPT) number.

**Results:** 57 patients (29 female, 50.8%) underwent ultrasound‐assisted liver biopsy. The mean age of the patients was 8.7 years (1‐208 months, with a mean of 104.5 months). The haemoglobin/Hematocrit levels of patients before procedure, 4 hours and 24 hours after biopsy appear in Table 1.

None of the patients experienced adverse events or bleeding, as evidenced by normal pre‐ and post‐haemoglobin/Hematocrit Levels. Histology revealed liver tissue with a length of 1,12 cm (0.5‐2 cm) and complete portal tract (CPT) 9 CPT (between 4‐5 to 15‐17 CPT).

**Conclusion:** Ultrasound‐assisted liver biopsy was well tolerated and yielded samples that were technically and diagnostically successful in a pediatric population examined. Ultrasound‐assisted liver biopsy is safe and effective in the management of pediatric patients for whom a liver biopsy is indicated.


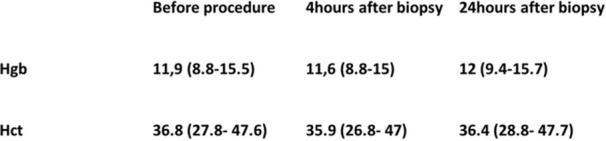


## 418 HYALURONAN FRAGMENTATION PATTERNS IN PEDIATRIC METABOLIC DYSFUNCTION ASSOCIATED STEATOTIC LIVER DISEASE ARE UNIQUE WHEN COMPARED TO ADULTS AND HEALTHY CONTROLS

*Voytek Slowik* ^*1*^, *Dorothea Erxleben* ^*2*^, *Adam Hall* ^*2*^, *Michele Pritchard* ^*3*^

^*1*^ *Pediatrics*, *Children's Mercy ‐ Kansas City*, *Kansas City*, *MO*; ^*2*^ *School of Biomedical Engineering and Sciences*, *Wake Forest University School of Medicine*, *Winston‐Salem*, *NC*; ^*3*^ *Department of Pharmacology, Toxicology and Therapeutics*, *The University of Kansas Medical Center*, *Kansas City*, *KS*

Metabolic dysfunction‐associated liver disease (MASLD) is a leading cause of liver disease in children and adults. However, there is a lack of predictive biomarkers and therapeutic targets, especially in pediatric MASLD. Hyaluronan (HA) is an important component of the extracellular matrix and total plasma HA content increases in adults with liver disease, including MASLD. Independent of its role as an adult liver disease biomarker, HA exhibits molecular weight (MW)‐dependent biological functions in animals and cell culture systems where high MW HA promotes tissue homeostasis and low MW HA drives inflammation. How plasma HA content and MW distribution differs in adult or pediatric MASLD (AM, PM) patients compared to healthy controls (AC, PC) is not known. To fill this knowledge gap, we measured total plasma HA using an ELISA‐like assay and then determined its MW distribution using solid‐state nanopore technology in AM (n = 8), AC (n = 6), PM (n = 7), and PC (n = 7). MW distribution data were then analyzed for correlation with disease state. Using the cumulative distribution curves for each specimen, a nonparametric Kruskal‐Wallis test was performed to evaluate global differences, followed by Fisher's Protected Least Significant Difference test to compare data between groups. These analyses showed that the distribution for PM was significantly different (p < 0.05) from that of both AM and AC in the MW range of 315 ‐ 658 kDa and from that of PC in the range of 379 ‐ 548 kDa. Moments of the MW distributions (mean, variance, skewness, and kurtosis) were also considered for each group. Results showed a significant difference (p < 0.05) in variance between the PM cohort and both the AM and AC groups, suggesting that pediatric MASLD HA MW distributions are more spread across the observable size range compared to the other groups. Our results demonstrate that, despite no increase in total plasma HA content, pediatric patients with MASLD have unique HA MW distributions when compared to healthy children and adults with and without MASLD. Future studies will be needed to determine if variance patterns of HA MW can be used as a reliable predictive biomarker or therapeutic target in pediatric MASLD.

## 419 EVALUATING THE IMPACT OF WEIGHT LOSS INTERVENTIONS ON METABOLIC DYSFUNCTION‐ASSOCIATED STEATOTIC LIVER DISEASE IN PEDIATRIC PATIENTS: A PRELIMINERAY ANALYSIS USING TRANSIENT ELASTOGRAPHY

*Danielle Statman* ^*4*^, *Stephan Myers* ^*5,3*^, *Ellen Mitchell* ^*1,2*^

^*1*^ *Pediatric Gastroenterology*, *St Christopher's Hospital for Children*, *Philadelphia*, *PA*; ^*2*^ *Gastroenterology*, *Drexel University*, *Philadelphia*, *PA*; ^*3*^ *Surgery*, *Drexel University*, *Philadelphia*, *PA*; ^*4*^ *Pediatrics*, *St Christopher's Hospital for Children*, *Philadelphia*, *PA*; ^*5*^ *Pediatric Surgery*, *St Christopher's Hospital for Children*, *Philadelphia*, *PA*

**Background/Introduction:** Due to its strong association with obesity nonalcoholic fatty liver disease (NAFLD), newly renamed as metabolic dysfunction‐associated steatotic liver disease (MASLD), has become the most common liver disease in children in the United States. MASLD can lead to progressive fibrosis and, in some cases, end‐stage liver disease making it one of the leading indications for liver transplantation in adults over the past decade. Despite its clinical significance, diagnosing MASLD in children remains a challenge. The current gold standard for diagnosing MASLD is a liver biopsy however there are always risks with an invasive procedure. Traditional ultrasound is only good at detecting severe steatosis in children but can miss mild to moderate cases. Transient elastography (TE), a non‐invasive ultrasound‐guided imaging technique, measures liver stiffness and fat deposition and is being used more commonly as a tool for screening and longitudinal assessment of MASLD. TE is increasingly being used in adult populations and is gaining traction in pediatric practice. However, more research is needed to validate its utility, accessibility, and accuracy in children and adolescents with MASLD.

**Objective/Purpose:** This study aims to assess the utility of TE in detecting and monitoring MASLD in obese pediatric patients undergoing weight loss interventions, including lifestyle changes, pharmacologic therapies (e.g., GLP‐1 agonists, Metformin, Topiramate) and bariatric surgery. The secondary aim is to assess whether liver stiffness, laboratory markers of liver function, cholesterol levels, hemoglobin A1c, and BMI improve following these interventions.

**Methods:** This is a prospective study designed to collect data over a three‐year period. All patients seen in the Pediatric Gastroenterology Office at St. Christopher's Hospital for Children with a BMI above the 95th percentile, age 12‐21, and speak English or Spanish, will be considered for recruitment to the study. As part of standard clinical care, all children with obesity will undergo screening blood work (alanine aminotransferase or ALT, lipid panel, hemoglobin A1C). Patients will be recruited to the study if ALT is twice the upper limit of normal (ALT > 52 for boys and > 44 for girls) indicating a diagnosis of MASLD. Those with other chronic liver diseases, significant comorbid conditions, use of medications that affect liver function, or have already started weight loss medications without obtaining a TE scan will be excluded.

Upon consent, TE scan will be ordered (if not already obtained) and patient will complete a lifestyle/diet survey. Based on standard of care practices, the provider and patient will decide if they will continue diet and exercise, start medication or enroll on the surgical track. They will receive regular follow‐up examinations at intervals recommended by the provider and all data will be collected. The goal is to obtain TE, standard blood work and survey annually.

**Preliminary Data/Current Progress**

The study is currently in the recruitment and consent phase. Consent started December 2024 and currently 15 patients are enrolled and ongoing (8 = lifestyle, 6 = medication, 1 = bariatric surgery). Among those who have completed TE, liver stiffness values have varied with corresponding ALT. Repeat TE scans have not yet been obtained, as insufficient time has passed since baseline measurements.

**Anticipated Impact/Future Direction**

As pediatric obesity rates continue to rise, so does the prevalence of MASLD, creating a critical need for effective diagnostic and management strategies. The American Academy of Pediatrics (AAP) now recommends weight loss medications as adjunct to lifestyle changes for adolescents over 12, and the American Society for Metabolic and Bariatric Surgery supports bariatric surgery for those with severe obesity, especially with comorbid conditions. Large studies like the “STEP TEENS STUDY” for use of GLP‐1 and “Teen‐LABS study” for bariatric surgery have shown that these interventions reduce BMI and improve hypertension, dyslipidemia and type 2 diabetes, but their impact on MASLD remains unclear.

With more pediatric patients being prescribed weight loss medications and undergoing bariatric surgery, it is increasingly important to understand how these interventions impact liver health. The findings of this study could help guide clinical decision‐making and support broader use of TE in pediatric care. The regular use of TE to monitor liver fibrosis may help prevent the serious complications of end‐stage liver disease by enabling earlier detection and treatment.

***Declaration of Generative AI and AI‐assisted technologies in the writing process***

*During the preparation of this work the author used Chat GPT in order to* organize content and refine language for clarity and coherence*. After using this tool, the author reviewed and edited the content as needed and takes full responsibility for the content of the publication*.

## 424 IMAGING COMPARED TO BIOPSY FOR DIAGNOSIS OF PEDIATRIC LIVER TUMORS

*Andrea Wright* ^*1*^, *Boaz Karmazyn* ^*2*^, *Kyla Tolliver* ^*3*^, *Jean Molleston* ^*3*^, *Chao Jarasvaraparn* ^*3*^

^*1*^ *Student*, *Indiana University School of Medicine*, *Indianapolis*, *IN*; ^*2*^ *Pediatric Radiology*, *Indiana University School of Medicine*, *Indianapolis*, *IN*; ^*3*^ *Pediatric Gastroenterology, Hepatology and Nutrition*, *Indiana University School of Medicine*, *Indianapolis*, *IN*

**Objectives:** Pediatric liver tumors are challenging to diagnose due to their varied clinical presentations. Traditionally, diagnosis is made through imaging and tissue sampling; however, technological advancements may reduce the need for tissue sampling through improved imaging accuracy. In this study, imaging reports were compared to biopsy reports to assess diagnostic accuracy.

**Methods:** This retrospective study included patients under 18 with liver tumors who underwent imaging prior to biopsy. We used the radiology informatics system from January 2007 to August 2024. Liver imaging reports (ultrasound, CT, MRI) were collected from reports to assess discrepant diagnosis of imaging using pathology as the gold standard.

**Results:** Among 50 patients, imaging and biopsy findings matched in 40 cases (80%, 40/50). Of these, 22 were benign and 18 were malignant. In 10 (20%, 10/50) discrepant cases, the most frequent misclassification (14%, 7/50) involved imaging diagnosis of malignancy that turned out to be benign on pathology. In three (6%, 3/50) cases, imaging suggested a benign lesion; hepatocellular adenoma, regenerative nodule and indeterminate benign lesion while biopsy revealed neuroendocrine tumor, hepatocellular carcinoma, and hepatoblastoma, respectively. Of the 40 cases with matched benign and malignant category of tumors in pathology by imaging and pathology, in 3 cases the specific type of tumor diagnosed by imaging was different than the type of tumor found in pathology. MRI was the most accurate modality matching biopsy in 82% of cases.

**Conclusions:** Imaging and biopsy diagnoses differed in tumor classification (benign vs malignant) in 20% of cases. MRI showed the highest concordance with biopsy, reinforcing its value in complex diagnoses. The most clinically significant discrepancy occurred in 3 cases (6%), where imaging suggested a benign lesion without specific diagnostic features, but pathology confirmed malignancy. These findings underscore the need for standardized imaging criteria for liver tumors and highlight the importance of follow‐up or biopsy in cases where imaging suggests a benign diagnosis without characteristic features.


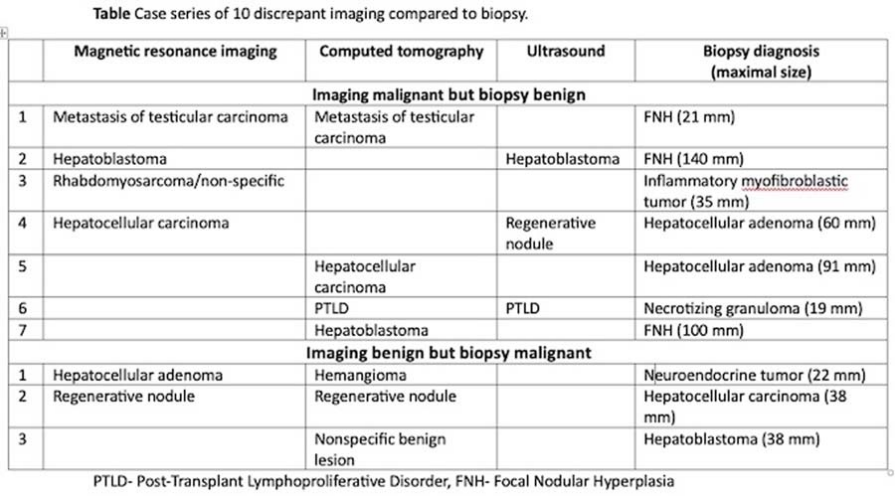


## 428 EFFECTIVENESS OF SURGICAL SHUNTS IN PEDIATRIC PATIENTS WITH NON‐CIRRHOTIC PORTAL HYPERTENSION: A 10‐YEAR EXPERIENCE AT A TERTIARY CENTER

*Arturo Cantu Kawas*, *Dr. José Francisco Cadena León*, *Roberto Cervantes Bustamante*, *Karen Ignorosa‐Arellano*, *Flora Zarate‐Mondragon*, *Erick Toro‐Monjaraz*, *Ericka Montijo‐Barrios*, *Martha Martínez Soto*, *Jaime Ramirez‐Mayans*

*Pediatric Gastroenterology and Nutrition*, *Instituto Nacional de Pediatria*, *Mexico City*, *CDMX*, *Mexico*

**Introduction:** Portosystemic shunts and mesorex are surgical procedures frequently used in pediatric patients with prehepatic portal hypertension, mainly indicated as secondary prophylaxis for variceal bleeding and for management of hypersplenism. It should be considered that a patent and therefore functional shunt is expected to significantly reduce variceal bleeding episodes, complications associated with hypersplenism, and overall morbidity and mortality in this population. The mesorex shunt (MRS) is recognized as the most physiological and has been associated with greater outcomes when compared to other commonly used portosystemic shunts, such as distal splenorenal (DSRS) and mesocaval (MCS). However, the effectiveness of these procedures is largely dependent on institutional experience and appropriate patient selection, underscoring the need for studies that compare shunt outcomes across different settings.

**Methods:** We conducted a retrospective, observational study of pediatric patients (0‐18 years) who underwent diagnostic and therapeutic evaluation at the Nacional Institute of Pediatrics between 2013‐2023. Al patients had a diagnosis of prehepatic or presinusoidal portal hypertension confirmed by clinical features (variceal bleeding, splenomegaly), biochemical findings (cytopenia's and normal liver function tests), radiological images (splenomegaly, portosystemic shunts, portal thrombosis o cavernous transformation) and histology (normal liver architecture or portal fibrosis with preserved acinus). Included patients underwent one of the following surgical procedures: distal splenorenal shunt (DSRS), mesocaval shunt (MCS) or mesorex shunt (MRS). To evaluate episodes of variceal bleeding, we assess endoscopic findings and presence of bleeding recurrence. Hypersplenism resolution was measured based on spleen size and presence of cytopenia's. These variables were assessed before surgery and during 12‐month follow‐up period. Data was obtained from electronic medical records, entered an Excel database, and analyzed using SPSS v21.

**Results:** A total of 112 patient records with portal hypertension were reviewed: 45.5% (n=51) had prehepatic portal hypertension (47 with cavernous transformation of the portal vein and 4 with portal vein thrombosis), and 12.5% (n=14) had presinusoidal intrahepatic portal hypertension (10 with hepatoportal sclerosis and 4 with congenital hepatic fibrosis). 32 patients met full criteria (28 with prehepatic and 4 with presinusoidal) and underwent the following surgical shunt procedures: 75% (n=24) underwent DSRS, 15.6% (n=5) MRS, and 9.4% (n=3) MCS. In 21.4% (n=6) of patients a secondary intervention was required: mesocaval 14.3% (n=4), distal splenorenal in 3.6% (n=1) and thrombectomy in 3.6% (n=1). Outcomes for the four patients with hepatoportal sclerosis were described separately, excluding 2 DSRS and 2 MCS cases from the main analysis. Chi‐square test were used for categorical variables. Fisher exact test revealed no statistically significant associations between shunt type and recurrence of variceal bleeding (p=1.3) or hypersplenism resolution (p=0.62). However, a trend in decreasing recurrence of bleeding was observed, with a recurrence of 22.7% in DSRS and 40% in MRS. A repeated‐measures ANOVA were also performed for hematologic parameters where results will be discussed in detail during presentation.

**Conclusion:** In this study of pediatric patients with prehepatic portal hypertension, no statistically significant differences were found in resolution of hypersplenism and recurrence of bleeding. However a trend to lower recurrence of variceal bleeding after surgical shunts was noted. The small sample size was a limiting factor, highlighting the need for further studies with larger cohorts to obtain more definitive conclusions.

## 430 EFFICACY OF ETRASIMOD AS FIRST‐LINE ADVANCED TREATMENT FOLLOWING FAILURE OF AMINOSALICYLATES ONLY: DATA FROM THE ELEVATE UC 52 PHASE 3 CLINICAL TRIAL

*David Rubin* ^*1*^, *Charlie Lees* ^*2,3*^, *Filip Baert* ^*4*^, *Maria Kudela* ^*5*^, *Abhishek Bhattacharjee* ^*6*^, *Krisztina Lazin* ^*7*^, *Martina Goetsch* ^*7*^, *Arcangelo Abbatemarco* ^*8*^, *Karolina Wosik* ^*9*^, *John Marshall* ^*10*^

^*1*^ *University of Chicago Medicine Inflammatory Bowel Disease Center*, *Chicago*, *IL*; ^*2*^ *University of Edinburgh*, *Edinburgh*, *United Kingdom*; ^*3*^ *Western General Hospital, NHS Lothian*, *Edinburgh*, *United Kingdom*; ^*4*^ *AZ Delta*, *Roeselare*, *Belgium*; ^*5*^ *Pfizer Inc*, *Cambridge*, *MA*; ^*6*^ *Pfizer Healthcare India Private Ltd*, *Chennai*, *India*; ^*7*^ *Pfizer AG*, *Zürich*, *Switzerland*; ^*8*^ *Pfizer Inc*, *New York*, *NY*; ^*9*^ *Pfizer Canada*, *Kirkland*, *QC*, *Canada*; ^*10*^ *Farncombe Family Digestive Health Research Institute, McMaster University*, *Hamilton*, *ON*, *Canada*

**Introduction:** Etrasimod is an oral, once‐daily, selective sphingosine 1‐phosphate (S1P)_1,4,5_ receptor modulator for the treatment of moderately to severely active ulcerative colitis (UC). In patients with moderately to severely active UC, treatment guidelines recommend early use of advanced therapies rather than gradual step up after failure of 5‐aminosalicylate (5‐ASA).^1^

**Methods:** This prespecified subgroup analysis assessed the efficacy of etrasimod vs placebo in patients in ELEVATE UC 52 (NCT03945188) with, and without, prior oral 5‐ASA failure only (defined as inadequate response, loss of response, or intolerance to previous treatment with oral 5‐ASA but not to any other previous UC medication). Efficacy endpoints assessed at Weeks 12 and 52 included clinical remission, endoscopic improvement, symptomatic remission, and composite histological endpoints.

**Results:** In total, 45 and 29 patients receiving etrasimod and placebo, respectively, had prior 5‐ASA failure only, and 244 and 115 patients did not. At Weeks 12 and 52, among those who had failed prior 5‐ASA only, significantly more patients receiving etrasimod vs placebo achieved clinical remission (Week 12, % difference [Δ]: 38.3%, *p < *0.001; Week 52, Δ: 45.0%, *p < *0.001), endoscopic improvement (Week 12, Δ: 54.7%, *p < *0.001; Week 52, Δ: 47.8%, *p < *0.001), symptomatic remission (Week 12, Δ: 38.6%, *p < *0.001; Week 52, Δ: 45.0%, *p < *0.001), and endoscopic improvement‐histologic remission (Week 12, Δ: 35.6%, *p < *0.001; Week 52, Δ: 31.1%, *p < *0.001; Table). Significantly more patients receiving etrasimod vs placebo in this subgroup achieved endoscopic normalization‐histologic remission (Week 12, Δ: 19.9%, *p = *0.002; Week 52, Δ: 24.3%, *p < *0.001) and disease clearance (Week 12, Δ: 15.6%, *p = *0.008; Week 52, Δ: 27.8%, *p < *0.001). Among those who had failed other therapies, significantly more patients receiving etrasimod vs placebo also achieved all endpoints at Weeks 12 and 52 (*p* < 0.01).

**Conclusion:** Etrasimod demonstrated robust efficacy as first‐line advanced therapy in patients who had failed oral 5‐ASA only, across all evaluated endpoints including stringent composite histological endpoints, consistent with the overall ELEVATE UC population. Etrasimod shows efficacy when used early in the UC treatment algorithm.

**Declaration of Generative AI and AI‐assisted technologies in the writing process:** During the preparation of this work the authors used Pfizer's generative artificial intelligence tool MAIA to assist production of the abstract first draft. After using this tool, the authors reviewed and edited the content as needed and take full responsibility for the content of the publication.

**Reference:**

1. Singh S et al. Gastroenterology 2024; 167: 1307–1343.


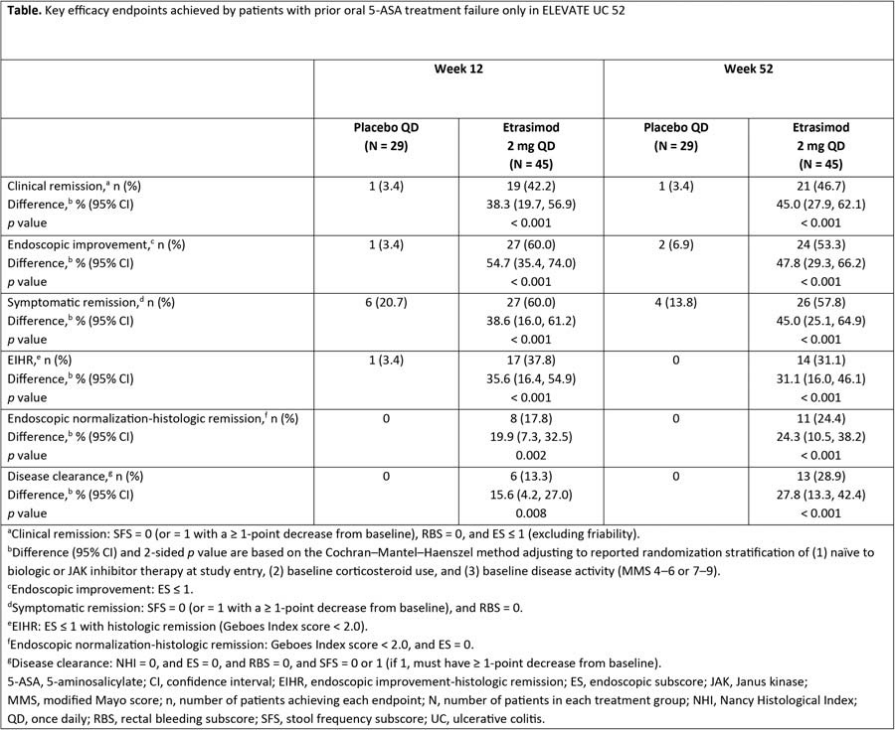


## 432 COMPUTED TOMOGRAPHY BASED EVALUATION OF MUSCLE DENSITY IN PEDIATRIC INFLAMMATORY BOWEL DISEASE: A SINGLE‐CENTER EXPERIENCE

*Sharef Al‐Mulaabed*, *Justin Baba*, *Jeffrey Bornstein*, *Akash Pandey*

*Center for Digestive Health and Nutrition*, *Orlando Health Arnold Palmer Hospital for Children*, *Orlando*, *FL*

**Background and Aim:** Inflammatory bowel disease (IBD), including Crohn's disease, ulcerative colitis, and indeterminate colitis, is a chronic condition that can significantly impact nutrition and growth in pediatric patients. Beyond gastrointestinal symptoms, IBD is associated with systemic complications such as osteopenia and sarcopenia. Currently, there are no universally established reference values for computed tomography (CT) based muscle density to define sarcopenia in children. The study aims to evaluate muscle density in pediatric IBD patients using routinely acquired CT scans, comparing findings to age and sex matched healthy controls. By assessing muscle mass through existing imaging, we explore a potentially cost‐effective approach to musculoskeletal evaluation in this high‐risk population.

**Methods:** We conducted a retrospective review of electronic medical records and CT scans from pediatric IBD patients (below 21 years old) followed at Arnold Palmer Hospital for Children (Orlando, FL), between January 2020 and May 2025. We selected age and sex matched controls from children and adolescents who presented with acute appendicitis and underwent a CT scan during their visit. CT scans were analyzed to assess psoas and paraspinal muscle density at lumbar vertebrae L4–L5, at the mid‐level of each vertebra, taking the average of right and left sides. Muscle density was measured using Hounsfield Units (HU)

**Results:** A total of 120 subjects were included, with a mean (SD) age of 14.3 (3.6) years, 77 (64%) were male. Among these, 71 patients had IBD [57 (80%) patients with Crohns disease, and 14 (20%) with ulcerative colitis], while 49 were considered as controls. There was no significant difference between IBD and controls in terms of age (p=0.409) and gender (p=0.236).

There was significantly lower **psoas muscle** density at both L4 and L5 levels in IBD patients [L4 psoas density mean (SD) of 61.6 (5.3) HU in IBD compared to 65.4 (5.1) HU in controls, p<0.001, and L5 psoas density mean (SD) of 62.9 (6.0) HU in IBD compared to 65.8 (5.4) HU in controls, p=0.007, figure 1. **Paraspinal muscle** density at L5 level was also significantly lower in IBD patients [L4 paraspinal muscle density mean (SD) of 63.8 (7.4) HU in IBD compared to 65.8 (5.3) HU in controls, p=0.038]. On the other hand, **paraspinal muscle** density at L4 level was similar in both groups, p=0.586.

Compared to Crohn's disease, patients with ulcerative colitis had significantly lower psoas muscle density at both L4 (p=0.020) and L5 (p=0.036). Additionally, paraspinal muscle density was lower in ulcerative colitis patients, with trend towards significance (p=0.053 at L4, and p=0.094 at L5).

**Conclusions:** In our study, pediatric patients with IBD, particularly those with ulcerative colitis, demonstrate significantly reduced muscle density in both psoas and paraspinal regions on CT imaging compared to age and sex matched controls, indicating possibility of sarcopenia in this population. These findings highlight the potential utility of routinely acquired CT scans as a valuable and cost‐effective tool for assessing musculoskeletal health in pediatric patients with IBD. Larger multicenter studies may help validate these findings and support the use of CT imaging to identify sarcopenia, thereby enabling timely nutritional and therapeutic interventions to improve growth and long‐term outcomes in IBD patients.


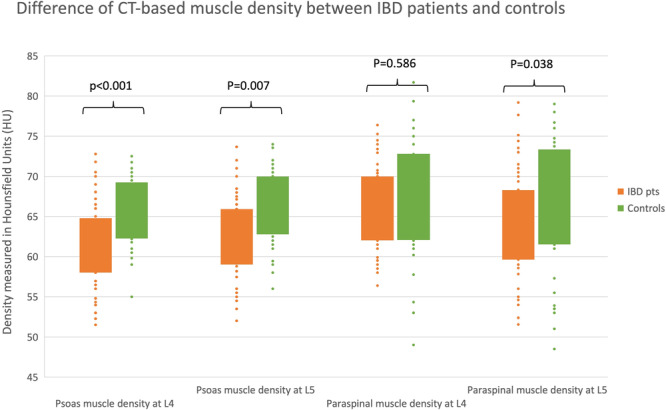


**Figure 1:** Difference of CT‐based psoas and paraspinal muscle densities between patients with inflammatory bowel disease and controls at levels of lumbar vertebrae L4‐L5, n=120.

## 433 HEALTHCARE RELATED QUALITY OF LIFE IN INFLAMMATORY BOWEL DISEASE: DOES AGE OF ONSET MATTER?

*Amala Alenchery* ^*1*^, *Benjamin Cohen* ^*2,3*^, *Jacob Kurowski* ^*1*^, *Jessica Philpott* ^*2,3*^, *Jessica Barry* ^*1*^, *Sandra Kim* ^*1*^

^*1*^ *Pediatric Gastroenterology, Hepatology and Nutrition*, *Cleveland Clinic*, *Cleveland*, *OH*; ^*2*^ *Center for Inflammatory Bowel Disease, Department of Gastroenterology, Hepatology and Nutrition*., *Cleveland Clinic*, *Cleveland*, *OH*; ^*3*^ *Digestive Disease Institute*, *Cleveland Clinic*, *Cleveland*, *OH*

**Background:** Inflammatory bowel diseases (IBD) are chronic immune‐mediated inflammatory diseases involving the gastrointestinal tract, often with extraintestinal manifestations (EIMs). When diagnosed in childhood, IBD can lead to heightened disease–related burden, including a more severe disease course, impaired growth and nutritional status, and psychosocial comorbidities. The relationship between psychological well‐being and disease activity is well‐established in both children and adults with IBD. However, limited research has specifically examined whether age at disease onset impacts healthcare related quality of life (HRQOL) and associated psychosocial outcomes in young adults living with IBD.

**Aims:** The primary aim was to assess differences in HRQOL among young adults with pediatric‐ vs adult‐onset IBD, when adjusted for disease severity. Secondary aims included assessment of psychosocial factors (stress, fatigue, and pre‐existing behavioral health diagnoses) based on age of disease onset and severity, as well as their relation to HRQOL.

**Methods:** This was a single center, cross‐sectional pilot study of young adults (21‐35 years) with an established diagnosis of IBD followed at a quaternary IBD center recruited at routine clinic or infusion visits. They were subdivided into two cohorts based on age at IBD diagnosis: pediatric (< 18 years) or adult (>/= 18 years). After informed consent was obtained, participants completed self‐reported surveys for quality of life (Short IBD Questionnaire: SIBDQ), fatigue (PROMIS‐Short Form) and perceived stress (Perceived Stress Scale: PSS). Demographic data (age, gender, education, employment, marital status, insurance – public/private), disease characteristics (IBD type and phenotype; physician global assessment or PGA, treatment/surgeries) and behavioral health co‐morbidities were obtained by retrospective chart review. The groups were compared using Wilcoxon rank sum tests for continuous and Chi‐square test for categorical variables. Multiple regression analyses were used to adjust for possible confounders with a significance level, p <0.05.

**Results:** Sixty‐one participants (n=31 pediatric‐onset; n=30 adult‐onset) completed the surveys. PGA scores were available for 59 patients. Demographic and disease characteristics were comparable between the two groups except for increased biologic treatment failure (p=0.034) and medication‐related side effects (p=0.001) in pediatric onset disease. Strong correlations between all three self‐reported surveys (p<0.001) were observed. Multivariate regression analysis revealed active disease (p<0.001), and higher self‐reported stress (p=0.002) were significantly associated with lower HRQOL. Active disease (p=0.04), public insurance (p=0.04), high self‐reported stress (p=0.001) and presence of behavioral health comorbidity (p= 0.02) were associated with higher levels of fatigue. Increased self‐reported stress was significantly impacted by the presence of behavioral health comorbidity (p=0.01) and active disease (p=0.04). Interestingly, among those with active disease, self‐reported stress levels were significantly heightened in patients with adult‐onset IBD compared to their pediatric‐onset counterparts (p=0.03).

**Conclusion:** This is the first study to examine the impact of age at disease onset (pediatric vs adult) on HRQOL and psychosocial factors in young adults with IBD. Our findings demonstrated no significant differences in HRQOL based on age of disease onset. Notably, active disease emerged as the strongest predictor for lower HRQOL, as well as greater fatigue and self‐reported stress. Additional factors associated with increased fatigue and stress included female gender, behavioral health comorbidities, and lower socioeconomic status. Heightened self‐reported stress was linked to both lower HRQOL and higher fatigue. Future directions include expansion to additional IBD centers to determine whether these initial findings can be extrapolated on a broader scale. Furthermore, quality improvement initiatives with standardized screening for fatigue and stress as well as behavioral health assessments at key timepoints (initial diagnosis, transition of care and annual health maintenance) during the IBD course can facilitate improved referral for behavioral health support, which can in turn improve HRQOL. These clinical strategies may also be applicable to other chronic disease states impacting young adults (liver transplant; eosinophilic esophagitis; celiac disease), underscoring the potential broader impact of this work.

## 435 ADVANCING PEDIATRIC GI: INSIGHTS FROM THE NICKLAUS CHILDREN'S HOSPITAL INTESTINAL ULTRASOUND PROGRAM

*Julieta Benitez*, *Martin Varea Romo*, *Luis Caicedo*, *Lina Maria Felipez Marrero*

*Pediatric Gastroenterology, Hepatology, and Nutrition*, *Nicklaus Children's Hospital*, *Miami*, *FL*

**Intro:** Intestinal Ultrasounds (IUS) is a non‐invasive diagnostic tool that provides real‐time assessment. Does not require sedation or preparation, identifies inflammation, structural abnormalities, and can help monitor progression of specific diseases such as inflammatory bowel disease (IBD). It is a great alternative for pediatric patients, vulnerable to traditional testing such as MRI. IUS helps recognize abnormal bowel wall thickening and its changes in response to therapy. It is an essential tool for monitoring pediatric patients with IBD.

**Methods:** We reviewed Nicklaus Children's (NCH) IUS database which compiles all IUS performed since the implementation of the program in August 2024. The associated diagnosis prompting IUS was used to categorize the procedures allowing for systematic organization of the data. Descriptive analysis was conducted to evaluate trends across the diagnosis. This approach provided insights into the utilization patterns and diagnostic indications for IUS.

**Results:** A total of 562 (males = 276, females =286) IUS performed from August 2024 to April 2025. Mean age 12 y/o (19 mo. – 26 y/o). Most frequent diagnosis was IBD with 291 (CD = 200, UC = 91). The second diagnosis was non‐IBD related GI disorders: chronic abdominal pain, chronic diarrhea, IBS all classified as disorders of gut‐brain interaction with 181 cases and third elevated calprotectin with 26 cases. Of the 291 IBD patients, 62 had medication changes (dose and/or frequency change). Restaging procedures were postponed for 9 patients with IBD. Of the 181 cases of non‐IBD related disorders, 19 had an endoscopy and/or colonoscopy. 13 new diagnoses of IBD were made at the time of IUS later confirmed with pathology after endoscopy and colonoscopy.

**Discussion:** The findings from the NCH IUS program, initiated in August 2024, highlight the utility and growing impact of this non‐invasive diagnostic tool in pediatric GI. The high volume of IUS procedures performed (August 2024 to April 2025) indicates the value for real‐time assessment of intestinal health in children. The youngest patient in our cohort was 19 months, suggests this is a viable alternative for this age group.

Our results shows that IUS is most frequently utilized for patients with IBD. Consistent with IUS's established role in assessing inflammation, monitoring disease progression, and evaluating treatment response in IBD. 62 IBD patients had medication changes based on IUS findings supports its utility in guiding therapeutic decisions, allowing for timely adjustments to optimize patient outcomes and reduce disease activity. The ability to postpone restaging procedures for 9 patients demonstrates IUS's potential to reduce the burden of invasive procedures, particularly beneficial for the pediatric population.

The second most common diagnosis for IUS utilization was disorders of gut‐brain interaction. This interesting finding, suggesting that IUS is not solely being used for overt inflammatory conditions but also to investigate functional gastrointestinal disorders where structural abnormalities might be suspected or need to be ruled out. The low rate of subsequent procedures in this group (19/181 cases) emphasizes IUS's role as a screening/initial diagnostic tool, avoiding unnecessary invasive procedures when IUS findings are reassuring.

The third indication, elevated calprotectin, reflects IUS's role in investigating objective markers of inflammation.

13 new diagnoses of IBD made at the time of IUS underscore the diagnostic power of this tool. This highlights its ability to identify early signs of IBD, facilitating prompt initiation of therapy, and potentially altering the course of disease. The absence of sedation and preparation makes IUS an ideal first‐line imaging modality in a pediatric setting, particularly when IBD is suspected.

Our initial data strongly support IUS as an invaluable asset in pediatric GI. Its non‐invasive nature, real‐time assessment capabilities, and ability to influence clinical decision‐making, including medication adjustments and avoidance of invasive procedures, make it an excellent alternative to traditional testing for children. The program's early success at NCH lays the groundwork for further integration of IUS into routine clinical care for pediatric patients with a wide range of gastrointestinal concerns.

**Conclusion:** The implementation of the IUS Program at NCH has rapidly demonstrated its significant value as a non‐invasive, real‐time diagnostic and monitoring tool in pediatric GI. The high volume of procedures and diverse diagnostic indications, particularly for **IBD**, emphasize its immediate clinical utility. IUS is instrumental in guiding treatment decisions, identifying new IBD diagnoses, and reducing the need for more invasive procedures like endoscopy and colonoscopy in a vulnerable pediatric population. These early results position IUS as an essential and effective component of comprehensive care for children with intestinal disorders.

## 437 ETRASIMOD FOR THE TREATMENT OF ULCERATIVE COLITIS: UP TO 4 YEARS OF SAFETY DATA FROM THE GLOBAL CLINICAL PROGRAM

*Séverine Vermeire* ^*1*^, *David Rubin* ^*2*^, *Miguel Regueiro* ^*3*^, *Ken Takeuchi* ^*4*^, *Alissa Walsh* ^*5*^, *Paulo Kotze* ^*6*^, *Aline Charabaty* ^*7*^, *Martina Goetsch* ^*8*^, *Krisztina Lazin* ^*8*^, *Joseph Wu* ^*9*^, *Georgios Tsamos* ^*10*^, *Michelle Segovia* ^*11*^, *Diogo Branquinho* ^*11*^, *Silvio Danese* ^*12*^

^*1*^ *University Hospitals Leuven*, *Leuven*, *Belgium*; ^*2*^ *University of Chicago Medicine Inflammatory Bowel Disease Center*, *Chicago*, *IL*; ^*3*^ *Cleveland Clinic*, *Cleveland*, *OH*; ^*4*^ *Tsujinaka Hospital Kashiwanoha*, *Kashiwa*, *Chiba*, *Japan*; ^*5*^ *Oxford University Hospital*, *Oxford*, *United Kingdom*; ^*6*^ *Pontifícia Universidade Católica do Paraná (PUCPR)*, *Curitiba*, *Brazil*; ^*7*^ *Johns Hopkins School of Medicine*, *Washington*,; ^*8*^ *Pfizer AG*, *Zürich*, *Switzerland*; ^*9*^ *Pfizer Inc*, *Cambridge*, *MA*; ^*10*^ *Pfizer, Thessaloniki*, *Central Macedonia*, *Greece*; ^*11*^ *Pfizer Inc*, *New York*, *NY*; ^*12*^ *IRCCS San Raffaele Hospital and Vita Salute San Raffaele University*, *Milan*, *Italy*

**Introduction:** Etrasimod is an oral, once‐daily, selective sphingosine 1‐phosphate (S1P)_1,4,5_ receptor modulator for the treatment of moderately to severely active ulcerative colitis (UC). The long‐term safety, tolerability, and efficacy of etrasimod are being evaluated in an ongoing open‐label extension (OLE) study.^1^ We report an updated cumulative safety analysis from the etrasimod UC clinical program with a maximum of 4 years of exposure.

**Methods:** Patients who received etrasimod in completed phase 2 (OASIS; OASIS OLE), phase 3 (ELEVATE UC 52; ELEVATE UC 12), and ongoing ELEVATE UC OLE (data cutoff Aug 30, 2023) and ES101002 OLE (data snapshot Aug 30, 2022) studies were included. Treatment‐emergent adverse event (TEAE) frequency and exposure‐adjusted incidence rates (IRs) per 100 patient‐years (PY) were analyzed.

**Results:** A total of 1,196 patients received ≥1 dose of etrasimod 1 mg or 2 mg once daily with a mean (standard deviation) exposure of 70.66 (54.36) weeks, and 1,619.5 PYs of total exposure. Demographics and baseline characteristics are reported in Table 1. Most TEAEs were nonserious and rarely led to discontinuation. The IRs of TEAEs of interest were generally low (Table 2). Serious infections and herpes zoster infections were infrequently reported (all IR <2.0). Three patients experienced four nonserious events of macular edema (0.3%, IR 0.18), including one event leading to discontinuation that resolved. One patient experienced two nonserious events of cystoid macular edema (<0.1%; IR 0.06) that resolved. Malignancies were uncommon and included five patients with serious events (0.4%), two patients with squamous cell carcinomas, and one with basal cell carcinoma. Eleven patients (0.9%) had alanine aminotransferase (ALT) levels >3 times the upper limit of normal (ULN) at two consecutive post‐baseline visits. Notably, no patients exhibited >3× ULN in either ALT or aspartate aminotransferase and >2× ULN in total bilirubin (laboratory criteria for Hy's law). TEAEs leading to death were reported in three patients (all deemed unrelated to treatment). No serious adverse events of hypertension or bradycardia were reported. No events of second‐degree Mobitz type 2 atrioventricular (AV) block or third‐degree AV block occurred.

**Conclusion:** Etrasimod remains well tolerated in patients with moderately to severely active UC, with a favorable safety profile that has not changed with longer‐term treatment exposure for up to 4 years.

**Declaration of Generative AI and AI‐assisted technologies in the writing process:** During the preparation of this work the authors used Pfizer's generative artificial intelligence tool MAIA to assist production of the abstract first draft. After using this tool, the authors reviewed and edited the content as needed and take full responsibility for the content of the publication.

**Reference:**

1. Vermeire S et al. J Crohns Colitis 2023; 17: i619–i620.


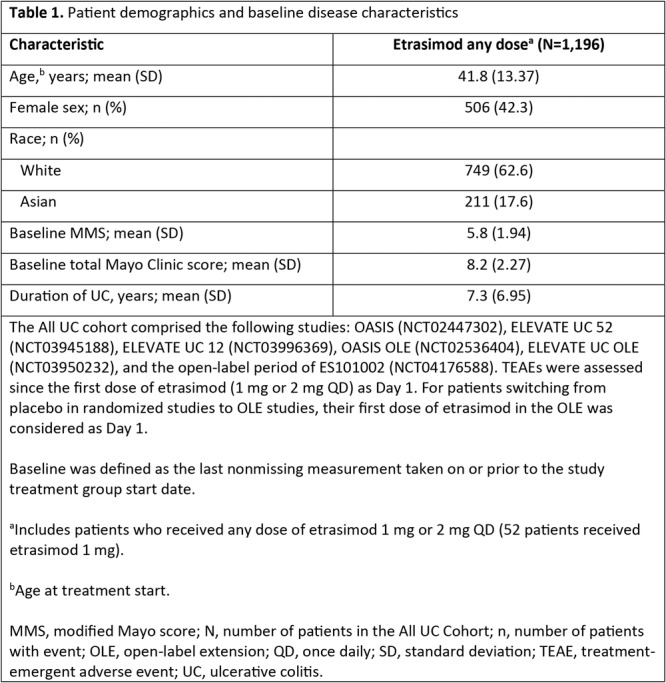



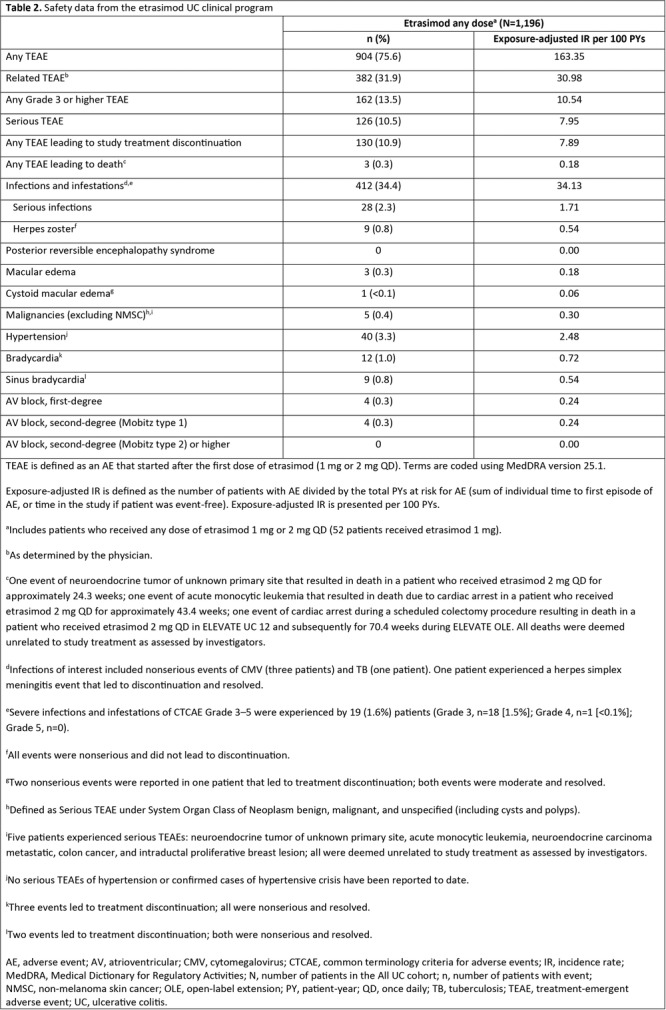


## 438 PREDICTORS FOR READMISSION IN NEWLY DIAGNOSED PEDIATRIC INFLAMMATORY BOWEL DISEASE

*Nathan Bryan*, *Kibileri Williams*, *Diana Moya*, *Himanshu Khangwal*, *Christopher Hayes*

*Gastroenterology*, *Children's National Hospital*, *Washington*

**Background:** Hospital readmission in pediatric inflammatory bowel disease (IBD) occurs frequently with estimates from 15‐20%. Most research to date has focused on adult and surgical patients rather than the pediatric population. Past researchers have found anxiety, depression, TPN use, and steroid use to be risk factors. In this study we provide a focus on risk factors for 30 day and 90 day readmission in newly diagnosed, hospitalized pediatric patients with IBD.

**Methods:** This retrospective cohort study included all newly diagnosed pediatric IBD patients admitted and diagnosed at our center between 2015‐2024. Data collection involved review of all electronic health records using ICD10 codes for IBD, Crohn's disease (CD), and ulcerative colitis (UC). Once collected, data underwent statistical modeling to create odds ratios (OR) to identify risk and protective factors for 30‐day and 90‐day readmission.

**Results:** Our initial results are included in Table 1. In total 170 patients were identified as meeting criteria for new diagnosis IBD requiring hospital admission. The rate of 30‐day readmission was 17.1% and the rate of 90‐day readmission was 28.2%. Statistically significant predictors for readmission included: no IBD specific treatment started during admission (OR = 3.08, p = 0.046, 30 d), requiring antibiotics during admission (OR 2.33, p =0.03, 90 d), and low vitamin D levels <20 ng/ml at presentation (OR 2.88, p=0.01, 30 d). Cross sectional imaging with enteral contrast via MRE or CTE during admission was found to be protective (OR 0.45, p = 0.045, 90 d). 5ASA use was also protective (OR 0.12, p=0.041 30 d, OR 0.29, p=0.028).

**Discussion:** While not initiating treatment for IBD upon diagnosis unsurprisingly leads to readmission, the use of antibiotics and the predictive value of vitamin D levels present a more interesting story. The typical reason for the 43 patients requiring antibiotics was intraabdominal infection in the setting of penetrating CD. Less often antibiotics were for concomitant c. diff infections, and rarely (1 patient) were antibiotics for non‐gastrointestinal related infection. There was correlation between stricturing/penetrating CD and antibiotic use (Pearson's 0.46+) suggesting antibiotic use as a possible marker for disease severity. However, severe endoscopic disease phenotypes (structuring/penetrating CD or severe pancolitis UC) did not independently predict readmission, though the ORs did show a trend without meeting the level of statistical significance. Conversly, 5‐ASA's protective feature (OR 0.12) likely represents mild disease. Low vitamin D levels (<20 ng/ml) may also reflect disease severity and TI mucosal involvement. Alternatively, vitamin D's role in the immune system might suggest a different modulating role in these patient's presentations. Many laboratory values often used to monitor disease or calculate severity scores provided no predictive value including ESR, CRP, albumin and fecal calprotectin. Conterintuitively anemia (Hbg <10) even appeared to be protective (OR 0.39 p=0.003). Predictors for readmission found in other studies including obesity, TPN use, and length of stay were also not found to be significant in our data. Patients with anxiety or depression prior to IBD diagnosis did show a trend towards readmission, however findings were also not statistically significant (OR 2.39, p=0.11). Along with further review and investigation of this population, next steps will involve validation of vitamin D as a potentially modifiable biomarker, phenotype specific risk profiling, and development of predictive models aimed at minimizing readmission.


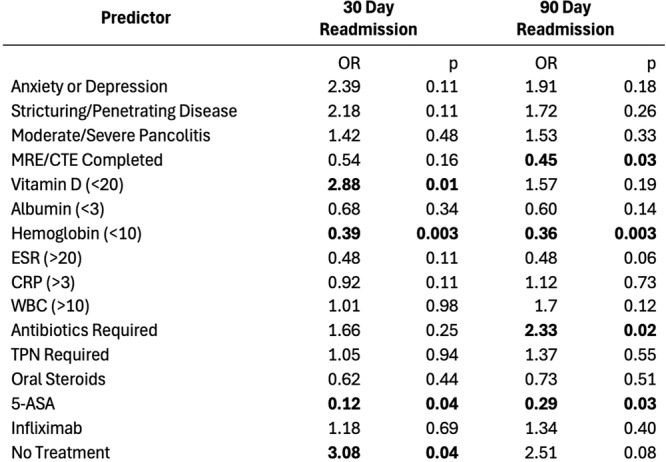


Risk Factors For Readmission

## 439 EFFICACY OF ETRASIMOD AT WEEK 52 AMONG BIOLOGIC/JANUS KINASE INHIBITOR‐NAÏVE PATIENTS WITH ULCERATIVE COLITIS WHO REACHED CLINICAL RESPONSE AT WEEK 12: POST HOC ANALYSIS OF THE PHASE 3 ELEVATE UC 52 RANDOMIZED TRIAL

*Séverine Vermeire* ^*1*^, *Bruce Sands* ^*2*^, *Marla Dubinsky* ^*3*^, *Brian Feagan* ^*4,5*^, *Remo Panaccione* ^*6*^, *Vipul Jairath* ^*7*^, *Andres Yarur* ^*8*^, *Michael Chiorean* ^*9*^, *Alissa Walsh* ^*10*^, *Martina Goetsch* ^*11*^, *Joseph Wu* ^*12*^, *Burak Sahin* ^*13*^, *Arcangelo Abbatemarco* ^*14*^, *Christina Cognata* ^*15*^, *Subrata Ghosh* ^*16*^

^*1*^ *University Hospitals Leuven*, *Leuven*, *Belgium*; ^*2*^ *Dr. Henry D. Janowitz Division of Gastroenterology, Icahn School of Medicine at Mount Sinai*, *New York*, *NY*; ^*3*^ *Susan and Leonard Feinstein IBD Center, Icahn School of Medicine at Mount Sinai*, *New York*, *NY*; ^*4*^ *Western University*, *London*, *ON*, *Canada*; ^*5*^ *Alimentiv Inc*, *London*, *ON*, *Canada*; ^*6*^ *University of Calgary*, *Calgary*, *AB*, *Canada*; ^*7*^ *Western University*, *London*, *ON*, *Canada*; ^*8*^ *Cedars‐Sinai Medical Center*, *Los Angeles*, *CA*; ^*9*^ *Swedish Medical Center*, *Seattle*, *WA*; ^*10*^ *Oxford University Hospital*, *Oxford*, *United Kingdom*; ^*11*^ *Pfizer AG*, *Zürich*, *Switzerland*; ^*12*^ *Pfizer Inc*, *Cambridge*, *MA*; ^*13*^ *Pfizer Ltd*, *Tadworth*, *United Kingdom*; ^*14*^ *Pfizer Inc*, *New York*, *NY*; ^*15*^ *Pfizer inc*, *Collegeville*, *PA*; ^*16*^ *University College Cork*, *Cork*, *Ireland*

**Introduction:** When conventional ulcerative colitis (UC) therapy fails to achieve or maintain remission, advanced UC therapies should be considered to improve patient outcomes. The responder re‐randomization trial, in which efficacy at the end of the maintenance period is reported only for the subset of patients who achieved clinical response at the end of the induction period, is a common design used in drug development of advanced UC therapies. Etrasimod is an oral, once‐daily (QD), selective sphingosine 1‐phosphate (S1P)_1,4,5_ receptor modulator for the treatment of moderately to severely active UC. The etrasimod ELEVATE UC 52 trial employed a treat‐through design, where efficacy at the end of the maintenance period was reported for the total enrolled baseline population. This post hoc analysis evaluated the efficacy of etrasimod vs placebo at Week 52 among biologic/Janus kinase inhibitor (bio/JAKi)‐naïve patients with moderately to severely active UC who achieved clinical response at Week 12.

**Methods:** ELEVATE UC 52 (NCT03945188) comprised a 12‐week induction phase followed by a 40‐week maintenance phase with a treat‐through design. Patients were randomized 2:1 to receive etrasimod 2 mg or placebo QD. Week 12 clinical response was defined as a ≥2‐point and ≥30% decrease from baseline in modified Mayo score (MMS), and a ≥1‐point decrease or absolute value ≤1 in rectal bleeding subscore. Efficacy outcomes evaluated in Week 12 bio/JAKi‐naïve responders with a baseline MMS of 5–9 were the achievement of clinical remission, clinical response, symptomatic remission, endoscopic improvement, endoscopic improvement‐histologic remission, and corticosteroid‐free clinical remission at Week 52.

**Results:** Among bio/JAKi‐naïve patients in ELEVATE UC 52, clinical response at Week 12 was achieved by 132/194 (68.0%) receiving etrasimod and 35/93 (37.6%) receiving placebo. Among Week 12 bio/JAKi‐naïve responders, significantly greater proportions receiving etrasimod vs placebo achieved clinical remission (51.5% vs 17.1%; *p*<0.001), clinical response (75.0% vs 54.3%; *p*=0.026), and symptomatic remission (70.5% vs 45.7%; *p*=0.009) at Week 52 (Figure 1). Similar findings were observed for other Week 52 efficacy endpoints (all *p*<0.05; Figure 2).

**Conclusion:** As a first‐line advanced UC therapy, etrasimod demonstrated significant efficacy over placebo in all Week 52 efficacy outcomes in bio/JAKi‐naïve induction treatment responders. These findings help contextualize data from the ELEVATE UC clinical program for patients receiving their first advanced UC therapy within a treat‐through trial design.

**Declaration of Generative AI and AI‐assisted technologies in the writing process:**

During the preparation of this work the authors used Pfizer's generative artificial intelligence tool MAIA to assist production of the abstract first draft. After using this tool, the authors reviewed and edited the content as needed and take full responsibility for the content of the publication.


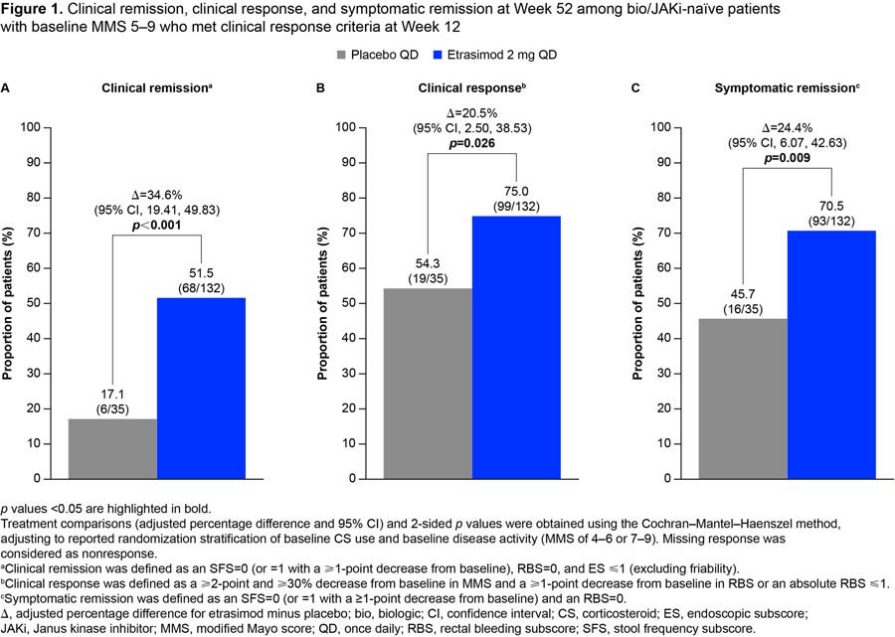



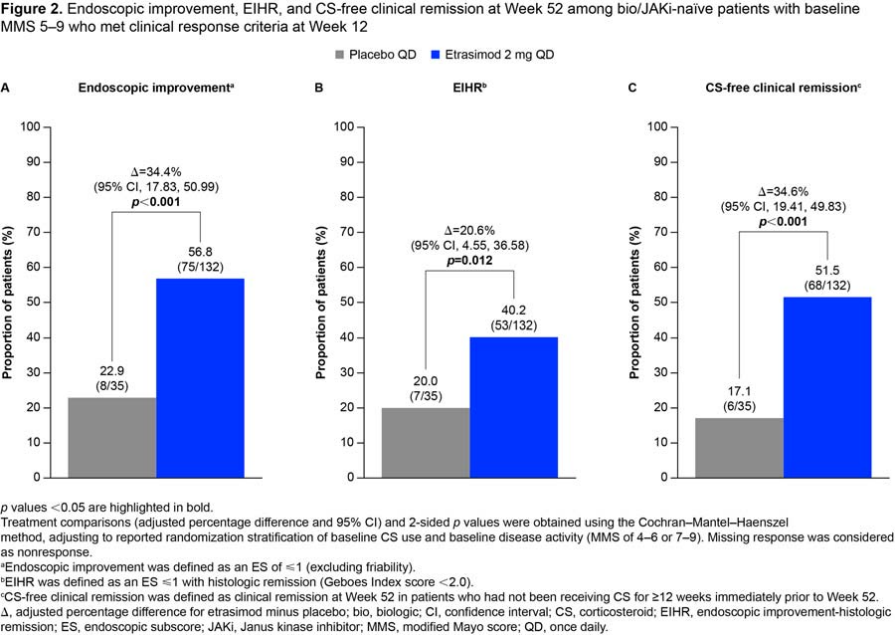


## 442 PREVALENCE OF DYSMENORRHEA IN PEDIATRIC IBD: A WIDELY UNDER RECOGNIZED AND UNDERTREATED CONDITION

*Katsiaryna Dubrouskaya* ^*4*^, *Evan Witz* ^*2*^, *Amanda Wenzel* ^*1*^, *Jose Cabrera* ^*1*^, *Joshua Noe* ^*1*^, *Joann Samalik* ^*1*^, *Jessica Francis* ^*3*^, *Abdul Elkadri* ^*1*^

^*1*^ *Pediatric Gastroenterology*, *Medical College of Wisconsin*, *Milwaukee*, *WI*; ^*2*^ *Mathematics*, *Wisconsin Lutheran College*, *Milwaukee*, *WI*; ^*3*^ *Obstetrics and Gynecology*, *Medical College of Wisconsin*, *Milwaukee*, *WI*; ^*4*^ *Pediatrics*, *Medical College of Wisconsin*, *Milwaukee*, *WI*

**Background:** Primary dysmenorrhea is one of the most common causes of pelvic pain in female patients, with equal rates across all ages for menstruating females. Female inflammatory bowel disease (IBD) patients commonly report changes in symptoms around and during their menses. Currently, there is no published data using systematic dysmenorrhea screening in the female pediatric IBD population, as well as no data on long term follow up of their outcomes with treatment for dysmenorrhea.

**Objective:** To determine the prevalence of dysmenorrhea and the extent of female IBD patients receiving dysmenorrhea treatment. A secondary objective is to investigate the relationship of dysmenorrhea symptoms and methods to improve the pain experienced by these patients.

**Design:** Female pediatric patients 10 years and older with IBD received a 6‐question dysmenorrhea survey at routine IBD clinic visits at a tertiary care academic center. The questionnaire assessed pain severity, change in bowel habits during menses, days of school missed due to symptoms, and if patients were already receiving treatment for dysmenorrhea. If a patient screened positive, a referral to adolescent gynecology was discussed with patients and families. A retrospective chart review was performed to follow outcomes of dysmenorrhea treatment.

**Results:** A total of 232 questionnaires were completed between November 2023 to March 2025 with 132 unique patients. 27% (n=36) patients had a positive screen for dysmenorrhea based on pain criteria. 66% (n=24) of patients who screened positive were not already treated for these symptoms. 26% (n=34) of total patients reported already being treated for dysmenorrhea. Crohn's disease (CD) patients (28.2%) reported higher rates of pain ≥5 than Ulcerative Colitis (UC) patients (19.5%). The most common forms of birth control reported included oral contraceptives and IUD. A total of 6 referrals were sent to adolescent gynecology. 44 patients reported not having started menses, with an average age of this group being 12.74 (SD ±2).

**Conclusion:** Dysmenorrhea is common in the female pediatric IBD population, and a quarter of patients have significant pain which could present with flare type symptoms, such as a change in bowel movements. Our study found that a majority of patients who screened positive for dysmenorrhea have received no treatment for their increased pain symptoms. Although the difference in number of patients endorsing dysmenorrhea was not statistically significant, there appears to be a clinically significant higher number of CD patients than UC endorsing symptoms which requires further investigation. Pediatric gastroenterologists should be aware of how to screen for dysmenorrhea. Referral to adolescent gynecology may be warranted for further evaluation and treatment.

## 443 DURABILITY AND PREDICTORS OF CLINICAL OUTCOMES FOLLOWING INFLIXIMAB USE IN HOSPITALIZED PEDIATRIC PATIENTS WITH ACUTE SEVERE ULCERATIVE COLITIS: A SINGLE‐CENTER COHORT STUDY

*Jose Erazo*, *Brianna Evans*, *Marla Dubinsky*, *Elizabeth Spencer*

*Pediatric Gastroenterology*, *Mount Sinai, Icahn School of Medicine*, *Brooklyn*, *NY*

**Background:** Hospitalized pediatric patients with acute severe ulcerative colitis (ASUC) are commonly treated with infliximab (IFX), yet long‐term durability and predictors of treatment failure remain poorly defined. These questions have become increasingly relevant in the context of an expanding therapeutic landscape, including Janus kinase inhibitors (JAKi), which have shown promise in hospitalized ASUC.

**Methods:** We conducted a retrospective cohort study of pediatric patients (<18 years) hospitalized with ASUC at a single tertiary care center between 2016 and 2024 who received IFX during admission. Co‐primary outcomes were: (1) a composite clinical failure outcome—defined as colectomy, corticosteroid re‐initiation, or IBD‐related re‐hospitalization after IFX initiation—and (2) IFX durability, defined as continued IFX use at last follow‐up without switch or discontinuation. Secondary outcomes included clinical response (Pediatric UC Activity Index (PUCAI) decrease ≥20 points) by Day 5 on IFX, clinical (PUCAI<10), steroid‐ and colectomy‐free remission at last follow‐up, attainment of pharmacokinetic induction targets (IFX trough ≥27 µg/mL at infusion 2 and/or ≥17 µg/mL at infusion 3), and transition to a JAKi. Statistical analyses included Kaplan‐Meier survival, Fisher's exact, Wilcoxon rank‐sum, and univariable analyses. Medians are presented with interquartile ranges [IQR].

**Results:** Among 27 patients (**Table 1**, median age 13.7 years [12.0–16.4], 26% male), IFX was initiated a median of 2 days [1–3] after admission and 0.4 years [0.1–1.1] after UC diagnosis. All patients received concomitant IV methylprednisolone (median dose 0.76 mg/kg [0.62–1.0]). At a median follow‐up of 2.1 years [0.8–4.0], 56% (15/27) experienced the composite failure outcome, including 1 colectomy (4%) and 9 re‐hospitalizations (33%). Median time to failure was 1.0 year [0.7–3.5], with 1‐year failure‐free survival of 75% (**Figure 1 A**).

IFX durability at last follow‐up was 48% (**Figure 1B**). Among those who discontinued IFX, second‐line advanced therapies included upadacitinib (n=6), ustekinumab (n=5), vedolizumab (n=2), and tofacitinib (n=1). Despite limited IFX durability, 88% (24/27) of the children achieved clinical, steroid‐ and colectomy‐free remission at last follow‐up.

Day 5 clinical response was observed in 91% of patients who remained on IFX and 92% of those who eventually discontinued it (p = 1.0). Patients who did not experience the composite clinical failure outcome had a significantly longer duration on infliximab (median 420 vs. 72 days, p = 0.002), but no other clinical or demographic variables were significantly associated with this outcome (**Table 1**). Among 18 patients with available IFX trough levels during induction, 8/18 were below pharmacokinetic targets; IFX durability was 50% (4/8) in those meeting targets vs. 30% (3/10) in those who did not (p = 0.63).

In a subgroup comparison of patients who transitioned to UPA by last follow‐up (n=9) versus those who remained durably on IFX (n=13), the UPA group had numerically higher baseline ESR (median 64 [37–81] vs. 36 [17–48], p = 0.13) and lower baseline albumin (median 2.9 g/dL [2.5–3.5] vs. 3.0 g/dL [2.9–3.4], p = 0.64), though differences were not statistically significant.

**Conclusions:** In this single‐center pediatric ASUC cohort, fewer than half of patients maintained long‐term IFX durability. However, colectomy was rare—occurring in only one patient over a median follow‐up of two years. These findings suggest that while IFX durability is often limited, effective second‐line therapies such as JAKi may mitigate the need for surgery. Further prospective multicenter studies are needed to guide therapeutic sequencing in hospitalized pediatric ASUC.


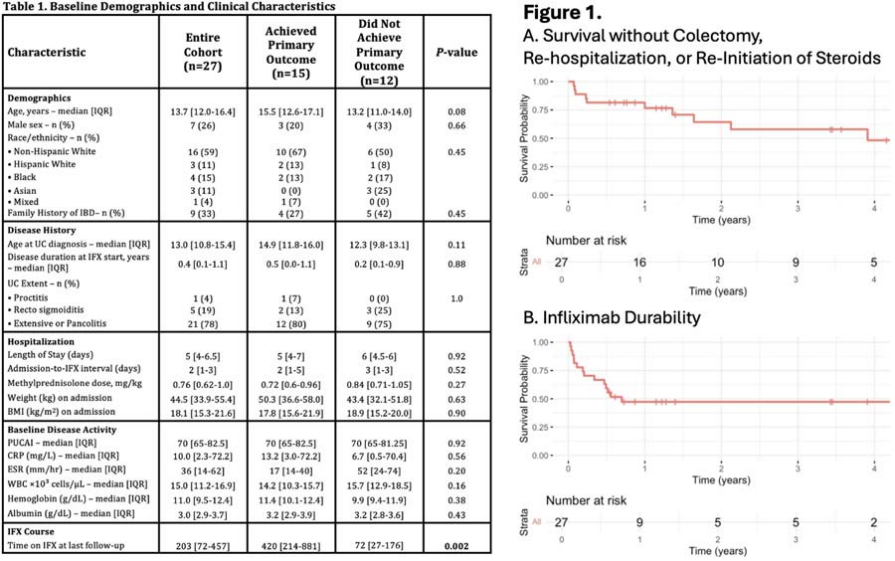


## 444 ANTIMICROBIAL USE AMONG PEDIATRIC PATIENTS WITH PERIANAL FISTULIZING CROHN'S DISEASE

*Samir Gadepalli* ^*2,3*^, *Alison Tribble* ^*4*^, *Moshiur Rahman* ^*3*^, *Lauren Manning* ^*3*^, *Jeremy Adler* ^*1,3*^

^*1*^ *Pediatric Gastroenterology*, *University of Michigan Michigan Medicine*, *Ann Arbor*, *MI*; ^*2*^ *Pediatric Surgery*, *University of Michigan Michigan Medicine*, *Ann Arbor*, *MI*; ^*3*^ *Susan B. Meister Child Health Evaluation and Research Center*, *University of Michigan Michigan Medicine*, *Ann Arbor*, *MI*; ^*4*^ *Pediatric Infectious Disease*, *University of Michigan Michigan Medicine*, *Ann Arbor*, *MI*

**Background:** Nearly 30% of children with Crohn's disease (CD) commonly develop perianal fistulizing CD (PFCD), more often than among adults. PFCD worsens quality of life and increases healthcare utilization, including hospitalization and surgery.

Although the efficacy of antimicrobials in the era of biologic therapy has not been well characterized, society guidelines recommend antimicrobials for first‐line PFCD treatment. However, antimicrobials can increase risk of infection with *Clostridioides difficile* and other resistant organisms, alter the microbiome leading to dysbiosis, and may adversely impact CD activity. Evidence‐based approaches to inform antimicrobial use for PFCD are lacking. We report the early findings from Standardization of Evaluation to Treatment of pediatric perianal CD and improving Outcomes through Networking (SETON), the first study of antimicrobial stewardship for PFCD.

**Methods:** Using Merative Marketscan (US‐based administrative database inclusive of commercial and Medicaid), we identified children (1‐18 yr) diagnosed with CD, and identified PFCD (including perianal fistula and/or abscess) using validated case definitions. We required enrollment 1 yr before and 4 yr after CD diagnosis. We categorized the timing of PFCD as “before/at Dx” as 1 yr before to 30 d after CD diagnosis, and “after Dx” as 31 d to 4 yr after CD diagnosis. Antimicrobials evaluated included metronidazole, ciprofloxacin, levofloxacin, amoxicillin ±clavulanate, nitazoxanide, rifaximin, and doxycycline. We used descriptive statistics including chi‐square and Wilcoxon rank sum to compare groups.

**Results:** 7,161 patients with CD were included (57% male, 80% commercial insurance). Age at CD diagnosis was 63% 15‐18 yr, 28% 10‐14 yr, 8% 5‐9 yr, and 1% 1‐4 yr. A total of 1,226 (17.1%) developed PFCD from 1 yr before to 4 yr after CD diagnosis, more commonly among males (18.0%) than females (16.0%; p=0.03), and more commonly among those diagnosed 15‐18 yr (18.4%) compared to 10‐14 yr (15.7%), 5‐9 yr (11.9%) and 1‐4 yr (14.9%; p<0.001). There were no differences in overall PFCD development by insurance (p=0.18). Dividing by timeframe of PFCD development, 548 (7.7%) patients developed PFCD before/at Dx, and 678 (9.5%) who developed PFCD after Dx.

Antimicrobial use was common among all with CD but more frequent in those who developed PFCD at any time (1 yr before to 4 yr after CD diagnosis), 1,126 (91.8%) compared with 5,156 (86.9%) of those without PFCD (p<0.0001). Patients who developed PFCD had more antimicrobial prescription fills (mean 6.9, median 5.0, IQR 2‐9) compared to those without PFCD (mean 3.8, median 2, IQR 1‐5; p < 0.0001) during the study period. Focusing on timing of antimicrobial use, among all patients with CD, antimicrobials were used by 3,029 (42.3%) before/at Dx, and 4,887 (68.2%) after Dx. Among those who developed PFCD before/at Dx, 368 (67.2%) received antimicrobials before/at Dx compared to 2,661 (40.2%) without early PFCD (p < 0.0001).

**Discussion:** Antimicrobial use is common in pediatric patients with CD in general, and especially among those with PFCD. PFCD before/at CD diagnosis increases antimicrobial use 1.7‐fold. However, antimicrobial use with PFCD is not universal as about 30% of patients with PFCD did not use antimicrobials. Variation in use related to abscesses and procedures, and associations with outcomes and adverse events are yet to be characterized in the ongoing study, and we aim to report these in the near future.

## 447 INSURANCE IQ: DEMYSTIFYING HEALTH INSURANCE FOR YOUNG PEOPLE WITH IBD

*Laura Gilligan* ^*1,2*^, *Jeannie Huang* ^*2,1*^

^*1*^ *Pediatrics*, *University of California San Diego*, *La Jolla*, *CA*; ^*2*^ *Gastroenterology*, *Rady Children's Hospital‐San Diego*, *San Diego*, *CA*

**Background & Objective:** Access to healthcare in the United States is closely tied to health insurance coverage, yet many adolescents and young adults with inflammatory bowel disease (AYA‐IBD) lack essential knowledge about how insurance works. Prior research has identified significant deficits in health insurance literacy within this population. This study aimed to enhance health insurance knowledge among AYA‐IBD patients through a targeted intervention delivered via the electronic medical record (EMR) system.

**Methods:** From January to May 2025, adolescents and young adults (15 y and older) with inflammatory bowel disease (AYA‐IBD) who had previously completed a 10‐item Health Insurance Knowledge (HIK) assessment (score range 0‐10) and had an upcoming IBD clinic visit were identified. Eligible participants received a MyChart message containing a brief educational video on health insurance and an infographic outlining 10 fundamental health insurance concepts (Figure 1). At their subsequent clinic visit, participants completed the HIK assessment again. Changes in HIK scores were calculated and compared based on whether the participant viewed the MyChart communication. Descriptive statistics were used to summarize demographic characteristics, and group differences were analyzed using the Wilcoxon rank sum test.

**Results:** A total of 85 AYA‐IBD participated in the intervention. Participants were 58.8% male with a median (interquartile (IQR)) age of 18 (17‐19) years and had diagnoses of Crohn's disease (54.1%), ulcerative colitis (35.3%), and IBD‐unclassified (10.6%) with a median (IQR) disease duration of 4.2 (2.2‐8.0) years. At baseline, AYA‐IBD scored a median (IQR) HIK score of 3 (0‐6) correct. Of the 85 participants, 26 viewed the electronic medical education communication, while the remaining 59 did not. Statistically significant improvements in HIK scores were observed among AYA‐IBD who engaged with the educational content, compared to those who did not (P=0.03, Table 2). These improvements were particularly evident among male participants (P=0.04), individuals with government health insurance (P<0.01), and those with a shorter duration of disease (P=0.03).

**Conclusion:** AYA‐IBD who accessed EMR portal educational content demonstrated significant improvements in health insurance knowledge, with the greatest gains observed among vulnerable subgroups. These findings highlight the potential of EMR‐integrated educational communications as a scalable, targeted, and effective strategy for delivering health education to youth with chronic conditions. This approach may help reduce disparities in health literacy and enhance patient empowerment in managing their care.


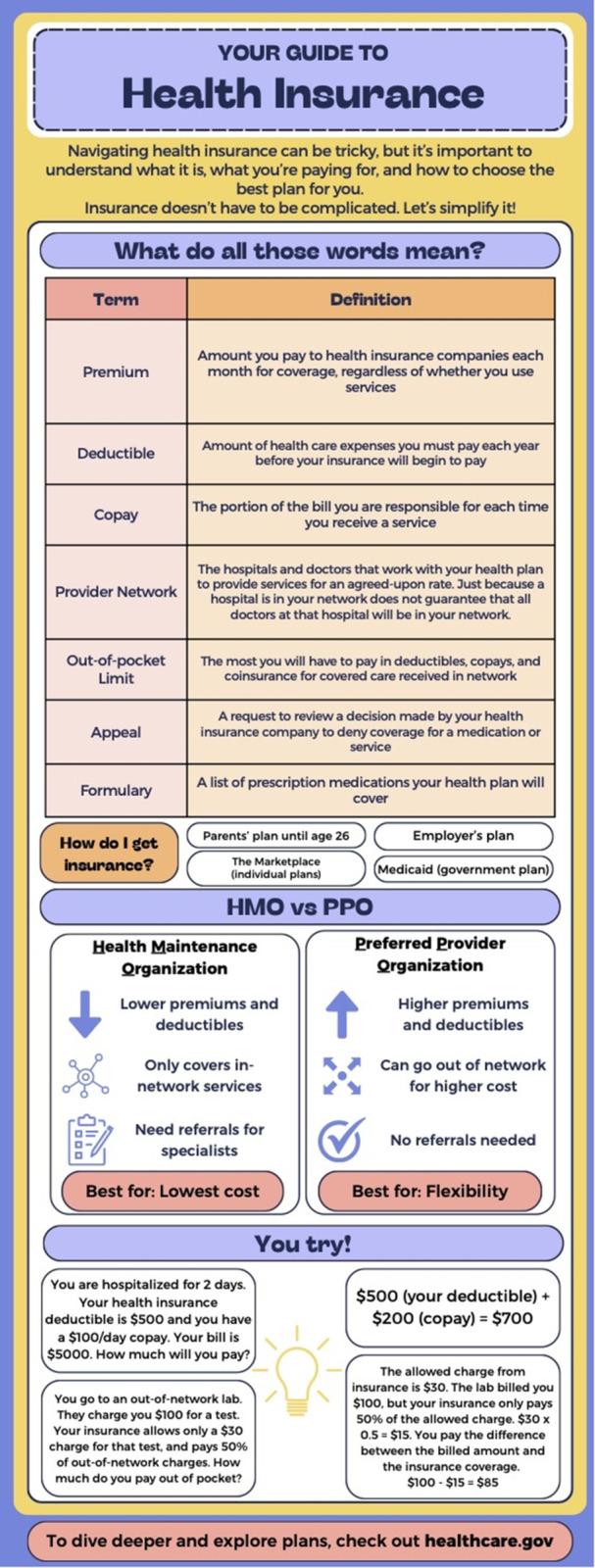



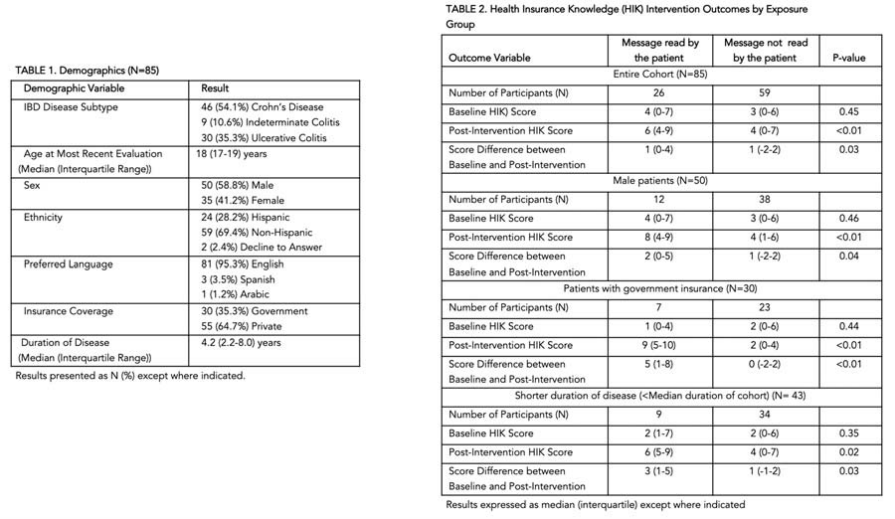


## 448 INJECTABLE DRUG‐ELUTING BIODEGRADABLE HYDROGEL FOR INFLAMMATORY BOWEL DISEASE TREATMENT

*Rafael Gonzalez* ^*1*^, *Neil Baugh* ^*2*^, *Narelli Paiva* ^*2*^, *Carly Celebrezze* ^*3*^, *Siavash Shariatzadeh* ^*3*^, *Pamela Emengo* ^*3*^, *Chih‐Hsin Chen* ^*3*^, *Anne‐Laure Thomas* ^*3*^, *Sarah Heilshorn* ^*2*^, *James Dunn* ^*3*^

^*1*^ *Pediatric Gastroenterology*, *Stanford University*, *Stanford*, *CA*; ^*2*^ *Materials Science and Engineering*, *Stanford University*, *Stanford*, *CA*; ^*3*^ *Pediatric Surgery*, *Stanford University*, *Stanford*, *CA*

**BACKGROUND:** Inflammatory bowel diseases (IBD) are a group of chronic inflammatory disorders of the gastrointestinal (GI) tract that include ulcerative colitis and Crohn's disease. IBD is a lifelong disease that requires continuous medical intervention. Current IBD treatments involve systemic corticosteroids and immunosuppressive medications to control symptoms, which many short and long‐term side effects. Treatments would benefit from direct delivery to the inflamed bowel, limiting side effects through reduced dosing and localized treatment. We developed an injectable gel composed of hyaluronic acid and elastin‐like protein hydrogel loaded with liposomal, drug‐eluting nanoparticles (NP gel), which can be delivered locally to the inflamed bowel to create a reservoir of medications. These medications will be sustainably released within the inflamed bowel for an extended time, increasing the duration of effect and preventing unwanted systemic side effects. Our objective is to deliver a corticosteroid (budesonide) and an immunosuppressive medication (infliximab) through this innovative NP gel in a mouse IBD model. We will demonstrate the efficacy of our treatment by monitoring the improvement of the animals’ IBD symptoms and histologic changes of bowel after therapy.

**METHODS:** ELISA assays were utilized to measure drug release from NP gel. Colitis was induced in mice by feeding them 2% dextran sulfate sodium water for 7 days resulting in weight loss and loose, bloody stools. A laparotomy was performed and initially the wall of the colon was injected with NP gel. However, this resulted in obstructions and perforations in the mice. The decision was made to perform injections in the cecum because of its wider caliber, which was less likely to obstruct. Utilizing a 33‐gauge needle, 10 microliters of non‐drug containing NP gel were injected into the cecal wall of three mice (image 1 A). Mice were observed for seven days then euthanized. An additional mouse was injected with fluorescently tagged NP gel which enabled us to monitor the presence of the gel over time in a non‐invasive manner with an in‐vivo imaging system (IVIS). This mouse was observed for over 1 month to test if gel remained in bowel over longer periods of time.

**RESULTS:** NP gel drug‐eluting ELISA assays calculated the release of 163 ng of infliximab for every 5 μL of gel. After cecal injections, mice quickly recovered, regained weight and showed no signs or symptoms of obstruction or perforation. At euthanasia on post‐operative day (POD) 7 the injections appeared to remain present within the cecal wall. Tissue specimens of the cecum were processed for histology which confirmed that the gel was successfully injected into the bowel wall (image 1B). IVIS imaging showed gel present soon after injection on POD‐3 (image 2 A) and POD‐41 (image 2B), proving that it remains in the bowel wall for potential sustained release of medications over weeks.

**CONCLUSIONS:** Drug eluting NP gels can be safely injected into the wall of inflamed cecum potentially serving as a treatment for IBD. At time of abstract submission, the next steps are to deliver injections of NP gel containing medications. Our plan is to inject mice with 10 microliters NP gel containing infliximab subcutaneously to test tolerance at this dose. We will also perform cecal wall injections of NP gel + infliximab, NP gel + budesonide and NP gel + budesonide + infliximab. We will observe mice for seven, euthanize and send injected tissue for histology.


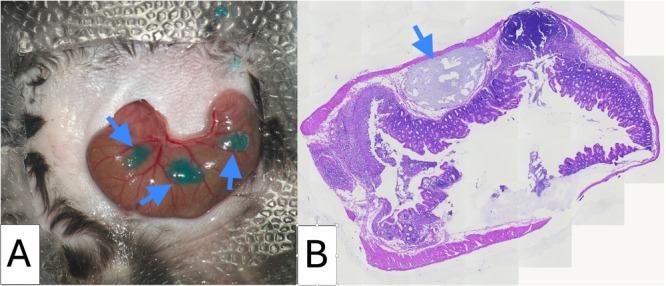



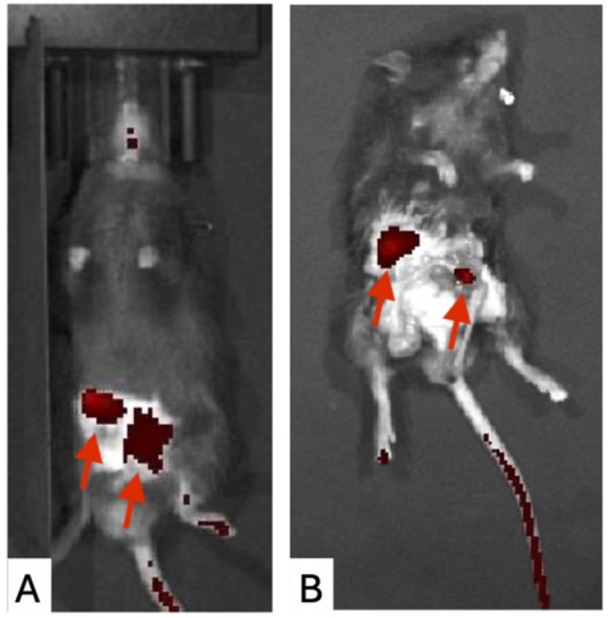


## 450 CLINICAL OUTCOMES OF ADALIMUMAB NONMEDICAL SWITCHES IN PEDIATRIC PATIENTS WITH INFLAMMATORY BOWEL DISEASE

*Daniel Himelstein* ^*1*^, *Megan McNicol* ^*1*^, *Mahmoud Abdel‐Rasoul* ^*2*^, *Brendan Boyle* ^*1*^, *Hilary Michel* ^*1*^, *Ross Maltz* ^*1*^

^*1*^ *Pediatric Gastroenterology*, *Nationwide Children's Hospital*, *Columbus*, *OH*; ^*2*^ *Department of Biomedical Informatics*, *The Ohio State University Wexner Medical Center*, *Columbus*, *OH*

**Objectives:** Tumor necrosis factor (TNF) inhibitors are first‐line therapies for Crohn's disease and second line therapies for ulcerative colitis. Historically, the introduction of biosimilars has significantly reduced the cost of both the originator biologics and biosimilars. Previous studies have demonstrated comparable safety and efficacy between adalimumab biosimilars and the originator in patients with inflammatory bowel disease (IBD), as well as similar clinical outcomes following a switch to the biosimilar. However, research on adalimumab biosimilars in the pediatric population remains limited, and no studies to date have examined the efficacy and safety of switching from the adalimumab originator to a biosimilar in children. This study aims to evaluate the clinical outcomes of pediatric and young adult patients with IBD who have undergone a nonmedical, insurance‐driven switch from the adalimumab originator to a biosimilar.

**Methods:** A single‐center retrospective chart review was conducted amongst patients with IBD who underwent a nonmedical switch from the adalimumab originator to a biosimilar. Data collected included demographic information, physician global assessments (PGAs), laboratory results, as well as drug and antibody levels approximately 12 months prior to and up to 6 months following the switch. At the 6‐month follow‐up, patients were evaluated to determine if they remained on the biosimilar medication, and if not, what led to the change in therapy. Various statistical methods were employed to compare variables before and after the switch, including McNemar's exact test (PGA values), linear mixed effect models (lab values), and paired T tests (dose and interval).

**Results:** Fifty patients underwent a nonmedical switch from the adalimumab originator to a biosimilar between June 2019 and October 2024. At six months post‐switch, 38 patients (76%) remained on the adalimumab biosimilar. Among the 12 patients who were no longer on the biosimilar, 4 (8%) experienced disease worsening, 2 (4%) reported adverse reactions, and 1 (2%) had a decreased drug level. Additionally, 3 patients (6%) had worsening disease prior to the switch to the biosimilar and 2 patients (4%) discontinued for reasons unrelated to the biosimilar. Among all patients, laboratory markers including CRP, albumin, ESR, and hemoglobin remained stable before and after the switch. Although adalimumab levels significantly decreased post‐switch (from 18 to 15 ug/mL), the reduction was unlikely to have clinical significance. Among the 42 patients with PGA scores recorded both before and after the switch, 36 (86%) demonstrated either stable or improved PGA scores.

**Conclusion:** A nonmedical, insurance‐driven switch from the adalimumab originator to a biosimilar resulted in comparable clinical, laboratory, and pharmacologic outcomes in children and young adults with IBD, and the majority of patients remained on the biosimilar at 6 months post‐switch. However, a quarter of patients were no longer on the biosimilar 6 months post‐switch suggesting need for larger pediatric studies to clarify whether these findings are associative versus causitive.

## 451 EVALUATING THE UTILITY OF INTESTINAL ULTRASOUND IN VERY EARLY ONSET INFLAMMATORY BOWEL DISEASE

*Katelynn Ho*, *Hengqi Zheng*, *David Suskind*, *Ghassan Wahbeh*

*Pediatrics*, *University of Washington System*, *Seattle*, *WA*

**Background:** Intestinal ultrasound (IUS) is a noninvasive modality used to assess disease activity in inflammatory bowel disease (IBD). Bowel wall thickness (BWT) and hyperemia are the primary measures that determine intestinal inflammation. While IUS is routinely used in adult IBD, its tolerability and accuracy in the very early onset inflammatory bowel disease population (diagnosis before age 6) has not been well defined.

**Objectives:** The aim of this study was to review IUS indications, image quality, concordance with endoscopy, and IUS challenges in patients with very early onset IBD (VEO‐IBD).

**Methods:** We conducted a single‐center retrospective observational study of VEO‐IBD patients who had an intestinal ultrasound completed between November 2024 and May 2025. Data on indications, image quality, concordance with endoscopy, and IUS challenges was extracted from medical records. Images were obtained using the in the GE Venue Go R5 (inpatient setting; Linear L4‐12t and Microconvex 8C‐RS probes) and the GE Logiq S8 (outpatient setting; Linear ML6‐15 and Convex C1‐6 probes) at the Seattle Children's Hospital IBD center. Image quality was subjectively rated from ‘very poor’ to ‘good’. Ultrasound concordance with endoscopy was evaluated when the image quality was ‘moderate’ or ‘good,’ the endoscopy was completed within 14 days of IUS, and the exam captured at least 4/5 standard bowel segments.

**Results:** Eight patients with VEO‐IBD were identified. The mean age at IBD diagnosis was 35 ± 22.5 months and the mean age at the time of IUS was 41.7 ± 23 months. The mean disease duration at the time of IUS was 6.4 ± 7 months. IUS images were ‘good’ in 6/8 (75%) of studies and used to support clinical decision making, while images ‘very poor’ to ‘moderate’ were excluded. Five ultrasounds were compared for endoscopic concordance and in all endoscopies, there was inflammation in the sigmoid through the ascending colon and no inflammation in the terminal ileum. Endoscopic concordance increased with lower bowel wall thickness cutoffs of <3 mm (table 1). Challenges encountered included breathing artifact, small bowel peristalsis, fussiness/agitation, probe size too large for longitudinal measurements relative to abdominal size, and lack of definition/inability to measure BWT with the convex probe (table 2).

**Conclusion:** Intestinal ultrasound can successfully be completed with useful image quality in the majority of children with VEO‐IBD with primary limitations in obtaining useful images being fussiness and probe size. Normal bowel wall thickness in the VEO‐IBD population may be lower than both the adult and proposed pediatric cutoffs. Larger population studies are needed to better define IUS parameters and optimal scanning standards in VEO‐IBD.


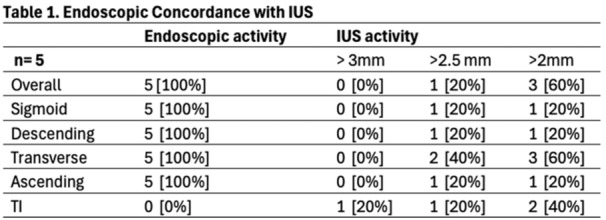



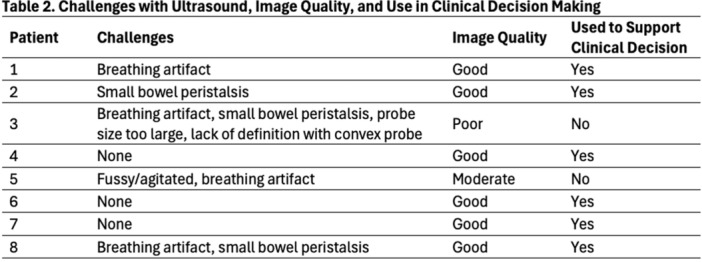


## 453 CLINICAL CHARACTERISTICS OF PEDIATRIC PATIENTS WITH IBD WHO DEVELOPED EOE SUBSEQUENT TO STARTING THERAPY: A COHORT PILOT STUDY

*Judy Jasser*, *Maria Amendola, MD*, *Elizabeth Sinclair*, *Whitney Sunseri*

*UPMC*, *Pittsburgh*, *PA*

**Background:** Eosinophilic esophagitis (EoE) and inflammatory bowel disease (IBD) are chronic, immune‐mediated gastrointestinal disorders with overlapping features. In some pediatric patients, EoE is diagnosed after initiation of IBD therapy, raising questions about whether IBD treatment could serve as a trigger for EoE. Additionally, it remains unclear whether these patients respond differently to EoE‐directed therapies compared to those diagnosed independently of IBD treatment. A better understanding of this subgroup is essential for optimizing care and guiding future research.

**Methods:** We conducted a retrospective study of 25 pediatric patients diagnosed with EoE after IBD treatment had been initiated, identified between 2010 and 2022. Patients with synchronous diagnosis of IBD and EoE—defined as EoE identified on the diagnostic endoscopy performed for initial IBD workup—were excluded. Demographic, clinical, therapeutic, and histologic data were collected. We evaluated IBD subtypes, treatment exposures (biologics[%1], immunomodulators[%2], others), and response to EoE‐directed therapy.

**Results:** Among the 25 patients (17 male, 8 female), 19 (76%) had Crohn's disease, 4 (16%) had ulcerative colitis, and 2 (8%) had indeterminate colitis. Seven of 19 Crohn's patients (36.8%) had perianal involvement. The average age at IBD diagnosis was 12.3 years, and the average age at EoE diagnosis was 15.8 years. Fourteen patients (56%) had a past medical history of atopic disease, and 8 of 24 (33.3%) had a family history of atopy.

Biologic therapy (infliximab/adalimumab) [%3] had been administered to 13 patients (52%), methotrexate to 4 patients (16%), and other therapies to 8 patients (32%). Of the 24 patients with follow‐up data, 18 (75%) received treatment for EoE. Among these, 6 (33.3%) achieved histologic remission. Of the 6 untreated patients, 1 (16.7%) achieved remission without intervention.

**Conclusion:** In this cohort of pediatric patients diagnosed with EoE after IBD therapy initiation, a majority had been exposed to biologic‐containing treatment. While some patients responded to EoE‐directed therapy, a notable proportion did not achieve remission, and a smaller subset achieved remission without treatment. These findings highlight the complexity of EoE development following IBD therapy and underscore the need for prospective studies to evaluate causality, treatment response, and the underlying immunologic mechanisms in this unique population.


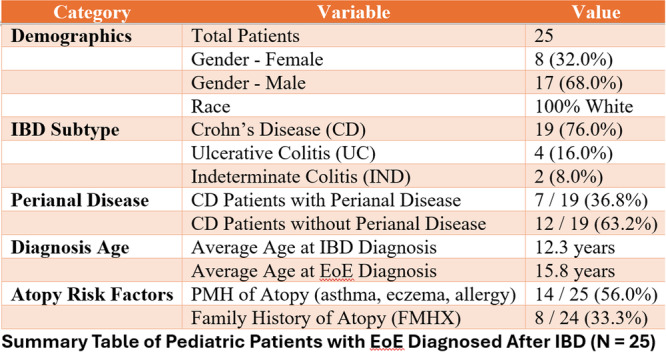



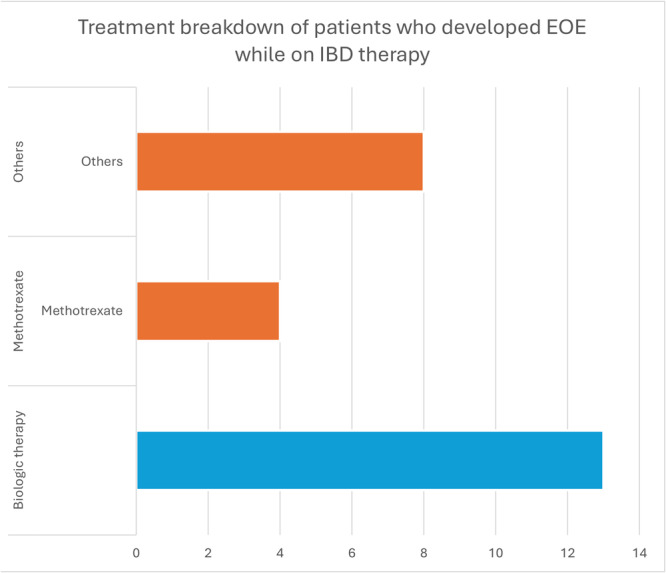


## 455 GENETIC RISK VARIANTS ARE COMMON IN PEDIATRIC INFLAMMATORY BOWEL DISEASE PATIENTS FROM MINORITIZED BACKGROUNDS

*Shahzaib Khan*, *Shagun Sharma*, *Melissa Ramirez Escobar*, *Anne Levine*

*Pediatric Gastroenterology & Hepatology*, *SUNY Downstate Health Sciences University*, *New York*, *NY*

**Introduction:** Inflammatory bowel disease (IBD), comprised of Crohn's disease (CD) and ulcerative colitis (UC), is a chronic immune‐mediated disorder, which is traditionally thought of as affecting individuals of European and Ashkenazi Jewish heritage. However, the incidence of IBD is increasing worldwide and it is fast becoming a global disease, affecting patients of all heritages. There is a significant genetic component to disease risk, and genome‐wide association studies (GWAS) have identified over 200 risk genes. However, most studies draw from populations with relatively few non‐Caucasian patients. At our center, the majority of IBD patients are of Afro‐Caribbean descent, and many are first‐ or second‐generation immigrants. Although the genetic architecture of IBD in patients of African descent strongly resembles that of Caucasians, some risk alleles are ethnicity specific, and others exert different effect sizes across populations. The frequency and significance of many risk alleles is unknown in our population; thus, we aimed to describe the frequency of a limited number of risk alleles.

**Methods:** We conducted a retrospective chart review of individuals aged 5‐21 diagnosed with IBD at SUNY Downstate Medical Center between 2009‐2024. Data collected from the electronic medical record (EMR) included demographics, including ethnic background and immigration status, clinical characteristics, disease phenotype, and laboratory results, including the Prometheus Laboratories SGI panel, which reports genotypes for known IBD risk variants in *ATG16L1*, *ECM1*, *NKX2*‐*3*, and *STAT3*. Pediatric patients with histologically confirmed diagnoses of IBD were included in the study.

**Results:** A total of 19 patients met inclusion criteria and had sufficient data available for analysis. Four were native‐born (21%), 3 first‐generation immigrants (16%) and 12 second‐generation immigrants (63%). The majority of patients were African American (10, 53%), with 2 Hispanics (10.5%), and 7 from other ethnic backgrounds (37%). There were no Caucasian patients (Table 1). We noted a high frequency of disease‐associated variants in *ATG16L1* and *STAT3*, with less frequency in *ECM1* and *NKX2*‐*3*. Second‐generation immigrants exhibited the highest genotypic diversity across all genes, particularly with variants in *ATG16L1* and *STAT3* (Table 2). African Americans showed the highest genetic diversity, predominantly within the second‐generation immigrant group, with significant representation of both *ATG16L1* and *STAT3* (Figure 1).

**Discussion:** These gene variants are known risk alleles for developing IBD in Caucasian and Asian populations. Less is known about the frequency and effect size of these risk alleles in our patient population. Our data shows that they common in patients of African and Caribbean backgrounds, though the degree of effect is unclear. More research is needed to identify the contribution of these markers to genetic risk in non‐Caucasian populations.


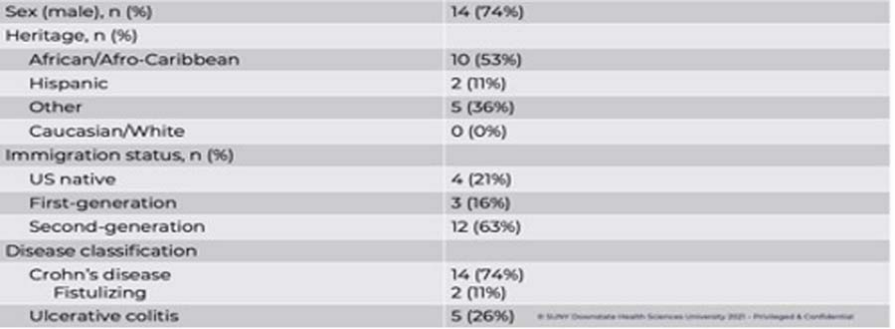


Table 1. Demographics of included patients


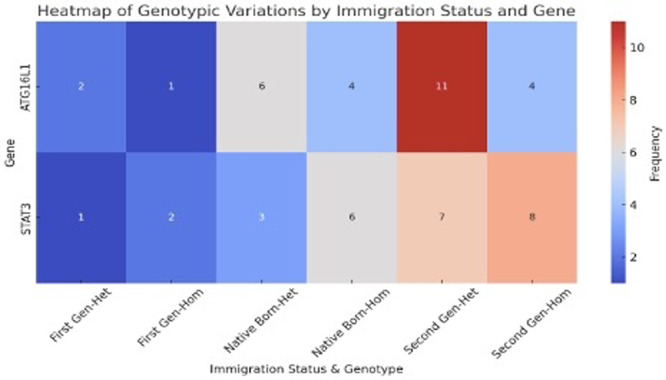


Figure 1. Genotypic variation by immigration status

## 456 COMPARATIVE ANALYSIS OF CLINICAL CHARACTERISTICS AND OUTCOMES BETWEEN CHILDREN WITH VERY EARLY ONSET INFLAMMATORY BOWEL DISEASE (VEOIBD) AND NON‐VEOIBD: A SINGLE‐CENTER EXPERIENCE IN JAPAN

*Taku Kimura*, *Kazuhiro Yasumoto*, *Kazuki Homma*, *Naoya Tsumura*, *Tatsuki Mizuochi*

*Department of Pediatrics and Child Health*, *Kurume University School of Medicine*, *Kurume*, *Fukuoka*, *Japan*

**Background:** Very early onset inflammatory bowel disease (VEOIBD) is often challenging to diagnose and tends to be resistant to treatment.

**Objective:** This study aimed to clarify the clinical characteristics and outcomes of children with VEOIBD by comparing them to those with non‐VEOIBD (diagnosed between 6 and 15 years of age).

**Methods:** We retrospectively reviewed the medical records of pediatric patients (under 16 years old) newly diagnosed with IBD at Kurume University Hospital in Japan between January 2011 and March 2024. The study cohort included ulcerative colitis (UC), Crohn's disease (CD), IBD unclassified (IBDU), and monogenic IBD. We compared disease activity at diagnosis, rates of biologics use and surgeries such as total colectomy and intestinal resection, and mortality between VEOIBD and non‐VEOIBD groups. Disease activity was assessed using the Pediatric Ulcerative Colitis Activity Index (PUCAI) for UC and the Pediatric Crohn's Disease Activity Index (PCDAI) for CD, IBDU and monogenic IBD.

**Results:** A total of 150 patients were included: 86 with UC (median age, 10 years), 53 with CD (12 years), 8 with IBDU (10 years), and 3 with monogenic IBD (0 years). Monogenic IBD included patients with IL10RA deficiency, Hermansky‐Pudlak syndrome type 1, and chronic granulomatous disease. Thirty patients (20%) were classified as having VEOIBD. The proportions of VEOIBD were 27% in UC, 8% in CD, 0% in IBDU, and 100% in monogenic IBD. VEOIBD was significantly more common in UC than in CD (P=0.007). The median PUCAI scores at diagnosis for UC were 35 in the VEOIBD group and 45 in the non‐VEOIBD group, with the latter showing significantly higher disease activity (P=0.049). The median PCDAI scores at diagnosis for CD were 30 in VEOIBD and 37.5 in non‐VEOIBD, with no significant difference (P=0.756). The rates of biologic use and surgery were 47% and 3% in the VEOIBD group, and 61% and 1% in the non‐VEOIBD group, respectively, with no significant differences (P=0.214 and P=0.316). No deaths were observed in either group.

**Conclusions:** VEOIBD accounted for 20% of pediatric IBD patients in our cohort, with a significant higher rate in UC than CD. While disease activity at diagnosis and rates of biologics use and surgery were largely comparable between VEOIBD and non‐VEOIBD, non‐VEOIBD UC patients showed significant higher disease activity at diagnosis. No deaths were observed in this study.

## 460 COMPARATIVE RISK OF INFECTIONS WITH INFLIXIMAB AND ADALIMUMAB IN PEDIATRIC PATIENTS WITH INFLAMMATORY BOWEL DISEASE

*Ning Lyu* ^*1,2*^, *Micheala Tracy* ^*5,3*^, *Sebastian Schneeweiss* ^*1,3*^, *Timothy Savage* ^*1,4*^

^*1*^ *Division of Pharmacoepidemiology and Pharmacoeconomics*, *Brigham and Women's Hospital Department of Medicine*, *Boston*, *MA*; ^*2*^ *Harvard Medical School*, *Harvard‐MIT Center for Regulatory Science*, *Boston*, *MA*; ^*3*^ *Harvard Medical School*, *Department of Medicine*, *Boston*, *MA*; ^*4*^ *Boston Children's Hospital*, *Division of Infectious Diseases*, *Boston*, *MA*; ^*5*^ *Boston Children's Hospital*, *Division of Gastroenterology, Hepatology, and Nutrition*, *Boston*, *MA*

**Background:** The prevalence of pediatric inflammatory bowel disease (IBD)—including Crohn disease (CD), ulcerative colitis (UC)—continues to rise in the U.S., affecting 122 per 100,000 children. Infliximab and adalimumab (and their biosimilars) are the only biologics currently approved by the US FDA for use in children with IBD. Comparative safety data of these medications in children is limited. We compared the risk of infections between infliximab and adalimumab in children with IBD using nationwide real‐world data.

**Methods:** This new‐user, active comparator cohort included children aged 6 to 17 years old with IBD who initiated adalimumab (exposure) or infliximab (referent) in either of two US commercial insurance claims databases, MarketScan (2016‐2023) and Optum Clinformatics (2016‐2024). We excluded patients with prior use of IBD‐related biologics, other autoimmune conditions, and other indications for biologic therapy. Patients were followed for 180 days from treatment initiation to evaluate for the outcomes of serious infections requiring hospitalization (meningitis, osteomyelitis, bacteremia, pneumonia, pyelonephritis, serious gastrointestinal infection, and skin and soft tissue infection) and outpatient infections with receipt of antimicrobial treatment (bacterial, mycobacterial, yeast, and viral infections). Propensity scores with overlapping weights were used for confounding control.

**Results:** We identified 2,907 children with IBD initiating biologic therapy in MarketScan (1,731 infliximab; 1,175 adalimumab) and 1,426 children in Optum (804 infliximab; 622 adalimumab). In both cohorts most subjects were aged 12–17 years (78% in MarketScan; 76% in Optum). Serious infection rates ranged from 20 to 38 per 1,000 person‐years for adalimumab and 30 to 32 per 1,000 person‐years for infliximab (RR: 1.17 [95% CI: 0.64–2.15] in MarketScan, RR: 0.68 [95% CI: 0.22–2.12] in Optum). Outpatient infection rates ranged from 360 to 415 per 1,000 person‐years for adalimumab and 333 to 352 per 1,000 person‐years for infliximab (RR: 1.25 [95% CI: 1.02–1.52] in MarketScan and RR: 1.03 [95% CI: 0.77–1.37] in Optum).

**Conclusions:** In this large national cohort of biologic‐naïve children with IBD initiating infliximab or adalimumab, serious infections were infrequent, and the risks were comparable between infliximab and adalimumab across databases. Outpatient infections were more frequent overall, with no clear difference in risk between treatments with heterogeneity across databases. These findings provide real‐world safety data to support treatment decisions in pediatric patients with IBD.


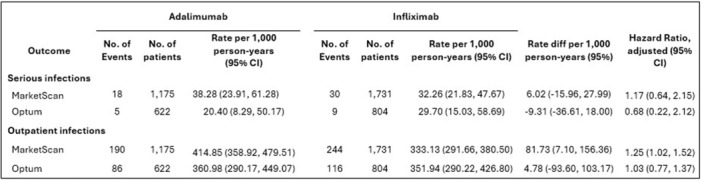


Table 1

## 461 HLA‐DQA1*05 GENOTYPE AND THE RELATIONSHIP WITH ANTI‐TNF TREATMENT FAILURE IN PEDIATRIC PATIENTS WITH INFLAMMATORY BOWEL DISEASE

*Patrick Morency*, *Kristin Capone*, *Jordan Whatley*, *Emily Campbell*, *Carmine Suppa*

*Pediatrics*, *Medical University of South Carolina*, *Charleston*, *SC*

**Background:** Anti‐TNF therapies (infliximab and adalimumab) are cornerstone treatments for moderate‐to‐severe pediatric inflammatory bowel disease (IBD), yet 30% of patients have primary non‐response, with an additional 45% experiencing loss of response within 12 months. Recent adult studies suggest that the HLA‐DQA1*05 allele is associated with anti‐drug antibody (ADA) development and treatment failure. However, this relationship has not been investigated in pediatric IBD populations.

**Methods:** This ongoing pilot study is enrolling pediatric patients (<21 years) with ulcerative colitis, Crohn's disease, or inflammatory bowel disease unclassified (IBD‐U) who have received infliximab or adalimumab therapy and are currently receiving care at the Medical University of South Carolina. Participants are being divided into two groups: those who have failed anti‐TNF treatment (defined as failing to develop an initial response, loss of therapeutic response to the medication, or not tolerating the medication due to side effects, all requiring change to alternate therapy) and those who have maintained successful treatment for at least one year. This study excludes participants who have not received anti‐TNF therapy and those who are responding well to anti‐TNF medication but have taken it for less than one year. Samples are being collected from participants and tested using the Prometheus 'RiskImmune' laboratory test to determine if they are variant carriers of HLA‐DQA105 (rs2097432). These samples are collected during venipuncture for already scheduled standard‐of‐care laboratory work. Clinical data are being extracted from the medical record including demographics, disease characteristics, treatment history, concomitant medications, and treatment outcomes. The primary outcome is the association between HLA‐DQA1*05 genotype and anti‐TNF treatment failure. Secondary outcomes include the relationship between HLA‐DQA1*05 and anti‐drug antibody development. The study aims to enroll 70 participants (40 non‐responders, 30 responders) over 12 months. Statistical analysis will include chi‐square testing to compare categorical variables, descriptive statistics for demographics and clinical characteristics, and logistic regression to assess associations between HLA‐DQA1*05 genotype and treatment outcomes adjusting for potential confounders (age, disease type, and concomitant immunomodulator use).

**Results:** Twenty pediatric IBD patients have been enrolled to date: 13 with anti‐TNF treatment failure (non‐responders) and 7 with treatment success (responders). This preliminary cohort had a mean age of 14.7 years, with 55% female participants and 90% having Crohn's disease. HLA‐DQA1*05 was present in 5/13 (38.5%) patients who failed anti‐TNF therapy compared to 2/7 (28.6%) who responded successfully. Anti‐drug antibodies developed in 5/13 (38.5%) of non‐responders versus 1/7 (14.3%) of responders. Among patients with HLA‐DQA1*05, 1/7 (14.3%) developed anti‐drug antibodies, while 5/13 (38.5%) of HLA‐DQA1*05 negative patients developed antibodies. Full statistical analysis will be completed once the recruitment phase has been completed.

**Discussion:** This pilot study seeks to provide an initial step in understanding the relationship between the HLA‐DQA1*05 allele and anti‐TNF treatment outcomes for pediatric patients. If HLA‐DQA1*05 is found to be associated with treatment failure in children, HLA testing results may eventually be used to guide initial therapy selection or the initiation of adjunctive immunomodulators in the future. Conversely, if it is found that there is no relationship between HLA‐DQA1*05 and anti‐drug antibody formation and treatment failure in the pediatric population, these results would highlight an important difference from the current adult literature.

## 463 OPTIMIZING ANEMIA SCREENING AND TREATMENT IN PEDIATRIC INFLAMMATORY BOWEL DISEASE

*Sydney Kuzoian*, *Viven Solomon*, *Pyae Naing*, *Giselle Davila Bernardy*, *Logan Jerger*

*Gastroenterology*, *Connecticut Children's Medical Center*, *Hartford*, *CT*

**Aim:** To improve the frequency of screening and standardize the treatment of iron deficiency anemia in pediatric inflammatory bowel disease (IBD) at Connecticut Children's.

**Background:** Anemia is common in IBD and is often multifactorial. Screening with laboratory studies is suggested every 3 months for those with active IBD and every 6‐12 months for those with inactive disease. Our division's data showed frequent blood count monitoring but limited use of recommended iron studies. Additionally, the treatment of iron deficiency, once identified, was inconsistent. We sought to improve the frequency of screening and standardize the approach to treatment based on national recommendations and cost‐conscious care.

**Root Causes:** 1) Variable knowledge of national recommendations for the screening and management of anemia in IBD 2) Inconsistent laboratory evaluation for anemia in IBD 3) Varied treatment for iron deficiency anemia in IBD.

**Methods:** We reviewed national recommendations for the screening and treatment of iron deficiency anemia in pediatric IBD and developed best practice suggestions for our GI division. Using Epic SlicerDicer reviewed divisional data for this at‐risk population. Inconsistencies among providers illustrated the need for unified efforts and aided in developing improvements in iron deficiency anemia screening. An Epic order set of the recommended screening labs was created. The project was presented to our division with accompanying education. Our order set became available in February 2024, and we began collecting data on the frequency of screening labs via Epic SlicerDicer; we compared this to data prior to order set implementation. Beginning in December 2024, we instituted an accompanying pathway that summarized the above screening information and the recommended treatment if iron deficiency was identified. Our evidence‐based recommendations included dosing guidelines and follow‐up recommendations. We collected data on the frequency and type of treatment via Epic SlicerDicer and chart review.

**Results:** CBC and CRP testing remained consistent at 100% and 98% pre‐ and post‐intervention (p‐value 0.56 and 0.66 respectively). Iron testing increased from 17% to 25% (p‐value <0.001), ferritin from 26% to 31% (p‐value 0.019), and reticulocyte count from 4% to 7% (p‐value 0.016) among our IBD population. The percentage of patients with low ferritin that were prescribed treatment improved from 58% to 64% pre‐and post‐intervention (p‐value 0.58). Patients with low ferritin who were subsequently treated with IV iron decreased from 77% to 67% (p‐value 0.46) while those prescribed oral iron increased from 23% to 33% (p‐value 0.46). Our results show an overall improvement in iron deficiency screening and subsequent treatment thus far. We suspect the simultaneous decrease in IV iron prescriptions, and increase in oral iron prescriptions, is secondary to our treatment pathway that identified the recommended clinical setting for each modality.

**Act:** Our results demonstrate a modest yet impactful change when considering patient experience, evidence‐based testing, cost‐benefit analysis, and others. There is a continued need for improvement of iron deficiency screening and treatment within our department. We intend to continue data collection alongside regular reminders and education to our providers.

**Recommendations:** Our project illustrates that implementing an order set and treatment pathway can positively influence providers’ laboratory ordering and medication prescribing behaviors. Obstacles in changing providers’ practices exist, however. Additional work is needed to ultimately overcome these obstacles, improve testing/treatment, and impact patient care and outcomes.


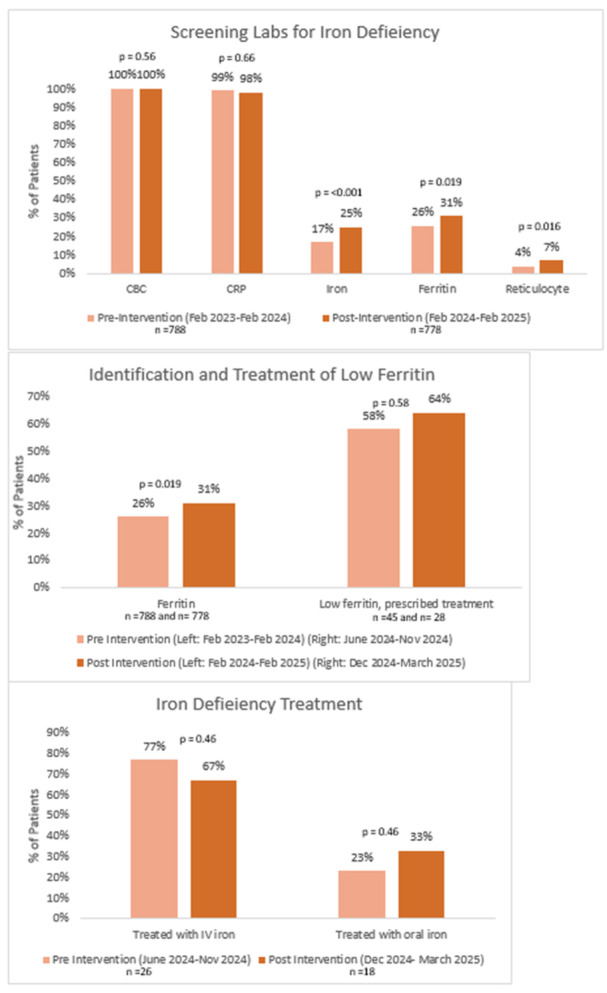


## 464 CYTOMEGALOVIRUS COLITIS IN PEDIATRIC PATIENTS WITH INFLAMMATORY BOWEL DISEASE

*Y. Dana Neugut*, *Lindsey Albenberg*

*The Children's Hospital of Philadelphia*, *Philadelphia*, *PA*

**Background:** Cytomegalovirus (CMV) colitis is a rare but potentially significant cause of refractory colitis in patients with inflammatory bowel disease (IBD). Much remains unknown about the impact of CMV‐mediated colitis in IBD patients, particularly among pediatric populations. Our study aims to explore the utility of endoscopic evaluation in diagnosing CMV colitis and its impact on IBD management in a pediatric population.

**Methods:** This single‐center retrospective cohort study included endoscopic procedures performed on the lower gastrointestinal (GI) tract with CMV mucosal testing, between April 15, 2016, and December 31, 2024, on IBD patients ages 0‐18 years. Electronic medical record data was extracted and validated. Charts were reviewed manually to investigate which patients with CMV positivity received and responded to antiviral medication, and to further characterize these patients.

**Results:** Of a total 428 procedures included in this study (Table 1), thirty‐six (8.4%) had mucosal tissue that tested positive by CMV polymerase chain reaction (PCR), five (1.2%) of which had evidence of CMV on immunohistochemistry (IHC) testing. In total, sixteen (3.7%) patients with positive CMV PCR were trialed on antiviral therapy, and five (1.2%) improved with antiviral therapy and thus completed a multi‐week course; this included three with positive IHC and two with negative IHC staining. These five patients all received concomitant steroids. Their characteristics are summarized in Table 2.

**Conclusion:** This study assesses the prevalence of CMV colitis in pediatric IBD patients presenting with acute colitis symptoms, and response to antiviral treatment, in a large tertiary care center in North America. Of 428 lower GI tract procedures for pediatric IBD patients with endoscopic CMV testing, only five (1.2%) tested positive for CMV colitis and responded well to antiviral therapy. These five patients included those with and without positive IHC testing. These findings suggest that CMV colitis testing may not be necessary as frequently as it is currently performed, and that when testing is performed, CMV PCR may be a reliable indicator for consideration of antiviral treatment. Further research is needed regarding the risks and benefits of endoscopic testing for CMV colitis in symptomatic IBD patients, and can help inform whether current testing practices should be modified.


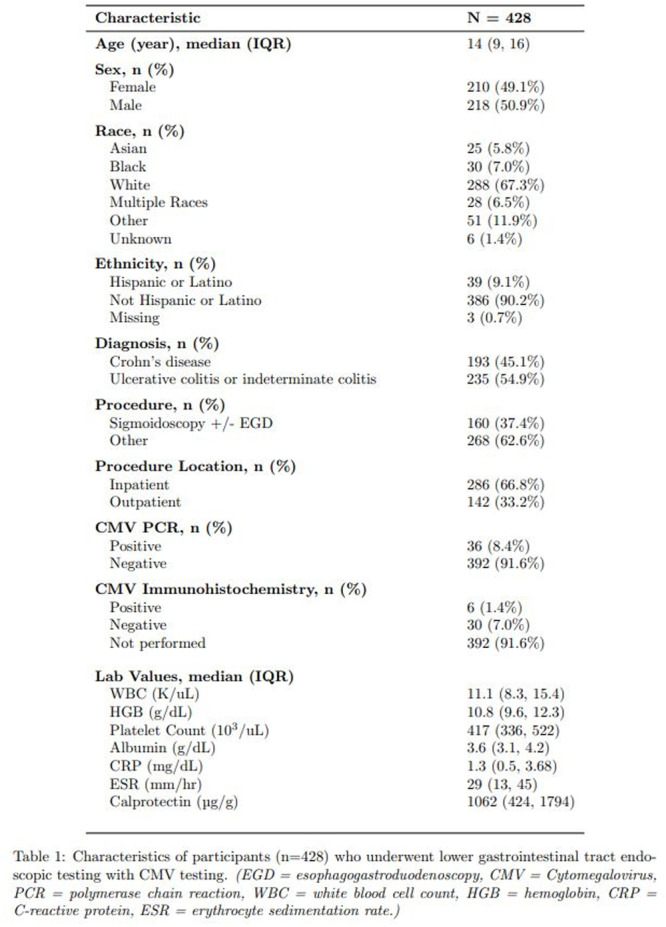



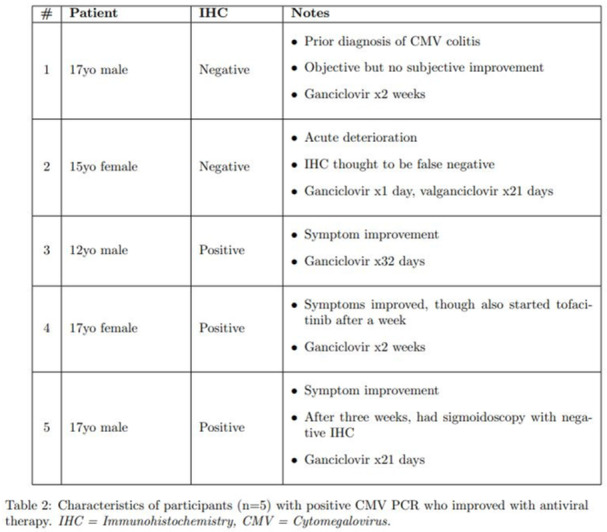


## 468 GLP‐1 RECEPTOR AGONISTS APPEAR TO HAVE PROTECTIVE EFFECTS IN PEDIATRIC INFLAMMATORY BOWEL DISEASE

*Melissa Ramirez Escobar* ^*1*^, *Thomas Wallach* ^*2*^, *Anne Levine* ^*2*^

^*1*^ *Pediatrics*, *SUNY Downstate Health Sciences University*, *New York*, *NY*; ^*2*^ *Pediatric Gastroenterology & Hepatology*, *SUNY Downstate Health Sciences University*, *New York*, *NY*

Introduction: Inflammatory bowel disease (IBD) is a chronic inflammatory intestinal condition driven by immune dysregulation in genetically predisposed individuals. Although traditionally associated with weight loss and malnutrition, as the prevalence of both IBD and obesity in the pediatric population have risen, 20‐30% of pediatric IBD patients are now overweight or obese. Obesity is a pro‐inflammatory condition and has been linked to more severe outcomes in IBD. Effective targeting of inflammation in IBD has been shown to improve both disease progression and symptom management. Glucagon‐like peptide‐1 receptor agonists (GLP‐1RAs) were originally developed for the treatment of type 2 diabetes and obesity. These drugs affect glucose homeostasis through central nervous system pathways, leading to improvement in glycemic control and weight loss. They may also have systemic and gut‐specific anti‐inflammatory properties via immune signaling, maintenance of gut microbiota, and intestinal barrier integrity. However, data on the use of GLP‐1RA in patients with IBD remain limited, particularly in the pediatric population. This study aims to evaluate the impact of GLP‐1 RA on pediatric patients with IBD.

**Methods:** We conducted a retrospective cohort study using the Global Collaborative TriNetX Network. Pediatric patients (age 0–18 years) with a diagnosis of IBD were identified and stratified into two cohorts: those treated with GLP‐1 RA and those not. Clinical outcomes assessed included the incidence of strictures, fistulas, abscesses, hospital admissions, and colonoscopy rates as indirect markers of disease activity. A second retrospective cohort study was conducted using data from the same Network. Pediatric patients (age 0–18 years) with a diagnosis of Overweight or Obesity were identified and stratified into two cohorts: those treated with GLP‐1 RA and those not. Clinical outcomes evaluated included the incidence of UC and CD diagnoses after starting GLP‐1 RA. Propensity score matching was performed to balance cohorts by gender and race, minimizing confounding variables. Kaplan‐Meier survival analysis was used to compare event‐free survival between cohorts, with results reported as hazard ratios (HRs) and corresponding 95% confidence intervals.

**Results:** The first study included 1,631 pediatric patients with IBD treated with GLP‐1 RA and 1,631 IBD patients not receiving a GLP‐1 after propensity score matching by sex and race (White, Asian, African American) for balance. The mean follow‐up duration in the GLP‐1 cohort was 506.97 days (SD 437.29), with a median follow‐up of 399 days. The non‐GLP‐1 cohort had a mean follow‐up of 1,792.21 days (SD 1,582.90) and a median of 1,468 days. There were no hospital admissions or colonoscopies in the GLP‐1 cohort. There were no statistically significant differences between cohorts in the incidence of strictures (HR 1.85; 95% CI 0.10–31.49), abscesses (HR 1.04; 95% CI 0.63–1.70), or fistulas (HR 1.04; 95% CI 0.63–1.70).

The second study included 28,338 pediatric patients with Overweight or Obesity treated with GLP‐1 RA and 28,338 patients with Overweight or Obesity not receiving a GLP‐1 after propensity score matching by sex and race for balance. The mean follow‐up duration in the GLP‐1 analogue cohort was 608 days (SD 604.41), with a median follow‐up of 460 days. The non‐GLP‐1 cohort had a mean follow‐up of 1566 days (SD 1446.37) and a median of 1215 days. GLP‐1 RA use in pediatric obese and overweight patients was associated with a 64.7% lower risk of developing UC (HR=0.353, 95% CI: 0.187‐0.668), and a 54.2% lower risk of developing CD (HR=0.458, 95% CI: 0.256‐0.817) compared with patients not on a GLP‐1.

**Discussion:** To our knowledge, this is the first study to suggest a potential protective effect of GLP‐1 RA in pediatric IBD, as evidenced by a lower incidence of IBD diagnoses among obese and overweight patients on GLP‐1 RA, as well as the absence of hospital admissions and colonoscopies in patients with IBD diagnoses prior to GLP‐1 RA initiation. While the utility of GLP‐1 RA in metabolic disease is well established, their use in pediatric IBD is both controversial and underexplored. Adult data is also inconclusive, with some studies demonstrating no reduction in IBD‐related hospitalization, or clinical and endoscopic response, and others showing a protective effect of GLP‐1 RA. Further prospective, controlled studies are needed to evaluate the true impact of these medications on disease susceptibility, disease activity, and long‐term outcomes in pediatric IBD.

## 470 UNMET SOCIAL NEEDS ARE ASSOCIATED WITH HIGHER RATE OF MISSED MEDICAL APPOINTMENTS IN PEDIATRIC PATIENTS WITH INFLAMMATORY BOWEL DISEASE

*Kayla Pryce* ^*1*^, *Faria Hasan* ^*2*^, *Samantha Levano* ^*3*^, *Kathleen Shea* ^*1*^, *Inessa Normatov* ^*1*^, *Yolanda Rivas* ^*1*^, *Kevin Fiori* ^*3*^, *Gitit Tomer* ^*1*^

^*1*^ *Department of Pediatrics, Division of Pediatric Gastroenterology and Nutrition*, *Albert Einstein College of Medicine*, *New York*, *NY*; ^*2*^ *Kaiser Permanente Fontana Medical Center*, *Fontana*, *CA*; ^*3*^ *Department of Pediatrics, Division of Community & Population Health*, *Albert Einstein College of Medicine*, *Bronx*, *NY*

**Background:** Health‐related social needs (HRSNs) are individual factors, such as housing insecurity, financial instability, and lack of access to healthy food, that put people at risk for worse health outcomes. The goals of this study were to systematically use a novel HRSNs screening tool to identify unmet HRSNs in pediatric patients with inflammatory bowel disease (IBD), assess the prevalence of these HRSNs and their association to missed medical appointments.

**Methods:** This was a pilot study conducted in the outpatient pediatric gastroenterology clinic and infusion center at the Children Hospital at Montefiore in the Bronx, NY, from December 2021 to November 2024. During their visit, patients with IBD were given a standardized 8‐item HRSNs screen. Patients were 21 years old or younger and the screen was completed by parents or guardians. The results were entered into electronic health records. The primary outcome was the number of missed medical appointments one year prior to the screen. Negative binomial regression models adjusting for the number of completed medical appointments as an offset were used. Bivariate and multivariate models without and with adjustment for socio‐demographic characteristics were used.

**Results:** A total of 191 patients with IBD were screened. The median age of participants at the time of screening was 16 years, and most patients were male (68.59%). Of the 191 patients, 34 patients (17.8%) had a positive HRSNs screen; 18 patients had one HRSN and 16 patients had 2 or more HRSNs. Out of 191 patients with IBD, 184 patients had at least one completed medical appointment in the year prior to the HRSNs screen, 54% (99/184) had 1 or more missed appointments. 46% (85/184) had 0 missed appointments. Patients with HRSNs had a 63% (IRR: 1.63, 95% CI: 1.11, 2.38) higher rate of missed medical appointments compared to patients without HRSNs (p=0.0121). HRSNs were significantly associated with missed medical appointments after adjusting for race/ethnicity, age and sex (IRR: 1.56, 95% CI: 1.07, 2.28, p=0.0212). Bivariate analysis revealed that having 2 or more HRSNs was significantly associated with missed medical appointments (IRR: 1.936, 95% CI: 1.25, 3.00, p=0.0032). Inadequate housing (8.38%), followed by food insecurity (6.81%) were the most prevalent risk factors in our pediatric population. Our bivariate analysis also showed that unmet needs related to utilities (p=0.0013), transportation (p=0.0014) and healthcare cost (p=0.0282) were also significantly associated with missed appointments.

**Conclusions:** In our cohort, HRSNs were significantly associated with the higher rate of missed medical appointments. Future research should aim to develop strategies that address specific HRSNs as well as personalized interventions tailored to individual patient circumstances.

## 472 PEDIATRIC GASTROENTEROLOGISTS PRACTICE STYLES AND ADHERANCE TO INTERNATIONAL GUIDELINES FOR PEDIATRIC CELIAC DISEASE

*Ashwin Agrawal* ^*1,2*^, *Ahmed Elhatw* ^*2*^, *Jonathan Teitelbaum* ^*1,2*^

^*1*^ *Pediatric Gastroenterology*, *RWJBarnabas Health*, *Eatontown*, *NJ*; ^*2*^ *Department of Pediatrics*, *Monmouth Medical Center*, *Long Branch*, *NJ*

**Introduction:** There has been a concerted effort by organizations to create consensus and evidence‐based guidelines for the diagnosis and management of pediatric celiac disease (CeD). In 2016, NASPGHAN published a clinical report on CeD. In 2020, ESPGHAN published its updated CeD guideline.

To date, there has been no study assessing pediatric gastroenterologists’ adherence with national/international guidelines. This study aims to assess pediatric gastroenterologists’ adherence to NASPGHAN/ESPGHAN guidelines to diagnose CeD, as well as elucidate clinical practices about diagnosis and treatment of CeD.

**Methods:** An anonymous survey with 26 questions was designed to assess practice areas of CeD care and compare responses to guidelines by NASPGHAN/ESPGHAN. The survey was distributed via the listserve for pediatric gastroenterology providers pedgi@list.uvm.edu. The survey was open for responses for 4 weeks between January 14, 2025 and February 11, 2025.

**Results:** 203 respondents completed the survey. Most responses were from the United States of America (USA) (72.9%); however, there were respondents from 27 unique countries. 78.% of respondents were attending physicians, 9.9% advanced practice providers, and 11.3% fellows/trainees. 36.5% had more than 15 years of practice while 25.1% had 1‐4 years of experience. 81.8% worked at tertiary or quaternary care centers.

Selected results of the survey are presented in Table 1. Almost all clinicians biopsied the second portion of the duodenum (99.0%) and the duodenal bulb (98.5%). When taking biopsies of the distal duodenal 93.6% took 3 or more biopsies. In the proximal duodenum, 72.4% of clinicians took 2 biopsies, 15.3% took 4 biopsies, 6.9% took 3 biopsies, 3.9% took one biopsy, and 1.5% took more than 4 biopsies.

For a gluten challenge prior to testing for CeD, NASPGHAN recommends 3‐6 grams of gluten, while ESPGHAN recommends 15 grams or more gluten. Respondents recommended 15 or more grams daily (47%), 10‐14 grams (36%), 5‐9 grams (31%), and 0‐4 grams (9.9%).

43.3% of respondents utilized the NASPGHAN 2016 guidelines while 47.3% of respondents utilized the ESPGHAN 2020 guidelines. 6.9% of respondents stated they do not follow either of the guidelines. Subgroup of respondents in the USA showed 54.7% used the NASPGHAN 2016 guidelines, 33.1% utilized the ESPHGAN 2020 guidelines, 9.5% did not use either guideline and 2.7% used a combination. For pediatric gastroenterologists practicing outside of the USA, 12.7% utilized NASPGHAN 2016 guidelines, 85.5% utilized ESPGHAN 2020 guidelines.

**Conclusion:** Our study highlights the adherence to guidelines and practices among pediatric gastroenterologists in managing CeD. There was good adherence to the use of TTG IgA testing for screening of CeD, however, disagreement in what age is considered a young child for additional testing. Good adherence is also found with typical follow‐up intervals for a child with CeD. There was a diversity of opinions of what is considered IgA deficiency. Most providers adhered to 10 grams or more of gluten for gluten challenge as suggested by ESPGHAN and few following NASPGHAN recommendations.

Majority of non‐USA respondents follow ESPGHAN 2020 guidelines. Interestingly, one third of USA pediatric gastroenterologists also followed ESPGHAN 2020 guidelines.

The use of non‐biopsy diagnosis when TTG IgA > 10x UNL is low in the USA with 7.5% respondents not often or never performing endoscopy compared to 45.4% of non‐USA respondents. However, USA respondents that always perform endoscopy in this situation is also low at 32.4% indicating many USA providers are not consistently following NASPGHAN recommendations.

Our study suggests that ESPGHAN guidelines are more widely utilized internationally. In addition, only half of USA providers refer to NASPGHAN recommendations. Further studies are needed to investigate why NASPGHAN guidelines are less utilized to help improve consistent evidence‐based care for CeD in children.


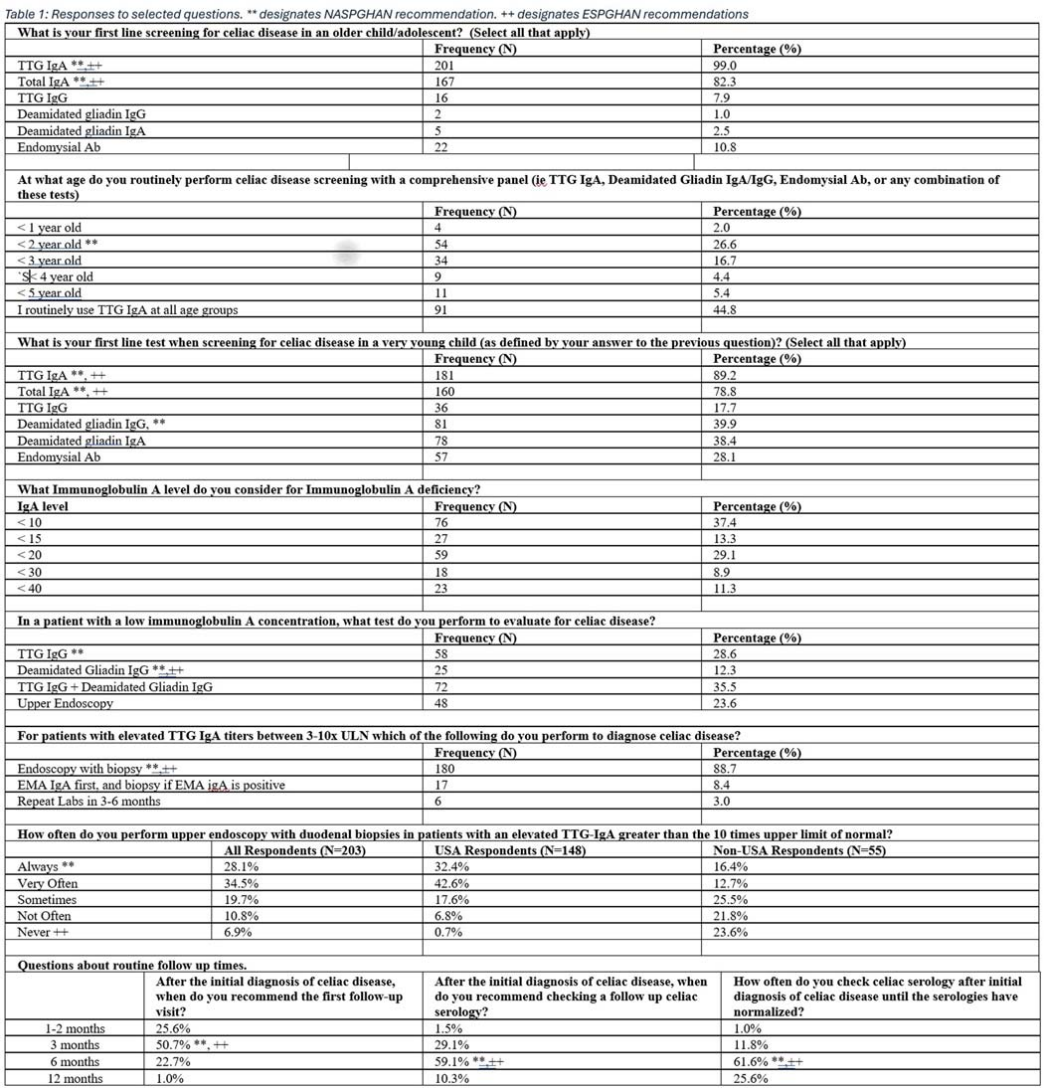


## 473 TRANSITION OF CELIAC DISEASE CARE FROM PEDIATRICS TO ADULTHOOD: A SINGLE‐CENTER EXPERIENCE

*Ebtihal Ahmed*, *Mohammad karam Chaaban*, *Joel A Hickman*, *Roland C Hentz*, *Imad Absah*

*Pediatric*, *Mayo Clinic Minnesota*, *Rochester*, *MN*

**Background:** Celiac disease (CeD) is a chronic autoimmune condition with both intestinal and extraintestinal manifestations, requiring lifelong adherence to a gluten‐free diet and ongoing medical follow‐up. Timely and structured transition of care from pediatric to adult services is essential to optimize outcomes and prevent long‐term complications. However, prior studies suggest that the transition process in North America is often suboptimal. This study aims to evaluate the transition of care practices for pediatric celiac disease patients at our institution.

**Methods:** We conducted a retrospective chart review of patients diagnosed with CeD before the age of 18 who reached adulthood (>18 years) between 2000 and 2025 at the Mayo Clinic. Demographic data, laboratory results, pathology reports, and clinical documentation were analyzed. Patients were categorized into three groups 1) Transitioned: Patients followed by an adult gastroenterologist (GI) or primary care provider (PCP) with documented CeD care, 2)Non‐transitioned: Patients followed by PCPs with no documentation of CeD care and, 3) Lost to follow‐up: Patients with no available adult health records at our institution. We compared the recurrence of symptoms and positive serology between those transitioned to adult GI vs PCP. Non‐transitioned patients were excluded from this comparison due to a lack of documented celiac‐related care.

**Results:** a total of 98 patients met the inclusion criteria. The mean age at CeD diagnosis was 11.16 years (SD 4.14); 93% were non‐Hispanic White and 60% were female. Among these, 48 patients (49%) had documented transition of care (20 to adult GI, 28 to adult PCP), 30 were seen by a PCP with no documented CeD care, and 20 were lost to follow‐up.

Among the transitioned cohort, the mean duration from CeD diagnosis to transition was 8.0 years (SD 4.4). The average age at first adult CeD‐related visit was 21 years (SD 3.0). Fifteen transitioned patients (31%) experienced symptom recurrence, and 9 (19%) had recurrent positive celiac serology. There was a trend toward higher recurrence rates among patients followed by adult GI compared to those followed by PCPs. Factors such as age at diagnosis, sex, and family history of CeD were not associated with the type of provider overseeing adult care. Repeat small bowel biopsy was performed in 9 of 20 (45%) patients transitioned to adult GI; two patients underwent biopsies as part of a clinical trial. Transitioning to adult GI, the likelihood of being recruited into CeD clinical trial is higher if patients transitioned to adult GI care.

**Conclusion:** Fewer than half of pediatric CeD patients continued to receive documented celiac‐related care into adulthood. Recurrence of symptoms and positive serology was common, underscoring the need for structured and consistent follow‐up. Our findings highlight the importance of improving the transition process to ensure continuity of care and optimize long‐term outcomes for patients with celiac disease.

Table 1. Comparison Between CeD Patients Transitioned to Adult GI vs Primary Care

Variable Adult GI (n=20) Primary Care (n=28)

Demographics

Female, n (%) 13 (65%) 18 (64%)

White, n (%) 19 (95%) 26 (92%)

Celiac Disease History

Age at diagnosis, mean (SD) 13.3 (3.4) 11.7 (4.0)

Age at transition, mean (SD) 21.3 (3.0) 22.5 (3.6)

Time from diagnosis to transition, mean (SD) 8.0 (4.4) 10.5 (4.5)

Age at most recent care, mean (SD) 24.5 (4.4) 23.1 (3.6)

Family history of celiac disease, n (%) 9 (45%) 14 (50%)

Follow‐Up and Disease Activity

Recurrence of symptoms, n (%) 9 (45%) 6 (21.4%)

Recurrence of positive serology, n (%) 6 (37.5%) 3 (25%)

Repeat biopsy performed, n (%) 9 (45%) 0 (0%)

Declaration of Generative AI and AI‐assisted technologies in the writing process:

Statement: During the preparation of this work the author(s) used ChatGPT in order to fotmat the table. After using this tool/service, the author(s) reviewed and edited the content as needed and take(s) full responsibility for the content of the publication.

## 474 ENHANCING FOOD INSECURITY SCREENING IN PEDIATRIC CELIAC DISEASE USING A QUALITY IMPROVEMENT FRAMEWORK

*Telly Cheung*, *Mala Setty*, *Erica Riray*, *Bradley Green*, *Sharad Wadhwani*, *Namrata Patel‐Sanchez*

*Pediatrics*, *University of California San Francisco*, *San Francisco*, *CA*

**Background:** Food insecurity (FI) affects more than 18 million households in the United States. Children with celiac disease (CeD) depend exclusively on the gluten‐free diet (GFD), yet gaps persist in screening for food‐insecure households who could benefit from interventions. We developed a system to identify and address barriers to FI screening in a pediatric clinic for CeD.

**Methods:** Using the Model for Improvement, a theoretical framework for designing and implementing changes, we conducted quality initiatives for households presenting in‐person to the Celiac Disease Clinic at Benioff Children's Hospital Oakland between 1/1/2024‐1/31/2025. We surveyed the medical team to identify barriers to FI screening and designed a key driver diagram to inform plan‐do‐study‐act cycles. Our primary drivers included identifying children with CeD in clinic, assessing FI risk, integrating FI screening into workflow, and providing GFD resources to at‐risk households. We implemented a 1) validated screening form, 2) clinic flowsheet, 3) educational module, and 4) visual aid in the electronic health records (EHR). We measured the process of FI screening and documentation. We tracked outcomes on positive screens for general and gluten‐free FI.

**Results:** Among 100 eligible children, the majority were female (63.0%), Other race (55.0%), non‐Hispanic (66.0%), and primary English‐speakers (80.0%); and 66 received FI screening (66.0%). We identified top team‐reported barriers to FI screening: language barriers, lack of FI resources, forgetting, and discomfort with asking questions. Applying the Model for Improvement, we improved median FI screening from 0.0% to ≥75.0% within 12 months and sustained screening rates for ≥6 months. Median screening rates increased from 0.0% to 60.0% within 1 month of introducing the patient FI screening form; to 80.0% within 2 months of creating a clinic flowchart and educational module; and to 100.0% within 2 months of incorporating closed loop communication for each intervention and establishing a visual EHR reminder. The average general and gluten‐free FI among screened households was 19.7% and 14.8%, respectively.

**Conclusion:** We implemented a systematic framework to address team‐reported barriers, helping to increase FI screening for children with CeD. Developing interventions to address FI may improve poor outcomes among food‐insecure households.


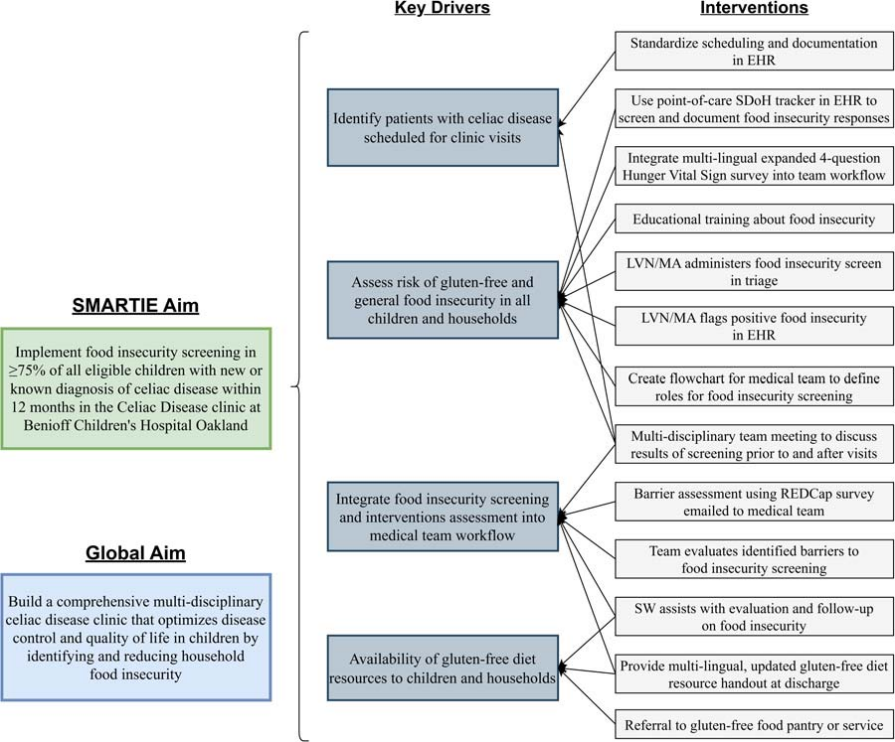


Key driver diagram represents the theory of change for improving food insecurity screening contextualized by a specific, measurable, achievable, relevant, time‐bound, inclusive, and equitable (SMARTIE) aim.

*Electronic health records (EHR), social determinants of health (SDoH), licensed vocational nurse (LVN), medical assistant (MA), social worker (SW)


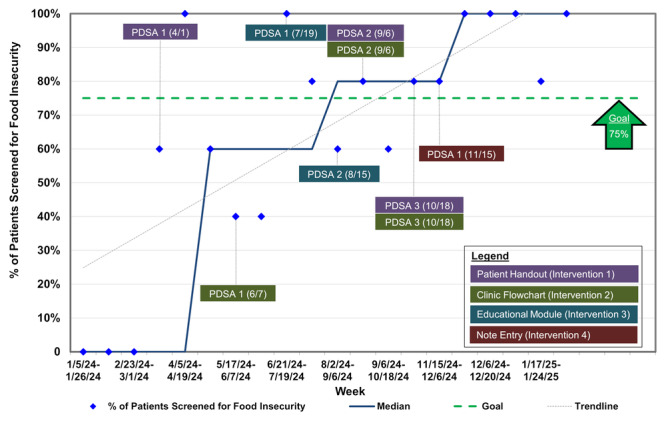


Run chart displays the implementation of initiatives to improve food insecurity (FI) screening between 1/1/2024‐1/31/2025. Each point represents the percentage of children (per 5) screened and documented. The median line shifted every 5 points. The dotted line and green arrow indicate the goal rate of ≥75.0%. **Patient handout (purple):** Plan‐Do‐Study‐Act (PDSA) 1 – introduction, PDSA 2 – printed on yellow‐colored paper, PDSA 3 – included signatures. **Clinic flowchart (green):** PDSA 1 – introduction, PDSA 2 – added inclusion criteria and roles, PDSA 3 – setup validation steps. **Educational module (blue):** PDSA 1 – shared recording, PDSA 2 – initiated huddles. **Note entry (brown):** PDSA 1 – launched visual cue.

## 475 SEBELIPASE ALFA IMPROVES LIVER AND LIPID PARAMETERS IN PATIENTS WITH LYSOSOMAL ACID LIPASE DEFICIENCY: RESULTS FROM THE INTERNATIONAL LAL‐D REGISTRY

*Lorenzo D'Antiga* ^*1,2*^, *Don Wilson* ^*3*^, *Jennifer Evans* ^*4*^, *Florian Abel* ^*4*^, *Emilio Ros* ^*5*^, *William Balistreri* ^*6*^

^*1*^ *Department of Medicine and Surgery*, *University of Milano–Bicocca*, *Milan*, *Italy*; ^*2*^ *Pediatric Hepatology, Gastroenterology and Transplantation*, *Hospital Papa Giovanni XXIII*, *Bergamo*, *Italy*; ^*3*^ *Endocrinology*, *Cook Children's Medical Center*, *Fort Worth*, *TX*; ^*4*^ *Alexion, AstraZeneca Rare Disease*, *Boston*, *MA*; ^*5*^ *Institut d'Investigacions Biomèdiques August Pi Sunyer (IDIBAPS)*, *Barcelona*, *Spain*; ^*6*^ *UC Department of Pediatrics*, *Cincinnati Children's Hospital Medical Center*, *Cincinnati*, *OH*

**Objective and Study:** Liver transaminase abnormalities and dyslipidemia are common in patients with lysosomal acid lipase deficiency (LAL‐D). This study examined alanine and aspartate aminotransferase (ALT, AST) and low‐ and high‐density lipoprotein cholesterol (LDL‐C, HDL‐C) levels in pediatric patients to determine prevalence and severity of baseline abnormalities and to report results over 3 years follow‐up among those treated with sebelipase alfa enzyme replacement therapy.

**Methods:** This observational study included patients diagnosed with LAL‐D before 18 years of age who were enrolled in the International LAL‐D Registry (NCT01633489), excluding those who initiated treatment before 6 months of age. Baseline liver and lipid measures were assessed. A subanalysis was performed among patients with baseline data and 3 consecutive annual follow‐up assessments. Descriptive results are presented.

**Results:** As of October 2024, 99 patients were included (41% female, 94% White, 9.2 year median age at baseline). Baseline ALT and AST levels were above the upper limit of normal for most patients: 81/86 (94%) for ALT and 75/83 (90%) for AST. Baseline LDL‐C was >130 mg/dL (median: 210 mg/dL) for 69/80 (86%) patients. Baseline HDL‐C was below the lower limit of normal for 61/78 (78%) patients. Among 29 treated patients with baseline data and 3 consecutive annual follow‐up measures, few patients had ALT, AST, LDL‐C, and HDL‐C within normal limits at baseline (7%, 7%, 10%, 10%, respectively); results improved over the first year (45%, 45%, 35%, 28% of patients, respectively, had normal values), with benefits sustained over 3 years. Most patients (66%) had ≥1 adverse event; most AEs were mild/moderate. One death occurred during follow‐up (roadway accident).

**Conclusions:** Baseline liver and lipid measures were abnormal among most patients with LAL‐D diagnosed at <18 years of age. Liver and lipid parameters improved in a large proportion of patients receiving sebelipase alfa over 3 years.


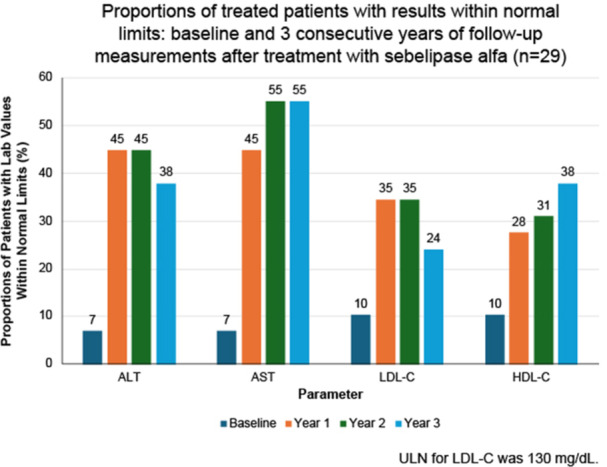


## 478 ESTIMATING HEALTH UTILITIES IN PEDIATRIC INTESTINAL FAILURE

*Daniela Gattini* ^*1*^, *Vikram Raghu* ^*2*^, *Varsha Lilman* ^*3*^, *Lisa Lakkis* ^*4*^, *Jessie Hulst* ^*1*^, *Yaron Avitzur* ^*1*^, *Wendy Ungar* ^*3*^, *Paul W Wales* ^*5*^

^*1*^ *Paediatrics*, *The Hospital for Sick Children*, *Toronto*, *ON*, *Canada*; ^*2*^ *UPMC*, *Pittsburgh*, *PA*; ^*3*^ *The Hospital for Sick Children*, *Toronto*, *ON*, *Canada*; ^*4*^ *UPMC*, *Pittsburgh*, *PA*; ^*5*^ *Cincinnati Children's Hospital Medical Center*, *Cincinnati*, *OH*

**Background:** Health utilities, a preference‐based measure of health‐related quality of life (HRQoL) required to conduct cost‐utility analyses, are lacking in children with intestinal failure (IF). Current economic evaluations extrapolate utilities from adults which may not accurately reflect the pediatric population. This study aims to estimate health utilities in children with IF on parenteral nutrition (PN) compared to children with IF that have achieved enteral autonomy (EA). Secondary objectives were to compare the non‐preference based generic and disease specific HRQoL between children with IF on PN and children with IF that achieved EA.

**Methods:** Children with IF aged 5‐17 years were recruited from two large intestinal rehabilitation centers in North America. Patients were excluded if they had other medical conditions that could directly impact health‐related quality of life (HRQoL), such as an independent life‐limiting condition, severe developmental delay, mental health disorders, intercurrent illness, recent hospital admission, other organ failure, and status post transplantation. Preference‐based HRQoL instruments were administered by interview to 50 children on PN and 57 children that achieved EA. Proxy‐assessments were conducted for children aged 5‐7 years, while self‐assessments were used for children aged 8‐17 years. Utilities, ranging from 0 (death) to 1 (perfect health), were calculated using the Child Health Utility 9 dimensions (CHU9D) questionnaire and the Health Utilities Index (HUI). Non‐preference based generic and disease specific HRQoL between groups were estimated using the Pediatric Quality of Life Inventory^TM^ (PedsQL^TM^) Generic Core Scales and the PedsQL^TM^ Gastrointestinal Symptoms Module, respectively. Differences in mean utilities and non‐preference based HRQoL between PN and EA groups were assessed with t‐test. An alpha value of <0.05 was considered statistically significant. A Minimal Clinically Important Difference (MCID) of 0.03 was considered significant when comparing overall utility scores between different groups based on previously reported MCID values for the HUI.

**Results:** For children aged 5‐7 years, mean utilities on PN were 0.735 (SD 0.208), while those who achieved EA had mean utilities of 0.821 (SD 0.169), with no statistically significant differences (p=0.269). For children aged 8‐17 years, mean utilities on PN were 0.789 (SD 0.186), and for EA, 0.836 (SD 0.140), with no statistically significant differences (p=0.375). However, while the differences between groups were not statistically significant, they were considered clinically significant as per previously established MCID.

**Conclusion:** This multicenter study involving 107 children with IF provides the first estimation of pediatric health utilities. Estimated utilities for children with IF on PN are notably higher than published adult estimates (0.36 [SD 0.35]), while utilities of children that achieved EA align with adult estimates (0.82 [0.22]). These findings are crucial for advancing economic evaluations in pediatric IF.

## 479 DIAGNOSING CONGENITAL SUCRASE‐ISOMALTASE DEFICIENCY IN CHILDREN: AN ALGORITHM USING COMBINED BREATH TESTING

*Jeanette Freeman* ^*3*^, *Wikrom Karnsakul* ^*2*^, *Shaija Kutty* ^*2*^, *Steven Miller* ^*2*^, *Jennifer Smith* ^*2*^, *Ann Scheimann* ^*2*^, *Brett Hoskins* ^*1*^

^*1*^ *Division of Pediatric Gastroenterology, Hepatology, and Nutrition, Department of Pediatrics*, *Indiana University School of Medicine*, *Indianapolis*, *IN*; ^*2*^ *Division of Pediatric Gastroenterology, Hepatology, and Nutrition, Department of Pediatrics*, *The Johns Hopkins University School of Medicine*, *Baltimore*, *MD*; ^*3*^ *Department of Medicine*, *The Johns Hopkins University School of Medicine*, *Baltimore*, *MD*

**Background:** Disaccharidase deficiencies, including sucrase‐isomaltase deficiency, can cause chronic gastrointestinal symptoms in children. While duodenal biopsies remain the diagnostic gold standard, results may be confounded by specimen handling variability or secondary mucosal injury. The noninvasive ^13^C sucrose breath test (^13^CSBT) accurately detects sucrase deficiency, including congenital sucrase‐isomaltase deficiency (CSID), while the Trio‐Smart® breath test (BT) identifies small intestinal bacterial overgrowth (SIBO), a potential cause of secondary enzyme deficiency.

**Methods:** We conducted a retrospective review of 25 pediatric patients with disaccharidase deficiencies on duodenal biopsy and normal villous architecture. Patients underwent ^13^CSBT and/or Trio‐Smart® BT to evaluate for true CSID or SIBO. Clinical outcomes and treatment responses were assessed.

**Results:** Of 21 patients with low sucrase activities who completed ^13^CSBT, only 7 (33%) had abnormal results consistent with CSID. Six patients received sacrosidase, with three reporting symptom improvement. Of 15 patients who underwent Trio‐Smart® breath testing, 9 (60%) had abnormal results suggestive of SIBO and responded to antimicrobial treatment. Two patients had abnormal results on both tests. Interestingly, low palatinase levels were associated with abnormal ^13^CSBT in some cases, though not consistently.

**Conclusion:** Biopsy‐based diagnosis may overestimate true CSID due to secondary causes or technical artifacts. Combined use of the ^13^CSBT and Trio‐Smart® BT provides a noninvasive strategy to distinguish primary or secondary sucrase deficiency, improving diagnostic accuracy and avoiding unnecessary lifelong enzyme therapy. We propose a diagnostic algorithm that integrates biopsy and BT results to guide evaluation and reduce misclassification of CSID.


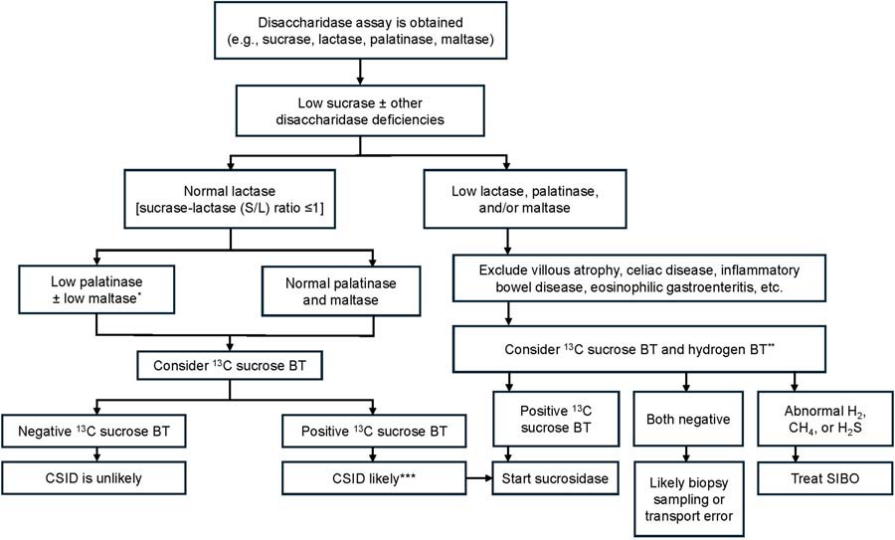


Figure: Diagnostic algorithm for sucrase deficiency: Distinguishing CSID from secondary causes.


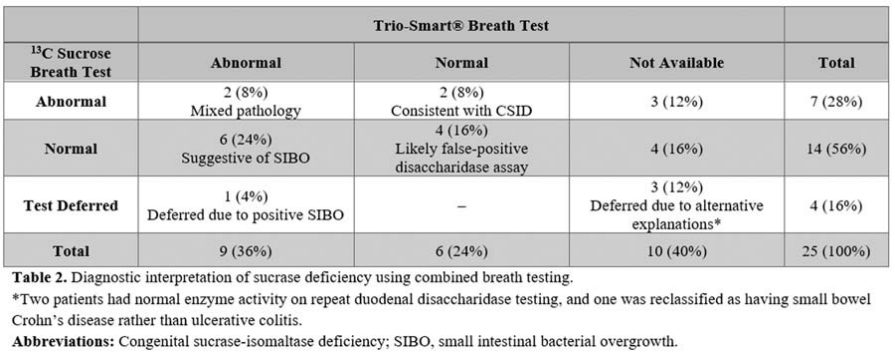


Table: Diagnostic interpretation of sucrase deficiency using combined breath testing.

## 480 DEVELOPING A NOVEL PEDIATRIC INTESTINAL FAILURE DISEASE ACTIVITY INDEX

*Juliana Kennedy* ^*1*^, *Joanne Lai* ^*1*^, *Kishore Iyer* ^*3,2*^

^*1*^ *Pediatric Gastroenterology*, *Icahn School of Medicine at Mount Sinai Department of Pediatrics*, *New York*, *NY*; ^*2*^ *Surgery*, *Icahn School of Medicine at Mount Sinai*, *New York*, *NY*; ^*3*^ *Recanati/Miller Transplantation Institute at Mount Sinai*, *New York*, *NY*

**Introduction:** At this time, there is no objective short or medium‐term measure of pediatric intestinal failure (IF) disease activity. Surrogate measures we sometimes use such as freedom from parenteral nutrition (PN), hospitalizations, survival etc., do not apply to all IF patients and may not capture the full spectrum of disease. We aim to create a pediatric IF disease activity index (PIFDAI) using clinical measures that can be scored by any clinician at the point of care. The PIFDAI score will correlate well with underlying phenotypic disease severity and will be sensitive to short‐ and medium‐term changes in disease activity.

**Methods:** The PIFDAI is currently being developed via the Delphi method. The multi‐stage anonymous surveys in the Delphi process allow for expert opinions to form group consensus. International expert participants were recruited from various intestinal rehabilitation (IR) networks and the global IR program contact list (as gathered by D. Galloway).

A web‐based online survey platform, Welphi.com, is hosting the Delphi rounds to ensure anonymity and help streamline analysis of survey results between rounds. The primary research team created a list of suggested criteria to start the first Delphi round. The Wilcoxon signed rank test is used between rounds to test the stability of the results for each criterion.

Entry into the first online Delphi survey served as voluntary consent for participation. This study was determined to be exempt from formal review by the Mount Sinai Institutional Review Board (STUDY‐23‐01159).

**Results:** A diverse cohort of 43 pediatric IF experts were recruited to participate. The participants include physicians (20), registered dieticians (5), nurses (8), pharmacists (2), a patient (1), parents of pediatric patients (5), and IF patient advocates (2) from IR programs in the US (38) and abroad (5). Participants were verified as experts by our lead research team.

Delphi rounds 1‐4 were conducted between December 2024 and May 2025. Round 1, with an 81% response rate, allowed participants to provide open ended comments on the list of suggested criteria and contribute recommendations for additional criteria. In the second round, with an 81% response rate, participants used a Likert scale to provide their opinion on if the criterion should be included. Round 3, with an 84% response rate, was the second rating round where participants were asked to solidify their opinions using the Likert scale. Round 4 had a 79% response rate and presented participants with the criteria not yet meeting consensus for inclusion or statistical stability. In addition to the Likert scales used during rounds 2‐4, participants also had the opportunity to provide comments and feedback on the criteria. At the end of round 4, the PIFDAI is poised to include 13 metrics for IF disease activity (Table 1).

**Conclusion:** While the PIFDAI is still being developed, the preliminary Delphi rounds reveal 13 criteria to be included based on iterative expert consensus. The next rounds will proceed with complex multi‐criteria decision analysis to develop scales and values for each criterion. We anticipate that some criteria will hold more weight (i.e. contribute more points to the disease activity index) than others. The Delphi rounds are expected to conclude by NASPGHAN's annual meeting 2025. Following the Delphi process, the final version will then undergo validation at multiple IR programs.


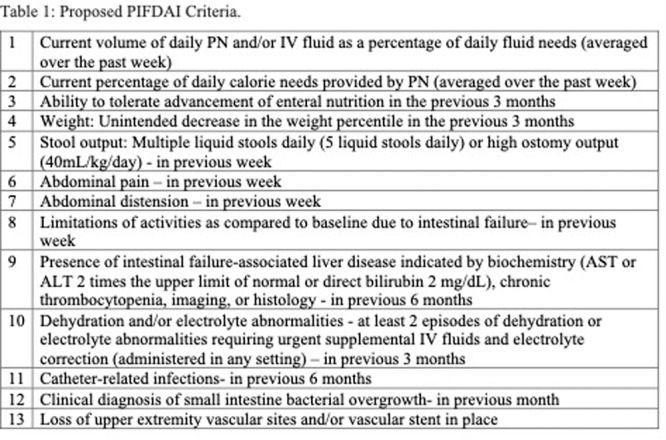


Table 1: Proposed PIFDAI Criteria

## 483 OUTCOMES OF CATHETER SALVAGE THERAPY FOR CENTRAL LINE INFECTIONS IN PEDIATRIC PATIENTS WITH SHORT BOWEL SYNDROME ON PARENTAL NUTRITION

*Lubna Rahman*, *Lubna Rahman*, *Joanne Thio*, *Sharef Al‐Mulaabed*, *khaled bittar*

*Pediatric Gastroenterology*, *Orlando Health Arnold Palmer Hospital*, *Clermont*, *FL*

**Background:** Children with short bowel syndrome (SBS) often rely on parenteral nutrition (PN) due to inadequate absorption of nutrients from the gastrointestinal tract. Central venous lines (CVLs) are critical for long‐term PN delivery. However, CVLs increases the risk of central line‐associated bloodstream infections (CLABSIs), which can be severe and even life‐threatening. In SBS, bacterial translocation is thought to contribute to recurrent CVL infections. CLABSIs in SBS patients contribute to morbidity, hospital readmissions, and increased healthcare costs. Repeated CVL replacements due to infections can lead to challenges in future venous access, a critical factor for patients requiring intestinal transplantation. Consequently, international guidelines recommend a catheter‐salvage strategy in select cases to preserve venous access.

When clinically safe, catheter salvage using systemic antibiotics and antibiotic lock therapy may avoid the risks associated with surgical CVL replacement, such as pneumothorax or venous thrombosis, while preserving future venous access. Our study aims to explore the long‐term outcomes of catheter salvage therapy in pediatric patients with SBS, an area where research remains limited.

**Objective:** To evaluate the short‐ and long‐term outcomes of catheter salvage therapy in managing central line‐ associated bloodstream infections in patients with short bowel syndrome, with a focus on infection, relapse and recurrence rates

**Methods:** This is a retrospective study design. The study includes the analysis of data on management of catheter‐related bloodstream infections in pediatric population 0‐21 yrs with SBS patient on TPN over the past 6 years between 2018 to 2024. Catheter salvage will be defined as successful treatment of the infection with antibiotics while retaining the central venous catheter (CVC) at discharge. Repeated infections caused by the same microbial species will be categorized as relapses if they occur within 30 days or as recurrent infections if they occur between 30–100 days.

**Results:** Out of 104 patients with SBS on TPN, 84 infections were documented, with 42 managed using salvage therapy and antibiotics instead of line removal. None of the salvage therapy cases experienced relapse within 30 days, while recurrence within 100 days occurred in 8 patients (16%). In comparison, patients who underwent line removal had no instances of relapse or recurrence.

**Conclusion:** Catheter salvage therapy demonstrated excellent outcomes in managing CLABSIs in pediatric SBS patients, with zero relapse within 30 days and a recurrence rate of only 16% within 100 days. In contrast, line removal eliminated relapse and recurrence but sacrificed critical venous access. These results highlight the importance of salvage therapy as a preferred strategy for balancing effective infection management and the preservation of central venous access. Preserving venous access is particularly vital for patients with SBS who are dependent on long‐term TPN and may require future intestinal transplants, where access options are already limited. Salvage therapy not only reduces the burden of invasive procedures but also aligns with long‐term goals of maintaining vascular access essential for life‐saving interventions.

## 484 COST COMPARISON OF GLUTEN‐FREE AND GLUTEN‐CONTAINING FOODS IN MAJOR GROVERY STORE CHAINS ACROSS THE UNITED STATES

*Annemarie Rompca* ^*1,2*^, *Katelynn Ho* ^*1,2*^, *Timothy Adamos* ^*1,2*^, *Kai Kokesh* ^*3*^, *Iman Ahmad* ^*3*^, *Aadya Syal* ^*4*^, *Erin Walsh* ^*4*^, *Angela Zhang* ^*4,2*^, *Dale Lee* ^*1,2*^

^*1*^ *Pediatric Gastroenterology, Hepatology, and Nutrition*, *Seattle Children's Hospital*, *Seattle*, *WA*; ^*2*^ *University of Washington System*, *Seattle*, *WA*; ^*3*^ *University of Washington School of Medicine*, *Seattle*, *WA*; ^*4*^ *Seattle Children's Hospital*, *Seattle*, *WA*

**Introduction:** Celiac disease is an autoimmune enteropathy triggered by gluten ingestion. The only treatment is a strict and life‐long gluten‐free (GF) diet. GF food items are typically more expensive and more difficult to access. These obstacles create barriers to patient adherence and economic burden on patients and families. The aim of this study was to analyze the cost difference between GF and gluten‐containing (GC) foods across three grocery store chains throughout the country.

**Methods:** We created a food basket of 19 standard grocery items. GF name brand (NB) and store brand (SB) items were identified, along with equivalent GC NB and SB items. The prices were collected at three different national grocery store chains (Chain A, Chain B, Chain C) in the same or neighboring zip codes across 14 regions of the United States. Locations were determined to consider most regions in the United States, though limited to areas with all three stores in proximity. Locations included Seattle WA; Dallas TX; Phoenix AZ; Los Angeles CA; Indianapolis IN; Anchorage AK; Denver CO; Atlanta GA; Nashville TN; Baltimore MD; Charlottesville VA; Little Rock AR. Two regions were included for Seattle WA and Dallas TX to assess differences in cost within the same city. The cost of each item was determined by search at the corresponding store website with the appropriate zip code; only items available for pick‐up were considered. The cost was measured in price per ounce, and percent differences in cost were calculated for each item at every location.

**Results:** When comparing all GF NB and GC NB foods, the highest percent mean difference (± SD) was found in Anchorage at Chain A with 69 ± 91%. The lowest percent difference of GF NB and GC NB foods was found in Nashville at Chain A with 17 ± 14%. The widest range of percent difference between grocery chains for GF NB and GC NB foods was found in Denver, ranging 24 ± 34% at a Chain C to 64 ± 93% at Chain A. Table 1 includes the percent difference across all locations, with corresponding graphic Figure 1. When assessing food category items, GF NB and GC NB were reviewed. The highest percent difference was seen in the instant noodle food item at Chain A with 1089 ± 556%. This difference was accentuated in the comparison of GF NB and GC SB food items, with instant noodles at Chain A having the highest percent difference as well, 3347 ± 1299%. GF NB and GF SB items were compared based on location. Chain C in Indianapolis had the highest percent difference at 64 ± 153%, compared to Chain A and Chain B in the same location at 14 ± 12% and 10 ± 8% respectively. Seattle Chain A had the lowest percent difference in GF NB and GF SB items at 7 ± 10%. GF NB and GC SB items had highest percent difference at Chain A in Anchorage 85 ± 97% and lowest percent difference in Nashville Chain A at 23 ± 16%.

**Conclusion:** The percentage increase in cost of GF foods over GC foods is highly variable for different food categories, grocery store chains, and different geographical regions. The highest cost difference between food items across locations was between GF NB and GC SB items. NB items are typically higher cost compared to SB items, though may not always be selected by patients with celiac disease due to brand familiarity, leading to increased cost. The future direction of this study will be to collect and analyze data at each location once a month over the course of a year.


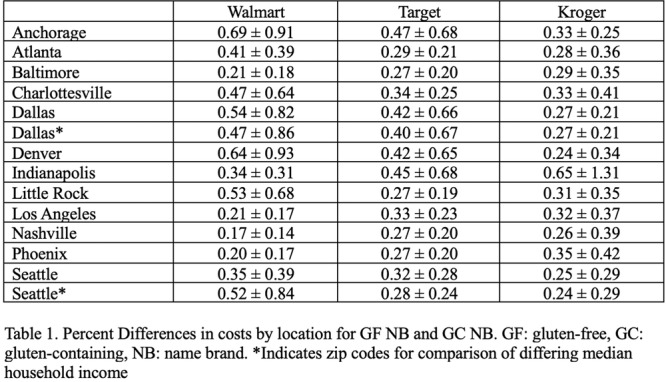



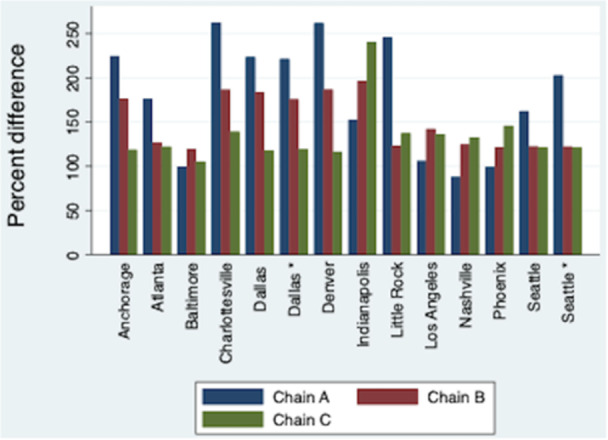


Figure 1. Comparison of percent difference in GF‐NB vs GC‐NB, by location across three stores. *Indicates zip codes for comparison of differing median household income

## 485 EVALUATION OF THE EFFECTIVENESS OF IN‐LINE IMMOBILIZED LIPASE CARTRIDGE IN ENTERALLY FED PATIENTS WITH SHORT BOWEL SYNDROME: A RETROSPECTIVE CASE SERIES

*Elizaveta Khenner* ^*3*^, *Jennifer Morton* ^*1,2*^, *Laura Green* ^*1,2*^, *Ashlee Yoder* ^*1*^, *Ann Remmers* ^*4*^, *Kayla Paul* ^*5*^, *William San Pablo* ^*1*^

^*1*^ *Gastroenterology*, *Children's Mercy Kansas City*, *Kansas City*, *MO*; ^*2*^ *Nutrition Services*, *Children's Mercy Kansas City*, *Kansas City*, *MO*; ^*3*^ *Pediatrics*, *Children's Mercy Kansas City*, *Kansas City*, *MO*; ^*4*^ *Clinical Research*, *Alcresta Therapeutics Inc*, *Waltham*, *MA*; ^*5*^ *Medical Affairs*, *Alcresta Therapeutics Inc*, *Waltham*, *MA*

Patients with short bowel syndrome (SBS) experience nutrient malabsorption. The clinical manifestations of malabsorption are heterogeneous due to the variety of congenital and acquired anatomic abnormalities that result in SBS. Because of various gastrointestinal pathophysiologic alterations, fat malabsorption is an important nutritional factor to consider. The Intestinal Rehabilitation Program at Children's Mercy has been providing a comprehensive approach to caring for children with SBS, and has incorporated RELiZORB (Alcresta Therapeutics, Inc.) immobilized lipase cartridge (ILC) into their treatment armamentarium. ILC is a single‐use digestive enzyme cartridge that connects in‐line with enteral feeding circuits to hydrolyze triglycerides in enteral formulas. Although ILC use has been associated with improved fat absorption and enteral feeding tolerance in patients with exocrine pancreatic insufficiency (EPI) and cystic fibrosis, there are no published reports of ILC use in patients with SBS.

**Objectives and Methods:** The primary objective of this retrospective single‐center case series was to evaluate the effectiveness of ILC use in children with SBS. Patients whose care was managed by the Intestinal Rehabilitation team at Children's Mercy Kansas City from 2015 to August 2024 were included in the study if they were diagnosed with SBS, between the ages of 2 and 21 years at the initiation of ILC use and had used ILC for a minimum of 3 months. Two patients were excluded from the analysis: one patient was prescribed ILC for EPI associated with acute pancreatitis, and one patient had poor compliance (<60%) with ILC use.

**Results:** Growth, enteral nutrition (EN) by tube feeding, and parenteral nutrition (PN) were analyzed for fourteen children; ten were PN and EN dependent and 4 were EN dependent. Etiologies of SBS included necrotizing enterocolitis (n=3), volvulus (n=3), gastroschisis (n=2), atresia (n=2), gastroschisis and atresia (n=2), and trauma (n=2). The majority of patients had Type III SBS. Mean age at ILC start was 6 years (range 2 to 15 years). Mean ILC use was 2 (range 1 to 3) per day, with up to 500 mL EN per device. The estimated mean residual small bowel length in 13 patients was 66 ± 54 cm (range 11 to 190 cm). Fifty percent of the patients retained their ileocecal valve and 50 % had colonic resection. Of the 8 patients with baseline fecal elastase testing, 75% had normal values (>200 µg/g). None of the patients were receiving teduglutide during the analysis period.

Within the first 3 months of ILC use:

↓ in PN use (kcal/kg/day) in 7 of 10 patients

↑ in EN use (kcal/kg/day) in 5 of 10 patients

↑ weight z‐score in 6 of 10 patients

These results suggest an increased tolerability to tube feeding with ILC use resulting in an initial trend towards decreasing PN dependence.

In the four patients dependent on EN (no PN), within the first 2 months of ILC use:

↑ weight z‐score in 3 of 4 patients with a mean improvement in z‐score of 0.40

Minimal mean change (<5%) in EN use (kcal/kg/day)

These results suggest increased nutrient absorption with ILC use.

**Conclusions:** In this single‐center case series, ILC use in pediatric patients with SBS receiving enteral nutrition demonstrated promising outcomes.

In patients requiring PN, improved EN tolerability and a reduction in PN dependence were observed in the first 3 months of ILC use.

In patients with enteral autonomy and SBS, an improvement in weight z‐score was observed within 2 months of ILC use.

These findings suggest that fat malabsorption plays a role in EN tolerance in pediatric patients with SBS.


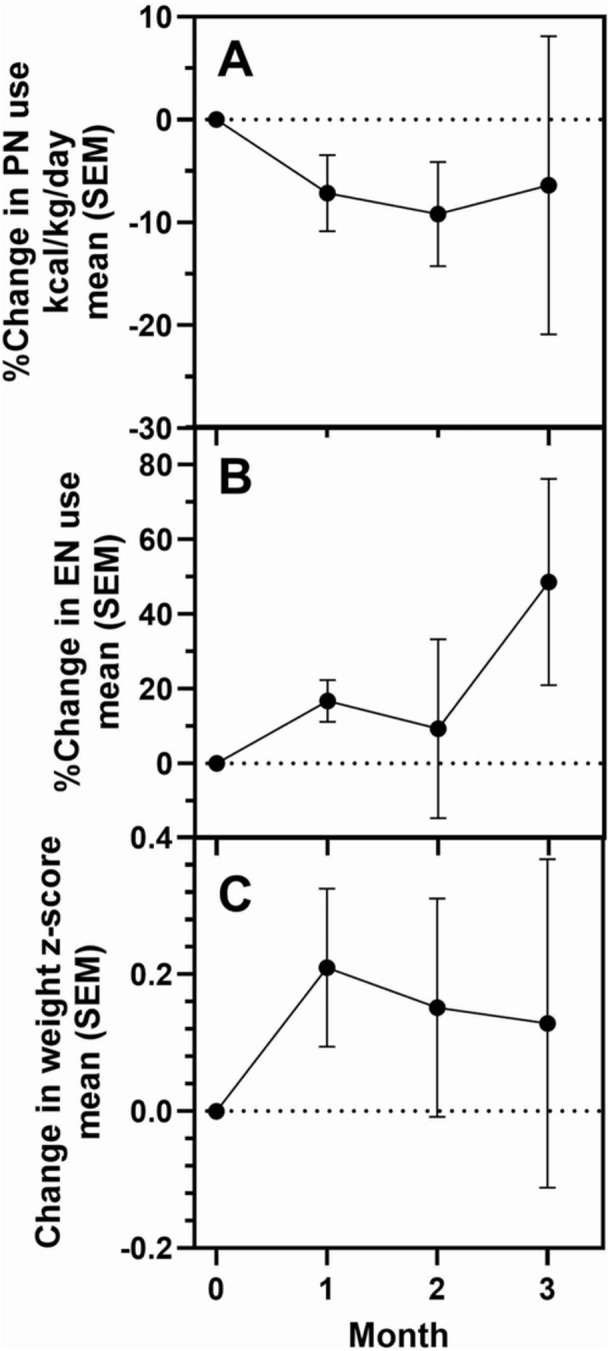


Figure 1: In the PN‐dependent population, A) Percent mean (SEM) change in PN use, B) Percent mean (SEM) change in EN use, and C) Change in mean (SEM) weight z‐score over the first 3 months of ILC use.

## 490 SURVIVAL ACHIEVED IN INFANTS WITH RAPIDLY PROGRESSIVE LAL‐D VIA SEBELIPASE ALFA ERT: RESULTS FROM THE INTERNATIONAL LAL‐D REGISTRY

*Suresh Vijay* ^*1*^, *Jennifer Evans* ^*2*^, *Simon Jones* ^*3*^, *Florence Lacaille* ^*4*^, *Florian Abel* ^*2*^, *Javier de las Heras* ^*5,6*^

^*1*^ *University Hospitals Birmingham NHS Foundation Trust*, *Birmingham*, *England*, *United Kingdom*; ^*2*^ *Alexion, AstraZeneca Rare Disease*, *Boston*, *MA*; ^*3*^ *Genomic Medicine*, *St Mary's Hospital*, *Manchester*, *United Kingdom*; ^*4*^ *Hôpital Universitaire Necker‐Enfants Maladies*, *Paris*, *France*; ^*5*^ *Biocruces Bizkaia Health Research Institute*, *Division of Pediatric Metabolism (CIBER‐ER), Cruces University Hospital*, *Barakaldo*, *Spain*; ^*6*^ *Department of Pediatrics*, *University of the Basque Country (UPV/EHU)*, *Leioa*, *Spain*

In infants, lysosomal acid lipase deficiency (LAL‐D; historically Wolman disease) is characterized by hepatosplenomegaly, growth failure, malabsorption, and liver failure, typically leading to death by 6 months of age. Previous clinical trials have shown that enzyme replacement therapy (ERT) with sebelipase alfa is well tolerated and can prolong survival and improve growth, hematologic parameters, and liver parameters. We analyzed data from the International LAL‐D Registry to report long‐term outcomes in 29 patients who were symptomatic in infancy with rapidly progressive LAL‐D. Of the 29 patients treated with sebelipase alfa, 12 were male; 8 were followed from clinical trials. Median (Q1, Q3) age was 2.3 (1.8, 3.1) months at treatment initiation. Patients received a starting dose of ≤1 or ≥3 mg/kg/wk, with observed trends showing higher starting doses in more recent years. Overall, 27 (93%) patients survived over a median (min, max; Q1, Q3) observation time of 6.2 years (0.0, 11.7; 3.5, 8.4); total patient‐years of exposure was 184.1. Median (min, max) age at last follow‐up was 6.5 (0.3, 12.0) years. Growth data are best viewed on an individual basis and will be presented as such. Adverse events (AEs) occurred in 23 (79%) patients, with 11 (38%) experiencing AEs that were potentially related to sebelipase alfa; these were generally mild to moderate in severity and resolved in most patients. Two deaths were reported: one patient died due to liver cirrhosis/failure at 3.8 years of age after 3.5 years of treatment, the second patient died prior to registry enrollment at 3.5 months of age after 0.5 months of treatment. Four of 7 patients tested positive for neutralizing antidrug antibodies. The observational International LAL‐D Registry confirmed the benefit of sebelipase alfa ERT; patients with rapidly progressive LAL‐D who were symptomatic in infancy demonstrated long‐term survival with an acceptable safety profile.

## 491 THE ASSOCIATION BETWEEN HLA GENOTYPE AND SYMPTOM PRESENTATION IN PATIENTS WITH CELIAC DISEASE: A CROSS‐SECTIONAL ANALYSIS

*Denise Dias* ^*3*^, *Caitlin Mungall* ^*3*^, *Clay Allan* ^*2*^, *Michael Miller* ^*1*^, *Tejas Desai* ^*1,2*^, *Andréanne Zizzo* ^*1,2*^

^*1*^ *Paediatrics*, *Western University*, *London*, *ON*, *Canada*; ^*2*^ *Paediatrics*, *Children's Hospital, LHSC*, *London*, *ON*, *Canada*; ^*3*^ *PROGrS Program*, *Western University*, *London*, *ON*, *Canada*

**Background:** Celiac disease (CeD) is an immune‐mediated enteropathy triggered by gluten in genetically predisposed individuals, most commonly those carrying HLA‐DQ2 and/or HLA‐DQ8 haplotypes. While these genetic markers are necessary for disease development, their presence alone does not predict symptom manifestation. A proportion of individuals with biopsy‐confirmed CeD remain asymptomatic ("silent carriers"), raising questions about genetic contributions to clinical presentation. Currently, periodic rescreening of at‐risk family members every 3–5 years is recommended. A more targeted approach based on genetic risk for symptomatic disease may improve diagnostic strategies and minimize unnecessary repeat screening. We aimed to determine whether specific HLA haplotypes are associated with symptomatic vs. asymptomatic presentations in individuals with confirmed CeD.

**Methods:** This cross‐sectional study included 174 patients with biopsy‐confirmed CeD who underwent HLA haplotyping. Patients were categorized as symptomatic (having at least one gastrointestinal [GI] or non‐GI symptom at diagnosis) or asymptomatic. The distribution of HLA haplotypes was compared between groups. Logistic regression was used to assess the association between HLA haplotype group (DQ2 only, DQ8 only, or both DQ2 and DQ8) and symptomatic presentation. Due to the small number of asymptomatic cases (n = 13), a univariable model was used to avoid overfitting. DQ2‐only patients (n = 127) served as the reference group; there were 24 patients with DQ8 only and 23 with both haplotypes.

**Results:** Of the 174 patients, 92.5% were symptomatic at diagnosis, including GI symptoms only (17.8%), non‐GI symptoms only (9.8%), or both (64.9%), while 7.5% were asymptomatic. HLA‐DQ2.5 was the most common haplotype, present in 53.4% of patients; 86% had at least one HLA‐DQ2 gene and 27% had at least one HLA‐DQ8 gene. In the logistic regression model, both DQ8‐only and DQ2+DQ8 groups had lower odds of symptomatic presentation compared to the DQ2‐only group. The odds ratio for the DQ8‐only group was 0.41 (95% CI: 0.10–1.71; p = 0.22) and for the DQ2+DQ8 group was 0.39 (95% CI: 0.09–1.63; p = 0.20). These associations were not statistically significant.

**Conclusions:** Although not statistically significant, these findings may suggest a potential trend toward asymptomatic presentation in patients with the HLA‐DQ8 haplotype. A larger cohort is needed to support this association. If confirmed, HLA typing may help stratify risk not only for CeD development but also for symptom expression, supporting more personalized rescreening intervals for at‐risk individuals. Until then, even if the absence of a positive screen, at‐risk family member carrying either one of the genes associated with a predisposition for developing CeD should continue to have ongoing screening every 3‐5 years, regardless of symptoms.


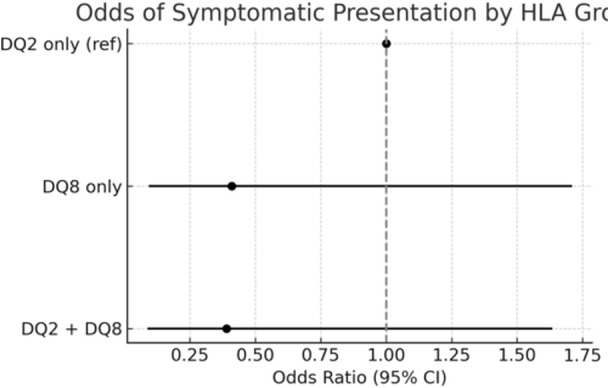


## 492 EXAMINING THE JOURNEY FOR UNDERREPRESENTED STUDENTS IN MEDICINE PROGRAM (JUMP‐START) INITIATIVE IN SUPPORTING UNDERREPRESENTED STUDENTS IN MEDICAL SCHOOL APPLICATIONS AND INCREASING MATRICULATION: A LONGITUDINAL STUDY UPDATE

*Jiwon Choi* ^*1*^, *Rhea Jacob* ^*1*^, *Vikita Patel* ^*1*^, *Nu Bui* ^*1*^, *Lauren Winkle* ^*1*^, *Rahul Shah* ^*1*^, *Qwynton Johnson* ^*1*^, *Deborah Dele‐Oni* ^*1*^, *Samantha Cooley* ^*1*^, *Reetom Bera* ^*1*^, *Ginger Chant* ^*1*^, *Alexis Kirk* ^*1*^, *Mira Basuino* ^*1*^, *Larry Segars* ^*2*^

^*1*^ *Medical Student*, *Kansas City University College of Osteopathic Medicine*, *Kansas City*, *MO*; ^*2*^ *Kansas City University College of Osteopathic Medicine*, *Kansas City*, *MO*

**Introduction:** Underrepresentation in medicine persists among both aspiring students and the physician workforce, perpetuating disparities in healthcare. The JUMP‐Start program at KCU addresses this gap by providing marginalized pre‐medical students with targeted workshops and mentorship to enhance diversity in medical education. This study evaluates JUMP‐Start's efficacy in preparing underrepresented students in medicine (URiM) for the medical school application process and examines early admission outcomes. We hypothesize that participants will demonstrate improved confidence, preparedness, and understanding of admissions requirements.

**Materials and Methods:** This longitudinal study employed two surveys: a post‐program assessment and a follow‐up survey. Fifty undergraduate participants were recruited through outreach to college counselors and pre‐medical interest groups. Surveys collected demographic data, measured perceived skill development, assessed confidence in application components (e.g., personal statements, interviews), and identified barriers to medical school admission.

**Results:** Among 31 post‐program respondents (across three cohorts), 65% identified as underrepresented racial/ethnic minorities, and 42% identified as LGBTQIA+. High satisfaction rates were reported for key metrics: 71% felt "very satisfied" with their understanding of the admissions process, and 87% expressed confidence in personal statement writing. All participants (100%) agreed JUMP‐Start reduced barriers to medical education. Of 16 follow‐up respondents, 81% endorsed satisfaction with program resources, and 56% reported applying skills during their application cycle. Among three 2024–2025 applicants, two received interview invitations, and one secured acceptances.

**Discussion:** JUMP‐Start significantly improved participants’ confidence and competence in navigating medical school applications, particularly in personal statement writing and admissions strategy. The universal perception of reduced barriers underscores the program's value for URiM students. While follow‐up data were limited, early outcomes suggest promise. Future directions include expanded longitudinal tracking and nuanced subgroup analysis (e.g., underrepresented Asian populations, who face unique disparities).

**Conclusion:** JUMP‐Start effectively equips URiM students with the tools to succeed in the medical school application process, fostering equity in medical education. These results advocate for its scalability as a model for diversifying the physician workforce.

## 493  A QUALITY APPRAISAL OF THE NORTH AMERICAN SOCIETY OF PEDIATRIC GASTROENTEROLOGY, HEPATOLOGY AND NUTRITION CLINICAL PRACTICE GUIDELINES

*Chinenye Dike* ^*1*^, *Lauren Klein* ^*2,3*^, *Kacie Denton* ^*2*^, *Irene Gamra* ^*4*^, *Aamer Imdad* ^*5*^

^*1*^ *Pediatrics, Division of Gastroenterology, Hepatology and Nutrition*, *University of Alabama at Birmingham Health System*, *Birmingham*, *AL*; ^*2*^ *Monroe Carell Jr. Children's Hospital at Vanderbilt, Department of Pediatrics, D. Brent Polk Division of Pediatric Gastroenterology, Hepatology, and Nutrition*, *Nashville*, *TN*; ^*3*^ *Vanderbilt University Medical Center, Vanderbilt Institute for Global Health*, *Nashville*, *TN*; ^*4*^ *Pediatrics, Division of Gastroenterology, Hepatology and Nutrition*, *University of Florida*, *Gainesville*, *FL*; ^*5*^ *Department of Pediatrics, Division of Pediatric Gastroenterology, Pancreatology, Hepatology and Nutrition*, *Stead Family Department of Pediatrics, Carver College of Medicine, University of Iowa*, *Iowa City*, *IA*

**Background:** The North American Society For Pediatric Gastroenterology, Hepatology and Nutrition (NASPGHAN) is the professional association for pediatric gastroenterologists in North America. NASPGHAN solicits guidelines from its committees to guide the practice of evidence‐based care. These guidelines are required to be systematically constructed with high quality and clinical impact.

**Aim:** To appraise the quality of published NASPGHAN guidelines using the Appraisal of Guidelines for Research & Evaluation II (AGREE II) instrument.

**Methods:** We evaluated NASPGHAN guidelines published from 2005‐2024. A training‐focused guideline was excluded because its purpose and content did not align with the intended scope of the AGREE II instrument. Each included guideline was assessed by at least 4 reviewers using the AGREE II instrument, which measures methodological quality and transparency across six domains (*D1‐D6*).

*Scope and purpose (D1)* examines the overall objectives of the guideline, the specific health questions addressed, and the target population. *Stakeholder involvement (D2)* evaluates whether relevant professional groups were included, whether the views and preferences of the target population were considered, and whether the intended users were clearly defined. *Rigor of development (D3)* assesses the process for evidence selection, description of evidence strengths and limitations, methods for formulating recommendations, consideration of benefits and risks, links between recommendations and evidence, external review procedures, and plans for guideline updates. *Clarity of presentation (D4)* focuses on the specificity, clarity, and identifiability of recommendations. *Applicability (D5)* considers potential barriers and facilitators to implementation, the availability of practical tools, resource implications, and monitoring or audit criteria. Finally, E*ditorial Independence (D6)* addresses the influence of funding sources and whether conflicts of interest were disclosed and managed appropriately. Each domain consists of 2 to 8 items, which are scored on a 7‐point Likert scale (1 = strongly disagree, 7 = strongly agree) based on how well the guideline meets specific criteria. Domain scores were standardized as percentages of the maximum possible score, with higher percentages indicating better quality.

**Results:** Twelve guidelines were published between 2005‐2024, and 11 were appraised; 8 of those were joint NASPGHAN/ESPGHAN guidelines. Guideline authors were primarily based in North America (83) and Europe (62), with smaller representation from South America (3), Middle East (3), and Asia (1). Guidelines evaluated covered a broad range of pediatric gastroenterology topics, including quality improvement in endoscopy, eosinophilic esophagitis and eosinophilic gastrointestinal disorders, gastroesophageal reflux, *Helicobacter pylori* (2016 and 2023), constipation, celiac disease, nonalcoholic fatty liver disease, neonatal jaundice, esophageal atresia and tracheoesophageal fistula, and hepatitis C. AGREE II scores varied across domains, as shown in violin plot (**Figure 1**), which illustrates the distribution of the scaled domain scores for the 11 guidelines. The highest scored domains were Scope and Purpose (mean: 83.8%, SD: 7.6) and *Clarity of presentation* (mean: 80.4%, SD: 9.9). The lowest scored domains were *Applicability* (mean 53.6%, SD: 5.1) and *Stakeholder involvement*. (mean: 58.1%, SD 7.8). The *Rigor of development* domain averaged 67.9% (SD: 7.7) with notable variability in scores across reviewers. Among all guidelines, only the 2023 *Helicobacter pylori* guideline achieved a score of ≥60% across all six AGREE II domains.

**Conclusions:** Appraisal of the NASPGHAN guidelines using the AGREE II instrument showed strengths in several domains, particularly in *Scope and purpose*, and *Clarity of presentation*. However, multiple domains, particularly in *Stakeholder involvement, Rigor of development* and *Applicability*, could be improved. Future guideline development should align more closely with AGREE II standards to enhance methodological rigor, transparency, and clinical utility.


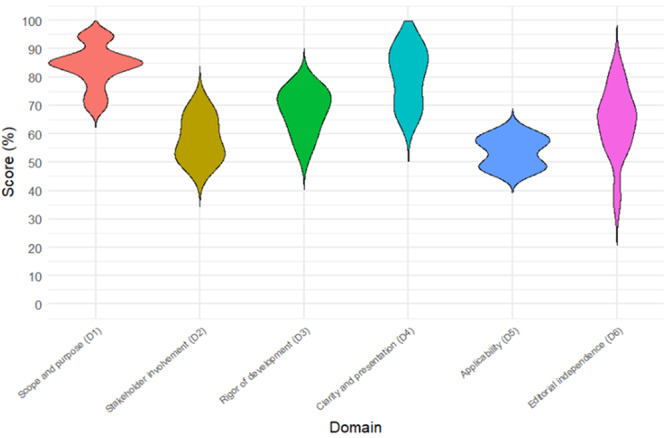


Violin Plot of AGREEII Scaled Domain Scores

## 494 ENHANCING PEDIATRIC GASTROENTEROLOGY FELLOWSHIP: THE POWER OF REAL‐WORLD MODELED ROTATIONS

*Nidhi Goyal*, *Elizabeth Yu*, *Mamata Sivagnanam*, *Jeannie Huang*

*Pediatric Gastroenterology*, *University of California San Diego*, *La Jolla*, *CA*

**BACKGROUND:** The relevance of real‐world applications is key to the effectiveness of medical education for adult learners, as adults learn best when they can directly apply their knowledge to real‐life situations. Training modules based on real‐world scenarios also offer foster skill development and deepen understanding. While pediatric gastroenterology fellowship training objectives have been identified, models by which such training occurs vary across programs.

**OBJECTIVE:** We aimed to assess the effectiveness of real‐world modeled rotations in a pediatric gastroenterology fellowship training program.

**METHODS:** The Pediatric Gastroenterology Fellowship at UC San Diego/Rady Children's was established in 1997. Restructuring of inpatient (to a model where fellows function as the attending) and the fellow continuity clinic (to a model where patients are scheduled under the fellow instead of under the attending) rotations was performed in 2010 and 2014 respectively, modeled after real‐world scenarios to promote adult learning. Fellowship graduates and faculty were surveyed about the fellowship training experience and specifically about these real‐world scenario rotations. Raters rated whether rotations promoted ownership in patient‐provider relationships and to what extent they prepared trainees for their future careers using a scale of 0 to 10; 10 being the highest. Answers are reported as median (interquartile range) and were analyzed using non‐parametric testing. A P‐value of 0.05 was used as the significance threshold.

**RESULTS:** Twenty out of 31 fellow graduates and 11 of 15 faculty completed the evaluation surveys. Of the surveyed fellow graduates, seven participated in continuity clinic with patients scheduled under an attending, while thirteen had patients scheduled under their own name. For the inpatient rotation, sixteen fellows served as attending while four did not. For both real‐world rotations, fellows rated the real‐world model higher than the standard model with regard to preparing them for their future careers (Table and Figure). Ownership in the patient‐physician relationship was also more highly rated by fellows in the real‐world v. standard model, although significance was reached only in the continuity clinic scenario.

Surveyed faculty reported that fellows demonstrated more ownership of patient relationships when training in real‐world rotations, particularly in continuity clinic. Overall, faculty felt very comfortable allowing fellows more autonomy to encourage preparedness for real‐world practice in all scenarios.

**CONCLUSION:** Real‐world modeled fellowship training rotations were highly rated by both trainees and faculty. These models appear to promote greater trainee autonomy and more effectively prepare trainees for future practice than standard training rotations. Real‐world modeling should be incorporated into clinical training models in pediatric gastroenterology fellowship programs.

## 495 USING LARGE LANGUAGE MODELS TO SIMULATE OVERNIGHT PHONE CALLS TO PEDIATRIC GASTROENTEROLOGY FELLOWS

*Christopher Moran* ^*1*^, *Kayla Hartjes* ^*1*^, *Katherine Baldwin* ^*2*^, *Michael Herzlinger* ^*3*^, *Adam Rodman* ^*4*^, *Christina Gao* ^*5*^, *Stephen Bacchi* ^*6*^

^*1*^ *Pediatrics*, *Massachusetts General Hospital*, *Dedham*, *MA*; ^*2*^ *Pediatric Gastroenterology*, *Connecticut Children's Medical Center*, *Hartford*, *CT*; ^*3*^ *Pediatric Gastroenterology*, *Brown University Warren Alpert Medical School*, *Providence*, *RI*; ^*4*^ *Beth Israel Deaconess Medical Center*, *Boston*, *MA*; ^*5*^ *University of Adelaide Graduate Research School*, *Adelaide*, *South Australia*, *Australia*; ^*6*^ *Neurology*, *Massachusetts General Hospital*, *Boston*, *MA*

**Background:** Pediatric gastroenterology (GI) fellows often receive urgent overnight phone calls from parents and physicians yet training for phone triage is often lacking or inconsistent. Standardized simulation exercises are effective in preparing learners for successfully managing novel clinical encounters. However, fellowship programs may have limited resources to provide such instruction. Automation through artificial intelligence (AI)‐based chatbot technology may be a useful, cost‐effective strategy for ensuring new pediatric GI fellows are equipped. This study evaluated the utility of a large language model (LLM)‐based virtual patient platform in training for these encounters.

**Methods:** Pediatric GI faculty and fellows at the primary center were surveyed to identify high‐yield clinical scenarios to create prompts. The study team wrote text prompts for the AI‐driven virtual patient platform on the TEACHABLE website (www.researchteaching.com). Current pediatric GI fellows (including 1st year, 2nd year, and 3rd year fellows) from 3 different institutions were invited to participate. Participants completed surveys regarding overall experience in Redcap including Likert scale‐based questions on relevance and helpfulness.

**Results:** The initial survey of 20 faculty and 6 fellows identified 9 potential topics. The study team created 7 scenarios for pilot testing and 12 current pediatric GI fellows were invited to participate. Ten fellows completed an average of 4.5 scenarios and six fellows completed follow‐up surveys. Thirty‐three percent (2/6) of pediatric GI fellow survey responders reported previous home‐call experience during their pediatric residency and both of those responders reported receiving previous training for those phone calls. The average time to complete the scenarios was 5.5 minutes per scenario. Participants rated the scenarios to be relevant (5.0/5), engaging (4.7/5), and believed they would be helpful to orient future pediatric GI fellows (4.5/5).

**Conclusion:** This pilot project provides evidence that an AI‐based virtual patient simulator simulating phone calls from parents and physicians can be useful to orient first year pediatric GI fellows to pager triage. Future studies will aim to evaluate this during orientation of new fellows.

## 496 VIDEO‐BASED LEARNING IN NUTRITION SUPPORT: EMPOWERING RESIDENTS TO MANAGE PN WITH CONFIDENCE

*Tierra Mosher* ^*1*^, *Stephanie Tran* ^*2*^, *Angela Nguyen* ^*2*^, *Shweta Namjoshi* ^*1*^, *Yasemin Cagil* ^*1*^

^*1*^ *Pediatric Gastroenterology*, *Stanford University*, *Stanford*, *CA*; ^*2*^ *Pediatrics*, *Stanford University*, *Stanford*, *CA*

**Background:** Parenteral nutrition (PN) is a complex yet critical aspect of inpatient pediatric gastroenterology. Many pediatric residents receive limited formal education on PN during training and often report a lack of confidence in optimizing PN for acute care patients on the pediatric gastroenterology inpatient service. To address this knowledge gap, we developed a series of short, high‐yield educational video modules aimed at improving resident confidence and knowledge in PN management.

**Methods:** We created a seven‐part video module curriculum (each video lasting 2‐5 minutes) highlighting key concepts such as PN macronutrients, micronutrients, ordering principles, patient monitoring, and safety concerns. The videos are being integrated into the pediatric gastroenterology inpatient rotation for residents at our institution over a three‐month pilot period. Residents will complete pre‐ and post‐intervention surveys, including: 1) a knowledge quiz on PN principles; 2) Likert‐scale questions assessing confidence and perceived usefulness of the videos; 3) open‐ended feedback on video content and format. Primary outcomes will include change in quiz scores and self‐reported confidence. Secondary outcomes will include qualitative themes identified from open‐ended responses. Quantitative data will be analyzed using Wilcoxon signed‐rank tests due to the small pilot group sample size (anticipated n~10). Effect sizes will be calculated. Qualitative responses will undergo thematic analysis.

**Results:** Data collection is ongoing, with video curriculum in its final stages. Final data on knowledge improvement and resident perceptions will be available at the time of NASPGHAN presentation.

**Conclusion:** This pilot study introduces a brief, accessible video‐based curriculum for teaching PN to pediatric residents. If shown to be effective, it may serve as a scalable tool to enhance nutrition education during inpatient training and will inform the next phase of implementation across future academic years. We therefore hope to use our preliminary data as proof of concept for a more extensive, nine‐month‐long implementation for which we will apply for funding.

## 497 IMPROVING HEALTH CARE ACCESS FOR PATIENTS WITH AUTISM IN PEDIATRIC ENDOSCOPY: A PROCESS IMPROVEMENT INITIATIVE

*Mojdeh Mostafavi*, *Kriston Ganguli*

*Pediatric Gastroenterology & Hepatology*, *Massachusetts General Hospital*, *Boston*, *MA*

**BACKGROUND:** Autism spectrum disorder (ASD) is a multifactorial neurodevelopmental disorder affecting 1 in 31 children in the United States. Defined by significant impairments in social interaction and communication, as well as restricted and repetitive behaviors, ASD is often associated with additional challenges. Beyond these core traits, individuals with ASD frequently experience significant sensory sensitivities, non‐core behavioral disturbances (e.g. self‐injury and aggression), comorbid psychiatric (e.g anxiety, depression, OCD) as well as comorbid medical conditions. Up to 70% of children with ASD experience gastrointestinal (GI) symptoms, a prevalence significantly higher than that of children without ASD or those with other developmental disabilities. In recent years, there has been growing recognition of the barriers individuals with ASD face in accessing healthcare, which contribute to increased morbidity and mortality.

**AIMS:** Traditional approaches to pre‐procedure preparation and procedural care may be ineffective for individuals with ASD due to common communication barriers, sensory sensitivities, and other unique needs. This project aims to improve health equity and access by first characterizing current practices with our pediatric endoscopy unit through process mapping in collaboration with providers, family/caregivers, and multidisciplinary team members (including anesthesia, nursing, and child life). Specific aims include: (1) creating developmentally appropriate materials for endoscopy preparation; (2) providing ASD‐focused education for clinicians and caregivers; (3) implementing a system to identify and screen patients with ASD undergoing endoscopy for accommodation needs; and (4) enhancing care coordination through development of a virtual pre‐procedure huddle.

**METHODS:** This project used the Plan‐Do‐Study‐Act (PDSA) model to iteratively improve care by first mapping current workflows, followed by targeted implementation of interventions. Family/caregiver feedback, along with individual patient feedback, when possible, was critical throughout and was gathered using mixed methods strategies including semi‐structured interviews.

**PRELIMINARY RESULTS:** In the last six months, more than 100 individuals with ASD have undergone endoscopy using the interventions developed in this project, informing continued refinement of our strategies. As an illustrative example, feedback from the family of a 13‐year‐old non‐verbal male patient with severe ASD, comorbid eosinophilic esophagitis and prior negative endoscopy experiences highlighted the positive impact of this work. Through early identification, ASD‐focused pre‐screening, use of a social story and day‐of schedule, as well as coordinated care, he was able to complete the procedure without physical restraint or IM sedation. Reflecting on this, his mother shared, *“I assumed it was going to be anxiety‐ridden and we were going to have to wrestle him to the floor… I had no confidence his autism would be addressed.”* However, thanks to these interventions, she said, *“I think this is an amazing thing that you're doing. I wish you could implement it in every hospital…it's a game changer. It was amazing. I couldn't even believe that it went the way that it did.”*

**PROJECT SIGNIFICANCE & NEXT STEPS:** To the best of our knowledge, this is the first process improvement project specifically aimed at improving endoscopy care and experiences for pediatric patients with ASD. This work is intended to serve as a foundation for more accessible and equitable healthcare for individuals with neurodevelopmental disabilities but ultimately has the potential to benefit all patients by creating a framework in which individualized needs are proactively identified and addressed through structured, patient‐centered planning. The core principles of this approach are adaptable and have the potential to be expanded across other areas of the healthcare system. Next steps include ongoing data collection and analysis through semi‐structured interviews with patients, parents/caregivers, and care teams; characterization of provider premedication prescribing practices; and evaluation of outcomes such as procedure tolerance, sedation use, and the impact on subsequent procedures. While this work reflects preliminary results, the early impact on patient experience and care delivery has been profound, underscoring the importance of sharing this work and continuing to expand its reach within the field of pediatric gastroenterology and beyond.

## 498 BREAKING BARRIERS FOR CHILDREN WITH AUTISM SPECTRUM DISORDER: DEVELOPMENT OF AN ENDOSCOPY SOCIAL STORY

*Mojdeh Mostafavi*, *Kriston Ganguli*

*Pediatric Gastroenterology & Hepatology*, *Massachusetts General Hospital*, *Boston*, *MA*

**Background:** In the United States, autism spectrum disorder (ASD) affects 1 in 31 children. ASD is defined by the DSM‐V criteria including impairments in social interaction and communication, as well as the presence of restricted, repetitive behaviors. Gastrointestinal symptoms affect up to 70% of children with ASD, a prevalence notably higher than in children without ASD or those with other developmental disorders. In recent years, there has been growing recognition of the barriers patients with ASD face in accessing healthcare, which contribute to increased morbidity and mortality.

**Social Stories for ASD:** “Social Stories” were first introduced in 1993 by educator and consultant Carol Gray. Originally designed to support patients with ASD in understanding and managing social interactions, the concept has since been adapted to enhance learning and communication more broadly. When combined with task analysis, social stories can target specific behaviors and support skill development. Importantly, they also serve as valuable tools in preparing patients with ASD for new or potentially stressful experiences by presenting information in a clear, step‐by‐step format, often accompanied by visual aids.

**Endoscopy Social Story:** The endoscopy social story was created as a direct response to concerns voiced by family/caregivers about the challenges faced by patients with ASD undergoing endoscopic evaluation. To ensure the tool reflected both the clinical reality and the needs of neurodiverse patients, development involved close collaboration with caregivers, child life specialists, behavioral consultants, and endoscopy staff. Content was refined through iterative feedback to ensure clarity, accuracy, and accessibility, with particular attention to the sensory and communication needs of patients with ASD. The resulting product was a patient‐centered narrative that aligns closely with the actual endoscopy experience, designed to support preparedness and reduce anxiety for both patients and their families.

**Preliminary Results:** Early feedback emphasizes that the social story itself is a crucial tool for preparing patients with ASD by setting clear expectations. However, its effectiveness depends heavily on the clinical experience aligning with what is described in the story. As one parent reflected:

"I believe the communication aspect of it was key because I could have gone over that social story with him a million times, but if we got in there and it wasn't what was on that social story, it would have been a different outcome. So, I think the communication, the way that the medical staff on the endoscopy floor was just so on it, I just think it all came together and it was for him exactly what he was anticipating.”

**Project Significance:** To the best of our knowledge, this is the first social story specifically designed for endoscopy procedures, filling a critical gap in healthcare. This social story is part of a broader process improvement initiative aimed at better supporting patients with ASD in accessing gastroenterological care. The project will continue to integrate other behavioral strategies— such as reinforcement/token boards, pre‐procedure site visits, and enhanced ASD‐specific pre‐screening processes—into the care model to further improve the patient experience. Traditional medical preparation methods frequently fall short for these patients, making accommodations like this social story essential.

**Collaboration for Success:** The involvement of medical providers is essential to the success of this project. Behavioral specialists alone cannot address the full scope of the needs of patients undergoing medical procedures. Due to the specialized nature of GI care and the complexity of endoscopy, the expertise of GI providers ensures that the social story is both medically accurate and effective. The social story will be publicly available for other institutions to adapt, improving the experience of children with ASD undergoing endoscopy nationwide.


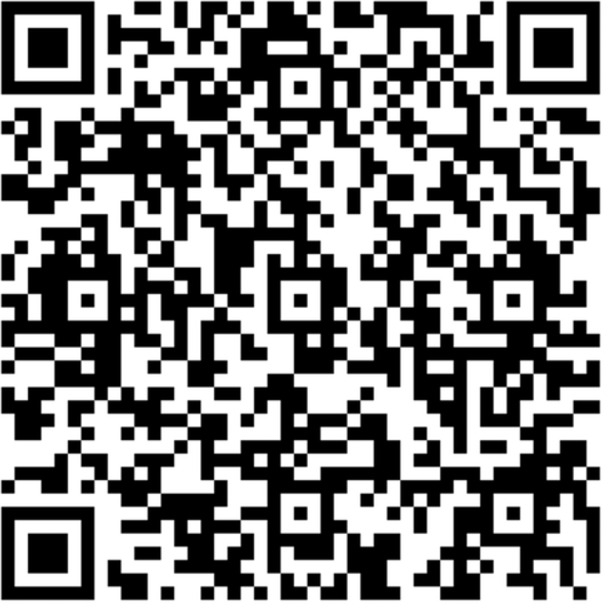


## 499 ENHANCING RESEARCH EXPOSURE WITH SUMMER MEDICAL STUDENT INTERNSHIPS‐ AN ACADEMIC PEDIATRIC GI EXPERIENCE

*Kanika Puri* ^*1*^, *Rebecca Goss* ^*1*^, *Anne Nguyen* ^*2*^, *Jean Molleston* ^*1*^

^*1*^ *Pediatric Gastroenterology, Hepatology and Nutrition*, *Indiana University School of Medicine*, *Indianapolis*, *IN*; ^*2*^ *Indiana University Indianapolis*, *Indianapolis*, *IN*

**Background:** Scholarly research training programs are crucial for developing research acumen and critical thinking in medical students (MS) and advancing medical knowledge. To foster research skills, medical schools offer for students to explore through summer research experiences or mandatory curricular components, globally. Indiana University School of Medicine (IUSOM) recruits 365 MS each year. MS are encouraged to be involved in summer research projects between year 1 and 2. Funding is obtained from various resources like Dean's offices, clinical departments, and various endowments.

The Makenna Van Laeken Endowment Fund (VLE) for Liver Research at Riley Children's Hospital in Indianapolis was established by the parents of a newborn who passed away due to liver failure. A summer research program was successfully rolled out by the Division of Pediatric Gastroenterology, Hepatology and Nutrition in 2019 and selected rising second year MS through an independent application process. In 2023, this process was merged with the Indiana University Medical Student Program for Research and Scholarship (IMPRS). Here we describe our center's experience with the program offered to IUSOM students from 2020‐2024.

**Methods:** 2 to 4 students were accepted into the research program in Pediatric GI each summer. VLE internships are a structured ten‐week summer experience aimed at fostering research skills and interest in clinician‐scientist careers. MS in good standing who are completing their first year are eligible to apply and paired with 2 faculty mentors with active research programs related to pediatric GI conditions.

The students work on 2 different research projects and develop one patient education handout. MS are encouraged to attend weekly half‐day educational conferences in the division and IMPRS seminar series. They have the chance to shadow faculty in clinics and procedures. They work in‐person on campus and may work remotely as well.

The students are required to submit a research abstract and participate in the poster symposium at the end of the IMPRS summer session with potential to submit to national meetings. The research student is also awarded co‐authorship on any published work arising from the research program. Qualitative feedback is received from faculty mentors on the MS performance and they can also give feedback on their experiences.

**Results:** Since its inception in 2020, the program has offered the summer internship opportunity to 12 medical students. Students have helped author 13 abstracts and poster submissions at regional and national meetings, three publications in peer‐reviewed journals, and six manuscripts in progress in various stages of submission as shown in Table 1.

Upon completion of the internship, students provided positive feedback mainly noting well‐rounded and valuable experience owing to the ability to work on different projects, exposure to educational endeavors (conference attendance, outpatient shadowing), research opportunities (introduction to biostatistics, REDCap tools) and mentoring from different GI faculty.

**Discussion:** Recently, there has been a downtrend in recruitment into pediatric residency and pediatric gastroenterology disciplines. Mentored research internships can have substantial benefits for medical students in the development of their research interests and persistent long‐term involvement in research activities. The early exposure can provide a catalyst for producing scholarly work and developing clinical skills through early mentoring. At the same time, the respective disciplines have an opportunity to recruit future clinicians who are driven to make long lasting clinical and academic impact. The partnership of the family endowment, the medical school, and the Pediatric GI Division had several positive impacts on the medical students and their mentors.


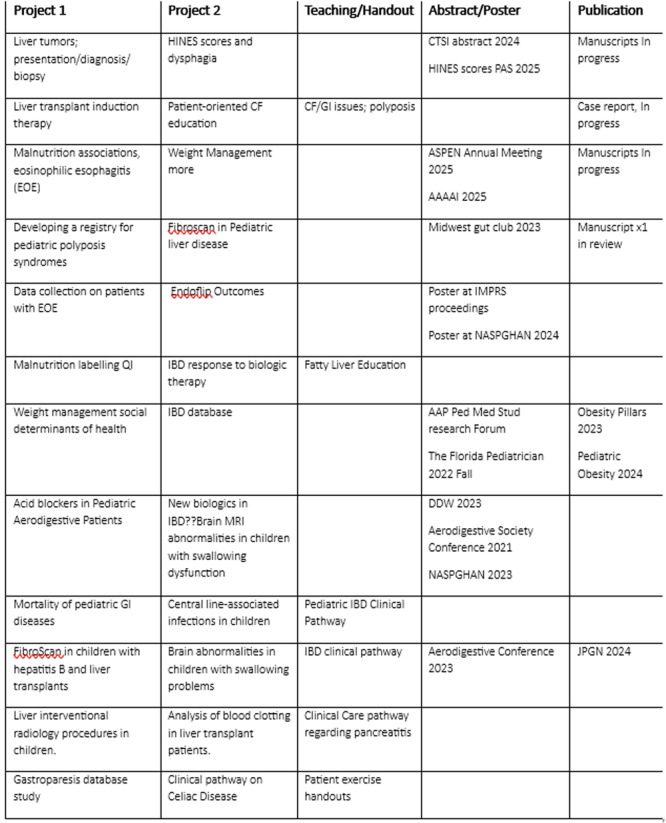


Table 1. Summary of project themes and outcomes of research

## 500 IMPLEMENTING A BEDSIDE STANDARDIZED CLINICAL SCORING SYSTEM FOR HOSPITALIZED PEDIATRIC PATIENTS WITH INFLAMMATORY BOWEL DISEASE

*Nasiha Rahim* ^*1*^, *Tessa George* ^*2*^, *Cynthia Alvarez* ^*1*^, *Andrea Berkemeyer* ^*1*^, *Amelia Kellar* ^*1*^

^*1*^ *Pediatric Gastroenterology*, *University of Chicago Division of the Biological Sciences*, *Chicago*, *IL*; ^*2*^ *Clinical Trials*, *The University of Chicago Division of the Biological Sciences*, *Chicago*, *IL*

**Background:** Inflammatory bowel disease (IBD) is a chronic autoimmune condition causing inflammation in the gastrointestinal tract.^1^ Symptoms are evaluated utilizing validated scoring systems: the Pediatric Ulcerative Colitis Activity Index (PUCAI) for Ulcerative Colitis^2^ and the Harvey‐Bradshaw Index (HBI) for Crohn's Disease^2^. It is important for pediatric residents to develop proficiency in utilizing standardized disease activity scoring and to incorporate them into clinical practice as frontline providers. These assessments enhance diagnostic accuracy, ensure consistency in care, and support evidence‐based decision‐making.

**Aim:** The primary objective of this prospective observational study was to assess the impact of an educational intervention on the utilization and documentation of PUCAI^2^ and HBI^2^ scoring tools by pediatric residents managing hospitalized IBD patients, as measured by compliance in clinical documentation. A secondary objective was to evaluate patient understanding and satisfaction with clinical care following implementation.

**Methods:** Pediatric hospital medicine (PHM) residents completed knowledge assessments before the intervention, and at 3‐ and 6‐months post‐intervention, using Likert scale responses, ranging from “Never” to “Always”. Standardized dot phrases were introduced to aid in documentation. Chart reviews evaluated compliance with PUCAI/HBI use one year before, and 3 and 6 months after the intervention. Fisher's Exact test assessed statistical significance in compliance at each time point. Patient feedback was collected through bedside symptom trackers and discharge surveys.

**Results:** Post educational intervention, there was an increase in utilization of the PUCAI/HBI^2^ in clinical documentation. The percentage of residents who “Always” ask about disease activity level increased from 15.8% pre‐intervention to 42.9% 3‐months post‐intervention, and 50.0% at the 6‐month follow‐up (Table 1). Inclusion of the PUCAI/HBI^2^ in clinical documentation significantly increased from 10.5% pre‐intervention, to 14.3% at the 3‐month follow up, and 16.7% at the 6‐month follow up. “Always” responses for both steroid use and guiding discharge planning rose from 5.3% to 7.1% and reached 16.7% at 6‐months.

A review of 224 PHM notes from the year prior to intervention indicated low utilization of the PUCAI/HBI^2^, at 9.4%. Among 236 GI notes, the scoring systems were documented in 27.1%. Three months post‐intervention, utilization increased to 16.4% in PHM notes and 56.6% in GI notes. At 6 months post‐intervention, 18.9% of hospitalist notes and 50.0% of GI notes included the HBI or PUCAI. These findings indicated a significant increase in utilization 6 months post‐intervention (PHM: *p* = 0.036, 95% CI [0.993‐4.87], OR = 2.25; GI: *p* < 0.001, 95% CI [1.55‐4.76], OR = 2.69).

Patient feedback gathered from discharge surveys thus far was consistently positive. All patients reported clear understanding of the reason for admission and treatment plans, and that providers addressed key symptoms during daily clinical assessments. 80% of patients “Strongly Agreed” the symptom tracker was helpful, and 100% “Strongly Agreed” the daily tracker improved symptom understanding and communication.

**Conclusion:** Following the education, compliance with the PUCAI/HBI^2^ significantly increased 6 months post‐intervention. Patients and caregivers reported improved understanding of treatment plans, and satisfaction with provider communication. Continued assessment and educational initiatives are planned to further enhance compliance, with the goal of improving diagnostic accuracy, promoting consistency in care, and supporting evidence‐based management of hospitalized pediatric IBD patients.

**References:**

1. McDowell C, Farooq U, Haseeb M. Inflammatory Bowel Disease. StatPearls Publishing; 2025 Jan.

2. Crohn's & Colitis Foundation. Pediatric Assessment Tools. Crohn's & Colitis Foundation; February 2023.


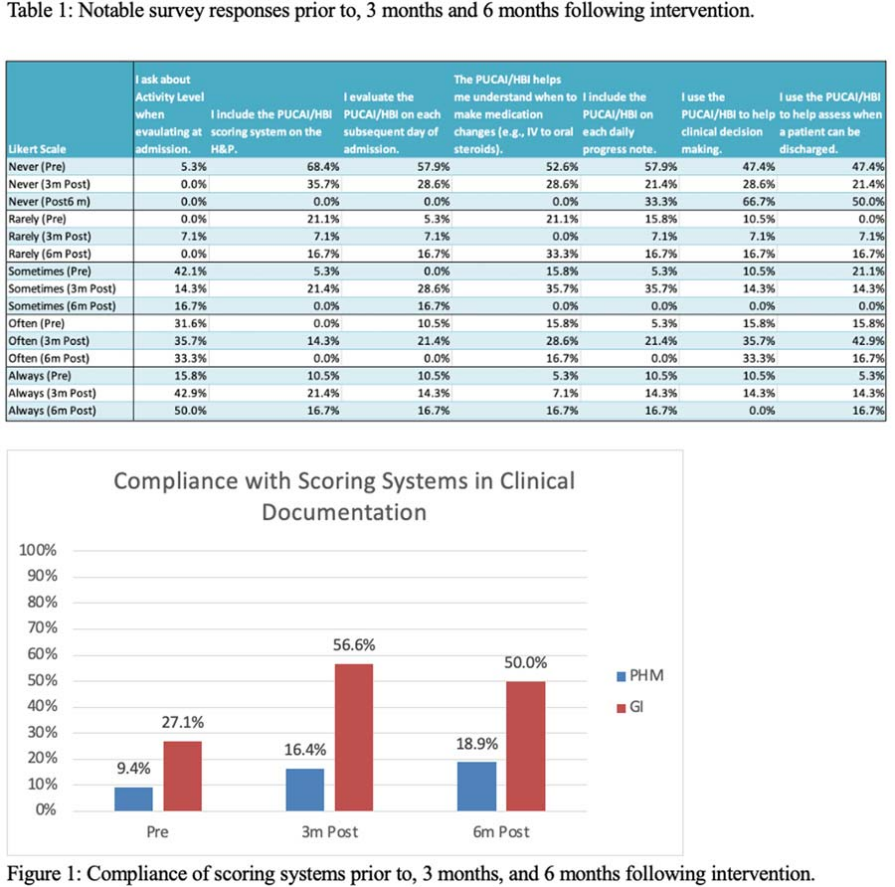


## 501 COMPARING DIAGNOSTIC PREFERENCES FOR BILIARY ATRESIA: A SURVEY OF PEDIATRIC SURGEONS AND GASTROENTEROLOGISTS

*Alexandra Stendahl*, *Henry Lin*

*Pediatric Gastroenterology*, *Oregon Health & Science University*, *Portland*, *OR*

**Background:** Biliary atresia (BA) is a critical condition to diagnose in the neonatal period, as a Kasai surgery before 60 days of life greatly increases survival with the native liver. The diagnosis of BA at our institution is a collaboration between medicine and surgery. We have historically used hepatobiliary scintigraphy (HIDA), which is highly sensitive (96%‐98%), but has lower specificity (70%‐74%). HIDA scans have the additional consideration of a recommended 5 days of premedication with phenobarbital to improve specificity, which may delay time to diagnosis. Given the limitations of a HIDA scan, the goal of this study was to review the practice of HIDA scans for neonatal cholestasis at our institution and assess pediatric gastroenterology and surgical provider clinical assessment and diagnostic testing for BA.

**Methods:** Pediatric GI providers and pediatric surgeons completed an assessment of 10 different clinical scenarios designed to assess their clinical suspicion of BA, and specifically which scenarios would prompt an intra‐operative cholangiogram versus additional diagnostic work up.

**Results:** On assessment of clinical suspicion of BA, 7 surgeons and 7 pediatric gastroenterologists completed the assessment (Table 1). Both GI providers and surgeons had high suspicion for BA with a positive PCC and liver biopsy and would proceed to BA. For many GI providers, the clinical scenario plus a liver biopsy, PCC, or non excreting HIDA scan alone was sufficient to recommend surgery. Surgeons differed primarily in their strong preference for PCC, as well as desire for a second confirmatory test (ie liver biopsy).

**Conclusion:** The difference between surgeon and GI provider preference highlights the need for collaboration. HIDA scans have lower specificity and can be time intensive due to premedication, making them poorer diagnostic tests. Several providers noted that if the clinical picture was suspicious for BA, they would request further testing or proceed to surgery even with an excreting HIDA, indicating poor clinical utility despite high sensitivity.

PCCs were the preferred test from the surgeons at our institution and have reported sensitivity of 100% and specificity of 87%. Given that the PCC is done by the interventional radiology team and can be coordinated with a liver biopsy, our institution has updated the diagnostic workflow of prioritizing PCC with a liver biopsy in the setting of a cholestatic infant with an elevated GGT and MMP7. We noted differing levels of confidence in serum biomarkers such as GGT and MMP7, and next steps include an educational session with faculty reviewing the utility of these tests.


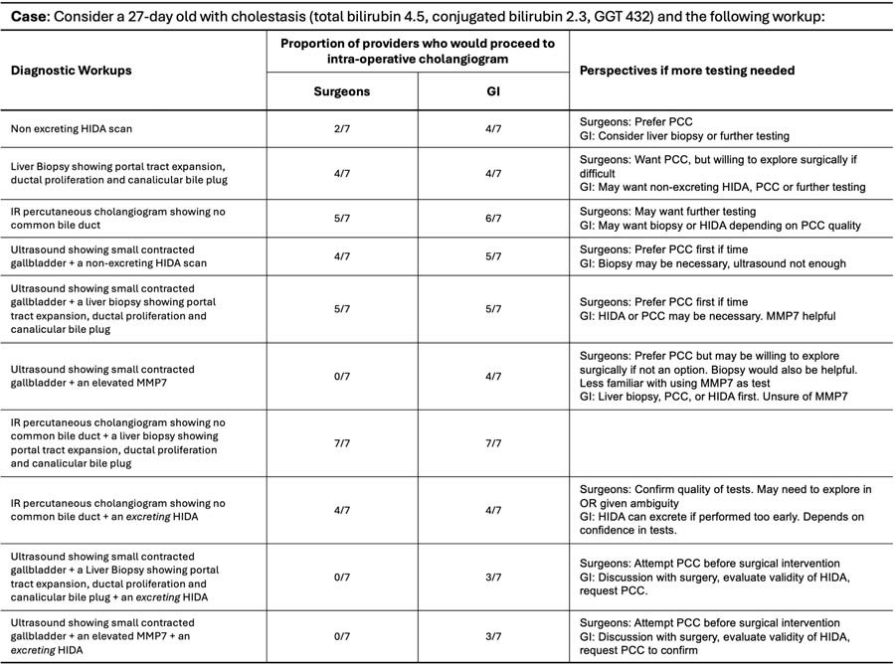


Table 1: Summary of survey sesponses to clinical scenarios.

## 502 CULINARY HEALTH EDUCATION FOR FAMILIES (CHEF): A PILOT PROGRAM FOR NUTRITIONAL EQUITY AND CULTURAL EMPOWERMENT; NASPGHAN CHILD NUTRITION AND HEALTH EQUITY WHITE HOUSE CHALLENGE AWARD PROGRESS UPDATE

*Mariaelena Terzis* ^*3*^, *Allison Grover* ^*3*^, *Kelly Gao* ^*3*^, *Anna Briley* ^*1*^, *Julia Fritz* ^*1,2*^

^*1*^ *Pediatric Gastroenterology*, *Maine Medical Center*, *Portland*, *ME*; ^*2*^ *Pediatrics*, *Tufts University*, *Medford*, *MA*; ^*3*^ *Pediatrics*, *Maine Medical Center*, *Portland*, *ME*

**Project Aims:**

‐ To provide child‐friendly cooking classes to the patients of the MaineHealth MMC Pediatric Clinic in partnership with community programs established to address nutritional insecurity

‐ To increase accessibility to local resources in a culturally sensitive manner

**Background:** Nationwide, Maine is ranked the 7th most food insecure state with 12.5% of the population and 20% of children facing hunger.The MaineHealth MMC Pediatric Clinic, which is also the MMC Pediatric Residency clinic, serves a diverse population of patients, many of whom are recent immigrants from a range of countries including Iraq, Democratic Republic of Congo, and Somalia. Only 37.3 % of the clinic population is English speaking with 39 other languages represented. Despite governmental and community resources to help these patients, they remain underserved and 20.9% of families screened positive for food insecurity in the last 12 months using the Hunger Vital Sign (a validated 2‐question food insecurity screening tool). In addition, many families have expressed difficulty using the resources available to them. This project will emphasize affordable childhood nutrition by offering child‐friendly cooking classes in conjunction with several community partners, providing a child‐friendly picture‐based recipe book and tools, and encouraging use of a healthy eating cooking application that translates recipes into the most common languages for our patient population.

**Project Details:** In developing this pilot program, we have drawn from successes described in the literature. Mauriello et al. (2019) outlines the importance of building a strong culinary medicine team including a physician, registered dietician, chef, and internal plus community partners along with an established curriculum, recipes, and suitable space (teaching kitchen or mobile alternatives). Our courses are designed within the Cooking Matters framework with recipes chosen with the input of a registered dietician as well as our community partners who have hosted Cooking Matters courses within the community in the past. We have also worked with the MaineHealth Food Pantry to ensure that recipes match what is readily available at the food pantry, where courses will be held. In addition to having the right team and structure for this endeavor – it must also be culturally relevant. In a scoping review on culinary and nutritional medicine interventions published between 2000 and 2019 across 5 databases, Villalona et al. (2022) found that only half of the nutritional interventions used were culturally tailored. The mobile phone culinary health cooking application that we are using not only provides recipes and instructions in the six most common languages in our clinic population, but allows for easy substitution of ingredients based on availability or preference.

We will offer 6 cooking classes over 18 months with a goal of including 5 families from the MaineHealth MMC Pediatric Clinic in each course. Our primary outcome will be uptake of this program measured by patient attendance at the cooking classes. In addition, we aim to evaluate if involvement in our program can improve diet quality and nutritional security (based on results of the Rapid Prime Diet Quality Screener and a validated 2‐question nutrition security screener) along with assesing cooking and food skills of participants using a validated tool. We will also collect qualitative assessments of the program during scheduled feedback sessions with our community partners as well as individualized interviews with class instructors (primarily pediatric residents). Our first class will be held June, 2025.

## 504 RAPID WEIGHT GAIN IN INFANTS: EXPLORING THE ROLE OF THE MICROBIOME IN EARLY DEVELOPEMENT

*Danielle Noles* ^*1*^, *Daniel Frank* ^*3*^, *Minghua Tang* ^*2*^

^*1*^ *ivision of Gastroenterology, Hepatology, and Nutrition, Department of Pediatrics*, *University of Colorado Anschutz Medical Campus*, *Aurora*, *CO*; ^*2*^ *Food Science and Human Nutrition*, *Colorado State University System*, *Denver*, *CO*; ^*3*^ *Infectious Disease*, *University of Colorado Anschutz Medical Campus*, *Aurora*, *CO*

**Background:** Rapid weight gain (RWG) during infancy has been associated with an elevated risk of both childhood and adult obesity. This increased risk of obesity in turn is linked to a range of unfavorable health outcomes including elevated lipid concentration and blood pressure. There is growing concern that microbial dysbiosis during the first year of life may contribute to rapid weight gain. This study aims to investigate the association between rapid weight gain in infants and the gut microbiota and to determine if the gut microbiota of infants with RWG differs by mode of feeding.

**Methods:** RWG defined as weight for age z‐score from birth to 5 months >0.67 was characterized amongst 211 infants (metro Denver, Colorado). Stool samples were collected around 5 months of age before solid foods were introduced and 16 s rRNA amplicon sequencing to characterize the gut microbiota. Mode of feeding, ethnicity, mode of delivery, and birth weight were also collected.

**Results:** In the first 5 months of life, 25% of the 211 infants experienced RWG. At 5 months of age, those with rapid weight gain had higher abundances of genera *Veillonella, Clostridioides* and *Lachnoclostridium*. There was a significant interaction in alpha diversity between RWG and mode of feeding (exclusively breastfed, exclusively formula‐fed and mixed‐fed), p=0.027. In brief, having rapid weight gain diminished alpha diversity in mixed‐fed infants, not in exclusively breastfed and formula‐fed infants. A significant interaction in beta diversity was also observed between RWG and mode of feeding (p=0.0072). Further evaluation using pair‐wise comparisons demonstrated that beta‐diversity differed by mode of feeding only in infants without RWG (p=1.00E‐05). For infants with RWG, their beta diversity did not differ by mode of feeding.

**Conclusion:** The prevalence of RGW in this cohort is consistent with previously reported rate. RWG appears to interact with mode of feeding to affect gut microbial composition and diversity. Infants with RWG, despite their mode of feeding, exhibit a convergence in gut microbiome beta diversity. This elucidates that RWG may exert a dominant influence over the mode of feeding on the infant gut microbiome.

## 506 DEVELOPMENT OF TAQMAN ASSAY TO DETECT TOTAL BACTERIAL LOAD FOR THE DIAGNOSIS OF SMALL INTESTINAL BACTERIAL OVERGROWTH IN CHILDREN

*Bassam Abomoelak*, *Noah Stoeckel*, *Mary Schreck*, *Nidhi Kapoor*, *Chirajyoti Deb*, *Khoa Pham*, *Devendra Mehta*, *Vijay Mehta*, *William Morgan*, *Edwin Ballelos*, *Katrina Tiqui*

*Peds Specialty Diagnostic Lab*, *Orlando Health*, *Orlando*, *FL*

**Background:** Small Intestinal Bacterial Overgrowth (SIBO) is associated with several gastrointestinal diseases in children and adults. The current hydrogen and methane breath test (BHT) offers early indications about SIBO, but has a very low sensitivity and specificity to diagnose SIBO. Furthermore, correlation studies have shown that BHT corelates poorly with symptoms of SIBO. Duodenal aspirates have more commonly been used for this purpose, with the clinical field adopting a recently defined cutoff of >10^3 colony‐forming units (CFU) per milliliter as the standard for diagnosing SIBO. This threshold was established based on studies in the adult population and relies on both aerobic and anaerobic culturing of duodenal aspirates. In children, SIBO lacks valid and accurate diagnosis, and it depends largely on BHT data that has apparent limitations. Our lab developed a duodenal mucosal brushing method that can be done during endoscopy on patients with symptoms of SIBO. Using duodenal brushing samples, our group has developed a semi‐quantitative molecular test, using a commercially available TaqMan^TM^ Microbe Detection assay (Applied Biosystems) targeting bacterial 16SrRNA gene. In this study, we compared the potential to diagnose SIBO using four different methods: the breath hydrogen test (BHT), quantitative culturing on selective aerobic and anaerobic media plates, semi‐quantitative Gram staining, and the 16S RNA gene TaqMan assay.

**Methodology:** 87 (mean age 12.2 ± 4.57 years) SIBO samples (duodenal brushing samples collected using a cytology brush) collected over the last two years in our laboratory, were used in this study. 66 of these samples were from pediatric patients who underwent BHT and quantitative microbial culturing for SIBO diagnosis. Aerobic and anaerobic culture data were also available along with Gram stain results for this patient cohort. In addition, clinical data on associated symptoms were collected. To establish a quantified standard curve for Taqman assay, serial dilutions of *Haemophilus influenzae* were used correlating CFU and bacterial DNA quantity. The total genomic DNA was extracted from 87 SIBO samples and the standard control using the Powersoil DNA extraction kit (Qiagen) and the Qiacube automated system. Real time PCR was performed using an inventoried TaqMan Microbe Detection Assay for 16S from Applied Biosystems (16S Pan‐bacterial Control Ba04230899_s1). With the established standard, we estimated the bacterial load in every sample at the CFU and DNA quantity levels and correlated the TaqMan qPCR data with BHT, Gram stain, and aerobic/anaerobic quantitative cultures. Additionally, a combination of logistic regression measurements and Receiver Operating Characteristic (ROC) was performed to determine the diagnostic potential of each method for SIBO.

**Results:** Of the 87 patients 66 had culture, Gram stain and BHT results. Logistic Regression with Gram stain results showed a statistically significant association between Bacterial DNA load (pg/mL) and SIBO diagnosis using Gram staining data. The ROC curve analysis suggests the threshold for diagnosis would be 309.297 pg/mL with a sensitivity of 0.7971 and specificity of 0.8235. There is a statistically significant association between Bacterial DNA load and diagnosis with culturing. The ROC curve suggests the threshold for diagnosis would be 605.48 pg/mL with a sensitivity of 0.333 and specificity of 0.91429. The ROC curves for Gram staining and culturing are not statistically significantly different from each other (Delong's test, D = 1.9104, df = 170, p‐value = 0.05776). For bacterial counts, the Bacterial DNA load more strongly correlated with aerobic growth than anaerobic growth. The Bacterial DNA count did not statistically significantly correlate with any of the hydrogen or methane variables from the BHT when we looked at all 66 individuals. This also held true when looking at subgroups: high baseline hydrogen vs normal, high baseline methane vs normal, and within diagnoses. For every 1 pg/mL that the Bacterial DNA load increases, the odds do not change. The area under the curve was (AUC=0.5), the same as chance.

**Conclusion:** A new molecular test based on TaqMan real‐time PCR detecting total bacterial load targeting bacterial 16S rRNA gene was compared to SIBO results obtained by quantitative bacterial culture, Gram stain, and BHT. The new test correlated better with culture and Gram stain than BHT. The threshold for diagnosing SIBO based on the Gram staining outcome was 309 pg/mL, which lead to a high sensitivity and high specificity while for culturing it was 605 pg/mL, which leads to a high specificity but low sensitivity.

## 507 LINKING INTESTINAL INFLAMATION, MICROBIOTA AND PSYCHOLOGICAL SYMPTOMS IN A PEDIATRIC COHORT

*Wesley Tom* ^*2*^, *Samantha Sack* ^*1*^, *Sharad Kunnath* ^*1*^, *Anna Trauernicht* ^*1*^, *M. Rohan Fernando* ^*2*^, *Rose Pauley* ^*1*^, *Tony Wilson* ^*3*^, *Jon Vanderhoof* ^*1,4*^

^*1*^ *Pediatric GI*, *Boys Town National Research Hospital*, *Boys Town, NE*; ^*2*^ *Center for Sensory Neuroscience*, *Boys Town National Research Hospital*, *Boys Town*, *NE*; ^*3*^ *Boys Town Institute for Human Neuroscience*, *Boys Town*, *NE*; ^*4*^ *Pediatric GI*, *Boston Children's Hospital*, *Boston*, *MA*

Children experiencing gastrointestinal discomfort often exhibit psychological symptoms such as anxiety and depression. In recent years, a growing body of research has emphasized the importance of the gut‐brain axis (GBA).The intestinal microbiota is an essential player in this bi‐directional system, as it influences neurodevelopment and behavior via neurotransmitter synthesis, immune modulation, and microbial metabolite signaling, including short‐chain fatty acids (SCFAs).

The objective of this study is to investigate the relationship between gut microbiota, psychological symptoms, and inflammation in children with and without GI complaints. The aim was to determine whether microbial taxa and functional pathways are associated with symptoms of anxiety and depression, if inflammation correlates with behavioral difficulties, and whether stool samples reflect broader microbial patterns along the intestinal tract that explain emotional dysregulation.

**Methods:** The study included 60 participants aged 11 to 18 years, divided into three groups: 30 healthy controls providing fecal samples, 25 patients referred for colonoscopy due to abdominal pain providing both fecal and cecal samples, and 5 asymptomatic individuals who showed elevated fecal calprotectin.Psychological assessment tools included the Patient Health Questionnaire‐9, Generalized Anxiety Disorder‐7, and the Behavioral Assessment System for Children. DNA was extracted and analyzed using shotgun metagenomic sequencing on the Illumina platform, with taxonomic and functional profiling conducted via HUMAnN3 and statistical correlation using MaAsLin2. Psychological scores on the BASC‐3 were interpreted as elevated when they exceeded one to two standard deviations above the normative mean.

**Results:** Symptomatic children demonstrated significantly higher rates of anxiety and depression compared to healthy controls, with anxiety (p < 0.05) and depression (p < 0.01) scores elevated on the GAD‐7 and PHQ‐9, respectively. These findings were mirrored on the BASC‐3, which also showed elevated scores in the corresponding anxiety and depression domains (p < 0.05). Eleven bacterial species were significantly lower in abundance in individuals with elevated PHQ‐9 depression scores; *Anaerostipes amylophilus*, *Blautia_A ammoniilytica, Blautia_A sp900066355, Parasutterella excrementihominis, Suilimivivens aceti, CAG‐170 sp003516765, Monoglobus pectinilyticus, Bifidobacterium adolescentis, Bifidobacterium sp959020155, Collinsella sp938005465, Collinsella sp900541245*. Interersingly, only two species were differentially abundant between fecal and cecal samples, *Lachnospira pectinoschiza_A* and *Hominilimicola fabiformis*, both of which were in higher abundance in feces compared to the cecum. Functional profiling revealed overrepresentation of microbial metabolic pathways involved in host‐immune interaction and neurochemical synthesis. These included coenzyme‐A biosynthesis, phosphopantothenate metabolism, and CMP‐legionaminate biosynthesis. An increase in sucrose biosynthesis from an unknown *Clostridium* species was observed, suggesting an uptick in energy‐producing microbial metabolism in samples associated with emotional dysregulation.

**Conclusion:** These results suggest that emotional symptoms, particularly anxiety and depression, are associated with specific microbial features. The enrichment of microbial pathways related to coenzyme metabolism, immune evasion, and fermentative energy production highlights plausible biological mechanisms through which gut microbes may influence mood and behavior. Analysis indicates that elevated markers of intestinal inflammation are correlated with emotional symptoms.


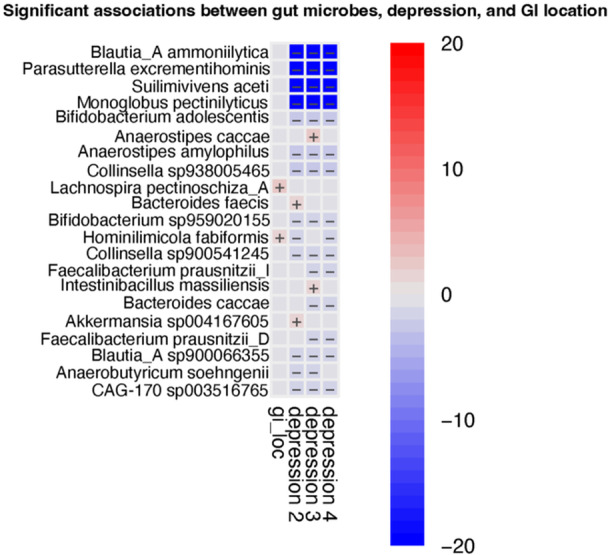


Figure 1. Significantly different microbial taxa as predicted by Maaslin2 differential abundance analysis. Cooler blue colors indicate negative correlation coefficients, meaning lower abundance in the gouping column. Red colors indicate positive correlation coefficients, indicating higher abundance in the grouping column.

## 508 IN CHILDREN WITH NEUROLOGICAL IMPAIRMENT, ARE GASTROESOPHAGEAL REFLUX HELPFUL TO SELECT WHO WILL BENEFIT WITH A FUNDOPLICATION WHEN A GASTROSTOMY HAS BEEN INDICATED?

*Judith Cohen Sabban* ^*2*^, *Jhoanna Adauto Luizaga* ^*1*^, *Maria Manin* ^*1*^, *Luis Benavidez* ^*1*^, *Florencia Ursino* ^*2*^, *Mauricio Urquizo* ^*3*^, *Marina Orsi* ^*2*^, *Veronica Busoni* ^*2*^

^*1*^ *Fellow in Pediatric Gastroenterology*, *Hospital Italiano de Buenos Aires*, *Buenos Aires*, *Buenos Aires*, *Argentina*; ^*2*^ *Pediatric Gastroenterology*, *Hospital Italiano de Buenos Aires*, *Buenos Aires*, *Buenos Aires*, *Argentina*; ^*3*^ *Pediatric Surgery Unit*, *Hospital Itlaliano*, *Buenos Aires*, *Argentina*

**Introduction:** The need of Nissen fundoplication (NF) in children with neurological impairment before a gastrostomy procedure is always a matter of discussion. In many centers surgical strategy is based on X Ray studies and clinical presentations. In the last decade we have implemented an endoscopy evaluation together with a 24 hr Impedance/Phmetry study (24 hr pH/MII) and shared surgical visit before a gastrostomy procedure.

**Objective:** To analyze the existence of GERD in patients with neurological impairment before a gastrostomy procedure (GTT). To select those for NF and to evaluate the proton pump inhibitors (PPI) requirement after the procedure.

**Methods:** A retrospective and observational study was conducted from January 2015 to December 2024. All pediatric patients with neurological impairment requiring GGT followed at a reference center were evaluated All of them underwent a 24 hr pH/MII study and an upper GI endoscopy off‐PPI. Los Angeles score was used to define endoscopic esophagitis and the total number of reflux episodes (acid/non‐acid), bolus clearance and acid exposure percentage in the pH/MII tracings to define GERD. Patients were divided according to symptoms: symptomatic (GI) and asymptomatic patients (GII). Symptoms in GI included vomiting, regurgitation, irritability, fuzzing, gagging and/or respiratory.

**Results:** A total of 73 children with neurological impairment were included, 38 male, median age 3.7 years (IQR 1.41‐8.65). Asymptomatic patients were 34% (25/73). Among symptomatic patients (48/73), the most common symptoms were vomiting/regurgitation (77%), irritability (21%) and respiratory symptoms (2%). 55% (40/73) underwent GTT alone and 45% (33/73) underwent GTT + NF. In upper GI endoscopy was observed esophagitis Los Angeles score C‐D: GI 9/48 (19%) and GII 1/25 (4%). In 24 hr pH/MII study were pathological: GI 23/48 (49%) and GII 5/25 (20%). According to the results of the complementary studies conducted on the patients, see table 1.

After the procedure, we observed that the patients who underwent GTT alone, 80% (32/40) remained asymptomatic and only 20% again required the need for the use of proton pump inhibitors (PPIs) vs 52% (17/33) of the GTT+NF group (p 0.02).

**Conclusion:** In this cohort of children with neurological impairment, the endoscopy results together with the 24 hr pH‐MII monitoring data and a shared surgical visit helped to better identify who would benefit from adding a NF to GGT procedure. The low PPI requirement in those without fundoplication reinforces the idea of being cautious when defining surgical strategies in this complex group of patients.


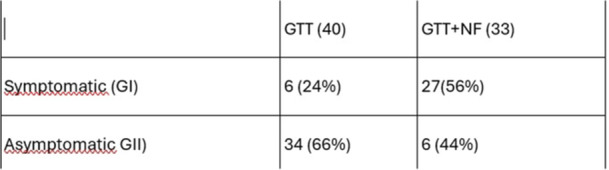


## 512 THE RELATIONSHIP BETWEEN GASTROESOPHAGEAL REFLUX AND CONSTIPATION

*Charlotte Banayan* ^*1*^, *Simon Rabinowitz* ^*2*^

^*1*^ *Pediatrics*, *SUNY Downstate Health Sciences University*, *Brooklyn*, *NY*; ^*2*^ *Pediatric Gastroenterology*, *SUNY Downstate Health Sciences University*, *New York*, *NY*

**INTRO:** Gastroesophageal reflux (R) and constipation (C) are two of the most common indications for pediatric gastroenterology or primary care visits. While several small series have linked these two dysmotility entities, the present report examines this relationship in a larger patient cohort.

**METHODS:** TriNetX, a worldwide database of electronic medical records from >100 million patients over >80 healthcare organizations, contains >20 million patients who have constipation (C+) and >10 million who have reflux (R+). C+ was based on ICD‐10 diagnoses (K59.0, K56.41, R15.0) or documented laxative (polyethylene glycol 3350, senna leaf extract, senna leaves, lactulose, psyllium, wheat dextrin, bulk‐forming laxatives, hyperosmotic laxatives, lubricant laxatives, stimulant laxatives, stool softener, prucalopride, and lubiprostone) use. R+ was defined by ICD‐10 diagnoses (K20, K21, R12, K44). A group of patients with R or who received acid suppression therapy (magnesium hydroxide, simethicone, sodium bicarbonate, rabeprazole, lansoprazole, esomeprazole, pantoprazole, omeprazole, dexlansoprazole, alginic acid, histamine antagonists, various antacids), was also analyzed (RA+). Exclusion criteria were drug‐induced constipation, Hirschsprung's disease, or congenital absence, atresia, or stenosis of the anus without fistula for C, and esophageal atresia without fistula or eosinophilic esophagitis for R and RA.

The total population (TP) in the database was divided into C+ and C‐, into R+ and R‐, and into RA+ and RA‐. The prevalence rates were then determined for each pair of clinical subgroups, and the ratio of the prevalence rates was defined as the associated risk. The subgroups are described below.

Among R+ and R‐, what proportion also had C: (C+R+)/R+ and (C+R‐)/R‐.

Among C+ and C‐, what proportion also had R: (R+C+)/C+ and (R+C‐)/C‐.

Among C+ and C‐, what proportion also had RA: (RA+C+)/C+ and RA+C‐)/C‐.

These associations were examined over the entire patient population and throughout childhood. The results are available in the included table.

**RESULTS:** C (22.4 million, 17.2% of TP) and R (10.8 million, 8.3% of TP) were common diagnoses. Each condition was diagnosed 4 to 7 times more frequently in patients diagnosed with the other (Table). For constipated children, the likelihood of receiving acid suppression was even more pronounced compared to those with normal bowel habits.

**CONCLUSION:** C and R appear to frequently coexist from early childhood through adolescence. For children with C or R, utilizing validated questionnaires and physical examination to diagnose if the second is also present appears to have clinical benefit. Treating both functional disorders in the same encounter could decrease these children's discomfort and improve their quality of life while also reducing the financial and psychosocial burden of either condition.


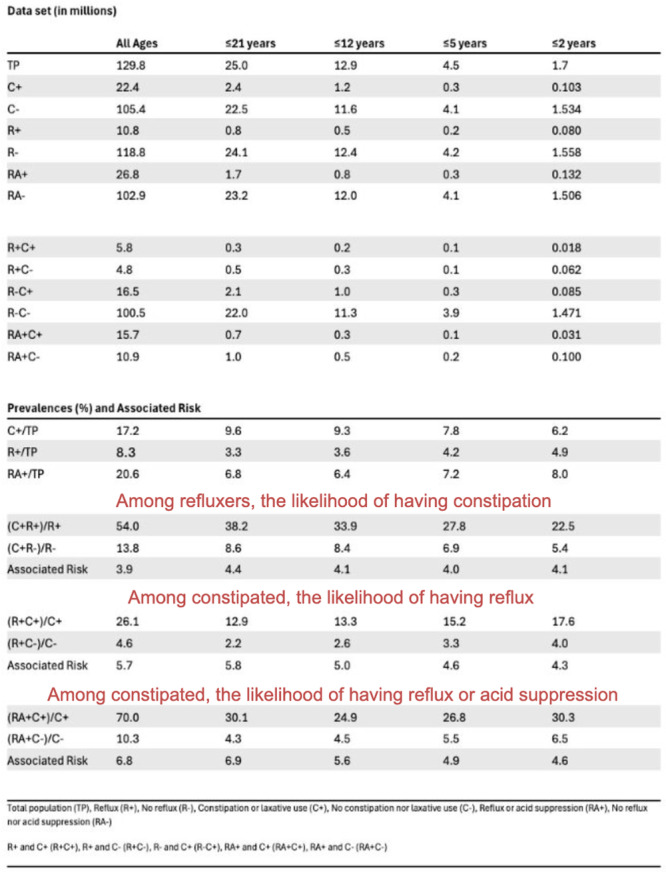


## 514 NAVIGATING THE CHALLENGES OF PEDIATRIC CONSTIPATION IN SCHOOLS: INSIGHTS FROM A SURVEY OF SCHOOL NURSES

*Andrew Chu* ^*1,2*^, *Eric Chiou* ^*1,2*^

^*1*^ *Pediatrics*, *Baylor College of Medicine*, *Houston*, *TX*; ^*2*^ *Gastroenterology, Hepatology & Nutrition*, *Texas Children's Hospital*, *Houston*, *TX*

**Objectives:** Constipation and encopresis are common pediatric conditions that can significantly impact student well‐being and school functioning. School nurses are frontline responders to these issues, yet their experiences are underreported. This study aimed to explore school nurses’ observations regarding constipation, identify barriers to student restroom access, and assess educational needs.

**Methods:** An anonymous 20‐question online survey was distributed to 1,000 nurses participating in a virtual school nurse continuing education series in May 2024. The survey assessed nurse demographics, frequency of encounters with constipation and encopresis, school restroom policies, barriers to restroom use, accommodation requests, and training in constipation management. Responses were collected over two weeks. Statistical analysis utilizing Fisher's exact test was performed using GraphPad Prism (Boston, MA).

**Results:** Of 1,000 invited, 125 nurses responded (12.5%). Most worked in public schools and covered Pre‐K through elementary levels. Nearly all (94%) encountered students with constipation at least monthly and 55% reported ≥6 encounters monthly; 60% reported seeing encopresis at least monthly. Collectively, middle and high school nurses were significantly less likely than Pre‐K and elementary nurses to encounter encopresis, with 73% of middle and high school nurses reporting no cases of encopresis in an average month compared with 26% of Pre‐K and elementary school nurses (p < 0.0001). Commonly perceived barriers to restroom use included unclean bathrooms (48%), bullying (42%), drug use (23%), vandalism (18%), and a variety of other concerns (42%) that included lack of privacy, fear of potential social media exposure, and fear of automatic toilets. Half of schools relied on teacher discretion for restroom access; only 37% of nurses were aware their schools had formal policies. Approximately 64% of respondents received accommodation requests from parents or medical providers at least monthly. The most common requests included increased restroom access (82%), permission for students to carry water bottles during the school day (81%), extended restroom time (54%), and access to a private restroom (51%). Only 38% had training in constipation management, and most nurses expressed interest in additional education. When asked about preferred educational resources, 88% of nurses expressed interest in learning how to identify and treat abnormal toileting habits, 83% wanted more information on childhood constipation, and 67% requested guidance on recommended fluid intake.

**Conclusions:** Constipation and encopresis are frequent concerns in schools, particularly in younger grades. Environmental and policy‐related barriers may contribute to toileting difficulties. School nurses report limited training and variable communication with providers, underscoring the need for targeted educational interventions and school‐wide policy development to support students with constipation.

*Statement: During the preparation of this work the authors used Elicit AI in order to assist in literature search. After using this tool/service, the authors reviewed and edited the content as needed and take full responsibility for the content of the publication*.


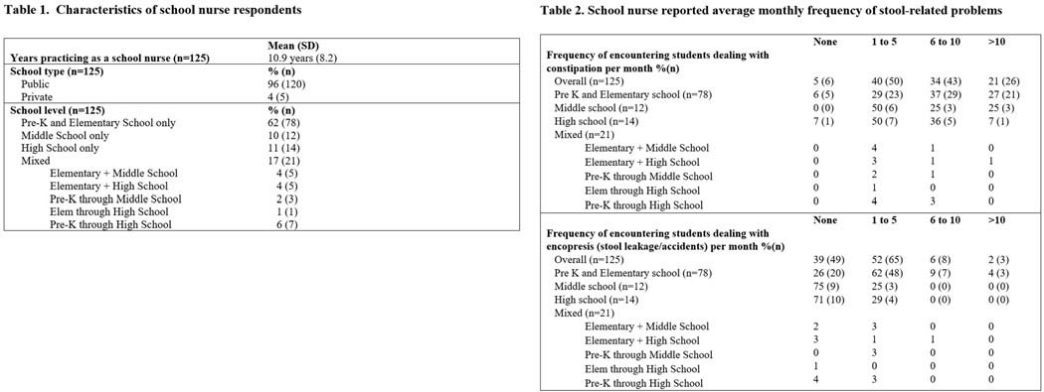


Tables 1 and 2


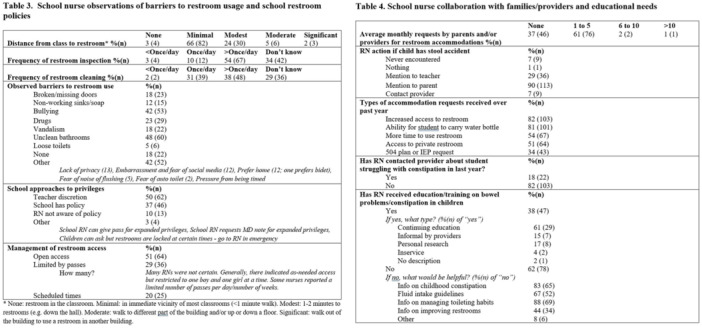


Tables 3 and 4

## 515 CHARACTERIZATION OF MANOMETRIC FINDINGS IN ADOLESCENT PATIENTS WITH DYSSYNERGIC DEFECATION

*Mustafa Sadek* ^*1,2*^, *Alaa Almallouhi* ^*1*^, *Olla Darwish* ^*1*^, *Louai Manini* ^*1*^, *Kristin Cole* ^*3*^, *Yamen Ezaizi* ^*1*^

^*1*^ *Pediatric Gastroenterology*, *Mayo Clinic Minnesota*, *Rochester*, *MN*; ^*2*^ *NCH Healthcare System*, *Naples*, *FL*; ^*3*^ *Division of Biomedical Statistics and Informatics*, *Mayo Clinic Minnesota*, *Rochester*, *MN*

**Introduction:** Dyssynergic defecation (DD) is a common but often underdiagnosed cause of constipation in children and adults. Diagnosis typically involves anorectal manometry (ARM) and rectal evacuation tests such as balloon expulsion time (BET), barium, or MR defecography. This study aims to describe and compare anorectal manometry findings in constipated adolescents with and without DD.

**Methods:** We retrospectively reviewed records of patients ≤18 years old who underwent ARM and/or BET between 2008 and 2019. Patients with prior pelvic floor therapy, inflammatory bowel disease, or colon surgery (e.g., ostomy, colectomy) were excluded. DD was diagnosed based on abnormal ARM (paradoxical anal contraction or inadequate anal relaxation [≤20%] during simulated evacuation) and/or an abnormal BET (>60 seconds). Manometric parameters were compared between patients with and without DD, and between those with abnormal vs. normal BET. We assessed performance of various rectoanal gradient cut‐offs determining abnormal BET sensitivity, specificity, positive predictive value (PPV), and negative predictive value (NPV). Wilcoxon rank sum tests compared continuous variables, and Chi‐square or Fisher's exact tests compared categorical data between groups.

**Results:** Of 187 adolescents with constipation, 69 (36.9%) met criteria for DD. The median age was 17 years (IQR 16–18), and 80.7% were female. Gastrointestinal symptoms did not differ between groups. Patients with DD had a significantly more negative rectoanal pressure gradient than those without DD [median (IQR): ‐49 mmHg (‐73.9, ‐31.4) vs. ‐25.2 mmHg (‐40.2, ‐9.8); p < 0.001]. Volume thresholds for first sensation, urge, and discomfort were similar. A rectoanal gradient cutoff of ‐45 mmHg showed the best predictive value for DD (sensitivity 58%, specificity 86%). Patients with DD also had significantly higher rates of depression (p < 0.05). Similarly, those with abnormal BET had more negative rectoanal gradients than those with normal BET [median (IQR): ‐48.6 mmHg (‐79.5, ‐33.2) vs. ‐29.4 mmHg (‐53.2, ‐13.4); p < 0.001].

**Conclusion:** In this cohort, over one‐third met criteria for DD, underscoring its clinical relevance in this population. The rectoanal pressure gradient appears to be a useful manometric parameter for identifying DD in constipated adolescents, particularly when BET is unavailable. Psychological factors such as depression may also be more prevalent in this group, highlighting the importance of psychosocial screening in adolescents with constipation.


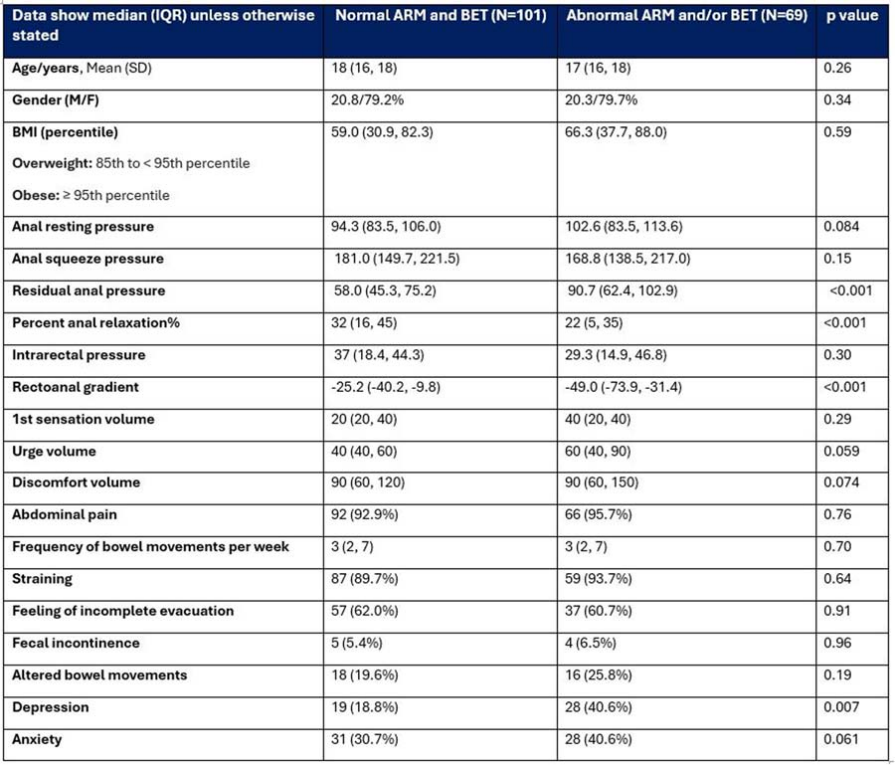


Table 1: Demographics, Symptoms and Co‐morbidities.


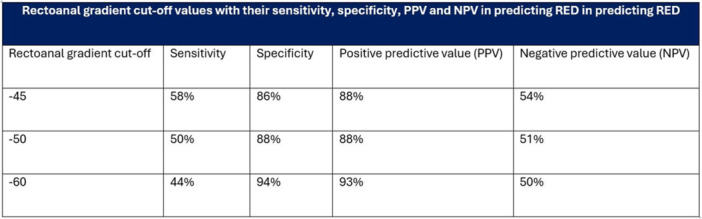


Table 2: Results of anorectal manometry.

## 516 TRENDS IN PEDIATRIC ED VISITS AND HOSPITALIZATIONS FOR GASTROINTESTINAL COMPLICATIONS IN PEDIATRIC PATIENTS WITH CYSTIC FIBROSIS PRE AND POST MODULATOR THERAPY ‐ A MULTICENTER RETROSPECTIVE COHORT ANALYSIS

*Christiana Ekezie* ^*1*^, *Cara Mack* ^*1*^, *Nicholas Antos* ^*1*^, *Ke Yan* ^*1*^, *Liyun Zhang* ^*1*^, *John Morrison* ^*2*^, *Racha Khalaf* ^*3*^

^*1*^ *Pediatrics*, *Medical College of Wisconsin*, *Milwaukee*, *WI*; ^*2*^ *Johns Hopkins All Children's Hospital*, *St. Petersburg*, *FL*; ^*3*^ *University of South Florida Morsani College of Medicine*, *Tampa*, *FL*

**BACKGROUND:** Recent studies have reported improvement in pulmonary outcomes in pediatric patients with Cystic Fibrosis (PwCF) since the introduction of highly effective modulator therapy (HEMT). However, HEMT effects on the gastrointestinal manifestations of CF, especially constipation and intestinal obstruction, remains largely unknown. This study aims to compare the rates of emergency department (ED) visits and hospitalizations for constipation and intestinal obstruction in PwCF in the eras pre and post HEMT.

**METHODS:** A retrospective cohort study on the rates of ED visits and hospitalizations for constipation or intestinal obstruction (surrogate for distal intestinal obstruction syndrome) in pediatric PwCF pre and post HEMT was performed using data from the Pediatric Health Information System (PHIS) ® database. The PHIS database includes medical information from ~45 children's hospitals; data on ages 2‐18 years of age was analyzed. Cohorts for comparison included PwCF from 2014‐2018 (pre‐HEMT) and PwCF from 2020‐2024 (post‐HEMT). Mann‐Whitney Wilcoxon test was used to examine the differences of continuous variables in the two groups and Chi‐square and Fisher's exact tests were used to examine the group differences of categorical variables. A p‐value<0.05 was considered statistically significant. All statistical analyses were performed using SAS 9.4(SAS Institute Inc., Cary, NC).

**RESULTS:** A total of 486 ED visits and 4197 hospitalizations in PwCF were identified with a primary or secondary diagnosis for constipation in the pre‐ and post‐HEMT cohorts. Demographics between the cohorts were similar except for payor mix and ethnicity. With constipation as a primary or secondary diagnosis in PwCF, a significant decline in ED visits was observed between the pre‐and post‐HEMT cohorts (295 ED visits pre‐HEMT, 191 ED post‐HEMT; p=0.012, Fig. 1 A). Inpatient hospitalizations also significantly decreased (2631 pre‐HEMT, 1566 post‐HEMT; p=0.012, Fig.1B). Sub‐analysis of PwCF cohorts where constipation was the primary diagnostic code revealed approximately a 2‐fold reduction in ED visits (252 ED pre‐HEMT, 155 ED post‐HEMT) and hospitalizations (297 pre‐HEMT, 161 post‐HEMT).

With regard to intestinal obstruction, a total of 13 ED visits and 173 hospitalizations were identified in PwCF in the pre‐ and post‐HEMT cohorts. A 2‐3‐fold decrease was observed in the post‐HEMT cohorts for both ED visits and hospitalizations (ED visits: 9 pre‐HEMT, 4 post‐HEMT; hospitalizations: 131 pre‐HEMT, 42 post‐HEMT).

**CONCLUSION:** Our results from a large national database demonstrate a significant reduction in rates of ED visits and hospitalizations for primary diagnoses of constipation and intestinal obstruction in the pediatric population following introduction of HEMT. These results are encouraging as these diagnoses are associated with a significant burden on quality of life in patients with CF. Future work will identify whether the inpatient management for these indications varied between the two time periods.


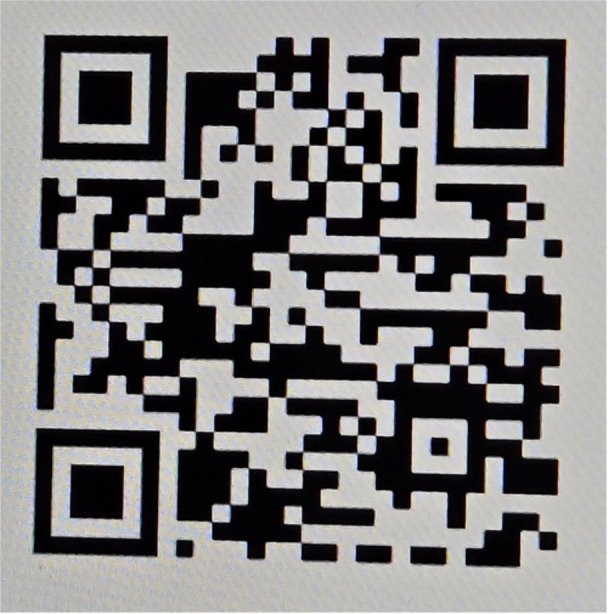



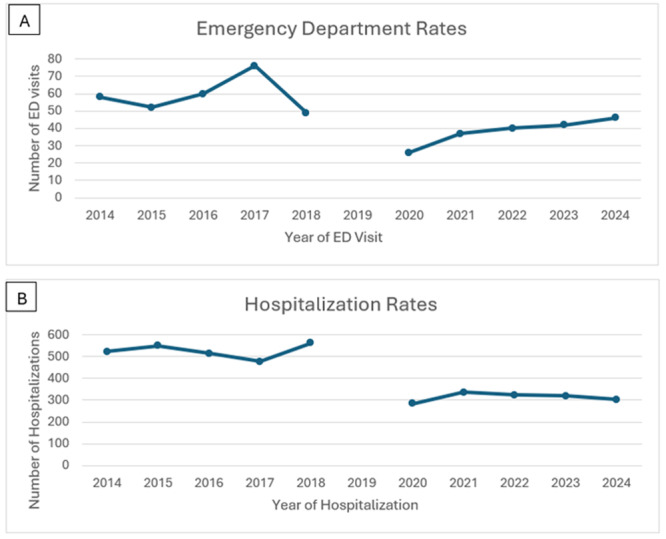


## 517 FUNCTIONAL NAUSEA IS NOT JUST NAUSEA: A MULTI‐CENTER STUDY

*Lauren Hagenstein* ^*1*^, *Khalil El‐Chammas* ^*2*^, *Asish Chogle* ^*5*^, *Neha Santucci* ^*2*^, *Kahleb graham* ^*2*^, *Lev Dorfman* ^*2*^, *Daniel Kelly* ^*4*^, *Jason Dranove* ^*4*^, *Rachel Rosen* ^*3*^, *Samuel Nurko* ^*3*^, *Joseph Croffie* ^*6*^, *Pippa Simpson* ^*1*^, *Katja Karrento* ^*1*^

^*1*^ *Department of Pediatrics*, *Medical College of Wisconsin Department of Pediatrics*, *Milwaukee*, *WI*; ^*2*^ *Cincinnati Children's Hospital Medical Center*, *Cincinnati*, *OH*; ^*3*^ *Boston Children's Hospital*, *Boston*, *MA*; ^*4*^ *Department of Pediatrics*, *Atrium Health Levine Children's Brenner Children's Hospital*, *Winston‐Salem*, *NC*; ^*5*^ *Department of Pediatrics*, *Children's Hospital of Orange County*, *Orange*, *CA*; ^*6*^ *Department of Pediatrics*, *Indiana University*, *Bloomington*, *IN*

**Background:** Chronic nausea is a common diagnostic and therapeutic dilemma among pediatric gastroenterologists. Studies have linked nausea associated with disorders of gut‐brain interaction (DGBI) with various comorbidities and poor quality of life. Rome IV criteria defines pediatric functional nausea (FN) as isolated nausea without emesis or meal‐related symptoms. Extra‐intestinal comorbidities are poorly characterized in children with FN. This multicenter study aimed to investigate the multi‐system symptom burden, quality of life and functioning in children with FN in comparison with children with non‐FN DGBI.

**Methods:** Children ages 8‐18 years with DGBI were enrolled in a prospective, multi‐center registry study across six different gastroenterology clinics. DGBI subtypes were classified by Rome IV criteria. Subjects who met criteria for FN underwent detailed symptom profiling. Subjects completed the following questionnaires with comparisons to non‐FN DGBI: 1) Children's Somatization Inventory (CSI), 2) Functional Disability Inventory (FDI), 3) PROMIS Global Health Scale (GHS) 4) PROMIS Pediatric Anxiety, 5) PROMIS Pediatric Depression, 6) Nausea Severity Scale (NSS) and 7) Abdominal Pain Index (API). For group comparisons, a Fisher exact test was used for analyzing categorical data and a Mann‐Whitney U test was used for continuous data.

**Results:** A total of 90 subjects, 50 with FN and 40 with non‐FN DGBI were analyzed. Majority (74%) of FN patients were female with median (IQR) age 16.5 (14.3, 18.7) years. Of the FN patients, 76% had chronic nausea for > 2 years and 61% had failed ≥4 pharmacological agents. A large proportion complained of chronic abdominal pain (79%), headaches (76%) and recurrent dizziness/lightheadedness (48%). 78% of the FN subset who underwent comprehensive autonomic testing were abnormal. Among FN patients, 41% had a formal mental health diagnosis and 36% were diagnosed with major depression. There was a trend towards higher CSI median score in FN compared to non‐FN group (p=0.069). Patients with FN had significantly greater functional disability compared to non‐FN: median (IQR) scores 19.5 (11.0, 27.3) vs. 13.0 (6.3, 21.3) respectively (p=0.018). Significant differences between the FN compared to non‐FN group included lower quality of life (PROMIS GHS; p=0.007), higher anxiety (p<0.001) and higher depression (p<0.001). There were no statistically significant group differences in NSS and API scores (p=0.29 and p=0.19 respectively).

**Conclusions:** Children who meet criteria for functional nausea do not suffer from isolated nausea. Majority of FN patients have chronic abdominal pain and display a high extra‐intestinal symptom burden. These comorbidities may contribute to the reduced functioning, worse quality of life and high frequency of mental health conditions seen in FN compared to non‐FN patients. Diagnostic categorizations and treatment interventions need to recognize this multi‐faceted symptom complex to improve outcomes.

## 520 CO‐DEVELOPMENT, FEASIBILITY, AND PRELIMINARY VALIDITY OF A DIGITAL GASTRIC SYMPTOM AND WELLBEING DIARY FOR ADOLESCENTS WITH GASTRODUODENAL DGBIS

*Gayl Humphrey* ^*1,2*^, *Mikaela Law* ^*1*^, *Armen Gharibans* ^*3,2*^, *Stefan Calder* ^*3,2*^, *Binghong Xu* ^*4*^, *Christopher Andrews* ^*5*^, *Alain Benitez* ^*4,6*^, *Hayat Mousa* ^*4,6*^, *Gregory O'Grady* ^*1,2*^

^*1*^ *Dept of Surgery*, *The University of Auckland*, *Auckland*, *New Zealand*; ^*2*^ *Alimetry, Ltd*., *Auckland*, *New Zealand*; ^*3*^ *Bioengineering*, *The University of Auckland*, *Auckland*, *Auckland*, *New Zealand*; ^*4*^ *The Children's Hospital of Philadelphia*, *Philadelphia*, *PA*; ^*5*^ *Gastroenterology*, *University of Calgary*, *Calgary*, *AB*, *Canada*; ^*6*^ *University of Pennsylvania*, *Philadelphia*, *PA*

**Background:** Self‐reported gastric symptoms and quality of life in adolescents are often collected via structured surveys but recall bias can distort reporting. Daily symptom tracking can mitigate this. Mobile applications provide a user‐friendly, accessible way to support real‐time, consistent, and accurate reporting. This study used a user‐centred design approach to develop and preliminary validate a daily gastric symptom and wellbeing diary App with adolescents aged 12–18 diagnosed with a gastroduodenal disorder of gut‐brain interaction (DGBI).

**Methods:** Phase 1 mapped the symptoms and well‐being concepts to validated pediatric questionnaires^1, 2^. Phase 2 interviewed adolescents to assess comprehension of gastric symptom pictograms^3^, and develop quality‐of‐life questions. Phase 3 developed a prototype app (Fig 1), and adolescents recorded their symptoms for 14 days. Feasibility, acceptability and preliminary construct validity were evaluated by comparing daily symptoms to a questionnaire completed on day 14.

**Results:** Phase 2 interviewed 5 adolescents (mean age 15; range 12‐17: female 3), confirmed pictograms were understandable, and refined and confirmed the well‐being questions. 14 adolescents (median age 15; range 12‐17: 8 female) with Functional Dyspepsia (FD) (n=2) and FD and Functional Constipation (n=2) used the App for 14 days; 13 completed the daily diary for all 14 days and 1 completed for 13 days. All reported that liked the App and it easy to use. The all preferred having a text reminder each day and agreed that setting their best time for that was important. Recalling belch and reflux events were reported as difficult and suggestions were to convert it to a severity scale instead of a count. The daily symptom scores recorded in the App were less severe than the recall scores for upper gut pain (mean App 78.7 (SD 10.6) v 45.7 (27.3), *p* <0.001), bloating (mean App 80.7 (SD 9.7) v 70.5 (SD 27.4), *p*=0.04) and excessively full (mean App 85.1 (SD 12.7) v 50 (SD 36.6) *p*=0.003). Similarly, daily reporting of Anxiety (98.6 (SD 2.99) v 58.9 (22.04), *p* <0.001) and quality of life (QoL) (95.3 (SD 5.97) v 79.8 (SD 8.5), *p*<0.001) were less severe than recall. Overall symptom severity was also lower using the App than when patients recalled severity of symptoms (Mean App 2.1 (SD 0.41) v 2.9 (SD 1.2), p= 0.027).

**Conclusion:** Reporting daily diary outcomes provided a clearer picture of symptoms experienced, severity and impact on QoL over 14 days than that of recall at 14 days using questionnaire responses. The poorer recall score outcomes may reflect a recall bias, where emotionally vivid events are remembered more than mundane ones. Daily symptom tracking can reduce this bias and be a valuable tool for monitoring treatment progress and assessing intervention effectiveness than the use of recall questionnaires.

1. Varni et al. (2015) PedsQL™ Gastrointestinal Symptoms Scales and Gastrointestinal Worry Scales in pediatric patients with functional and organic gastrointestinal diseases in comparison to healthy controls." Quality of life research **24**(2): 363‐378.

2. Varni et al. (2015) ealth‐Related Quality of Life in Pediatric Patients with Functional and Organic Gastrointestinal Diseases." The Journal of pediatrics **166**(1): 85‐90.e82.

3. Humphrey et al. (2024) Designing, Developing, and Validating a set of Standardized Pictograms to Support Pediatric‐reported Gastroduodenal Symptoms." The Journal of Pediatrics https://doi.org/10.1016/j.jpeds.2024.113922


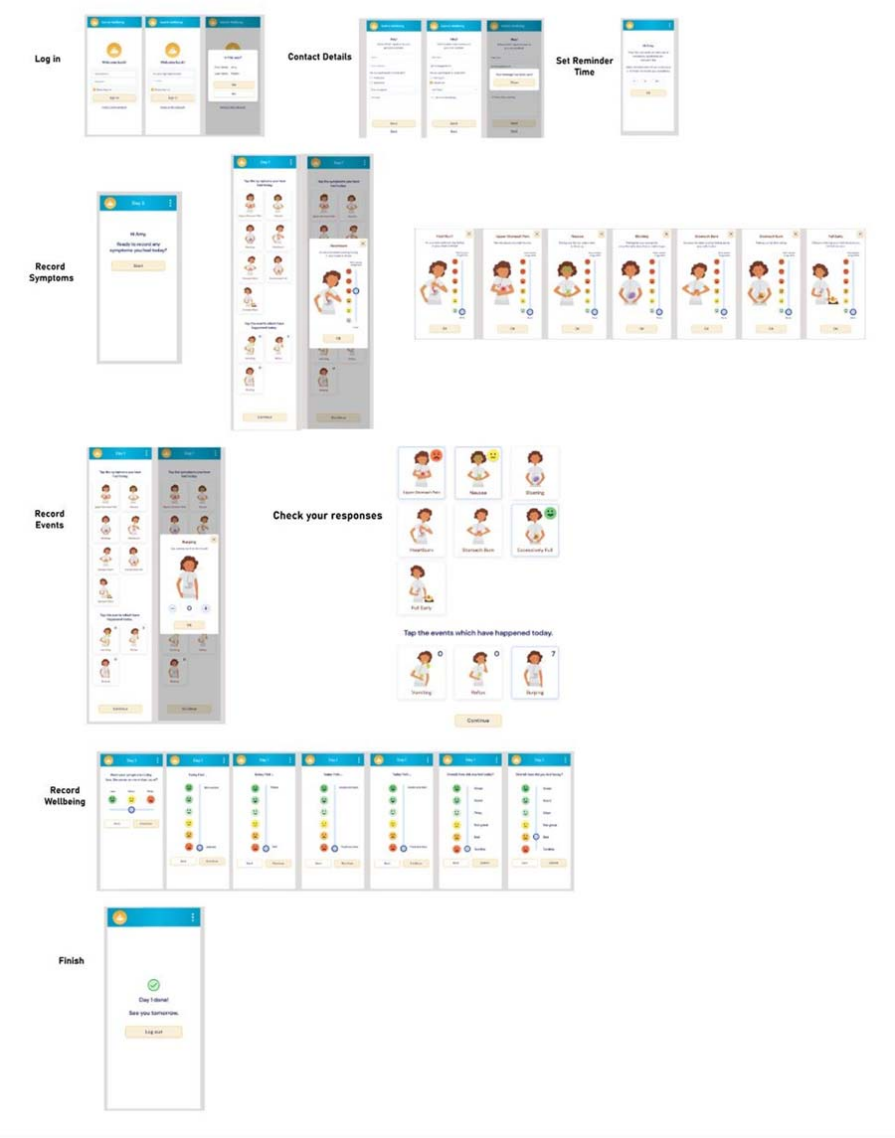


Figure 1. Daily Symptom and Wellbeing App

## 523 PATTERNS OF SECONDARY ESOPHAGEAL MOTILITY BY ENDOFLIP IN PEDIATRIC FEEDING DISORDER PATIENTS

*Gurleen Kahlon* ^*1*^, *Sara Karoli* ^*2*^, *Krisha Mansukhani* ^*1*^, *Lucia Mirea* ^*1*^, *Ricardo Medina‐Centeno* ^*1*^, *Dana Williams* ^*1*^

^*1*^ *Pediatric Gastroenterology*, *Phoenix Children's Hospital*, *Phoenix*, *AZ*; ^*2*^ *University of Arizona College of Medicine‐Phoenix*, *Phoenix*, *AZ*

**Background:** Pediatric Feeding Disorder (PFD) is defined as impaired oral intake that is not age‐appropriate and is associated with medical, nutritional, feeding skill, and/or psychosocial dysfunction. Inability to swallow due to esophageal motility dysfunction may drive PFD. Traditional esophageal motility studies are not feasible in young children. EndoFLIP is a minimally invasive diagnostic tool performed during esophagogastroduodenoscopy (EGD) under general anesthesia. There is no literature on EndoFLIP use for young patients with PFD.

**Objectives:** The primary aim is to describe and compare the patterns of esophageal contraction in children ages 6‐72 months with symptoms of PFD with and without dysphagia. The secondary aim is to gather data on pediatric EndoFLIP baseline parameters ‐Volume (V), Diameter (D), Distensibility Index (DI), and Pressures (P) of lower esophageal sphincter (LES) and upper esophageal sphincter (UES) as well as the presence of esophageal contractility‐ repetitive antegrade contraction (RAC) vs repetitive retrograde contraction (RRC).

**Methods:** This is a retrospective cohort observational single‐center study. Inclusion criteria included children aged 6‐72 months old with PFD with or without dysphagia. Patients with known motility disorders were excluded. Data was collected from January 2023 to January 2025. We performed EGD with biopsies under sedation (without using Precedex or Sevoflurane). EndoFLIP catheter was inserted (<5yo= 325 N/80 mm; >5yo= 322 N/160 mm) under direct visualization. The EndoFLIP balloon was filled to 10cc to find LES on the 3‐D geometric display on the screen. Volume increments of 5 to 10cc were added to obtain pressures of 18‐20 mm Hg to look for contractions and reach pressures below 50 mmHg. LES measurements were recorded for V, D, DI, and P. RRC, absence of contractions or LES DI <3 mm2/mmHg was considered abnormal. Balloon was deflated to 10 cc to safely find UES and the same steps were repeated to get similar UES measurements. UES DI <1 mm2/mmHg and decreasing DI with increased pressures was considered abnormal.

**Results:** A total of 150 patients were enrolled. 120 patients had dysphagia. Measurements of LES and UES were noted **(Figures 1 and 2).** 77% had normal esophageal contractility, 23.5% had inadequate esophageal contractions, 7.4% had restrictive LES and 30.9% had restrictive UES. The presence or absence of dysphagia did not alter the pattern of contractility in the statistical analysis (p >0.05).

**Conclusion:** EndoFLIP is an easy‐to‐perform, safe, and well‐tolerated procedure in young children. Our study describes the EndoFLIP protocol in children and highlights normative EndoFLIP values.This is the very first known study aiming to understand the patterns of secondary esophageal contractility using EndoFLIP in young patients with PFD.


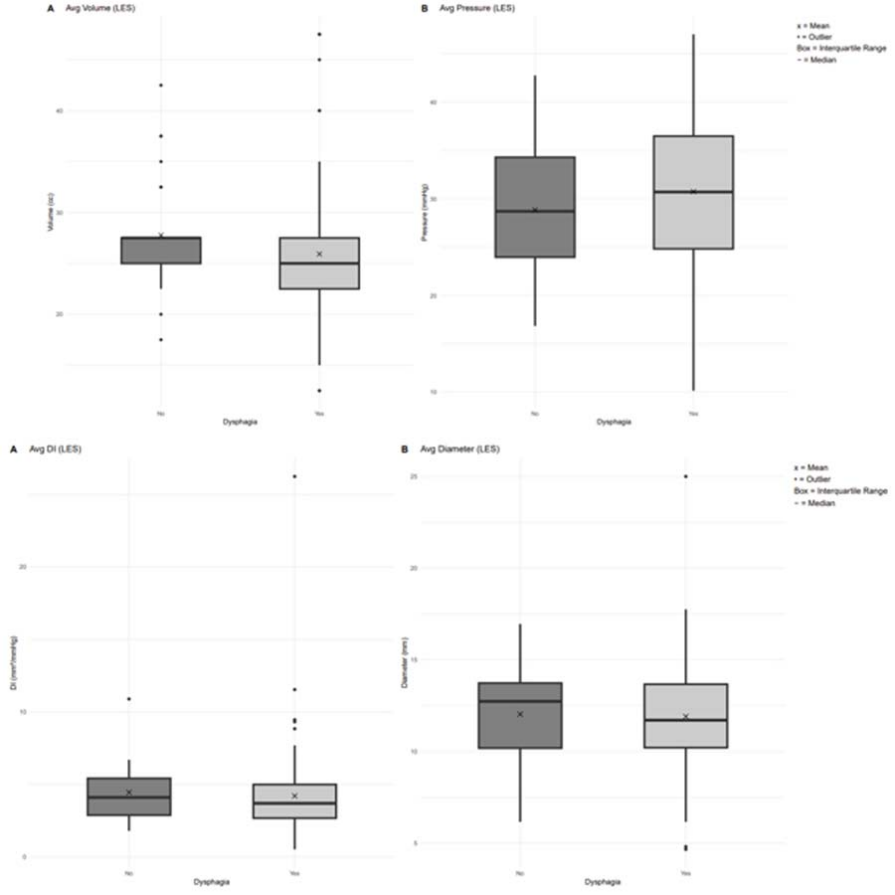


LES Average Measurements by Dysphagia Status in PFD Patients.


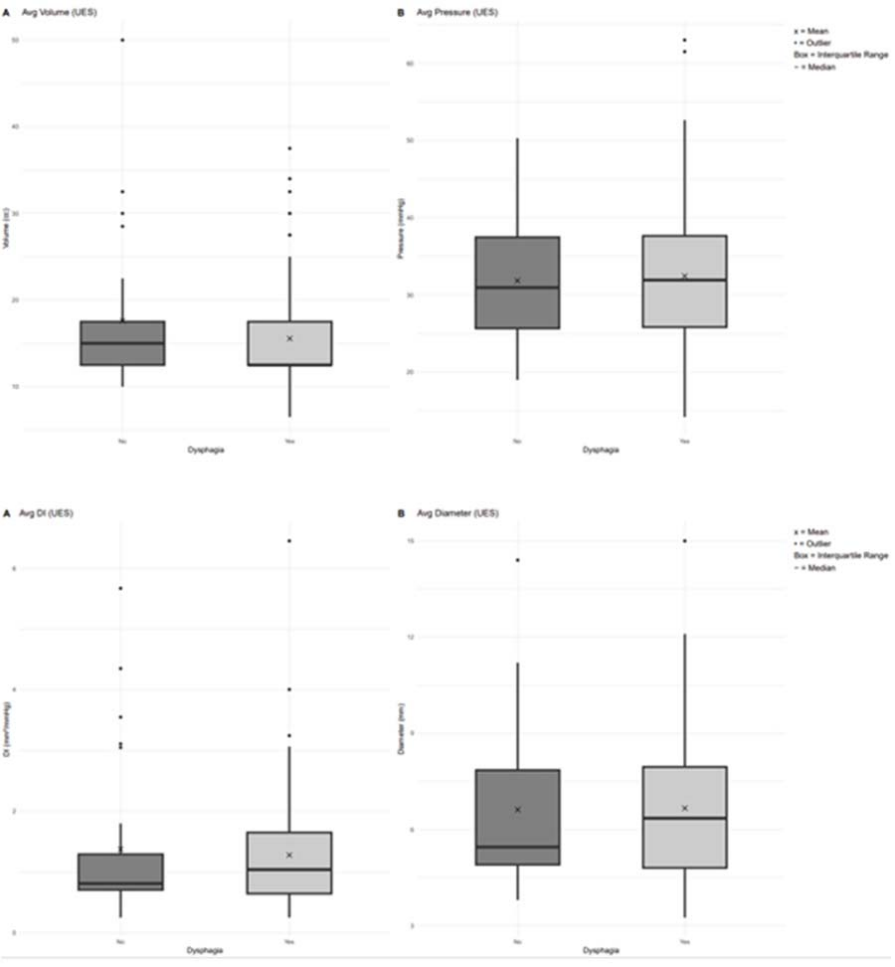


UES Average Measurements by Dysphagia Status in PFD Patients.

## 524 RADIO‐OPAQUE MARKER STUDY: RE‐DEFINING THE COLONIC TRANSIT TIME IN PEDIATRICS

*Prasanna Kapavarapu*, *Lexi Roshkovan*, *Carolyn Orians*, *Kimberly Konka*, *Alain Benitez*, *Hayat Mousa*

*Pediatric Gastroenterology*, *The Children's Hospital of Philadelphia*, *Philadelphia*, *PA*

**Background:** Radio‐opaque marker (ROM) study is used to measure colonic transit time (CTT) in adults with ingestion of a capsule with 24 radio‐opaque markers followed by obtaining an X‐Ray of the abdomen 120hrs after ingestion of the ROM capsule. However, in pediatrics the gut transit is faster compared to adults which requires the need to obtain X‐Ray sooner than 120hrs to characterize the type of constipation. In this study we aim to assess if day 3 X‐Ray (D#3 XR) is a better marker compared to day 5 X‐Ray (D#5 XR) to assess colonic transit time in pediatrics.

**Methods:** Retrospective review of patients who had ROM study were obtained during a 3‐year period from 2022 to 2025. At our institution there are two protocols (depending on ordering provider) for ROM study which includes either obtaining one XR on D#5 alone or 2 XR's on D#3 & D#5. The indications for ROM study are constipation with or without fecal incontinence, in children with a diagnosis of functional constipation with or without co‐morbidities including Hirschsprung disease, Anorectal malformation and Neurogenic bowel. The number of ROM were then counted on the XR based on the anatomic regions (right colon RC, left colon LC, Sigmoid S and Rectum R). Interpretation of ROM study was based on the number of retained markers: Normal (<5 ROM), Slow transit (majority ROM in RC), Intermediate transit (majority ROM in LC and S) and outlet dysfunction (majority ROM in R).

**Results (Table1):** 223 patients, ages ranging from 3 to 20 yrs, with 113 females and 110 males were identified. Out of 223 patients, 134 had one XR on D#5 and 89 had two XR's on D#3 & D#5.

Out of 89 patients who had two XR's on both D#3 & D#5,

28% (n=25) had normal CTT on both D#3 XR & D#5 XR, which means majority ROM were expelled on D#3 and the D#5 XR could have been avoided;

24% (n=21) had normal CTT on D#5 but not on D#3, which means by not doing D#3 XR these patients would have been labelled as normal CTT if they had only D#5 XR and hence these patients would not have had a characterization of the type of constipation without a D#3 XR;

18% (n=16) had outlet dysfunction on both D#3 & D#5 which means the D#3 XR had predicted the ROM retention to be the same as D#5 XR.

Overall, putting everything together 70% (n=62) of patients benefited from having a D#3 XR & D#5 XR instead of having a single D#5 XR.

**Conclusion:** In children to assess CTT with ROM study it may be best to always obtain a D#3 XR and to do a D#5 XR only if needed based on the results of D#3 XR. This approach will not only avoid unnecessary radiation exposure but also will also serve as a guide to robustly characterize the type of constipation in pediatrics.


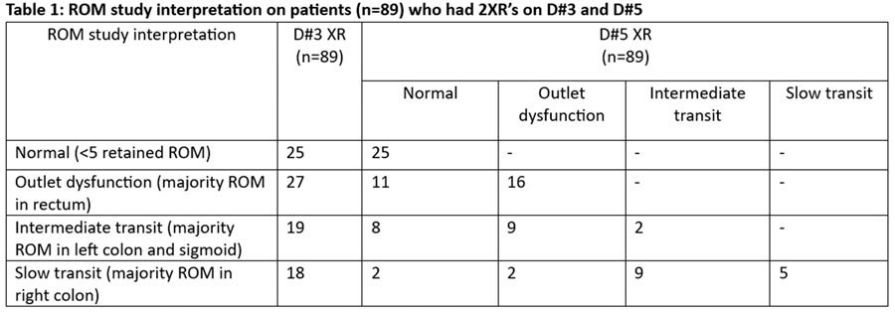


## 525 EFFECT OF BODY POSITION ON ESOPHAGEAL MOTILITY IN CHILDREN

*Karlo Kovacic* ^*1*^, *Mark Kern* ^*2*^, *Reza Shaker* ^*2*^

^*1*^ *Pediatric Gastroenterology*, *Medical College of Wisconsin*, *Milwaukee*, *WI*; ^*2*^ *Medical College of Wisconsin*, *Milwaukee*, *WI*

Children with suspected esophageal motility disorders are commonly assessed by high resolution esophageal manometry (HREM) with use of adult diagnostic algorithm summarized in the latest version of the Chicago Classification (CCv4.0). Per recommendation of North American Society for Pediatric Gastroenterology, Hepatology & Nutrition, HREM in children is frequently obtained while patients are sitting up. Given that CCv4.0 is based on adult data obtained while subjects were supine there is a concern that minor disorders of peristalsis are being misclassified during HREM in children. **Aim:** To compare the esophageal motility metrics of children with and without minor disorders of peristalsis (per CCv4.0) in supine and upright position. **Methods:** We studied 41 children (28 F, ages 7‐18) with dysphagia who underwent HREM. All studied children underwent placement of 8fr HREM catheter (Unisensor Inc, Attikon, Switzerland) either awake or under anesthesia. HREM studies were completed and captured after given adequate time (>10 min) needed for accommodation of upper esophageal sphincter. We studied ten 5 ml swallows supine and at least three 5 ml swallows while sitting upright of 0.45% NaCl in each child. We measured proximal contractile integral (PCI), transition zone gap (TZ), distal contractile integral (DCI) and 4 s IRP. Mann‐Whitney test was used to compare the results. **Results:** Median PCI and TZ were significantly affected with the change of the position (Fig 1.) (p=0.007 and p=0.045 respectively). While, DCI and 4 s IRP did not reach statistical significance (p=0.22 and 0.2 respectively). **Conclusions:** This is the first study that compares HREM metrics across different positions during HREM in children. We showed that PCI and TZ are significantly being affected by the position of the child during HREM. This is likely driven by pharyngeal drive of the bolus and gravity through the esophagus and has direct effect on HREM results, as such, it should be taken into account when assessing children. This stresses the need for universal pediatric HREM protocol driven by pediatric data.


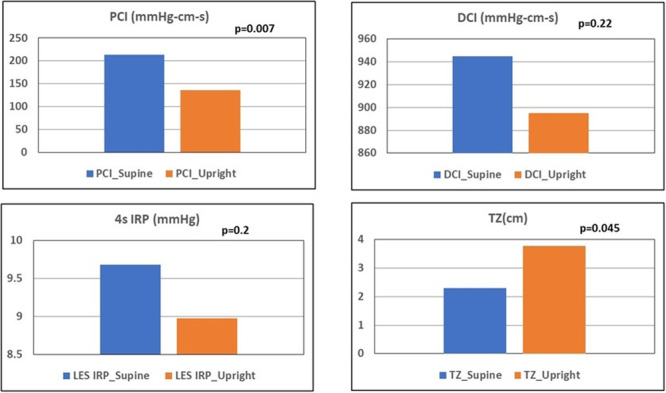


Figure 1

## 528 EFFICACY OF PROKINETIC TREATMENT IN PEDIATRIC INTESTINAL PSEUDO‐OBSTRUCTION

*Angela Lee* ^*1,2*^, *Eduardo Castillo Leon* ^*1*^, *Sujithra Velayuthan* ^*1*^, *Raul Sanchez* ^*1*^, *Neetu Bali Puri* ^*1*^, *Karla Vaz* ^*1*^, *Desale Yacob* ^*1*^, *Carlo Di Lorenzo* ^*1*^, *Peter Lu* ^*1*^

^*1*^ *Division of Gastroenterology, Hepatology and Nutrition, Department of Pediatrics*, *Nationwide Children's Hospital*, *Columbus*, *OH*; ^*2*^ *The Ohio State University College of Medicine*, *Columbus*, *OH*

**Background:** Although prokinetic medications are often used for children with pediatric intestinal pseudo‐obstruction (PIPO), our understanding of their efficacy remains limited. Our objective was therefore to evaluate the efficacy of prokinetic medications in children with PIPO.

**Methods:** We performed a retrospective review of children with PIPO seen at our institution in the past year. Diagnosis was made based on ESPGHAN criteria. We recorded demographics, medical history, and diagnostic testing. We identified the first instance each patient was treated with erythromycin, amoxicillin/clavulanic acid, prucalopride, or pyridostigmine and recorded symptoms before and 1 month after starting the medication.

**Results:** We included 39 patients (59% male). Median age of symptom onset was 1 year (IQR 0‐4) and median age at diagnosis was 4 years (IQR 1‐6). Nearly half (44%) had a history of prematurity and 26% a family history of a GI motility disorder. Comorbidities included short bowel syndrome (15%), intestinal malrotation (13%), and neurogenic bladder (13%). Four patients had megacystis‐microcolon‐intestinal hypoperistalsis syndrome (MMIHS). Of those with genetic testing, 23% had an abnormality associated with PIPO. Common presenting symptoms included constipation (47%), abdominal distention (41%), vomiting (38%), feeding intolerance (38%), and abdominal pain (28%). The majority (79%) had dilated loops of small bowel on prior imaging. Sixty‐seven percent underwent antroduodenal manometry, showing small bowel dysmotility in 88%. By their most recent follow up a median of 17 years after symptom onset, nearly all (92%) had at least 1 surgical procedure, including gastrostomy tube placement (84%), ileostomy (57%), jejunostomy tube placement (30%), colostomy (22%), and colonic resection (32%). Sixteen (41%) were receiving enteral nutrition, 9 (23%) required parenteral nutrition, and 17 (44%) received all nutrition by mouth. Seventeen patients were treated with erythromycin, 19 with amoxicillin‐clavulanic acid, 6 with prucalopride, and 13 with pyridostigmine. As shown in **Table 1**, changes in gastrointestinal symptoms before and after treatment with erythromycin and amoxicillin‐clavulanic acid were limited, with frequency of symptom resolution similar to development of new symptoms. Prucalopride led to a decrease in nausea and feeding intolerance. Pyridostigmine led to the most impressive response, with a decrease in nearly all gastrointestinal symptoms recorded. The percentage of patients with abdominal distention decreased from 92% to 38% after treatment with pyridostigmine (p=0.01).

**Conclusion:** In this cohort of children with PIPO, gastrointestinal symptoms improved with pyridostigmine and to a lesser degree with prucalopride. Response to prokinetic medications with a more targeted effect like erythromycin and amoxicillin‐clavulanic acid was less clear.


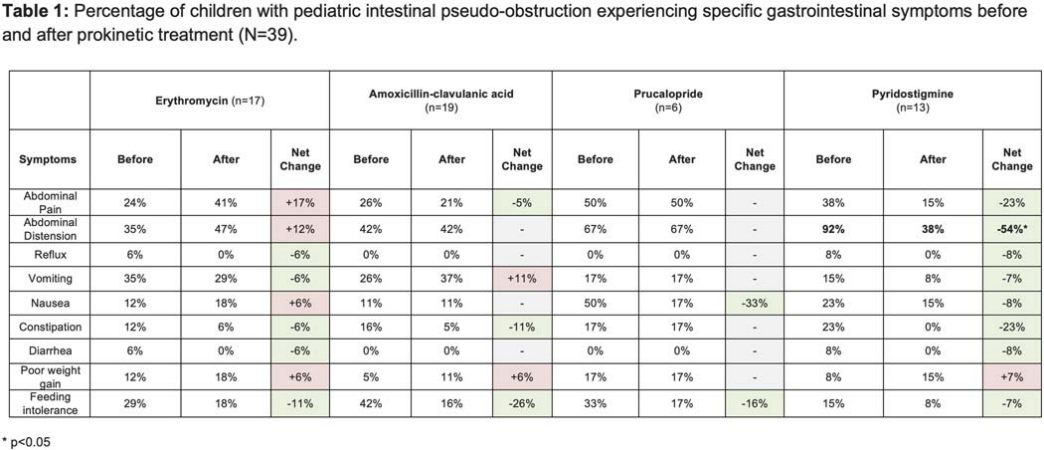


**Table 1:** Percentage of children with pediatric intestinal pseudo‐obstruction experiencing specific gastrointestinal symptoms before and after prokinetic treatment (N=39).

## 531 EFFECTS OF SPEECH‐LANGUAGE THERAPY INTERVENTION ON FEEDING SKILLS IN PRETERM NEWBORNS

*Andrea Medina‐Rodriguez* ^*1,2*^, *Rosa Mora‐Guerra* ^*1,2*^, *Carlos Velasco‐Benitez* ^*1,2*^

^*1*^ *Pediatrics*, *Universidad del Valle*, *Cali*, *Valle del Cauca*, *Colombia*; ^*2*^ *Pediatrics*, *Hospital Universitario del Valle “Evaristo García”*, *Cali*, *Valle del Cauca*, *Colombia*

**Introduction:** Preterm newborns (PTNB) present feeding difficulties due to the immaturity of the oromotor system. Speech therapy interventions aim to improve these skills through various techniques. This study aimed to demonstrate the effects of speech therapy intervention on the feeding skills of PTNB.

**Methods:** A descriptive longitudinal study was conducted in PTNB. An initial assessment of oromotor skills was performed. Then, a speech therapy intervention was implemented, including sensorimotor stimulation, non‐nutritive/nutritive sucking, and caregiver education. Outcomes were compared at the end to evaluate the effects of the intervention.

**Results:** A total of 96 PTNB were included; 52.1% were born between 32–34 weeks of gestation, 53.1% were male, and 64.6% had respiratory compromise. The average time from admission to speech therapy consultation was 7.3 ± 11.4 days. After the intervention, there was improvement in the number of sucks (p=0.0000), strong sucking (p=0.010), complete lip seal (p=0.000), adequate tongue coordination (p=0.000), mature sucking cycle (p=0.031), adequate oral sensitivity (p=0.011), and weight gain (p=0.0002). When comparing groups by gestational age (G1≤32 weeks; G2= 32–34 weeks; G3>34 weeks), the following was observed: G3 vs. G1 had greater weight gain (p=0.0000); G2 vs. G1 had earlier response to consultation (p=0.0001); and G3 vs. G1 had a higher number of sucks (p=0.0072). By the end of the treatment, 79.8% of the PTNB were feeding entirely by oral route.

**Conclusions:** Early speech therapy intervention in PTNB significantly improved oromotor skills and weight gain, allowing the majority to achieve full oral feeding. Gestational age influenced outcomes, with better results in newborns of higher gestational age. These findings highlight the importance of timely interdisciplinary care to optimize development and feeding in this vulnerable population.

## 532  A POTENTIAL SOLUTION FOR WATER BEAD INGESTION: NOVEL USE OF GASTROGRAFIN OSMOTICALLY SHRINKS WATER BEADS

*Naomi Patel*, *Cassidy Pham*, *John Schweitzer*

*Pediatrics*, *East Tennessee State University*, *Johnson City*, *TN*

Water beads are super‐absorbent polymers (SAPs) which are widely used as sensory toys. They have also led to thousands of emergency department visits due to their accidental ingestion by children. Accidental ingestion can lead to choking, but even more severe complications such as bowel obstruction, and even death. Due to their relatively small bowel lumen diameter, children are at higher risk of complications as the ingested water beads expand in their gastrointestinal tract. To address these complications, management often involves invasive procedures including endoscopic or surgical removal. However, non‐invasive interventions to reduce bead size in vivo may provide an alternative or complementary solution. This study investigates whether certain solutions can reduce bead size and weight over extended periods to inform potential clinical applications.

Water beads of six different colors from the brand “Leeche Waterbeads” were used and initially soaked in normal saline (NS) for 30 minutes to simulate gastrointestinal conditions. Subsequently, beads were immersed in four solutions: polyethylene glycol 3350 (MiraLAX), 2% Milk, diatrizoate contrast solution (Gastrografin), and normal saline. The diameter and weight of each water bead was recorded at 30‐minute intervals for up to 6 hours.

Beads soaked in MiraLAX had the largest expansion in diameter while those in gastrografin had the largest decrease in diameter (p<0.001). Beads soaked in normal saline and milk had similar trends, with a gradual increase in diameter. Gastrografin maintained an average bead weight of 0 grams throughout the entirety of the experiment. MiraLAX‐soaked beads had the largest increase in weight, expanding from 0 grams to 0.5 grams over the six hour period.

This study demonstrates that oral solutions variably affect water bead diameter and weight. Gastrografin had the most substantial effect, underscoring its potential clinical relevance and offering insight into strategies to prevent further expansion and complications such as intestinal obstruction. These findings suggest a promising avenue for non‐invasive management of water bead ingestion, potentially reducing the need for invasive procedures in selected cases, though future study is needed to evaluate long‐term effects and explore variability across different types of water beads.


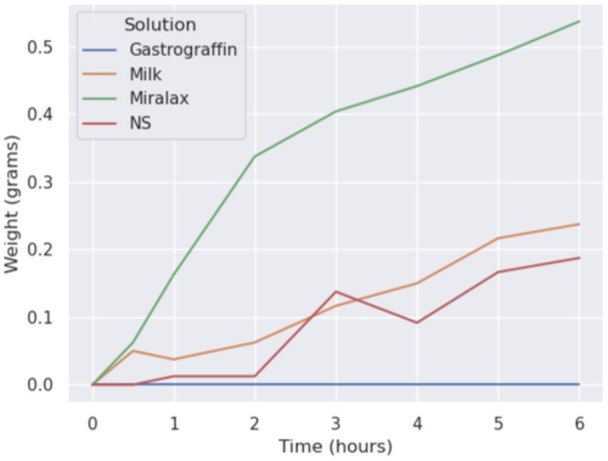



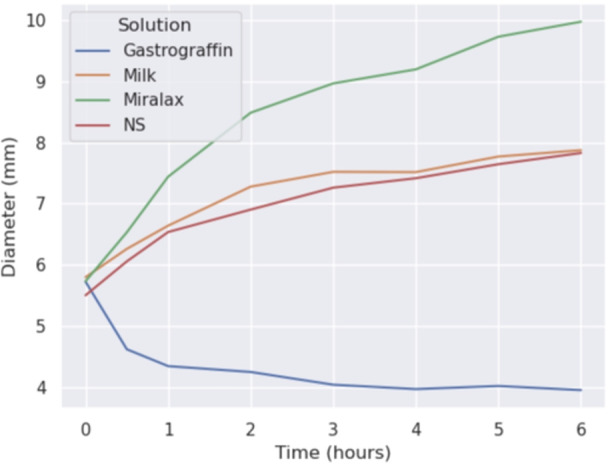


## 533 ESOPHAGEAL MOTILITY DISORDERS IN AUTOIMMUNE DISEASES: EVALUATION BY HIGH‐RESOLUTION MANOMETRY

*Fernanda Pérez Ortega*, *Erick Toro‐Monjaraz*, *Karen Ignorosa‐Arellano*, *Roberto Cervantes Bustamante*, *Jaime Ramirez‐Mayans*, *Dr. José Francisco Cadena León*, *Flora Zarate‐Mondragon*, *Ericka Montijo‐Barrios*, *Martha Martínez Soto*

*Gastroenterología y Nutricion Pediatrica*, *Instituto Nacional de Pediatria*, *Mexico City*, *CDMX*, *Mexico*

**Introdiction:** Scleroderma and dermatomyositis are autoimmune diseases that can manifest with dysphagia due to esophageal involvement. One of the cornerstone diagnostic tools for evaluating dysphagia in these patients is high‐resolution esophageal manometry (HRM), which provides a detailed assessment of esophageal motor function. Understanding the specific manometric patterns associated with each condition is essential for early diagnosis, therapeutic decision‐making, and monitoring disease progression.

**General Objective:** To analyze esophageal motility disorders using high‐resolution manometry in pediatric patients diagnosed with dermatomyositis or scleroderma.

Specific Objectives:

‐ To describe the demographic characteristics of the patients.

‐ To identify motility patterns through esophageal manometry.

‐ To compare manometric findings between the two groups.

**Study Design:** A retrospective analysis was conducted on patients treated at the National Institute of Pediatrics. Clinical and manometric data were collected. The following variables were included: age at diagnosis, sex, and manometric parameters such as integrated relaxation pressure (IRP), distal contractile integral (DCI), and lower esophageal sphincter (LES) length. Quantitative variables between groups were compared using the Mann‐Whitney U test, as appropriate for the sample size.

**Results:** Nineteen patients with dermatomyositis and four with scleroderma were analyzed. Patients with scleroderma had a significantly lower mean DCI of 98.65 mmHg s cm (range: 1– 260.6), compared to those with dermatomyositis, who had a mean DCI of 2226.33 mmHg s cm The mean IRP in scleroderma was 4.65 mmHg (range: 2.6–6.6), compared to 13.47 mmHg in dermatomyositis, with no statistically significant difference. The mean resting LES pressure was 38.85 mmHg (range: 40.3–170) in scleroderma and 29.6 mmHg (range: 24–660) in dermatomyositis. Esophageal aperistalsis was observed in patients with scleroderma.

**Conclusions:** High‐resolution manometry revealed distinct esophageal motility patterns in dermatomyositis and scleroderma. Patients with scleroderma showed severe hypocontractility, markedly reduced DCI, and esophageal aperistalsis, while those with dermatomyositis retained better esophageal motor function. These findings may be useful in the diagnosis and follow‐up of pediatric patients with autoimmune diseases.

## 534 THE RELATIONSHIP BETWEEN FUNCTIONAL LUMINAL IMAGING PROBE (FLIP) AND HIGH RESOLUTION ESOPHAGEAL MANOMETRY MEASUREMENTS IN CHILDREN ‐ CAN FLIP REPLACE HIGH RESOLUTION ESOPHAGEAL MANOMETRY?

*Tal Berger*, *Samuel Nurko*, *Elise Delaney*, *Chalmers Christopher*, *Grace Nemec*, *Evrim Ozcam*, *Rachel Rosen*

*Gastroenterology*, *Boston Children's Hospital*, *Boston*, *MA*

**Background:** As part of the diagnostic work up for children with dysphagia, esophageal manometry and functional luminal imaging probes (FLIP) panometry are jointly performed to assess for disorders of primary and secondary peristalsis. Additional pediatric studies to determine the degree of concordance between FLIP and esophageal high‐resolution impedance manometry (HRIM) in patients with well‐defined Chicago classification are needed.

**Methods:** We retrospectively reviewed the esophageal high‐resolution impedance manometry (HRIM) records and FLIP 2.0 tracings for 74 patients. In each FLIP tracing we obtained esophagogastric junction (EGJ) distensibility (DI), presence of RAC's and their characteristics. Each esophageal manometry was measured for integrated relaxation pressure (IRP) and distal contractile integral (DCI). The patients were classified according to the Chicago classification v4.0 and the HRIM measurements into 5 groups ‐ normal motility, ineffective peristalsis, absent peristalsis, achalasia and esophagogastric junction outflow obstruction (EGJOO). These groups were then compared to the FLIP tracings which were categorized as normal repetitive antegrade contractions (RAC), abnormal RACs (shape or frequency), and/or low EGJ distensibility (DI>2.8).

**Results:** The mean age of patients was 12.9 ± 6.4 years. The HRIM was normal in 23/74 patients (31%), 15/74 (20.2%) had achalasia, 11/74 (14.9%) had ineffective peristalsis, 14/74 (18.9%) had absent peristalsis and 11/74 (14.9%) had EGJOO. The table shows the concordance between HIRM and FLIP. There was a significant difference in the mean DCI in patients with abnormal or absent RACs (557 + 110 mmHg‐s‐cm) and patients with normal RACs (1089 + 168 mmHg‐s‐cm; p<0.012). There was a strong correlation between IRP and DI (r=0.449; p < 0.001). We then determined the relationship between primary peristalsis by HRIM and secondary peristalsis by FLIP (achalasia patients excluded); in the 23 patients with normal peristalsis by HIRM, 65% had normal RACs and, in the 25 patients with abnormal peristalsis,13 (52%) had an abnormal FLIP. We then determined the relationship between EGJ function by HRIM and FLIP; of the 48 patients with a normal IRP, 5 had an abnormal DI and, of the 11 patients with EJGOO by HRIM, 28% had an abnormal DI by FLIP.

**Conclusions:** FLIP diagnoses differed from the Chicago classification diagnoses in a significant proportion of children. Our data confirms that these tools are complementary and should not be used in isolation except for patients with untreated achalasia.

Table 1. The concordance between FLIP and HRIM findings in patients with well‐defined Chicago classification diagnoses (all patients)


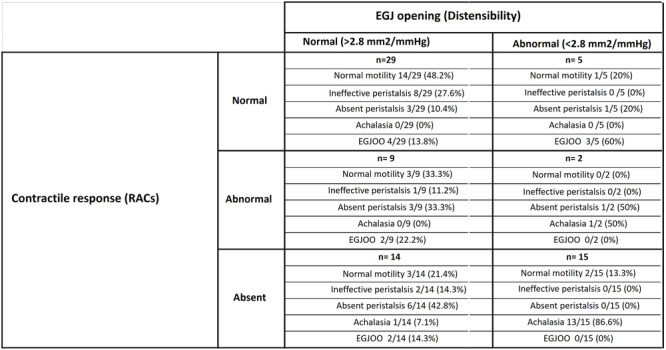


## 535  A VIRTUAL REALITY‐BASED MINDFULNESS INTERVENTION IMPROVES ABDOMINAL PAIN AND NAUSEA IN CHILDREN AND ADOLESCENTS WITH FUNCTIONAL ABDOMINAL PAIN DISORDERS

*Neha Santucci* ^*1,2*^, *Priyanshi Shah* ^*1*^, *Rashmi Sahay* ^*1*^, *Megan Miller* ^*1*^, *Jen Hardy* ^*1*^, *Linda Nguyen* ^*3*^, *Bonney Reed* ^*4*^, *Christopher King* ^*1,2*^

^*1*^ *Gastroenterology*, *Cincinnati Children's Hospital Medical Center*, *Cincinnati*, *OH*; ^*2*^ *Pediatrics*, *University of Cincinnati College of Medicine*, *Cincinnati*, *OH*; ^*3*^ *Gastroenterology*, *Stanford Medicine*, *Stanford*, *CA*; ^*4*^ *Emory University School of Medicine*, *Atlanta*, *GA*

**Introduction:** Pediatric disorders of gut‐brain interaction (DGBI) are associated with increased psychological comorbidities, disability, school absenteeism, and healthcare costs. Evidence suggests that non‐pharmacological interventions, including relaxation and guided mindfulness‐based therapies, can reduce the experience of pain and emotional distress in adults and children with chronic pain conditions. To extend this line of research, we aimed to assess the feasibility, acceptability, and efficacy of delivering a brief mindfulness intervention in children and young adults with functional abdominal pain disorders (FAPD) using virtual reality‐guided mindfulness (VR‐M). We also explored the impact of VR‐M on subjective and objective outcomes associated with several biopsychological domains previously associated with pain and relaxation.

**Methods:** Participants (8‐ 21 years) with FAPD (based on Rome 4 criteria) were recruited from the CCHMC DGBI clinic. Patients with organic GI disorders, conversion disorders, severe developmental delays, visual impairment, and seizure disorders were excluded. Demographics and medical history were obtained. After completing a series of patient‐reported outcomes, the study staff provided information to the participants about the Oculus VR gear headset. The VR‐M session lasted 10 minutes and participants used the “Mindful Aurora” guided mindfulness‐based application. Subjects rated their abdominal pain intensity, unpleasantness, nausea, nausea botherness and stress on a visual analog scale (VAS) before, immediately, and up to 30 minutes after the VR‐M session.

**Results:** Of 28 subjects (*M*_Age_ 14.7 ± 2.6, 60.7% female, 71.4% Caucasians), the most common diagnosis was functional dyspepsia (46.4%). Compared to baseline, ratings of abdominal pain intensity (p = 0.02), pain unpleasantness (p = 0.002), nausea (p = 0.002), and stress (p = 0.003) were lower at all timepoints after VR‐M session completion (Figure 1). About half of the sample found it feasible (46.4%) and felt more relaxed (53.6%). A small percentage of participants reported VR‐M as an ideal intervention for improving their pain (17.9%) and interest in using VR mindfulness again using a home‐based approach (35.7%).

**Conclusion:** This study demonstrates that a single VR‐M session is feasible and acceptable, with marked reductions in abdominal pain intensity, unpleasantness, nausea, and stress in children and young adults with FAPD. This is promising intervention to improve compliance with behavioral techniques for adolescents who might otherwise not engage in non‐pharmacologic therapies. While over half of participants reported relaxation, variability in individual responses highlights the need for personalized therapeutic approaches.


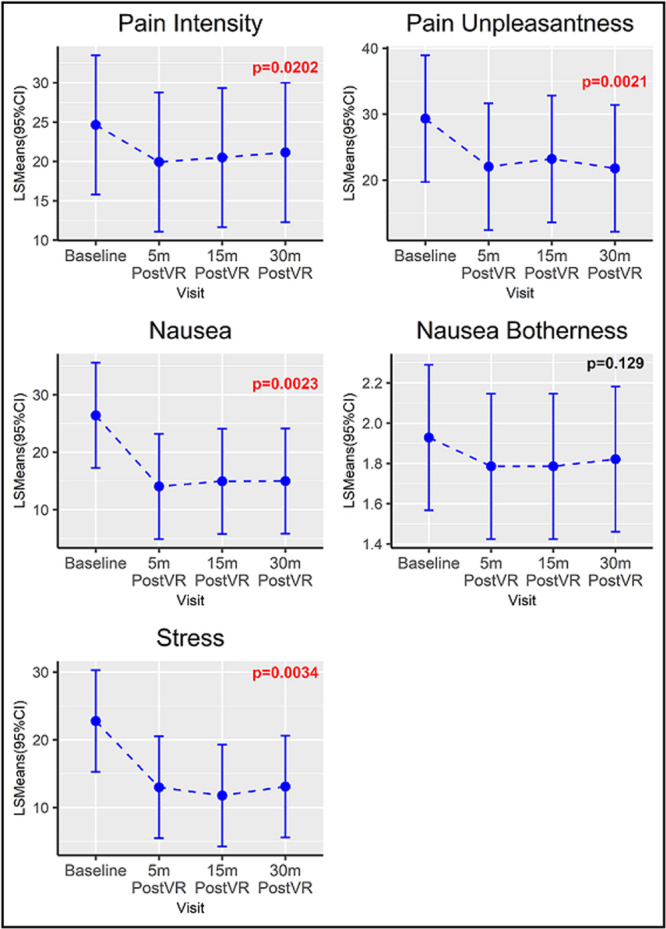


**Changes in GI symptoms post‐VR guided mindfulness**

## 537 INCREASED DISABILITY, POST‐TRAUMATIC STRESS DISORDER, SLEEP DISTURBANCE, AND RESTRICTED EATING PATTERNS POST‐PANDEMIC IN CHILDREN AND ADOLESCENTS WITH DISORDERS OF GUT‐BRAIN INTERACTION

*Mila Colizza* ^*2*^, *Jen Hardy* ^*1*^, *Rashmi Sahay* ^*3*^, *Neha Santucci* ^*1,2*^

^*1*^ *Gastroenterology*, *Cincinnati Children's Hospital Medical Center*, *Cincinnati*, *OH*; ^*2*^ *University of Cincinnati College of Medicine*, *Cincinnati*, *OH*; ^*3*^ *Biostatistics*, *Cincinnati Children's Hospital Medical Center*, *Cincinnati*, *OH*

**Background:** The COVID‐19 pandemic has been associated with an increase in disorders of gut‐brain interaction (DGBI) and DGBI disease severity. There has been an increase in GI symptoms, virtual schooling patterns, and extra‐intestinal symptoms including long COVID post‐pandemic. However, the impact of the pandemic on DGBI‐associated comorbidities is not well‐established. We aimed to characterize clinical outcomes and comorbidities in DGBI patients in the years before, during, and after the pandemic.

**Methods:** We reviewed the charts of patients ages 6‐21 y who met Rome 4 criteria for DGBI and were seen in the multidisciplinary DGBI clinic between the years of 2017 (year of inception of the clinic) and 2022. Patients with organic GI disorders and systemic illnesses were excluded. Data was collected on demographics, medical history, nutritional disturbances, and responses to the following questionnaires: Abdominal Pain Index (API), Functional Disability Inventory (FDI), and Nausea Severity Scale (NSS). Responses were compared between DGBI patients seen pre‐pandemic (before 2020), during the pandemic (2020‐2021), and post‐pandemic (after 2021). Since the questionnaires were only implemented in 2020, the clinical responses were compared during and post‐pandemic.

**Results:** Of 331 total DGBI patients, 88.2% were age 12‐21 y, 79.2% were female, and 89.1% were Caucasian. The most common symptoms were abdominal pain (91.8%), nausea (72.8%), and constipation (41.4%). The most common types of DGBI were functional dyspepsia (58%) and irritable bowel syndrome (57.4%).

Of the total cohort, 52 were seen before 2020, 173 between 2020 and 2021, and 106 after 2021. In the <2020 cohort, the mean age was 14 y ± 3.19, 73.1% were female, and 94.2% were Caucasian. In the 2020‐2021 cohort, the mean age was 15.98 y ± 2.34, 82.7% were female, and 89.6% were Caucasian. In the >2021 cohort, the mean age was 15.92 y ± 2.47, 76.4% were female, and 85.9% were Caucasian.

The most common comorbidity across all three cohorts was anxiety (51.9%, 65.7%, and 57.6%) followed by orthostatic intolerance in the <2020 cohort (26.9%), depression in the 2020‐2021 cohort (37.2%), and sleep disturbance in the >2021 cohort (42.5%, Figure 1).

In comparing measures of disease severity during and after the pandemic, FDI scores were found to increase significantly from 17.7 to 22.4 (p = 0.0096). API and NSS scores did not statistically differ during and after the pandemic.

Over the three time periods, the prevalence of restricted eating was found to increase significantly, from 35.4% pre‐pandemic to 49.1% in the pandemic years to 56.7% post‐pandemic (p = 0.0499, Figure 1). The proportion of DGBI patients with post‐traumatic stress disorder (PTSD) also increased significantly over these three periods, from 1.9% to 4.7% to 11.3% (p = 0.0311). Sleep disturbance had a strong trend for increase from 23.1% to 34.7% to 42.5% (p=0.055). Finally, a significant variation in the prevalence of POTS was identified, affecting 5.9% and 5.7% of DGBI patients pre‐ and post‐pandemic compared to 14.5% of patients during the pandemic (p = 0.0356).

**Conclusion:** Our data suggests that DGBI patients exhibit increased restricted eating, PTSD, and sleep disturbance post‐pandemic. While GI symptom severity itself did not change, worse functional disability was noted post‐pandemic, which could likely be from increased comorbidities, making DGBI harder to manage. This may be reflective of an increased prevalence of these comorbid conditions in the general population as well. It is prudent for clinical providers to be aware of this overlap to accurately diagnose and treat these conditions as they may have a bearing on clinical outcomes.


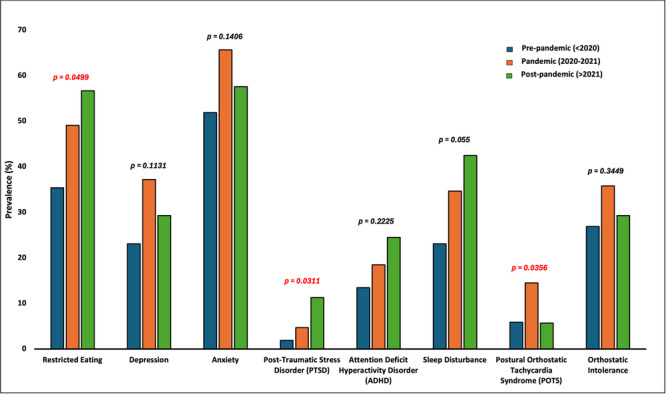


Figure 1. Change in GI comorbidities before, during, and after the pandemic.

## 538 VALIDATION OF CLAIMS‐BASED ALGORITHMS FOR PEDIATRIC ABDOMINAL PAIN‐ASSOCIATED DISORDERS OF GUT‐BRAIN INTERACTION

*Ayesha Sujan* ^*1*^, *Ryan Ma* ^*1*^, *AnnMing Yeh* ^*2*^, *Brian Bateman* ^*1*^, *Jennifer Rabbitts* ^*1*^

^*1*^ *Department of Anesthesiology, Perioperative and Pain Medicine*, *Stanford University School of Medicine*, *Stanford*, *CA*; ^*2*^ *Department of Pediatrics*, *Stanford University School of Medicine*, *Palo Alto*, *CA*

Introduction: Abdominal pain‐associated disorders of gut‐brain interaction (AP‐DGBIs) are a major source of disability for youth and have been linked to functional impairments, psychological distress, and increased healthcare utilization. However, evidence‐based protocols for pediatric AP‐DGBI treatment are lacking. Therefore, research is critically needed to understand the treatment needs of youth with AP‐DGBI and establish safe, evidence‐based treatments for pediatric AP‐DGBI. Claims‐based databases provide a valuable resource for investigating health‐related questions, including those concerning pediatric AP‐DGBI, by providing comprehensive information on routine and specialized care for a large proportion of the population. The first step to utilizing claims data for research purposes is to develop valid algorithms to identify the health conditions of interests. Published research to date has not reported on the validity of claims‐based algorithms for AP‐DGBI.

Objective: The objective of this study was to develop and validate claims‐based algorithms for identifying pediatric AP‐DGBI diagnoses, specifically irritable bowel syndrome (IBS), functional dyspepsia (FD), and abdominal migraines (AM).

**Methods:** We identified patients between the ages of 4‐ and 21‐years receiving care at Stanford University School of Medicine with >2 International Classification of Disease (ICD), version‐10 codes for IBS, FD, or AM given at least 90 days apart. We then randomly selected 50 cases with each condition for chart review. Two reviewers coded each chart to determine if cases were correctly identified and then met to resolve discrepancies. A third reviewer arbitrated any discrepancies between the first two reviewers. To quantify the validity of the claims‐based algorithms, we calculated positive predictive values and corresponding 95% confidence intervals for each condition using the chart reviews as the gold standard.

**Results:** 86% (95% confidence interval [CI]:76%‐96%) of the IBS cases identified with the claims‐based algorithm were confirmed by chart review. 74% (95% CI: 62%‐86%) of the FD cases identified with the claims‐based algorithm were confirmed by chart review. 90% (95% CI:82%‐98%) of the AM cases identified with the claims‐based algorithm were confirmed by chart review.

Discussion: Results provide strong support for the validity of claims‐based algorithms for IBS and AM. The FD algorithm was approaching the cutoff for what is generally considered valid (i.e., a positive predictive value of > 80%). Future directions include testing the validity of other claims‐based algorithms for FD, as well as using validated AP‐DGBI claims‐based algorithms to characterize youth with AP‐DGBI and evaluate the safety and effectiveness of currently utilized AP‐DGBI treatments in youth.

## 539 INCREASED RISK OF INFLAMMATORY BOWEL DISEASE IN PEDIATRIC PATIENTS WITH HIRSCHSPRUNG'S DISEASE: A PROPENSITY SCORE‐MATCHED ANALYSIS

*Aravind Thavamani* ^*1,2*^, *Senthilkumar Sankararaman* ^*3*^, *Thomas Sferra* ^*1,4*^, *Sujithra velayuthan* ^*3*^

^*1*^ *Pediatrics*, *University Hospitals Rainbow Babies & Children's Hospital*, *Cleveland*, *OH*; ^*2*^ *University Hospitals Health System*, *Cleveland*, *OH*; ^*3*^ *Gastroenterology, Hepatology and Nutrition*, *Cleveland Clinic Children's Hospital*, *Cleveland*, *OH*; ^*4*^ *Case Western Reserve University*, *Cleveland*, *OH*

**Background:** Prior case studies have reported inflammatory bowel disease (IBD) in patients with Hirschsprung's disease (HD). Various etiologies such as dysmotility, genetic mutations and dysbiosis have been postulated for this association. However, the epidemiology of this association is not well studied. We hypothesized that the prevalence of IBD in HD is higher compared to the control population.

**Methods:** We analyzed data from TrinetX database, a global federated health research network providing access to electronic medical across large healthcare organizations (HCOs). We analyzed all pediatric patients less than 18 years old with a diagnosis of HD using the International Classification of Diseases (ICD) diagnostic codes. Patients with chronic idiopathic constipation (CIC) were chosen as control population given the commonality of symptoms (constipation). Any patient with preexisting diagnosis of IBD before HD or CIC diagnosis were excluded. Propensity score matching (PSM) was done for various demographic factors such as age, gender and race. The outcome variable was the development of IBD (Crohn's disease or Ulcerative colitis).

**Results:** We analyzed a total of 8,134 patients with HD and approximately 128,500 patients with CIC. Patients with HD were younger, belonged to African American race and more often males. The prevalence of IBD was 2.2% (186 patients) in the HD group and 0.6% in the CIC group. After propensity score matching there were 7,337 patients in each group, with comparable demographic characteristics other than gender (Table 1). Mean duration of follow up were 1520 days and 992 days in the HD and CIC group respectively. After propensity score matching the odds of developing IBD was 5.48 (3.474, 8.649) times higher in the HD group compared to the CIC group, P<0.001. The absolute risk difference was 0.013 (95% CI: 0.010 to 0.016, P<0.001) between the HD and CIC group.

**Conclusion:** Our study elucidated that pediatric patients with HD are at increased risk of developing IBD compared to CIC. Further prospective studies are needed to better understand the pathophysiology behind this increased association.


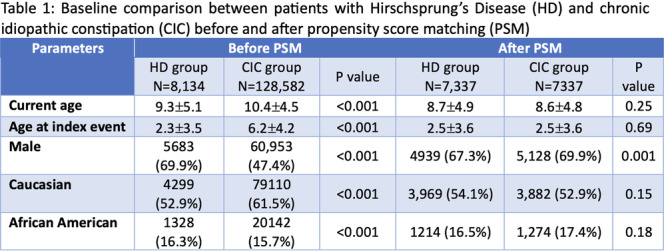


## 542 THE USE OF LUBIPROSTONE FOR PEDIATRIC FUNCTIONAL CONSTIPATION: A SYSTEMATIC REVIEW AND SINGLE‐ARM META‐ANALYSIS

*Nágila Alves Lima* ^*2*^, *Jennifer Warner* ^*1,5*^, *Beatriz Ximenes Mendes* ^*2*^, *David Abraham Batista da Hora* ^*4*^, *Francisco Willamy Pedrosa Alves Filho* ^*3*^, *John Rosen* ^*5,1*^

^*1*^ *Pediatrics*, *University of Arkansas for Medical Sciences*, *Little Rock*, *AR*; ^*2*^ *Centro Universitario Christus*, *Fortaleza*, *CE*, *Brazil*; ^*3*^ *Universidade Federal do Ceara*, *Sobral*, *CE*, *Brazil*; ^*4*^ *Universidade Federal do Amazonas*, *Manaus*, *AM*, *Brazil*; ^*5*^ *Arkansas Children's Hospital*, *Little Rock*, *AR*

**Background:** Pediatric Functional Constipation (PFC) is common, affecting 30% of the pediatric population and accounting for approximately 25% of the demand for care by pediatric gastroenterologists. Only 70% of children with PFC achieve long‐term improvement with the behavioral, dietary, and pharmacological measures proposed by guidelines. Therefore, new therapies are needed. Lubiprostone is a type 2 chloride channel activator that increases intestinal fluid secretion and decreases stool transit time, but has unclear evidence for its effect on PFC.

**Objective:** We aimed to evaluate the effectiveness of lubiprostone in children with functional constipation.

**Methods:** We systematically searched PubMed, Embase, and Cochrane. Outcomes were the prevalence of spontaneous bowel movement (SBM), headache, nausea, and abdominal pain. Because of few reports and a lack of comparison groups, a single‐arm meta‐analysis was conducted in R software (v3.6.2), using a random‐effects model with 95% confidence intervals and the generic inverse variance method. Heterogeneity was assessed with I2 statistics. This systematic review and meta‐analysis were conducted using the Cochrane Handbook for Systematic Reviews of Interventions and reported following the Preferred Reporting Items for Systematic Reviews and Meta‐Analyses (PRISMA) guidelines.

**Results:** 711 participants from 2 randomized controlled trials (RTCs) and 2 non‐randomized cohorts were included. The dose of lubiprostone used in patients weighing less than 50 kg ranged from 8 to 12 mcg/day, while patients weighing more than 50 kg received doses ranging from 24 to 48 mcg/day. The median age was 10.99 years, 52.4% were female, and the median body weight of participants was 45.29 kg. The baseline SBM was 15.34+‐4.79 frequency. Follow‐up time ranged from 4 to 24 weeks in the included studies. The prevalence of SBM was 23.8% (95% CI [7.18; 61.93], I2=96.2%, p<0.0001). The prevalence of headache was 8.4% (95% CI [4.57; 11.09], I2= 52.5%, p=0.09). The use of lubiprostone was associated with nausea in 9.1% (95% CI [2.72; 16.16], I2=72.2%, p=0.02) and abdominal pain in 10.5% (95% CI [0.38; 19.94], I2=91.4%, p<0.0001) of patients.

**Conclusion:** In this systematic review and meta‐analysis, the use of lubiprostone may be associated with a modest improvement in SBM in PFC. Lubiprostone was statistically associated with nausea and abdominal pain, but it was not associated with headache in the pediatric population studied. Due to the high heterogeneity, more randomized studies are needed to ensure the effectiveness and safety of this medication in the pediatric population.

**References:**

Benninga MA, Hussain SZ, Sood MR, Nurko S, Hyman P, et al. Lubiprostone for Pediatric Functional Constipation: Randomized, Controlled, Double‐Blind Study With Long‐term Extension. Clin Gastroenterol Hepatol. 2022 Mar;20(3):602‐610.e5.

Elkaragy ES, Shamseya MM, Metwally RH, Mansour ER, Lashen SA. Efficacy of lubiprostone for functional constipation treatment in adolescents and children: Randomized controlled trial. J Pediatr Gastroenterol Nutr. 2024 Apr;78(4):800‐809.

Fedele F, Fioretti MT, Scarpato E, Martinelli M, Strisciuglio C, et al. The ten "hard" questions in pediatric functional constipation. Ital J Pediatr. 2024 Apr 8;50(1):64.

Hussain SZ, Labrum B, Mareya S, Stripling S, Clifford R. Safety of Lubiprostone in Pediatric Patients With Functional Constipation: A Nonrandomized, Open‐Label Trial. J Pediatr Gastroenterol Nutr. 2021 Nov 1;73(5):572‐578.

Hyman PE, Di Lorenzo C, Prestridge LL, Youssef NN, Ueno R. Lubiprostone for the treatment of functional constipation in children. J Pediatr Gastroenterol Nutr. 2014 Mar;58(3):283‐91.

Oak AA, Chu T, Yottasan P, Chhetri PD, Zhu J, et al. Lubiprostone is non‐selective activator of cAMP‐gated ion channels and Clc‐2 has a minor role in its prosecretory effect in intestinal epithelial cells. Mol Pharmacol. 2022 Jun 9;102(2):106–15.


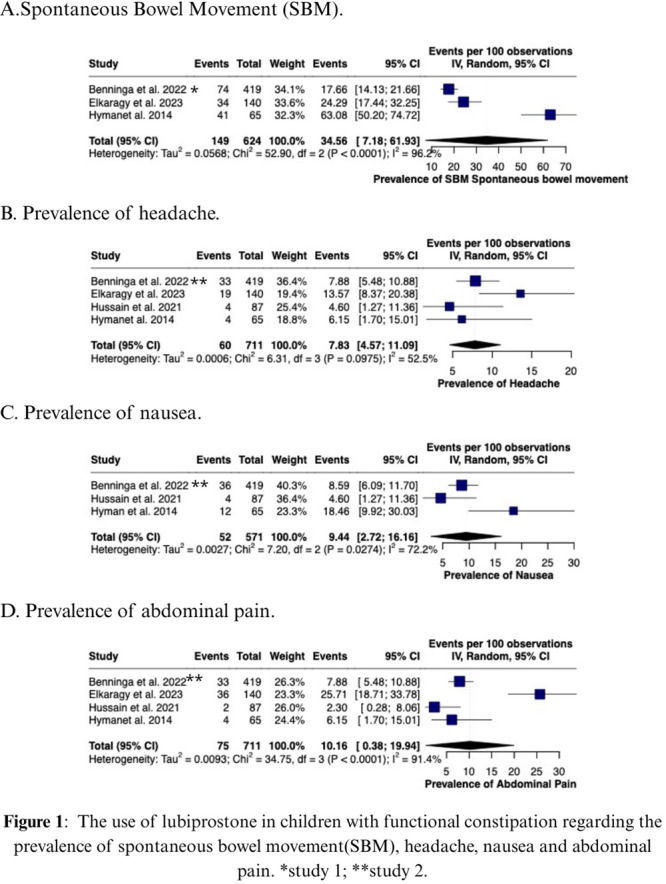


## 543 CURRENT PRACTICES AND BARRIERS IN TRANSITIONING ADOLESCENTS AND YOUNG ADULTS WITH NEUROGASTROENTEROLOGY AND MOTILITY DISORDERS: RESULTS OF A SURVEY STUDY

*Sharon Wolfson* ^*1,3*^, *Dhiren Patel* ^*2*^, *Lusine Ambartsumyan* ^*4*^, *Neha Santucci* ^*5*^, *Julie Khlevner* ^*6*^, *Miguel Saps* ^*7*^

^*1*^ *Department of Gastroenterology, Hepatology and Nutrition*, *The Children's Hospital of Philadelphia*, *Philadelphia*, *PA*; ^*2*^ *SSM Health Cardinal Glennon Children's Hospital*, *St. Louis*, *MO*; ^*3*^ *University of Pennsylvania Perelman School of Medicine*, *Philadelphia*, *PA*; ^*4*^ *Seattle Children's Hospital*, *Seattle*, *WA*; ^*5*^ *University of Cincinnati College of Medicine*, *Cincinnati*, *OH*; ^*6*^ *Columbia University Vagelos College of Physicians and Surgeons*, *New York*, *NY*; ^*7*^ *University of Miami Miller School of Medicine*, *Miami*, *FL*

**Introduction:** Pediatric Neurogastroenterology and Motility disorders (NGM) are chronic and complex, often persisting into adulthood. Transition from pediatric to adult care can be challenging not only for adolescents and young adults (AYA), but also their parents and caretakers, and their health care team. Delayed or poorly coordinated transition can lead to unpreparedness and worse outcomes. This study aimed to assess existing transition of care practices, barriers to successful transition, and institutional resources allocated to transition of care across pediatric centers in North America.

**Methods:** A cross‐sectional survey consisting of 29 questions was distributed to the members of the North American Society of Pediatric Gastroenterology, Hepatology and Nutrition listserv. The survey collected data on transition of care practices including institutional structure, transition age, adult provider availability, transition assessment methods, and perceived needs for successful transition.

**Results:** Fifty‐five responses were received from 40 distinct institutions, with 54 attending and 1 fellow physician completing the survey. Respondents were predominantly pediatric NGM experts (62%, n=34), followed by general gastroenterologists (GI) (25%, n=14). Institutional types included adult and pediatric medical centers (40%, n=22), pediatric‐only centers (40%, n=22), and pediatric centers embedded within adult facilities (20%, n=11). Institutions were categorized as: Academic (72.5%, n=29), Community (12.5%, n=5), Private practice (7.5%, n=3) and Other/Mixed model (7.5%, n=3).

The most common age to begin transition was 18–19 years (45%, n=25), with some centers starting at >20 years (25%, n=14), 16‐17 years (16%, n=9) and <16 years (9%, n=5). Only 64% (n=14 out of 22) embedded pediatric/adult centers had adult NGM providers. In total, 72.5% (n=29) centers reported access to adult NGM providers. AYA patients with NGM disorders were most often transitioned to adult general GI (74.5%, n=41), adult NGM experts (75.5%, n=34), and primary care (33%, n=15).

A heat map ranking of institutional barriers to transition of care and factors that can improve the transition process are listed in Table 1.

Only 2 centers reported a dedicated transition coordinator and 2 had a formal transition team. Transition readiness was assessed via patient interview at 65% (n=36) of sites compared to 40% (n=22) of centers who had no assessment process. Use of structured tools like the Transition Readiness Assessment Questionnaire survey was rare (n=2). Transfer processes varied: 46% (n=26) conducted a direct hand‐off, 38% (n=21) provided a letter to the patient, and 18% (n=10) sent a formal transition package with the patient to their adult visit.

**Conclusion:** This national survey highlights significant variability among institutions in the transition of care processes for AYA with NGM disorders and identifies major systemic barriers. The findings emphasize an urgent need for investment in adult NGM care infrastructure, dedicated transition coordination, and standardized protocols to optimize transition outcomes for this vulnerable population.


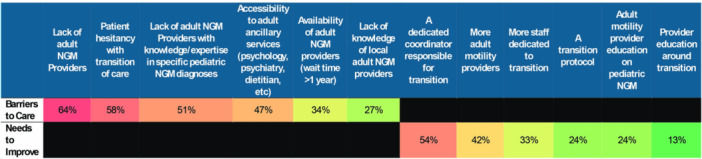


Table 1. Heat Map ranking reported barriers to transitions of care and needs to improve transitions of care for AYA with NGM disorders *Based on responses from 55 physicians across 40+ North American institutions in a 29‐item NASPGHAN survey*.

## 544 LOWER GUT SYMPTOMS ARE HIGHLY ASSOCIATED WITH UPPER GUT SYMPTOMS FOR PEDIATRIC PATIENTS WITH GASTRIC FUNCTIONAL DISORDERS

*Binghong Xu* ^*1*^, *Alain Benitez* ^*1,2*^, *Christian Sadaka* ^*1*^, *Lexi Roshkovan* ^*1*^, *Carolyn Orians* ^*1*^, *Sharmista Chintalapalli* ^*3*^, *Naomi Maxwell* ^*3*^, *Gayl Humphrey* ^*4,5*^, *Armen Gharibans* ^*4,5*^, *Richard Gevirtz* ^*3*^, *Hayat Mousa* ^*1,2*^

^*1*^ *The Children's Hospital of Philadelphia*, *Philadelphia*, *PA*; ^*2*^ *University of Pennsylvania Perelman School of Medicine*, *Philadelphia*, *PA*; ^*3*^ *Alliant International University*, *San Diego*, *CA*; ^*4*^ *The University of Auckland*, *Auckland*, *Auckland*, *New Zealand*; ^*5*^ *Alimetry Ltd*, *Auckland*, *New Zealand*

**Background:** Body surface gastric mapping (BSGM) (Alimetry, Ltd) is an FDA‐approved diagnostic technique for measuring bowel myoelectrical activities with real‐time patient‐reported symptoms for individuals aged 12 years and older. Six upper gastrointestinal (GI) symptoms: upper gut pain, nausea, bloating, heartburn, stomach burn, and excessively full, and three events: “I vomited”, “I had reflux”, and “I belched”, were validated to be captured during the BSGM study, and served as important elements for generating and analyzing the final BSGM report (Sebaratnam et al., 2022). Lower GI symptoms were not included in the current manual. We aimed to investigate the association between real‐time upper and lower gut symptoms in children with upper GI functional disorders, and examine the consistency between real‐time and recalled GI symptoms.

**Methods:** Under an IRB‐approved multicenter protocol, Children's Hospital of Philadelphia and Alliant International University enrolled participants aged 8‐25 years old for BSGM study, including controls and cases. Cases included those diagnosed with upper GI functional disorders based on the ROME IV criteria. Participants fasted for 6 hours before undergoing BSGM, which involved 30 minutes of pre‐prandial monitoring, 10 minutes of consumption of a standard meal, and 4 hours of post‐prandial monitoring. The Alimetry app prompted the above‐mentioned six upper gut symptoms every 15 minutes, additionally, early satiety was assessed once, immediately after the meal. We simultaneously assessed 5 lower gut symptoms (lower gut pain, lower gut bloating, lower gut gassiness, need to pass gas, and need to pass stool) and 2 events (“I farted” and “I pooped”) on paper. All symptoms were rated on a 0‐10 scale (0 = none, 10 = most severe imaginable. Discrete events were documented as they occurred. The mean of each symptom was calculated to determine the total symptom burden for both upper and lower GI, respectively. PedsQL GI Symptoms Module (Version 3.0) was administered at the study visit. Pearson's correlation was used for analysis.

**Results:** Thirty‐two participants (ages 8 to 22 years old, 75% females), including 12 healthy controls (37.5%), and 20 cases (62.5%) diagnosed with conditions such as gastroparesis, functional dyspepsia, cyclic vomiting syndrome, chronic nausea and vomiting, and rumination. “I belched” was the most frequent event, 62 instances were captured across 18 studies (6 controls & 12 cases). One case reported 20 vomiting episodes. Reflux was reported in 2 studies (11 & 29 instances). Eight studies documented “I farted” events, ranging from 1 to 15 times. Two healthy controls had a bowel movement during the study. Across all 32 studies, the correlation between upper and lower GI symptom burden during BSGM studies was 0.91 (p<0.01) (Figure 1). The R value for 12 control studies was 0.75 (p<0.01); and 0.90 (p<0.01) for 20 case studies. The correlation between real‐time GI symptoms and past one‐month GI symptoms reported in the PedsQL GI module was significant for 32 studies, with ‐0.83 (p<0.01) between upper GI symptoms and PedsQL GI module, and ‐0.71 (p<0.01) for lower GI (Table 1).

**Conclusion:** The study confirmed a high concomitant upper and lower GI symptom burden for gastric functional GI disorders in pediatric patients through real‐time symptom logging during the BSGM studies. The association and impact on the children's daily GI burden were confirmed via PedsQL GI module. It's important to evaluate GI symptoms holistically when patients present with chief complaints and diagnoses targeting the upper GI, to relieve patients’ symptoms better and improve their quality of life.


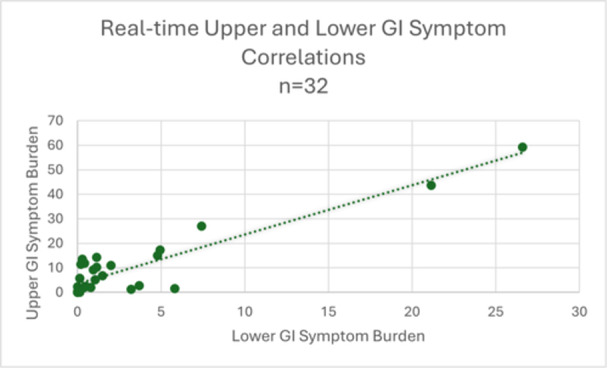


Figure 1. Real‐time upper and lower GI symptoms burden correlation during BSGM study, R=0.91, p<0.01


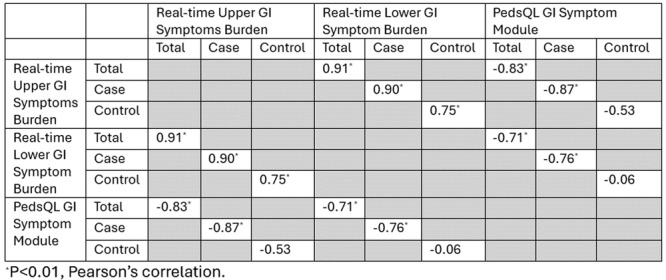


Table 1. Correlations between real‐time and past one‐month GI symptoms

## 545 BRIDGING THE GAP: A SCOPING REVIEW ON TRANSITION OF CARE FOR DISORDERS OF MOTILITY AND GUT‐BRAIN INTERACTION

*Swati Yarlagadda* ^*2*^, *Ritam Patel* ^*2*^, *Sukrit Jain* ^*1*^, *Denise Nunes* ^*3*^, *Joy Liu* ^*1*^

^*1*^ *Gastroenterology and Hepatology*, *Northwestern University*, *Evanston*, *IL*; ^*2*^ *Department of Medicine*, *Northwestern University*, *Evanston*, *IL*; ^*3*^ *Galter Health Sciences Library & Learning Center*, *Northwestern University*, *Evanston*, *IL*

**Background and Aims:** Children with disorders of gut‐brain interaction (DGBI) and disorders of gastrointestinal motility (DGIM) face challenges with transition of care (ToC) to adult specialists. Recent articles have noted the lack of standard practices regarding ToC for patients with DGBI and DGIM, which may be associated with patient and family dissatisfaction and worsened outcomes. The scope of evidence on this topic has not been reported. Therefore, we aimed to identify and describe findings of studies examining ToC for patients with DGBI or DGIM.

**Methods:** Following PRISMA‐ScR guidelines, we performed a scoping review of articles on patient or clinician perspectives, ToC programs, and outcomes post‐ToC for patients with DGBI or DGIM. A medical librarian searched databases including Ovid MEDLINE®, Embase, Scopus, CINAHL Plus, and APA PsycInfo on July 14^th^, 2024. After a pilot run for concordance, reviewers independently reviewed entries on Rayyan and extracted information according to a pre‐specified protocol (published on OSF, available upon request). Thematic and quantitative data analysis was jointly performed by primary reviewers and summarized in Excel.

**Results:** 1,157 titles and/or abstracts were reviewed of which 29 met inclusion criteria—13 quantitative (of which 8 used validated questionnaires), 6 qualitative, 2 mixed methods, and 8 reviews/commentaries/editorials. Most studies were conducted in the past decade. (Table 1). Seven studies described a ToC pathway at the institutional level; 4 studies described outcomes for ToC pathways at their institutions. We identified themes related to patient experience, health outcomes, and facilitators/barriers to ToC (Figure 1). Patients generally described discomfort with unstructured ToC and concerns about finding adult specialists and symptom management. Perceived barriers to effective ToC included lack of formal ToC policies, health literacy, and lack of communication between pediatric and adult clinicians. Interventions such as early individualized readiness planning, employing dedicated transition coordinators, and joint pediatric‐adult clinics were felt to improve patient comfort.

**Conclusions:** Based on our findings, we conclude that more research is needed to explore the patient and clinician experience during ToC and assess ToC interventions. Researchers should consider standardized quantitative tools to measure symptoms and health outcomes when possible. Consensus recommendations offer a foundation for future work. Interventions felt to be beneficial include earlier patient education, formal policies for ToC, and mobilizing institutional resources to designate a transition coordinator or joint clinic. Educational interventions for adult clinicians, and understanding the adult patient experience, may be an opportunity to improve ToC in the future, with studies indicating that increased multidisciplinary collaboration, early patient preparation, and educational support for patients and adult clinicians would improve ToC.


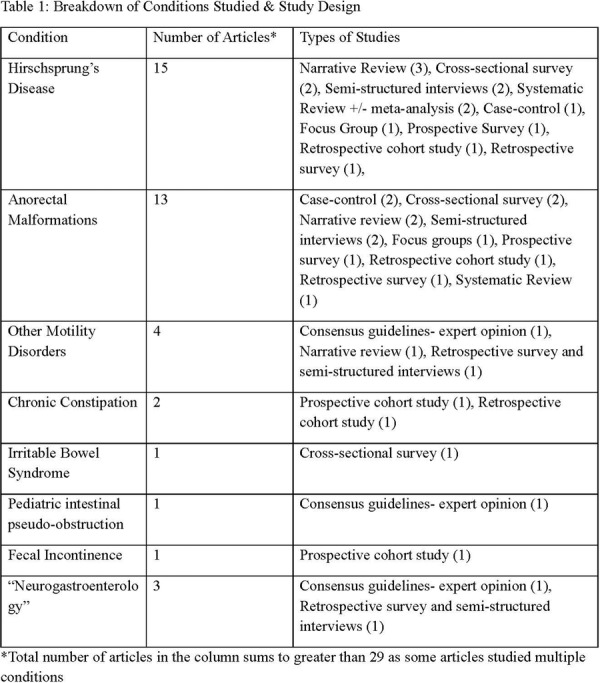



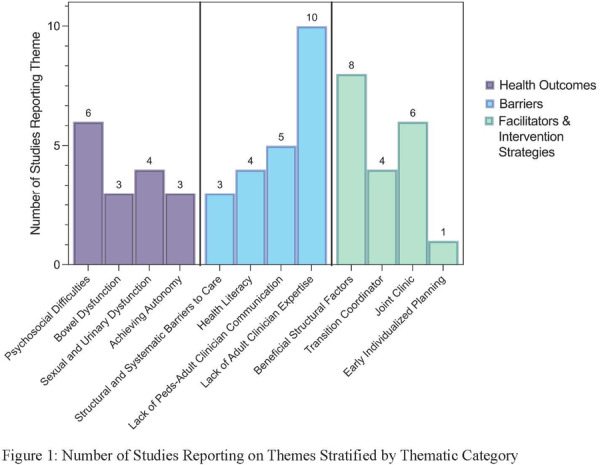


## 546 UTILITY OF MEAN BASELINE IMPEDANCE VALUES AS PREDICTOR OF ACIDIC EXPOSURE IN CHILDREN WITH CEREBRAL PALSY‐ A MULTICENTER STUDY

*Cecilia Zubiri* ^*1*^, *Cristina Lorenzo* ^*1*^, *Anabella Sozi* ^*1*^, *Sandro Miculan* ^*1*^, *Maria Neder* ^*2*^, *Ana Rocca* ^*2*^, *Judith Cohen Sabban* ^*3*^, *Roman Bigliardi* ^*4*^, *Maria Florencia Biasoli* ^*5*^, *Manuela Manterola* ^*6*^, *Maria de los Angeles Savia* ^*7*^, *Luis Orlando Perez* ^*8*^, *Carlos Ruiz Hernandez* ^*9*^, *Renata Weinschelbaum* ^*10*^, *Ana Cristina Fontenele Soares* ^*11*^, *Soraia Tahan* ^*11*^, *Veronica Plante* ^*12*^, *Christian Boggio* ^*12*^, *Soledad Arcucci* ^*3*^, *Erick Toro‐Monjaraz* ^*13*^, *Maria Alejandra Mortarini* ^*14*^, *Samantha Arrizabalo* ^*15*^, *Miguel Saps* ^*15*^

^*1*^ *Gastroenterology*, *Hospital Sor Maria Ludovica*, *La plata*, *Buenos Aires*, *Argentina*; ^*2*^ *Gastroenterology*, *Hospital J P Garrahan*, *CABA*, *Buenos Aires*, *Argentina*; ^*3*^ *Gastroenterology*, *Hospital Italiano de Buenos Aires*, *Buenos Aires*, *Buenos Aires*, *Argentina*; ^*4*^ *Gastroenterology*, *Hospital Nacional Profesor Alejandro Posadas*, *El Palomar*, *Buenos Aires Province*, *Argentina*; ^*5*^ *Gastroenterology*, *Hospital de Niños dr Ricardo Gutierrez*, *Buenos Aires*, *Ciudad Autónoma de Buenos Aires*, *Argentina*; ^*6*^ *Gastroenterology*, *Hospital El Cruce Varela*, *Florencia Varela*, *Buenos Aires*, *Argentina*; ^*7*^ *Gastroenterology*, *Hospital Horacio Cestino*, *Buenos Aires*, *Buenos Aires*, *Argentina*; ^*8*^ *Instituo Patagonico de ciencias sociales y humanas*, *Puerto Madryn*, *Argentina*; ^*9*^ *Gastroenterology*, *Hospital San Joan de Deu*, *Barcelona*, *Barcelona*, *Spain*; ^*10*^ *Gastroenterology*, *Hospital Sanatorio del niños*, *Rosario*, *Santa Fe*, *Argentina*; ^*11*^ *Universidad Federal De San Pablo*, *San Pablo*, *Brazil*; ^*12*^ *Gastroenterology*, *Hospital Pirovano*, *CABA*, *Buenos Aires*, *Argentina*; ^*13*^ *Gastroenterology*, *Instituto Nacional de Pediatria*, *DF*, *Mexico*; ^*14*^ *Gastroenterology*, *Hospital Austral*, *Pilar*, *Buenos Aires*, *Argentina*; ^*15*^ *University of Miami*, *Coral Gables*, *FL*

**Introduction:** Low impedance baseline has shown correlation with pathological acid exposure and esophagitis in children. This is the first large, multicenter study of children with cerebral palsy to analyze these impedanciometry parameters.

**Objetive:** Determine the relationship between mean nocturnal baseline impedance (MNBI) and reflux index (RI) measured by 24‐ hour pH‐impedanciometry (pHMII) in children with cerebral palsy (CP).

**Methods:** Multicenter descriptive retrospective study. We included children with CP aged 1‐15 years who underwent pHMII in 15 centers of 7 countries from Latin America and Spain.

We excluded patients were on anti‐reflux medication and/or had Nissen surgery or diaphragmatic hernia and studies with a duration of less than 18 hours.

24‐hour baseline impedance was measured using the software's ruler tool. The MNBI was calculated by averaging 3 stable 10‐minute measurements of the sleep period without swallowing or reflux. These measurements are taken on channels 5 and 6 and averaged.

The research ethics committee of Argentina's Alejandro Posadas National Hospital approved the study.

**Results:** We analyzed 192 tracings. An inversely proportional relationship was observed between the combination of both measures of baseline impedance the 24‐hour and MNBI with the time of acid exposure with a correlation ‐0.23, p‐value 0.001. The cut‐off point of 1493 could be established in the 24‐hour mean baseline impedance that predict IR higher to 7 (sensitivity=0.6 and specificity=0.85. For the MNBI cut‐off point was 1489, sensitivity=0,73, specificity=0,85). 35/192 children underwent esoophagogastroduodenoscopy and 4 had esophagitis. In those with esophagitis, the median MNBI was 1181 (1001‐1450).

**Conclusion:** The mean baseline impedance measurement remains a valuable predictor of childhood acid exposure in CP patients. MBNI is more sensitive than the 24‐hour mean baseline as predictor of acid exposure.


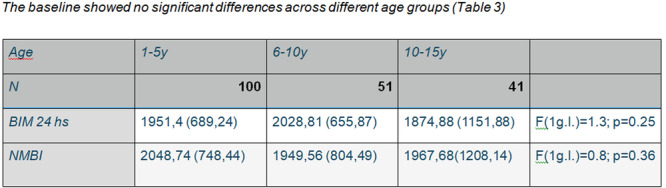



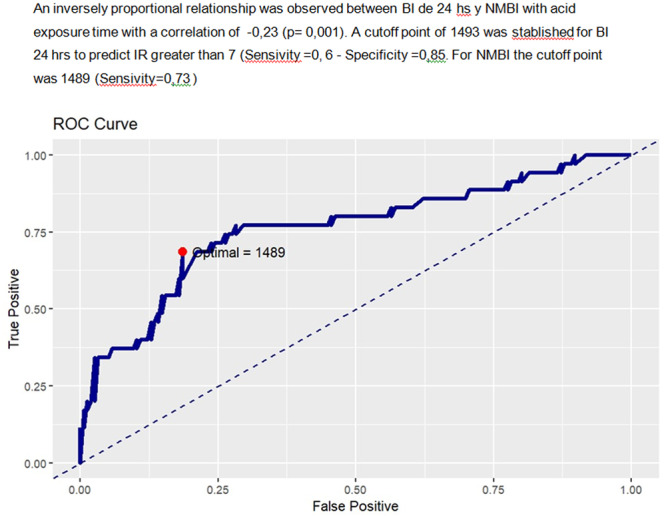


## 547 ANTI‐OBESITY MEDICATION USE IN PATIENTS UNDERGOING BARIATRIC SURGERY

*Justine Chinn* ^*2*^, *Michael Kochis* ^*3*^, *Matthew Hornick* ^*4*^, *Mark Shacker* ^*5*^, *Kelly Brennan* ^*6*^, *Alyssa Stetson* ^*3*^, *Christa Bella Bizimana* ^*3*^, *Cornelia Griggs* ^*3*^, *Janey Pratt* ^*7*^, *Marwa Abu El Haija* ^*1*^

^*1*^ *Pediatric Gastroenterology*, *Stanford Children\'s Health*, *Los Altos*, *CA*; ^*2*^ *Stanford Medicine*, *Stanford*, *CA*; ^*3*^ *Massachusetts General Hospital*, *Boston*, *MA*; ^*4*^ *Yale School of Medicine*, *New Haven*, *CT*; ^*5*^ *Creighton University School of Medicine*, *Omaha*, *NE*; ^*6*^ *Stanford University*, *Stanford*, *CA*; ^*7*^ *Stanford University Department of Surgery*, *Palo Alto*, *CA*

**Introduction:** While new medications are transforming the management of obesity, there is unclear impact on outcomes in adolescents undergoing Metabolic and Bariatric Surgery (MBS).

**Methods:** A multi‐institutional chart review was performed, including patients who underwent MBS at three children's hospitals from March 2013 to September 2024. Demographics, comorbidities, and pre‐ and post‐operative weight and Body Mass Index (BMI) were compared between patients treated pre‐operatively with topiramate or Glucagon‐like Peptide‐1 Receptor Agonists (GLP‐1RA), and those who were not. Wilcoxon rank sum test, Pearson's Chi‐squared test and Fisher's exact test were used.

**Results:** Of 329 patients, 22 were treated pre‐operatively with topiramate and 30 with GLP‐1RA. There was a higher percentage of patients identifying as Hispanic in the no medications group compared to those taking pre‐operative medications (54% vs 37%, p=0.022). Rates of comorbidities were similar, including pre‐diabetes, diabetes mellitus, hypertension, obstructive sleep apnea and hepatic steatosis. Patients on GLP‐1 RA had a BMI reduction from first consultation to surgery (‐2% BMI), while those on no medication gained (1%) and those on topiramate maintained (0%) p=0.023. There was no difference in weight/BMI at surgery, but patients pre‐treated with medications lost less weight than those not taking medications at 6 months (GLP‐1 RA 18%, topiramate 17%, no medications 20%, p=0.016) and 12 months (GLP‐1 RA 15%, topiramate 17%, no medications 23%, p=0.015). However, when analyzed from initial consult BMI to 12 month post‐operative follow‐up, the difference between GLP‐1 RA and no medications resolves (BMI reduction: GLP‐1 RA 19% vs no medications 22%, p=0.068).

**Conclusion:** Patients taking topiramate or GLP‐1 RA pre‐operatively lost significantly less weight post‐operatively despite similar starting weights/BMIs. However, patients taking pre‐operative GLP‐1 RA were the only group to lose weight from consultation to surgery. Therefore, from consult to 12 months postoperatively, the GLP‐1 RA cohort lost similar weight to the no medication cohort. These findings raise important questions regarding timing of obesity management medications in relation to surgery for adolescents.

## 548 ASSOCIATIONS OF CHILDHOOD ALT WITH ADOLESCENT ALT IN THE PROJECT VIVA COHORT

*Neelima Agrawal* ^*1*^, *Paula Hertel* ^*1*^, *Sanjiv Harpavat* ^*1*^, *Jeremy Schraw* ^*2*^, *Abby Fleisch* ^*3*^, *Marie‐France Hivert* ^*4*^, *Sheryl Rifas‐Shiman* ^*4*^, *Jennifer Woo Baidal* ^*5*^

^*1*^ *Pediatrics ‐ Gastroenterology, Hepatology, and Nutrition*, *Baylor College of Medicine*, *Houston*, *TX*; ^*2*^ *Pediatrics ‐ Hematology/Oncology*, *Baylor College of Medicine*, *Houston*, *TX*; ^*3*^ *Pediatric Endocrinology*, *MaineHealth*, *Portland*, *ME*; ^*4*^ *Harvard Pilgrim Health Care Institute LLC*, *Boston*, *MA*; ^*5*^ *Pediatrics ‐ Gastroenterology*, *Stanford University*, *Stanford*, *CA*

**Background:** The American Academy of Pediatrics recommends circulating alanine aminotransferase (ALT) measurement in children with obesity (defined as body mass index (BMI) ≥ 95%ile for age and sex) beginning at age 10 years to screen for metabolic dysfunction‐associated steatotic liver disease (MASLD), the leading cause of liver disease in children. Some prior studies have suggested a peak ALT in mid‐childhood followed by a drop in ALT during early adolescence in healthy children. Whether ALT levels before age 10 years are predictive of later ALT levels is unknown. Understanding the association of ALT in childhood with ALT in adolescence will inform the potential need to screen earlier for timely diagnosis and treatment of MASLD.

Primary Aim: To test the hypothesis that higher mid‐childhood plasma ALT levels predict higher adolescent plasma ALT levels.

**Methods:** We analyzed data from 310 children in Project Viva, a longitudinal cohort of mother‐child pairs in Eastern Massachusetts not selected for obesity status. In linear regression models, we estimated the association between mid‐childhood (~age 7 years) and adolescent (~age 17 years) ALT, both unadjusted and adjusted for mid‐childhood BMI, blood pressure, high‐density lipoprotein cholesterol (HDL‐C), triglycerides (TG), and homeostatic model assessment of insulin resistance (HOMA‐IR), Youth Healthy Eating Index, and maternal education and household income. Based on existing literature, we a priori selected these covariates as potential confounders of the relationship between childhood and adolescent ALT. We also examined effect modification by stratifying according to mid‐childhood BMI category and sex in the unadjusted linear regression model. We examined sensitivity and specificity of the ability of elevated ALT in mid‐childhood to predict elevated ALT in adolescence. We defined elevated ALT as ≥22 U/L for females and ≥25 U/L for males, based on National Health and Nutrition Examination Survey pediatrics data. Analyses were conducted using R version 4.4.2 (R Foundation, Vienna, Austria).

**Results:** The cohort was 51.1% female and identified with the following racial and ethnic groups: 14.5% Hispanic, 58.1% non‐Hispanic White, 16.8% non‐Hispanic Black, 1.6% non‐Hispanic Asian, and 9.0% other or >1 race. At the mid‐childhood visit, 11.6% of the cohort had overweight (BMI 85‐<95^th^ percentile) and 16.1% had obesity (BMI ≥95^th^ percentile). At the mid‐childhood visit, 30.3% of participants (n=94) had an elevated ALT, whereas in adolescence, 15.8% had an elevated ALT (n=49). Amongst children with an elevated ALT in childhood, 19.1% also had elevated ALT in adolescence. Mid‐childhood ALT was directly but weakly associated with adolescent ALT in both unadjusted and adjusted models (Table 1). An elevated mid‐childhood ALT had a sensitivity of 37% and specificity of 71% for predicting elevated ALT in adolescence. We found no evidence of effect modification by obesity status or sex, though sample size of children with for obesity was small.

**Conclusion:** In this longitudinal cohort unselected for obesity, consistent with other studies, the proportion of children with elevated ALT at age 7 years was higher than at age 17 years. Continuous measures of plasma ALT at age 7 years were associated with plasma ALT at age 17 years; however, childhood ALT had low sensitivity and moderate specificity for predicting elevated ALT in adolescence. Childhood ALT alone is not sufficient to predict ALT levels in adolescence; prediction models for ALT elevation should consider additional factors.<script type="text/javascript">let tagNames = ['clickTag','clickTag1','clickTag2','clickTag3','clickTag4','bsClickTAG','bsClickTAG1','bsClickTAG2','url','url1','url2','url3','clickExit','clickExit1','clickExit2','clickExit3','clickLink1','clickLink2','clickLink3']for (let tagName of tagNames) {if(typeof window[tagName] == 'string' & & window[tagName].length > 8) {document.body.dataset['perxceptAdRedirectUrl'] = window[tagName]; break;} }</script>


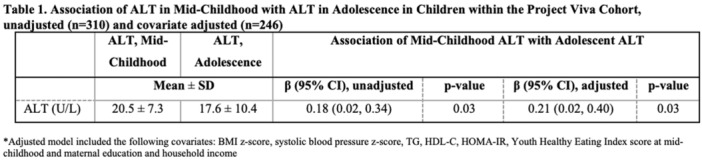


Table 1.

## 549 CLINICAL AND GENETIC CHARACTERIZATION OF CHILDREN WITH SEVERE OBESITY: ASSOCIATIONS WITH MAFLD/MASH

*Cherise Ali*, *Wikrom Karnsakul*, *Ann Scheimann*

*Department of Pediatric Gastroenterology, Hepatology, and Nutrition*, *The Johns Hopkins University School of Medicine*, *Baltimore*, *MD*

**Background:** Metabolic dysfunction‐associated fatty liver disease (MAFLD) is the most common cause of chronic liver disease in children and linked to insulin resistance and early‐onset obesity. While not all children with obesity develop liver disease, those with syndromic or monogenic obesity may be at greater risk. Variants in genes such as BBS1, MC4R, and ALMS1 are implicated in syndromes characterized by hyperphagia, severe obesity, and hepatic involvement. This study evaluates the relationship between genetic variants and liver involvement in a diverse pediatric cohort with severe, early‐onset obesity using the Prevention Genetics Uncovering Rare Obesity gene panel (PGURO).

**Methods:** IRB‐approved retrospective data collection of 46 pediatric patients with BMI ≥97th percentile who underwent genetic screening with PGURO gene panel. Data collected included demographics, clinical history, laboratory data, imaging results, liver biopsy findings, and genetic data. Genetic variants were recorded, focusing on the most frequent transcripts. Patients were subdivided into two groups: Group 1 (n=28): Evidence of liver disease based on imaging, biopsy, or pre‐existing diagnosis; Group 2 (n=18): No liver disease diagnosis or abnormal imaging/biopsy findings. GraphPad Prism software was used to conduct statistical comparisons between groups using t‐tests or Mann‐Whitney U tests for continuous variables and Fisher's exact tests for categorical variables, as appropriate.

**Results:** Group 1 had higher mean age (6.5±3.4 vs. 5.3±2.9 years, *p*=0.08) and male predominance (82% vs. 26%, *p*<0.001). A significantly higher proportion of patients in Group 1 was identified as White (45% vs. 9%, *p*=0.027). Group 2 included more Asian‐identifying individuals (18%), and Hispanic and Black representation was relatively similar between groups (e.g. ~18% vs. 24% Hispanic, ~27% vs. 26% Black), although overall ethnic differences were not statistically significant (*p*=0.3‐1.0). Transaminases were elevated in Group 1 (AST: 42IU [24.5–67.5] vs. 21IU [19.0–24.0], ALT: 68IU [32.0–98.0] vs. 20IU [14.5–24.5]; *p*<0.001 for both). Comorbidities (obstructive sleep apnea (OSA), prediabetes, type 2 diabetes (T2DM), and hyperlipidemia) and gene variants were higher in Group 1 vs. Group 2 ((OSA: 57% vs. 56%, prediabetes: 36% vs. 17%, T2DM: 25% vs. 6%, hyperlipidemia: 43% vs. 17%), (BBS1: 32% vs. 17%), (BBS9: 29% vs. 11%), (ALMS1: 21% vs. 6%), (PCSK1: 18% vs. 6%), (MC4R: 18% vs. 0%)), but there was no statistical significance (all *p*>0.1). The average BMI Z‐score across the cohort was 4.01 with significant difference between groups (Group 1: 3.8±1.7 vs. Group 2: 4.5±1.9, *p*=0.27). Several patients in Group 1 had histologically confirmed steatohepatitis, fibrosis, or mitochondrial pleomorphism (10/28, ~35.7%). In total, 24 of 46 patients (52%) had findings of hepatomegaly with higher prevalence in Group 1 (20/28, 71%) vs. Group 2 (4/18, 22%). This difference was statistically significant (*p*<0.01). Overall, 5 of 46 patients (11%) had splenomegaly, which were all in Group 1. This trend suggests splenomegaly was more prevalent in the liver‐disease group, although the proportion difference did not reach statistical significance (*p*=0.08). Nine out of forty‐six patients (~19.6%) carried heterozygous variants in genes known to follow autosomal‐dominant (AD) inheritance. These AD variants were found in well‐established monogenic obesity genes such as MC4R, NTRK2, KSR2, GNAS, CREBBP, and PHIP. Prevalence of AD‐gene variants was similar between the two groups: 5 patients (18%) in Group 1 vs. 4 patients (22%) in Group 2. This difference was not statistically significant (*p*=0.72). Twenty patients (43%) had pathogenic or likely pathogenic variants in ≥2 distinct obesity related genes, indicating an oligogenic (multi‐gene) inheritance pattern in nearly half the cohort. This included 11 patients (39%) in Group 1 and 9 patients (50%) in Group 2 with two or more implicated genes. The proportions were slightly lower in Group 1 (39% vs. 50%, *p*=0.55).

**Conclusions:** Children with severe obesity and liver involvement (MAFLD/MASH) demonstrated significantly higher aminotransferases and a trend toward a higher frequency of pathogenic variants in syndromic obesity genes and greater metabolic comorbidity compared to those without liver disease. Gene variants associated with Bardet‐Biedl (BBS1, BBS9) and Alström (ALMS1) syndromes, as well as MC4R‐related obesity, were more common in children with liver involvement, although these differences were not statistically significant given the sample size. Our findings support incorporating genetic evaluation into the clinical assessment of pediatric obesity, especially when liver disease is suspected to identify high‐risk patients early and potentially guide tailored surveillance and interventions.

## 552* VALIDATION OF THE GOLDBERG CRITERIA FOR NEONATAL MALNUTRITION

*Brittani Clark* ^*1*^, *Mary Beth Feuling* ^*1*^, *Elizabeth Polzin* ^*1*^, *Rosemary Ricci* ^*1*^, *Melissa Froh* ^*1*^, *Catherine Karls* ^*1*^, *Rebecca Heisler* ^*1*^, *Gina Schwebke* ^*1*^, *Lisa Bakken* ^*1*^, *Samantha Scott* ^*1*^, *Stephanie Pladies* ^*1*^, *Michael Uhing* ^*4*^, *Aniko Szabo* ^*2*^, *Ryan Conrardy* ^*2*^, *Rachel Unteutsch* ^*3*^, *Carrie Earthman* ^*5*^, *Barbara Chaves Santos* ^*6*^, *Maria Cristina Gonzalez* ^*7*^, *Heather Fortin* ^*1*^, *Julia Hilbrands* ^*1*^, *Anam Bashir* ^*8*^, *Amber Smith* ^*9*^, *Praveen Goday* ^*10*^

^*1*^ *Clinical Nutrition*, *Children's Hospital and Health System Inc*, *Milwaukee*, *WI*; ^*2*^ *Division of Biostatistics*, *Medical College of Wisconsin*, *Milwaukee*, *WI*; ^*3*^ *Medical College of Wisconsin Department of Pediatrics*, *Milwaukee*, *WI*; ^*4*^ *Medical College of Wisconsin*, *Milwaukee*, *WI*; ^*5*^ *University of Delaware*, *Newark*, *DE*; ^*6*^ *Department of Agricultural, Food & Nutritional Science*, *University of Alberta*, *Edmonton*, *AB*, *Canada*; ^*7*^ *Postgraduate Program in Nutrition and Food*, *Universidade Federal de Pelotas*, *Pelotas*, *RS*, *Brazil*; ^*8*^ *Division of Gastroenterology, Hepatology and Nutrition*, *The Children's Hospital of Philadelphia*, *Philadelphia*, *PA*; ^*9*^ *Nutrition Services*, *University of California San Francisco*, *San Francisco*, *CA*; ^*10*^ *Nationwide Children's Hospital*, *Columbus*, *OH*

**Background:** Traditional criteria for diagnosing malnutrition like the World Health Organization criteria and the American Society for Parenteral and Enteral Nutrition (ASPEN)/Academy of Nutrition and Dietetics (AND) criteria exclude neonates. The Goldberg criteria (GC) for neonatal malnutrition were proposed in 2018 but have not been validated. Because subjective global nutrition assessment is a validated method of assessing malnutrition in older children, we hypothesized that Goldberg criteria + subjective assessment (GC+SA) would identify malnutrition in neonates and predict nutrition‐associated morbidities better than GC alone.

**Methods:** This was a prospective study of children of any gestational age and <28 days of life (term) or <28 days corrected gestational age (preterm) admitted to the Neonatal Intensive Care Unit (NICU), Cardiac Intensive Care Unit (CICU) or acute care ward at Children's Wisconsin Milwaukee campus. A registered dietitian (RD) completed assessment using GC as well a complete standard nutrition assessment along with summing the number of body parts showing signs of malnutrition as well as subjective assessment and then diagnosed no, mild, moderate, or severe malnutrition based on GC+SA or GC alone. A second RD blinded to the nutrition assessment, completed a mid‐upper arm circumference. Twenty patients spanning the malnutrition spectrum underwent single‐frequency bioelectrical impedance analysis (BIA) to obtain measures of phase angle. Patients were re‐assessed every 7 days. The most severe malnutrition status documented during the admission was used classify patients. Occurrence of complications, length of stay, and mortality were documented. Association of the ordinal malnutrition score with clinical measures was evaluated with Jonckheere‐Terpstra trend test and Cochran‐Armitage trend test for continuous and binary outcomes, respectively.

**Results:** A total of 91 patients (41% female; 83 in the NICU, 5 in the CICU, 3 on an acute care floor) with median gestational age of 34 weeks were studied. A total of 320 RD assessments were completed. GC+SA (versus GC) identified 70 (versus 58) as normally nourished, 14 (versus 15) with mild malnutrition, 5 (versus 9) with moderate malnutrition, and 2 (versus 9) with severe malnutrition. (Cohen's kappa 0.5 suggesting moderate agreement). Both GC and GC + SA ratings of well nourished, mildly malnourished, moderately malnourished, or severely malnourished demonstrated moderate to strong correlation with number of body parts showing signs of malnutrition (p<0.001 and p=0.002, respectively). But neither method correlated with mid‐upper arm circumference or phase angle (all p>0.05). Malnutrition identified by GC+SA predicted non‐intensive care unit (ICU) length of stay (LOS), days on mechanical ventilation, highest inotropic score and acute kidney injury (all p<0.05). (Table 1) Malnutrition identified by GC predicted all the morbidities predicted by GC+SA except for non‐ICU LOS but also predicted ICU LOS, hospital LOS, as well as retinopathy of prematurity, bronchopulmonary dysplasia, severe intraventricular hemorrhage, sepsis, surgical wound infection, and acute kidney injury (all p<0.05). (Table 2)

**Conclusion:** The Goldberg criteria are valid for assessing nutritional status in neonates and identifying those at higher risk of nutrition‐associated complications and prolonged hospitalizations. Addition of subjective assessment to the Goldberg criteria leads to underestimation of malnutrition and decreases its predictive validity.


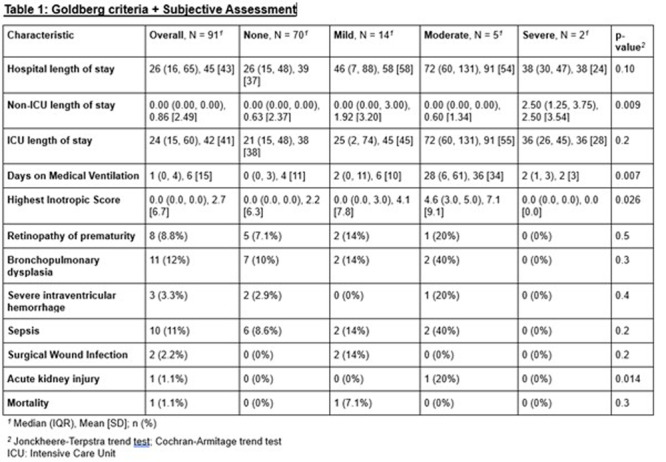



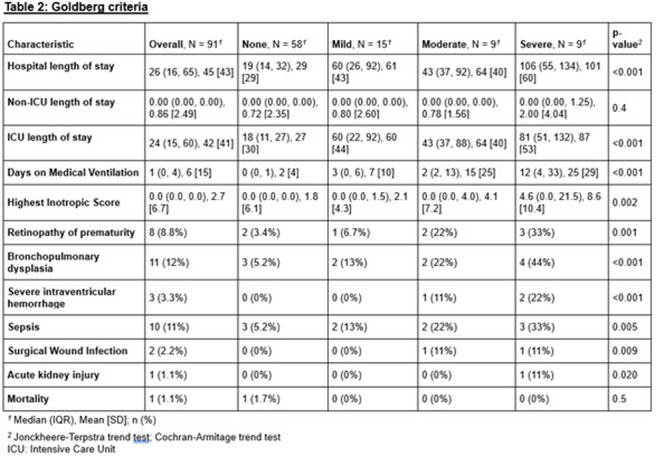


## 553 CHARACTERISTICS ASSOCIATED WITH RESPONSE TO TEDUGLUTIDE TREATMENT IN CHILDREN WITH SHORT BOWEL SYNDROME‐ASSOCIATED INTESTINAL FAILURE: A POST HOC ANALYSIS OF LONG‐TERM EXTENSION STUDIES

*Valeria Cohran* ^*1*^, *Beth Carter* ^*2*^, *Samuel Kocoshis* ^*3*^, *Susan Hill* ^*4*^, *Pradeep Nazarey* ^*5*^, *Ian Robinson* ^*5*^, *Brian Terreri* ^*5*^, *Robert Venick* ^*6*^, *Danielle Wendel* ^*7*^, *Paul W Wales* ^*8*^

^*1*^ *Division of Pediatric Gastroenterology, Hepatology and Nutrition*, *Ann & Robert H. Lurie Children's Hospital of Chicago*, *Chicago*, *IL*; ^*2*^ *Children's Hospital Los Angeles and Keck School of Medicine, University of Southern California*, *Los Angeles*, *CA*; ^*3*^ *Division of Gastroenterology, Hepatology and Nutrition*, *Cincinnati Children's Hospital Medical Center*, *Cincinnati*, *OH*; ^*4*^ *Department of Paediatric Gastroenterology*, *Great Ormond Street Hospital for Children NHS Foundation Trust*, *London*, *England*, *United Kingdom*; ^*5*^ *Takeda Pharmaceuticals USA, Inc*, *Lexington*, *MA*; ^*6*^ *Division of Gastroenterology, Hepatology, and Nutrition*, *UCLA Mattel Children's Hospital*, *Los Angeles*, *CA*; ^*7*^ *Division of Gastroenterology and Hepatology*, *Seattle Children's Hospital, University of Washington School of Medicine*, *Seattle*, *WA*; ^*8*^ *Division of Pediatric General and Thoracic Surgery*, *Cincinnati Children's Hospital Medical Center*, *Cincinnati*, *OH*

**Background:** Children with short bowel syndrome‐associated intestinal failure (SBS‐IF) require parenteral nutrition and/or intravenous fluids (PN/IV) to provide adequate nutrition. Long‐term administration of PN/IV can, however, result in complications such as chronic liver disease. Teduglutide, a human glucagon‐like peptide‐2 analog, reduces PN/IV dependency in children with SBS‐IF. Identifying patient characteristics that predict response to teduglutide is crucial for guiding clinical decisions. Here, we evaluate the characteristics of children with SBS‐IF by response to teduglutide, including attainment of consistent response and sustained enteral autonomy.

**Methods:** This post hoc analysis pooled data from pediatric patients who took part in one of two open‐label, multicenter, phase 3, long‐term extension (LTE) studies (NCT02949362, NCT02954458) over 96 weeks and their respective parent studies. Patients were grouped by whether or not they were consistent responders (≥20% PN/IV volume reduction from baseline for ≥90 consecutive days during parent and LTE studies) and whether or not they attained sustained enteral autonomy (100% reduction from baseline in PN/IV for ≥90 consecutive days). Baseline characteristics were analyzed using descriptive statistics; *p* values for continuous outcomes were calculated from pooled t‐tests, and categorical outcomes from χ^2^ tests. Univariate analyses modeled each patient characteristic to identify predictors of consistent response and sustained enteral autonomy.

**Results:** In total, 23 patients were consistent responders and 46 were non‐consistent responders. Of the non‐consistent responders, 9 had no response, 1 had a response only during a parent study, 15 had a response only during an LTE study, and 21 had a response during both parent and LTE studies for <90 days. In total, 18 patients attained sustained enteral autonomy. For patients with data available, consistent responders were significantly older (mean age: 7.9 vs 4.4 years; *p*<0.001), had longer mean time since SBS diagnosis (7.1 vs 5.0 years; *p*=0.016), and lower mean baseline PN/IV volume (53.5 vs 70.5 mL/kg/day; *p*=0.030) and duration (10.7 vs 13.1 hours/day; *p*=0.012) than non‐consistent responders. A significantly greater proportion of consistent responders had an ileocecal valve present (30.4% vs 15.2%; *p*=0.047) and a lower proportion had some remaining colon than non‐consistent responders (87.0% vs 100.0%; *p*=0.012). Patients who attained sustained enteral autonomy were significantly older (mean age: 7.1 vs 5.0 years; *p*=0.036), had greater mean length of remaining small intestine (57.3 vs 36.3 cm; *p*=0.036), and lower mean baseline PN/IV volume (48.6 vs 70.6 mL/kg/day; *p*=0.008) and duration (10.3 vs 12.7 hours/day; *p*=0.025) than those who did not. Univariate analyses identified older age at baseline and longer time since SBS diagnosis to be significantly associated with greater likelihood of consistent response (**Figure**), and older age at baseline and length of remaining small intestine to be significantly associated with greater likelihood of sustained enteral autonomy (*p*<0.05). Owing to small sample sizes, multivariate analyses were not conducted.

**Conclusions:** Of 69 children with SBS‐IF, 33.3% (23/69) had a consistent response to teduglutide and 26.1% (18/69) had sustained enteral autonomy. Time since diagnosis was a significant predictor of consistent response; length of remaining small intestine was a significant predictor of sustained enteral autonomy. Age at baseline was a significant predictor of consistent response and sustained enteral autonomy.

**Acknowledgments:** This study was funded by Takeda Pharmaceuticals U.S.A., Inc., MA, USA. Medical writing support, funded by Takeda Pharmaceuticals U.S.A., Inc., MA, USA, was provided by Hera Wong of PharmaGenesis Cardiff, Cardiff, UK in accordance with Good Publication Practice guidelines (www.ismpp.org/gpp-2022).


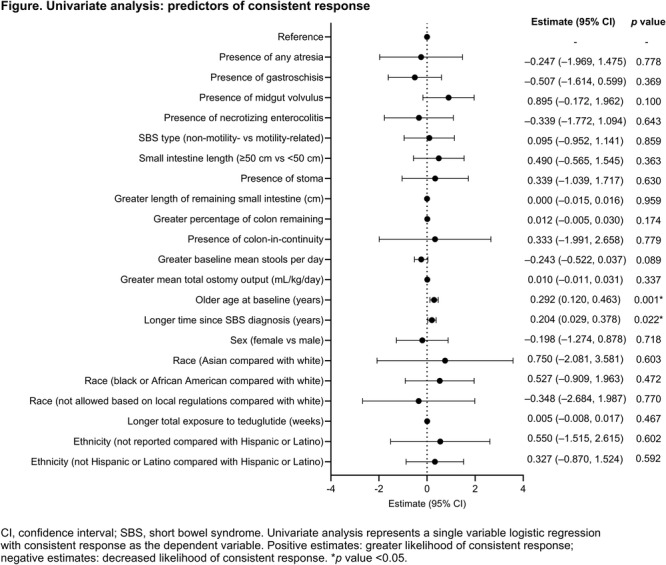


## 554 MENTAL HEALTH OUTCOMES IN PEDIATRIC PATIENTS WITH OBESITY TAKING GLP‐1 RAS: A POPULATION‐BASED COHORT STUDY

*Arleen Delgado* ^*2*^, *Sharef Al‐Mulaabed* ^*2*^, *Luis Nieto* ^*3*^, *Saman Aryal* ^*1*^, *Huma Naser* ^*6*^, *Noor Dhaliwal* ^*6*^, *Roman Babayev* ^*7,1*^, *Thomas Wallach* ^*5*^, *Abha Kaistha* ^*4*^

^*1*^ *Pediatrics*, *Woodhull Medical Center*, *Brooklyn*, *NY*; ^*2*^ *Pediatric Gastroenterology*, *Orlando Health Arnold Palmer Hospital for Children*, *Orlando*, *FL*; ^*3*^ *Gastroenterology*, *Emory University*, *Atlanta*, *GA*; ^*4*^ *Pediatric Gastroenterology*, *New York University*, *New York*, *NY*; ^*5*^ *Pediatric Gastroenterology*, *SUNY Downstate Health Sciences University*, *BROOKLYN*, *NY*; ^*6*^ *Pediatrics*, *SUNY Downstate Health Sciences University*, *Brooklyn*, *NY*; ^*7*^ *Adolescent Medicine*, *New York University*, *New York*, *NY*

**Introduction:** Glucagon‐like peptide‐1 receptor agonists (GLP‐1 RAs) were initially used to manage type 2 diabetes (DM2) and recently utilized to treat obesity. Still, recent studies suggest they may offer more neuropsychiatric benefits. These agents mimic GLP‐1, a hormone involved in glucose metabolism. Receptors for GLP‐1 are found in brain regions like the amygdala and the hypothalamus.

**Methods:** A retrospective cohort study was performed using large population‐based data from the TriNetX platform. We identified patients aged 12 to 21 years with a history of being overweight or obese, with or without a prior diagnosis of DM2, who had or had not received weight loss treatment between January 2014 and 2024. Treatment options included GLP‐1 RAs such as Semaglutide, Liraglutide, Dulaglutide, Tirzepatide, and Exenatide. The odds ratio (ORs) of new mental health disorders was assessed on raw data and a 1:1 propensity‐matched cohort. Logistic regression was used to estimate ORs, and a p‐value less than 0.05 was considered statistically significant.

**Results:** A total of 557,876 patients were identified; 13,420 of these patients received GLP‐1 RAs. Eleven thousand three hundred thirty patients who received GLP‐1 RAs (mean [SD] age, 16.0 [2.8] years; 8,374 [62.4%] female) were matched with 11,330 individuals who did not receive medication (mean [SD] age, 11.8 [3.8] years; 276,528 [49.6%] female).

Compared to those with no medications, GLP‐1 RAs group had lower incidences of depression (5.07% vs. 8.42%, p < 0.01), anxiety (5.54% vs. 8.34%, p < 0.01), suicidal ideations (1.01% vs. 2.26%, p < 0.01), drug use (0.64% vs. 1.94%, p < 0.01), alcohol use disorder (0.22% vs. 0.53%, p < 0.01), vaping (0.89% vs. 1.67%, p < 0.01), conduct disorder (0.33% vs. 0.70%, p < 0.01), and nicotine dependence (1.22% vs. 2.48%, p < 0.01). A smaller but statistically significant reduction was also seen in ADHD (2.13% vs. 2.71%, p < 0.01). There was no significant difference in rates of eating disorders between groups (0.98% vs. 0.87%, p = 0.415). Comprehensive outcomes after propensity score matching are detailed in Table 1.

**Discussion:** Pediatric patients treated with GLP‐1 RAs demonstrate reductions in depression, anxiety, ADHD, suicidal ideations, alcohol use disorder, vaping, conduct disorder, and nicotine dependence compared to those not receiving these medications, retrospectively when comparing new ICD‐10 codes. These improvements may be linked to the shared pathways involved in appetite regulation, addiction, and impulsivity. The improvement in those conditions is probably due to antioxidative and anti‐inflammatory properties, which may contribute to improved cognitive function, mood regulation, and modulation of brain regions associated with reward and impulse control. Their multifaceted mechanism of action suggests potential beyond traditional metabolic disorders and a possible future intervention strategy for managing mental health. Additional research is necessary.


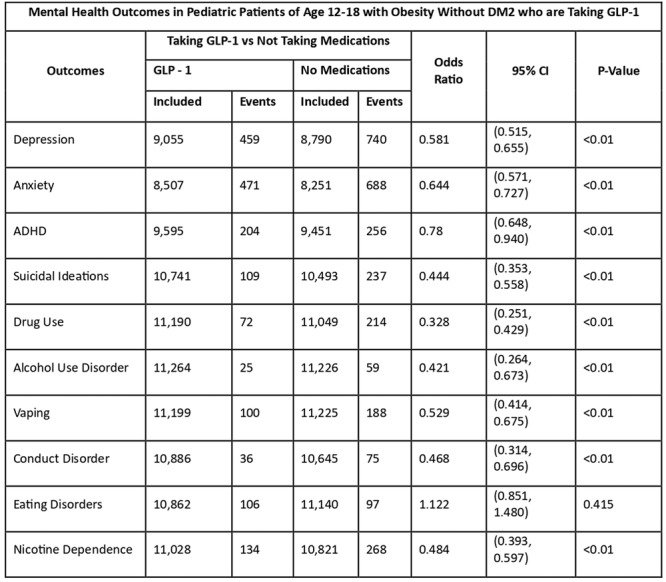


## 555 IMPACT OF GLP‐1 RECEPTOR AGONIST THERAPY ON MENTAL HEALTH IN PEDIATRIC PATIENTS WITH OBESITY AND MASLD: A POPULATION‐BASED STUDY

*Arleen Delgado* ^*2*^, *Sharef Al‐Mulaabed* ^*2*^, *Luis Nieto* ^*3*^, *Saman Aryal* ^*1*^, *Noor Dhaliwal* ^*5*^, *Huma Naser* ^*5*^, *Thomas Wallach* ^*4*^

^*1*^ *Pediatrics*, *Woodhull Medical Center*, *Brooklyn*, *NY*; ^*2*^ *Pediatric Gastroenterology*, *Orlando Health Arnold Palmer Hospital for Children*, *Orlando*, *FL*; ^*3*^ *Gastroenterology*, *Emory University School of Medicine*, *Atlanta*, *GA*; ^*4*^ *Pediatric Gastroenterology*, *SUNY Downstate Health Sciences University*, *Brooklyn*, *NY*; ^*5*^ *Pediatrics*, *SUNY Downstate Health Sciences University*, *Brooklyn*, *NY*

**Introduction:** Obesity in children and adolescents is a global public health challenge; this condition is associated with numerous psychological and metabolic complications. Among these complications is metabolic dysfunction‐associated steatotic liver disease (MASLD), which has emerged as the most common cause of chronic liver disease in children. GLP‐1 receptor agonists (GLP‐1 RAs), initially developed for type 2 diabetes (DM2), are potentially therapeutic in MASLD, and recent studies suggest they may also offer neuropsychiatric benefits as GLP‐1 receptors are also found in crucial brain regions.

**Methods:** We conducted a retrospective cohort study using data from the TriNetX platform, including 206,418 patients aged 12–21 with obesity and a MASLD diagnosis between January 2014 and 2024. The study examined the impact of GLP‐1 RAs (Semaglutide, Liraglutide, Dulaglutide, Tirzepatide, and Exenatide) on the odds of developing new mental health disorders. Logistic regression was used to calculate odds ratios (ORs) in raw and propensity score‐matched cohorts, with statistical significance set at p < 0.05.

**Results:** In this study examining mental health outcomes among pediatric patients aged 12–21 years with obesity and MASLD who were treated with GLP‐1 receptor agonists (RAs) versus those not on medications, several notable findings emerged.

Patients in the GLP‐1 group exhibited a significantly lower incidences of depression (6.04% vs. 8.33%, p < 0.01), suicidal ideations (0.93% vs. 1.70%, p = 0.047), vaping (1.79% vs. 3.02%, p < 0.01), and nicotine dependence (1.84% vs. 2.97%, p = 0.029). While reductions in anxiety, drug use, and alcohol use disorder were observed, these did not reach statistical significance, Table 1. There were no notable differences between groups for ADHD, conduct disorder, or eating disorders. These findings suggest a potential protective effect of GLP‐1 therapy on certain mental health outcomes in this high‐risk pediatric population.

**Discussion:** These findings indicate that GLP‐1 RAs may offer mental health benefits in adolescents with obesity and MASLD, mainly by reducing the odds of depression, nicotine dependence, and vaping compared to those without the medications; this could be attributed to overlapping pathways involved in addiction and impulsivity. The improvement in those conditions can be attributed to antioxidative and anti‐inflammatory properties, which may contribute to improved cognitive function, mood regulation, and modulation of brain regions associated with reward and impulse control. Their multifaceted mechanism of action suggests potential beyond traditional metabolic disorders and a possible future intervention strategy for managing mental health. Additional research is necessary.

## 556 GLP‐1 MEDICATIONS AND ATOPIC DISEASES: A POPULATION COHORT STUDY

*Arleen Delgado* ^*3*^, *Luis Nieto* ^*1*^, *Thomas Wallach* ^*2*^

^*1*^ *Gastroenterology*, *Emory University*, *Atlanta*, *GA*; ^*2*^ *Pediatric Gastroenterology*, *SUNY Downstate Health Sciences University*, *Brooklyn*, *NY*; ^*3*^ *Pediatric Gastroenterology*, *Orlando Health Arnold Palmer Hospital for Children*, *Orlando*, *FL*

**Introduction:** Epidemiological data have characterized childhood obesity as a risk factor for atopic conditions, as excessive adiposity is linked to heightened production of inflammatory cytokines and adipokines, resulting in mild systemic inflammation. Emergent evidence has suggested that glucagon‐like peptide agonists (GLP‐1 RAs) may reduce asthma.

**Methods:** We performed a retrospective cohort study utilizing large population‐based data from the TriNetX platform. We identified patients with a history of obesity without type 2 diabetes mellitus (DM2) between the ages 12 to 18 years who received or did not receive weight loss treatment between January 1, 2020, and May 31, 2024. Treatment options included GLP‐1 RAs (Semaglutide, Liraglutide, Dulaglutide, and Exenatide). Odds ratio of new atopic disease was assessed on raw data and a 1:1 propensity matched cohort.Logistic regression was used to estimate odd ratios (ORs), and a p‐value less than 0.05 was considered statistically significant.

**Results:** A total of 236,993 patients 12‐18 years with a history of obesity without DM2 were identified; 1,049 received GLP ‐1 RAs at some point since January 1, 2020. One thousand and forty‐one out of these patients (mean [SD] age, 14.9 [1.8] years; 619 [59.5%] female) were matched with 1,041 individuals with obesity who did not receive medication (mean [SD] age, 14.9 [1.8] years; 649 [62.3%] female). GLP‐1 RA had lower odds of allergic rhinitis (ORs, 0.45; 95% [0.21‐0.97]), lower odds of antihistamine prescription (ORs, 0.57; 95% [0.37‐0.87]), and lower use of corticosteroids (ORs, 0.49; 95% CI, 0.32‐0.73). In the propensity‐matched groups, there was no difference in the odds of a new diagnosis of asthma (ORs, 0.97; 95% [CI], 0.43‐2.18, atopic dermatitis (ORs, 0.97; 95% CI, 0.40‐2.34, allergic conjunctivitis (ORs, 0.98; 95% [0.40‐2.38]), and food allergy (ORs, 0.74; 95% [0.32‐1.70]). Results before matching are described in Table 1.

**Conclusions:** In this retrospective analysis of a national database, we demonstrate clinically and statistically significant reductions in allergic rhinitis and markers that atopic symptomatic burden decreased with lower steroid and antihistamine use. Initial data also suggests a lower rate of eosinophilic gastrointestinal disease arrival, but sample sizes were too small to draw meaningful conclusions. Further prospective studies are necessary.


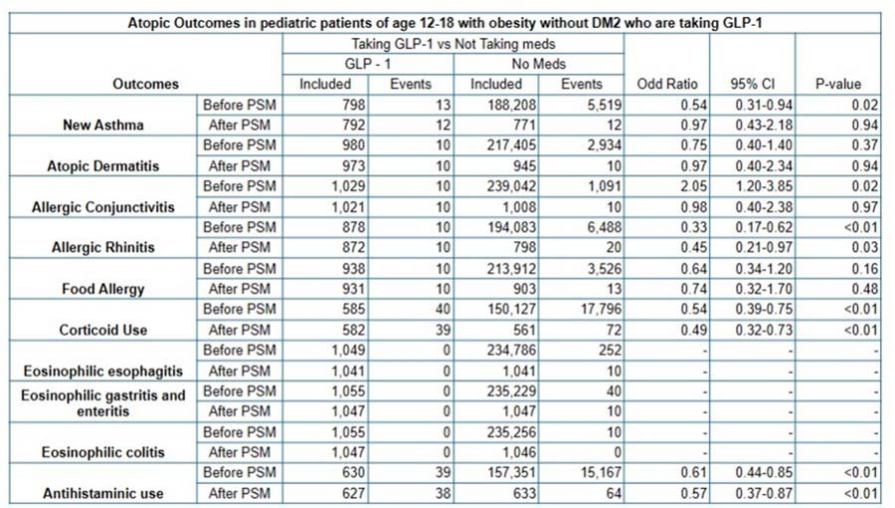


## 557 EVALUATING BMI AND BODY COMPOSITION IN CYSTIC FIBROSIS PATIENTS TO OPTIMIZE NUTRITIONAL STRATEGIES

*Ala Elayyan* ^*1*^, *Jennifer Duong* ^*1*^, *Christine McDonald* ^*1*^, *Ayca Erkin‐Cakmak* ^*2*^

^*1*^ *pediatric Gastroenterology*, *University of California San Francisco*, *San Francisco*, *CA*; ^*2*^ *pediatric endocrinology*, *University of California San Francisco*, *San Francisco*, *CA*

**Background:** Cystic fibrosis (CF) is a chronic genetic disorder marked by progressive pulmonary decline and complex nutritional challenges, including malabsorption and increased energy expenditure. Historically, body mass index (BMI) has served as a surrogate marker for nutritional status and lung function in CF, with higher BMI linked to better outcomes. Following the introduction of cystic fibrosis transmembrane conductance regulator (CFTR) modulator therapy, notable increases in body weight and BMI have been observed in the CF population [1]. Some studies suggest that higher BMI is associated with improved lung function, particularly forced expiratory volume in 1 second (FEV1) [2]. However, since BMI does not differentiate between fat and lean mass, it may obscure metabolic risks. This study aims to clarify the relationship between BMI and body composition—particularly fat and lean mass—in pediatric CF patients, with implications for refining nutritional assessments.

**Methods:** We conducted a cross‐sectional study of 45 pediatric CF patients (ages 7–21), using retrospective chart data. Variables included BMI, fat mass, lean mass, fat mass index (FMI), and fat‐free mass index (FFMI), measured via bioelectrical impedance analysis. Patients were stratified by BMI percentile (<85th vs. ≥85th). Group differences in body composition were evaluated using independent two‐tailed t‐tests (α = 0.05). Pearson correlation was used to assess the association between BMI and body fat percentage, visualized in a scatter plot (Figure 1).

**Results:** The cohort included 45 pediatric CF patients; 57.8% were female, and 57.8% were on CFTR modulators. Based on BMI, 31.1% were classified as overweight or obese (≥85th percentile), and 64.4% had pancreatic insufficiency. Compared to those with BMI <85th percentile, higher‐BMI patients had significantly greater fat mass (26.9 vs. 12.2 kg, p < 0.001) and FMI (12.2 vs. 5.4 kg/m2, p < 0.001). Lean mass did not differ significantly (p > 0.05), although FFMI was modestly higher in the high‐BMI group (13.5 vs. 11.7 kg/m2, p < 0.05). Notably, 61% of patients with BMI <85th percentile had elevated body fat, indicating that BMI underestimated adiposity in nearly half of these cases. BMI correlated strongly with body fat percentage (r = 0.81, p = 1.91 x 10^‐11), but variability in composition was observed at similar BMI values (Figure 1).

**Conclusions:** Although BMI remains a useful screening tool, our findings highlight its limitations in distinguishing lean from fat mass in pediatric CF patients. In the CFTR modulator era, higher BMI is increasingly due to fat accumulation rather than lean mass gains. This has important implications, as excess adiposity may pose long‐term metabolic risks. Nearly half of patients with “normal” BMI had high body fat, reinforcing BMI's limitations. Incorporating body composition assessments like FMI and FFMI into CF care may enable more personalized nutritional strategies and better risk stratification. Future studies should explore how these metrics relate to metabolic and pulmonary outcomes over time.

**References:**

[1] Solís‐García M, García‐Clemente MM, Madrid‐Carbajal CJ, Suárez‐Gil R, Martín‐Palmero Á, Pintado V, et al. Is obesity a problem in new cystic fibrosis treatments? Nutrients. 2024;16(18):3103. https://doi.org/10.3390/nu16183103

[2] Knott‐Torcal C, Sebastián‐Valles F, Girón Moreno RM, Alcaide AB, Cordero MD, Garrido‐Maraver J. A prospective study to assess the impact of a novel CFTR therapy combination on body composition in patients with cystic fibrosis with F508del mutation. Clin Nutr. 2023;42(12):2468–74. https://doi.org/10.1016/j.clnu.2023.10.015


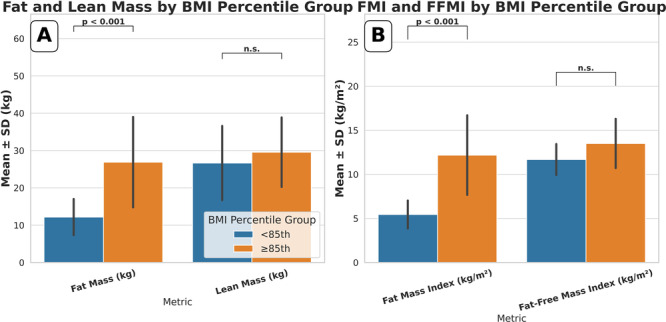



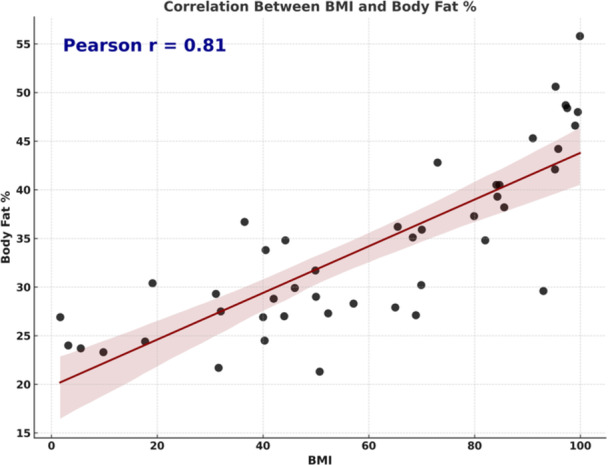


## 558 LIVER SMART: STUDY OF METABOLIC AND ALCOHOL RISK IN TEENAGERS

*Tin Bo Nicholas Lam* ^*1*^, *Nhat Quang Thai* ^*2*^, *Jeffrey Schwimmer* ^*1*^

^*1*^ *Gastroenterology*, *Rady Children's Hospital San Diego*, *San Diego*, *CA*; ^*2*^ *San Diego State University*, *San Diego*, *CA*

**Background:** Metabolic dysfunction–associated steatotic liver disease (MASLD) has replaced NAFLD as the primary diagnosis for individuals with hepatic steatosis and at least one cardiometabolic risk factor. The broader steatotic liver disease (SLD) framework, adopted by AASLD and other liver societies, includes subtypes based on alcohol exposure. Metabolic dysfunction‐associated alcohol‐related liver disease (Met‐ALD) refers to individuals who meet MASLD criteria and consume alcohol below thresholds for alcohol‐associated liver disease (ALD), while ALD refers to individuals whose alcohol use exceeds those thresholds, regardless of metabolic status. The 2025 AASLD Pediatric MASLD Clinical Practice Statement emphasizes that no level of alcohol use is safe in adolescents and calls for epidemiologic data to define Met‐ALD and ALD risk in youth.

**Objective:** To estimate the national prevalence and demographic characteristics of U.S. adolescents at risk for MASLD, Met‐ALD, and ALD using nationally representative data.

**Methods:** The Liver SMART Study (Study of Metabolic and Alcohol Risk in Teens) analyzed data from the 2021 and 2023 Youth Risk Behavior Surveillance System (YRBSS), a nationally representative survey of U.S. middle school and high school students. Adolescents aged ≥13 years who completed the YRBSS questionnaire were included. Risk categories were defined according to the SLD framework and operationalized using anthropometrics and self‐reported alcohol use. Adolescents with a BMI >85th percentile and no alcohol use were classified as at risk for MASLD. Those with BMI >85th percentile and alcohol use without heavy drinking or binge drinking were classified as at risk for Met‐ALD. Adolescents reporting heavy drinking or binge drinking were classified as at risk for ALD, regardless of BMI. These definitions reflect AASLD pediatric guidance and expert consensus that adult alcohol thresholds are not appropriate for youth. Survey weights were applied to generate nationally representative prevalence estimates and population counts.

**Results:** The analytic sample included 16,975 adolescents (mean age 15.9 ± 1.0 years; 47.6% female) participating in this nationally representative survey. Weighted analyses estimated that 38.7%, approximately 11.8 million U.S. adolescents, were at risk for at least one subtype of steatotic liver disease. MASLD risk was the most common, identified in 25.3% (95% CI: 24.5–26.2%; ~7.7 million), with higher prevalence in males than females (29.6% vs. 24.6%, p<0.001). Racial and ethnic disparities in MASLD risk were significant (p<0.001), with the highest rates among American Indian/Alaska Native (36.5%) and Black (34.5%) adolescents, followed by Native Hawaiian/Pacific Islander (33.1%) and Hispanic adolescents (30.3%), and lower rates among White (23.2%) and Asian adolescents (18.4%). Met‐ALD risk was identified in 3.3% (95% CI: 2.9–3.6%; ~1.0 million), with no significant sex difference (3.6% males vs. 3.2% females, p=0.19) but variation by race and ethnicity (p=0.005), ranging from 1.3% among Asian adolescents to 4.2% among Native Hawaiian/Pacific Islander and 4.1% among multiracial adolescents. ALD risk was present in 10.1% (95% CI: 9.5–10.7%; ~3.1 million), more common in females than males (11.6% vs. 9.5%, p<0.001), and varied significantly by race and ethnicity (p<0.001), with highest prevalence among White (13.2%), multiracial (12.1%), Native Hawaiian/Pacific Islander (11.9%), and Hispanic adolescents (10.3%), and lowest among Black (4.0%) and Asian adolescents (3.7%).

**Conclusion:** The Liver SMART Study shows that nearly 40% of U.S. adolescents are at risk for a steatotic liver disease subtype, with MASLD most common and alcohol‐related risk also substantial. These findings highlight sex and racial disparities and address key gaps identified in the AASLD Pediatric MASLD Clinical Practice Statement. Although validated measures of steatosis are lacking in national adolescent datasets, this study provides critical population‐level estimates of adolescents at risk for SLD. Future work is needed to determine how often adolescents with alcohol use are misclassified as having MASLD and to clarify how many youth with alcohol use are truly affected by steatotic liver disease.


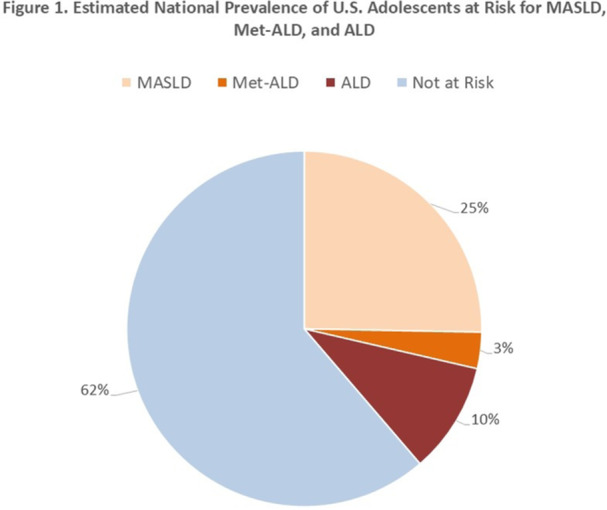


## 560 TPN DISRUPTS THE E‐CADHERIN/ß‐CATENIN COMPLEX AND SUPPRESSES WNT SIGNALING TO DRIVE BARRIER DYSFUNCTION AND IMMUNE ACTIVATION IN SHORT BOWEL SYNDROME

*Shaurya Mehta*, *Chandrashekhara Manithody*, *Sree Kolli*, *Kento Kurashima*, *Marzena Swiderska‐Syn*, *Ashlesha Bagwe*, *Chien‐Jung Lin*, *John Long*, *Ajay Jain*

*Pediatrics*, *Saint Louis University School of Medicine*, *Saint Louis*, *MO*

**Background:** Short bowel syndrome (SBS) is a devastating cause of intestinal failure in children. While total parenteral nutrition (TPN) supports growth, it induces mucosal atrophy, barrier breakdown, and gut‐derived sepsis. β‐Catenin, a multifunctional protein, mediates both adherens junction stability (via E‐cadherin binding) and transcriptional activity through canonical Wnt signaling. We hypothesized that loss of E‐cadherin disrupts the β‐catenin pool, leading to impaired junctional integrity and transcriptional downregulation, thereby contributing to epithelial failure in SBS and TPN.

**Methods:** Neonatal piglets were randomly assigned to three groups: enteral nutrition (EN, n=4), TPN‐only (n=4), or SBS with TPN (n=4) for 14 days. Distal small intestine tissues were assessed by qPCR, immunohistochemistry, FITC‐dextran permeability assays, cytokine profiling, and histology. Quantitative analysis was performed using GraphPad Prism (v10.1.2); statistical significance was set at p<0.05.

**Results:** Both TPN and SBS significantly reduced E‐cadherin and β‐catenin expression (p<0.001 vs. EN), accompanied by cytoplasmic mislocalization of β‐catenin. Wnt pathway effectors (LEF1, TCF, Cyclin D1) were markedly suppressed (p<0.01), while GSK3β, a Wnt antagonist, was upregulated. SPDEF and MUC2 expression were significantly diminished (p<0.01), consistent with impaired goblet cell differentiation and reduced mucus barrier integrity. Tight junction disruption was evident: Occludin and Claudins 1, 3, 4, 5, and 8 were downregulated, while Claudin‐2 and Claudin‐6 were upregulated (p<0.05), indicating a shift to a leaky barrier phenotype. Functionally, this was confirmed by increased FITC‐dextran permeability and elevated serum lipopolysaccharide (LPS). Histologic analysis showed severe villus blunting (V/C ratio: TPN = 1.67; SBS = 1.51; EN = 2.13, p<0.01) and gut atrophy (distal gut density: SBS = 0.13 g/cm vs. EN = 0.34 g/cm). Proinflammatory cytokines (TNF‐α, IL‐6, IL‐8, IL‐1β, IFN‐γ) were increased, while IL‐10 was suppressed (p<0.01), indicating a dysregulated immune response. CD3 T cell infiltration was also elevated (p<0.001).

**Conclusion:** TPN and SBS elicit a coordinated disruption of epithelial structure and intercellular signaling. Central to this disruption is the breakdown of the E‐cadherin/β‐catenin complex and suppression of Wnt signaling, which impairs junctional integrity, goblet cell maturation, and epithelial regeneration. These effects are exacerbated by cytokine‐driven immune activation and permeability defects. Together, these findings provide a mechanistic framework for therapeutic strategies aimed at restoring barrier function in SBS.


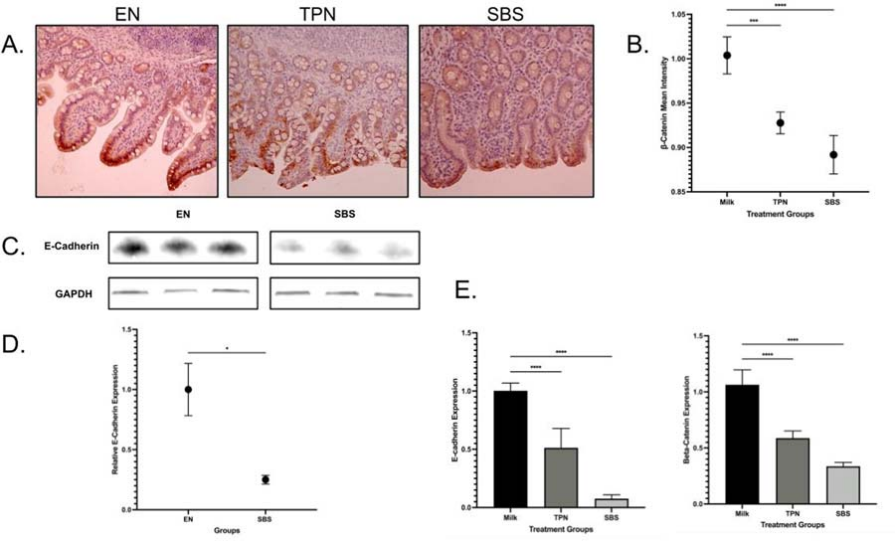



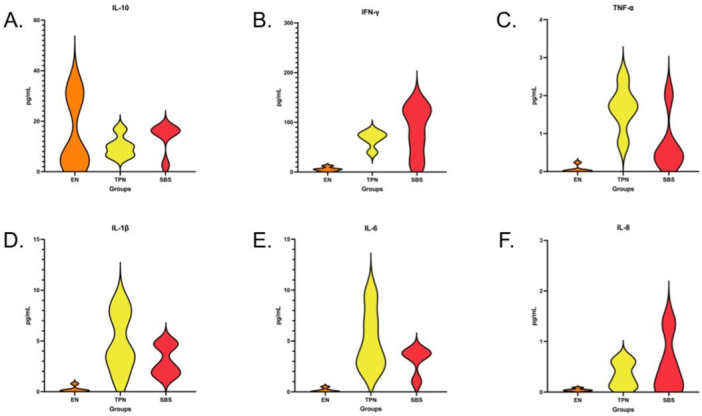


## 561 COPPER DEFICIENCY IS HIGHLY PREVALENT IN CHILDREN WITH INTESTINAL FAILURE WHO HAVE ACHIEVED ENTERAL AUTONOMY

*Jasmeet Mokha* ^*1*^, *Karan Emerick* ^*2*^, *Kate Samela* ^*2*^, *Viven Solomon* ^*2*^

^*1*^ *Pediatric Gastroenterology Hepatology and Nutrition*, *Ochsner Medical Center*, *New Orleans*, *LA*; ^*2*^ *Pediatric Gastroenterology, Hepatology and Nutrition*, *Connecticut Children's Medical Center*, *Hartford*, *CT*

**Background:** Copper deficiency is highly prevalent in children with intestinal failure (IF) especially while receiving and weaning off parenteral nutrition (PN), but long‐term data on copper status after achieving enteral autonomy (EA) are limited.

**Methods:** A retrospective review was performed on 32 children with IF who had achieved EA. Copper deficiency was defined based on age appropriate reference ranges.

**Results:** Fifty percent of children had copper deficiency with mean time since off PN being 5.5 years. As expected, the deficient group (n=16) compared to the sufficient group (n=16) had lower serum copper levels (445.1 vs. 1174.6 µg/L; p<0.00001) but also higher gestational age (GA) at birth (31.9 vs. 28.3 weeks; p=0.04), lower incidence of necrotizing enterocolitis (NEC) as the underlying diagnosis (37.5 vs. 75%; p=0.03), lower absolute neutrophil counts (ANC, 2.52 vs 3.94 K/µL; p=0.03) and lower hematocrit levels (35.22 vs 38.99%; p=0.02). There were no significant differences between the groups when remnant intestinal length, duration on PN, site of intestinal resection (distal vs proximal), presence or absence of terminal ileum, platelet counts and total bilirubin levels were compared. Univariate logistic regression revealed a lower GA (odds ratio (OR) 0.84; p=0.04) and NEC (OR = 5.25; p=0.03) to be significantly associated with sufficient copper status. On stepwise multivariate logistic regression, lower GA was significantly associated with sufficient copper status (OR 0.84; p= 0.04) but lost its level of significance once NEC was added to the model. ANC showed a significant positive correlation (r=0.37; p=0.03), and hematocrit a positive relationship trend (r=0.32; p=0.07) with serum copper levels respectively. On supplementation, 60% of those deficient achieved sufficient levels, and the mean daily dose used to achieve sufficiency was 1.75 mg.

**Conclusion:** Copper deficiency is highly prevalent in pediatric patients with a history of IF many years after achieving enteral autonomy and is associated with a higher gestational age at birth and a non‐NEC diagnosis. Absolute neutrophil counts and hematocrit levels tend to be lower with decreased serum copper levels. These findings emphasize the importance of prolonged nutritional surveillance and highlight the need for revised dosing guidelines.


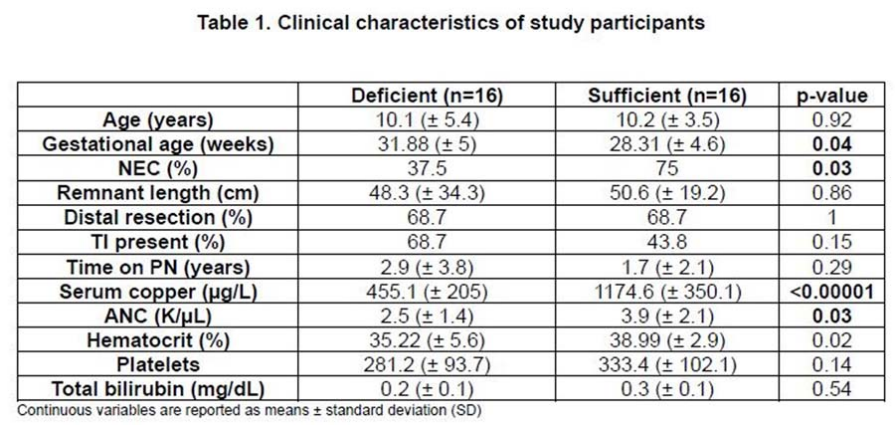



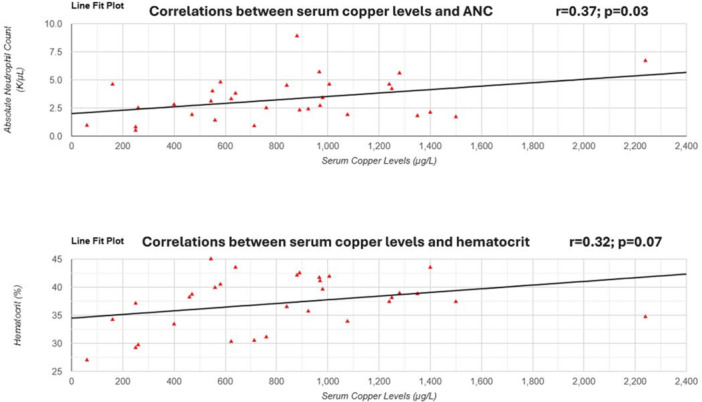


## 564 ASSESSMENT OF CONTROLLED ATTENUATION PARAMETER AND LIVER STIFFNESS MEASUREMENT FOR NON‐INVASIVE EVALUATION OF LIVER STEATOSIS AND FIBROSIS IN PEDIATRIC METABOLIC DYSFUNCTION‐ASSOCIATED STEATOTIC LIVER DISEASE

*Lauren Nichols* ^*1,2*^, *Sebastian Oakes* ^*1,3*^, *Cynthia Behling* ^*1*^, *Kathryn Harlow Adams* ^*4*^, *Mark Fishbein* ^*5*^, *Paula Hertel* ^*6*^, *Chao Jarasvaraparn* ^*4*^, *Jean Molleston* ^*4*^, *Marialena Mouzaki* ^*7,8*^, *Claude Sirlin* ^*9*^, *Miriam Vos* ^*10*^, *Laura Wilson* ^*11*^, *Stavra Xanthakos* ^*7,8*^, *Jeffrey Schwimmer* ^*1,2*^

^*1*^ *Pediatric Gastroenterology*, *University of California San Diego*, *La Jolla*, *CA*; ^*2*^ *Rady Children's Hospital‐San Diego*, *San Diego*, *CA*; ^*3*^ *University of California San Diego Earl Warren College*, *La Jolla*, *CA*; ^*4*^ *Riley Hospital for Children at Indiana University Health*, *Indianapolis*, *IN*; ^*5*^ *Ann & Robert H Lurie Children's Hospital of Chicago*, *Chicago*, *IL*; ^*6*^ *Texas Children's Hospital*, *Houston*, *TX*; ^*7*^ *Cincinnati Children's Hospital Medical Center*, *Cincinnati*, *OH*; ^*8*^ *University of Cincinnati College of Medicine*, *Cincinnati*, *OH*; ^*9*^ *Radiology*, *University of California San Diego*, *La Jolla*, *CA*; ^*10*^ *Emory University*, *Atlanta*, *GA*; ^*11*^ *Department of Epidemiology*, *Johns Hopkins University Bloomberg School of Public Health*, *Baltimore*, *MD*

**Background:** Metabolic dysfunction‐associated steatotic liver disease (MASLD) is an increasingly prevalent disease in children. Despite being pervasive, MASLD is often underdiagnosed, leading to increased risk of related complications and progression to more severe conditions. Vibration‐controlled transient elastography (VCTE) offers a non‐invasive alternative to liver biopsy, using controlled attenuation parameter (CAP) to estimate steatosis and liver stiffness measurement (LSM) for fibrosis. However, pediatric data with histological validation are limited. This study evaluated the ability of CAP and LSM to assess the severity of steatosis and fibrosis in children with MASLD.

**Methods:** This was a prospective, multicenter study with centrally reviewed liver histology as the outcome measure. Children with histologically confirmed MASLD enrolled in the NIDDK NASH CRN Database 3 study underwent VCTE within six months of liver biopsy. Trained operators performed VCTE examinations using a standardized protocol. CAP was evaluated for its correlation with steatosis grades, and LSM for its correlation with fibrosis stages. The diagnostic performance of LSM in distinguishing fibrosis stages was analyzed using histological findings as the reference standard.

**Results:** The mean age of participants (n=92) was 13 ± 3 years, and the median interval between liver biopsy and VCTE was 82 days (IQR 62). Among children with MASLD, CAP values showed substantial overlap across steatosis grades, with median scores of 325 dB/m (grade 1), 310 dB/m (grade 2), and 323 dB/m (grade 3), and no significant correlation with histological steatosis grades (p = 0.422). Median LSM values increased with fibrosis stages (6.0 to 8.8 kPa), but overlap was substantial; only the comparison between no fibrosis (stage 0) and stage 3 fibrosis was significant (p = 0.037). The figure shows the receiver operating curves for each of three separate dichotomous classifications of fibrosis stage. For distinguishing nonfibrotic (F0) from fibrotic (F1–F4) stages, the area under the receiver operating characteristic (AUROC) curve was 0.70 with sensitivity 50%, specificity 88%, positive predictive value (PPV) 95%, and negative predictive value (NPV) 27%, with an overall diagnostic accuracy of 43%. For advanced fibrosis (stages 3–4), the AUROC was 0.67, with sensitivity 67%, specificity 76%, PPV 40%, NPV 90%, and a diagnostic accuracy of 33%.

**Conclusion:** In this prospective, multicenter study, VCTE shows limited accuracy in assessing liver steatosis and fibrosis in children with MASLD. There was no significant relationship between CAP and histological steatosis grade and only a weak association between LSM and fibrosis stage. The diagnostic accuracy of LSM in differentiating fibrosis stages was only modest. These findings suggest that while VCTE is often utilized in adult practice in the evaluation of MASLD, its reliability in pediatric MASLD is limited. Further studies are necessary to establish more accurate and dependable noninvasive techniques for assessing liver disease severity in children with MASLD.


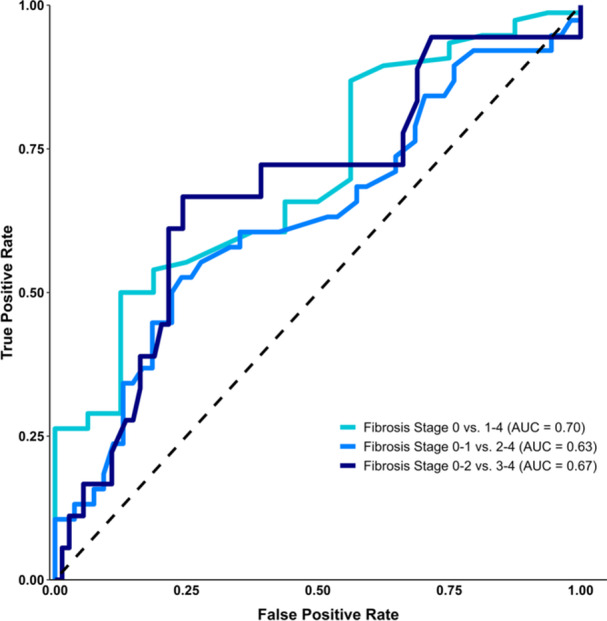


**Receiver Operating Characteristic Curves Liver Stiffness Measurement by Fibrosis Stage**

## 567* DISRUPTING DEVELOPMENTAL PROGRAMMING OF METABOLIC DYSFUNCTION THROUGH GLP‐1 RECEPTOR AGONISM DURING PREGNANCY

*Ananthi Rajamoorthi* ^*1*^, *Rebecca Simmons* ^*2*^

^*1*^ *Department of Gastroenterology, Nutrition and Hepatology*, *The Children's Hospital of Philadelphia*, *Philadelphia*, *PA*; ^*2*^ *Department of Neonatology*, *The Children's Hospital of Philadelphia*, *Philadelphia*, *PA*

**Background:** Maternal metabolic dysfunction during pregnancy increases the risk of gestational diabetes and programs long‐term metabolic complications in offspring, including insulin resistance and obesity. While several interventions are available to treat metabolic dysfunction, therapeutic strategies that prevent or disrupt the intrauterine programming of metabolic dysfunction are lacking. Glucagon‐like peptide‐1 receptor agonists (GLP‐1 RAs) are currently the most effective FDA‐approved medications in promoting weight loss and improving glucose homeostasis, however, their safety and efficacy during pregnancy remain unclear. Here, we investigated the therapeutic potential of semaglutide on maternal and offspring metabolic health before and during gestation.

**Methods:** Six‐week‐old female C57BL/6 mice were fed a chow (n=10) or high‐fat diet (HFD) (n=20) for 10 weeks to induce metabolic dysfunction, then treated daily with vehicle or semaglutide (3 nmol/kg) for 5 days. On day 5, mice were bred, and semaglutide was continued at a lower dose (1 nmol/kg) throughout gestation. Maternal food intake and body weight were monitored during pregnancy. Glucose and insulin tolerance tests were performed at embryonic days (E)14.5 and E16.5. Body composition was assessed by MRI at sacrifice on E17.5. Pregnancy and fetal outcomes—including conception rate, fetal viability, and fetal‐to‐placental weight ratio—were evaluated. Tissue markers of lipid metabolism, insulin signaling, inflammation, and oxidative stress in maternal liver, adipose tissue, placenta, and fetal liver were analyzed via histology, qPCR, and western blotting.

**Results:** Semaglutide treatment reduced food intake and body weight in HFD‐fed female mice prior to pregnancy. During gestation, GLP‐1 RA treatment at a lower dose improved glycemic control and insulin sensitivity in both chow‐ and HFD‐fed mice. While appropriate late gestational weight gain was preserved, HFD‐fed mice treated with semaglutide gained significantly less weight than vehicle‐treated controls during pregnancy. Body composition analysis revealed a significant reduction in fat mass without changes in lean mass in HFD‐fed mice treated with semaglutide. Semaglutide treatment did not affect rate of pregnancy or fetal viability in either dietary group. Notably, semaglutide significantly increased fetal‐to‐placental weight ratio—a marker of placental efficiency—in chow‐fed mice, with a similar trend observed in HFD‐fed mice.

**Conclusions:** GLP‐1 RA treatment reduced adiposity while preserving lean mass in female mice fed a high‐fat diet, and improved glycemic control as well as hepatic and peripheral insulin sensitivity during pregnancy. Importantly, lower dosing during pregnancy supported appropriate gestational weight gain and did not impair fetal development or placental function. In fact, our data suggest that GLP‐1 receptor agonism may improve placental efficiency and offspring outcomes. Overall, this study highlights GLP‐1 receptor agonism as a promising strategy to improve maternal metabolic health and potentially prevent intergenerational transmission of metabolic disease.


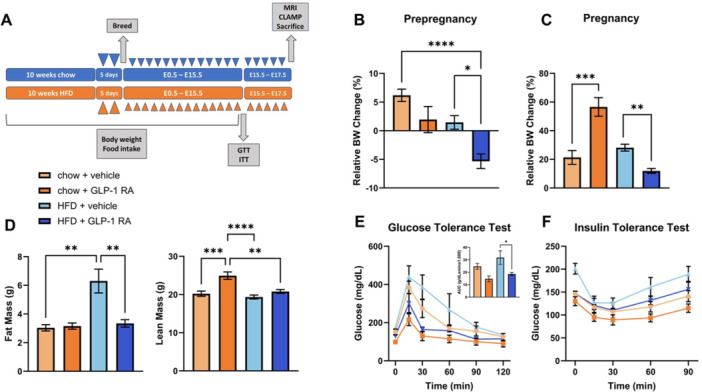


Figure 1: Maternal metabolic outcomes. (A) Experimental design, large and small arrowheads indicate 3 nmol/kg and 1 nmol/kg daily semaglutide treatment, respectively. (B) Relative body weight (BW) change during initial 5‐day treatment at 3 nmol/kg (prepregnancy). (C) Relative BW change during pregnancy. (D) Body composition. (E) Glucose and (F) insulin tolerance tests. Data are shown as means ± s.e.m. (*n* = 5‐8). *p ≤ 0.05, **p ≤ 0.01, ***p ≤ 0.001, ****p ≤ 0.0001.


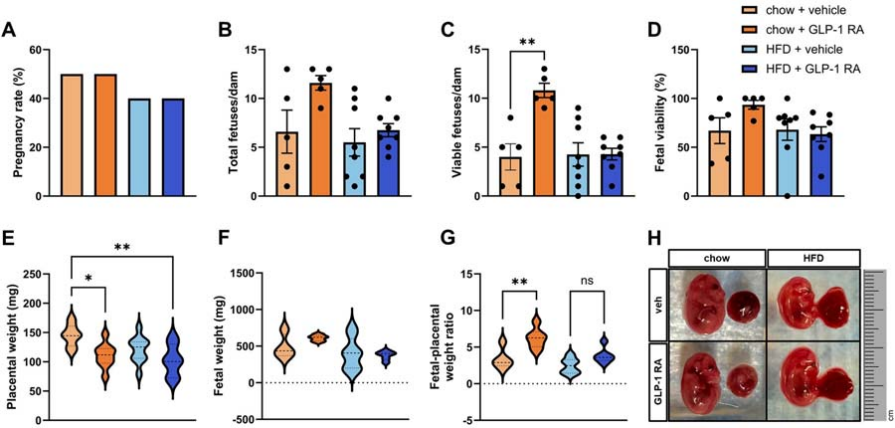


Figure 2: Pregnancy and fetal outcomes. (A) Pregnancy rate. (B) Litter size. (C) Viable fetuses per dam. (D) Fetal viability (%). (E) Placental weight. (F) Fetal weight. (G) Fetal‐to‐placental weight ratio. (H) Representative images of fetus with placenta at embryonic day 17.5.

## 568 TRENDS IN GLP‐1 ANALOGUE PRESCRIPTIONS IN THE PEDIATRIC POPULATION: A DESCRIPTIVE ANALYSIS FROM 2021 TO 2024

*Melissa Ramirez Escobar*, *Huma Naser*, *Alaaelden Hassan*, *Thomas Wallach*

*Pediatric Gastroenterology*, *SUNY Downstate Health Sciences University*, *Brooklyn*, *NY*

**Introduction:** Pediatric use of Glucagon‐like peptide‐1 (GLP‐1) analogues for weight management and metabolic disorders has increased, yet comprehensive data on demographic and diagnostic prescribing practices remain limited. The 2023 American Academy of Pediatrics (AAP) clinical practice guidelines recommend GLP‐1 analogues for children and adolescents with class II and III obesity, while the 2021 North American Society for Pediatric Gastroenterology, Hepatology and Nutrition (NASPGHAN) guidelines support their use in cases of severe obesity and obesity‐related comorbidities such as non‐alcoholic fatty liver disease (NAFLD), type 2 diabetes mellitus (T2DM), and hypertension. This study aims to describe trends of GLP‐1 analogue prescribing patterns in pediatric patients from 2021 to 2024 using data from a Global Collaborative Research Network.

**Methods:** We conducted a retrospective cohort study using the Global Collaborative TriNetX Network. Pediatric patients aged 0–18 years who were prescribed GLP‐1 receptor analogues between January 1, 2021, and December 31, 2024, were identified. Data was stratified by age, sex, race, and ethnicity. Baseline demographic and clinical characteristics were described. Outcomes of interest were identified including diagnosis of overweight and obesity, Type 2 Diabetes Mellitus (T2DM), weight loss, and metabolic syndrome and other insulin resistance.

**Results:** The study cohort included 14,962 pediatric patients aged 0–18 years, with a mean age of 14 ± 4 years. Females represented 54.5% of the population, males 45.2%, and 0.3% were of unknown sex. Ethnicity data showed that 40.5% were non‐Hispanic/Latino, 15.7% Hispanic/Latino, and 43.7% unknown. In terms of race, 48.1% identified as White, 25.9% as Black or African American, 8.6% as Other, 1.5% as Asian, 0.7% as American Indian/Alaska Native, 0.3% as Native Hawaiian or Pacific Islander, and 14.9% were of unknown race.

Among patients with available anthropometric data, 45% had a recorded mean body mass index (BMI) of 37.9 ± 9.4 kg/m^2^. BMI percentiles indicated that 47% of the cohort fell between the 95th and 120th percentiles, 2% between the 120th and 140th percentiles, and 3% exceeded the 140th percentile.

In terms of prescription indication, 76% had non‐specific symptoms, signs, or clinical/laboratory findings not otherwise classified. Symptoms related to food and fluid intake were reported in 31%, while 25% had a documented diagnosis of abnormal weight gain.

Abnormal blood glucose levels were identified in 42% of the cohort. Of these, 31% had a diagnosis of diabetes mellitus, including 29% with T2DM and 17% with prediabetes.

Skin and subcutaneous tissue disorders were noted in 49% of patients, with acanthosis nigricans comprising 28% of this subgroup. Cardiorespiratory symptoms were also reported in 49%, including abnormal breathing patterns (28%), cough (22%), snoring (21%), and dyspnea (10%).

Metabolic disorders were documented in 40% of patients, including hyperlipidemia (27%) and metabolic syndrome or insulin resistance (15%). Additionally, 40% of the cohort had mental, behavioral, or neurodevelopmental disorders.

**Conclusions:** There is an increasing trend in the use of GLP‐1 analogues among pediatric patients. While current AAP and NASPGHAN guidelines support their use for obesity and type 2 diabetes in adolescents aged 12 years and older, real‐world prescribing patterns suggest broader utilization beyond these recommended indications. Our data fills this knowledge gap. Further research is needed to evaluate the efficacy, safety, and clinical outcomes of GLP‐1 analogue use beyond approved indications in pediatric population.

## 569 HEALTHCARE PROFESSIONAL PERSPECTIVES ON BARRIERS TO ORAL FEEDING IN THE NICU: A MIXED METHOD STUDY

*Willow Schanz* ^*1*^, *Loulwa Soweid* ^*2*^, *DeShauna Jones* ^*2*^, *Aunum Akhter* ^*3*^, *Georgette Richardson* ^*4*^, *William Story* ^*5*^, *Tarah Colaizy* ^*3*^, *Aamer Imdad* ^*6*^

^*1*^ *The University of Iowa Roy J and Lucille A Carver College of Medicine*, *Iowa City*, *IA*; ^*2*^ *Institute for Clinical and Translational Science*, *University of Iowa Hospitals and Clinics*, *Iowa City*, *IA*; ^*3*^ *Division of Neonatology*, *The University of Iowa Stead Family Children's Hospital*, *Iowa City*, *IA*; ^*4*^ *Division of Pediatric Psychology*, *The University of Iowa Stead Family Children's Hospital*, *Iowa City*, *IA*; ^*5*^ *The University of Iowa College of Public Health*, *Iowa City*, *IA*; ^*6*^ *Division of Gastroenterology, Hepatology, and Nutrition*, *The University of Iowa Stead Family Children's Hospital*, *Iowa City*, *IA*

**Background:** Oral feeding difficulties in preterm infants remain a significant contributor to prolonged hospitalization, parent stress, delayed discharge, and increased cost. Although structured oral feeding protocols aim to standardize care, healthcare professionals (HCPs) often face challenges balancing guideline adherence with individualized, cue‐based care. This study explored NICU HCPs’ perceptions of a structured oral feeding protocol for preterm infants to identify barriers to oral feeding, areas of inconsistency, and opportunities for improved implementation.

**Methods:** This was a sequential mixed method study conducted in two phases. In Phase I, a cross‐sectional electronic survey was distributed to all NICU healthcare professionals, including nurses, nurse practitioners, neonatologists, speech‐language pathologists, dietitians, and lactation consultants. The survey assessed knowledge, agreement, comfort, and satisfaction with various components of the unit's oral feeding protocol. It included multiple‐choice, Likert‐scale, and open‐ended items focused on factors influencing oral feeding decisions.

In Phase II, we conducted semi‐structured interviews with a purposive sample of 23 HCPs representing a range of disciplines and years of experience. Interview questions explored participants’ perceptions of the protocol's utility, barriers to implementation, alignment with cue‐based care, and how feeding decisions are made in practice. Interviews were recorded, transcribed verbatim, coded and analyzed using thematic analysis. Three coders independently reviewed transcripts and met regularly to refine codes and resolve discrepancies, ensuring rigor and trustworthiness.

Quantitative data from the survey was analyzed descriptively. Qualitative data were used to complement and expand upon survey findings, highlighting the complex factors shaping how HCPs interpret and apply feeding guidelines at the bedside.

**Figure 1** ‐ Distribution of Healthcare Roles in Survey Participants

**Results:** 83 survey responses were collected with diverse healthcare role representation (Figure 1). While most respondents reported high understanding of the protocol (94%), only 61% agreed with the current initiation approach, and just 34.9% supported initiating feeds by 34 weeks postmenstrual age (PMA). Feeding infants on CPAP drew mixed responses: 57.8% were comfortable, 20.5% were conditionally comfortable, and 19.3% were not comfortable. Major concerns included risk of aspiration (56.3%) and disruption of the suck‐swallow‐breathe reflex (87.5%). Key decision‐making factors included infant cues (96.3%), respiratory support (95.1%), and cardiorespiratory stability (95.1%). Qualitative findings were rich, with four of the central themes described below:

1. Protocol Confusion and Inconsistencies: HCPs reported confusion between the initiation and advancement scales, leading to delays and inconsistent assessments.

2. Tension Between Standardization and Clinical Judgment: HCPs valued protocol structure but noted disagreement on definitions of “medical readiness” and gestational benchmarks. Some advocated for earlier, cue‐based feeding; others referenced safety concerns.

3. Limited Family Engagement: While caregiver involvement was viewed as critical, scheduling conflicts and lack of parent education limited consistent participation.

4. Structural Barriers: Staffing shortages, time constraints, and interprofessional communication challenges hindered effective feeding practices and team coordination.

**Conclusions:** Despite protocol awareness, NICU HCPs vary in how they interpret and implement feeding guidelines—particularly regarding feeding while on CPAP and initiation before 34 weeks postmenstrual age. These findings suggest a need for enhanced interdisciplinary collaboration, clearer staff protocol education, and more flexible, cue‐based strategies. Increasing family involvement and addressing workflow limitations may also improve consistency and outcomes for preterm infant oral feeding in the NICU.


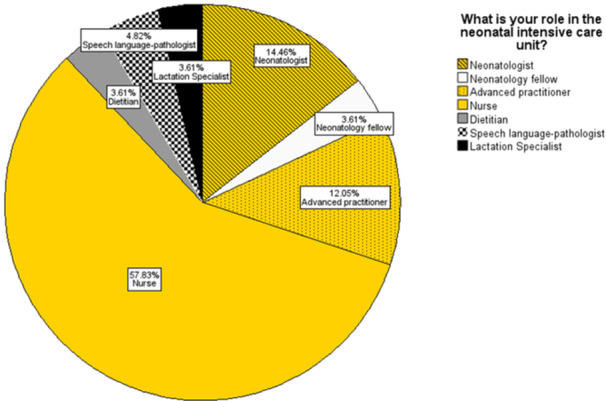


## 570 MANAGEMENT OF SMALL INTESTINAL BACTERIAL OVERGROWTH (SIBO) IN PEDIATRIC SHORT BOWEL SYNDROME (SBS) ‐ PERSPECTIVES OF AN INTERNATIONAL DELPHI PANEL

*Wenjing Zong* ^*2*^, *Zev Minkoff* ^*3*^, *Clarivet Torres* ^*4*^, *Vahe Badalyan* ^*4*^, *David Galloway* ^*5*^, *Valeria Cohran* ^*6*^, *Reena Alame* ^*2*^, *Nikhil Pai* ^*1*^

^*1*^ *Gastroenterology/Hepatology/Nutrition*, *Children's Hospital of Philadelphia*, *Philadelphia*, *PA*; ^*2*^ *Pediatric GI/Hepatology/Nutrition*, *The University of Texas Southwestern Medical Center*, *Dallas*, *TX*; ^*3*^ *Pediatric Gastroenterology, Hepatology and Nutrition*, *Valley Children's Hospital*, *Madera*, *CA*; ^*4*^ *Pediatric Gastroenterology, Hepatology and Nutrition*, *Children's National Hospital*, *Washington*,; ^*5*^ *Pediatric Gastroenterology/Hepatology/Nutrition*, *Children's of Alabama*, *Birmingham*, *AL*; ^*6*^ *Pediatric Gastroenterology/Hepatology/Nutrition*, *Ann and Robert H Lurie Children's Hospital of Chicago Foundation*, *Chicago*, *IL*

**Introduction:** Small intestinal bacterial overgrowth (SIBO) is a challenging complication in pediatric short bowel syndrome (SBS), contributing to malabsorption and inflammation. However, its management remains highly variable due to the lack of robust, evidence‐based guidelines. Differences in diagnostic practices, access to medications, and availability of diagnostic tools further complicate standardized care. Additionally, emerging evidence supports the long‐term impacts of antibiotic‐induced dysbiosis. This study aimed to gather expert consensus on management approaches for SIBO in pediatric SBS, and to identify areas of uncertainty in current practice.

**Method:** An international panel of pediatric gastroenterologists, surgeons, and nutrition physicians with expertise in intestinal rehabilitation was assembled using a modified Delphi approach. Experts were invited based on criteria including >5 years of relevant clinical experience and active management of >15 pediatric patients with SBS. The consensus process involved 3 iterative e‐Delphi survey rounds and 2 in‐person focus group discussions. Consensus was defined as ≥75% agreement on a given statement a priori. Statements were revised throughout each round based on panel feedback and qualitative thematic analysis (Figure 1). Pharmacist, infectious disease and Delphi methodology experts also contributed to the final recommendations.

**Result:** Over 70% of invited experts participated in each round of Delphi (n ≈ 35). Consensus was reached on statements related to (Table 1):

**Diagnostic criteria** (e.g., symptoms, signs, high risk scenarios, etc);

**Treatment approaches** (e.g., choice and duration of antibiotics, rotating antibiotics, treatment response monitoring, etc);

Experts acknowledged that current practices are challenged by the limited interpretability of both clinical symptoms and diagnostic tests such as breath testing and jejunal aspirates. The panel recognized the diagnostic value of clinical symptomatology despite its subjectivity, and concurred that a standardized, symptom‐based treatment trial may be appropriate in select cases. While some panelists raised concern about the limitation of certain diagnostic tests, including breath testing and jejunal aspirates, there was no consensus on this issue. There was consensus on the importance of regular reassessment to monitor treatment response and recurrence.

**Conclusion:** Using a modified Delphi approach, we achieved expert consensus on several key components of SIBO management in pediatric SBS. Based on panel responses, we present the first pediatric‐specific diagnostic algorithm. These findings highlight current practice patterns, shared challenges, and highlight areas in need of further research. The consensus statements may serve as a foundational framework for future guideline development, validation of the proposed diagnostic algorithm, and establishment of ongoing clinical trials, aiming to harmonize care and improve outcomes in this vulnerable population.


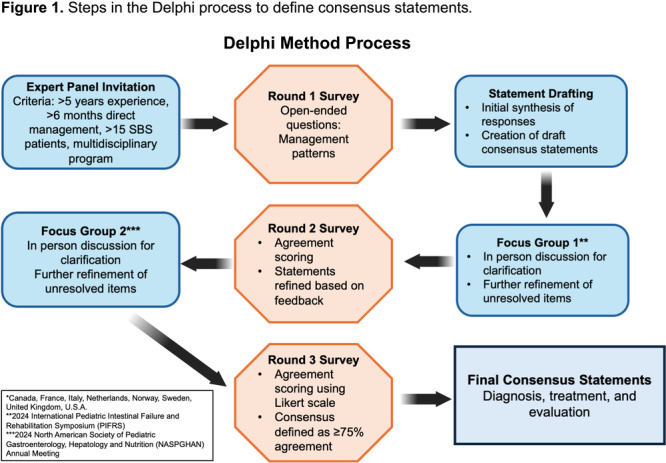


Figure 1. Steps in the Delphi process to define consensus statements.


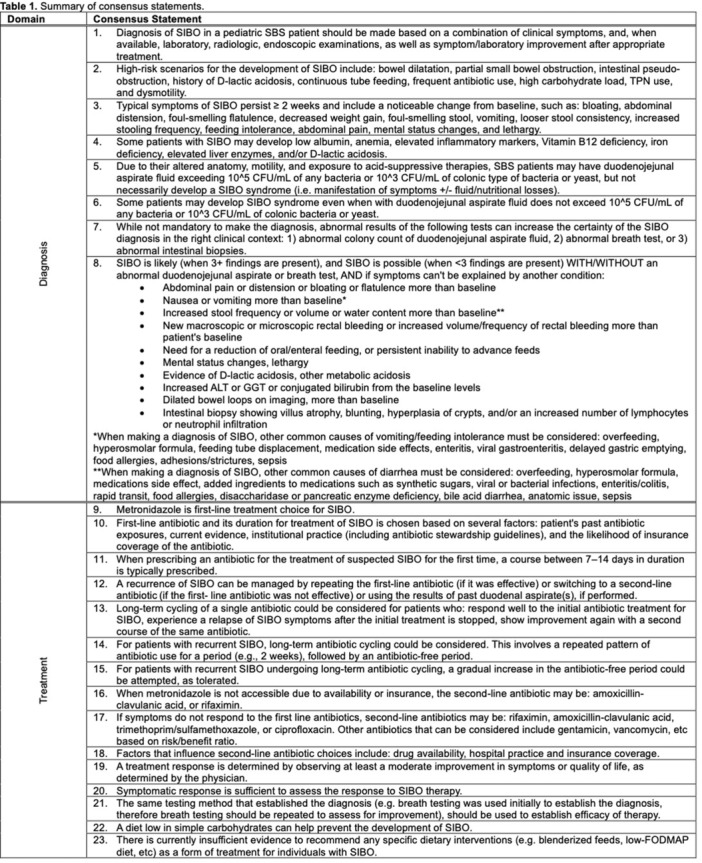


Table 1. Summary of consensus statements.

## 572 IMPORTANCE OF OPTIMIZING NUTRITIONAL STATUS OF A PEDIATRIC PATIENT WITH DRUG INDUCED PANCREATITIS

*Janvi Bhavsar* ^*1*^, *Vivian Tang* ^*2*^, *Raj Shah* ^*3*^

^*1*^ *Pediatrics*, *Maimonides Medical Center*, *Brooklyn*, *NY*; ^*2*^ *Pediatric Gastroenterology*, *Maimonides Medical Center*, *New York*, *NY*; ^*3*^ *The University of Kansas Medical Center*, *Wichita*, *KS*

**Introduction:** Acute pancreatitis (AP) in pediatric patients is a significant health concern, with special concern for Drug Induced Pancreatitis (DIP) having poor outcomes. Protein‐energy malnutrition (PEM) is a well‐documented predictor of mortality in AP. Children with PEM have higher rates of severe complications, including respiratory failure, mechanical ventilation, acute kidney injury, sepsis, and pseudocyst formation, which prolong hospital stays and increase healthcare costs. Factors associated with poor outcomes in DIP are not well documented. This study aims to highlight the critical role of nutritional status in patients with DIP and the potential benefits of optimizing nutrition to reduce mortality.

**Methods:** We used logistic regression and STATA BE 18.0 software to conduct an analysis using the National Inpatient Sample (NIS) 2021 to examine the demographic distribution and prevalence of PEM in patients with DIP and patients with Non‐Drug Induced Pancreatitis (NDIP). We only included patients between ages 1 month and 18 years. We used ICD‐10 codes to isolate patients who were hospitalized for AP and then divided them into two cohorts of DIP and NDIP.

**Results:** There were 8290 pediatric patients hospitalized for acute pancreatitis in 2021. Out of these patients, 215 (2.6%) were hospitalized for DIP and 8075 (97.4%) for NDIP. Mean age of patients with DIP was 10 years, and of patients with NDIP was 12 years, a statistically significant association. There was no racial or socioeconomic disparity on analysis of the demographic trends of the two populations.

Prevalence of PEM in patients with DIP was 23% vs 6% in patients with NDIP and it was a statistically significant association. Obesity had a prevalence of 9% in DIP but 22% in NDIP.

Mortality rate in DIP was 9% and 2% in NDIP. The length of stay was 16 days in DIP and 8 days in NDIP, which was a statistically significant association (p=0.03).

Mechanical ventilation was 14% in DIP and 6% in NDIP. (p=0.02)

On multivariate logistic regression patients with PEM had aOR of 3.51 [CI 1.49‐8.25] for patients with DIP, and mortality aOR 5.02 [CI 2.09‐12.03]. Age was a protective factor aOR 0.93 [CI 0.88‐0.99] for DIP.

**Conclusion:** Through these results we conclude that patients with DIP have a higher prevalence of PEM and mortality than patients with NDIP, even when adjusted for variables such as demographic data, median household income and insurance type. This motivates us to enhance the nutrition status of patients being prescribed drugs that commonly cause DIP, which may be an important preventative measure against mortality caused by it.

## 575 DEGREE OF COMMON BILE DUCT DILATATION MAY FURTHER PREDICT CHOLEDOCHOLITHIASIS IN INTERMEDIATE RISK PEDIATRIC PATIENTS WITH SYMPTOMATIC CHOLELITHIASIS

*Caroline Chinchilla Putzeys* ^*1*^, *Owais Salahudeen* ^*2*^, *Estefania Lopez* ^*2*^, *Grace Yoshiba* ^*1*^, *Ted Swing* ^*2*^, *Joshua Carroll* ^*1,2*^, *Mark McOmber* ^*1,2*^, *Shahan Fernando* ^*1,2*^

^*1*^ *Gastroenterology*, *Phoenix Children's Hospital*, *Phoenix*, *AZ*; ^*2*^ *The University of Arizona College of Medicine Phoenix*, *Phoenix*, *AZ*

**INTRODUCTION:** Choledocholithiasis (CDL) in the pediatric population with symptomatic cholelithiasis is relatively uncommon (2%‐6%), yet urgent condition, that is rising in prevalence. The risk stratification of CDL based on established adult risk factors categorizes patients into three broad categories that guide management: high, intermediate, and low risk. Endoscopic retrograde cholangiopancreatography (ERCP) is the mainstay of CDL treatment in high‐risk patients. While ERCP is relatively safe, it is not without significant risks. In our study, we focus on the risk factors and outcomes of intermediate risk pediatric patients, which are an area of uncertainty with regards to clinical and endoscopic management.

**OBJECTIVE:** Review predictors of choledocholithiasis in pediatric patients evaluated for symptomatic cholelithiasis. Specifically, we evaluate patient outcomes in those stratified as “intermediate risk” who did and did not undergo an ERCP to further explore the utility of current predictors of choledocholithiasis.

**STUDY DESIGN:** This is a retrospective single center study of pediatric patients with suspected CDL between January 2021 to December 2024. Inclusion criteria were all pediatric patients (<19 years old) with symptomatic cholelithiasis who had cholecystectomy during admission at Phoenix Children's Hospital. Exclusion criteria include patients who had ERCP for other than suspected choledocholithiasis. Previous ERCP intervention and patients with hemolytic disease.

**RESULTS:** In total, 443 charts were reviewed, 46 were excluded, and 397 pediatric patients were included in the study. Of these, 196 (49%) patients were classified as intermediate risk using the 2010 ASGE guidelines, compared to 232 (58%) following the 2019 ASGE guidelines (p value = 0.124). The discrepancy in these numbers reflects the broader capture of intermediate risk patients under the updated guidelines. Among the 232 intermediate‐risk patients, 76 (33%) underwent ERCP, with 51 (67%) having a stone removed. Therefore, 25 patients had an ERCP that was not needed and conveyed unnecessary risks.

Across all risk categories, a CBD diameter greater than 6 mm was predictive of stone presence in 76.6% of patients, with an odds ratio (OR) of 2.6 (p<0.05). Specifically, within the intermediate‐risk cohort, a CBD diameter greater than 8 mm had a stronger correlation with stone presence in 85.2% of patients, yielding an odds ratio of 4.3 (p<0.05).

Direct bilirubin (DB) and GGT were additional variables analyzed in this study, particularly in intermediate‐risk patients. Patients with a DB greater than or equal to 2 mg/dL were found to have a stone 84% of the time (OR 2.69, p<0.05), consistent with findings from previous studies. Unique to our study, results indicated that 80% of patients with a GGT greater than or equal to 50 U/L also predicted the presence of a stone (OR 2.28, p<0.05).

Multivariate analysis identified independent predictors using the three variables: CBD > 8 mm, DB ≥ 2 mg/dL, and GGT ≥50 U/L. CBD was the strongest predictor (OR 3.22, p<0.05), followed by DB (OR 2.33, p<0.05) and GGT (OR 2.20, p<0.05).

Finally, the predictive value of stone presence was assessed based on having 0, 1, 2, or all 3 of the statistically significant CDL predictors. In patients with a combination of two risk factors, 84.9% were found to have a stone, with a sensitivity of 54% (CI 45.2% ‐ 62.7%) and a specificity of 78.3% (CI 65.0% ‐ 88.5%), p<0.001. Patients with only one risk factor had a stone 68.2% of the time, with increased sensitivity to 90.3% (95% CI: 84.3–94.7) but reduced specificity to 32.6% (95% CI: 20.3–46.8), yet still statistically significant (p value < 0.022). The presence of all three risk factors resulted in the highest proportion of stone detection (91.7%) with the greatest specificity of 95.7% (CI: 87.2%‐99.3%), but sensitivity decreased to 17.7% (95% CI: 11.7–25.1). However, this finding was only trending towards statistical significance (p = 0.180), likely due to the small sample size of this subset of patients, as GGT is not routinely ordered during admission at our institution.

**CONCLUSION:** Moderate dilation in CBD, in addition to elevated direct bilirubin and GGT, may further increase the probability of finding intraductal stones on ERCP, especially in patients stratified as intermediate risk based on current adult guidelines. As more pediatric centers are utilizing ERCP in the management of patients with suspected choledocholithiasis, pediatric‐specific guidelines are necessary to mitigate the risks of ERCP and improve the detection of intraductal stones.

## 578 FLUID AND NUTRITION MANAGEMENT AND LENGTH OF STAY IN PEDIATRIC ACUTE PANCREATITIS: A SYSTEMATIC REVIEW AND META‐ANALYSIS

*Parker Giroux* ^*1*^, *Oluwagbemiga Dadematthews* ^*4*^, *Adefunke Dadematthews* ^*3*^, *Megan Bell* ^*5*^, *Kathryn Kaiser* ^*2*^, *Chinenye Dike* ^*1*^

^*1*^ *Division of Pediatric Gastroenterology, Hepatology, and Nutrition*, *The University of Alabama at Birmingham Department of Pediatrics*, *Birmingham*, *AL*; ^*2*^ *The University of Alabama at Birmingham School of Public Health*, *Birmingham*, *AL*; ^*3*^ *Auburn University College of Human Sciences*, *Auburn*, *AL*; ^*4*^ *Louisiana State University School of Kinesiology*, *Baton Rouge*, *LA*; ^*5*^ *The University of Alabama at Birmingham Libraries*, *Birmingham*, *AL*

**Background:** Acute pancreatitis (AP) in the pediatric population is a steadily growing area of investigation thanks to increased awareness by clinicians and scientists. Like adults, children are susceptible to severe disease, complications and poor outcomes, yet data on management strategies that improve clinical outcomes in children are limited. A systematic review is needed to synthesize the available data and better understand the research landscape in pediatric AP management.

**Objective:** The purpose of this systematic review is to evaluate management strategies and clinical characteristics that improve outcomes such as length of stay (LOS), cost of hospitalization (COH), severity, complications, ICU stay, and mortality in children with AP.

**Methods:** Multiple electronic databases were last searched on 10/4/2024 to retrieve studies that investigated the association of LOS and COH (outcome) in pediatric patients with AP (population) with exposures and controls such as IV fluid type (normal saline vs. Lactated Ringer's), IV fluid rate (<1.5x maintenance, 1.5‐2x maintenance, >2x maintenance), timing of enteral nutrition initiation, route of enteral nutrition, and nutritional status (obesity, malnutrition, normal). A pre‐registered protocol was submitted to the PROSPERO registry (CRD42024578304). Two authors independently screened titles and abstracts, reviewed full texts, and extracted data from the studies that met preliminary inclusion criteria. A third author resolved conflicts and provided consensus at each stage. Risk of bias analysis was performed on included randomized controlled trials (RCTs) by two independent reviewers using Cochrane ROB‐2. The Risk Of Bias In Non‐randomized Studies – of Interventions, version 2 (ROBINS‐I V2) tool was used to assess the included non‐randomized studies. Statistical analysis was performed via SPSS v30.0.0.0.

**Results:** We screened 395 studies from 7 electronic databases. Twenty‐six unique studies met criteria: 5 RCTs (4 articles, 1 abstract; Table 1) and 21 observational studies (17 articles, 4 abstracts), comprising a total of 82,409 patients or hospital encounters. Included studies recruited children up to 21 years of age. Female was the most prevalent sex in the majority of articles. Most studies were conducted in the USA. All studies were conducted in the inpatient setting. Preliminary results from full text manuscripts with respect to our exposures of interest were as follows: 4 studies on IV fluid type; 4 studies on IV fluid rate; 13 studies on enteral nutrition timing; 1 study on enteral nutrition route; 7 studies on nutritional status. Primary outcomes of LOS and COH were reported by 26 and 7 studies, respectively. The number of articles reporting secondary outcomes were: severity (8), ICU stay (6), complications (11), and mortality (7). Meta‐analysis of RCT data on IV fluid type and timing of nutrition (<24 h vs >24 h) did not show a significant difference between groups. Eight out of twenty‐one observational studies were included in a preliminary meta‐analysis. For nutritional status, underweight BMI was associated with a statistically significant longer LOS when compared to normal BMI (p = 0.04). Further, early feeding (within 48 h) significantly reduces LOS compared to feeding beyond 48 h (p = 0.01; Fig. 2). Among the 4 RCTs with complete reports, risk of bias was highest in the domain for measurement of the outcome due to lack of blinding of caregivers.

**Conclusions:** Pediatric data on AP management strategies that affect clinical outcomes are limited to mostly observational studies. Our preliminary meta‐analysis supports early feeding and normal nutritional status to significantly reduce LOS in children with AP. Further data may reveal areas needing higher quality evidence to guide future investigation, change clinical practice and improve outcomes in children.


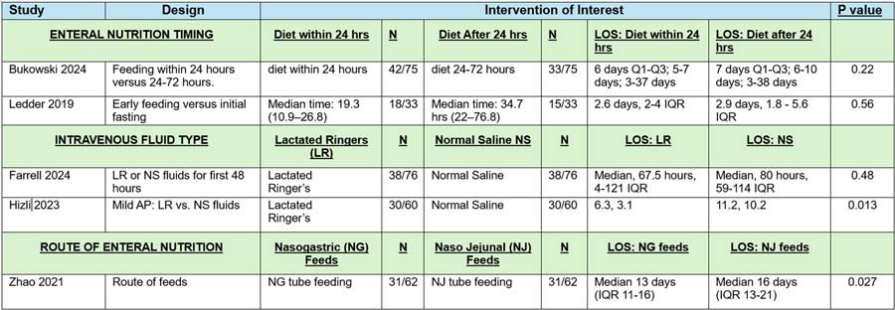


Table 1: RCTs that met inclusion criteria


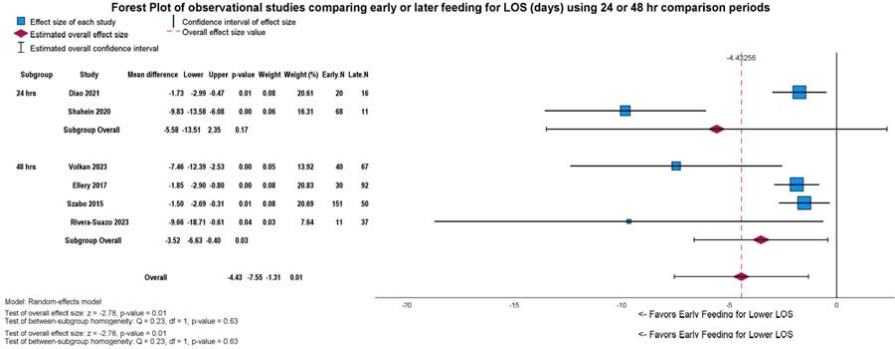


Figure 1: Preliminary meta‐analysis of observational studies meeting inclusion criteria that reported data on LOS and timing of enteral nutrition

## 579 USE OF QUANTITATIVE MRI MARKERS TO IDENTIFY EARLY CHANGES POST‐ACUTE PANCREATITIS AND PREDICT DIABETES MELLITUS RISK: A PROSPECTIVE STUDY

*Lucia Gonzalez‐Llanos* ^*1*^, *Emrah Gecili* ^*3*^, *Jonathan Dudley* ^*2*^, *Andrew T. Trout* ^*2*^, *Maisam Abu‐El‐Haija* ^*1*^

^*1*^ *Gastroenterology, Hepatology, and Nutrition*, *Cincinnati Children's Hospital Medical Center*, *Cincinnati*, *OH*; ^*2*^ *Radiology*, *Cincinnati Children's Hospital Medical Center*, *Cincinnati*, *OH*; ^*3*^ *Biostatistics and Epidemiology*, *Cincinnati Children's Hospital Medical Center*, *Cincinnati*, *OH*

**Background:** While most children recover from a single episode of acute pancreatitis (AP), some develop acute recurrent pancreatitis (ARP), chronic pancreatitis (CP), or metabolic complications such as diabetes mellitus (DM) or prediabetes (Pre‐DM). Quantitative MRI markers, including segmented pancreatic volumes, T1 mapping, and proton density fat fraction (PDFF), may offer noninvasive tools to assess structural and functional pancreatic changes. This study evaluated MRI markers among children with AP, ARP, and DM/Pre‐DM to identify phenotypic differences and better understand disease progression.

**Methods:** This IRB‐approved, single‐institution prospective study included patients from three diagnostic groups: AP (single episode), ARP (≥2 episodes), or DM/Pre‐DM (HbA1c ≥5.7 or fasting glucose >100 per ADA guidelines, after ≥1 AP episode). Patient characteristics were collected via EMR, and comorbid conditions (e.g., seizure disorder, Schwachman‐Diamond syndrome, IBD, hypothyroidism, asthma, cystic fibrosis, hypertension, obesity, cyclic vomiting syndrome, vesicoureteral reflux, and central hypotonia) were documented. Research MRI was performed ≥4 weeks post‐AP; laboratory data were collected at imaging. Pancreas volume, T1 relaxation time (MOLLI), PDFF, pancreas:spleen T1 signal intensity ratio (T1SIR), and liver PDFF were measured on MRI, blinded to group. Group comparisons were conducted using ANOVA or Kruskal–Wallis tests with post hoc Dunn tests and Holm adjustment. Chi‐square or Fisher's exact tests were used for categorical variables. Pancreas volume was adjusted for body surface area (BSA) using analysis of covariance.

**Results:** The study sample included 60 participants (AP: 21, ARP: 23, DM/Pre‐DM: 16). Age, sex, and time from first AP to MRI did not differ significantly across groups (p > 0.05). BMI percentile differed significantly (p = 0.016) among groups: AP=80.5 (60.2, 93.9), ARP=82.3 (48.1, 95.1), DM/Pre‐DM=95.9 (87.2, 98.7); post hoc tests showed the DM/Pre‐DM group had higher values than both AP and ARP (all p < 0.05), suggesting an association between metabolic risk and endocrine complications.

Comorbidity prevalence varied significantly (p = 0.027), highest in DM/Pre‐DM (56%), followed by AP (48%) and ARP (17%).

BSA‐adjusted pancreatic volume and T1SIR did not differ significantly between groups. T1 relaxation time was lower in AP than ARP (p=0.015).

Pancreatic PDFF was higher in ARP (3.7) than AP (1.7) (p=0.021), indicating fat accumulation with recurrent injury. Liver PDFF was significantly higher in the DM/Pre‐DM group (9.9) compared to ARP (2.7) (p=0.024).

A PCA was conducted to reduce redundancy among quantitative MRI biomarkers and explore underlying structure but did not reveal clear, well‐separated clusters to strongly discriminate the clinical groups.

**Conclusions:** Children with AP, ARP, and DM/Pre‐DM show distinct clinical and imaging profiles. Notably, patients with AP had lower T1 relaxation times and reduced PDFF compared to ARP, suggesting tissue remodeling and fat deposition following repeated pancreatic injury. In contrast, children with DM/Pre‐DM displayed features of metabolic dysfunction, including increased liver fat content and elevated BMI, although pancreatic changes were not observed. These findings support using advanced MRI as a noninvasive tool to monitor pancreatic health, assess risk for endocrine dysfunction, and guide early intervention, though further studies are needed to validate these markers for risk stratification in pediatric pancreatitis.


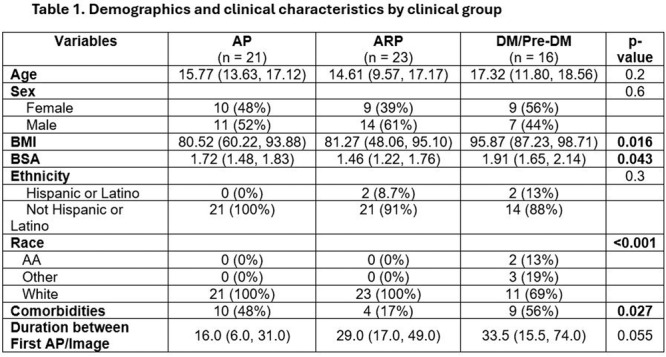



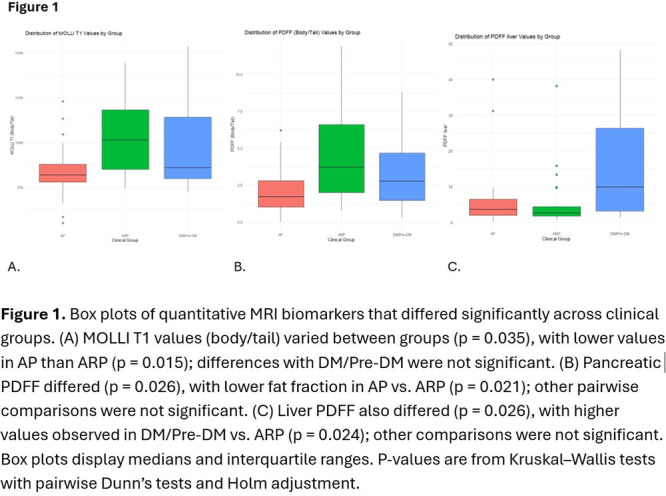


## 585 METABOLOMIC PROFILING IN GROWTH IMPAIRED PEDIATRIC LIVER TRANSPLANT RECIPIENTS

*Luis Lopez* ^*4*^, *Bridget Whitehead* ^*1*^, *Saeed Mohammad* ^*2*^, *Estella Alonso* ^*1*^, *Divakar Mithal* ^*3*^

^*1*^ *Pediatric Gastroenterology, Hepatology and Nutrition*, *Northwestern University Feinberg School of Medicine*, *Chicago*, *IL*; ^*2*^ *Pediatric Gastroenterology, Hepatology and Nutrition*, *Vanderbilt University Medical Center*, *Nashville*, *TN*; ^*3*^ *Pediatric Neurology*, *Northwestern University Feinberg School of Medicine*, *Chicago*, *IL*; ^*4*^ *Northwestern University Feinberg School of Medicine*, *Chicago*, *IL*

**Introduction:** Children with chronic liver disease are at risk for sarcopenia and growth failure. Many, but not all children have improvement and catch‐up growth following liver transplantation. The drivers of growth impairment (GI) in children after liver transplant (LT) are not fully understood and warrant further investigation.

**Methods:** We enrolled children ages 5‐17 years old in the outpatient setting who were at least 6 months post LT from 10/2016‐5/2018. Data collection included demographics, graft function, medications and nutritional assessment (length, body mass index (BMI), mid upper arm circumference (MUAC)). Body composition was measured using air displacement plethysmography in the BodPod from which fat free mass index (FFMI) was calculated. From serum samples we measured cytokines (TNF‐α, IL‐1β, IL‐6), IGF‐1 using liquid chromatography/mass spectrometry and performed untargeted metabolomic profiling of 306 metabolites. Patients were categorized by presence or absence of GI by growth measure (length, BMI or MUAC) Z scores below the 10%ile or <‐1.28. Comparison of clinical data, cytokines and IGF‐1 Z scores by GI status were compared by chi‐square and Mann‐Whitney U tests. We performed unsupervised hierarchical analysis followed by comparison of individual metabolite levels by GI status. Significant differences between metabolite levels were defined by a fold change (FC) >2 and p value of <0.1.

**Results:** A total of 40 patients with a median age 10 years (7,14) old were enrolled with a median time from transplant 71 months (39,109). The most common diagnosis was biliary atresia (48%) and median ALT was 21 (16,36). The majority (88%) were taking a calcineurin inhibitor (CNI) and only 15% of patients were on corticosteroids. GI was present in 27.5% (11/40) of patients and median length, BMI and MUAC Z scores of the cohort were ‐0.37, 0.30, and ‐0.21 respectively. There was not a significant relationship between GI and immunosuppression medications (CNI, steroids, or >1 immunosuppressant), ALT, IGF‐1 Z score, IL‐1β or IL‐6 levels. Patients with GI had higher TNF‐α levels although this did not meet the threshold for statistical significance: 23.4 pg/mL (15.3, 50.0) vs 16.5 pg/mL (6.8, 22.9), p=0.057. We found differences in FFMI which trended toward significance with GI patients having lower FFMI: 13.3 (12.6,15.0) vs 14.1 (13.4, 15.6) *p = *0.148. Unsupervised clustering of serum metabolites demonstrated 2 groups with the majority of GI patients in one cluster (Figure 1). Comparison of individual metabolite levels between patients with and without GI identified 13 distinct metabolites that were significantly different including spermidine (fold change [FC] = 2.52, *p* = .001), N1‐acetylespermine (FC = 4.57, *p = *0.002) and malic acid (FC=4.74, p=0.003).

**Conclusions:** We found GI was common in children post LT, but this did not have a relationship to graft function or immunosuppression medications. Children with GI had evidence of increased systemic inflammation and decreased fat free mass although these differences did not reach statistical significance. Using metabolomics, we found children with GI have specific metabolites that are abnormally upregulated. Although they do not all fit within a singular metabolic pathway, the polyamine synthesis pathway was highly represented. Previous studies in humans and animal models have suggested that increased levels of spermidine may have a protective effect against obesity. Spermidine is thought to act through activation of adipose tissue thermogenesis. Upregulation of this polyamine pathway in pediatric LT recipients may reflect a maladaptive response with a negative impact on growth. Further investigation into the causal effects of spermidine in growth patterns of pediatric LT recipients is needed.


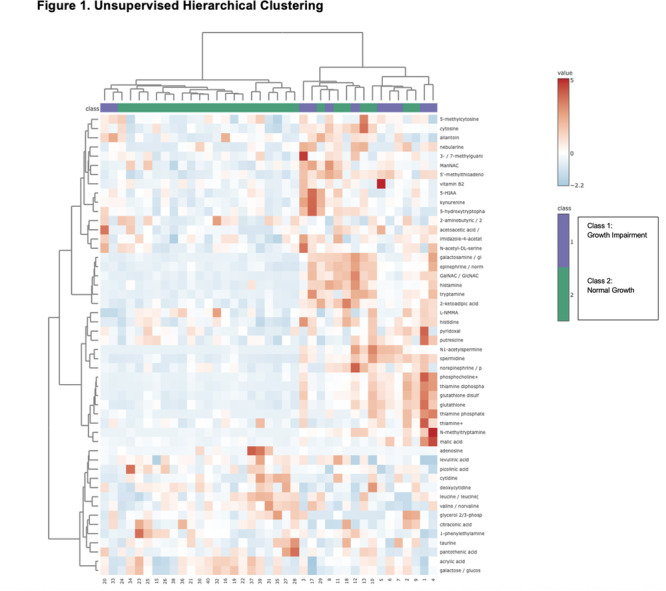


## 586 CHARACTERISTICS AND OUTCOMES OF FONTAN ASSOCIATED LIVER DISEASE IN CHILDREN FROM A MULTICENTER ‐ INITIAL ANALYSIS OF 8 CENTERS IN USA

*Chaowapong Jarasvaraparn* ^*2*^, *Henry Lin* ^*4*^, *Sindhu Pandurangi* ^*3*^, *Kim Liss* ^*1*^, *Steven Lobritto* ^*5*^, *Adebowale Adeyemi* ^*6*^, *Sara Kathryn Smith* ^*8*^, *Amal Aqul* ^*3*^, *R. Mark Payne* ^*2*^, *Katryn N. Furuya* ^*7*^, *Wikrom Karnsakul* ^*8*^, *Jean Molleston* ^*2*^

^*1*^ *Division of Pediatric Gastroenterology, Hepatology and Nutrition, Department of Pediatrics*, *Washington University School of Medicine*, *Saint Louis*, *MO*; ^*2*^ *Riley Hospital for Children at Indiana University Health*, *Indianapolis*, *IN*; ^*3*^ *The University of Texas Southwestern Medical Center*, *Dallas*, *TX*; ^*4*^ *Oregon Health & Science University Doernbecher Children's Hospital*, *Portland*, *OR*; ^*5*^ *Columbia University*, *New York*, *NY*; ^*6*^ *Nemours Children's Hospital Delaware*, *Wilmington*, *DE*; ^*7*^ *University of Wisconsin‐Madison*, *Madison*, *WI*; ^*8*^ *Johns Hopkins University*, *Baltimore*, *MD*

**Background:** Fontan associated liver disease (FALD) is a universal sequela of the Fontan procedure. In this study, we aimed to 1) characterize severity of pediatric FALD, 2) evaluate non‐invasive markers of liver fibrosis, and 3) describe long‐term outcome of FALD in children.

**Methods:** This is a multi‐center study (PED‐FALD) collecting data prospectively and retrospectively since January 2020 of FALD patients aged 10‐ 20 years. All imaging, cardiac catheterization, and transient elastography were collected. Laboratory tests were collected within 6 months of the liver biopsy. Liver histologic data was abstracted from the pathology reports and utilized the congestive hepatic fibrosis score (stage 0‐4).

**Results:** 251 patients with Fontan procedure were identified at 8 centers with mean age at 14.3 ± 3.3 years and mean age at Fontan procedure of 3.7 ± 1.7 years. The majority were male (153; 61%), and white (129; 51%). 51 patients (20%) had liver biopsy with the most common indication being surveillance (44; 86%). 24 patients (47%) had stage 3 or 4 liver fibrosis, and 9 patients (18%) had stage 4 liver fibrosis (cirrhosis) from liver biopsy. Patients with biopsy proven cirrhosis had significantly lower platelet count and higher GGT than patients without cirrhosis (155.5 vs. 202.8 x10^3^/uL; p = 0.03 and 78.6 vs. 49.2 units/L; p = 0.04, respectively). There was significant correlation between liver fibrosis score and non‐invasive fibrosis markers (FIB‐4, Forn's index; Spearman 0.28, p=0.05, 0.48, p=0.05), and free hepatic venous pressure (0.4, p=0.03). There was no significant association with histologic severity and liver stiffness measurements from FibroScan or elastography. 242 patients (96%) had imaging and only 15 patients (6%) had cirrhosis from imaging, including 9 from ultrasound, 1 CT, and 5 MRI. 5 patients died (2%), 37 patients were diagnosed with failing Fontan (15%) and 24 patients (10%) ended up with isolated heart transplant.

**Conclusion:** This is the first multi‐center study in children with FALD with ongoing data registry. Lower platelet counts and high GGT are good markers of cirrhosis. Overall, there was a low mortality rate at 2%. The fact that only 20% of the children in this study underwent liver biopsy demonstrates the need for more sophisticated measures of liver injury. The PED‐FALD network will help us understand how to monitor progression of the disease and to optimize treatment decisions in children with FALD.

## 587 MITOCHONDRIAL DNA CONTENT IN PERIPHERAL BLOOD CELLS AS A MARKER OF DISEASE PROGRESSION IN PEDIATRIC METABOLIC DYSFUNCTION ASSOCIATED STEATOTIC LIVER DISEASE (MASLD)

*Joseph Chapman* ^*1*^, *Michelle Ewart* ^*2*^, *Preeti Viswanathan* ^*1*^

^*1*^ *Pediatric Gastroenterology*, *Children's Hospital at Montefiore*, *New York*, *NY*; ^*2*^ *Department of Pathology*, *Montefiore Einstein Medical Center*, *New York*, *NY*

**Background:** Metabolic Dysfunction Associated Steatotic Liver Disease (MASLD) is the most common cause of chronic liver disease in childhood. Progression from fatty liver (FL) to steatohepatitis (MASH) greatly increases the risk of adverse consequences such as cirrhosis, hepatocellular carcinoma, and liver failure. Convenient approaches for early identification of children at risk for progression are a priority area in the field. Liver biopsies are impractical for routine monitoring, especially in children. Liver tests are convenient, but do not reliably predict disease progression. A unique aspect of childhood MASLD is its development in the context of ongoing liver growth, during which time the integrity of cellular DNA, both nuclear and mitochondrial, is critical. Cellular DNA is susceptible to damage from the byproducts of cellular metabolism, and the oxidative stress (ROS) generated during the metabolism of excess free fatty acids. Our previous work found that the integrity of hepatic DNA was an important determinant of disease progression in children. Specifically, hepatic nuclear DNA damage and reduction of hepatic mitochondrial content correlated closely with the presence of inflammation and fibrosis at diagnostic biopsy, when compared to healthy donor controls. Peripheral neutrophils (PMN) are recruited to the liver in MASLD, whereby they are susceptible to the same oxidative stress‐induced DNA damage. We therefore hypothesized that cellular DNA damage in PMN could be a mechanism‐based tool to identify children at risk for MASH ± fibrosis. We conducted a pilot study to examine nuclear and mitochondrial DNA in peripherally circulating neutrophils (PMN) for associations with hepatic steatosis, inflammation, ballooning, and fibrosis.

**Patients and Methods:** We analyzed blood and liver histology of children with biopsy proven MASLD in 2024‐2025 (N=17), liver histology from healthy donors (N=12) (NIH, NIDDK), and blood from non MASLD controls (N=17, obtained from obese and non‐obese children aged 2‐18 from our general pediatric gastroenterology clinic without known chronic illness and not on hepatotoxic medications). Biopsies were graded for steatosis, inflammation, ballooning, and fibrosis by a pediatric pathologist. PMN were isolated by magnetic beads (MACXpress) and efficient separation confirmed by morphology (Wrights stain) and FACS. Nuclear DNA damage was assessed by immunostaining with phospho‐histone H2AX, a marker for DNA double strand breaks. Mitochondrial DNA integrity was evaluated by mitochondrial DNA content via RT‐PCR of mitochondrial gene TL1 relative to nuclear gene RNAseP. Continuous variables reported as means ± SEM. Intergroup comparisons were through 2‐tailed Student's t test, Mann Whitney or ANOVA with post hoc adjustments as appropriate. p < 0.05 was considered significant.

**Results:** MASLD patients ranged in age from 11.9 ± 2.6 y vs 10.0 ± 6.1 y for non MASLD controls (p>0.05). Average BMI at biopsy was 96.3% ± 5.5 for MASLD patients versus 67.2% ± 33.5 for non MASLD controls (p<0.05). Average ALT was 230 ± 130 IU/L vs 16 ± 4 IU/L for non MASLD controls (p<0.05). Biopsy specimens had a NAFLD activity score of 4.43 ± 1.15, with 69% of specimens demonstrating grade 2 fibrosis or higher. Cellular DNA damage was significantly higher in PMN from MASLD patients compared to non MASLD controls. 95%± 7% of PMN from MASLD patients were positive for H2AX vs 0‐5% in non MASLD controls. Mitochondrial DNA content of PMN from MASLD patients was 696 ± 324 copies vs 2089 ± 874 copies in non MASLD controls (p<0.05). Additionally, mitochondrial DNA content correlated inversely with the degree of inflammation and >grade1 fibrosis on biopsy. Interestingly, neither H2AX staining nor mitochondrial DNA content of PMN had any correlation with serum ALT.

**Conclusion:** Cellular DNA damage in PMN was significantly more in MASLD compared to controls and correlated with severity of disease. These findings could be further developed in future longitudinal studies for early identification of children at risk for MASLD progression.

## 588 PEDIATRIC LIVER TRANSPLANT OUTCOMES AND THE ASSOCIATION WITH SOCIOECONOMIC STATUS: A POPULATION‐BASED COHORT STUDY OVER 30 YEARS

*Toshifumi Yodoshi* ^*1,6*^, *Ellen Kuenzig* ^*2,3*^, *Aaron Tang* ^*6*^, *Andréanne Zizzo* ^*3,4*^, *Vicky Ng* ^*1,5*^, *Eric Benchimol* ^*1,5*^

^*1*^ *Gastroenterology, Hepatology and Nutrition*, *The Hospital for Sick Children*, *Toronto*, *ON*, *Canada*; ^*2*^ *Child Health Evaluative Sciences*, *SickKids Research Institute*, *Toronto*, *ON*, *Canada*; ^*3*^ *Schulich School of Medicine and Dentistry*, *Western University*, *London*, *ON*, *Canada*; ^*4*^ *Division of Pediatric Gastroenterology & Hepatology*, *London Health Sciences Centre Children's Hospital*, *London*, *ON*, *Canada*; ^*5*^ *Pediatrics*, *University of Toronto*, *Toronto*, *ON*, *Canada*; ^*6*^ *ICES (the Institute for Clinical Evaluative Sciences)*, *Toronto*, *ON*, *Canada*

**Background:** Pediatric liver transplantation (LT) offers good long‐term survival, yet recipients face lifelong morbidities. Population‐based data on the association between socioeconomic status (SES) and very long‐term outcomes (including graft type interactions) after pediatric LT are lacking. We aimed to compare outcomes over three decades (LT vs. controls; deceased donor, DDLT vs. living donor, LDLT) and test if SES modulates these associations.

**Methods:** This was a retrospective population‐based cohort study from Ontario, Canada. Two centers conduct pediatric LT in the province. We linked all available pediatric (<18 y) clinical data from these centers (SickKids: 1991–2021; LHSC: 1996–2011) of first isolated LT to provincial administrative data. LT recipients were matched 1:5 by age and sex to general population controls with index dates defined as LT date and assigned as the same date in controls. Outcomes were: mortality, graft failure, de novo cancer, nine chronic comorbidities, childbirth. Analyses compared: (1) LT recipients vs. controls; (2) LDLT vs. DDLT; and (3) the interaction between group (LT vs. controls; LDLT vs. DDLT) and SES (dichotomized mean neighborhood income quintile [MNIQ] and material deprivation index [MDI]) (lowest two quintiles vs. highest two quintiles). Time‐to‐event outcomes used multivariable Cox or Fine‐Gray models, adjusted for key baseline factors.

**Results:** Among 449 LT recipients (median age 1.9 y at LT, follow up 14 y) matched to 2245 controls, LT had higher mortality, comorbidity, and cancer risks than controls (see Table); childbirth outcomes were comparable. DDLT (N=260) was associated with higher adjusted hazard of mortality, graft failure, and chronic kidney disease (CKD) versus LDLT (N=189) (Table); other outcomes were similar. SES did not modify the association between LT status and mortality. However, within LTs, low MNIQ (HR=2.1, 95%CI 1.2‐3.7) and high MDI (HR=2.7, 95%CI 1.1‐6.8)) was associated with mortality. The association between graft type and outcomes was modified by SES. Among DDLT recipients, low MNIQ increased mortality (HR 2.0, 95%CI 1.1‐3.8; MNIQ*Graft‐type p=0.002) and high MDI increased graft failure risk (MDI*Graft‐type p<0.001). These associations were non‐significant among LDLT recipients. Lower SES was linked to increased mood disorders only in LDLT (e.g., high MDI sHR 2.9 95%CI 1.6–5.4; MDI*Graft‐type p=0.02). For most other comorbidities, cancer, and childbirth, such interaction analyses were precluded by sparse events.

**Conclusion:** This three‐decade Ontario study showed pediatric LT survivors in universal healthcare faced high mortality, multimorbidity, and cancer risks; childbirth potential was preserved. LDLT offered better survival and lower CKD risk than DDLT. Critically, SES impacted outcomes. Overall, lower SES predicted increased mortality in all LT recipients. Furthermore, SES demonstrated novel, graft‐specific heterogeneity—lower SES worsened mortality/graft failure in DDLT recipients but was strongly linked to mood disorders in LDLT. These distinct vulnerabilities demand targeted, graft‐specific social and psychological support.


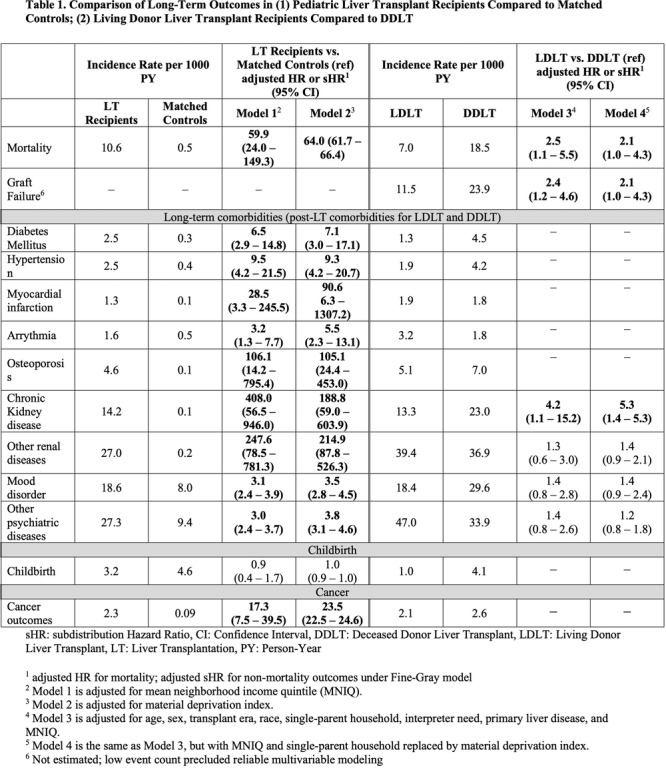


## 589 OUTCOMES OF VEDOLIZUMAB THERAPY IN BIO‐NAIVE VERSUS BIO‐EXPOSED CHILDREN WITH INFLAMMATORY BOWEL DISEASE

*Casey Konys* ^*1*^, *Joseph Runde* ^*2*^, *Jennifer Strople* ^*2*^, *Jeffrey Brown* ^*2*^

^*1*^ *Pediatrics*, *Ann & Robert H Lurie Children's Hospital of Chicago*, *Chicago*, *IL*; ^*2*^ *Pediatric Gastroenterology, Hepatology, and Nutrition*, *Ann & Robert H Lurie Children's Hospital of Chicago*, *Chicago*, *IL*

**Background:** The incidence of inflammatory bowel disease (IBD) in children is rising. Approved advanced therapies in children, like infliximab, have risks of adverse effects and treatment failure due to nonresponse, loss of response, and the development of anti‐drug antibodies. Vedolizumab is a monoclonal antibody approved in adults which targets the α4β7 integrin, offering a gut‐specific mechanism with fewer systemic side effects. Vedolizumab is considered first‐line therapy in adults, however, access in pediatric IBD is limited and often only available following failure of anti‐TNF therapy. Adult studies have demonstrated a lower likelihood of response when vedolizumab is used after anti‐TNF therapy. We aim to characterize the differences in response to vedolizumab between bio‐naive and bio‐exposed children with IBD.

**Methods:** We reviewed electronic medical records of children with IBD treated with vedolizumab at Ann & Robert H. Lurie Children's Hospital between 2014 and September 2024. Patients were excluded if they were over 18 years old when starting vedolizumab or if they did not complete induction dosing. Data collected included demographics, diagnosis, disease extent, and prior therapies. Outcomes, including need for dose escalation, concomitant therapies, endoscopic response and remission, and surgical outcomes, were compared between children who had prior exposure to biologic therapy ("bio‐exposed") to those who were naive to biologics ("bio‐naive").

**Results:** We identified 143 patients with IBD treated with vedolizumab, of which 98 were eligible for inclusion. 60% of patients were male. 28% had Crohn's Disease (CD), 49% had Ulcerative Colitis (UC), and 23% had IBD unclassified (IBD‐U). 52 (53%) were identified as bio‐naive (BN) and 46 (47%) were considered bio‐exposed (BE). The mean starting age (years) for vedolizumab was 12.9 [8.1,17.7] in BN and 12.3 [8.6,16] in BE. For the BE subjects, 63% had prior treatment with one biologic, 30% with two, and 7% with three or more. Compared to BE, fewer BN patients were on concomitant therapy (39% vs. 58%) and a lower ratio received escalated dosing of vedolizumab (25% vs 54%). Endoscopic response and remission were more common in the BN group (77% and 46%, respectively), compared to those who were BE (44% and 19%). Surgery was less common for BN vs. BE (8% vs 41%). Side effects occurred in 13% of patients and were mild.

**Conclusion:** Our large single center cohort demonstrates that vedolizumab is both safe and effective for pediatric patients with IBD regardless of previous biologic exposure. While data analysis is ongoing and may identify differences between the groups, our findings suggest that subjects starting vedolizumab as their first biologic therapy have improved outcomes, with less concomitant therapy, a lower likelihood of dose intensification, higher rates of mucosal healing and decreased rates of surgery. This comports with adult experience describing decreased efficacy of vedolizumab after anti‐TNF therapy and suggests consideration and increased uptake of vedolizumab as first line biologic therapy for children with IBD.


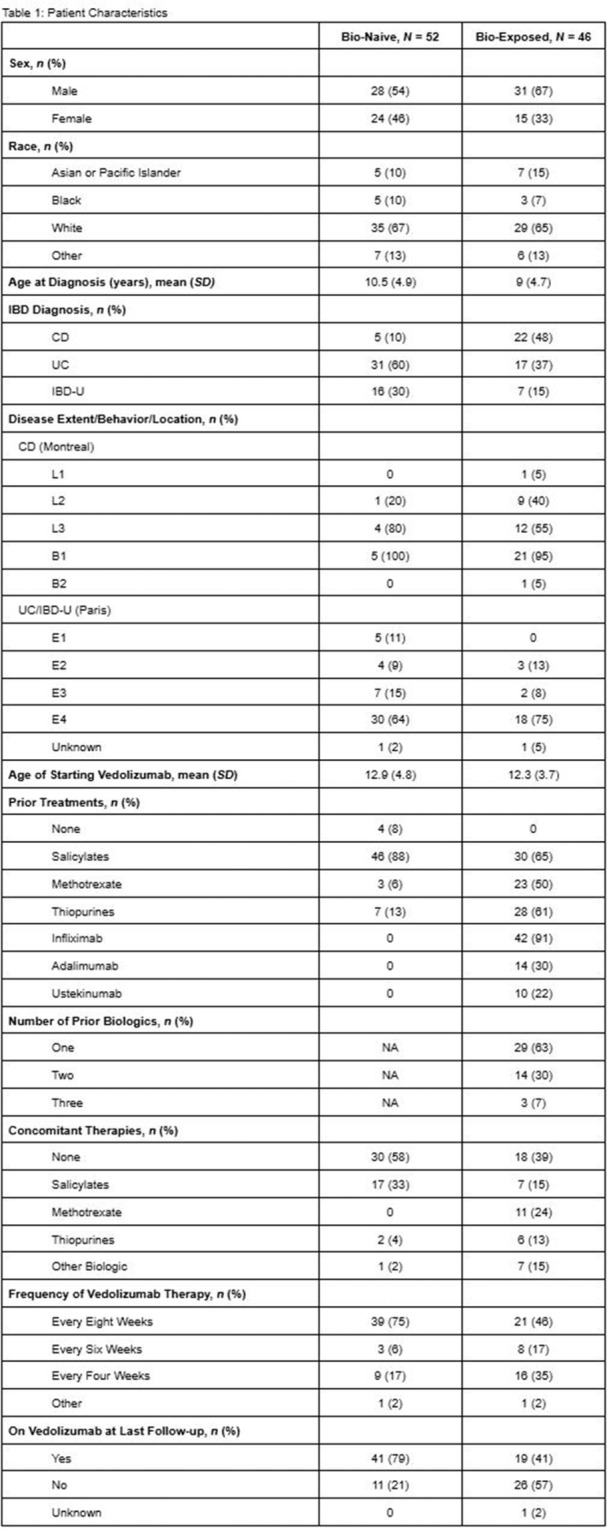


Patient Characteristics


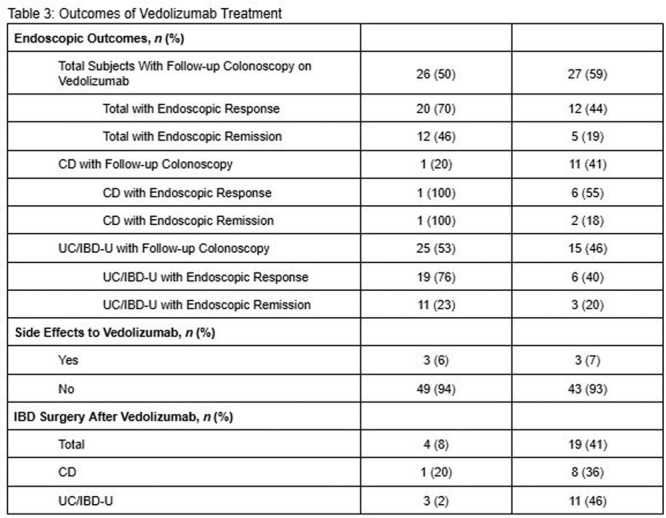


Outcomes of Vedolizumab Treatment

## 590 NATIONAL TRENDS IN COLECTOMY RATES IN PEDIATRIC PATIENTS WITH ULCERATIVE COLITIS AND IBD‐UNCLASSIFIED IN THE PRE AND POST BIOLOGIC ERA

*Emily Lai*, *Janet Wojcicki*, *Matt Pantell*, *Melvin Heyman*

*Pediatric Gastroenterology*, *University of California San Francisco*, *San Francisco*, *CA*

**Background:** Inflammatory bowel disease (IBD) is a chronic inflammatory disorder of the gastrointestinal tract, including Crohn's disease (CD), ulcerative colitis (UC), and IBD‐unclassified (IBD‐U). While many children respond to medical therapy, those with refractory disease may require colectomy for disease control.

Biologic therapies were first approved for pediatric IBD in 2011, marking the start of the “biologic era.” Since then, outcomes have been variable from international and U.S. single‐center studies —some citing rising colectomy rates, others reporting stable rates but increasing complications.

The Kids’ Inpatient Database (KID) is the largest publicly available all‐payer pediatric inpatient database in the U.S., offering national estimates of utilization and outcomes. We conducted a pre‐post analysis using KID data from 1997–2022, comparing colectomy rates, demographics, and hospitalization characteristics in pediatric UC and IBD‐U patients before and after biologic approval.

**Methods:** Hospitalizations for patients aged 0–18 with a diagnosis of of pediatric ulcerative colitis (pUC) or IBD‐unclassified (pIBD‐U) were identified using ICD‐9 and ICD‐10 codes for total colectomy.

Cohorts were defined by hospitalization year: Pre‐Biologic Era (PreB, <2011) and Post‐Biologic Era (PostB, ≥2011). Colectomy rates were calculated by dividing the weighted number of colectomies by the annual U.S. population <18 years, based on Census data.

Comparisons between cohorts used Chi‐squared tests for categorical and Wilcoxon rank‐sum tests for continuous variables. All analyses applied discharge‐level weights to generate nationally representative estimates.

**Results:** A total of 2,278 colectomies were identified among pediatric UC and IBD‐U patients. Of these, 1,465 (65%) occurred in the PreB cohort, and 639 (35%) in the PostB cohort. The overall median age at colectomy was 15 years (IQR 12–17).

Significant differences between cohorts were seen in sex (p = 0.01), race (p < 0.01), income quartile (p = 0.04), and age group (p = 0.01).

Hospital characteristics differed in primary payer type (p < 0.01), teaching status (p < 0.01), bed size (p < 0.01), and length of stay (p < 0.01), but not geographic region.

Figure 2 shows colectomy rates peaking in 2009 (0.05 per 100,000 patient‐years) with a decline during the PostB era (2012–2022). The median rate of decline from 2009 to 2022 was 0.016 colectomies per 100,000 patient‐years.

Table 1 summarizes demographic and hospital characteristics overall and by cohort.

**Conclusion and Discussion:** In this national analysis of pediatric UC and IBD‐U hospitalizations (1997–2022), colectomy rates peaked in 2009 and declined thereafter, suggesting a potential impact of biologics in reducing surgical intervention. Demographic and hospital‐level changes may reflect evolving disease patterns, care access, and systemic shifts in healthcare delivery.

Limitations include the encounter‐level nature of KID, which lacks longitudinal detail on presurgical course and exposures. Future studies should explore medical therapy's impact on colectomy risk and outcomes to better understand disparities and optimize care.


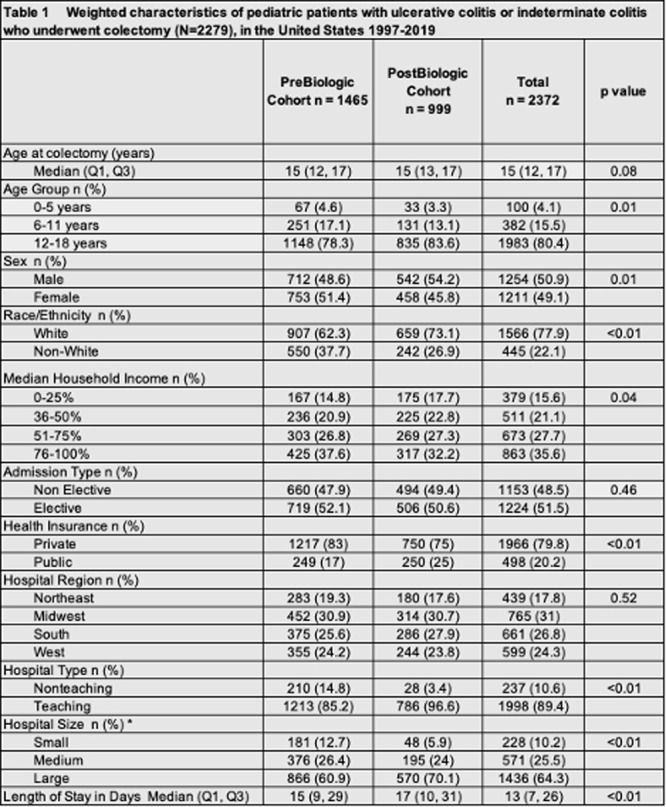



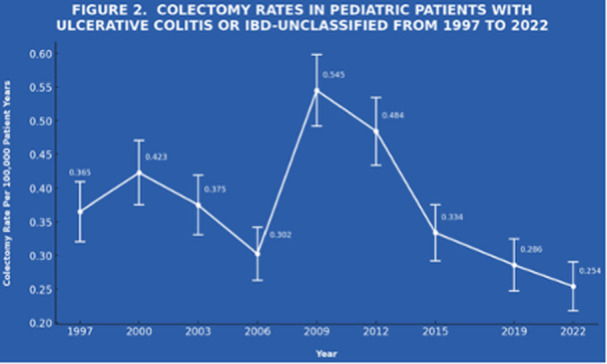


## 591 AN INNOVATIVE DREAM PROMOTES INTESTINAL ADAPTATION AND PREVENTS LIVER INJURY IN SHORT BOWEL SYNDROME

*Shaurya Mehta* ^*1*^, *Kento Kurashima* ^*1*^, *Chandrashekhara Manithody* ^*1*^, *ASHLESHA BAGWE* ^*1*^, *Marzena Swiderska‐Syn* ^*1*^, *Austin Sims* ^*1*^, *Sree Kolli* ^*1*^, *Aditya Jain* ^*2*^, *Miguel Guzman* ^*1*^, *Sherri Besmer* ^*1*^, *Sonali Jain* ^*1*^, *Paula Buchanan* ^*1*^, *Keith pereira* ^*1*^, *Chien‐Jung Lin* ^*1*^, *John Long* ^*1*^, *Matthew McHale* ^*1*^, *Chelsea Hutchinson* ^*1*^, *Ajay Jain* ^*1*^

^*1*^ *Pediatrics*, *Saint Louis University School of Medicine*, *Saint Louis*, *MO*; ^*2*^ *Johns Hopkins University*, *Baltimore*, *MD*

**Background:** Short bowel syndrome (SBS) prevents sustenance of nutritional needs via regular enteral nutrition (EN) and requires parenteral nutrition, resulting in detrimental side effects. Intestinal adaptation (IA), which is driven by EN remains a major goal. We have developed a revolutionary DREAM system (Distal Recirculation of Enteral contents Augmented Mechanically, US patent 63/413,988), Fig 1‐1, which allows full EN despite SBS and tested its impact on SBS related complications.

**Methods:** 20 neonatal pigs were randomly allocated to EN, SBS (75% bowel resection) or DREAM. Weight gain, serum chemistries, cytokines, intestinal morphology, tight junction integrity, hepatic histology, and gene expression were analyzed.

**Results:** DREAM animals demonstrated significant attenuation of liver and intestinal injury compared to SBS. DREAM reduced serum bilirubin (0.11 mg/dL vs. SBS 5.14 mg/dL, p = 0.0008), GGT (23.2 IU/L vs. SBS 114.6 IU/L, p < 0.0001), and total bile acids (9.7 µmol/L vs. SBS 39 µmol/L, p = 0.0026), Fig 1‐2. DREAM also normalized inflammatory cytokines (e.g., IFN‐γ, p = 0.0296; IL‐1β, p = 0.0349; IL‐6, p = 0.0189) and decreased LPS levels (p = 0.0130) versus SBS, Fig 1‐3. DREAM preserved hepatic expression of BSEP and Cyp7A1 (p = 0.0034 vs. SBS), restored intestinal EGF and TGR5 signaling (p < 0.01), and reduced hepatic cholestasis scores (p = 0.0012 vs. SBS), Fig 1‐4. IA markers, including linear gut density (proximal: 0.237 g/cm vs. SBS 0.121, p = 0.0042), villus‐to‐crypt ratio (p = 0.0026), Fig 1‐5, and tight junction protein expression (e.g., Occludin, p < 0.0001; E‐cadherin, p = 0.0002) mirrored EN animals and significantly improved with DREAM, Fig 1‐6. DREAM reduced transcriptional dysregulation, Fig 1‐7 and reactivated key functional pathways, resulting in a gene expression profile more similar to EN, Fig 1‐8. Macronutrient absorption from recirculated enteral feeds was rapid and sustained, with >80% absorption of protein and fat by 6 hours (p < 0.0001), Fig 1‐9

**Conclusion:** DREAM enables full EN despite SBS to restore nutrient absorption and gut derived signaling, mitigating intestinal and hepatic injury. This innovative, translation system highlights a major advancement in SBS management bringing a paradigm shift to life saving strategies for SBS patients.


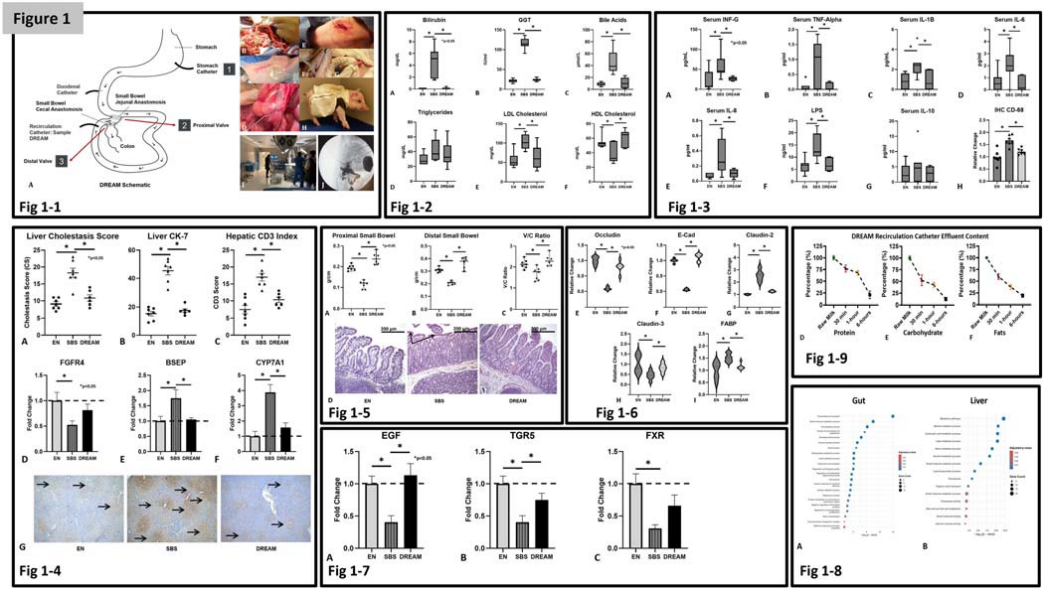


## 592 SALIVARY SUBSTANCE P IS A SENSITIVE AND SPECIFIC BIOMARKER OF ASPIRATION IN INFANTS AND TODDLERS

*Daniel Duncan* ^*1*^, *Clare Golden* ^*1*^, *Michael Kim* ^*1*^, *Tina Samuels* ^*2*^, *Alexandra Vandenberg* ^*2*^, *Nikki Johnston* ^*2*^, *Rachel Rosen* ^*1*^

^*1*^ *Aerodigestive Center/Gastroenterology*, *Boston Children's Hospital*, *Boston*, *MA*; ^*2*^ *Otolaryngology*, *Medical College of Wisconsin*, *Milwaukee*, *WI*

**Background:** Despite being a cause of severe aerodigestive symptoms in children, the current approach to diagnosis and management of oropharyngeal dysphagia is hindered by: 1) lack of sensitivity of observed feedings for diagnosing aspiration and 2) limitations of the gold standard videofluoroscopic swallow study (VFSS), including radiation exposure, limited timeslots available, and need for patient cooperation for reliable results. Small studies in adult populations suggest that substance P can be measured in saliva. We hypothesize that this biomarker could be used to predict aspiration risk in children.

**Methods:** We recruited children under 2 years of age undergoing evaluation for oropharyngeal dysphagia at Boston Children's Hospital who had VFSS performed within 3 months of study enrollment. Children with oropharyngeal/craniofacial anomalies or history of oropharyngeal/gastrointestinal procedures were excluded. Saliva samples were collected using infant or child‐size absorbent saliva collection devices with tip placed in the subject's mouth (salimetrics.com). Saliva levels were measured by ELISA for substance P (abcam.com). Medical records were reviewed for subject characteristics, comorbidities and VFSS results. We used the student's t‐test compare values between subjects with aspiration vs those without aspiration. Receiver operating characteristic (ROC) analyses were used to determine optimal cut‐off values and to calculate values for sensitivity and specificity compared to gold standard VFSS results.

**Results:** The cohort included 42 subjects with mean age 8.5 ± 0.01 months at the time of sample collection. Apart from aspiration status, there were no significant differences in baseline characteristics, comorbidities or medications between the groups as in Table 1. Substance P was 254.5 ± 41.8 pg/ml for those with aspiration compared to 48.4 ± 8.5 pg/ml for those without aspiration (p<0.001) as shown in Figure 1 A. There were no differences in substance P level when comparing by prematurity, neurologic comorbidities, cardiac comorbidities, or PPI or H2RA treatment status (all p>0.28) but aspirating female subjects had higher levels compared to aspirating male subjects (363.6 ± 67.6 vs 131.1 ± 16.7, p=0.004). On ROC analysis, area under the curve (AUC) was 0.963 (95% CI 0.899 – 1.03, p<0.001), as shown in Figure 1B. Using a cutoff of 63.9 pg/ml provides 97% sensitivity and 90% specificity for predicting aspiration in children.

**Conclusions:** Salivary substance P is a novel biomarker that can predict aspiration in infants and toddlers with high sensitivity and specificity. Levels are significantly higher in children with aspiration compared to children without aspiration.


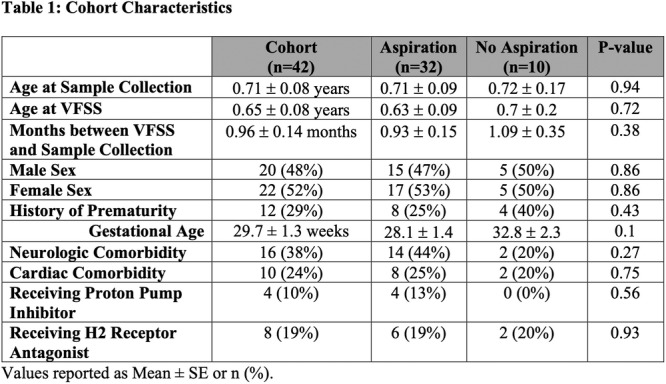



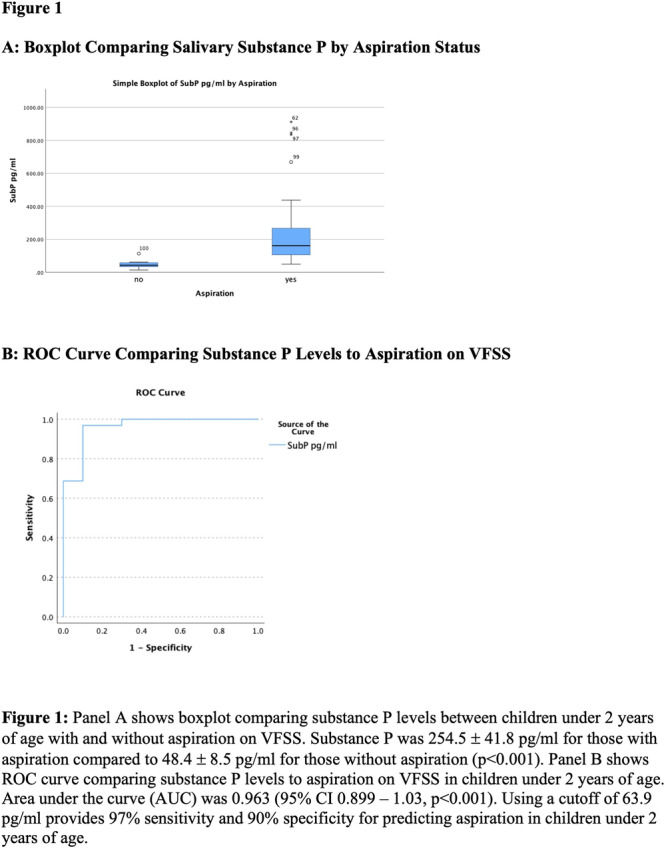


## 593 GASTRIC PERORAL ENDOSCOPIC MYOTOMY (GPOEM) PERFORMED BY A PEDIATRIC GASTROENTEROLOGIST IS SAFE AND EFFECTIVE IN THE TREATMENT OF PEDIATRIC GASTROPARESIS

*Ashley Croker‐Benn*, *Samuel Nurko*, *Peter Ngo*

*Division of Gastroenterology*, *Boston Children's Hospital*, *Boston*, *MA*

**Introduction:** Gastric peroral endoscopic myotomy (GPOEM) is an emerging therapeutic option for the treatment of gastroparesis in adult and pediatric populations. Adult literature reports a decrease in Gastroparesis Cardinal Symptom Index (GCSI) score greater than or equal to 50% post‐GPOEM in approximately 60% of adult patients. To date, there have been no published reports of GPOEM performed by a pediatric gastroenterologist and only a single published case report of GPOEM for the treatment of gastroparesis in a pediatric patient. This study aims to evaluate the safety and efficacy of GPOEM performed by a pediatric gastroenterologist for the treatment of gastroparesis in both adult and pediatric patients.

**Methods:** A retrospective review of all GPOEM procedures performed by PN between 12/1/2023 ‐ 5/22/2025 at a single pediatric center. Data collected included patient demographics, GCSI scores, Gastric Emptying Scan (GES) results, procedure details, and adverse events. A two‐tailed paired T‐test was used to compare pre‐ and post‐GPOEM GCSI scores with subgroup analysis for patients equal to or under and over the age of 21. A linear regression analysis was performed to evaluate the association between case order and operative time. P‐value <0.05 was considered significant.

**Results:** A total of 12 GPOEM procedures were performed. All GPOEMs were successfully completed and performed with a T‐type hybrid knife along the greater curvature of the stomach with longitudinal mucosal incisions closed with hemostatic clips. Median patient age was 20 years (range 9‐33, 5 greater than 21 years, IQR 13.5, 26.5). Median procedure length was 101 minutes (IQR 96, 155). All patients had gastroparesis symptoms with baseline median GCSI score of 2.69 (IQR 2.11, 3.08) consistent with moderate gastroparesis. Prior gastroparesis therapies included use of prokinetics and intra‐pyloric onabotulinum toxin A injections in all patients with transient clinical improvement after injection. Baseline 4‐hour solid food GES showed delay (>10% retained at 4 hours) in 6/9 patients (66.7%). Pre‐ and post‐GPOEM GCSI scores with minimum 1 month follow up were available for 10 patients. GCSI scores decreased post‐GPOEM in 9/10 (90%, Fig.1) with mean follow up of 6 months (range 1 to 17 months). Mean pre‐ and post‐GPOEM GCSI scores decreased (2.73, 1.22, p<0.01) in the group of all 10 patients (Fig.1) and in both subgroups of those under (n=6) and over (n=4) 21 years of age (2.69, 1.52, p=0.026; 2.78,0.78, p=0.047). A 50% or greater decrease in GCSI score was observed in 6/10 patients (60%).

Endoscopic functional lumen impedance planimetry (EndoFLIP) was performed in 12 patients at the time of GPOEM with pre‐ and post‐myotomy data available for 8 patients. Mean pre‐ and post‐myotomy pyloric diameter and distensibility at matching maximum balloon volumes were unchanged (17.1 mm, 18.1 mm, p=0.17; 5.08mm^2^/mmHg, 5.78 mm^2^/mmHg, p=0.28).

No major complications were reported with only a single inconsequential mucosal injury that was closed with clips. There were no unsatisfactory closures of the mucosal incision. All patients had a fluoroscopic contrast study demonstrating intact mucosal closure without leak on post‐operative day (POD) 1 and discharge on POD1.

Linear regression showed a significant decrease in operative time with increasing case number with operative time decreasing by approximately 9 minutes per case (slope= ‐9.06, R^2^ = 0.54, p<0.01), Fig. 2.

**Conclusions:** We demonstrate the safety and efficacy of GPOEM performed by a pediatric gastroenterologist with the largest reported number of pediatric patients undergoing GPOEM for symptoms of gastroparesis with careful patient selection. GPOEM was successfully performed in all patients with a significant decrease in GCSI scores without any serious adverse events. In contrast to POEM for achalasia, EndoFLIP, measurements may have limited utility in evaluating GPOEM myotomy intra‐operatively consistent with reported adult findings. Post‐GPOEM improvement in GCSI scores in both adult and pediatric patients were comparable to results in adult literature. Increased operative experience is associated with decreased operative time.


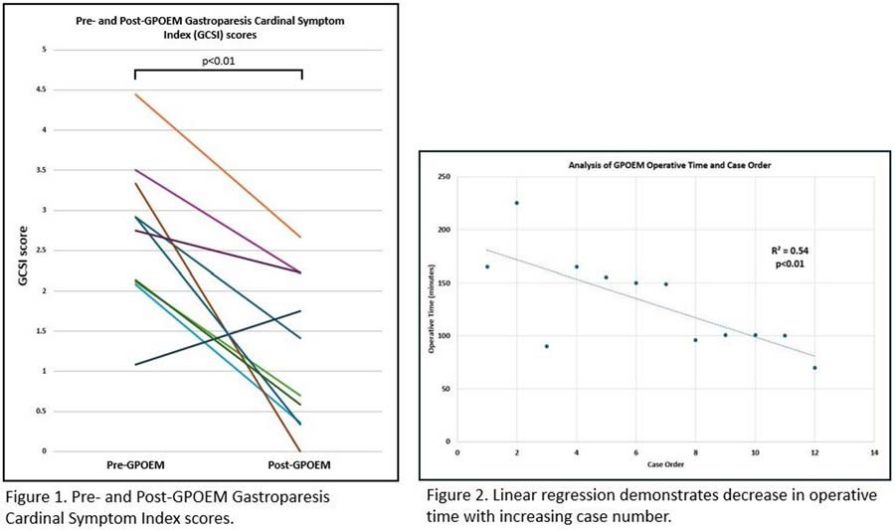


## 594  A NOVEL TRUNCATING EP300 MUTATION IMPAIRS INTESTINAL EPITHELIAL DIFFERENTIATION IN PATIENT TISSUE AND ORGANOIDS AND CAN BE RESCUED BY CHEMICAL HDAC INHIBITION

*Lauren Collen* ^*1*^, *Katlynn Bugda Gwilt* ^*1*^, *Noah Hoffman* ^*2*^, *Jaclyn Siegel* ^*4*^, *Michael Field* ^*1*^, *Lily Gillette* ^*1*^, *Maiya Whalen* ^*1*^, *Ashrita Iyengar* ^*1*^, *Jeffrey Goldsmith* ^*3*^, *Scott Snapper* ^*1*^, *Jay Thiagarajah* ^*1*^

^*1*^ *Gastroenterology*, *Boston Children's Hospital*, *Boston*, *MA*; ^*2*^ *Gastroenterology*, *Maine Medical Center*, *Portland*, *ME*; ^*3*^ *Pathology*, *Boston Children's Hospital*, *Boston*, *MA*; ^*4*^ *Gastroenterology*, *The University of Texas Health Science Center at Houston*, *Houston*, *TX*

**Background:** Very Early Onset Inflammatory Bowel Disease (VEOIBD) is defined as disease onset at age <6. A subset of patients with VEOIBD have causal monogenic variants that disrupt epithelial homeostasis. These patients may have disease that is severe and refractory to conventional therapies. *EP300* encodes p300 – a histone acetyltransferase and essential regulator of chromatin accessibility and transcription. We describe *EP300* as a novel monogenic cause of VEOIBD, and employ patient‐derived organoids to characterize effects of partial loss of p300 function on the intestinal epithelium and validate a precision therapy approach.

**Methods:** The Very Early Onset IBD Consortium (veoibd.org) has enrolled >200 patients with VEOIBD into its research pipeline for generation of multi‐omic data and patient‐derived organoids. Organoid growth and morphology are assessed using high‐content imaging. Targeted organoid experimentation, including evaluation of transcript (qRT‐PCR) and protein (Western blot) levels are performed on relevant patients and age‐matched non‐IBD controls. Evaluation of monogenic gene candidates is carried out through analysis of genomic sequencing, GeneMatcher for additional case identification, and orthogonal data from the VEOIBD Consortium.

**Results:** A 20‐month‐old female presented with severe, secretory diarrhea and associated hypovolemic shock and electrolyte derangements, necessitating prolonged intensive care. She was parenteral nutrition (PN)‐dependent and her endoscopic evaluation was notable for intestinal edema with loss of vascular patterning. Intestinal histology exhibited striking abnormalities, with severe chronic mucosal injury in the colon, and near‐complete loss of goblet cells, metaplasia (including clusters of pancreatic acinar cells and gastric oxyntic cells), and grossly abnormal mucosal architecture in the duodenum. Collectively, histologic findings pointed to possible defects in epithelial cell differentiation and identity. Whole genome sequencing revealed a novel, heterozygous truncating variant in *EP300*, which in combination with hallmark dysmorphic features, confirmed a diagnosis of Rubinstein‐Taybi syndrome (RTS). Gastrointestinal inflammation or metaplasia have not previously been described in RTS, but two additional subsequently identified cases of RTS displayed features of gastrointestinal inflammation with metaplasia/dysplasia. We therefore hypothesized that partial loss of p300 function favors decreased acetylation of chromatin, leading to impaired cellular differentiation and abnormal epithelial maturation.

Analysis of the index *EP300* variant patient duodenoids showed severely impaired viability, abnormal morphology, and reduced growth. Western blot analysis revealed reduced histone H3 acetylation, consistent with defective p300 histone acetyltransferase activity. Transcriptional analysis showed aberrant upregulation of Pepsinogen (gastric) and HNF4A (pancreas), and reduced EPCAM (intestine), suggestive of abnormal ectopic lineage differentiation. Baseline expression of ALPI and NEUROG3 were 2‐fold lower than controls, indicating impaired absorptive and enteroendocrine lineage specification. LGR5, ASCL2 and OLFM4 expression were reduced, suggesting reduced stemness. Potential cancer‐associated markers BIRC5, MYC, and BMI1 were also upregulated.

We hypothesized that histone deacetylase inhibition (HDACi) may be able to compensate for reduced *EP300* function by favoring a state of histone acetylation, and therefore, chromatin accessibility. Valproic acid (VPA) is an HDACi approved for use in pediatric patients for neurologic indications. EP300 variant duodenoids treated with VPA exhibited increased viability, normalization of morphology, and increased H3 acetylation. VPA also modulated cell differentiation with reduction in HNF4A and loss of Pepsinogen expression.

**Conclusion:** We identified *EP300* loss of function variants as a novel monogenic cause of IBD. p300 loss of function and reduced histone acetylation leads to significant epithelial disorganization, gastrointestinal metaplasia/dysplasia and altered cell identity in intestinal tissue and enteroids. In vitro application of the approved HDACi VPA resulted in significant improvement in epithelial viability and a reduction in ectopic lineage differentiation, supporting its use as a precision therapy for our index *EP300* patient. These results suggest that *EP300* function is critical for defining intestinal epithelial differentiation programs and cellular identity. Our work supports a precision medicine approach for epigenetic therapies in genetically defined VEOIBD and highlights the translational utility of organoids to guide individualized clinical care.

## 595 THE CONTEXT DEPENDENT ROLE OF CELLULAR SENESCENCE IN BILIARY ATRESIA

*Katie Conover* ^*2*^, *Franziska Lammert* ^*1*^, *Eric Katz* ^*2*^, *Ron Sokol* ^*2*^, *Sarah Taylor* ^*2*^

^*1*^ *Universitatsklinikum Aachen*, *Aachen*, *NRW*, *Germany*; ^*2*^ *University of Colorado Anschutz Medical Campus School of Medicine*, *Aurora*, *CO*

**Background:** Cellular senescence supports tissue repair but can become dysregulated and contribute to disease, in part by immune cell modulation. Increased senescence is present in biliary atresia (BA), but its role in disease progression remains poorly defined. We aimed to explore immune‐senescent cell networks based on BA disease stage and patient outcome.

**Methods:** We used multiplex histology to identify cell densities in liver tissue (cholangiocytes: CK19; senescence: P21; T regs: CD3, FOXP3; TREM2+ macrophages [Mφ]: CD68, TREM2) in BA at diagnosis (BADx, n=17), BA at transplant (BATx, n=22), cholestatic controls (CC, n=13), and non‐cholestatic controls (NCC, n=7). Targeted serum proteomics detected 61 senescence‐associated secretory proteins (SASPs) and 39 BA‐related proteins in BADx (n=12), BATx (n=12), CC (n=12) and NCC (n=10). Statistical analysis compared all groups and BA groups by outcome (survival with native liver ≥2 years [favorable outcome] vs <2 years [poor outcome]).

**Results:** Among all groups, cholangiocyte density was highest in the BA groups (p=0.02), but there were no significant differences in senescent or immune cell subsets. BADx with favorable outcome had greater senescent cholangiocytes (p=0.01), TREM2+ Mφ (p=0.01), and T reg densities (p=0.04). Total Mφs, TREM2+ Mφs, and T regs correlated with senescent cells (r=0.654, r=0.761, r=0.639 respectively, p<0.05 for all).

Among all groups, 30 SASPs and 17 BA‐associated proteins significantly differed (p adj<0.05). Nine of 10 SASPs differing between BADx and BATx were higher at transplant (p adj<0.05, FC>1.5, **Figure 1 A**). BADx patients separated discreetly by outcome (**Figure 1B**). Of the top 20 proteins driving this separation (**Figure 1 C**), most (9 of 11) SASPs were associated with poor outcome. Pathway analysis revealed BADx patients with poor outcome had IL‐6/JAK/STAT3 activation (p adj 7.45 e‐7), which is known to regulate oxidant‐induced senescence in fibroblasts. BADx patients with favorable outcome had HIF‐1a stabilization (p adj 0.02), which could be related to increased antioxidant gene expression.

**Conclusion:** In BADx, liver tissue senescence is associated with favorable outcome, while SASPs are associated with poor outcome and progress over disease course. Our data highlight the dynamic and context‐dependent nature of cellular senescence. Impaired redox homeostasis may modulate senescence and contribute to discrepant BA outcomes, warranting exploration of senescent cell driven oxidative‐stress modulating therapies.


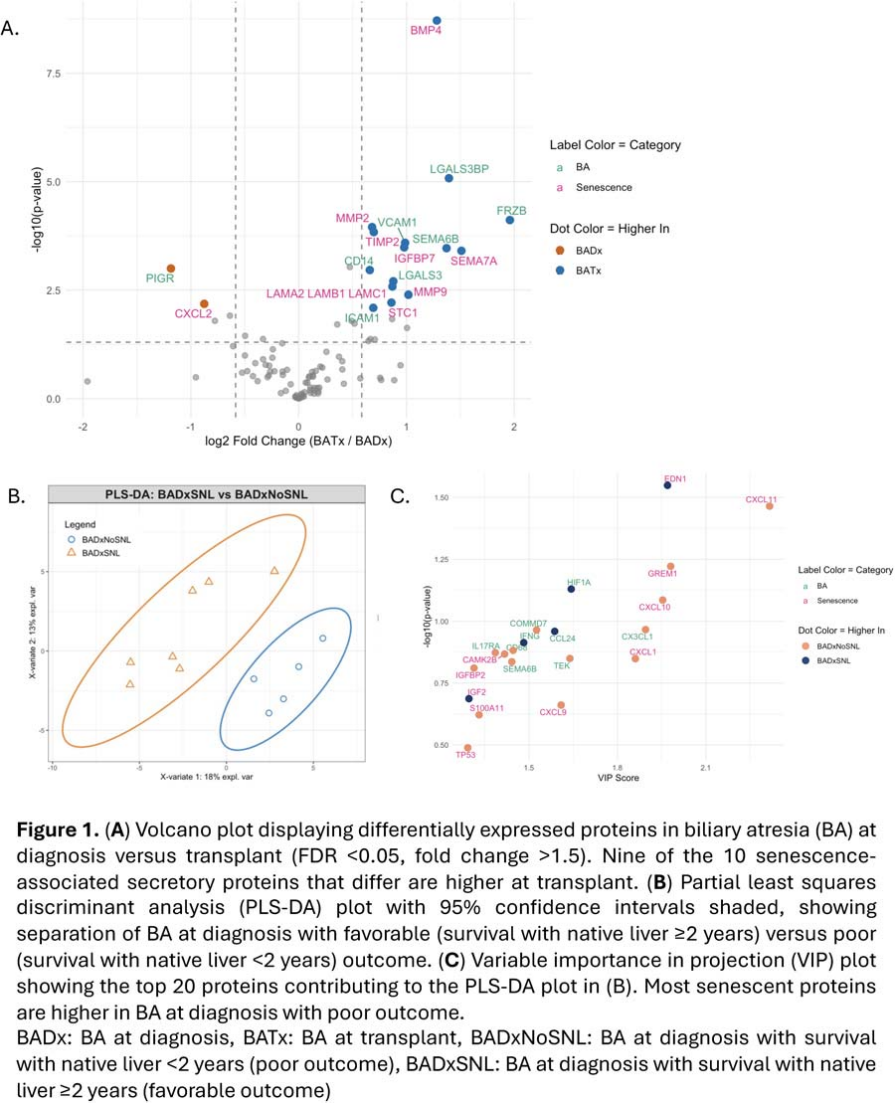


## 596 IMPACT OF APREPITANT USE ON LENGTH OF STAY AND REPEAT HOSPITALIZATION FOR CHILDREN WITH CYCLIC VOMITING SYNDROME

*Sergio Cobos* ^*2*^, *Peter Lu* ^*1*^, *Neetu Bali Puri* ^*1*^, *Karla Vaz* ^*1*^, *Raul Sanchez* ^*1*^, *Carlo Di Lorenzo* ^*1*^, *Desale Yacob* ^*1*^

^*1*^ *Pediatric GI*, *Nationwide Children's Hospital*, *Columbus*, *OH*; ^*2*^ *Pediatric Residency*, *Nationwide Children's Hospital*, *Columbus*, *OH*

**Background:** Cyclic vomiting syndrome (CVS) is a disorder of gut‐brain interaction (DGBI) that often leads to hospitalization. Our understanding of factors that influence length of stay and need for further hospitalizations is limited. Aprepitant has been shown to be effective as an abortive and prophylactic medication for children with CVS. Our objective was to evaluate whether aprepitant administration at presentation decreases length of stay and likelihood of repeat hospitalization for children with CVS.

**Methods:** We performed a retrospective chart review. We identified all hospital encounters with a diagnosis of CVS and selected the most recent hospitalizations for children with CVS episodes. We recorded demographic data, medical history, age of onset and diagnosis of CVS, medications for CVS, information on care in the Emergency Department (ED), and information on their hospitalization. Primary outcomes included length of stay and whether each patient required further hospitalizations for CVS after discharge. We compared hospitalizations where the patient received aprepitant versus hospitalizations where they did not.

**Results:** We included 20 children (85% female, median age 16 years, range 11‐19) hospitalized for CVS who received aprepitant and 14 children (57% female, median age 15, range 6‐17) hospitalized for CVS who did not receive aprepitant. Patient characteristics are shown in **Table 1**. Children who received aprepitant were more likely to be White race (18/20 vs. 7/14, p=0.02) and to have been diagnosed with cannabinoid hyperemesis syndrome (15/20 vs. 3/14, p<0.01). Patient characteristics were otherwise similar, including comorbid DGBI and psychiatric conditions, use of home abortive and prophylactic medications, and intravenous fluid administration in the ED. There was no difference in the hospital length of stay between the hospitalized children who did and did not receive aprepitant in the ED (median of 2 days stay for both groups, p = 0.56). Likelihood and number of further hospitalizations for CVS after discharge were also the same at 50% and a median of 0.5 (p = 0.64) for both groups.

**Conclusion:** Once a child has been hospitalized for CVS, whether they received aprepitant in the ED or not was not associated with either length of stay or future hospitalization for CVS. Our findings support prior literature demonstrating that if initial abortive intervention is not successful in stopping a CVS episode and hospitalization is required, subsequent care is supportive and does not influence the duration of the episode.


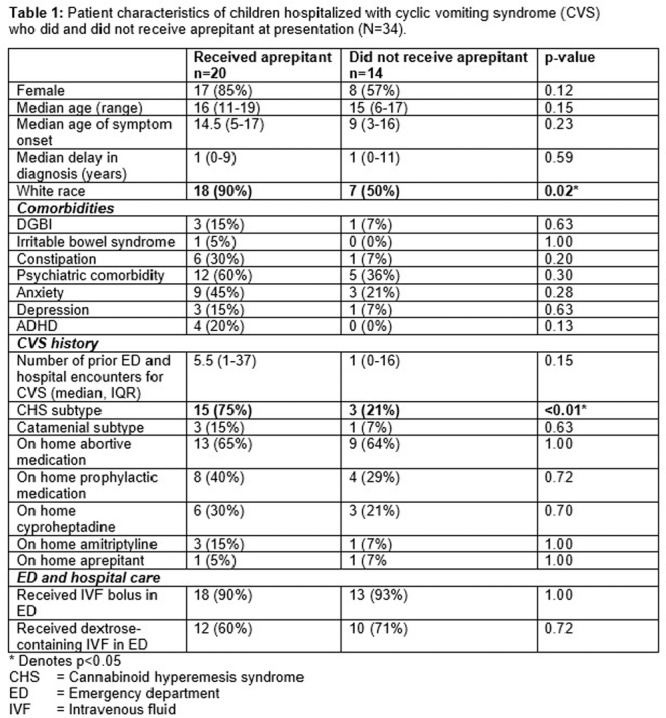


## 597  A LARGE‐SCALE ELISA‐BASED STUDY TO DETERMINE THE DIAGNOSTIC THRESHOLD OF HUMAN PEPSIN A AS A BIOMARKER OF MICROASPIRATION

*Nidhi Kapoor* ^*2*^, *Noah Stoeckel* ^*2*^, *Mary Schreck* ^*3*^, *Melanie Paredes* ^*1*^, *Bassam Abomoelak* ^*2*^, *Chirajyoti Deb* ^*2*^, *Devendra Mehta* ^*4*^

^*1*^ *University of Central Florida*, *Orlando*, *FL*; ^*2*^ *specialty diagnostic and translational lab*, *Orlando Health*, *Orlando*, *FL*; ^*3*^ *OHMG Peds Spec Prac Resources*, *Orlando Health*, *Orlando*, *FL*; ^*4*^ *APH Gastroenterology Practice*, *Orlando Health Arnold Palmer Hospital for Children*, *Orlando*, *FL*

**Background:** Pepsins are aspartate proteases secreted by gastric chief cells as inactive *pepsinogens*. Their enzymatic activity is optimal at pH 1.5‐2.5 but remains stable up to pH 8. Reactivation of pepsin has been reported in laryngopharyngeal reflux disease following successive acid reflux events. Therefore, measuring pepsin protein may serve as a more reliable indicator of microaspiration. Although pepsin A has gained attention as a surrogate marker of microaspiration, its clinical utility remains controversial due to inconsistent data arising from variable detection methods. Accurate enzymatic detection requires active pepsin A, which can be irreversibly inactivated by factors such as sample collection, storage conditions, and pH ‐ potentially leading to false negatives. In our previous pilot study, we demonstrated that compared to enzymatic assay, ELISA‐based method was more sensitive and was able to accurately detect microaspiration in patients who tested false negative by enzymatic method. In this study, we measured pepsin A protein levels in bronchoalveolar lavage (BAL) samples from a larger cohort of patients and determined a diagnostic threshold for its use as a biomarker of microaspiration.

**Methodology:** Bronchoalveolar lavage (BAL) samples were collected from 228 patients, presented with clinically indicated symptoms for microaspiration. Samples were centrifuged for 5 min at 13,000 rpm at 4^o^C, and the supernatant was analyzed for pepsin A using a novel ELISA method. Based on clinical evaluation, 120 patients were classified as having low (normal) microaspiration, while 108 patients were suspected to have moderate to high (abnormal) microaspiration. A commercially available quantitative ELISA kit with a detection range of 37 to 4000 pg/ml was used for pepsin A quantification. This assay is specific for human pepsin A and does not cross‐react with pepsinogen A or pepsinogen C/pepsin C. Two statistical methods‐ logistic regression and the percentile method were employed to determine the diagnostic threshold of pepsin A.

**Results:** Pepsin A levels were measured in all 228 BAL samples by ELISA. The abnormal group exhibited significantly higher pepsin A level (mean 41.9 ng/ml) compared to the normal group (mean 2.71 ng/ml). Logistic regression analysis yielded a receiver operating characteristic (ROC) curve with an area under the curve (AUC) of 0.9139. The optimal cutoff value was determined to be 8.69 ng/ml with a sensitivity of 77% and specificity of 91%. Using the 3^rd^ percentile method based solely on the normal group, a similar cut‐off of 8.6 ng/ml with a sensitivity of 77% and specificity of 93% was obtained.

**Conclusion:** Using this novel ELISA method, we successfully quantified pepsin A levels in BAL samples from patients that had symptoms and suspicion of microaspiration. In a large cohort of 228 patients, we established a clinically relevant diagnostic threshold of 8.69 ng/mL, using two independent statistical approaches. This threshold demonstrated strong sensitivity and specificity, supporting the utility of pepsin A as biomarker of microaspiration. As tracheal microaspiration of gastric pepsin A can have significant clinical consequences, this new sensitive ELISA based test for pepsin A holds a better promise to help with accurate diagnosis of microaspiration, improved symptom managements, and better treatment outcomes.


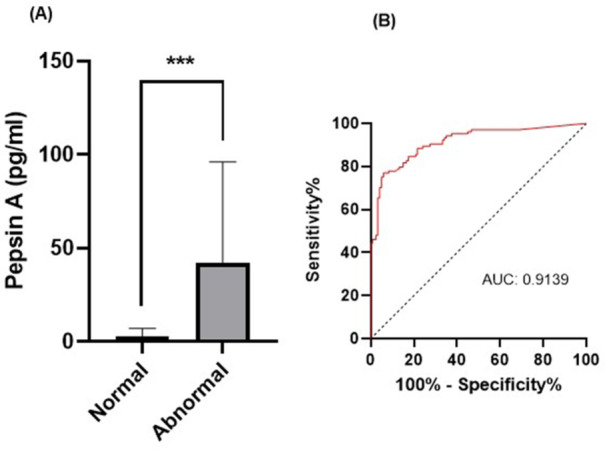


**Figure 1:** Mann‐Whitney Test shows a statistically significant higher concentration of pepsin A in the abnormal group compared to the normal group of patients. (*p

## 598 DECODING CILIARY DYSFUNCTION IN HEPATOBILIARY DEVELOPMENT: MECHANISTIC INSIGHTS INTO BILIARY ATRESIA PATHOGENESIS

*Prasanna Venkatesh Ramachandran* ^*1,3*^, *Wolfram Goessling* ^*2,3*^

^*1*^ *Pediatric Gastroenterology*, *Boston Children's Hospital Division of General Pediatrics*, *Boston*, *MA*; ^*2*^ *Massachusetts General Hospital*, *Boston*, *MA*; ^*3*^ *Harvard Medical School*, *Boston*, *MA*

**Background:** **Biliary atresia (BA) is a life‐threatening pediatric liver disease marked by progressive bile duct obstruction, liver fibrosis, and eventual failure**. Despite being the leading indication for pediatric liver transplantation, the underlying mechanisms of BA remain poorly understood. Emerging evidence suggests primary cilia – vital organelles for cellular signaling – play a critical role in liver development and biliary epithelial cell (BEC) integrity. Aberrations in cilia‐associated pathways, including Hedgehog (Hh), Wnt, and Notch, have been implicated in BA pathogenesis. Notably, pathogenic variants in ciliary genes occur in over 30% of BA patients, and liver‐specific knockout of the ciliary gene polycystin‐1‐like 1 (*PKD1L1*) replicates features of BA in a mouse model. How *PKD1L1* deficiency disrupts ciliary function and hepatobiliary development is unknown.

**Methods:** To investigate the role of primary cilia in hepatobiliary development, we employed a zebrafish (*Danio rerio*) model, leveraging its conserved liver development pathways and optical clarity allowing for direct visualization of early hepatobiliary development *in vivo*. We performed morpholino‐mediated knockdown of *pkd1l1* and confirmed dose‐dependent effects. We employed high‐resolution spinning‐disk confocal microscopy to visualize phenotypic consequences on hepatobiliary structures using transgenic reporters (*fabp10a:CFP* labeling hepatocytes; *tp1:GFP*labeling BECs). We also visualized ciliogenesis and ciliary architecture using a cilia‐specific reporter (*arl13:GFP*), and measured transcriptional responses to *pkd1l1* deficiency by qRT‐PCR from dissected livers.

**Results:** Knockdown of *pkd1l1* resulted in severe hepatobiliary malformations, including a 60% reduction in BEC density, fragmented biliary networks, and a 2.1‐fold expansion in hepatocyte populations, mimicking the cholangiopathy observed in BA (Figure 1A‐C). High‐resolution imaging revealed significant structural disorganization and misorientation of cilia in *pkd1l1*‐deficient BECs. Transcriptomic analysis confirmed aberrant Hh signaling activation (Figure 1D). These findings directly link ciliary dysfunction to disrupted biliary tree development.

**Conclusions:** **Our study highlights the pivotal role of primary cilia in maintaining hepatobiliary integrity** and elucidates how *pkd1l1* deficiency amplifies Hh signaling to drive biliary dysmorphogenesis. By pinpointing **specific mechanisms underlying BA pathogenesis**, these results lay the groundwork for targeted therapies aimed at mitigating ciliary‐driven bile duct damage in pediatric patients. Ongoing efforts, including single‐cell and spatial transcriptomic analyses, aim to further dissect intrahepatic and extrahepatic gene expression changes in *pkd1l1*‐deficient livers.


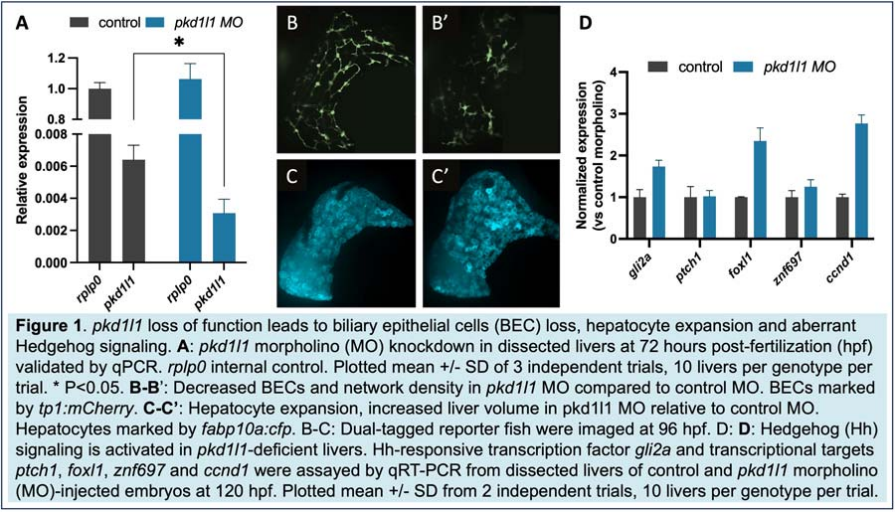


## 599 CHILDREN WITH ANTRAL HYPOMOTILITY OR SMALL BOWEL DYSMOTILITY HAVE A GREATER RESPONSE TO GASTRIC ELECTRICAL STIMULATION

*Eduardo Castillo Leon* ^*1*^, *Amina Usman* ^*1*^, *Md‐Rejuan Haque* ^*2*^, *Karen Diefenbach* ^*3*^, *Raul Sanchez* ^*1*^, *Neetu Bali Puri* ^*1*^, *Karla Vaz* ^*1*^, *Desale Yacob* ^*1*^, *Carlo Di Lorenzo* ^*1*^, *Peter Lu* ^*1*^

^*1*^ *Gastroenterology*, *Nationwide Children's Hospital*, *Columbus*, *OH*; ^*2*^ *Department of Biomedical Informatics, Center for Biostatistics*, *The Ohio State University*, *Columbus*, *OH*; ^*3*^ *Center for Colorectal and Pelvic Reconstruction*, *Nationwide Children's Hospital*, *Columbus*, *OH*

**Background:** Gastric electrical stimulation (GES) improves symptoms and quality of life in children with refractory nausea and vomiting. However, characteristics of suitable patients for GES remain unclear. Our objective was to evaluate whether antroduodenal manometry (ADM) can predict response to GES.

**Methods:** We completed a prospective cohort study. We identified patients <21 years old who underwent GES placement at our institution between 2011‐2024 who had ADM before GES. Encounters were selected at baseline before GES and at follow‐up at 1 week, 2 months, 6 months, and 12 months. Patients completed the Symptom Monitor Worksheet (SMW) and Pediatric Quality of Life Inventory (PedsQL) at each encounter. ADM results were categorized as antral hypomotility, enteric neuropathy, or normal based on review of reports and/or manometry tracings. Clinical response to GES was defined as >1 point improvement in average SMW score from baseline to last follow up. We used a linear mixed effects regression model to assess changes during the first year.

**Results:** We included 48 patients with a median age of 14.9 years old (Q1‐Q3, 11–17.1) who were mostly female (68.7%) and White (95.8%). At baseline, 83.3% had a diagnosis of gastroparesis, 47.7% had delayed gastric emptying found on a gastric emptying study, 46.6% had functional dyspepsia, 35.4% had postural orthostatic tachycardia syndrome, and 72.9% had anxiety or depression. At baseline, 71.8% were receiving supplemental enteral feeds and 15.6% were receiving parenteral nutrition. Twenty‐nine (60.4%) patients had abnormal ADM results; 15 (31.2%) had antral hypomotility and 17 (35.4%) had enteric neuropathy, with 3 patients having both. At baseline, there were no significant differences in patient characteristics between patients with antral hypomotility and normal ADM. However, patients diagnosed with enteric neuropathy were more likely to have a diagnosis of pediatric intestinal pseudo‐obstruction and were more likely to have a diagnosis of gastroparesis, although likelihood of having a documented delay in gastric emptying was similar between groups (35.2% vs 52.6%, respectively) (**Table**). During the first year of GES treatment, patients with antral hypomotility (p=0.005) or enteric neuropathy (p=0.01) experienced statistically significant improvement in their total SMW score by the end of the year (**Figure**). Patients with normal ADM showed improvement in their SMW score, but this was not statistically significant (p=0.19). All three groups improved in quality of life by the end of the year. Nineteen of 29 patients with either antral hypomotility or enteric neuropathy met criteria for clinical response with GES by their last follow‐up while only 5/14 patients with normal ADM met criteria (p=0.04).

**Conclusion:** Children with refractory nausea and vomiting and abnormal ADM with either antral hypomotility or enteric neuropathy are more likely to experience greater symptom response with GES when compared to children with normal ADM.


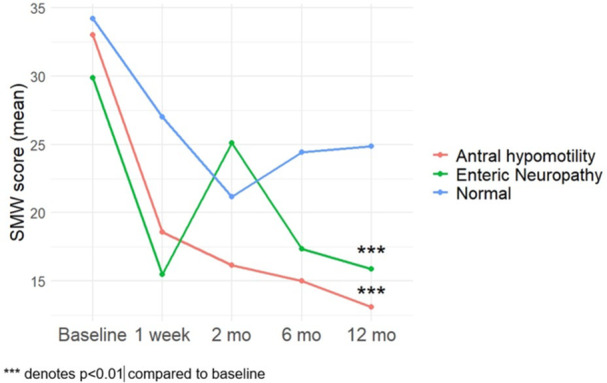


**Figure.** Line graphs show the mean value of the Symptom Monitor Worksheet (SMW) at every encounter grouped by antroduodenal manometry results.


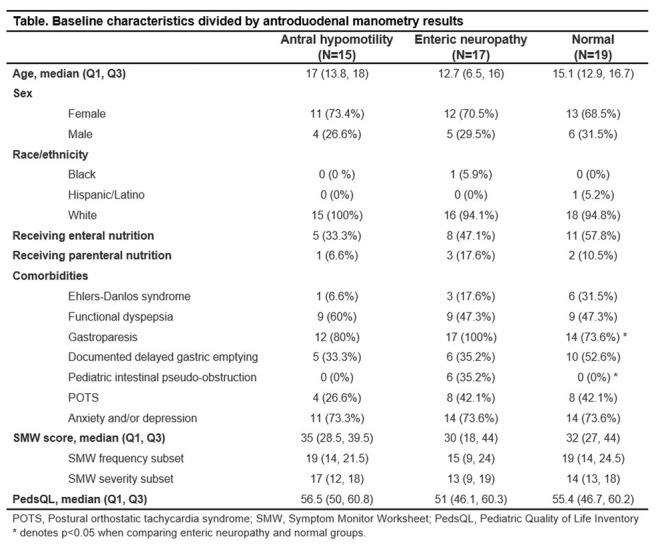


Table. Baseline characteristics divided by antroduodenal manometry results

## 600 ENTEROCYTES REGULATE HEPATIC BILE ACID SYNTHESIS IN CO‐CULTURED GUT AND LIVER ORGANOIDS WITH INTERRUPTION UPON SIRNA TRANSFECTION

*ASHLESHA BAGWE*, *Chandrashekhara Manithody*, *Kento Kurashima*, *Shaurya Mehta*, *Sree Kolli*, *Miguel Guzman*, *Uthayashanker Ezekiel*, *Ajay Jain*

*Pediatrics*, *Saint Louis University School of Medicine*, *Saint Louis*, *MO*

**Background:** Intestinal failure‐associated liver disease (IFALD) is a major complication of long‐term parenteral nutrition, characterized by cholestasis and bile acid dysregulation. Fibroblast growth factor 19 (FGF19), secreted from enterocytes in response to farnesoid X receptor (FXR) activation, is a key regulator of hepatic CYP7A1 and bile acid synthesis. To explore gut‐liver crosstalk in this context, we developed a human enteroid–liver organoid co‐culture system and integrated FXR‐targeted siRNA studies using porcine enteroids.

**Methods:** Human small bowel–derived 3D enteroids were co‐cultured with human liver organoids using a transwell platform to mimic the gut‐liver axis. FXR signaling was induced using CDCA (25–50 µM), and hepatic CYP7A1 expression was measured. Parallel experiments with porcine enteroids utilized FXR‐targeted DsiRNA transfection and CDCA treatment. FXR expression, bile acid uptake, and efflux were quantified via real‐time PCR and functional assays.

**Results:** CDCA‐treated human enteroids upregulated FXR (~2X), which corresponded with suppression of CYP7A1 in co‐cultured liver organoids, validating an enterocyte‐driven hepatic response. In porcine enteroids, CDCA produced a dose‐dependent increase in FXR mRNA (up to 2.8‐fold), enhanced nuclear localization, increased bile acid uptake, and decreased efflux. FXR knockdown using siRNA resulted in 68% expression reduction, impaired bile acid uptake, and increased efflux, confirming FXR's central role in regulating bile acid dynamics.

**Conclusion:** Our human enteroid–liver organoid co‐culture system models key molecular events of enterohepatic communication and reveals how intestinal FXR signaling modulates hepatic bile acid metabolism. These findings underscore the gut's active regulatory role in IFALD pathogenesis and highlight FXR as a potential therapeutic target for restoring bile acid homeostasis in liver disease.

## 601 PEDIATRIC‐SPECIFIC HIGH‐RESOLUTION ESOPHAGEAL MANOMETRY PROTOCOLS ARE ESSENTIAL: EVIDENCE FROM A MULTICENTER COHORT STUDY

*Trevor Davis* ^*1,3*^, *Benjamin Rogers* ^*2*^, *Alejandro Llanos Chea* ^*4*^, *Amornluck Krasaelap* ^*5*^, *Darnna Banks* ^*6*^, *Lusine Ambartsumyan* ^*5*^, *Raul Sanchez* ^*7*^, *Corey Baker* ^*8*^, *ADRIANA PRADA REY* ^*9*^, *Chaitri Desai* ^*10*^, *Aaron Rottier* ^*2*^, *Mayuri Jayaraman* ^*11*^, *Camila Khorrami* ^*12*^, *Lev Dorfman* ^*13*^, *Khalil El‐Chammas* ^*13*^, *Sherief Mansi* ^*13*^, *Eric Chiou* ^*14*^, *Bruno Chumpitazi* ^*15*^, *Neetu Bali Puri* ^*7*^, *Leonel Rodriguez* ^*16*^, *Jose Garza* ^*17*^, *Miguel Saps* ^*18*^, *Dhiren Patel* ^*11*^, *C. Prakash Gyawali* ^*3*^

^*1*^ *Pediatric Gastroenterology*, *Washington University School of Medicine*, *St. Louis*, *MO*; ^*2*^ *University of Louisville*, *Louisville*, *KY*; ^*3*^ *Washington University in St Louis School of Medicine*, *St. Louis*, *MO*; ^*4*^ *The University of Texas Southwestern Medical Center*, *Dallas*, *TX*; ^*5*^ *Seattle Children's Hospital*, *Seattle*, *WA*; ^*6*^ *Ochsner Health*, *New Orleans*, *LA*; ^*7*^ *Nationwide Children's Hospital*, *Columbus*, *OH*; ^*8*^ *Connecticut Children's Medical Center*, *Hartford*, *CT*; ^*9*^ *Universidad El Bosque*, *Bogotá*, *Bogota*, *Colombia*; ^*10*^ *Children's Mercy Kansas City*, *Kansas City*, *MO*; ^*11*^ *Saint Louis University School of Medicine*, *St. Louis*, *MO*; ^*12*^ *Washington State University*, *Pullman*, *WA*; ^*13*^ *Cincinnati Children's Hospital Medical Center*, *Cincinnati*, *OH*; ^*14*^ *Texas Children's Hospital*, *Houston*, *TX*; ^*15*^ *Duke Children's Hospital and Health Center*, *Durham*, *NC*; ^*16*^ *Yale School of Medicine*, *New Haven*, *CT*; ^*17*^ *Emory University*, *Atlanta*, *GA*; ^*18*^ *Miami University*, *Oxford*, *OH*

**Background:** High‐resolution manometry (HRM) study quality, artifacts, positioning of markers, utilization of provocative maneuvers and resultant findings can alter the final motility diagnosis. We evaluated the impact of centralized interpretation of de‐identified pediatric HRM studies from US motility centers.

**Methods:** Clinical and manometric characteristics of children undergoing esophageal HRM during 2021‐2022 were collected from 12 pediatric motility centers across the US. Each center submitted corresponding deidentified HRM studies for centralized interpretation, and provided their interpretation when possible. Each study was blinded prior to centralized interpretation and reviewed in a swallow‐by‐swallow manner to collect motor metrics, study adequacy, presence of artifacts, Chicago Classification (CC) version 4.0 diagnosis, adherence to CC protocol, and use of provocative/supportive testing.

**Results:** From 281 pediatric HRM studies, 260 were de‐identified and randomized for blinded review (median age 15.0 years, 62.7% female, body mass index 20.8 kg/m^2^). The remaining 21 HRM studies were missing and thus excluded. The majority (190, 73.1%) utilized Laborie, while the remainder were Medtronic studies (70, 26.9%). Of these, 88 (33.8%) studies were deemed optimal for interpretation. Of 172 (66.2%) studies with at least one non‐critical artifact, 123 (71.5%) had double swallows, 120 (69.8%) had short intervals between swallows, 12 (7.0%) had an inadequate gastric baseline, 5 (2.9%) poor UES visualization, and 10 (5.8%) had an incomplete supine swallow complement. The complete CCv4.0 protocol was followed in only 15 of 260 patients (5.8%). Centralized analysis changed interpretation to a non‐actionable diagnosis (i.e., normal HRM or IEM) more often than to an actionable diagnosis (i.e., achalasia, EGJOO) (p<0.01). Among studies that included MRS (200, 76.9%) only 53 (20.4%) had all three MRS performed. Of those with at least one multiple rapid swallow (MRS) sequence, 150 (57.7%) demonstrated contraction reserve. Rapid drink challenge (RDC) was performed in 18 patients (6.9%) and 38 (14.6%) underwent upright swallows, although paste and/or cookie swallows were much more common (153, 58.8%). The diagnosis of rumination was made in 19 (7.3%). Relevant provocative maneuvers were incomplete in 77 (29.6%), predominantly from inadequate MRS (15, 19.5 %), no MRS (9, 11.7%) no RDC (17, 22.1%), no upright swallows (1, 1.2%), and no upright swallows or RDC (35, 45.5%).

**Conclusions:** The current CC protocol is not followed in the majority of pediatric HRM studies. Artifacts are common, but non‐critical, and interpretation is mostly accurate. Changes in diagnosis from blinded centralized interpretation were often toward non‐actionable diagnoses and normal manometry. Missed provocative/supportive maneuvers were observed in approximately one‐third of interrogated studies. Our findings indicate a need for pediatric‐specific manometric protocols to ensure uniform diagnostic evaluation.

## 602 IMPACT OF PEDIATRIC INTESTINAL ULTRASOUND ON COLONOSCOPY UTLIZATION IN PATIENTS WITH SUSPECTED INFLAMMATORY BOWEL DISEASE: A RETROSPECTIVE COHORT STUDY FROM A TERTIARY CENTER IN FLORIDA

*Christian Martinez* ^*1*^, *Monica Ramirez* ^*1*^, *Gabriel Cardenas* ^*2*^, *Lina Maria Felipez Marrero* ^*3*^, *Luis Caicedo* ^*3*^

^*1*^ *Pediatrics*, *Nicklaus Children's Hospital*, *Miami*, *FL*; ^*2*^ *Research*, *Nicklaus Children's Hospital*, *Miami*, *FL*; ^*3*^ *Pediatric Gastroenterology*, *Nicklaus Children's Hospital*, *Miami*, *FL*

**Background:** Intestinal ultrasound (IUS) is an emerging noninvasive imaging tool increasingly utilized in pediatric inflammatory bowel disease (IBD) management. While evidence suggests IUS can reduce reliance on invasive procedures such as colonoscopy, data quantifying clinical and economic impacts of IUS in pediatric settings are limited.

**Objective:** To evaluate the impact of pediatric IUS implementation on colonoscopy utilization, provider‐specific procedural changes, and associated cost implications at a tertiary pediatric gastroenterology center.

**Methods:** A retrospective pre/post panel design assessed colonoscopy utilization in inpatient and outpatient pediatric patients with suspected IBD across two matched 9‐month periods: pre‐IUS (8/1/2023–4/30/2024) and post‐IUS implementation (8/1/2024–4/30/2025). IUS was utilized based on clinical judgment for suspected IBD diagnosis, monitoring disease activity, or evaluation of complications. An additional subgroup analysis evaluated the odds of undergoing colonoscopy among patients seen by the two IUS utilizers (Providers 1 and 2) before and after IUS implementation. Physician paired t‐tests compared total encounters pre‐ and post‐IUS implementation to control for encounter volume differences. Odds ratios (OR) assessed the odds of undergoing colonoscopy pre‐ versus post‐IUS. The charge cost for colonoscopy ($3,211/procedure) was used to calculate return on investment (ROI).

**Results:** Total colonoscopy procedures declined significantly from 207 (pre‐IUS) to 127 (post‐IUS) with a 38.65% reduction. The odds of undergoing colonoscopy post‐IUS implementation significantly decreased (OR 0.58; 95% CI: 0.46–0.72) among all 7 pediatric gastrointestinal (GI) providers. In addition, IUS utilizing providers revealed a significant reduction in the odds of undergoing colonoscopy post‐IUS implementation (OR 0.68; 95% CI: 0.50–0.94). Together, they reduced colonoscopies from 97 to 68 (−30%), performing 443 IUS exams. Intestinal ultrasound implementation reduced colonoscopy rates from 2.02% to 1.18% (Absolute Risk Reduction 0.84%), resulting in a Number Needed to Scan (NNS) of 119, and approximately 6 ultrasounds performed for each colonoscopy avoided. Economically, actual expenditures for 443 IUS exams totaled $123,154, compared to hypothetical colonoscopy costs of $1,422,668, yielding total cost savings of approximately $1,299,514 (91.34%). ROI analysis revealed that every $1 invested in IUS resulted in approximately $10.55 in savings (ROI = 1055%). Physician paired t‐tests comparing total encounters between groups showed no statistically significant difference in patient encounters pre‐ (mean ± SD: 1463.43 ± 284.96) versus post‐IUS (mean ± SD: 1537.43 ± 217.48; p = 0.33).

**Discussion:** IUS implementation led to a substantial, statistically significant reduction in colonoscopy utilization across all GI providers. The analysis demonstrated significantly lower odds of colonoscopy use among IUS‐utilizing providers, further validating the clinical impact of IUS in clinical decision‐making. This significant reduction underscores a shift toward less invasive monitoring strategies, aligning with existing literature supporting IUS as an accurate, patient‐friendly, and cost‐effective modality in pediatric IBD management. The economic analysis underscores that integrating intestinal ultrasound may optimize hospital resources and enhance patient‐centered care. Additionally, improvements in patient comfort, procedural anxiety, and overall patient experience associated with IUS versus invasive procedures warrant further exploration. Future prospective studies should evaluate clinical outcomes such as disease remission rates or therapeutic decision‐making influenced directly by IUS findings, as well as specific patient‐level factors influencing decisions to utilize IUS versus colonoscopy.

Limitations include the retrospective, single‐center design which may limit generalizability and introduce selection bias. Diagnostic accuracy and therapeutic implications were not directly assessed, necessitating future prospective studies. Procedural and patient‐level characteristics that may influence decision‐making were not explored in depth.

**Conclusion:** The introduction of pediatric intestinal ultrasound significantly reduced colonoscopy utilization and demonstrated substantial cost savings with an exceptional return on investment. These findings support broader adoption of IUS in pediatric gastroenterology, highlighting its potential to decrease procedural burdens and healthcare costs. Further research should aim to refine patient selection criteria and identify optimal strategies for integrating IUS into clinical practice to maximize both clinical and economic outcomes.

## 603 GLUCAGON‐LIKE PEPTIDE‐1 AGONISM AMELIORATES COLONIC INFLAMMATION IN A MURINE MODEL OF ULCERATIVE COLITIS

*Biren Desai* ^*1,2*^, *Kendra Francis* ^*1,2*^, *Caeley Bryan* ^*2*^, *Kelly Kadlec* ^*2*^, *Jessie Connolly* ^*2*^, *Thanushree Karunagaran* ^*2*^, *Maria Cristina Pacheco* ^*3*^, *Gregory Morton* ^*2*^, *Michael Schwartz* ^*2*^, *Jarrad Scarlett* ^*1,2*^

^*1*^ *Pediatric Gastroenterology*, *Seattle Children's Hospital*, *Seattle*, *WA*; ^*2*^ *Diabetes Institute*, *University of Washington*, *Seattle*, *WA*; ^*3*^ *Department of Labratories*, *Seattle Children's Hospital*, *Seattle*, *WA*

**Introduction:** Inflammatory Bowel Disease (IBD) is a chronic inflammatory disorder of the gastrointestinal (GI) tract that is increasing in prevalence worldwide. The etiology is considered multifactorial, involving interactions between genetic pre‐disposition, abnormalities with the intestinal/colonic microbiome, environmental/food triggers, and intestinal immune dysregulation causing disruption of the intestinal barrier that progresses to chronic mucosal injury. Current IBD therapies act primarily by suppressing the immune system, but in doing so increase the risk of infections and neoplastic complications. Therefore, an unmet need exists to identify novel therapies that treat IBD that do not suppress the immune system. Glucagon‐Like Peptide‐1 receptor agonist drugs (GLP‐1RAs) have emerged as highly efficacious medical treatments of obesity and type 2 diabetes, conditions associated with heightened risk of IBD. More recently, the anti‐inflammatory effects of these drugs have been linked to improved cardiovascular, renal, and hepatic complications of these disorders. In the current work, we sought to determine if GLP‐1RAs have salutary effects on IBD, as suggested by epidemiologic evidence of improved IBD outcomes in IBD patients taking GLP‐1RAs. To this end, we tested whether the GLP‐1RA Semaglutide impacts colitis severity in the murine dextran sodium sulfate (DSS) model of ulcerative colitis.

**Methods:** After wild‐type male C57BL/6 J mice were placed on a high‐fat diet (HFD) for 8 weeks to produce diet induced obesity (DIO), they underwent subcutaneous implantation of an osmotic minipump containing either Semaglutide (Sema, 0.04 mg/kg) or saline vehicle (veh). Food intake, blood glucose, and body weight measurements were monitored daily until weight loss in the Sema treatment groups plateaued. At 14 days, mice were started on 2% DSS water or control drinking water (veh) for 10 days to induce colitis, yielding four treatment groups: veh/veh, sema/veh, veh/DSS, and sema/DSS (n= 6‐12/group). Body weight, food and water intake, and clinical signs of IBD (amount of weight loss, rectal bleeding, and stool consistency, range of 0‐8) were monitored daily. Animals were euthanized after 10 days of DSS treatment, and colon length and spleen weight were recorded. Colon tissue was swiss‐rolled, formalin fixed, embedded in paraffin, and mounted onto slides. Statistical significance was determined by one‐way ANOVA followed by Tukey post‐hoc‐testing.

**Results:** By design, sema treatment resulted in weight loss compared to veh groups (sema/DSS vs veh/DSS: ‐18%, P <0.01 and sema/veh vs veh/veh: ‐18% 2‐way Anova Tukey post‐hoc, P = 0.023) that was associated with a pronounced reduction of food intake (sema/DSS: 1.075 g/mouse/d vs. veh/DSS: 2.35 g/mouse/d, 2‐way Anova Tukey post‐hoc, P <0.0001. Sema/veh: 0.84 g/mouse/d vs. veh/veh 2.32 g/mouse/d, 2‐way Anova Tukey post‐hoc, P = 0.0019). After Semaglutide induced weight loss plateaued (Day 14), DSS was initiated for an additional 10 days showing comparable weight loss (veh/DSS: ‐7.36% vs sema/DSS: ‐7.91%, P = 0.99) and suppression of food intake in both groups. However, clinical IBD scores were markedly reduced among mice in the sema/DSS group (sema/DSS: 4.16 vs veh/DSS: 7.08, P < 0.0001; as expected, colitis was not detected in the veh/veh or sema/veh groups). Pathologic markers of disease activity including colon length, which shortens in response to mucosal inflammation, also showed lower disease activity in the sema/DSS vs. veh/DSS group (8.19 cm vs. 6.85 cm, P < 0.0001). Unexpectedly, colon length was increased by treatment with sema alone compared to veh/veh groups that did not receive DSS (sema/veh: 9.18 cm vs. veh/veh: 7.88 cm, P = 0.0037). The spleen‐weight‐to‐body‐weight ratio, which increases in response to systemic inflammation, was also reduced among DSS mice treated with sema (sema/DSS: 3.51 mg/g10^‐3^ vs. veh/DSS: 4.33 mg/g10^‐3^ body weight ratio, P <0.0001). Histologic damage scoring in colon tissues and biochemical analysis of intestinal and systemic inflammatory burden are pending.

**Discussion:** Our results show that in the DSS mouse model, colitis severity is robustly blunted by administration of a GLP‐1RA drug. Specifically, continuous subcutaneous Semaglutide administration significantly improved clinical and pathological IBD outcomes over a 10‐day course of oral DSS administration. Although additional study is needed to control sema‐induced weight loss prior to DSS, the beneficial effect of sema was not associated with differences of food intake or body weight during DSS administration. One potential mechanism to explain the protective effect of GLP‐1RA agonism is that it suppresses DSS induced activation of natural killer T cells and intraepithelial lymphocytes in the intestine. These findings offer compelling preliminary support for a clinical trial to establish efficacy and safety of GLP‐1RA drugs for IBD patients, particularly those with type 2 diabetes or obesity.

## 604 CHILDREN WITH SICKLE CELL DISEASE HOSPITALIZED AT CHILDREN'S HOSPITALS WITH ACUTE PANCREATITIS HAVE A LONGER LENGTH OF STAY AND REQUIRE MORE PROCEDURES COMPARED TO THOSE WITHOUT SICKLE CELL DISEASE

*Olawale Oduru* ^*1*^, *Fazlur Rahman* ^*2*^, *Kondal Kyanam Kabir Baig* ^*2*^, *Ali Ahmed* ^*2*^, *Saskia D'Sa* ^*1*^, *Shaundra Blakemore* ^*1*^, *Chinenye Dike* ^*1*^

^*1*^ *Gastroenterology*, *Children's of Alabama*, *Birmingham*, *AL*; ^*2*^ *University of Alabama at Birmingham Health System*, *Birmingham*, *AL*

**Background:** Sickle cell disease (SCD) affects about 100,000 Americans, particularly those with African ancestry. Children with SCD are at significantly higher risk of developing gallstones‐one of the most common etiologies of acute pancreatitis (AP). We hypothesized that children with SCD admitted to children's hospitals for AP will have an increased prevalence, length of stay (LOS), and need for procedures compared to those without SCD.

**Aims:** i) determine the prevalence of SCD in a cohort of children admitted with AP in several children's hospitals ii) Evaluate the LOS in children with SCD hospitalized with AP compared to those without SCD iii) Assess the need for procedures, and transfusions in children with SCD admitted with AP compared to those without SCD.

**Methods:** Retrospective study of children 0 ‐ 21 years admitted with a diagnosis of AP in the pediatric health information system database from 2012 to 2023. We excluded children with co‐morbidities and those with complex medical conditions. We used descriptive statistics to describe baseline demographics and prevalence. Rates of categorical variables such as readmissions within or after 30 days, transfusions, need for procedures (ERCP/EUS and cholecystectomy) were compared between SCD vs non SCD groups using chi‐square or Fisher's exact test as appropriate. Wilcoxon test was used to compare the median age and LOS between groups. All analyses were performed using SAS version 9.4 software (SAS Institute, Inc.; Cary, NC) and a p‐value <0.05 was considered statistically significant.

**Results:** A total of 18,213 distinct subjects (17,927 (non SCD) and 286 (SCD) children were included in the analysis for a total of 23,265 encounters (22908 (non SCD) and 357 (SCD). The prevalence rate of SCD in this cohort was ~ 2%. The non SCD and SCD cohorts did not differ by sex. Children in the SCD cohort were older (median age 14 years (IQR‐ 10‐17 years)) than the non SCD cohort (median age 13 years (IQR 9‐16 years) (p=0.0004). Most children in the SCD cohort were Non‐Hispanic Black (88%) while 60% of the non SCD cohort were non‐Hispanic White (p=<0.0001). Children in the SCD cohort had a longer LOS (median of 5 days (IQR: 3‐9 days)) compared to children in the non SCD cohort (median LOS of 4 days (IQR: 2‐6 days)) (p=< 0.0001). There was an increased percentage of ERCP/EUS performed in the SCD cohort 15% vs 1% in the non SCD cohort (p=<0.0001). Children in the SCD cohort had a higher rate of cholecystectomy 23% vs 3% in the non SCD cohort (p=<0.0001). Also, children in the SCD cohort received more pRBC transfusions at 3% compared to the non SCD cohort at 0% (p=<0.001). There were no differences in the readmission rates between the groups.

**Conclusion:** Children with SCD hospitalized with AP are older, have a prolonged LOS and require more pRBC transfusions, procedures including ERCP/EUS, and cholecystectomy when compared to children without SCD. Further studies aimed at reducing the burden of AP in children with SCD are desperately needed.


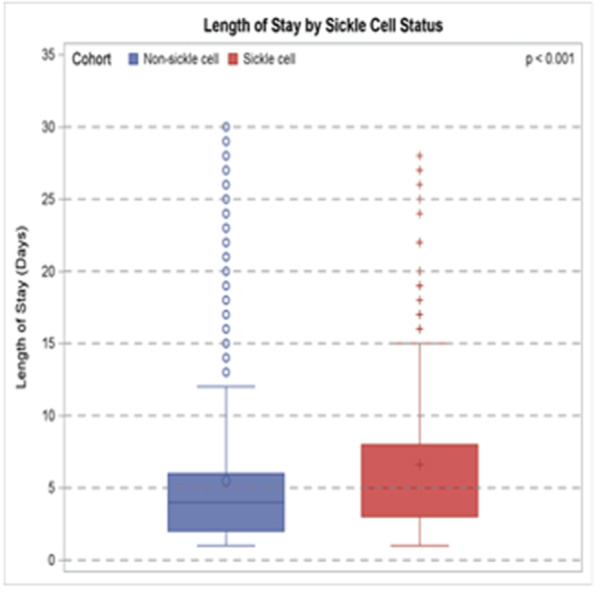



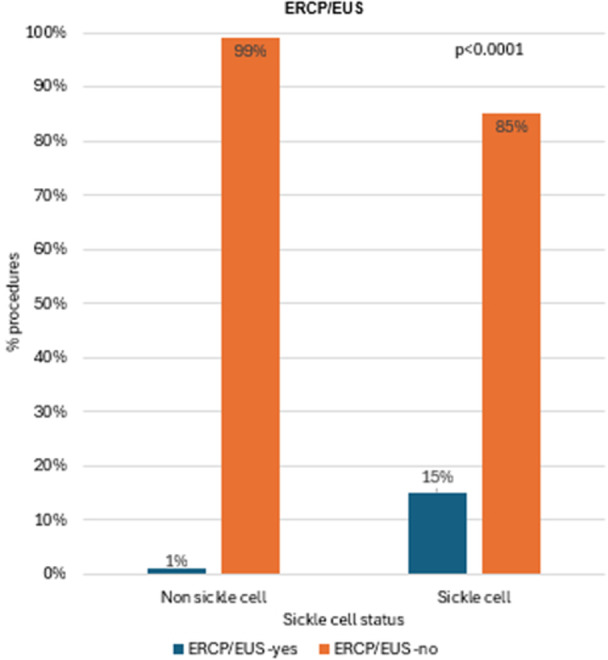


## 605 USING MACHINE LEARNING TO PREDICT CHILDHOOD BMI FROM DATA FROM THE FIRST 1000 DAYS OF LIFE

*Aamer Imdad* ^*1*^, *Uzma Rani* ^*1*^, *Samantha Pothitakis* ^*2*^, *Yash Vora* ^*2*^, *William Story* ^*3*^, *Donna Santillan* ^*4*^, *Yanan Liu* ^*5*^

^*1*^ *Stead Family Department of Pediatrics*, *University of Iowa Health Care*, *Iowa City*, *IA*; ^*2*^ *Computer Science*, *University of Iowa Health Care*, *Iowa City*, *IA*; ^*3*^ *College of Public Health*, *University of Iowa Health Care*, *Iowa City*, *IA*; ^*4*^ *Obstetrics and Gynecology*, *University of Iowa Health Care*, *Iowa City*, *IA*; ^*5*^ *Iowa Institute of Artificial Intelligence*, *University of Iowa Health Care*, *Iowa City*, *IA*

**Background:** Childhood obesity affects approximately 1 in 5 children and adolescents in the United States. Once obesity develops, it is challenging to reverse, and even when reversed, it often recurs, posing significant treatment challenges. A major barrier to prevention is the limited understanding of the factors that predispose children to obesity, particularly during the first 1,000 days of life, from conception to two years of age. Our objective was to design a novel predictive deep machine learning algorithm utilizing data from the first 1,000 days of life to predict body mass index (BMI) at 3 years of age.

**Methods:** Our primary exposures included factors from the first 1,000 days of life, and the outcome was BMI at 3 years of age. We utilized a merged dataset comprising over 30,000 patients from the University of Iowa's Intergenerational Health Knowledgebase and electronic health records. We had health information from 40,000 variables from the first 1,000 days of life and that included information on maternal pre‐pregnancy health, pregnancy health including information on morbidities such as gestational diabetes, pre‐eclampsia, smoking, gestational weight gain, and information for newborn including mode of delivery, birthweight, and information from first two years of life such as breastfeeding, formula feeding, complementary feeding, medication such as antibiotics. Missing values were imputed using mean imputation for numerical variables and mode imputation for categorical variables, preserving as much information as possible while maintaining consistency across the dataset. In the first phase, feature selection was conducted using a pipeline that combined least absolute shrinkage and selection operator (LASSO) regression, univariate testing, and domain expertise (Figure 1). In the second phase, two Support Vector Regression (SVR) models with Radial Basis Function (RBF) kernels were trained: one utilizing 90 features and the other using a clinically curated subset of 14 features. Model performance was assessed through 5‐fold cross‐validation, and results are reported with test set mean absolute error (MAE).

**Results:** The final sample size, after applying exclusion criteria, was 13,000 patients. The 90‐feature model achieved a test mean absolute error (MAE) of 0.7726 (SD = 0.6564), while the 14‐feature model (table) performed comparably with an MAE of 0.7866 (SD = 0.6799). MAE represents the average absolute difference between the predicted and actual BMI values; in this context, an MAE of approximately 0.78 indicates that, on average, the model's BMI predictions were within 0.78 BMI units of the true values. Shapley Additive Explanations (SHAP) analysis identified prior BMI at ages 1 and 2, birth weight, and maternal health indicators, such as obesity, as the most influential predictors of BMI at age 3.

**Conclusions:** Data from the first 1,000 days of life were predictive of BMI at 3 years of age, with the most predictive 14 features that included potentially modifiable features such as infant nutrition, use of antibiotics during infancy and pregnancy, and maternal health, such as obesity. This study sets the stage for future studies that should focus on improving predictions for the dichotomous outcome of obesity with the goal of finding clinically meaningful and modifiable risk factors for the prevention of childhood obesity.


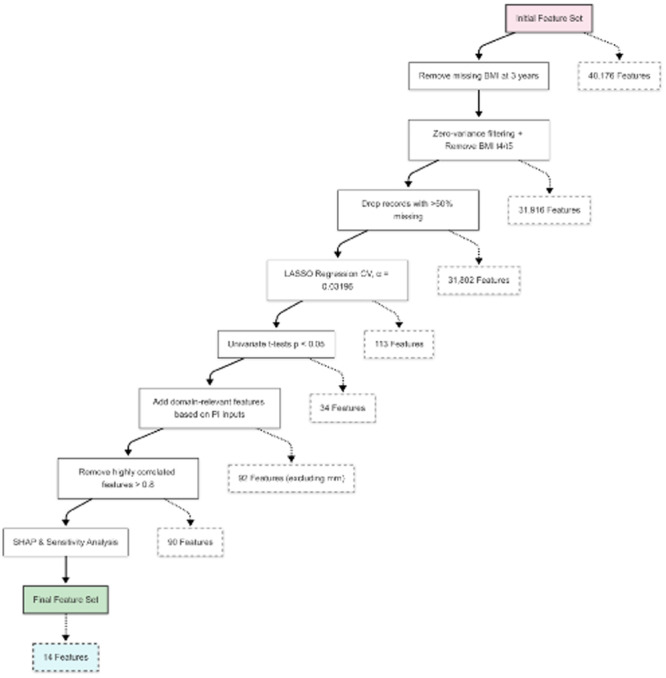



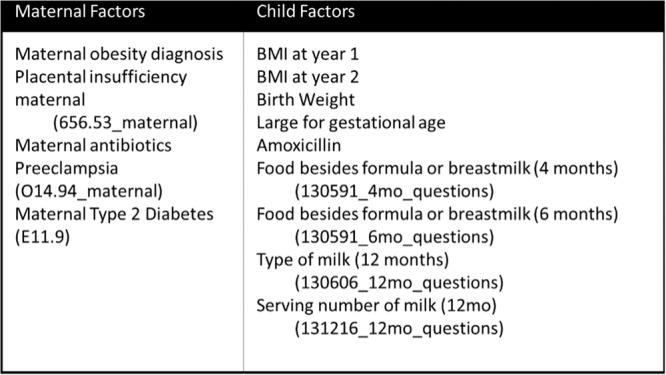


## 606 CHAMPIONING GENDER EQUITY: ADJUSTING OUTPATIENT PRODUCTIVITY TARGETS FOR LACTATING PHYSICIANS

*Lisa Fahey* ^*1,2*^, *Lauren Boldizar* ^*3*^, *Pinney Sara* ^*3,2*^, *Michelle DeLucia* ^*3*^, *Angela Ellison* ^*3,2*^, *Patricia DeRusso* ^*1,2*^

^*1*^ *Pediatrics; Division of Gastroenterology*, *The Children's Hospital of Philadelphia*, *Philadelphia*, *PA*; ^*2*^ *Perelman School of Medicine*, *University of Pennsylvania*, *Philadelphia*, *PA*; ^*3*^ *The Children's Hospital of Philadelphia*, *Philadelphia*, *PA*

**Background:** Breastfeeding in academic medicine is challenging. Significant obstacles include lack of time to pump, decreased productivity and decreased pay for many physicians that pump.

To address this inequity, a few healthcare organizations implemented lactation programs to protect time and adjust outpatient productivity targets for clinicians to accommodate pumping without penalty. The Children's Hospital of Philadelphia (CHOP) implemented a similar program for general pediatricians, but there was an opportunity to support subspecialists.

**Objective:** The aims were to 1) conduct a lactation needs assessment to determine opportunities to further support breastfeeding attending physicians as it relates to protected time needed to pump, productivity targets, and incentive opportunities; 2) Design a lactation program to address ongoing needs including time and financial disparity, in order to support gender equity, diversity and inclusion. 3) Conduct a 6‐month post‐launch assessment of the new outpatient lactation program

**Methods:** We examined the existing CHOP employee lactation policy, surveyed division leadership and physician well‐being champions across all Departments and conducted qualitative interviews in Fall 2023 to assess opportunities to further support breastfeeding attending physicians. We also engaged with key stakeholders including our Human Resources, Finance and Legal teams. We then designed a lactation program to reduce clinical effort and adjust productivity targets for up to 12 months post‐childbirth for CHOP outpatient attending physicians who express breastmilk. Specifically, there is now a 30‐ minute clinical effort reduction per 4‐hour outpatient clinical session, and the productivity target within the incentive is adjusted in order to protect the incentive target. The program was launched in July 2024. A 6‐month post‐launch assessment was then conducted to assess the impact of the program.

**Results:** *Pre‐Program Launch Survey Data*: Prior to the lactation program launch, the existing CHOP lactation policy did not address clinical productivity targets for attending physicians. In addition, pre‐ launch data indicated that 65% (26/41) reported protected time for lactating physicians to pump (69% protect time as needed, 8% protect time every 4 hours, 4% protect time every 3 hours and 19% had no set process). 82% (22/41) reported that the annual productivity targets and incentive targets were NOT adjusted to account for time needed to pump. 85% reported that there was a specific space in the clinical area designated for pumping.

*6‐month Post‐Program Launch Survey Data*: The six‐month post‐program launch survey data indicated that 71% of attending physicians and 100% of division leadership teams were aware of the new lactation program. 92% of divisions implemented the new program, including the gastroenterology division. Protected pumping time improved from 65% to 83%, with 90% of divisions reporting existence of a specific process to adjust schedules to protect time to pump (from 52% pre‐program launch).

91% of division leadership and 77% of attending physicians reported adjusted productivity targets to account for time needed to pump. 90% of division leadership and 24% of attending physicians reported adjusting the productivity target within the incentive opportunity for time needed to pump in order to protect the incentive opportunity. 100% of division leadership reported that the new lactation program has helped to provide additional support for outpatient attendings who are pumping.

**Conclusions & Practical Implications:** Most CHOP breastfeeding attending subspecialists did not have an adjustment in their annual productivity targets or incentive opportunities to account for time needed to pump prior to initiation of the lactation program. This highlights a significant disparity for this group of female physicians. Additionally, the financial inequity primarily applied to physicians in the outpatient setting. Furthermore, many physicians noted during qualitative interviews that they stopped pumping soon after returning to work due to time constraints. The new outpatient attending lactation program is a financially sustainable program. Ongoing education is needed to raise awareness about the program.

In conclusion, supporting breastfeeding is critically important for gender equity and diversity, can increase an organization's retention of physician parents, and helps to create a more supportive environment for physicians.

## 607 EFFECTS OF BILE FLOW ON THE DEVELOPMENT OF THE GUT MICROBIOME IN INFANTS WITH BILIARY ATRESIA

*Mary Elizabeth Tessier* ^*1*^, *Laurel Cavallo* ^*1*^, *Cynthia Tsai* ^*1*^, *Niviann Blondet* ^*2*^, *Christopher Gayer* ^*3*^, *Stephen Guthery* ^*4*^, *Kathleen Loomes* ^*5*^, *Kyla Tolliver* ^*6*^, *James Squires* ^*9*^, *Shikha Sundaram* ^*7*^, *Joseph Petrosino* ^*8*^, *Benjamin Shneider* ^*1*^

^*1*^ *Pediatrics, Division of Pediatric Gastroenterology, Hepatology and Nutrition*, *Baylor College of Medicine Department of Pediatrics*, *Houston*, *TX*; ^*2*^ *Seattle Children's Hospital*, *Seattle*, *WA*; ^*3*^ *Pediatric Surgery*, *Children's Hospital Los Angeles*, *Los Angeles*, *CA*; ^*4*^ *University of Utah Health*, *Salt Lake City*, *UT*; ^*5*^ *The Children's Hospital of Philadelphia*, *Philadelphia*, *PA*; ^*6*^ *Indiana University School of Medicine*, *Indianapolis*, *IN*; ^*7*^ *Children's Hospital Colorado*, *Aurora*, *CO*; ^*8*^ *Molecular Virology & Microbiology*, *Baylor College of Medicine*, *Houston*, *TX*; ^*9*^ *UPMC*, *Pittsburgh*, *PA*

**Background:** In biliary atresia (BA), a fibro‐obliterative disorder of the extrahepatic bile duct of infants, intestinal bile flow can be restored with a Kasai portoenterostomy (KP). However, only about 50% of infants undergoing KP have successful clearance of jaundice (COJ), characterized by a total bilirubin (TB) <2 mg/dL by 3 months after KP. Our previous pilot single‐center microbiome data (n=8) suggested that COJ vs not after KP differentially changed the diversity and structure of the developing microbiome. Here we aimed to examine this relationship in an additional 20 infants with BA as part of a multi‐center investigation.

**Methods:** Stool samples were longitudinally collected from infants (n=20) at 5 days, 1, 3, and 6 months after KP and 1 year of life at 7 sites across the US. Whole genome shotgun metagenomic sequencing was performed. Clinical metadata was obtained from an ancillary study to the Childhood Liver Disease Research Network's Prospective Database of Infants With Cholestasis (PROBE; NCT00061828), The microbiome was compared between infants who attained COJ (TB <2 mg/dL by 3 months after KP) vs nCOJ (TB >/=2 mg/dL at 3 months after KP) and between good (TB<2 mg/dL), intermediate (TB =2‐6 mg/dL) and poor bile flow (TB >/=6 mg/dL) at 3 months after KP.

**Results:** 9 (45%) infants attained COJ, 9 (45%) did not, and 2 (10%) had missing data. 9 (45%) had good bile flow (TB<2 mg/dL), 4 (20%) had intermediate flow and 5 (25%) had poor bile flow. Average age at KP was 63.3 +/‐ 23.0 days. Overall, there were trends towards increased diversity (observed OTUs) over time in infants with COJ vs not. Overall community structural differences were seen between COJ vs nCOJ (p=0.001) with trends toward differences at the 5‐day point between infants who went on to obtain COJ vs not (p=0.068) These community differences were more pronounced between infants with good and poor bile flow (5‐day p=0.022, 3mo p=0.065, 6 mo p=0.085). At the species levels, *Bifidobacterium breve* relative abundance (RA) was higher in COJ (FDR‐adj p=0.02) and *Enterococcus faecalis* was higher in non‐COJ (FDR adj p<0.001) when all time points were combined. RA of *Escherichia coli* was also significantly higher in poor bile flow (FDR adj p=0.028). At each time point, trends to increased RA of *B. breve* in good bile flow and *E. coli* in poor bile flow were seen. Additionally, *E coli* RA positively correlated with serum TB levels at the 1‐ month (R^2^ r=0.63, p=0.003) and 3‐mo time points (R^2^ r=0.57, p=0.003). MaAsLin2 analysis demonstrated associations between *B. breve* and COJ both overall (p<0.001) and at the 5‐day time point (p= 0.01). LeFSE and MaAsLin2 analysis of Kegg pathways additionally demonstrated associations between good bile flow and secondary bile acid synthesis (p=0.02). This Kegg pathway was found in several taxa of *Bifidobacterium*, such as *B. breve*.

**Conclusions:** In these preliminary analyses, there are differences in the longitudinal microbiomes of infants with BA who have COJ vs not. *B. breve* associates with good bile flow and *E. coli* associates with poor bile flow after KP. Secondary bile acid synthesis associated with good bile flow, suggesting that bile flow may select for bacteria such as *B. breve* and overall impact the development of the infant microbiome. Ongoing prospective studies with additional participants will help confirm these findings.


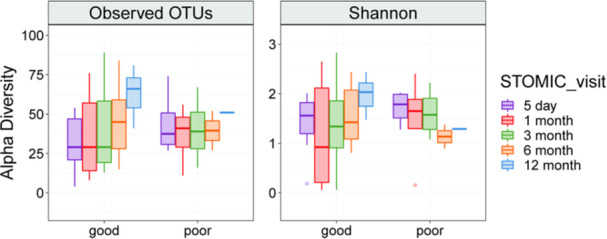


Figure 1. Alpha diversity metrics over time in infants with biliary atresia who attain good (TB 6 mg/dL) bile flow at 3 months after KP.

## 608 PREBIOTIC INULIN‐TYPE FRUCTANS MODULATES CLOSTRIDIOIDES DIFFICILE ABUNDANCE IN CHILDREN WITH INFLAMMATORY BOWEL DISEASE: SUB‐ANALYSIS FROM A DOUBLE‐BLIND RANDOMIZED CONTROLLED TRIAL

*Jibraan Fawad* ^*1*^, *Naomi Wilson* ^*1*^, *Ceylan Tanes* ^*1*^, *Connor Tiffany* ^*2*^, *Katharine Hewlett* ^*2*^, *Robert Baldassano* ^*1*^, *Jessica Breton* ^*1,4*^, *Lindsey Albenberg* ^*1*^, *Joseph Zackular* ^*2,3*^

^*1*^ *Gastroenterology, Hepatology and Nutrition*, *The Children's Hospital of Philadelphia*, *Philadelphia*, *PA*; ^*2*^ *Pathology and Laboratory Medicine*, *The Children's Hospital of Philadelphia*, *Philadelphia*, *PA*; ^*3*^ *The Center for Microbial Medicine*, *The Children's Hospital of Philadelphia*, *Philadelphia*, *PA*; ^*4*^ *Division of Pediatric Gastroenterology*, *Universite de Montreal*, *Montreal*, *QC*, *Canada*

**Background:** *Clostridioides difficile* infection (CDI) is a significant public health concern with approximately 500,000 infections annually in the United States with an estimated 30,000 deaths each year. There has been a worrisome rise in community‐associated CDI in patients who are at risk of severe disease. Children with inflammatory bowel disease (IBD) are disproportionally susceptible to *CDI* comparable with rates of infection in adults with IBD. Antibiotics are mainstay for treatment though they can increase the risk of developing disease as well. Therefore, there is a growing need to identify alternative preventative and therapeutic strategies for CDIs including non‐pharmacologic interventions.

**Methods:** A single‐center double‐blind, randomized placebo‐controlled trial was performed at the Children's Hospital of Philadelphia. Participants included patients with inflammatory bowel disease in clinical remission (PCDAI or PUCAI <10) and with fecal calprotectin between 50 and 500 mcg/g. Participants were randomly assigned to either an oligofructose‐enriched inulin (OFI) or a maltrodextin placebo for 8 weeks. Shotgun metagenomic sequencing of stool samples and rectal swabs was performed at baseline, week 4, 8 and 16. Sub‐analysis from microbiome analysis (shotgun metagenomic sequencing) was completed to compare relative abundance of *C. difficile* at these time points.

**Results:** The study randomized 68 patients, and 59 were included in the efficacy analyses. Relative abundance of *C. difficile* in stool samples was decreased in patients receiving OFI at 8 weeks (‐0.22, p=0.021). 8 weeks after discontinuation of OFI, this difference in relative abundance was lost (Figure 1).

**Conclusion:** OFI supplementation in children with inflammatory bowel disease can decrease the relative abundance of *C. difficile* in stool. Dietary interventions may provide a safe preventive intervention to prevent CDIs.


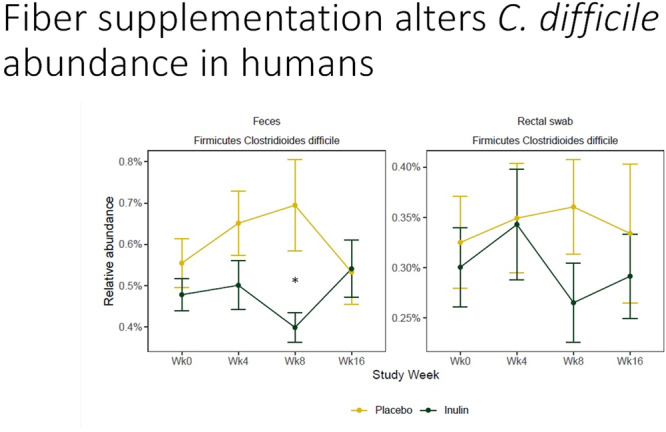


## 609 INTESTINAL TISSUE METABOLOME REVEALS DEPLETION OF HYDROXYBUTYRATE AND ELEVATION OF INDOXYL SULFATE IN PATIENTS WITH NECROTIZING ENTEROCOLITIS

*Joann Romano‐Keeler* ^*1*^, *Muthusamy Thiruppathi* ^*1*^, *Hua Geng* ^*1*^, *Xiao‐Di Tan* ^*1*^, *De‐Ann Pillers* ^*1*^, *Joern‐Hendrik Weitkamp* ^*2*^

^*1*^ *Pediatrics*, *University of Illinois Chicago*, *Chicago*, *IL*; ^*2*^ *Pediatrics*, *Vanderbilt University*, *Nashville*, *TN*

Necrotizing enterocolitis (NEC) remains a devastating gastrointestinal emergency affecting 15% of infants <1500 grams. While metabolomic studies have profiled metabolite derangements associated with this disease, no studies, to our knowledge, have conducted this research in intestinal tissue, the primary injury site. We conducted untargeted metabolomics on intestinal tissue from NEC patients and surgical controls to identify metabolic pathways implicated in NEC onset and progression.

Twenty‐three patients (8 surgical controls, 13 NEC cases) were included in this study. Tissue samples were collected at the time of surgery from both cases or controls and stored in ‐80 degrees Celcius. Samples underwent metabolomics analysis using ultrahigh performance liquid chromatography‐tandem mass spectroscopy. Raw data was extracted, peak‐identified, and QC processed. Compounds were identified by comparison to library entries of purified standards or recurrent known entries. Following normalization for mass extracted and log transformation, Welch's two sample t‐test and matched pairs t‐test were used to identify biochemicals that achieved statistical significance.

There were no statistically significant differences in gender, gestational weeks, days of life, early and late antibiotic use, and type of feeds between control and NEC patients. Of 1,039 detected compounds, 243 were upregulated and 29 downregulated in NEC. Principal coordinate analysis demonstrates a distinct clustering of NEC samples versus control patients with PC1 and PC2 accounting for 23% and 13% of variance, respectively. Hierarchical clustering further supported differentiation between groups. Indole‐containing compounds were elevated in NEC patients, including a greater than 100‐fold increase in 3‐indoxyl sulfate, a metabolite of tryptophan known to increase intestinal permeability and cause epithelial barrier dysfunction. NEC patients had a marked increase in caffeine and xanthine‐related metabolites, including theophylline, theobromine, and 1,3‐dimethylurate. A trend towards decreased 3‐hydroxybutyrate in NEC patients was observed, however, not statistically significant.

This is one of the only studies evaluating metabolites in intestinal tissue of infants with and without NEC. Distinct metabolite profiles were detected, with patients clustering based on disease state. Notably, indole‐containing compounds, known to incite bowel injury, were elevated in NEC patients. Elevated caffeine metabolites suggest vasoconstriction may play a role in NEC pathogenesis. The observed trend toward decreased butyrate supports its potential gut‐protective role. These findings underscore the importance of tissue‐based studies in understanding NEC pathophysiology.


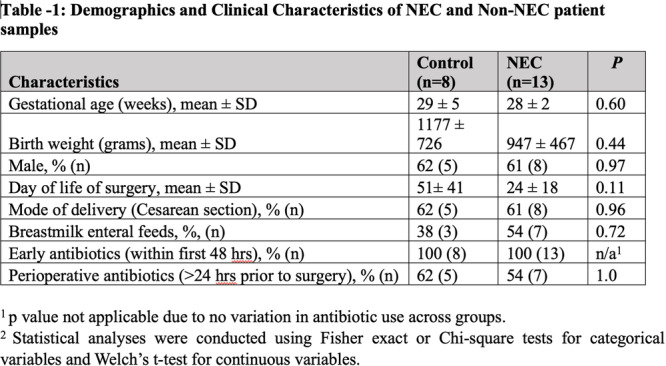



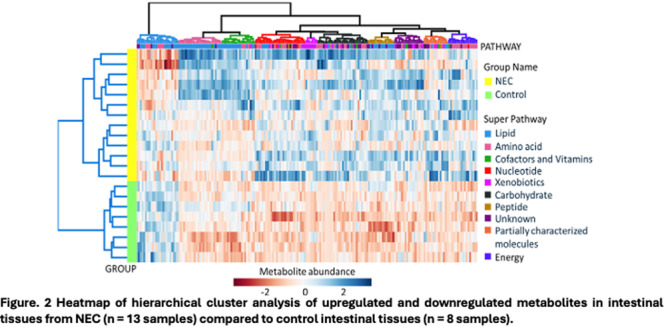


Metabolite expression across super pathways is represented with distinct color labels along the horizontal axis. The sample groups, NEC and control, are shown vertically with blue and pink labels, respectively. A color bar at the bottom indicates metabolite abundance. Welch's two‐sample t‐test and Ward's linkage method were used for statistical analysis and hierarchical clustering, respectively.

## 610 GASTRIC MOTOR DYSFUNCTION BY ^13^ C‐SPIRULINA GASTRIC EMPTYING BREATH TEST AND DYNAMIC GASTRIC MRI IN ADOLESCENTS WTIH DYSAUTONOMIA VS HEALTHY CONTROLS

*Fatima Khamissi* ^*1*^, *Lauren Williams* ^*2*^, *Matthew Plunk* ^*2*^, *Dylan Applin* ^*2*^, *Liyun Zhang* ^*3*^, *Pippa Simpson* ^*3*^, *Katja Karrento* ^*1*^

^*1*^ *Department of Pediatrics*, *Medical College of Wisconsin Department of Pediatrics*, *Milwaukee*, *WI*; ^*2*^ *Radiology*, *Medical College of Wisconsin Department of Pediatrics*, *Milwaukee*, *WI*; ^*3*^ *Biostatistics*, *Medical College of Wisconsin Department of Pediatrics*, *Milwaukee*, *WI*

**Background:** The autonomic nervous system (ANS) closely regulates GI tract motility. Many patients with dysautonomia suffer from postprandial GI symptoms frequently dismissed as functional or psychosomatic. There is a lack of diagnostic modalities that accurately assess gastric motor function, particularly in children and relative to healthy, age and gender‐matched norms. The aim of this study was to evaluate 1) gastric emptying using the ^13^ C‐*Spirulina* gastric emptying breath test (^13^C‐GEBT) and 2) gastric motor function using a novel, dynamic MRI protocol in adolescents with ANS imbalance compared to cohorts of age and gender‐matched healthy controls (HC).

**Methods:** Adolescent females ages 11‐18 years with chronic, post‐prandial symptoms and suspected gastric dysmotility were prospectively enrolled from a tertiary care clinic. After overnight fast and withholding any promotility drugs x 72hrs, subjects underwent the ^13^C‐GEBT with collection of 10 breath samples across 4 hours after a standardized test meal. On a separate day, a subset underwent a 60 min gastric MRI protocol with anatomical and motility scans before and after ingestion of 300 ml formula inside scanner. Gastric motor function was quantified by measuring change in gastric volume (GV), gastric wall thickness, superior mesenteric artery (SMA) blood flow and contractility [spatio‐temporal motility mapping (STMM) score] in response to meal. Two cohorts of gender and age‐matched HC underwent the same protocols (n=216 ^13^C‐GEBT and n=14 MRI).

**Results:** 58 females with ANS dysfunction, median (IQR) age 16.3 (14.7, 17.5) years, were enrolled. Controls were similar in demographics, BMI and pubertal stage. 71% had hypermobile Ehlers‐Danlos Syndrome/Hypermobile Spectrum Disorder and 63% psychological comorbidities. Patients suffered from median (IQR) 7 (6, 8) extra‐intestinal comorbidities. 20% required enteral nutrition support. 58 patients underwent ^13^C‐GEBT per protocol of which n=20 also completed gastric MRI. 65% had delayed gastric emptying based on recently established ^13^ C‐GEBT reference values for females >11 years (unpublished data based on n=216 HC). Those with delayed gastric emptying did not have a higher frequency of specific comorbidities but were more likely to require enteral nutrition (p=0.025). A greater total GV on MRI correlated positively with higher BSA (r=0.567). Patients had a smaller total GV change in response to meal compared to HC (p=0.032), which did not correlate with delayed gastric emptying by ^13^C‐GEBT (p=0.692). Patients on oral contraceptives vs. those not, had smaller relative change in SMA blood flow (p=0.027) and gastric fundus wall thickness (p=0.024) in response to meal. There was no significant difference in pre‐post meal motility score (STMM) between subjects and HC. Additional analyzes of fundus volume change in response to meal are pending.

**Conclusions:** This is the first study to report on specific gastric motor function alterations in adolescents with ANS dysfunction compared to age, gender and BMI matched healthy controls. A large proportion have delayed gastric emptying while a separate cohort displays reduced meal‐induced gastric volume change (i.e. gastric accommodation). Subjects taking oral contraceptives have altered meal response based on several MRI‐based gastric function parameters. Findings may have therapeutic implications for dysautonomia patients with post‐prandial distress.


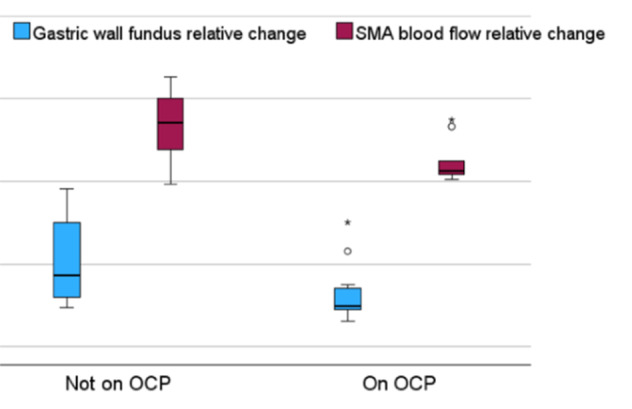


Fig. 1. Diffferences in gastric fundus wall thickness and superior mesenteric artery (SMA) blood flow change in response to meal in patients not on oral contraceptives (OCPs) vs. on OCPs (0.024 and 0.027 respectively).

## 611 CONTINENT OSTOMY DEVICE

*Sara Fidanza* ^*1,2*^, *Devon Horton* ^*3*^, *Lily Williams* ^*4*^, *steven moulton* ^*5*^

^*1*^ *Digestive Health*, *Children's Hospital Colorado*, *Aurora*, *CO*; ^*2*^ *University of Colorado System*, *Denver*, *CO*; ^*3*^ *Division of Pediatric Surgery, Department of Surgery*, *University of Colorado Anschutz Medical Campus School of Medicine*, *Aurora*, *CO*; ^*4*^ *Division of Pediatric Surgery, Department of Surgery*, *University of Colorado Anschutz Medical Campus School of Medicine*, *Aurora*, *CO*; ^*5*^ *Division of Pediatric Surgery, Department of Surgery*, *University of Colorado Anschutz Medical Campus School of Medicine*, *Aurora*, *CO*

An ostomy is an external facing, surgically diverted segment of bowel that results in the formation of a temporary or permanent stoma. This procedure is commonly performed for conditions such as inflammatory bowel disease (IBD), bowel dysmotility, abdominal trauma, bowel obstruction, pelvic and perineal tumors, and rarely intestinal cancer. Ostomy care requires a pouching system that includes an adhesive wafer and a heavy‐duty plastic bag that collects feces that uncontrollably spill from the stoma.

Current pouching systems are obtrusive, malodorous, noisy, and occasionally leak gas and fecal matter causing skin irritation and embarrassment. As a result, ostomy patients face significant emotional and social challenges, including diminished self‐esteem, embarrassment, body image maladjustment, and grief related to loss of bowel control. Hence, there is a huge unmet need for a continent ostomy device that reduces leakage and controls fecal expulsion from a stoma. Such a device would enhance the quality of life and overall well‐being of patients, who require a temporary or permanent stoma.

Currently, the ostomy market is dominated by traditional pouch systems, which have remained unchanged for 70+ years. This $3+ billion global ostomy market is dominated by five traditional pouch systems companies. Approximately one million Americans live with an ostomy and in 2023 the US ostomy market was valued at $543.68 million dollars with a CAGR of 4.0%. To date, there are two implantable devices in the market developed to address ostomy continence: 1) the Transcutaneous Implant Evacuation System (TIES), a surgically implemented device patented and evaluated in three clinical studies; and 2) StomaLife, a partially implanted pressure relief system with a magnetic holder that is surgically implanted to secure the device to the skin without adhesives and barriers. StomaLife, is patented in Australia but is not available to the consumer market. Two additional exterior‐facing devices in literature are SphinX and CapsuleCap with no commercialization information available.

To address this unmet need, a team made up of bioengineers, a gastroenterology advance practice nurse and a surgeon, met to develop a continent ostomy device (Twistomy). This device is minimally invasive, composed of a soft flexible inner ring, twistable thin plastic sleeve and a low‐profile external housing. The inner ring and sleeve, inspired by the Alexis retractor, are inserted through the stoma and into the lumen of the bowel. The outer portion of the plastic sleeve is secured to the external housing, enabling users to twist the conduit closed, thus creating a valve to control the expulsion of fecal matter. The low‐profile external housing attaches to the body via a standard stoma wafer. To evacuate a stoma, the user attaches an ostomy pouch to the housing unit and untwists the conduit, opening the channel for fecal expulsion in a controlled and effective manner. This design ensures an air‐ and effluent‐tight seal to minimize leakage and malodor and eliminate the need to always wear an ostomy pouch.

This continent ostomy device has undergone significant pre‐clinical development and testing. Initially, the problem was defined, design requirements determined, a continent ostomy device conceptualized, then engineered, progressing through several stages of device design, verification, and validation utilizing an ostomy model. Twistomy withstands 70 mmHg of effluent pressure in its sealed state, which corresponds to the intraluminal pressure typically found in the bowel. When the device is untwisted to the open position, it allows the bowel to effectively evacuate. Twistomy's components are easy to handle and color coded, allowing assembly and disassembly in under three minutes.

To date, all prototype development and testing has been performed in a laboratory setting. The next step is to hire an FDA consultant and meet with the FDA to gain a better understanding of the regulatory pathway and the studies that will be required in animals and/or humans to gain clinical clearance. The regulatory pathway (510 K vs de novo) will influence the amount of funding required for R&D efforts and clinical trial support, which in turn will influence manufacturing, corporate development, sales, and marketing costs. The timing required to bring Twistomy to commercial sales will depend on how quickly funds can be raised to support and conduct clinical trials. The amount could easily exceed $3‐4 M; however, the potential benefits to patient care and investor returns are significant.

It is unknown whether Twistomy will follow a 510(k) or de novo regulatory pathway. Neither do we know if animal testing will be required, or if pre‐clinical testing with a high‐fidelity model will allow us to bypass animal studies and move directly to human clinical studies, to gather safety and effectiveness data required for FDA clearance with the goal to improve the lives of ostomy patients.

## 612 HELICOBACTER PYLORI IN CHILDREN: RISK OR PROTECTION?

*Jessica Merino* ^*1*^, *Idalmis Matos* ^*1*^, *Daime Guilarte Falcon* ^*1,2*^, *Elsa García Bacallao* ^*1*^, *Maria Pérez Rodríguez* ^*1*^, *Enrique Galbán García* ^*1*^, *Licet González Fabián* ^*1*^

^*1*^ *Plaza de la Revolución*, *Instituto de Gastroenterologia*, *Havana*, *Havana*, *Cuba*; ^*2*^ *Gastroenterology and Endoscopy*, *St. Joseph Mercy Hospital*, *Georgetown*, *Demerara‐Mahaica*, *Guyana*

**INTRODUCTION:** Helicobacter pylori infection requires research to identify the true risks or potential beneficial effects currently being debated in childhood.

**OBJECTIVE:** To identify the prevalence and possible associations of Helicobacter pylori infection with gastrointestinal and extragastrointestinal diseases in pediatric patients.

**METHOD:** A descriptive, cross‐sectional study with an analytical component was conducted in children under 19 years of age who underwent upper gastrointestinal endoscopy at the Institute of Gastroenterology from January 2019 to June 2023. The sample consisted of 195 patients. Age, sex, clinical manifestations, endoscopic and histological diagnoses, and the presence of comorbidities were the main variables studied. Numbers and percentages were used as well as measures of central tendency and dispersion. Statistical analysis was performed using Mantel‐Haenszel chi‐square test, Fisher exact test, Student's t‐test, and univariate odds ratio analysis with 95% confidence intervals.

**RESULTS:** The overall prevalence was 44%, higher in those over 10 years of age (p=0.03); The main symptoms were epigastralgia (87.6%), nausea (58.4%) (p>0.05); asthma and other allergies were the most frequent diseases in those infected (p 0.05). Nodular gastritis and pangastritis were statistically associated (p<0.001); as was the intensity of the moderate and severe chronic infiltrate (p<0.001) and histological activity in the antrum (43%) and in the antrum and body (26.7%) (p<0.01); No premalignant lesions were diagnosed.

**CONCLUSIONS:** Clinical manifestations are nonspecific, and complications in children are less frequent than in adults. This study does not demonstrate an association between infection and the diseases studied.

## 613 ONLINE DIGITAL E&M SERVICES FOR HOME PARENTERAL NUTRITION MANAGEMENT: INTEGRATION INTO CLINICAL PRACTICE

*Katherine Brennan*

*Gastroenterology, Hepatology, and Nutrition*, *Ann and Robert H Lurie Children's Hospital of Chicago*, *Chicago*, *IL*

Care of medically complex pediatric patients often extends beyond traditional face‐to‐face clinic visits to address their individual needs. This indirect provider‐level care, is primarily non‐billable and highly time‐consuming. In the Intestinal Rehabilitation Program, we specialize in managing patients with complex, chronic GI conditions. Many of these patients experience intestinal failure and rely on home parenteral nutrition (HPN). HPN necessitates frequent lab work and patient monitoring by a highly skilled team. Historically, a significant portion of HPN management has been non‐billable, APP patient care. The introduction of Online Digital E&M Services allows APPs to capture charges for provider‐level HPN management. These billable encounters can be applied across a range of pediatric subspecialty practices. I will present an analysis of the HPN management charges and payments generated from this initiative, offering insights and a practical billing implementation experience for other institutions to consider.


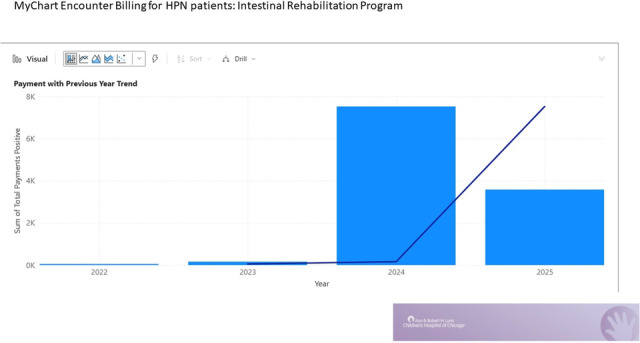


Payments


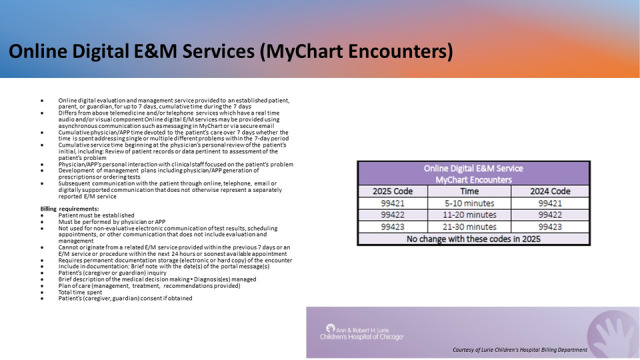


Billing Information

## 614 PEDIATRIC GI TRIAGE ALORITHM SYSTEM: GETTING THE RIGHT PATIENT SEEN BY THE RIGHT PROVIDER

*Debra Browne*, *Jaya Punati*

*Gastroenterology, Hepatology, Nutrition*, *Children's Hospital Los Angeles*, *Los Angeles*, *CA*

The GI department at CHLA has been working to maximize provider productivity related to new patient referrrals based on a detailed written algorithm. Prior to the implementaton of the current GI Triage process any new pt could be scheduled in any open provider appointment slot, without regard to the clinical expertise of the provider. This led to NPs having complex patients on their shcedule and Physicians seeing non‐complex patients. This resulted in fncreasing frustration among all of the providers and patients.

The department developed a written triage algorithm so that the RN triage nurses would be able to consistently assign the new patinet referrals to the most appropriate provider and also determine if the new patient referral needed to be seen urgently, based on this algorithm.

If the RN triage team has a question regarding urgency or how to scheudle the patient, the question is sent to the GI Department Nurse Practitioner Triage coordinator. This role is filled by 1 NP and provides consistency with regard to patient direction/decision making.

The algorithm outcomes are designed :

1. To assign specialty patients (ie IBD, Motility, Intestinal Rehab) to a specialty Nurse Care Manger who can then review the referral with the specialty team to determine how to schedule the patient.

2. Allows an Urgent referral to be scheduled in an Urgent appointment slot, as the patient meets predetermined "urgent" triage criteria.

3. Matches the skill set of the provider with the patient's referral question.

4. Decreases the number of times patients need to be rescheduled

5. Builds expertise in the NP provider group because they now care for a consistent group of specific General Pediatric GI problems.

6. Gets the right kid on the right scheudle.

7. Improves provider satisfation.

8. improves patient satisfaction.

The poster will outline the specific triage guidelines and the decion making process. The framework of this system can be easily used by other GI Departments or specilaty teams.

## 615 ESOPHAGEAL DISSECANS: A RARE FINDING ON AN ENDOSCOPY

*Patricia Bierly*

*GI and Nutrition*, *The Children's Hospital of Philadelphia*, *Philadelphia*, *PA*

**Introduction:** Esophagitis is inflammation in the esophagus. Symptoms of esophagitis include painful, difficulty swallowing and chest pain with eating. Causes of esophagitis include stomach acid irritating the esophagus, oral medications, allergies such as eosinophilic esophagitis, and infections. This case presents a patient with esophagitis dissecans superficialis which is rare occurrence.

**Background/Literature Review:** Esophagitis dissecans superficialis (EDS) is a rare, benign endoscopic finding, characterized by mucosal sloughing into the esophageal lumen. At Children's Hospital of Philadelphia the were no other reports of EDS. Most cases are found incidentally and in adults.

Findings/Case Description GH is a 13‐year‐old female with a complex medical history including past medical history of failure‐to‐thrive, cardiac abnormality, growth hormone deficiency, ADHD, anxiety, spontaneous perforated duodenal ulcer, past C. difficile infection, and hematemesis. GH presented to the emergency room with complaints of epigastric pain and vomiting. GH underwent an Upper Endoscopy and Colonoscopy which showed the following.

‐ Early necrosis of the superficial portions of the mucosa with formation

of small bullae and minimal inflammation, most suggestive of esophagitis dissecans superficialis.

**Summary/Recommendations** Esophagitis dissecans superficialis can be caused by medications, autoimmune disease, or medications (such as NSAIDs) most are idiopathic. EDS is a rare endoscopic finding necrosis of the superficial portions of the mucosa with formation of small bullae and minimal inflammation, most suggestive of esophagitis

dissecans superficialis. Our patient was treated with dietary changes, Omeprazole and Carafate with a repeat Endoscopy several months later showed resolution.

## 616 MANAGEMENT OF MAINTENANCE INTRAVENOUS IRON IN THE HOME PARENTERAL NUTRITION DEPENDENT PATIENT

*Jennifer McClelland* ^*1,3*^, *Margaret Murphy* ^*2,3*^, *Matthew Mixdorf* ^*1*^, *Alexandra Carey* ^*1,3*^

^*1*^ *Gastroenterology, Hepatology & Nutrition*, *Boston Children's Hospital, Boston Children's Hospital, Boston, MA, US, hospital/children*, *Boston*, *MA*; ^*2*^ *Pharmacy*, *Boston Children's Hospital*, *Boston*, *MA*; ^*3*^ *Home Parenteral Nutrition Program*, *Boston Children's Hospital*, *Boston*, *MA*

**Background:** Iron deficiency anemia (IDA) is common in patients with intestinal failure (IF) dependent on parenteral nutrition (PN). If severe, can require hospital admission, blood transfusion, and/or infusion center visits which decreases quality of life. Treatment with enteral iron is preferred; however, may not be tolerated or efficacious. In these cases, intravenous (IV) iron is a suitable alternative. Complications include adverse reactions and infection, though low when using low‐molecular‐weight (LMW) formulations. In a home PN (HPN) program, an algorithm (Figure 1) was developed by nurse practitioner to treat IDA utilizing IV iron.

**Methods:** Retrospective chart review was conducted in large HPN program (150 patients annually) from Jan 2019 ‐ Apr 2024 prescribed IV iron following an algorithm. Laboratory studies were analyzed looking for instances of ferritin >500 ng/mL indicating potential iron overload, as well as transferrin saturation 12‐20% indicating iron sufficiency. In instances of ferritin levels >500 further review was conducted to understand etiology, clinical significance and if IV iron algorithm was adhered to.

**Algorithm:** HPN patients are diagnosed with IDA based on low iron panel (low hemoglobin and/or MCV, low ferritin, high reticulocyte count, serum iron and transferrin saturation and/or high total iron binding capacity (TIBC). If the patient can tolerate enteral iron supplementation, dose of 3‐6 mg/kg/day is initiated. If patient cannot tolerate enteral iron, the IV route is initiated. Initial IV dose is administered in the hospital or infusion center for close monitoring and to establish home maintenance administration post repletion dosing. Iron dextran is preferred as it can be directly added into the PN and run for duration of the cycle. Addition to the PN eliminates an extra infusion and decrease additional CVC access. Iron dextran is incompatible with IV lipids, so the patient must have one lipid‐free day weekly to be able to administer. If patient receives daily IV lipids, iron sucrose is given as separate infusion from the PN. Maintenance IV iron dosing is 1 mg/kg/week, with dose and frequency titrated based on clinical status, lab studies and trends. Iron panel and C‐reactive protein (CRP) are ordered every 2 months. If lab studies are below the desired range and consistent with IDA, IV iron dose is increased by 50% by dose or frequency; if studies are over the desired range, IV iron dose is decreased by 50% by dose or frequency. Maximum home dose is < 3 mg/kg/dose; if higher dose needed, patient is referred to an infusion center. IV iron is suspended if ferritin >500 ng/mL due to risk for iron overload and deposition in the liver.

**Results:** Ferritin results (n = 4165) for all patients in HPN program from Jan 2019‐Apr 2024 reviewed looking for levels >500 ng/mL indicating iron overload. Twenty‐nine instances of ferritin >500 ng/mL (0.7% of values reviewed) were identified in 14 unique patients on maintenance IV iron. In 9 instances, high ferritin level occurred with concomitant acute illness and elevated CRP; elevated ferritin in these cases thought to be related to an inflammatory state vs. iron overload. In 2 instances, IV iron dose was given the day before lab draw, rendering a falsely elevated result. Two patients had 12 instances (0.28% of values reviewed) of elevated ferritin thought to be related to IV iron dosing in the absence of inflammation, with normal CRP levels. During this period, there were no recorded adverse events.

**Conclusion:** IDA is common in patients with IF dependent on PN. Iron is not a standard component or additive in PN. Use of maintenance IV iron in this population can increase quality of life by decreasing need for admissions, visits to infusion centers, or need for blood transfusions in cases of severe anemia. IV iron can be safely used for maintenance therapy in HPN patients with appropriate dosing and monitoring.


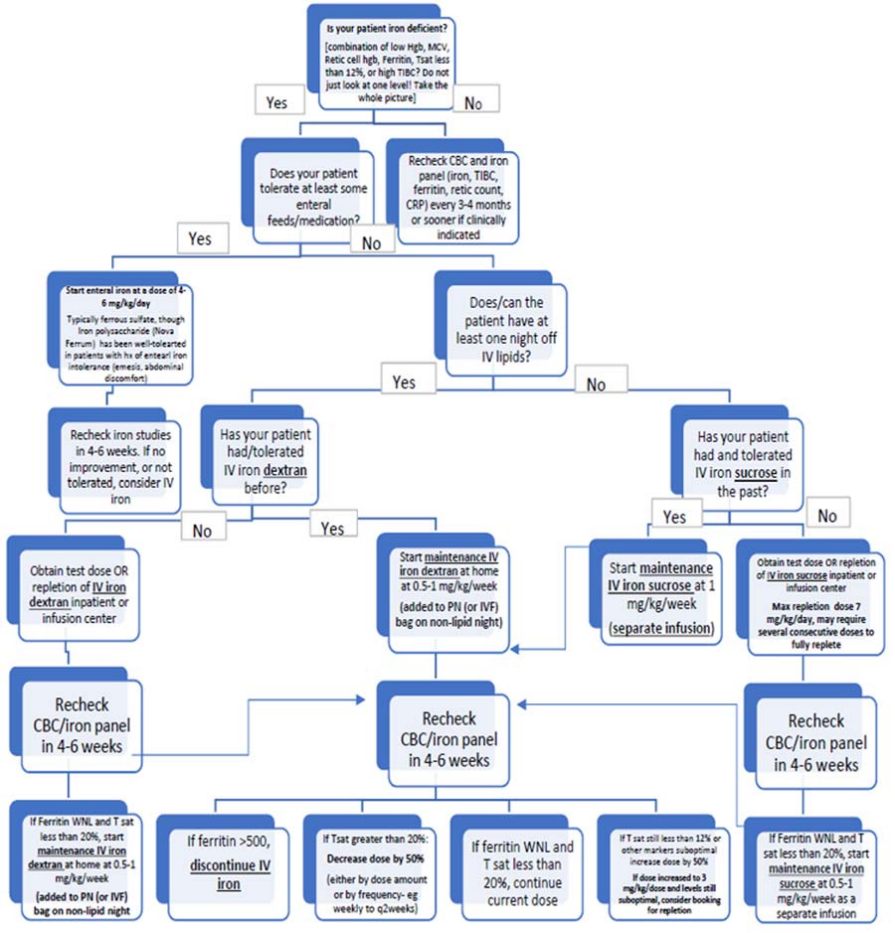


Maintenance IV Iron Algorithm

## 617 IMPLEMENTING A NOVEL PATIENT‐PROVIDER CODE OF CONDUCT FOR YOUNG ADULTS WITH INFLAMMATORY BOWEL DISEASE (IBD) TO BETTER FACILITATE TRANSITION TO THE ADULT IBD CENTERS: A PILOT STUDY

*Billi Marie Meli*, *Vikram Raghu*, *Whitney Gray*, *Whitney Sunseri*

*Gastroenterology*, *UPMC Children's Hospital of Pittsburgh*, *Pittsburgh*, *PA*

**Background:** Those 18‐22 years of age with IBD are known to be a vulnerable population. Without proper transition and transfer of care they are likely to get lost to follow up, discontinue or fail to adhere to prescribed medications and over utilize the emergency room. This same cohort has higher frequency of missed appointments without notification in the Pediatric IBD office where access is a precious resource. We aimed to identify the frequency of missed appointments in young adults with IBD and to pilot a novel code of conduct agreement to reduce missed visit frequency.

Method: The Improve Care Now (ICN) database was queried to identify all patients 18 years and older that are currently being cared for at the UPMC Children's Hospital of Pittsburgh (CHP) IBD Center. Patient charts were reviewed and the number of missed appointments or cancelations < 48 hours in advance were collected. A stakeholder team that included the CHP IBD medical team and the UPMC legal team developed the Code of Conduct intervention. Implementation data was collected.

**Results:** 295 patients aged 18 years and older were identified, with 116 falling within the 22–27‐year age group, 65 of whom are currently under care. This cohort of active patients collectively missed or cancelled appointments with less than 48 hours notice a total of 194 times since turning 18 years old. Created from the stakeholder team meetings, the Code of Conduct (CHP IBD University Acceptance Letter, Figure 1) outlines expectations (Figure 2) for patients, including attendance at biannual appointments, communication through the myCHP portal, notification of changes, and compliance with prescribed care. A clause was included to specify that failure to adhere to expectations would result in dismissal and early transition and transfer to an adult IBD facility. In the last 60 days since its inception, 50 patients have been approached, and all have signed the agreement with no missed appointments after signing code of conduct.

**Conclusion:** Young adult patients with IBD are high risk for no show or cancellation of appointments. This novel Patient‐Provider Code of Conduct was created to establish guidelines for increasing accountability, identify cause for dismissal, and to streamline transition/transfer to the Adult IBD centers. It has been received well by patients and families. Future studies will determine if this initiative improves healthcare utilization, adherence to treatment plans, increased TRAQ scores, and ultimately a successful transition/transfer of the patient to an Adult IBD center.


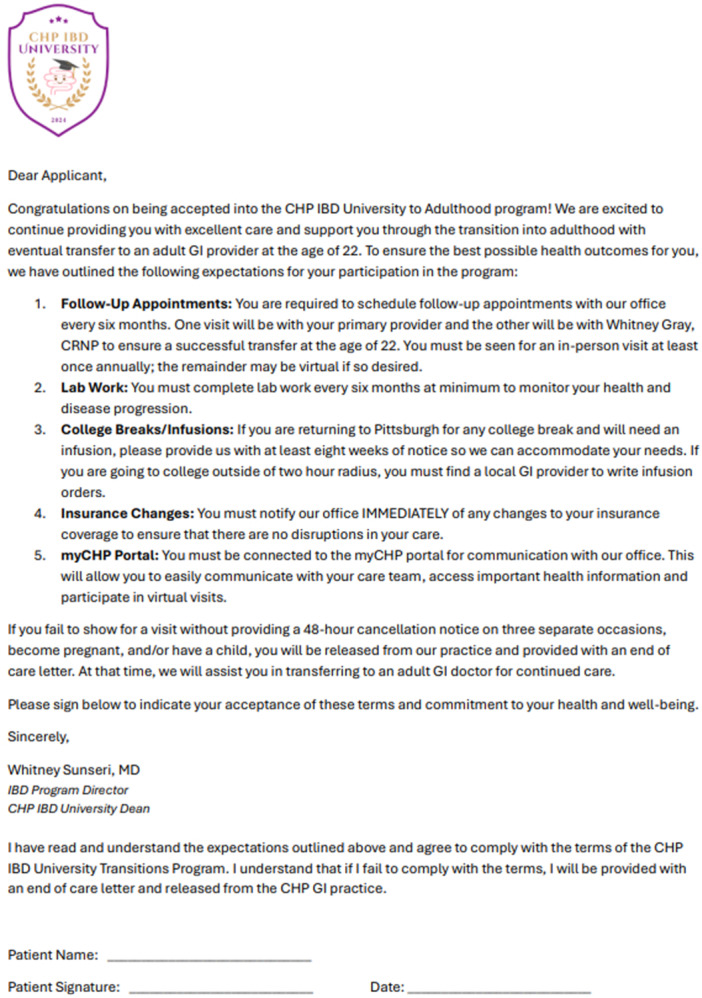


CHP IBD University Acceptance Letter Example


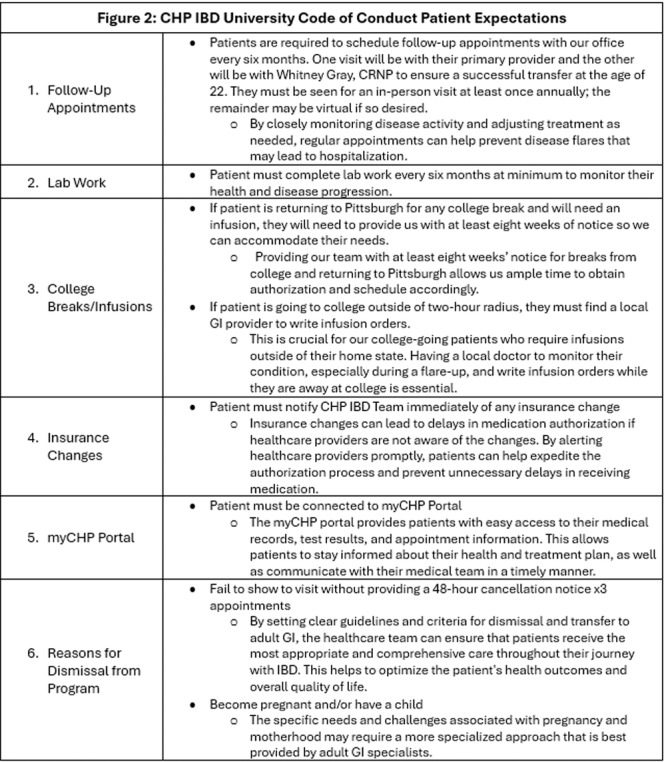


CHP IBD University Code of Conduct Patient Expectations

## 618 ENHANCING PATIENT EXPERIENCE AND OUTCOMES OF ESOPHAGEAL MANOMETRY WITH ENDOSCOPIC CATHETER PLACEMENT: A NURSING PERSPECTIVE

*Sarah Bandurski*, *Elizabeth Kearns*, *Vibha Sood*

*Gastroenterology*, *UPMC Children's Hospital of Pittsburgh*, *Pittsburgh*, *PA*

**Background:** High‐resolution esophageal manometry (HREM) is a widely used diagnostic tool for assessing esophageal motility in various clinical conditions, including achalasia, diffuse esophageal spasm, and systemic sclerosis. The standard method for placing the high‐resolution manometry (HRM) catheter involves transnasal insertion while the patient is awake. This approach can cause discomfort and anxiety, which may not be tolerable for certain patient populations. An alternative technique is to place the HRM catheter during a baseline upper endoscopy (EGD), which has proven effective in more complex cases.

**Method:** We conducted a retrospective review of patients aged 10 to 21 years who underwent EGD with HREM catheter placement within the past year. We compared this group to a similar cohort that underwent manometry catheter placement and testing while awake. The small cohort included four patients with systemic sclerosis who required both pre‐transplant and post‐transplant evaluations of esophageal motility. We standardized processes for pre‐procedure patient education, equipment setup, procedural protocols, and reporting of results. The duration of the procedures was determined through an extensive review of perioperative nursing documentation.

**Results:** The study cohort comprised eight patients, with both endoscopic (n=4) and transnasal (n=4) placements, resulting in a 100% success rate for catheter insertion. In the group undergoing endoscopic catheter placement, there were no immediate complications, and patients reported significantly lower levels of anxiety and discomfort. Additionally, nursing staff noted improvements in workflow efficiency, patient cooperation, and overall tolerability. The total duration of the procedures was comparable between the two groups. Furthermore, three patients in the endoscopic group benefited from the concurrent EGD, which would have otherwise required a separate procedure, leading to reductions in cumulative time, costs, and staffing requirements. A subset of patients underwent additional pH impedance probe testing. Retrospective analysis could not effectively distinguish the time allocated for endoscopic manometry placement from that designated for awake manometry catheter placement.

**Conclusion:** Preliminary findings from this study suggest that endoscopic manometry placement is a safe and viable alternative to the traditional awake approach. It yielded no immediate complications while maintaining the quality of the studies. The reported advantages include reduced patient discomfort and anxiety, the ability to directly visualize esophageal landmarks for accurate positioning, enhanced procedural workflow, and potential time and cost efficiencies, especially when EGD and manometry procedures were combined into a single session. Additionally, the combined approach allows for the collection of biopsies, facilitating the evaluation of mucosal pathology and visualization of structural abnormalities. To further assess patient‐perceived benefits and satisfaction, structured experience surveys will be administered in future assessments.

## 619 THE USE OF TELEHEALTH IN TRANSITION OF CARE PLANNING FOR ADOLESCENTS WITH INFLAMMATORY BOWEL DISEASE

*Anna Jones*

*Manning Family Children's, Pediatric Gastroenterology*, *LCMC Health*, *New Orleans*, *LA*

Pediatric patients with Inflammatory Bowel Disease (IBD) require detailed planning when transitioning to adult gastroenterology. At our facility, there was no standard protocol in place to assess and facilitate transitioning these patients to new providers. Our practice was to place a referral and rely on the adult provider to bridge the gap in care and education with minimal disruption to therapy. While there is literature available regarding the needs of adolescents transitioning to adult care with IBD, there is little regarding the use of telehealth in this process.

A virtual transition of care clinic was piloted by an Inflammatory Bowel Disease Nurse Practitioner (NP) to help ease the transition to adult care. This clinic sought to evaluate the adolescent's readiness to transition by assessing their current knowledge base, stopping for education as appropriate, and creating a concrete plan post‐pediatric care through a virtual telehealth visit with a survey administered by a gastroenterology NP. Patients aged 17‐21 were selected by referral from the primary gastroenterologist and scheduled for a 1‐time 1‐hour long telehealth visit with the NP. The visit was structured as a standard IBD visit incorporating current symptoms, response to treatment, and health maintenance but focusing the majority of the visit with a verbal questionnaire of 41 questions administered by the NP. Self‐reported and corrected answers were typed and available to the patient to review along with a transition of care plan sent in the patient portal for future reference. Each visit was structured to the patient's individual case and required a considerable amount of preparation and chart review.

A total of 30 patients were scheduled from June 2024 to April 2025. 18 patients attended transition of care visits, 17 virtually and 1 in‐person. A total of 4 patients attended their initial visit to gastroenterology, 1 has an upcoming appointment, 3 are scheduled or have active plans for close‐future transitions. All but 1 patient was on a biologic medications at the time of the transition visit. Patients and parents expressed overwhelmingly positive feedback regarding this clinic and addressing topics such as re‐educating on medication safety, discussing complications of IBD, planning for adult insurance coverage, how to effectively set up a phone calendar to keep track of appointments, and even planning for future childbearing and fertility to name a few. Referring GIs verbally expressed positive feedback regarding patients being reeducated on their disease and medications and prepared for conversations regarding referrals and college accommodations. Patients and referring GIs were also empowered to pursue adult GI referral when a college or career decision was made. The virtual care model allowed the visit to be informal and non‐threatening to adolescents being questioned about their healthcare. Visits were conducted with and without parents, in the home setting, college dormitories, summer jobs, high school libraries, and even at an active construction site.

This virtual clinic allowed patients to familiarize themselves their case, tailored with individualized recommendations, re‐education regarding their disease and medications, planning for college or career with discussing available providers, insurance, medications, and health maintenance strategies in a familiar, accessible setting. A future direction for this clinic would be to re‐develop the survey to include a measurable numerical outcome for assessing knowledge base and referring provider satisfaction, begin administering the survey at an earlier age to assess knowledge and areas for the primary GI to focus on education, and expanding to engage the adult providers to assess the adolescent's readiness post‐telehealth visit. The use of telehealth shows a promising avenue to facilitate the transition of care for Pediatric Inflammatory Bowel Disease patients to adult gastroenterology.

## 620 ADDRESSING SOCIAL DETERMINANTS OF HEALTH (SDOH) IN PEDIATRIC CONSTIPATION: A QUALITY IMPROVEMENT PROJECT

*Lauren Wegrowski* ^*1,2*^, *Robert Sylvester* ^*2*^, *Amy Thorsen* ^*1*^, *Catherine McDonald* ^*3*^

^*1*^ *Pediatrics*, *University of Utah Health*, *Salt Lake City*, *UT*; ^*2*^ *College of Nursing*, *University of Utah Health*, *Salt Lake City*, *UT*; ^*3*^ *Primary Children's Hospital*, *Salt Lake City*, *UT*

**Background:** Pediatric constipation is a prevalent issue that disproportionately affects children from low socioeconomic backgrounds, where factors such as food insecurity and financial barriers can exacerbate symptoms. Routine management may not fully address social determinants of health (SDOH), which can impact treatment adherence and patient outcomes.

**Local Problem:** The project site provided general recommendations for managing pediatric constipation—such as increasing fiber intake, fluid consumption, and using laxatives—are commonly provided. However, these recommendations did not account for the unique social and economic barriers the patient population faced. Furthermore, there was no structured process of integrating SDOH into personalized care plans for patients with constipation. This quality improvement project aimed to integrate SDOH considerations into clinical workflows, supporting comprehensive and accessible care for underserved pediatric populations.

**Methods:** The project team modified existing constipation management protocols using the Johns Hopkins Evidence‐Based Practice Model to incorporate interventions informed by SDOH. The team collected baseline data on SDOH screening frequency and documentation through retrospective chart reviews, assessing existing documentation practices and integration of SDOH factors in patient encounters prior to implementation. Evaluation metrics included the frequency and timeliness of SDOH screenings documented in the electronic health record (EHR), frequency of SDOH‐informed documentation in clinic notes, and utilization of SDOH‐informed intervention Smart Phrases. A pre‐intervention survey assessed provider‐reported barriers to SDOH documentation and SmartPhrase use; however, no post‐survey responses were received.

**Interventions:** Key interventions included updates to the constipation action plan to improve clarity and usability and the creation of SmartPhrases in the EHR to facilitate SDOH‐informed documentation. To support dietary modifications, Women, Infants, and Children and Supplemental Nutrition Assistance Program eligible high‐fiber recipes were introduced, ensuring accessibility for families with limited resources. Additionally, a visual laxative guide was developed to address low health literacy and paired with a pharmacy benefit quick response code to provide access to real‐time coupons, reducing medication costs and promoting treatment adherence by minimizing financial barriers. Monthly provider interviews and chart audits guided iterative adjustments to these interventions, ensuring alignment with clinical workflows and meeting provider needs.

**Results:** After implementation, SDOH screening documentation improved significantly, increasing from 62.3% to 96.1%, with a chi‐square value of χ^2^ (1, N = 112) = 18.28, p < .001. Screening timeliness also improved, with the proportion of documented screenings completed within the past year rising from 52.6% to 100%, χ^2^ (1, N = 87) = 29.27, p < .001. These findings reflect improved integration of SDOH data into the electronic health record during patient rooming. However, consistent use of documentation tools remained limited. SmartPhrases were used in only 9 of 51 post‐intervention encounters and did not show a statistically significant increase from baseline, p = 0.2902. Similarly, ICD‐10 coding remained low, with only 11.8% of encounters including SDOH‐related codes, p = 0.138. Provider documentation of social risk factors was inconsistent, with no documentation observed for high‐risk food insecurity cases.

**Conclusion:** Addressing social factors in pediatric constipation management can reduce health disparities by supporting treatment adherence in vulnerable populations. This project highlights the potential of SDOH‐informed interventions used to enhance care quality and provider confidence. Future efforts will focus on refining these strategies and expanding their application across diverse clinical settings, providing a model for addressing SDOH in other chronic pediatric conditions.

*Keywords*: constipation, social determinants of health, SDOH, clinical action plan, quality improvement


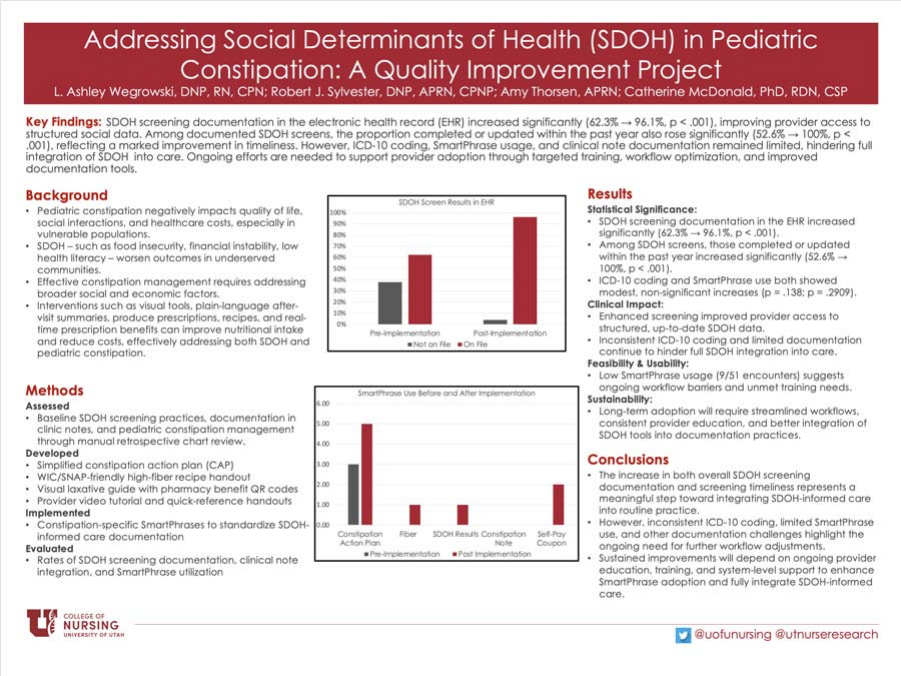


## 621 CVS ACTION PLAN FOR PATIENT‐CENTERED QUALITY CARE

*Julie Banda* ^*1*^, *Antice Dare* ^*2*^, *Kendra Otten* ^*3*^, *Bernadette Vitola* ^*1*^

^*1*^ *Pediatric Gastroenterology*, *Medical College of Wisconsin Department of Pediatrics*, *Milwaukee*, *WI*; ^*2*^ *Milwaukee Hospital‐Children's Wisconsin*, *Milwaukee*, *WI*; ^*3*^ *College of Nursing*, *Marquette University*, *Milwaukee*, *WI*

**Background:** Children with cyclic vomiting syndrome (CVS) have complex medication and lifestyle management needs. Medications are often under‐utilized due to complexity of treatment plans, resulting in more vomiting, missed school and work, and increased health care utilization. The CVS Pictograph Action Plan (CVSAP) is an interactive medication management tool available to the public through the Darnall Medical Library for creation of an individualized treatment plan at a fifth grade reading level with adjacent pictographs using green, yellow, and red zones to help patients understand when to take medications based on symptoms. The primary objective of this quality improvement (QI) project is to offer patient‐centered medication education meeting health literacy preferences of patients to assist with patient understanding of complex treatment plans. The secondary objective is to document patient acceptability of the CVSAP to promote sustainable evidence‐based practice change. The intervention was developed in the nursing context of the Individual and Family Self‐Management Theory (IFSMT). The QI model utilized is the Iowa Model of Evidence‐Based Practice to Promote Excellence in Healthcare.

**Aims:**

1. To offer CVSAP to 90% of patients with CVS seen in clinic within 3 months.

2. To document patient acceptability rates of the CVSAP by families in clinic the first visit and at subsequent visits to promote sustainability and continued use by clinic staff based on understanding of patient preferences.

3. To gather feedback from families in the context of patient‐centered care.

**Methods:** The office of nursing research at Children's Wisconsin deemed this project to be quality improvement rather than human subject research. This project implements offering the CVSAP vs. standard discharge instructions to CVS patients in clinic, documenting if they accept it, if it was the first time they received it, and feedback provided by families. The CVSAP has been piloted in the NP led clinic with support of a nurse practitioner student and in the second phase is being rolled out in MD led clinic with support of nursing staff.

**Results:** The CVSAP was offered to >90% of patients with chief complaint of CVS in the first 3 months. 79% of patients with CVS as chief complaint were given a CVSAP based on patient preferred instructions within the first 3 months, and 92% of these received the CVSAP for the first time. Feedback from families included that it would be helpful for the patient, parent, other caregivers and school to keep track of medicines and that it is easier to follow for each phase of CVS than standard instructions. Reasons that families did not accept the offered CVSAP included having a smaller number of medications and already improved CVS status.

**Discussion:** Limitations include that the CVSAP is only available in English, and time constraints in clinic. Time constraints limiting sustainability of evidence into practice are also a driver of this QI project and are addressed by the CVSAP replacing other written medication instructions in clinic, and multidisciplinary team‐based care in clinic. As the NP student supporting this project has graduated, RN support will be crucial for maintaining commitment to the practice change. The successful implementation of this practice change and feedback that patients prefer the CVSAP over standard education also promotes continued utilization of this patient‐centered health literacy tool. Future cycles include expanding this intervention into MD led CVS clinic and evaluation of patient preferences for receiving CVSAP again vs. written instructions at subsequent visits.

## 622 MITIGATING FOOD INSECURITY IN WASHTENAW COUNTY SCHOOLS: A PILOT STUDY IMPLEMENTING A COMMUNITY‐CENTRIC INTERVENTION

*Rija Awan* ^*1*^, *Fatema Dohadwala* ^*2*^

^*1*^ *Medical School*, *University of Michigan Michigan Medicine*, *Ann Arbor*, *MI*; ^*2*^ *The University of Texas at Austin Dell Medical School*, *Austin*, *TX*

**Background:** Food‐insecure children are twice as likely to be in fair or poor health, are more prone to hospitalization, and more likely to experience mental health issues. In Washtenaw County, MI, 10% of the population faces food insecurity, with students at Title I schools in Washtenaw County facing heightened food insecurity challenges. To address this issue, we established Washtenaw County's first‐ever “Care Closets” in six Title I elementary classrooms and assessed their impact on food insecurity and child behavior. We hypothesized that establishing care closets within schools would mitigate food insecurity during the school day and subsequently improve student behavior.

**Description:** Through community fundraising and grants, our team created Care Closets with nutritious, non‐perishable snacks in six Carpenter Elementary classrooms. Post‐implementation, teachers completed a survey to evaluate the impact of Care Closets on student behavior and food security.

**Results:** Three out of six classrooms participated in the post‐implementation survey, with 86 students accessing the Care Closets. Initially, 66% of classrooms reported 1‐5 students lacking food, and 33% reported 6‐10 students without food. After the implementation of the Care Closets, 100% of classrooms reported only 1‐5 students without food, indicating a potential decrease in food insecurity. An average of 10 students in each classroom used the Care Closet weekly. Most notably, 100% of teachers observed improved student mood, 66% noted increased focus, and 33% reported fewer behavioral issues, suggesting positive impacts on student behavior and educational outcomes.

**Conclusion:** Care Closets offer a creative approach to mitigating food insecurity among students. Further community‐engaged programs are essential to tackle the underlying causes of food insecurity in underserved communities, which will impact the long‐term educational and health outcomes of students.


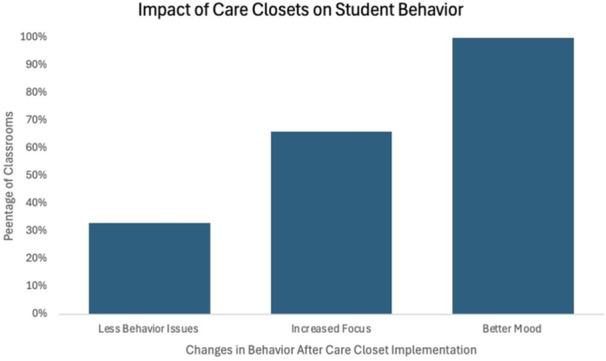


## 623 CARING FOR CHILDREN WITH DOWN SYNDROME WHO HAVE NUTRITION AND FEEDING DIFFICULTIES: IMPLICATIONS FOR CLINICIANS

*Emma Marston* ^*1*^, *Michele Polfuss* ^*1,2*^

^*1*^ *School of Nursing*, *University of Wisconsin‐Milwaukee*, *Milwaukee*, *WI*; ^*2*^ *Milwaukee Hospital‐Children's Wisconsin*, *Milwaukee*, *WI*

**Background:** Children with Down syndrome have high risks for feeding, nutrition, and digestive challenges. However, there is limited research on nutrition and feeding for children with Down syndrome, including how interactions with healthcare providers contribute to how families manage their children's feeding, nutrition, or digestive concerns. The purpose of this study was to explore mothers’ experiences related to food and feeding their children with Down syndrome.

**Methods:** Twenty‐nine mothers of children ages 3‐10 years with Down syndrome across the United States participated in this virtual qualitative study. Data collection methods included semi‐structured interviews and photo‐elicitation. The interview guide included questions on how family, school, and healthcare provider interactions contribute to daily family management of their children's feeding and nutrition. Thematic analysis methods were used to develop themes and subthemes from the data.

**Results:** Of the 29 participants’ children with Down syndrome, 28 experienced some type of feeding or nutritional challenge. Challenges ranged from mild to severe and included food selectivity, texture sensitivity, swallowing and choking problems, food allergies, and difficulties eating too little or too much. Nine children required daily calorie supplementation, and four children had a feeding tube at the time of the study. Thirteen children had a feeding tube at some point in their life. Nine children had food allergies or intolerances. Two children had celiac disease, and two children had Food Protein‐Induced Enterocolitis Syndrome (FPIES).

Families interacted with healthcare providers frequently to manage their children's feeding, nutritional, and digestive needs. Three main themes related to healthcare provider interaction included 1) Access, Provider Expertise, and Maternal Concern; 2) Infant Feeding Experiences Matter; and 3) Mothers are Experts on and Advocates for Their Children. Participants described challenges accessing providers who were knowledgeable about caring for children with Down syndrome. Many participants’ children experienced feeding challenges during infancy, and mothers did not always feel supported by clinicians in meeting their feeding goals when children were infants. Mothers were strong advocates for their children's needs and described limiting assumptions that healthcare providers conveyed about their child's abilities due to the diagnosis of Down syndrome.

**Conclusion:** Findings from this study highlight unmet healthcare needs for children with Down syndrome and their families. Implications for current practice include the need for provider education on caring for children with Down syndrome specific to feeding, nutrition, and digestion, and the importance of listening to parents and providing evidence‐based clinical recommendations to families.

## 624  A HYBRID CULINARY MEDICINE MODEL ENHANCES ACCESS AND NUTRITION EDUCATION FOR YOUTH WITH OBESITY

*Sharon Weston* ^*1,4*^, *Maura McNamara* ^*1,4*^, *Brucaj Lirona* ^*2*^, *Erin McShane* ^*1,4*^, *Sarah Federoff* ^*1,4*^, *Emily Rizzitano* ^*3*^

^*1*^ *Center for Nutrition*, *Boston Children's Hospital*, *Peabody*, *MA*; ^*2*^ *Boston University*, *Boston*, *MA*; ^*3*^ *Boston Children's Hospital*, *Boston*, *MA*; ^*4*^ *Department of Endocrinology*, *Boston Children's Hospital*, *Boston*, *MA*

**Objective:** Culinary Medicine (CM) sessions provide patients with practical tools to support dietary adherence. This is particularly critical during weight loss interventions, where optimizing nutrient intake and preventing the loss of lean body mass are key clinical goals. Our aim was to increase access to CM sessions in the Boston Children's Hospital Empower program by utilizing a hybrid approach for the cooking component of the classes.

**Methods:** The Empower Program expanded its virtual CM classes to include two formats: (1) class which incorporated live interactive cooking sessions, where patients cook simultaneously in their homes while following a registered dietitian who instructed from a kitchen, and (2) class which incorporated pre‐recorded instructional videos demonstrating recipe preparation, where patients can choose to cook simultaneously or prepare the recipe at their convenience, and the instructor did not require to be in a kitchen to lead class. Sessions were offered weekly to accommodate varying patient schedules. Each class was led or supervised by a registered dietitian, and billing was conducted using CPT code 97804 for group medical nutrition therapy (2 units, 60 minutes). Classes highlight a monthly theme, review nutrition concepts consistent with weight loss, provide key dietary strategies when using anti‐obesity medications (AOMs) including glucagon‐like peptide‐1 receptor agonists (GLP‐1 RAs), and feature recipes consistent with the nutrition prescription (Table 1). Tips from the Boston Children's Hospital Fit Kit (https://www.childrenshospital.org/programs/new-balance-foundation-obesity-prevention-center/boston-childrens-fit-kit) are also featured to discuss exercise, stress, sedentary life, and sleep (Table 2). Recipes are prepared and recorded by a registered dietitian, and the video content is integrated into the class presentation slides. Each recipe is analyzed for nutritional content using Cronometer to generate a Nutrition Facts label. Recipes are distributed to patients one week in advance of each session to allow time to shop for recipe ingredients. Following the class, both the recorded video and corresponding recipes are made accessible via the hospital's social media homepage.

**Results:** The implementation of recorded CM segments significantly increased program capacity, expanding patient access from a minimum of 25 participants per month up to 125 participants per month. The dual‐format approach allowed for greater scheduling flexibility and enabled patients to engage with the material at their own pace. The model proved feasible and sustainable within clinical operations, with reimbursement available through standard nutrition group billing codes.

**Conclusions:** Integrating recorded CM sessions into existing virtual programming is an effective approach to increasing patient access while maintaining clinical quality. This hybrid delivery model enhances patient flexibility, supports adherence to nutrition prescriptions, and serves as a practical tool in preserving nutritional status and lean body mass during weight loss interventions. The approach is reproducible and can be implemented across diverse healthcare settings to support long‐term dietary behavior change.


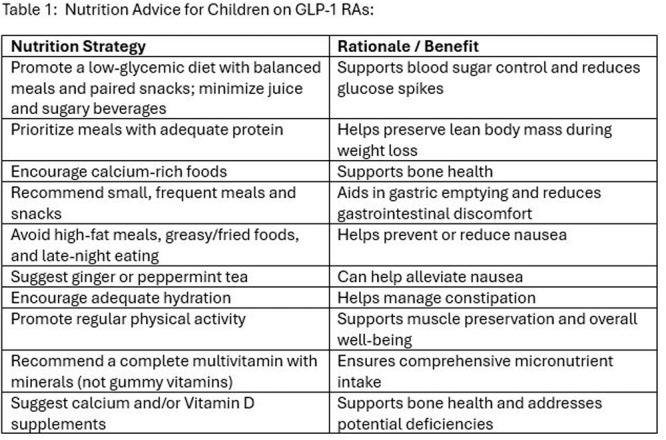


Table 1


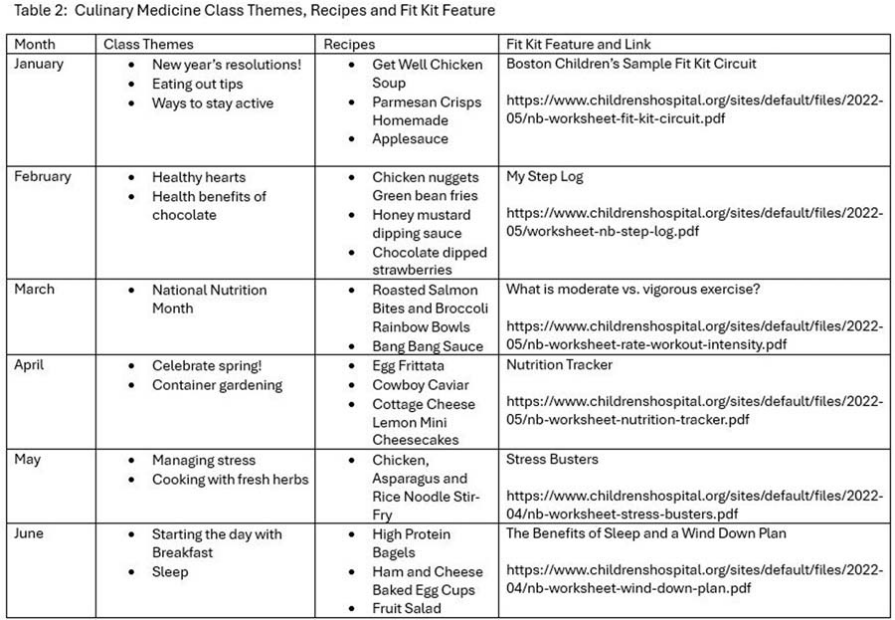


Table 2

## 625 ROUTINE NUTRITIONAL BIOMARKER SCREENING IN NEWLY DIAGNOSED PEDIATRIC INFLAMMATORY BOWEL DISEASE PATIENTS: A COMPARATIVE ANALYSIS BASED ON MALNUTRITION STATUS

*Julia Caron*, *Julia Driggers*

*Nutrition*, *The Children's Hospital of Philadelphia*, *Philadelphia*, *PA*

**Background:** Pediatric inflammatory bowel disease (IBD), including Crohn's disease (CD) and ulcerative colitis (UC), is frequently associated with nutrient deficiencies due to chronic inflammation, decreased intake, and malabsorption. These nutritional impairments can significantly impact growth, immune function, and disease outcomes. While malnutrition is a well‐recognized complication of IBD, micronutrient deficiencies may also be present in patients who are not definitively malnourished. Routine screening of key biomarkers such as 25‐hydroxyvitamin D (Vitamin D), zinc, and methylmalonic acid (MMA) — a functional marker of vitamin B12 status — may provide early identification of deficiencies and improve clinical management. However, the utility and yield of routine screening in the context of pediatric malnutrition status at diagnosis has not been fully studied.

**Objective:** To evaluate the prevalence and clinical utility of routine screening for Vitamin D, zinc, and MMA in newly diagnosed pediatric IBD patients and compare deficiency rates between those with and without pediatric malnutrition at diagnosis.

**Methods:** A retrospective cohort study was conducted involving pediatric patients (age <18 years) newly diagnosed with IBD during inpatient admission at Children's Hospital of Philadelphia (CHOP) between January 2024 and May 2025.

Inclusion criteria included documented diagnosis of IBD confirmed by clinical, endoscopic, radiographic, and histologic findings, and availability of laboratory assessments for Vitamin D, zinc, and MMA within 30 days of diagnosis. Patients were stratified into two groups based on the presence or absence of malnutrition at diagnosis, defined using AND and ASPEN pediatric malnutrition criteria.

Patients with very early onset (VEO‐IBD) subtype were excluded.

Demographic data, anthropometric measurements, and inflammatory markers (CRP or ESR, albumin) were collected. Nutritional biomarkers were assessed using standardized clinical laboratory methods. Deficiency cutoffs were defined as: Vitamin D <30 ng/mL (deficient), Zinc <60 µg/dL (deficient), and MMA >400 nmol/L (elevated).

**Results:** A total of 20 pediatric patients were included (CD: n=15; UC: n=2; indeterminate: n=3), with 18 (90%) meeting criteria for pediatric malnutrition at diagnosis (50% severe malnutrition). Overall prevalence of deficiencies: Vitamin D (80%), zinc (30%), and elevated MMA (0%).

**Conclusions:** Micronutrient deficiencies, particularly Vitamin D and zinc, are prevalent at the time of IBD diagnosis in pediatric patients. Malnutrition does not appear to be an dependent factor of vitamin D deficiency, which highlights the importance of routine micronutrient screening in all newly diagnosed pediatric IBD patients.

Of note, 85% of patients had zinc levels <80 µg/dL. Our patients may benefit from higher achieving higher serum levels to promote intestinal healing and replete zinc losses from stool output. Patients with Crohn's Disease had a higher incidence of zinc deficiency compared to UC and indeterminate subtypes.

No patients were identified with vitamin B12 deficiency across all subtypes and degree of malnutrition.

Limitations include laboratory assay accuracy. Due to the inflammatory nature of IBD patients present at new diagnosis, they are likely to have elevated pro inflammatory markers (CRP, ESR). Zinc is an acute phase reactant and serum zinc levels checked at first diagnosis should be interpreted with this consideration.

Early identification and correction of deficiencies may have implications for disease progression, immune response, and growth outcomes. We recommend incorporating routine assessment of vitamin D, zinc into standard diagnostic workups for pediatric IBD, regardless of nutritional status at presentation. We do not recommend routine monitoring of MMA levels at this time. Vitamin B12 status is to be assessed on a case‐by‐case basis depending on portion of disease involvement (ie: terminal ileum), CBC (MCV) results, and baseline diet recall.

## 626 CAREGIVER‐REPORTED BARRIERS AND FACILITATORS TO EARLY AND SUSTAINED ALLERGENIC FOOD INTRODUCTION IN INFANTS: A MIXED‐METHODS SYSTEMATIC REVIEW

*Audrey Su* ^*1*^, *Camille Lyu* ^*1*^, *Edmond Chan* ^*2,3*^, *Lianne Soller* ^*2,3*^, *Jennifer Protudjer* ^*4,5*^, *Stephanie Erdle* ^*2,3*^, *Brock Williams* ^*2,3*^

^*1*^ *Food, Nutrition and Health*, *The University of British Columbia*, *Vancouver*, *BC*, *Canada*; ^*2*^ *Pediatrics*, *The University of British Columbia*, *Vancouver*, *BC*, *Canada*; ^*3*^ *BC Children's Hospital Research Institute*, *Vancouver*, *BC*, *Canada*; ^*4*^ *Department of Pediatrics and Child Health*, *University of Manitoba*, *Winnipeg*, *MB*, *Canada*; ^*5*^ *University of Manitoba Children's Hospital Research Institute of Manitoba*, *Winnipeg*, *MB*, *Canada*

**Background:** For the primary prevention of food allergy, several national professional organisations, including the Canadian Paediatric Society and American Academy of Pediatrics, recommend that commonly allergenic foods, particularly peanut and egg, are introduced in non‐choking forms to infants at higher risk for food allergy around 4‐6 months of age, and to infants at lower risk around 6 months of age. Guidelines emphasize that once introduced, these foods should be fed regularly to maintain tolerance. However, evidence suggests that caregivers may struggle to adhere to recommendations for early introduction and sustained feeding once commonly allergenic foods have been introduced.

**Objective:** To examine caregiver‐reported barriers and facilitators to the early introduction and sustained feeding of commonly allergenic foods (egg, cow's milk, peanut, tree nuts, wheat, sesame, fish, shellfish, and soy) in infants.

**Methods:** We searched 5 databases (MEDLINE, EMBASE, CENTRAL, CINAHL, and PsycInfo) for relevant articles published between 2008 and July 2024. Quantitative, qualitative, and mixed methods studies that identified caregiver‐reported experiences feeding an infant aged ≤12 months commonly allergenic foods were eligible for inclusion. Extracted data from selected studies were synthesized using the Joanna Briggs Institute (JBI) convergent integrated approach and then analyzed using a reflexive thematic synthesis approach. JBI Critical Appraisal Checklists were used to assess study quality. This review is registered with PROSPERO (Registration Number: CRD42023444861).

**Results:** A total of 4161 references from 4153 unique studies were identified. After removing duplicates (N=1353), 2800 records were screened by title and abstract, and 40 full‐ text articles were assessed for eligibility. Nine studies met the inclusion criteria: five cross‐sectional studies, two qualitative studies, one randomized clinical trial, and one cohort study. All studies were appraised using the relevant JBI quality assessment checklists and deemed suitable for inclusion. The included studies were conducted in the United States of America (N=4), Europe (N=4), and Australia (N=1). More barriers than facilitators were identified. Five main themes related to barriers in introducing and feeding commonly allergenic foods to infants were identified: (1) parental concerns and fears, (2) practical and lifestyle constraints, (3) infant‐related feeding difficulties, (4) healthcare system challenges, and (5) parental beliefs and trust in recommendations. One main theme related to facilitators was identified, namely educational and healthcare supports.

**Conclusions:** Parental, infant, and system‐level barriers continue to limit adherence to guidelines for early and sustained feeding of commonly allergenic foods. Support from healthcare professionals and accessible, practical guidance may help address these challenges. Targeted interventions are also warranted to promote the uptake of evidence‐based feeding practices.

## 627 IMPROVED TOLERANCE, METABOLIC STABILITY, AND QUALITY OF LIFE IN A MEDICALLY COMPLEX PATIENT WITH GASTROINTESTINAL DYSMOTILITY USING A TAPIOCA CARBOHYDRATE‐BASED JUNIOR AMINO ACID FORMULA

*Madden Wilson* ^*2*^, *Kelli Miller* ^*1*^

^*1*^ *Medical Affairs*, *Ajinomoto Cambrooke*, *Ayer*, *MA*; ^*2*^ *GI for Kids*, *Knoxville*, *TN*

**Background:** Following a 13‐month hospitalization for congenital heart disease repair with multiple complications, a 3‐year‐old female transitioned to a pediatric gastroenterology clinic for outpatient care. Post surgical complications resulted in multiple diagnoses including feeding difficulties and gastrointestinal (GI) dysmotility requiring a gastro‐jejunal feeding tube (GJT), chronic renal disease (CKD), and necrotizing pancreatitis. This report investigates using a junior amino acid formula with tapioca as the carbohydrate source following poor tolerance of other junior amino acid‐based formulas.

**Case Presentation:** Upon presentation to the GI clinic, the patient's enteral feeding regimen was a combination of a junior amino acid formula with corn syrup solids as the carbohydrate source and a protein‐ free modular via GJT for 18 hours/day. Patient's GI symptoms included watery diarrhea 6‐8 times per day and persistent vomiting despite continual venting of GT (output of 100‐130 mL/day). Medications included long acting and sliding scale insulin (SSI), pancreatic enzyme replacement cartridge used in conjunction with tube feeding for pancreatic insufficiency, and a gastric motility agent.

At the time of initial evaluation by the registered dietitian, the patient's calorie provisions had recently been decreased due to BMI‐for‐age measurement > 99%ile (z‐score: 2.03 SD) and ongoing rapid weight gain. Enteral nutrition provided 62 kcal/kg and 1.3 g protein/kg. Despite stage 3b CKD diagnosis, nephrology confirmed patient was not on a specific protein restriction. Notable labs included BUN 64 and creatinine 0.76. Blood glucose was noted to be 318.

One month after the initial evaluation, the nephrologist inquired about changing the amino acid formula to reduce the intake of corn syrup solids and the patient was then transitioned to a junior amino acid formula with tapioca as the carbohydrate source.

Within 1‐2 weeks of being on the new formula, the overall clinical picture improved. Urine uric acid levels decreased from 14 to 12. GI symptoms had improved based on caregiver report of 1‐2 loose bowel movements per day and minimal emesis. Within 1 month, the patient was able to transition from GJT to GT feeds due to improved formula tolerance. Patient was also able to wean off long‐acting insulin due to improved blood glucose control.

While establishing insurance coverage for the new formula, the patient trialed a peptide pea protein‐based formula that resulted in increased vomiting. Vomiting then resolved after transitioning back to the junior amino acid formula with tapioca as the carbohydrate source.

After 3 months, clinical improvement and enhanced quality of life were evident. It was noted that the patient had not used SSI in >1 month. Lab values including BUN 30 and creatinine 0.6 indicated stable or improved renal function. Due to improved GI symptoms, patient was able to slowly increase the rate of infusion via GT and compress feeding schedule to run over 16.4 hours/day.

At the 4‐month follow‐up, patient was receiving 68 kcal/kg and 1.91 g protein/kg daily from feeding regimen of the junior amino acid formula with tapioca as the carbohydrate source combined with a protein‐free modular. Observations following the change in feeding regimen and formula included achievement of desired weight loss as well as improved linear growth. BMI‐for‐age improved to 75.9%ile (z‐score: 0.7 SD).

As this patient continues to be followed on the new formula, the next steps include weaning off the motility agent, adjustment of calorie provisions to promote desired growth, and plans to advance oral diet.

**Discussion:** In this case, a junior amino acid formula with tapioca as the carbohydrate source was well tolerated and may have contributed to an improved overall clinical picture. GI symptom improvement, specifically resolution of vomiting and transition to gastric feeds, as well as decreased diarrhea and diaper rash were noted. Within the overall clinical picture, average blood glucose levels decreased from the 300 s to the 100 s in addition to weaning insulin. Higher protein content of the formula was noted to be tolerated from a renal standpoint. Additionally, the pancreatic enzyme replacement cartridges were successfully used with this formula, despite this formula not currently being included on the product's formula compatibility list. This case highlights the role of a tapioca carbohydrate‐based junior amino acid formula in a medically complex pediatric patient with GI dysmotility with observed outcomes of improved tolerance, metabolic parameters, and quality of life.

## 628 ASSOCIATION OF FOOD INSECURITY WITH FEEDING TUBE DEPENDENCE AND INCREASED HEALTHCARE UTILIZATION IN PEDIATRIC GASTROENTEROLOGY

*Nicole Misner* ^*1*^, *Athanasios Tsalatsanis* ^*2*^, *Chaitanya Chaphalkar* ^*2*^, *Racha Khalaf* ^*1*^

^*1*^ *Gastroenterology, Hepatology, and Nutrition*, *USF Health*, *Tampa*, *FL*; ^*2*^ *University of South Florida Morsani College of Medicine*, *Tampa*, *FL*

**Introduction:** Food insecurity (FI), characterized as limited or uncertain access to adequate food, represents a substantial public health challenge, with demonstrable adverse effects on children's physical health, growth trajectories, and developmental outcomes. Previous research has reported feasibility of universal FI screening in pediatric gastroenterology clinics.

The aims of the study were 1) to characterize differences between individuals who screen positive versus negative for FI seen in a pediatric gastroenterology clinic including demographics, anthropometric data, gastrointestinal diagnoses, and presence of dietary restrictions and 2) identify specific gastrointestinal diagnoses associated with greater health care utilization among individuals who screen positive for FI.

**Methods:** We performed a retrospective study of visits with FI screens from August 2022 – March 2025 in the outpatient clinics of the Division of Pediatric Gastroenterology at the University of South Florida (USF). Patients are universally screened in this division for FI using the two‐item, Hunger Vital Sign™ Screener embedded in the electronic medical record at every visit. Data collected include the screener, demographics, anthropometrics, gastrointestinal diagnoses, self‐reported food allergies, emergency department (ED) utilization, hospitalizations, and no showed or missed office visits. Exception wavier was received by the USF Institution Review Board for this retrospective chart review.

**Results:** A total of 3,527 visits had screens for FI completed for 1,789 unique individuals during the study period. Demographics are described in Table 1. Individuals that screened positive for FI were more likely to be Black or African American (P<0.001), Hispanic or Latino (P<0.001), report Spanish as their preferred language (P<0.001), and use Medicaid insurance (P<0.001). Table 2 compares gastrointestinal diagnoses between the cohorts. FI was associated with a diagnosis of enteral feeding tube, (P<0.001). The FI cohort had a greater percent of individuals with missed office visits (43% vs 28%, P<0.001), emergency department visits (27% vs 16%, P<0.001), and hospitalizations (15% vs 8%, P<0.001). Additional analysis excluding those with feeding tubes, showed the food insecure cohort continued to have an increased percent of individuals with missed office visits (38% v 24%, P<0.001), ED visits (17% vs 13%, P=0.035), and hospitalizations (9% vs 5%, P=0.038).

**Conclusion:** We sought to identify characteristics of individuals screening positive for FI in a pediatric GI clinic to determine whether associations exist. Individuals that screened positive for FI were more likely to be Black or African American, Hispanic or Latino, report Spanish are their preferred language and use Medicaid insurance. FI was associated with feeding tube dependence. In addition, FI was associated with increased missed office visits, ED visits, and hospitalizations in pediatric GI patients.


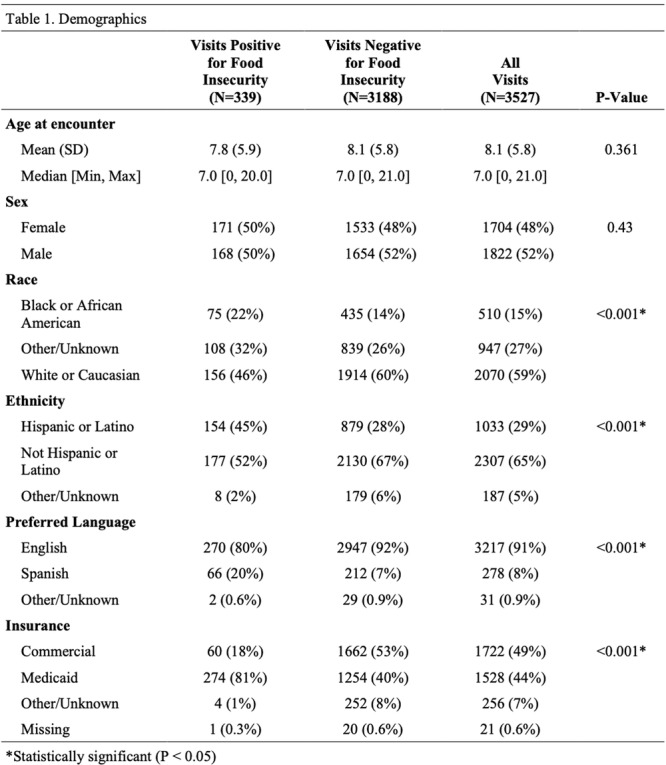



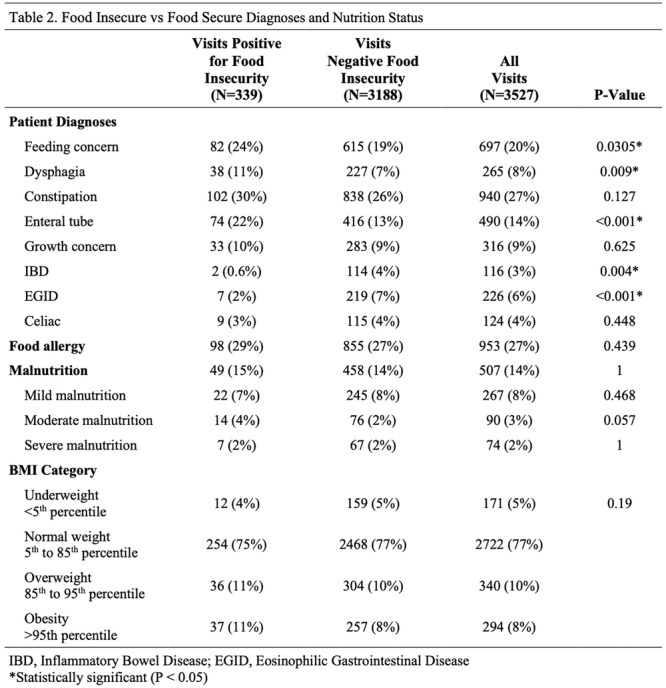


## 629 ARE PRACTICE GUIDELINES FOR NUTRITION COUNSELING IN ADOLESCENTS ON GLP‐1RAS A PIPE DREAM? A SURVEY OF CURRENT PRACTICE

*Christy Figueredo* ^*1*^, *Amy Braglia‐Tarpey* ^*2*^, *Venus Kalami* ^*3*^

^*1*^ *Pediatric Gastroenterology*, *University of Miami*, *Coral Gables*, *FL*; ^*2*^ *Director, Nutrition*, *Amerita Specialty Infusion*, *Green Wood Village*, *CO*; ^*3*^ *Medical Science Liaison*, *Nutricia North America Inc*, *White Plains*, *NY*

**Background:** Glucagon‐like peptide‐1 receptor agonists (GLP‐1 RAs) have been approved for adolescent obesity management since 2022. Research to date in adolescents has largely focused on the impact of GLP‐1 RAs on weight and BMI, and to a lesser degree, nutritional quality of pediatric diets. The American Academy of Pediatrics (AAP) recommends that weight loss pharmacotherapy be used in conjunction with 26 contact hours of nutrition, exercise, and lifestyle counseling within a 3‐12 month period. However, research regarding the extent of lifestyle counseling offered in conjunction with GLP‐1 RAs use is limited.

**Research Question:** What are current practices and experiences of RDNs providing lifestyle counseling to adolescents prescribed GLP‐1 RAs for weight loss indications?

**Methods:** A sixteen‐question survey was developed and distributed to pediatric RDNs through professional listservs, emails, and LinkedIn. Responses were collected from January –April 2025. Professionals who were not credentialed as a RDN and/or who did not primarily work with patients < 18 years old were excluded.

**Results:** 64 RDNs responded to all questions. Results demonstrate that patients do not consistently receive lifestyle counseling before prescription of GLP‐1 RAs (Table 1). RDNs are not consistently involved in the care of patients on GLP‐1 RAs. When RDNs are involved, their roles are varied and include nutritional screenings, assessments, and counseling; as well as screening for other social and environmental drivers of health.

Additionally, responses suggest that reimbursement pathways for nutrition services and GLP‐1RAs are unclear to RDNs and generally inconsistent in nature (Table 2). Most RDNs (72%) endorse that there is inadequate staffing and resources available to support the AAP's recommendation for 26 hours of lifestyle counseling within 3‐12 months.

**Discussion:** These findings raise concerns for: gaps in pediatric nutrition care, inability of families to access and afford lifestyle counseling, systemic health inequities, guideline recommendations mismatched with institutional capacity, and lack of representation of RDN perspectives in providing lifestyle counseling.

Strengths of this study include a robust survey sample size, in addition to thorough survey questions, and representation from experts in nutrition and lifestyle counseling (RDNs) from various settings across the country, who have generally been underrepresented in the academic discourse around GLP‐1 RA use and best practices in adolescents. Limitations include self‐selection bias, possible dual entries from a single facility, as well as lack of detailed demographic data.

Future research studies should seek to explore gaps in nutrition and lifestyle counseling for adolescent patients on weight loss pharmacotherapy, as well as, build resources such as a decision tree for the pediatric clinician to help better guide nutritional care and protocols. Clinical investigation of the impact of GLP‐1RAs on nutritional quality and sufficiency of the adolescent diet should also be undertaken in order to develop better guidance for monitoring and nutrition intervention during and after their use.


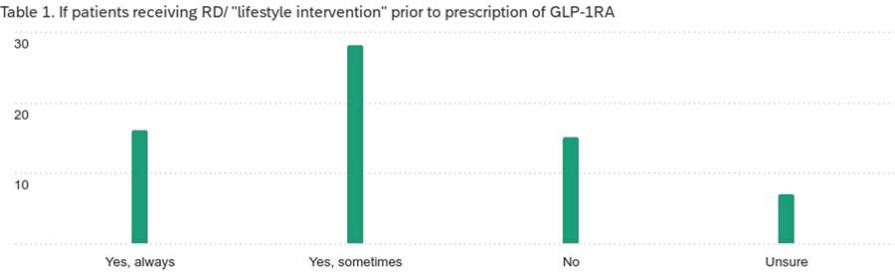



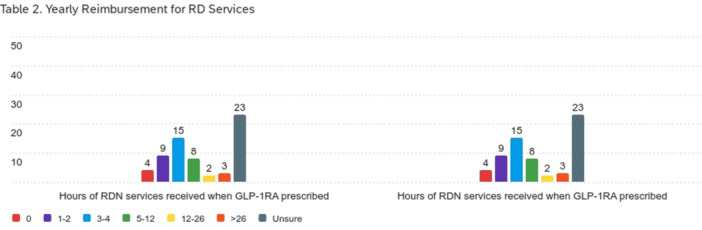


## 630 OPTIMIZING PEDIATRIC CELIAC DISEASE FOLLOW UP: WHAT FAMILIES WANT FROM PROVIDERS AND TIMING OF CARE

*Sadie Nagle* ^*1*^, *Monique Germone* ^*1,4*^, *Adyson Hill* ^*2,3*^, *Michelle Knees* ^*4*^

^*1*^ *Colorado Center for Celiac Disease, Digestive Health Institute*, *Children's Hospital Colorado*, *Aurora*, *CO*; ^*2*^ *Creighton University*, *Omaha*, *NE*; ^*3*^ *Peds Gastro Genops*, *University of Colorado Anschutz Medical Campus School of Medicine*, *Aurora*, *CO*; ^*4*^ *University of Colorado Anschutz Medical Campus School of Medicine*, *Aurora*, *CO*

**Background:** Celiac disease (CD) is a common, lifelong autoimmune disorder that causes nutrient malabsorption due to gluten‐induced intestinal damage. The only treatment is a strict gluten‐free diet (GFD), which, while effective in reducing complications, is burdensome, costly, and negatively affects quality of life. Current guidelines recommend dietary education with a dietitian, follow‐up with a gastroenterologist, and undergoing a standard dietitian evaluation to assess dietary adherence. However, little is known about what pediatric patients and their caregivers want and benefit from, particularly regarding multidisciplinary support to alleviate the burdens of treatment. Of the few studies conducted, most adult patients felt they could follow up annually with their primary care provider (77%), followed by a gastroenterologist (57%) and dietitian (43%). No studies have investigated the preference and impact in pediatric populations, nor have they included care with a behavioral health providerdespite evidence to support the psychosocial needs in pediatric CD. The current study aims to identify pediatricpatients' preference of provider type and timing of care provided in a pediatric multidisciplinary CD center.

**Setting and Population:** Participants include all pediatric patients (anticipated ages 1–17 years, n ≈ 166) seen between May 2025–October 2025 in a multidisciplinary CD center within a Western tertiary Children's Hospital. Center providers include expert gastroenterologists, a nurse practitioner, a dietitian, and a psychologist. A unique feature of the center is an integrated joint visit that focuses on treatment adherence with both the dietitian and psychologist.

**Methods:** This is a single‐site, cross‐sectional, observational study. Caregivers of pediatric CD patients are asked to complete anonymous web‐based surveys following each visit with a provider at the CD center. Surveys assess perceived importance of provider visits (gastroenterologist, dietitian, psychologist), preferences for frequency of follow‐up, and satisfaction with integrated care.

**Results:** Data collection is ongoing and will be updated for the poster presentation. Based on prior research, it is anticipated that caregivers of pediatric patients, prior to a formal CD diagnosis, will report a preference for meeting with the gastroenterologist and dietitian. At the initial follow‐up visit with the gastroenterologist, caregivers will report that the recommended 3‐month interval is both beneficial and preferred. Consistent with existing literature, annual follow‐up visits may be preferred thereafter. As of the date of this abstract submission, 100% of eligible caregivers (n=10) participated in the survey regarding the integrated dietitian and psychologist adherence visit. All respondents who answered the question about provider preference (100%, n=9) indicated they would prefer to meet with the dietitian and psychologist together in an integrated visit, rather than separately. When asked how important it was to meet with a dietitian before the integrated visit, caregiver responses (n=9) ranged from not important (n=2, 22%) to very important (n=3, 33%), with an average stating it was important. Following the integrated visit, caregivers reported viewing the dietitian visit as somewhat important (n=1, 11%) to very important (n=6, 66%), with an average stating it was important. When asked how important it was to meet with the psychologist before the integrated visit, caregiver responses (n=9) ranged from somewhat important (n=4, 44%) to very important (n=1, 11%), with an average of important. After the integrated visit, caregivers reported that meeting with a psychologist was important (n=2, 22%), to very important (n=5, 55%), with an average of very important.

**Conclusions:** This study addresses a critical gap in understanding caregiver preferences for multidisciplinary care in pediatric CD, a population where such insights have been largely unexplored. Identifying how families perceive and value medical, as well as integrated nutrition and behavioral health services, is essential to improving adherence, satisfaction, and outcomes. Preliminary findings suggest that while caregivers initially perceived nutrition and behavioral health services as less critical, their perceived value increased following integrated visits. This highlights the importance of multidisciplinary care in managing pediatric CD and supports the continued integration of nutrition and behavioral health into routine follow‐up. Ongoing data collection will further clarify caregiver preferences regarding provider type and timing of care, with the potential to inform clinical guidelines and optimize patient‐centered care models for pediatric CD. These findings may help shape future models of care to reduce the burden of care, improve access to supportive services, and strengthen long‐term adherence to the GFD in pediatric CD.

## 631 MATERNAL ELIMINATION DIETS IN THE SETTING OF FOOD PROTEIN‐INDUCED ALLERGIC PROCTOCOLITIS

*Diana Schnee*, *Senthil Sankararaman*

*Gastroenterology*, *Cleveland Clinic Children's Hospital*, *Cleveland*, *OH*

**Background:** Food protein‐induced allergic proctocolitis (FPIAP) is characterized by inflammation in the distal colon due to immune‐mediated reactions against one or more food proteins. The common manifestations of FPIAP include blood and mucus in stools of infants. Milk and soy are the mostly commonly implicated foods in the pathogenesis of FPIAP. In breastfed babies, elimination of these foods from the maternal diet is often the initial step in the management of FPIAP. If the blood in the stool does not resolve, then subsequent practices vary among clinicians due to lack of guidelines. Some may initiate a protein hydroxylate (partial or fully hydrolyzed) or even amino acid‐based formulas, while others may pursue further maternal dietary elimination. These practices should be carefully chosen and must be individualized as there could be implications in disruption to maternal/newborn bonding, additional financial burden for the family from expensive formulas, and adverse effects on maternal physical and mental health.

**Case description:** The purpose of this presentation is to review three exclusively breastfed infants with bloody stools who had complete resolution with maternal dietary elimination of egg proteins alone. After consultation with breastfeeding medicine/pediatric gastroenterology specialists, mothers were initially advised to avoid milk and soy, which had no effect on bloody and mucus stools. Later, mothers were advised to initiate hypoallergenic/amino acid‐based formulas for supplemental feeding, and in one infant, cessation of breast feeding was recommended altogether and replace it with an amino acid‐based formula. The mothers of all three infants wished to pursue breastfeeding and were referred to a pediatric dietitian. As eggs were the third most common food protein incriminated in FPIAP, the elimination of eggs from maternal diet was recommended by the dietitian in addition to continuation of exclusive breastfeeding. All three mothers resumed milk and soy in their diet. Within a week, the infants had complete resolution of symptoms.

**Discussion:** Milk is the most common food implicated in FPIAP followed by soy. The role of egg proteins is still under‐recognized in FPIAP. In breast‐fed babies with refractory colitis symptoms due to FPIAP, elimination of eggs and other food proteins should be strongly considered before recommending cessation of breastfeeding. In general, dietary elimination of eggs is considered relatively easy compared to other food proteins such as soy or wheat. In the described cases, the dietitian played an important role in the successful treatment of allergic proctocolitis by egg elimination from the maternal diet, thereby augmenting maternal‐infant bonding and supporting the additional benefits of breast feeding. Further, the family did not have to buy expensive hypoallergenic formulas that were previously recommended. There is urgent need for additional research and framing of an evidence‐based, stepwise approach for instituting maternal dietary elimination in the setting of FPIAP as well as a systematic process for reintroduction of the trigger food. These strategies can help optimize maternal and fetal nutritional intake and allergen exposure.

## 632 PARENTERAL NUTRITION WEANING AND ENTERAL FEEDING ADVANCEMENT WITH THE USE OF SERACAL

*Katherine Bennett* ^*2*^, *Tracy Ruvolo* ^*3*^, *Carmyn Thompson* ^*1*^

^*1*^ *Private Practice*, *Chicago*, *IL*; ^*2*^ *Children's Hospital of Orange County*, *Orange*, *CA*; ^*3*^ *Vytala*, *Fort Lauderdale*, *FL*

This clinical vignette describes a brief course of CC, a 19 year old parenteral nutrition (PN) dependent male having difficulty tolerating and gaining weight on enteral nutrition who is being trialed on Seracal. Seracal is a medical food composed of long chain fat, monoglyceride, and lecithin containing choline that creates a micelle and transports fat and fat‐soluble vitamins across the intestinal enterocytes without the need for bile salts and lipase. CC has a history of Trisomy 21, developmental delay, gastrostomy dependence and short bowel syndrom after a cecal volvulus resulted in the removal of his terminal ileum, ileocecal valve, and half of his colon. CC has been PN dependent since 4/2018. After multiple formula trials, CC had the most success with a real food based formula that allowed him to wean PN to 2‐3 days per week. He has remained on PN 2‐3 days/week for the last two years despite multiple attempts at weaning. Seracal was introduced 4/2025 and has increased to 9 TBSP daily. He continues to tolerate feeds and the goal is to trial off PN in the next 1‐2 months with the expectation that he will be able to maintain his weight off PN and with all enteral nutrition with the use of Seracal. This information will be updated with patient progress and additional outcomes well in advance of the poster in November.

## 633 CAN FAT ABSORPTION BE ENHANCED WITH A NOVEL MEDICAL FOOD?

*Cassandra Brown*

*Gastroenterology*, *Children's Medical Center Dallas*, *Dallas*, *TX*

A medical food (MF) (Seracal) consisting of pre‐digested fat with essential fatty acids, monoglycerides, and choline‐containing phospholipids has been developed to enhance fat/nutrient absorption. It has been clinically shown to enhance weight gain in patients with cystic fibrosis (CF). The MF comes in a powdered form, mixed with food, and consumed throughout the day or used as part of a tube feeding regimen. One case example demonstrates gastrointestinal symptoms improvement in non‐CF, malabsorption diagnosis.

A 4‐year‐old with history of Rett Syndrome, feeding difficulties, G‐tube with intolerance to various enteral nutrition formulas eventually transitioned to home blenderized feeds, continued issues with diarrhea and vomiting and Total Parenteral Nutrition (TPN) dependence. The patient receives 2 bolus feeds of home blenderized formula providing ~33% total calorie needs with the remaining nutrient requirements being met by TPN. The MF was added to the home blenderized formula and an immediate improvement in bowel movement frequency and consistency was reported.

An observational study of the MF for patients greater than 1 year of age with diagnoses such as short bowel syndrome, chronic diarrhea, and malabsorption is being conducted to further substantiate these findings.

## 634 PRESCRIPTION TRENDS, GUIDELINE COMPLIANCE, AND REPORTED SIDE EFFECTS OF PROTON PUMP INHIBITOR USE IN INFANTS UNDER ONE YEAR AT A TERTIARY HOSPITAL IN JORDAN

*Eyad Altamimi* ^*1*^, *Dalia Abulaila* ^*1*^, *Shahdan Al Dabbas* ^*1*^, *Karim Zaghloul* ^*1*^, *Sarah Alkhasawneh* ^*1*^, *Abeer AbuAlrub* ^*1*^, *Lina Abulaila* ^*1*^, *Mohammad Albatayneh* ^*2*^

^*1*^ *Pediatrics and Neonatology*, *Jordan University of Science and Technology*, *Irbid*, *Irbid*, *Jordan*; ^*2*^ *Jordan University of Science and Technology Faculty of Medicine*, *Irbid*, *Irbid Governorate*, *Jordan*

**Background:** Proton pump inhibitors (PPIs) are commonly prescribed in infants despite limited evidence for efficacy and safety in this age group. This study aimed to evaluate prescription trends, adherence to clinical guidelines, and reported adverse effects of PPIs in infants under one year at King Abdullah University Hospital (KAUH), Irbid, Jordan.

**Methods:** We retrospectively reviewed medical records and e‐prescriptions of 498 infants (<12 months) prescribed PPIs. Data included demographics, presenting symptoms, indications, dosing, duration of therapy, and reported side effects. Each case was evaluated for compliance with published guidelines.

**Results:** The mean age at PPI initiation was 4.9 ± 3.5 months; 37.1% were female. While dosing was appropriate in 90.4% of cases, only 48.4% had appropriate indications, and just 46.6% had guideline‐compliant duration. PPIs were frequently prescribed as gastric protection, particularly in patients receiving steroids (20.9%) or postoperatively (15.1%), despite limited indication. Common presenting symptoms included respiratory distress (38.8%), vomiting (29.9%), and poor feeding (23.3%). Reported side effects were uncommon but included), abdominal distention (1.4%), diarrhea (1.0%), allergic reaction (0.8%), and others.

**Conclusions:** Despite appropriate dosing, over half of PPI prescriptions in infants at our institution were not aligned with guideline‐recommended indications or duration. PPIs were often prescribed liberally for presumed gastric protection, particularly in the context of steroid use and postoperative care. Improved education and stewardship programs are needed to promote safe, evidence‐based PPI use in infants.


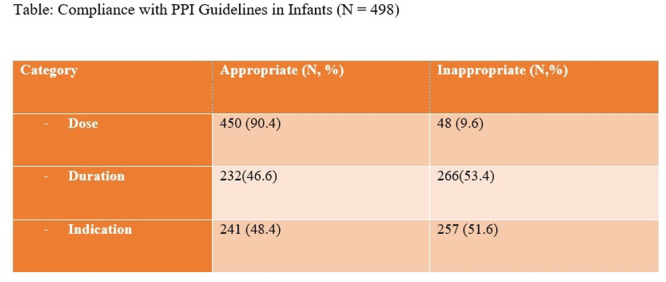


## 635 DEMOGRAPHIC DISTRIBUTION OF ENDOSCOPY IN INFLAMMATORY BOWEL DISEASE BEFORE AND AFTER THE COVID PANDEMIC

*Charlotte Banayan* ^*1*^, *Klaudia Cios* ^*2*^, *Maile Ray* ^*3*^, *Shahzaib Khan* ^*1*^, *Ashley Shayya* ^*3*^, *Sandra McGinnis* ^*3*^, *Thomas Wallach* ^*1*^

^*1*^ *SUNY Downstate Health Sciences University*, *Brooklyn*, *NY*; ^*2*^ *Maimonides Medical Center*, *New York*, *NY*; ^*3*^ *University at Albany*, *Albany*, *NY*

**INTRO:** Endoscopy is essential for the diagnosis and management of inflammatory bowel disease (IBD). Commonly used endoscopies for Crohn's Disease (CD) and Ulcerative Colitis (UC) include esophagogastroduodenoscopy (EGD), colonoscopy (COL), and sigmoidoscopy (SIG). We previously illustrated disparities in the distribution of endoscopic evaluation by race before and after the COVID pandemic. Here, we seek to assess what variation, if any, exists in the distribution of endoscopy by sex, ethnicity, and race prior to the COVID pandemic (2019) and after the pandemic (2024) in pediatric patients with CD and UC.

**METHODS:** We used TriNetX, a database of electronic medical records from >120 million patients across >50 healthcare organizations, to identify endoscopy utilization among patients ≤22 years of age before (2019) and after (2024) the COVID pandemic. We evaluated the total pediatric CD and UC populations and assessed who received each procedure within the same timeframe. We utilized Chi‐square analysis with a p‐value <0.05 to determine statistical significance.

Data was available for sex, ethnicity, and race. Sex categories were male or female. Ethnic categories were Hispanic or Latino (HL), Not Hispanic or Latino, and unknown ethnicity (UE). Racial subgroups included White, Black or African American (BAA), unknown race (UR), other race (OR), Asian, American Indian or Alaska Native (AIAN), and Native Hawaiian or Other Pacific Islander (NHOPI).

**RESULTS:** Prior to the pandemic in 2019, we identified 12,788 patients with CD (21% had EGD, 25% had COL, 1% had SIG) and 6,990 patients with UC (21% had EGD, 28% had COL, 4% had SIG). After the pandemic in 2024, we identified 14,115 patients with CD (22% had EGD, 26% had COL, 1% had SIG) and 8,201 patients with UC (20% had EGD, 30% had COL, 3% had SIG). Tables 1 and 2 provide percentage breakdown of sex, ethnicity, and race for each procedure as they relate to CD and UC in 2019 and 2024.

Among patients with CD in 2019, there is a significant difference among those who underwent EGD and those who underwent COL to those that did not by sex, ethnicity, and race; the difference in ethnicity and race persisted into 2024. There was also a significant difference in those who underwent SIG to those that did not by ethnicity in 2019.

We observed an increase in ethnic representation and percentage of SIG in HL patients with CD from 2019 to 2024. While the gap narrowed, HL representation in this group remained below HL representation of SIG. Similarly, there was a reduction in the amount of overrepresentation of procedures in the non‐HL population of CD patients in 2024.

Among patients with UC in both 2019 and 2024, there is a significant difference in demographics among patients who separately underwent EGD, COL, and SIG and those that did not by ethnicity and race. There is also a significant difference among patients who received COL to those that did not by sex.

We observed a decrease in percentage of White patients with CD and UC from 2019 to 2024 with less overrepresentation in procedures relative to the total respective disease population. The percentage of BAA with CD remained the same pre‐ and post‐pandemic; however, the percentage BAA with UC increased (as did those who underwent EGD and COL) in 2024.

**CONCLUSION:** Among patients with IBD in 2019 and 2024, there appears to exist a variety of demographic disparities among patients who received almost all scope types in 2019 and 2024 separately, and when comparing 2019 to 2024. In most cohorts, White patients are overrepresented in endoscopic frequency, and BAA patients underrepresented. Given that BAA patients are known to have worse treatment outcomes in pediatric IBD, we are concerned that disparities in endoscopic frequency observed herein may be a contributing factor in ongoing disparate outcomes.


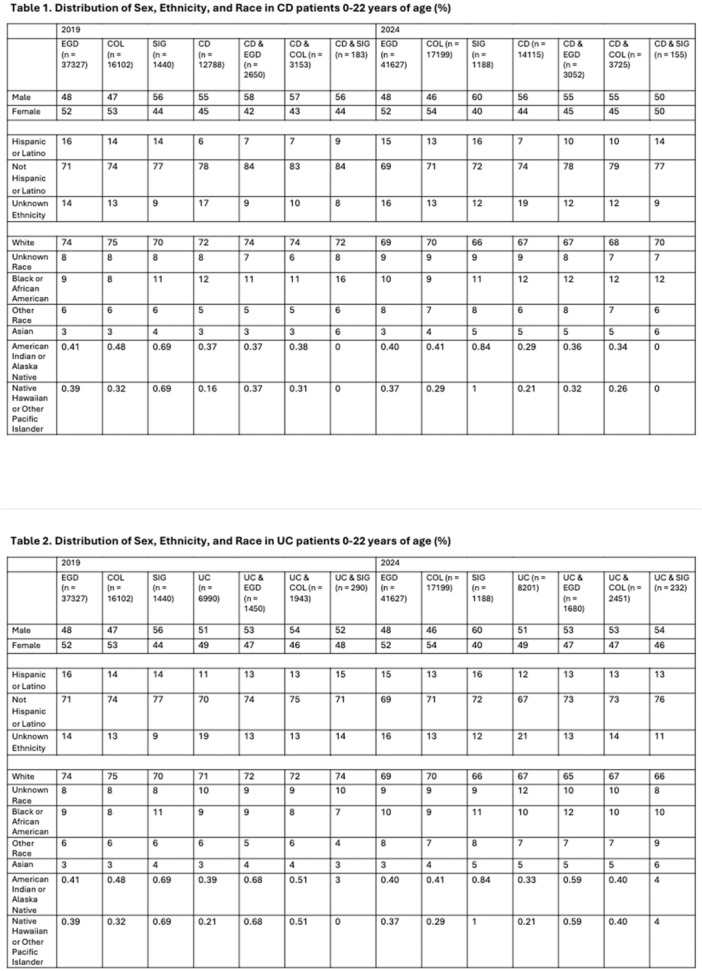


## 637 PROGRESS AND PERSISTENT INEQUITIES IN CHILDHOOD CHRONIC MALNUTRITION IN ECUADOR: A SIX‐YEAR NATIONAL TREND ANALYSIS (2018–2024)

*Camila Gallegos Caicedo* ^*1*^, *Ariel Vargas* ^*2*^, *David Estrella Granda* ^*3*^

^*1*^ *Pediatrics*, *Nicklaus Children's Hospital*, *Miami*, *FL*; ^*2*^ *Pediatrics*, *Cincinnati Children's Hospital Medical Center*, *Cincinnati*, *OH*; ^*3*^ *School of Medicine*, *Universidad San Francisco de Quito*, *Quito*, *Pichincha*, *Ecuador*

**Background and Aims:** Ecuador has one of the highest rates of chronic malnutrition in children under 2 years old in the region. Likely due to limited healthcare access in rural areas, unsafe food and water sources, and rising poverty. This study analyzes the trends in childhood chronic malnutrition over a six‐year period.

**Methods:** We analyzed public access national data from 2018 to 2024 on chronic malnutrition in children under 2 years of age stratified by region, urban vs. rural settings, and ethnicity. Data were obtained from the National Institute of Census and Statistics (INEC), the 2018 National Health and Nutrition Survey (NHHS), and the 2023‐2024 National Survey on Child Malnutrition.

**Results:** In 2018, chronic malnutrition affected 27.2% of children under two years of age, according to the NHHS. By 2023, this rate had declined to 20.1%, based on data from the first round of the National Survey on Child Malnutrition, representing a 7.1 percentage point drop and a 26% relative reduction. The second round of the survey, conducted in 2024, reported a further decline to 19.3%, marking a total reduction of 7.9 percentage points, or approximately 29%, over six years.

Chronic malnutrition remained more prevalent among males, with a male‐to‐female ratio of 3:2. Rural areas showed a higher prevalence (21.9%) compared to urban areas (18.9%) (Figure 1). Indigenous children were the most affected, with a rate of 33.4%, followed by mestizo children at 19.2% (Figure 2). Regionally, the Andean highlands had the highest prevalence (23.9%), followed by the Amazon region (19.6%), while the Coastal and Insular regions reported the lowest figures.

**Conclusions:** This study reveals a 7.9 point decrease in chronic malnutrition among children under 2 in Ecuador over six years, an encouraging outcome that reflects the impact of targeted public health interventions. However, the burden remains disproportionately high in rural areas, among Indigenous populations, and in specific regions. These disparities underscore the urgent need for sustained, equity‐driven policies that expand access to nutrition, clean water, education, and culturally sensitive healthcare.


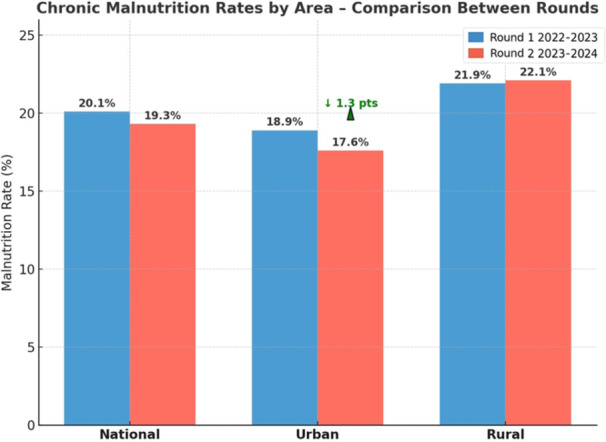


**Figure 1.** Chronic malnutrition rates in urban vs. rural areas across survey rounds.


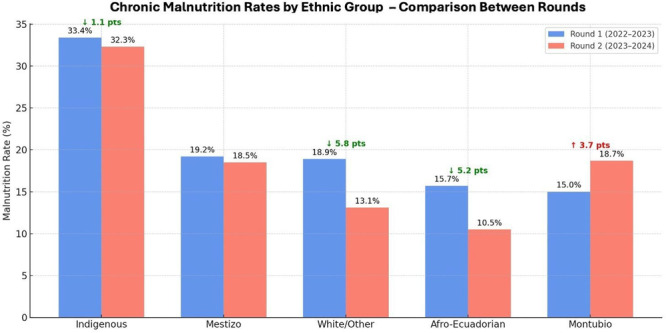


**Figure 2.** Changes in chronic malnutrition rates by ethnic group across survey rounds.

## 638 TITLE: RACIAL DISPARITIES AMONG PEDIATRIC PATIENTS WITH CELIAC DISEASE DEMONSTRATES HIGHER RISK OF TYPE 1 DM IN AFRICAN AMERICAN PEDIATRIC CELIAC PATIENTS: A PROPENSITY SCORE‐MATCHED ANALYSIS USING TRINETX

*Wadan Khan*, *Shahzaib Khan*, *Alaaelden Hassan*, *Ammar al wardi*, *Annie Levine*, *Thomas Wallach*

*SUNY Downstate Health Sciences University*, *New York*, *NY*

**Background:** Celiac disease is an autoimmune condition triggered by the intake of gluten in genetically predisposed individuals. Celiac disease is associated with several comorbidities, including but not limited to Type 1 Diabetes Mellitus (T1DM), anemia, failure to thrive (FTT), depression, osteopenia, autoimmune thyroid and osteoporosis. However, given previous evidence of significant racial disparities in diagnosis and management of gastrointestinal diseases, there is concern there may be variable risks in the celiac disease population. This study primarily examines the prevalence and risk of common celiac comorbidities in black and white pediatric CD patients.

**Methods:** Using the TriNetX Research Network, pediatric patients (ages 0–18 at time of dx) diagnosed with CD (ICD‐10: K90.0) within the past 20 years were identified. Black and African American patients (N = 1,180) were propensity score matched 1:1 with white patients based on age, sex, and encounter type, with a five year window after index event reviewed. The primary outcomes were diagnosis of T1DM. Secondary outcomes included anemia, failure to thrive (FTT), depression, emergency room (ER) visits, osteopenia/osteoporosis, hepatitis, and mortality. Statistical analysis was completed using Kaplan‐Meier for diagnoses, and t‐test comparisons for events.

**Results:** Black and African American pediatric patients with CD exhibited a significantly higher prevalence of T1DM, with 217 cases (18.4%) compared to 103 cases (8.7%) in white patients. The survival probability without T1DM at study end was markedly lower in Black patients (73.95%) than in whites (85.93%), with a highly significant difference (log‐rank χ^2 = ^40.099, p < 0.001). The hazard ratio (HR) for developing T1DM was 2.097 (95% CI: 1.659–2.651. Black patients further had significantly higher rates of anemia (HR = 2.619), failure to thrive (HR = 2.008), depression (HR = 1.394), and emergency room visits (mean 4.43 vs. 2.64; p = 0.004). No statistically significant differences were observed in osteopenia/osteoporosis (10 cases per group; p = 0.366), hepatitis (19 vs. 17 cases; p = 0.959), autoimmune thyroid (55 vs 49 cases; p=0.912 for hyperthyroid and for 43 vs 47 cases; p=0.386 for hypothyroid) or mortality (10 deaths each; p = 0.670) between the cohorts.

**Conclusion:** This study highlights a pronounced racial disparity in T1DM among pediatric patients with celiac disease, with Black and African American children facing more than double the risk compared to white peers. These findings underscore the urgent need for targeted screening and management strategies focused on T1DM in this vulnerable population, while also recognizing broader health disparities in anemia, growth failure, and mental health. It is highly likely that socioeconomic determinants of health may be impacting anemia/FTT/mental health concerns, however variation in T1DM disease suggests a biological difference and need for future exploration.

## 639 ANALYSIS OF CLINICAL OUTCOMES AND HEALTHCARE DELIVERY IN PEDIATRIC ENDOSCOPIC PROCEDURES: A SINGLE CENTER STUDY

*Julie Luna‐Torres* ^*1,2*^, *Kaitlin Olson* ^*2*^, *Lisa DeCamp* ^*2,3*^, *Robert Kramer* ^*1,2*^

^*1*^ *Digestive Health Institute*, *Children's Hospital Colorado*, *Aurora*, *CO*; ^*2*^ *Department of Pediatrics*, *University of Colorado Anschutz Medical Campus*, *Aurora*, *CO*; ^*3*^ *Adult and Child Center for Health Outcomes Research and Delivery Science*, *University of Colorado System*, *Aurora*, *CO*

**Background:** Clinical studies have documented variations across multiple pediatric gastroenterology outcomes associated with race, ethnicity, and preferred healthcare language. Prior studies have identified lower bowel preparation quality scores among pediatric patients with non‐English as preferred language and those with public insurance^1^. There remains a gap in comprehensive assessment of endoscopic quality metrics, particularly terminal ileum (TI) intubation rates, across different patient populations.

**Objectives:** The aim of this study is to assess differences in endoscopic quality metrics and healthcare delivery by race/ethnicity, preferred healthcare language, and patient residence.

**Methods:** We conducted a retrospective cohort study of pediatric patients (<18 years) who underwent endoscopic procedures at our institution from February 2023 to August 2024 using data extracted from Epic Lumens, an endoscopy‐specific module within the Epic electronic health record. Sociodemographic variables included race/ethnicity, preferred healthcare language, and zipcode of patient residence (in Colorado vs. outside of Colorado). Quality and healthcare delivery outcomes assessed were Boston Bowel Preparation Scale scores (categorical, 0‐9), TI intubation rates, adverse events (categorized from parent phone calls requiring reassurance only, to emergency evaluations with discharge home, to complications requiring hospital admission or surgical management), and patient satisfaction ratings from post‐endoscopy surveys (categorical, 1‐5). Statistical analysis used Fisher's Exact Test for categorical variables and Wilcoxon Rank Sum/Kruskal‐Wallis tests for numerical variables.

**Results:** Among 6,198 patients, racial/ethnic distribution was 62.5% White, 25.9% Hispanic/Latino, 4.9% Black/African American, 2.4% Asian, 1.0% American Indian/Alaska Native, and 3.3% Other. Preferred healthcare language was English for 93.8% and Spanish/Other for 6.2%.

In the colonoscopy subset (n=1599), bowel preparation scores showed significant differences between racial/ethnic groups (p=0.018). 12.3% of Non‐Hispanic/Latino Non‐White patients had inadequate bowel preparation scores (≤3) compared to 7.7% of Non‐Hispanic/Latino White patients and 7.5% of Hispanic or Latino patients. TI intubation rates differed by race/ethnicity (p=0.006): 96.0% for Non‐Hispanic/Latino White patients versus 94.7% for Hispanic/Latino and 90.1% for Non‐Hispanic/Latino Non‐White patients. Patients with English as preferred language had higher TI intubation rates than those preferring other languages (95.3% vs. 89.5%, p=0.036). Colonoscopies that reached the TI had higher median bowel prep scores than those that did not (8 vs. 4, p<0.001). In univariate analysis, patients preferring non‐English languages had lower odds of reaching the TI (OR: 0.4; 95% CI: 0.2, 0.9; p=0.021); however, after adjusting for bowel prep score, this relationship was no longer statistically significant (OR: 0.48; 95% CI: 0.21, 1.26; p=0.104).

Adverse events (n=237, 3.8% overall) showed no significant differences by race/ethnicity (p=0.689) or preferred language (p=0.073).

Post‐endoscopy satisfaction survey data (n=960, 15.5% of total) showed no significant differences by race/ethnicity (p=0.169) or preferred language (p=0.688), with over 50% reporting highest satisfaction.

Patients with Colorado zip codes had higher TI intubation rates than those from outside Colorado (95.4% vs. 91.0%, p=0.028).

**Conclusions:** Our study identified significant differences in colonoscopy quality metrics across demographic groups. Non‐Hispanic/Latino Non‐White patients had higher rates of inadequate bowel preparation and lower TI intubation rates. Similarly, patients preferring non‐English languages had lower TI intubation rates in initial analysis, though this effect was largely explained by differences in bowel preparation quality. While adverse events and post‐procedure satisfaction showed no significant differences across demographic groups, the satisfaction survey results should be interpreted cautiously due to selection bias. Geographic factors may also influence outcomes, as shown by differences in TI intubation rates based on patient residence. Implementing targeted quality improvement measures—including tailored pre‐procedure education in multiple languages, culturally appropriate instructions, and addressing logistical challenges for patients from outside the immediate area—could help standardize outcomes across patient populations.

## 640 RARE DISEASE, REAL BARRIERS : LIVER TRANSPLANTATION IN CHILDREN WITH PCNQ

*Marie‐Frédérique Paré*

*Peaditric*, *Centre Hospitalier Universitaire Sainte‐Justine*, *Montreal*, *QC*, *Canada*

**Background:** Progressive cholestasis of Northwestern Quebec (PCNQ) is a rare, autosomal recessive cholestatic liver disease affecting children from the Cree, Anishinabe, and Atikamekw nations in Quebec. Caused by a homozygous R565W mutation in the *CIRH1A* gene, the disease typically presents with transient neonatal cholestasis and progresses to biliary cirrhosis in childhood. To this day, liver transplantation (LT) is the only curative treatment. Despite its severe clinical course, PCNQ remains poorly understood due to its rarity and phenotypic variability.

**Objective:** To describe the complete global cohort of patients (n = 17) who underwent liver transplantation for PCNQ, and to assess whether their post‐transplant outcomes are comparable to those of patients transplanted for other pediatric liver diseases. We also aimed to examine the role of social determinants of health in shaping disease progression, access to care, and transplant outcomes in this geographically and socially marginalized population.

**Methods:** We conducted a retrospective chart review of 17 patients with genetically or histopathologically confirmed PCNQ who received liver transplants at CHU Sainte‐Justine between the 1970s and 2023. These 17 patients represent the entirety of known PCNQ cases worldwide who have undergone transplantation. Clinical, laboratory, transplant‐related, and sociodemographic data were extracted from medical records. Descriptive analyses explored the relationship between social disadvantage and clinical outcomes.

**Results:** All patients originated from remote communities in Northwestern Quebec. Median age at diagnosis and transplantation varied widely, reflecting differences in disease progression and access to specialized care. Post‐transplant outcomes, including survival and graft function, were generally comparable to those of children transplanted for other indications. However, socioeconomic disadvantage, geographic isolation, and family structure appeared to influence time to diagnosis and timing of referral for transplantation.

## 641 IMPROVING ACCESS TO PEDIATRIC GASTROENTEROLOGY FOR RURAL COMMUNITIES UTILIZING A PRIMARY CARE HYBRID CLINIC MODEL

*Claire Tuquero* ^*2*^, *Mihir Palan* ^*3*^, *Henry Lin* ^*1*^, *Ariel Porto* ^*1*^

^*1*^ *Division of Pediatric Gastroenterology*, *Oregon Health & Science University Doernbecher Children's Hospital*, *Portland*, *OR*; ^*2*^ *School of Nursing*, *Simmons University*, *Boston*, *MA*; ^*3*^ *Oregon Health & Science University School of Medicine*, *Portland*, *OR*

**Background:** Advances in pediatric healthcare have led to more children living with chronic health conditions, increasing the demand for multidisciplinary care involving pediatric subspecialists. Access to pediatric gastroenterology remains limited in many rural regions of the United States. In 2019, over 5 million children lived more than 80 miles from a pediatric gastroenterologist. Despite improvements in national subspecialist density to 2.4 per 100,000 children in 2023, these providers remain clustered in urban areas. In Oregon, 17 of the state's 19 board‐certified pediatric gastroenterologists practice in a single urban county, leaving many rural areas underserved. These geographic disparities are associated with poorer health outcomes, longer wait times, and greater barriers to care.

At our institution, a hybrid telehealth model has been piloted for children with metabolic dysfunction‐associated steatotic liver disease (MASLD). The model includes scheduled clinic appointments in local practices, where patients are seen in person by a pediatric cardiologist while a pediatric gastroenterologist joins the visit virtually to provide subspecialty consultation during the same visit.

**Objective:** The objective of this study is to assess rural PCP and pediatric GI perspectives on the hybrid care model as an approach to improve access to subspecialty care, identify barriers and facilitators to implementation, and explore its potential for broader applications in pediatric gastroenterology care.

**Methods:** Rural pediatric primary care clinics were identified using a state directory. A total of 17 pediatric PCPs participated in semi‐structured interviews to discuss referral challenges, subspecialty access, and impressions of the hybrid model. Additional interviews were conducted with the MASLD hybrid program clinical team. Responses were analyzed for common themes and summarized descriptively.

**Results:** Among the 17 PCPs interviewed, 15 (88%) expressed interest in implementing the hybrid model at their clinic. The two providers who did not show interest referred fewer than 4 patients to pediatric GI per year. Across the cohort, 15 providers (88%) reported feeling comfortable conducting in‐person physical exams with guidance from a remote pediatric gastroenterologist. Those who did not feel comfortable expressed reasons including having majority adult patients in their family practice as well as being unfamiliar with the important aspects of a pediatric gastroenterology physical examination. Interviews with subspecialists currently involved in the MASLD hybrid clinic emphasized the model's value in reducing patient burden, improving collaboration, aiding diagnostic clarity, and facilitating more efficient triage of complex cases. Limitations identified in interviews include that the hybrid model does not yet resolve long wait times for patient's to be evaluated by a subspecialist and that many patients are uncomfortable navigating technology for virtual follow‐up communication and appointments.

**Discussion:** As the demand for pediatric GI specialists increases, novel strategies are needed to help lower the barriers to care for both patients and referring PCPs, especially for those in low access areas and those who frequently refer to pediatric GI specialists. Rural PCPs in Oregon expressed strong interest in the hybrid model, which addresses several major barriers to subspecialty care including geographic distance, limited access, and lack of direct collaboration opportunities with distant specialists. These perceived benefits are reflected in the feedback from the providers participating in the MASLD hybrid clinic. However, logistical challenges to consider based on the MASLD clinic experience include scheduling complexity and technology literacy for families to receive follow‐up care with remote subspecialists. In conclusion, this survey of rural PCPs supports expanding the model beyond MASLD to other common GI conditions. Future efforts should focus on pilot implementation, workflow refinement, and outcome evaluation to scale this model across additional rural communities.

## 642 EVALUATING THE IMPACT OF MECONIUM ILEUS ON DIAGNOSIS AND CLINICAL TRAJECTORIES IN PEOPLE WITH CYSTIC FIBROSIS

*Alexandra Pottorff* ^*1,4*^, *Savannah Knight* ^*2,3*^, *MinJae Lee* ^*1*^, *Fadel Ruiz* ^*2,3*^, *Donna Beth Willey‐Courand* ^*5*^, *Meghana Sathe* ^*1,4*^

^*1*^ *The University of Texas Southwestern Medical Center*, *Dallas*, *TX*; ^*2*^ *Pediatric Pulmonology*, *Baylor College of Medicine*, *Houston*, *TX*; ^*3*^ *Texas Children's Hospital*, *Houston*, *TX*; ^*4*^ *Pediatric Gastroenterology*, *Children's Medical Center Dallas*, *Dallas*, *TX*; ^*5*^ *The University of Texas Health Science Center at San Antonio*, *San Antonio*, *TX*

**Background:** Meconium ileus (MI) is the initial presentation of Cystic Fibrosis (CF) in up to 20% of People with CF (PwCF)[1]. MI in PwCF has been associated with reduced growth and increased rates of *Pseudomonas aeruginosa* (PsA) infections. Recognition of MI and early diagnosis of CF is important. Hospitals with less exposure to MI and PwCF may have difficulty recognizing MI as a clinical sign of CF. Prior studies have found that Immunoreactive Trypsinogen (IRT) is less sensitive for screening for CF in PwCF with MI, and the newborn screen (NBS) may miss PwCF with MI[2]. Recognition of MI prenatally or soon after birth and early involvement with a CF center could provide the opportunity for engagement with the state NBS program to obtain earlier genetic testing regardless of IRT. This project is a retrospective review to assess the timeliness of diagnosis of CF in PwCF and MI compared to those without MI and evaluate for disparities in CF related outcomes.

**Methods:** Retrospective review of PwCF cared for at Dallas Children's and Texas Children's Hospital CF centers from 1/1/2010‐ 5/23/2016 was performed. Data was collected regarding NBS results, genetic testing, and timeliness to first CF event, sweat test, and diagnosis. Anthropometric outcomes, hospitalizations, and PsA growth for the first 5 years of life were collected. We performed univariable comparisons of outcomes by PwCF with MI versus those without using Chi‐square test for categorical variables and ANOVA (or non‐parametric Kruskal‐Wallis test) for continuous variables.

**Results:** PwCF with MI had a higher prevalence of premature birth and NICU stay compared to those without MI (Table 1). There was not a statistically significant difference in prevalence of normal IRT based on MI status. PwCF with MI had a significantly higher prevalence (40%) of state genetic testing performed from first NBS. Only 1/7 non‐White/non‐Hispanic PwCF with MI had genetic testing sent with first NBS, compared to 5/17 White non‐Hispanic PwCF with MI. PwCF and MI were found to have a longer median time to first sweat chloride test but had an earlier time to first CF event and final diagnosis. PwCF with MI had significantly lower growth parameter z‐scores at 3 months. Differences persisted even at 5 years but were not statistically significant. Prevalence of gastrostomy tube (GT), hospitalization frequency, and rates of growth of PsA were higher in PwCF and MI.

**Conclusion:** Collaboration between birth hospitals, CF centers, and the state NBS program to diagnose CF in infants born with MI is evidenced by earlier state genetic testing, time to first CF event, and age of diagnosis in PwCF with MI. However, 60% of PwCF with MI did not undergo earlier testing and disparities based on race/ethnicity were found, suggesting aims for future improvements. This study supports that PwCF with MI have significant morbidities compared to those without MI. Next steps will be to perform review from 5/24/2016‐1/1/2023.

**References:**

[1] Tan SMJ, Coffey MJ, Ooi CY. Differences in clinical outcomes of paediatric cystic fibrosis patients with and without meconium ileus. Journal of Cystic Fibrosis. 2019;18:857‐62.

[2] Rusakow LS, Abman SH, … & Accurso FJ. Immunoreactive trypsinogen levels in infants with cystic fibrosis complicated by meconium ileus. Screening. 1993;2:13‐17.


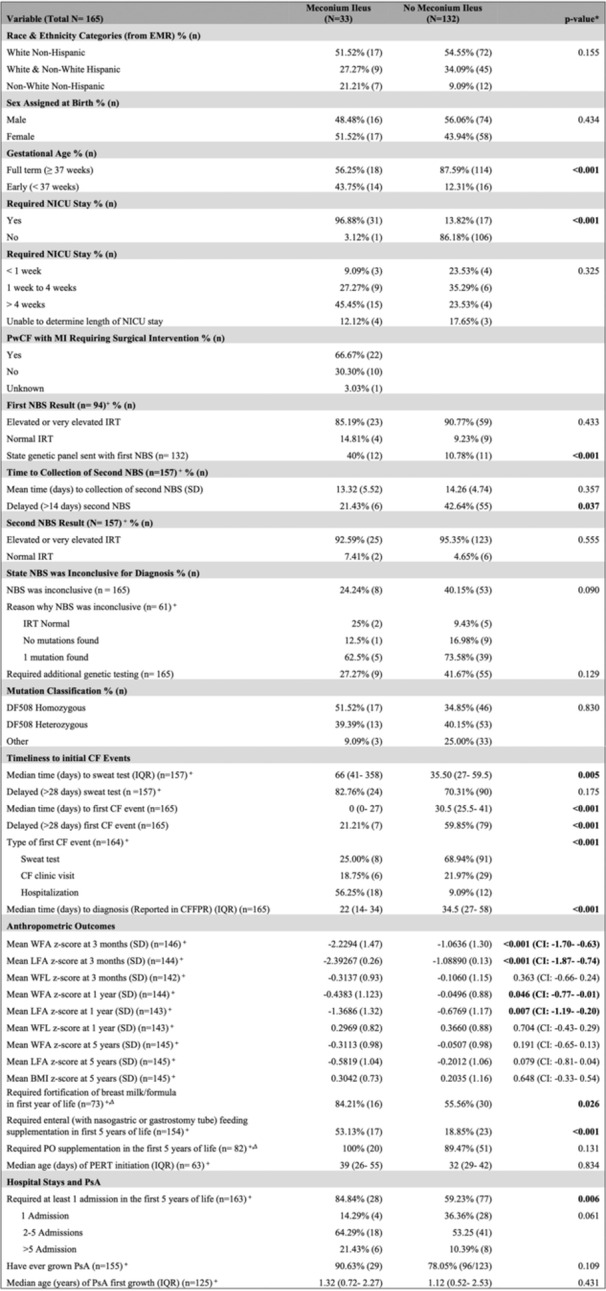


**Table 1. Demographics, timeliness to CF events, anthropometric outcomes, and hospital stays for PwCF with MI and without MI.:** IQR= Interquartile Range. SD = Standard Deviation. CFFPR= CF Foundation Patient Registry. WFA = Weight for Age. LFA = Length for Age. WFL = Weight

for Length. BMI = Body Mass Index.

*Chi‐square test for categorical variables, ANOVA or its non‐parametric counterpart Kruskal‐Wallis test for continuous variables.

+ Total sample size was 165, if specific sample size (n) is not listed n=165. If sample size was different (e.g. missing values, specific variable) it is noted.

△Data only collected at Dallas Children's CF Center.

## 644 PSYCHOSOCIAL DETERMINANTS OF HEALTH IN PEDIATRIC LIVER TRANSPLANT CANDIDATES: A SINGLE CENTER QUALITATIVE ANALYSIS FROM THE PATIENT'S PERSPECTIVE

*Shruti Sakhuja* ^*1,2*^, *Deborah Thompson* ^*3*^, *Salma Musaad* ^*3*^, *John Goss* ^*3*^, *Krupa Mysore* ^*1*^, *Nhu Galvan* ^*3*^

^*1*^ *Gastroenterology, Hepatology, and Nutrition*, *Texas Children's Hospital*, *Houston*, *TX*; ^*2*^ *The University of Chicago Division of the Biological Sciences*, *Chicago*, *IL*; ^*3*^ *Baylor College of Medicine*, *Houston*, *TX*

Racial and socioeconomic disparities in liver transplantation (LT) are well documented. These disparities are compounded by psychosocial factors including family dynamics and neuropsychological functioning, which are crucial to adherence to medical protocols. Despite this, there is currently no standardized pre‐transplant psychosocial assessment tool for pediatric LT patients. This study aims to develop a tool, grounded in the patient experience, to identify psychosocial challenges facing this population.

We used qualitative interviewing methods to understand the lived experiences of LT patients. Data was gathered through virtual interviews and focus groups until data saturation was achieved. Thematic analysis of the data was performed using NVivo software, and the extracted themes were used to develop an item pool for our tool.

Twenty‐three LT patients and parents of patients who had undergone LT participated in the study. Thematic analysis highlighted major areas of focus. Key findings included the importance of social support as well as the challenges associated with financial burden, transportation, and medication side effects. Adolescents additionally reported difficulties with medication adherence, social reintegration, and self‐esteem due to physical changes.

This study highlights the multifaceted psychosocial challenges faced by pediatric LT patients and their families, and emphasizes the need for a comprehensive approach to LT care. Development of the tool is a step toward standardizing the psychosocial assessment of pediatric LT candidates, thereby identifying high‐risk patients and tailoring interventions accordingly. Limitations include the small, single‐center sample and potential reporting bias. Future studies may pilot the tool to further assess its impact on patient outcomes.


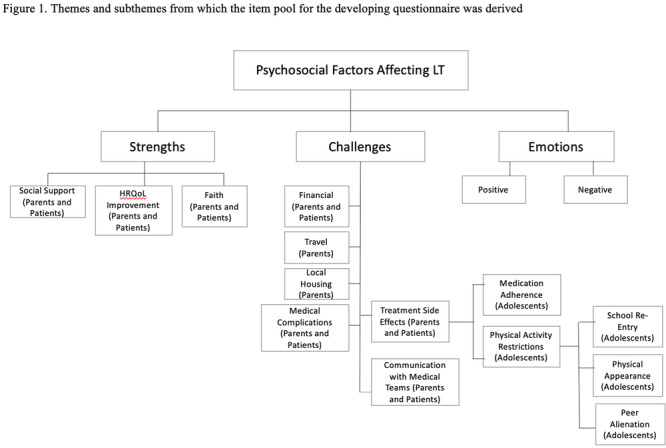


Figure 1. Themes and subthemes from which the item pool for the developing questionnaire was derived.


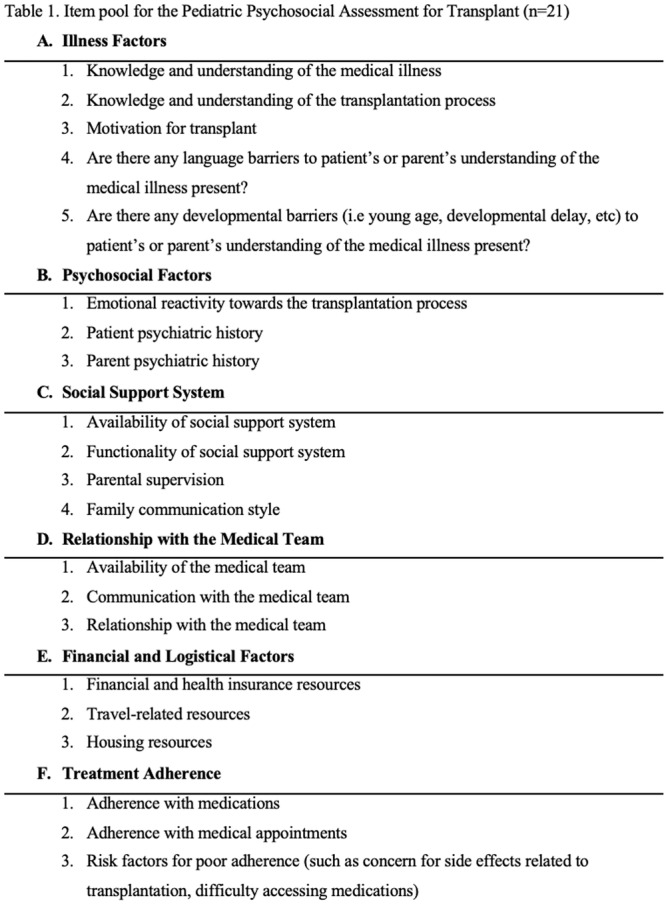


Table 1. Item pool for the Pediatric Psychosocial Assessment for Transplant

## 645 EVALUATING THE IMPACT OF AN AERODIGESTIVE CLINIC ON AT‐RISK NEIGHBORHOODS USING THE CHILDHOOD OPPORTUNITY INDEX: A RETROSPECTIVE ANALYSIS

*Natalia Youssef* ^*1*^, *Julie Khlevner* ^*2*^

^*1*^ *Albany Medical College*, *Albany*, *NY*; ^*2*^ *Pediatric Gastroenterology, Hepatology and Nutrition*, *Columbia University*, *New York*, *NY*

**Introduction:** The Pediatric Aerodigestive Clinic (ADC) at Columbia University Irving Medical Center manages patients with complex gastrointestinal/respiratory conditions and is located in Washington Heights, an area with significant linguistic barriers and low adult education levels. There is limited data on the impact of an ADC in such neighborhoods. To quantify barriers faced by pediatric patients, the Childhood Opportunity Index (COI) compiles education, health/environment, and social/economic data from U.S. neighborhoods and categorizes zip codes into five levels: "very low" (VL), "low" (L), "moderate" (M), "high" (H), and "very high" (VH). We sought to examine whether social determinants of health, as determined by COI levels, impact clinical outcomes in patients referred to the ADC from January 2022 to December 2023.

**Methods:** A retrospective chart review was conducted on 144 patients seen at the ADC during the study period, with 27 meeting inclusion criteria of relevant medical records at least one year before and after initial ADC evaluation. COI levels were combined into two cohorts: “Low” (VL +L) and “High” (M+H+VH). Clinical outcomes were examined one year before and after the initial ADC visit, as indicated by the number of emergency room (ER) visits, hospitalizations, and Δweight‐for‐age percentile (WAP) Z‐score.

**Results:** The “High” cohort showed improvement one year post‐initial ADC visit in the number of ER visits (‐38.7%), hospitalizations (‐45.9%), and mean ΔWAP Z‐score (+966.7%) compared to one year pre‐initial ADC visit. Conversely, the “Low” cohort experienced a reduction in hospitalizations (‐33.7%) but saw an increase in ER visits (+20%) and a decrease in mean ΔWAP Z‐score (‐66.7%) one year post‐initial ADC visit. A trend suggests that a higher COI level may lead to better clinical outcomes in a cohort of patients referred to ADC.

**Discussion:** This study highlights the importance of interdisciplinary ADC teams recognizing that patients from lower COI levels may derive comparatively fewer benefits from ADC interventions than those from higher COI levels. Identifying resources to address specific barriers contributing to outcome disparities in low COI neighborhoods is crucial for ADC teams.

Further research with a larger cohort size is needed to increase the power of the study and draw statistically significant conclusions to support these trends. Collaborating with other ADC clinics nationwide in similar neighborhoods may help achieve this.


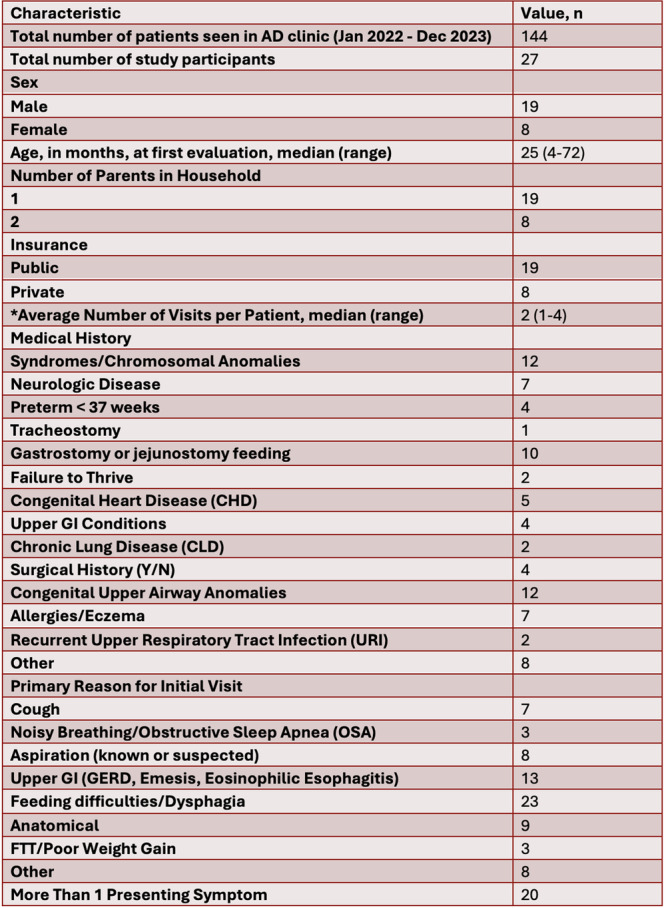


Table 1. Demographics of Study Participants

*During study period from January 2022 ‐ December 2023.


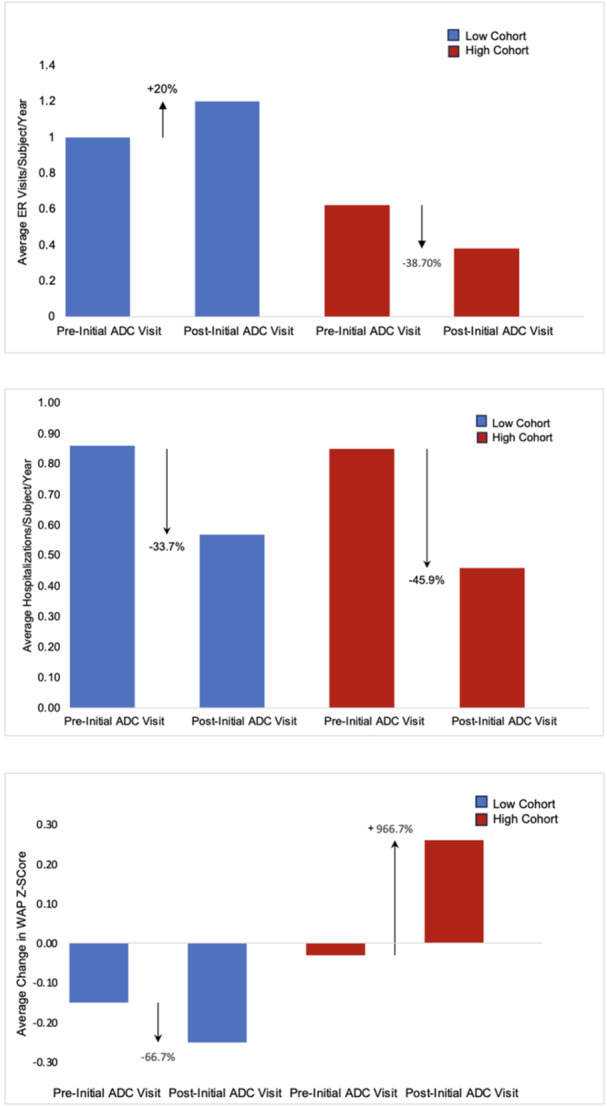


Figure 1: (a) Percentile Change in Average ER visits/subject/year by COI Level (1 Year Pre‐ vs. Post‐Initial ADC Visit). (b) Percentile Change in Average Hospitalization visits/subject/year by COI Level (1 Year Pre‐ vs. Post‐Initial ADC Visit). (c) Percentile Change in Average △WAP Z‐Scores by COI Level (1 Year Pre‐ vs. Post‐Initial ADC Visit).

## 647 FEEDING OUTCOMES IN NICU GRADUATES WITH HOME NASOGASTRIC OR GASTROSTOMY TUBES: A RETROSPECTIVE COHORT STUDY

*Sophie Halpern* ^*1*^, *Vibha Sood* ^*2*^, *Feras Alissa* ^*2*^, *Abeer Azzuqa* ^*3*^, *Arcangela Balest* ^*3*^

^*1*^ *Pediatrics Residency Program*, *UPMC Children's Hospital of Pittsburgh*, *Pittsburgh*, *PA*; ^*2*^ *Division of Pediatric Gastroenterology, Hepatology and Nutrition*, *UPMC Children's Hospital of Pittsburgh*, *Pittsburgh*, *PA*; ^*3*^ *Division of Newborn Medicine*, *UPMC Children's Hospital of Pittsburgh*, *Pittsburgh*, *PA*

**Background:** Studies and clinical observations suggest that home nasogastric (NG) tube feeding programs may be a beneficial alternative to gastrostomy tube (GT) feedings, supporting a successful transition to full oral (PO) feedings in NICU graduates with ongoing feeding difficulties.

**Purpose:** This study aimed to investigate the success rates of oral feeding in patients discharged from a tertiary care NICU using home NG tube or GT feeds. Secondary outcomes included the rates of patients requiring continuous feeds, the number of visits to the feeding team, gastroenterology‐related visits, and the number of ancillary medications.

**Methods:** This was a retrospective cohort study that followed a pilot sample of 30 NICU graduates from a single center for outcomes at 12 months post‐discharge. Statistical analysis included paired t‐test or Mann‐Whitney U tests for quantitative variables and Chi‐squared analysis or Fisher's Exact test of categorical variables. Logistic regression was used to identify possible confounding variables such as comorbidities or demographic data.

**Results:** 100% of patients discharged with a home NG tube, and 20% of patients discharged with a G‐tube achieved total oral feeding success within 12 months of NICU discharge. 0% of the patients in the NG tube group compared to 100% in the G‐tube group required continuous feeds during the 12 months following NICU discharge. The documented percentage of oral intake at the time of NICU discharge and concerns for aspiration were different between the two groups. This disparity may represent confounding variables that could impact their outcomes.

**Conclusion:** Patients with home NG tubes have a higher success rate of oral feedings within 12 months of NICU discharge than patients with a G‐tube, although many of their predisposing comorbidities and demographic factors may not be significantly different.

## 650 INPATIENT FEEDING PROGRAM FOR A VULNERABLE, UNDERSERVED, PEDIATRIC POPULATION: A PILOT STUDY

*Shahzaib Khan*, *David Inzerillo*, *Annie Levine*, *Thomas Wallach*, *Simon Rabinowitz*

*Pediatric Gastroenterology*, *SUNY Downstate Health Sciences University*, *New York*, *NY*

**Background:** Pediatric feeding disorders (PFD) encompass a variety of conditions that interfere with a child's ability to consume a developmentally appropriate diet essential for growth. These disorders, often found in children with simultaneous behavioral and medical problems, lead to nutritional deficiencies, social/behavioral delays and poor physical growth. Unfortunately, services such as intensive, multidisciplinary feeding programs have traditionally been limited for underserved populations. Our study describes a pilot multidisciplinary program highlighted by a comprehensive inpatient stay in an inner‐city university medical center.

**Methods:** We describe 14 children between ages 8 months to 9 years with a diagnosis of failure to thrive or avoidant restrictive food intake disorder (ARFID) who underwent 1‐week intensive in‐patient feeding therapy. All patients were evaluated prior to admission by a pediatric gastroenterologist and had baseline laboratory testing (CBC, CMP, CRP, ESR, Vitamin D 25OH, iron, B12 and folate levels) as part of the baseline evaluation. Intensive feeding therapy sessions were conducted twice a day with a trained pediatric speech language pathologist (SLP) who assessed barriers to oral feeding. A variety of feedings problems were initially identified followed by sensory and behavioral techniques to improve acceptance of age‐appropriate foods. Specific techniques employed included desensitization, texture fading, portion fading, demand fading, allowing choices and providing preferred foods. Primary outcomes recorded were number and type of new foods accepted, consistency and characteristics of new foods accepted, increased intake during mealtimes, strategies that were effective in individual patients as well as the efficacy of caregivers to replicate successful strategies. Patients were also monitored for changes in weight, total caloric intake, and the caregiver's quantitative ability to replicate the SLP's success. If indicated, a Behavioral and Development consultation assessed current developmental needs to optimize service provision.

**Results:** A total of 14 patients (10 males) participated in our program; 5 had ARFID, 4 had FTT and 5 had both. 8 out of the 9 (88%) patients with FTT gained weight during the in‐patient admission week with an average weight gain of 560 g (range 200g‐1400g). 13 out of 14 patients had an increase in the number of foods they accepted and had successful carry‐over of feeding strategies to caregiver. Within our cohort, a total of 60 new foods and 17 new textures were successfully introduced and tolerated. After discharge, 11 patients were transitioned to an out‐patient feeding therapy program. 5 patients were referred for further consultation to neurology and behavioral and developmental specialists.

**Conclusions:** Our condensed, one week‐long feeding program was able to successfully demonstrate weight gain in a high proportion of children with PFD and FTT, as well as an increased acceptance of a variety of foods with carry‐over of feeding strategies to caregivers.


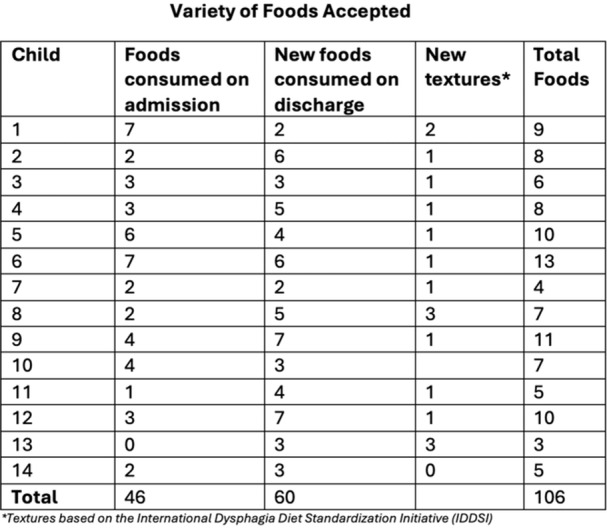


Table 1: Variety of Foods Accepted

## 652 REDUCING RATE OF FEEDS IS MORE EFFECTIVE THAN THICKENING THEM IN EXTREMELY PREMATURE INFANTS WITH ASPIRATION

*Cristina Soto* ^*1*^, *Abeer Abdulaziz Al‐Raddadi* ^*2*^, *Demiana Azmy* ^*2*^, *Sarah Mauraase* ^*3*^, *Aimee Seiderer* ^*4*^, *laura chen* ^*4*^, *Arik Alper* ^*2*^

^*1*^ *Pediatrics*, *Yale University*, *New Haven*, *CT*; ^*2*^ *Gastroenterology, Hepatology, and Nutrition*, *Yale University*, *New Haven*, *CT*; ^*3*^ *ENT*, *Yale University*, *New Haven*, *CT*; ^*4*^ *Yale New Haven Hospital*, *New Haven*, *CT*

**Introduction:** Oropharyngeal aspiration is defined as the involuntary entry of food, liquid, saliva, or gastric contents into the airway during the act of swallowing. This problem is prevalent among extremely premature infants, particularly those delivered before 28 weeks of gestation, attributed to their underdeveloped neuromuscular and respiratory systems. The modified barium swallow (MBS) study serves as the gold standard diagnostic tool for this condition. Treatment approaches may involve modifying feeding rates and viscosity of oral feeds or initiating non‐oral feeding methods.

There is a notable lack of literature on oropharyngeal aspiration in extremely premature infants, creating a critical gap in understanding and managing feeding difficulties in this vulnerable population. We aim to share our experience regarding this problem, assess the success rate of current treatment modalities, and identify risk factors for abnormal swallow evaluation in this cohort.

**Methods:** We performed a retrospective chart review of children aged 21 years and younger who were assessed at the aero‐digestive clinic in Yale New Haven Children's Hospital from 2013 to 2021 due to concerns for oropharyngeal aspiration. Extreme prematurity was identified when the gestational age was less than 28 weeks. We conducted a review of medical records and collected demographic, clinical, and radiological data. For the purpose of statistical analysis, we employed the independent t‐test and chi‐square test.

**Results:** The study included 484 patients, of whom 77 (16%) presented with extreme prematurity (≤ 28 weeks gestational age) out of which 49 (10%) had MBS within their first year of life due to clinical concerns regarding aspiration. From these patients, 80% had an abnormal swallow study. Infants with an abnormal swallow study demonstrated a significantly lower mean gestational age in comparison to those with a normal evaluation (25 ± 1.5 versus 26.1 ± 1.4, p = 0.02; see Table). No additional risk factors were identified. The majority of abnormal studies showed improvement when efforts were made to decrease the flow rate, as opposed to thickening feeds (46% versus 18%, respectively).

**Discussion:** Oropharyngeal aspiration represents a significant concern for infants born extremely prematurely. This study underscores gestational age as the sole risk factor for abnormal swallow evaluation, rather than the age of the infant at the time of study. This finding may facilitate improved utilization of MBS studies, considering the associated radiation risk. Furthermore, reducing the flow rate during the evaluation proved to be more effective than thickening in normalizing swallow function. In light of the associated risks of commercial thickeners, this study emphasizes the importance of flow reduction as the initial step in managing oropharyngeal aspiration. These findings may contribute to the development of guidelines for the evaluation and management of dysphagia in preterm infants.


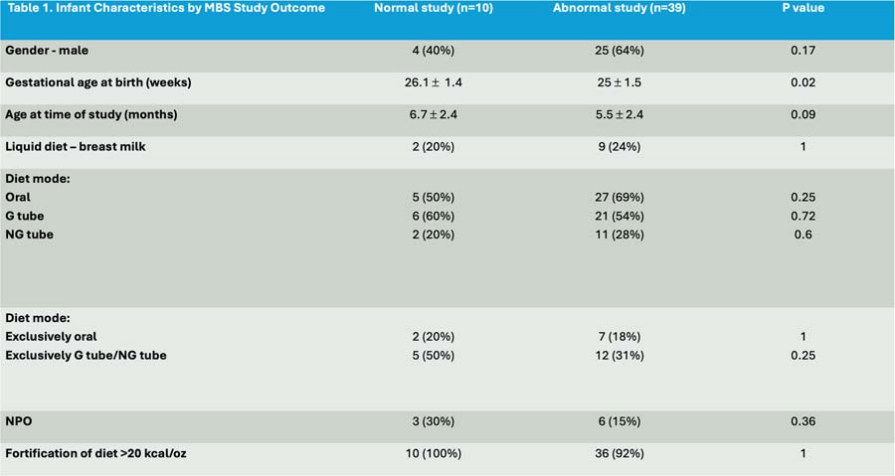


## 653 REFERRAL PATTERNS AND PATIENT CHARACTERISTICS IN PEDIATRIC FEEDING DISORDER CARE AT A MULTIDISCIPLINARY OUTPATIENT CLINIC

*Edward Tannenbaum* ^*1*^, *Riad Rahhal* ^*2*^, *Willow Schanz* ^*1*^, *Adam Vaske* ^*1*^, *Linder Wendt* ^*3*^, *Dina Al‐Zubeidi* ^*2*^

^*1*^ *The University of Iowa Roy J and Lucille A Carver College of Medicine*, *Iowa City*, *IA*; ^*2*^ *Pediatrics‐Gastroenterology*, *University of Iowa Health Care*, *Iowa City*, *IA*; ^*3*^ *Institute for Clinical and Translational Science*, *University of Iowa Health Care*, *Iowa City*, *IA*

**Objectives:** Pediatric feeding disorders are increasingly prevalent in the United States. A multidisciplinary approach has been shown to effectively address the nutritional, feeding skill, medical, and psychosocial needs of these patients. However, limited research has examined referral characteristics for outpatient feeding programs. Understanding these patterns can help optimize resource allocation and improve patient care. This study aims to analyze patient referral characteristics based on referral patterns, patient demographics, and treatment interventions.

**Methods:** A retrospective review was conducted of pediatric patients (ages 6 months to 17 years) evaluated in a multidisciplinary outpatient feeding clinic at an academic institution from 2021 to 2024. Data collected from chart reviews include demographics, height, weight, BMI, diagnoses, tube feeding use, appetite‐stimulating medication use, referral reasons, referral sources, and endoscopy procedure details.

**Results:** Primary care referrals were the most common referral source to our pediatric feeding program. Specialist referrals were associated with patients with a higher rate of comorbidities and the use of tube feeding compared to primary care referrals. Oral aversion was common when feeding tube was present regardless of the type but was less in the nasogastric tube group.

**Conclusions:** Few studies have examined referral patterns and patient characteristics in U.S. outpatient feeding clinics. This study will address a critical knowledge gap and provide insights into how referral patterns impact patient characteristics and outcomes. Findings will help inform future resource allocation, particularly given the limited availability of feeding specialists and multidisciplinary feeding clinics.

## 656 CAPSULE ENDOSCOPY: A NEW HORIZON IN THE DIAGNOSIS OF GASTROINTESTINAL GRAFT‐VERSUS‐HOST DISEASE

*María del Rosario Alvarado Cifuentes*, *Ericka Montijo‐Barrios*, *Karen Ignorosa‐Arellano*, *Dr. José Francisco Cadena León*, *Flora Zarate‐Mondragon*, *Roberto Cervantes Bustamante*, *Jaime Ramirez‐Mayans*, *Erick Toro‐Monjaraz*, *Martha Martínez Soto*

*Gastroenterology*, *Instituto Nacional de Pediatria*, *Mexico City*, *CDMX*, *Mexico*

**Background:** Gastrointestinal graft‐versus‐host disease (GI‐GVHD) is a severe complication of allogeneic hematopoietic cell transplantation (allo‐HCT), associated with high morbidity and mortality. Conventional endoscopy with biopsy carries risks in critically ill patients, while video capsule endoscopy (VCE) offers a non‐invasive alternative for small bowel evaluation. Limited data exist on VCE use in adults, and none in pediatrics.

**Objective:** To compare findings of VCE, upper and lower endoscopy, and histopathology in pediatric patients with suspected GI‐GVHD.

**Methods:** A case series of four pediatric patients with suspected GI‐GVHD was conducted. Upper and lower endoscopy findings were compared with VCE results and histopathology.

**Results:** Four male patients with combined immunodeficiency or leukemia underwent allo‐HCT. Upper endoscopy showed mild findings (non‐erosive pangastritis, mucosal edema), and lower endoscopy revealed edema and erythema, inconclusive for GVHD. VCE demonstrated severe lesions in three patients (deep ulcers, sloughed mucosa); one patient had normal findings. Histopathology confirmed GVHD in the three patients with abnormal VCE and was normal in the fourth.

**Conclusions:** VCE identified significant small bowel lesions not detected by conventional endoscopy, correlating strongly with histopathology. VCE may enable earlier GI‐GVHD diagnosis in pediatric patients, improving management where invasive procedures pose high risk.


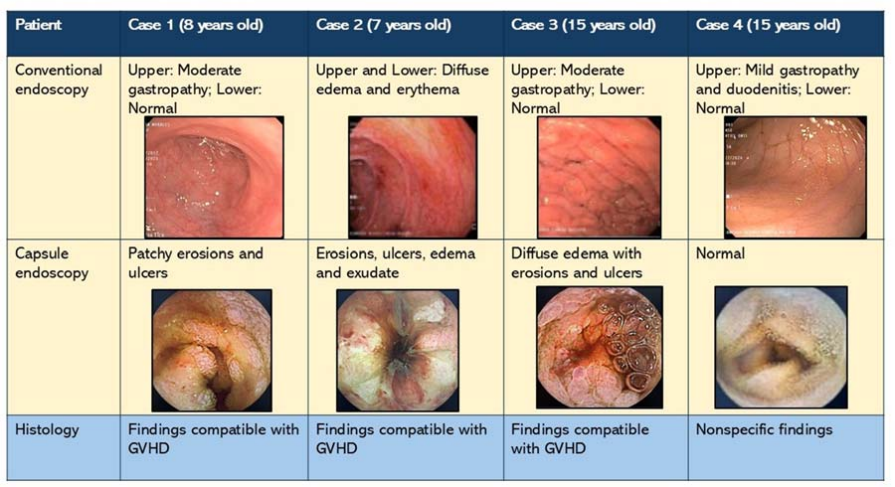


## 659 PRACTICE PATTERNS AND CLINICAL FINDINGS IN INFANT ESOPHAGOGASTRODUODENOSCOPY

*Tyler Babinski* ^*1*^, *Zoe Rosoff‐verbit* ^*1*^, *Michael Manfredi* ^*1*^, *Matthew Ryan* ^*1*^, *Jonathan Berken* ^*1,2*^

^*1*^ *Gastroenterology, Hepatology and Nutrition*, *The Children's Hospital of Philadelphia*, *Philadelphia*, *PA*; ^*2*^ *Neonatology*, *The Children's Hospital of Philadelphia*, *Philadelphia*, *PA*

**Background:** The role of esophagogastroduodenoscopy (EGD) in infants is evolving with emerging diagnostic and therapeutic applications. This study aims to fill the current gap surrounding the usage of EGD in infants, where current data are sparse and primarily derived from single‐center reports. Recognizing the limitations of single‐center experiences, there exists an opportunity to characterize national practice patterns, therapeutic approaches, and endoscopic findings to inform clinical decision‐making and future research.

**Objective :** To characterize national patterns in indications, pre‐procedural pharmacologic management, and endoscopic findings in infants undergoing EGD using a multicenter endoscopic database.

**Methods:** We performed a multicenter retrospective cohort study using version 4 of the NIDDK Clinical Outcomes Research Initiative National Endoscopic Database (CORI‐NED), encompassing procedures performed between 2010 and 2014. This dataset is the most up‐to‐date multicenter resource available. We identified infants under 12 months of age who underwent an EGD, capturing a total of 968 procedures across four centers: an academic medical center in Texas (n = 106) and community hospitals in Arizona (n = 685), Virginia (n = 145), and Maine (n = 32). The Maine site discontinued participation in 2012. All data were de‐identified and thus exempt from institutional review board approval. Due to variability in documentation, not all data elements were available for certain cases.

**Results:** Among the 743 procedures with available setting data, 69.7% (n = 518) were performed in outpatient endoscopy suites, while 17.9% (n = 133) occurred in inpatient settings. The most frequently documented indications included feeding difficulties and gastroesophageal reflux as shown in Table 1.

Acid suppression therapy was documented in 30.1% of cases (n = 291/966). Proton pump inhibitors were recorded in 218 cases, with lansoprazole being the most common agent (69.7%; n = 152/218; X^2^ = 246.0, *p* < 0.05). H_2_ receptor antagonists were recorded in 98 cases, primarily ranitidine, used in 88.8% of those cases (n = 87/98; X^2^ = 135.51, *p* < 0.05). Bethanechol was documented in 3.31% of procedures (n = 32/966).

Endoscopic findings were reported in 17.6% of procedures (n = 170), comprising 90 esophageal and 80 gastric/duodenal evaluations. Among the esophageal evaluations, linear furrowing and mucosal congestion suggestive of eosinophilic esophagitis, white plaques suspicious for *Candida* esophagitis, and esophagitis characterized by swollen, congested, friable mucosa, and adherent whitish patches were present in 24.4% of cases (n = 28). Structural abnormalities observed included patulous lower esophageal sphincter, diffuse submucosal hemorrhages, and sliding hiatal hernia were seen in 16.6% of cases (n = 15). Discrete reflux, edema, erosions, and erythema were noted in the mid and distal esophagus, sometimes related to mechanical irritation from foreign bodies were noted in 35.5% (n = 32) of cases. In the gastric antrum, body, and duodenum at least one abnormality: erythema, friable mucosa, edema, hemorrhage, or nodularity was observed in 97.5% of cases (n = 78).

**Conclusion:** EGD remains a key diagnostic modality for evaluating upper gastrointestinal symptoms in infants. This multicenter analysis highlights significant variability in indications, pre‐procedural pharmacologic management, and endoscopic findings across diverse clinical settings. However, the study is limited by the retrospective design, inconsistent and often incomplete documentation, and limited granularity of the dataset, such as lack of detailed infant age stratification, nutritional information, and comprehensive clinical variables. Additionally, the cohort is overrepresented by community hospital data, which may impact generalizability.

Despite these challenges, this study provides valuable real‐world insights into infant EGD practice patterns and underscores the urgent need for evidence‐based guidelines and innovation in diagnostic approaches for this vulnerable population.


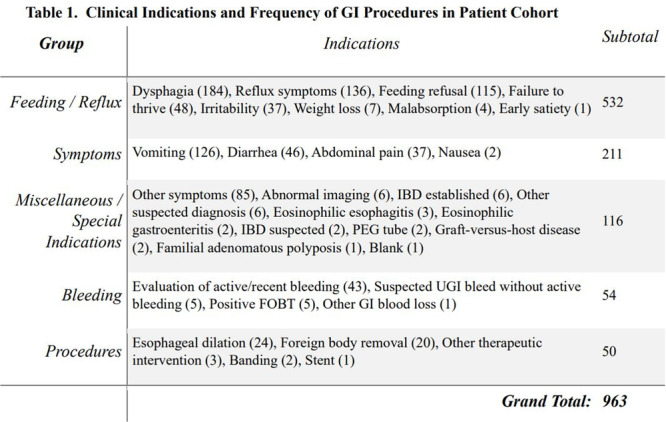


## 660 SECOND VICTIM SYNDROME IN PEDIATRIC GASTROENTEROLOGY: A PREVALENT PROVIDER WELLNESS ISSUE WITH A QUALITY‐OF‐CARE IMPACT

*Monique Barakat* ^*1,2*^, *Alka Goyal* ^*2*^, *Roberto Gugig* ^*2*^

^*1*^ *Pediatrics & Medicine*, *Stanford University School of Medicine*, *Stanford*, *CA*; ^*2*^ *Lucile Salter Packard Children's Hospital at Stanford*, *Palo Alto*, *CA*

**Introduction:** Second victim syndrome (SVS) refers to the emotional distress and psychological burden experienced by healthcare professionals after their patient is affected by an unanticipated adverse event or medical error. SVS can limit the scope of practice and overall wellness of the provider and tends to be more severe in those who care for pediatric patients. Surgical and critical care specialties recognize SVS and have strategies to address and prevent it, but this has never before been studied in pediatric gastroenterology. In the present era of escalating endoscopic interventions performed for pediatric patients, we evaluate the prevalence and impact of SVS within our pediatric gastroenterology program faculty and fellows.

**Methods:** We distributed a validated survey tool, the second victim experience and support tool (SVEST) developed to detect the prevalence of SVS, assess its impact across seven dimensions (psychological distress, physical distress, colleague support, supervisor support, institutional support, nonwork‐related support, and professional self‐efficacy) and evaluate two outcome measures relating to SVS (turnover and absenteeism). The electronic survey instrument was distributed to all faculty and fellows at our institution via email and responses analyzed using standard descriptive statistics.

**Results:** The survey was notable for a 72.7% (8/11) response rate from fellows and an 81.3% (26/32) response rate from faculty. Faculty respondents were distributed among junior (9, 34.6%), mid‐career (9, 34.6%) and established (8, 30.8%) faculty. Respondent characteristics are depicted in Figure 1. 33/34 (97.1%) respondents reported experiencing an endoscopy‐associated adverse event during a procedure they performed. Of the seven dimensions evaluated, psychological distress was most commonly experienced (82.4%, 28/34). Physical distress was experienced by 44.1% (15/34) of respondents. A detrimental impact on self‐efficacy was noted in 50% (17/34) of respondents, which included 75% (6/8) of fellows, 77.8% (7/9) of junior faculty, 33.3% (3/9) mid‐career faculty and 12.5% (1/8) of established faculty. Respondents reported a relatively low level of perceived support following prior adverse events, with only 14.7% (5/34) reporting adequate support after the adverse event. 20.6% (7/34) respondents considered leaving their current position following a second victim event. Additionally, in free text comments, faculty noted that the SVS experience limited the complexity of procedures they were willing to perform, their willingness to order and perform procedures, and the quality of care they could provide to subsequent patients. Most respondents (85.3%, 29/34) desired additional institutional or interpersonal support, with specific modalities of support they desired delineated in Table 1.

**Discussion:** In pediatric endoscopy, the vulnerable patient population raises the stakes for endoscopic success. When complications inevitably arise, trainees and gastroenterologists often struggle with self doubt, feelings of inadequacy and fear and/or a sense of judgment from peers and families—all consistent with SVS. While to some extent this represents the realities of growth and practice in a procedure‐based specialty, this can lead to burnout, impaired quality of life and job satisfaction, and even impact the quality of care provided to future patients.

For the first time we present objective data demonstrating a high prevalence of SVS and associated emotional distress among pediatric gastroenterology faculty and fellows. These data highlight SVS as an area in need of attention. Fellows and junior faculty are particularly vulnerable to the self‐efficacy impact of SVS and this can negatively impact their scope of practice and the quality of care they provide.

Addressing second victim syndrome involves creating a supportive environment where healthcare professionals can access resources to cope with their feelings. This is an area worthy of further multi‐institutional and society level study to understand broader patterns and formulate recommendations for initiatives to mitigate the impact of SVS and promote resilience in pediatric gastroenterology.


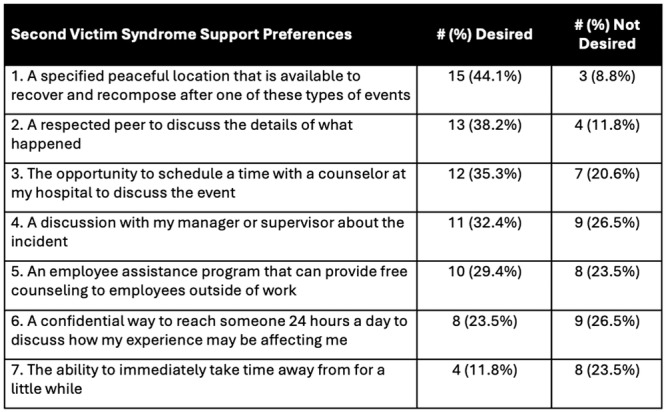


Table 1

## 661 STANDARDIZING A PROTOCOL FOR HELICOBACTER PYLORI GASTRIC BIOPSY CULTURE: FROM IMPLEMENTATION TO SUSTAINED PRACTICE

*Kim Ruiz Arellanos* ^*1*^, *Loida Estrella‐Pimentel* ^*3*^, *Jill Joeger* ^*3*^, *Jeff Cardini* ^*4*^, *Silvana Bonilla* ^*5,2*^

^*1*^ *Pediatrics*, *Boston Children's Hospital*, *Boston*, *MA*; ^*2*^ *Harvard Medical School*, *Boston*, *MA*; ^*3*^ *Laboratory Medicine*, *Boston Children's Hospital*, *Boston*, *MA*; ^*4*^ *Nursing*, *Boston Children's Hospital*, *Boston*, *MA*; ^*5*^ *Gastroenterology*, *Boston Children's Hospital*, *Boston*, *MA*

**Background:** The 2023 joint NASPGHAN/ESPGHAN guidelines for the management of *Helicobacter pylori* (*H. pylori*) infection continue to recommend against a test‐and‐treat approach. Instead, they recommend initial diagnosis via endoscopy with biopsies (EGD) when there is a valid indication, and strongly recommend gastric biopsy culture, when available, to confirm diagnosis and guide treatment. We previously reported on a quality improvement (QI) initiative to increase the rate of successful primary gastric biopsy culture at our hospital after identifying multiple factors affecting culture success^1^. We present follow‐up data and describe changes made to support implementation, which may inform similar efforts at other centers.

**Methods:** Using the Plan‐Do‐Study‐Act (PDSA) framework, interventions were implemented to: (1) consolidate specialty laboratory processing to a single outside laboratory (Mayo Laboratories), and (2) educate and provide reminders to gastroenterologists, endoscopy suite personnel, and laboratory staff. Descriptive statistics were performed on all collected variables. Differences in culture positivity by year, and before and after consolidation to a single specialty laboratory, were assessed using logistic regression, and a p‐chart was constructed to determine variation. All analyses were conducted using R (version 2025.05).

**Results:** Between November 1, 2019, and March 31, 2025, we observed a consistent increase in the number of gastric biopsy cultures obtained by gastroenterologists each year (55 in 2020, 95 in 2021, 109 in 2022, 146 in 2023, and 154 in 2024. Among patients with positive histology, a logistic regression model demonstrated a significant association between calendar year and the odds of a positive culture (OR 2.28 times (95% CI: 1.75–3.05, p < 0.001). (Table 1) A marked improvement in culture positivity was observed following the intervention to consolidate processing through a single outside specialty laboratory (Figure 1).

**Conclusion:** The interventions implemented through the QI initiative, such as staff education, standardized checklists, and a change in laboratory provider, may have led to sustained improvements in the success of primary gastric biopsy cultures at our hospital.

^*1*^ *Bonilla S, Bousvaros A, Cardini J, et al. Lessons From a Quality Improvement Project to Standardize the Process of Gastric Biopsy Culture for Helicobacter pylori. JPGN Rep. 2021 Aug*


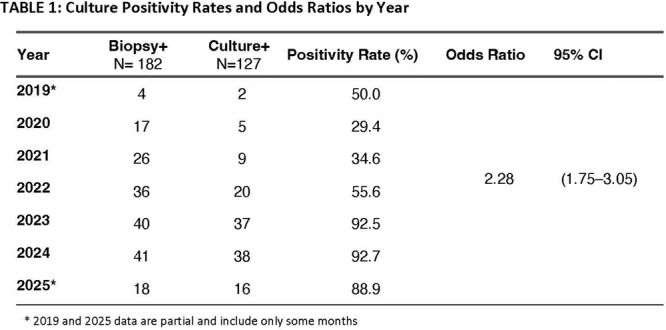



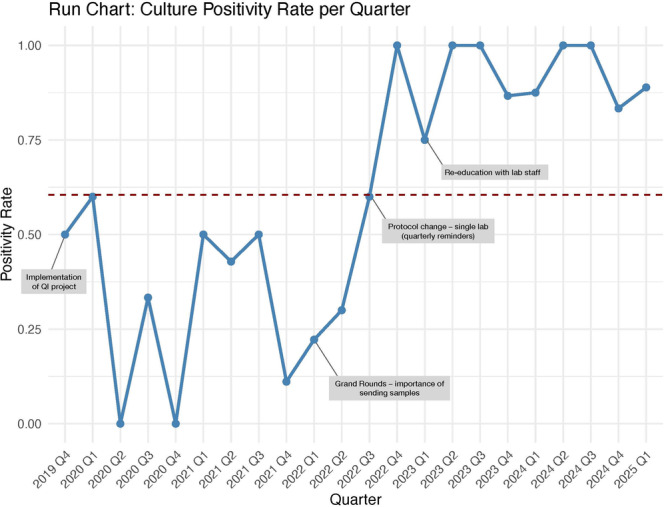


## 667 UPPER GASTROINTESTINAL ENDOSCOPY REPORTS; QUALITY OF DESCRPTION OF ABNORMAL FINDINGS

*Prisha Desai*, *Vrinda Bhardwaj*, *Thirumazhisai Gunasekaran*

*Pediatrics*, *Children's Hospital Los Angeles Department of Pediatrics*, *Los Angeles*, *CA*

North American and European Societies of Pediatric Gastroenterology have published guidelines on indicators and standards for safe endoscopy in children. It recommends, when describing lesions to use standardized disease‐related terminology and/or scales, when available. When procedures are done by endoscopist, who may share the care with partners in same group or with other group it becomes crucial to have a detailed and standard way of describing lesions. This results in seamless transfer of information resulting in safe patient care. There are guidelines for these in colonoscopy findings but few or none for upper gastrointestinal (UGI) finding.

So, the aim of this study is to assess the quality of UGI endoscopy reports focusing if a standardized terminology and/or scales were used to describe abnormal findings.

**Methods:** UGI endoscopy reports done at Children's Hospital of Los Angeles done over a period of 6 months (2‐2024 to 7‐2024) were included and were evaluated for the following quality metrics; indications, completing the procedure (examination up to II part of the duodenum), segment description (esophago‐ gastric junction and description of lesions; i) EoE (EREFS), ii) peptic esophagitis (Los Angeles Classification), iii) Hiatal hernia (from the esophageal view‐ Z line and diaphragmatic pinch and gastric view (Hill's classification) iv) caustic/button battery injury (Zargar's classification), v) strictures (location, extent, permeability of endoscope (outer diameter ‐OD) and description of mucosa post dilation, vi) esophageal varices (classification and findings of risk of bleeding), vii) polyps (location, type, and size), viii) erosions/ulcers (location, size, borders, and bed (Forrest classification of bleeding ulcers) and ix) mucosal injury from malabsorption‐ celiac disease. When reports had all features it was scored as complete and if any features were absent as incomplete. All reports were included except the following: post surgery changes of stomach, gastropathy of portal hypertension and achalasia dilation.

**Results:** Total reports reviewed were 596, excluded reports;8 and final reports reviewed were 588. All reports showed indication and were completed to II part of the duodenum. Reports with normal findings; 239 (40.65%) and abnormal findings 349 (59.35%). 3.3% of total reports documented the Z line and diaphragmatic pinch.

Rest of the abnormal findings were grouped and the details of complete and incomplete documentation in each group are given in Table 1. p values are scored but with the small sample size in each group it may not give a conclusive significance.

**Discussion:** UGI endoscopy, both diagnostic and therapeutic are done safely with quality indicators and metrics given to us resulting in high quality service delivered to the children. This should be reflected in a detailed description in the report in a standard way, which show cases that the gastroenterologist did a careful examination. This is the first step towards that goal. We will follow up with educating our GI Fellows/gastroenterologist and then will do a follow up analysis.

**Conclusion:** ‐Of the 588 reports reviewed 59.35% of reports had abnormal findings

‐Ulcers of duodenum/stomach, eosinophilic esophagitis and esophageal varices were the top three reports comprising around 10% on their own and 30% all together, of the abnormal findings

‐Eosinophilic esophagitis reports had the most complete findings and strictures the lowest.

‐This is the first step in improving the quality of UGI endoscopic abnormal findings

**References:**

1.Lisoa‐ Gonclaves P, et al. Quality of reporting in UGI endoscopy. GE Port J Gastroenterol 2019;26:24‐32

2.Zargar SA, et al. Ingestion of corrosive acids, spectrum of injury. Gastroenterology.

1989;97:702‐707


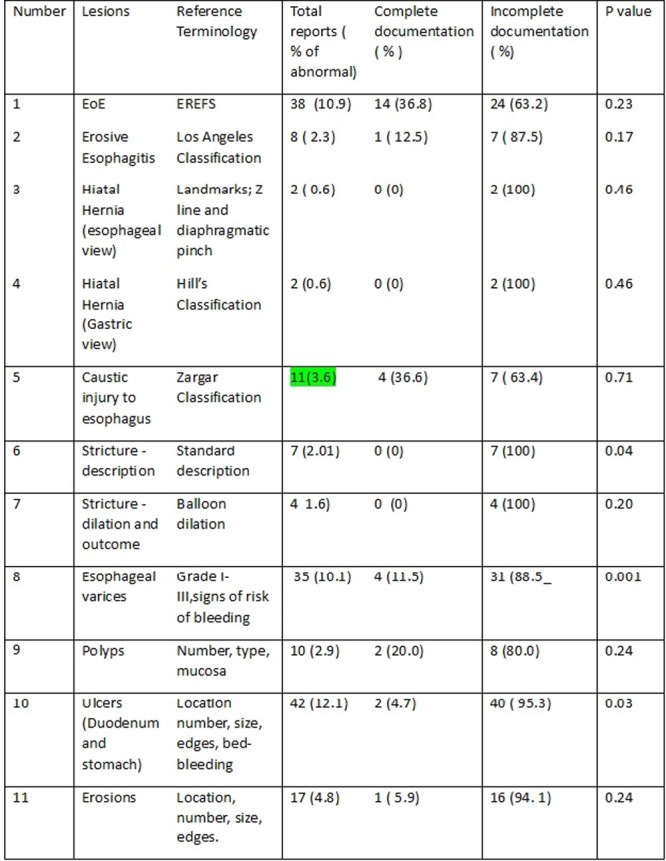


## 669 STRUCTURED PEDIATRIC ENDOSCOPY TRAINING: LESSONS FROM CURRICULUM INNOVATION

*Brett Hoskins*

*Division of Pediatric Gastroenterology, Hepatology, and Nutrition, Department of Pediatrics*, *Indiana University School of Medicine*, *Indianapolis*, *IN*

**Background:** Traditional pediatric endoscopy training often follows an informal apprentice‐based model, leading to variability in skill acquisition—especially for therapeutic procedures. Simulation and competency‐based frameworks have gained recognition but remain underutilized in many pediatric programs. A need existed for a formalized curriculum to standardize training and enhance technical proficiency.

**Objective:** To evaluate the impact of a structured, multimodal endoscopy curriculum on fellows’ perceptions of training adequacy and procedural comfort.

**Methods:** A structured endoscopy curriculum was implemented for six pediatric gastroenterology fellows over one academic year. The intervention included:

‐ A restructured first‐year introductory endoscopy month with didactics, simulation using the Thompson Endoscopic Skills Trainer®, consistent faculty educators, and an “endoscopy passport” to track progress.

‐ A second‐year curriculum focused on advanced therapeutic techniques, including stricture dilation, polypectomy, endoscopic mucosal resection, deep enteroscopy, exposure to ERCP and third space endoscopy, and others.

‐ Quarterly simulation sessions for all fellows.

‐ On‐demand educational endoscopy video tutorials and peer‐reviewed journal opporunities.

Pre‐ and post‐intervention surveys assessed fellows’ perceived training adequacy and procedural comfort using a five‐point Likert scale.

**Results:** The curriculum significantly improved fellows’ perceptions of formal endoscopy training adequacy (mean score: 2.2 pre‐ vs. 4.2 post‐intervention; *p* = 0.003). Perceptions of informal training remained unchanged (3.3 vs. 3.7; *p* > 0.05). Comfort with therapeutic endoscopy improved, though skill‐specific gains did not reach statistical significance.

**Conclusions:** A structured endoscopy curriculum enhances fellows’ confidence and perceived adequacy of training, particularly in formal endoscopy training. Simulation‐based learning, a clear tracking system, and faculty standardization may contribute meaningfully to skill development. Future work should include objective competency assessments, larger cohorts, and integration of emerging technologies like virtual reality to further improve pediatric endoscopy training.


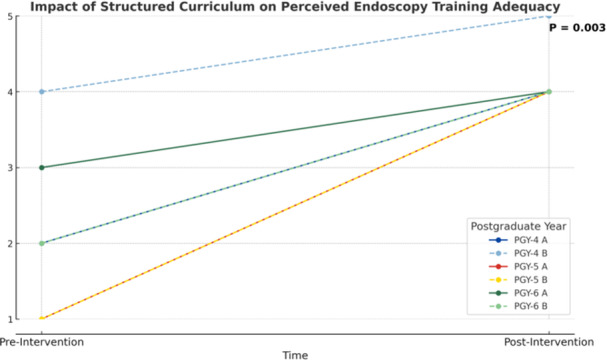


## 672 CAUSTIC SUBSTANCE INGESTION IN PEDIATRIC PATIENTS: CLINICAL AND ENDOSCOPIC CHARACTERISTICS, TREATMENT, AND COMPLICATIONS IN A TERTIARY CARE HOSPITAL

*Sabrina Abigail Medina Rodriguez*, *María del Rosario Alvarado Cifuentes*, *Ericka Montijo‐Barrios*, *Karen Ignorosa‐Arellano*, *Jose Francisco Cadena*, *Flora Zarate‐Mondragon*, *Erick Toro Monjaraz*, *Roberto Cervantes Bustamante*

*Gastroenterologia*, *Instituto Nacional de Pediatria*, *Mexico City*, *CDMX*, *Mexico*

**Introduction:** Accidental ingestion of caustic substances is a significant global problem due to its potential to cause severe injuries and complications that affect quality of life. 80% of these cases occur in children, with the majority being accidental. The extent of esophageal damage is related to the nature of the caustic substance. There is a lack of published data on this topic in the Mexican pediatric population, making it essential to document the findings obtained in the management of these patients in recent years.

**Objective:** Describe the clinical and endoscopic characteristics, treatment, and complications of caustic ingestion in pediatric patients.

**Methods:** Observational, retrospective, cross‐sectional, and descriptive study including patients with caustic ingestion treated in the Pediatric Gastroenterology and Nutrition Service from January 2019 to July 2024. Statistical analysis: descriptive analysis was performed. The results were analyzed using measures of central tendency to obtain percentages, mean, and average.

**Results:** A total of 91 patients were included over a period of 5 years, of which 61.53% were male and 38.46% female, with an average age of 47.9 months (8 m ‐ 205 m). The most common age group was older infants (36%). The most frequently ingested substance was caustic soda. The symptoms presented were sialorrhea, vomiting, irritability, and odynophagia (45%, 40%, 22%, and 12% respectively). Table 1 shows the percentage of stenosis presented in these patients.

**Discussion:** According to the literature, the risk of developing stenosis is directly proportional to the initial injury, which is similar to the findings in our study. The clinical presentation is variable; the severity of symptoms and their relationship to the severity of esophageal lesions is uncertain. Gorman et al. found that symptoms such as vomiting, sialorrhea, and stridor were predictive of severe esophageal injury.

**Conclusion:** The cases of esophageal stenosis observed are related to the degree of the Zargar Classification, which corresponds to the reported literature; present in 62.5% in Zargar 2B and 100% of patients with Zargar 3 A and 3B. Despite steroid treatment, all patients with Zargar 3 A and 3B developed stenosis. Therefore, it is important to perform an initial endoscopic evaluation to establish the treatment and prognosis of these patients.

**References:**

1.Hoffman RS, Burns MM, Gosselin S. Ingestion of caustic substances. *The New England Journal of Medicine* [Internet]. 2020;382(18):1739–1748. Available from: https://doi.org/10.1056/NEJMra1810769

2.Sutherland J, Bowen L. Ingestion of foreign bodies and caustic substances in children. BJA Educ [Internet]. 2023;23(1):2–7. Available from: https://doi.org/10.1016/j.bjae.2022.09.003

3.Pierre R, Neri S, Contreras M, Vázquez R, Ramírez LC, Riveros JP, et al. Guía de práctica clínica Ibero‐Latinoamericana sobre la esofagitis cáustica en Pediatría: Fisiopatología y diagnóstico clínico‐endoscópico (1 a. Parte). Rev Chil Pediatr [Internet]. 2020;91(1):149–57. Available from: https://doi.org/10.32641/rchped.v91i1.1288

## 674 THE IMPACT OF ENDOSCOPIC FINDINGS ON CLINICAL MANAGEMENT IN INFANTS UNDER 12 MONTHS: A RETROSPECTIVE REVIEW AT A SINGLE INSTITUTION

*Carey Johnson*, *Shirisha Reddy Sandadi*, *David Gremse*

*Pediatrics*, *University of South Alabama Health System*, *Mobile*, *AL*

**Purpose:** Gastrointestinal endoscopy is increasingly used in infants under 12 months to evaluate persistent gastrointestinal symptoms. Previous studies reported erosive esophagitis in this age group to be approximately 5.5% (Gilger, 2009).

**Objective:** To evaluate how endoscopic findings influence changes in clinical management in patients younger than 12 months at a single local hospital.

**Methods:** A retrospective chart review was conducted of all infants aged <12 months who underwent upper gastrointestinal endoscopy at the University of South Alabama Children and Women's Hospital between January 2020 and December 2023. Data collected included patient demographics, indications for endoscopy, endoscopic and histologic findings, pre‐ and post‐endoscopy diagnosis, and change in treatment management. The primary outcome was the proportion of patients diagnosed with endoscopic findings of conditions in addition to gastroesophageal reflux disease (GERD). Secondary outcomes included the types of management changes and associations between specific findings and treatment alterations.

**Results:** A total of 587 patients met the inclusion criteria. The most common indications for endoscopy were vomiting, poor weight gain, and blood in stool while on a hypoallergenic formula. Endoscopic findings included GERD (469), hiatal hernia (32), gastritis (10), erosive esophagitis (8), eosinophilic esophagitis (8), candida esophagitis (7), eosinophilic enteritis (7), and peptic ulcer (4 gastric, 1 duodenal). Erosive esophagitis was identified in 8 patients (1.4%). Overall, clinical management was modified in 90% (529/587) of patients following endoscopy. The most frequent management changes included initiating a different hypoallergenic formula (7%, 44/587), changing acid suppression therapy (82%, 484/587), swallowed topical corticosteroids (1.4%), and antifungal therapy (1.2%).

**Discussion:** In this cohort of infants under 12 months, erosive esophagitis was identified in only 1.4% of cases but endoscopic findings led to notable changes in clinical management, including initiation of acid suppression therapy, swallowed topical corticosteroids, antifungal therapy, and dietary modifications. The incidence of erosive esophagitis was lower in this retrospective study than in previous reports. This difference may be explained by increased use of acid suppression therapy in infants or may be explained by a difference in a community‐based referral population compared to a tertiary referral center population of patients.

**Conclusion:** Although endoscopy is indicated in only a small percentage of infants, findings on endoscopy may provide clinically actionable information, supporting the selective use of endoscopic evaluation in appropriately selected symptomatic infants.

## 675 THE ROLE OF INTERVENTIONAL ENDOSCOPY IN POST‐BARIATRIC SURGERY PEDIATRIC PATIENTS

*Michael Joseph*, *Jacob Mark*, *Robert Kramer*

*Pediatric Gastroenterology*, *University of Colorado Anschutz Medical Campus*, *Aurora*, *CO*

**Background:** Pediatric obesity and subsequently bariatric surgery continue to be on the rise in the United States. Sleeve gastrectomy and Roux‐en‐Y gastric bypass are the most common bariatric surgical procedures performed today. While efficacious, these procedures are not without complications such as: gastroesophageal reflux disease (GERD), sleeve stenosis or anastomotic leak/stricture. In the adult population, there are interventional techniques that are employed to treat these post‐surgical complications. To date, there is no pediatric data describing techniques or outcomes of these interventions.

**Methods:** Chart review was performed on patients at Children's Hospital Colorado who underwent interventional GI procedures (balloon dilation or gastric endovacuum) after bariatric surgery from 2020‐2025. Demographic, radiographic, procedural variables were all collected. Descriptive statistics were performed.

**Results:** Seven patients were included in our study. Six patients (85.7%) underwent a sleeve gastrectomy with the other patient having a Roux‐en‐Y gastric bypass. Five patients (71.4%) were female. Mean age was 16.0 +2.4 years. 57.1% of the cohort had gastroesophageal reflux disease (GERD) prior to surgery. Table 1 depicts pre‐endoscopy symptoms including: GERD, emesis, early satiety, abdominal pain and dysphagia. There was a total of 21 endoscopies performed. 76.2% (n=16) were done for gastric sleeve stenosis. Average time from surgery to initial EGD was 14.2 months. All patients had a fluoroscopic study (Upper GI) as part of the initial evaluation but, only 54.5% (6/11) studies noted any signs of sleeve stenosis. All dilations were performed with a Boston Scientific CRE balloon with one patient undergoing pneumatic dilation in their last endoscopy. Duration of symptomatic improvement from endoscopic dilation was highly variable (1‐18 months). Average EGDs per patient was 3 with a range of 1‐5 for those with sleeve stenosis. One patient with post‐surgical leak underwent 4 total endoscopies with 3 rounds of gastric endo‐vacuum therapy. Adverse event rate was 19% of EGDs with 2 requiring ED/PCP visit and one other requiring admission all for abdominal pain. No patients had post‐EGD perforation.

**Conclusion:** This is the first pediatric study to describe interventional procedures aimed at treating post‐bariatric surgery complications. Sleeve stenosis was the most common indication for intervention with Upper GI series not being a reliable non‐invasive test. We report a 19% adverse event rate (with no perforations) which must be taken into consideration pre‐operatively but, these procedures still provide benefit if persistent symptoms given the risks of re‐operation. Given the wide range of symptomatic improvement duration, balloon dilation does have therapeutic value even without radiographic evidence of sleeve stenosis and should be offered to post‐sleeve gastrectomy patients with significant GERD or dysphagia. Further multicenter data is needed to better elucidate the symptomatic benefits of interventional dilation for gastric sleeve stenosis.


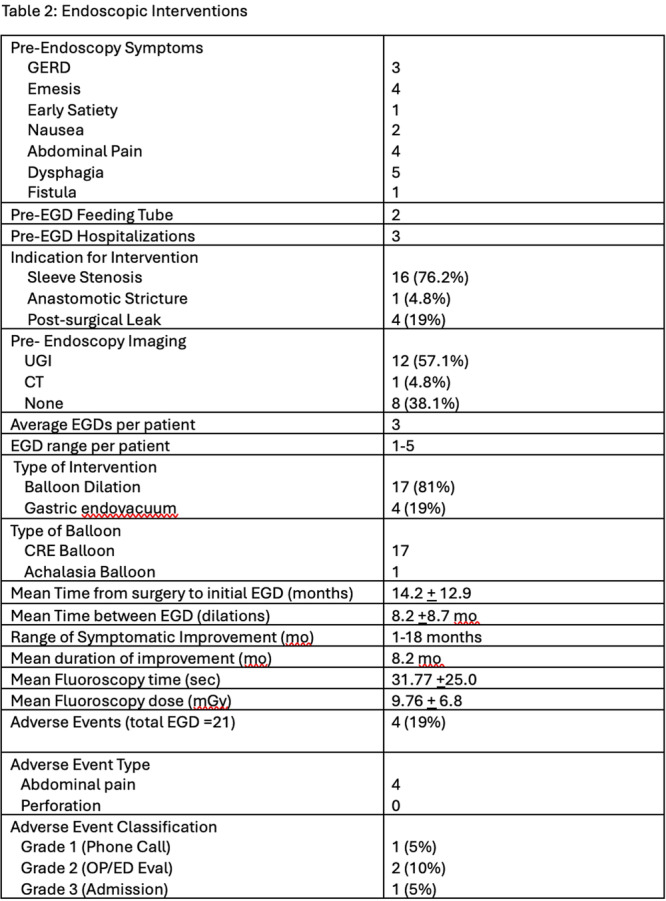


## 680 SEDATION‐FREE TRANS‐NASAL ESOPHAGOSCOPY IS SWEET: SAFE, WELL‐TOLERATED, EFFECTIVE, EFFICIENT, AND A TOTAL COST SAVER

*John Lyles* ^*1*^, *Katharine Newton* ^*2*^, *Bridget Dickinson* ^*2*^, *Bruno Chumpitazi* ^*1*^

^*1*^ *Duke University School of Medicine*, *Durham*, *NC*; ^*2*^ *Duke University Hospital*, *Durham*, *NC*

**Background:** Access to sedated endoscopy is limited, requires families to spend multiple hours in the hospital while missing work/school afterward, is expensive, and exposes patients to anesthesia. Sedation‐free trans‐nasal esophagoscopy (TNE) is a safe, well‐tolerated, effective, efficient, and less expensive alternative to sedated esophagogastroduodenoscopy (EGD).

**Aims and Measures:** Through TNE, this project aimed to increase patient access to endoscopy, eliminate risks of repeated anesthesia, decrease patient time in hospital, and reduce cost of care for families. To assess access to endoscopy, the number of TNEs and EGDs per month were measured and compared over time. To assess the length of stay in the hospital, the time spent in the hospital during TNEs and EGDs were measured and compared over time. To assess the cost of care, the charge for TNE was compared to charge for EGD. Demographics of patients undergoing TNE and tolerance of the procedure via TNEase, a provided recorded assessment of 1 (with ease) to 5 (procedure terminated), were also collected.

**Results:** Since May 2024, 46 (94%) patients successfully completed TNE at our institution, of which 7 have had multiple procedures. Patients were 6‐21 years old (median age 13 years old) with male predominance (73%). Procedures were well tolerated with median TNEase score of 1 (IQR 1, 2). Since implementation, TNE has increased access to endoscopy without impacting the number of EGDs per month (Figure 1) and decreased patient length of stay by 2 hours (Figure 2). Patients who underwent TNE were charged 55% less than patients who underwent EGD due to elimination of anesthesia associated costs.

**Discussion:** As an implementation feasibility project, TNE eliminated risk of anesthesia for select patients, was well‐tolerated with only 3 of 49 patients terminating the procedure (2 before scope insertion), increased access to endoscopy, decreased length of stay, and saved the families money. Areas for improvement include increasing knowledge of TNE to referring providers and families seeking care while planning for program expansion to multiple providers offering cases weekly.


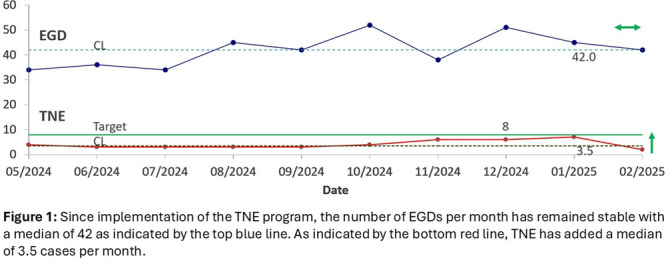



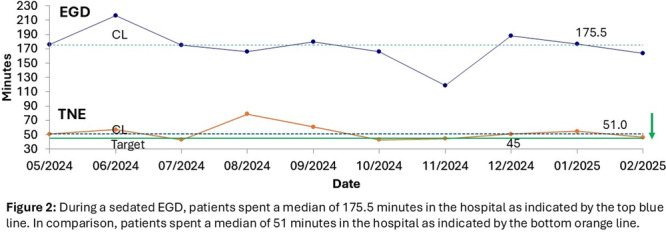


## 681 ALCIAN BLUE POSITIVITY IN ESOPHAGO ‐ CARDIAC MUCOSAL BIOPSIES IN PEDIATRIC AGE GROUP, A COMPREHENSIVE CLINICOPATHOLOGICAL STUDY IN 2587 CASES OF ESOPHAGEAL BIOPSIES

*Fatemeh Mahjoub* ^*1*^, *Mehri Najafi Sani* ^*2*^, *Shamim Nazemi Qeshmi* ^*1*^, *Mehrnaz Farasat* ^*3*^, *Pegah Pakzadian* ^*1*^, *Fatemeh Farahmand* ^*2*^, *Azizollah Yousefi* ^*4*^, *Farzaneh Motamed* ^*2*^

^*1*^ *Pathology*, *Tehran University of Medical Sciences School of Medicine*, *Tehran*, *Tehran Province*, *Iran (the Islamic Republic of)*; ^*2*^ *Gastroenterology*, *Tehran University of Medical Sciences Children's Hospital*, *Tehran*, *Tehran Province*, *Iran (the Islamic Republic of)*; ^*3*^ *Internal medicine*, *Tehran University of Medical Sciences School of Medicine*, *Tehran*, *Tehran Province*, *Iran (the Islamic Republic of)*; ^*4*^ *Pediatric gastroenterology*, *Iran University of Medical Sciences Faculty of Medicine*, *Tehran*, *Tehran Province*, *Iran (the Islamic Republic of)*

**Aim of Study:** Esophageal biopsies are obtained in most upper endoscopies performed in pediatric age group. It is taken from lower third of esophagus in more than 95% of cases. Cardiac mucosa is defined as a metaplastic change by some investigators although others believe that it is a normal part of stomach. Incidence of presence of columnar epithelium in biopsies taken from esophagus in pediatric age group is not vastly investigated. Moreover, Alcian blue staining of this type of epithelium is studied mainly in adults. The purpose of current study is to describe esophago‐cardiac biopsies in pediatric group in terms of Alcian blue positivity and it's correlation with clinical and upper endoscopic findings in 2587 cases.

**Material and Methods:** In this retrospective cross sectional study, biopsies obtained from esophagus by upper endoscopy were assessed. All the cases which include cardiac mucosa or cardio oxyntic type of mucosa were stained with Alcian blue and positivity was recorded. Also demographic and upper endoscopic findings were recruited from patient files.

**Results:** 2587 esophageal biopsies were obtained and sent to our lab. Mean age was 6 years (5 months ‐14 years) and 51 % of the cases were male. In 605 cases (23%) cardiac or cardio‐oxyntic mucosa was also seen (300 cases were esophago‐cardiac mucosa and 305 were esophago‐cardio oxyntic mucosa). Alcian blue stain was positive in 207 cases (8%) of total esophageal biopsies and 34% of total cases which were stained with Alcian blue. The positivity was recorded as focal (under 30% of areas) and more than focal and also the grade of intensity (weak, more than weak and strong). Most cases stained focally (92% of total positive cases) and from these 37% showed strong staining. Hiatal hernia was reported in 339 cases (13%) and in those with positive Alcian blue staining the incidence was 14.5% of total positive cases.

**Discussion:** Esophageal cancer is among the deadliest cancers in the world. One of the predisposing factors for this type of cancer is prolonged irritation of lower esophageal mucosa by acid due to esophago‐gastric reflux which is rather common in the pediatric age group. One of the initial steps in precancerous change is histologic properties of mucus which can be shown by Alcian blue stain. In this study we report 34% positive Alcian blue stain in pediatric age group which is relatively high. Although mostly the staining is seen focally (less than 30% of areas), however this can be an alarm sign for the prolonged irritation which can consequently lead to intestinal metaplasia. In 14% of all cases hiatal hernia was reported in upper endoscopy which is high and in 32% of such cases the hiatal hernia was medium to large. In this study we did not find statistically significant correlation between age (P e: 0.36) and sex (P : 0.07) with Alcian blue positivity. Interestingly, our study demonstrated a significant correlation between hiatal hernia and alcian blue positivity (P=0.002).

**Conclusion:** Incidence of altered mucus property is rather high in esophageal biopsies and we recommend to report Alcian blue positivity in these cases in order to warn clinicians of the possible chronicity and to emphasize that adherence is crucial to avoid metaplastic changes in lower esophagus.


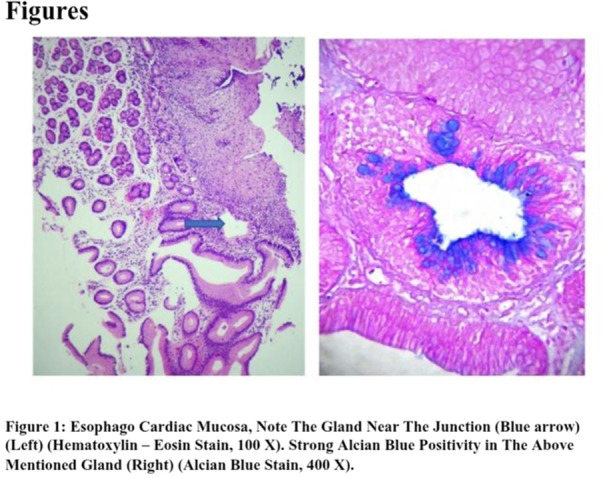


## 685 CLEEN: COMPLETING LOWER ENDOSCOPY EFFICIENTLY THROUGH INNOVATION

*Reshma Patel* ^*1*^, *Kristina Nazareth‐Pidgeon* ^*2*^, *Bruno Chumpitazi* ^*1*^, *John Lyles* ^*1*^

^*1*^ *Pediatric Gastroenterology, Hepatology, and Nutrition*, *Duke University Medical Center*, *Durham*, *NC*; ^*2*^ *Pediatric Hospital Medicine*, *Duke University Medical Center*, *Durham*, *NC*

**Introduction:** Colonoscopy is vital for diagnosis and treatment in pediatric gastroenterology. However, the efficiency and utility of the procedure are impacted by the quality of pre‐procedure bowel preparation. Nearly half of the patients undergoing colonoscopy at our institution have inadequate bowel preparation according to Boston Bowel Preparation Scale (BBPS). Patients with inadequate bowel preparation are at risk for increased exposure to anesthesia, incomplete mucosal evaluation, and aborted procedures. This quality improvement study aimed to improve the percentage of adequate BBPS scores during outpatient colonoscopies from 50% to 70% by October 2025.

**Methods:** Chart abstraction was completed on all pediatric patients who underwent outpatient colonoscopy since March 2024. Exclusion criteria included history of bowel resection or transplantation. The primary measure was the frequency of adequate BBPS scores, with adequate defined as ≥6 on a scale of 0 to 9. Demographic data (age, gender, insurance type, and race) was also recorded. Pareto charts and Key Driver Diagrams drove root cause analysis and corresponding interventions (Figure 1). Interventions included updating bowel preparation instructions, BBPS education for proceduralists, and access to purchase bowel preparation kits at the hospital pharmacy.

**Results:** Pareto charts demonstrated no difference in bowel preparation quality by procedure day or clinic. When assessing BBPS scores by insurance type, 66% of patients with Medicaid had inadequate BBPS scores compared to 44% of patients with commercial insurance. No difference was noted in BBPS by age, gender, or race.

Despite multiple interventions, the median frequency of adequate BBPS scores remained at 45%. Data remained within control limits and gradually approached the center line over time. For the most recent colonoscopies, there was a sustained improvement in the frequency of adequate BBPS scores to greater than 70% (Figure 2). After implementation of the BBPS as a quality measure, documentation by proceduralists was initially high, but gradually decreased to as low as 50%. While BBPS re‐education briefly improved documentation, it later declined again until a systematic requirement to record BBPS scores was added to procedure documentation software.

**Discussion:** CLEEN addresses the issue of inadequate bowel preparation prior to pediatric colonoscopy at our institution. While interventions targeting education and process standardization did not immediately improve BBPS scores, there has been a positive trend for the last 30 colonoscopies. Delayed improvement of BBPS scores may be due to slower adoption of changes in addition to a lead time of 4‐6 weeks between ordering a colonoscopy to the day of procedure. Implementation of mandatory BBPS documentation within procedural software represents a system‐level change that is expected to be more sustainable. The disparity in bowel preparation adequacy based on insurance status underscores potential socioeconomic barriers to preparation compliance. Future interventions should address access, affordability, and health communication tailored to diverse populations. While promising, the sustainability and generalizability of these findings require further study. Limitations include potential bias in BBPS scoring and the limited sample size in the final subgroup showing improvement. Future steps involve assessment of inter‐observer reliability of recorded BBPS scores and use of digital navigation applications to improve patient communication.


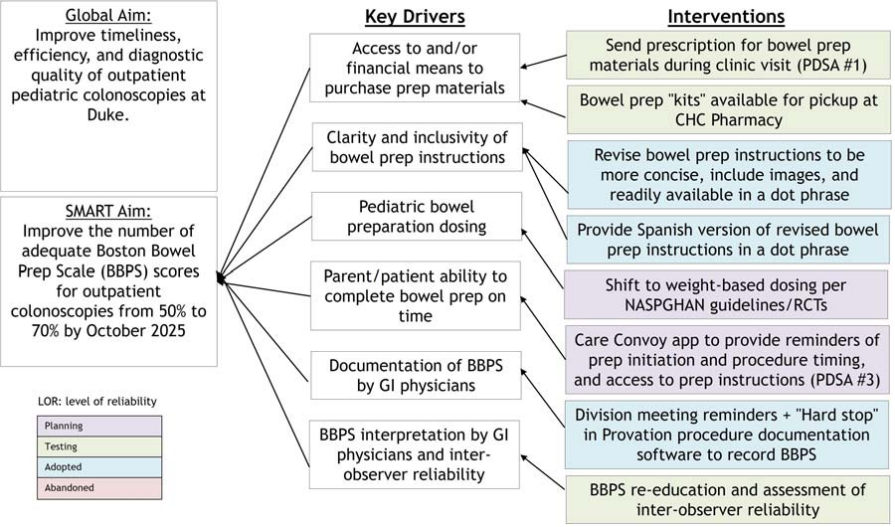


Key Driver Diagram with corresponding interventions.


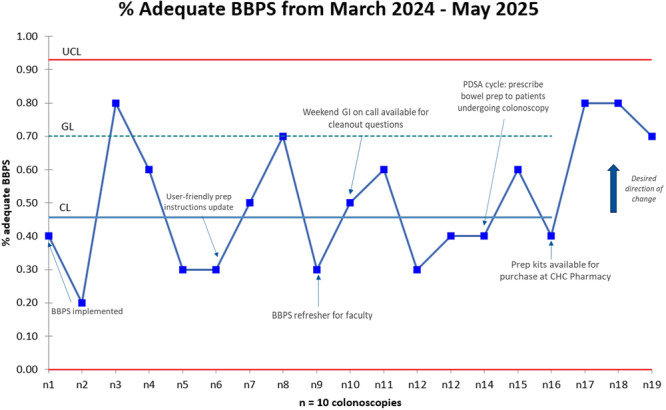


Control chart demonstrating improved process control and improving bowel preparation quality.

## 690 SAFETY OF RECTAL MIDAZOLAM AS A SINGLE AGENT SEDATIVE IN INFANT ESOPHAGOGASTRODUODENOSCOPY ‐ A SINGLE CENTER RETROSPECTIVE STUDY

*Shirisha Reddy Sandadi*, *Carey Johnson*, *David Gremse*

*Pediatrics*, *University of South Alabama*, *Mobile*, *AL*

**BACKGROUND:** Pediatric endoscopy is done for diagnostic and therapeutic benefits. Sedation is an important predictor of procedural success and ensures patient safety, comfort and cooperation. Whether infants need sedation for medical procedures depends on the potential for pain and the need for immobility. General anesthesia is typically used to achieve the goals of pain relief and immobility when endoscopy is performed. There have been concerns based on animal studies that exposure to general anesthesia in early life may affect neurodevelopmental outcomes due to neuroapoptosis or synaptogenesis disruption. Midazolam is effective and commonly used for sedation in infants. Studies have shown the safety and efficacy of benzodiazepine and opioid combinations such as midazolam/meperidine and ketamine with/without midazolam.

**OBJECTIVE:** To assess the safety of rectal midazolam when used as a single agent sedative in infant esophagogastroduodenoscopy (EGD).

**METHODS/DESIGN:** This study involves a single center IRB approved retrospective chart review of elective, diagnostic EGDs done in infants (age ≤1year) at USA Health Children's and Women's Hospital during 2020‐2023. Midazolam was administered per rectum at a dose of 1 mg/kg in infants 2‐12 months of age and a lower dose of 0.75 mg/kg for infants 0‐2 months of age. Infants were continuously monitored including heart rate, blood pressure and oxygen saturation. The data collected included age, gender, type of sedation, immediate complications, delayed complications (≤30days ER visit/admission). Patients admitted to inpatient service prior to procedure, age >1 yr, EGD done for foreign body removal, lost to follow up and sedation other than midazolam were excluded from the study. Immediate complications were characterized by intra/postprocedural respiratory/cardiovascular instability requiring intravenous fluids or oxygen, prolonged recovery due to lethargy. Delayed complications were characterized by poor feeding/vomiting/altered mental status/respiratory symptoms not related to previous GI issues or infections.

**RESULTS:** A total of 582 patient records were reviewed out of which 559 (96%) received rectal midazolam, 20 (3.4%) received general anesthesia and 3 (0.5%) had no data on sedation. Descriptive analysis showed that immediate complications occurred in 1 infant (0.2%) and delayed complications were seen in 50 patients (8.9%). Out of the total 51 complications seen, 7(1.2%) were likely sedation‐related with a CI of (0.3 ‐ 2.1) and 44(7.9%) were non‐sedation related. A binomial test revealed that the complications with midazolam were significantly lower than test proportion with a P value of <0.001. Fischer's Exact test revealed that the proportion of sedation‐related complications were significantly lower than non‐sedated related ones with a P value <0.001. None of the infants needed flumazenil to reverse the effects of rectal midazolam.

**LIMITATIONS:** Retrospective nature of study, lack of documentation at times and misclassification bias due to a non‐blinded study are some of the limitations for this study.

**DISCUSSION:** The goal of sedation in pediatric endoscopy is to minimize discomfort and improve patient cooperation, thereby completing the procedure effectively and to minimize need for repeat endoscopy. This can be achieved using various levels of sedation. Conscious sedation with midazolam as a single agent is effective in providing the level of sedation required with minimal complications. It is also cost effective and requires less personnel and equipment while also minimizing post operative pain needing analgesia with agents like morphine, reduces trauma associated with intubation required for general anesthesia. Midazolam also has an antagonist called flumazenil if needed. Topical anesthetic sprays containing benzocaine can be used on the pharynx to minimize the discomfort and gag reflex associated when the endoscope is inserted.

**CONCLUSION:** Midazolam can be a safe and effective option for sedation of infants for endoscopy.

## 696 IMPROVING STANDARDIZED ENDOSCOPIC DOCUMENTATION IN PEDIATRIC EOSINOPHILIC ESOPHAGITIS: A QUALITY IMPROVEMENT INITIATIVE TO IMPLEMENT EREFS SCORING

*Martin Varea Romo* ^*1*^, *Christian Martinez* ^*2*^, *Annette Medina* ^*1*^

^*1*^ *Pediatric Gastroenterology*, *Nicklaus Children's Hospital*, *Miami*, *FL*; ^*2*^ *Pediatrics*, *Nicklaus Children's Hospital*, *Miami*, *FL*

**Background:** Eosinophilic esophagitis (EoE) is a chronic immune‐mediated condition increasingly recognized in children. The Endoscopic Reference Score (EREFS) is a validated classification system that standardizes esophageal findings, quantifies disease severity, improves clinical communication, and assesses response to therapy. Despite its utility and broad acceptance, EREFS was not a routine practice at our institution. This underutilization created gaps between microscopic and macroscopic documentation, limiting objective tracking of disease activity and treatment response.

**Objective:** To assess baseline use of EREFS in esophagogastroduodenoscopy (EGD) reports of patients with EoE during diagnostic or surveillance endoscopy at a tertiary pediatric care center over a 6‐month period and, through targeted interventions, increase scoring system utilization to a minimum of 80% over a 12‐month period.

**Methods:** Plan‐Do‐Study‐Act (PDSA) quality improvement model was applied from July 2023 to May 2025 at Nicklaus Children's Hospital. We reviewed EGDs performed between July and December 2023 to establish baseline EREFS usage. Interventions began in December 2023 and included provider education via didactics and case‐based teaching, laminated reference guides at workstations, integration of an EREFS template in the EndoVault endoscopy system, and regular email updates to encourage use. Post‐intervention EGDs from December 2023 to April 2025 were then reviewed to assess changes.

**Results:** During the pre‐intervention phase, 76 EGDs were reviewed, with 30 patients (39.4%) having biopsy‐confirmed EoE. Of those, 22 had a prior EoE diagnosis. One report (1.3%) included an EREFS score, and among all EoE patients, 1 of 30 (3.3%) had EREFS documented. In contrast, 90% of pathology reports included eosinophil counts, highlighting the inconsistency between endoscopic and histologic reporting. In the post‐intervention phase, 78 EGDs were reviewed. 36 patients (46.2%) had biopsy‐confirmed EoE; 22 had prior diagnoses and 14 were newly diagnosed. Among known EoE patients, 11 of 22 reports (50%) included EREFS scoring; an approximate 1001% relative increase. In newly diagnosed patients, 4 of 14 (28.6%) had EREFS documented during initial endoscopy.

**Discussion:** The most notable improvement occurred between January and September 2024, coinciding with structured education and the integration of the EREFS template into the documentation system. Sustained improvement continued through April 2025, despite the absence of additional educational efforts, suggesting that system‐level integration and consistent email reminders effectively supported long‐term behavior change. This suggests that documentation gaps may have been influenced more by challenges with workflow integration and differences in provider familiarity, rather than diagnostic uncertainty. The improvement among both previously diagnosed and newly diagnosed patients suggests that even low‐cost, targeted interventions can significantly shift provider practice and improve standardization.

**Limitations:** This project was conducted at a single center, which may limit the generalizability of the findings. A correlation between EREFS scoring and clinical or histologic outcomes was not assessed. Sustainability beyond the observation period remains to be evaluated.

**Conclusion:** A structured, multi‐phase intervention significantly increased use of EREFS documentation in pediatric EoE patients, from 3% to 50% in those with known disease and 28.6% in new diagnoses. This approximate 1001% relative increase highlights the effectiveness of targeted quality improvement efforts. EREFS enables objective disease tracking and enhances communication across providers. Broader implementation of this tool could support more standardized care and improve outcomes for children with EoE.


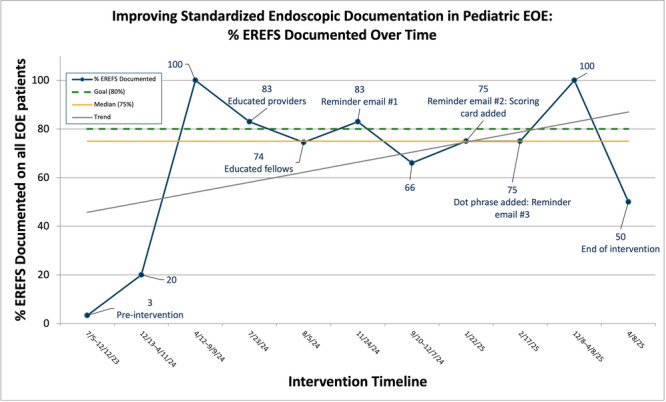


Figure 1: Percentage of EREFS documentation in pediatric EoE during the quality improvement initiative. Baseline documentation was 3% (“Pre‐intervention”), increasing after targeted interventions including provider and fellow education, reminder emails, a scoring card, and dot phrase integration. Intervention dates are labeled along the timeline. The green dashed line represents the documentation goal (80%), and the solid orange line represents the median (75%). The gray trend line highlights improvement over time.

## 698 REAL‐WORLD OUTCOMES OF OFF‐LABEL DUPILUMAB USE IN PEDIATRIC EOSINOPHILIC ESOPHAGITIS

*Sasha Jane Abi Aad* ^*1*^, *Grace Dittmar* ^*2*^, *Nicole Misner* ^*1*^, *Sharon Albers* ^*3*^, *Athanasios Tsalatsanis* ^*5*^, *Panida Sriaroon* ^*4*^, *Racha Khalaf* ^*1*^

^*1*^ *Pediatric Gastroenterology*, *University of South Florida Morsani College of Medicine*, *Tampa*, *FL*; ^*2*^ *Pediatrics*, *University of South Florida Morsani College of Medicine*, *Tampa*, *FL*; ^*3*^ *Department of Pediatrics, Division of Dermatology, University of South Florida Morsani College of Medicine*, *Tampa*, *FL*; ^*4*^ *Pediatric Allergy and Immunology*, *University of South Florida Morsani College of Medicine*, *Tampa*, *FL*; ^*5*^ *Research Methodology and Biostatistics Core, University of South Florida Morsani College of Medicine*, *Tampa*, *FL*

**Introduction** Eosinophilic esophagitis (EoE) is a chronic inflammatory disease that often presents in very young children with nonspecific symptoms such as vomiting, food aversion, and failure to thrive, which can delay diagnosis. There is no single standard treatment, and current management includes dietary elimination, proton pump inhibitors (PPIs), swallowed corticosteroids, and dupilumab, a monoclonal antibody that inhibits IL‐4 and IL‐13. Initially approved for atopic dermatitis in adults and later extended to children as young as 6 months and ≥ 5 kg, dupilumab received approval by the US Food and Drug Administration for patients with EoE ≥ 12 years in 2022 and later for children ≥ 1 year or ≥ 15 kg in 2024. Our study aimed to investigate the impact of dupilumab on EoE outcomes in patients < 1 year or < 15 kg who received this therapy off‐label for concurrent atopic dermatitis.

**Methods:** This is a retrospective single‐center study. We queried the electronic medical records to identify individuals less than 1 year of age or less than 15 kg, diagnosed with EoE (ICD‐10 K20.0) and prescribed dupilumab, at the University of South Florida Pediatric specialty clinics which include pediatric gastroenterology, dermatology and allergy immunology. Patients were included in the results if they had an esophagastroduodenoscopy (EGD) pre‐ and three to six months post‐dupilumab initiation. Student's t‐test and chi‐squared test were used (significance p < 0.05), and a patient‐level plot was designed to visualize individual changes in peak eosinophils along the esophageal mucosa.

**Results:** Fifteen patients with EoE <1 year or <15 kg on dupilumab were identified between 2021‐2025. Five were excluded due to missing follow‐up EGD. Among the 10 included, 60% were male, 60% identified as white, and 80% as not‐hispanic or Latino. The mean age at dupilumab initiation was 27.8 ± 13.3 months, and the mean weight was 12.3 ± 2.7 Kg. Following dupilumab treatment, there was a significant decrease in the eosinophils per high‐power field (hpf) in the distal esophagus (47.8 ± 39.1 vs. 4.9 ± 8.0 p = 0.009) and in the mean peak eosinophils across the esophageal mucosa (64.1 ± 63.1 vs. 5.7 ± 8.8 p = 0.006). All patients showed a reduction in peak eosinophils per hpf, with 80% demonstrating <15 eosinophils/hpf on their second EGD (Figure 1). However, the Eosinophilic Esophagitis Endoscopic Reference (EREF) score remained unchanged. Before dupilumab, 70% had trialed PPI and/or swallowed steroids and failed, and 70% (n=7) were on dietary therapy for EoE. At follow‐up, 57% of those patients (n=4) had liberalized their diet, and 100% (n=10) had a documented increase in their oral intake. Additionally, there was a reduction in atopic dermatitis lesions, with a significant decrease in body surface area (BSA) involved (15.8 ± 18.6 % vs 3.1± 6.9 %, p=0.022) (Table 1).

**Conclusion:** Early initiation of dupilumab at the atopic dermatitis dose may be a safe and effective therapy for EoE; however, further research is needed to confirm our results and evaluate for adverse events.


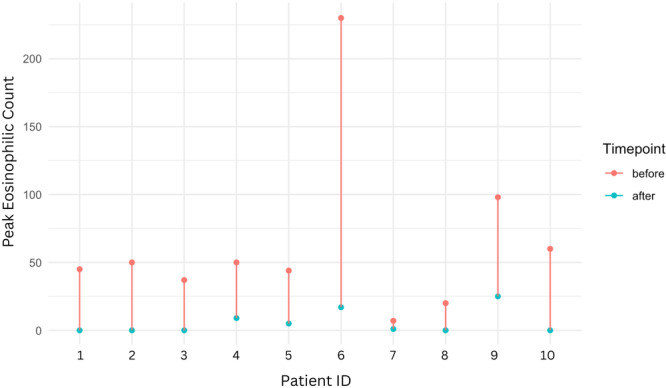


Figure 1: Individual changes in peak eosinophils per high‐power field before and after dupilumab treatment in pediatric patients with eosinophilic esophagitis under 1 year of age or less than 15 kg.


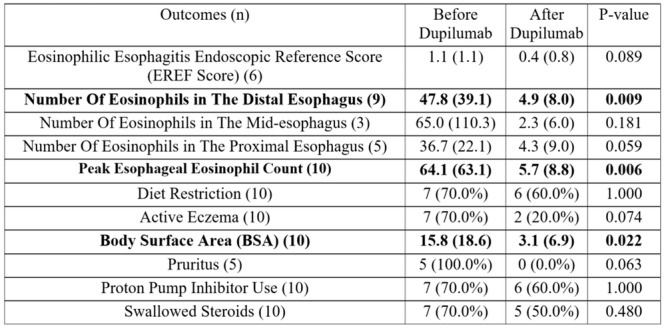


Table 1: Patient outcomes before and after 3 to 6 months of dupilumab treatment in pediatric patients with eosinophilic esophagitis under 1 year of age or less than 15 kg.

## 699 BONE MINDERAL DENSITY AMONG CHILDREN AND ADOLESCENTS WITH EOSINOPHILIC ESOPHAGITIS: A SINGLE CENTER EXPERIENCE

*Sharef Al‐Mulaabed*, *Hiba Hajissa*, *Yamen Smadi*

*Center for Digestive Health and Nutrition*, *Orlando Health Arnold Palmer Hospital for Children*, *Orlando*, *FL*

**Background and Aim:** Eosinophilic esophagitis (EoE) is a chronic, immune‐mediated inflammatory disease of the esophagus that causes dysphagia, which may impair oral intake and nutrition. Children and adolescents with EoE are at increased risk for low bone mineral density (BMD), primarily due to dietary restrictions, treatment with corticosteroids or PPI, as well as the underlying inflammatory process itself. This study aimed to assess BMD in pediatric patients with EoE and explore associations with demographic factors and therapeutic interventions.

**Methods:** We retrospectively reviewed data from all patients with EoE who underwent dual‐energy X‐ray absorptiometry (DEXA) between January 2020 and May 2025. A BMD Z score ≤ ‐2.0 was considered as "low for age", per the International Society for Clinical Densitometry (ISCD) and the American Academy of Pediatrics (AAP) guidelines. BMD measurements at the lumbar spine (average of L1‐L4) with their Z scores were retrieved, along with demographic and clinical characteristics and therapies of EoE.

**Results:** A total of 49 EoE patients were included; 39 (78%) males, with a mean (±SD) age at diagnosis of 6.6 (±4.7) years. DEXA scan was performed at median (interquartile ratio) of 1.75 (0.75‐3.3) years after EoE diagnosis. Mean (±SD) Z score bone density in our cohort was ‐1.07 (±1.22). Low BMD was identified in 9 out of 49 EoE patients (18.4%). There were no significant differences in sex, age at diagnosis, age at the time of the DEXA scan, or time since diagnosis in those with and without low BMD, table 1.

Patients with low BMD had lower mean weight Z scores (Mean ± SD of ‐1.08 ±1.61) compared to those with normal BMD (‐0.04 ± 1.02), with a trend toward significance (p=0.092). On the other hand, there was no significant difference in BMI Z scores between the two groups (p=0.569), table 1. There is a significant positive correlation found between DEXA Z scores and both weight Z scores (r=0.429, p=0.002) and BMI Z scores (r=0.317, p=0.028)”.

Although patients who are not on steroid therapy had a higher proportion of low BMD (36.4%) compared to those on steroid (13.5%), the difference was not statistically significant (p = 0.180). Among those using steroid users, there was no significant difference in low BMD prevalence between once‐daily (11.1%) and twice‐daily (14.3%) dosing (p = 1.000).

**Conclusions:** There was a considerable degree of low BMD Z scores among children and EoE patients in our single center cohort, with correlation mainly with weight and BMI suggesting that nutritional status may play a key role in bone health of those patients. Ongoing monitoring of nutritional status and bone health should be considered in all EoE patients, especially during their active growth.


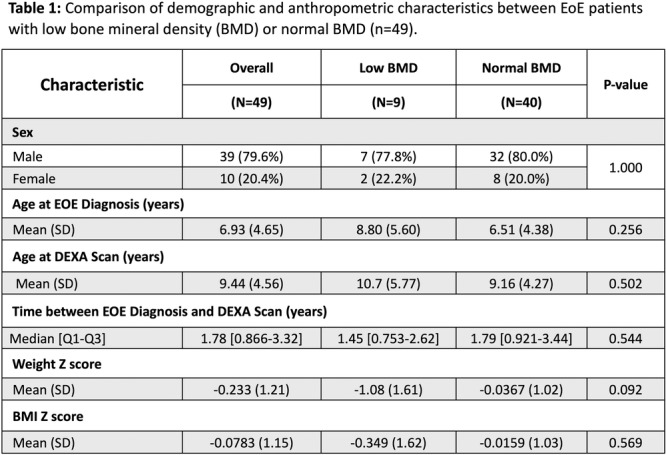



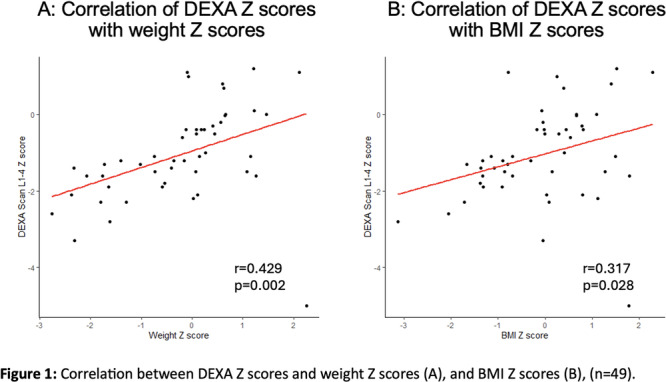


## 700 EOSINOPHILIC ESOPHAGITIS IN INFANTS AND CHILDREN PRESENTING TO AN URBAN MULTIDISCIPLINARY FEEDING CLINIC

*Victoria Berger*, *Sarah Elzayat*, *Melanie Greifer*, *Abha Kaistha*

*Pediatric Gastroenterology and Hepatology*, *New York University*, *New York*, *NY*

**Background:** Pediatric feeding disorders in infants and children can present as vomiting, failure to thrive, feeding difficulties and delayed transition to age‐appropriate foods. These were the main presenting symptoms in infants and children seen in our outpatient multidisciplinary feeding clinic. Routine evaluation for patients with a history of atopy and allergies included endoscopy with biopsy to diagnose EoE. Our results demonstrate that infants and children presenting with feeding difficulties and predisposing allergies and atopy benefit from a multidisciplinary approach incorporating EoE evaluation along with feeding therapy.

**Methods:** A retrospective chart review of 91 patients presenting to our multidisciplinary feeding clinic was performed. 36 patients were identified as having atopy or allergies. We further identified patients who underwent endoscopy with biopsy and had positive EoE findings. Those with positive EoE findings underwent treatment with oral proton pump inhibitors, viscous budesonide, elemental diet, or combination therapy with simultaneous feeding therapy.

**Results:** Out of the 36 patients identified, 29 [81%] had food allergies, 21 [58%] had eczema, 5 [14%] had environmental allergies, and 4 [11%] had asthma. There was a predominance of males identified in our patient population (75%), which is consistent with the literature. 22 of our 36 [61%] patients underwent EGD and 14 [64%] were diagnosed with EoE. Out of the 14 patients diagnosed with EoE, 2 [14%] were less than 12 months old, 6 [43%] were 12‐24 months old, and 4 [29%] were 24‐36 months old. All patients with EoE underwent appropriate treatment. 10 out of 14 [71%] patients diagnosed with EoE were found to have oral stage of swallowing impairment and delayed development in feeding skills. All patients with EoE underwent appropriate treatment and concurrent feeding therapy.

**Conclusions:** We demonstrated that a high prevalence of EoE exists in infants and children with feeding difficulties and presence of atopy and allergies. Infants as young as 11 months old with atopy and allergies can have underlying EoE. Prompt EoE diagnosis and subsequent treatment with concurrent feeding therapy was shown to improve presenting symptoms in our population. Although this is a selective population presenting to our multidisciplinary feeding clinic, it is important that infants and children presenting with pediatric feeding disorders should be further investigated to rule out presence of EoE.


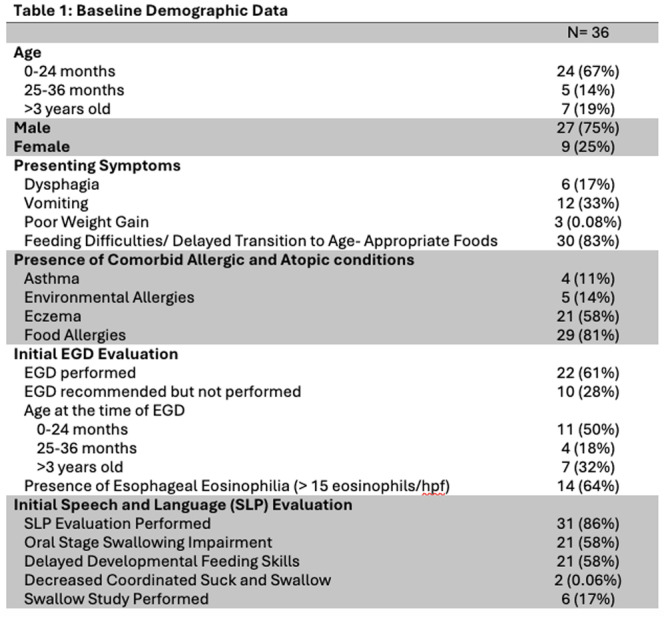



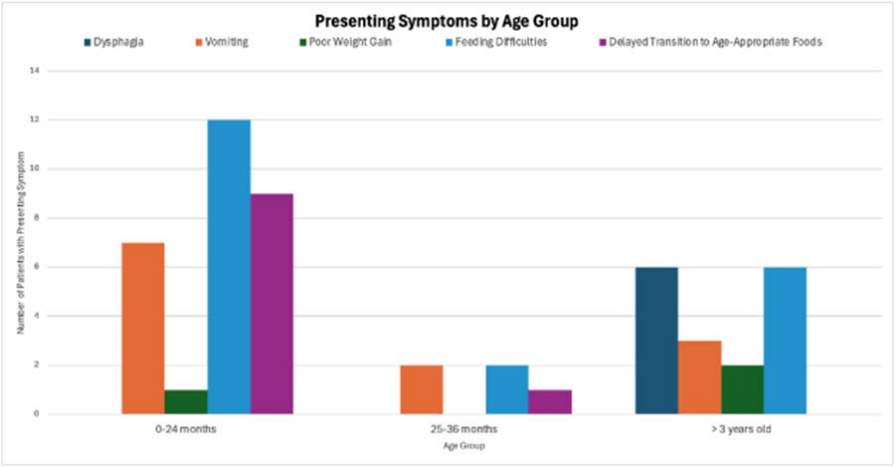


## 701 UTILIZATION OF QUERY LANGUAGE AND AI‐BASED TOOLS TO IMPROVE IDENTIFICATION OF NEW PEDIATRIC EOSINOPHILIC ESOPHAGITIS CASES ACROSS A LARGE HEALTH CARE SYSTEM

*Paola Blanco* ^*1*^, *Aubrey Muller* ^*1*^, *Tina Wadhwa* ^*3*^, *Krista Grant* ^*4*^, *Ashley Wegrowski* ^*1*^, *Allison Smego* ^*2*^, *Kathryn Weirich* ^*1*^, *Jacob Robson* ^*1*^

^*1*^ *Pediatric Gastroenterology*, *University of Utah Health*, *Salt Lake City*, *UT*; ^*2*^ *Pediatric Endocrinology and Diabetes*, *University of Utah Health*, *Salt Lake City*, *UT*; ^*3*^ *Enterprise Analytics*, *Intermountain Health*, *Salt Lake City*, *UT*; ^*4*^ *Pediatric Pathology*, *University of Utah Health*, *Salt Lake City*, *UT*

**Background:** Manual chart review has been the gold standard for retrospectively identifying diagnoses of eosinophilic esophagitis (EoE), but these methods are time and labor intensive, especially within large health care systems performing thousands of upper endoscopies annually. ICD‐based medical code searches for EoE cases are specific but low sensitivity, limiting their utility for population‐based epidemiology research. With the growing burden of EoE, there is a critical need for case identification methods that are scalable, efficient, accurate and reproducible. We aimed to evaluate and compare two automated strategies for identification of new pediatric EoE diagnoses across a large, integrated healthcare system: 1. a structured query language (SQL)‐based code and 2. an artificial intelligence (AI)‐based tool. We compared these methods against the previously defined gold standard method of patient chart review by a trained clinician.

**Methods:** We conducted a retrospective review of all pediatric upper endoscopies with esophageal biopsies performed from January 2024 through June 2024 for patients under age 18 across the Intermountain Health (IH) system. This study was approved by the University of Utah & IH Institutional Review Board. The majority (>90%) of pediatric endoscopic procedures in the Intermountain West region (Utah and parts of the surrounding states) are performed at IH facilities. The gold standard was established via comprehensive manual chart and pathology record review of all patients who had an upper endoscopy. A new pediatric EoE diagnosis was defined by meeting the following criteria: presence of esophageal symptoms, a biopsy with ≥15 eosinophils per high power field and a thoughtful assessment for other systemic causes of esophageal eosinophilia. Two automated search strategies were tested: 1. SQL‐based code to parse unstructured pathology reports for eosinophil patterns and 2. An AI tool, GPT 4o mini, trained to extract and interpret eosinophil counts from free‐text pathology notes. Both the SQL‐based code and GPT 4o mini queries ran on the IH‐enterprise data platform which stores pathology data extracted from the electronic medical record. Case identification performance was assessed using sensitivity, specificity, positive predictive value, negative predictive value, and accuracy.

**Results:** A total of 1,193 pediatric upper endoscopy procedures were identified over the 6‐month study period. Manual case review by trained clinicians identified 138 new pediatric EoE cases, representing 11.5% of all upper endoscopies with esophageal biopsy. Both the number of endoscopies and new pediatric EoE diagnoses were consistent with previously published epidemiology trends for this region. Iterative application of SQL‐based code search ultimately yielded high sensitivity and accuracy, though there were 6 false positives and 15 false negatives. The AI model was superior to the SQL‐based code, with specificity, sensitivity and accuracy all above 99% and zero false negatives.

**Conclusion:** Both the SQL and AI‐based tools demonstrate strong potential to efficiently and accurately identify new pediatric EoE diagnoses across large health care systems with an enterprise‐wide data warehouse. These automated methods significantly reduce time required to perform manual chart review and may allow for collaboration across centers to improve estimates of disease incidence and prevalence at the state and nationwide level. The AI model was able to consistently identify eosinophil count patterns in unstructured pathology text, outperforming the SQL approach in sensitivity, while maintaining high specificity. The main issue with both the SQL and AI models were that they falsely identified new EoE diagnoses in pediatric patients that should have been excluded due to presence of another condition such as candidal esophagitis, Crohn disease or lack of esophageal symptoms. Overall, the excellent performance of these models is very promising. Quickly and correctly characterizing pediatric EoE epidemiology at the population‐level can support advocacy for appropriate staffing for clinical care, community education resources, and research funding allocation. Identifying cases at the population‐level can also allow for deeper analysis of exposures (particularly environmental) and other risk factors contributing to EoE disease development. Next steps are to ensure these types of searches are reliable and reproducible across other healthcare systems to aid in collaboration across centers and better identify true pediatric EoE incidence and prevalence at the population level.


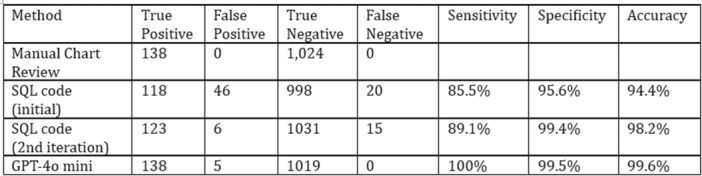


## 703 CLINICAL AND THERAPEUTIC COMPARISON OF EOSINOPHILIC ESOPHAGITIS IN PEDIATRIC AND ADULT POPULATIONS IN COLOMBIA: A MULTICENTER CROSS‐SECTIONAL STUDY

*MICHELLE HIGUERA* ^*12,13*^, *FABIAN JULIAO‐BAÑOS* ^*5*^, *NATALI GONZALEZ ROZO* ^*2*^, *WILSON DAZA* ^*3*^, *AILIM CARIAS* ^*1,18*^, *JOSE VERA* ^*1*^, *JUAN PABLO RIVEROS LOPEZ* ^*4*^, *CATALINA ORTIZ PIEDRAHITA* ^*5*^, *CARLOS AUGUSTO CUADROS MENDOZA* ^*6*^, *STEPHANIA PEÑA HERNANDEZ* ^*7*^, *CESAR AUGUSTO MORENO SERRANO* ^*8*^, *OTTO GERARDO CALDERON GUERRERO* ^*9*^, *FERNANDO ALONSO MEDINA MONROY* ^*10*^, *ADRIANA PRADA REY* ^*11*^, *JUANITA HIGUERA* ^*13*^, *MONICA MARIA CONTRERAS RAMIREZ* ^*5*^, *CARLOS TIMOSSI* ^*26*^, *Pablo Vasquez* ^*14*^, *Jhon Camacho‐Cruz* ^*14*^, *Adán Lúquez‐Mindiola* ^*15*^, *Alvaro Gómez‐Venegas* ^*5,16*^, *Viviana Parra‐Izquierdo* ^*6*^, *Jhon Carvajal‐Gutiérrez* ^*5*^, *Brenda Arturo‐Arias* ^*17*^, *Pedro Aponte‐Ordoñez* ^*15*^, *Viviana Parra‐Vargas Parra‐Vargas* ^*19*^, *Jerónimo Toro‐Calle* ^*20*^, *Robin Prieto‐Ortiz* ^*21*^, *Rafael Carmona‐Valle* ^*22*^, *Alejandra Castro‐Rodríguez* ^*23*^, *Fabio Gil‐Parada* ^*24*^, *Jhonny Castaño‐Morales* ^*25*^, *Jorge Donado‐Gómez* ^*5*^, *William Otero‐Regino* ^*6*^

^*1*^ *gastroenterology*, *Fundacion Santa Fe de Bogota*, *Bogotá*, *Bogota*, *Colombia*; ^*2*^ *Hospital Universitario Erasmo Meoz Cucuta*, *Cúcuta*, *North Santander*, *Colombia*; ^*3*^ *UNIDAD DE GASTROENTEROLOGIA PEDIATRICA Y NUTRICION GASTRONUTRIPED*, *BOGOTA*, *Colombia*; ^*4*^ *Colegio Colombiano de Gastroenterologia, Hepatologia y Nutricion Pediatrica*, *BOGOTA*, *Colombia*; ^*5*^ *Hospital Pablo Tobon Uribe*, *Medellín*, *Antioquia*, *Colombia*; ^*6*^ *Hospital Internacional de Colombia*, *Bucaramanga*, *Santander Department*, *Colombia*; ^*7*^ *Fundacion Hospital de la Misericordia*, *Bogota*, *Colombia*; ^*8*^ *Hospital Departamental de Villavicencio ESE*, *Villavicencio*, *Meta*, *Colombia*; ^*9*^ *Clinica Imbanaco*, *Cali*, *Valle del Cauca*, *Colombia*; ^*10*^ *Centro Medico UGANEP*, *Bucaramanga*, *Colombia*; ^*11*^ *Clinica del Country*, *Bogotá*, *Bogota*, *Colombia*; ^*12*^ *PEDIATRIA*, *Universidad Nacional de Colombia*, *Bogotá*, *Bogota*, *Colombia*; ^*13*^ *PEDIATRIA*, *Universidad El Bosque Facultad de Medicina*, *Bogotá*, *Bogota*, *Colombia*; ^*14*^ *pediatria*, *Fundacion Universitaria de Ciencias de la Salud*, *Bogotá*, *Bogota*, *Colombia*; ^*15*^ *gastroenterólogia*, *instituto de salud digestiva gut medical*, *Bogota*, *Colombia*; ^*16*^ *gastroenterólogia*, *Universidad de Antioquia*, *Medellín*, *Antioquia*, *Colombia*; ^*17*^ *gastroenterologia*, *SES Hospital Universitario de Caldas*, *Manizales*, *Caldas*, *Colombia*; ^*18*^ *PEDIATRIA*, *Universidad de los Andes*, *Bogotá*, *Bogota*, *Colombia*; ^*19*^ *gastroenterólogia*, *Clinica Colsanitas SA*, *Bogotá*, *Bogota*, *Colombia*; ^*20*^ *GASTROENTEROLOGIA*, *Clinica CES*, *BOGOTA*, *Colombia*; ^*21*^ *GASTROENTEROLOGIA*, *Centro de Enfermedades Hepáticas y Digestivas CEHYD*, *BOGOTA*, *Colombia*; ^*22*^ *Clinica Medihelp Services Cartagena*, *Cartagena*, *Bolívar*, *Colombia*; ^*23*^ *GASTROENTEROLOGIA*, *Gastroplus*, *BOGOTA*, *Colombia*; ^*24*^ *GASTROENTEROLOGIA*, *CLINICA UNIVERSITARIA COLOMBIA*, *BOGOTA*, *Colombia*; ^*25*^ *Clinica Medellin*, *Medellín*, *Antioquia*, *Colombia*; ^*26*^ *Research and development Miramar*, *DF*, *Mexico*

**Introduction:** Eosinophilic esophagitis (EoE) is a chronic, progressive inflammatory disease with increasing global prevalence and incidence, affecting both pediatric and adult populations.

**Objective:** To compare the epidemiological, clinical, and therapeutic characteristics of two multicenter Colombian cohorts comprising pediatric and adult patients diagnosed with EoE.

**Materials and Methods:** A descriptive, cross‐sectional comparative study was conducted using two independent cohorts of pediatric and adult patients from multiple Colombian centers. Data were consolidated into an electronic database, evaluating demographic variables, clinical features, treatment modalities, and complications.

**Results:** A total of 286 EoE patients were analyzed, including 143 children and 143 adults, with a male predominance (61.9%). Cesarean delivery (50.3% vs. 5.6%, P < 0.001), atopic conditions (27.3% vs. 11.9%, P < 0.001), allergic rhinitis (34.3% vs. 11.9%, P < 0.001), and family history (4.9% vs. 0.7%, P < 0.001) were more prevalent among children. Symptom duration prior to diagnosis differed significantly (P = 0.003), with adults seeking medical attention earlier (<6 months: 42.0% vs. 22.8%). Abdominal pain and vomiting were more common in children, whereas adults exhibited higher rates of dysphagia (69.9% vs. 32.2%, P < 0.001), food impaction (21.0% vs. 2.8%, P < 0.001), and weight loss (25.2% vs. 4.2%, P < 0.001). Regarding treatment, adults were more frequently prescribed corticosteroids (29.4% vs. 0.0%, P < 0.001) and less frequently proton pump inhibitors (72.7% vs. 88.1%, P < 0.001) compared to children. No significant difference was observed in dietary therapy (41.3% vs. 43.4%, P = 0.35). Table 1:

**Conclusions:** EoE in Colombia affects both pediatric and adult populations, with notable differences in clinical history, symptomatology, treatment approaches, and time to diagnosis. These findings underscore the need for national guidelines and tailored educational strategies for each demographic group.


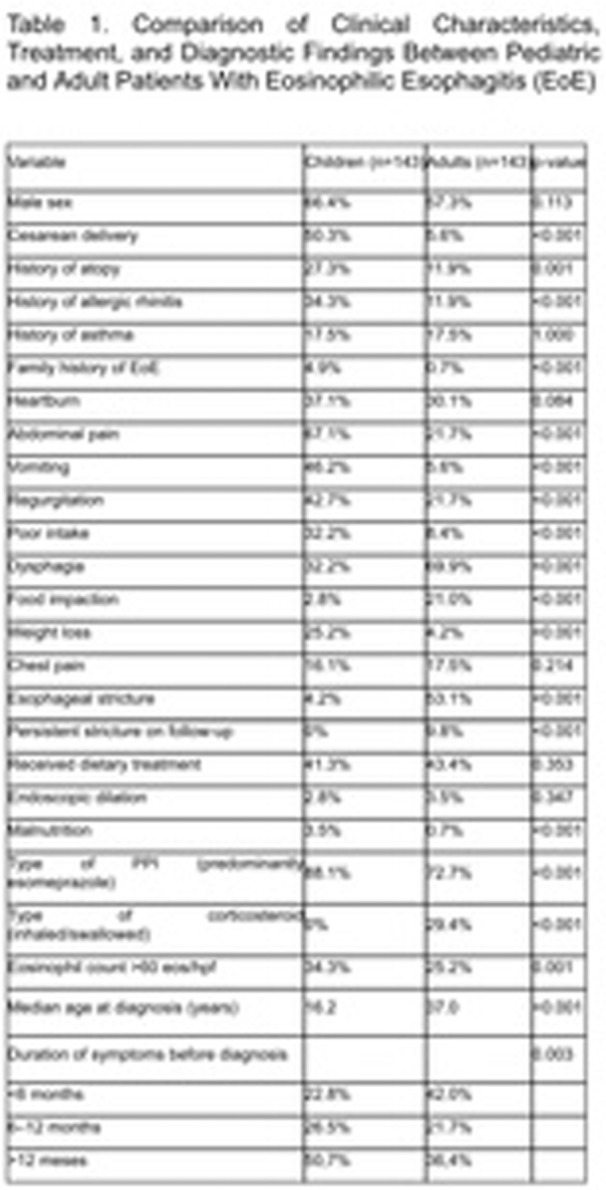


## 704 BONE MINERAL DENSITY CHALLENGES IN PEDIATRIC AND YOUNG ADULTS WITH EOSINOPHILIC ESOPHAGITIS: A CLOSER LOOK AT RISKS AND INSIGHTS

*Shreeya Chugh* ^*1*^, *Uzzam Khawaja* ^*2*^, *Barrett Barnes* ^*1*^, *Emily McGowan* ^*3*^, *Madhusmita Misra* ^*2,4*^

^*1*^ *Pediatric Gastroenterology*, *University of Virginia*, *Charlottesville*, *VA*; ^*2*^ *Pediatrics*, *University of Virginia*, *Charlottesville*, *VA*; ^*3*^ *Allergy/Immunology*, *University of Virginia*, *Charlottesville*, *VA*; ^*4*^ *Pediatric Endocrinology*, *University of Virginia*, *Charlottesville*, *VA*

**Background:** Eosinophilic esophagitis (EoE) is a chronic, immune‐mediated esophageal disease characterized by eosinophil‐predominant inflammation (≥15 eosinophils per high‐power field) and esophageal dysfunction. It frequently coexists with atopic conditions, and has a male predominance. Chronic inflammation is known to impact bone health deleteriously in other conditions. The goal of this study was to evaluate the impact of EoE on bone mineral density (BMD) in pediatric and young adult patients, and to identify key risk factors associated with low BMD.

**Methods:**This study was approved by our Institutional Review Board (IRB). A retrospective chart review was conducted for 123 patients with EoE, aged 5–25 years (98 males, 25 females), who underwent dual‐energy X‐ray absorptiometry (DXA) for BMD assessment. 103 patients were younger than 18 years, and 20 were 18 years or older. Data were collected from electronic medical records spanning January 2020, to January 2025 (60 months). Data management was performed using REDCap, and statistical analysis using JMP Pro 18.0.2.

**Results:** Patients with EoE had a median age of 11.3 years (IQR: 9–16) and median disease duration of 47 months (IQR: 20–77). The median BMI was 19.0 (IQR: 17–22) kg/m2, and BMI z‐score (BMIz) 0.48 (IQR: ‐0.54 to 1.34).Hip BMD Z‐scores (n=76) had a median of ‐0.67 (IQR: ‐1.59 to 0.26), with 37% below ‐1 SD and 16% below ‐2 SD. Spine BMD Z‐scores (n=105) had a median of ‐0.48 (IQR: ‐1.16 to 0.38), with 30% below ‐1 SD and 12% below ‐2 SD. Total body less head BMD Z‐scores (n=44) had a median of ‐0.82 (IQR: ‐1.69 to 0.25), with 45% below ‐1 SD and 21% below ‐2 SD.In a multivariate model that included age, sex, BMIz, duration since diagnosis, and whether or not EoE had remitted, associations were identified of (i) hip BMD Z‐scores with BMIz (p = 0.016), sex (p = 0.004) and age (p=0.004), (ii) spine BMD Z‐scores with BMIz (p<0.0001), and (iii) total bod y less head BMD Z‐scores with BMIz (p=0.004). 25OHD levels were not associated with BMD Z‐scores.Males had lower BMD Z‐scores than females at both the hip (p = 0.001) and spine (p = 0.021), underscoring sex‐based differences in bone health. A larger proportion of males vs. females had a diagnosis of autism (18.4% vs. 4.0%, p=0.045), a known risk factor for low BMD, but a diagnosis of autism was not independently associated with BMD Z‐scores.

**Conclusions:** Patients with EoE exhibited a high prevalence of low BMD, particularly at the hip and spine, with significant sex‐based differences. BMIz was the strongest predictor of BMD across all sites, and males had lower BMD than females. These findings emphasize the need for early BMD screening and targeted interventions to mitigate long‐term skeletal risks in this population. Further research is warranted to explore the longitudinal impact of EoE‐related factors on bone health and to develop preventive strategies.


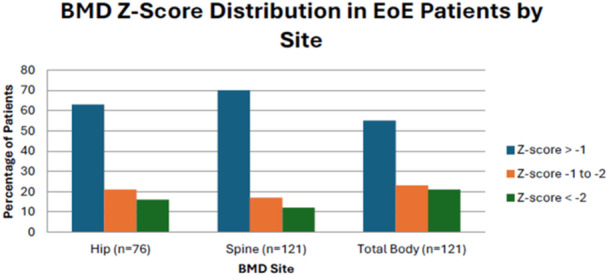


BMD z‐score distribution across EoE patients by site


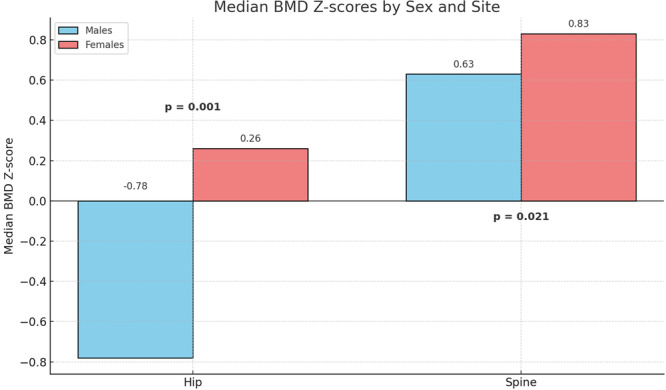


Median BMD z‐scores by sex and site

## 708 CHARACTERIZING PEDIATRIC PATIENTS WITH CONCURRENT CELIAC DISEASE AND EOSINOPHILIC ESOPHAGITIS: INSIGHTS INTO DIAGNOSIS AND MANAGEMENT

*Judy Jasser*, *Elizabeth Sinclair*

*Pediatrics*, *UPMC*, *Pittsburgh*, *PA*

**Background:** Eosinophilic esophagitis (EoE) and celiac disease (CD) are both immune‐mediated gastrointestinal disorders with overlapping clinical and histologic features. While a pathophysiologic relationship between the two conditions has been hypothesized, there are currently no clear guidelines on how to manage patients with both diagnoses.

**Methods:** We identified 21 pediatric patients with a dual diagnosis of EoE and CD. Demographics, family history, symptoms, diagnostics, treatments and outcomes were assessed including treatment with gluten‐free diet (GFD) alone and combination therapy.

**Results:** Demographic data is presented in Table 1. All 21 patients were of White race. The most commonly reported symptoms were abdominal pain and bloating. Diagnoses were synchronous in 71% of cases. In 23% of patients, EoE was diagnosed prior to CD, and in 5%, CD was diagnosed first. On initial diagnostic endoscopy, 57% of patients had abnormal esophageal findings. Histologic analysis revealed lamina propria fibrosis in 38% of patients at the time of EoE diagnosis. These patients were significantly more likely to have persistent histologic abnormality (71%) compared to those without fibrosis (30%). 95% of patients underwent repeat endoscopy. All patients were placed on a GFD following celiac diagnosis. Overall, 77% of patients received EoE treatment in addition to a GFD. 55% of patients reached EoE histologic remission. Table 2 details EoE histologic remission rates in response to GFD alone as well at GFD plus additional EoE therapy. Nearly all patients had repeated celiac serologic titers following diagnosis. Of these, 90% achieved serologic normalization. The mean time to serologic normalization was 17 months, and the median was 20 months. Two of the 20 patients had persistently elevated celiac titers; one of these achieved complete histologic remission of EoE, while the other had persistent eosinophilic inflammation.

**Conclusion:** In this pediatric cohort of patients with dual diagnosis of EoE and CD, only a minority responded to GFD as monotherapy. Additionally, a notable number presented with lamina propria fibrosis at EoE diagnosis and had a higher rate of histologic persistence of EoE, emphasizing the need to follow‐up EoE in patients with dual diagnosis. Due to overlap of symptoms and long‐term consequences of untreated CD and EoE, endoscopic evaluation should be considered for both disorders.


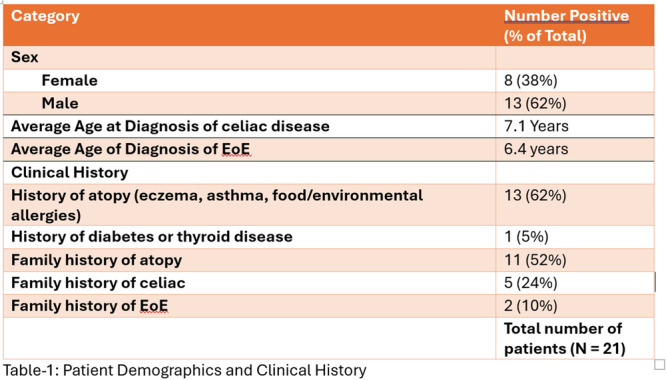



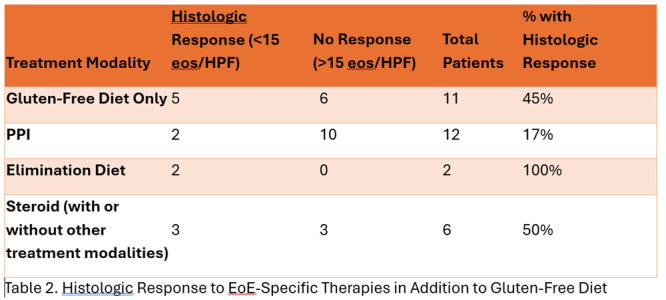


## 709 REEVALUATING BIOPSY FORCE IN EOE: INSIGHTS ON TREATMENT IMPACT AND ESOPHAGEAL REMODELING USING A DIGITAL FORCE GAUGE

*Kimera Joseph* ^*1,2*^, *Eric Pasman* ^*3,2*^, *Steve Min* ^*1,2*^

^*1*^ *Pediatrics*, *Walter Reed National Military Medical Center*, *Bethesda*, *MD*; ^*2*^ *Pediatrics*, *Uniformed Services University of the Health Sciences*, *Bethesda*, *MD*; ^*3*^ *Pediatrics*, *Naval Medical Center San Diego*, *San Diego*, *CA*

**Background:** We recently published a study describing a safe and reliable novel technique using a digital force gauge during routine esophageal biopsies to quantify the “tug” or “pull” sign appreciated by endoscopist in patients with eosinophilic esophagitis (EoE). We found that patients with EoE required significantly increased peak tug forces to biopsy their esophageal mucosa when compared to non‐EoE patients (20 N vs 15 N). Our goal was to further describe the tug forces required to obtain biopsies in patients with EoE, specifically whether treatment outcomes and endoscopic variations affect those tug forces.

**Methods:** Data was collected from an EoE Registry database maintained through an approved IRB at the Walter Reed National Military Medical Center. Only patients with biopsy confirmed EoE are enrolled in the registry. Data includes demographics, presence of atopic conditions, Eosinophilic Esophagitis Endoscopic Reference Score (EREFS), Newton force measurements of up to 6 esophageal biopsies, and pathology reports for total eosinophils per high power field (eos/hpf) and presence of fibrosis. Data obtained from the EoE subjects from our original tug sign study was included as well. Patients with diagnosed connective tissue disorders, inflammatory bowel disease, or tracheoesophageal fistula were excluded. We used the Kruskal‐Wallis rank sum test to compare biopsy forces.

**Results:** 38 pediatric patients aged 1‐18 years were enrolled. Patients with known EoE on any treatment (N=20) compared to no treatment (N=18) had no statistical difference in the peak force required for biopsies [median (IQR) 18.6 N, (14.35‐22.85) vs 20.7 N, (8.6‐32.8), p=0.226]. There was no statistically significant difference in force required for biopsy on a specific treatment modality (Figure 1). There was a moderate positive correlation between EREFS and peak forces (R=0.6, p=0.00012). For patients who had multiple EGDs with tug measurements, the peak force following each treatment showed no difference.

**Conclusions:** While our original study demonstrated that higher forces were required to obtain biopsies in EoE vs non‐EoE patients, here, we found that among EoE patients there were no significant differences in peak tug forces required for biopsies following treatment, regardless of outcome or therapeutic modality. Our study confirmed a positive association of higher EREFS with higher biopsy force measurements, which may indicate persistent esophageal remodeling despite adequate treatment. Continued enrollment of EoE patients with evaluation including tensile force measurements may provide additional insight on the impact of various treatment modalities and long‐term prognosis in EoE.


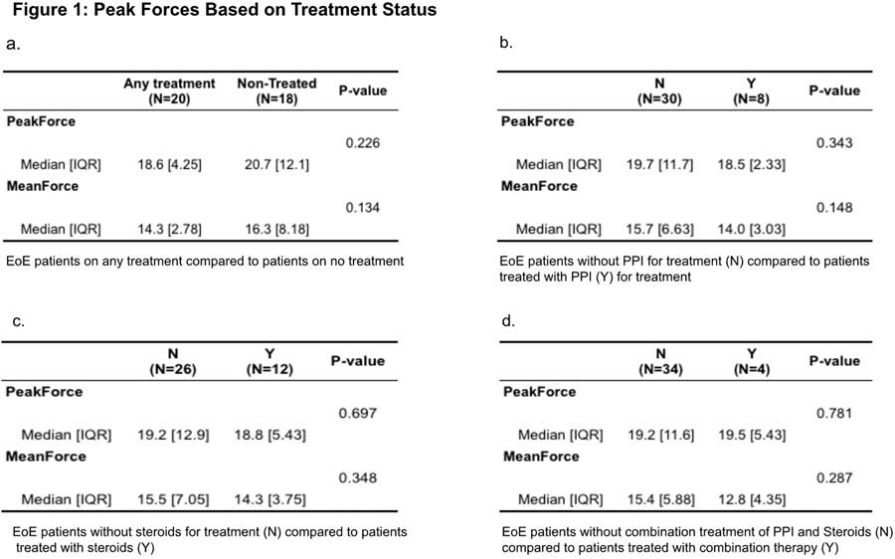



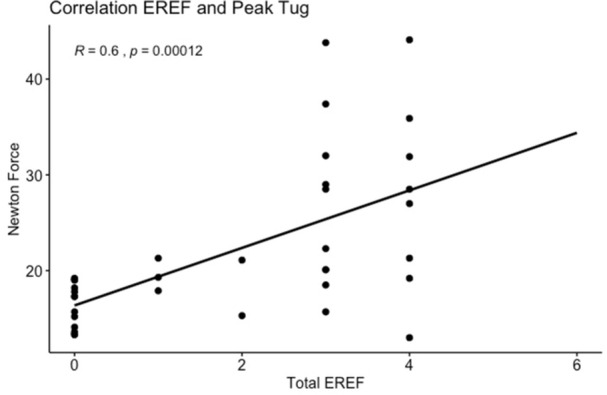


## 710 CHARACTERISTICS OF PEDIATRIC PATIENTS PRESCRIBED DUPILUMAB FOR EOSINOPHILIC ESOPHAGITIS AT A SINGLE ACADEMIC MEDICAL CENTER

*Amanda Kibbons* ^*1*^, *Charlotte Haskell* ^*2*^, *Nicolas Gargurevich* ^*3*^, *Autumn Zuckerman* ^*1*^, *Leena Choi* ^*3*^, *Katie Cruchelow* ^*1*^

^*1*^ *Specialty Pharmacy*, *Vanderbilt University Medical Center*, *Nashville*, *TN*; ^*2*^ *Lipscomb University College of Pharmacy and Health Sciences*, *Nashville*, *TN*; ^*3*^ *Vanderbilt University Medical Center*, *Nashville*, *TN*

**BACKGROUND:** Dupilumab improves symptoms and induces histologic remission in pediatric patients with eosinophilic esophagitis (EoE), but its role in EoE treatment is undefined by established guidelines. With limited guidance, insurers have varying criteria for dupilumab's place in therapy leading to insurance delays or denials. This study evaluated baseline symptoms and disease characteristics in pediatric patients prescribed dupilumab for EoE and factors affecting time to insurance approval.

**METHODS:** Single‐center retrospective cohort study of pediatric patients prescribed dupilumab for EoE from a gastroenterology clinic in the Southeast US from February 1, 2023 to August 1, 2024. Patients >18 years or who never started dupilumab were excluded. The primary outcome was time from referral to insurance approval. Secondary outcomes were EoE characteristics at baseline, concomitant medication changes post‐initiation, and reasons for insurance denial. Baseline characteristics were reported descriptively. Wilcoxon Rank Sum test compared time from referral to final insurance approval between patients with versus without insurance denial. A multivariable logistic regression evaluated the relationship between insurance denial and dysphagia controlling for insurance type, weight z‐score, and PPI or steroid use at baseline. A Cox proportional hazards model evaluated factors potentially associated with time to approval, including dysphagia, insurance type, weight z‐score, and PPI or steroid use at baseline.

**RESULTS:** In 117 patients included, the median age was 13 (Interquartile Range [IQR] 10, 16) years, with 77% Male, and 81% White. Most patients had commercial insurance (48%) or Medicaid (44%). The most common signs and symptoms reported at baseline were dysphagia (50%), stomach pain (25%), and compensatory mechanisms (18%). Dupilumab was prescribed a median of 3 (IQR 1.1, 5) years after diagnosis, mostly due to failure of PPI and steroid (53%). Before starting dupilumab, 68% of patients were prescribed PPI, 66% steroids, 12% no concomitant medications, and 5% dupilumab for a condition other than EoE. After starting dupilumab, of patients taking PPI or steroids at baseline (n=80), 26% discontinued PPI and 55% discontinued steroid. Initially, 35% of patients were denied dupilumab by insurance, most commonly due to patients being below FDA‐approved age‐weight (44%). Ultimately all patients were approved after appeal. Patients initially denied dupilumab received approval for medication access in a median of 22 (IQR 17, 29) days, compared to a median 2 (IQR 1,5) days p<0.001) for patients initially approved dupilumab. Patients with dysphagia at baseline were less likely to be denied (OR 0.45, p=0.047) and were significantly more likely to receive insurance approval sooner (OR 1.51, p=0.03).

**CONCLUSION:** Pediatric patients with EoE commonly have symptoms other than dysphagia at baseline and limited or no response to previous therapies. Patients with EoE symptoms other than dysphagia have delayed access to dupilumab requiring an appeal for approval. Future studies should examine how prescribing practices and insurance approval criteria change over time as real‐world data emerges.


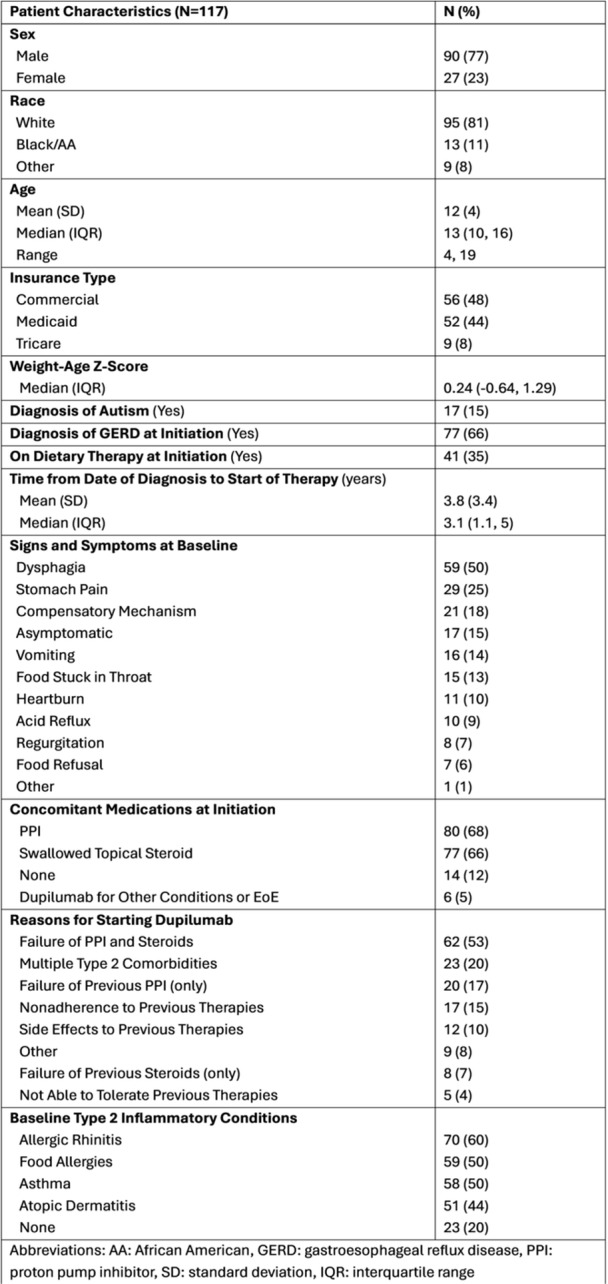


Patient Characteristics


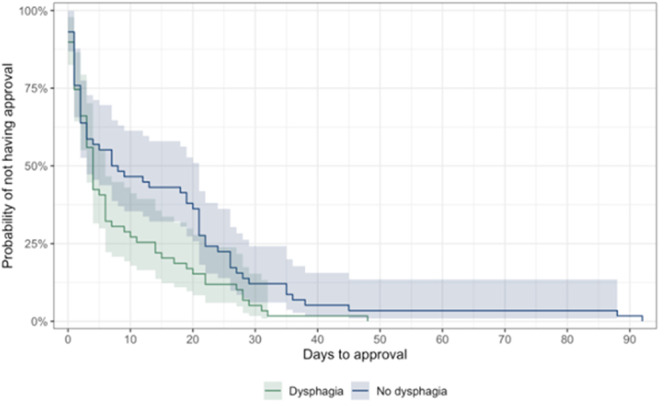


Days to insurance approval plotted over time in days for patients with dysphgia and patients without dysphagia

## 711 EVALUATION OF FACTORS ASSOCIATED WITH TREATMENT RESPONSE IN PEDIATRIC EOSINOPHILIC ESOPHAGITIS: A RETROSPECTIVE SINGLE CENTER STUDY

*Erica Levin* ^*1*^, *Gabrielle Ro* ^*2*^, *Melissa Weidner* ^*1*^

^*1*^ *Pediatrics*, *Rutgers Robert Wood Johnson Medical School*, *New Brunswick*, *NJ*; ^*2*^ *Rutgers Robert Wood Johnson Medical School*, *New Brunswick*, *NJ*

**Purpose:** Well‐established clinical phenotypes to guide treatment in eosinophilic esophagitis (EOE) do not exist. There is currently no data available regarding which treatment strategy has advantage as primary treatment for EOE. We performed a retrospective chart review to evaluate demographic, clinical, endoscopic, and histologic factors associated with treatment response in a cohort of pediatric patients with EOE within a single center.

**Methods:** Data was collected from patients diagnosed with EOE followed by the pediatric gastroenterology group at Rutgers‐ Robert Wood Johnson over 10 years (1/1/2014–12/31/2024), including results from the initial endoscopy and first follow‐up endoscopy after treatment initiation. Demographic, clinical, endoscopic, and histologic factors were recorded. Histologic remission (responders) was based on accepted definition (< 15 eosinophils per high power field (HPF)). Patients were stratified by treatment type. Histologic, endoscopic, clinical, and demographic features were compared between responders and non‐responders.

**Results:** 126 EOE patients were identified (74.0% male). Racial/ethnic distribution: 48.0% White, 15.7% Black or African American, 7.1% Asian, 29.9% Other; 85.8% were non‐Hispanic/Latino. There were no significant differences between treatment responders and non‐responders in sex, age, race, or ethnicity (Table 1). Following initial endoscopy, 92.4% started a proton pump inhibitor (PPI), 16.9% dietary restriction, 9.3% swallowed topical corticosteroids (STC), and 3.4% dupilumab. On follow‐up endoscopy, 48.4% (n = 61) achieved histologic remission. Response rates were 65% with dietary restriction (13/20), 48.5% with PPI (52/107), 40% with STC (4/10), and 100% with dupilumab (4/4). Eosinophilic Esophagitis Endoscopic Reference Scores (EREFS) were significantly higher in non‐responders at both baseline (p = 0.0002) and follow‐up (p < 0.0001) endoscopy. Overall, follow‐up endoscopy showed significantly lower peak eosinophils (p < 0.0001) and EREFS scores (p = 0.0003). Patients with baseline EREFS score ≤ 2 were more likely to achieve histologic remission (Figure 1) and had significantly decreased peak eosinophils on follow‐up endoscopy (EREFS = 0, p = 0.000153; EREFS = 1, p = 0.000011; EREFS = 2, p = 001077).

**Conclusion:** Currently, EOE treatment is individualized but not targeted based on phenotypes or other clinical data. Treatment algorithms exist, but response can be 50% or less. Therefore, treatment often requires adjustment with repeat endoscopy to evaluate response. Although PPI is a convenient and relatively well‐tolerated treatment, response rates of roughly 50% question its use as first line. In our cohort, remission rate of STC was lower than prior studies, although dose of STC and compliance could have contributed to the relatively low remission rate noted in the STC group. Challenges to compliance to both medication and diet modification must be considered. Further studies evaluating factors associated with histologic remission, including EREFS score, are needed to better guide initial treatment and reduce treatment‐related burden on patients and their families.


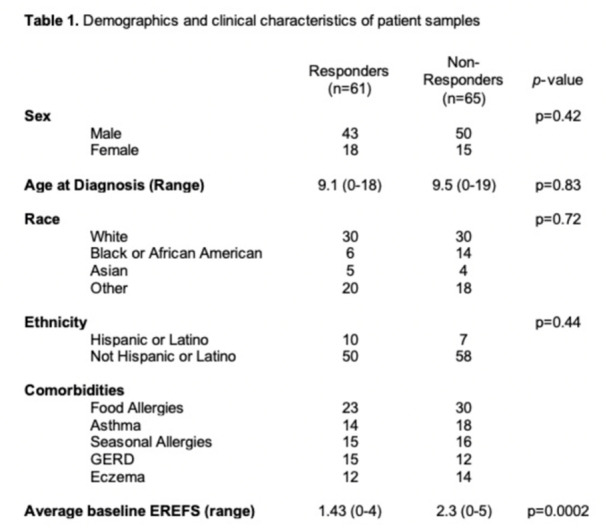



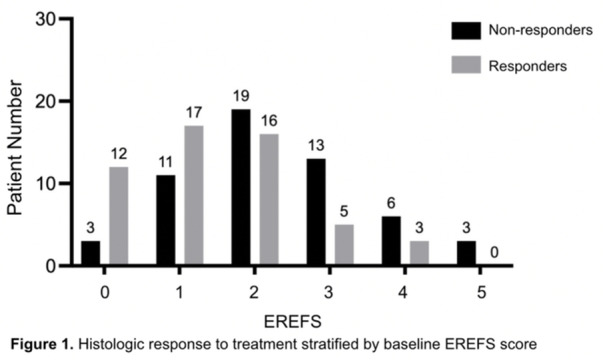


Histologic response defined as < 15 eosinophils per high power field (HPF) on follow‐up endoscopy.

## 712 EVALUATING TRENDS IN PEDIATRIC EOSINOPHILIC ESOPHAGITIS ADMISSIONS: A MULTICENTER RETROSPECTIVE COHORT STUDY

*Sarah Masten* ^*2*^, *John Morrison* ^*3,2*^, *Racha Khalaf* ^*1*^

^*1*^ *Pediatric Gastroenterology, Hepatology and Nutrition*, *University of South Florida Morsani College of Medicine*, *Tampa*, *FL*; ^*2*^ *Pediatrics*, *Johns Hopkins All Children's Hospital*, *Saint Petersburg*, *FL*; ^*3*^ *Johns Hopkins University*, *Baltimore*, *MD*

**Objectives and Study:** The prevalence of eosinophilic esophagitis (EoE) is increasing, and existing studies of nationally representative, insurance‐payer‐based databases have reported a rise in charges related to EoE that outpace inflation. However, these studies are limited in that they are unable to evaluate encounter‐level data to accurately describe diagnostic, pharmacologic, and procedural resource utilization during admissions. Furthermore, studies to date have not described the broader hospitalization course (i.e. length of stay (LOS)) and return to care (i.e. return to the emergency department and hospital readmission) after an inpatient encounter related to EoE. While the majority of EoE care takes place in the outpatient setting, this study evaluates if the increased prevalence of EoE correlates to increased hospitalizations. This study aims to 1) identify trends in EoE‐related encounters at hospitals within the Pediatric Health Information System® (PHIS) registry, and 2) identify factors associated with hospital LOS.

**Methods:** We conducted a multicenter retrospective cohort study using the PHIS registry, an administrative dataset containing de‐identified information on clinical and health resource utilization from 48 children's hospitals. Patients 0‐21 years of age discharged between 1/1/2010‐12/31/2024 were eligible. Included hospitalizations for EoE were identified using ICD9/10 codes for EoE as a principal diagnosis or a secondary diagnosis for EoE with an accompanying principal diagnosis of a prominent symptom of EoE (ex: dysphagia). Our outcomes of interest included rates of EoE hospitalization per 1,000 total encounters at each institution and total hospital LOS in days. The association between hospital LOS for the first eligible encounter for each patient and various demographic and clinical predictors of interest were assessed using multivariate linear regression.

**Results:** 3,542 individuals admitted with EoE were included for study. Hospitalization rates per 1,000 encounters did not increase significantly from 2010‐2024 (Figure 1). Our cohort was 71% male with a median (IQR) age of 10 (5‐15) years. Only 26% of the cohort had comorbid chronic complex conditions. Overall, 56% of patients had a hospital LOS of 1 day. Patients were frequently prescribed systemic (41%) or topical/swallowed (32%) corticosteroids and proton pump inhibitors (57%). Individuals who had prolonged LOS of ≥2 days more often received swallowed steroids or PPIs. In our multivariate model, undergoing abdominal or chest radiography, and fluoroscopic studies were associated with an increased LOS (1.23 days, 95% CI 0.84‐1.61). From a procedural standpoint, while undergoing esophagogastroduodenoscopy (EGD) or colonoscopy was associated with prolonged LOS, (0.58 days, 95% CI 0.17‐1 days) and (0.97 days, 95% CI 0.35‐1.59 days), respectively, foreign body removal was associated with shorter LOS (–0.86 days, 95% CI –1.7, ‐0.06 days). Undergoing an esophageal dilation during admission was not associated with a significant change in LOS.

**Conclusions:** Despite a recognized rise in the prevalence of EoE over time, hospitalization rates per 1,000 encounters remained stable, suggesting that improvements in disease recognition and effective outpatient management strategies may be mitigating the need for inpatient care. We identified several factors with significant associations as predictors of LOS, such as administration of swallowed steroids and PPIs, and undergoing imaging studies and endoscopic procedures. Further investigation is needed to understand why these factors are associated with a change in LOS, as this may reflect efficacy of acute management and severity of illness.


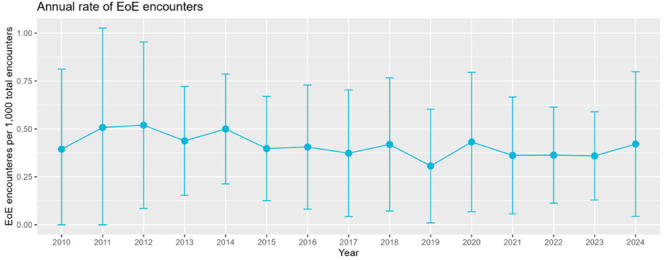


Figure. Annual rate of encounters for eosinophilic esophagitis per 1,000 total encounters. Error bars represent one standard deviation from the mean annual rate. Only hospitals reporting data during the entire study period were incorporated (N=36).

## 713 CORRELATION ANALYSIS OF PATIENT‐REPORTED OUTCOMES AND ENDOSCOPIC SEVERITY SCORES IN PEDIATRIC EOSINOPHILIC ESOPHAGITIS TREATED WITH DUPILUMAB VERSUS CONVENTIONAL THERAPIES

*Sukrita Mysore* ^*1*^, *Anupama Kewalramani* ^*2*^, *Jennifer Hong* ^*3*^

^*1*^ *Pediatric Gastroenterology*, *University System of Maryland*, *Baltimore*, *MD*; ^*2*^ *Pulmonology, Allergy and Immunology*, *University of Maryland Baltimore School of Medicine*, *Baltimore*, *MD*; ^*3*^ *Pediatric Gastroenterology*, *University of Maryland Baltimore School of Medicine*, *Baltimore*, *MD*

**Introduction:** Eosinophilic Esophagitis (EoE) is a chronic type 2 inflammatory disease of the esophagus. Treatment is more standardized with proton pump inhibitors (PPI), swallowed topical steroids, and recent FDA approval of Dupilumab. However, there remain gaps in literature regarding assessing treatment response. EoE is a multifaceted disease with clinical, endoscopic, and histologic markers of disease activity with both biologic and patient reported outcomes (PROs). Use of PROs is complicated by symptom variability in young children and adolescents. PROs have become increasingly important in the treatment of chronic disease, and Pediatric EoE symptom score (PEESS v2.0) is a validated tool to assess severity of symptoms as well as treatment response during disease monitoring. EoE Endoscopic Reference Score (EREFS) is a validated score in adults that assesses disease activity. EREFS in children is recommended to be used in combination with histological and clinical features to evaluate treatment response.

**Objective:** The objective of this pilot study is to evaluate the therapeutic response to dupilumab in comparison with other conventional treatments (proton pump inhibitors (PPIs), swallowed topical corticosteroids, and restricted diets) in pediatric patients with EoE. Additionally, the study aims to investigate the correlation between changes in patient‐reported symptom scores (PEESS v2.0) and endoscopic findings (EREFS) during treatment and follow‐up.

**Methods:** We recruited pediatric and adolescent patients aged 2‐20 years with a confirmed diagnosis of EoE (n=27) between February‐May 2025 receiving treatment in the Eosinophilic Gastrointestinal Disorders Multidisciplinary Clinic at University of Maryland Children's Hospital. Informed consent and, when appropriate, assent was obtained from all participants. Demographic data, including age, sex, and ethnicity, were collected for analysis. Symptom burden was assessed using the PEESS® v2.0, reported either by patients or parent proxies. A retrospective chart review was conducted to obtain corresponding EREFS and evaluate treatment‐related endoscopic responses.

Descriptive statistics were used to summarize demographic and clinical data. Continuous variables, including age, PEESS® v2.0 scores, and EREFS, were reported as means with range. PEESS® v2.0 score between the Dupilumab and non‐Dupilumab groups was conducted using Welch's t‐test. The correlation between PEESS® v2.0 and EREFS within each group was conducted using Pearsons's correlation coefficient. A p‐value of <0.05 was considered statistically significant.

**Results:** A total of 27 pediatric patients with eosinophilic esophagitis (EoE) were included in the study. The mean age of participants was 11.8 years (range: 3–18 years). The cohort had a nearly equal sex distribution, with 51.8% male (n = 14). Within the cohort, 16 (59%) patients were on dupilumab therapy and 11 patients receiving alternative conventional therapies: proton pump inhibitors (PPIs; 11%, 3/27), swallowed topical corticosteroids (15%, 4/27), no treatment (11%, 3/27), restricted diet (4%, 1/27).

The average PEESS® v2.0 symptom score was lower in the dupilumab group (mean: 15.6; range: 0–33.75) compared to the non‐Dupilumab group (mean: 29.55; range: 2.5–60), which and the difference was statistically significant (p=0.05). Mean EREFS were low in both groups, with an average of 0.28 (range: 0–3) in the dupilumab group and 0.37 (range: 0–1) in the comparator group, which was not statistically significant (p=0.75). The Pearson coefficient between PEESS and EREFS in the dupilumab group was – 0.48 (p=0.86) in the dupilumab group and + 0.375 (p=0.36) in the conventional therapy group.

**Discussion and Conclusion:** In this pilot study, pediatric patients diagnosed with EoE who received dupilumab demonstrated a lower symptom burden, as measured by PEESS® v2.0 scores, compared to those treated with alternative conventional therapies. Endoscopic severity, quantified using EREFS, remained consistently low across both treatment cohorts. These findings suggest a symptomatic benefit associated with dupilumab therapy which was statistically significant. However, no statistically significant correlation was observed between symptom scores and endoscopic findings in either cohort. Within the dupilumab‐treated cohort, a negative correlation between symptom scores and endoscopic severity was identified, suggesting that symptomatic improvement may precede or occur independently of endoscopic healing. Although these trends did not reach statistical significance in this pilot sample, a larger cohort may yield more definitive correlations. Further investigation incorporating histologic data will be essential to assess whether symptom and endoscopic scores align with underlying histologic disease activity. Collectively, these findings offer real‐world insight into therapeutic effects of dupilumab and conventional therapies in treatment of pediatric EoE.

## 716 NATURAL HISTORY AND NUTRITIONAL IMPACT OF PEDIATRIC EOE: A SINGLE CENTER PROSPECTIVE REGISTRY

*Aliza Solomon* ^*1*^, *Hana Flaxman* ^*2*^, *Cecilia Dalmau* ^*1*^, *Ayelet Goldberg* ^*1*^, *charlene thomas* ^*2*^, *jennifer lentine* ^*1*^

^*1*^ *Pediatric Gastroenterology Hepatology and Nutrition*, *Weill Cornell Medicine*, *New York*, *NY*; ^*2*^ *Weill Cornell Medicine*, *New York*, *NY*

**Background:** The natural history of eosinophilic esophagitis (EoE) in children remains poorly defined, with variable clinical presentations and uncertain long‐term outcomes. Emerging data suggest a potential association between EoE, restrictive eating behaviors, and micronutrient deficiencies; however, this relationship remains underexplored. Understanding disease trajectory and nutritional impact is critical to improving care and informing future guidelines.

**Objective:** To establish a single‐center pediatric EoE registry using prospective observational methods to evaluate the natural history of EoE and to investigate the prevalence and potential drivers of vitamin and trace element deficiencies in this population.

**Methods:** A prospective cohort enrolling from 2021–2031 of pediatric patients diagnosed with EoE (≥15 eosinophils/hpf). Data are collected at diagnosis, 3, 6, and 12 months post‐diagnosis, and annually thereafter. Variables include demographics, clinical symptoms, endoscopic findings, treatment history, dietary patterns, supplement use, and lab data.

Nutritional intake was assessed through Cronometer dietary app logs, and dietitian evaluations. Blood samples are analyzed for vitamin levels. All data are coded and stored securely in REDCap.

**Statistical Analysis: needs to be confirmed by Charlene:** To describe this patient cohort, continuous variables were summarized using the median and interquartile range (IQR), while categorical variables were reported as counts and percentage [n(%)]. Differences in demographics, endoscopic findings, and laboratory data between EoE patients and the control patients were assessed using the Wilcoxon rank sum test for continuous variables and the chi‐square test or Fisher's exact test for categorical variables, as appropriate. For this abstract, only data from the first time point were analyzed. All analyses were performed using R Version 4.2.3. A two‐sided p‐value < 0.05 was considered statistically significant. As this study was exploratory, p‐values were not corrected for multiple comparisons. A retrospective power indicated that with 17 EoE patients and 109 control patients, the study had 80% power to detect an effect size of 0.73.

**Results:** We enrolled 126 subjects (17 EoE and 109 controls). Demographics are noted in Table 1. Preliminary analysis on baseline characteristics was performed. Presenting complaints are listed in table 2.

Chronometer analysis was performed on available date showing no difference between EoE and controls with report of any nutrient intake except inadequate manganese intake in EoE 6/10 (60%) vs Control 7/33 (21.1%) p=0.044. There were no differences in folate, iron, B12 and Vitamin C levels between EoE and controls. Vitamin D was deficient or insufficient in 4/18 (22%) controls and 1/10 (10%) EoE subjects which was independent of intake.

**Conclusion:** This registry provides a structured framework for evaluating the longitudinal course of pediatric EoE and its nutritional consequences. Contrary to retrospective data, there was in vitamin levels between EoE and controls regarding blood levels or intake except manganese. However, data was incomplete for Vitamin D for the control group which limited analysis for significance. Further analysis on serial data points will be performed. Findings may inform clinical management strategies and guide future research on risk stratification and dietary interventions.


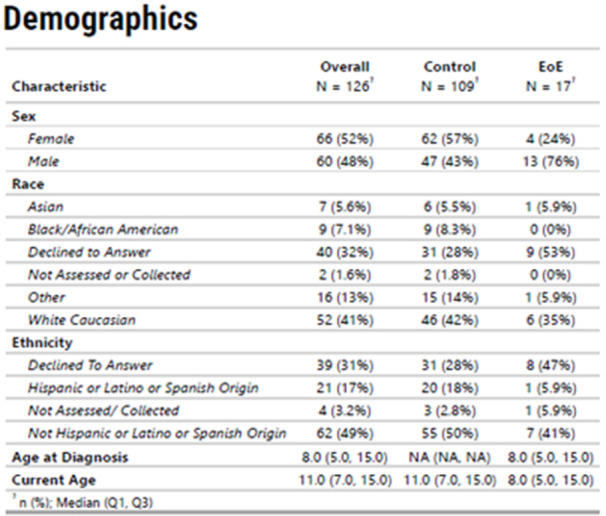



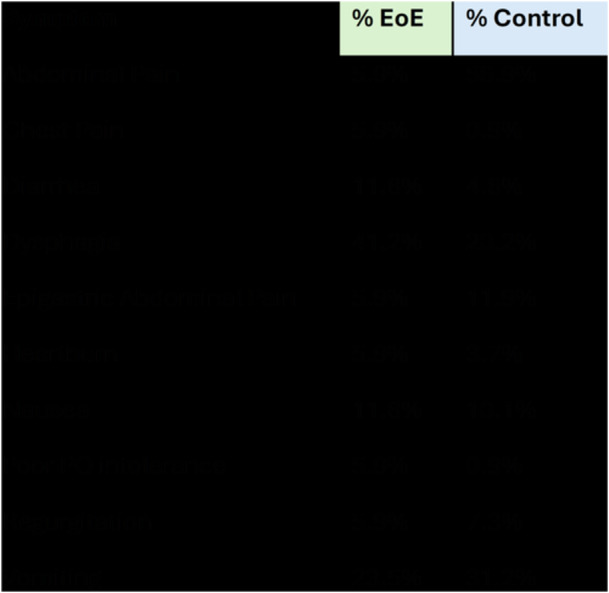


## 717 EXAMINING PREVALENCE OF EOSINOPHILIC ESOPHAGITIS IN CHILDREN WITH AUTISM SPECTRUM DISORDER WHO ARE EVALUATED FOR INTENSIVE FEEDING THERAPY: A SINGLE CENTER EXPERIENCE

*Joanne Thio*, *Jessica Martin*, *Sharef Al‐Mulaabed*, *Justin de Boer*

*Pediatric Gastroenterology*, *Orlando Health*, *Orlando*, *FL*

**Background:** Eosinophilic esophagitis (EoE) is a chronic immune mediated disease of the esophagus and is one of the leading causes of dysphagia in children. It is estimated that EoE affects approximately 1/1000 people in the US. It has been reported that in children with autism spectrum disorder (ASD) the prevalence of EoE is higher. Children with ASD have a higher rate of feeding difficulties and are often referred to feeding therapy. Their feeding difficulties may be attributed to behavioral issues however, feeding difficulties may be the presenting symptom of EoE in children with ASD. We aim to see the prevalence of EoE in children with ASD compared to children without ASD who are evaluated for feeding therapy at our Pediatric Feeding Difficulties Center (FDC).

**Methods:** We conducted a retrospective review of children who were evaluated for intensive feeding therapy between January 2023 and September 2024 at Pediatric Feeding Difficulties Center in Orlando, Fl. Children who had undergone esophagogastroduodenoscopy (EGD) evaluation were included. Data included demographics, medical history, and EGD findings.

**Results:** The cohort contained 254 patients, of which 69.7% (177/254) were male with a mean age of 4.43 (SD 2.91) years. Overall, 45.7% (116/254) of patients had an autism diagnosis when first evaluated in the program. Of the EGD outcomes, 56.3% (143/254) were normal, 31.5% (80/254) had reflux, and 12.2% (31/254) showed EOE. For patients with ASD, 53.4% (62/116) had normal EGD findings, 35.3% (41/116) had reflux, and 11.2% (13/116) had EOE while for non‐ASD patients, 58.7% (81/138) had normal findings, 28.3% (39/138) had reflux, and 13% (18/138) had EOE. There was no statistical association between ASD diagnosis and EGD outcome (χ^2^=1.4866, df=2, p=0.4755).

**Conclusion:** In our cohort of children undergoing evaluation for intensive feeding therapy, we did not find children with ASD to have a higher prevalence of EoE compared to children without ASD. This study did however show that in this cohort of children with ASD, they have similar rates of upper GI conditions compared to the patients without ASD and feeding disorders. GI conditions are common in children with feeding difficulties and should be evaluated.


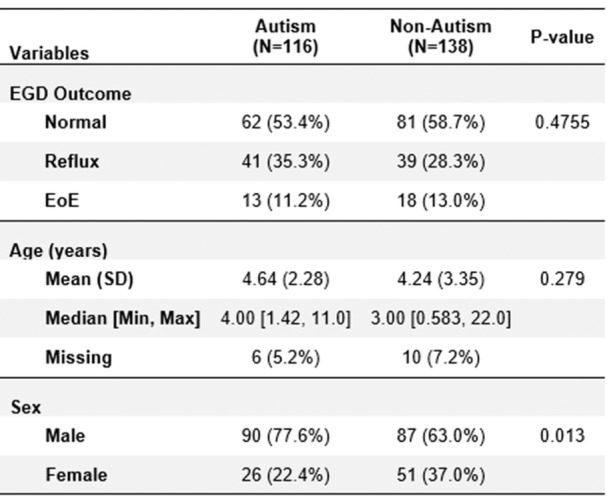


Table 1. Comparison of demographic and endoscopic finding between autism and non‐autism patients.

## 719 ASSESSMENT OF BONE HEALTH AND GROWTH IN PEDIATRIC INFLAMMATORY BOWEL DISEASE (IBD) PATIENTS: A SINGLE CENTER RETROSPECTIVE CHART REVIEW

*Justin Baba*, *Sharef Al‐Mulaabed*, *Jeffrey Bornstein*, *Vijay Mehta*, *Akash Pandey*

*Pediatric Gastroenterology*, *Orlando Health Arnold Palmer Hospital for Children*, *Orlando*, *FL*

**Background:** Inflammatory bowel disease (IBD) is a chronic inflammatory condition of the gastrointestinal tract, encompassing Crohn's Disease (CD), Ulcerative Colitis (UC), and Indeterminate Colitis (IC), with a rising incidence in the pediatric population globally. Children with IBD often face challenges such as malnutrition, growth failure, and altered body composition due to persistent intestinal inflammation, increased metabolic demands, and reduced nutrient absorption. Malnutrition during critical growth periods can lead to long‐term consequences, including delayed puberty, reduced bone mineral density, and impaired muscle development. Therefore, optimizing nutritional therapy is essential for improving clinical outcomes and supporting normal growth in pediatric IBD patients.

Studies have shown that patients with IBD have high prevalence of low bone mineral density, however data is limited in the pediatric population. Important factors that may be associated with worsened bone health status include low body mass index, small bowel involvement of disease, anemia, hypocalcemia, and immunosuppressive therapies.

This study aims to review electronic medical records to assess bone mineral density in patients aged 5–21 years with IBD. By examining dual‐energy X‐ray (DEXA) scan results alongside laboratory markers and growth data, we aim to understand how IBD, its treatments, and nutritional factors affect bone health. By data analysis, we aim to identify high‐risk patients and guide early interventions. Better screening and personalized care could help prevent long‐term bone problems and improve outcomes for kids with IBD.

**Methods:** We retrospectively reviewed data from all patients with IBD who underwent DEXA scan between January 1, 2010 to April 1, 2025. A Z‐score between ‐1 and ‐2.5 was identified as osteopenia and a Z‐score ‐2.5 or lower was identified as osteoporosis as per standardized guidelines. Bone mineral density (BMD) measurements at the lumbar spine (average of L1‐L4) with their Z‐scores were retrieved, along with demographic and clinical characteristics and therapies for IBD.

**Results:** A total of 102 patients were included in the study, of whom 64 (63%) were male and 38 were female (37%). The mean age at the time of DEXA scan was 13.8 years with a standard deviation (SD) of 3.2 years. Among the cohort, 70 patients (69%) had CD, 28 (27%) had UC, and 4 (4%) were diagnosed with IC.

The mean Z‐score for L1–L4 bone mineral density (BMD) was ‐0.248 (SD 1.386). BMD results were classified as normal in 62 patients (60.8%), osteopenia in 36 patients (35.2%), and osteoporosis in 4 patients (4%).

There was a statistically significant positive correlation between BMD Z‐scores and weight Z‐scores (r = 0.490, p < 0.001), height Z‐scores (r = 0.298, p = 0.003), and BMI Z‐scores (r = 0.403, p < 0.001). Additionally, a significant negative correlation was observed between BMD Z‐scores and platelet count (PLT) (r = ‐0.224, p = 0.024). A trend toward a positive correlation with hemoglobin (r = 0.173, p = 0.082) was noted, although it did not reach statistical significance. No significant correlations were found between BMD Z‐scores and erythrocyte sedimentation rate (ESR), albumin, total protein, hemoglobin (Hgb), or hematocrit (Hct).

There was no significant difference in BMD Z‐scores between patients with CD and those with UC (p = 0.425). Although a higher percentage of patients had abnormal BMD Z‐scores in CD (41.9%) when compared to those with UC (32.1%), this was not statistically significant. Similarly, no significant differences were observed between patients in clinical remission (p = 0.940) or those in both clinical and histological remission (p = 0.525).

**Conclusions:** In our single center retrospective study, almost 40% of patients had an abnormal BMD. BMD Z‐scores positively correlated with Z‐scores for weight, height, and BMI, suggesting that nutritional status may play a key role in bone health of those patients. Low BMD may be another indicator of poor nutrition due to poor absorption and altered metabolic state associated with inflammation. Larger studies with attention to disease activity status, length of symptoms, and reassessment of bone density over time in relation to disease state may better delineate response of BMD Z‐scores with treatment of IBD.

## 720 IMPROVING TIME TO MR‐ENTEROGRAPHY IN PATIENTS NEWLY DIAGNOSED WITH INFLAMMATORY BOWEL DISEASE

*Serina Beydoun*, *Kristen Cares*

*Pediatric Gastroenterology*, *Children's Hospital of Michigan*, *Detroit*, *MI*

**Background:** Magnetic resonance enterography (MRE) is a radiological technique that uses magnetic resonance imaging (MRI) to assess the small bowel, following distension with an oral contrast agent. It's main indication is to evaluate small bowel involvement in patients with Crohn's disease (CD) and undefined inflammatory bowel disease (IBD), as well as to assess complicatiosn and help risk‐stratify disease severity. One of its main advantages is that it involves no ionizing radiation and provides high contrast resolution allowing for detailed evaluation of bowel wall changes. Additionally, it allows comparable evaluation to colonoscopy for evaluation of known or suspected Crohn's disease and is noninvasive.

A lot of research has been done on the use of MRE in regards to its use compared to different imaging modalities and its ability to be used to distinguish between penetrating, inflammatory, and stricturing disease. In fact, many institutions now request and/or obtain MRE prior to diagnosis and treatment of CD. At our institution, an MRE is commonly requested while the patient is admitted or shortly after diagnosis is made via endoscopy and prior to beginning treatment. However, due to scheduling and financial conflicts, it is often performed many weeks after initial diagnosis. While waiting for the MRE patients are routinely started on treatment which can include steroids and other medications that may potentially alter the findings on the MRE and thus inaccurately depict the severity of IBD and prognosis for the patient leading to a more severe course.

**Objectives:** This is a quality improvement study aiming to decrease the length of time between diagnosis and completion of MRE study at our institution.

**Study Design:** The PDSA cycle was utilized for the study design of this project.

**Plan:** A retrospective chart review of all patients diagnosed with IBD over the last 13 years at our institution was conducted. Patients included in the study were those diagnosed with IBD and had an MRE completed between January 1, 2018 and December 31, 2023. The total number of days between the date an MRE was ordered and the date it was completed was determined for each patient. The average number of days from MRE order and completion was calculated for all patients and used as a baseline.

**Do:** A meeting was scheduled between GI nurses, GI physicians, radiologists and radiology schedulers to review current scheduling practices and identify areas to streamline the process. Decision was made that instead of faxing MRE orders to radiology scheduling office the order will be emailed directly to one scheduler who will reach out to family within one week to make an appointment and MRE completed within 2 weeks of order date.

**Study:** Patients with MRE starting January 7, 2024 to present were followed with length of time between MRE order and completion documented. After 1 year (January 2025) data was again reviewed between GI team and radiology team to address new concerns and further streamline the process.

**Act:** Feedback from the meeting included concerns of patients no‐showing to scheduled appointment or not answering phone call to schedule, leading to delay in scheduling. Additionally, radiologist have cut back the number of slots allotted to MRE's weekly dramatically decreasing availability and leading to increased wait times. Further interventions are on‐going at this time to address these concerns.

**Results:** Prior to initial intervention patients had an average wait of 60 days between order date of MRE and completion of MRE. After initial intervention average wait time was decreased to 51 days. There was no statistical significance between the two groups. Results for the second phase of the intervention are pending.

**Discussion:** There are significant barriers to obtaining an MRE in a timely manner at our institution. In an effort to improve time between MRE order and completion of MRE we began working with our radiology team to streamline the process. Despite this, barriers to completing an MRE continue to exist including lack of patient understanding regarding the importance of imaging and systemic issues such as limited availability of MRE time slots. As we continue to work with our institution to address these barriers and provide more patient education we hope to meet our goal of a MRE completion within 14 days of order date. We hope that would help us better assess patient's disease severity at onset to provide appropriate treatment leading to less complications and decreased disease severity. Additionally, we plan to further elucidate the impact delayed MRE's have on the course of our patient's treatment and disease course, which may provide a basis for systemic change at our institution.

## 721 EOSINOPHILIA IN VERY‐EARLY ONSET INFLAMMATORY BOWEL DISEASE: DOES IT PREDICT DISEASE SEVERITY?

*Serina Beydoun*, *Kristen Cares*

*Pediatric Gastroenterology*, *Children's Hospital of Michigan*, *Detroit*, *MI*

**Introduction:** Inflammatory bowel diseases, including ulcerative colitis (UC), Crohn's Disease, and IBD‐ unclassified are lifelong disorders affecting the gastrointestinal tract and characterized by relapsing‐remitting behavior. The pathogenesis is not fully understood but thought to be multifactorial and its incidence has significantly increased in recent decades. Very‐early onset IBD (VEO‐IBD) is defined as children diagnosed 6 years or younger and makes up about 6‐15% of total cases. Typically, these children have higher rates of resistance to conventional therapy and can have significantly more prolonged courses with remission difficult to achieve. Patients with VEO‐IBD are believed to have different features than later onset IBD patients making traditional guidelines for diagnosis and classification inaccurate.

It has been noted in the adult population that eosinophilia at the time of diagnosis is correlated with greater disease severity and a more complicated course. Our study hopes to elucidate if a similar pattern can be identified in patients diagnosed with VEO‐IBD, which may help guide treatment and help patients achieve remission sooner.

**Objective:** This study aims to determine if there is a relationship between eosinophilia either on biopsy or in the blood and the severity of the course of very‐early onset inflammatory bowel disease. We hypothesize that patients with peripheral eosinophilia and/or eosinophilia on colonic biopsy will have a more severe disease course with earlier treatment failure.

**Methods:** This study is a retrospective chart review at a single site, Children's Hospital of Michigan. Patients who have a diagnosis of Crohn's disease will be included. A chart review to collect data regarding usage of MRE and management of the disease via different medication regimens.

**Study Population:**

**Inclusion Criteria:**

Males and Females age 1‐6 who were diagnosed with Very‐ Early Onset Inflammatory Bowel Disease.

Diagnosed with inflammatory bowel disease in the last 13 years.

**Exclusion Criteria:**

Males and Females who lost follow up.

Males and females older than 26 years of age.

Males and females with a previous diagnosis of inflammatory bowel disease that has now changed.

**Primary Outcome:**

To determine if peripheral and/or mucosal eosinophilia at diagnosis predicts disease severity.

Severity at diagnosis based on: Mayo 3, SESCD (more than 15 is severe), perianal disease (abscess or fistula), structuring/penetrating disease

Severity based on course: blood transfusions, steroids (more than 2 bursts), hospitalizations (more than 2 in a year), more than 1 biologic, need to change medications w/in first year of diagnosis

**Secondary Outcomes:**

To determine if there is a difference in outcomes with peripheral eosinophilia vs mucosal eosinophilia.

To determine if there is a difference in outcomes in pts with eosinophilia in w/UC vs. Crohn's Disease

To identify other markers which can predict disease severity.

**Results:** There were 40 patients who met inclusion criteria for the study. 16 patients were diagnosed with Crohn's Disease, 19 patients were diagnosed with Ulcerative Colitis, and 5 patients were diagnosed with IBD‐ unspecified. The average age of diagnosis was 4.7 years old, the average current age of the patients included in the study is 12.7 years old. 34 total patients had any eosinophilia present, either peripheral, mucosal, or both. A majority of patients with any eosinophilia present were classified with mild disease at diagnosis (19/34, 56%) a majority of patients with eosinophilia required a change in medication within the first year of diagnosis (24/34, 70%). Most of these changes happened within the first 6 months of diagnosis (13/34. 38%) and a majority of patients needed more than one change to achieve remission during their course (11/34, 32%). There were 25 total patients how were classified as having a severe course and 88% (22/25) had either peripheral or mucosal eosinophilia at diagnosis. None of our results had a p‐value <0.05.

**Conclusions:** Children with very early onset IBD have higher rates of resistance to conventional therapy and can have significantly more prolonged courses with remission difficult to achieve. Identifying lab values that may predict disease severity and course is essential to optimizing treatment for these patients early on and inducing early remission. Eosinophilia is likely to play a role in disease severity and as our study shows patients with eosinophilia, either mucosal, peripheral, or both, are more likely to require escalation in treatment and have a course. While our results are not statistically significant they are clinically significant. Our small sample size is a major limitation of the this study and we would hope to engage in a multi‐site study to further elucidate the potential role of eosinophilia in VEO‐IBD.

## 724 DIAGNOSTIC PERFORMANCE OF VIDEOCAPSULE ENDOSCOPY, MAGNETIC RESONANCE ENTEROGRAPHY AND INTESTINAL ULTRASOUND IN PEDIATRIC PATIENTS WITH INFLAMMATORY BOWEL DISEASE: A LATIN AMERICAN SINGLE‐CENTER EXPERIENCE

*Veronica Busoni* ^*1*^, *Guillermo Vera Alvarado* ^*1*^, *Cristian Demeco* ^*2*^, *Judith Cohen Sabban* ^*1*^, *Maria Julieta Gallo* ^*1*^, *Maria Soledad Arcucci* ^*1*^, *Adauto Jhoanna* ^*1*^, *Marina Orsi* ^*1*^

^*1*^ *Pediatric Gastroenterology and Hepatology*, *Hospital Italiano de Buenos Aires Departamento de Pediatria*, *Buenos Aires*, *Buenos Aires*, *Argentina*; ^*2*^ *Hospital Italiano de Buenos Aires Servicio de Diagnostico por Imagenes*, *Buenos Aires*, *Buenos Aires*, *Argentina*

**Introduction:** Videocapsule endoscopy (VCE) is a valuable tool to diagnose and monitor small bowel Crohn's disease (CD). While VCE offers superior accuracy in detecting subtle mucosal lesions, magnetic resonance enterography (MRE) is more effective in revealing intramural inflammation, strictures and extraintestinal involvement. The intestinal ultrasound (IUS) has gained popularity as a bedside method due to its non‐invasive and radiation free technique, but may be less sensitive in identifying early mucosal changes.

**Aim:** To analyze the diagnostic yield of VCE, MRE and IUS in detecting small bowel lesions in children with pediatric inflammatory bowel disease (PIBD).

**Methods:** Retrospective analysis from 9/2020 to 5/2025 of patients with PIBD, referred to a University Hospital in Argentina. Inclusion criteria: established CD regardless of mid‐gut involvement. Demographic data were collected for all included patients. All of them underwent VCE (using PillCam™ SB3), MRE and/or IUS at the same period of time. For analysis purposes, lesions in duodenum, jejunum and/or ileum registered by VCE and/or MRE, as well as ileal lesions detected by IUS, were considered. All patients with paired studies (VCE + MRE and VCE + IUS) were included in comparative analysis. Mc Nemar's test was used to assess differences in detection rates between paired methods.

**Results:** A total of 107 VCE were performed in PIBD patients. We analysed 66 VCE from 60 patients with a median age of 13 years (IQR 10.3–16.3), 45% male, with confirmed CD. 36/60 patients underwent both VCE and MRE, and 35/60 underwent both VCE and IUS. VCE identified small bowel lesions in 24 of 36 patients (67%) compared to 11 of 36 (31%) with MRE (p = 0.0036). Segmental analysis showed that VCE detected more lesions than MRE in each small bowel segment: ileum (22 vs. 9 patients), jejunum (15 vs. 0), and duodenum (16 vs. 3). Similarly, VCE detected lesions in 25 of 35 patients (71%) compared to 11 of 35 (31%) with IUS (p = 0.0037). In both comparisons, VCE significantly outperformed the other modalities in lesion detection. Capsule retention occurred in 4 patients (6%), all of whom required medical or endoscopic retrieval.

**Conclusion:** In this PIBD cohort, VCE was significantly more accurate than both MRE and IUS, in detecting even subtle small bowel lesions at an earlier stage. These findings highlight the superior sensitivity of VCE, especially in detecting proximal and minimal mucosal lesions and support its role in the diagnostic and therapeutic decision‐making process when evaluating small bowel involvement in PIBD.

## 726 VENOUS THROMBOEMBOLISM WITHIN 30 DAYS PRE‐ OR 90 DAYS POST‐ PEDIATRIC INFLAMMATORY BOWEL DISEASE DIAGNOSIS

*Caroline Chinchilla Putzeys* ^*1*^, *Nina Gautam* ^*6*^, *Andrew Ritchey* ^*1,6*^, *Lucia Mirea* ^*1,6*^, *Victoria Bernaud* ^*1*^, *Umesh Sharma* ^*4*^, *Laura Hamant* ^*5*^, *Hilary Michel* ^*5*^, *Elizabeth Hilow* ^*1*^, *Ashish Patel* ^*1*^, *Sabina Ali* ^*4*^, *Jeremy Adler* ^*3*^, *Jonathan Moses* ^*2*^, *Brad Pasternak* ^*1*^

^*1*^ *Gastroenterology*, *Phoenix Children's Hospital*, *Phoenix*, *AZ*; ^*2*^ *Stanford University School of Medicine*, *Stanford*, *CA*; ^*3*^ *University of Michigan Health System*, *Ann Arbor*, *MI*; ^*4*^ *University of California San Francisco*, *San Francisco*, *CA*; ^*5*^ *Nationwide Children's Hospital*, *Columbus*, *OH*; ^*6*^ *The University of Arizona College of Medicine Phoenix*, *Phoenix*, *AZ*

**Background:** Inflammatory bowel disease (IBD), a prevalent chronic condition among pediatric patients, is associated with a hypercoagulable state that increases the risk of venous thromboembolism (VTE), particularly around the time of diagnosis. Although pediatric patients generally exhibit lower VTE risk compared to adults, specific clinical and laboratory factors can significantly elevate this risk. To date, the incidence and risk factors for VTE in pediatric patients with IBD remain underexplored, and the absence of definitive guidelines for VTE prophylaxis in this patient population presents a critical gap in clinical practice.

**Methods:** This matched case‐control study examined pediatric patients aged 0‐18 years with a new diagnosis of inflammatory bowel disease (IBD) between 2019 and 2024 from five tertiary pediatric institutions. Cases comprised individuals who developed venous thromboembolism (VTE) within 30 days prior to or 90 days following their IBD diagnosis. Controls were defined as patients with newly diagnosed IBD who did not develop VTE. Matching (1 up to 6) was based on the type of IBD diagnosis and the age at diagnosis (±2 years). Data collected included demographics, laboratory values, prothrombotic risk factors and treatment. Analyses compared factors between cases and controls using Wilcoxon signed rank sum or McNemar's test, as appropriate for the matched data.

**Results:** Study patients included 70 matches (15 VTE cases and 52 controls). Cases had a mean (standard deviation) age of 12 (3.61) and included 10 (66.7%) diagnosed with ulcerative colitis and 5 (33.3%) with Crohn's disease. Overall, gender was approximately equal, with subjects self‐reporting their race as White (71.6%), Asian (11.9%) or Hispanic (10.4%), and majority reporting ethnicity as non‐Hispanic (88.1%). The mean (standard deviation) of body mass index (BMI) was 19.75 (6.69).

No significant differences between cases and controls were detected in gender, race, and ethnicity.

VTE cases had significantly (p<0.05) lower mean hemoglobin and albumin, and higher mean C‐reactive protein (CRP), platelet count, body mass index (BMI), and more likely to have anticoagulant/antiplatelet use, steroid use, central line at diagnosis, any surgery within 90 days of IBD diagnosis.

**Conclusions:** The possible risk factors for VTE identified included hemoglobin, platelet count, CRP, and albumin levels, as well as BMI, anticoagulant/antiplatelet use, steroid use, central line at diagnosis, any surgery within 90 days of IBD diagnosis.

Our findings highlight the importance of targeted chemoprophylaxis for pediatric patients at high risk for VTE formation. These factors may be used in a future prediction model to stratify newly diagnosed pediatric IBD patients on their risk for VTE development.

Further, these findings highlight the importance of targeted chemoprophylaxis for patients at greater risk, as defined by systemic inflammation levels at the time of IBD diagnosis. Developing clinical guidelines for VTE prophylaxis in this population could enhance risk stratification and management strategies.

## 727 UPADACITINIB FOR THE TREATMENT OF PEDIATRIC INFLAMMATORY BOWEL DISEASE: A SINGLE CENTER EXPERIENCE

*Clifton Dietrick*

*pediatrics*, *Medical University of South Carolina*, *Charleston*, *SC*

**Introduction:** Inflammatory bowel disease (IBD), which consists of Crohn's disease (CD), ulcerative colitis (UC), and indeterminate colitis (IBDU) is a group of chronic conditions that involve inflammation within the gastrointestinal tract potentially leading to lifelong debilitating symptoms and complications. Treatment includes diet, medical therapy, and surgery or combinations of the three. Specifically, the use of advanced therapeutics including biologics and small molecules, have helped improve patient outcomes and advance the field of IBD treatment. Though numerous advanced therapeutics are FDA approved for the treatment of adult IBD, FDA approval for the treatment of pediatric IBD remains limited to one drug class, tumor necrosis factor inhibitors. As such, pediatric off‐label use is common and acquiring insurance approval for use of novel therapies is a mounting struggle for pediatric providers. Clinical data and more multi‐center studies on safety and efficacy is needed to advance approval options in pediatrics. Upadacitinib, a selective JAK 1 inhibitor, is approved for the treatment for adult CD and UC and is becoming more commonly used as a second line medication for moderate to severe pediatric IBD as well. Despite its more common use, pediatric literature remains scarce.

**Methods:** To better understand the safety and efficacy of upadacitinib, we are conducting a retrospective cohort study utilizing chart review of the electronic medical records of children with inflammatory bowel disease at the Medical University of South Carolina. To date, our study includes 12 patients diagnosed with CD, IBDU, and UC who were initiated on upadacitinib from the years 2022‐2024. Primary outcomes include clinical remission/response at 8 or 12 weeks depending on type of IBD and steroid‐free remission at 1 year. Clinical remission was defined as a Pediatric UC/CD Activity Index (PUCAI/PCDAI) <10, and clinical response as a decrease in PUCAI by 20% and a decrease in PCDAI by at least 12.5 points.

Secondary outcomes where information is available include: One year colectomy rates, clinical disease activity and biochemical assessment (CRP, Hg, albumin, fecal calprotectin) at start of medication use, 8 weeks and 12 weeks for UC/IBDU and CD, respectively, 6 months and 1 year. Where available, we report adverse events or reason for medication discontinuation will be recorded.

**Results:** Twelve pediatric patients with inflammatory bowel disease (IBD) initiated treatment with upadacitinib and were included in this retrospective cohort study. The mean age was 13.7 years, with 58% (7/12) female. Diagnoses included ulcerative colitis (UC) in 75% (9/12), Crohn's disease (CD) in 17% (2/12), and IBDU in 8% (1/12). Prior to upadacitinib, 42% (5/12) had failed one advanced therapeutic agent, and 58% (7/12) had failed more than one. Disease severity at initiation was classified as mild in 33% (4/12), moderate in 17% (2/12), and severe in 50% (6/12).

At 8–12 weeks post‐initiation, 33% (4/12) achieved clinical remission and 42% (5/12) achieved clinical response. Two patients (17%) worsened clinically, and one (8%) discontinued due to adverse effects (headache, chest pain). At 1 year, 33% (4/12) remained in steroid‐free remission, with an additional 2 patients in remission for less than a year. Four patients (33%) discontinued therapy—two due to non‐response and two due to adverse events (hair loss, migraines). Colectomy was avoided in 92% (11/12) of patients; one underwent elective colectomy related to IBD‐associated anxiety.

Clinical activity scores demonstrated marked improvement among UC/IBDU patients, with mean PUCAI decreasing from 52.2 at baseline to 22.2 at week 8. Among CD patients, mean PCDAI remained stable at 10. Biochemical markers also showed favorable trends: mean fecal calprotectin levels decreased from 1,260 mcg/g to 553 mcg/g, while mean serum albumin levels increased from 3.4 g/dL to 4.0 g/dL over the same period.

**Discussion:** This single‐center experience supports upadacitinib as a potentially effective and well‐tolerated treatment option for pediatric patients with moderate‐to‐severe IBD who have failed prior advanced therapies. Early clinical and biochemical improvements were observed, particularly among patients with ulcerative colitis. While the remission and response rates are encouraging, a subset of patients experienced adverse events or lack of efficacy, underscoring the need for careful patient selection and monitoring. These preliminary findings highlight the importance of real‐world data in informing treatment strategies and underscore the need for larger, multi‐center studies to further evaluate the safety and efficacy of upadacitinib in this population.

## 728 EFFICACY AND SAFETY OF UPADACITINIB IN PERIANAL CROHN'S DISEASE

*Niki Viradia*, *Joann Samalik*, *Jose Cabrera*, *Joshua Noe*, *Amanda Wenzel*, *Abdul Elkadri*

*Medical College of Wisconsin*, *Milwaukee*, *WI*

**Background and aim:** Pediatric Crohn's Disease (CD) is a chronic immune dysregulation resulting in inflammation of the gastrointestinal tract. Perianal CD is a severe form of CD with complications such as perianal fistulization and/or perianal abscess formation requiring antibiotics, surgical intervention and fecal stream diversion with an ileostomy. This results in significant pain and fecal incontinence, with downstream quality of life repercussions. There are two currently approved medications for pediatric CD, infliximab and adalimumab which both target Tumor Necrosis Factor alpha (TNFα). Children not responding to anti‐TNF therapy face numerous barriers including lack of insurance approval, lack of safety data and lack of evidence of efficacy of newer therapies. There has been evidence of efficacy of JAK pathway inhibition in CD and more specifically perianal CD, with some evidence of efficacy of JAK inhibition also being shown in Pediatric CD in the form of small case series. Upadacitinib, the first oral JAK inhibitor approved for moderately to severe adult CD, has recently been shown to have efficacy in adult perianal CD in ad‐hoc analysis of phase 3 adult trial data. We evaluated the efficacy of upadacitinib in a well‐characterized cohort of children with severe perianal CD at a major pediatric tertiary care center in Milwaukee, Wisconsin.

**Methods:** We performed a retrospective chart review of patients diagnosed under the age of 18 with perianal CD between January 2020 and May 2025. Patients were phenotyped using the Paris Modification of the Montreal Classification. Patients having failed a biologic therapy who were started on upadacitinib were selected, reviewing response in disease activity using Physicians’ Global Assessment (PGA), endoscopy data, Magnetic Resonance Imaging (MRI) and lab evaluation where available. Patients who had quiescent perianal disease on previous biologic therapy were excluded. Fistula response in patients with perianal fistulae was assessed by clinical examination, based on resolution of drainage, closure of all or any fistula openings, ability to taper from induction dosing of upadacitinib, and evidence of response on MRI.

**Results:** 13 patients were identified with perianal CD. All had previously had exposure to infliximab, with 3 failing only 1 biologic (23.1%), 6 failing two biologics (38.5%), 4 failing 3 biologics (30.8%) and 4 failing 4 biologics (7.7%). 5 had required ileostomy (38.5%), with only 3 needing seton placement (23.1%) and 6 using concurrent antibiotics while on upadacitinib (46.2%). Induction dosing was 45 mg in 9 patients (69.2%), with one patient started at 30 mg (7.7%) and 3 patients who could not swallow pills who used liquid preparations at 6 mg twice daily (23.1%). For pill formulations, 6 needed maintenance dosing changed back to 45 mg (60.0%), 3 patients were weaned to 30 mg daily (30.0%), and only 1 was able to be weaned to 15 mg daily (10.0%). For the 3 children requiring liquid formulations, 2 needed dose escalation to 12 mg twice daily (66.7%). 5 of the 13 patients had endoscopic re‐evaluation (38.5%), with all showing continued inflammation endoscopically and microscopically, though overall significantly improved. 7 patients had repeat MRI evaluation (53.8%), with 3 showing some improvement (42.9%) and all showing continued activity. 11 of the 13 patients (84.6%) reviewed showed significant clinical response to upadacitinib with regards to systemic and luminal disease activity. 3 patients (23.1%) had perianal abscess formation without the development of fistula, and one had not had repeat examination yet (7.7%). Fistulization completely responded in 3 of 9 patients (33.3%), with 2 experiencing complete fistula closure (22.2%) and 8 having closure of some fistulization (88.8%). Overall, no severe adverse reactions were noted, though 5 experienced acne (38.5%), 5 had severe dizziness or vertigo (38.5%) and 2 had significant nausea (15.4%). All minor side effects improved with lower dosing of upadacitinib.

**Conclusion:** Children with perianal CD who have failed anti‐TNF therapy experience clinical response to upadacitinib in 84%, with one third experiencing complete resolution of fistulizing disease. Partial response of fistulization with closure is seen in 88% of children. No severe adverse reactions were noted, suggesting that upadacitinib is an effective and safe form of treatment for this difficult‐to‐treat population of patients with limited treatment options.

## 729 IPEER2PEER: VIRTUAL MENTORING FOR FRENCH SPEAKING ADOLESCENTS WITH INFLAMMATORY BOWEL DISEASE: RECRUITEMENT UPDATE

*Hugo Gagnon* ^*7*^, *Marie‐Pier Vermette* ^*2*^, *Djouher Nait Ladjemil* ^*7*^, *Anthony Otley* ^*1*^, *Sara Ahola Kohut* ^*6*^, *Colette Deslandres* ^*7*^, *Véronique Groleau* ^*7*^, *Jessica Breton* ^*7*^, *Stéphanie Privé* ^*7*^, *Marie‐Josée Girard* ^*7*^, *Rilla Schneider* ^*2,3*^, *Julie Castilloux* ^*4*^, *Brigitte Moreau* ^*5*^, *Kelly Grzywacz* ^*7*^

^*1*^ *Dalhousie University Faculty of Medicine*, *Halifax*, *NS*, *Canada*; ^*2*^ *McGill University Faculty of Medicine and Health Sciences*, *Montreal*, *QC*, *Canada*; ^*3*^ *Gastroenterology*, *Montreal Children's Hospital*, *Montreal*, *QC*, *Canada*; ^*4*^ *Pediatric Gastroenterology*, *Centre Hospitalier de l'Universite Laval*, *Sainte‐Foy*, *QC*, *Canada*; ^*5*^ *Pediatric Gastroenterology*, *Hotel‐Dieu de Sherbrooke*, *Sherbrooke*, *QC*, *Canada*; ^*6*^ *Gastroenterology*, *The Hospital for Sick Children*, *Toronto*, *ON*, *Canada*; ^*7*^ *Pediatric Gastroenterology*, *Centre Hospitalier Universitaire Sainte‐Justine*, *Montreal*, *QC*, *Canada*

**BACKGROUND:** Inflammatory bowel disease (IBD) is increasing in prevalence among youth, with an annual standardized incidence of 13 per 100,000 in Quebec. Managing IBD is complex and impairs health‐related quality of life (HRQL) across physical, emotional, social, academic, and occupational domains. These impacts are especially pronounced during adolescence, a critical period for gaining independence and forming a stable self‐identity.

Emerging evidence from pediatric chronic illness research highlights the value of peer mentoring in enhancing social support, promoting positive identity formation, and fostering engagement in disease self‐management. The iPeer2Peer program, which connects adolescents with trained peer mentors via live video calls, has shown promising outcomes in conditions such as juvenile idiopathic arthritis.

This study aims to assess the feasibility of implementing the iPeer2Peer program in French‐speaking clinical settings in Quebec and to evaluate its impact on self‐management and HRQL in both mentors and mentees.

**METHODS:** Peer mentors were recruited from four tertiary care pediatric centers in the province of Quebec by clinicians and research coordinators. All mentors were former patients who recently transitioned to adult care. Mentors completed a validated 2‐day in‐person training led by a psychologist and social worker at the CHU Sainte‐Justine. Forty French‐speaking youth aged 12‐17 years with IBD are expected to be enrolled. IPeer2Peer provides emotional support, promotes self‐management, and delivers disease‐related education. The program includes up to ten tailored video call sessions (~20–30 minutes) and ad hoc text messaging over 15 weeks. Impact on self‐management and HRQL is measured at baseline (T1), program completion (T2), and six months later (T3) using validated questionnaires.

**RESULTS:** The mentors consist of 11 women and 2 men aged 18‐23, 9 with Crohn's disease, 3 ulcerative colitis, and 1 has IBD‐unclassified. Eleven mentees have been matched with mentors, and early evaluations suggest that the program is feasible and well received. The primary objective is to evaluate the feasibility of implementing the iPeer2Peer mentorship program. Mentors completed four validated questionnaires, with median T‐scores aligning with population norms (50.0 ± 10.0) (Table 1). Mentees completed six assessments (Table 2). Using the PUCAI score, 6 of 11 mentees had mild disease activity, and 5 were in remission. In the IMPACT III survey, 5 mentees reported they could “rarely” or “sometimes” talk to someone about their IBD, underscoring the relevance of peer support. On the PROMIS Pediatric Peer Relationships Scale, the median T‐score was 41.99, indicating scores slightly below the population mean. Four out of eleven youth scored above 70 on the RCADS, indicating clinically significant levels of depression and anxiety. Recruitment and data collection are ongoing. Follow‐up analyses will compare the baseline questionnaire scores with those obtained at the end of the program to assess changes over time.

**CONCLUSION:** The iPeer2Peer program has been initiated, with positive early feedback. While the study is ongoing, baseline data indicate that participants’ HRQL aligns with population norms. We expect individual improvement through program participation, given its previous success in chronic conditions. A knowledge translation plan will share findings and promote awareness of peer support in IBD care. This initiative could establish the first French‐language peer mentorship program for adolescents with IBD in Canada.


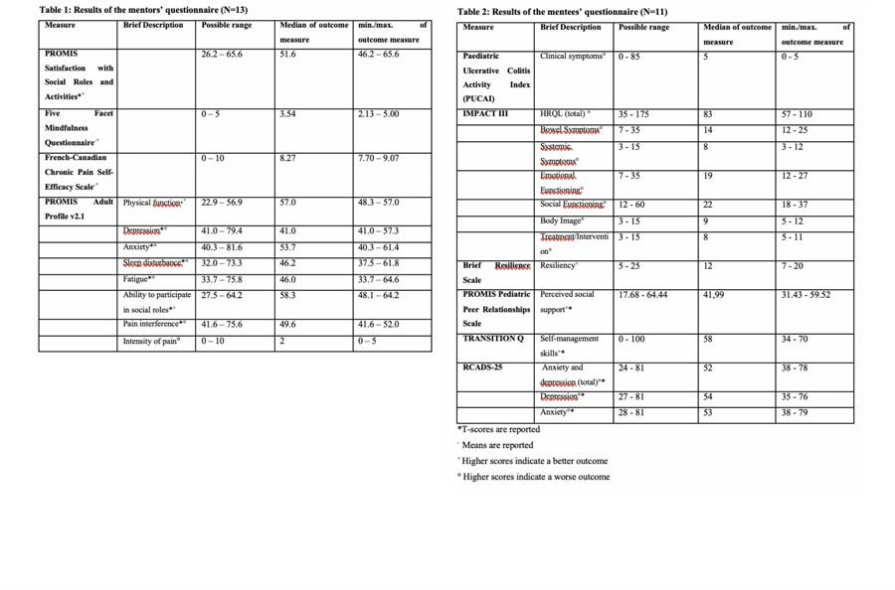


## 730 VITAMIN D DEFICIENCY IN PEDIATRIC INFLAMMATORY BOWEL DISEASE: PREVALENCE AND ITS CORRELATION WITH DISEASE ACTIVITY

*Amrita Gujar* ^*1*^, *Aashay Dharia* ^*2*^, *Noah Kondamudi* ^*1*^

^*1*^ *Pediatrics*, *Brooklyn Hospital Center*, *New York*, *NY*; ^*2*^ *Internal medicine*, *Brooklyn Hospital Center*, *New York*, *NY*

**Introduction:** Inflammatory bowel disease (IBD), encompassing Crohn's disease and ulcerative colitis, is a chronic inflammatory condition of the gastrointestinal tract. Emerging evidence suggests a potential link between Vitamin D status and IBD pathogenesis, as Vitamin D is known to modulate immune responses and maintain intestinal barrier integrity. Despite this, the prevalence of Vitamin D deficiency in IBD and its association with disease activity remain underexplored, particularly in pediatric population. This study aims to evaluate the prevalence of Vitamin D deficiency in IBD patients and assess its correlation with disease severity.

**Methods:** Study Design and Participants.

A retrospective cross‐sectional analysis was conducted on 51 IBD patients (40 Crohn's disease, 11 ulcerative colitis) attending a gastroenterology clinic. Data collected included age, sex, disease type, Vitamin D levels (nmol/L), Vitamin D deficiency status (defined as ≤20 nmol/L), and disease activity scores based on Pediatric Crohn's Disease Activity Index (PCDAI) and Pediatric Ulcerative Colitis Activity Index (PUCAI). Disease activity was categorized as inactive, mild, moderate, or severe based on the scores.

**Statistical Analysis:** Descriptive statistics were calculated for prevalence estimates. Chi‐square tests assessed associations between Vitamin D deficiency and disease activity (active vs. inactive). Pearson correlation was used to assess the relationship between Vitamin D levels and severity scores. Analyses were performed using Python (Pandas, SciPy), with significance at p < 0.05.

**Results:** Prevalence of Vitamin D Deficiency.

The overall prevalence of Vitamin D deficiency in the cohort was 55%. Among patients with active disease, 78.1% had Vitamin D deficiency, compared to 15.8% in the inactive disease group. The chi‐square test demonstrated a significant association between Vitamin D deficiency and active disease (p<0.0001).

Correlation Between Vitamin D Levels and Disease Severity

Pearson correlation analysis revealed a significant negative correlation between Vitamin D levels and disease severity scores (r = ‐0.35, p = 0.012). Subgroup analysis showed this correlation was significant in Crohn's disease (r = ‐0.38, p = 0.016) but not in ulcerative colitis (r = ‐0.43, p = 0.19).

**Discussion:** This study demonstrates a high prevalence of Vitamin D deficiency in IBD patients, particularly among those with active disease. The significant inverse correlation between Vitamin D levels and disease severity suggests a potential role for Vitamin D in modulating disease activity, especially in Crohn's disease. These findings align with previous research highlighting Vitamin D's anti‐inflammatory properties and its involvement in gut immunity. In comparison, the correlation in ulcerative colitis was not statistically significant, likely due to a smaller sample size; the trend warrants further investigation.

This study's limitations include its retrospective design, limited sample size, and potential confounding factors (e.g., seasonal variation, dietary intake). Nonetheless, the results underscore the clinical importance of monitoring Vitamin D status in IBD patients and considering Vitamin D supplementation as a potential adjunct in disease management. Future prospective studies with larger cohorts and interventional trials are recommended to determine the impact of Vitamin D correction on IBD outcomes.

**Conclusion:** Vitamin D deficiency is common among IBD patients, particularly in those with active disease. Lower Vitamin D levels are associated with increased disease severity, suggesting a potential role for Vitamin D in the pathogenesis and progression of IBD. Regular assessment of Vitamin D status and appropriate supplementation should be considered as part of comprehensive IBD care.


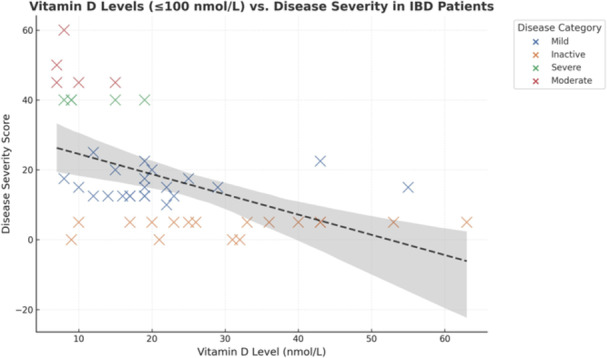


Scatter plot showing negative correlation (r = ‐0.35, p = 0.012) between Vitamin D deficiency and IBD severity


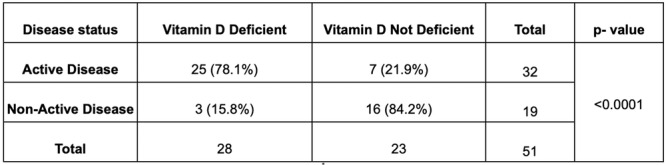


2x2 table showing Vitamin D deficiency vs Disease activity status

## 731 CHARACTERIZING THE ROLE OF THE PERI‐APPENDICEAL MUCOSA IN CHILDREN AND YOUNG ADULTS WITH ULCERATIVE COLITIS

*Diane Hsu*, *Eduardo Contijoch*, *Elizabeth Costello*, *Dorsey Bass*, *David Relman*

*Stanford University*, *Stanford*, *CA*

**Background:** While medical therapy has advanced, up to 30% of patients with ulcerative colitis (UC) will require staged colectomy. Recent prospective studies show appendectomy may be effective in achieving clinical remission in 30% of patients with treatment‐refractory UC, and medical therapy plus appendectomy is superior to medical therapy alone in achieving clinical remission, suggesting a role for the appendix in UC pathogenesis and progression. Biofilm progressively decreases from the appendix to the distal colon, and its potential disruption in UC may lead to invasion of pathobionts from the appendix into intestinal epithelial cells. In a pilot study, CD4+, CD8+, CD69+, and HLA‐DR+ T cell populations in periappendiceal biopsies and samples of resected appendix were similar in each patient, suggesting the orifice could reflect appendiceal tissue. Our objective is to examine the relationship between peri‐appendiceal microscopic inflammation and the mucosa‐associated microbiome in pediatric UC, CD, and in non‐inflammatory bowel disease (IBD) controls.

**Methods:** From February 2021‐December 2023, we performed a prospective pilot study of treatment‐naïve children and young adults < 21 years of age who were undergoing diagnostic colonoscopy. Rectal and peri‐appendiceal cecal biopsies were obtained for immunophenotyping using imaging mass cytometry, and peri‐appendiceal inflammation was classified using the Nancy histologic index. Blood samples were obtained for suspension mass cytometry, and mucosal brushings were obtained from the peri‐appendiceal orifice, at 2‐3 cm increments both approaching and moving away from the orifice, and rectosigmoid for microbiome analysis with amplicon sequencing of the V4 region of the bacterial 16S rRNA gene.

**Results:** We recruited 52 subjects including 9 individuals with UC, 8 with CD, 1 with IBD‐unclassified, and 34 non‐IBD controls, and 423 mucosal microbiome samples were collected. Subjects with UC (8/9, 89%) had higher rates of microscopic inflammation at the peri‐appendiceal orifice compared to those with CD (3/8, 38%) and non‐IBD group (0/34, 0%). Although prior studies using fecal microbiome samples have demonstrated decreased alpha diversity in the microbiome of individuals with IBD compared to controls, we found higher alpha diversity in patients with UC compared to CD (p=0.042) and in female compared to male patients (p=0.004), but no statistically significant difference between CD and non‐IBD controls or sampling location in our mucosal brushing samples (Figure 1). Similarly, when incorporating histologic data from our biopsy specimens, we did not observe a significant difference in mucosal microbiome diversity in mucosal samples with active inflammation compared to those without inflammation. Subject was the most important factor in explaining differences in the mucosa‐associated microbiome, more so than disease status or body site (Figure 2).

**Conclusions:** Our data do not reveal significant mucosa‐associated microbiome differences that can be attributed to disease status or body site but demonstrate high inter‐individual variability. While the number of patients with IBD was low, our study adds to the growing literature on the mucosa‐associated microbiome in IBD, as the majority of prior studies are of fecal samples. Additional analyses incorporating plasma and imaging mass cytometry are underway. Subsequent studies are needed to better understand the complex relationships between the appendiceal mucosal microbiome, local and systemic immune system, and development and progression of inflammatory bowel disease.


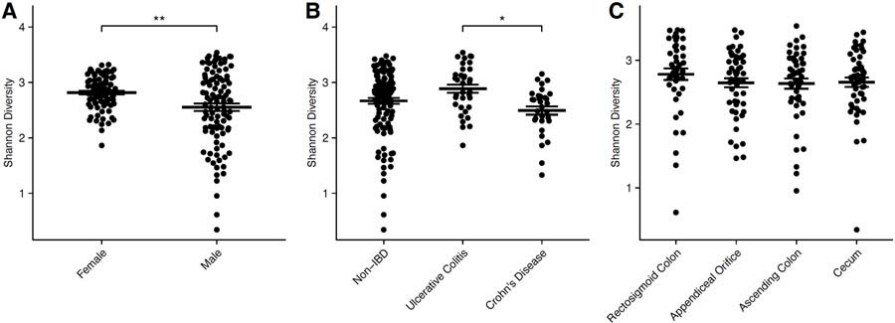


Figure 1. Shannon diversity by (A) sex, (B) disease status, and (C) body site.


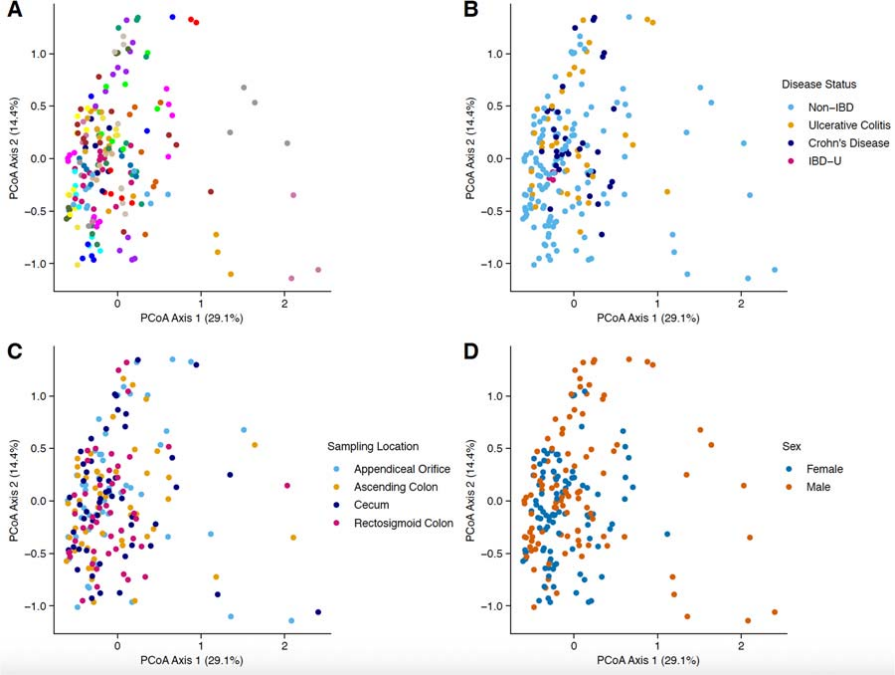


Figure 2. Principal coordinates analysis of unweighted UniFrac distances by (A) individual subjects, (B) disease status, (C) sampling location, and (D) sex.

## 733 ADJUNCTIVE HYPERBARIC OXYGEN THERAPY IN PEDIATRIC PERIANAL CROHN'S DISEASE

*Sneha Magesh*, *Arthur Kastl*

*The Children's Hospital of Philadelphia*, *Philadelphia*, *PA*

**Background:** Perianal disease (PD) occurs in nearly 25% of children with Crohn's disease (CD), often causing substantial physical, psychological, and social impairment. Perianal manifestations are often complex and may be refractory to conventional medical treatments and surgical interventions. Hyperbaric oxygen therapy (HBOT) has been used in certain clinical settings, including different phenotypes of inflammatory bowel disease (IBD). HBOT provides 100% oxygen under pressure to target tissue hypoxia, recruit local stem cells, and thereby modify inflammatory pathways with the goal of healing the affected soft tissues. Literature regarding the use of HBOT in children with perianal complications from CD is very limited. The aim of this study was to describe the perianal outcomes in children receiving adjunctive HBOT for management of refractory PD.

**Methods:** We preformed a retrospective descriptive case series of children with established CD and perianal manifestations, and who received HBOT specifically for their PD. The participants must have had a diagnosis of CD before 18 years of age, based on validated ICD‐9 or ICD‐10 diagnostic coding, and received gastroenterology care at the Children's Hospital of Philadelphia during the study period of 1/2014‐12/2024. HBOT sessions were done at an academic hospital setting with a protocol of 2.0 atmospheres absolute with 100% oxygen for 120 minutes. Abstracted clinical metadata included demographic information, IBD characteristics and treatments, radiologic studies, endoscopic reports, and operative notes. Immunosuppressive therapies for CD must have been at stable dosing during the course of HBOT. The PD course following completion of HBOT was then defined as improved, stable, or worsened. PD was defined as improved if there was improvement in perianal symptoms and imaging characteristics after its initiation, relative to PD characteristics upon entering HBOT. If there was no appreciable change in perianal symptoms and characteristics via imaging, but without need for subsequent surgical intervention over the following year, then PD was determined to be stable while on HBOT. Lastly, if there was subsequent PD complication requiring surgical intervention, then PD was determined to have worsened while on HBOT.

**Results:** A total of 5 participants were included, with 4 males and 1 female. The median age at diagnosis of CD was 11 years (range 8‐14) and median age of diagnosis of PD was 12 years (range 8‐14). All were on biologic therapy, and all had complex refractory PD that required several surgical interventions prior to starting HBOT (range 5‐10), including abscess drainage, seton placement, and/or fistulotomy. One participant required diverting colostomy for management of refractory PD. Historical PD characteristics prior to HBOT involved deep fistulae (100%), superficial fistulae (20%), deep abscess (100%), major skin breakdown (40%), minor skin breakdown (60%), and anal fissure (20%). The total number of HBOT sessions received ranged from 19‐90, with 3 participants receiving a typical 40 session course. HBOT improved the PD in 3 participants, while 1 participant showed no overall change, and 1 participant had worsened PD. No participant had a respiratory or other adverse event as a result of HBOT. The patient who had worsened PD developed a perirectal abscess requiring drainage less than 1 month after completion of HBOT, though had imaging evidence of a small abscess a few months prior to receiving HBOT. The patient who had no appreciable change received only 19 of the planned 40 HBOT sessions, discontinuing early due to weariness with travelling to and completing sessions. Of the 3 participants who had a favorable response to HBOT, 2 had sustained improvement for at least one year before re‐developing an abscess, and the other participant had a long‐lasting improvement following HBOT. For one of the participants who re‐developed an abscess, they received another course of HBOT and responded favorably again.

**Conclusion:** Complex PD may be refractory to conventional medical and surgical management, and more treatment options are needed in this small but challenging subset of patients. Herein, we found that HBOT is potentially helpful in improving PD characteristics in select patients, though the effect may not be sustained long‐term. Further evaluation is needed to determine efficacy HBOT in different PD phenotypes.

## 736 PATIENT AND CAREGIVER PERCEPTIONS OF INFLAMMATORY BOWEL DISEASE AND IRRITABLE BOWEL SYNDROME: A CROSS‐SECTIONAL SURVEY STUDY

*Sapna Khemka*, *Jarrett Rardon*, *Peter Lu*, *Carlo Di Lorenzo*, *Brendan Boyle*, *Ross Maltz*, *Hilary Michel*

*Pediatric Gastroenterology*, *Nationwide Children's Hospital*, *Columbus*, *OH*

**Background:** Inflammatory bowel disease (IBD) and irritable bowel syndrome (IBS) can both present with abdominal pain, diarrhea, and weight loss. While IBD is an inflammatory disorder with multifactorial pathogenesis, IBS is a disorder of gut‐brain interaction (DGBI). DGBIs are equally prevalent among children with IBD compared to the general pediatric population (~25%). Previous studies demonstrated that both adult patients with IBD or IBS and medical students perceive higher levels of stigma towards IBS compared to IBD. This is concerning, as disease stigma has been shown to impact health‐seeking behaviors among patients and care delivery among providers. Our study aims to assess perceptions of IBD and IBS among patients with IBD and their caregivers.

**Methods:** We performed a cross‐sectional survey study of patients with IBD, ages 12 to 21 years, and their caregivers. Participants were excluded if unable to complete the survey in English. Recruitment occurred in person at routine clinic or infusion visits and via the patient portal. The 29‐item survey was designed by a multidisciplinary team, modeled after the previously mentioned medical student bias survey, and completed electronically via REDCap. It included demographics, medical history, and questions assessing knowledge and attitudes toward IBD and IBS. Descriptive statistics and Fisher's exact tests were utilized for analysis of responses.

**Results:** Of 287 participants, 8 were excluded as they did not meet inclusion criteria or complete the survey, leaving 140 patients and 139 caregivers for analysis (demographics summarized in Table 1). Most participants knew that IBS presents with abdominal pain and that patients can have both IBS and IBD, however, fewer knew that IBS does not have diagnostic testing. The largest proportions of patients felt that IBD is more difficult to be diagnosed with, but that IBD and IBS are equally difficult to manage. In contrast, the largest proportion of caregivers felt that IBS is more difficult to be diagnosed with and IBD more difficult to manage. When asked about illness severity, patients and caregivers agreed that patients with IBD exhibit worse symptoms, are sicker, and have a worse prognosis compared to patients with IBS. Additionally, similar proportions of patients and caregivers believed that patients with IBD are equally as likely to demonstrate patience, optimism, resilience, and honesty regarding their condition as those with IBS. Results are summarized in Table 1. While participants did not perceive significantly higher stigma toward IBD versus IBS amongst healthcare providers, they did perceive significantly higher levels of stigma towards IBS in the general population (Figure 1).

**Conclusions:** Knowledge and attitudes toward IBD and IBS vary amongst patients with IBD and their caregivers, though IBD was generally perceived as a more severe disorder. Participants did not appear biased toward patients with IBD versus IBS and did not perceive healthcare providers to be either, perhaps related to them receiving care at an institution with centers of excellence for both IBD and DGBIs. However, a significant proportion perceived bias toward patients with IBS in the general population, emphasizing the importance of healthcare providers in providing accurate education to decrease stigma and improve healthcare delivery for patients with these unique conditions.


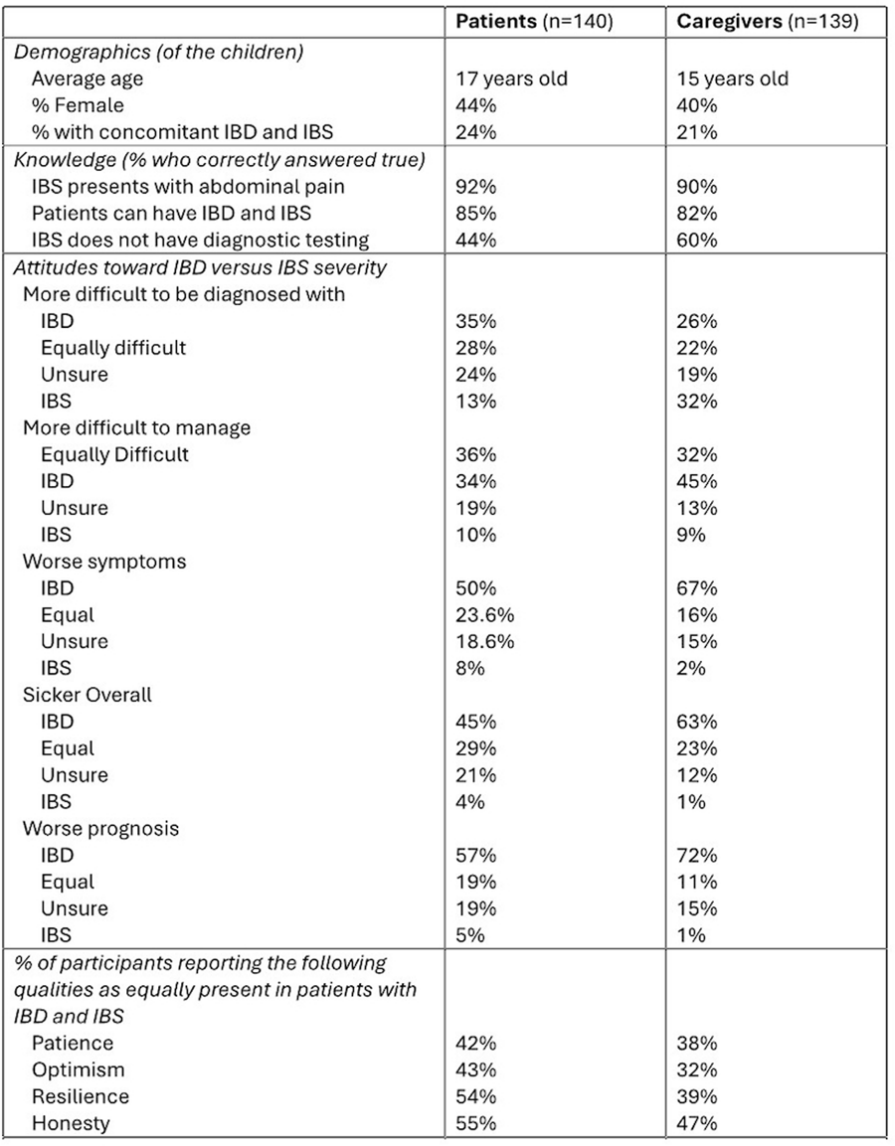


**Table 1.** Summary of survey Responses


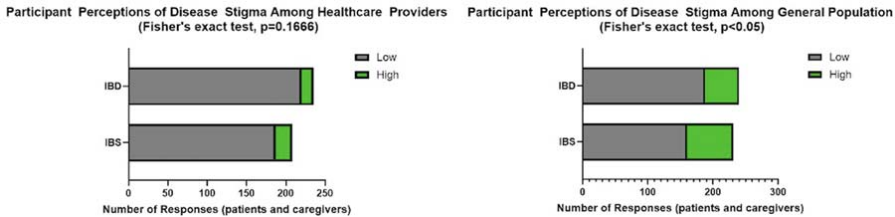


**Figure 1.** Participant perceptions of disease stigma towards patients with IBD versus IBS

## 737 EFFICACY AND SAFETY OF SUBCUTANEOUS VEDOLIZUMAB FOR ULCERATIVE COLITIS OR CROHN'S DISEASE IN YOUNG ADULT PATIENTS: A POST HOC ANALYSIS OF VISIBLE 1 AND 2

*Sandra Kim* ^*1*^, *Stephen Jones* ^*2*^, *Sharif Uddin* ^*2*^, *Abbey Wojtowicz* ^*2*^, *Lisa Young* ^*2*^, *Marla Dubinsky* ^*3*^

^*1*^ *Pediatric Gastroenterology, Hepatology & Nutrition*, *Cleveland Clinic Children's*, *Cleveland*, *OH*; ^*2*^ *Takeda Pharmaceuticals U.S.A., Inc*., *Cambridge*, *MA*; ^*3*^ *Division of Pediatric Gastroenterology*, *Icahn School of Medicine at Mount Sinai*, *New York*, *NY*

**Introduction:** Vedolizumab delivered subcutaneously (SC) following two intravenous (IV) infusions is approved for the treatment of moderate to severe ulcerative colitis (UC) and Crohn's disease (CD). Young adults (18–25 years of age) may benefit from the convenience and reliability of SC administration, particularly for college or travel. We aimed to determine the efficacy and safety of vedolizumab SC in young adults from the VISIBLE 1 (NCT02611830) and VISIBLE 2 (NCT02611817) phase 3 clinical trials.

**Methods:** During the VISIBLE 1 and 2 trials, patients received open‐label vedolizumab 300 mg IV at weeks 0 and 2. Those with a clinical response at week 6 were randomized as follows: vedolizumab 108 mg once every 2 weeks SC, vedolizumab 300 mg once every 8 weeks IV (reference arm, VISIBLE 1 only), or placebo up to week 52.

In this post hoc analysis, patients with moderate to severe UC (VISIBLE 1) or moderate to severe CD (VISIBLE 2) who received vedolizumab SC were grouped by age at baseline (≤ 25 years or > 25 years). The primary objective was to assess the proportion of patients with clinical remission at week 52. Secondary objectives included the proportion of patients with a clinical response, mucosal healing (VISIBLE 1 only), and an enhanced clinical response (VISIBLE 2 only) at week 52. The proportion of patients with each efficacy outcome was analyzed by age group, and the percentage differences with associated 95% confidence intervals were reported. Safety outcomes and numerical differences in baseline characteristics between age groups were also reported.

**Results:** A numerically greater proportion of patients aged > 25 years than those ≤ 25 years had a severe Mayo score (9–12) in VISIBLE 1 (58.0% vs 50.0%) or a Crohn's Disease Activity Index score > 330 in VISIBLE 2 (44.1% vs 32.7%) at baseline. Similar proportions of patients aged ≤ 25 years and > 25 years had prior anti‐tumor necrosis factor α (TNFα) failure in VISIBLE 1 (38.9% vs 37.5%), but a numerically greater proportion of patients aged ≤ 25 years than those > 25 years had prior anti‐TNFα failure in VISIBLE 2 (60.0% vs 53.6%). There were no statistically significant differences in the proportion of patients who had clinical remission, clinical response, or mucosal healing at week 52 in VISIBLE 1 (**Table 1**), or clinical remission, clinical response, or enhanced clinical response at week 52 in VISIBLE 2 (**Table 2**) between patients aged ≤ 25 years and > 25 years who received vedolizumab SC. The overall response and remission rates for the placebo and vedolizumab IV groups are listed in **Table 1** and **Table 2**. In both trials, a greater proportion of patients receiving vedolizumab SC achieved these efficacy endpoints than the placebo group regardless of age. For VISIBLE 1, overall efficacy was similar for vedolizumab SC and IV. No new safety signals were identified when grouping by patient age.

**Discussion:** This post hoc analysis of two phase 3 clinical trials demonstrates that vedolizumab SC is similarly efficacious in patients aged ≤ 25 years and > 25 years. Vedolizumab SC may be a suitable option for young adults seeking a convenient and effective treatment to accommodate their lifestyle and ensure ongoing adherence to therapy. The limited number of patients aged ≤ 25 years requires additional research to understand outcomes in this age group in real‐world settings.


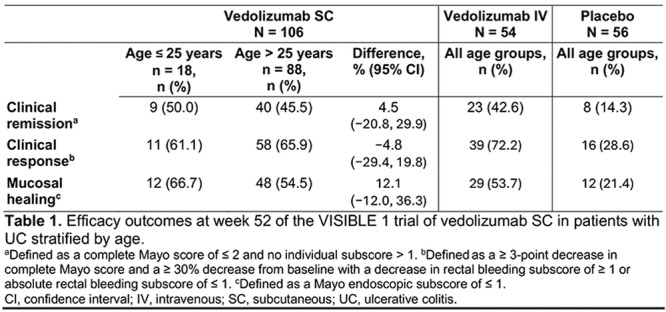



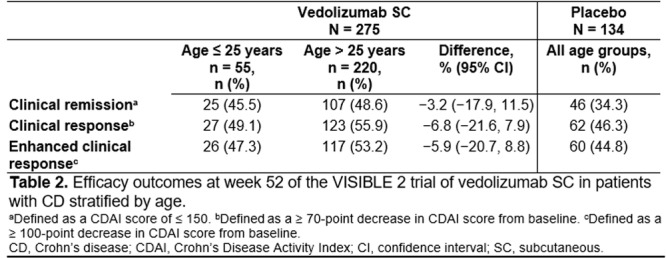


## 738 MEDICATION CHANGES FOR INFLAMMATORY BOWEL DISEASE AFTER TRANSITION OF CARE FROM PEDIATRIC TO ADULT GASTROENTEROLOGY

*Alexander Lyons* ^*1*^, *Jeremy Adler* ^*1,2*^

^*1*^ *Pediatric Gastroenterology*, *University of Michigan Michigan Medicine*, *Ann Arbor*, *MI*; ^*2*^ *Susan B. Meister Child Health Evaluation and Research Center*, *University of Michigan Michigan Medicine*, *Ann Arbor*, *MI*

**Background:** Despite recent approvals for new drugs to treat adults with inflammatory bowel disease (IBD), there are only two FDA approved advanced treatment options for children which include infliximab and adalimumab. Children with IBD commonly have rapid drug clearance and require higher doses of infliximab and adalimumab than adults with IBD. Contemporary dose escalation strategies and proactive therapeutic drug monitoring improve efficacy while resulting in higher treatment doses than are often used in adult care. There has been increasing efforts focused on improving transition readiness, patient self‐efficacy, and education. However, changes in medical therapy after transferring from pediatric to adult care have not been well characterized. It is unclear if high‐dose therapy is maintained or how often it is reduced after transfer to adult care.

**Objectives:** Our aim was to determine the frequency and predictors of treatment change within 1 year after transfer to adult care.

**Methods:** We performed a retrospective cohort study of pediatric patients with IBD who transferred care from pediatric to adult GI from January 2010‐December 2023 within Michigan Medicine. All patients had to have a minimum follow up period of 12 months after transferring to adult care. Patients diagnosed with IBD at an outside institution prior to transfer to the University of Michigan and who had an incomplete diagnostic record were excluded from the study. We compared medication use, medication dosage before and after transferring care. We used Chi‐square and Fisher's exact test for bivariate comparisons. We used logistic regression to evaluate treatment changes using independent variables of age at transition, sex, race, gap (in days) between pediatric and adult visits. Michigan Medicine Institutional Review Board approved considered this retrospective study exempt.

**Results:** We obtained preliminary data from 19 patients enrolled with a median age of 21.8 years (interquartile range [IQR] 20.7‐22.8), 58% male, 84% White, 79% with Crohn's disease. 18 patients were on a medication at time of transition from pediatric to adult care. The gap between last pediatric visit and first adult visit had a median of 169 days (IQR 111‐356; Figure 1). Overall, 38.9% of patients had a medication change or dose adjustment within 1 year of establishing adult care, which included 4 medication changes and 3 dose or interval adjustments. 2 dose adjustments occurred in infliximab‐treated patients, 1 in a ustekinumab‐treated patient; all which were dose or interval escalations. Of the medication changes, 2 involved a switch from adalimumab to ustekinumab; 1 patient had transitioned from infliximab and methotrexate to ustekinumab, and another switched from infliximab to ustekinumab (using upadacitinib as bridging therapy). 85% of medication changes or dose adjustments involved patients with Crohn's disease. Multivariate logistic regression found no association between medication changes and age at transition, sex, race, days in between visits (p=0.64). In descriptive review, we found the majority of patients who had treatment changes had done so due to active disease.

**Conclusion:** Among pediatric patients with IBD on advanced therapies, we found nearly 40% underwent treatment change after transferring from pediatric to adult care. Changes were mostly treatment escalation and were more common among patients with active disease, particularly with Crohn's disease. No changes were made based on convenience moving from intravenous medications to subcutaneous or oral options. Data collection is ongoing. With a larger sample size, we anticipate a more nuanced evaluation characterizing treatment changes.


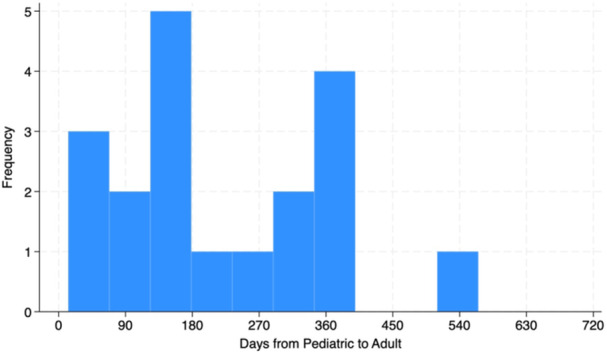


Figure 1: Number of Days in Between Appointments from Last Pediatric Visit to First Adult Appointment

## 739 ASSESSING RESILIENCE IN YOUTH WITH INFLAMMATORY BOWEL DISEASE

*Catherine Marchetta* ^*1*^, *Amanda Stone* ^*2,3*^

^*1*^ *Pediatric Gastroenterology, Hepatology, and Nutrition*, *Monroe Carell Junior Children's Hospital at Vanderbilt*, *Nashville*, *TN*; ^*2*^ *Pediatrics*, *Vanderbilt University Medical Center*, *Nashville*, *TN*; ^*3*^ *Anesthesiology*, *Vanderbilt University Medical Center*, *Nashville*, *TN*

Anxiety and depression are common in patients with IBD (~15‐20%). As the prevalence of pediatric IBD continues to rise, identifying resilience factors that may mitigate mental and physical impacts of this disease is essential. Resilience is multidimensional and comprised of resources, behaviors, and psychosocial factors that help someone bounce back from difficulties. Understanding how resilience factors relate to IBD symptoms and mental health comorbidities could help inform future preventive interventions in childhood.

The goal of this research study is to evaluate the relation between psychosocial resilience constructs, disease and symptom severity, and psychological symptoms in pediatric IBD patients to identify potential resilience‐based targets for future brief interventions. We enrolled pediatric patients (8‐17 years of age) with IBD who were treated at Monroe Carell Jr. Children's Hospital at Vanderbilt within the last 18 months (n = 58; current abstract reflects an interim analysis at n = 38) and a caregiver to complete a one‐time ~20 minute survey. Additional eligibility criteria included the ability to complete a survey in English independently or with minimal assistance. The surveys selected were validated for use in pediatric populations and included: Pediatric PROMIS measures (Family Relationships, Peer Relationships, Anxiety, Sleep Disturbance, Depressive Symptoms, Life Satisfaction, Stigma Psychological Stress Experiences), IBD Symptom Inventory, Brief Resilience Scale, Children's Hope Scale, Children's Revised Impact of Event Scale, Abdominal Pain Index, and Functional Disability Inventory. Participant's IBD disease status (quiescent vs. mild/moderate/severe) was extracted from their medical record. The relationship between psychosocial resilience factors and IBD symptoms, functioning, and mental health comorbidities were analyzed using Pearson's r correlations. To evaluate the extent to which the correlations between resilience factors and mental and physical health outcomes were accounted for by current IBD symptoms, we ran partial correlations controlling for IBD symptoms severity. Independent samples T‐tests evaluated whether psychosocial risk and resilience constructs differed by IBD disease status (quiescent vs. active disease).

Resilience factors (i.e. hope, resilience, peer relationships, family relationships, and life satisfaction) were associated with lower IBD‐related symptoms and impairment (Figure A). When controlling for IBD symptom severity, almost all resilience constructs continued to show significant correlations with anxiety and depressive symptoms. However, correlations between resilience constructs in relation to abdominal pain and functional impairment were no longer significant. Patients with mild or moderate disease compared to those with quiescent disease reported significantly lower life satisfaction, family relationships, and hope, but did not differ on other factors (Figure B).

Psychosocial resilience appears to be a potential target for improving mental health comorbidities in youth with IBD regardless of current disease status. Brief interventions that foster resilience in children could serve as a protective effect against potential adverse psychosocial outcomes and improve disease course, but further longitudinal research is needed. One actionable takeaway is that hope, which relates to anxiety and depressive symptoms, is significantly lower in youth with active disease. Medical providers can strive to instill a sense of hope to strengthen resilience in patients even during brief clinical encounters.


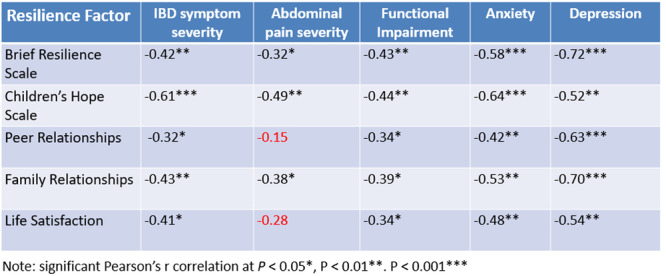


**Figure A.** Resilience factors associated with IBD‐related symptoms and impairment


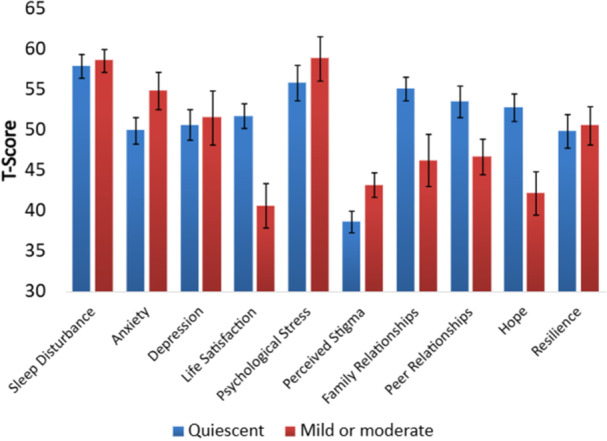


**Figure B.** Resilience and stress factors as they relate to disease severity in children with IBD

## 742 LEAN BODY MASS DEFICITS IN NEWLY DIAGNOSED PEDIATRIC CROHN'S DISEASE REVERSED WITHIN 8 WEEK OF EXCLUSIVE ENTERAL NUTRITION THERAPY

*Jillian Owens* ^*1*^, *Mary Zachos* ^*2,1*^, *Heather Mileski* ^*1*^, *Mary Sherlock* ^*2,1*^, *Muhanad Al Ruwaithi* ^*2*^, *Stephanie Atkinson* ^*2*^, *Jenna Dowhaniuk* ^*2,1*^

^*1*^ *McMaster Children's Hospital*, *Hamilton*, *ON*, *Canada*; ^*2*^ *Pediatrics*, *McMaster University*, *Hamilton*, *ON*, *Canada*

**Purpose:** Exclusive enteral nutrition (EEN) is first line therapy for pediatric Crohn's disease with established improvement in weight gain and disease activity. The observed weight gain following EEN can be related to fat mass or lean‐body mass, with the preference for lean body mass gain following nutritional rehabilitation. Dual‐energy X‐ray absorptiometry (DXA) is considered the gold standard modality to assess body fat, lean and bone mass. The primary aim was to review body composition changes following a standard 8‐week treatment with EEN for children with newly diagnosed Crohn's disease.

**Methods:** Pediatric patients with newly diagnosed Crohn's disease completed baseline and post‐treatment anthropometric measurements, whole body DXA scan (QDR®4500 series Hologic Inc. Discovery^TM^ Waltham, MA) and bi‐pedal BIA (Tanita BF‐350). Height (statiometer) and weight (Tanita BF‐350) were measured to determine BMI z‐score (WHO). Included patients received EEN and did not receive concomitant steroids.

**Results:** 18 children (78% male) were included in the study. Average age was 12.33 years (SD 3.14). Disease distribution included predominantly ileal or ileocolonic disease (Paris L1 or L3; 94.4%), non‐stricturing non‐penetrating disease (Paris B1 88.9%). 44% has perianal disease. At diagnosis, mean BMI z‐score was ‐1.25 (SD 0.87). The median percent ideal body weight was 87.9% (SD 7.21). Participants received EEN for 8.37 weeks (mean). DEXA demonstrated increase in mean lean body and fat mass between groups by 3291.2 g (95% CI; 2193.10, 4389.30(p <0.001) and 1369.24(95% CI;985.42, 1753.06 (P<0.001), respectively with EEN therapy. Numerous secondary outcomes were significant including improvement in weight (p<0.001), BMI (p<0.001), mid upper arm circumference (MUAC) (p<0.001) and triceps skin folds (TSF) (p<0.001).

**Conclusions:** This study highlights the statistically significant and greater improvement in lean body mass by DEXA as well as weight, BMI, MUAC, and TSF within 8 weeks of EEN. Further studies comparing body composition change to other therapies are needed.

## 743 DIAGNOSTIC PERFORMANCE AND CLINICAL UTILITY OF INTESTINAL ULTRASOUND IN A LARGE PEDAITRIC IBD COHORT

*Stephanie Rager* ^*2,1*^, *Hillary Moore* ^*2,1*^, *Neal LeLeiko* ^*2,1*^, *Joseph A Picoraro* ^*2,1*^

^*1*^ *NewYork‐Presbyterian Morgan Stanley Children's Hospital*, *New York*, *NY*; ^*2*^ *Columbia University Vagelos College of Physicians and Surgeons*, *New York*, *NY*

Monitoring of intestinal inflammation in IBD has historically relied on invasive and costly methods such as endoscopy or magnetic resonance enterography (MRE). Intestinal ultrasound (IUS) offers a low‐cost, patient‐centered alternative for the visual evaluation of IBD, however large‐scale data on IUS findings within the pediatric population remain limited.

This retrospective study evaluated 163 point‐of‐care IUS exams performed for pediatric patients with confirmed IBD as a routine part of clinical care, including initial diagnosis, treatment response, and disease monitoring (**Table 1**). IUS was performed by a single IBUS‐certified pediatric gastroenterologist and included standardized examination of the sigmoid colon, descending colon, transverse colon, ascending colon, and terminal ileum with measurement of the following parameters for each segment of examined bowel: bowel wall thickness (BWT), degree of hyperemia, loss of bowel wall stratification (BWS), presence of inflammatory fat, and presence of lymphadenopathy. Any abscesses, fistulas, and strictures were also documented. Disease subtypes examined included Crohn's disease (CD), ulcerative colitis (UC) and very‐early onset IBD (VEOIBD).

IUS showed strong diagnostic utility, as abnormal findings on IUS were significantly correlated with biomarkers of disease activity (C‐reactive protein, erythrocyte sedimentation rate, fecal calprotectin, albumin), lack of endoscopic remission, and symptoms at time of exam (**Fig 1**). Notably, symptomatic presentation at time of exam was a significant predictor of hyperemia on IUS across all disease subtypes and bowel segments. Neither body mass index (BMI) nor age were significantly correlated with BWT of any bowel segment. Concurrent steroid therapy at time of exam did not correlate with decreased BWT or mask the presence of hyperemia; conversely, steroid usage was significantly associated with increased hyperemia within the sigmoid colon, descending colon, and transverse colon, likely confounded by disease severity. IUS also revealed several significant differences among disease subtypes, such as increased BWT of the terminal ileum in those with CD compared to VEOIBD. Additionally, agreement between IUS and endoscopic or radiographic findings varied significantly by subtype; patients with CD had greatest concordance between modalities while those with VEOIBD had lowest concordance. Clinically, abnormal IUS findings were significantly correlated with changes in management at time of exam, defined as medication adjustments or recommendations for repeat imaging.

Overall, these findings support IUS as a useful tool for the evaluation of pediatric IBD that correlates well with other markers of disease activity and may enable timely changes to patient management. This large dataset further elucidates how standard IUS measurements vary with key patient demographics, which is essential for informed interpretation of IUS results in clinical practice. Altogether, this work highlights the promising role of IUS in the personalized care for children with IBD while also underscoring the need for further prospective research to optimize integration into existing clinical frameworks.


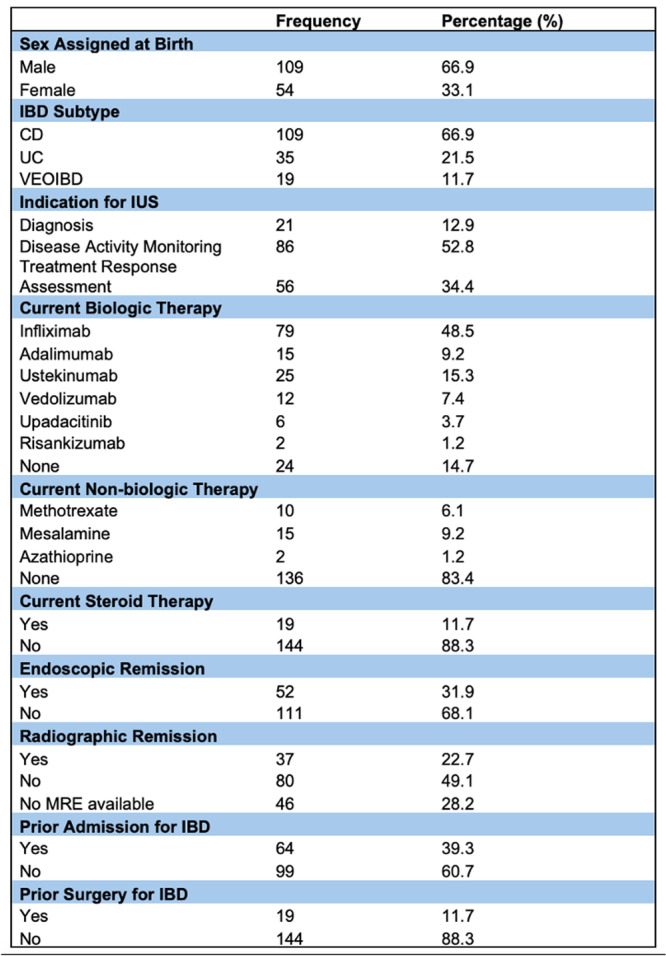


Patient characteristics.


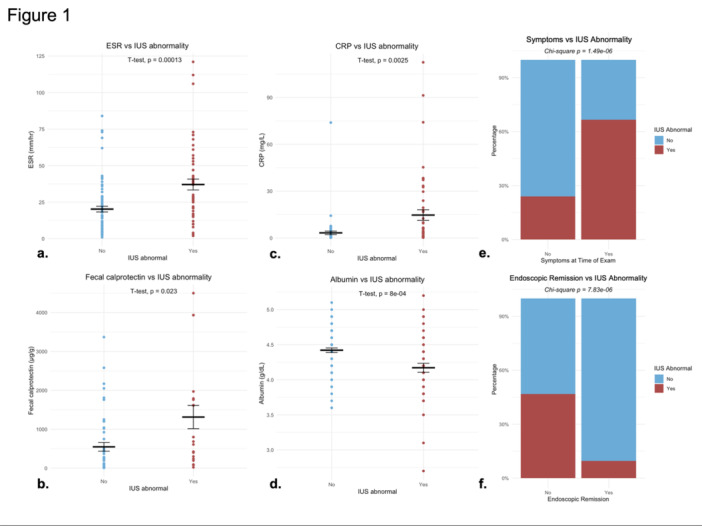


## 746 UNCOVERING THE COMPLEXITIES OF VEO‐IBD: TREATMENT RESPONSES, MUCOSAL HEALING, AND LONG‐TERM OUTCOMES

*Anita Sharma* ^*1,2*^, *David Katibian* ^*1,2*^, *Laura Bauman* ^*1,2*^, *D. Brent Polk* ^*1,2*^, *Jeannie Huang* ^*1,2*^

^*1*^ *Pediatric Gastroenterology*, *Rady Children's Hospital‐San Diego*, *San Diego*, *CA*; ^*2*^ *University of California San Diego*, *La Jolla*, *CA*

Very early‐onset inflammatory bowel disease (VEO‐IBD) is a recognized subset of pediatric IBD. Cohort studies to date have shown that youth with VEO‐IBD exhibit a wide range of responses to a variety of treatments from aminosalicylate to anti‐tumor necrosis factor therapies. Refractory disease is especially common among those with monogenic disease or immune dysfunction.

Given the recent expansion of advanced treatment options, we evaluated the outcomes of our VEO‐IBD patient cohort at a pediatric tertiary care center via a retrospective chart review of patients seen from 2010 to 2024. Descriptive statistical analyses were utilized to report results.

Our VEO‐IBD cohort includes 51 youth. The median age at diagnosis was 4 (interquartile range [IQR]: 3–5) years, with a follow‐up period of 7 (3‐13) years. Thirty (58.8%) are male, 37 (72.5%) White, 7 (13.7%) Hispanic, and 49 (96.1%) prefer to communicate in English. Most (35/51) have Crohn's disease (Table 1). Nine had identified gene mutations potentially predisposing to more severe disease and five had demonstrated immune dysfunction on immunologic testing.

Most patients (41/51, 80.4%) are in steroid‐free clinical remission. Among these, 24 (58.5%) have achieved mucosal healing (MH), and 11 (26.8%) have achieved both MH and histologic remission. Of the remaining 10 patients, 4 have undergone colectomy, 1 is in steroid‐induced clinical remission, 2 have mild disease, 2 moderate disease, and 1 moderate‐severe disease, as determined by physician global assessment (Table 1). Most (32/51, 62.7%) are receiving advanced therapies (Table 2). 36% of the 32 on advanced therapies receive higher‐than‐standard dosing. Among the 33 patients who have tried advanced therapies, the median age at therapy initiation was 7.0 (4.5‐11.8) yrs. Of these, 14 (42.4%) responded to their 1st therapy. Of the 19 who did not respond, 9 did not respond to one advanced therapy, 7 to two advanced therapies and 3 to three advanced therapies. Therapeutic drug monitoring (TDM) was performed in 32 patients, of whom 19 (57.6%) demonstrated low levels requiring dose adjustments or immunomodulator therapy. Responses to therapies attempted are detailed in Table 2.

Achieving MH was not universally associated with sustained treatment response. Among the 43 VEO‐IBD youth who underwent repeat endoscopy, 32 (74.4%) achieved MH. However, four (12.5%) lost response to the therapies on which they had achieved MH.

Our cohort of VEO‐IBD patients highlights key treatment considerations. Less than half responded to their initial biologic therapy. Aggressive biologic dosing was imperative for achieving therapeutic drug levels in our cohort. Also, MH did not always correlate with sustained treatment response. Future research will focus on comparing this cohort with our general pediatric IBD population to further explore treatment outcomes in VEO‐IBD.


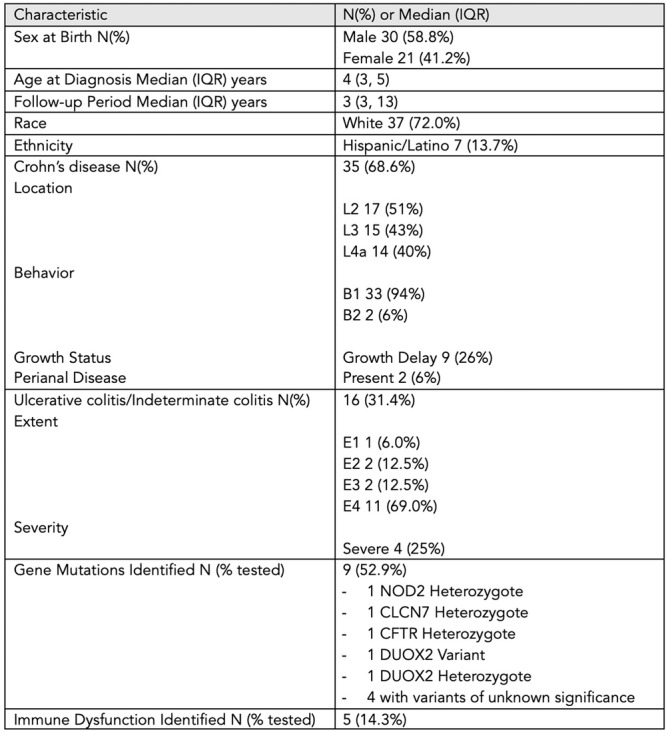


Baseline Characteristics of VEO‐IBD Cohort (N=51)


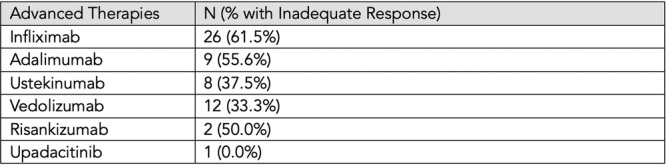


Advanced Therapies Attempted in the VEO‐IBD Cohort and Response

## 747 GENETIC RISK VARIANTS FOR INFLAMMATORY BOWEL DISEASE ARE LESS COMMON IN IMMIGRANT THAN NATIVE‐BORN PATIENTS

*Shagun Sharma* ^*1*^, *Shahzaib Khan* ^*1*^, *Melissa Ramirez Escobar* ^*2*^, *Rebecca Berger‐Gutierrez* ^*3*^, *Anne Levine* ^*1*^

^*1*^ *Pediatric Gastroenterology & Hepatology*, *SUNY Downstate Health Sciences University*, *New York*, *NY*; ^*2*^ *Pediatrics*, *SUNY Downstate Health Sciences University*, *New York*, *NY*; ^*3*^ *SUNY Downstate Health Sciences University*, *New York*, *NY*

**Introduction:** Inflammatory bowel disease (IBD) is a chronic, immune‐mediated disorder with a significant genetic component to disease risk. Less is known about non‐genetic factors that increase risk, though previous studies have shown that patients who immigrate from low to high prevalence countries soon assume local disease risk. At our center, the majority of IBD patients are of Afro‐Caribbean descent, and many are first‐ or second‐generation immigrants. Previous work by our group showed high frequencies of disease‐associated variants in several risk genes, but little data exists regarding the clinical impact of these variants or immigration status on disease outcomes. We aimed to characterize the genetic burden of IBD risk in our population, as well as disease severity and outcomes by immigration status.

**Methods:** We conducted a retrospective chart review of children age 5‐21 diagnosed with IBD at SUNY Downstate Medical Center from 2009‐2024. Data collected from the electronic medical record (EMR) included demographics, ethnic background, immigration status, clinical characteristics, disease phenotype, laboratory results, and the Prometheus Laboratories SGI panel, which reports single nucleotide polymorphisms (SNPs) for IBD risk variants in *ATG16L1*, *ECM1*, *NKX2‐3*, and *STAT3*. We also collected data on disease complications, escalation in therapy (defined as increase in dose, decrease in interval, or change in medication), or surgery. Patients with histologically confirmed IBD and complete SGI panels were included.

**Results:** A total of 25 patients were included, comprising 4 native‐born, 4 first‐generation immigrants, and 17 second‐generation immigrants. Disease parameters are shown in Table 1, including age, BMI percentile, and laboratory values at diagnosis (hemoglobin, albumin, sedimentation rate (ESR), C‐reactive protein (CRP), and fecal calprotectin). There were no significant differences in disease activity between groups. Genetic burden, defined as the total number of SNPs present in each patient, was significantly different between groups (p=0.045). Linear regression showed that genetic burden is higher in native‐born patients than in immigrant patients (p=0.050). We also evaluated disease outcomes between immigration status, including perianal disease, fistula, stricture, need for surgery, and need for therapeutic escalation. Perianal disease was significantly more prevalent in native‐born patients (p=0.037). No significant difference was observed in fistula formation or need for surgery, and no patients developed strictures. Escalation was required in 75% of native‐born, 100% of first‐generation, and 53% of second‐generation patients, with a trend towards less escalation in second‐generation immigrants, but did not reach significance.

**Discussion:** While we previously showed that genetic risk variants are common in our patient population, we now show that native‐born patients with IBD appear to have a significantly higher genetic burden compared to first‐ and second‐generation immigrants. Interestingly, native born patients also appeared to have more perianal disease and require more frequent escalation in therapy. These findings may be due to epigenetic or environmental factors. Native‐born patients may be habituated to the local environment, or have accumulated protective epigenetic changes such that IBD develops in the setting of increased genetic risk, which also may contribute to more complicated or treatment‐resistant disease. Environmentally or epigenetically naïve immigrant patients conversely may develop disease with fewer risk genes in the setting of new deleterious exposures. However, caution is required in interpreting these results as our sample size is small and only a small number of SNPs were included. Large registry‐based genetic and epigenetic population studies are needed.


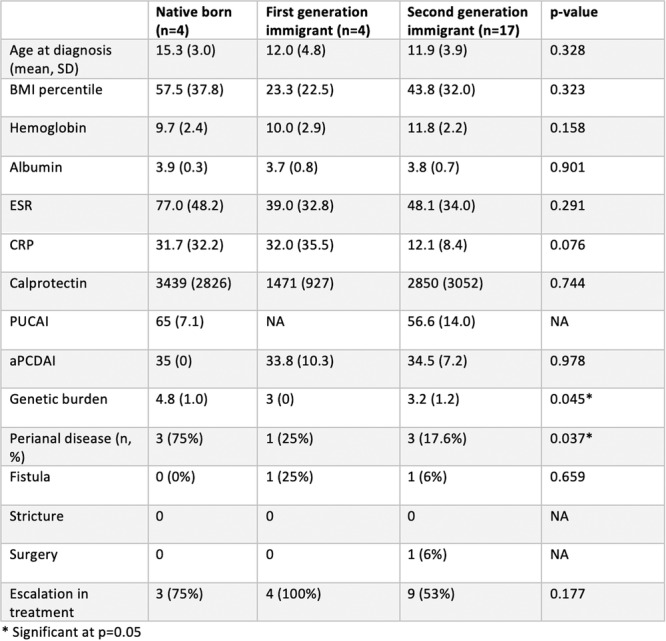


Table 1. Disease characteristics of patients

## 749 CLINICAL OUTCOMES OF TACROLIMUS THERAPY IN PEDIATRIC STEROID‐RESISTANT ULCERATIVE COLITIS: A RETROSPECTIVE SINGLE‐CENTER STUDY

*Yugo Takaki*, *Yoshihiko Sugino*, *Takahiro Yamashita*

*Department of Pediatric Gastroenterology and Hepatology*, *Kumamoto Sekijuji Byoin*, *Kumamoto*, *Kumamoto Prefecture*, *Japan*

**Background:** In recent years, the number of pediatric cases of steroid‐resistant ulcerative colitis (UC) requiring molecular‐targeted therapies has been increasing. Tacrolimus (Tac), a calcineurin inhibitor that acts on T lymphocytes, remains an important treatment option due to its rapid onset and efficacy, even in the current era of molecular‐targeted therapies.

**Aim:** To evaluate the prognosis of children with steroid‐resistant UC treated with Tac and to assess the appropriateness of initial remission induction therapy.

**Methods:** This retrospective study included pediatric patients with steroid‐resistant UC who received Tac at our institution between April 2018 and May 2025. Data collected included sex, age at diagnosis, highest Pediatric Ulcerative Colitis Activity Index (PUCAI) score from first visit to Tac initiation, disease extent, duration of Tac treatment, remission induction rate, adverse events, relapse after Tac discontinuation, current maintenance therapy, and need for surgical intervention.

**Results:** A total of 10 patients (male:female ratio 1:1; median age 12 years, range 2–15) were included. The median PUCAI score was 57.5 (range 30–85). Eight patients had E4‐type UC, and two had E2‐type. The median duration of Tac therapy was 73 days (range 4–98), and remission was achieved in all but one patient who discontinued treatment due to hypertensive encephalopathy. No other adverse events were observed. Relapse occurred in four patients after Tac withdrawal. Among the five non‐relapsing cases, one maintained remission with thiopurines, and four had already initiated molecular‐targeted therapy during Tac treatment. At the time of analysis, seven patients were on molecular‐targeted therapy, two on traditional Chinese herbal medicine, and one on thiopurines. No patients required total colectomy.

**Conclusion:** Most patients treated with Tac eventually required molecular‐targeted therapy for sustained remission. Initiating molecular‐targeted therapy during Tac administration was associated with prolonged remission. Although cost‐effectiveness should be considered, these findings suggest that post‐Tac maintenance strategies warrant reevaluation.

## 755 CREATING CELIAC‐FRIENDLY SCHOOLS: ENHANCING ENVIRONMENTS FOR CHILDREN'S HEALTH

*Shanaz Daneshdoost*, *Peacha Sokzini*, *Ashley Dunn*, *Anava Wren*, *Farah Mardini*, *Javier Lopez Rivera*, *Nasim Khavari*, *Hilary Jericho*

*Pediatric Gastroenterology, Hepatology, and Nutrition*, *Stanford University School of Medicine*, *Stanford*, *CA*

**Background:** Strict adherence to a gluten‐free diet (GFD) is the only effective treatment for celiac disease (CeD), yet maintaining this diet is especially difficult for children and adolescents in school settings, where limited gluten‐free options and social pressures are common. Non‐adherence can result in severe CeD symptoms and reduced quality of life. Despite these challenges, research on the impact of school environments on GFD adherence is limited and often fragmented. This mixed methods study uses an integrated design to examine the personal, social, and structural barriers to GFD adherence in school settings. A cross‐sectional survey, paired with interviews and focus groups, will identify high‐risk situations and predictors of non‐adherence in school. Findings will inform the development of targeted GFD educational resources for families, educators, and school personnel, addressing current gaps in support.

**Methods:** Participants will include Stanford Children's Health patients (ages 4–18) enrolled in California schools with a confirmed diagnosis of celiac disease (CeD), as well as school personnel with a minimum of two years in their current role. Surveys will assess school‐specific adherence behaviors (adapted from the CeliacKids Study), food security (Hunger Vital Sign), and CeD‐specific quality of life (CDDUX). Quantitative analyses will include descriptive statistics, t‐tests, and chi‐square tests to identify factors associated with dietary non‐adherence. Qualitative data from in‐depth interviews with participants will be analyzed thematically using NVivo, with methodological rigor ensured through independent coding and peer debriefing. Integrated analysis will triangulate survey and interview data to identify convergent barriers and facilitators of GFD adherence.

**Anticipated Results:** Power analyses indicate that with an expected response rate of 50% from 311 eligible patients in the Stanford Children's Health Epic Slicer Dicer registry, the study will achieve 80% power to detect associations between key psychosocial, demographic, and environmental factors and GFD adherence, with effect sizes as small as 0.32 (continuous variables, t‐test) and 0.25 (categorical variables, chi‐square). Data collection is ongoing as of May 15, 2025 (current N=52). Integrated findings from quantitative and qualitative data will inform the development of structured, evidence‐based educational resources using Kern's 6‐Step Curriculum Development framework.

**Conclusion:** This mixed methods study will highlight the personal, social, and structural barriers to GFD adherence in school settings. The integrated findings from quantitative and qualitative data will address a critical gap in support mechanisms for pediatric patients with CeD and inform the development of structured, evidence‐based educational resources. These resources will be tailored to the needs of families and school personnel, with the goal of improving dietary adherence and student well‐being. Additionally, study results will inform clinical care and support policy development to better support pediatric patients with CeD. By bridging traditional silos between healthcare, education, and public health, this work provides a scalable model for enhancing chronic disease management in school settings.

## 757  A SINGLE CENTER RETROSPECTIVE ANALYSIS OF THE UTILITY OF DISACCHARIDASE TESTING DURING UPPER ENDOSCOPY

*Rudy El Asmar* ^*1*^, *Adam Himmelrick* ^*1*^, *Stephen Roper* ^*2*^, *Phillip Tarr* ^*1*^, *Laura Duckworth* ^*1*^

^*1*^ *Pediatrics*, *Washington University in St Louis School of Medicine*, *St. Louis*, *MO*; ^*2*^ *Laboratory and Genomic Medicine*, *Washington University in St Louis School of Medicine*, *St. Louis*, *MO*

**Background:** Enzymes such as lactase, sucrase, palatinase/isomaltase, and maltase, split disaccharides into monosaccharides prior to absorption in the small intestine^[1]^. Deficiencies of these disaccharidases (DS) can cause nonspecific gastrointestinal symptoms such as abdominal pain, bloating, or diarrhea^[1]^, or be asymptomatic. There are multiple methods of testing for DS deficiency, including empiric trials (dietary elimination and assessing symptom response), breath testing, and enzyme activity measurement in duodenal tissue (obtained via esophagogastroduodenoscopy (EGD))^[2]^. Testing during EGD requires > two additional biopsies from the duodenum and adds cost to the procedure, but the utility of these results and their effect on subsequent medical management is not well understood^[1]^. Here, we report a single center series focused on the performance and interpretation of tissue DS activity obtained during EGD in children and adolescents, to evaluate the reliability and clinical relevance of the resulting values.

**Methods:** We conducted a retrospective review of children who underwent DS testing on tissue obtained during EGD at St. Louis Children's Hospital between May 2022 and August 2024. Demographic variables, clinical indications, DS (lactase, sucrase, palatinase/isomaltase, maltase) activity, duodenal histology, and management outcomes were collected. The cutoff values for the normal range of each DS were laboratory‐specific (lactase <14, sucrase<25, maltase<135, palatinase<8.5 nmol/min/mg protein). All tissue was assayed using an enzymatic spectrophotometric method after shipment on dry ice.

**Results:** A total of 1,280 DS assays from 1,274 patients were analyzed. The median age was 14 years (range: 4 months to 23 years). Activities of all four enzymes were below the cutoffs for normal in 495 (38.7%) patients; with 311 (24.3%) of these >25% below the lower limit of normal. Of these 311 with pan‐low results, 275 had normal duodenal histopathology. Isolated lactase deficiency, defined as < the cut‐off for this enzyme with all other enzyme activities normal, was documented in 185 patients (14.5%). Tissue from only 265 (20.7%) of the patients had normal activity across all four enzymes. Results stratified by age are presented in Table 1, and enzyme‐specific findings are illustrated in Figure 1.

**Conclusions:** Our center's rate of pan‐disaccharidase deficiency is greater than previously reported ^[2]^, though the normal histology accompanying most of these biopsies argues against such an entity as being related to pathology. Improper specimen handling can reduce DS activity, but our center follows rigorous protocols that include obtaining two distal small bowel biopsies and immediate placement of specimens on dry ice in pre‐chilled polypropylene. Another possibility is that our customary sampling protocol recovers inadequate tissue to accurately determine concentrations. Further investigation is warranted to determine the clinical relevance of these findings with a focus on how often test results alter patient management, if such management improves outcomes, and if disaccharidase testing is superior to empiric withdrawal of lactose from the diet or breath hydrogen testing. Future directions include stratifying results by race and ethnicity, evaluating outcomes and management strategies in patients with PDD, and considering a cost‐benefit analysis of DS testing in routine clinical practice.

**References:**

[1]Reed RC, Pacheco MC. Clinical and histopathologic predictors of disaccharidase deficiency in duodenal biopsy specimens. *Am J Clin Pathol*. 2019;152:742.

[2]Daileda T, et al. Disaccharidase activity in children undergoing esophagogastroduodenoscopy: a systematic review. *World J Gastrointest Pharmacol Ther*. 2016;7:283.

[3]Viswanathan L, Rao SS. Intestinal disaccharidase deficiency in adults: evaluation and treatment. Curr Gastroenterol Rep. 2023;25:134.


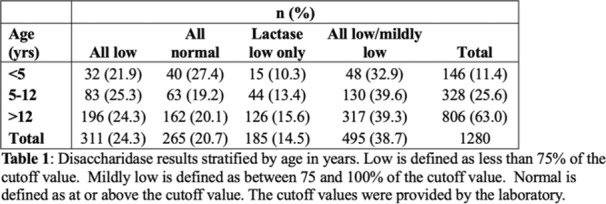



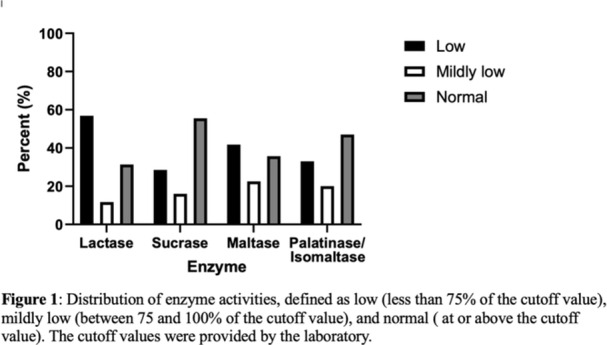


## 759 UNDERSTANDING CELIAC DISEASE IN ADOLESCENTS: A SURVEY EXPLORING CHALLENGES IN DIAGNOSIS AND ADHERENCE TO THE GLUTEN‐FREE DIET

*Simon Gidrewicz* ^*2*^, *James King* ^*3*^, *Dominica Gidrewicz* ^*1*^

^*1*^ *Pediatrics*, *University of Calgary Cumming School of Medicine*, *Calgary*, *AB*, *Canada*; ^*2*^ *Renert Centre*, *Calgary*, *AB*, *Canada*; ^*3*^ *Centre for Health Informatics*, *University of Calgary*, *Calgary*, *AB*, *Canada*

Adolescents with celiac disease (CeD) often struggle to follow a strict gluten‐free diet (GFD). This study aimed to explore their experiences and challenges.

**Methods:** A 34‐question survey targeting adolescents aged 12 to 19 with a confirmed diagnosis of celiac disease was distributed through Celiac Canada's nationwide mailing list and shared on their social media platforms. The survey evaluated various topics, including demographics, symptoms at diagnosis, peer pressure, eating habits, and quality of life.

**Results:** 134 adolescents from across Canada completed the survey, of which 67% were female. The mean age was 15.2 +/‐ 2.3 years. The majority were diagnosed via blood work and intestinal biopsy (81, 60.4%), 46 (34%) via blood work alone, 9 (6.7%) via another diagnostic method. Almost half of the participants (61, 45.5%) were symptomatic for greater than 2 years before diagnosis. The most common symptoms at diagnosis included abdominal pain/cramping (74.6%, of which 14.0% had unresolved symptoms on a GFD), bloating or gas (67.9%, 17.6% unresolved symptoms), fatigue (68.7%, 25.0% unresolved symptoms) and poor weight gain/growth (60.5%, 18.5% unresolved symptoms) (Table 1). Neurological symptoms were also common at diagnosis, including headaches/migraines (48.5%), difficulty concentrating (54.5%), self‐reported depression/anxiety (51.5%) (Table 1). While most respondents reported strict adherence to a GFD (107, 79.9%), one‐third face major difficulties adhering to their diet (44, 32.8%). Suprisingly, 52 (38.8%) stated they would break their GFD if they remained asymptomatic after consuming gluten. When asked about reasons for breaking their diet, 101 (75.3%) cited accidental gluten consumption. However, 18 (13.4%) reported peer pressure as a factor, noting they consumed gluten to fit in socially. Feelings of exclusion were also common: 90 respondents (66.7%) reported feeling left out when others shared non‐GF food, and 39 (43.3%) admitted that this sense of exclusion led them to break their GFD.

**Summary:** Delays in CeD diagnosis are common among adolescents, and atypical, including neurologic, symptoms are frequent in this age group. Raising awareness of these non‐classical presentations among primary care providers is essential for timely diagnosis. Many adolescents struggle to follow a GFD, and surprisingly over 1/3 would abandon it if they remained asymptomatic after gluten ingestion. Feelings of isolation and exclusion related to their dietary restrictions are also common. These results underscore the need for continued support and advocacy to improve the long‐term well‐being of adolescents living with CeD.


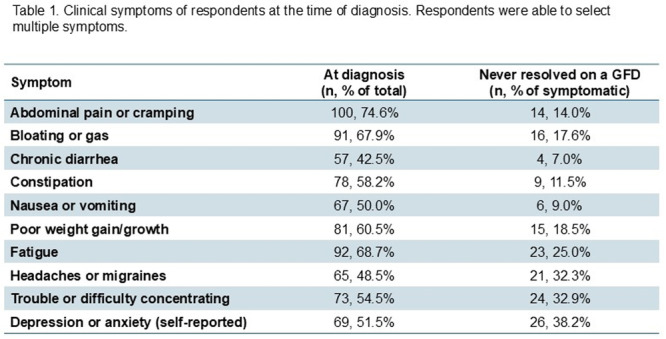


## 761 GETTING TO THE HEART OF CELIAC DISEASE: INCREASED RISK OF CARDIOMYOPATHY IN HOSPITALIZED CHILDREN

*Benjamin Jack* ^*1*^, *Brian Hughes* ^*1*^, *Anthony Marrama* ^*2*^, *Apryl Susi* ^*2*^, *Cade Nylund* ^*1*^

^*1*^ *Pediatrics*, *Walter Reed National Military Medical Center*, *Bethesda*, *MD*; ^*2*^ *Henry M. Jackson Foundation for the Advancement of Military Medicine*, *Bethesda*, *MD*

**Background:** Celiac disease (CD) is a chronic autoimmune disorder triggered by the ingestion of gluten in genetically predisposed individuals. It primarily affects the small intestine, but its systemic manifestations have been increasingly recognized. The etiology of cardiomyopathy, a condition characterized by the weakening of the heart muscle, is thought to be multifactorial and is associated with such as genetic conditions, autoimmune conditions, congenital heart disease, metabolic disease, and environmental or infectious exposures. However, its association with CD in children has not been well established. Understanding this potential relationship is crucial for early detection and management of cardiovascular complications in pediatric patients with CD. This study aimed to evaluate the prevalence and association of cardiomyopathy in children with celiac disease using a large, nationwide pediatric hospitalization dataset.

**Methods:** We conducted a retrospective cohort study using the Healthcare Cost and Utilization Project Kids' Inpatient Database (HCUP‐KID) from 1997 to 2022, which includes data from over 26 million pediatric hospitalizations across the United States. We identified hospitalizations involving children aged 1–20 years with a diagnosis of CD (ICD‐9 code 579.0; ICD‐10 code K90.0) and cardiomyopathy diagnosis (cardiomyopathy, dilated cardiomyopathy, restrictive cardiomyopathy, or viral cardiomyopathy), excluding hypertrophic cardiomyopathy (ICD‐9 codes: 425.4, 425.9, 425.8; ICD‐10 codes: I43, I42.0, I42.5, I42.8, I42.9 and B3324) were identified. Multivariable logistic regression models were used to assess the association between CD and cardiomyopathy while adjusting for age, sex, race, hospital region and insurance payer type. Appropriate survey analysis methods were used to calculate national estimates and statistical tests.

**Results:** Among the estimated 21,201,301 US national pediatric hospitalizations, within the study age range, we identified 18,863 hospitalizations with a diagnosis of CD and 44,604 with cardiomyopathy (Table 1). Of those with CD, 0.34% (n=64) had a concurrent diagnosis of cardiomyopathy. In comparison, the group without CD had a cardiomyopathy rate of 0.21% (n=44,540). Of note, 0.0014% of those with cardiomyopathy also had a diagnosis of CD. Subjects who were male (adjusted odds ratio [aOR], 2.17; 95% confidence interval [CI] 2.10‐2.23), Black race vs White race (aOR, 1.63, 95% CI, 1.58‐1.68) and Asian or Pacific Islander vs White race (aOR,1.83; 95% CI, 1.70‐1.97) were at increase odds of hospitalization with a diagnosis of cardiomyopathy. Children with CD had a significantly higher odds of being diagnosed with cardiomyopathy (aOR, 1.80; 95% CI, 1.27–2.54, p<0.001).

**Conclusions:** Although rare, this large‐scale analysis of pediatric hospitalizations demonstrates a significant association between CD and cardiomyopathy in hospitalized children. These findings suggest the possible need for heightened or routine cardiovascular surveillance in pediatric patients with CD. However, screening for CD among those with newly diagnosed cardiomyopathy is unlikely to be beneficial. Further studies are warranted to elucidate the underlying mechanisms of this association and to assess whether early interventions may reduce the risk of cardiomyopathy in these patients.


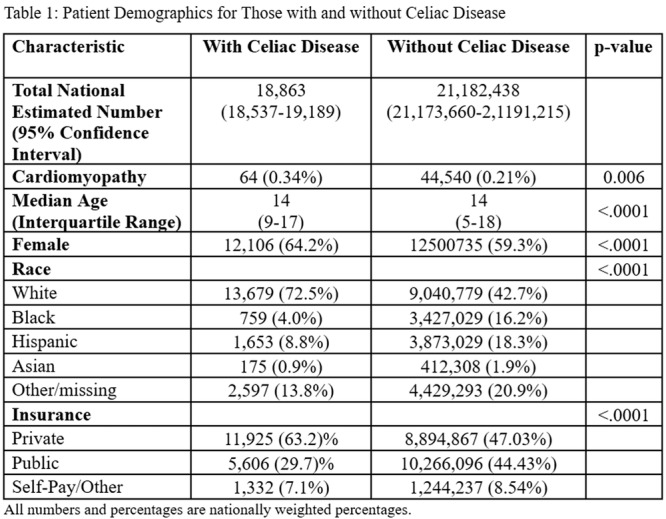


## 763 COLONIC DIVERTICULITIS IN THE PEDIATRIC POPULATION: A RETROSPECTIVE STUDY OF NINETEEN PATIENTS

*Jessica Lat‐Pfeiffer* ^*1*^, *Neha Malhotra* ^*2*^, *Graciela Wetzler* ^*2*^, *Vivian Tang* ^*2*^

^*1*^ *Pediatrics*, *Maimonides Medical Center*, *New York*, *NY*; ^*2*^ *Pediatrics Gastroenterology*, *Maimonides Medical Center*, *New York*, *NY*

**Objective:** Colonic diverticulitis (CD) is a prevalent condition among adults but remains rare and poorly defined in the pediatric population. Unlike adults, children present with symptoms mimicking appendicitis, which can delay accurate diagnosis. While the conservative management of acute uncomplicated CD with antibiotics is well‐supported in adults, no standardized guidelines currently exist in the pediatric population. This study examines the characteristics, diagnosis and management of CD in children.

**Methods:** This is a single‐center, retrospective review of 19 patients under 21‐years‐old diagnosed with diverticular disease (DD) from January 2013 to May 2023 at Maimonides Children's Hospital. Data collected and analyzed were patient demographics, symptoms, blood tests, diagnostic imaging, lower endoscopy and therapeutic management.

**Results:** Nineteen patients were diagnosed with DD through abdominal CT scans, with 11 cases (58%) identified as CD and 8 cases with diverticulosis. The cohort consisted of 11 male patients (58%), with a median age of 18 years, ranging from 12 to 20 years. Abdominal pain was the most common presenting symptom for patients with CD followed by nausea and diarrhea. Abdominal pain was caused by a different diagnosis for those with diverticulosis and most common presenting symptoms was constipation. Only one patient developed fever. There were 47% of cases requiring hospital admission for intravenous antibiotics. Six patients underwent abdominal ultrasound, three of whom were diagnosed with CD. One patient underwent MRE, and 4 patients had a colonoscopy. Only 1 patient (5%) developed recurrence and complicated diverticulitis.

**Conclusion:** DD in the pediatric population is rare, but its prevalence is increasing. Diverticulosis presents with non‐toxic symptoms and abdominal pain is commonly caused by a different etiology. CD present with abdominal pain but unlike in surgical cases, other symptoms are less toxic appearing. Abdominal CT scans consistently identified DD, and most patients responded well to antibiotic treatment. The use of abdominal US could be explored further as a safer, non‐invasive diagnostic tool for CD. However, MRE and colonoscopy did not provide additional information in patient care. Further studies and collaboration are needed to establish standardized clinical guidelines for the management of CD in children, to reduce variability and enhance our understanding of the disease process in this population.


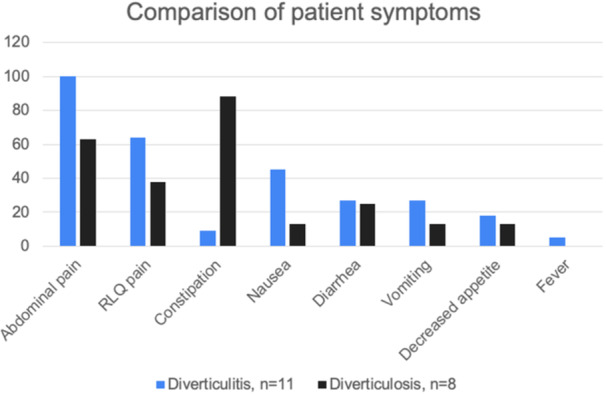


Fig 1. Patient symptoms CD vs diverticulosis


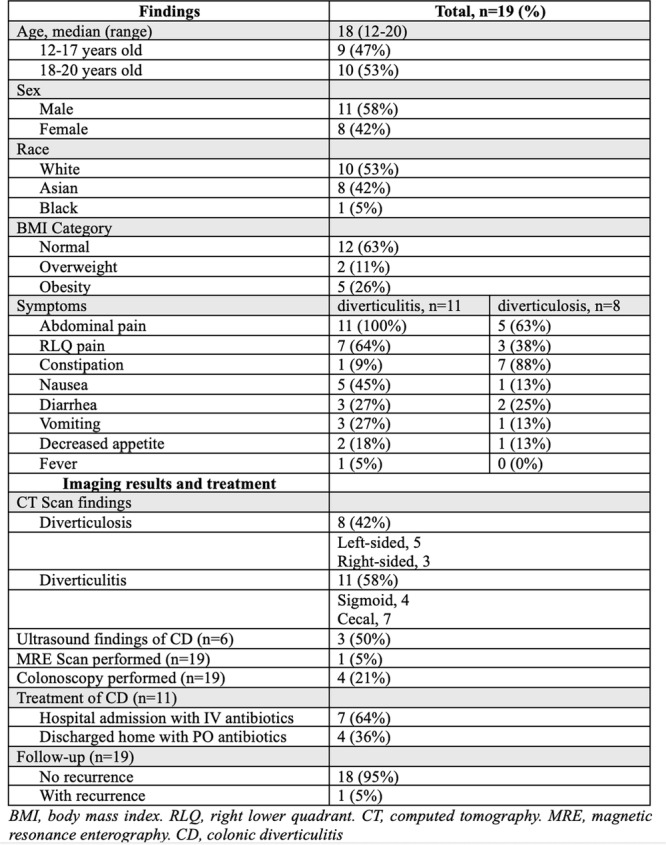


Table 1. Demographics, signs and symptoms, diagnostic studies and management of patients with CD and diverticulosis

## 764 IN THE SHADOW OF CARE ‐ A CLOSER LOOK AT THE IMPACT ON SIBLINGS OF CHILDREN LIVING WITH CELIAC DISEASE

*Danica Mathew* ^*1*^, *Priyanka Chugh* ^*2*^, *Shannon Meng* ^*1*^, *Ritu Verma* ^*1*^, *Aanya Gupta* ^*1*^, *Ainsley DeRosa* ^*1*^, *Rebecca Auburn* ^*1*^, *Rachel Lieberman* ^*1*^, *Lindsey Yeakle* ^*1*^

^*1*^ *Pediatric Gastroenterology*, *The University of Chicago Division of the Biological Sciences*, *Chicago*, *IL*; ^*2*^ *Boston Medical Center*, *Boston*, *MA*

**Introduction:** Much research has focused on the physiology and management of celiac disease. However, not much research has looked at family dynamics of celiac disease, particularly siblings that do not carry the diagnosis. Studies in other chronic diseases have also reported that healthy siblings of children with chronic diseases have higher susceptibilities to emotional and behavioral difficulties. This study evaluates self‐reported psychosocial effects on non‐celiac siblings of patients with celiac disease, particularly related to annoyance with making lifestyle changes and receiving less attention from parents.

**Methods:** A cross‐sectional survey was developed and piloted to 8 non‐celiac siblings of celiac disease patients. The survey was then electronically disseminated to families who have established an affiliation with the University of Chicago Celiac Disease Center. Respondents were all non‐celiac siblings of celiac disease patients diagnosed before the age of eighteen. Each participant completed a survey including questions on family demographics, medical family history, and questions related to the effect of the celiac diagnosis on the non‐celiac sibling. Questions focused on feelings of less attention, annoyance around the sibling with the celiac disease diagnosis, anxiety around the siblings' diagnosis, disordered eating patterns of the non‐celiac sibling, and episodes of disruptive behavior related to the diagnosis.

**Results:** This study included 174 participants. The non‐celiac sibling ages ranged from 6‐26 years old. The majority (56%) of the sibling respondents were in the 10‐14 year range. It was found that 58% of non‐celiac siblings aged 10‐14 years old generally felt they received less attention from parents compared to their sibling with celiac disease. We additionally found that 69% of non‐celiac siblings age 10‐14 years old felt annoyance with changes in their daily lives (such as maintaining a safe home, limiting choices in restaurants, and creating new family traditions related to food) Finally, of this group we found 100% of this age cohort did not feel guilty for not having celiac disease.

**Discussion:** This study highlights that non‐celiac siblings of patients with celiac disease often have psychosocial effects related to making changes in the home and feeling they receive less attention. This detailed evaluation of non‐celiac siblings aged 10‐14 years old illustrates the importance of addressing the needs of siblings in managing chronic illnesses. Based on this study we have found that a chronic condition, such as celiac disease, that involves making changes in the home, can be associated with feelings of guilt and annoyance from siblings. We would recommend extending resources for the entire family and addressing the emotional aspects, alongside clinical care needs for the patient. Future investigation will focus on these mental health concerns and family dynamics to develop support systems and create interventions for healthy siblings.

**Sources for abstract:**

Posner, E.B., Haseeb, M. Celiac Disease. [Updated 2023 Aug 8]. In: StatPearls [Internet]. Treasure Island (FL): StatPearls Publishing; 2024 Jan‐. Available from: https://www.ncbi.nlm.nih.gov/books/NBK441900/

Russo, C., Wolf, R.L., Leichter, H.J. *et al*. Impact of a Child's Celiac Disease Diagnosis and Management on the Family. *Dig Dis Sci* **65**, 2959–2969 (2020). https://doi.org/10.1007/s10620-020-06316-0

Keywords: sibling, celiac disease, educational program development, educational program, celiac, teen care


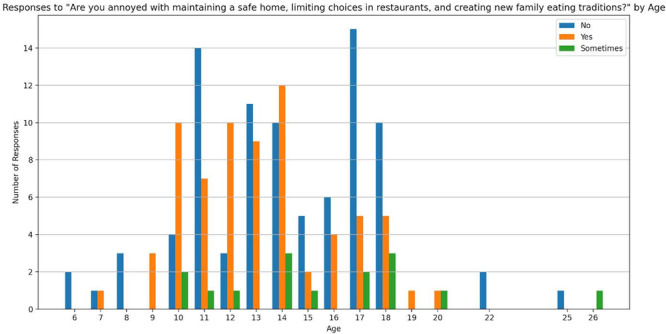


## 766 EXPOSOMIC INSIGHTS INTO ENVIRONMENTAL FACTORS INFLUENCING CELIAC DISEASE RISK

*Max Manwaring‐Mueller* ^*2*^, *Victoria Kenyon* ^*1*^, *Kevin Schneider* ^*3*^, *Matias Fuentealba* ^*3*^, *Francesco Valitutti* ^*4*^, *Ali Zomorrodi* ^*1*^, *Daniel Winer* ^*3*^, *Alessio Fasano* ^*1*^, *Maureen Leonard* ^*1*^, *David Furman* ^*3*^

^*1*^ *Pediatric GI*, *MGH*, *Boston*, *MA*; ^*2*^ *Buck Institute for Research on Aging Furman Lab*, *Novato*, *CA*; ^*3*^ *Buck Institute for Research on Aging*, *Novato*, *CA*; ^*4*^ *Pediatrics Section, Depart. of Medicine and Surgery, University of Perugia*, *Perugia*, *Italy*

**Introduction:** Approximately 30% of the general population carries the HLA‐DQ2 or DQ8 alleles, yet celiac disease (CeD) incidence continues to rise, suggesting a role for environmental factors. Studies on factors like breastfeeding and gluten introduction have yielded conflicting results, raising questions about whether feeding practices meaningfully influence CeD pathogenesis. Using data from a multi‐center, prospective study of infants at risk for CeD, we conducted multivariate survival analyses to identify environmental factors associated with CeD risk and applied linear mixed‐effects models to examine longitudinal relationships between these factors, microbiota composition, and CeD outcomes.

**Methods:** Using data from the Celiac Disease Genomic Environmental Microbiome and Metabolomic (CDGEMM) study, we evaluated the influence of 62 early‐life environmental exposures on CeD conversion, derived from longitudinal, parent‐completed questionnaires encompassing demographics, medical history, environmental exposures, and dietary habits. Multivariate Cox proportional hazards models, adjusted for genotype, nationality, sex, birth order, and dietary factors (e.g., breastfeeding, gluten intake), identified environmental exposures linked to CeD risk (Figure 1). Sample sizes varied due to missing data. To explore potential mechanisms, we then used linear mixed‐effects models to assess associations between environmental factors and microbiota shifts in matched CeD cases (n=31) and controls (n=31) at the time of conversion (Figure 2).

**Results:** Among 423 subjects, 56 (age 38.4 ±18.8 months; 70% female) seroconverted to CeD. Key environmental factors during the first 15 months were identified (Figure 1). Breastfeeding within the first six months was a nominally protective factor (HR: 0.59, p=0.068) and significant among Italians (HR: 0.42, p=0.041). Increased gluten intake between 12–15 months raised CeD risk (HR: 1.31, p=0.014).

Mixed‐effects models revealed nominally significant microbiota differences in cases vs. controls and in relation to breastfeeding. Breastfeeding between 0–6 months was associated with enrichment of *Bacteroides fragilis* and reduction of *Ruminococcus gnavus* (Figure 2). Notably, *R. gnavus* and *Mediterraneibacter* were enriched in converters. These findings suggest breastfeeding may influence gut microbiota in ways that modulate CeD risk.

**Conclusions:** Increased gluten intake between 12–15 months elevates CeD risk, while breastfeeding in the first six months appears protective. Breastfeeding was associated with microbiota shifts that may protect against the enrichment of inflammatory taxa such as *R. gnavus*. These findings highlight the importance of revisiting environmental factors in CeD pathogenesis, particularly the role of infant feeding practices in shaping microbiota trajectories.

Future work will apply the Metagenomic Estimation of Dietary Intake (MEDI) pipeline to refine dietary exposure estimates, conduct mediation analyses to quantify the role of microbiota in CeD risk, and examine microbiome diversity leading up to conversion.


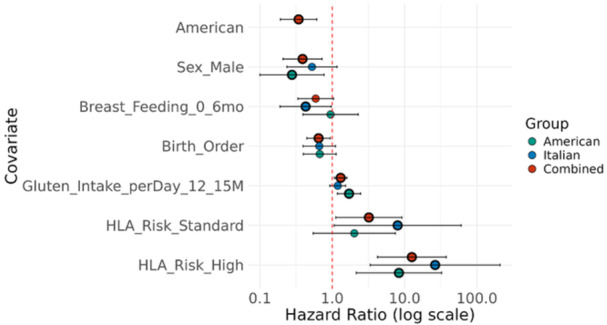


**Figure 1. Multivariate Cox Analysis of Celiac Disease Risk Factors (First 15 Months Post‐Birth)** Breastfeeding within the first six months was a borderline protective factor in the full cohort (HR: 0.59, p=0.068, 95% CI: 0.335–1.04) and statistically significant among Italians (HR: 0.42, p=0.041, 95% CI: 0.189–0.969). Higher gluten intake between 12–15 months increased CeD risk (HR: 1.31, p


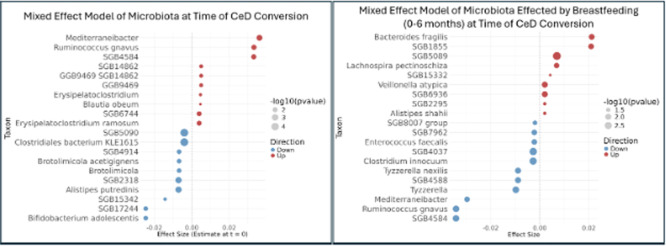


**Figure 2. Microbiota Differences at CeD Conversion and in Relation to Breastfeeding (0–6 Months):** Longitudinal mixed‐effects models revealed nominally significant differences in gut microbiota composition at CeD conversion. The left panel shows taxa differing between cases and controls; the right panel compares microbiota in children breastfed during the first six months vs. not, controlling for conversion. Circle size indicates association strength (‐log10 p‐value), color shows direction (red = enriched, blue = reduced).

## 768 CHRONIC DIARRHEA IN INFANTS: DIAGNOSTIC UNCERTAINTY, RARE DISEASE BURDEN, AND MORTALITY IN THE ICD‐10 ERA

*Paul Wasuwanich* ^*2*^, *Wikrom Karnsakul* ^*1*^, *Sara Nandolia* ^*1*^, *Brett Hoskins* ^*3*^

^*1*^ *Pediatric Gastroenterology*, *Johns Hopkins University*, *Baltimore*, *MD*; ^*2*^ *Department of Internal Medicine*, *Naples Comprehensive Health*, *Naples*, *FL*; ^*3*^ *Division of Pediatric Gastroenterology, Hepatology, and Nutrition, Department of Pediatrics*, *Riley Hospital for Children at IU Health*, *Indianapolis*, *IN*

**Abstract**

**Background:** Chronic diarrhea (>3 weeks) in infants presents diagnostic complexity due to overlapping features, rare causes, and reliance on delayed or invasive tests. Many admissions remain coded as “unspecified,” potentially masking congenital, genetic, or immune‐mediated conditions.

**Methods:** We analyzed U.S. National Inpatient Sample (NIS) data from 2015–2022 for hospitalizations in children ≤2 years with chronic diarrhea. Diagnoses included congenital sucrase‐isomaltase deficiency (CSID), lactase deficiency, inflammatory bowel disease (IBD), food protein‐induced syndromes, and unspecified diarrhea. Incidence was calculated per 10,000,000 U.S. population. Median hospital length of stay (LOS), total charges, and in‐hospital mortality were summarized per hospitalization, not per individual.

**Results:** Lactase deficiency had the highest incidence (154.5/10 M) with 0% mortality, likely reflecting secondary disaccharidase deficiency and not as the primary cause of diarrhea. CSID remained rare (18.2/10 M) but incurred higher median LOS (6 days, IQR 2–19) and charges ($32,623; IQR $19,105–$90,004). Ulcerative colitis showed the highest burden per admission (LOS 8 days, charges $76,792) with a 4.08% mortality rate. Crohn's disease had a 1.75% mortality rate. Notably, unspecified diarrhea was the most frequent diagnosis and accounted for 225 deaths, with a per‐admission mortality rate of 0.37%. Genetic conditions such as CSID and congenital lactase deficiency are now detectable on commercial panels, but slow turnaround (3–4 weeks) limits impact during index admission.

**Conclusions:** In the ICD‐10 era, chronic diarrhea remains frequently undercoded or unspecified in infants, leading to diagnostic delay and measurable in‐hospital mortality. Hospital‐level data reflect readmissions and complex care trajectories. Earlier pattern recognition, coupled with faster access to genetic and histologic diagnostics, may reduce cost and mortality while supporting targeted management.


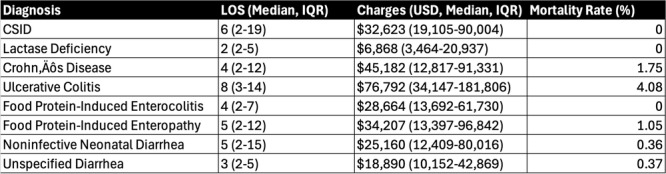



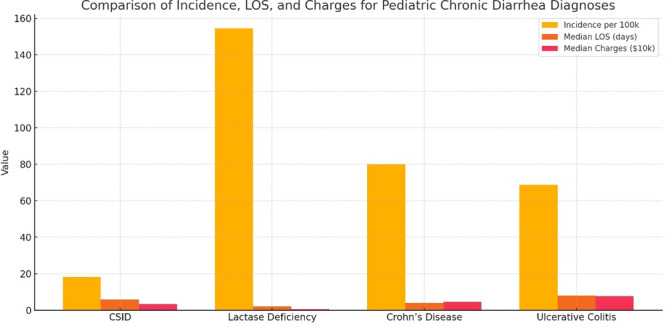


## 771 FEASIBILITY OF SEDATION‐FREE TRANSNASAL ENDOSCOPY IN THE DIAGNOSIS OF CELIAC DISEASE

*Yamen Smadi*, *Sharef Al‐Mulaabed*, *Vijay Mehta*

*Pediatric Gastroenterology*, *Orlando Health Arnold Palmer Hospital for Children*, *Orlando*, *FL*

**Background:** Small bowel endoscopy and biopsies are an important tool in the evaluation of celiac disease. We aimed to evaluate the feasibility of transnasal esophagogastroduodenoscopy (TN‐EGD) using ultra‐slim, single use, gastroscopes in the diagnosis of celiac disease.

**Methods:** Subjects ages 8‐22 years of age with suspected or confirmed celiac disease based on abnormal tissue transglutaminase (TTG) were recruited to participate. Sedation‐free TN‐EGD was performed as previously described using the EvoEndo Model LE gastroscope (Grayslake, Il) in either a sitting or left lateral position. Size and age‐appropriate dosing of lidocaine 4% spray in the nose and throat was used along with virtual reality distraction and dissociation when preferred by the subject. Outcome was measured by successful completion of the procedure, tolerance as measured by TNEase Score, convenience and time to complete procedure. Study was approved by IRB # 23.152.08 and gastroscopes were granted by EvoEndo, Inc.

**Results:** Six subjects (3 males) were enrolled (mean age 14 years, range 10‐20 years, mean weight 51 kg). TN‐EGD was successful in 4 subjects (67%). Both subjects who failed duodenal intubation had type 1 diabetes. All duodenal biopsies were adequate for pathology analysis. Average duration of the procedure was 34 minutes (range 25‐45). All patients and their caregivers were satisfied by the procedure. TNEase score was rated as 1 from 1 to 3 with 1 being with ease. Average endoscopy team satisfaction rate was 4.6 on a scale from 1 to 5 with five being most satisfied (Table 1).

**Conclusions:** In this pilot study, sedation‐free TN‐EGD was successful in the diagnosis of celiac disease in 2 thirds of the subjects. Comorbidity of type 1 diabetes was associated with unsuccessful duodenal intubation. Unsedated TN‐EGD saved patients sedation in cases of false positive elevated TTG.

**Acknowledgement:** Thank you to Joel Friedlander from EvoEndo, Inc.


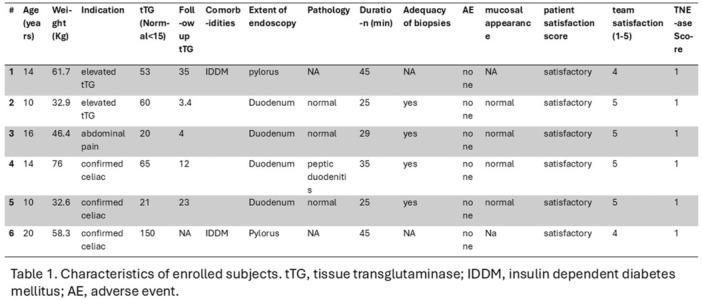


## 772 CHARACTERISTICS OF PEDIATRIC PATIENTS WITH INTESTINAL FAILURE ENROLLED IN A DIRECT‐TO‐PATIENT REGISTRY OF IMMOBILIZED LIPASE CARTRIDGE USERS

*Jason Soden* ^*1*^, *Justin Neal* ^*3*^, *Megan Aarnio‐Peterson* ^*2*^, *Ann Remmers* ^*2*^, *David Recker* ^*2*^

^*1*^ *Department of Pediatrics*, *University of Colorado Anschutz Medical Campus School of Medicine*, *Aurora*, *CO*; ^*2*^ *Alcresta Therapeutics Inc*, *Waltham*, *MA*; ^*3*^ *PRO‐spectus*, *Huntington Beach*, *CA*

RELiZORB (Alcresta Therapeutics, Inc.) immobilized lipase cartridge (ILC), a single‐use digestive enzyme cartridge that connects in‐line with enteral feeding circuits to hydrolyze triglycerides in enteral formulas, is cleared by the FDA for use in pediatric (including neonates and infants) and adult patients. Although ILC use has been associated with improved fat absorption and enteral feeding tolerance in patients with exocrine pancreatic insufficiency and cystic fibrosis, there are no published reports of ILC use in patients with intestinal failure (IF) or Short Bowel Syndrome (SBS).

A prospective direct‐to‐patient observational registry was initiated in July 2024 to collect real‐world data including medical history, anthropometric measurements, quality of life (caregiver‐as‐proxy), and progression towards enteral autonomy in pediatric patients with IF receiving enteral nutrition (EN) administered through an ILC. A total of 103 patients have been enrolled from 52 US clinics as of April 2025. Data provided by healthcare providers from a subset of patients diagnosed with SBS (n=92) and subpopulations who are parenteral nutrition (PN)‐dependent (n=55) or who have enteral autonomy (n=37) was analyzed to evaluate baseline patient characteristics prior to the development of a formal study protocol (Table 1). As expected, patients with Type III SBS were more likely to have achieved enteral autonomy compared to those who are PN‐dependent (p= 0.0252 two‐tailed Fisher's Exact test).

Caregiver‐reported PedsQL Generic Core and Gastrointestinal (GI) Symptoms Diarrhea Module Scores for the registry and healthy control populations are shown in Table 2. The PedsQL total score (73) in the registry population with SBS was comparable to caregiver reported scores (67 – 79) from an 11‐center study in the US and Canada of 336 pediatric patients with IF (Modi J Pediatrics (2025), doi: https://doi.org/10.1016/j.jpeds.2025.114566). Registry patients with enteral autonomy had psychosocial health scores 9 points higher (95% CI: 19.4, ‐1.3) than PN‐dependent patients (79.04 vs. 70.0). Registry patients with enteral autonomy had a psychosocial health mean score (79) was not statistically significantly different (p=0.3828 unpaired two‐tailed t‐test) from that of healthy controls (Varni 2007). Pediatric patients with SBS enrolled in the registry have a significantly worse mean diarrhea symptom score (50.8) than patients with other types of gastrointestinal diseases (77.4, Varni 2015).

It is anticipated that data collected through this registry will facilitate research on quality of life in patients with SBS, as well as the impact of ILC use on growth, progression towards enteral autonomy, and quality of life.


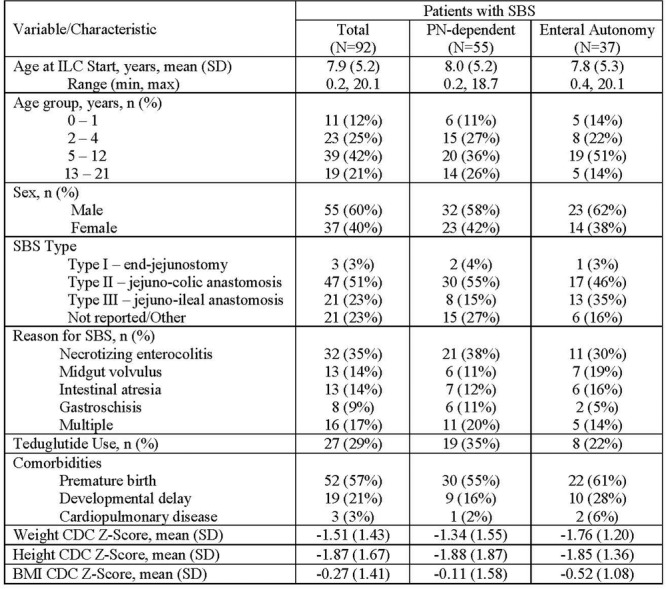


Table 1 Patient Demographics and Baseline Characteristics of Patients Diagnosed with Short Bowel Syndrome


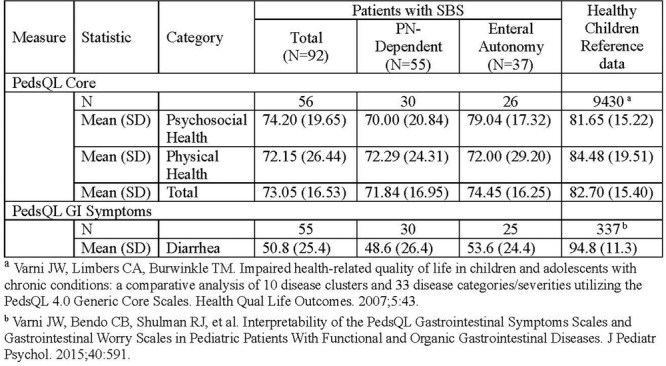


Table 2 Baseline Caregiver‐Reported Quality of Life Scores

## 773 NAVIGATING CELIAC DISEASE IN COLLEGE: RESULTS FROM A NATIONAL STUDENT SURVEY ON DINING, HOUSING, AND SUPPORT SERVICES

*Vanessa Weisbrod* ^*1*^, *Meghan Donnelly* ^*1*^, *Emma Kowzun* ^*1*^, *Marilyn Geller* ^*1*^, *Cynthia Alvarez* ^*2*^, *Ritu Verma* ^*2*^

^*1*^ *Education*, *Celiac Disease Foundation*, *Needham*, *MA*; ^*2*^ *The University of Chicago Medicine*, *Chicago*, *IL*

**Background:** College presents unique challenges for students with celiac disease, a chronic autoimmune disorder requiring strict lifelong adherence to a gluten‐free diet. Despite increased awareness of food allergies and dietary restrictions, limited data exist how students with celiac disease navigate campus life.

**Methods:** The Celiac Disease Foundation conducted a national survey between March 14 and April 2, 2025. The 35‐question survey explored student experiences with dining accommodations, housing, support services, social experiences, and mental health. Responses were solicited via the Foundation's email and social media platforms.

**Results:** A total of 324 students attending colleges or universities in the United States completed the survey. Survey respondents represented diverse academic settings, with 54% attending public universities, 41% private institutions, and 5% community colleges. A majority (58%) lived in on‐campus housing, and 55% participated in a college meal plan. However, campus dining posed significant challenges:

**‐ Dining Safety and Confidence:** 53% reported gluten exposure in campus dining halls. Confidence in dining safety was low with only 7% reported feeling “very confident” food was gluten‐free, while 60% were either “not very confident” or “not confident at all.”

**‐ Dining Hall Use and Satisfaction:** Nearly half of students rarely or never used the dining hall, citing lack of variety, poor quality, and inconvenience. Over 60% expressed dissatisfaction with the variety of gluten‐free options available.

**‐ Mental Health and Food Access:** 63% experienced frequent stress or anxiety related to managing celiac disease on campus. Nearly 90% of students endorsed skipping meals due to the absence of safe gluten‐free options, and 53% avoided social events out of concern for food safety.

**‐ Disability Accommodations and Support:** Only 32% of students had registered with their school's disability services for celiac‐related accommodations. Among them, just 20% found the office “very helpful” in securing safe housing or meal accommodations. Common accommodations included kitchen access, private housing, and extended time on assignments or exams.

**‐ Perceived Campus Support:** Only 4.4% of students felt their campus community clearly understood or were very supportive students with celiac disease.

**Conclusions:** College students with celiac disease face persistent barriers to safe, inclusive participation in campus life. These include inadequate gluten‐free dining options, lack of housing accommodations, and insufficient institutional support. The high rates of gluten exposure, meal‐skipping, and associated stress underscore the need for policy‐driven solutions to ensure equal access to food, health, and education for this vulnerable population. Institutions of higher education must improve gluten‐free safety protocols, housing flexibility, and clear disability accommodations to support the well‐being of students with celiac disease.

## 778 ANTIBIOTIC SENSITIVITY FOR CLABSI IN CHILDREN WITH PEDIATRIC INTESTINAL FAILURE RECEIVING HOME TPN

*Chenthan Krishnakumar*, *Aashka Patel*, *Gabriela Moraru*, *Saurabh Talathi*

*Pediatrics*, *The University of Oklahoma Health Sciences*, *Oklahoma City*, *OK*

**Background:** Patients with PIF receiving home TPN (total parenteral nutrition) via a central venous catheter are at risk for central line associated bloodstream infections (CLABSI). Institutional/regional analysis of the antibiotic sensitivity patterns can help choosing the appropriate empiric antibiotics coverage for these patients.

**Objective:** To identify antibiotic sensitivity patterns in CLABSI in pediatric patients followed in the intestinal rehabilitation program of tertiary facility at a single center.

**Methods:** Retrospective cohort study determining the type of microbes associated with CLABSI and their antibiotic sensitivity patterns.

**Results:** From February 1, 2018, to July 30, 2024, there were 167 CLABSI episodes among 37 TPN‐dependent children with median age 2.3 years old. 75.4% of patients carried the diagnosis of short bowel syndrome. Organisms isolated during these infections included 52.1% Gram‐positive bacteria, 38.3% Gram‐negative bacteria, and 9.6% fungi.

Among all staphylococcal isolates, 20.7% were methicillin resistant but 100% sensitive to vancomycin. Among enteric Gram‐negative organisms, 99.5% were sensitive to cefepime and only 82% were sensitive to Ceftriaxone. Current institutional empiric therapy (Cefepmie + Vancomycin) was appropriate for 98.8% of bacterial CLABSI infections.

Fungal infections were found in 9.6% of patients and all of them were due to candida sp and sensitive to micafungin.

**Conclusions:** Empiric antimicrobial therapy for suspected CLABSI in TPN‐dependent children should include therapy for methicillin‐resistant staphylococci as well as enteric Gram‐negative organisms – based on institutional antimicrobial sensitivity data. Fungal coverage needs to be added to the therapy for those not responding for initial empiric therapy or if clinically indicated.

## 779 DIFFERENCES IN GUT MICROBIOTA ASSSOCIATED WITH OBESITY IN LATINO CHILDREN BEFORE 3 YEARS OF AGE

*Sarah Maxwell* ^*1*^, *Lu Yang* ^*2*^, *Abdur Rahim Khan* ^*2*^, *James Bayrer* ^*1*^, *Philip Rosenthal* ^*1*^, *Emily Perito* ^*1*^, *Jennifer Price* ^*3*^, *Janet Wojcicki* ^*1*^

^*1*^ *Pediatrics*, *University of California San Francisco*, *San Francisco*, *CA*; ^*2*^ *The Benioff Center for Microbiome Medicine*, *University of California San Francisco*, *San Francisco*, *CA*; ^*3*^ *Medicine*, *University of California San Francisco*, *San Francisco*, *CA*

**Background:** Previous studies have found that the gut microbiota of children who later develop obesity may show important differences as early as infancy, including a greater abundance of *Bifidobacterium* in children who maintain a normal weight and a higher abundance of *Staphlycoccus aureus* in those who develop obesity. However, few studies have characterized microbial patterns across multiple time points during infancy and the preschool years in relation to early childhood obesity. We characterized the gut microbiome of Latino infants and preschool children with obesity and compared it to those without obesity.

**Methods:** In a longitudinal cohort of 87 Latino babies, stool samples were collected at 6 months, 1, 2, and 3 years of age, for a total of 215 observations. Obesity assessed at each time point was defined as a weight‐for‐length (WFL) or body mass index (BMI) ≥95% for age and sex. Stool microbial communities were profiled using 16S ribosomal RNA gene sequencing and analyzed for alpha diversity (using linear regression‐based t‐tests), beta diversity (using PERMANOVA), and differential taxonomic abundance (using ANCOM‐BC) to assess associations with obesity, adjusting for potential confounders.

**Results:** A high percentage of children in the cohort had obesity in early childhood (19.4% at 6 months, 2 years, and 3 years, and 41.9% at 1 year of age). Children with obesity had greater alpha diversity at 6 months of age (Shannon p=0.03 and Inverse Simpson p<0.01), but these differences were no longer significant at 1, 2 or 3 years of age. Differences in genera between children with and without obesity became apparent only at 2 years of age, with greater abundance of *Agathobacter* in children with obesity (FDR <0.01). At 3 years of age, children with obesity showed higher abundance of several genera belonging to the phyla *Actinobacteria and Firimcutes*, including *Collinsella, Dorea*, *Clostridium_sensu_stricto_1*, *and Ruminococcus* compared to children without obesity (FDR <0.1). At 3 years of age, children without obesity showed higher abundance of *Sellimonas* compared to children with obesity (FDR <0.1).

**Conclusions:** In our longitudinal study of Latino children with and without obesity, early differences in alpha diversity were no longer significant by 1 year of age, though changes in genera surfaced by 2 years of age. Some of our findings correspond with other obesity studies (e.g. *Sellimonas*, *Dorea, Ruminococcus*) and others are associated with low fiber intake (*Collinsella*). Early intervention for obesity may be required before 2 years of age when there are already significant differences in microbiota by obesity status.

## 780 TRENDS IN PROBIOTIC THERAPY IN HOSPITALIZED PEDIATRIC PATIENTS BEFORE AND AFTER THE 2023 UPDATED ESPGHAN GUIDELINES

*Pranjali Muppidi*, *Yasmika Rasakumar*, *Andrew Osten*

*Department of Pediatrics*, *SUNY Upstate Medical University Hospital*, *Syracuse*, *NY*

**Introduction:** The ESPGHAN updated their guidelines in 2023, stating that healthcare providers may recommend the use ofprobiotic therapy, particularly with acute gastroenteritis, hospital‐acquired diarrhea, Helicobacter pylori infection, necrotizing enterocolitis in premature infants, and antibiotic‐associated diarrhea. While prior studies suggest probiotics can reduce symptom duration and hospital stay, real‐world data on utilization trends and clinical impact remain limited. This study analyzes multicenter data to assess trends in probiotic use, aiming to inform quality improvement initiatives.

The primary objective of this study is to evaluate the patterns of temporal trends in probiotic use in hospitalized pediatric patients diagnosed with acute gastroenteritis, hospital‐acquired diarrhea, Helicobacter pylori infection, necrotizing enterocolitis, or those receiving antibiotics for ≥7 days. The study will also assess whether probiotic administration correlates with improved clinical outcomes by evaluating the length of hospital stay.

**Methods:** This retrospective study utilizes de‐identified data from the Pediatric Health Information System (PHIS) database. Patients under 20 years of age hospitalized between January 2020 and December 2024 with a diagnosis of acute gastroenteritis, hospital‐acquired diarrhea, Helicobacter pylori infection, premature infants at risk of necrotizing enterocolitis, or those receiving antibiotics for ≥7 days were included. Data collection included demographics, probiotic use, antibiotic exposure, length of hospital stay, and ICU admissions. Interrupted time series analysis, regression modeling, and t‐tests were used to assess temporal trends and associations.

**Results:** Data analyses revealed no significant difference in probiotic use before vs. after the 2023 guidelines for patients admitted with acute gastroenteritis (9.46% vs. 8.65%, p = 0.072), or preterm infants at risk for NEC (3.88% vs. 2.83%, p = 0.077). However, a significant decrease was observed in patients receiving antibiotics for ≥7 days (10.7% vs. 9.0%, p <0.001), with hospital‐acquired diarrhea (11.2% vs. 9.39%, p = 0.0076) and with H. pylori (3.88% vs. 2.83%, p = 0.045). Linear regression analyses showed overall stable or decreased use of probiotics after the guidelines, with a notable decline in use in preterm infants at risk for NEC.

Length of stay (LOS) was significantly longer (p < 0.001) in pediatric patients receiving probiotics compared to those who did not, across all indications. However, the group sizes between probiotic recipients and non‐recipients differed substantially, which may contribute to the observed statistical significance. It is also important to note, no adjustments were made for baseline differences between groups.

**Discussion/Conclusion:** Despite updated ESPGHAN guidelines, probiotic use in hospitalized pediatric patients has remained stable or declined over the past two years. While NASPGHAN did not update recommendations for each condition examined in this study, the 2023 joint ESPGHAN‐NASPGHAN H. pylori guideline similarly had little impact on probiotic prescribing patterns. In the NICU, although no statistically significant change was observed overall, time series analysis revealed a sharp decline in probiotic use beginning in late 2023, coinciding with the FDA warning regarding the risk of sepsis and potentially fatal disease in preterm infants. These findings raise important questions about provider hesitancy in probiotic use and whether alternative recommendations are being followed.

Regarding the LOS data, substantial differences in group sizes and the absence of adjustments for baseline characteristics suggest that residual confounding may be influencing the results. However, the findings of extended LOS in probiotic recipients likely reflects their use in more complex cases rather than a direct effect of the treatment. This highlights the challenge of indication bias in observational data. Future research using more rigorous methods, such as matched cohorts or instrumental‐variable analyses, is necessary to clarify probiotic benefits. Our results also point to a gap between guideline recommendations and clinical practice, underscoring the need for targeted strategies and stronger evidence to define the optimal use of probiotics in hospitalized children.

## 783 MUC2 TRANSPORT ACROSS PEYER'S PATCHES IN THE CFTR KO INTESTINE

*Lillian Caldwell* ^*1*^, *Ashley Stocksick* ^*1*^, *Rowena Woode* ^*1,2*^, *Lane Clarke* ^*1,2*^

^*1*^ *Dalton Cardiovascular Research Center*, *University of Missouri*, *Columbia*, *MO*; ^*2*^ *Department of Biomedical Sciences*, *University of Missouri*, *Columbia*, *MO*

**Background:** Cystic fibrosis (CF) is a genetic disorder caused by mutations in the cystic fibrosis transmembrane conductance regulator (CFTR) gene. In the intestine, loss of CFTR function leads to the accumulation of thick, sticky mucus resulting in dysbiosis and intestinal obstruction. This abnormally viscous mucus, referred to as "mucoviscidosis," may also contribute to the immune abnormalities seen in CF including chronic inflammation and an increased prevalence of food allergies. Previous studies have shown that Muc2, the primary mucin in the gastrointestinal tract, is sampled by dendritic cells (DCs) in the intestinal Peyer's patches (PP) to promote oral tolerance to food antigens. Mucoviscidosis in the CF intestine may alter Muc2 uptake across PP, thereby affecting the tolerogenic properties of the underlying DCs.

**Methods:** Cftr KO mice and their sex‐matched wild‐type (WT) littermates were maintained on a nutritionally complete liquid diet (Peptamen) and tap water to prevent intestinal obstruction. PP from pairs of Cftr KO and WT mice between 2 and 3 months of age were collected, fixed with paraformaldehyde, and embedded in OCT compound. Jejunal PP were sectioned at 11 microns and Muc2 was detected via indirect immunofluorescence staining. Images were captured using confocal microscopy and analyzed using Fiji image processing software to quantify Muc2 within each PP. Muc2 fluorescence was measured in mean gray value, which is the sum of the intensity of pixels within the grayscale image divided by the number of pixels within the region of interest. Statistical analysis was performed using an unpaired t‐test in SigmaPlot® 14.0 to compare PP Muc2 content between Cftr KO and WT mice. The threshold for statistical significance was set at p < 0.05 and results are reported as mean ± standard error.

**Results:** 5 WT and 6 CF mice were included in analysis. Muc2 in the PP was assessed in the sub‐dome region (0–50 µm from the epithelium) and in the interior (50–150 µm from the epithelium). Muc2 quantity was significantly greater in the sub‐dome region in the Cftr KO PP (7.829 ± 1.859) than in the WT PP (1.485 ± 0.702; p=0.0213). There was not a significant difference in the amount of Muc2 within the interior 50‐150 µm region between Cftr KO (5.018 ± 1.734) and WT (2.184 ± 0.817) PP (p=0.170).

**Discussion:** A greater amount of Muc2 was observed in the subepithelial region of Cftr KO PP compared to the WT controls. Given that Muc2 has been demonstrated to confer tolerogenic properties to DCs during antigen presentation, altered dynamics in the Cftr KO intestine may contribute to the immune dysregulation present in CF. Further research is needed to explore the potential relationship between increased Muc2 and antigen sampling and studies with larger sample sizes are warranted to validate these findings. One limitation of our study was the strong autofluorescence that persisted in samples despite quenching efforts. Employing an alternative method to quantify Muc2, such as western blotting, would be beneficial to support these results.

## 791 RISK FACTORS FOR GASTROINTESTINAL BLEEDING IN PEDIATRIC PATIENTS ON VENTRICULAR ASSIST DEVICE THERAPY: A SINGLE‐CENTER RETROSPECTIVE ANALYSIS

*Timothy Marshall* ^*1*^, *Carmine Suppa* ^*2*^, *Nagraj Kasi* ^*2*^, *Heather Henderson* ^*3*^, *Jordan Whatley* ^*2*^

^*1*^ *Pediatric Gastroenterology Fellowship Program*, *Medical University of South Carolina*, *Charleston*, *SC*; ^*2*^ *Division of Pediatric Gastroenterology, Hepatology, and Nutrition*, *Medical University of South Carolina*, *Charleston*, *SC*; ^*3*^ *Division of Pediatric Cardiology*, *Medical University of South Carolina*, *Charleston*, *SC*

**Background:** Mechanisms of gastrointestinal bleeding (GIB) in ventricular assist device (VAD) therapy include changes in blood properties, altered shear forces, platelet effects, angiodysplasia, mucosal injury, and depletion of essential clotting proteins. Adult studies have found several significant risk factors for GIB in patients on VAD therapy. However, studies identifying risk factors for pediatric patients remain limited. This single center retrospective analysis seeks to identify and elucidate preimplantation risk factors for GIB after VAD implantation in pediatric patients.

**Methods:** This is an ongoing retrospective 8‐year study from 2017 to 2024 at a single center with a primary endpoint of gastrointestinal bleeding within 12 months of ventricular assist device implantation in pediatric patients (n=40 for preliminary data included from 2017 to 2021). Multivariable logistic regression was used to determine independent predictors of GI bleeding in a variety of continuous and categorical variables.

**Results:** In the preliminary analysis, GIB occurred following 12 of the 40 VADs placed. Baseline continuous variables prior to VAD placement including BUN, creatinine, platelet count, INR, ALT, BMI, and hemodynamic data were not significantly different between the GIB group and non‐GIB group (Table 1). Pre‐VAD categorical variables were also assessed (Table 2). Patients with history of single ventricle pathology, prior Berlin with Pedimag VAD implant, prior GI consult, acute kidney injury, enoxaparin use at implantation, or proton pump inhibitor use at implantation had significantly higher rates of GIB when compared to the GIB rate of the entire cohort (P<0.05). Patients on histamine receptor‐2‐blocker therapy or with history of dilated cardiomyopathy had significantly lower rates of GIB (P<0.05). Patients with history of other cardiomyopathies, prior cardiac surgeries, prior GIB, other prior VADs, or aspirin use at time of VAD placement did not have a significantly different rate of bleeding when compared to the entire cohort.

**Conclusion:** Patients with history of dilated cardiomyopathy had a lower number of expected GIB events, whereas patients with history of single ventricle pathology had an increase number of GIB events. Unlike patients with dilated cardiomyopathy, patients with single ventricle congenital heart disease who have underwent Fontan procedure have circulation which predisposes them to venous congestion and formation of vascular anomalies in GI tract that can rupture and bleed, especially in setting of anticoagulation. History of prior GI consultation was associated with an increase in GIB events. Of the 7 bleeding events with prior GI consultation, 4 events occurred in patients who had prior GI consultation for monitoring for Fontan‐associated liver disease.

Patients with history of acute kidney injury had a higher incidence of bleeding than expected as did patients with history of enoxaparin use. Enoxaparin is known to be renally cleared and can accumulate in patients with renal dysfunction. Thus, based on this preliminary data, anticoagulation with enoxaparin use in patients with VADs and concomitant renal dysfunction may confer an increased risk of GIB.

The differences in bleeding rates between proton pump inhibitor (PPI) use and histamine‐2 (H2) blocker were not an expected finding as PPIs are more effective in management of upper GIB. However, a possible explanation may be that H2 blockers were used more in patients felt to have a lower risk of GIB.


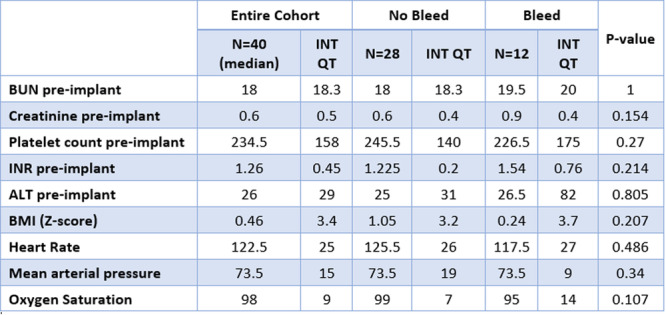



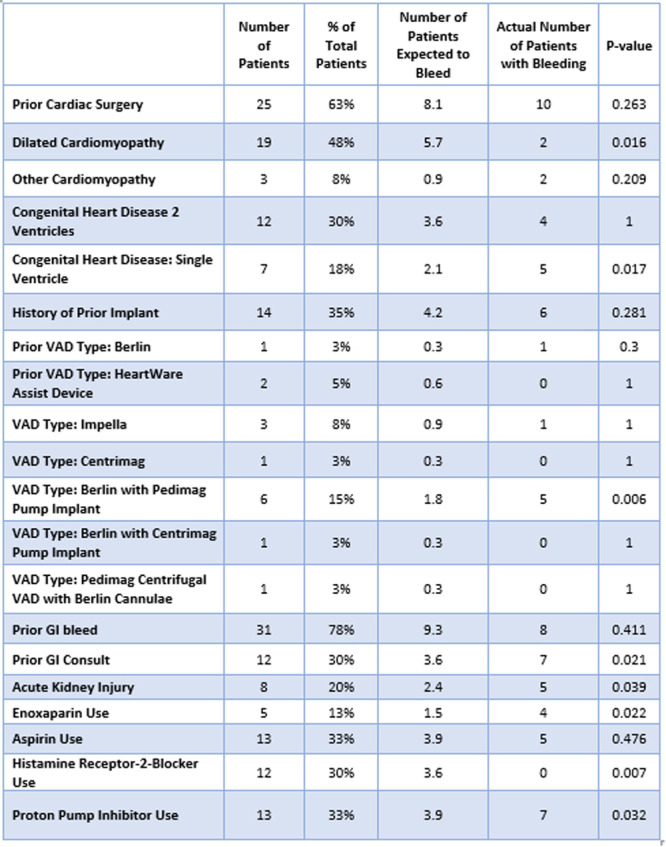


## 798  A PILOT STUDY INVESTIGATING IMPROVEMENT IN FUNCTIONAL NAUSEA AFTER PERCUTANEOUS ELECTRICAL NERVE FIELD STIMULATION (PENFS)

*Mohamad Abi Nassif*, *Khalil El‐Chammas*, *Kahleb graham*, *Rashmi Sahay*, *Neha Santucci*

*GASTROENTEROLOGY*, *Cincinnati Children's Hospital Medical Center*, *Cincinnati*, *OH*

**Background:** Chronic nausea is a distressing symptom in children, affecting between 15% and 23% of school‐aged children in the United States. The Rome IV criteria define functional nausea as 1) frequent and bothersome nausea at least twice weekly, usually unrelated to meals, 2) not consistently accompanied by vomiting, and 3) unexplained by other medical conditions after appropriate evaluation. Auricular percutaneous electrical nerve field stimulation (PENFS) is FDA cleared for treating pediatric Disorders of the Gut‐Brain Interaction (DGBI). Yet, evidence regarding its effectiveness in managing nausea is still scarce. We aimed to evaluate the changes in nausea over the course of PENFS visits.

**Methods:** We reviewed the medical records of patients aged 7‐21 y who met criteria for functional nausea and underwent PENFS at Cincinnati Children's Hospital between 2017 and 2024. We gathered information on baseline demographic and anthropometric data, diagnostic interventions, and associated comorbidities as well as outcomes. Outcomes were classified as resolution, improvement, worsening, or no change based on nausea intensity and frequency, assessed after each PENFS visit through subjective patients’ responses documented in their charts.

**Results:** Of the 16 patients with functional nausea, median age was 17 y (15.5,18.5), 75% females and 100% Caucasian. Of these, 9 (56.25%) patients had a comorbid psychiatric disorder, of which 4 (44.4%) were on psychotropic medications and 2 (12.5%) were receiving cognitive behavioral therapy (CBT).

During the second, third and fourth PENFS visit, 56.25%, 68% and 75% reported improvement or resolution of nausea respectively (p‐value=0.5811). A trend for higher odds of nausea getting resolved was noticed with every visit (p‐value= 0.0618).

**Conclusion:** In our small cohort, 75% of children with functional nausea report improvement or resolution of symptoms after PENFS placement. Future prospective studies are required to assess the durability of response.

## 799 OUTCOMES OF SURGICAL VERSUS CONSERVATIVE TREATMENT IN PEDIATRIC MEDIAN ARCUATE LIGAMENT SYNDROME (MALS)

*Mohamad Abi Nassif* ^*1*^, *Juan Gurria* ^*2*^, *Daniel Mallon* ^*1*^, *Rashmi Sahay* ^*1*^, *Lev Dorfman* ^*1*^, *Emily Vore* ^*2*^, *Alexander Nasr* ^*1*^, *Kaitlin Whaley* ^*1*^, *Neha Santucci* ^*1*^

^*1*^ *GASTROENTEROLOGY*, *Cincinnati Children's Hospital Medical Center*, *Cincinnati*, *OH*; ^*2*^ *Division of Pediatric General and Thoracic Surgery*, *Cincinnati Children's Hospital Medical Center*, *Cincinnati*, *OH*

**Background:** Median arcuate ligament syndrome (MALS) includes clinical signs and symptoms from compression of celiac artery by the median arcuate ligament. We assessed outcomes after surgical and conservative treatment in children with MALS.

**Methods:** We reviewed charts (demographics, surgical and medical history) of MALS patients aged 5‐18 y diagnosed by ultrasonography and/or CT angiogram. Patient outcomes were categorized as resolved, improved, unchanged, or worsened at the most recent follow‐up (FU) and compared between those who underwent surgical treatment and those managed conservatively.

**Results:** Of 34 patients [median age: 15 y (13,17), 79.4% females and 94% Caucasian], abdominal pain (97%) and nausea (70.5%) were the most common symptoms, 45% of patients had a psychiatric disorder (89% on psychotropic medications), 100% met criteria for a DGBI.

Ten patients underwent conservative management [pharmacotherapy: amitriptyline, cyproheptadine or others (100%), behavioral therapies (80%), endoscopic pyloric botulinum toxin injection (30%), auricular neurostimulation (20%)]. Of these, 27% reported resolution, 26% improved, and 36% remained unchanged (median FU:5 months, 1‐22 months, Fig 1).

After surgery [n=24, length of stay: 4 days (1‐15 days)], complications (29%) included rash, splenic artery rupture, decreased oral intake and hematochezia. At the post‐operative FU (median 20 days, range:7‐39 days), 50% reported resolution and 33% improved (p‐value=0.009). At a median FU of 9.8 months (range: 7 days ‐ 4.2 years), 21% had symptom resolution, 33% improved, 17% were unchanged and 29% worsened (p‐value =0.199).

More patients in the conservative group had generalized or periumbilical pain while surgical patients had more lower or upper abdominal pain (p=0.005). The groups did not differ based on demographics or long‐term response (p>0.05). Refer to table 2. Baseline comorbidities and interventions did not predict treatment response in medical or surgical groups (p > 0.05).

**Conclusion:** In our small cohort, 64% of children with MALS improved with conservative treatment for DGBI while 54% improved with decompression surgery at long‐term FU despite a robust post‐operative response. Future prospective studies are required for selection of patients and assess the durability of response.


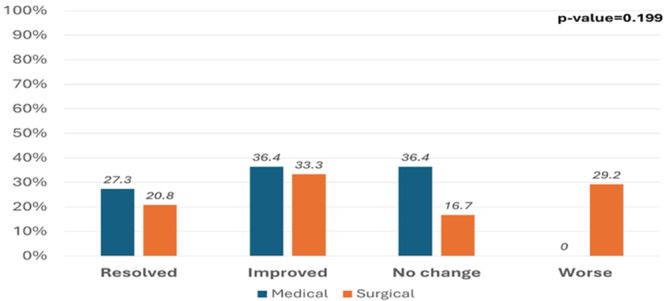


## 802 ASSOCIATION OF PREMATURITY AND DELIVERY MODE WITH PEDIATRIC DISORDERS OF GUT‐BRAIN INTERACTION

*Samantha Arrizabalo* ^*1*^, *Natalia Palma* ^*2*^, *Carlos Velasco‐Benitez* ^*3*^, *Daniela Velasco‐Suarez* ^*3*^, *Miguel Saps* ^*1*^

^*1*^ *Pediatrics Gastroenterology, Hepatology and Nutrition*, *University of Miami Miller School of Medicine*, *Miami*, *FL*; ^*2*^ *Universidad Peruana de Ciencias Aplicadas*, *Lima District*, *Lima Region*, *Peru*; ^*3*^ *Universidad del Valle*, *Cali*, *Colombia*

**Background/Objectives:** Disorders of gut‐brain interaction (DGBI) are highly prevalent worldwide and are considered to be multifactorial in origin. Early life events have been proposed as a possible factor in their etiopathogenesis. Current data states that early life events, including method of delivery and gestational age of birth, significantly influence early gut colonization and the development of long‐term diseases. Despite this clear association, data on the relationship between mode of delivery, prematurity, and the development of DGBI in children remains limited. This study aims to determine the possible relationship between mode of delivery, prematurity, and DGBI development remains unclear. This study aims to assess whether cesarean delivery and prematurity contribute to the development of DGBI in early childhood.

**Methods:** Caregivers of children aged 1 month to 4 years —categorized into two groups (1–12 months and 1–4 years) — who were attending pediatric outpatient clinics at public and private hospitals from four geographically diverse Colombian cities were invited to participate in a cross‐sectional study. Children with a history of organic diseases were excluded. Data was obtained by interviewing the parents and the pediatrician completed the Spanish‐validated Questionnaire of Pediatric Gastrointestinal Symptoms Rome IV (QPGS‐IV) during the child's appointment. We assessed the relationship between prematurity, cesarean delivery, and the development of DGBI by comparing the prevalence of DGBI in children with and without these factors. Demographic data was analyzed using measures of central tendency, including mean ± standard deviation (SD). Univariate analysis was conducted to calculate odds ratios (OR) with 95% confidence intervals (95% CI). The significance level was set at P < 0.05.

**Results:** A total of 1468 caregivers participated in the study, with a median age of 24.2 ± 15 months and 50.7% were male. In total, 390 (26.6%) participants were diagnosed with at least one DGBI, with functional constipation (FC) being the most prevalent (22.3%). Among children born via cesarean section (54.3%), 30.4% of them were diagnosed with DGBI (OR=1.54, 95% CI=1.20–1.96, p=0.00), and 26.3% with FC (OR=1.29, 95% CI=1.29–2.18, p=0.00). Prematurity was observed in 12.6% of participants and was associated with a higher prevalence of DGBI (35.7%, (OR=1.64, 95% CI=1.16–2.29, p=0.00), with FC affecting 30.8% (OR=1.66, 95% CI=1.16–2.35, p=0.00). Both prematurely and cesarean section was identified in 138 (9.4%) children. Among them, 55 (40%) were diagnosed with DGBI (OR=1.67, 95% CI=1.11–2.48, p=0.00), and 47 (34.1%) were diagnosed with FC (OR=1.57, 95% CI=1.03‐2.36, p=0.02).

**Conclusion:** This is the first epidemiological study in infants and preschool‐aged children focused on whether cesarean delivery and prematurity contribute to early childhood DGBI. Our results suggest that Cesarean delivery and prematurity may increase the risk of developing DGBI in early childhood, particularly FC. These findings highlight the need for further research to explore potential mechanisms and confirm these associations.


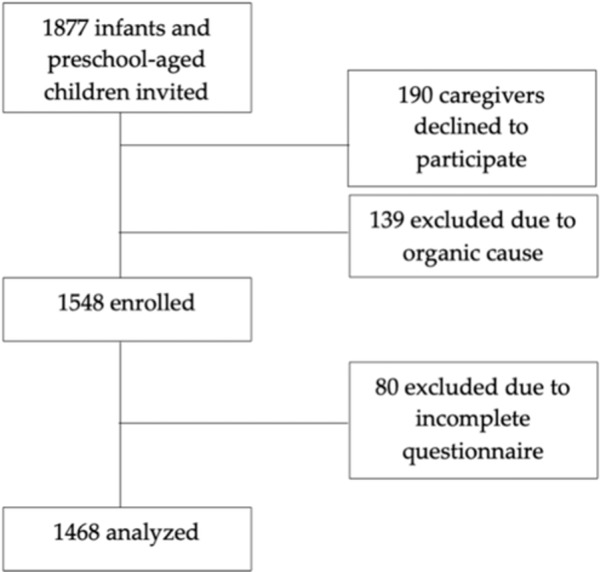



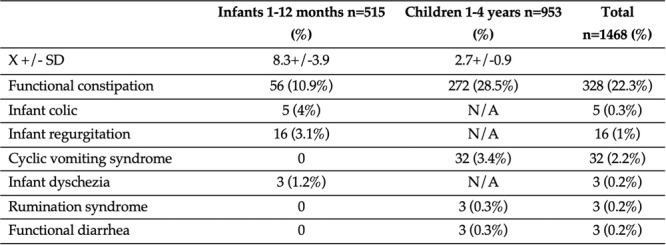


## 803 MENSTRUAL INFLUENCE ON IBS DIAGNOSIS IN ADOLESCENTS

*Samantha Arrizabalo* ^*1*^, *Krisia Banegas* ^*1*^, *Carlos Velasco‐Benitez* ^*2*^, *Daniela Alejandra Velasco‐Suarez* ^*2*^, *Miguel Saps* ^*3*^

^*1*^ *Pediatrics*, *Holtz Children's Hospital*, *Miami*, *FL*; ^*2*^ *Gastroenterology*, *Universidad del Valle Cali*, *Cali*, *Colombia*; ^*3*^ *Gastroenterology, Hepatology and Nutrition*, *University of Miami Miller School of Medicine*, *Miami*, *FL*

**Background:** Disorders of gut–brain interaction (DGBI) affects 25%–30% of children and adolescents worldwide. Among these, irritable bowel syndrome (IBS) is one of the most reported disorders especially in girls. IBS is diagnosed clinically per the Rome IV criteria. These criteria do not consider abdominal pain or stool changes associated with menstruation, which are commonly reported by adolescent girls. Understanding the association of the menstrual period with IBS symptoms can potentially define subgroups of IBS in females and guide personalized treatments.

**Objectives:** This study aimed:

Assess the influence of menstrual‐related symptoms on the diagnosis of IBS

**Hypothesis:** A subset of patients who meet the criteria for IBS experience symptoms exclusively during the menstrual period.

Compare the prevalence of IBS between postmenarchal and premenarchal females.

**Hypothesis:** Postmenarchal adolescents will exhibit a higher prevalence of IBS compared to their premenarchal counterparts.

**Methods:** A cross‐sectional study was conducted among female students aged 10–16 years from public and private schools across seven Colombian cities. Participants completed the QPGS‐Rome IV questionnaire. Females with a history of organic disease were excluded. Analyses were performed using descriptive statistics and Fisher's exact test, with significance set at p < 0.05.

**Results:** A total of 3521 female school‐aged children and adolescents were invited to participate Data from 2140 participants who reported experiencing abdominal pain on at least one day were analyzed (Figure 1), the mean age was 13.7 ± 1.7 years; 1877 (87.7%) had reached menarche and 263 (12.3%) had not. Among postmenarchal females, 609 (32.5%) met criteria for a DGBI, 212 (11.3%) had an abdominal pain–related disorder, and 45 (2.4%) were diagnosed with IBS. Of those with IBS, 62.2% reported experiencing abdominal pain exclusively during menstruation (p = 0.13). 80 (30.4%) of premenarchal females had a DGBI, 27 (10.3%) had an abdominal pain–related disorder, and 12 (4.5%) met criteria for IBS. IBS prevalence was significantly higher in premenarchal than in postmenarchal participants (4.5% vs. 2.4%, p = 0.04).

**Conclusion:** The majority of postmenarchal females have IBS symptoms exclusively during the menstrual period. IBS is more common in premenarchal females compared with postmenarchal females. If the results of our study are confirmed in future larger investigations, it is possible that hormonal changes may influence the diagnosis of IBS. Menstrual‐associated IBS should be possible considered as a form of IBS with a specific diagnosis and treatment.


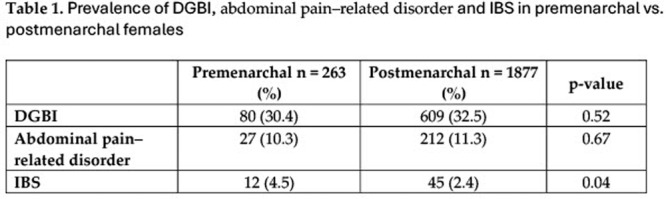



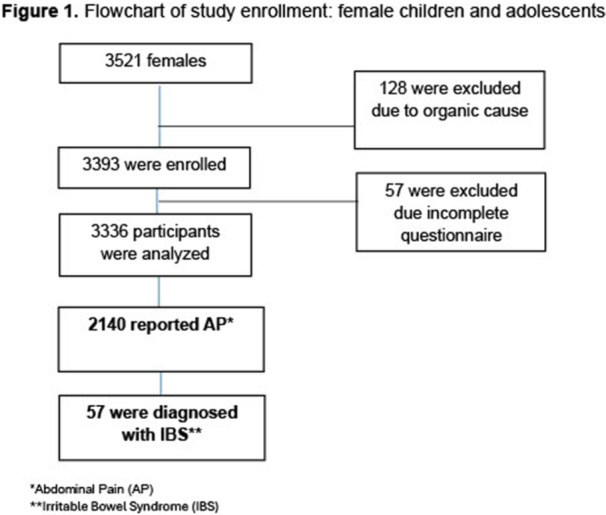


## 804 DO CHILDREN AND ADOLESCENTS MEET THE SAME ROME IV CRITERIA FOR FUNCTIONAL CONSTIPATION?

*Adrian Baca‐Arzaga* ^*1*^, *Samantha Arrizabalo* ^*2*^, *Carlos Velasco‐Benitez* ^*3*^, *Daniela Velasco‐Suarez* ^*3*^, *Miguel Saps* ^*2*^

^*1*^ *Pediatrics*, *University of Miami Department of Pediatrics*, *Miami*, *FL*; ^*2*^ *Division of Pediatric Gastroenterology, Hepatology and Nutrition*, *University of Miami Department of Pediatrics*, *Miami*, *FL*; ^*3*^ *Universidad del Valle Facultad de Salud*, *Cali*, *Valle del Cauca*, *Colombia*

**Background:** Functional constipation (FC) is among the most prevalent disorder of gut‐brain interaction (DGBI) in children and adolescents, affecting approximately 9.5% of the pediatric population. Additionally, follow‐up studies reveal that up to 25% of children treated for FC continue to experience symptoms into adolescence and adulthood. FC is diagnosed based on at least two of the seven (one of them being a composed criterion: painful or hard stool) Rome IV criteria. Understanding the prevalence of each criterion is important for optimizing treatment strategies and guiding clinical research, including participant recruitment and outcome selection. However, data on the prevalence of individual items on the Rome criteria across different age groups remain limited.

**Aim:** 1‐ To assess the prevalence of each criterion of the Rome IV criteria for FC in school‐aged children (8 to 12 years old) and adolescents (13 to 18 years old). 2‐To determine whether the prevalence of each criterion differ by age group.

**Hypothesis:** The frequency of each individual criterion vary with age.

**Methods:** Children aged 8–18 years were recruited from 10 schools (eight public, two private) across seven Colombian cities. Exclusion criteria included a history of organic disease. Informed consent was obtained from caregivers and assent from children prior to participation. Students completed the self‐report validated QPGS‐Rome IV questionnaire (QPGS‐IV). Instructions were provided by trained staff, who remained available to answer questions. The study was approved by the Ethics Committee of Hospital Universitario del Valle (024‐2019, 02/27/2020). Demographic data were analyzed using means and standard deviations for continuous variables, and frequencies and percentages for categorical variables. Group comparisons were performed using 2×2 contingency tables and Fisher's exact test, with significance set at p < 0.05.

**Results:** A total of 6797 school‐aged children and adolescents participated in the study from which 849 met criteria for FC and were included in this analysis. Most participants were adolescents (70.1%), 66.1% were female, 51.4% identified as mixed race and the mean age was 13.7 ± 2.2 years. Adolescents more frequently met FC criteria based on a combination of only two diagnostic criteria compared to school‐aged children (63.5% vs. 53.5%, p < 0.00) (Table 1). The two most frequently reported individual criteria across both groups were two or fewer stools per week and history of painful bowel movement (BM). However, the prevalence of each criterion varied by age group. Among adolescents, the most common criterion was frequency: having two or fewer BM per week (64.5%), while school‐aged children most frequently reported painful BM (68.1%) (Table 2). Fecal incontinence was more common in school‐aged children than in adolescents (11% vs. 5.2%, p < 0.00).

**Conclusion:** As hypothesized, most children and adolescents with FC fulfilled the same two Rome IV criteria: two or fewer stools per week and history of painful BM. However, their relative prevalence varied by age group.


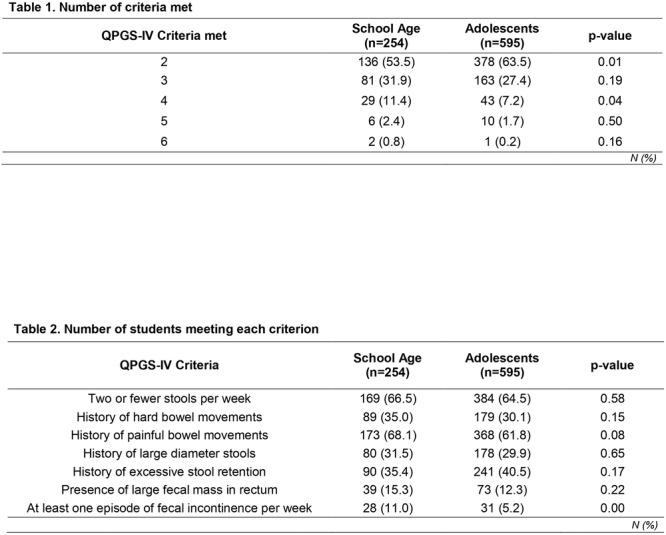


Tables 1 and 2

## 805 TOWARD TARGETED TREATMENT IN PEDIATRIC CVS: CHARACTERIZING PHYSICAL AND PSYCHOSOCIAL FACTORS TO INFORM MULTIDISCIPLINARY CARE

*Kimberly Brown*, *Julie Banda*, *Brittany Gresl*, *B Li*, *Katja Karrento*

*Pediatrics*, *Medical College of Wisconsin*, *Milwaukee*, *WI*

**Background:** Cyclic vomiting syndrome (CVS) is a disorder of gut‐brain interaction (DGBI) manifested by recurrent episodes of vomiting lasting hours to days between periods of baseline health. The multidisciplinary pediatric CVS Program at Children's Wisconsin uses the PROMIS Pediatric Global Health Scale (PGH‐7) child‐ and parent proxy‐reports to assess overall physical and mental health of patients and guide clinical care. In this quality improvement project, we characterized demographics, clinical features, and global health using the PROMIS PGH‐7 to identify factors that may improve multidisciplinary services.

**Methods:** We conducted a retrospective chart review to collect demographics and baseline self‐ and caregiver‐reported PROMIS PGH‐7 scores from patients evaluated in the CVS Program between April 2022 and March 2025.

**Results:** Data from 182 children of mean age 11.29 (*SD* = 3.55) years (57.1% female, 85.7% White) were analyzed. Families were from 19 US states (73.6% Wisconsin) and one Canadian territory. The most common medical diagnoses were CVS (91.2%), other or co‐occurring DGBI (47.3%), and dysautonomia (32.4%). Over half (56%) had a documented anxiety diagnosis. PGH‐7 T‐scores were below standardized average (self‐report *M* = 44.09, caregiver report *M* = 41.71). Patient and caregiver reports were strongly correlated (*r* = 0.91, *p*<0.001). Older age was associated with lower PGH‐7 scores for self‐ (*r* = ‐0.23, *p*=0.005) and caregiver report (*r* = ‐0.3, *p*<0.001). There were no significant differences in PGH‐7 scores by sex, ethnicity, home state, or visit type (new versus follow‐up). Patient and caregiver‐reported PGH‐7 scores were higher in those with a diagnosis of CVS than those without (*p*=0.006, *p*<0.001, respectively), and lower in those with a co‐occurring DGBI (*p*=0.01, *p*=0.001, respectively) and/or dysautonomia (*p'*s<0.001). Patients and caregivers indicated lower global health in those with concurrent diagnoses of anxiety (*p'*s<0.001), depression (*p*=0.02, *p*=0.002, respectively), and ADHD (*p*=0.02, *p*=0.01, respectively) compared to those without.

**Conclusions:** Older age, presence of comorbid DGBI and dysautonomia, and anxiety, depression, and ADHD are related to worse global health as measured by PROMIS PGH‐7 in children with CVS. These data help guide clinical decision making by identifying patients who may benefit from additional supports and reinforce the value of a multitargeted, multidisciplinary approach to improve quality of life in children with CVS.

## 806 INTRAOPERATIVE HIGH‐RESOLUTION ESOPHAGEAL MANOMETRY AND ENDOLUMINAL FUNCTIONAL IMAGING PROBE EFFECTIVELY AND SAFELY GUIDE HELLER MYOTOMY AND DOR FUNDOPLICATION IN PEDIATRIC ACHALASIA

*Gabriela Báez‐Bravo* ^*1,2*^, *Price Edwards* ^*3*^, *Duc Nguyen* ^*2*^, *Kexin Guo* ^*2*^, *Kristy Rialon* ^*4,2*^, *Bruno Chumpitazi* ^*5*^, *Eric Chiou* ^*1,2*^

^*1*^ *Pediatric Gastroenterology, Hepatology and Nutrition*, *Texas Children's Hospital*, *Houston*, *TX*; ^*2*^ *Pediatrics*, *Baylor College of Medicine*, *Houston*, *TX*; ^*3*^ *Pediatric Gastroenterology*, *The University of Tennessee Health Science Center*, *Memphis*, *TN*; ^*4*^ *Pediatric Surgery*, *Texas Children's Hospital*, *Houston*, *TX*; ^*5*^ *Pediatric Gastroenterology, Hepatology and Nutrition*, *Duke University School of Medicine*, *Durham*, *NC*

**Background:** Achalasia is a rare esophageal motility disorder characterized by impaired relaxation of the esophagogastric junction (EGJ) and lack of peristalsis through the esophageal body. The use of high‐resolution esophageal manometry (HREM) and endoluminal functional imaging probe (EndoFLIP) has been described in adult populations to guide interventions for achalasia. However, the feasibility, impact, and safety of both HREM and EndoFLIP in guiding therapy in pediatric patients with achalasia undergoing laparoscopic Heller myotomy (LHM) and Dor fundoplication is not well established.

**Methods:** A retrospective review was conducted for children with achalasia who underwent LMH and Dor fundoplication with intraoperative HREM and EndoFLIP guidance between January 2020 and December 2024. Esophagogastric junction (EGJ) parameters were assessed at three surgical time points: pre‐myotomy, post‐myotomy (before fundoplication), and post‐fundoplication. HREM was used to measure mean baseline pressure at all time points, whereas EndoFLIP was used to measure the minimum cross‐sectional area (CSA) and distensibility index (DI) at 40 and 50 mL balloon volumes pre‐myotomy and post‐fundoplication. Measurements were obtained throughout the procedure to guide intraoperative decision‐making, allowing the surgical team to make real‐time adjustments.

**Results:** Nineteen patients were included, with a median age of 14 years (interquartile range: 11.00–16.00). Intraoperative HREM measurements were obtained in all nineteen patients; of these, seventeen had complete data across all three time points. Intraoperative EndoFLIP measurements were available for seventeen patients, with fourteen having both pre‐myotomy and post‐fundoplication measurements. Median HREM EGJ pressure significantly decreased pre‐myotomy vs post‐myotomy (36 mmHg vs 13 mmHg, p<0.001), with no significant difference observed between post‐myotomy and post‐fundoplication values (Figure 1). Intraoperative EndoFLIP demonstrated significant increases in EGJ distensibility pre‐myotomy vs post‐fundoplication, with the median DI rising from 1.6 to 3.9 mm^2/mmHg at 40 mL (p=0.002) and from 1.8 to 4.8 mm^2/mmHg at 50 mL balloon distension (p=0.003) (Table 1). Median CSA also increased significantly pre‐myotomy vs post‐fundoplication at both 40 and 50 mL balloon inflations (Table 1). There were no complications associated with the use of intraoperative HREM or EndoFLIP.

**Conclusion:** Our findings demonstrate that intraoperative HREM and EndoFLIP can successfully and safely guide LHM and Dor fundoplication in pediatric patients. These tools provide valuable real‐time physiologic assessment of EGJ dynamics that may guide intraoperative decision‐making. However, further studies are needed to evaluate the correlation between intraoperative measurements and clinical outcomes.


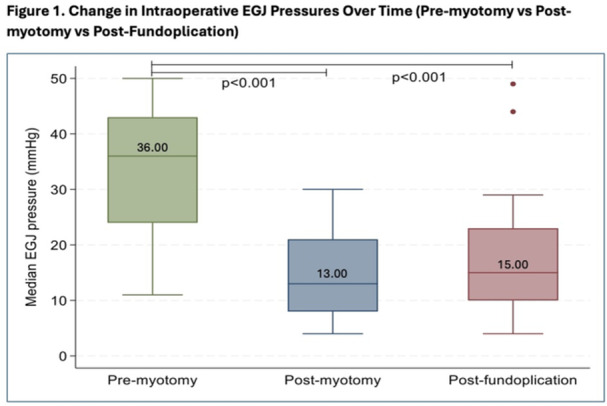



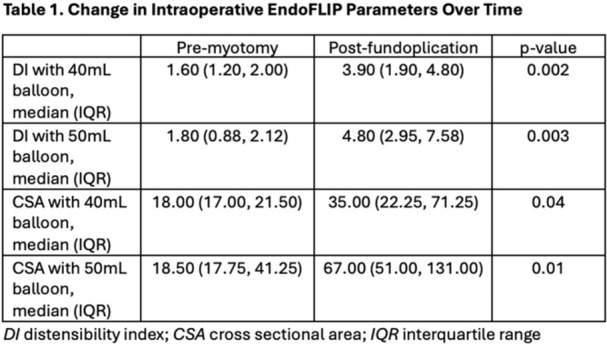


## 807 KNOWLEDGE, ATTITUDES, AND PRACTICES OF PEDIATRICIANS AND PEDIATRIC GASTROENTEROLOGISTS REGARDING FUNCTIONAL CONSTIPATION IN CHILDREN

*Ricardo Chanis Aguila* ^*1*^, *Carlos Velasco‐Benitez* ^*2*^, *FINDERS group* ^*2*^

^*1*^ *Pediatrics*, *Hospital del Niño Dr. José Renán Esquivel*, *Panama city*, *Panama*; ^*2*^ *Pediatrics*, *Universidad del Valle*, *Cali*, *Valle del Cauca*, *Colombia*

**Introduction:** The prevalence of functional constipation (FC) is 12.0%. Children with FC frequently visit emergency services. Several guidelines provide recommendations for managing FC in children. The aim of this study was to identify the knowledge, attitudes, and practices related to FC in children among Latin American pediatricians and pediatric gastroenterologists.

**Methods:** Prospective cross‐sectional study. A survey was conducted addressing knowledge, attitudes, and practices regarding FC. Measures of central tendency and dispersion were calculated. A p‐value <0.05 was considered statistically significant.

**Results:** A total of 893 professionals participated, 80.2% were pediatricians and 19.8% pediatric gastroenterologists. Of them, 25.0% were Colombian; 54.5% worked in public institutions; 52.4% in outpatient care; and 36.8% had more than 15 years of experience. When comparing general knowledge between pediatric gastroenterologists and pediatricians, it was found that gastroenterologists more frequently use the Rome IV Criteria (p=0.000), place greater importance on physical examination (p=0.001), better correlate stool types using the Bristol Stool Scale (p=0.007), rarely request referrals (p=0.000); request abdominal X‐rays to assess treatment response (p=0.032); prescribe osmotic (p=0.048) and stimulant laxatives (p=0.000), saline enemas (p=0.002), and higher doses of PEG (p=0.0221). Pediatricians more frequently use abdominal X‐rays as a diagnostic tool for FC (p=0.000), mainly to identify the cause of severe abdominal pain (p=0.008), more often resort to complementary treatments (p=0.000), and are less familiar with quality of life scales (p=0.000).

**Conclusions:** Differences in knowledge, attitudes, and practices regarding FC in children were identified between pediatricians and pediatric gastroenterologists. These findings highlight the need to strengthen continuing education on FC in children.

## 808 PREDICTORS OF TREATMENT RESPONSE FOR CHILDREN WITH RUMINATION SYNDROME

*Toby Chen* ^*1,2*^, *Ashley Kroon van Diest* ^*3*^, *Dennis Yang* ^*1*^, *Janice Khoo* ^*1*^, *Raul Sanchez* ^*1*^, *Neetu Bali Puri* ^*1*^, *Karla Vaz* ^*1*^, *Desale Yacob* ^*1*^, *Carlo Di Lorenzo* ^*1*^, *Peter Lu* ^*1*^

^*1*^ *Division of Gastroenterology, Hepatology and Nutrition, Department of Pediatrics*, *Nationwide Children's Hospital*, *Columbus*, *OH*; ^*2*^ *Washington State University Elson S. Floyd College of Medicine*, *Spokane*, *WA*; ^*3*^ *Department of Pediatric Psychology and Neuropsychology*, *Nationwide Children's Hospital*, *Columbus*, *OH*

**Background:** Rumination syndrome (RS) is a disorder of gut‐brain interaction (DGBI) that can be challenging to treat. Predictors of treatment response are unknown. Therefore, our objective was to evaluate potential predictors of treatment response in children diagnosed with RS.

**Methods:** We performed a retrospective review of children diagnosed with RS at our institution. Demographic data, medical and surgical history, and clinical symptoms at baseline and follow‐up were recorded. Treatment response was defined as no longer having any vomiting (rumination leading to expulsion). Baseline factors potentially associated with treatment response were analyzed using Fisher's exact and Wilcoxon rank‐sum tests.

**Results:** We included 148 children with RS (60% female, median age 13 years). The median age at diagnosis was 13 years, and the age at symptom onset was 11 years. Most children also had additional DGBI diagnoses (35%), including functional dyspepsia (7%), irritable bowel syndrome (19%), functional abdominal pain (3%), cyclic vomiting syndrome (3%), and functional nausea/vomiting (30%). Additionally, 42% had anxiety/depression, 10% had a history of developmental delay, and 5% had a prior diagnosis of an eating disorder. Children required gastric tube feeding (5%), jejunal tube feeding (14%), and parenteral nutrition (1%). At baseline, 35% had seen a psychologist, 29% had tried behavioral treatment, and 5% had tried baclofen. Anxiety about eating was explicitly noted in 8% of patients. School attendance varied, with most (63%) attending school in person, 14% participating in home‐bound instruction, 3% attending school online, and 10% not attending school. Twenty‐one percent were reluctant to accept their diagnosis and/or treatment. Following their initial evaluation, outpatient treatment was recommended for 70%, intensive outpatient program for 14%, and inpatient treatment for 17%. Symptoms at baseline and after treatment (median follow‐up 4 months) are shown in **Table 1**. We found that children and parents who were reluctant to accept the diagnosis of RS were numerically less likely to respond to treatment, but this did not meet our criteria for statistical significance (39% vs. 58%, p=0.07). The remaining baseline factors we evaluated were not associated with response, including concurrent diagnosis of DGBI, anxiety/depression, developmental delay, eating disorder, supplemental nutrition, prior or regular psychology visits, anxiety about eating, school attendance, and skipping meals. Frequency of re‐swallowing at baseline also was not associated with treatment response.

**Conclusion:** Children with RS and parents who are reluctant to accept the diagnosis and/or treatment may be less likely to respond to treatment. This finding highlights the importance of proper education during diagnosis.


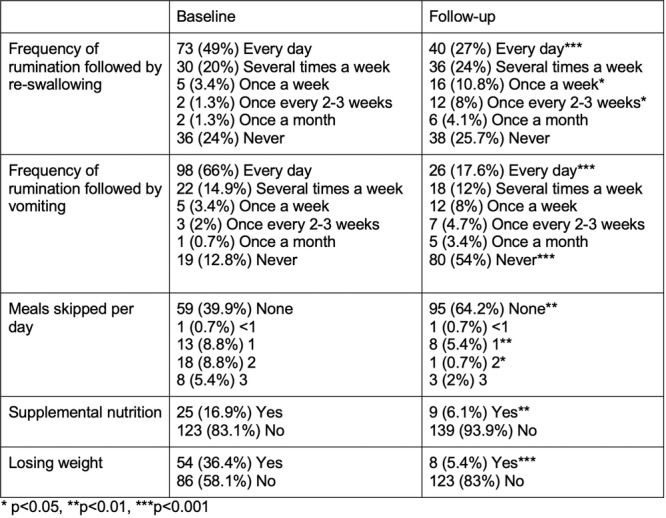


**Table 1:** Symptoms at baseline and follow‐up after treatment (N=148, median follow‐up 4 months).

## 809 MEAN NOCTURNAL BASELINE IMPEDANCE IN CHILDREN WITH GASTROESOPHAGEAL REFLUX DISEASE ‐ A SINGLE‐CENTER EXPERIENCE

*Donna Cheung*, *Aamer Imdad*

*Pediatric Gastroenterology*, *The University of Iowa*, *Iowa City*, *IA*

**Background:** Additional pH‐Impedance Monitoring (pH‐MII) metrics, like the Mean Nocturnal Baseline Impedance (MNBI), are thought to improve the diagnosis of gastroesophageal disease (GERD). Adults have a cutoff value for MNBI of 2292 Ω. A cut off has not been established in children but limited studies suggest a lower MNBI is associated with GERD. The aim of this study is to evaluate the value of MNBI in children with GERD.

**Methods:** A retrospective chart review was done from July 2019 to April 2025 at the University of Iowa. Demographic data, clinical data, and pH‐MII metrics including MNBI (if available) were collected. Children under 21 years of age who underwent pH‐MII during this period were included. Children without MNBI results were excluded. pH‐MII were considered to have an abnormal acid exposure time (AET) when Reflux Index (RI) was high for age (>7 for children, >10 for infants). MNBI data was summarized using medians and interquartile ranges (IQR). The Wilcoxon rank‐sum test was performed to compare differences between groups, given its appropriateness for non‐normally distributed data. A subgroup analysis was considered for a cohort of children without eosinophilic esophagitis (EoE) or esophageal anatomical abnormalities and use of antacid medications. A two‐sided p‐value of less than 0.05 was considered statistically significant. All analyses were conducted using STATA version 14.

**Results:** Ninety‐seven pH‐MII were completed in 94 children, of which 63 pH‐MII in 62 children met inclusion and exclusion criteria. One additional study was reported to have faulty impedance sensors which was excluded, leaving a total of 62 studies for analysis. The study population included children aged 1 month to 19 years (median age 12 years) with 56% female and 87% white.

Of the 62, twenty‐three were included in the subcohort after excluding children with EoE/esophageal anatomical abnormalities, children who were on antacids (n=24), and children whose antacid status was unknown (n=15) during pH‐MII. The subcohort included children aged 1 month to 17 years (median age 12 years) with 61% female and 83% white.

In all children, the median MNBI in children with abnormal AET (n = 7) was 338 (IQR 293‐1937) and it was 2505 (IQR 1907‐3060) in children with normal AET (n=55) (p <0.05) [See box plot 1]. In the subcohort, the median MNBI was also 435 (IQR 131‐ 3299) in children with abnormal AET (n=3) and 2343.5 (IQR 2074.5‐3259.5) in children with normal AET (n=20) (p >0.05) [See box plot 2].

**Conclusions:** Children with GERD defined as AET on pH‐MII have lower MNBI than those without. This was shown in both the overall study population and in the subcohort and was statistically significant in the former. The subcohort excluding children with EoE/esophageal anatomic abnormalities and including only children documented to be off antacids during pH‐MII likely did not reach statistical significance because of the sample size. There is value in using MNBI on pH‐MII to evaluate GERD in children. Additional research is needed to establish a cut off for MNBI in children.


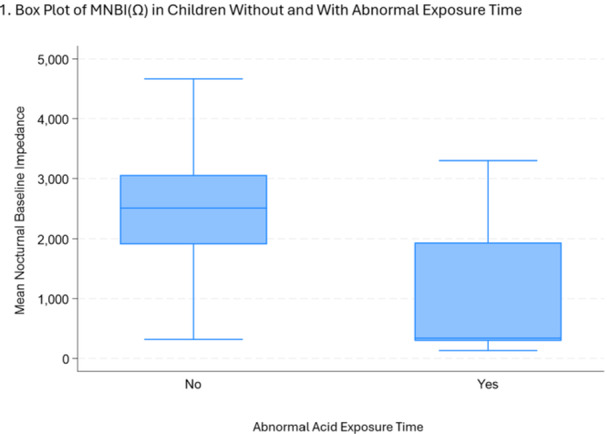


1. Box Plot of MNBI(Ω) in Children Without and With Abnormal Exposure Time


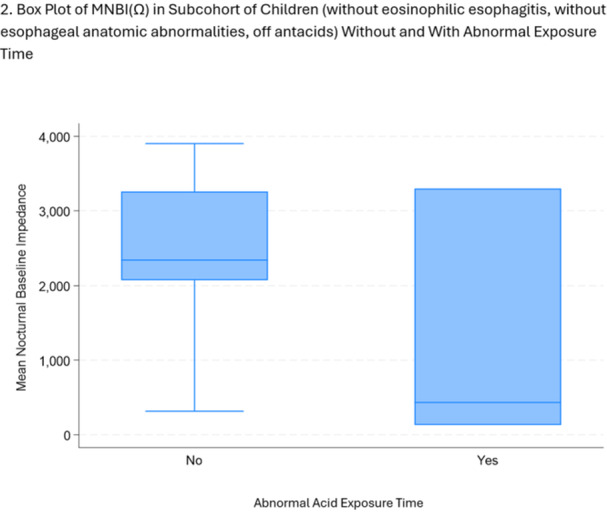


2. Box Plot of MNBI(Ω) in Subcohort of Children (without eosinophilic esophagitis, without esophageal anatomic abnormalities, off antacids) Without and With Abnormal Exposure Time

## 811 USING A STANDARDIZED TREATMENT PLAN FOR THE TREATMENT OF CHRONIC CONSTIPATION: A QUALITY IMPROVEMENT PROJECT AT A LARGE PEDIATRIC INSTITUTION

*Trevar Dahl* ^*1*^, *Sapna Khemka* ^*3*^, *Kaelin Kiss* ^*1*^, *Christine Carter‐Kent* ^*2*^, *Kevin Watson* ^*2*^

^*1*^ *Pediatric Residency*, *Akron Children's Hospital*, *Akron*, *OH*; ^*2*^ *Akron Children's Hospital*, *Akron*, *OH*; ^*3*^ *Nationwide Children's Hospital*, *Columbus*, *OH*

Introduction: Constipation is a common diagnosis in the pediatric population. Studies have shown that a structured bowel management plan can standardize practice and help decrease costs for constipation related health care. Generalized treatment plans have been created at other pediatric institutions, but Akron Children's did not have a structured plan. The investigators sought to develop and implement a Constipation Treatment Plan (CTP) in the emergency department, outpatient clinics, and general inpatient units with the goal of reaching a 10% utilization rate by June 30, 2025, for patients who have a diagnosis of constipation.

**Methods:** The improvement was undertaken at a large midwestern free‐standing children's hospital. A team was formed that included GI providers and residents using the Model for Improvement for quality improvement (QI) methodology. Key interventions included creating a standardized CTP and incorporating it into the EPIC electronic medical record as a standard treatment plan available to any patient with a diagnosis of constipation, educating providers on availability, use, and recommendations through live virtual demonstrations, in person discussions with various stakeholders, and placing physical reminders to use the CTP in resident workrooms. This plan was created among other treatment plans already in use within the hospital system for asthma, allergies, and migraines, and is found with these treatment plans on the electronic medical record. The target audience includes 50 primary care practices, 3 emergency rooms, a large hospitalist group, as well as 16 medical specialties. The primary outcome measure was the percentage of patients with a principal diagnosis of constipation who were provided with a CTP.

**Results:** Inpatient usage of the CTP increased to approximately 10%. Outpatient usage has varied from 1‐2%. ED usage has increased and is approaching 10%. The inpatient usage increased and stayed at around 10% after placing physical notes on all resident workstations to serve as a reminder to use the CTP.

**Conclusion:** The CTP has been met with positive feedback, yet varying usage rates, with higher usage rates in the hospital setting compared to outpatient. Virtual education sessions proved helpful to initially introduce the CTP, but further work will be required to continue to increase its implementation both inside and outside the hospital. With continued utilization, the QI team will plan to evaluate if the CTP affects GI referrals for constipation.


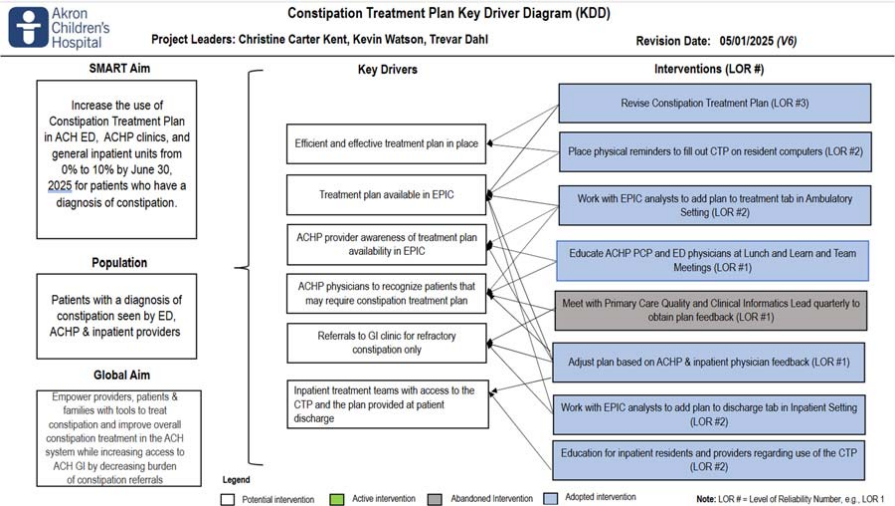


Key Driver Diagram


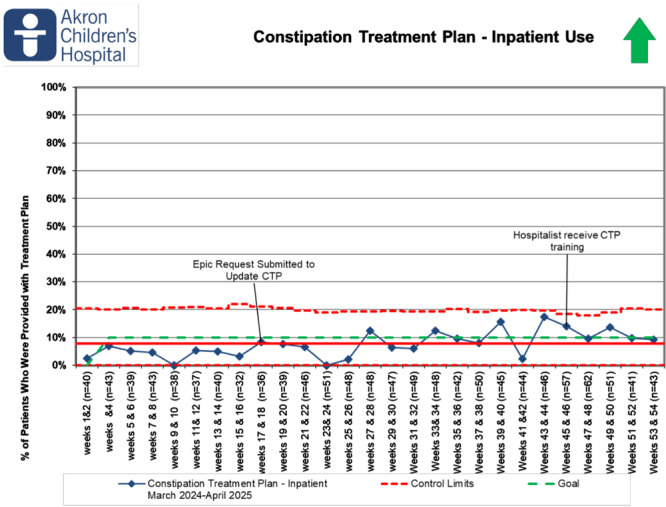


Inpatient P Chart

## 814 CLINICAL OUTCOMES IN CHILDREN AND ADOLESCENTS WITH DISORDERS OF GUT‐BRAIN INTERACTION (DGBI) SEEN IN MULTIDISCIPLINARY DGBI VS GENERAL GASTROENTEROLOGY CLINICS

*Rocco Giegerich* ^*1*^, *Rashmi Sahay* ^*2*^, *Jen Hardy* ^*1*^, *Priyanshi Shah* ^*1*^, *Neha Santucci* ^*1,3*^

^*1*^ *Gastroenterology*, *Cincinnati Children's Hospital Medical Center*, *Cincinnati*, *OH*; ^*2*^ *Biostatistics*, *Cincinnati Children's Hospital Medical Center*, *Cincinnati*, *OH*; ^*3*^ *Pediatrics*, *University of Cincinnati College of Medicine*, *Cincinnati*, *OH*

**Background:** The Disorders of Gut Brain Interaction (DGBI) Clinic at Cincinnati Children's Hospital Medical Center (CCHMC) provides a multidisciplinary approach to treat children with complex DGBI presentations often not relieved by general treatments. This clinic provides integrated care with a neurogastroenterologist, psychologist and dietitian and utilizes state‐of‐the‐art treatments such as auricular neurostimulation and endoscopic pyloric botulinum toxin injection and dilation with standard of care treatments. In contrast, general gastroenterology (GI) clinics would, in general, treat patients with medications, refer to psychology for anxiety management and sometimes, pain coping. We retrospectively compared patient outcomes from the DGBI and general GI Clinics.

**Methods:** We reviewed demographics, medical history, symptoms, co‐morbidities, and treatments from charts of patients who met the Rome 4 criteria for a DGBI and were seen at the CCHMC DGBI and/or general GI clinics from 2021‐2022. Outcomes included validated questionnaire responses of the Pain Numeric Rating Scale and the Functional Disability Inventory (FDI) questionnaire. We also recorded subjective symptom ratings from physician‐documented notes at baseline and at the first two follow‐up visits. These were coded as resolved, improved, worsened and no change. Outcomes were examined over time for each group and compared between the two clinic groups.

**Results:** From the DGBI clinic cohort (n=271), mean age was 15.58 y ± 2.78, 74.2% were females and 88.2% were Caucasian. From the general GI clinic (n=205), mean age was 14.65 y ± 3.06, 70.7% were female and 89.3% were Caucasian. More Hispanic patients were seen in the general GI clinic (p=0.035). Diagnoses of functional dyspepsia (p<0.0001) and rumination (p=0.012) were more commonly seen in the DGBI clinic versus irritable bowel syndrome (p=0.002) and functional nausea and vomiting (p=0.0019) in the general GI clinic. The presence of cyclic vomiting syndrome (CVS) and other DGBI did not differ significantly between the types of clinics (p=0.118).

More patients from the DGBI clinic experienced co‐morbidities including psychological disorders (p=0.042), eating disorders (p=0.026), joint hypermobility, orthostatic intolerance, migraine, sleep disturbance, and musculoskeletal pain (p<0.0001 for all groups). Patients from the DGBI clinic also had more non‐GI related hospital admissions (p=0.002).

While assessing objective questionnaires, DGBI clinic patients had worse pain numeric rating scale components (higher baseline, worst pain, average pain, least pain, and pain at the time of visit) and disability (FDI) scores (p<0.0001 for all, Table 1). Both groups showed clinical improvements in worst pain and average pain over the first two follow‐up visits (p<0.0001 for all groups, Table 2). Comparing baseline to second follow‐up visit, the mean disability scores (FDI) in DGBI clinic improved from 22.4 to 17.9 (p<0.0001) while disability scores for general GI clinic did not significantly change (13.5 vs 11.0, p=0.120).

In contrast, the general GI clinic patients more frequently reported subjective improvements (from physician documented notes) in abdominal pain (p=0.033), regurgitation (p=0.036), and diarrhea (p=0.010) than DGBI clinic patients.

**Conclusion:** Patients presenting to multidisciplinary DGBI clinics presented with worse symptom severity and comorbidities. DGBI clinics improved functional disability more than general GI clinics. However, patients from the general GI clinic reported greater subjective improvements in abdominal pain, regurgitation, and diarrhea than from the DGBI clinic. Thus, the current standard of care treatments may hold value for typical and less severe cases of DGBI while multidisciplinary clinics will benefit those with more complex and severe presentations of DGBI, particularly targeted at improving functioning in this population.


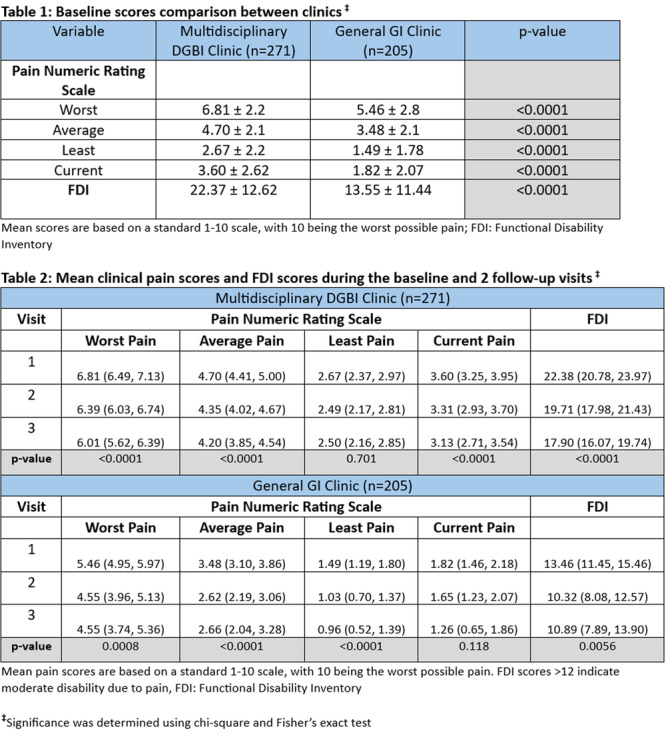


## 815 PREVALENCE AND POSSIBLE ASSOCIATIONS OF RUMINATION SYNDROME IN TODDLERS, SCHOOLCHILDREN AND ADOLESCENTS ACCORDING TO THE ROME IV CRITERIA

*Natali Gonzalez Rozo* ^*1,2*^, *Carlos Velasco‐Benitez* ^*1*^, *Daniela Velasco‐Suarez* ^*1*^

^*1*^ *Gastrohnup Research Group*., *Universidad del Valle Facultad de Salud*, *Cali*, *Valle del Cauca*, *Colombia*; ^*2*^ *Epidemiology*, *Universidad Libre ‐ Campus Cali*, *Cali*, *Valle del Cauca*, *Colombia*

**Introduction:** According to the Rome IV Criteria, disorders of the gut‐brain interaction (DGBIs) in schoolchildren and adolescents have a global prevalence of 23.0%. The prevalence of rumination syndrome (RS) is 1.0%. In Latin America, reports related to RS and its potential associations are lacking. The aim of this study was to describe its prevalence in children from three Latin American countries and identify possible associations.

**Methods:** An analytical, observational, and descriptive study was conducted in children aged 4–18 years old from schools in Colombia (n=67858), Mexico (n=2380), and Ecuador (n=951). Familial, clinical, and nutritional variables were considered. After obtaining informed consent/assent, participants completed the Spanish version of the Questionnaire for Pediatric Gastrointestinal Symptoms Rome IV (QPGS‐IV). The analysis included measures of central tendency, univariate and bivariate analyses, and multiple logistic regression, with corresponding odds ratios (OR) and 95% confidence intervals (95% CI). A p‐value <0.05 was considered statistically significant.

**Results:** A total of 10089 children were included (mean age 12.7±3.1 years; 58.2% adolescents; 52.1% of mixed race); 82.7% attended public school; 67.0% were Colombian; 16.5% were only children; 40.6% were firstborn; 40.0% had separated/divorced parents; 2.3% had a family history of DGBIs; 34.1% were born by cesarean section; 13.5% were premature; 29.2% were undernourished according to BMI and 12.2% according to height‐for‐age. The prevalence of any DGBI was 21.9%, with RS in 0.4%. RS was more likely in girls (OR=2.10; 95%CI=1.24–3.66; p=0.0032) and in those with overlap (OR=29.69; 95%CI=18.11–48.46; p=0.0000). Associated factors for RS included sex (OR=1.59; 95%CI=0.95–2.67; p=0.0076) and overlap (OR=26.44; 95%CI=16.50–42.2; p=0.000).

**Conclusions:** 1) Early identification of RS allows for timely diagnosis; 2) RS in infants is clinically distinct from that in older children and tends to have a better prognosis; 3) High‐resolution impedance manometry is useful for diagnosis; 4) Studies on RS management should include non‐pharmacological treatment based on high‐intensity diaphragmatic breathing; 5) Our prevalence is similar to global reports, with risk factors including female sex and symptom overlap.

## 816 SOME ADULT DISORDERS OF GUT‐BRAIN INTERACTION MAY OCCUR IN CHILDHOOD

*Natali Gonzalez Rozo* ^*1*^, *Michelle Higuera* ^*3,4*^, *Carlos Velasco‐Benitez* ^*2*^, *Daniela Velasco‐Suarez* ^*2*^

^*1*^ *Pediatrics*, *Hospital Universitario Erasmo Meoz Cucuta*, *Cúcuta*, *North Santander*, *Colombia*; ^*2*^ *Gastrohnup Research Group*, *Universidad del Valle*, *Cali*, *Valle del Cauca*, *Colombia*; ^*3*^ *Pediatrics*, *Universidad Nacional de Colombia*, *Bogotá*, *Bogota*, *Colombia*; ^*4*^ *Pediatrics*, *Universidad El Bosque*, *Bogotá*, *Bogota*, *Colombia*

**Introduction:** Some disorders of the gut‐brain interaction (DGBIs) are well‐defined in adults but are not yet included in the pediatric Rome IV Criteria. These include functional dysphagia (FD), functional diarrhea (FDr), chest pain (CP), biliary pain (BP), burning pain (BPn), and proctalgia fugax (PF). The aim of this study was to determine the prevalence of FD, FDr, CP, BP, BPn, and PF using the Questionnaire for Pediatric Gastrointestinal Symptoms Rome IV (QPGS‐IV), adapted from the adult version.

**Methods:** After presenting the project at the 2024 LASPGHAN research competition on pediatric gastroenterology, hepatology, and nutrition for young investigators in Latin America, a prospective cross‐sectional study was conducted in children from public schools in three Colombian cities: Cucuta, Maicao, and Corozal. Data were collected using the adapted QPGS‐IV questionnaire, which included additional questions related to adult‐model DGBIs. Sociodemographic, clinical, nutritional, and family‐related variables were included. Statistical analysis involved descriptive statistics, univariate and bivariate analysis, and multivariate logistic regression. Odds ratios with 95% confidence intervals were calculated, considering p<0.05 as statistically significant.

**Results:** A total of 789 children were included (13.7±2.8 years old; 52.8% male; 45.4% from Cucuta; 50.8% mixed race). Among them, 68.7% were only children; 57.6% firstborn; 7.4% had separated/divorced parents; 54.8% were born via cesarean section; 14.7% were premature; and 25.7% and 12.2% were malnourished according to BMI and height‐for‐age, respectively. Regarding DGBIs described in adults, the children showed the following prevalence: FD (0.6%), FDr (0.8%), CP (0.4%), BP (0.1%), BPn (1.0%), and PF (3.4%). Factors associated with the presence of any DGBIs included depression (OR=1.87; 95%CI=1.09–3.21; p=0.022) and school absenteeism (OR=3.04; 95%CI=1.75–5.27; p=0.000). For PF specifically, being schoolchildren (p=0.017) and exhibiting depressive traits (p=0.003) were significant factors.

**Conclusions:** Using the adapted QPGS‐IV, the presence of FD, FDr, CP, BP, BPn, and PF was identified in this pediatric population. These findings suggest that such DGBIs should be considered in future revisions of the Rome Criteria, along with attention to psychosocial and behavioral factors in their assessment.

## 817 THE CVS FREQUENCY‐SEVERITY‐DURATION SCALE AS A MEASURE OF CYCLIC VOMITING SYNDROME BURDEN: PRELIMINARY VALIDATION IN A PEDIATRIC SAMPLE

*Annie Gottinger* ^*1*^, *Keri Hainsworth* ^*1*^, *Julie Banda* ^*1*^, *W. Hobart Davies* ^*2*^, *Kimberly Brown* ^*1*^, *B Li* ^*1*^, *Katja Karrento* ^*1*^

^*1*^ *Department of Pediatrics*, *Medical College of Wisconsin Department of Pediatrics*, *Milwaukee*, *WI*; ^*2*^ *Psychology*, *University of Wisconsin‐Milwaukee Libraries*, *Milwaukee*, *WI*

**Background and Preliminary Data:** Cyclic vomiting syndrome (CVS) is a debilitating disorder characterized by episodes of intense nausea and vomiting interspersed with periods of normal health. Due to the episodic nature of CVS, standard outcome surveys do not effectively quantify the significant burden of CVS on patients and families. A recent study (Bujarska, et al., JPGN 2025) indicated that vomiting episode characteristics such as emesis frequency, severity, and duration are key to accurate diagnosis of CVS. As a first step toward developing a comprehensive pediatric CVS burden outcome measure, the aim of the current study is to evaluate the strength of a CVS composite score versus individual parameters in relationship to indices of CVS burden.

**Objective:** Evaluate the strength of a CVS composite score (CVS Frequency‐Severity‐Duration; CVS FSD) vs. individual factors (episode frequency, severity, and duration) in relationship to indices of functioning: PedMIDAS disability measure, vomiting severity, and headache severity. We hypothesized that the CVS composite score has a stronger association with indices of CVS burden than individual factors alone. We further hypothesized that all associations would be stronger in patients with CVS compared to patients with similar symptoms but not diagnosed as CVS (i.e. non‐CVS group).

**Methods:** This study was a secondary analysis of data from a larger trial. The cohort consisted of 108 patients between ages 3‐18 years who presented with episodic nausea and vomiting +/‐ other symptoms (e.g. abdominal pain, headache, photophobia) to the Emergency Department, outpatient Gastroenterology clinic, or inpatient ward at Children's Wisconsin hospital. All patients were diagnosed with CVS or non‐CVS causes of vomiting. Patients and caregivers completed specific questions regarding the features of vomiting episodes (frequency, severity, duration). A CVS composite score was created by calculating the product of three individual parameters: Frequency of vomiting episodes, Severity of abdominal pain, and Duration of vomiting episodes (F*S*D). As indices of CVS burden, patients also completed a validated migraine disability measure (PedMIDAS), self‐reported headache pain intensity, and a validated measure of nausea severity. Pearson correlations were used to assess associations between the CVS composite score and the individual measures, with PedMIDAS (primary index of burden), headache severity, and nausea severity (secondary indices of burden).

**Results:** For the 108 patients enrolled, the CVS composite score was more strongly associated with pedMIDAS (*r*=0.58, *p*<0.001) than any of the individual factors, including frequency (*r*=0.37,0.001), severity (*r*=0.16, *p*=0.146), or duration (*r*=0.43, *p*<0.001). Considering the CVS composite and individual parameters, only abdominal pain severity was associated with headache severity (*r*=0.33, *p*=0.003) and nausea severity (*r*=0.33, *p*=0.003). Subgroup analyses: Within the CVS group, the CVS composite score was more strongly associated with PedMIDAS scores (*r*=0.62, *p*<0.001) than frequency (*r*=0.41, *p*=0.011), severity (*r* =0.13, *p*=0.426), or duration (*r*=0.39, *p*=0.016) alone. Similarly, within the non‐CVS subgroup, CVS composite score was more strongly associated with PedMIDAS (*r*=0.65, *p*<0.001) than frequency (*r*=0.37, *p*=0.014), severity (*r*=0.20, *p*=0.189) or duration (*r*=0.51, *p*<0.001) alone. As with the whole sample, only the abdominal pain severity parameter was associated with headache severity for the CVS subgroup (*r*=0.36, *p*=0.029) and with nausea severity for the non‐CVS subgroup (*r*=0.46, *p*=0.002).

**Conclusions:** This study is an initial step towards the development and validation of a CVS burden scale for children and adolescents that can be applied in future clinical trials. Analyses showed that a CVS composite score has a stronger association with PedMIDAS disability measure than any individual factors alone. Further work is needed to develop other disease‐specific outcome metrics. We conclude that a CVS composite score would more efficiently capture overall disease burden than individual parameters.

## 818 CHARACTERIZATION AND CLINICAL EVOLUTION OF INEFFECTIVE ESOPHAGEAL MOTILITY IN PEDIATRIC PATIENTS: RETROSPECTIVE STUDY IN A REFERENCE CENTER

*Ana Guerrero*, *Erick Toro Monjaraz*, *Flora Zarate‐Mondragon*, *Roberto Cervantes Bustamante*, *Dr. José Francisco Cadena León*, *Ericka Montijo‐Barrios*, *Karen Ignorosa‐Arellano*, *Jaime Ramirez‐Mayans*

*Pediatric Gastroenterology*, *Universidad Nacional Autonoma de Mexico*, *Mexico City*, *CDMX*, *Mexico*

**Objectives and Study:** Ineffective esophageal motility (IEM) is considered the most common esophageal motor disorder. Due to its high frequency among motor disorders and there are no studies in pediatric patients regarding its evolution. This study aims to describe the evolution, clinical and manometric characteristics of pediatric patients with a diagnosis of IEM.

**Methods:** Cross‐sectional, observational, retrospective and analytical study in a third level hospital with a total of 11 patients from 14 to 18 years with a diagnosis of IEM confirmed by a high‐resolution esophageal manometry study were included, in a period from 2017 to 2023. We evaluated the presence or absence of gastrointestinal symptoms and its evolution 6 to 12 months after diagnosis.

**Results:** In every patient, the diagnosis of IEM was established by manometry study according to the Chicago classification 4.0, where we observed the following findings: Mean upper esophageal sphincter length was 3.5 cm (SD 0.522), while the pressure The mean upper esophageal sphincter was 130.1 mmHg (SD 1.859), the mean length of the esophagus was 17.7 cm (SD 0.750), mean pressure of the lower esophageal sphincter of 16 mmHg (SD 0.944), the mean length of the lower esophageal sphincter was 4 cm (SD 0.447), with a mean pressure reversal point of 36.4 (SD 0.750). SD 0.603), as well as a mean intra‐abdominal length of 3.3 cm (SD 1.513) and an average DCI of 337 mmHg.s.cm (SD 0.934), finally an average IRP of 12.3 mmHg (SD 0.674). Subsequently, the symptoms at the time of diagnosis were evaluated, where 45.4% of the patients presented vomiting, while 54.5% presented dysphagia, the presence of reflux and heartburn occurred in 81.8%, while abdominal pain and distension only occurred in 36.3% and it was found that none of the patients presented alterations in the evacuation pattern. Finally, the symptoms after diagnosis were evaluated from 6 to 12 months with an average of 9.2 months, 81.8% of the patients didn't present vomiting, while in 72% the dysphagia had resolved, the presence of reflux and heartburn persisted in 36.3%, while abdominal pain and distension were still present in only 9%.

**Conclusions:** IEM is a motor disorder with not many information in the pediatric population. In our study we observed that dysphagia was not the pivotal symptom, but rather the symptoms related to gastroesophageal reflux such as heartburn and regurgitation. In addition, during follow‐up a large percentage of patients decreased the frequency of symptoms overall.

## 820 USE OF ANORECTAL MANOMETRY IN THE EVALUTION AND MANAGEMENT OF ENCOPRESIS IN CHILDREN

*Serena Haver* ^*1*^, *Mariana Middelhof* ^*1,2*^, *Catherine Chao* ^*1,2*^

^*1*^ *Inova Health System Office of Continuing Medical Education*, *Falls Church*, *VA*; ^*2*^ *Pediatric Specialists of Virginia*, *Fairfax*, *VA*

**Background and Objectives:** Encopresis affects approximately 4% of children in the United States between the ages of 4 and 17 years. Anorectal manometry (ARM) is a diagnostic tool that can aid in evaluating chronic constipation and guiding individualized treatment plans. This single center study aimed to assess how frequently patients with encopresis are referred for ARM and to evaluate clinical outcomes following the procedure.

**Methods:** We conducted a single‐center retrospective chart review of patients from 6 to 18 years old who were diagnosed with encopresis at a pediatric gastroenterology outpatient clinic between June 1, 2024 and June 30, 2024. We collected data on symptom resolution or persistence at follow‐up visits, ARM referrals, and outcomes post‐ARM.

**Results:** Of the 34 patients identified, 1 was excluded due to significant autism. Of the remaining 33, 27 returned for at least one follow‐up visit. Thirteen achieved symptom resolution by the first follow‐up, and another 3 achieved resolution at subsequent follow‐ups. Eleven children never achieved symptom resolution. Among the 14 patients with persistent symptoms at the first follow‐up, 3 were referred for ARM. All 3 were found to have megarectum and were treated with escalated senna dosing and biweekly maintenance cleanouts. Two achieved resolution by their first post‐ARM follow‐up, while the third showed a marked reduction in soiling frequency.

**Conclusions:** ARM proved useful in identifying underlying pathology and guiding management in children with persistent encopresis. While most patients responded to standard therapy, those with persistent symptoms benefitted from ARM‐directed treatment. The next phase of work will implement an encopresis algorithm to include earlier ARM referral after the first follow‐up visit in the cases of treatment failure, with the goal of reducing time to resolution.

Declaration of Generative AI and AI‐assisted technologies in the writing process:

During the preparation of this work the author(s) used ChatGPT to assist in editing the abstract. After using this tool/service, the author(s) reviewed and edited the content as needed and take(s) full responsibility for the content of the publication.

## 821 PREVENTING THE INCORRECT ADMINISTRATION OF OVER‐THE‐COUNTER RECTAL CONSTIPATION THERAPIES IN CHILDREN: A QUALITY IMPROVEMENT PROJECT IN RESPONSE TO A CRITICAL PATIENT SAFETY EVENT

*Stephanie Hum* ^*1*^, *Andrea Burnside* ^*2*^, *Astrela Moore* ^*3*^, *Elizabeth Maxwell* ^*1,4*^, *Manoj Mittal* ^*5,4*^, *Gina Murray* ^*2*^, *Kimberly Bennett* ^*2*^, *Kirsten Walaski* ^*1*^, *Keely McManamon* ^*6*^, *Janine McDermott* ^*1*^, *Angelica Del Grippo* ^*1*^, *Haley Pearlstein* ^*1*^, *Jennifer Webster* ^*1,4*^

^*1*^ *The Children's Hospital of Philadelphia Division of Gastroenterology Hepatology and Nutrition*, *Philadelphia*, *PA*; ^*2*^ *Department of Pediatrics*, *The Children's Hospital of Philadelphia*, *Philadelphia*, *PA*; ^*3*^ *Department of Pharmacy*, *The Children's Hospital of Philadelphia*, *Philadelphia*, *PA*; ^*4*^ *University of Pennsylvania Perelman School of Medicine*, *Philadelphia*, *PA*; ^*5*^ *The Children's Hospital of Philadelphia Division of Emergency Medicine*, *Philadelphia*, *PA*; ^*6*^ *The Children's Hospital of Philadelphia*, *Philadelphia*, *PA*

**BACKGROUND:** Over‐the‐counter (OTC) rectal constipation therapies, including suppositories and enemas, are commonly used to manage fecal impaction in children with constipation. Sodium phosphate enemas, which are commonly labeled “saline” enemas, can cause severe electrolyte derangements and dehydration leading to acute kidney injury, arrhythmias, and death. This risk is higher in younger children and with repeated use. In 2014, the FDA issued a safety announcement for sodium phosphate products after 29 serious adverse events in children, including one fatality, were reported between 1969 and 2012. At our institution, a recent safety event occurred in a 15‐month‐old child who was inadvertently given an adult‐sized OTC sodium phosphate enema by parents after a Pedia‐Lax saline enema had been recommended. The child was admitted to the pediatric intensive care unit with severe hypocalcemia causing tetany, hyperphosphatemia, and hypokalemia. This prompted a quality improvement project to identify root causes contributing to the incorrect administration of rectal constipation therapies and develop targeted interventions to address them.

**METHODS:** We conducted an anonymous survey of pediatric healthcare providers in primary care (PC), urgent care, the emergency department (ED), and the gastroenterology division (GI) at our large tertiary pediatric center. The survey asked about demographics, prescribing practices, and active ingredients in common OTC rectal constipation therapies based on product images. An interdisciplinary team of quality improvement advisors, pharmacists, physicians (ED, GI, and urgent care), advanced practice providers (PC and GI), and triage nurses (PC and GI) performed a root cause analysis using process mapping, a fishbone diagram, and a driver diagram to develop interventions to prevent the incorrect administration of OTC rectal constipation therapies in children.

**RESULTS:** A total of 247 pediatric healthcare providers who treat constipation responded to our survey. Of these, 62% (153/247) prescribed outpatient enemas with 44% doing so more than 1‐2 times per year. Even though 36% of providers instructed patients to buy enemas over the counter, only 26% of them used standardized patient educational materials. Among providers that prescribe enemas, only 15‐26% were able to correctly identify three sodium phosphate enema products. Over 50% misidentified sodium phosphate enemas as containing normal saline as the active ingredient. Knowledge improved slightly for providers who prescribed enemas more than 1‐2 times per year. Root cause analysis identified four key contributors to the incorrect administration of rectal constipation therapies: (1) inadequate provider knowledge, (2) inadequate family or patient knowledge, (3) misleading product labeling and limited availability of pediatric enemas, and (4) inadequate ordering safeguards in the electronic medical record (Figures 1 and 2). Interventions with the highest impact to effort ratio included updating and creating clinical decision‐making tools for constipation such as pathways and order sets, creating standardized educational materials for patients and providers, and implementing ordering safeguards in the electronic medical record (Figure 2).

**CONCLUSION:** Providers’ knowledge of OTC rectal constipation therapies is inadequate and is one of many factors driving incorrect administration in children. Addressing the identified root causes by implementing high impact interventions will increase provider knowledge and reduce the risk of future patient safety events.


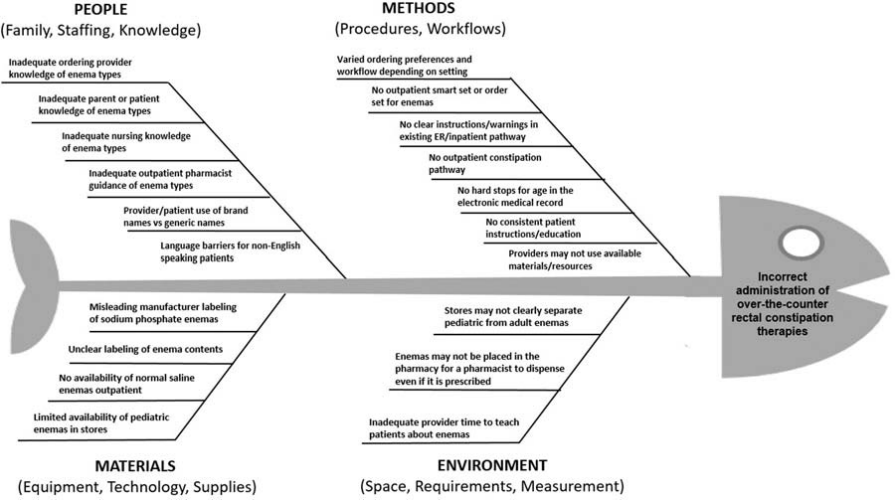


Figure 1. Fishbone diagram illustrating root causes of incorrect administration of rectal constipation therapies in children


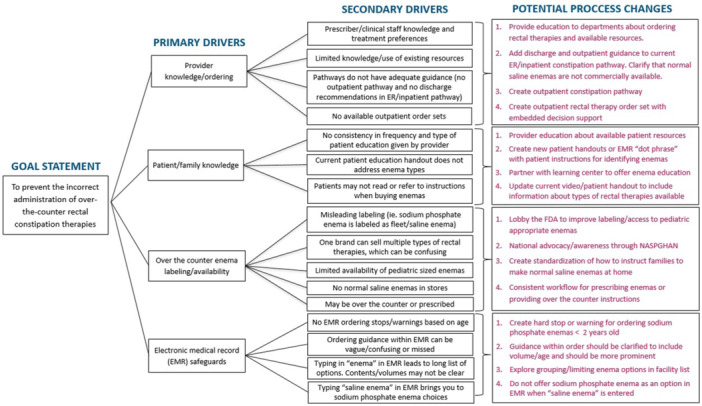


Figure 2. Driver diagram of root causes and change ideas to prevent incorrect administration of rectal constipation therapies in children

## 823 SLEEP RELATED BREATHING DISORDERS IN PATIENTS WITH HIRSCHSPRUNG DISEASE

*Marcos Mendoza* ^*1,4*^, *David Greis* ^*1,4*^, *Danny Del Cid‐Linares* ^*2,4*^, *Jaya Punati* ^*3,4*^, *Iris Perez* ^*2,4*^

^*1*^ *Pediatric Pulmonology and Sleep Medicine; Pediatric Gastroenterology*, *University of Southern California Keck School of Medicine*, *Los Angeles*, *CA*; ^*2*^ *Pulmonology and Sleep Medicine*, *Children's Hospital Los Angeles*, *Los Angeles*, *CA*; ^*3*^ *Gastroenterology, Hepatology, and Nutrition*, *Children's Hospital Los Angeles*, *Los Angeles*, *CA*; ^*4*^ *Children's Hospital Los Angeles*, *Los Angeles*, *CA*

**Background:** Congenital Central Hypoventilation Syndrome (CCHS) is a rare disorder characterized by alveolar hypoventilation and autonomic nervous system dysfunction. Patients with CCHS present with obstructive and central apneas, hypoventilation and hypoxemia. About 30% of patients with CCHS have Hirschsprung Disease. Although the prevalence of Hirschsprung Disease in CCHS is defined, the prevalence of Hirschsprung Disease patients with sleep related breathing disorders (SRBD) is unclear.

**Objective:** To determine the prevalence of sleep related breathing disorders in children with Hirschsprung Disease. We hypothesize that children with Hirschsprung are at increased risk of sleep related breathing disorders.

**Methods:** Retrospective chart review of 355 patients with Hirschsprung Disease seen at Children's Hospital Los Angeles between 2000‐2024. Demographics, Hirschsprung diagnosis, medical conditions, and polysomnographic data were collected.

Fishers exact test was used to analyze categorical data.

**Results:** Eighteen of 355 patients with Hirschsprung disease (5%) had polysomnography (PSG). Average age of first PSG was 7.1 years ± 5.7, earliest PSG was 3 months old, earliest OSA diagnosis was 8 months. 12/18 (67%) were male with average body mass index percentile (BMI) of 70.9% ± 31.9 (1 overweight, 4 obesity). 4 patients had CCHS, 4 had trisomy 21, 2 had chromosome 22 abnormality, 1 had fetal alcohol syndrome, 1 had cartilage hair syndrome, 1 had Aarskog syndrome/Mowat Wilson syndrome, 2 had ganglioneuroblastoma, and 5 had epilepsy/seizures.

Polysomnographic data of the cohort (n=18) can be found in Table 1. 72% (n=13) had obstructive sleep apnea (OSA), 33% were classified as mild (n=6), 22% as moderate (n=4), and 17% as severe (n=3). 2 patients had central sleep apnea and 1 had hypoventilation independently of CCHS.

Of the patients with obstructive sleep apnea (n=13), the average obstructive apnea hypopnea index (events per hour) was 25.5±68.2, central apnea index (events per hour) 1.9±2, baseline PetCO_2_(mmHg) 41.4±4.1, maximal end tidal CO_2_(mmHg) 48.6±5.2, time spent with CO_2_ >50 mmHg (%) 0.3±0.8, baseline SpO_2_(%) 94.5±2.6, lowest SpO_2_(%) 82.5±7.8, time spent with <90% O_2_ (%) 4.9±8.8.

5/11 patients with OSA were overweight/obese. There was no difference in OSA between those with BMI <85 percentile and BMI >85 percentile (p=0.6).

**Conclusion:** Patients with Hirschsprung disease do not routinely obtain polysomnographic studies assessing for sleep related breathing disorders. Our study showcases that patients who did receive PSG commonly had sleep related breathing disorders, obstructive sleep apnea being the most common. Obesity did not affect the presence of OSA. Our findings suggest screening for sleep related breathing disorders including polysomnography in patients with Hirschsprung disease.


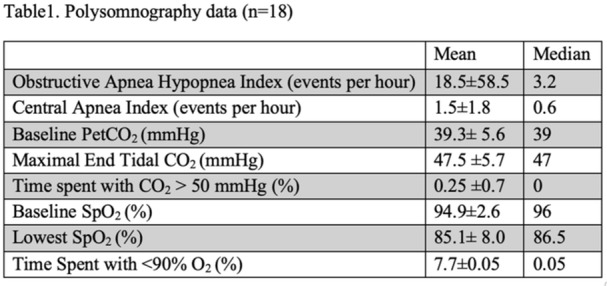


## 825 PREVALENCE AND POSSIBLE ASSOCIATIONS OF DISORDERS GUT‐BRAIN INTERACTION IN CHILDREN AGED 4 TO 18 YEARS WITH EPILEPSY

*Luz Murillas* ^*1*^, *Christian Rojas‐Ceron* ^*1,2*^, *Carlos Velasco‐Benitez* ^*1*^

^*1*^ *Pediatrics*, *Universidad del Valle*, *Cali*, *Valle del Cauca*, *Colombia*; ^*2*^ *Pediatrics*, *Hospital Universitario del Valle. Cali, Colombia*, *Cali*, *Valle del Cauca*, *Colombia*

**Introduction:** The prevalence of epilepsy in Colombia is 1.3%, and the global prevalence of disorders of the gut‐brain interaction (DGBIs) is 23.0%. The gut–brain–microbiota–epilepsy connection has been explored in preclinical studies, but clinical evidence remains limited. The presence of DGBIs in children with epilepsy has not been sufficiently investigated. The aim of this study was to determine the prevalence of DGBIs and potential associations in children with epilepsy.

**Methods:** A prospective, observational, descriptive study was conducted in children aged 4–18 years old who attended the neurology outpatient clinic at Hospital Universitario del Valle in Cali, Colombia. The Spanish‐validated version of the Questionnaire for Pediatric Gastrointestinal Symptoms Rome IV was used to identify DGBIs. Sociodemographic, clinical, and dietary variables were included. The analysis comprised measures of central tendency, univariate and bivariate analyses, with 95% confidence intervals (95% CI) and odds ratios (OR). A p‐value <0.05 was considered statistically significant.

**Results:** A total of 161 children were included (mean age 11.3±4.1 years); 55.3% were male; 5.6% were immigrants. Focal epilepsy was present in 66.9%, focal motor seizures in 54.3%, and intractable epilepsy in 26.1%. Valproic acid was used by 41.0% of the children; 1.9% had undergone epilepsy surgery, and 1.9% were on a ketogenic diet. The prevalence of DGBIs was 36.0%, with postprandial functional dyspepsia (FD) (16.8%) and functional constipation (FC) (13.0%) being the most common. Multiple DGBIs were reported in 10.6% of cases. Children attending private schools had a higher prevalence of DGBIs compared to those in public schools (40.5% vs. 59.5%, p=0.008). The risk of FD was higher in children with epilepsy and cerebral palsy GMFCS level III (7.4% vs. 92.6%, p=0.038). A history of epilepsy surgery was associated with a higher risk of FC (25.2% vs. 74.8%, p=0.005).

**Conclusions:** Our DGBI prevalence of 36.0% is similar to that reported in Turkish children, where the most common DGBI was irritable bowel syndrome. In conclusion, one‐third of children with epilepsy present with at least one DGBI, the most frequent being postprandial FD. Potential risk factors include cerebral palsy, epilepsy surgery, and type of educational institution.

## 828 ACHALASIA IN CHILDREN: A 10‐YEAR REVIEW OF SURGICAL OUTCOMES, NUTRITIONAL RECOVERY AND RECURRENCE

*Karla Ramirez‐Beltran*, *Erick Toro‐Monjaraz*, *Flora Zarate‐Mondragon*, *Ericka Montijo‐Barrios*, *Karen Ignorosa‐Arellano*, *Dr. José Francisco Cadena León*, *Martha Martínez Soto*, *Roberto Cervantes Bustamante*, *Jaime Ramirez‐Mayans*

*Pediatric Gastroenterology and Nutrition*, *Instituto Nacional de Pediatria*, *Mexico City*, *CDMX*, *Mexico*

**Background:** Achalasia is a rare esophageal motor disorder characterized by ineffective esophageal peristalsis and failure of the lower esophageal sphincter to relax. The first‐line treatment is Heller myotomy; however, peroral endoscopic myotomy (POEM) has emerged in recent years as an alternative, although it remains unavailable in many low‐resource settings. The main complications associated with treatment are esophageal perforation and gastroesophageal reflux during long‐term follow‐up (1,2).

**Objective:** To evaluate the nutritional status, treatment outcomes, complications, and persistent symptoms in pediatric patients diagnosed with achalasia at a tertiary care center.

**Methods:** A retrospective cohort study was conducted on pediatric patients diagnosed with achalasia between 2014 and 2024 at the National Institute of Pediatrics in Mexico City. All patients met the manometric diagnostic criteria according to the Chicago Classification versions 3.0 and 4.0. Data collected included demographics, nutritional status, achalasia subtype, manometric metrics, surgical approach, complications, and symptom recurrence during follow‐up.

**Results:** A total of 26 patients were diagnosed with achalasia, with Type II being the most common (73%, n=19). The median age at diagnosis was 12 years, and gender distribution was nearly equal (57.7% male, 42.3% female). Associated comorbidities included Down syndrome (n=3), Allgrove syndrome (n=3), Marfan syndrome (n=1), and autoimmune disease (n=1).

Nutritional status at diagnosis was assessed in 23 patients: 39.1% had severe malnutrition, 26.1% moderate, 13% mild, and 21.7% were eutrophic. High‐resolution esophageal manometry showed a mean IRP of 43.64 mmHg, DCI of 1820 mmHg.s.cm, and panesophageal pressurization in 78.3% (n=18).

Nineteen patients underwent Heller myotomy, with 17 receiving concomitant fundoplication. The Dor technique was used in 52.9% (n=9).

Primary complications included esophageal perforation in 15.8% of patients, with 11.5% occurring intraoperatively. Esophageal stenosis developed in 15.8% within one month postoperatively. Additional complications included pneumothorax (n=1), splenic rupture (n=1), and hyperfunctioning fundoplication.

At 6‐month follow‐up (n=16), 3 patients (11.5%) had persistent dysphagia to solids, 4 (15.4%) had vomiting related to feeding, and 5 (19.2%) reported thoracic pain. At 1‐year follow‐up (n=12), 3 patients (11.5%) continued with solid food dysphagia, and 1 patient each (3.8%) had dysphagia to liquids, vomiting, or thoracic pain.

Nutritional follow‐up was available for 15 patients: 30% showed nutritional improvement, while 20% remained severely malnourished, 20% moderately, 33.3% mildly, and 26.7% achieved normal nutritional status.

Postoperative manometry was performed in 4 patients, with a mean IRP of 4 mmHg, DCI of 730 mmHg.s.cm, and panesophageal pressurization in 75%. Four patients with postoperative strictures underwent additional interventions including balloon dilation.

**Conclusions:** Achalasia remains a rare diagnosis in pediatrics, with Type II as the predominant form. Most patients presented with severe malnutrition at diagnosis. Despite surgical intervention, a subset of patients continued to exhibit poor weight gain and persistent symptoms. Esophageal perforation was the most common immediate complication in our population, while postoperative esophageal stenosis contributed to the persistence or recurrence of symptoms such as dysphagia and emesis. Although gastroesophageal reflux is frequently reported in the literature, persistent dysphagia was the most common long‐term symptom in this cohort.

**References:**

1. Jarzebicka, D.; Czubkowski, P.; Sieczkowska‐Golub, J.; Kierkus, J.; Kowalski, A.; Stefanowicz, M.; Oracz, G. Achalasia in Children—Clinical Presentation, Diagnosis, Long‐Term Treatment Outcomes, and Quality of Life. J. Clin. Med. 2021, 10, 3917. https://doi.org/10.3390/jcm10173917.

2. Saliakellis E, Thapar N, Roebuck D, Cristofori F, Cross K, Kiely E, et al. Long‐term outcomes of Heller's myotomy and balloon dilatation in childhood achalasia. European Journal of Pediatrics. 2017 May 23;176(7):899–907. doi:10.1007/s00431-017-2924-x

## 829 HOW WELL DO ESOPHAGRAMS PREDICT ESOPHAGEAL MOTILITY DISORDERS? A SINGLE‐CENTER REVIEW COMPARING PEDIATRIC HIGH RESOLUTION ESOPHAGEAL MANOMETRY AND FLUOROSCOPIC ESOPHAGRAM

*Kevin Ratnasamy*, *Shikib Mostamand*

*Division of Pediatric Gastroenterology, Hepatology, and Nutrition*, *Stanford University School of Medicine*, *Palo Alto*, *CA*

Fluoroscopic esophagram is often part of the diagnostic evaluation for dysphagia. These reports note findings of gastroesophageal reflux, hiatal hernia, and may include comments on abnormal esophageal peristalsis observed during the study. However, esophagrams are transit studies which provide a snapshot in time and while may be suggestive, they do not definitively assess esophageal mucosa or function. In this retrospective study, our aim was to evaluate the sensitivity and specificity of fluoroscopic findings compared to the current gold standard for measuring esophageal peristalsis, high‐resolution esophageal manometry (HREM).

This study reviewed all HREM performed from September 2021 to May 2025 (n = 104). Of these, 67 patients also underwent fluoroscopic esophagram or upper GI series in which the radiologist interpretation commented on the esophagus. All fluoroscopic studies were reviewed for reflux, hiatal hernia, and any discussion regarding peristalsis (“dysmotility”, “impaired motility”, “lack of peristalsis”, or comments on primary, secondary, or tertiary contractions). HREM reports were reviewed for indication, recorded manometric values, and characterization of esophageal motility utilizing the Chicago Classification 4.0 (CC4.0).

The average age of this cohort was 13 years (range 1 – 23). There were 45 male and 22 female patients. The most common indication for HREM was dysphagia (n = 52, 77.6%). Other noted indications included globus (n = 3, 4.5%), regurgitation (n = 2, 3%), inability to belch (n = 2, 3%), vomiting (n = 2, 3%), odynophagia (n = 2, 3%), and abnormal imaging (1 with an abnormal esophagram, 1 with abnormal endoscopic visualization of motility).

Of 67 patients with dual fluoroscopy and HREM evaluations, 18 had discordant results between the simplified fluoroscopy interpretation (normal vs abnormal) and HREM interpretation. 64 of the 67 fluoroscopic studies had complete results, with 33 (49.3%) of these being interpreted as abnormal. 66 of the 67 HREM studies had complete assessments, 30 being abnormal (45.5%). Chicago Classification diagnoses included achalasia (n = 12, 17.9%), ineffective esophageal motility (n = 7, 10.4%), absent contractility (n = 6, 9%), EGJ obstruction (n = 3, 4.5%), and 2 were classified as other (3%). Assuming HREM is the gold standard, the sensitivity of fluoroscopy was 0.78, specificity was 0.68, positive predictive value was 0.64 and negative predictive value was 0.81.

Prior studies have compared esophagram and HREM in adults, but to our knowledge there has not been a comparable analysis in pediatric patients. Both esophagram and HREM can be technically challenging or limited studies in pediatrics due to patient cooperativity. Additionally, many of the patients who undergo esophagram with or without HREM have notable prior pathology or surgical history including tracheoesophageal fistulas or Nissen fundoplications. Further studies are needed as the adult‐based Chicago Classification has limitations for interpretation of pediatric studies, and standard reference ranges for HRM in pediatrics are not currently established. In conclusion, our data demonstrates low specificity (0.68) and positive predictive value (0.64) for esophageal motility disorders when comparing fluoroscopic findings and HREM.

## 830 FIRST FORMALIZED NEUROGASTROENTEROLOGY AND MOTILITY CURRICULUM LEADS TO INCREASED CONFIDENCE GAINS AMONG PEDIATRIC GASTROENTEROLOGY FELLOWS

*Raul Sanchez* ^*1*^, *Janice Khoo* ^*2*^, *Hilary Michel* ^*1*^, *Md‐Rejuan Haque* ^*3*^, *Peter Lu* ^*1*^, *Ashley Kroon van Diest* ^*4*^, *Neetu Bali Puri* ^*1*^, *Desale Yacob* ^*1*^, *Carlo Di Lorenzo* ^*1*^, *Karla Vaz* ^*1*^

^*1*^ *Gastroenterology*, *Nationwide Children's Hospital*, *Columbus*, *OH*; ^*2*^ *Gastroenterology*, *Children's Hospital of Orange County*, *Orange*, *CA*; ^*3*^ *Biomedical Informatics*, *The Ohio State University*, *Columbus*, *OH*; ^*4*^ *Psychology*, *Nationwide Children's Hospital*, *Columbus*, *OH*

**Background:** Neurogastroenterology and motility (NGM) disorders are commonly encountered in pediatric gastroenterology. However, no formal education exists in NGM separate from a 1‐year fellowship has been published. With six physicians in our division who specialize in motility disorders, we created a curriculum to build the foundational knowledge of NGM disorders for our pediatric gastroenterology (GI) fellows.

**Methods:** We designed a comprehensive curriculum that consisted of monthly lectures given by attending physicians and a GI psychologist. Topics were chosen after completion of a needs assessment of 9 pediatric GI fellows in 2023. Additional surveys were completed by attendings to identify key topics to include. The Entrustable Professional Activity for NGM disorders published in 2021 was also reviewed to further guide curriculum topics. Prior to and after completing the curriculum, GI fellows completed a survey ranking confidence in their knowledge on 29 NGM topics on a Likert scale ranging from 1 (not confident at all) to 5 (completely confident). Additionally, after each lecture, the fellows completed speaker evaluations to provide feedback on ways to improve the curriculum. The survey topics were grouped into 7 categories by specific area of the gastrointestinal tract or NGM topics ‐ esophageal, stomach/small bowel, colon/anorectal, disorders of gut‐brain interaction, medications, neuromodulation, and rumination. Descriptive statistics were presented as means with 95% confidence intervals. Data was analyzed via Wilcoxon signed‐rank test for each of the variables and were adjusted by the Benjamini and Hochberg method.

**Results:** All 9 GI fellows completed surveys before the first lecture on 8/21/23 and after the last lecture on 5/20/24. The fellows’ overall mean ranked confidence was 1.99 (1.20 – 2.72) before the curriculum and 2.97 (2.38 – 3.24) after completing the curriculum (p = 0.004). Additionally, for each of the 7 categories, there was significant improvement in confidence rankings (Table 1).

**Conclusion:** Our innovative and novel NGM curriculum was successful in increasing pediatric GI fellows’ confidence in their knowledge of NGM disorders. In all of our designated areas of learning, fellows demonstrated improvement in confidence scores by the end of the curriculum year.


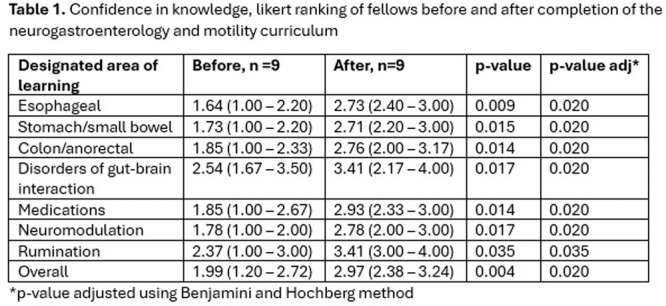


## 832 ALKALINE REFLUX IN CHILDREN: A HIDDEN CULPRIT IN ESOPHAGEAL MUCOSAL DAMAGE?

*Mariana Robles Linares Calleros*, *Erick Toro‐Monjaraz*, *Flora Zarate‐Mondragon*, *Jaime Ramirez Mayans*, *Ericka Montijo‐Barrios*, *Karen Ignorosa‐Arellano*, *Dr. José Francisco Cadena León*, *Roberto Cervantes Bustamante*, *Martha Martínez Soto*

*Instituto Nacional de Pediatria*, *Mexico City*, *CDMX*, *Mexico*

**Abstract:** Alkaline reflux is an important area of research in gastroenterology. In pediatrics, some studies have linked alkaline reflux with respiratory symptoms, while in adults it has been associated with duodenogastric reflux, particularly involving bile salts. However, to date, no studies have evaluated the impact of alkaline reflux on esophageal mucosal integrity, as assessed by endoscopy or by measuring baseline nocturnal impedance. Nocturnal baseline impedance has been proposed as a marker of esophageal mucosal integrity. The aim of this study was to evaluate the association between alkaline reflux and esophagitis, as well as its relationship with nocturnal baseline impedance.

**Methods:** We conducted an observational, descriptive, cross‐sectional, and retrospective study of pH‐impedance studies performed over a two‐year period at a tertiary care hospital in Mexico City. We included a pH‐impedance studies from patients who also underwent upper endoscopy within no more than two weeks of the impedance study. Additionally, only studies with a total recording time longer than 18 hours were considered. The pH‐impedance studies were divided into two groups: those with erosive esophagitis confirmed by endoscopy and those without endoscopic evidence of esophagitis. All pH and impedance metrics were analyzed, including the assessment of mean nocturnal baseline impedance (MNBI), following current recommendations. Statistical analysis was performed using SPSS version 28.0. Both univariate and bivariate analyses were conducted. Depending on the distribution of continuous variables, either Student's t‐test or the Mann–Whitney U test was applied. Correlation analyses were conducted between numerical variables, particularly between nocturnal baseline impedance and the number of acid, weakly acidic, and alkaline reflux episodes.

**Results:** We included 124 pH‐impedance studies of which 41 patients belong to the group with esophagitis, while 83 belong to the group without esophagitis. The mean age of the patients was 6 years, with a predominance of males. Comparison between patients with and without esophagitis showed a statistically significant association only with alkaline reflux (p < 0.024); no significant association was found with acid or weakly acidic reflux.When analyzing the relationship between nocturnal baseline impedance and the types of reflux, a weak negative correlation was observed between MNBI and alkaline reflux (r = –0.177; p = 0.05). A stronger negative correlation was found between acid reflux and MNBI (r = –0.3; p < 0.001). No statistically significant correlation was found for weakly acidic reflux (r = –0.092).Among clinical symptoms, only epigastric pain showed a significant association with alkaline reflux (p < 0.05). No significant differences were found for other symptoms such as vomiting, hiccups, cough, globus sensation, choking, recurrent pneumonia, dysphagia, chest pain, cyanosis, nausea, heartburn, or fullness.

**Conclusion:** This study suggests that alkaline reflux may play a significant role in esophageal mucosal damage in children, as evidenced by its association with erosive esophagitis and lower nocturnal baseline impedance. While acid reflux showed a stronger correlation with impaired mucosal integrity, alkaline reflux was the only type significantly associated with esophagitis and epigastric pain. These findings underscore the need for greater clinical awareness of non‐acid reflux in pediatric patients and support further investigation into its pathophysiological relevance and diagnostic evaluation.

## 834 SOCIAL FACTORS ARE ASSOCIATED WITH TREATMENT OUTCOMES IN CHILDREN WITH ABDOMINAL PAIN RELATED DISORDERS FOR GUT BRAIN INTERACTION

*Miranda van Tilburg* ^*1,2*^, *Tasha Murphy* ^*3*^, *Katherine Lamparyk* ^*4*^, *Kendra Kamp* ^*5*^, *David Huh* ^*3*^, *Margaret Heitkemper* ^*5*^, *Rob Shulman* ^*6*^, *Rona Levy* ^*3*^

^*1*^ *Gastroenterology and Hepatology*, *University of North Carolina*, *Chapel Hill*, *NC*; ^*2*^ *Cape Fear Valley School of Medicine*, *Methodist University*, *Fayetteville*, *NC*; ^*3*^ *School of Social Work*, *University of Washington*, *Seattle*, *WA*; ^*4*^ *Akron Children's Hospital*, *Akron*, *OH*; ^*5*^ *School of Nursing*, *University of Washington*, *Seattle*, *WA*; ^*6*^ *Baylor College of Medicine*, *Houston*, *TX*

**Introduction:** Psychological and dietary treatments are often recommended for children with Abdominal Pain related Disorders of Gut Brain Interaction (**AP‐DGBI**). However, these treatments require access to trained clinicians, and food options that may be difficult to access in certain areas. We aimed to examine if social and economic factors predicted outcomes of psychological and dietary treatments.

**Methods:** This was a secondary data analysis of a randomized controlled trial. 163 children with AP‐DGBI were randomized to either parent‐delivered Cognitive Behavioral Therapy (**CBT**; n=82)) or the low Fermentable Oligosaccharides, Disaccharides, Monosaccharides and Polyols Diet (**LFD**; n=81). Treatments were given for three weeks; both groups received a weekly session with a therapist or a dietitian, respectively. The LFD group also received a few samples of LFD foods such as gluten free bread and pasta. This was not enough food for the entire treatment period.

Outcome variables included parent‐reported abdominal pain intensity and frequency (2‐week diary) and health‐related quality of life (child report Pediatric Quality of Life Inventory; PedsQL) before and after treatment. Social/economic factors included child neighborhood inequities (Child Opportunity Index, a composite index based on census tract of access to education, health/environment, and social/economic opportunities), living in a food desert (based on census tract living > 1 mile from the supermarket), living in poverty (based on zip codes, income federal poverty threshold), parent education level (college graduation), and parent race (white vs non‐white).

Moderation analyses were conducted using generalized estimating equations to evaluate whether baseline patient characteristics predicted differences in pre‐ to post‐outcome change. We evaluated the prospective effect of each baseline moderator variable on (1) Differences in outcome change within each intervention, and (2) The between‐group differences in pre‐ to post‐outcome change for CBT vs. LFD. Results are presented as differences in outcome variables between those with high or low score (greater than ‐1 to +1 standard deviation for continuous variables) on moderator variables.

**Results:** The sample consisted of 60.1% girls, mean age 10.0 ± 1.8 yrs., 47.2% white/Caucasian, 32.5% Hispanic/Latino. Caregivers were primarily female (95.7%). As can be seen from Table 1, social/economic factors were not associated with differences in pre to post treatment quality of life. Poverty rate was associated with more change in pain frequency and parent employment was associated with more change in pain intensity with CBT treatment. LFD was more effective than CBT in reducing pain frequency for participants living in a food desert than for participants not living in a food desert. This was the only significant between group comparison.

**Discussion:** Some social factors may impact treatment outcomes in children with AP‐DGBI. CBT was more effective in reducing child pain if parents were employed and lived in low poverty areas. These families may have better access to healthcare and more time for treatment involvement such as homework. Children living in a food desert appear to benefit more from LFD than CBT. In this trial some LFD foods were sent to families, which may have been the most helpful to those living in food deserts were LFD foods are scarce. Further research is needed to determine why social factors can predict treatment response to different treatments.

This work was supported by R01NR016786 (Shulman/Levy).


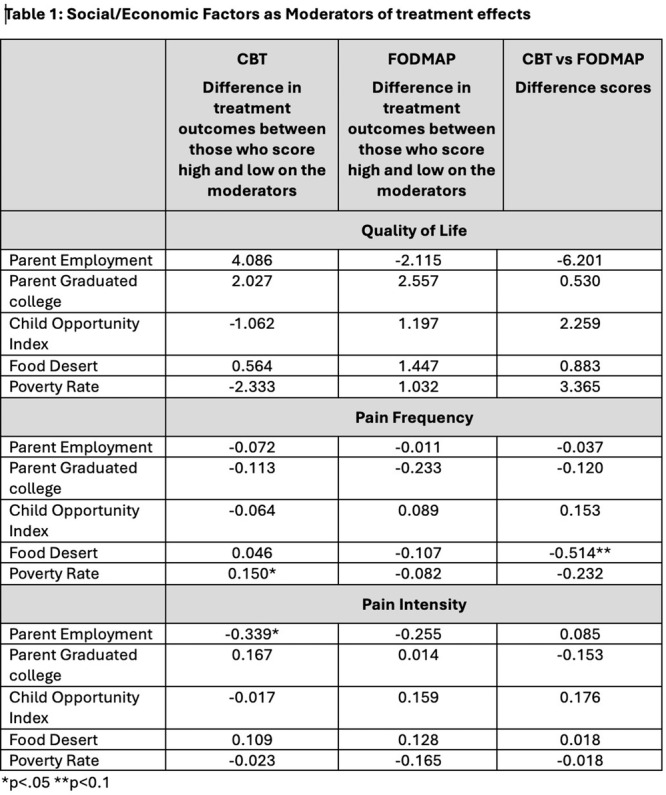


## 835 KNOWLEDGE, ATTITUDES, AND PRACTICES REGARDING INTRACTABLE CONSTIPATION AMONG A GROUP OF LATIN AMERICAN AND IBERO‐AMERICAN PEDIATRIC GASTROENTEROLOGISTS

*Carlos Velasco‐Benitez*, *Daniela Velasco‐Suarez*, *FINDERS group*

*Gastrohnup Research Group*, *Universidad del Valle*, *Cali*, *Valle del Cauca*, *Colombia*

**Introduction:** The definition of intractable constipation (IC), according to the 2014 NASPGHAN/ESPGHAN guidelines, requires a deeper understanding of key concepts that would allow for better comprehension of the condition, optimization of treatments, improved quality of life, and reduced costs. The aim of this study was to assess the knowledge, attitudes, and practices regarding IC among a group of Latin American and Ibero‐American pediatric gastroenterologists.

**Methods:** A 7‐question survey was administered, previously translated and validated into Spanish, designed to evaluate concepts that support the definition and use of a unified term for IC. The survey was distributed among LASPGHAN members. Collected data were analyzed using descriptive, univariate, and bivariate statistics, considering p<0.05 as statistically significant.

**Results:** A total of 429 pediatric gastroenterologists participated: 45.7% from LASPGHAN Latin America (Group 1), 16.3% from LASPGHAN Ibero‐America (Group 2), and 38.0% were not LASPGHAN members (Group 3). These Latin American and Ibero‐American gastroenterologists most frequently consider the time during which the child fails to have bowel movements despite receiving “optimal medical treatment” (94.6%); they believe that “optimal medical treatment” should last between 1–3 months (69.2%); they consider the use of two laxatives as a marker of “treatment failure” (51.7%); they choose tertiary care level before diagnosing IC (43.1%); they believe that follow‐up of “optimal medical treatment” should be carried out by a pediatrician or specialist (86.7%), and they prefer the term "refractory constipation" over IC (86.7%).

When comparing Group 1 vs. Group 2, Spanish gastroenterologists more often prefer the term “treatment‐resistant constipation” (OR=1.92; 95%CI=1.06–3.47; p=0.0195); and in the comparison between Group 1 and Group 3, non‐LASPGHAN gastroenterologists more frequently prefer these children to be treated at a secondary care level (OR=1.75; 95%CI=1.11–2.76; p=0.0097).

**Conclusion:** This group of Latin American and Ibero‐American pediatric gastroenterologists mainly prefers to change the term “IC” to “refractory/treatment‐resistant constipation” and to recommend that these children be treated at a secondary care level. They also support maintaining the criterion of “optimal medical treatment,” consider a treatment duration of 1–3 months, define therapeutic failure as the use of two laxatives, multiple enemas or medications, and recommend that follow‐up be conducted by a pediatrician or specialist.

## 836 PREMATURITY AND CESAREAN SECTION AS RISK FACTORS FOR INFANT REGURGITATION

*Daniela Velasco‐Suarez*, *Carlos Velasco‐Benitez*, *Carlos Jimenez*

*Gastrohnup Research Group*., *Universidad del Valle Facultad de Salud*, *Cali*, *Valle del Cauca*, *Colombia*

**Introduction:** The prevalence of infant regurgitation (IR) in Latin American infants under 12 months of age, according to Rome IV, is 6.8%. It is important to identify risk factors for IR in preterm newborns (PTNB) compared to full‐term newborns (TNB). The objective of this study was to determine the risk factors for IR in PNB compared to TNB.

**Methods:** A case‐control study was conducted with PTNB (n=105) and TNB (n=105) whose parents reported infant regurgitation through the Questionnaire for Pediatric Gastrointestinal Symptoms Rome IV. The risk factors analyzed included sociodemographic variables (sex, ethnicity, and age group); clinical variables (cesarean section); and nutritional variables (breastfeeding, bottle feeding, and complementary feeding). Statistical analysis included measures of central tendency, univariate and bivariate analysis (OR and 95%CI), with p<0.05 considered statistically significant.

**Results:** A total of 210 infants under 12 months of age (mean age 6.9 ± 3.5 months old) participated. Of these, 51.4% were male and 53.3% were of mixed race. Cesarean delivery was reported in 54.3%, and 30.0% were both preterm and born via cesarean section. The prevalence of IR was 5.7% in TNB and 14.3% in PTNB (p=0.032). Risk factors for IR in PTNB compared to TNB included age under 6 months old (OR=7.0; 95%CI=1.9–38.2; p=0.0006), Afro‐descendant ethnicity (OR=4.2; 95%CI=1.2–12.7; p=0.0031), cesarean delivery (OR=5.7; 95%CI=2.0–17.5; p=0.0001), and breastfeeding (OR=4.1; 95%CI=0.9–37.5; p=0.0472). Complementary feeding was a protective factor (OR=0.4; 95%CI=0.1–1.1; p=0.0466).

**Conclusions:** IR was more common in PTNB compared to TNB. Identified risk factors included younger age, cesarean delivery, Afro‐descendant ethnicity, and breastfeeding, while complementary feeding appeared to be a protective factor.

## 837 PREVALENCE AND FACTORS ASSOCIATED WITH FUNCTIONAL CONSTIPATION IN INFANTS, TODDLERS, SCHOOLCHILDREN AND ADOLESCENTS ACCORDING TO THE ROME IV CRITERIA

*Daniela Velasco‐Suarez*, *Claudia Araque Mora*, *Carlos Velasco‐Benitez*

*Gastrohnup Research Group*., *Universidad del Valle Facultad de Salud*, *Cali*, *Valle del Cauca*, *Colombia*

**Introduction:** According to the Rome IV Criteria, disorders of the gut‐brain interaction (DGBIs) in infants, toddlers, schoolchildren, and adolescents have a global prevalence of 22.0–23.0%, with functional constipation (FC) reported as the most common DGBI. In Latin America, possible associations of FC remain unknown. The aim of this study was to describe the prevalence of FC in Latin American children and its potential associations.

**Methods:** This was a descriptive, observational, and analytical study conducted in children aged 0–18 years from schools in Colombia (n=9415), Ecuador (n=1396), El Salvador (n=201), Cuba (n=93), Panama (n=139), Nicaragua (n=65), and Mexico (n=2571). Sociodemographic, familial, clinical, and nutritional variables were taken into account. After obtaining informed consent/assent, participants completed the Spanish version of the Questionnaire for Pediatric Gastrointestinal Symptoms Rome IV (QPGS‐IV). The analysis included measures of central tendency, univariate, bivariate, and multiple logistic regression analyses, with corresponding ORs and 95%CI. A p‐value <0.05 was considered statistically significant.

**Results:** A total of 14831 participants were included. FC was present in 15.0% of children aged 0–4 years old (n=4742) and in 12.9% of children aged 5–18 years old (n=10,088). Infants and toddlers had a higher likelihood of presenting FC if they had started complementary feeding (OR=3.48; 95%CI=1.72–2.53; p=0.012) or had a family history of DGBIs (OR=2.32; 95%CI=1.34–3.97; p=0.0009). Among schoolchildren and adolescents, FC was more likely in females (OR=1.48; 95%CI=1.34–1.63; p=0.0000) and those attending private schools (OR=1.46; 95%CI=1.28–1.67; p=0.0000). Risk factors for DGBIs included older age, female sex (OR=1.66; 95%CI=1.36–2.03; p=0.0000), country of origin (OR=3.39; 95%CI=1.46–2.84; p=0.004), and family history of DGBIs (OR=2.47; 95%CI=1.12–1.98; p=0.048).

**Conclusions:** Our prevalence of 12.9–15.0% is consistent with global studies, with FC being the most common DGBI. FC increases with age but shows better outcomes in infants and toddlers. Early identification of FC allows for timely diagnosis. Risk factors for FC include, in infants and toddlers: initiation of complementary feeding, origin from Nicaragua, and family history of DGBIs; and in schoolchildren and adolescents: female sex and attending a private school.

## 839 ASSOCIATION BETWEEN YOUTH‐ AND PARENT‐ REPORTED SYMPTOMS IN DGBI AND SCHOOL ABSENTEEISM

*Zara Zaidi* ^*1*^, *Sofia Wicker Velez* ^*1*^, *Melanie Brown* ^*2*^, *Ching‐Yuan Wang* ^*3*^, *Seoyeon Yoo* ^*3*^, *Shaija Kutty* ^*1*^, *Erika Chiappini* ^*3*^

^*1*^ *Department of Pediatric Gastroenterology Hepatology and Nutrition*, *Johns Hopkins Medicine*, *Baltimore*, *MD*; ^*2*^ *Johns Hopkins University*, *Baltimore*, *MD*; ^*3*^ *Psychiatry and Behavioral Sciences*, *Johns Hopkins University*, *Baltimore*, *MD*

**Background:** Chronic abdominal complaints are common in school‐aged children. Most affected children do not have underlying organic diseases but suffer from disorders of gut‐brain interaction (DGBI). Suffering from DGBI can have very real and negative effects on a child's quality of life and are also associated with increased rates of anxiety and depression symptoms. It has been suggested that children with DGBI are more likely to experience significant school absenteeism than children suffering from organic disease, as are youth with anxiety and depression. As a result, many children with chronic conditions turn to online or homeschool settings to better manage their symptoms. Chronic school absenteeism is associated with poor educational and health outcomes. We examined the association between youth‐ and parent‐ reported symptoms and type of school attended and number of school days missed based on this known literature.

**Method:** Participants were 67 youth with chronic abdominal pain and/or IBS who attended a consultation visit in a multidisciplinary clinic. A retrospective review of patient charts was completed to assess school type (in‐person, online, homeschool, home and hospital) and school absenteeism (i.e., number of days missed). Youth and their parents completed measures of somatic symptoms (Child Somatic Symptom Inventory; CSSI), functional impairment (Functional Disability Index; FDI), and measures of anxiety and depression symptoms (PROMIS measures). The relationship between youth‐ and parent‐rated somatic symptoms, functional disability, and anxiety and depression symptoms on school absences and school type (in‐person, home school, online, etc.) was examined using linear and logistic regressions.

**Results:** Youth predominantly identified as female (59.7%) and white (83.6%). Diagnoses included abdominal pain (64.2%), IBS (47.8%), nausea (38.8%), and/or constipation (26.9%). 59.7% had an anxiety disorder diagnosis, 22.4% were diagnosed with depression, and 26.9% diagnosed with ADHD. Related to school, 61.2% were enrolled in an in‐person school, 16.4% in online/cyberschool, 13.4% were homeschooled, and 9.0% were enrolled in home‐and‐hospital. Attending school at home (online, homeschool, or H&H), was significantly predicted by youth‐ (OR: 1.10, p=.03) and parent‐report of somatic symptoms (OR: 1.12, p=.01) and parent‐report of functional disability (OR: 1.04, p=.04); it was not significantly predicted by anxiety or depressive symptoms. Of those attending in‐person school, the mean days missed in the current school year was 20.52 days (SD=25.30 days). Number of missed school days was significantly predicted by parent‐report of the child's depressive symptoms (B=0.92, t(19)=2.42, p=.03); it was not significantly predicted by parent‐ or child‐report of anxiety, somatic symptoms, or functional disability or youth‐report of depressive symptoms. Youth report of somatic symptoms significantly predicted missing >30days of school (OR: 1.10, p=0.04), as did parent‐report of somatic symptoms (OR: 1.13, p=.03) and functional disability (OR: 1.05, p=.04).

**Conclusions:** Youth with DGBI are more likely to complete schooling at home when they experience greater somatic symptoms (parent and youth reported) and greater functional disability (parent reported). Additionally, youth with DGBI attending in‐person school miss more days of school when they report greater somatic symptoms (youth and parent report) as well as greater parent report of functional disability. Only parent‐report of depressive symptoms predicted days missed. Our study highlights the utility of patient and parent reported somatic symptoms and functional disability to better understand school choice and absences. Possible future research could include extrapolating this study to larger patient populations and other centers and/or diverse populations. Further understanding of predictors of school absenteeism in DGBI may support better intervention in this population.

## 843 COMPARATIVE ANALYSIS OF GASTROINTESTINAL OUTCOMES IN PEDIATRIC TPN PATIENTS BY AGE GROUPS

*Saman Aryal* ^*1*^, *Arleen Delgado* ^*1*^, *Luis Nieto* ^*2*^, *Ricardo Mora* ^*3*^

^*1*^ *Pediatrics*, *NYC Health + Hospitals*, *New York*, *NY*; ^*2*^ *Gastroenterology*, *Emory University*, *Atlanta*, *GA*; ^*3*^ *Neonatology*, *NYC Health + Hospitals*, *New York*, *NY*

**Introduction:** Total parenteral nutrition (TPN) is vital for children who cannot meet nutritional needs through enteral feeding due to medical conditions. While TPN supports growth, its long‐term impact on gastrointestinal health is unclear. Children receiving TPN are at risk for liver dysfunction, metabolic disorders, and other issues. Younger children may have different outcomes due to developmental and physiological factors. This study examines the age‐related risk of gastrointestinal outcomes in children receiving TPN.

**Methods:** The study did not specify a single TPN lipid formulation. Patients included in the analysis received various types of lipid emulsions, including those based on soybean oil, fish oil (omega‐3 enriched), and mixed lipid emulsions combining plant and animal sources. These formulations were used according to individual patient needs and institutional protocols. However, detailed stratification based on lipid type was beyond the scope of this analysis. Patients included in the study were receiving TPN as part of their treatment for chronic medical conditions requiring long‐term nutritional support.These conditions primarily included short bowel syndrome, congenital or acquired gastrointestinal abnormalities, severe malabsorption syndromes, and prolonged ileus where enteral nutrition was insufficient or impossible. The duration of TPN use ranged from several weeks to multiple years, depending on the underlying condition and recovery status. Specific time frames of TPN exposure were not detailed in this analysis, but the population represented is predominantly reliant on extended TPN therapy.

We performed a retrospective cohort study utilizing extensive population‐based data from the TriNetX platform. Patients between the ages of 1 to 10 years who received total parenteral nutrition (TPN) between January 2010 and December 2024 were identified and categorized into two cohorts: those aged 1–5 years and those aged 6–10 years. Data from 45,750 patients in the 1–5 years cohort and 87,278 patients in the 6–10 years cohort were analyzed. Outcomes included gastrointestinal conditions such as abnormal liver function tests (LFTs), metabolic syndrome, inflammatory bowel disease (IBD), jaundice, intestinal dysbiosis, vomiting, gastroesophageal reflux disease (GERD), and irritable bowel syndrome (IBS). Propensity score matching was performed to balance baseline characteristics between the two cohorts, resulting in matched groups of 41,922 patients each. Logistic regression was used to estimate odds ratios (ORs) with 95% confidence intervals (CIs). A p‐value less than 0.05 was considered statistically significant.

**Results:** A total of 133,028 patients were included. After matching, each group had 41,922 patients. The 1–5 years cohort had lower odds of abnormal LFTs (OR 0.498, 95% CI, 0.417–0.595, p < 0.001) and metabolic syndrome (OR 0.238, 95% CI, 0.119–0.474, p < 0.001). IBD was also less common (OR 0.390, 95% CI, 0.274–0.555, p < 0.001). Jaundice was less frequent (OR 0.615, 95% CI, 0.498–0.761, p < 0.001), and the younger cohort had reduced intestinal dysbiosis (OR 0.858, 95% CI, 0.767–0.960, p = 0.007), vomiting (OR 0.602, 95% CI, 0.576–0.630, p < 0.001), GERD (OR 0.714, 95% CI, 0.671–0.759, p < 0.001), and IBS (OR 0.354, 95% CI, 0.220–0.569, p < 0.001).

**Discussion:** Younger children showed a lower risk for TPN‐related gastrointestinal conditions, likely due to metabolic and developmental differences. Their adaptable gastrointestinal tracts may reduce oxidative stress and inflammation, limiting complications like abnormal LFTs and metabolic disorders. In contrast, older children face cumulative TPN damage, leading to higher risks of GERD, IBS, and gut health issues. Protective factors such as hepatic regeneration and immune adaptation might explain the lower incidence of jaundice and vomiting in younger children. These findings highlight the need for age‐specific monitoring and interventions to reduce risks, especially for older patients. Limitations include the study's retrospective design and potential data biases. Future prospective studies are needed for validation and to explore underlying mechanisms.

**References:**

1. Smith J, Brown L, et al. Long‐term outcomes of pediatric patients receiving total parenteral nutrition: A review. J Pediatr Gastroenterol Nutr. 2020;70(4):e45‐e52.

2. Jones K, Patel R, et al. Age‐related risks associated with total parenteral nutrition in children. Pediatr Res. 2018;84(2):203‐209.

3. Lee M, Park S, et al. The impact of early nutritional support on gastrointestinal health in pediatric patients. Nutr Clin Pract. 2017;32(1):48‐55.

4. Miller A, Thompson B, et al. Gastrointestinal complications in children on total parenteral nutrition. Arch Dis Child. 2019;104(3):263‐268.

5. Davis L, White C, et al. Metabolic and gastrointestinal outcomes in pediatric TPN: A cohort analysis. Clin Nutr. 2021;40(5):1423‐1429.

## 844 CORRELATION BETWEEN NECK CIRCUMFERENCE AND CENTRAL ADIPOSITY WITH METABOLIC ALTERATIONS IN CHILDREN WITH OBESITY

*Ana Gabriela Ayala‐Germán*, *Carlos Medina‐Campos*

*Pediatrics*, *UMAE Hospital De Especialidades*, *Mérida*, *Yucatán*, *Mexico*

**Background:** Childhood overweight and/or obesity is a major global public health issue. In children over 2 years of age, the anthropometric diagnosis is established using body mass index (BMI), with thresholds at the ≥85th and ≥95th percentiles according to WHO/CDC growth charts for overweight and obesity, respectively. It is estimated that by the end of 2025, approximately 206 million children aged 5–19 years will be living with obesity. In Mexico, the prevalence reached 34.2% as reported in 2023. Upper‐body adiposity has been associated with an increased risk of metabolic disorders. Neck circumference (NC) has emerged as an alternative method to estimate adiposity, offering advantages such as minimal circadian and postprandial variation, which positions it as a potential indirect marker of central adiposity and related metabolic disturbances, including elevated alanine aminotransferase (ALT) levels and vitamin D deficiency. Objective To determine the correlation between NC and abdominal circumference (AC), waist‐to‐height ratio (WHtR), as well as serum levels of ALT and vitamin D in children with overweight or obesity. Materials and methods This was a correlational (observational, comparative, cross‐sectional, and prospective) study. A sample of 22 pediatric patients with a diagnosis of overweight or obesity was included. Anthropometric measurements of NC, AC, and WHtR were recorded, and serum levels of ALT and vitamin D were collected. Pearson or Spearman correlation coefficients were calculated depending on the distribution of the variables. Results: Of the 22 participants, 41% (n=9) were male and 59% (n=13) were female. Regarding nutritional status, 4.5% (n=1) were overweight, and 54.5% (n=12), 18.2% (n=4), and 22.7% (n=5) had obesity class I, II, and III, respectively. There were no statistically significant differences by sex (p=0.422). Mean anthropometric values were as follows: NC: 36 cm (±5.3), AC: 99.5 cm (±13.1) and WHtR: 0.65 (±0.07) Median serum values were ALT: 76.5 U/L and vitamin D: 22.5 ng/mL. A strong positive correlation was found between NC and AC (Pearson 's R: 0.60; p<0.01). A moderate negative correlation was found between WHtR and ALT levels (Spearman's R: ‐0.46; p=0.03), and a strong negative correlation in females (Spearman's R: ‐0.706; p<0.01). No significant correlations were observed between NC and WHtR, ALT, or vitamin D levels. Conclusion: Neck circumference is positively correlated with abdominal circumference, supporting its use as a marker of adiposity. However, no correlations were found between NC and WHtR, ALT, or vitamin D levels. Interestingly, WHtR was inversely associated with ALT levels, contrary to findings reported in the literature. Further studies including non‐obese pediatric populations are recommended to better understand the directionality of this latter correlation.

## 846 IMPACT OF SUPERVISED EXERCISE AND NUTRITION AT A PEDIATRIC OBESITY CENTER

*Rosen Ling* ^*1*^, *Anupama Chawla* ^*5*^, *Amy Canterella* ^*2*^, *Brianna Burghard* ^*5*^, *Rachel Valenti* ^*5*^, *Peter Morelli* ^*3*^, *Sharon Martino* ^*4*^, *Ada Lee* ^*5*^

^*1*^ *Pediatrics*, *Stony Brook University Hospital*, *Stony Brook*, *NY*; ^*2*^ *Adolescent Medicine*, *Stony Brook University Hospital*, *Stony Brook*, *NY*; ^*3*^ *Pediatric Cardiology*, *Stony Brook University Hospital*, *Stony Brook*, *NY*; ^*4*^ *Physical Therapy*, *Stony Brook University*, *Stony Brook*, *NY*; ^*5*^ *Pediatric Gastroenterology*, *Stony Brook University Hospital*, *Stony Brook*, *NY*

**Objectives:** A comprehensive multidisciplinary intervention to increase opportunity for exercise and nutrition is the foundation of Stony Brook's Healthy Weight and Wellness Center (HWWC) and Fit Kid's For Life program (FKFL). FKFL offers supervised exercise and hands‐on nutrition. The purpose of this study is to perform a novel investigation in a tertiary pediatric obesity center to compare the effects of nutrition and exercise counseling versus counseling with the addition of supervised exercise and hands‐on nutrition.

**Methods:** This retrospective study, conducted at a single pediatric obesity center, analyzed patients enrolled in the HWWC program with or without FKFL between January 2014 and December 2020. Inclusion criteria were patients aged 7‐17, BMI ≥ 85th percentile for age and sex, and with at least 6 months of follow‐up. Exclusion criteria were no follow‐up within 6 months. The study included 40 HWWC patients, 20 with FKFL and 20 without, cohorts matched by age and sex. Pre‐ and post‐treatment data on lab work (ALT, lipid panel, HbA1c, insulin, fasting glucose), blood pressure, heart rate, and anthropometrics were collected and analyzed using SPSS with t‐tests.

**Results:** After participation in a 10‐week session of FKFL and HWWC, systolic blood pressure significantly decreased (p = 0.035) compared to HWWC alone. When our patients were subdivided into two groups, children ages 7‐12 and adolescents ages 13‐17, a statistically significant (p‐value =0.037) decrease in systolic blood pressure was noted in children ages 7‐12 but not in adolescents ages 13‐17. No significant differences were found in diastolic blood pressure, heart rate, weight, BMI, ALT, or lipid panel. Body fat percentage and HbA1c decreased on average, but not significantly, in FKFL with HWWC participants.

**Conclusion:** Our study found that pediatric patients who participated in FKFL and HWWC had a statistically significant decrease of systolic blood pressure, and an average decrease in body fat percentage and HbA1C that was not statistically significant. This suggests supervised exercise and hands‐on nutrition may be more effective than standard counseling in reducing systolic blood pressure in pediatric patients with obesity. Larger‐scale, longer‐term studies are warranted to further evaluate the clinical outcomes for patients receiving interactive interventions with traditional counseling.


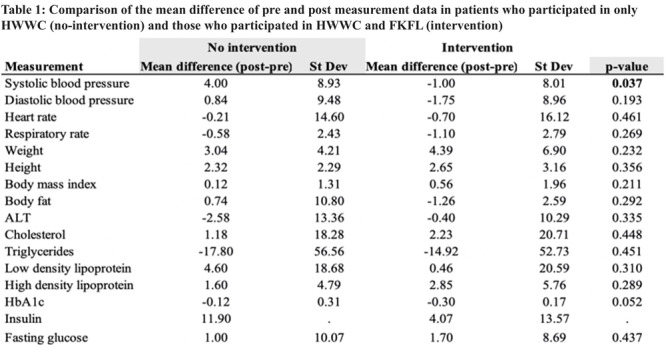


## 847 ASSESSING ADDED SUGAR INTAKE AND SOURCES OF ADDED SUGAR USING A VALIDATED FOOD FREQUENCY QUESTIONNAIRE IN A GENERAL AND SPECIALTY PEDIATRIC CLINIC

*John DeCaro* ^*1,3*^, *Amer Al‐Nimr* ^*2*^

^*1*^ *Pediatric Gastroenterology*, *University of Michigan*, *Ann Arbor*, *MI*; ^*2*^ *Pediatrics Gastroenterology*, *Dartmouth Health*, *Lebanon*, *NH*; ^*3*^ *Pediatrics*, *Dartmouth Health*, *Lebanon*, *NH*

**Background:** Obesity is an increasingly prevalent disease that, as of 2021, affected nearly twenty percent of children under the age of 18 years. Obesity places these patients at risk for many diseases including cardiovascular disease, steatotic liver disease, and decreased quality of life. There are limited proven dietary interventions to decrease BMI; however, some studies suggest that decreasing added‐sugar intake decreases BMI. According to recent National Health and Nutrition Examination Survey (NHANES) data, the average added‐sugar intake in the United States greatly exceeds the recommended daily intake.

**Objective:** This study was conducted to compare the added‐sugar intake of a sample of the pediatric population in the general and specialty clinics at Dartmouth Health Children's (DHC) to the US average according to NHANES, and to determine which sources of added‐sugar were most commonly consumed so that informed dietary interventions can be subsequently performed.

**Methods:** To measure added‐sugar intake, this study used the Block Kids Food Screener (BKFS), a food‐frequency questionnaire (FFQ) that was validated against a 24‐hour diet recall. To our knowledge the BKFS is the only validated FFQ intended for use in a pediatric population in the United States. The BKFS was converted to an electronic format, using the software RedCap, and given to children ages 2‐17 years in the DHC clinics, excluding those with dietary restrictions due to medical problems. The BKFS results were converted into quantitative estimates of added‐sugar intake per day, using NutritionQuest software, who created and validated the BKFS. Clinic patients were asked, in person, to participate in this study as they were leaving the clinic. Patients were recruited and enrolled after their actual clinic visits. Those patients were consented, given an electronic tablet hosting the survey, and asked to take the survey in the waiting room of the clinic. This study was approved by Dartmouth Health's Institutional Review Board. Data collection occurred between 3/5/24 and 5/4/24.

**Results:** Surveys were completed by 33 respondents. 19 of 33 respondents were male (57.6%). The median age of the sample was 7 years (range of 2 years to 17 years). Mean added‐sugar intake per day in that sample was 17.245 grams, with a standard deviation of 8.681 grams. Male subjects took in a statistically significant greater amount of added sugar than female patients (19.8 g vs 13.7 g, p= 0.043). Modal sources of added‐sugars were: 1. Granola bars, protein bars, breakfast bars, etc. 2. Ice cream 3a. Applesauce and fruit cocktail, and 3b. Candy and candy bars.

**Conclusions:** The sample population in this survey reportedly took in markedly less added‐sugar than in the NHANES sample from 2017‐2020 (17.245 g vs 75.642 g). Male subjects took in more sugar than female subjects, which we expect is due to differences in diet patterns. The main sources of added‐sugar in the sample population included bars, ice cream, candy and fruit sauce/cocktail. The study was limited by low sample size, recall bias, response bias, participation bias, and by possible inherent underestimation by BKFS. We considered repeating the study using the ASA 24 food recall survey. This information could be used to give targetting nutrition counselling based off of local dietary practices and gender/age‐based patterns.

## 848 MICRONUTRIENT IMBALANCES AND BODY COMPOSITION IN PEDIATRIC CYSTIC FIBROSIS IN THE ERA OF CFTR MODULATOR THERAPY

*Ala Elayyan* ^*1*^, *Christine McDonald* ^*1*^, *Jennifer Duong* ^*1*^, *Ayca Erkin‐Cakmak* ^*2*^

^*1*^ *pediatric Gastroenterology*, *University of California San Francisco*, *San Francisco*, *CA*; ^*2*^ *pediatric endocrinology*, *University of California San Francisco*, *San Francisco*, *CA*

Cystic fibrosis (CF) is a multisystem genetic disorder caused by mutations in the *Cystic Fibrosis Transmembrane Conductance Regulator (CFTR)* gene, leading to chronic pulmonary infections, pancreatic insufficiency, and complex nutritional and metabolic challenges. Historically, CF patients have experienced persistent malnutrition and fat‐soluble vitamin deficiencies due to malabsorption, systemic inflammation, and elevated energy demands. The introduction of CFTR modulator therapy has significantly improved nutrient absorption, weight gain, and overall nutritional status [1]. However, emerging data suggest that these improvements are often accompanied by disproportionate gains in fat mass and increasing reports of hypervitaminosis, particularly of vitamins A and D [2]. These changes raise concerns about evolving micronutrient imbalances and potential long‐term metabolic complications in the modulator era. This study aims to characterize the shifting nutritional phenotype of pediatric CF patients receiving CFTR modulators by evaluating the relationships between serum micronutrient levels, body composition metrics, and the duration of CFTR modulator therapy. The overarching goal is to identify key risk factors that are associated with metabolic complications in pediatric CF patients undergoing modulator therapy. The study aims to inform personalized, data‐driven strategies such as adjusted vitamin supplementation protocols, targeted dietary counseling, and early metabolic screening to optimize long‐term nutritional management.

b. Study Design

The study leverages integrated clinical records, including dual‐energy X‐ray absorptiometry (DXA)‐derived body composition metrics (FMI and FFMI), serum micronutrient levels (e.g., vitamins A, D, E, zinc, selenium), BMI trajectories, and detailed CFTR modulator therapy data. Pediatric CF patients will be stratified by BMI category (<85th vs. ≥85th percentile) and pancreatic status (PI vs. PS). Multivariate regression models, adjusted for age, sex, and pancreatic insufficiency, will be used to examine associations between body composition and micronutrient status, and to identify predictors of metabolic risk profiles.

c. Patient Selection Criteria

The study includes pediatric CF patients aged 8–19 years who had at least one DXA scan and a corresponding serum micronutrient panel. Eligible patients were required to have complete clinical data, including records of CFTR modulator therapy use, dietary intake assessments, BMI data, and enzyme supplementation. Patients were stratified based on CFTR modulator therapy status (on vs. not on modulators) and age and sex adjusted BMI percentile (<85th vs. ≥85th).

d. Primary and Secondary Endpoints

Primary endpoints include the correlation between serum levels of vitamins A, D, E, zinc, selenium, and magnesium and DXA‐derived measures of fat mass index (FMI) and fat‐free mass index (FFMI). The prevalence of micronutrient imbalances, particularly hypervitaminosis, is also evaluated.

Secondary endpoints assess how CFTR modulator therapy duration, adherence, and BMI trajectory affect serum micronutrient levels. The accuracy of predictive models using serum biomarkers and clinical variables to estimate FMI and FFMI is also evaluated, with validation against DXA results.

e. Interim Results

Preliminary analysis of 45 pediatric CF patients shows a strong correlation between BMI and body fat percentage (r = 0.81, p < 0.001), with 31% classified as overweight/obese (BMI % ≥85th) and 46.7% of patients with normal BMI (<85th percentile) exhibiting elevated fat mass. Patients with BMI ≥85th percentile had significantly higher FMI (12.2 vs. 5.4 kg/m2, p < 0.001), while lean mass was not significantly different between groups These findings will inform the need for more nuanced, composition‐based assessments beyond BMI to guide nutritional management in CF. Data analysis is currently underway, and results are expected by July‐ August/2025], which will help refine clinical strategies based on body composition and micronutrient profiles.

**Reference:**

1. Academy of Nutrition and Dietetics: 2020 Cystic Fibrosis Evidence Analysis Center Evidence‐Based Nutrition Practice Guideline. McDonald CM, Alvarez JA, Bailey J, et al. Journal of the Academy of Nutrition and Dietetics. 2021;121(8):1591‐1636.e3. doi:10.1016/j.jand.2020.03.015.

2. The Impact of Elexacaftor/¬Tezacaftor/¬Ivacaftor on Fat‐Soluble Vitamin Levels in People With Cystic Fibrosis. Hergenroeder GE, Faino A, Bridges G, et al. Journal of Cystic Fibrosis : Official Journal of the European Cystic Fibrosis Society. 2023;22(6):1048‐1053. doi:10.1016/j.jcf.2023.08.002.

## 849 NAVIGATING AVOIDANT RESTRICTIVE FOOD INTAKE DISORER (ARFID): CAREGIVER EDUCATION AND FEEDBACK

*Olivia Eldredge*, *Lisa Mancini*, *Annette Schille*, *Sarah Salazar*, *Elana Bern*

*Pediatrics*, *Harvard Medical School*, *Boston*, *MA*

Avoidant/Restrictive Food Intake Disorder (ARFID) is a feeding and eating disorder defined by the Diagnostic and Statistical Manual of Mental Disorders (5^th^ edition) in 2013, characterized by a persistent failure to meet adequate nutritional and/or energy intake with significant impact on physical, psychological, and/or social health. Subtypes include limited interest in food and/or eating, avoidance of specific sensory characteristics, and/or fear of aversive consequences. Family functioning plays a role in development and progression of eating disorders; Furthermore, family support, including patient family centered care (PFCC), can play a key role in therapeutic success through empowerment of families to understand and support their child with ARFID, in collaboration with their care team^1,2^. The current pilot study aimed to evaluate the feasibility and preliminary efficacy of a novel ARFID‐Family Education (AFE) class that was delivered in a one‐time virtual synchronous participatory format. The goal was to provide comprehensive education to families, increase awareness regarding medical and nutritional concerns associated with ARFID, as well as foster a sense of confidence in caregivers to implement strategies that will help families manage ARFID in their day‐to‐day lives.

**Methods:** A panel consisting of ARFID specialized physicians, dietitians, behavioral medicine providers, and administrators, identified topics of interest to families of children diagnosed with ARFID. Patients were identified through our ARFID Program triage process or via internal provider referral. All patients were either seen by a Boston Children's Hospital (BCH) gastroenterologist within the last 3 years or by the BCH ARFID team for consultation. Families attended one of 19 scheduled AFE classes held via zoom telemedicine (February 29^th^, 2024, to May 1^st^, 2025). Participants (n=120) included families of identified patients (5 to19 years old), with an average of 6 families in each class. The class was directed towards caregivers, but patients could observe, if they were interested. The 90‐minute class consisted of two segments: 1) Three didactic sections each presented by a specialized gastroenterologist, dietitian, and social worker, respectively. 2) Live question and answer (Q and A) dialogue amongst speakers and caregivers. Participants were educated about the proposed pathogenesis of ARFID, potential medical and nutritional consequences of ARFID, and general nutritional and behavioral recommendations for family supported care. Caregivers were asked to complete a pre‐evaluation survey (Pre‐S) prior to starting the class and post‐evaluation survey (Post‐S), once the Q and A completed. Survey responses were entered and stored into a REDCap electronic data capture tool provided through the BCH External Platform.

**Results:** Pre‐S and Post‐S response rate was 67.5% and 77.5%, respectively; 81.7% respondents shared they “definitely would” recommend the class to other families or patients with ARFID. 76% respondents preferred the interactive component of this presentation over a pre‐recorded video option. Among Post‐S respondants, 98.9% "agreed or strongly agreed" to having a good understanding of what ARFID is, compared to 71% Pre‐S, respondants. 86% Post‐S respondants "agreed or strongly agreed" to have an increased understanding of how they can help manage their child's ARFID compared to 29% Pre‐S respondants. Our results suggest an increase in the understanding and confidence of caregivers on how to manage their child's ARFID, following engagement in the class. Meanwhile, positive agreement statements regarding understanding how ARFID impacts their child's diet and nutritional state, exhibited limited change (80% Pre‐S to 81% Post‐S) suggesting a higher understanding of nutritional impact of ARFID prior to the class.

**Conclusion:** We present a family centered, single‐session, and ARFID specific education program delivered by a trained multidisciplinary team. Our live interactive virtual class for caregivers provides an engaging opportunity to educate and support families of children and adolescents with ARFID. Pre and post survey responses from caregivers were compared, and our data suggests that interactive family education and group participation can play a meaningful role in supporting families with ARFID. Our study begins to provide a framework for developing targeted education‐based interventions aimed at improving health outcomes in children with feeding and eating disorders. Further studies are needed to evaluate feeding behavior modifications‐initiated post group and clinical follow‐up.

**References:**

Erriu, M., et al (2020). The Role of Family Relationships in Eating Disorders in Adolescents: A Narrative Review. *Behavioral sciences,10*, 71.

Shimshoni, Y., & Lebowitz, E. R. (2020). Childhood Avoidant/Restrictive Food Intake Disorder: Review of Treatments and a Novel Parent‐Based Approach. *Journal of Cognitive Psychotherapy*, *34*, 200.

## 851 FITTING THE FUTURE: GASTROJEJUNOSTOMY TUBE LENGTHS FOR PEDIATIRC PATIENTS

*Molissa Hager* ^*1*^, *Erin Alexander* ^*1*^, *Drayna Megan* ^*2*^, *Schmidt Cameron* ^*2*^, *Olson Rachel* ^*3*^, *Dana Steien* ^*1*^

^*1*^ *Pediatric Gastroenterology*, *Mayo Clinic Minnesota*, *Rochester*, *MN*; ^*2*^ *Mayo Clinic School of Health Sciences*, *Rochester*, *MN*; ^*3*^ *University of Minnesota Rochester*, *Rochester*, *MN*

**Background:** Gastrojejunostomy tubes (GJ) provide nutrition to children who are intolerant of gastric feedings. When a new GJ is placed or replaced, the size of the GJ tube must be decided. This includes the jejunal limb (J‐limb) length. Available J‐limb lengths include: 10 cm, 15 cm, 22 cm, 30 cm, and 45 cm. Although GJ tubes are commonly used in pediatrics, there are no guidelines for choosing the J‐limb length. It has been hypothesized that J‐limbs lengths that are too short or too long may increase the risk of complications, such as coiling, clogging, breakage, malfunction, or dislodgement. In addition, most GJ placements in children require sedation and radiation, creating additional risks and increasing healthcare utilization and costs.

**Methods:** This was a retrospective study completed at Mayo Clinic Children's in children ages 0‐19 years old who underwent GJ placement or replacement from 2018 to 2024. Demographic data, anthropometric data, J‐limb length and reason for GJ placement or replacement were recorded. Linear regression models were performed to determine the relationship between J‐limb length and patients’ heights and weights. T‐tests were performed for each J‐limb length to evaluate the occurrence of complications based on patients’ heights and weights. T‐tests were also performed to compare anthropometrics of individuals who experienced GJ complications versus those who did not, for each J‐limb length.

**Results:** 96 patients, ages 6 months to 19 years of age had 367 GJ tubes placed. GJ tubes were placed by 3 divisions: interventional radiology, pediatric surgery, and pediatric gastroenterology. 62 (17%) were initial GJ placements and 305 (83%) were replacements. Of the 305 replacements, 193 (63%) were scheduled while 109 (36%) were done related to complications. It was found that children's heights and weights correlated to the chosen J‐limb length (R2=0.9586, R2==0.9983). The 30 cm J‐limb length was the most commonly used (158, 43%). Children who experienced complications with the 30 cm J‐limb had lower heights, compared to those who did not have complications (p=0.096).

**Conclusion:** J‐limb lengths impact GJ complications rates in pediatric patients. Children's heights and/or weights should likely be used to help determine the J‐limb length, when GJ tubes are needed. Creating standard guidelines for choosing the J‐limb length, for each individual child, would streamline care practices. Undoubtedly, this would improve efficiency and consistency among medical providers. It would also likely decrease GJ tube complication rates, which would improve patient satisfaction, reduce healthcare costs, and utilization.

## 852 ANEMIA IN OVERWEIGHT AND OBESE CHILDREN IN A URBAN CLINIC

*Emily Kahoud*, *Robert Abbott*, *Iona Monteiro*, *Hanan Tanuos*

*Pediatrics*, *Rutgers New Jersey Medical School*, *Newark*, *NJ*

**Introduction:** According to the WHO, worldwide adult obesity doubled since 1990, and adolescent obesity quadrupled. Despite the overabundance of calories in the diets of children/adolescents who are overweight, there may be a deficiency of essential vitamins and minerals due in part to intake of calorie dense, but nutrient‐poor foods. Numerous studies report iron deficiency among overweight/obese children and adolescents. A 2024 systematic literature review suggests that the chronic inflammatory state associated with obesity leads to production of IL‐6, which upregulates the synthesis of hepcidin and ultimately leads to iron deficiency. Obese children and adolescents who are iron deficient may respond poorly to iron supplementation, yet this same population responds to therapeutic intervention programs focusing on weight loss, lending further support to the role of chronic inflammation. Other proposed factors that relate obesity to iron deficiency and resistance to iron supplementation include genetic influences, elevated blood volume secondary to increased adipose tissue mass leading to enhanced iron requirements, physical inactivity that putatively results in decreased myoglobin breakdown with less iron being released into the serum, and metabolic effects.

**Objective:** The objective was to evaluate the presence of anemia in our pediatric population of overweight and obese children.

**Methods:** Patient records (n=500) were selected from a clinic database, from 2020 – 2024, with a listed ICD code for BMI in overweight or obese range (Z68.53 and Z68.54, respectively). Nine were excluded due to insufficient laboratory data. The height, weight, age, and gender of the patients were used to verify BMI and determine whether overweight or obese based on the percentile. Hemoglobin (Hb) levels were used to determine presence of anemia based on the reference ranges per 2025 UpToDate guidelines: 11.2 ‐ 14.5 for 6‐12 years of age (y), 11.4 ‐ 14.7 for females 12‐18 y, and 12.4 ‐16.4 for males 12‐18 y.

**Results:** 491 patient records were analyzed (male 53.4 %, female 46.6%), with 460 out of 491 (93.7%) patients between the ages of 12‐18 y; of the remaining 31 patients, 24 were between ages of 9 and 11, and 7 were between the ages of 6 and 8 y. Of these 491 patients, 205 were stratified into the overweight group and 286 into the obese group. Based on criteria listed above, 41/205 were found to be anemic in the overweight group and 38/286 in the obese group, representing 20% and 13.3%, respectively. The groups had average Hb levels of 12.8 for the overweight group and 13.1 for the obese group. 1/205 (0.5%) of the overweight patients and 7/286 (2.4%) of the obese patients were found to have Hb levels above the normal reference range. The results were analyzed using a two‐tailed z‐score to test for statistical significance of the higher level of anemia seen in the patients in the overweight group as compared to the obese group. The z‐score for this test was 1.997, corresponding to a p‐value of .0455, a statistically significant finding at p<0.05.

**Discussion:** Iron deficiency is a nutritional disorder of particular concern in our developing pediatric population due to its detrimental cognitive impact. Although the literature demonstrates an association between obesity and iron deficiency in children, there are many mechanisms that play a role, though the etiology has not yet been elucidated. Studies have demonstrated a reduced response to oral iron supplementation among an obese pediatric population with a role for hepcidin as a putative intermediary. We found that our overweight population was more predisposed to anemia than our obese population, in contrast to previously reported association of obesity and iron deficiency. Our inner‐city pediatric practice consists predominantly of African American and Hispanic patients, thus there is a lack of ethnic diversity. Despite economic limitations facing our population as well as ethnic factors, there may be dietary predispositions that have impacted our results. We also tend to advocate for vitamin supplementation at our pediatric practice, typically a multivitamin with fluoride, which may also explain a decreased prevalence of anemia overall, although neither dietary predilections nor supplementation explain the paradoxical finding of less anemia in our obese patients. Ascorbic acid directly enhances iron absorption, we speculate that multivitamin use may also contribute to iron absorption indirectly through microbiome modification and hence decrease anemia in our patients.

**Conclusion:** Anemia in our predominantly African American and Hispanic patients was higher in the overweight compared to the obese patients. Further research using a more diverse population may give a better understanding of our findings. Investigating the possible role of multivitamin and/or probiotic supplementation on the hepcidin level may prove valuable.

## 853 NEWBORN IM VITAMIN K REFUSAL RATES AND PARENTAL REASONING IN AN ACADEMIC WELL BABY NURSERY

*Anmol Kundlas* ^*1,2*^, *George Ye* ^*1,2*^, *Aimee LaRivere* ^*2,1*^, *Dhristi Raval* ^*3*^, *Anna Kerkush* ^*3*^, *Kosha Ravani* ^*3*^, *Melissa Weidner* ^*2,1*^

^*1*^ *Pediatrics*, *Rutgers University New Brunswick*, *New Brunswick*, *NJ*; ^*2*^ *Pediatrics*, *Robert Wood Johnson University Hospital*, *New Brunswick*, *NJ*; ^*3*^ *Pharmacy*, *Rutgers Ernest Mario School of Pharmacy*, *Piscataway*, *NJ*

**Background:** Since 1961, the American Academy of Pediatrics (AAP) has recommended a single intramuscular dose of Vitamin K be administered to all infants within 6 hours of birth to prevent vitamin K deficiency bleeding (VKDB), previously known as hemorrhagic disease of the newborn (Hand and Noble 2022). Vitamin K is essential for the hepatic synthesis of several vitamin K–dependent coagulation factors, including factors II (prothrombin), VII, IX, and X, as well as proteins C and S. Neonates are particularly susceptible to vitamin K deficiency due to several factors: limited placental transfer of vitamin K, low concentrations of vitamin K in breast milk, an immature neonatal liver with reduced capacity for prothrombin synthesis, and a sterile gut lacking the bacteria that synthesize vitamin K (Johnson 2024). However, despite its proven safety profile, there has been a marked increase in parental refusal nationwide, ranging from 0‐3.2% in large academic centers (Loyal et al 2018). This presents multiple challenges to public health including increased risk of early, classic, and late VKDB and subsequent morbidity and mortality (Marcewicz et. al., 2017).

**Methods:** We performed a retrospective chart review for all babies born between February 15, 2023, and February 15, 2024, discharged from the well‐baby nursery at Robert Wood Johnson University Hospital in New Brunswick, NJ. We then gathered information on the rate of refusal and reasons given by parents for this decision. Lastly, we performed a subgroup analysis for each of the newborn nursery attendings, recording differences in approach by physicians when discussing and educating hesitant parents on vitamin K, to be used for a future quality improvement initiative.

**Results:** 18 out of 2038 (0.88%) newborns who met inclusion criteria, did not receive IM vitamin K due to parental refusal. There was an increase in the rate of vitamin K refusal between the first and second six‐month intervals from 0.67 to 1.10%, but it was not statistically significant (p= 0.305, Chi‐squared). Per chart review, the five most common reasons provided by families were: unspecified (8/18), refusing all routine newborn medications, vaccinations or prophylaxis (5/18), wanting to wait until pediatrician appointment to discuss (2/18), wanting to give oral rather than IM dose (2/18), and unaware vitamin K is not a vaccine (1/18). A subgroup analysis performed for each of the newborn nursery attending physicians did not identify statistically significant differences in refusal rate for each, however key differences in documentation of the patient encounter as well as counseling process with parents were identified.

**Conclusions:** Data from a 1‐year period at our institution showed rates of VKDB prophylaxis refusal consistent with those of past nationwide studies and meta‐analyses (Isennock 2023). However, inconsistencies in education and documentation for parents who are initially hesitant have been identified. The information in this study will form the basis for a future quality improvement study aimed at reducing vitamin K refusal rates and providing parents with evidence‐based, internally consistent counseling.


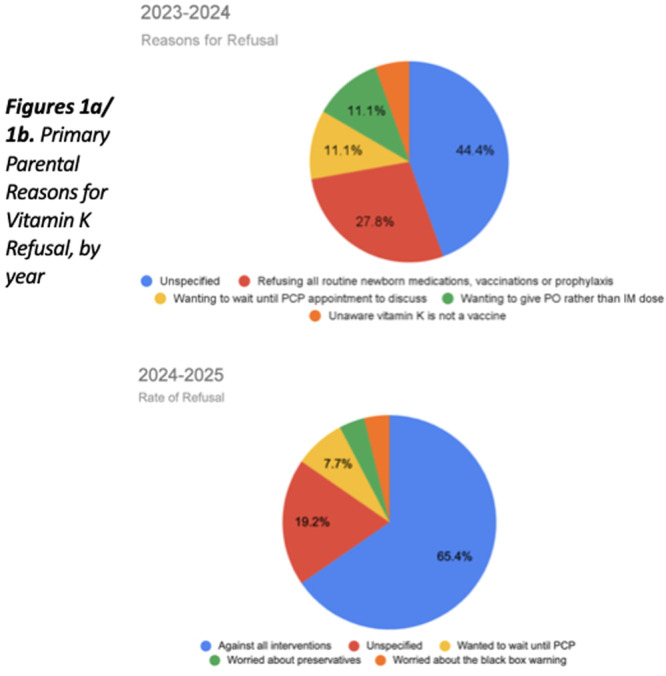



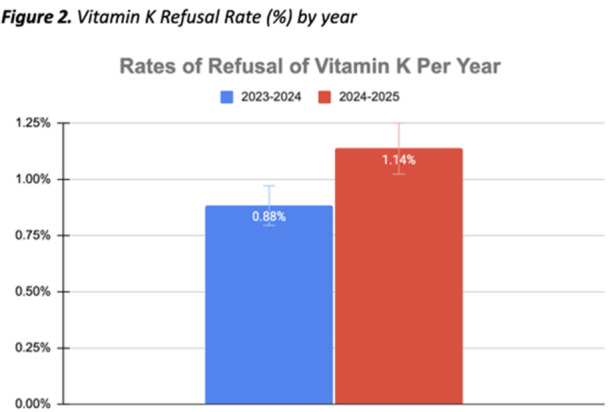


## 854  A TWO‐YEAR FOLLOW‐UP ON A NATURAL EXPERIMENT COMPARING ADOLESCENT METABOLIC AND BARIATRIC SURGERY TEACHING SESSION OUTCOMES

*Tierra Mosher* ^*1*^, *Justine Chinn* ^*2*^, *Marwa Abu El Haija* ^*1*^, *Janey Pratt* ^*2*^

^*1*^ *Pediatric Gastroenterology*, *Stanford University*, *Stanford*, *CA*; ^*2*^ *General Surgery*, *Stanford University*, *Stanford*, *CA*

**Introduction:** Adolescents referred for Metabolic and Bariatric Surgery (MBS) at Stanford Children's Health attend a teaching session to learn more about obesity and its treatment. Patients and their parents then choose which treatment to pursue. This study aimed to evaluate the effects of treatment choice after teaching sessions on patient anthropometrics two years post‐session.

**Methods:** This is a retrospective chart review for all patients attended teaching session from January 2016 to July 2022. Data regarding long term outcome such as number of patients chose MBS, pursued medical management or lost to follow up were collected. Short‐term outcomes in this cohort were presented at ASMBS 2023.

**Results:** Of the 247 eligible patients, 214 had two‐year follow‐up data. Among those, 125 underwent MBS, 29 received medical or lifestyle management, and 60 dropped out. The average ages of patients by treatment type were 15.3 (MBS), 14.8 (medical/lifestyle), and 14.8 years (dropout). Gender and ethnicity were not significant across the groups. MBS patients lived significantly farther from the hospital (mean distance 125 miles, p=0.020) as compared to medical management (50 miles). The initial BMI values were similar across groups (46.8 kg/m^2^ ‐ 47.9 kg/m^2^), however the average BMI reduction was greatest in MBS patients (‐11.6 kg/m^2^), compared to no change in BMI two years post‐teaching session for those in the medical/lifestyle group, and an increase of 2 kg/m^2^ noted in those who dropped out (p<0.001). MBS patients reduced their BMI percentage of the 95^th^ percentile by an average of ‐45%, compared to ‐8% in those in the medical/lifestyle group, and 0% in the dropout group (p<0.001).

**Conclusion:** Two years post‐teaching session, patients who underwent MBS had significantly greater BMI reduction than those managed with medication or who dropped out. These findings may help adolescents deciding on treatment options for obesity.


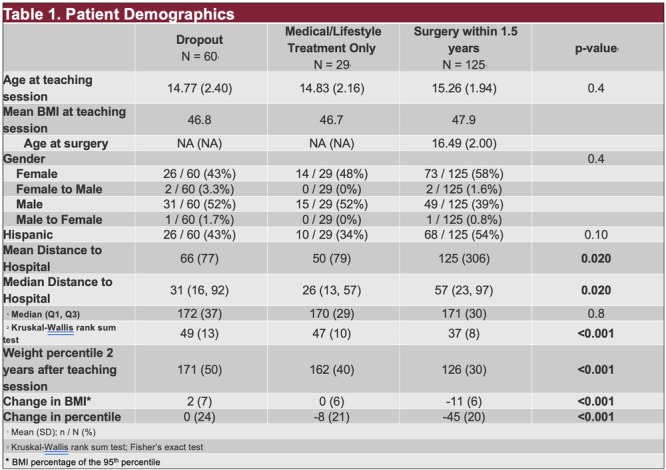


## 856 OPTIMIZING THE NUTRITION STATUS OF CHILDREN WITH CEREBRAL PALSY

*Judy‐April Murayi* ^*1*^, *Laurie Glader* ^*2*^, *Richard Stevenson* ^*3*^, *Praveen Goday* ^*1*^

^*1*^ *Pediatric Gastroenterology, Hepatology and Nutrition*, *Nationwide Children's Hospital*, *Columbus*, *OH*; ^*2*^ *Department of Complex Care*, *Nationwide Children's Hospital*, *Columbus*, *OH*; ^*3*^ *Developmental and Behavioral Pediatrics*, *University of Virginia*, *Charlottesville*, *VA*

**Objective:** The ideal nutrition status for children with cerebral palsy (CP) remains unknown. This study investigated the association between weight‐for‐age percentiles on the CP‐specific growth curves with clinical outcomes.

**Methods:** This was a retrospective cohort study of patients with CP (Gross Motor Classification System III‐V). The cohort was divided into patients ‘above’ and ‘not above’ the 20th percentile weight‐for‐age on CP‐specific growth curves based on patients being above/not above for at least 80% of the study period. Repeat measurements were collected on each patient once annually. We assessed for between‐group differences in emergency department (ED) or urgent care visits, hospital admissions, ICU admissions, fracture risk, surgery risk, and mortality.

**Results:** Of the 133 patients (63.9% male, age range: 3‐20 years), 118 were above and 15 were not above the 20th percentile weight‐for‐age. Those not above had an estimated 137% greater mean number of ED and urgent care visits compared to those that were above the threshold (p <0.001, 95% CI 1.36 to 4.14). The not above group also had 15.4% higher odds of requiring surgery (p < 0.001, 95% CI 1.15 to 1.16). There were 5 deaths (4.2%) in the above group, and 4 deaths (26.7%) in the not above group.

**Conclusion:** Pediatric patients with moderate‐to‐severe CP are at increased risk for requiring more acute care services and surgeries. They may also have an increased risk of mortality. Achieving a weight‐for‐age above the 20th percentile may be an important target for clinicians that care for patients with CP.


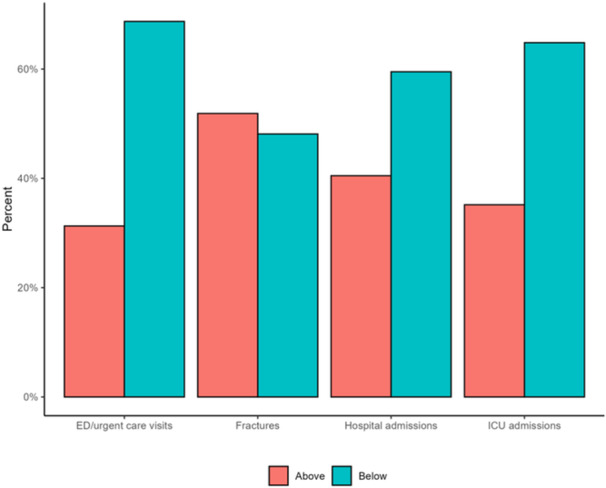


Healthcare Utilization by Weight Category

## 857 FOOD INSECURITY, FOOD ENVIRONMENT, AND BMIZ IN RURAL YOUTH: AN EXPLORATORY PATH ANALYSIS FROM IAMHEALTHY, A FAMILY‐BASED OBESITY INTERVENTION STUDY

*Hide Okuno* ^*2,6*^, *Anne Chuning* ^*2,6*^, *Madeline Maholland* ^*2,3*^, *Zachary Bricken* ^*2,6*^, *Brittany Lancaster* ^*2,4*^, *Bethany Forseth* ^*2,7*^, *Megan Olalde* ^*5,3*^, *Kandace Fleming* ^*1*^, *Rebecca Swinburne Romine* ^*1*^, *Ann Davis* ^*2,3*^

^*1*^ *Lifespan Institute*, *The University of Kansas*, *Lawrence*, *KS*; ^*2*^ *Center for Children's Healthy Lifestyles & Nutrition*, *Kansas City*, *KS*; ^*3*^ *Department of Pediatrics*, *The University of Kansas Medical Center*, *Kansas City*, *KS*; ^*4*^ *Department of Psychology*, *Mississippi State University*, *Mississippi State University*, *MS*; ^*5*^ *Center for Children's Healthy Lifestyles & Nutrition*, *Kansas City*, *KS*; ^*6*^ *Department of Clinical Child Psychology*, *The University of Kansas*, *Lawrence*, *KS*; ^*7*^ *Department of Physical Therapy, Rehabilitation Science, & Athletic Training*, *The University of Kansas Medical Center*, *Kansas City*, *KS*

**Objective:** Pediatric obesity represents a growing public health concern in the United States, particularly in rural areas. Children in rural areas are 30% more likely to have overweight or obesity (OW/OB) compared to their urban peers, underscoring a significant health disparity in rural communities. Multiple environmental and behavioral factors are associated with a child's body mass index z‐scores (BMIz), such as caloric intake, access to grocery stores and fast‐food outlets, and household food security. Food insecurity, paradoxically, has been identified as a factor for elevated obesity rates in children. However, there remains a paucity of research examining the relationships among these variables within rural populations. This study addresses this gap by exploring pathways through which food environment and caloric intake relate to BMIz among youth in rural areas. This study employed an exploratory path analysis to examine the associations between food insecurity, access to grocery stores, fast‐food restaurant density, caloric intake, and BMIz scores in youth with OB/OW living in rural communities. The aim was to identify potential pathways through which environmental and nutritional factors jointly contribute to pediatric obesity in rural settings.

**Methods:** Participants included 113 children (M age = 8.9 ± 0.85 years; 54.9% female) recruited from 15 elementary schools in rural Kansas as part of the iAmHealthy family‐based obesity intervention. Baseline data were analyzed. The participants’ home addresses were converted to Rural‐Urban Commuting Area (RUCA) code using the 2010 Federal Information Processing Standard code. RUCA code 4 to 10 was included in this study. BMIz scores were derived from height and weight measurements collected by trained school personnel. Food insecurity was measured using a parent‐reported six‐item Household Food Security Scale. The child's average caloric intake was estimated using three‐day dietary recall interviews with parents. Geographic food access indicators were sourced from the USDA Food Environment Atlas and linked to participants via census tract data corresponding to home address. Two measures of grocery store access were used: whether a grocery store is located more than 10 or 20 miles away. Fast‐food outlet density was sourced from USDA Food Access Research Atlas; the number of fast‐food outlets per 1,000 residents in the participants’ counties of residence. Path analysis tested a model in which caloric intake and food insecurity predicted BMIz, with mediation by grocery store access and fast‐food outlet density, controlling for gender.

**Results:** Mean caloric intake was 1787.80 kcal ± 393.21. Mean household food insecurity scale was 1.16 ± 1.69 (out of 6) points. The BMIz mean was 1.87 ± .52. Of the sample, 26.5% (n=30) lived in census tract where a grocery store access at 10 mile and 12.4% (n = 14) were at 20 miles. The path model showed acceptable fit indices (*χ* ^2^(10) = 16.52, *p* > 0.05; CFI = 0.90; TLI = 0.79; RMSEA = 0.08). Caloric intake was not significantly associated with BMIz through any direct or indirect pathway (*p*s > 0.05). The food insecurity was directly associated with limited grocery store access (≥10 miles; β = −0.18, *p* < 0.05), and the limited grocery store access (≥10 miles) was associated with fast food outlet density. (β = −0.54, *p* < 0.01). The fast‐food outlet density was associated with the child's BMIz (β = 0.20, *p* < 0.05). The model accounted for 35.3% of the variance in fast‐food access, 8.3% in grocery store access (≥10 miles), 0.5% in grocery store access (≥20 miles), and 3.9% in BMIz scores.

**Discussion:** The current exploratory path analysis suggests that household food insecurity plays an important role in affecting BMIz scores in youths with OB/OW in rural settings. This is crucial as families in rural areas are more likely to experience food insecurity. The pathway posits that food insecurity was associated with reduced access to grocery stores, which was indirectly associated with BMIz via the mediating role of fast‐food outlet density. Though previous studies had examined the relationship between food environments and BMIz in urban areas with mixed results, the current study explored further by adding the relationship with caloric intake and food insecurity. The study also revealed that there was no direct path from limited grocery store access to BMIz in rural population, rather, the access to fast‐food access plays a key role. As this study was exploratory in nature, these results require cross‐validation by an independent sample; however, the results provide a glimpse to the complex relationships between various factors that may contribute to BMIz scores for OB/OW youths living in rural communities.

## 858 SUMMARY OF CONSENSUS ON THE DIAGNOSIS, TREATMENT AND FOLLOW‐UP OF OVERWEIGHT AND OBESITY IN CHILDREN AND ADOLESCENTS: REPORT FROM LASPGHAN OBESITY WORKING GROUP

*Yunuen Rivera‐Suazo* ^*1*^, *Julia Alberto‐Meléndez* ^*2*^, *Jaime Ernesto Alfaro‐Bolaños* ^*1*^, *Felipe de Jesús Álvarez‐Chávez* ^*3*^, *Ana Gabriela Ayala‐Germán* ^*4*^, *María de Jesús Galaviz‐Ballesteros* ^*5*^, *Michelle Higuera‐Carillo* ^*6*^, *Claudia Lorena Taquez‐Castro* ^*7*^, *Alejandra Lorena Villa‐Gómez* ^*3*^, *Britta Ninoscka Villaroel‐Ibarra* ^*8*^, *Bertha Alejandra Alvarado‐Cárcamo* ^*9*^, *Fátima Azereth Reynoso‐Zarzosa* ^*10*^, *Yazmín Berenice Quiñones‐Pacheco* ^*11*^, *Carlos Marcelo Timossi‐Baldi* ^*12*^, *Rodrigo Vázquez‐Frias* ^*13*^

^*1*^ *Pediatric Gastroenterology*, *Centro Médico Nacional 20 de Noviembre, Instituto de Seguridad y Servicios Sociales de los Trabajadores del Estado*, *Mexico City*, *Mexico City*, *Mexico*; ^*2*^ *Pediatric Gastroenterology*, *Hospital Zafiro*, *Tegucigalpa*, *Tegucigalpa*, *Honduras*; ^*3*^ *Pediatric Gastroenterology*, *Hospital General de Zona No. 1, Instituto Mexicano del Seguro Social*, *Tepic*, *Nayarit*, *Mexico*; ^*4*^ *Pediatric Gastroenterology*, *Unidad Médica de Alta Especialidad, Instituto Mexicano del Seguro Social*, *Mérida*, *Yucatán*, *Mexico*; ^*5*^ *Pediatric Gastroenterology*, *Hospital General de Occidente*, *Zapopan*, *Jalisco*, *Mexico*; ^*6*^ *Pediatrics*, *Universidad El Bosque*, *Bogotá*, *Bogotá*, *Colombia*; ^*7*^ *Centros Médicos Colsanitas*, *Medellín*, *Medellín*, *Colombia*; ^*8*^ *Research*, *Incubadora de Investigación en Salud*, *Cochabamba*, *Cochabamba*, *Bolivia, Plurinational State of*; ^*9*^ *Hospital Comarcal Sant Jaume de Calella*, *Barcelona*, *Barcelona*, *Spain*; ^*10*^ *Hospital Angeles Puebla, Universidad UPAEP*, *Puebla*, *Puebla*, *Mexico*; ^*11*^ *Hospital Dr. Agustín O'Horán, Secretaría de Salud*, *Mérida*, *Yucatán*, *Mexico*; ^*12*^ *Research and Development*, *Miramar MedCom*, *Mexico City*, *Mexico City*, *Mexico*; ^*13*^ *Hospital Infantil de Mexico Federico Gomez*, *Mexico City*, *Mexico City*, *Mexico*

**Objectives and Study.** To provide clear and standardized guidelines on the diagnosis, treatment and follow‐up of overweight and obesity in children and adolescents in Latin America.

**Methods.** Six working subgroups were formed (15 specialists). 34 specialists (21 countries) were convened according to criteria of the California Evidence Code: 17 pediatric gastroenterologists, 7 pediatric endocrinologists, 9 pediatricians, 1 pediatric nutritionist; and voted using a 3‐point Likert scale: 1) agree, 2) abstain, 3) disagree. An anonymous voting process was conducted using Delphi method; statements were accepted if they achieved > 80% agreement, Cronbach's alpha coefficient >0.88 was reached.

**Results.** After two rounds of voting, 22 statements were obtained: definition, epidemiology and etiology (3 statements), diagnosis (no consensus), comorbidities (3 statements), metabolic evaluation (6 statements), treatment (5 statements) and prevention (5 statements). Statements are summarized in Table 1 and Figures 1‐5.

**Conclusions.** Although Latin America is a region that shares ethnic and cultural characteristics, there is a lack of uniformity regarding growth charts and cut‐off points to be used in the pediatric age group for clinical diagnosis of overweight and obesity, as well as different considerations regarding the biochemical evaluation protocol in these patients.

With this consensus, the authors attempt to unify the criteria for a multidisciplinary approach, with the aim of preventing and reducing the associated comorbidities.

It is imperative to promote, from all levels of care, a healthy lifestyle.


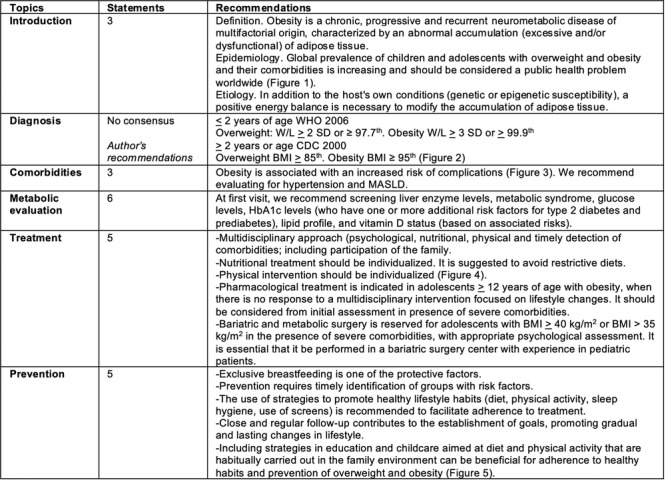


Table 1. Statements


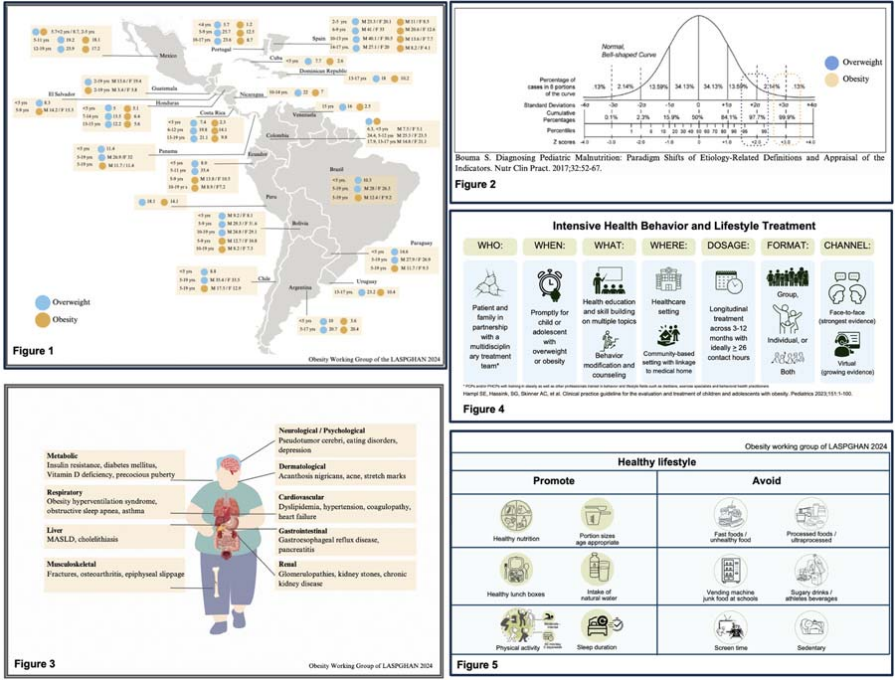


Figures 1‐5.

## 859 ASSOCIATION OF EATING HABITS AND PHYSICAL ACTIVITY WITH PEDIATRIC METABOLIC‐DYSFUNCTION ASSOCIATED STEATOTIC LIVER DISEASE (MASLD) IN CHILDREN WITH OVERWEIGHT (OW) AND OBESITY (OB) ‐ THROUGH A SELF‐COMPLETION QUESTIONNAIRE

*Mary José Huitron‐García* ^*2*^, *Frida Chin López‐Alvarado* ^*2*^, *Jaime Ernesto Alfaro‐Bolaños* ^*1*^, *Yunuen Rivera‐Suazo* ^*1*^

^*1*^ *Pediatric Gastroenterology*, *Centro Medico Nacional 20 de Noviembre*, *Mexico City*, *CDMX*, *Mexico*; ^*2*^ *Pediatrics*, *Centro Medico Nacional 20 de Noviembre*, *Mexico City*, *CDMX*, *Mexico*

**Background:** The rise in Ow and Ob in children worldwide is strongly associated with the rise in MASLD, which is the most common chronic liver disease in children and is characterized by hepatic steatosis with at least one cardiometabolic risk factor. In Mexico, the prevalence of Ow and Ob is 36.5% in school‐children and 40.4% in adolescents. The development of MASLD is associated with eating habits, and the characteristics of dietary intake are a relevant risk factor.

We aim to examine the correlation between anthropometric parameters, biochemical markers and screen time with the scores of a self‐completion questionnaire on dietary habits and physical activity.

**Methods:** A cross‐sectional study was conducted including children (8‐18 years old) with Ow and Ob. MASLD was diagnosed according to multisociety statement. A 31‐item self‐completion questionnaire was implemented. Section 1 included 4 items (6 questions) addressing the frequency and quantity of recommended food consumption. Section 2 contained 7 items (9 questions) focused on the intake of non‐recommended foods. Section 3 included 3 items (12 questions) related to the frequency, setting, and companionship during mealtimes. Section 4 assessed physical activity (frequency, if it is performed outside of school, and the overall type of lifestyle). For the evaluation of dietary habits and physical activity, a score of 0‐3 points was assigned for items consisting of a single question and 0‐1.5 points for items containing two or more questions. For section 1, the maximum score was 12 points, for section 2 it was 21 points, and for section 3 it was 18 points, giving a maximum of 51 points for the evaluation of dietary habits. For section 4, the maximum was 12 points. Dietary habits: inadequate < 25.5, partially inadequate > 25.5 to < 38.5, and adequate > 38.5. Physical activity: inadequate < 6, partially inadequate > 6 to 9, and adequate > 9.

**Results:** This study included 31 pediatric patients (Table 1). The mean age was 149 ± 33.7 months. Most participants (71%) were classified as obese, with 48.4% in class 1, 19.4% in class 2, and 3.2% in class 3. Elevated ALT (> 22 UI/L for females, >26 UI/L for males) were found in 67.7%. MASLD was diagnosed in 61.3% patients, with significantly higher ALT levels in this group (60.03 vs. 23.1 U/L; *p=0.048*). Total dietary habits score showed significant negative correlations with BMI (*r=–0.397*), waist circumference (*r=–0.478*), and liver stiffness (*r=–0.462*). HOMA‐IR was positively correlated with section 1 (*r=0.59*). Screen time was inversely associated with healthy eating and lifestyle behaviors. It negatively correlated with fruit and vegetable intake, portion sizes, and total dietary score (*r=–0.507*). A significant inverse correlation was also found with section 4 score (*r=–0.407*), including physical activity (*r=–0.404*), extracurricular activities (r*=–0.356*), and overall lifestyle (*r=–0.437*).

**Conclusions:** These findings underscore the importance of multidisciplinary interventions targeting dietary habits, screen time, and physical activity to prevent MASLD progression in children with Ow and Ob.

Contact. rivera.suazo@outlook.com


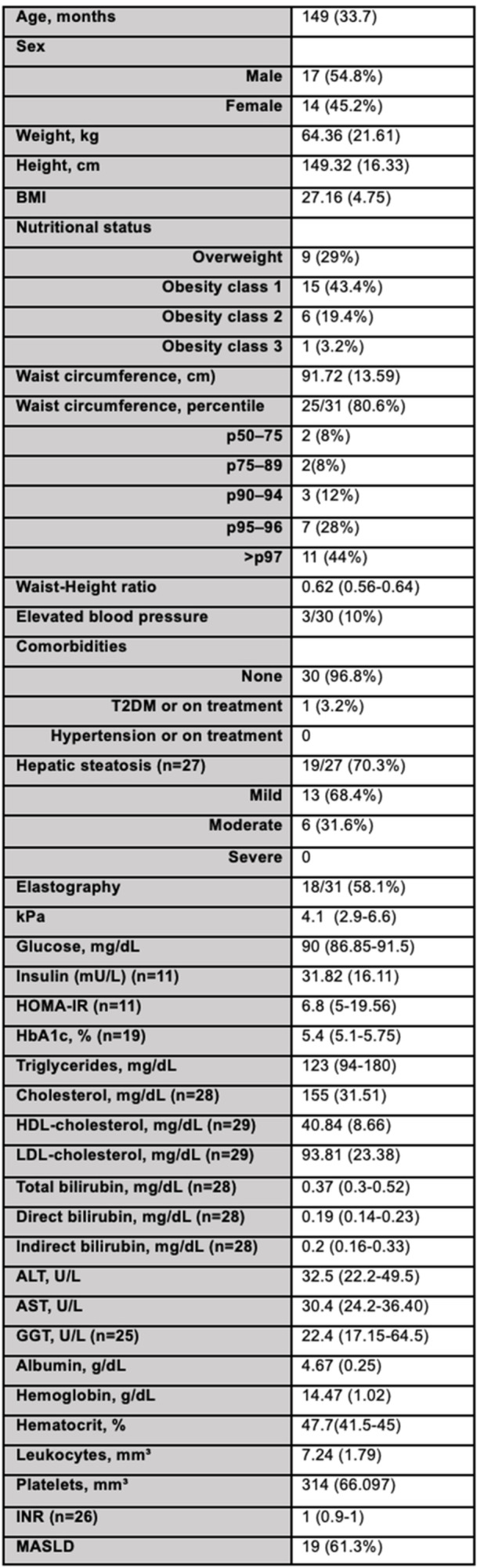


**Table 1. Characteristics of patients in the study population.:**


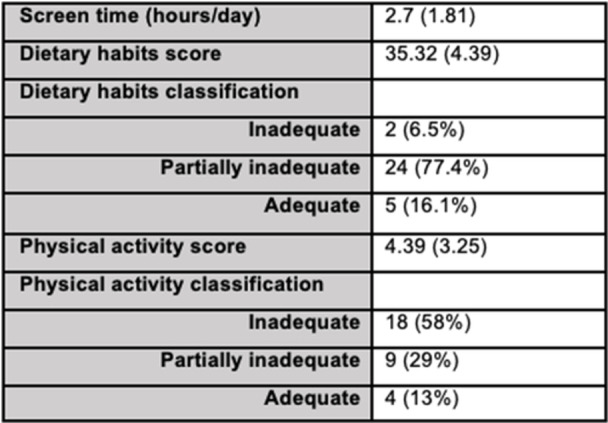


Table 2. Eating habits and physical activity.

## 861 LOW‐DOSE VERSUS STANDARD‐DOSE SOYBEAN‐BASED INTRAVENOUS LIPID EMULSIONS IN NEONATES: A SYSTEMATIC REVIEW AND META‐ANALYSIS

*Nágila Alves Lima* ^*3*^, *Jennifer Warner* ^*1,2*^, *Leticia Rocha Campos* ^*4*^, *Beatriz Ximenes Mendes* ^*3*^, *Francisco Willamy Pedrosa Alves Filho* ^*5*^, *Megan Butler* ^*1,2*^, *Matthew Kelly* ^*1,2*^

^*1*^ *Pediatrics*, *University of Arkansas for Medical Sciences*, *Little Rock*, *AR*; ^*2*^ *University of Arkansas for Medical Sciences*, *Little Rock*, *AR*; ^*3*^ *Centro Universitario Christus*, *Fortaleza*, *CE*, *Brazil*; ^*4*^ *Universidade de Ribeirao Preto*, *Ribeirao Preto*, *SP*, *Brazil*; ^*5*^ *Universidade Federal do Ceara*, *Sobral*, *CE*, *Brazil*

**Background:** Infants with gastrointestinal disorders often require long‐term parenteral nutrition (PN), which increases the risk of PN‐associated liver disease (PNALD), including cholestasis. Prolonged use of soybean oil‐based intravenous lipid emulsions (S‐ILE) is a recognized contributing factor. Although lipid restriction strategies—such as reducing S‐ILE dosage—have been proposed to lower this risk, the evidence remains unclear. This meta‐analysis evaluated whether a reduced S‐ILE dose (1 g/kg/day) decreases the incidence of PN‐associated cholestasis compared to standard dosing (2–3 g/kg/day) in neonates.

**Objective:** We aimed to evaluate the use of low‐dose versus standard‐dose of S‐ILE in neonates receiving parenteral nutrition.

**Methods:** We systematically searched PubMed, Embase, and Cochrane. Outcomes were: cholestasis, peak levels of direct bilirubin (DB), days of parenteral nutrition, weight velocity, discharge weight, and mortality. Risk ratios (RRs) with 95% confidence intervals (CIs) were calculated using a random effects model and the statistical method was inverse variance. Heterogeneity was assessed with I2 statistics and mean difference was used for effect measure. Review Manager v5.4.1 was used for statistical analyses. This systematic review and meta‐analysis were conducted by using the Cochrane Handbook for Systematic Reviews of Interventions and reported following the Preferred Reporting Items for Systematic Reviews and Meta‐Analyses (PRISMA) guidelines. The protocol was prospectively registered in PROSPERO (CRD420251057906).

**Results:** 721 participants from 6 randomized controlled trials (RTCs) and 3 non‐randomized cohorts were included. All patients in the low‐dose group used a dose of 1 g/kg/day, while in the standard‐dose group, it varied between 2‐3 g/kg/day. The mean gestational age in the low‐dose group was 32.2±5.13 weeks and in standard‐dose was 32.17+‐5.22 weeks (mean+‐SD). Approximately 47% of the patients had gastroschisis as a previous disease and 43.3% of the study population was male. There were no differences between low‐dose of S‐ILE and standard dose of S‐ILE regarding the outcomes of: cholestasis (43.7% vs 46.4%; RR 0.94; 95% CI [0.66, 1.34]; p=0.73; I2=63%); peak levels of DB (MD ‐0.30 mg/dl; 95% CI [‐0.90,0.30]; p=0.33; I2=0%); mortality (7.8% vs 6.1%; RR 1.24; 95% CI [0.63, 2.46]; p=0.53; I2=0%); days of parenteral nutrition (MD ‐1.97 days; 95%CI [‐6.66, 2.73]; p=0.41; I2=0%); discharge weight (MD‐114.57 g; 95% CI [‐460.5, 231.3]; p=0.52; I2=0%) and weight velocity (MD ‐1.87 g/day; 95% CI [‐6.13, 2.4]; p=0.39; I2=0%).

**Conclusion:** There is no difference between the use of low doses and standard doses of S‐ILE in parenteral nutrition in neonates regarding outcomes: cholestasis, peak levels of DB, mortality, days of parenteral nutrition, discharge weight, and weight velocity.

**References:**

Calkins KL, Havranek T, Kelley‐Quon LI, Cerny L, Flores M, et al. Low‐Dose Parenteral Soybean Oil for the Prevention of Parenteral Nutrition‐Associated Liver Disease in Neonates With Gastrointestinal Disorders. JPEN J Parenter Enteral Nutr. 2017 Mar;41(3):404‐411.

Cober MP, Killu G, Brattain A, Welch KB, Kunisaki SM, at el. Intravenous fat emulsions reduction for patients with parenteral nutrition‐associated liver disease. J Pediatr. 2012 Mar;160(3):421‐7.

Gupta K, Wang H, Amin SB. Soybean‐Oil Lipid Minimization for Prevention of Intestinal Failure‐Associated Liver Disease in Late‐Preterm and Term Infants With Gastrointestinal Surgical Disorders. JPEN J Parenter Enteral Nutr. 2021 Aug;45(6):1239‐1248.

Huff KA, Cruse W, Vanderpool C. Lipid strategies to prevent intestinal failure‐associated liver disease in neonates: A pilot trial. JPEN J Parenter Enteral Nutr. 2023 May;47(4):482‐493.

Levit OL, Calkins KL, Gibson LC, Kelley‐Quon L, Robinson DT, et al. Low‐Dose Intravenous Soybean Oil Emulsion for Prevention of Cholestasis in Preterm Neonates. JPEN J Parenter Enteral Nutr. 2016 Mar;40(3):374‐82.

Maselli KM, Carter IC, Matusko N, Warschausky S, Blackmer AB, et al. Prevention of Parenteral Nutrition‐associated Cholestasis Using Reduced Dose Soybean Lipid Emulsion: A Multicenter Randomized Trial. J Pediatr Surg. 2024 Jul;59(7):1369‐1373.

Nehra D, Fallon EM, Carlson SJ, Potemkin AK, Hevelone ND, et al. Provision of a soy‐based intravenous lipid emulsion at 1 g/kg/d does not prevent cholestasis in neonates. JPEN J Parenter Enteral Nutr. 2013 Jul;37(4):498‐505.

Rollins MD, Ward RM, Jackson WD, Mulroy CW, Spencer CP, et al. Effect of decreased parenteral soybean lipid emulsion on hepatic function in infants at risk for parenteral nutrition‐associated liver disease: a pilot study. J Pediatr Surg. 2013 Jun;48(6):1348‐56.

Sanchez SE, Braun LP, Mercer LD, Sherrill M, Stevens J, et al. The effect of lipid restriction on the prevention of parenteral nutrition‐associated cholestasis in surgical infants. J Pediatr Surg. 2013 Mar;48(3):573‐8

## 862 TOWARD A SINGLE SOURCE OF TRUTH: THE CASE FOR CENTRALIZED NUTRITION DOCUMENTATION IN EMR'S

*Jocelyn Young* ^*1,2*^, *Mary Beth Feuling* ^*3*^, *Jeannie Huang* ^*1,2*^

^*1*^ *Pediatric Gastroenterology, Hepatology, and Nutrition*, *Rady Children's Hospital San Diego*, *San Diego*, *CA*; ^*2*^ *Pediatrics*, *University of California San Diego School of Medicine*, *La Jolla*, *CA*; ^*3*^ *Clinical Nutrition*, *Children's Wisconsin*, *Milwaukee*, *WI*

**Objective:** Nutrition is an essential component of care for all patients. However, electronic medical record (EMR) systems often lack a centralized location for nutrition‐related information. This fragmentation contributes to miscommunication, inappropriate nutrition therapy, and preventable safety events. The absence of standardized documentation and workflows is misaligned with key domains of healthcare quality as defined by the Institute of Medicine—namely, safety, patient‐centeredness, and efficiency. This study aims to identify opportunities for EMRs to enhance nutrition evaluation, management, and safety.

**Methods:** A 12‐item online survey was developed to assess current practices in EMR documentation of nutrition information, identify nutrition‐related adverse events, and elicit suggestions for EMR improvements. Most survey item responses were categorical, with one question utilizing a Likert scale ranging from 1 (not well) to 10 (very well). Additionally, two open‐ended questions were included: one exploring participants’ suggestions for improving nutrition data sharing within the EMR, and another inquiring about the nature of nutrition‐related adverse events.

Survey distribution targeted clinicians who work with children with nutritional concerns and who utilize an EMR system to deliver care. Invitations for study participation were distributed via email to select members of NASPGHAN, including CPNP, the Nutrition Committee, as well as the Intestinal Rehabilitation and the Electronic Health Record Special Interest Groups.

Descriptive statistics and the Kruskal‐Wallis test were utilized to analyze quantitative survey data. Qualitative data were analyzed thematically to identify key concerns and a core theory reflecting respondents’ perspectives.

**Results:** One‐hundred and fifteen participants (out of the 510 invited) completed the survey, resulting in a response rate of 22.5%. Only 54% of participants reported having a standardized EMR workflow for documenting food allergies and intolerances. Notably 86% of respondents reported using free text to share nutrition information. Participants noted multiple challenges in locating nutrition plans within the EMR, including the absence of a standard location for nutrition information, lack of order interoperability across care settings, and inconsistent or outdated information. Most respondents (73%) reported awareness of at least one nutrition‐related safety event.

**Conclusion:** There is a critical need to optimize and standardize where nutrition information is stored and documented within EMRs. Healthcare providers are calling for a unified, integrated system that clearly distinguishes a patient's feeding/nutrition plan, food allergies and intolerances, enhances the visibility of essential nutrition data, and links dietary information across departments and care settings to improve patient safety and care coordination.


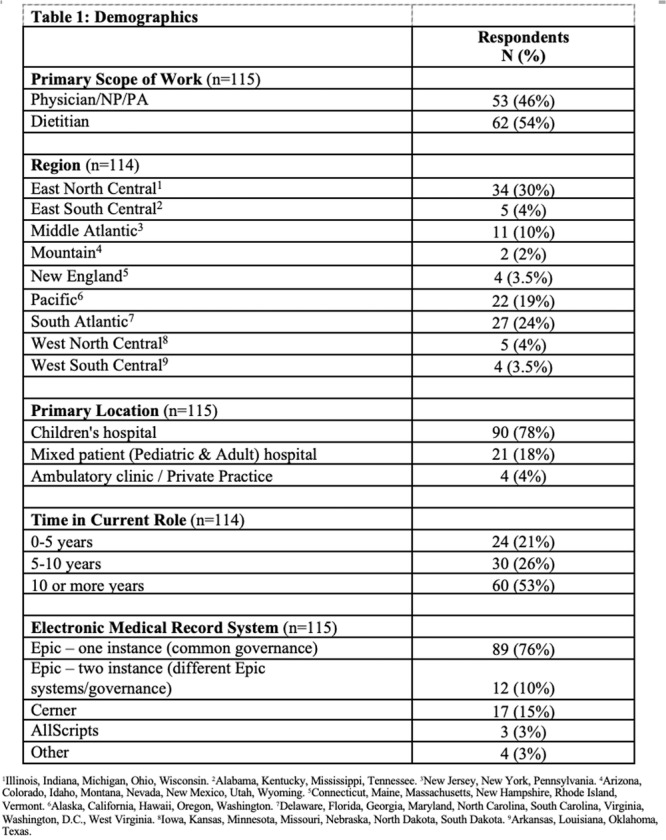


TABLE 1. Demographics


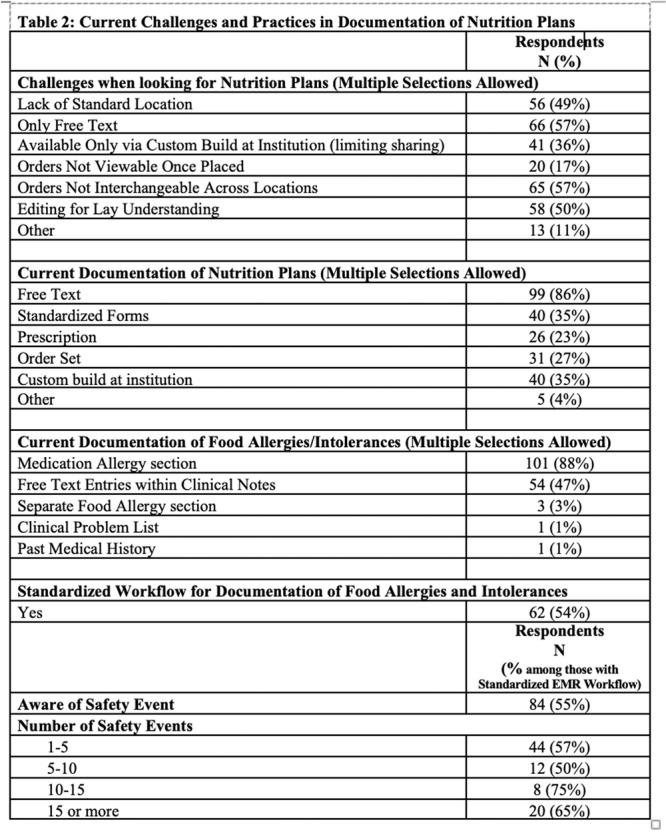


TABLE 2. Current Challenges and Practices in Documentation of Nutrition Plans

## 863 USING A SPANISH‐LANGUAGE INTRODUCTION PROTOCOL TO SUPPORT ENGAGEMENT IN PEDIATRIC DGBI CARE

*Anthony Cifre* ^*1,2*^, *Leonel Rodriguez* ^*1*^, *Maggie Stoeckel* ^*1,2*^

^*1*^ *Pediatrics*, *Yale School of Medicine*, *New Haven*, *CT*; ^*2*^ *Child Study Center*, *Yale School of Medicine*, *New Haven*, *CT*

**Background:** Disorders of gut‐brain interaction (DGBIs) present unique challenges in pediatric care due to their biopsychosocial complexity. In integrated GI clinics, clear explanations of the gut‐brain connection and psychology's role are important for family engagement, especially for Spanish‐speaking families, who may face added linguistic and cultural barriers. These families may also experience distrust or skepticism toward psychological services, stemming from cultural stigma, past negative experiences, or unfamiliarity with integrated models of care. This pilot project aims to develop and implement a structured Spanish‐language introduction protocol to support families’ understanding of DGBIs, facilitate buy‐in to psychological services, and promote collaborative care.

**Protocol Overview:** The protocol includes a brief explanation of DGBIs using accessible metaphors, culturally responsive psychoeducation about the gut‐brain connection, and clarification of the psychologist's role within the GI team. It was developed and will be delivered by Spanish‐speaking providers (attending physician and psychology trainee) in an interdisciplinary pediatric DGBI clinic. Implementation focuses on the initial visit and follow‐up sessions as needed. Sample phrases used in the protocol include:

“El estómago y el cerebro están conectados como si hablaran por teléfono, y a veces esa señal se interrumpe.”

“No es que los síntomas estén ‘en la cabeza’ — enseñamos estrategias que ayudan a calmar el cuerpo y mejorar la conexión entre el cerebro y el intestino.”

“Trabajamos juntos por unas pocas sesiones para practicar herramientas como respiración, manejo del estrés y mejorar el sueño.”

**Clinical Implementation:** We will describe preliminary implementation in a small sample of Spanish‐speaking families (target sample = 5*) seen in an integrated DGBI clinic. Families are invited to share their impressions of the protocol, with attention to understanding, engagement, and readiness to participate in behavioral strategies. Preliminary themes will be shared regarding family reactions, common questions, and cultural considerations.

**Conclusions:** This project highlights the need for intentional, linguistically appropriate, and culturally sensitive approaches to introducing integrated care to Spanish‐speaking families. As pediatric GI teams increasingly serve diverse populations, tools such as a Spanish DGBI introduction protocol may enhance access, understanding, and clinical outcomes.

* Data collection to be completed by Fall 2025 for presentation at the national conference

## 864* TSLP EXPRESSION AND ACTIVITY PRE‐ AND POST‐EOSINOPHIL DEPLETION WITH BENRALIZUMAB TREATMENT IN PATIENTS WITH EOSINOPHILIC ESOPHAGITIS: INSIGHTS FROM THE MESSINA STUDY

*Christopher Nazaroff* ^*1*^, *Patrick Gavin* ^*1*^, *Joseph Sherrill* ^*1*^, *Wendy White* ^*2*^, *Margaret Collins* ^*3*^, *Marc Rothenberg* ^*4*^, *Evan Dellon* ^*5*^, *Albert Bredenoord* ^*6*^, *Carlos Seminario* ^*7*^, *Harald Fjällbrant* ^*8*^, *Hanna Grindebacke* ^*8*^, *Daniel Muthas* ^*9*^, *Adam Platt* ^*10*^, *Christopher McCrae* ^*1*^

^*1*^ *Translational Science and Experimental Medicine, Research and Early Development, Respiratory and Immunology, BioPharmaceuticals R&D*, *AstraZeneca*, *Gaithersburg*, *MD*; ^*2*^ *Clinical Pharmacology and Quantitative Pharmacology, Clinical Pharmacology and Safety Sciences, BioPharmaceuticals R&D*, *AstraZeneca*, *Gaithersburg*, *MD*; ^*3*^ *Department of Pathology & Laboratory Medicine, University of Cincinnati College of Medicine*, *Cincinnati Children's Hospital Medical Center*, *Cincinnati*, *OH*; ^*4*^ *Division of Allergy and Immunology, Department of Pediatrics*, *Cincinnati Children's Hospital Medical Center*, *Cincinnati*, *OH*; ^*5*^ *Center for Esophageal Diseases and Swallowing, Division of Gastroenterology and Hepatology*, *The University of North Carolina at Chapel Hill School of Medicine*, *Chapel Hill*, *NC*; ^*6*^ *Department of Gastroenterology and Hepatology*, *Amsterdam University Medical Center*, *Amsterdam*, *Netherlands*; ^*7*^ *Clinical, Late‐stage Development, Respiratory and Immunology, BioPharmaceuticals R&D*, *AstraZeneca*, *Gaithersburg*, *MD*; ^*8*^ *Late Stage Respiratory and Immunology, BioPharmaceuticals R&D*, *AstraZeneca*, *Gothenburg*, *Sweden*; ^*9*^ *Translational Science and Experimental Medicine, Research and Early Development, Respiratory and Immunology, BioPharmaceuticals R&D*, *AstraZeneca*, *Gothenburg*, *Sweden*; ^*10*^ *Translational Science and Experimental Medicine, Research and Early Development, Respiratory and Immunology, BioPharmaceuticals R&D*, *AstraZeneca*, *Cambridge*, *United Kingdom*

**Background:** Eosinophilic esophagitis (EoE) is a chronic inflammatory disorder characterized by eosinophil‐predominant inflammation and esophageal dysfunction. Benralizumab, an anti‐interleukin‐5 receptor α monoclonal antibody, induces eosinophil depletion. In the phase 3 MESSINA trial (NCT04543409), benralizumab achieved a histologic response but did not significantly improve symptoms or endoscopic features versus placebo in patients with EoE. Thymic stromal lymphopoietin (TSLP), an upstream epithelial cytokine, is associated with EoE pathogenesis. We evaluated TSLP expression and activity at baseline and following eosinophil depletion with benralizumab.

**Methods:** MESSINA was a multicenter, double‐blind, placebo‐controlled trial that enrolled patients (12–65 years) with active EoE. Patients were randomized 1:1 to receive benralizumab 30 mg (n=103) or placebo (n=107) subcutaneously every 4 weeks for 24 weeks. Distal esophageal biopsies were evaluated at baseline and week 24 using bulk RNA sequencing and immunohistochemistry. Patients were grouped by high or low TSLP activity based on median gene set activity scores obtained using a TSLP‐stimulated myeloid dendritic cell‐derived gene signature previously shown to be enriched in EoE. Differentially expressed genes (DEGs) were identified using DESeq2 software, followed by gene set enrichment analysis using fgsea software. Spearman's rank correlation coefficients were determined, and *p* values calculated using a Wilcoxon test or ANOVA.

**Results:** Esophageal TSLP gene expression and activity were weakly correlated at baseline (*r*=0.21; *p*=0.015) and were unchanged following benralizumab treatment (Fig. 1). At baseline, 1215 DEGs were identified in the high TSLP activity subgroup (n=35) versus the low TSLP activity subgroup (n=34) that were significantly associated with activation of multiple inflammatory cell pathways, including those involving natural killer T cells, T helper cells and B cells; high TSLP activity was associated with activation of similar inflammatory cell pathways following benralizumab treatment (Fig. 2). High TSLP activity was associated with higher peak tissue and blood eosinophil counts at baseline versus the low TSLP activity subgroup (*p*=0.0001 and *p*=0.016, respectively). Patients with high TSLP activity had a higher median [IQR] total Endoscopic Reference Score than those with low TSLP activity (baseline: 10.5 [8.0–12.2] and 8.0 [6.0–10.0], respectively; *p*=0.013; week 24: 9.0 [7.0–11.0] and 6.0 [3.2–10.0], respectively; *p*=0.024).

**Conclusions:** Despite esophageal eosinophil depletion with benralizumab, high TSLP activity persisted that is associated with multiple inflammatory pathways that may underlie severe clinical features of EoE. The phase 3 CROSSING study (NCT05583227) of tezepelumab, an anti‐TSLP antibody, in patients with EoE is ongoing.

**Funding:** This study was funded by AstraZeneca. Medical writing support was provided by Richa Tripathi, PhD, of PharmaGenesis, London, UK, with funding from AstraZeneca.


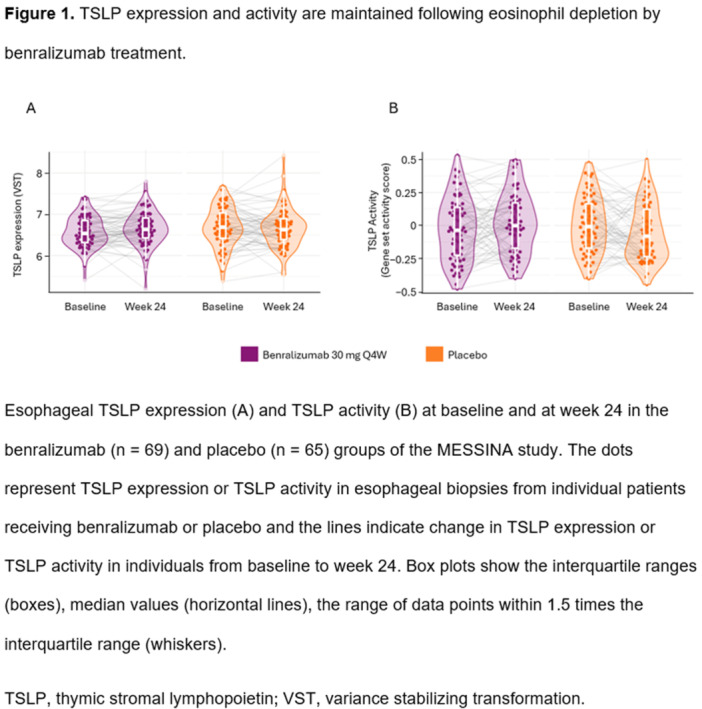



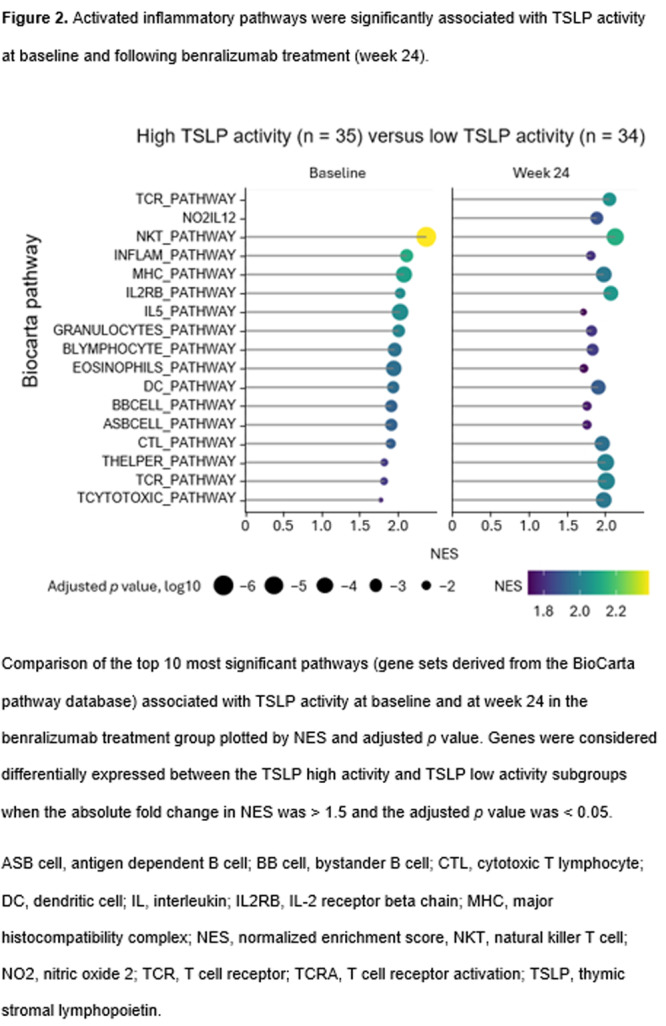


## 865 EXPLORATORY SINGLE‐ARM, OPEN‐LABEL NON‐RANDOMIZED TRIAL EVALUATING THE SAFETY AND EFFECTIVENESS OF CROFELEMER IN PEDIATRIC PATIENTS WITH INTESTINAL FAILURE

*Mohammad Miqdady* ^*1*^, *Pravin Chaturvedi* ^*2*^, *Amal Omar* ^*1*^

^*1*^ *Pediatrics*, *Sheikh Khalifa Medical City*, *Abu Dhabi*, *Abu Dhabi*, *United Arab Emirates*; ^*2*^ *Napo Therapeutics*, *San Francisco*, *CA*

**Background and Aim:** Congenital intestinal diseases and short bowel syndrome (SBS) are common causes of intestinal failure (IF), resulting in inadequate intestinal malabsorption requiring total parenteral nutrition (TPN). SBS IF patients have reduced absorptive surface and microvillus inclusion disease (MVID) causes malabsorption due to hypoplastic villus atrophy and defective brush border assembly and differentiation; both diseases requiring lifelong intravenous nutrition which may increase life expectancy but may also lead to significant morbidity and mortality. Currently there is no curable therapies. Crofelemer, an FDA‐approved novel antisecretory drug, it improves stool consistency through modulation of two gastrointestinal chloride ion channels; cystic fibrosis transmembrane conductance regulator (CFTR) and calcium‐activated chloride channel (CaCC). Crofelemer was evaluated for reduction in TPN needs and diarrhea in a 12‐week open‐label clinical trial in pediatric IF patients.

**Methods:** IF patients >1 to <18 years requiring PS for >60 days were enrolled.; Exclusion criteria included hypersensitivity to crofelemer; malignancy; bowel surgery <6 months; use of immunosuppressants; and active infection. Seven days after screening, oral crofelemer dosing was initiated for 12 weeks, with maximum participation duration of 16 weeks. Liquid formulation of crofelemer was administered orally, three times per day (TID) in this dose‐escalation study at dose levels of 3 mg/kg TID for the initial 2 weeks; followed by 6 mg/kg/dose TID for the next 2 weeks; and 9 mg/kg/dose TID for the last 8 weeks. Patients were eligible for reinitiation of crofelemer dosing following protocol‐mandated cessation of treatment in case of worsening IF symptoms at the investigator's discretion.

**Primary objectives:** assessments of safety and effectiveness of oral crofelemer in pediatric IF patients, in reducing their need for parenteral support (PS) (PN + intravenous fluids). Safety assessments included medical history, physical examination, vital signs, height & weight, and laboratory assessments. Effectiveness was assessed from daily diaries for PS volume, oral intake, stool frequency and consistency. Secondary objectives included improvements in oral intake, weight gain and reduction in loose or watery stool output and/or frequency.

**Results:** trial is still ongoing, three patients of the planned 6 IF patients, have completed treatment, one with MVID and two with SBS‐IF. Crofelemer was well tolerated with no physical or laboratory abnormalities. Following dose‐escalation to 9 mg/kg TID, crofelemer demonstrated significant reductions in weekly TPN for IF patients ranging from 12.5‐27% during the 12‐week treatment period. Reductions in loose/watery stool output, improved hydratioj status with increased urine output were observed. No adverse safety signals were observed during 12 weeks of high‐dose therapy, except for occasional, red‐colored urine in the MVID‐IF patient. Urinalysis showed no adverse findings and dosing with crofelemer was not interrupted. More results will be presented at the time of the congress.

**Conclusions:** Oral crofelemer in pediatric IF patients at dose levels of 3, 6 and 9 mg/kg TID were well tolerated with no crofelemer‐related adverse events. PS needs were reduced for IF patients between 12.5‐27% over the 12‐week treatment together with reduced stool output and increased urine output. These findings support continued evaluation of crofelemer to reduce PS needs for pediatric IF patients.

## 866 EVALUATION OF THE PHARMACOKINETICS AND SAFETY OF VONOPRAZAN IN CHILDREN AGED ≥6 TO

*Darcy Mulford* ^*1*^, *David Gremse* ^*2*^, *Yu‐Ming Chang* ^*1*^, *Eckhard Leifke* ^*1*^, *Thomas Wagner* ^*3*^, *Axel Facius* ^*3*^, *Galen Witt* ^*1*^

^*1*^ *Clinical Pharmacology*, *Phathom Pharmaceuticals LLC*, *Florham Park*, *NJ*; ^*2*^ *Department of Pediatrics*, *University of South Alabama*, *Mobile*, *AL*; ^*3*^ *thinkQ2 AG*, *Baar*, *Switzerland*

**Introduction:** Proton pump inhibitors (PPIs) are a pharmacological mainstay for the treatment of gastroesophageal reflux disease (GERD) in children. Vonoprazan is a potassium‐competitive acid blocker (P‐CAB) and has a pharmacological profile of more rapid, potent, and durable elevations in gastric pH compared to PPIs in adults and may be a highly effective treatment option for children with GERD. This is the first study to evaluate the pharmacokinetic (PK) and safety profiles of vonoprazan in children ≥6 to <12 years of age with symptomatic GERD (NCT06106022).

**Methods:** Pediatric subjects (aged ≥6 to <12 years) with symptomatic GERD were randomized to receive vonoprazan 10 mg or vonoprazan 20 mg once daily for 14 days. Plasma samples were collected on both Days 7 and 14 for measurement of concentrations of vonoprazan using liquid chromatography with tandem mass spectrometry. A two‐compartment population PK (popPK) model previously developed with PK data from adults and adolescents was used to analyze the data. Individual post hoc empirical Bayes estimates of PK parameters including individual oral clearance (CL) and central volume of distribution (V_c_) were used to predict dense plasma concentration profiles at steady state. From these predicted values, individual exposure parameters of maximum concentration at steady state (C_max,SS_) and area under the drug concentration‐time curve during the dosing interval τ at steady state (AUC_τ,SS_) were calculated. Individual apparent oral clearance (CL/F) and central volume of distribution (V_c_/F) were also calculated from the model‐based estimates for these parameters. Additionally, vonoprazan exposures in children, adolescents, and adults were computed using dose and individual model‐based CL estimates.

**Results:** Among 22 subjects enrolled, 11 were randomized to vonoprazan 10 mg once daily and 11 were randomized to vonoprazan 20 mg once daily. The mean age of the subjects was 8.9 years, 54.5% of the subjects were female, and their mean body weight was 38.6 kg. One subject was excluded from the PK analysis due to concerns regarding the subject's self‐administered dosing records and being enrolled despite not meeting protocol specified criteria. Individual PK and exposure paramters for children ≥6 to <12 years of age are summarized in **Table 1**. There was a slightly greater than dose proportional increase for both C_max,SS_ (1.3 times) and AUC_τ,SS_ (1.42 times) between the 10 and 20 mg doses of vonoprazan. As expected, CL/F and V_c_/F values were similar for both doses. A separate model‐based assessment of exposure showed that mean AUC_τ,SS_ (dose‐normalized to 20 mg vonoprazan) for children ≥6 to <12 years of age (215 ng•h/mL) was only slightly greater than for adolescents (198 ng•h/mL) and adults (209 ng•h/mL). One subject in the vonoprazan 10 mg group reported 4 treatment‐emergent adverse events (TEAEs). None of the TEAEs were serious or resulted in treatment discontinuation.

**Conclusions:** Exposures of vonoprazan in children ≥6 to <12 years of age were not meaningfully different from exposures observed in adolescents and adults, confirming the appropriateness of vonoprazan 10 mg and 20 mg doses in children ≥6 to <12 years of age.


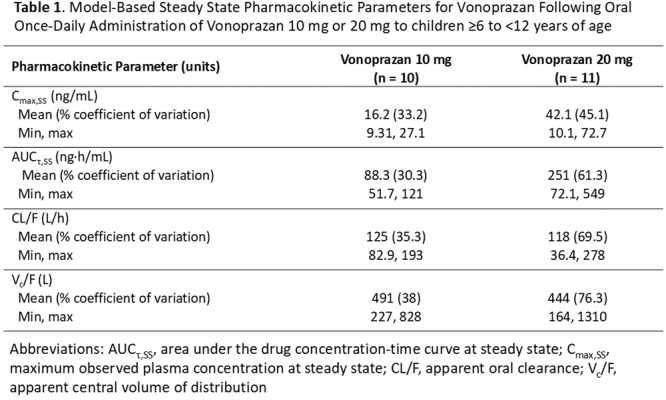


## 867 PREDICTIVE MODEL FOR TACROLIMUS ADHERENCE IN PEDIATRIC POST‐LIVER TRANSPLANT PATIENTS BASED ON SOCIAL DETERMINANTS OF HEALTH FACTORS

*Blake Rosenthal*, *Shreena Patel*

*Gastroenterology*, *Children's Hospital Los Angeles*, *Los Angeles*, *CA*

Disparities in race, socioeconomic status, language, distance to transplant center, and level of family support have been shown to affect liver transplant outcomes in the pediatric population.^1‐3^ In a retrospective study of 208 pediatric liver transplant patients, Thammana et al. demonstrated that non‐White children had higher rates of graft failure and mortality when adjusted for demographic, clinical and socioeconomic characteristics.^2^ In another single‐center retrospective cohort of pediatric acute liver failure patients requiring liver transplant, the cohort of children that died had the largest proportion of non‐English speaking patients and parents who worked full time with limited level of family support.^3^

Medication adherence post‐transplant, particularly immunosuppressant medications, is critical in preventing rejection and promoting graft success. High Tacrolimus Medication Level Variability Index (MLVI) >2.0 is reflective of less consistent medication compliance and has been associated with increased risk for rejection in pediatric and adult studies.^4‐6^

In this retrospective study, our objective is to identify social determinants of health (SDOH) factors that are most influential in predicting MLVI >2.0 to better identify and assist children and families who need additional support. Low socioeconomic neighborhood status has been shown to be associated with elevated MLVI, but there is less data on other SDOH factors. Wadhwani et al. show in their secondary analysis of MALT (Medication Adherence in children who had a Liver Transplant) prospective multi‐site study data that neighborhood socioeconomic deprivation was associated with elevated MVLI. About 25% of patients from the lowest quartile socioeconomic neighborhood had MLVI ≥2.5 compared to 12% in the remaining 3 quartiles.^6^

We will use multivariable weighted regression analysis to determine a predictive score for MLVI >2.0 based on collected SDOH factors, anticipating inclusion of 3‐5 factors in our score based on the expectation of approximately 300 patients meeting inclusion criteria. Inclusion criteria include patients who received liver transplantation at CHLA between January 1, 2000 and December 31, 2023, Tacrolimus prescribed during the first year of transplant, and at least 3 serum tacrolimus levels at intervals of approximately 3 months recorded between the first 3 months to 1‐year post‐transplant. Patients with more than one transplant will be excluded.

Data collection and analysis is ongoing. We anticipate analysis and score development to be complete by August 2025 with results to be presented at the 2025 NASPGHAN conference. Part two of this study will involve prospective enrollment of pre‐transplant patients with the goal of testing the accuracy of our score in predicting MLVI >2 post‐transplant.

1. Ebel NH, Lai JC, Bucuvalas JC, Wadhwani SI. A review of racial, socioeconomic, and geographic disparities in pediatric liver transplantation. *Liver Transpl*. 2022 Sep;28(9):1520‐1528.

2. Thammana RV, Knechtle SJ, Romero R, Heffron TG, Daniels CT, Patzer RE. Racial and socioeconomic disparities in pediatric and young adult liver transplant outcomes. *Liver Transpl*. 2014 Jan;20(1):100‐15.

3. Ascher Bartlett J, Barhouma S, Bangerth S, et al. Finance, race, ethnicity, and spoken language impact clinical outcomes for children with acute liver failure. *Pediatr Transplant*. 2024;28(1):e14686.

4. Shemesh E, Bucuvalas JC, Anand R, Mazariegos GV, Alonso EM, Venick RS, Reyes‐Mugica M, Annunziato RA, Shneider BL. The Medication Level Variability Index (MLVI) Predicts Poor Liver Transplant Outcomes: A Prospective Multi‐Site Study. *Am J Transplant*. 2017 Oct;17(10):2668‐2678.

5. Supelana S, Annunziato RA, Schiano TD, Anand R, Vaidya S, Chuang K, Zack Y, Florman S, Shneider BL, Shemesh E. Medication level variability index predicts rejection, possibly due to nonadherence, in adult liver transplant recipients. *Liver Transpl*. 2014 Oct;20(10):1168‐77.

6. Wadhwani SI, Bucuvalas JC, Brokamp C, Anand R, Gupta A, Taylor S, Shemesh E, Beck AF. Association Between Neighborhood‐level Socioeconomic Deprivation and the Medication Level Variability Index for Children Following Liver Transplantation. *Transplantation*. 2020 Nov;104(11):2346‐2353.

## 868 INTERDISCIPLINARY VIRTUAL THERAPY PROGRAM TO PROMOTE ORAL FEEDING IN TODDLERS WITH FEEDING TUBES AND TRACHEOSTOMIES

*Karen Dilfer*, *Erika Meier*, *Julia Sadowski*, *Sarah Sobotka*

*Pediatrics*, *The University of Chicago Division of the Biological Sciences*, *Chicago*, *IL*

**Background and Objectives:** Long‐term feeding tube (FTs) use and Pediatric Feeding Disorders (PFDs) are highly prevalent in children with tracheostomies as a result of prematurity, delayed feeding experiences and past medical trauma. Despite families prioritizing eating by mouth, due to respiratory vulnerabilities including risk of aspiration pneumonia, families and providers are often hesitant to advance oral feeding and reduce tube feeding without expert guidance. Children with FTs and tracheostomies often do not receive needed therapies in the community through early intervention. Travel to tertiary care centers for therapies can be challenging for families of children with medical technologies, and therefore an approach which incorporates virtual therapies is the most feasible habilitative therapy model. To our knowledge, there are no studies which examine feeding interventions specifically for children with FTs and tracheostomies. Our objectives are to study the preliminary impact of an interdisciplinary virtual feeding intervention on advancing oral intake and reducing feeding tube dependency.

**Study Participants and Setting:** Children with FTs and tracheostomies under 3 years of age were recruited through a state‐wide agency and subspecialty clinics to participate in a randomized controlled trial of an interdisciplinary virtual therapy model. If determined safe to advance oral feeding after an in‐home assessment by the study team (a Developmental and Behavioral Pediatrician, Occupational Therapist (OT), Speech and Language Pathologist (SLP), and Registered Dietitian) children were randomized into intervention and control groups. All subsequent touch points, therapies, feeding groups and interval assessments occurred over video conferencing. The study is ongoing.

**Methods:** Children in the intervention group had oral and tube feeding actively managed by the study team, which may have included reducing the frequency or amount of tube feeding and/or increasing frequency or types of oral intake. The intervention group received biweekly feeding therapy conducted over zoom by a feeding therapist (OT or SLP) with a parent or caregiver present. These therapies used a parent coaching model to promote responsive feeding interactions, engage in developmentally appropriate sensory food experiences, promote skill development, and support mealtime participation. Both intervention and control‐group parents were offered the opportunity to participate in a weekly virtual parent support group led by an SLP not involved with the assessment team. All existing community therapies were continued. Children were assessed in the home at enrollment and virtually every 3 months while in the 1‐year intervention using clinical assessment and validated survey tools which included The Children's Eating and Drinking Activity Scale (CEDAS) and The

Pediatric Eating Assessment Tool (PediEAT), which is intended to assess observable symptoms of problematic feeding in children.

**Results:** Recruitment and participation are ongoing. Sixteen children enrolled in the study and were randomized into equal groups. Half are male, 43.8% Non‐Hispanic Black, 37.5% Hispanic, and 18.8% Non‐Hispanic White. The mean(range) age of children at enrollment was 24.4(16.6‐35.8) months. Their median(range) National Area Deprivation Index was 52(10‐92). Three‐fourths of the cohort (n=12) were CEDAS level 2 at enrollment, meaning they used the FT use for all nutrition and hydration and had oral intake for experience and/or pleasure only. One fourth (n=4) were CEDAS level 3 meaning oral intake partially met nutritional and/or hydration needs. CEDAS levels were equal between control and intervention groups. On the PediEAT, 9(56%) of children had concerning physiologic symptoms, 4(25%) had concerning problematic mealtime behaviors, 7(44%) had concerning selective or restrictive eating, and 2(13%) had concerning oral processing skills. Of those who have completed 6‐month follow‐up (n=12), 2 of 7 children in the treatment group transitioned to all calories taken by mouth; 7 of 7 had reduced tube feeding volumes and increased oral experiences. No children in the control group had weaned from tube feeding and 4 of 5 had increased tube feeding volumes as recommended by their existing care teams.

**Conclusions/Significance:** Our study is early and ongoing and therefore broad conclusions are premature before the full cohort and final outcomes are collected. However, our intervention shows that children with FTs and tracheostomies have complex feeding disorders which likely require interdisciplinary approaches to address. Our initial cohort demonstrates promise of acceptability and retention of children with FTs and tracheostomies in a virtual intervention. Early findings suggest this model may potentially have success over traditional approaches for weaning FTs and increasing oral feeding in a medically complex and hard‐to‐reach population.

## 869 FEEDING SUPPORT GROUPS FOR CHILDREN WITH FEEDING TUBES AND TRACHEOSTOMIES

*Karen Dilfer*, *Carolyn Jaynes*, *Sarah Sobotka*

*Pediatrics*, *The University of Chicago Division of the Biological Sciences*, *Chicago*, *IL*

**Background and Objectives:** Long‐term feeding tube (FTs) use has negative impacts on parental stress and family well‐being. Parents report profound anxiety around mealtimes and guilt related to their children's eating struggles. Parents of children with FTs as compared to other chronic diseases report higher stress and twice as much hands‐on caregiving. Parents of children with FTs and tracheostomies also describe feeling socially isolated and report gaps in feeding therapies. Expert‐facilitated virtual feeding support groups have the potential to effectively offer education and training while additionally providing therapist and peer support to isolated families. Our study objective was to evaluate the feasibility, acceptability, and preliminary impacts of a pilot feeding support group program for children with FTs and tracheostomies.

**Study Participants and Setting:** Virtual feeding support groups were a weekly resource offered to all participants in an ongoing randomized trial to improve oral feeding for children with FTs and tracheostomies. Children were eligible if less than 3 years of age with a FT, tracheostomy, and assessed as safe to advance oral feeding. IRB approval and consent were obtained. This is a secondary analysis of the feeding support groups. The overall study is ongoing.

**Methods:** Weekly virtual parent support groups were facilitated by a Speech‐Language Pathologist (SLP) with feeding expertise and offered to all participants enrolled in a virtual feeding therapy trial. Participant attendance was tracked to measure acceptability. Session topics were parent‐directed with parents initiating conversation topics before steering to a predetermined topic if time allowed. The SLP took notes describing content discussed and participants were surveyed to provide feedback. Participant and SLP responses were compiled and analyzed for main themes by study investigators.

**Results:** Sixteen parents were invited to groups and 12 (75%) participated in at least one or more groups during their observed study period. The majority (11/92%) were mothers. Six participants (50%) identified as Black, 3(25%) as Hispanic, 2(13%) as Non‐Hispanic White, and 1(8%) as American Indian/Alaskan Native. Their median(range) National Area Deprivation Index was 52.75 (10‐84). For those that engaged, median participation was 50% of sessions. Common support group topics included: sharing parent and child experiences, strategies to build oral interest thru exploring sensory components of food, family routines for mealtime, troubleshooting common challenges, introducing different formulas and blenderized foods via FT, and focused FT and tracheostomy education (e.g. one‐way valve and oral care). Parents also discussed how they navigated having multiple caregivers in the home and helping their child transition from Early Intervention to a school program. Parents expressed appreciation for hearing the similar lived experiences of other parents and ideas on how to work toward goals. One parent shared in written feedback: “we can all give each other suggestions on what worked for us that can maybe help with improvement of feeding.” Families often wanted future sessions to continue the prior discussion topics. One parent stated: “I would like to learn more…so we can keep help the kids progress at a smoother pace when it comes to eating food by mouth and start transitioning them into what comes next.”

**Conclusions/Significance:** In a pilot cohort of parents of children with FTs and tracheostomies who were enrolled in a virtual trial, the vast majority actively engaged with virtual support groups. Parent‐driven session topics focused on eating behaviors and feeding tube strategies, but also addressed unique challenges of living with a child with medical complexity and medical technology dependence. These sessions demonstrate the range of shared experiences for parents of children with FTs and tracheostomies which may benefit from a virtual support model. Parent feedback was positive; they valued hearing others’ stories and learning new ideas to support oral feeding. Future studies ought to explore the impacts of virtual group interventions on individual feeding outcomes in order to determine their potential effectiveness as a therapeutic intervention. Given the practicalities of scarce resources for feeding tube weaning and feeding therapy for children with medical complexity who struggle to travel away from home with medical technologies, virtual parent groups may have the potential to provide support and increased connection for this vulnerable and often isolated population.

## 870 EFFICACY OF LANSOPRAZOLE ORAL DISINTEGRATING TABLETS IN THE TREATMENT OF PEDIATRIC GASTROESOPHAGEAL REFLUX DISEASE: A DOSE‐RESPONSE STUDY

*Eru Sujakhu* ^*1*^, *David Gremse* ^*2*^

^*1*^ *University of South Alabama*, *Mobile*, *AL*; ^*2*^ *University of South Alabama*, *Mobile*, *AL*

**Background:** Gastroesophageal reflux disease (GERD) is a prevalent condition in the pediatric population affecting almost 26.9% of children aged 1‐5 years, characterized by the retrograde flow of gastric contents into the esophagus, leading to discomfort and various complications. Lansoprazole is one of the proton pump inhibitors (PPIs), that are licensed for use in the treatment of GERD in children. The standard recommended daily dose is 15 mg for children weighing less than 30 kg and 30 mg for children weighing more than 30 kg. Lansoprazole oral disintegrating tablets (ODT) are a useful formulation for young children who cannot swallow pills. This study aims to assess the dose response of Lansoprazole ODT, comparing weight‐based dosing of a low versus high dose in alleviating GERD symptoms in children aged 1 to 5 years.

**Methods:** This is a single‐center, randomized, multi‐dose treatment study conducted over 10 weeks, involving 8 children between 1 and 5 years of age diagnosed with GERD. Participants were randomly assigned to two groups: one group receiving a low dose, i.e., 0.75 mg/kg/day (Group 1), and the other receiving a high dose i.e. 2.5 mg/kg/day (Group 2) of Lansoprazole ODT once daily for 8 weeks. PPI therapy was discontinued after 8 weeks, then a final symptom assessment was obtained 2 weeks after PPI withdrawal. The primary endpoint was improvement in GERD symptoms, measured using the Gastroesophageal Reflux Symptom Questionnaire (GASQ).

**Results:** Treatment outcomes were similar between the groups after 4 weeks, with p‐values of 0.08 and 0.6 for the low and high doses of Lansoprazole ODT, respectively. This outcome aligns with previous studies indicating that up to 50% of children with erosive esophagitis may continue to experience abdominal pain despite four weeks of Lansoprazole therapy. Notably, the symptom scores in the low‐dose group were similar at 4 and 8 weeks of treatment. In contrast, the high‐dose group receiving 2.5 mg/kg/day exhibited a significant reduction in symptom scores between weeks 4 and 8 (p = 0.042), achieving the lowest recorded GASQ scores. While there was a slight rebound in symptoms by week 10 in both treatment groups, the scores remained significantly lower than baseline, suggesting a sustained therapeutic benefit. Analysis of standard deviations further revealed that the higher dose led to greater symptom relief with more consistent outcomes across participants, underscoring its reliability and effectiveness.

**Conclusion:** Although early treatment outcomes did not show statistical significance, the 2.5 mg/kg/day dose of Lansoprazole ODT demonstrated a more significant improvement in symptom scores after 8 weeks of treatment than patients who received a dose of 0.75 mg/kg/day. These findings suggest that a higher dose regimen may be more effective in managing symptoms of erosive esophagitis in pediatric patients, providing both greater relief and consistency in therapeutic response. These results support further exploration of indications for the use of higher doses and the safety of lansoprazole ODT in treating GERD in young children.


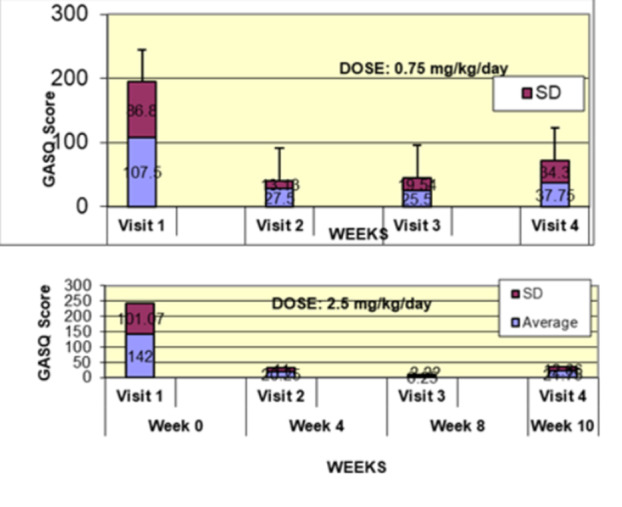



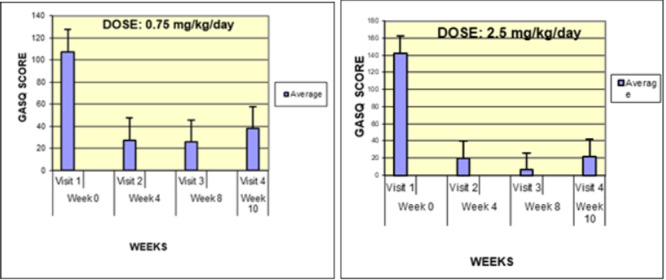


## 871 SAFETY AND TOLERABILITY OF TENAPANOR IN PEDIATRIC PATIENTS WITH IRRITABLE BOWEL SYNDROME WITH CONSTIPATION: AN ANALYSIS OF BLINDED SAFETY DATA FROM A PHASE 3 STUDY AND ITS OPEN‐LABEL EXTENSION

*Nastassja Williams* ^*1*^, *Thomas Wallach* ^*2*^, *Mihaela Ringheanu* ^*3*^, *Ana Roig Cantisano* ^*4*^, *Yang Yang* ^*1*^, *Karishma Raju* ^*1*^, *Jocelyn Tabora* ^*1*^, *Susan Edelstein* ^*5*^

^*1*^ *Ardelyx, Inc*., *Newark*, *CA*; ^*2*^ *SUNY Downstate Health Sciences University*, *New York*, *NY*; ^*3*^ *Texas Digestive Specialists*, *Harlingen*, *TX*; ^*4*^ *Florida Pharmaceutical Research & Associates*, *Miami*, *FL*; ^*5*^ *Ardelyx, Inc*., *Waltham*, *MA*

**Background:** Irritable bowel syndrome (IBS) is a gastrointestinal disorder that affects approximately 5% of pediatric patients (pts) in the United States, with IBS with constipation (IBS‐C) being the most common subtype. Tenapanor (TEN) is a first‐in‐class, minimally absorbed inhibitor of intestinal sodium/hydrogen exchanger isoform 3 approved for the treatment of IBS‐C in adults. The phase 3 R ALLY study is underway to assess the efficacy, safety, and tolerability of TEN in pediatric pts aged ≥12 and <18 years. Pts who complete the 12‐week R‐ALLY randomized treatment period (RTP) may be able to enter a 40‐week open‐label safety extension (OLE) study. We present preliminary, blinded, interim safety data from R‐ALLY and its OLE.

**Methods:** R‐ALLY is a randomized, double‐blind, placebo‐controlled study in pts (aged ≥12 to <18 years) meeting the Rome IV criteria for child/adolescent diagnosis of IBS‐C. After a 2‐week screening period, eligible pts were randomized 1:1:1 to receive TEN low dose (25 mg twice daily [bid]), TEN high dose (50 mg bid), or matching placebo bid for 12 weeks. During the 12‐week RTP, pts are assessed on site every 2 or 4 weeks for safety. Adverse events (AEs) and concomitant medications are recorded. Pts who complete the 12‐week RTP may enter a 40‐week OLE study (TEN‐01‐306), where AE assessments are performed every 4 weeks and pts will return approximately every 6 weeks for safety data collection.

**Results:** As of November 1, 2024, 143 pts were screened, 77 randomized, 58 completed the RTP, and 56 completers entered the OLE. There were 10 early dropouts from the RTP. **Table 1** summarizes baseline demographics and treatment‐emergent AEs (TEAEs) for the safety analysis set (SAS), which included 77 randomized and treated pts from the RTP. In the RTP, no serious TEAEs were reported, and all TEAEs were resolved or resolving and considered unrelated to study drug except diarrhea. Of the 56 completers from R‐ALLY who entered the OLE, 26 completed the 40‐week study as of November 1, 2024, with 6 early dropouts. **Table 2** summarizes baseline demographics and TEAEs for the 56 enrolled and treated pts (SAS) from the OLE. In the OLE, no serious TEAEs were reported, all TEAEs were considered unrelated to study drug except diarrhea, and all TEAEs were resolved except 2 mild events: 1 overflow incontinence and 1 diarrhea.

**Conclusion:** Tenapanor's efficacy and safety are currently being investigated in the R‐ALLY study in pediatric patients with IBS‐C. Preliminary safety results of this blinded study and its open‐label safety extension are consistent with those from previous studies of tenapanor in adult patients with IBS‐C. Diarrhea was the only adverse event related to study drug, which is consistent with tenapanor's mechanism of action. To date, no unexpected safety concerns have been observed.


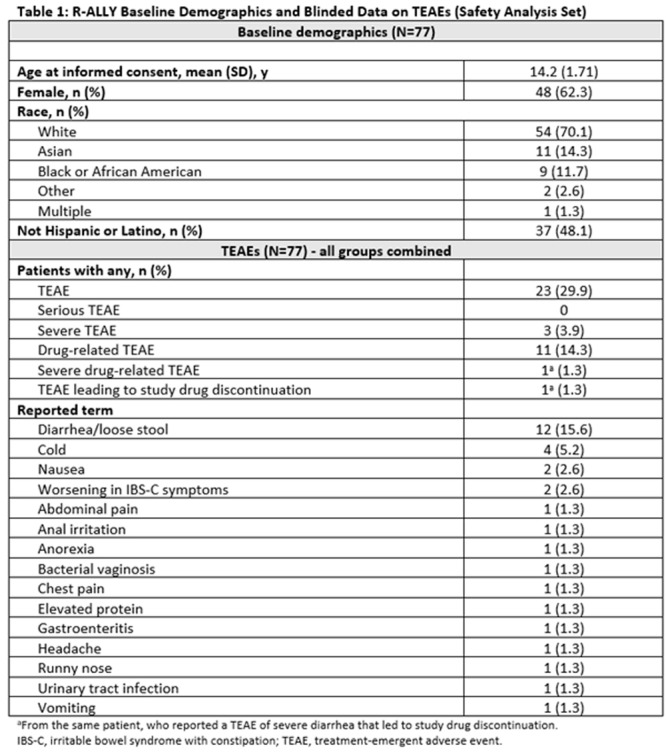



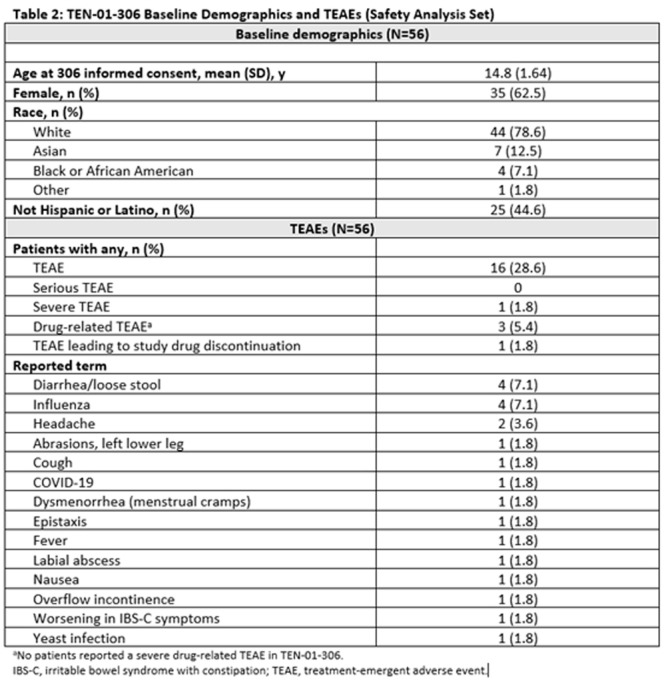


## 872 POSITIVE PSYCHOLOGICAL FACTORS AND PAIN IN PEDIATRIC INFLAMMATORY BOWEL DISEASE: EXPLORING THE POTENTIAL PROTECTIVE ROLE OF MINDFULNESS, ACCEPTANCE, AND QUALITY OF LIFE

*Ashley Dunn* ^*1,2*^, *Javier Lopez Rivera* ^*1,2*^, *Hannah Boyd* ^*1,2*^, *Nicole Neiman* ^*3*^, *Anava Wren* ^*1,2*^

^*1*^ *Pediatrics Gastroenterology, Hepatology, and Nutrition*, *Stanford University School of Medicine*, *Stanford*, *CA*; ^*2*^ *Stanford Medicine Children's Health Center for IBD and Celiac Disease*, *Stanford Medicine*, *Stanford*, *CA*; ^*3*^ *Lincoln Memorial University*, *Harrogate*, *TN*

**Background:** Pain is a prevalent and complex symptom in pediatric inflammatory bowel disease (IBD), frequently persisting despite clinical remission and contributing to worse psychosocial and health outcomes. While the detrimental effects of psychological distress—such as depression, anxiety, and stress—on pain are well‐established, the protective role of positive psychological factors remains underexplored. Preliminary research has shown associations between positive psychological variables (e.g., mindfulness and disease acceptance) and improved pain and health outcomes in pediatric populations. Exploring the relationship between positive psychological variables and key IBD outcomes such as pain may offer valuable insights into how these positive constructs support health and resilience in pediatric IBD populations.

**Aim:** This study aimed to (1) examine associations between positive psychological variables (e.g., mindfulness, disease acceptance, global health‐quality of life (GH‐QoL)) and pain outcomes (intensity, interference), and (2) explore the extent to which these positive psychological factors predict the likelihood of reporting pain, pain intensity, and pain interference after adjusting for demographic and disease‐related covariates.

**Methods:** A cross‐sectional analysis was conducted among pediatric patients (N = 149) with a confirmed diagnosis of IBD, including Crohn's disease, ulcerative colitis, and IBD‐unclassified (mean age = 18 ± 2.8 years; 58% female; 51% Crohn's disease; 63% White, 13% Hispanic, 17% Asian, 7% Other). Participants completed the validated PROMIS Pediatric Profile‐25, including fatigue, pain interference and a single item measuring pain intensity. Positive psychological constructs included mindfulness (Children's Acceptance and Mindfulness Measure), disease‐specific acceptance (IBD‐Acceptance), and global health‐related quality of life (PROMIS Pediatric Global Health‐7; GH‐QoL). Disease severity was evaluated using the Pediatric Ulcerative Colitis Activity Index (PUCAI) and the Crohn's Disease Activity Index (CDAI). Pearson correlations were used to examine associations between psychological measures and pain variables. Logistic regression modeled the binary outcome of reported pain in the past two weeks (yes/no) and linear regression modeled pain intensity and pain interference; adjusting for demographic factors (sex, race, IBD diagnosis) and disease severity with separate models testing each positive psychological variable independently.

**Results:** Among the 149 participants with IBD, 80 (54%) reported experiencing pain within the past two weeks. Correlational analyses revealed that mindfulness was negatively associated with pain interference (r = –.25, p < .05). Similarly, IBD acceptance was negatively correlated with pain intensity (r = –.38, p < .01) and pain interference (r = –.28, p < .05). Lastly, GH‐QoL was negatively correlated with pain intensity (r = –.34, p < .01) and pain interference (r = –.43, p < .001). Regression models showed mindfulness was significantly associated with reduced odds of reporting pain (β = –.0610, FDR= .062), decreased pain intensity (β = ‐.0367, FDR = .083), and decreased pain interference (β = ‐.2051, FDR = .001). IBD acceptance was significantly associated with a decrease in pain interference (β = ‐.0564, FDR = .0838). GH‐QoL was significantly associated with reduced odds of reporting pain (β = –.0765, FDR = .017), decreased pain intensity (β = ‐.0609, FDR= .001), and decreased pain interference (β = ‐.2307, FDR = .0003).

**Discussion:** This study examined whether important positive psychological variables are associated with pain in pediatric IBD. Mindfulness, disease acceptance, and GH‐QoL were all inversely correlated with pain. Further, increases in mindfulness and GH‐QoL predicted lower odds of reporting pain, decreased pain intensity, and decreased pain interference. These findings are particularly important because pain is a key symptom of IBD. Additionally, there is growing recognition that a subset of youth experience persistent pain even when their disease is in remission, and this pain is linked to poorer psychosocial and health outcomes. These preliminary findings support past research demonstrating the potential benefit of positive psychological variables on health outcomes in pediatric chronic illness populations. Future research should be conducted in larger and more diverse pediatric IBD cohorts exploring temporal relationships in longitudinal study designs. Another important future direction is the design of behavioral interventions that cultivate these psychological constructs to explore their potential improvements on health outcomes in pediatric IBD.

## 873 EPITHELIAL STEM CELL DEFECTS PRECEDE THE DEVELOPMENT OF HIRSCHSPRUNG ASSOCIATED ENTEROCOLITIS IN THE PIEBALD LETHAL MODEL OF HIRSCHSPRUNG DISEASE

*Virginia Chinn* ^*2*^, *Sophie Sax* ^*2*^, *Katherine Beigel* ^*2*^, *Benjamin Wilkens* ^*3*^, *Jessica Raab* ^*1*^, *Joshua Eisenberg* ^*2*^, *Robert Heuckeroth* ^*2*^, *Kathryn Hamilton* ^*2*^, *Naomi Tjaden* ^*2*^

^*1*^ *Gastroenterology*, *Children's Hospital of Philadelphia Pediatrics Residency Program*, *Philadelphia*, *PA*; ^*2*^ *Gastroenterology*, *The Children's Hospital of Philadelphia*, *Philadelphia*, *PA*; ^*3*^ *Pathology*, *The Children's Hospital of Philadelphia*, *Philadelphia*, *PA*

**Background and Aims:** Hirschsprung disease (HSCR) is a congenital disorder caused by the failure of enteric neural crest cells to colonize the distal bowel, resulting in aganglionosis (absence of the enteric nervous system). Absence of the enteric nervous system causes a functional obstruction characterized by symptoms including intractable constipation and risk of “Hirschsprung‐associated enterocolitis” (HAEC). When HAEC occurs, bacteria cross the protective epithelial barrier leading to potentially fatal sepsis. Primary HSCR treatment is surgical removal of aganglionic bowel, however postoperative complications occur in 40% of patients including HAEC. The existing data from mouse and human patient sample studies of HSCR point to abnormal goblet cell phenotypes. There are no studies examining the effects of aganglionosis on colonic epithelial stem cell fitness or differentiation. We hypothesize that epithelial colonoids derived from *Piebald sl/sl* (mutant phenotype) may exhibit abnormal stem cell function that may predispose to abnormal goblet cell differentiation.

**Methods:** *Piebald lethal sl/sl* mice and their *s/s* littermate controls were euthanized at P20. Colonic epithelial morphology (colon length, crypt length) and enterocolitis scoring was assessed. Colonic mucus integrity was evaluated by ability to exclude microbes (visualized by 16S FISH). Immunofluorescence of Ki67, TUNEL, and Muc2 at P20 were conducted. Organoid formation efficiency, stem cell and goblet cell gene expression were measured in tissue‐derived colonoids generated from colon epithelium of *Piebald lethal* mice at P20. These were compared to bulk RNA sequencing of colonic epithelium isolated from P20 *sl/sl* and *s/s* mice.

**Results:** Colon length, enterocolitis scores, and 16S localization did not differ between groups. Ki67+ cells in distal colon crypts were decreased in the *Piebald sl/sl* compared to *s/s (s/s* mean = 128 Ki67+/hpf, *sl/sl* mean = 68 Ki67+/hpf, P=<0.0001), however crypt depth was unchanged. Muc2 was unchanged. Neither group had TUNEL staining localized to the crypts. Organoid formation efficiency and Lgr5 expression was decreased in *sl/sl* in early organoid passages. OFE in *sl/*sl was 4.8% compared to the 14.5% of *s/s* controls, representing a 66% decrease (p =0.0009). Accordingly, Lgr5 expression was decreased in *sl/sl*, also by 66% (p<0.05). However, the phenotype reverted to wildtype in later passages. In early passages, goblet cell‐associated transcription factor Atoh1 was increased (p<0.05) by a factor of two in the *sl/sl*. An additional transcription factor, Spdef also appeared to be increased in *sl/sl*. Muc2 expression also appeared to be increased in *sl/sl* vs *s/s*. Bulk RNA seq showed significant differential gene expression in inflammatory cytokines in *sl/sl* compared to *s/s*, notably a 5‐fold increase in expression of various mast cell proteases and Ang4. We confirmed Ang4 expression was increased in *sl/sl* organoids.

**Conclusions:** There was a pre‐existing knowledge gap about colonic epithelial stem cell characteristics in Hirschsprung disease. This study identifies a novel stem cell defect in a mouse model of HSCR and posits a potential mechanism. Normal mucus phenotyping prior to onset of megacolon or enterocolitis features (P20), with early organoid passage decreased stem cell fitness, and early presence of inflammatory transciptome signature in RNA sequencing indicate that epithelial stem cell defects may underlie HAEC pathogenesis. Loss of significant differences between *s/s* and *sl/sl* stem cell phenotype suggests there is an *in‐situ* co‐driver of this pathology (such as regional neuron abundance, microbes, blood flow or other non‐epithelial compartment contributors). Absence of early mucus abnormalities suggest that early defects in mucus formation are not the driving HAEC development in *Piebald lethal* HAEC. However the products of goblet cells remain of interest‐‐notably the high expression of Ang4 at P20. Ang4 is produced by murine goblet cells during infection. At high concentrations, it can impact Lgr5 stem cell survival by promoting apoptosis. Future directions include investigating whether changes in Ang4 expression in late passages correspond with changes in OFE and Lgr5 expression.

## 874 UNRAVELING THE MECHANISM BEHIND COW'S MILK PROTEIN ALLERGY/FOOD PROTEIN‐INDUCED ALLERGIC PROCTOCOLITIS IN A LARGE PROSPECTIVE OBSERVATIONAL COHORT STUDY

*Timothy Sun* ^*1*^, *Chen Goldstein* ^*3*^, *Itamar Lavy* ^*3*^, *Yamini Virkud* ^*4*^, *Wayne Shreffler* ^*2*^, *Qian Yuan* ^*1*^, *Moran Yassour* ^*3*^, *Victoria Martin* ^*1*^

^*1*^ *Pediatric Gastroenterology and Nutrition*, *Massachusetts General Hospital*, *Cambridge*, *MA*; ^*2*^ *Pediatric Allergy*, *Mass General Brigham Inc*, *Boston*, *MA*; ^*3*^ *Microbiology and Molecular Genetics*, *The Hebrew University of Jerusalem*, *Jerusalem*, *Jerusalem District*, *Israel*; ^*4*^ *Pediatric Allergy*, *University of North Carolina*, *Chapel Hill*, *NC*

**INTRODUCTION:** Food protein‐induced allergic proctocolitis (FPIAP) (also referred to as cow's milk protein allergy) is an early, common manifestation of gastrointestinal food allergy, yet the underlying pathophysiology is poorly understood. It is characterized as a non‐IgE‐mediated process yet is associated with a two‐fold increase in IgE‐mediated food allergies later in childhood. Using a large prospective observational cohort study (the GMAP study), we aim to uncover the pathophysiologic mechanisms underlying FPIAP. We previously reported dysbiosis in infants with FPIAP at the family level characterized by enrichment of *Enterobacteriaceae* prior to and during symptom onset. Here we utilized shotgun metagenomic sequencing to identify strain level microbial differences, examine functional microbial pathways, and investigate the microbial genomes for mechanistic insights into the pathogenesis of FPIAP.

**METHODS:** We prospectively identified 84 infants with a clinical diagnosis of FPIAP (including blood in the stool), and 79 infants without, from the observational GMAP cohort study. Infants were matched by age and sampling density, and each contributed a median of four longitudinal stool samples during the first year of life. 740 stool samples were analyzed here. DNA was extracted (Qiagen DNeasy PowerSoil HTP 96 Kit), bead‐beaten on a TissueLyser II to ensure lysis of diverse microbial cell types, then we performed deep, paired‐end metagenomic sequencing (median 14~ million paired reads per sample). Microbial taxonomic profiling was performed with MetaPhlAn 4, metagenomic assembled genomes (MAGs) were created using Metaspades, minimap2, MetaBat2. Relevant genes were analyzed using BioCyc databases and Roary. We used HUMAnN 3.0 with MetaPhlAn 4‐generated taxonomic profiles to improve mapping specificity and functional profiling utilized the UniRef90 database, and pathway abundances were normalized to relative abundance using HUMAnN's “renorm” utility. Downstream statistical analysis was performed with MaAsLin2, using the same covariates as in taxonomic models. Significance thresholds were set at p < 0.05 and q < 0.25.

**RESULTS:** In examining the earliest age window (0–2 months), we observed a clear enrichment of *Escherichia* in infants with AP (coefficient = 0.136; p = 0.016; q = 0.068), driven principally by *Escherichia coli* (coefficient = 0.168; p = 0.005; q = 0.035). *E. coli* strain clustering based on both marker gene phylogenies and MAGs indicated the existence of FPIAP‐enriched lineages. Notably, E. coli MAGs from infants with FPIAP were more similar to each other than to those from control infants, and these strain‐level distinctions were supported by gene content differences. The most frequent function in multiple enrichment tests, different in children with FPIAP, were those related to cell adhesion. Functional pathway analysis revealed consistent alterations in microbial fermentation processes associated with FPIAP. The propane‐1,2‐diol degradation pathway was enriched in infants with FPIAP across early and resolved stages (coef = 0.011, p = 0.001, q = 0.003), indicating shifts in microbial substrate utilization. Succinate fermentation to butanoate, an important step in butyrate production, was modestly but repeatedly elevated in FPIAP infants (coef = 0.010, p = 0.004, q = 0.022), suggesting altered short‐chain fatty acid metabolism. Conversely, the acetyl‐CoA to butyrate fermentation pathway showed reduced abundance in FPIAP (coef = ‐0.008, p = 0.005, q = 0.020).

**CONCLUSION:** Our findings reveal novel taxonomic and functional differences in the microbiome of infants with FPIAP, some of which are detectable prior to symptom onset suggesting an underlying microbial signature that is not solely a consequence of inflammation. Enrichment of *E. coli*, specifically strains with gene enrichment affecting cell adhesion, were identified in infants with FPIAP. Altered short‐chain fatty acid metabolism were also identified, including butyrate and succinate pathways, aligning with prior evidence in infants with IgE‐mediated milk allergy but this has not previously been examined in FPIAP. This underscores a possible shared pathophysiology between FPIAP and IgE‐mediated food allergies which has important clinical implications and novel opportunities for food allergy prevention. Connecting these intestinal microbiome findings to immune cell phenotypes in the periphery of the infants with FPIAP in this cohort to further delineate this mechanistic pathway is ongoing.

## 875 ANESTHESIA‐FREE TRANSORAL ENDOSCOPY USING A MODIFIED PACIFIER IN EARLY INFANCY

*Jonathan Berken* ^*1,2*^, *Zoe Rosoff‐verbit* ^*1*^, *Tyler Babinski* ^*1*^, *Elizabeth Silvestro* ^*3*^, *Michael Manfredi* ^*1*^, *Matthew Ryan* ^*1*^

^*1*^ *Pediatric Gastroenterology, Hepatology, and Nutrition*, *The Children's Hospital of Philadelphia*, *Philadelphia*, *PA*; ^*2*^ *Neonatology*, *The Children's Hospital of Philadelphia*, *Philadelphia*, *PA*; ^*3*^ *The Children's Hospital of Philadelphia*, *Philadelphia*, *PA*

**Background:** Neonates and infants frequently require esophagogastroduodenoscopy (EGD) for diagnostic evaluation of upper gastrointestinal disorders. Standard EGD typically involves general anesthesia, which increases procedural time, costs, and potential neurodevelopmental risk. An anesthesia‐free alternative would represent a significant advance in infant endoscopic care.

**Objective:** To evaluate the feasibility of a novel anesthesia‐free transpacifier endoscopy (TPE) technique in neonates and infants ≤6 months of age. Primary outcomes included procedural success, biopsy adequacy, physiologic stability, and caregiver and physician attitudes toward TPE compared to standard EGD.

**Methods:** Infants ≤6 months old with an intact suck reflex, receiving inpatient or outpatient gastroenterology care at the Children's Hospital of Philadelphia (CHOP), were eligible if clinically indicated for endoscopic evaluation. Exclusion criteria included mechanical ventilation, epilepsy, hemodynamic instability, suspected gastrointestinal bleeding, or a need for therapeutic EGD. Participants were identified through medical record review and gastroenterologist referral.

TPE was performed using a 3.1 mm bronchoscope introduced through a custom 3D‐printed pacifier flavored with Sweet‐ease (sucrose‐water solution) for nonpharmacologic analgesia. The pacifier was secured without anesthesia. Vital signs were continuously monitored. Adverse events, procedural outcomes, and attitudes from caregivers and endoscopists were collected.

**Results:** To date, TPE has been successfully performed in 6 infants. All procedures were completed without the need for conversion to sedated endoscopy or early termination. Infants remained hemodynamically stable, with no clinically significant fluctuations in vital signs or serious adverse events. Visualization was optimal in most cases, with the exception of one procedure in which secretions limited mucosal assessment. Esophageal and gastric biopsies were consistently adequate for histopathologic interpretation; notably, rare intraepithelial eosinophils were identified in 2 cases. In one infant, treatment with bethanechol was initiated following the procedure. Gastroenterologists reported high satisfaction with both endoscopic visualization and tissue yield. Caregivers uniformly expressed positive attitudes, particularly in appreciation of the anesthesia‐free approach.

**Discussion:** These preliminary findings support the feasibility and tolerability of unsedated TPE in infants. The technique consistently yielded diagnostically adequate biopsies, was well‐tolerated without pharmacologic analgesia or sedation, and received positive feedback from both caregivers and clinicians. TPE may represent a safe, cost‐effective, and developmentally appropriate alternative to conventional sedated EGD in early infancy. Ongoing enrollment aims to expand the cohort to further assess safety, reproducibility, clinical utility, and broaden potential indications.

## 876 TRENDS IN INPATIENT HEALTHCARE UTILIZATION IN CHILDREN WITH FEEDING DIFFICULTIES

*Suzanna Hirsch*, *Enju Liu*, *Rachel Rosen*

*Gastroenterology*, *Boston Children''s Hospital*, *Boston*, *MA*

**Background:** Feeding difficulties affect 5‐20% of children and are associated with morbidity and decreased quality of life. Although the patient impact is significant, no prior studies have evaluated resource utilization in children with symptoms severe enough to merit hospitalization. The aims of this study were: 1) to evaluate national trends in admissions, length of stay (LOS), and charges for children with feeding difficulties and 2) to compare resource utilization in children with and without feeding difficulties.

**Methods:** This was a cross‐sectional analysis of non‐birth hospitalizations for children < 21 years in the Kids’ Inpatient Database (KID) from 2009 to 2022. Children with feeding difficulties were identified based on ICD codes. Data were weighted for population estimates. Outcomes included the proportion of hospitalizations associated with feeding difficulties, mean LOS, and mean hospital charges in 2022 dollars. Covariates included age, gender, race, complex chronic conditions (CCC), insurance status, income quartile, and hospital characteristics (region, location/teaching status, control/ownership, and free‐standing children's hospital status). Trends and group comparisons were analyzed using multivariable linear regression.

**Results:** We identified 462,381 hospitalizations for children with feeding difficulties. The mean age was 4.65 years with 71% from 0‐6 years, 11% from 7‐12 years, and 17% from 13‐21 years. Hospitalizations in children with feeding difficulties increased from 75,389 in 2009 to 95,844 in 2022, and the weighted proportion of hospitalizations associated with feeding difficulties increased from 3.4% to 6.1% (Figure 1). Mean LOS in children with feeding difficulties increased by 4 days (10.49 in 2009 vs. 14.52 days in 2022), and mean charges nearly doubled ($98,223 in 2009 vs. $192,287 in 2022). The effect of time on both LOS and charges was significant in multivariable linear regression (*P*<0.001). When comparing hospitalizations for children with and without feeding difficulties, LOS and charges were 3 times higher in children with feeding difficulties (Figure 2). There were baseline group differences, including in complexity with 64.0% of the feeding difficulty group having > 1 CCC vs. 29.6% in the comparator group (*P*<0.001 on chi square); however, the effect of feeding difficulties on LOS and charges remained significant after adjusting for potential confounders (*P*<0.001).

**Discussion:** Children with feeding difficulties accounted for an increasing proportion of hospitalizations, bed days, and charges over this 10‐year period. LOS and charges were 3 times higher in children with feeding difficulties, with group differences still seen after controlling for patient and hospital characteristics. These findings highlight feeding difficulties as a growing and costly condition, and future research should include efforts to reduce inpatient healthcare burden in this population.


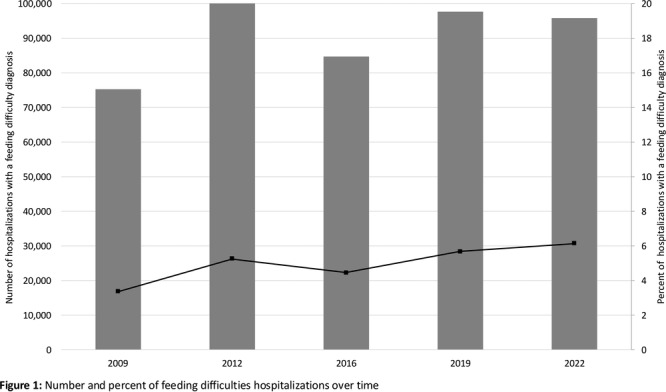


**Figure 1.** Number and percent of feeding difficulties hospitalizations over time


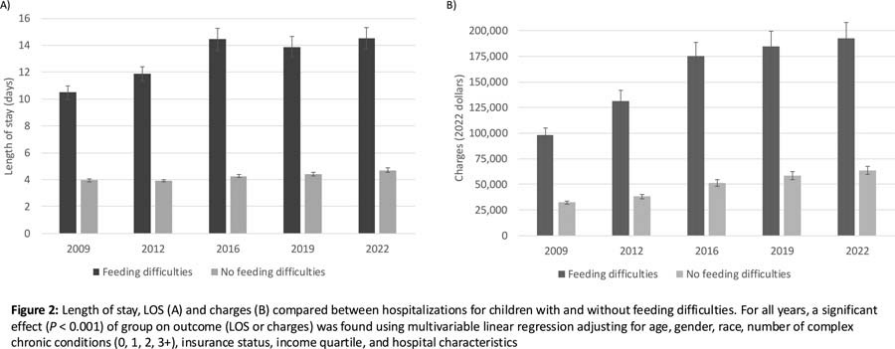


**Figure 2:** Length of stay, LOS (A) and charges (B) compared between hospitalizations for children with and without feeding difficulties. For all years, a significant effect (*P* < 0.001) of group on outcome (LOS or charges) was found using multivariable linear regression adjusting for age, gender, race, number of complex chronic conditions (0, 1, 2, 3+), insurance status, income quartile, and hospital characteristics

## 877 CLINICAL TRIAL OF WHOLE‐FAT BOVINE DAIRY TO LOWER HEPATIC STEATOSIS IN CHILDREN WITH METABOLIC DYSFUNCTION‐ASSOCIATED STEATOTIC LIVER DISEASE

*Megan Roach* ^*2*^, *Karenina Valdez* ^*1*^, *Jaret Skonieczny* ^*1*^, *Claude Sirlin* ^*4*^, *Jake Weeks* ^*4*^, *Lael Ceriani* ^*4*^, *Kimberly Newton* ^*1,3*^, *Nidhi Goyal* ^*1,3*^, *Jeffrey Schwimmer* ^*1,3*^

^*1*^ *Pediatrics*, *University of California San Diego*, *La Jolla*, *CA*; ^*2*^ *University of California San Diego*, *La Jolla*, *CA*; ^*3*^ *Rady Children's Hospital‐San Diego*, *San Diego*, *CA*; ^*4*^ *Radiology*, *University of California San Diego*, *La Jolla*, *CA*

**Background:** Metabolic dysfunction–associated steatotic liver disease (MASLD) affects roughly 10% of children and up to one‐quarter of those with obesity, yet optimal dietary management remains undefined. Lifestyle modification, particularly dietary change, is the cornerstone of MASLD therapy, but nutritional guidance among U.S. pediatric gastroenterologists varies widely. Existing pediatric obesity and liver‐disease guidelines offer few MASLD‐specific recommendations, reflecting a paucity of robust evidence. Emerging data suggest whole‐fat dairy may confer metabolic and hepatic benefits in this population, yet expert societies continue to advise low‐fat or nonfat dairy after age two. To address this gap, we conducted a 24‐week clinical trial testing our hypothesis that incorporating 2.5 servings of whole dairy daily reduces liver fat in children with MASLD.

**Methods:** Children (10–17 y) with biopsy‐confirmed MASLD, ALT ≥ 40 U/L, and hepatic steatosis ≥ 8% on magnetic resonance imaging–proton density fat fraction (MRI‐PDFF) completed a 24‐week, within‐participant trial comprising two distinct phases: a 12‐week observational period during which participants maintained their usual diet, followed by a 12‐week intervention involving incorporation of a minimum of 2.5 daily servings of whole‐fat dairy (milk and/or yogurt). This design allowed each participant to serve as their own control, reducing confounding from inter‐individual variability. Liver fat was quantified by MRI‐PDFF at weeks 0, 12, and 24; anthropometrics and fasting laboratory tests (ALT, AST, GGT, lipids, glucose, insulin, plasma fatty acids) were collected concurrently. The primary endpoint was the within‐participant difference in liver fat change during the intervention versus observational period. Paired t‐tests and mixed‐effects models (participant as random intercept, adjusted for baseline) evaluated absolute and relative changes; secondary outcomes were analyzed similarly. Two‐sided p < 0.05 denoted statistical significance.

**Results:** There were a total of 20 participants enrolled who all completed the study and adhered to the protocol. The cohort had a median age of 14.4 years (interquartile range, 4.9), was 70% male, and had a median body mass index of 32.5 kg/m^2^. Baseline mean liver fat was 21% (SD 9.0). Following incorporation of ≥ 2.5 daily servings of whole‐fat dairy, MRI‐PDFF liver fat significantly declined, with a mean absolute reduction of ‐5.6% (95% CI: ‐7.6 to ‐3.6; p < 0.001) (Figure 1) and a mean relative reduction of ‐29% (95% CI: ‐40% to ‐19%; p < 0.001). Triglycerides decreased from median 111 mg/dL to 99 mg/dL (p=0.0028), while total cholesterol and LDL‐C remained stable.

**Conclusions:** In this 24‐week clinical trial of children with MASLD, incorporating 2.5 servings/day of whole‐fat dairy produced an absolute 5.6 percentage‐point reduction and a relative 29% reduction in liver fat. This effect size aligns with ranges associated with clinically meaningful improvement. Liver fat remained stable during the observational phase, and the magnitude of change during the intervention suggests a beneficial role for whole‐fat dairy, though definitive causality requires larger, controlled studies. The intervention was well tolerated, with no adverse effects. As an accessible, familiar food group, whole‐fat dairy may offer a practical and safe strategy to reduce hepatic steatosis in pediatric MASLD. These proof‐of‐concept findings present a need for additional clinical trials and an examination of dietary guidelines for children with MASLD.


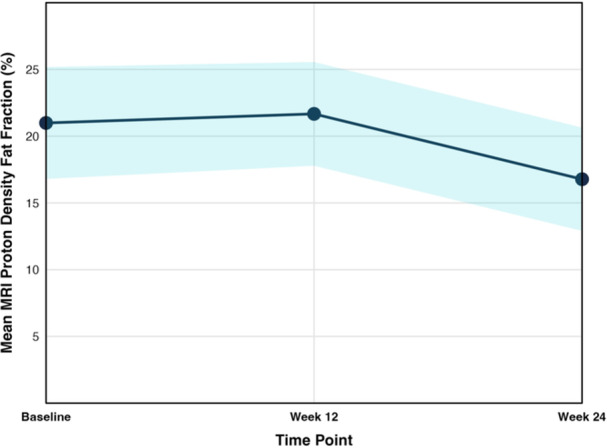


**Figure 1. Mean MRI‐PDFF Change from Baseline to Week 24 in Study Participants**

## 878 RELATIONSHIP OF DISTENSIBILITY TO INFLAMMATORY AND FIBROSTENOTIC OUTCOME METRICS OF SEVERITY IN PEDIATRIC EOSINOPHILIC ESOPHAGITIS

*Luis Lopez* ^*2*^, *Charlie Wang* ^*2*^, *Grant Edland* ^*2*^, *Michael White* ^*1,2*^, *Jose Peraza* ^*1*^, *Zeinab Dehghani* ^*1,2*^, *Sharon Tam* ^*1,2*^, *Peter Osgood* ^*1,2*^, *Barry Wershil* ^*1,2*^, *Natalie Hoffmann* ^*1,2*^, *Joshua Wechsler* ^*1,2*^

^*1*^ *Pediatrics*, *Ann & Robert H. Lurie Children's Hospital of Chicago*, *Chicago*, *IL*; ^*2*^ *Northwestern University Feinberg School of Medicine*, *Chicago*, *IL*

**Background:** Fibrostenosis is a complication of Eosinophilic Esophagitis (EoE) and often requires dilation, an indicator of severe disease in pediatric patients. Functional Lumen Imaging Probe (FLIP) can identify abnormalities in distensibility, which can be influenced by both inflammation and fibrosis. The Index for Severity of Eosinophilic Esophagitis (I‐SEE index) consists of symptom frequency, disease complications, diffuseness of disease, as well as inflammatory and fibrostenotic features found on endoscopy and histologic examination. The relationship of I‐SEE to distensibility and need for dilation in pediatric EoE is poorly understood.

**Methods:** We conducted a retrospective review of 317 patients aged 4‐22 who underwent upper endoscopy and FLIP between 3/2021‐5/2025. EoE was defined as a history of esophageal symptoms and biopsies with 15 or more eosinophils per high‐power‐field (EOS/HPF). Patients with chromosomal or anatomic abnormalities, history of thoracic/abdominal surgery, non‐EoE inflammatory disease (eg. Celiac, IBD), or peptic stricture were excluded. Patient demographics, medical history, treatments, symptoms, endoscopic reference score (EREFS), FLIP data, peak EOS/HPF, basal zone hyperplasia (BZH) grade, and presence of lamina propria fibrosis (LPF) were collected. The narrowest FLIP diameter in the esophageal body at the distensibility plateau where further increases in volume did not yield increased diameter was obtained. I‐SEE was scored, and patients were categorized: None‐Mild (0‐6), Moderate (7‐14), and Severe (≥15). The association of diameter and I‐SEE category to endoscopic and histologic measures was assessed along with the correlation of I‐SEE to diameter. ROC was generated to determine diameter to predict severe I‐SEE and dilation. Spearman, Chi‐square, Kruskal‐Wallis, and ANCOVA (to control for age) were utilized for statistical assessment.

**Results:** 41 controls and 276 EoE patients were assessed. Diameter was highest in controls regardless of inflammatory or fibrostenotic severity. Diameter had a weak inverse correlation with peak eosinophil count and a moderate inverse correlation with inflammatory endoscopic references score (iEREFS: sum of edema/exudate/furrows). Diameter was significantly decreased with increasing BZH grade, presence of lamina propria (LP) fibrosis, stricture, and increasing ring score. Among EoE patients, 122 had Mild I‐SEE scores, 47 had Moderate scores and 111 had Severe scores. Patients with severe I‐SEE scores had reduced BMI Z‐scores while patients with moderate I‐SEE scores had the highest EOS/HPF, BZH grade, and iEREFS score. LPF was increasingly common with higher I‐SEE severity and ring score was significantly increased with increasingly severe I‐SEE category. 24% of patients with severe I‐SEE scores had strictures. Diameter significantly decreased with increasing I‐SEE severity [Fig 1 A]. I‐SEE scores had a moderate inverse correlation with diameter [Fig 1B]. Dilation was very common among patients with severe I‐SEE scores [Fig 1 C]. Receiver operating characteristics (ROC) identified diameter was highly predictive of dilation and severe I‐SEE (AUC 0.9, p<0.001) with an optimal threshold of 14.7 [Fig 1D].

**Conclusions:** Distensibility abnormalities reflect both inflammatory and fibrostenotic burden. EoE severity based on I‐SEE is inversely correlated with FLIP distensibility. A diameter threshold of 14.7 mm effectively predicts severe disease and the need for dilation. Taken together, this study highlights the clinical utility of FLIP in assessing pediatric EoE severity.


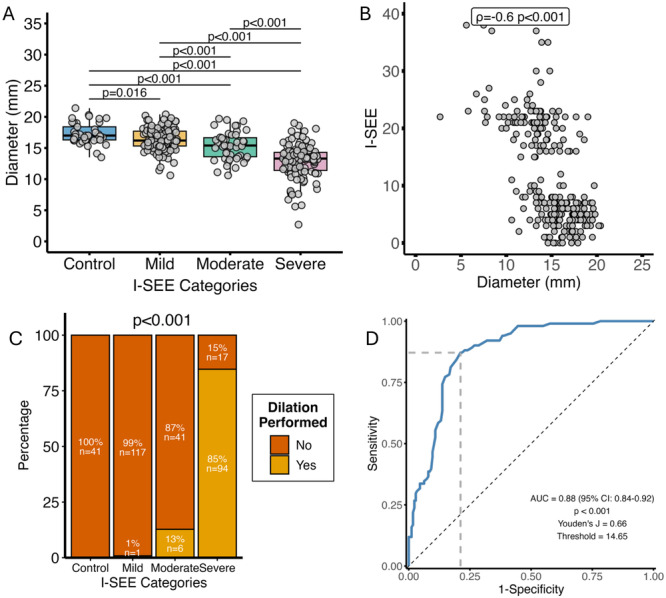


## 879 IMPROVEMENT IN DISTENSIBILITY IS GREATER WITH DUPILUMAB COMPARED TO OTHER TREATMENTS IN PEDIATRIC EOE INDEPENDENT OF DILATION

*Grant Edland* ^*2*^, *Michael White* ^*1,2*^, *Jose Peraza* ^*1*^, *Charlie Wang* ^*2*^, *Luis Lopez* ^*2*^, *Sharon Tam* ^*1*^, *Peter Osgood* ^*1*^, *Barry Wershil* ^*1,2*^, *Natalie Hoffmann* ^*1,2*^, *Joshua Wechsler* ^*1,2*^

^*1*^ *Pediatrics*, *Ann & Robert H. Lurie Children's Hospital of Chicago*, *Chicago*, *IL*; ^*2*^ *Northwestern University Feinberg School of Medicine*, *Chicago*, *IL*

**Introduction:** Multiple therapies can impact outcomes in Eosinophilic Esophagitis (EoE) including proton pump inhibitors (PPI), swallowed ‘topical’ corticosteroids (STC), diet elimination (Diet), dilation and Dupilumab. Fibrostenosis is the primary complication of EoE, and Functional Lumen Impedance Probe (FLIP) can identify abnormalities in esophageal diameter, which can be influenced by inflammation, fibrosis, and dilation. Longitudinal studies comparing the impact of treatment on esophageal diameter in pediatric EoE are lacking. We hypothesized that dupilumab is associated with greater improvement in diameter than non‐dupilumab treatment.

**Methods:** We performed a retrospective study of EoE patients who underwent FLIP twice at Lurie Children's Hospital between 8/2020‐5/2025. EoE was defined as a history of esophageal symptoms and esophageal biopsies with 15 or more eosinophils per high‐power‐field (EOS/HPF). Patient demographics, medical history, treatment, dilation history, endoscopic reference score (EREFS), FLIP data and peak EOS/HPF were collected. The narrowest FLIP diameter in the esophageal body at the distensibility plateau where further increases in volume did not yield an increase in diameter was obtained. Patients with <15 EOS/hpf at timepoint 1 were excluded along with history of autoimmune disease treated with a biologic (eg. Anti‐TNF). Wilcoxon Signed‐Rank test was used to assess paired differences between timepoints. ANCOVA was used to assess the magnitude (delta) of diameter change between timepoint 1 and 2 with control for potential confounders.

**Results:** 95 patients were included; At timepoint 1, treatments were PPI (53%), Diet (20%) and STC (38%), while at timepoint 2, treatments were PPI (43%), Diet (12%) and STC (26%), and dupilumab (49%). Dilation was performed in 60% of patients at timepoint 1. Dupilumab and non‐dupilumab groups had no differences in baseline diameter although dupilimab‐treated patients were older (14 vs 12) and more commonly had dilation (71% vs 49%) at timepoint 1. Between timepoint 1 and 2, both dupilumab and non‐dupilumab groups had a significant reduction in EOS/HPF and EREFS, however only the dupilumab group had a significant reduction in self‐reported dysphagia (Fig 1A‐C). Patients with dilation at timepoint 1 had a significant increase in diameter at timepoint 2 (Fig 1D). After controlling for age, treatment duration, and dilation/diameter at timepoint 1, we found dupilumab was associated with a greater increase in diameter than non‐dupilumab treatments (Fig 1E). Among patients who did not have dilation at timepoint 1, diameter significant increased only in the dupilumab group (Fig 1 F).

**Conclusions:** Dilation is associated with a significant longitudinal increase in esophageal diameter. Dupilumab is associated with a greater increase in distensibility in pediatric EoE compared to non‐dupilumab therapy independent of dilation and can be considered first‐line for fibrostenosis.


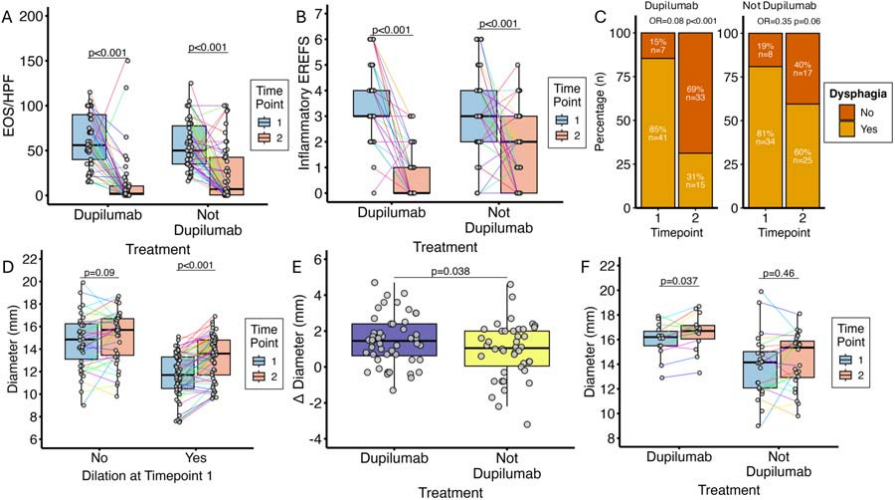


Figure 1: Impact of therapy on measures of inflammation and fibrostenosis in pediatric EoE. Both dupilumab and non‐dupilumab treatment improved A) EOS/HPF and B) Inflammatory EREFS (sum of edema/exudate/furrows). C) Only dupilumab improved presence of dysphagia. D) Diameter significantly improved with dilation. E) Magnitude of change in diameter between timepoints greater with dupilumab after control for baseline dilation/diameter, age and duration of treatment. F) Among pateints who did not have dilation at timepoint 1, only dupilumab was associated with increased diameter.

## 880 N‐ACETYLCYSTEINE MAY ATTENUATE FIBROSIS IN EOSINOPHILIC ESOPHAGITIS BY REDUCING FIBROBLAST ACTIVATION

*Matthew Buendia* ^*1*^, *Jennifer Pilat* ^*2*^, *Mitanshu Pandya* ^*2*^, *Anna Harris* ^*2*^, *Christopher Williams* ^*2,3*^, *Girish Hiremath* ^*1*^, *Yash Choksi* ^*2,3*^

^*1*^ *Pediatrics*, *Vanderbilt University Medical Center*, *Nashville*, *TN*; ^*2*^ *Medicine*, *Vanderbilt University Medical Center*, *Nashville*, *TN*; ^*3*^ *VA Tennessee Valley Healthcare System*, *Nashville*, *TN*

**Background:** Eosinophilic esophagitis (EoE) is a chronic inflammatory disorder of the esophagus characterized by intense eosinophilic infiltration and subepithelial fibrosis. Increased oxidative stress has been shown to contribute to the development of fibrosis in other organs. In addition, transforming growth factor beta (TGF‐β), which plays a central role in activating fibroblasts in EoE, has been shown to increase reactive oxygen species production in several cell types. However, little is known about the role of increased oxidative stress in EoE fibrosis. Thus, our aim was to determine if reducing oxidative stress with N‐Acetylcysteine (NAC), an antioxidant, could attenuate fibrosis in EoE.

**Methods:** Either immortalized FEF3 cells or primary esophageal fibroblasts established from 1 non‐EoE control patient and 1 active EoE patient were utilized. Primary fibroblast cells were derived from mucosal biopsies obtained during upper endoscopy. Active EoE was defined according to the AGREE 2018 guidelines. A ROS‐Glo Assay was completed on primary esophageal fibroblasts to quantify levels of intracellular H_2_O_2_. To assess fibroblast activation, collagen contraction assays were performed on either FEF3 cells or non‐EoE control primary fibroblasts. In experiments with FEF3 cells, 75,000 cells were suspended in collagen plugs, and for primary fibroblasts, 50,000 cells were used. Contraction was measured over 4 days with no treatment, 10 mM NAC, 10 ng/ml recombinant human TGF‐β, or both NAC and TGF‐β. For *in vivo* experiments, an experimental esophagitis murine model was used, and experimental mice received 1 µg of recombinant IL‐33 intraperitoneal injections daily for 7 days. To test if NAC could prevent histopathologic characteristics of fibrosis, mice received 3% NAC mixed in sucrose to the drinking water during the 7 days. Esophageal tissue was collected for histology and trichrome stain completed to measure collagen deposition.

**Results:** Primary esophageal fibroblasts from an active EoE patient displayed a 71% increase in intracellular H_2_O_2_ compared to a non‐EoE control patient (p=0.0079). As expected, FEF3 and primary esophageal control fibroblasts treated with recombinant human TGF‐β for 4 days led to a significant increase in collagen contraction as compared to baseline (1.00 v 0.65, p=0.007; 1.00 v 0.85, p=0.016, n=3‐4 independent experiments). However, when treated with NAC in addition to TGF‐β, collagen contraction was significantly less as compared to treatment with TGF‐β (1.01 v 0.65, p=0.0072; 1.09 v 0.85, p=0.0014) (Figure 1). Subepithelial esophageal collagen thickness in mice was significantly decreased in mice that received 3% NAC in their drinking water as compared with mice that did not receive NAC during induction of experimental EoE (15.49 µm v 10.75 µm, p=0.0003) (Figure 2).

**Conclusion:** Esophageal fibroblasts derived from a patient with active EoE exhibited higher levels of oxidative stress compared to esophageal fibroblasts from a non‐EoE control patient. *In vitro*, collagen contraction was increased in FEF3 and non‐EoE control fibroblasts treated with TGF‐β, and this was reduced with the addition of NAC, suggesting a critical role for oxidative stress in TGF‐β mediated fibroblast activation. *In vivo*, mice that received NAC in drinking water during IL‐33 induced experimental EoE had less subepithelial collagen as compared with mice that received only IL‐33. Thus, NAC may be useful in either treating or preventing fibrosis in EoE. Further studies, including more patient samples, investigating the role of oxidative stress in the development of fibrosis in EoE are needed.


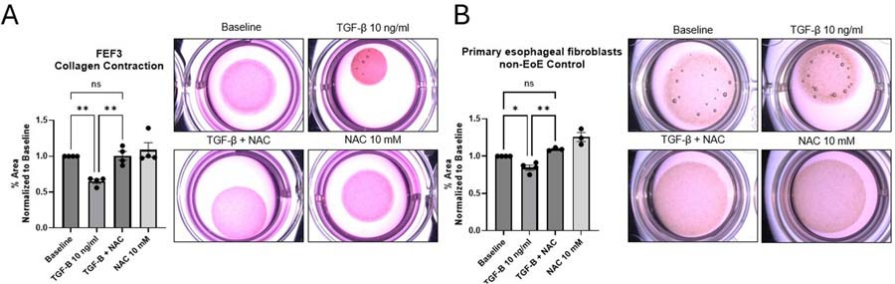


**NAC attenuates TGF‐β mediated fibroblast activation via collagen contraction assays**. TGF‐β reduces the area of the collagen plug as compared to baseline in both FEF3 and a control fibroblast line. The addition of NAC to TGF‐β treatment attenuates collagen contraction. No significant difference between NAC alone and baseline was observed. (*P<0.01)


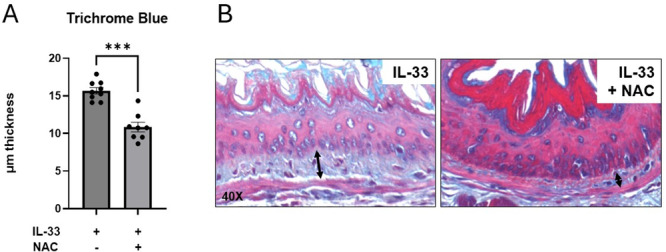


**NAC reduces collagen thickness in the subepithelium of IL‐33 induced experimental EoE mice**. When mice received 3% NAC in drinking water, the collagen layer was diminished compared to mice treated with IL‐33 without NAC (P<0.001).

## 881 UNIQUE GENE SIGNATURES IDENTIFY TREATMENT‐RESPONSE AND AGE‐SPECIFIC DIFFERENCES BETWEEN ADULTS AND CHILDREN WITH EOSINOPHILIC ESOPHAGITIS

*Krishan Chhiba* ^*2*^, *Michael White* ^*1,2*^, *Ming‐Yu Wang* ^*1,2*^, *Barry Wershil* ^*1,2*^, *Nirmala Gonsalves* ^*2*^, *Joshua Wechsler* ^*1,2*^

^*1*^ *Pediatrics*, *Ann & Robert H. Lurie Children's Hospital of Chicago*, *Chicago*, *IL*; ^*2*^ *Northwestern University Feinberg School of Medicine*, *Chicago*, *IL*

**Background:** Eosinophilic esophagitis (EoE) is a chronic inflammatory disease that affects children and adults. Numerous studies have confirmed an elimination diet (ED) with removal of specific food antigens improves symptoms and inflammation. The EoE diagnostic panel (EDP) is an age‐independent gene signature that can identify Active EoE (>15 eosinophils per high‐power‐field). However, gene signatures that distinguish adult and pediatric EoE as well as ED treatment response have not been identified.

**Methods:** To establish age‐specific gene signatures, we performed bulk RNA‐Seq on esophageal biopsies of 44 adults (Active EoE:38/Controls:6). Differentially expressed genes (DEGs) with LogFC>2 and adjusted p‐value<0.05 were determined with *limma* and overlapped with DEGs from 2 existing pediatric bulk RNA‐Seq datasets that compared Active EoE to control (GSE58640/GSE197702). Gene values were normalized and summed to form a score for comparison. Validation was performed on independent adult (GSE271128) and pediatric (GSE113341) RNAseq datasets that compared Active EoE to control. To identify a ED response signature, we performed RNA‐seq on esophageal biopsies taken before and after ED therapy for pediatric (N=33) and adult (N=22) EoE patients. RNA‐Seq was performed on 9 adult EoE ED‐responsive patients that had food reintroduction with Active EoE relapse. Shared DEGs among pediatric and adult ED treatment responders were compared between timepoints and to ED non‐responders.

**Results:** Distinct adult and pediatric gene signatures were identified and independently validated. The adult signature had 28 genes, while the pediatric signature had 41. Pediatric and adult EoE shared 87 genes that formed an age‐independent EoE signature. While the age‐independent and pediatric signatures overlapped with the EDP, the adult signature was distinct (Fig 1 A). Pathways analysis showed upregulation of NK‐mediated immunity and downregulation of intermediate filament organization in the adult signature while the pediatric signature had upregulation of anti‐viral processes (Fig 1B). The age‐independent signature had upregulation of mast cell and T‐cell activation and downregulation of epithelial differentiation. Pediatric and adult EoE patients that responded to ED shared a 52‐gene signature with upregulation of epithelial differentiation and down‐regulation of T‐cell and mast cell activation. The ED response signature had 13 genes not found in the EDP or age‐independent signature (Fig 2 A). The ED response signature was responsive to food removal and reintroduction in ED treatment responders (Fig 2B) and differentiated adult ED responders from non‐responders prior to treatment (AUC:0.7, p=0.002, Fig 2 C).

**Conclusion:** We identified unique transcriptional signatures specific to adult EoE and ED response that hold promise for identifying novel age‐specific and ED‐responsive pathways as well as a more personalized approach to dietary therapy in EoE.


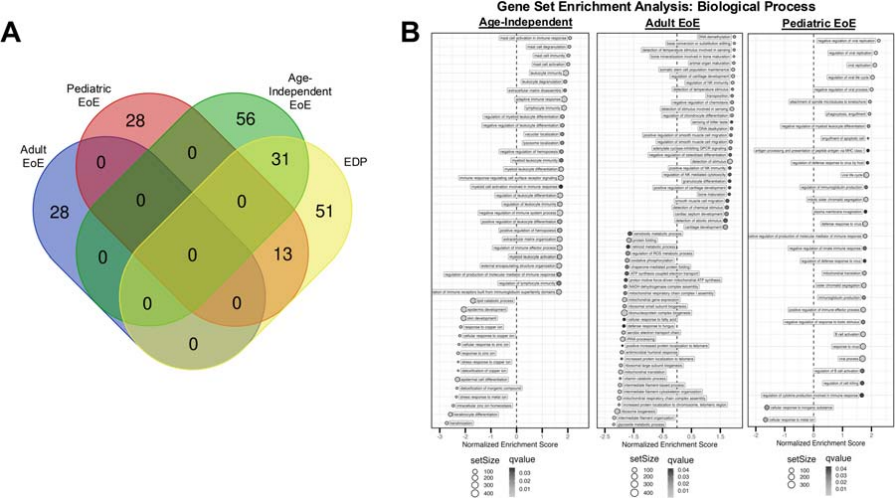


Fig 1. Overlap of gene signatures (A) with EDP and pathway analysis (B)


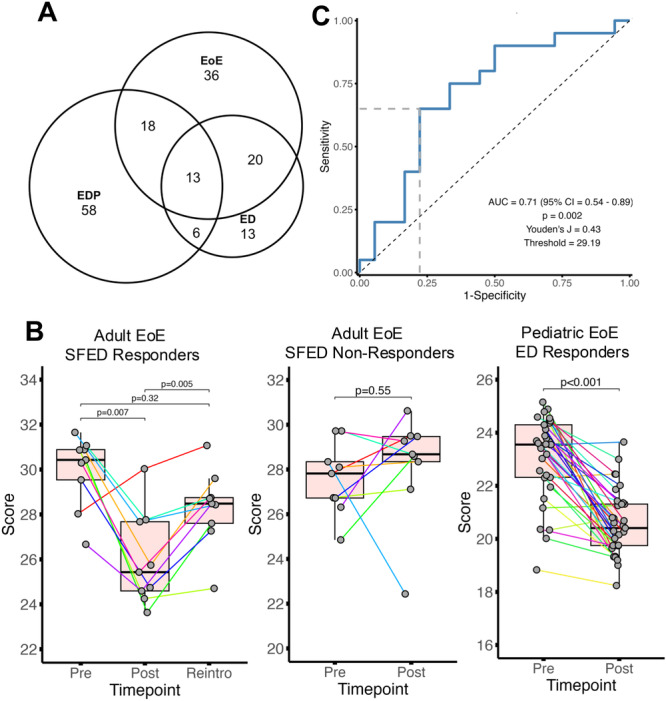


Fig 2. A) The ED response signature was characterized by 13 genes not found in the EDP or age‐independent signature. B) The ED response signature was responsive to food removal and reintroduction in ED treatment responders and C) differentiated adult DE responders from non‐responders prior to treatment.

## 882 ARE YOU SMARTER THAN AN AI CHATBOT? EVALUATING AI PERFORMANCE ON AAP PREP® GI SELF‐ASSESSMENTS

*Sharmilaa Babu*, *RESHMA PATEL*, *Annette Roberts*, *arun ajmera*

*Pediatric Gastroenterology*, *Duke University*, *Durham*, *NC*

**Introduction:** Generative AI tools like OpenAI's ChatGPT and Microsoft Copilot, powered by large language models (LLMs), have demonstrated the ability to pass general medical licensing exams. However, their performance on highly specialized assessments, such as the pediatric gastroenterology (GI) board exams, remains untested. Pediatric gastroenterology is a field where precision matters. Diagnoses hinge on subtle clinical clues, age‐specific presentations, and evolving treatment protocols. This study puts three AI chatbots—ChatGPT‐3.5, ChatGPT‐4o, and Microsoft Copilot—to the test using the AAP PREP® GI Self‐Assessments.

**Methods:** We tested the three chatbots on 216 questions from the 2022–2024 AAP PREP® GI Self‐Assessments. Each chatbot received the same questions in a simulated test‐taking environment, with a second attempt allowed after incorrect responses. Questions were categorized by content domain and analyzed for performance on media‐ and table‐based questions. Scores were compared to the AAP's passing threshold (>65%) and average first‐time test taker scores. Data were collected using REDCap and analyzed in Stata. Each chatbot session was reset every 18 questions to simulate a human test‐taking experience and avoid session length limitations. Media files and table formatting were lost during input, and no additional prompts were given after a second incorrect attempt. The questions were input between November 14 and 29, 2024, and categorized using the 2024 PREP® content domain categories for consistency across years.

**Results:** ChatGPT‐4o and Microsoft Copilot passed all three assessments. ChatGPT‐3.5 passed in 2023 and 2024 but failed in 2022. All chatbots performed best in anatomy, motility, and esophageal disorders, and worst in physiology, pharmacology, and liver/stomach/duodenum disorders. Despite the absence of images, chatbots answered over 50% of media‐based questions correctly, though with lower accuracy than text‐only questions. Table‐based performance varied, with GPT‐3.5 outperforming its overall score in 2023. Second‐attempt accuracy was significantly lower for GPT‐3.5 in 2023 compared to the other models. No significant differences were observed between chatbot types or across quarters. In 2024, human learners outperformed all three AI models. None of the chatbots consistently outperformed human first‐time test takers across all years. Additionally, performance on questions involving tables was generally lower than overall scores, except for GPT‐3.5 in 2023, which showed a slight improvement.

**Discussion:** While newer models like GPT‐4o and Copilot showed more stable performance, variability across years and domains suggests that AI performance is influenced by both model architecture and question content. Chatbots struggled most with questions requiring integrative reasoning or visual interpretation. Although they demonstrated contextual understanding, their limitations in handling media and nuanced clinical reasoning highlight the need for further development before clinical application. Domain‐level analysis revealed strengths in structured topics like anatomy and research, but weaknesses in conceptual areas like physiology and liver disorders. GPT‐3.5 showed inconsistent second‐attempt performance, raising concerns about adaptability. These findings underscore the importance of validating AI tools in domain‐specific contexts before educational or clinical integration. The inability to process visual data and the variability in performance across content types and years suggest that current LLMs, while promising, are not yet reliable substitutes for human expertise in high‐stakes medical assessments. Future research should explore the integration of multimodal capabilities and assess AI performance in real‐world clinical scenarios.

**Conclusion:** Advanced AI chatbots like ChatGPT‐4o and Microsoft Copilot show promise in passing pediatric GI board‐style assessments. However, their inconsistent performance across domains and inferiority to human learners in some areas highlight the need for cautious integration into medical education. These tools may serve as valuable adjuncts for board preparation and clinical reasoning exercises, but they should not be viewed as replacements for human expertise.